# Functionally Divergent Splicing Variants of the Rice AGAMOUS Ortholog OsMADS3 Are Evolutionary Conserved in Grasses

**DOI:** 10.3389/fpls.2020.00637

**Published:** 2020-05-25

**Authors:** Ludovico Dreni, Andrea Ravasio, Nahuel Gonzalez-Schain, Sara Jacchia, Glacy Jaqueline da Silva, Stefano Ricagno, Rosaria Russo, Francesca Caselli, Veronica Gregis, Martin M. Kater

**Affiliations:** ^1^Department of Biosciences, Università degli Studi di Milano, Milan, Italy; ^2^Plant Genomics and Breeding Center, Federal University of Pelotas, Pelotas, Brazil; ^3^Department of Pathophysiology and Transplantation, Università degli Studi di Milano, Milan, Italy

**Keywords:** AGAMOUS, alternative splicing, OsMADS3, floral organ identity, protein structure, rice, transcription factor, homeotic

## Abstract

Within the MADS-box gene family, the *AGAMOUS*-subfamily genes are particularly important for plant reproduction, because they control stamen and carpel identity. A number of studies in the last three decades have demonstrated that the *AGAMOUS* (*AG*) function has been conserved during land plant evolution. However, gene duplication events have led to subfunctionalization and neofunctionalization of *AG*-like genes in many species. Here we show that alternative splicing in *Oryza sativa* produces two variants of the AG ortholog OsMADS3 which differ in just one serine residue, S109. Interestingly, this alternative splicing variant is conserved and specific to the grass family. Since in eudicots the S109 residue is absent in AG proteins, stamen and carpel identity determination activity of the two rice isoforms was tested in *Arabidopsis thaliana*. These experiments revealed that only the eudicot-like OsMADS3 isoform, lacking the serine residue, had ability to specify stamens and carpels in *ag* mutant flowers, suggesting an important functional role for the serine residue at position 109 in AG proteins of grasses.

## Introduction

The evolution of flowers as reproductive structures is the most remarkable feature of angiosperms (flowering plants), which currently are by far the most dominating group of plants on Earth, counting over 350,000 species^1^. In its basic model, the flower is a bisexual structure composed of perianth organs surrounding the reproductive organs, the male stamens and the female gynoecium, which are located in the center of the flower. All organs are arranged either in a spiral phyllotaxy or in whorls, depending on the angiosperm lineage. The gynoecium can be composed of one or more pistils made by one carpel each, or by one pistil formed by multiple fused carpels. The pistil consists of a stigma, style and ovary which contains the ovules. The pistil upon pollination eventually becomes a fruit, and the ovules develop into seeds (Bowman, 1997).

The two most evolved and successful angiosperm *taxa* are referred to as eudicots and monocots, comprising *ca*. 75% and 20% of the living plant species, respectively. It is estimated that divergence between eudicot and monocot species occurred about 150 million years ago (MYA) (Magallon et al., 2015). Plants belonging to the two lineages evolved inflorescences and flowers that show an enormous variation in complexity and shapes, evidencing the forces of natural selection. Examples of structural diversity can be noticed by comparing the morphologies of two model species representing each lineage, the core eudicotyledon *Arabidopsis thaliana* (L.) Heynh. and the monocotyledon *Oryza sativa* L. However, if we compare the basic floral structure of these two species, a conserved organization emerges, in which four different types of floral organs are arranged into four concentric whorls. The two most outer whorls bear so-called perianth organs: (1) four sepals in Arabidopsis and the lemma and palea in rice (Lombardo and Yoshida, 2015), and (2) the petals or lodicules in Arabidopsis and rice, respectively. The male and female reproductive organs are located in the most inner two whorls. Both Arabidopsis and rice flowers develop six stamens in the third whorl, and in the innermost fourth whorl of Arabidopsis a bicarpellate pistil develops, whereas in rice the pistil is tricarpellate (Smyth et al., 1990; Yoshida and Nagato, 2011).

*AGAMOUS (AtAG)*, a member of the MADS-box TF family (Dreni and Kater, 2014), is essential for stamen and carpel development in Arabidopsis (Bowman et al., 1989, 1991; Yanofsky et al., 1990). The *ag* loss-of-function mutant completely lacks reproductive organs; petals replace stamens in the third whorl and a new *ag* flower replaces the carpel in the fourth whorl, resulting in a complete loss of floral meristem determinacy (FMD) (Yanofsky et al., 1990). AGAMOUS acquires its reproductive organ identity function through the formation of a complex with SEPALLATA (AtSEP) MADS-domain proteins (Pelaz et al., 2000). Complexes formed by AtAG and AtSEP proteins direct carpel development, whereas in the third whorl AtSEP transcription factors mediate the interaction between AtAG, APETALA3 (AtAP3) and PISTILLATA (AtPI) to form the MADS-domain stamen identity complex (Theissen, 2001; Theissen and Saedler, 2001).

The *AGAMOUS* subfamily is typically represented by two or more genes in each angiosperm species, having various degrees of functional redundancy and sub-functionalization, and is further divided into many paraphyletic lineages. The main division which is important to mention here is the one between the *AG* and *AGL11* lineages (Kramer et al., 2004; Zahn et al., 2006), which diverged before the most recent common ancestor of extant angiosperms. All members of the *AGAMOUS* subfamily cluster into one of these two groups. Generally speaking, the genes providing carpel and stamen identity and FMD function belong to the *AG* lineage, whereas the *AGL11*-like genes provide ovule identity (Kramer et al., 2004; Zahn et al., 2006; Dreni and Kater, 2014). However, despite their ancestral origin, functional redundancy between the two clades is common (Pinyopich et al., 2003; Brambilla et al., 2007; Dreni et al., 2011; Heijmans et al., 2012).

Interestingly, in monocot grasses (Poaceae), the *AG* and *AGL11* lineages are further duplicated to form four conserved clades which are named after their representative genes of rice. Among them, the *OsMADS3* and *OsMADS58* clades belong to the *AG* lineage whereas the *OsMADS13* and *OsMADS21* clades belong to the *AGL11* lineage (Kramer et al., 2004; Yamaguchi et al., 2006; Zahn et al., 2006; Dreni and Kater, 2014). Therefore, the rice genes *OsMADS3* and *OsMADS58* are both direct orthologous of Arabidopsis *AtAG* with a similar expression domain, and they redundantly control the reproductive organ identity function in rice. Like the Arabidopsis *ag* mutant, the *osmads3 osmads58* double mutant shows a complete loss of reproductive organ identity; stamens are replaced by lodicules and an small ectopic palea emerges in place of the carpel. Moreover, FMD is also lost and the flower continues to produce ectopic lodicules from its center (Dreni et al., 2011). The phenotypes of the single mutants suggest that *OsMADS3* has an important role in stamen development, whereas the two genes seem to be almost completely redundant for pistil development (Dreni et al., 2011; Hu et al., 2011).

Like the eudicot AG proteins, rice OsMADS3 and OsMADS58 form complexes with rice SEP*-*like factors (Favaro et al., 2002; Cooper et al., 2003; Hu et al., 2015; Wu et al., 2018).

Ectopic *AtAG* expression in Arabidopsis results in a homeotic conversion of sepals into carpel-like organs and of petals into stamens (Mizukami and Ma, 1992). Several functional studies, using *AG* orthologous genes from other eudicot, monocot or even gymnosperm species, showed that when ectopically expressed in Arabidopsis, they mimic the ectopic expression of *AtAG*, or in some cases they even rescue the *ag* loss of function mutants (Rutledge et al., 1998; Kitahara and Matsumoto, 2000; Zhang et al., 2004; Martin et al., 2006; Airoldi et al., 2010; Heijmans et al., 2012). Stunningly, the *AGAMOUS* subfamily gene from the gymnosperm *Cycas edentata* de Laub. was able to rescue the Arabidopsis *ag* mutant, despite the two species likely diverged about 300 MYA (Zhang et al., 2004). Another study shows very clearly the importance of the conservation of the domains required for protein complex formation. The ectopic expression of the snapdragon *AG* lineage genes *PLENA (AmPLE)* and *FARINELLI (AmFAR)* either in Arabidopsis or snapdragon revealed that the two genes have different abilities in organ specification: *AmPLE* can convert petals into stamens and induces carpelloid features on the sepals, similar to *AtAG* ectopic expression, while *AmFAR* only affects petal identity (Davies et al., 1999; Causier et al., 2005; Airoldi et al., 2010). Airoldi and colleagues showed that this different ability of AmPLE and AmFAR is due to a different affinity to interact with SEP proteins (Airoldi et al., 2010). Indeed AmFAR is only able to interact with Arabidopsis AtSEP3, which is not expressed in sepals, but not with the other Arabidopsis AtSEPs. Hence, when AmFAR is expressed in Arabidopsis sepals it can’t find any SEP interaction partner and remains therefore inactive. The restricted interaction ability of AmFAR is the result of a single glutamine insertion, Q173, near the end of the K domain. Q173 is the result of a three-base duplication of the splice acceptor site (CAG) at the 5′ end of AmFAR exon seven (Airoldi et al., 2010).

Extensive duplications of homeotic genes have likely contributed to the evolution of floral structures and their diversification in angiosperms. Besides gene duplication, variants of proteins can also arise via alternative splicing of transcripts from the same gene. For MADS-box genes of plants, a few cases of alternative splicing have been reported. Considering only the *AGAMOUS* subfamily, exon skipping gives rise to the double-flowered mutant of *Prunus lannesiana* (Carrière) E.H. Wilson (Rosaceae)(Liu et al., 2013). Alternative splicing of *AG* genes has also been reported in a few other angiosperms (Kitahara and Matsumoto, 2000; Tsaftaris et al., 2005; Lightfoot et al., 2008; Zhang et al., 2015), but the functional significance of these events is unknown. To date, a complete functional characterization of splicing variants has only been carried out for the *AG* lineage gene *PapsAG* from opium poppy (*Papaver somniferum* L.) (Hands et al., 2011). *PapsAG* undergoes alternative splicing generating two different transcripts, *PapsAG-1* and *PapsAG-2*, which are co-expressed in stamens and carpels and only differ in the last part of the *C*-terminal region. Even though they redundantly share the conserved functions of *AG*, *PapsAG-2* has distinctive roles in gynoecium development, particularly in the septum, ovule and stigma (Hands et al., 2011).

Here, we describe the identification of two alternative splicing isoforms of the rice *OsMADS3* gene, which differ for only three nucleotides in length, resulting from a three-base pair duplication of a splice acceptor site. The longer transcript encodes for an additional serine residue (S109) within the first predicted α-helix of the K-box, a domain whose length and amino acidic composition are highly conserved among angiosperms (Kaufmann et al., 2005). Search on public databases hints that the two splicing variants are broadly expressed in reproductive tissues, and probably ubiquitously within the whole *OsMADS3* clade of the grass family. Functional analysis revealed differences in their ability to rescue the *ag* mutant phenotype in Arabidopsis, which might be due to different affinities of the splice variants for the AtSEP1 protein.

## Materials and Methods

### Plant Materials

Mature wild-type (WT) inflorescences of *Arabidopsis thaliana* and *Oryza sativa ssp. japonica* cv. Nipponbare were used for total RNA isolation and gene cloning. The *ag-3* mutant in the Arabidopsis L*er* background (Bowman et al., 1991) was already available in our lab. Inflorescences from *A. thaliana* transgenic lines were used for total RNA isolation and Real Time PCR analysis.

### Bioinformatic Analysis of *AGAMOUS* Subfamily Member Splice Variants

To find suitable RNA-Seq samples from grasses and other Poales, we first interrogated the SRA database of Genebank^2^, with the names of species of interest, and we eventually selected the most suitable samples (i.e., reproductive stages, when available). Then, for each gene/species, we used the nucleotide BLAST tool (settings: optimize for somewhat similar sequences), to screen those data with a query cDNA sequence of 37-47 nucleotides, comprising the exon4-exon5 splicing region of *OsMADS3* and of its grass homologs, and downloaded all the homologous reads. Control query and read homologous sequences where then aligned with MAFFT, and edited manually with Genedoc. After removing the dubious or low quality reads (i.e., those too short or with more than one or two different bases) the alternative splicing events were counted, in order to calculate their relative abundance. The accession codes and available references of the RNA-Seq samples are listed in Table 1. All the selected reads are available in Supplementary Dataset 2 as Fasta format alignments. The monocot *AG*-lineage genes included in this work have been defined by sequence and phylogenetic analysis (Dreni et al., 2013; Dreni and Kater, 2014; data not shown) and their accession codes are available in Supplementary Table 3. The complete ORFs and protein products of *OsMADS3*^+*S*109^ and *OsMADS3* are also provided in Supplementary Dataset 1.

**TABLE 1:** Summary of the number of reads mapping on the exon 4-exon 5 junction of the *OsMADS3*-like genes from various grasses and from other families of the order Poales.

NCBI SRA Experiment	Species	Query sequence	Total high quality reads	Long splicing + S109		TAG deletion No S109	
SRX1332256; SRX507920; DRX000335; DRX000334; DRX000333 SRX100746 (Davidson et al., 2012)	***Oryza sativa ssp. japonica***	TAGCTTACAGAACGCAAACAG/TAG/GA CCATAGTGGGGGATTC	2021	**1238**	**61.3%**	**783**	**38.7%**
SRX472914	***Leersia perrieri***	CAGTAGCTTACAGAACGCAAACAA/GA CCATAGTGGGGGATTC	1205	**562**	**46.6%**	**643**	**53.4%**
SRX2342718; SRX2342716; SRX1583837; SRX1583838; SRX1583839 SRX100693; SRX100694; SRX100691 (Davidson et al., 2012)	***Brachypodium distachyon***	AGCCTGCAGAACTCAAACAG/TAG/GT CCTTAGTGAAGG	391	**324**	**82.9%**	**67**	**17.1%**
SRX375649; SRX378862 (Chen et al., 2014)	***Hordeum vulgare***	CTTGCAAAACTCAAACAG/TAG/GTCACTGG TGAGAGATT	39	**23**	**59%**	**16**	**41%**
SRX099185; SRX099141; SRX099021 (Davidson et al., 2012)	***Sorghum bicolor***	GCTTGCAAAACGCAAACAG/TAG/GACCATA GTGGGAGAT	223	**40**	**17.9%**	**183**	**83.1%**
SRX058598; SRX058600; SRX058607; SRX058605; SRX058604; SRX058601; SRX058599; SRX058597	***Zea mays***	ZMM2: CAGCTTGCAAAACGCAAACAC/TAG/GAAC ATAGTGGGAGATTC	496	**372**	**75%**	**124**	**25%**
		ZMM23: TAGCTTGCAAAACGCAAACAG/TAG/GACCA TAGTGGGAGATTC	118	**23**	**19.5%**	**95**	**80.5%**
SRX2375352; SRX2375353; SRX2375354 (Wang et al., 2017) SRX567871	***Phyllostachys edulis***	TAGCTTGCAGAACTCAAACAG/TAG/GAACT TAGTGGGGGATTC	83	**22**	**26.5%**	**61**	**73.5%**
SRX1639030	***Streptochaeta angustifolia***	TTACCACCTTGCAGAACAACAACAG/GACCA TAATGGGGGATTCTGTA	11	**1**	**9.1%**	**10**	**90.9%**
SRX1639019	***Ecdeiocolea monostachya*** ***(Ecdeiocoleaceae)***	TCACCAACTTGCAGAACTCCAATAG/CAG/GA CTATACAGGCAGGGGA	100	**24**	**24%**	**76**	**76%**
-	***Joinvillea ascendens (Joinvilleaceae)***	NO DATA AVAILABLE			
SRX1639020	***Flagellaria indica (Flagellariaceae)***	TACCAACTTGCAGAACTCAAATAG/GACTCTAC TGGGGGATTCA	23	**0**	**0%**	**23**	**100%**
ERX2099848; SRX1639024	***Elegia tectorum*** **and** ***E. fenestrata*** ***(Restionaceae)***	ACCAACCTACAGAACTCCAACAGTAGGATTT TTTTGGGGGAGTCT CTACAGAACTCCATCAGGATTTTGTTGGGGG AATGCCTTAGC	15	**2**	**13%**	**13**	**87%**
ERX1349704	***Luzula elegans (Juncaceae)***	AGCACATTAAATAACAGTAATAG/GAATTTGTT GGGCGAG	1921	**0**	**0%**	**1921**	**100%**
SRX1465570; SRX1465595	***Ananas comosus*** ***(Bromeliaceae)***	CAACCTCCAGAATTCAAACAG/GAATTTACT GGGTGAGTCTCT	232	**0**	**0%**	**232**	**100%**

*The percentage of reads indicating the presence or absence of the second TAG at the splicing acceptor site of intron 4 is reported. In each query sequence, the exon 4-exon 5 junction is indicated by a slash. All reads are available in Fasta format (Supplementary Dataset 1).*

**FIGURE 1:**
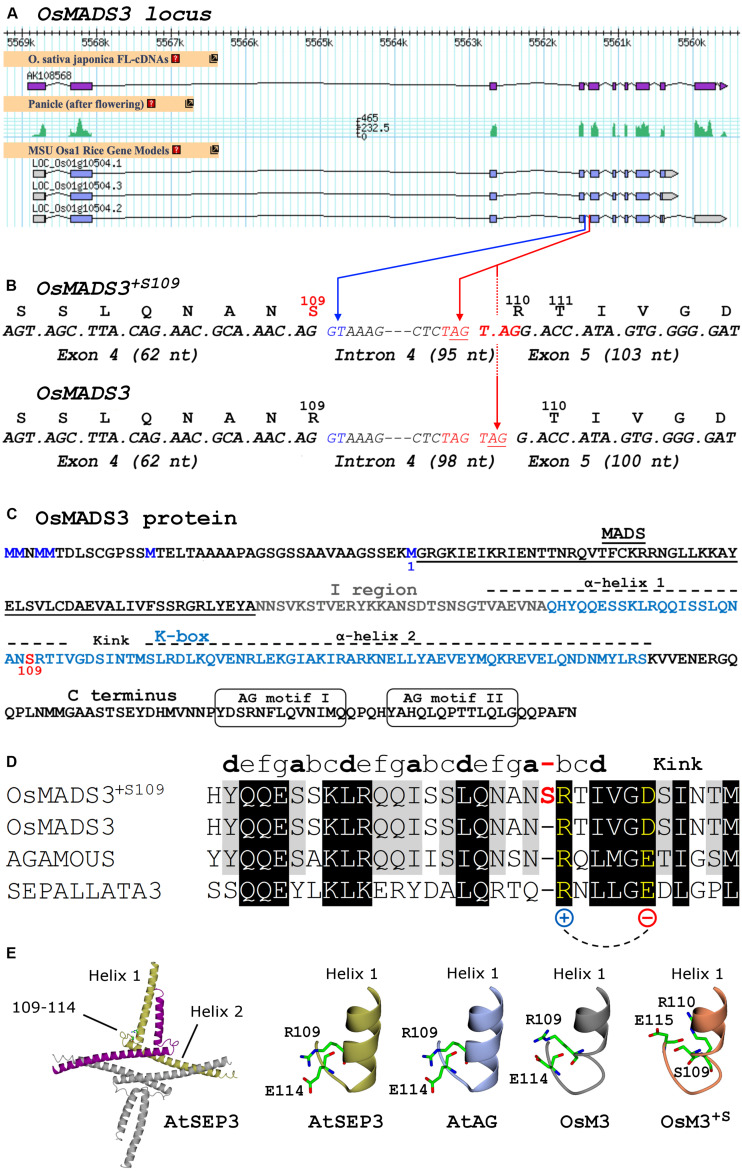
**(A)** Current gene models, mRNA-Seq and cDNA data of rice *OsMADS3* (*LOC_Os01g10504)* from the Rice Annotation Project Database (http://rapdb.dna.affrc.go.jp). **(B)** Detail of the *OsMADS3* locus around the fourth intron, including codons and translated protein isoforms of the two splicing variants. The blue arrow indicates the splicing donor site at the 5′ end of intron 4; the red arrows indicate the two alternative acceptor sites at the 3′ end of intron 4. **(C)** OsMADS3 protein sequence. The methionine residue preceding the MADS-domain (M1, blue), the serine at position 109 (red) and the main conserved motifs are highlighted. **(D)** Alignment of the region forming the first α-helix and the kink region, based on the crystal structure of AtSEP3 (Puranik et al., 2014), of AtSEP3 (bottom), AtAG, and the two isoforms of OsMADS3 (top). The partially conserved heptad repeats (abcdefg) of the first α-helix are indicated. The extra residue in OsMADS3^+*S*109^ (in red color) breaks the last partial heptad repeat between the positions ‘a’ and ‘b’, where ‘b’ is occupied by a conserved residue (in yellow) with positively charged side chain, which forms a salt bridge with a downstream conserved residue with negatively charged side chain (in yellow). This salt bridge contributes to the stabilization of the kink between the two α-helices (Puranik et al., 2014). **(E)** From the left to the right: cartoon presentation of the tetrameric structure of AtSEP3 K box; stick representation of the C-terminal region of helix 1 and the neighboring loop (kink) in the K box in AtSEP3, the position of R109, E110 and D115 side chains is shown. Structural models based on the AtSEP3 structure of the C-terminal region of helix 1 and the neighboring loop for AtAG, OsMADS3 and OsMADS3^+*S*109^. Putative conformations of residues 109, 110, 114, and 115 side chains are shown in sticks.

Open reading frames were controlled by Gene Runner. Images were processed with Gimp and Paint.NET. Structures were analyzed and Figure 1E were generated using COOT and CCP4mg software (Emsley and Cowtan, 2004; McNicholas et al., 2011).

### RNA Isolation

Total RNA was isolated using the LiCl precipitation method as previously described (Gregis et al., 2008). Total RNAs were converted into first-strand cDNA by using the ImProm-II Reverse Transcription System (Promega, Madison, WI, United States).

### Gene Cloning and Arabidopsis Transformation

The CDS of *OsMADS3*^+*S*109^, *OsMADS3* and *OsMADS58* were amplified in a thermocycler (MasterCycler EPGradient S, Eppendorf, Hamburg, Germany) using Phusion High-Fidelity DNA Polymerase (ThermoFisher Scientific, Waltham, MA, United States) using primers OsP337/OsP79 (for both *OsMADS3* isoforms) and OsP119/OsP120, respectively. The PCR products were isolated by gel electrophoresis, purified using NucleoSpin Gel Purification Kit (Macherey-Nagel, Duren, Germany) and cloned into pENTR/D-TOPO vector (Invitrogen). The recombination products were used to transform electrocompetent *E. coli* cells (DH10B strain) and colonies positive for the desired plasmids were isolated on LB-agar plates containing gentamycin (30 mg/L). Colony PCR was performed to confirm the presence of the constructs; in particular, colony PCR was performed to discriminate between colonies carrying *OsMADS3*^+*S*109^ and colonies carrying *OsMADS3*, using the reverse primers OsP428 and OsP429 with the forward primer OsP17. The different constructs have been sent for sequencing to the StarSEQ facility (Mainz, Germany). Suitable clones of *AtAG* in pDONR207 entry vector were already available in the laboratory.

Clones were transferred, using the Gateway LR Clonase II Enzyme (ThermoFisher Scientific, Waltham, MA, United States), into the pB2GW7 Gateway destination vector (VIB-Gent University) for expression under the control of the CaMV 35S promoter. The recombination products were used to transform electrocompetent *E. coli* cells (DH10B strain) and positive colonies were isolated on LB agar plates containing spectinomycin (100 mg/L). Colony PCR was performed to confirm the presence of the constructs. Plasmids were re-extracted using NucleoSpin Plasmid Isolation kit (Macherey-Nagel, Duren, Germany) and used to transform electrocompetent *A. tumefaciens* cells (EHA105 strain). Positive colonies were selected on LB-agar plates containing rifampicin and spectinomycin (100 mg/L). Colony PCR was performed to confirm the presence of the constructs.

*Agrobacterium* strains carrying *pB2GW7-AtAG, pB2GW7-OsMADS3*^+*S*109^, *pB2GW7-OsMADS3* and *pB2GW7-OsMADS58* were used to transform *ag-3/* + heterozygous plants in the *A. thaliana* L*er*. background (T_0_) with the floral dip method (Clough and Bent, 1998). The T_1_ of these plants was harvested and transformed plants were isolated through multiple rounds of BASTA selection. DNA extraction and PCR were performed on selected plants to confirm the presence of the transgene.

Primer sequences are available in Supplementary Table 1.

### Real-Time PCR

Inflorescences of *A. thaliana* were pooled by construct and background (WT, *ag-3*) and total RNA was extracted as described above.

The expression levels of *35S:AtAG, 35S:OsMADS3*^+*S*109^, *35S:OsMADS3* and *35S:OsMADS58* were evaluated by Real Time PCR using StoS Quantitative Master Mix (GeneSpin, Milano, Italy) and primers RT1898/RT1899, RT973/RT974 (for both *OsMADS3* isoforms) and RT975/RT976, respectively. For each construct, at least two independent lines were used (Supplementary Figure 4).

The expression levels of *SPOROCYTELESS (At4g27330), REM22 (At3g17010)* and *SHATTERPROOF1 (At3g58780)* were evaluated using primers RT1674/RT1675, RT1672/RT1673 and AtP650/AtP651, respectively.

For each experiment, three biological replicates were used and for each of these three technical replicates were done. Arabidopsis reference genes ubiquitin (*At4g36800*) and *ACT2-8* were used as an internal reference during the experiments. Primer sequences are listed in Supplementary Table 1.

### Yeast-2-Hybrid Assay

The analysis focused on the M, I, K and C domains of all proteins, therefore the putative *N-terminus* preceding the MADS domain in AtAG and OsMADS3 was not included in the clones. The CDS of the candidate MADS-box genes were amplified using the primers SEP1f/SEP1r (*AtSEP1*), AtP4929/AtP4930 (*AtSEP3*), and Atp7069/Atp7070 (*AtAG*) and cloned into the Gateway entry vectors pDONR201 and pDONR207 by BP reaction. Both *OsMADS3* splicing variants were amplified by the primers LD264/LD265 and cloned into pCR8/GW/TOPO TA Cloning Kit (Invitrogen); *AtSEP2* CDS was amplified by the primers AtP3141/AtP3142 and cloned into pENTR/D-TOPO vector (Invitrogen). All the primer sequences are available in Supplementary Table 1. After sequencing, the clones were transferred by LR reaction in the Gateway destination vectors *pGADT7* and *pGBKT7* plasmids for C-terminal fusion to the GAL4 activation domain (AD) and binding domain (BD), respectively. The recombination product was used to transform electrocompetent *E. coli* cells (DH10B strain) and positive colonies were isolated on LB agar plates containing ampicillin (100 mg/L) for *pGADT7* or kanamycin (50 mg/L) for *pGBKT7*.

Combinations of *pGADT7* and *pGBKT7* constructs (see Table 2) have been simultaneously used to transform chemo-competent *S. cerevisiae* cells (AH109 strain). Positive colonies for both plasmids were isolated on YSD-agar plates lacking leucine and tryptophan (YSD-L-W). Interaction assays were performed on YSD-agar plates lacking leucine, tryptophan and histidine and containing increasing concentrations of 3-amino-1,2,4-triazole (YSD-L-W-H + 3AT 1mM/2,5mM/5mM/10mM) or on YSD-agar plates lacking leucine, tryptophan and adenine (YSD-L-W-A). Growth for interaction assay was performed at 28°C for one week. As a positive control for interaction, *pGADT7-AtAG* and *pGBKT7-AtSEP3* constructs were used.

**TABLE 2:** Summary of yeast-2-hybrid assays.

pGAD/pGBK	-L-W-H	+ 1mM 3aT	+2.5mM 3aT	+ 5mM 3aT	+10mM 3aT	-L-W-A
1. Empty/OsMADS3 + S	+++	+	−	−	−	−
2. Empty/OsMADS3	++	+	−	−	−	−
3. Empty/AtAG	++	+	+	−	−	−
4. AtSEP1/Empty	++	+	−	−	−	−
5. AtSEP2/Empty	++	+	−	−	−	−
6. AtSEP3/Empty	+++	+	−	−	−	−
**7. AtSEP1/OsMADS3 + S**	** +++**	**+**	**−**	**−**	**−**	**−**
**8. AtSEP2/OsMADS3 + S**	** ++**	**−**	**−**	**−**	**−**	**−**
**9. AtSEP3/OsMADS3 + S**	** +++**	**+++**	** +++**	**+++**	** ++**	**++**
**10. AtSEP1/OsMADS3**	** +++**	**+++**	** +++**	**+++**	** ++**	**++**
**11. AtSEP2/OsMADS3**	** +++**	**−**	**−**	**−**	**−**	**−**
**12. AtSEP3/OsMADS3**	** +++**	**+++**	** +++**	**+++**	** ++**	**+++**
**13. AtSEP1/AtAG**	** +++**	**+++**	** +++**	**+++**	** +++**	**+**
**14. AtSEP2/AtAG**	**+++**	**+++**	**+**	**−**	**−**	**−**
**15. AtSEP3/AtAG**	** +++**	**+++**	** +++**	**+++**	** +++**	**+++**

*In each pair, the first protein is fused to GAL4 activation domain (AD) and the second protein is fused to GAL4 binding domain (BD). The symbols indicate strong growth (++ and +++), mild growth (+) or no growth (−) of yeast colonies on the different media.*

## Results

### *OsMADS3* Encodes Two Protein Isoforms Differing Only for One Serine Residue in the K-Box

*OsMADS3* (*LOC_Os01g10504*) is located on the short arm of rice chromosome 1, and is in the current MSU annotation represented by three gene models (Figure 1A), whose predicted protein products differ only for the few *C* terminal amino acidic residues after the conserved AG motif II (Kramer et al., 2004). The variants no. 2 and no. 3 have the same predicted protein product (Figure 1C), however no. 2 has at least one additional intron in the 3′UTR sequence, in addition to the eight conserved introns which are typical for *AG* lineage genes (Johansen et al., 2002; Figure 1A). The analysis of peaks of mRNA-seq reads and cDNA clones from public databases suggests that *LOC_Os01g10504.2* is the only correct and transcribed gene model (Figure 1A). We confirmed this by cloning the *OsMADS3* cDNA, but we noticed that some clones just lacked 3 nucleotides (TAG) when compared to the annotated transcript. These variants differed not for the C terminal region, but at the intron 4 – exon 5 splice junction which caused the loss of a serine (S) residue in the predicted OsMADS3 peptide (Figure 1B). Since the length of the N terminal region of AG lineage proteins has only been experimentally determined for Arabidopsis AtAG (Riechmann et al., 1999), we refer to this serine amino acidic position as S109, counting from the more conserved methionine residue just preceding the MADS domain (M1, Figure 1C). Based on known plant homologs, the lack of S109 is indeed the ancestral form of AG proteins. Therefore, we named the two protein variants OsMADS3^+*S*109^ (having S109) and OsMADS3 (lacking S109).

A deeper analysis revealed that in the *OsMADS3* locus residue S109 is encoded by an AGT codon which, in the pre-mRNA, is interrupted by the fourth intron (Figure 1B). The 3’ acceptor site of the fourth intron is duplicated into a TAGTAG repetition (Figure 1B). We found that the splicing machinery often recognizes the first AG-base pairs as acceptor site (*OsMADS3*^+*S*109^), confirming online annotations, but in a significant fraction of the transcripts the second AG-sequence is used instead, thus removing a TAG from the mature transcript and resulting in the absence of the serine residue in the encoded peptide (*OsMADS3*; Figure 1B). The existence of such two transcript variants was also confirmed by analyzing several mRNA-seq datasets from rice reproductive tissues which are available on the NCBI Sequence Read Archive (SRA). These data clearly show that wherever *OsMADS3* is expressed, roughly 39% of the transcripts carry this 3-nt deletion (Table 1). This amount varied substantially between different samples (range approx. 29–47%), but we could not find any obvious correlation with tissue type nor developmental stage, as the two transcripts were both present in young and mature whole panicles, stamens, ovaries and stigmas (not shown).

To better understand the role of residue S109 on the structural properties of OsMADS3, we analyzed the structure of the AtSEP3 K-domain, which is the only available crystal structure of a plant MADS-dimerization domain to date available (Puranik et al., 2014). In the AtSEP3 structure, which lacks S109, R109 is located at the C-terminal end of helix 1 close to a loop region which orientates perpendicularly to the two helices of the K-box (Figures 1D,E). In the AtSEP3 structure, R109 establishes a salt bridge with E114 stabilizing the orientation of helix 1 and the adjacent loop: this region is strongly involved in the AtSEP3 dimerization interface (Puranik et al., 2014). Given the high level of conservation in position 109 and 114, this salt bridge is likely present also in OsMADS3 and AtAG (Figures 1D,E). Conversely, the insertion of a S residue in position 109 should slide all residues one position forward and, consequently, R110 and E115 are not well positioned as in the absence of the S residue (Figure 1E). This likely results in a re-organization of the end of helix 1 and of the neighboring loop region. The overall high level of conservation of the adjacent residues (Figure 1D) suggests that such conformational change is local: it may involve only the loop region and likely allows the formation of the R110 – E115 salt bridge. Hence, the presence of S109 is expected to trigger a loop reorganization and this results in a minor modification of the dimerization interface compared to the one present in MADS-domain proteins devoid of S109 as AtAG and AtSEP3. These considerations suggest that the formation of mixed dimers between floral homeotic MADS-domain proteins carrying S109 could well be unfavored.

### The S109 Isoform Seems to Be Ubiquitously Present in Grass OsMADS3-Like Proteins

**FIGURE 2:**
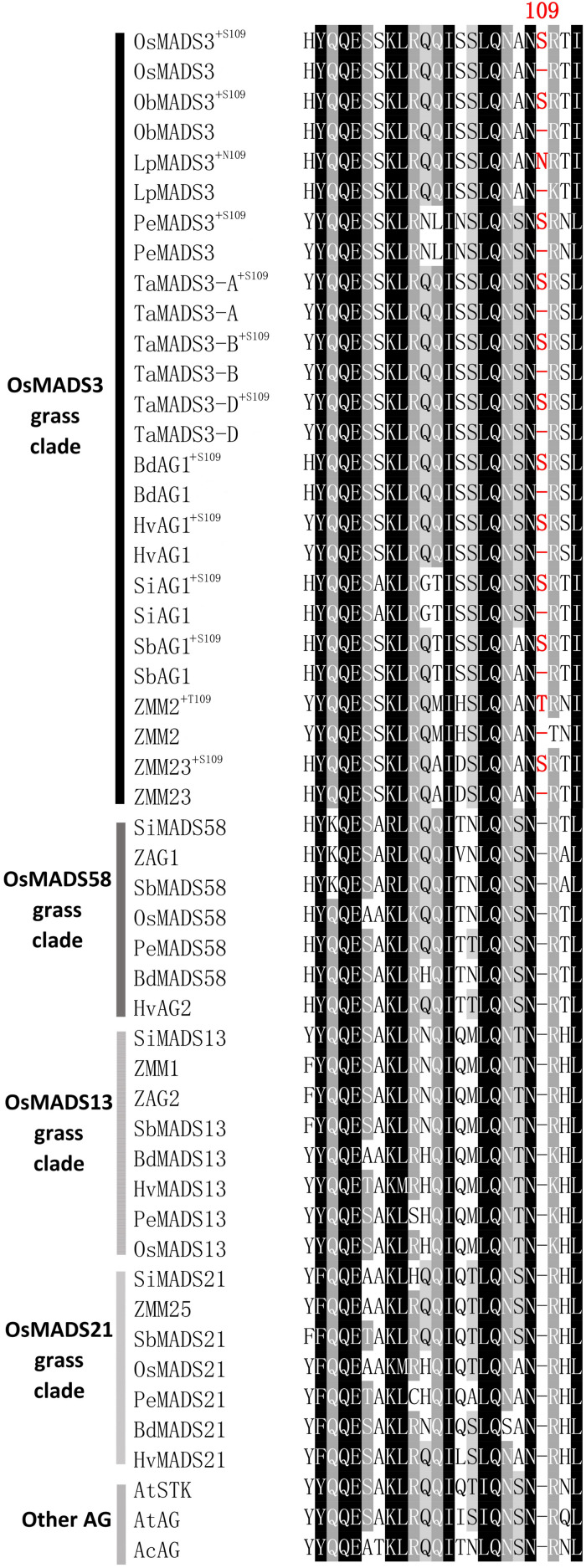
Alignment of the region forming the first α-helix of AGAMOUS subfamily proteins from different grasses, pineapple and Arabidopsis: Os, *Oryza sativa;* Pe, *Phyllostachys edulis;* Lp, *Leersia perrieri;* ZM, *Zea Mays;* Si, *Setaria Italica;* Sb, *Sorghum bicolor;* Ta, wheat (*Triticum aestivum*); Hv, *Hordeum vulgare;* Bd, *Brachypodium distachyon;* At, *Arabidopsis thaliana*; Ac, *Ananas comosus.* For each OsMADS3-like protein, both the isoforms with or without the additional amino acid residue 109 are shown. The protein sequences of AtSTK and AtAG from *A. thaliana* are included in the alignment as representatives of the dicotyledons.

To trace the origin of the *OsMADS3* alternative splicing, we did alignments of AGAMOUS subfamily protein sequences from several different angiosperms and gymnosperms. In angiosperms, the AGAMOUS subfamily divides into the AG and AGL11 lineages, which in the grass family are further duplicated into the OsMADS3 (AG), OsMADS58 (AG), OsMADS13 (AGL11) and OsMADS21 (AGL11) clades (Kramer et al., 2004; Zahn et al., 2006; Dreni and Kater, 2014). The alignments suggested that residue S109 is conserved in many OsMADS3 homologs from grass species, but it is absent in the other AGAMOUS subfamily proteins within the same grass species, including those from the OsMADS58 subgroup, which is sister to the OsMADS3 clade (Kramer et al., 2004; Yamaguchi et al., 2006; Zahn et al., 2006). The S109 residue was found to be absent outside the grass family, in all the flowering plants and gymnosperms that we analyzed (Figure 2 and Supplementary Figure 2).

**FIGURE 3:**
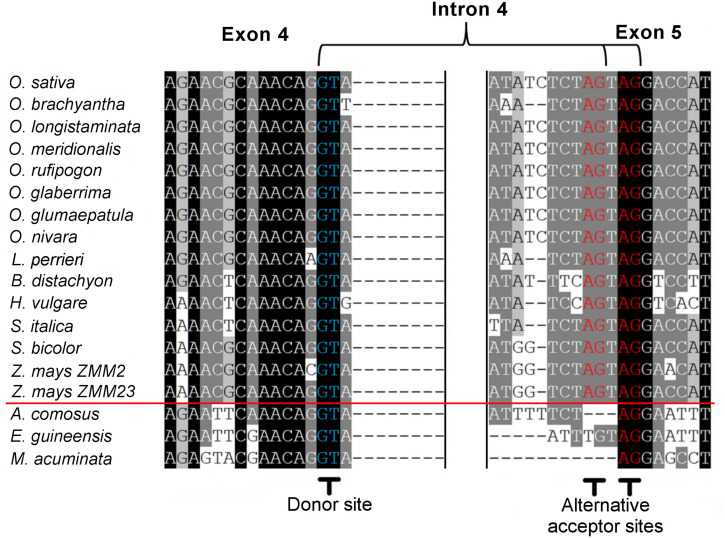
Alignment of corresponding fourth intron splicing sites from the pre-mRNA of *OsMADS3* and its direct orthologs from different monocotyledon species. The red line divides grasses (above) and non-grasses (below). The 5′ donor sites and the 3′ alternative acceptor sites are shown in blue and in red, respectively.

Within the grass *OsMADS3* clade, a search in the Phytozome and Gramene databases revealed that both splicing variants are currently annotated for rice *OsMADS3*, and for the *OsMADS3*-like genes of *Brachypodium distachyon* (L.) P.Beauv., barley (*Hordeum vulgare* L.), and maize (*Zea mays* L.) *ZMM2*. Both the variants of rice and *Brachypodium* are also reported on NCBI (XM_015777004, XM_015777012, XM_010232289 and XM_010232291). Furthermore, we noticed that this alternative splicing event corresponds to the one recently described for the *OsMADS3* homolog of *Aegilops tauschii* Coss (Wei, 2016), which is the D-genome donor of bread wheat (*Triticum aestivum* L.). The author interpreted the alternative splicing as involving an AGT instead of a TAG sequence, probably due to ambiguity in aligning the last two nucleotides ‘AG’ of the fourth exon in the short variant. However, only one of the two variants is annotated in other available grass genomes and in maize *ZMM23*. When we analyzed the genomic loci of various *OsMADS3* orthologs, we noticed that the TAGTAG duplication in the 3′ splice acceptor site of intron four, is conserved throughout the whole grass family, and only changes into CAGTAG in *Brachypodium* and barley (a selection of species is shown in Figure 3). This duplication is not observed in any of the three other clades of the *AGAMOUS* subfamily in grasses, i.e., *OsMADS13, OsMADS21* and *OsMADS58*. Therefore, we reasoned that the alternative splicing might occur ubiquitously in *OsMADS3* genes of grasses, without being fully represented in current databases. To verify this hypothesis, we extended our analysis of published mRNA-Seq reads to other species representative of the main grass evolutionary clades: *Streptochaeta angustifolia* Soderstr. (from the basal most subfamily Anomochlooideae), *Phyllostachys edulis* (Carrière) J. Houz. (Bambusoideae), *Leersia perrieri* (A.Camus) Launert (another species from Oryzoideae), *Brachypodium* and barley (Pooideae), *Sorghum bicolor* (L.) and maize (Panicoideae). Stunningly, the analysis revealed that the S109 alternative splicing is indeed conserved across grasses. Male, female and seed specific samples were available for *Brachypodium*, *Sorghum* and maize which indicated that, like in rice, the two transcript variants do not have any obvious tissue or stage specificity. The isoform lacking S109 was predominant only in *Streptochaeta*, *Leersia*, *Sorghum* and in maize *ZMM23*, on the contrary to maize *ZMM2* (Table 1).

Next, we traced the origin of this alternative splicing along the evolution of monocots. Outside the Poaceae family, the TAGTAG duplication allowing the alternative splicing doesn’t seem to exist. This is the case for other commelinids like *Musa acuminata* Colla, *Elaeis guineensis* Jacq. and *Ananas comosus* (L.) Merr., as well as any of the other angiosperms and gymnosperms that we analyzed (Figure 3). Within the order Poales, *Ananas comosus* (pineapple) is an excellent sister group to study the evolution of grass genomes, due to the availability of a high quality whole genome sequence (Ming et al., 2015), and because it diverged before the whole genome duplication (WGD) event which shaped all grass genomes (Paterson et al., 2004). In the only pineapple *AG* lineage gene, *AcAG*, there is no TAGTAG duplication in the fourth intron 3′ splicing acceptor site (Figure 3) and, accordingly, all its transcripts miss S109 (Table 1). Despite that no genomic data are yet publicly available from Poales other than grasses and pineapple, a limited amount of mRNA-Seq experiments allowed us to test the presence of the alternative splicing in most lineages. For example, nearly 2000 reads from *Luzula elegans* (Juncaceae) support only the conserved splicing typical of the *AGAMOUS* subfamily genes, like in pineapple (Table 1). The same result was obtained from all the main clades of Poales. Within Poales, the so called graminid clade includes grasses and three tiny sister families of just 11 species in total (Supplementary Figure 2). For two of these families, we did not find enough data to draw a conclusion, however, in Ecdeiocoleaceae we have found clear evidence of S109 alternative splicing (Table 1). Sister to the graminid clade is the family Restionacee (Supplementary Figure 2), where the S109 extra residue clearly exists in the genus *Elegia* (Table 1). Surprisingly, in the genera *Centrolepis* and *Aphelia*, a similar alternative splicing event removes the conserved R109 residue, rather than adding a S residue, generating protein isoforms lacking one amino acid when compared to typical AGAMOUS subfamily proteins (data not shown). Therefore, the alternative splicing of *AG* intron 4 possibly arose just before the most common ancestor of grasses and their sister families and Restionaceae (Supplementary Figure 2).

In most grasses, the additional amino acidic residue 109 is a serine, though some species show other polar neutral amino acids. For instance, in maize ZMM2 and in *Leersia* LpMADS3, the serine is replaced by threonine and asparagine, respectively (T109 and N109). This suggests that this residue is important not only for its position, but also for it physical/chemical properties.

In almost all the species that we considered, the presence or absence of the codon for S109 does not alter the translation of the surrounding codons, which usually encode an asparagine (upstream) and an arginine (downstream). In *Leersia perrieri*, a species very close to the *Oryza* subgroup, not only S109 is replaced by N109 in the long splicing variant, but in the short variant missing N109, the downstream arginine (R) is replaced by a lysine (K) (Figure 2), which is an amino acid with similar properties. An exception is maize ZMM2 where, in the short variant, splicing out the codon for T109 generates another codon for T, rather than for the highly conserved basic residue. Notably, based on RNA-Seq data, the short variant of ZMM2 has a much lower abundance. The opposite holds for the paralog ZMM23 for which the short variant keeps the basic conserved residue, and is much higher expressed (Table 1). This suggests that in maize the presence of two *OsMADS3*-like genes is favoring their subfunctionalization.

### OsMADS3 Isoforms Have Different Abilities to Induce Homeotic Changes in Arabidopsis Flowers

In order to compare the functionality of the two isoforms, *OsMADS3*^+*S*109^ and *OsMADS3* were expressed in Arabidopsis as an heterologous test system, along with *OsMADS58* and Arabidopsis *AtAG* as controls. The coding sequences were cloned downstream of the CaMV 35S promoter and these constructs were used to transform Arabidopsis plants which were heterozygous for the *ag-3* mutation (Bowman et al., 1991). In many previous publications, the effects of ectopic heterologous C-class gene expression have been evaluated in a WT background (Martin et al., 2006; Airoldi et al., 2010). In this study we used the progeny of a T_1_ generation segregating for the *ag-3* allele, because we were interested to observe the ability of rice proteins to complement the lack of *AtAG* activity in Arabidopsis.

By different rounds of transformation and BASTA selection, from 31 to 64 transgenic plants for each construct were selected. Of the selected transgenic plants 39% showed dwarfism, curled rosette leaves and were early flowering, except those expressing *OsMADS58* (Supplementary Table 2 and Supplementary Figure 3). These are typical phenotypes for plants ectopically expressing *AG*-like genes. Many of these plants also showed floral phenotypes with homeotic changes of floral organs, except those expressing *OsMADS58* which developed normally, as explained in a following paragraph.

**FIGURE 4:**
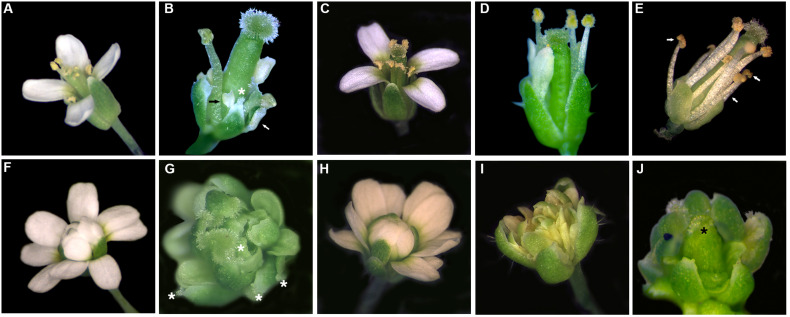
Flowers of plants expressing: **(B,G)** *35S:AtAG*, **(C, H)** *35S:OsMADS58*, **(D, I)** *35S:OsMADS3*^+*S*109^ and **(E, J)** *35S:OsMADS3* in WT **(B–E)** and *ag-3* **(G–J)** backgrounds, compared to WT **(A)** and *ag-3* mutant **(F)**. Ectopic stigmas on sepals (white asterisks), petal-to-stamen conversion (white arrows) and meristem determinacy recovery with formation of carpel-like organs (black asterisk) are highlighted.

**FIGURE 5:**
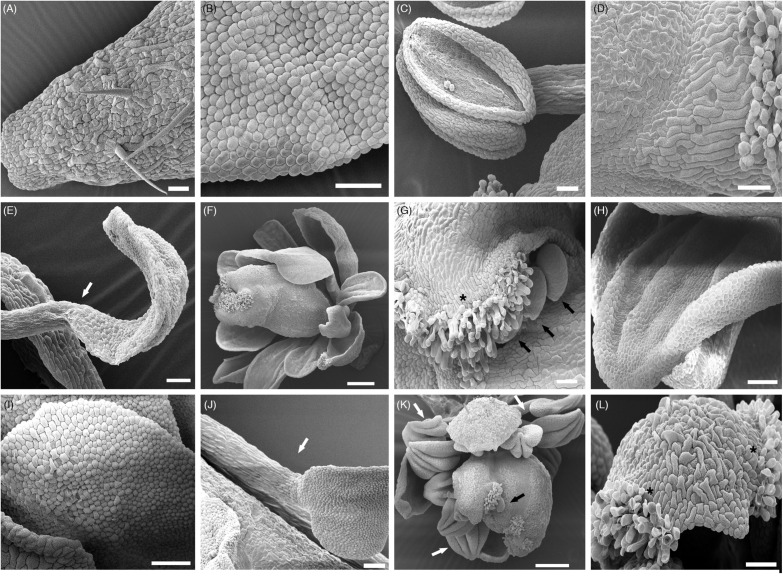
SEM analyses of ectopic organs. **(A)** WT sepal; **(B)** WT petal; **(C)** WT stamen; **(D)** WT carpel; **(E)** Staminoid-petal in *35S:AtAG* (WT background); **(F)** Meristem determinacy recovery in *ag-3 35S:AtAG*; **(G)** Ectopic stigmas and ovules on sepals in *ag-3 35S:AtAG*; **(H)** Mild petal-to-stamen conversion in *ag-3 35S:AtAG*, cells shape are more lobate than those in WT petals **(B)** resembling the shape of anther cells **(C)**; the organ acquires the typical folds of the anther; **(I)** Mild petal-to-stamen conversion in *ag-3 35S:OsMADS3*^+*S*109^, cells shape are more lobate than those in WT petals **(B)** resembling the shape of anther cells shown in **(C)**; **(J)** Petal-to-stamen conversion in *35S:OsMADS3;* **(K)** Staminoid-petals and meristem determinacy recovery in *ag-3 35S:OsMADS3;* an ectopic ovule is also visible; **(L)** Ectopic stigmas on sepals in *ag-3 35S:OsMADS3.* Ectopic stigmas on sepals (black asterisks), petal-to-stamen conversion (white arrows) and ectopic ovules (black arrows) are highlighted.

For each construct, a selection of T_1_ plants heterozygous for the *ag-3/* + mutation were chosen for further analysis in the T_2_ generation, so that we could observe the effect of the constructs in the WT and *ag-3* mutant backgrounds compared to untransformed controls (Figures 4A,F, 5A). We have also evaluated the expression level in two or more independent lines for each transgene (Supplementary Figure 4).

Probably due to the use of the heterologous 35S promoter lacking the correct spatial and temporal regulation of the endogenous promoter, *35S:AtAG* was not able to completely rescue the *ag* mutant phenotype.

In the WT and *ag-3/* + backgrounds, we could observe carpelloid sepals and a mild to strong conversion of second whorl petals into stamens or staminoid-like structures (Figures 4B, 5C,E). SEM images of anther-like petals in *35S:AtAG* plants showed that the lower part of the organs look very similar to anther filaments while the upper part of the organ resembles the shape of an anther, but the surface cells look spherical and resemble those of the petal surface (Figures 5B,C,E). Also, the shape of the upper part of the chimeric organ in the second whorl looks like a small petal (Figures 4B, 5E).

In the *ag-3* mutant background, *AtAG* expression from the 35S promoter resulted in petals that acquired a yellowish appearance and stigmatic tissues appeared on the sepals (Figure 4G). In addition, 30% of the plants recovered FMD almost completely. In these plants, the meristem terminated with a carpelloid organ arising from the fourth whorl, closed by stigmatic tissue at the tip (Figures 4G, 5F). None of the *35S:AtAG* lines in the *ag-3* background were able to produce seeds. Petals of *35S:AtAG ag-3* plants showed a mild conversion to anthers when observed by SEM (Figure 5H). The cell shape mostly recalled that of a normal petal except some group of cells which acquired a lobate shape typical of anther cells. Moreover the overall shape of the terminal part of these organs look like stamens. Another detail to notice is that, in the first whorl, the nature of the cells surrounding the ectopic stigmas in *35S:AtAG* lines in the *ag-3* mutant background varied from irregular sepal cells to smoother and longer cells which mostly recalled those of the style (Figures 5D,G). In some plants, ectopic ovules were visible near the stigmatic tissue on sepals (Figure 5G).

Plants transformed with the *35S:OsMADS58* construct never showed any change in phenotype, neither in WT nor in the *ag-3* background, regardless of the transgene expression level (Figures 4C,H). It is worth mentioning that the OsMADS58 protein lacks a conserved *C*-terminal AG II motif (Kramer et al., 2004; Dreni et al., 2013), which might affect its functionality in Arabidopsis.

In Arabidopsis plants expressing *35S:OsMADS3*^+*S*109^ in a WT background, the main phenotype was a strong reduction in sepal and petal length; as a result, reproductive organs were uncovered much earlier than usual, around stage 6–7 of flower development, while in WT flowers the bud opens around stage 13 (Figure 4D; stages indicated according to Smyth et al., 1990).

As mentioned before, the *ag-3* mutant flower is composed of a repetition of sepals – petals – petals – new flower, without any reproductive organ. *35S:OsMADS3*^+*S*109^ expression in the *ag-3* mutant background caused some petals to acquire a yellowish appearance (Figure 4I). SEM images show that the overall identity of these organs is that of a petal, even if some cells acquired a lobate shape typical of an anther-like cell (Figures 5C,I). Notably none of the plants were able to recover the FMD.

Interestingly, the plants that expressed *OsMADS3* from the 35S promoter had a floral phenotype that resembled the *35S:AtAG* plants. In the WT background they showed a strong petal-to-stamen conversion with cells having a lobate shape typical of anther-like cells (Figures 4E, 5B,C,J). In the *ag-3* background, stigmatic tissue was observed on outer sepals and about 60% of the plants also show FMD recovery and formation of carpel-like organs in the fourth whorl (Figures 4J, 5K,L). SEM images revealed that also in the *ag-3* background all petals were converted to stamens in an even more pronounced way than in plants expressing *35S:AtAG* (Figure 5K).

**FIGURE 6:**
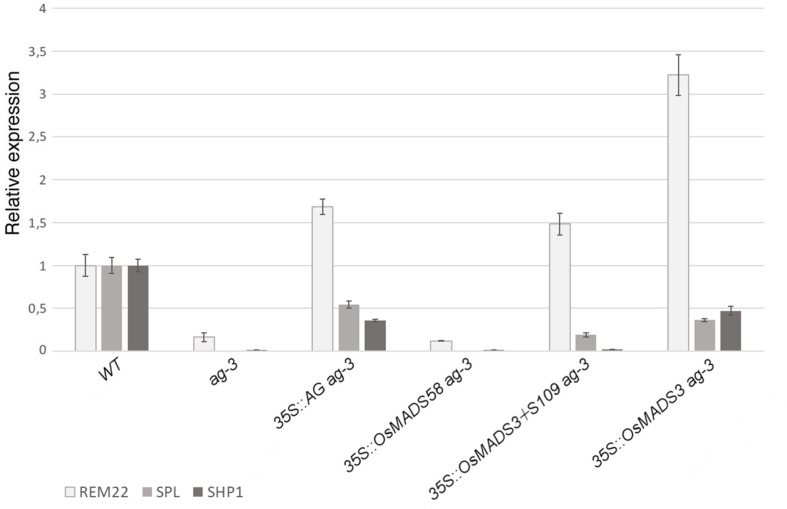
Expression of *REM22, SPL*, and *SHP1* in transgenic plants in the *ag-3* background. For each experiment, three biological replicates were used and for each of these three technical replicates were done. The expression levels have been normalized against the WT. SEM is indicated.

For each transgene, a representative line with the described phenotypes was chosen in order to test the expression of organ identity marker genes in the *ag-3* background, as a confirmation of the microscopical observations (Figure 6). Therefore, we performed qRT-PCR experiments to check the expression of three genes: *SPOROCYTELESS (SPL)* (Liu et al., 2009) and *REPRODUCTIVE MERISTEM 22 (REM22)* (Romanel et al., 2011), as markers for stamen identity, and *SHATTERPROOF1 (SHP1)*(Liljegren et al., 2000), as marker for carpel identity. These genes have been described as direct targets of *AtAG* (Gómez-Mena et al., 2005; Ó’Maoiléidigh et al., 2013) and they all appear strongly down-regulated in the inflorescence of *ag-3* (Figure 6).

In agreement with the observed phenotypes, transgenic plants containing *35S:AtAG* in the *ag-3* background, showed an upregulation of all marker genes (Figure 6). This suggests that, even if the mutant phenotype was not fully complemented, the floral program to produce stamens an carpels was activated, as shown by the microscopic analysis (Figure 5H).

The expression levels of marker genes in *ag-3* plants expressing *35S:OsMADS58* remained unaltered compared to the untransformed *ag-3* mutant (Figure 6) which perfectly correlates with the fact that no alteration in the flower morphology was observed in these lines.

In the *ag-3* plants expressing *35S:OsMADS3*^+*S*109^, qRT-PCR analysis showed upregulation of *REM22* and *SPL*, markers of stamen identity, despite that only a very mild homeotic transformation to stamens was observed in these plants (Figures 5C,I). The carpel identity marker *SHP1* remained unaltered compared to *ag-3*, and this is in line with the observed lack of carpel development, as confirmed by SEM analysis (Figure 5I).

The *ag-3* plants expressing *35S:OsMADS3*, as explained above, showed a phenotype that was very similar to those expressing *35S:AtAG.* This similarity is confirmed by the qRT-PCR analyses: not only the markers for stamen identity appeared upregulated, but also *SHP1* was upregulated in the *ag-3* background and this could account for the formation of carpel tissues which is not observed in plants expressing *35S:OsMADS3*^+*S*109^ (Figure 6).

### Comparative Interaction Studies of OsMADS3^+*S*109^ and OsMADS3 With SEP Proteins

The activity of AG proteins depends on the interaction with members of the SEP family. Since the difference between the two OsMADS3 isoforms is one amino acid in the first α-helix of the K region, which is known to function as the core dimerization interface between MADS-domain factors, we hypothesized that different interaction abilities may account for the difference in capacity to complement the *ag-3* mutant.

**FIGURE 7:**
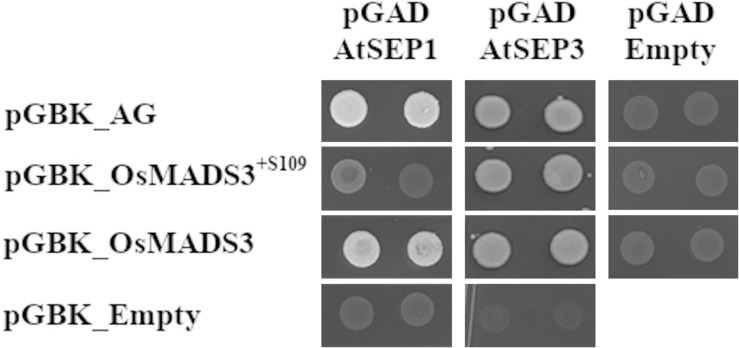
Yeast-2-hybrid interaction assay for AtAG, OsMADS3^+*S*109^, OsMADS3, AtSEP1 and AtSEP3, interactions on -W-L-H + 5 mM 3AT are shown. AtAG and OsMADS3 are able to interact with both AtSEP1 and AtSEP3, while OsMADS3^+*S*109^ can interact only with AtSEP3. As a positive control, the already published AtAG-AtSEP3 interaction was tested and, as a negative control, the vectors of interest were co-transformed with the empty pGAD or pGBK vectors.

We therefore tested the interaction of AtAG, OsMADS3^+*S*109^ and OsMADS3 with Arabidopsis AtSEP1, AtSEP2 and AtSEP3 proteins using the yeast-2-hybrid assay (Table 2 and Figure 7). The result was that AtAG (as expected), OsMADS3^+*S*109^ and OsMADS3 were all able to interact strongly with AtSEP3. However, OsMADS3^+*S*109^ was not able to interact with either AtSEP1 or AtSEP2, whereas OsMADS3 showed interaction with AtSEP1 but not with AtSEP2. The interaction between OsMADS3 and AtSEP1 had the same strength as the interaction observed for AtAG. These results suggest that the differences between OsMADS3^+*S*109^ and OsMADS3 in their ability to complement the *ag* phenotype, might be explained, at least in part, by their different capability to interact with AtSEP1.

## Discussion

All the proteins in the AGAMOUS subfamily, from both ancestral and evolved seed plant families, lack S, or any other additional residue, in the considered position 109 (Supplementary Figure 2), indicating that the insertion has occurred later during evolution. In particular, we provide strong evidence that it occurred after the differentiation of the monocot order Poales, but before the ρ WGD event which occurred in grasses (Supplementary Figure 1). Indeed we found evidence of the alternative splicing of *AG-*lineage intron 4 also in sister families of grasses in the graminid clade, whereas the grass ρ WGD has been dated after the separation of grasses from them (Supplementary Figure 1; McKain et al., 2016). Since this grass WGD caused the duplication of the *AG*-lineage into the *OsMADS3* and *OsMADS58* clades, we reason that the conserved alternative splicing arose in the pre-grass *AG*-lineage, but later in grasses it remained conserved only in the *OsMADS3* clade, and was lost in the *OsMADS58* clade. However, there are not yet sequenced genomes for the sister families of grasses, which would allow to better study the S109 evolution and to confirm that grasses do not share their ρ WGD and the *OsMADS3/OsMADS58* duplication with any sister family.

It looks like S109 has acquired an active and fundamental role in the functionality of OsMADS3-like proteins during evolution, since selective pressure seems to keep in place both isoforms. Despite the majority of *OsMADS3*-like genes are annotated only for one of the two isoforms, we showed that alternative splicing occurs widely in grasses. In most cases, the additional amino acidic residue is a serine, or another amino acid with a polar uncharged side chain. Therefore, the physical/chemical properties of this residue are likely to be important.

The TAG duplication that we report in grasses, is similar to the CAG duplication of a splice acceptor site in the snapdragon *FAR* gene, which results in an additional glutamine (Q173) in the last α-helix of the K-box (Airoldi et al., 2010). However, there is an important difference between the two cases: S109 is not present in all the OsMADS3 peptides because both the (T)AG sequences can work as splice acceptor sites whereas, based on current analysis, in *FAR* only the ‘new’ (C)AG repetition seems to be recognized as splice acceptor site (Airoldi et al., 2010).

It is widely accepted that the evolution and diversification of the flower went through the evolution of MADS tetrameric complexes and extensive gene duplications followed by sub- and neo-functionalization, which increased greatly the number of possible tetrameric combinations. A recent report on eudicot MADS-box genes revealed that in this evolutionary process the contribution of alternative splicing isoforms should not be ignored (Severing et al., 2012). However, the previously reported cases represent species-specific alternative splicing isoforms, with no or limited conservation within related species. To our knowledge, the alternative splicing that we reveal here is the first case, within the MADS-box family, of an alternative splicing which is highly conserved in a whole plant family, despite the fact that it causes a difference of just one amino acidic residue in the derived polypeptide. Its position in the first α-helix is also unusual, as previous cases involved the *C*-terminal part of the K-box or the C-terminus of the protein. Through a preliminary screen of grass genome databases, we found evidence of alternative splice isoforms in other MADS-box families, but none of them seemed to be conserved among species nor affected the first α-helix (LD, unpublished data). Therefore, we might have discovered a unique case within plant floral homeotic MADS-domain proteins or, on the contrary, similar events might be more common but simply passed unnoticed so far. With the massive and continuing increase in high-throughput plant genome and transcriptome data, large scale analysis across angiosperms are nowadays possible and may clarify the frequency by which this phenomenon occurs.

The level of conservation of this alternative splice event in grasses, suggests that both OsMADS3^+*S*109^ and OsMADS3 have specific functions. However, mRNA-seq data showed that the two alternative transcripts share similar expression profiles, thus apparently excluding tissue- or stage-specific functions for them, although our experiments can’t exclude cell specific functions for these two isoforms. Interestingly, also the results obtained from the expression and complementation experiments with the two rice *OsMADS3* isoforms revealed a clear difference in activity of these two proteins. The analysis of numerous Arabidopsis transformants revealed that *OsMADS3* complemented the *ag* mutant in a way that was comparable to the endogenous *AtAG* gene, whereas the *OsMADS3*^+*S*109^ encoded isoform lacked greatly this ability. Since both AtAG and OsMADS3 lack the serine residue at position 109, this result indicates that the absence of S109 facilitated the *ag* mutant complementation capability of these two proteins in Arabidopsis. Since S109 is located in the first α-helix of the K-box, a domain that is important for MADS-domain protein dimerization (Puranik et al., 2014), these results suggest that S109 influences the ability to interact with SEP-like proteins, because the interaction with these proteins has shown to be fundamental for AtAG to determine stamen and carpel identity in Arabidopsis (Pelaz et al., 2000).

The yeast 2-hybrid assays showed a role for residue S109 in the ability of the OsMADS3 isoforms to interact with Arabidopsis AtSEPs, since OsMADS3, OsMADS3^+*S*109^ and AtAG all interacted equally well with AtSEP3, but the two OsMADS3 isoforms had a different capability to interact with Arabidopsis AtSEP1. While OsMADS3 and AtAG were able to interact with this protein, no interaction was detected between OsMADS3^+*S*109^ and AtSEP1.

As observed by Puranik and co-workers (Puranik et al., 2014), the K-domains establish large dimerization interfaces, where the helix 1 and the neighboring loop are involved in such interactions. The insertion of a residue in position 109 is not likely to alter the overall dimer architecture, however, a local reorganization of the loop is needed to adjust the extra residue and this likely triggers a limited reorientation/rotation of helix 2 with respect to helix 1. This implies that MADS-box proteins devoid S109 may have distinct dimerization interfaces compared to the ones with S109. Based on these observations we propose that by slightly modifying the tetramer architecture, the presence/absence of S109 may modify the functional interactions of the OsMADS3 isoforms with their cellular partners in keeping with the Yeast 2-hybrid experiments with AtSEP1 and AtSEP3.

In conclusion, in their native background, OsMADS3^+*S*109^ and OsMADS3 could have diverse affinity to form different tetrameric complexes. Further studies in rice will be interesting to investigate the role of OsMADS3^+*S*109^ and OsMADS3, including the analysis of their crystal structures. Notably is that the evolutionary conservation of these splice variants in the grass family suggests that subtle changes in a MADS-domain transcription factor may lead to a divergence in function of these important regulators of carpel and stamen development.

## Data Availability Statement

All datasets generated and analyzed for this study are cited in the article/Supplementary Material.

## Author Contributions

LD discovered the alternative splicing and conceived the project and wrote the manuscript with contributions from all the authors. LD and AR performed the research. NG-S did expression analysis. FC and VG performed yeast interaction and SEM studies. SJ and GS helped in cloning and plant transformation. SR and RR did biochemical and structural analysis. MK supervised the whole project.

## Conflict of Interest

The authors declare that the research was conducted in the absence of any commercial or financial relationships that could be construed as a potential conflict of interest.

## Footnotes

1 http://www.theplantlist.org/

2 https://www.ncbi.nlm.nih.gov/genbank/
