# Supplementary material for: Functionally Divergent Splicing Variants of the Rice AGAMOUS Ortholog OsMADS3 Are Evolutionary Conserved in Grasses
Source: Front Plant Sci. 2020 May 25;11:637. doi: 10.3389/fpls.2020.00637 (PMC7261849; doi:10.3389/fpls.2020.00637)
Supplement: Supplementary file 1 [file Data_Sheet_1.PDF]

Supplementary Material

Supplementary Table 1. List of primers used.

Primer name	Sequence from 5' to 3'	Use
Osp17	TAATACGACTCACTATAGGGTGTGTGTACGTACGGTGTCTACAC	Both *OsMADS3* fw
Osp79	**TAATACGACTCACTATAGGG**TGGCACAATGGTGGCAAGGTC	Both *OsMADS3*
OsP119	CACCGAACACACCCCTCTCTTCC	*OsMADS58*
OsP120	CTGATCAGTCTCCTTGAAGAAGG	*OsMADS58*
OsP337	CACCAGTTAGCATACCCATCCATG	Both *OsMADS3*
Osp428	AATCCCCCACTATGGTCCTGT	*OsMADS3*$^{+S109}$ rev
Osp429	AATCCCCCACTATGGTCCTAC	*OsMADS3* rev
AtP650	GCTGTTAGTGGCAGAGATAGAG	*SHP1* qRT-PCR
AtP651	CCGGATTCGTAAACTGTCGTC	*SHP1* qRT-PCR
RT973	GGGATTCTATCAACACCATGAG	Both *OsMADS3* qRT-PCR
RT974	CTCAACTTCAGCATATAACAGC	Both *OsMADS3* qRT-PCR
RT975	CTGCTAAGCTGAAGCAACAG	*OsMADS58* qRT-PCR
RT 976	CTTCCAGCTGCTTAAGTTCTC	*OsMADS58* qRT-PCR
RT1672	AAGGTGCTTTGCTTGGTGAG	*REM22* qRT-PCR
RT1673	CACCAACCATCAAAGGGAAC	*REM22* qRT-PCR
RT1674	GACACTTGCTTCAAGAAGAAACGTTTGG	*SPL* qRT-PCR
RT1675	GAAGAAGATACTGATCGTAGCCGTTCAT	*SPL* qRT-PCR
RT1898	AACTTGGAAGGCAGATTAGAGAG	*AtAG* qRT-PCR
RT1899	GTGGCATAAGCTGCTCGTAG	*AtAG* qRT-PCR
SEP1f	**GGGGACAAGTTTGTACAAAAAAGCAGGCTtt**ATGGGAAGAGGAAGAGTAG	*AtSEP1* fw GW
SEP1r	**GGGGACCACTTTGTACAAGAAAGCTGGGTT**CAGAGCATCCACCCCGGGATG	*AtSEP1* rev GW
AtP_4929	**GGGGACAAGTTTGTACAAAAAAGCAGGCT**TCATGGGAAGAGGGAGAGTAG	*AtSEP3* fw GW
AtP_4930	**GGGGACCACTTTGTACAAGAAAGCTGGGT**CTCAAATAGAGTTGGTGTC	*AtSEP3* rev GW
Atp_7069	**GGGGACAAGTTTGTACAAAAAAGCAGGCTtt**atgGGGAGAGGAAAGATCGAAATC	*AtAG* fw GW
Atp_7070	**GGGGACCACTTTGTACAAGAAAGCTGGGT**TTACACTAACTGGAGAGCGGTTTG	*AtAG* rev GW
AtP3141	caccATGGGAAGAGGACGAGTGGAGCTGAAGCGGA	*AtSEP2 fw*
AtP3142	GATCTCATGGCAACCATGG	*AtSEP2 rev*
LD264	ATGGGGAGGGGGAAGATCGAG	Both *OsMADS3* fw
LD265	TTACTAATTGAAGGCCGGCTGCTG	Both *OsMADS3* rev

Supplementary Table 2. T1 lines obtained transforming *ag-3*/+ background plants with *35S::AtAG, 35S::OsMADS58, 35S::OsMADS3*$^{+S109}$ *and 35S::OsMADS3* constructs, respectively, and the resulting

phenotypes which are consistent with the reported *AG*-like genes overexpression phenotypes: dwarfism, curled rosette leaves, early flowering and, depending on the transgene, floral phenotypes.

**T0**	**Transgene**	**N° of T1 lines**	**Percentage of T1 lines with *AG* overexpression phenotypes**
ag-3/+	*35S::AtAG*	38	39,5%
ag-3/+	*35S::OsMADS58*	31	0%
ag-3/+	*35S::OsMADS3^{+S109}*	49	39%
ag-3/+	*35S::OsMADS3*	64	39%

Supplementary Table 3. Accession ID codes of the monocot *AG*-lineage genes included in this study.

Name	Species	MSU rice annotation	RAP rice annotation	Phytozome 13	Gramene / Ensembl Plants	NCBI GeneBank
OsMADS3	*Oryza sativa* ssp. *japonica*	LOC_Os01g10504.2	Os01t0201700-02 (3'UTR not complete)	LOC_Os01g10504.2	(+S109) (no S109)	XM_015777004 (+S109) XM_015777012 (no S109)
OsMADS58	*Oryza sativa* ssp. *japonica*	LOC_Os05g11414.1 (last exon and 3'UTR not correct)	Os05t0203800-02	LOC_Os05g11414.1 (last exon and 3'UTR not correct)		
ObMADS3	*Oryza brachyantha*				OB01G16550	
OlMADS3	*Oryza longistaminata*				KN538747.1_FGT006	
OmMADS3	*Oryza meridionalis*				OMERI01G06630	
OrMADS3	*Oryza rufipogon*				ORUFI01G07080	
OglaMADS3	*Oryza glaberrima*				ORGLA01G0061900	
OgluMADS3	*Oryza glumeapatula*				OGLUM01G07510	
OnMADS3	*Oryza nivara*				ONIVA01G08650	
LpMADS3	*Leersia perrieri*				LPERR01G06160	
LpMADS58	*Leersia perrieri*				LPERR05G05550	
BdAG1/MADS3	*Brachypodium distachyon*			Bradi2g06330.1	Bradi2g06330 KQK03211 (+S109) KQK03213 (no S109)	XM_010232289 (+S109) XM_010232291 (no S109)
BdMADS58	*Brachypodium distachyon*			Bradi2g32910.1	Bradi2g32910	
HvAG1/MADS3	*Hordeum vulgare*			HORVU3Hr1G026650.1	HORVU3Hr1G026650.1 (+S109) HORVU3Hr1G026650.2 (no S109)	
HvAG2/MADS58	*Hordeum vulgare*			HORVU1Hr1G029220.1	HORVU1Hr1G029220.1	

WAG-2	*Aegilops tauschii*				AET3Gv20300900	KT188778 (+S109) KT188779 (no S109)
TaMADS3-A	*Triticum aestivum*			Traes_3AS_55E9080C2	TraesCS3A02G314300	
TaMADS3-B	*Triticum aestivum*			Traes_3B_5C0E6A627	TraesCS3B02G157500	
TaMADS3-D	*Triticum aestivum*			Traes_3DS_C148D43AF	TraesCS3D02G140200	
SiAG1/MADS3	*Setaria italica*			Seita.5G143100.1		
SiMADS58	*Setaria italica*			Seita.3G073000.1		
SbAG1/MADS3	*Sorghum bicolor*			Sobic.003G027000.1		
SbMADS58	*Sorghum bicolor*			Sobic.009G075500		
ZMM2	*Zea mays*			Zm00001d008882_T001	Zm00001d008882_T001	
ZMM23	*Zea mays*			Zm00001d039434_T001	Zm00001d039434_T001	
ZAG1	*Zea mays*			Zm00001d037737_T001	Zm00001d037737_T001	
PeMADS3	*Phyllostachys edulis*					KJ002712.1
PeMADS58	*Phyllostachys edulis*					KJ002725.1
MaAG	*Musa acuminata*			GSMUA_Achr10T21480_001		
EgAG1	*Elaeis guineensis*					LOC105040564
AcAG	*Ananas comosus*			Aco009993.1		

Supplementary Figure 1. Distribution of the alternative splicing of *AG/OsMADS3*-like genes, within the monocot order of Poales (the families where we have found it are marked with a red asterisk), and its likely origin in the lineage of grasses (red star). The most recent whole genome duplication occurred in the grass evolution (ρ WGD), which caused the duplication of the *AG* lineage into the conserved *OsMADS3* and *OsMADS58* clades, is currently dated after the split of grasses from all its sister families (black star). The branching diagram showing the relationships between families, has been adapted from the Angiosperm Phylogeny Website, http://www.mobot.org/MOBOT/research/APweb/.

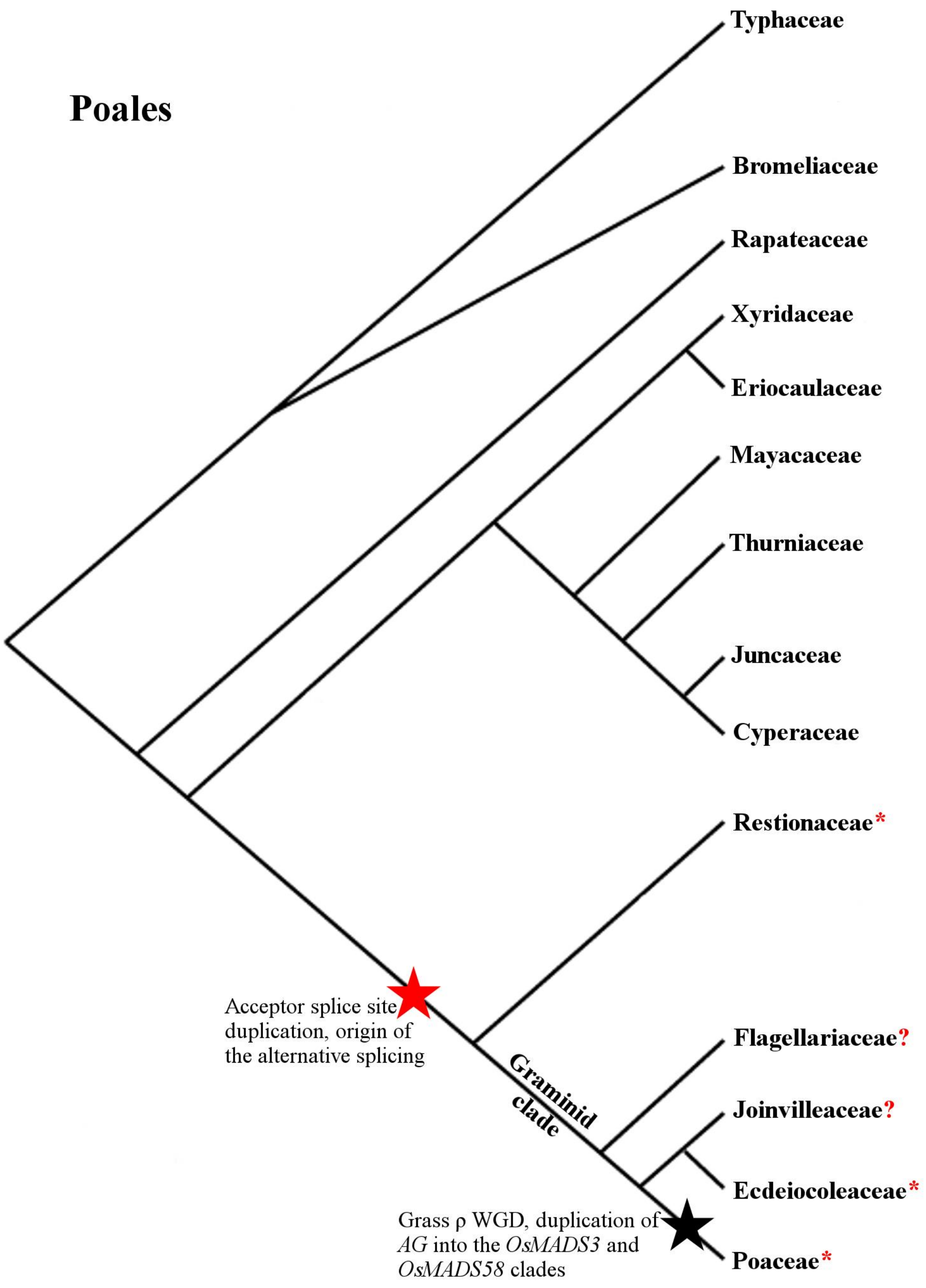

Supplementary Figure 2. Alignment of the region forming the 1st α-helix and the kink region in the two isoforms of OsMADS3 (top) and 100 other AGAMOUS subfamily proteins from non-grass monocots, Rosids, Asterids, basal angiosperms and gymnosperms. The partially conserved heptad repeats (abcdefg) of the predicted 1st α-helix, the S109 extra residue in OsMADS3^{+S109}, and the putative salt bridge contributing to the stabilization of the kink between the two α-helices, are indicated. The length and structure of this region are highly conserved, and isoforms with an additional amino acid residue are exclusive of OsMADS3-like proteins of grasses ad of closely related sister families.

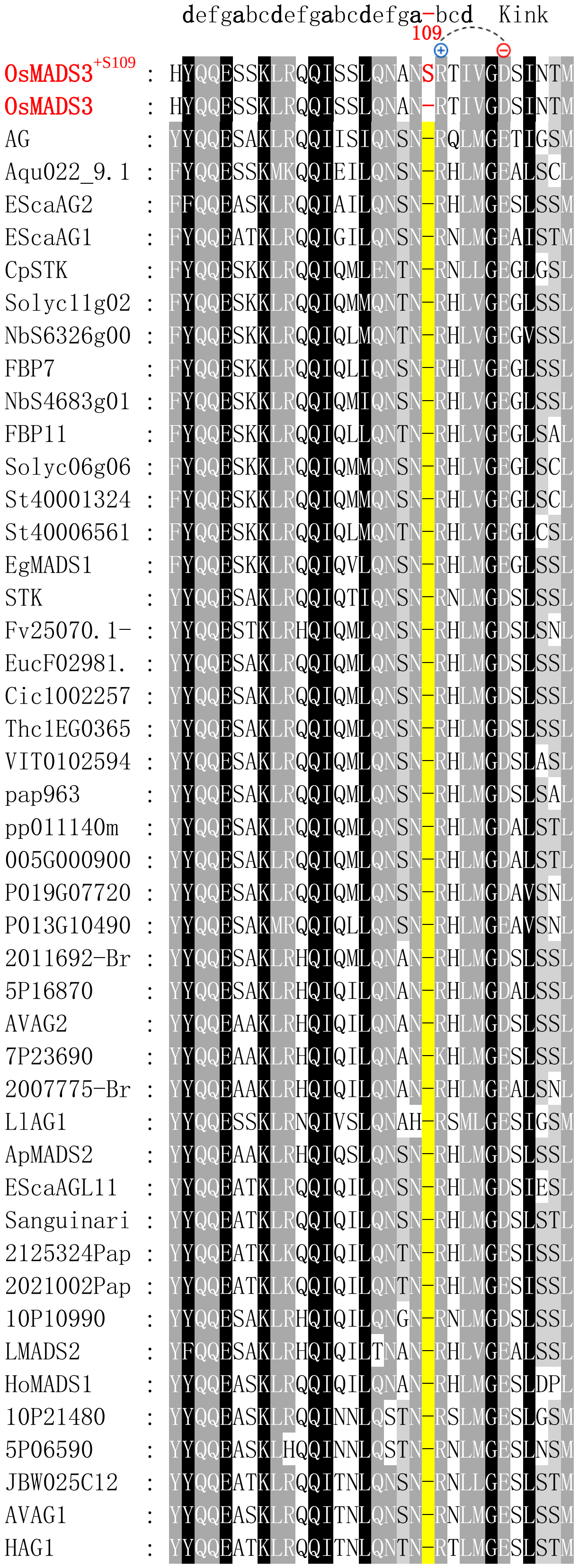

```
DcAG1       : YYLQEASKLRQQITNLQNSN-RNLMGEALSTM
Podocarpus  : YWQQEAAKLRQQIEILHNAN-RHLMGESLSNL
Stangeria   : YWQQEAGKLRQQIDIVQNAN-RHLMGDALTSL
002G243200  : FYQQEAAKLRVQISNLQNHN-RQMMGEALSNM
FARINELLI   : YYQQEASKLRAQISNLQNQN-RNMLGESLGAL
MoFAR       : YYQQEASKLRAQISNLQNQN-RNMLGESLGAL
St40007313  : YYQQEASKLRAQIGNLQNQN-RNMLGESLGSM
TfFAR       : YYQQEASKLRAQISNLQNHN-KNMLGEALGAL
pMADS3      : YYQQEASKLRAQIGNLQNQN-RNFLGESLAAL
Solyc02g07  : YYQQEASKLRAQIGNLMNQN-RNMMGEALAGM
pp010595m   : YYQQEAAKLRAQIGNLQNSS-RHMMGESLSSM
Thc1EG0295  : FYQQEAAKLRVQIGNLQNSN-RHMLGESLSAL
P011G07580  : FYQQEAAKLRSQIGNLQNSN-RNMLGESLSAL
P004G06430  : YYQQEAAKLRSQIGNLQNSN-RHMLGEALSSL
Cic1001642  : FYQQEAAKLRIQISNMQNSN-RNMLGESLSGL
006G169600  : FYQQEADKLRAQISNMQNNN-RQMMGESLGSM
DcAG2       : YYQQEAAKLRHQIQILHNTN-RHPMGEGLTSL
Aqu136_9.1  : FYQQEVTKLRNQIASLQNHN-RKLVGESLSNL
Aqu136_10.  : FYQQEATKLRNQIASLQNHN-RNLLGESLSNL
PLENA       : FYQQEANKLRRQIREIQTSN-RQMLGEGVSNM
MoPLE       : FYQQEANKLRRQIREIQTSN-RQMLGEGVGNM
TfPLE1      : FYQQEAAKLRRQIREIQNSN-RQILGEGVTSM
TfPLE2A     : FYQQEAAKFRRQIREIQKSN-RQILGEGVTGM
006G034400  : FYQQESSKLRRQIRDIQNLN-RHILGEALGSL
008G190600  : FYQQESSKLKRQIRDIQNLN-RHILGEALSSL
VIT0100080  : FYQQEASKLRRQIRDIQNLN-RHILGEALSSL
Thc1EG0018  : FYQQEASKLRRQIRDVQNMN-RHILGEALSTL
SHP2        : YYQQEASKLRRQIRDIQNLN-RHILGESLGSL
SHP1        : YYQQEASKLRRQIRDIQNSN-RHIVGESLGSL
Cic1003382  : FYQQEATKLRRQIREIQNLN-RHILGEALSTL
pp010578m   : FYQQESSKLRRQIREIQNSN-RHILGEALSTL
Fv24494.1-  : FYQQEASKLRRQIREIQNSN-RHILGEALSTL
pap50.73    : FYQQEATKLRRQIREIQNSN-RHILGEALGSL
FBP6        : YYQQEAAKLRRQIRDIQTYN-RQIVGEALSSL
St40004455  : YYQQEASKLRRQIRDIQTYN-RQIVGEALSSL
Solyc07g05  : YYQQEASKLRRQIRDIQTYN-RQIVGEALGSL
EucK01195.  : FYQQEASKLRRQIREIQVSN-RHILGEGISDL
peony       : FYQQESAKLRRQIREIQTSN-KQILGESLGVL
VIG0102130  : FYQQESSKLHQQIRNLQNSN-RHMLGESLGSL
AAT46102.1  : FYQQESLKLRQQIGNLQNLN-RHLMGEALGSM
2Ipomoea    : FYQQEANKLRQQISNLQNQN-RNYMGEGLGGF
10P14160    : HYQLESAKLRQQINNIQSTN-RNLMGEALSSM
6P14760     : HYQQESAKLQQQINHIQSTN-RSLMGEGLSSM
AAT46096.1  : YYQHEATKLRQQIQNLQIAN-RQLMGDSLSSL
EucE02863.  : FYQQESAKLQQQINNMQNNN-RQLVGDSIAGM
Cyperus     : YYEQEIAKLRHEIQALQIRN-SHYKGESLGNL
GGM3        : YWQQEAVKLKQQIDVLNNQI-RHYMGECLQSM
Pinus       : YWQQEAGKLRQQIDILQNAN-RHLMGDGLTAL
DAL2        : YWQQEAGKLRQQIEILQNAN-RHLMGDGLTAL
GBM5/GbMAD  : YWQQEAGKLRQQIDILQNAN-RHLMGDALTSL
Cycas       : YWQQEAGKLRQQIDILQNAN-RHLMGDALTSL
Taxus       : YWQQEAXKLRQQIENLENTN-RRLLGDGITNM
Juniperus   : YYQQEAARLRQQIDILINTN-DNLQGQGISDL
Cryptomeri  : FWQQEAAKLRQQIDILTNSN-GNLLGQGISDF
Thujopsis   : FWQQEAAKLRQQIDILTNSN-KNLLGQGISDL
```

Supplementary Figure 3. Phenotypes observed in T1 lines obtained in this study. **(A)** Example of T1 plants after selection, arrows indicate plants showing curly leaves phenotype. Example of **(B)** *35S::AtAG*, **(C)** *35S::OsMADS3*$^{+S109}$ and **(D)** *35S::OsMADS3* transformant showing dwarfism, curled rosette leaves, and early flowering.

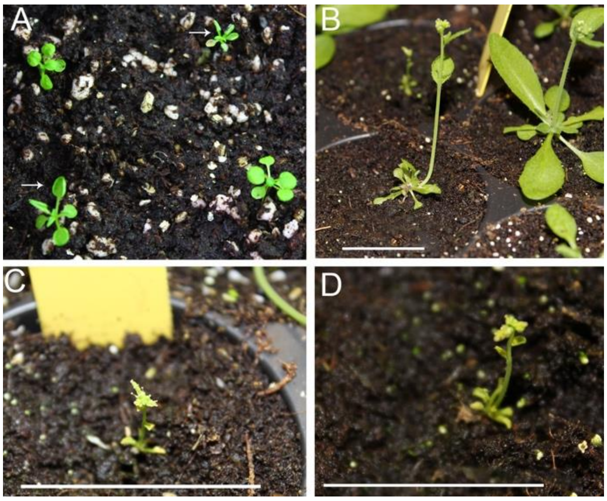

Supplementary Figure 4. Expression levels of *35S::AtAG*, *35S::OsMADS58*, *35S::OsMADS3*$^{+S109}$ and *35S::OsMADS3* in WT and *ag-3* background, compared to non-transgenic lines. For each transgene, two or more independent lines were used (indicated with #1, #2, etc.). **(A)** *AtAG* levels have been normalized against the WT level, where the endogenous *AtAG* is also detected; **(B)** *OsMADS58* levels have been normalized against the expression level of the transgene in the selected line *35S::OsMADS58 #2 ag-3*. **(C)** *OsMADS3* levels have been normalized against the expression level of the transgene in the selected line *35S::OsMADS3*$^{+S109}$ *#2 ag-3*. For each experiment, three biological replicates were used and for each of these three technical replicates were done. The expression of selected genes was normalized to that of *UBI* and *ACT8-2*. SEM is indicated.

A

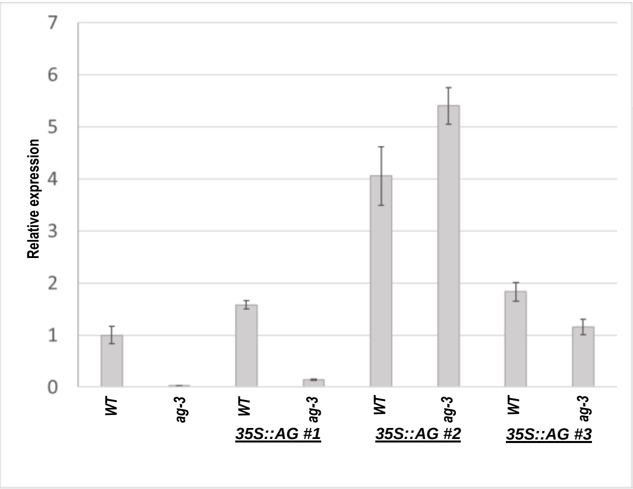

B

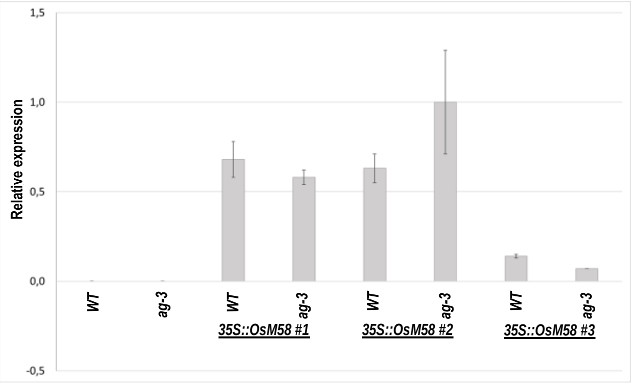

C

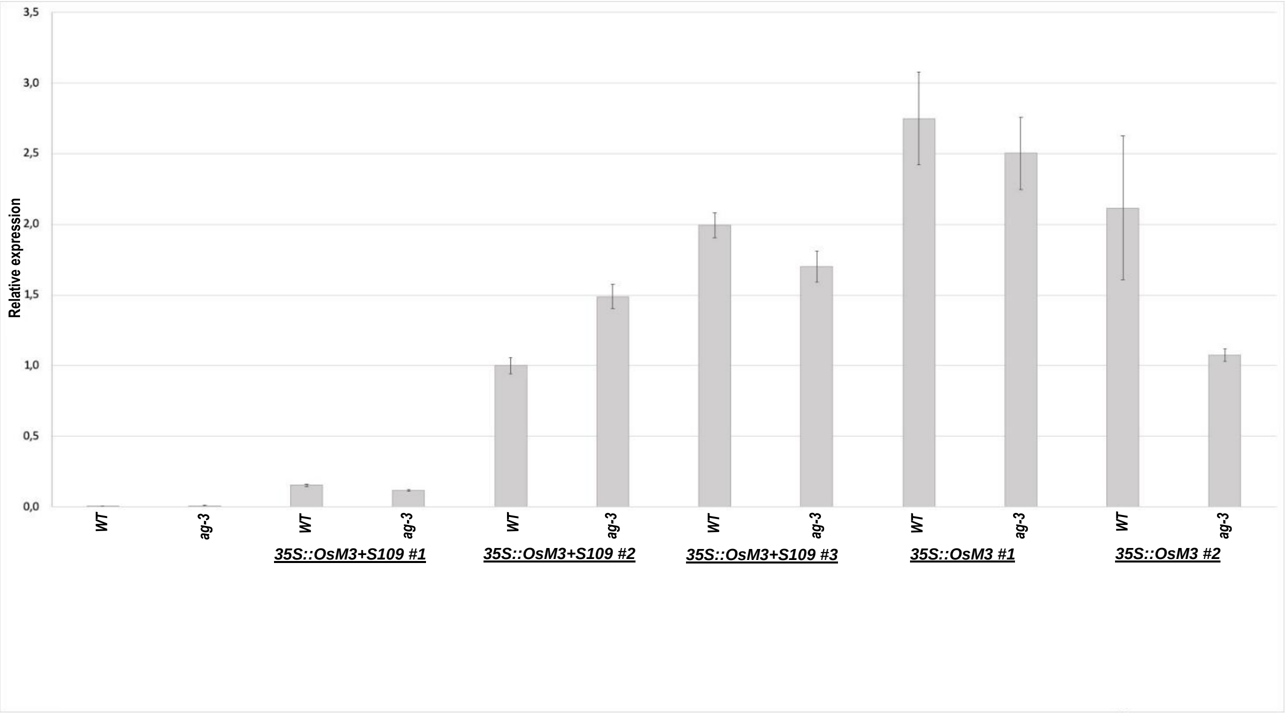

Supplementary Dataset 1. Complete ORFs and protein products of *OsMADS3*$^{+S109}$ and *OsMADS3* (putative N *terminus* included, in blue colour; S109 codon and amino acidic residue underlined in red colour).

OsMADS3^{+S109}

ATGATGAACATGATGACCGATCTGAGCTGCGGGCCATCGTCGATGACGGAGCTGACCGCGGCAGCGGCG
CCGGCTGGGTCAGGATCGTCGGCGGCGGTGGCGGCGGGGAGCAGCGAGAAGATGGGGAGGGGGAAGAT
CGAGATAAAGCGGATCGAGAACACGACGAACCGGCAGGTGACCTTCTGCAAGCGCCGCAATGGCCTCCT
GAAGAAGGCGTACGAGCTGTCCGTCCTCTGCGACGCCGAGGTTGCCCTCATCGTCTTCTCCAGCCGCGGC
CGCCTCTACGAGTACGCCAACAACAGTGTGAAATCCACCGTTGAGAGGTACAAGAAGGCAAACAGTGAC
ACCTCCAACTCTGGCACAGTTGCAGAAGTCAATGCCCAGCACTACCAGCAGGAGTCCTCCAAACTGCGCC
AACAAATCAGTAGCTTACAGAACGCAAACAGTAGGACCATAGTGGGGGATTCTATCAACACCATGAGCC
TCAGGGACCTTAAACAGGTAGAGAACAGGCTGGAGAAAGGCATAGCTAAGATAAGGGCTAGAAAGAAT
GAGCTGTTATATGCTGAAGTTGAGTACATGCAGAAAAGGGAAGTTGAGCTGCAGAATGACAACATGTAC
CTGAGGAGCAAGGTTGTTGAGAATGAGAGGGGACAGCAGCCACTGAACATGATGGGGGCAGCATCAACA
AGTGAATACGATCATATGGTTAATAACCCATATGATTCCAGGAACTTTCTTCAAGTGAACATCATGCAGC
AGCCTCAGCATTACGCCCATCAGCTGCAGCCAACTACCCTTCAACTCGGGCAGCAGCCGGCCTTCAATTA
G

OsMADS3^{+S109}

MMNMMTDLSCGPSSMTELTAAAAPAGSGSSAAVAAGSSEKMGRGKIEIKRIENTTNRQVTFCKRRNGLLKKA
YELSVLCDAEVALIVFSSRGRLYEYANNSVKSTVERYKKANSDTSNSGTVAEVNAQHYQQESSKLRQQISSLQ
NANSRTIVGDSINTMSLRDLKQVENRLEKGIAKIRARKNELLYAEVEYMQKREVELQNDNMYLRSKVVENER
GQQPLNMMGAASTSEYDHMVNNPYDSRNFLQVNIMQQPQHYAHQLQPTTLQLGQQPAFN

OsMADS3

ATGATGAACATGATGACCGATCTGAGCTGCGGGCCATCGTCGATGACGGAGCTGACCGCGGCAGCGGCG
CCGGCTGGGTCAGGATCGTCGGCGGCGGTGGCGGCGGGGAGCAGCGAGAAGATGGGGAGGGGGAAGAT
CGAGATAAAGCGGATCGAGAACACGACGAACCGGCAGGTGACCTTCTGCAAGCGCCGCAATGGCCTCCT
GAAGAAGGCGTACGAGCTGTCCGTCCTCTGCGACGCCGAGGTTGCCCTCATCGTCTTCTCCAGCCGCGGC
CGCCTCTACGAGTACGCCAACAACAGTGTGAAATCCACCGTTGAGAGGTACAAGAAGGCAAACAGTGAC
ACCTCCAACTCTGGCACAGTTGCAGAAGTCAATGCCCAGCACTACCAGCAGGAGTCCTCCAAACTGCGCC
AACAAATCAGTAGCTTACAGAACGCAAACAGGACCATAGTGGGGGATTCTATCAACACCATGAGCCTCA
GGGACCTTAAACAGGTAGAGAACAGGCTGGAGAAAGGCATAGCTAAGATAAGGGCTAGAAAGAATGAG
CTGTTATATGCTGAAGTTGAGTACATGCAGAAAAGGGAAGTTGAGCTGCAGAATGACAACATGTACCTGA
GGAGCAAGGTTGTTGAGAATGAGAGGGGACAGCAGCCACTGAACATGATGGGGGCAGCATCAACAAGTG
AATACGATCATATGGTTAATAACCCATATGATTCCAGGAACTTTCTTCAAGTGAACATCATGCAGCAGCCT
CAGCATTACGCCCATCAGCTGCAGCCAACTACCCTTCAACTCGGGCAGCAGCCGGCCTTCAATTAG

OsMADS3

MMNMMTDLSCGPSSMTELTAAAAPAGSGSSAAVAAGSSEKMGRGKIEIKRIENTTNRQVTFCKRRNGLLKKA
YELSVLCDAEVALIVFSSRGRLYEYANNSVKSTVERYKKANSDTSNSGTVAEVNAQHYQQESSKLRQQISSLQ
NANRTIVGDSINTMSLRDLKQVENRLEKGIAKIRARKNELLYAEVEYMQKREVELQNDNMYLRSKVVENERG
QQPLNMMGAASTSEYDHMVNNPYDSRNFLQVNIMQQPQHYAHQLQPTTLQLGQQPAFN

Supplementary Dataset 2. List of RNA-seq reads used, in Fasta format alignments.

SRX1332256

>control .

---tagcttacagaacgc
aaacagtaggaccatagtgggggattc---------------------------------

>gnl|SRA|SRR2638781.82119918.2 HWI-D00731:29:HGNNYADXX:1:2213:4627:21935.

----------cagcaggagtcctccaaactgcgccaacaaatcagtagcttacagaacgc
aaacagtaggaccatagtgggggattct--------------------------------
-atcaacaccatgagcctcagggaccttaaacagg---tagagagatcggaagagcg---
-----tcgtgtagggaaaga--gtgt----------------------------------

>gnl|SRA|SRR2638781.43464319.2 HWI-D00731:29:HGNNYADXX:1:2101:8560:7630.

-------------------------------agttgcagaagt----caatgc-------
ccagcactaccagcaggagtcctccaaactgcgccaacaaatcagtagcttacagaacgc
aaacagtaggaccatagtgggggattct--------------------------------
-atcaacaccatg------------------------------agatcggaagagcg---
-----tcgtgtagggaaaga--gtg-----------------------------------

>gnl|SRA|SRR2638781.25356923.2 HWI-D00731:29:HGNNYADXX:1:1203:11562:98140.

--------------------------------gttgcagaagt----caatgc-------
ccagcactaccagcaggagtcctccaaactgcgccaacaaatcagtagcttacagaacgc
aaacagtaggaccatagtgggggattct--------------------------------
-atcaacaccatg----------------------------agagatcggaagagcg---
-----tcgtgtagggaaaga--gt------------------------------------

>gnl|SRA|SRR2638781.17621657.2 HWI-D00731:29:HGNNYADXX:1:1114:9249:12221.

--------------------------------gttgcagaagt----caatgc-------
ccagcactaccagcaggagtcctccaaactgcgccaacaaatcagtagcttacagaacgc
aaacagtaggaccatagtgggggattct--------------------------------
-atcaacaccatgagcct----------------------cagagatcggaagagcg---
-----tcgtgtaggga--

>gnl|SRA|SRR2638781.12656820.2 HWI-D00731:29:HGNNYADXX:1:1110:14491:60877.

----------------------------------tgcagaagt----caatgc-------
ccagcactaccagcaggagtcctccaaactgcgccaacaaatcagtagcttacagaacgc
aaacagtaggaccatagtgggggattct--------------------------------
-atcaacaccatgagcctcag------------------ggacagatcggaagagcg---
-----tcgtgtagg--

>gnl|SRA|SRR2638781.11040750.2 HWI-D00731:29:HGNNYADXX:1:1109:20879:44378.

---gc-------
ccagcactaccagcaggagtcctccaaactgcgccaacaaatcagtagcttacagaacgc
aaacagtaggaccatagtgggggattct--------------------------------
-atcaacaccatgagcc--------------------------agatcggaagagcg---
-----tcgtgtagggaaaga--gtgtaga-----------------tctcggtg------

>gnl|SRA|SRR2638781.9615475.2 HWI-D00731:29:HGNNYADXX:1:1108:16844:40753.

---gc-------
ccagcactaccagcaggagtcctccaaactgcgccaacaaatcagtagcttacagaacgc
aaacagtaggaccatagtgggggattct--------------------------------
-atcaacaccatgagcc--------------------------agatcggaagagcg---
-----tcgtgtagggaaaga--gtgtaga-----------------tctcggtg------

>_R_gnl|SRA|SRR2638781.86839189.1 HWI-D00731:29:HGNNYADXX:1:2216:7339:40801.

--------------------------------gttgcagaagt----caatgc-------
ccagcactaccagcaggagtcctccaaactgcgccaacaaatcagtagcttacagaacgc
aaacagtaggaccatagtgggggattct--------------------------------
-atcaacaccatgagcctcagggaccttaaacaggtagagaacagg--------------

>gnl|SRA|SRR2638781.63170969.2 HWI-D00731:29:HGNNYADXX:1:2115:14888:25172.

--------------------------------gttgcagaagt----caatgc-------
ccagcactaccagcaggagtcctccaaactgcgccaacaaatcagtagcttacagaacgc
aaacagtaggaccatagtgggggattct--------------------------------
-atcaacaccatgagcctcagggaccttaaacaggtagagaacagg--------------

>gnl|SRA|SRR2638781.21702123.2 HWI-D00731:29:HGNNYADXX:1:1116:13221:93008.

--------------------------------gttgcagaagt----caatgc-------
ccagcactaccagcaggagtcctccaaactgcgccaacaaatcagtagcttacagaacgc
aaacagtaggaccatagtgggggattct--------------------------------
-atcaacaccatgagcctcagggaccttaaacaggtagagaacagg--------------

>_R_gnl|SRA|SRR2638781.21026351.1 HWI-D00731:29:HGNNYADXX:1:1116:5359:47145.

--------------------------------gttgcagaagt----caatgc-------
ccagcactaccagcaggagtcctccaaactgcgccaacaaatcagtagcttacagaacgc
aaacagtaggaccatagtgggggattct--------------------------------
-atcaacaccatgagcctcagggaccttaaacaggtagagaacagg--------------

>gnl|SRA|SRR2638781.13678178.2 HWI-D00731:29:HGNNYADXX:1:1111:8297:34277.

--------------------------------gttgcagaagt----caatgc-------

ccagcactaccagcaggagtcctccaaactgcgccaacaaatcagtagcttacagaacgc
aaacagtaggaccatagtgggggattct--------------------------------
-atcaacaccatgagcctcagggaccttaaacaggtagagaacagg--------------

>_R_gnl|SRA|SRR2638781.51197348.1 HWI-D00731:29:HGNNYADXX:1:2106:12713:92008.

-------------------------------agttgcagaagt----caatgc-------
ccagcactaccagcaggagtcctccaaactgcgccaacaaatcagtagcttacagaacgc
aaacagtaggaccatagtgggggattct--------------------------------
-atcaacaccatgagcctcagggaccttaaacaggtagagaacag---------------

>gnl|SRA|SRR2638781.79012821.2 HWI-D00731:29:HGNNYADXX:1:2211:10373:4515.

------------------------------cagttgcagaagt----caatgc-------
ccagcactaccagcaggagtcctccaaactgcgccaacaaatcagtagcttacagaacgc
aaacagtaggaccatagtgggggattct--------------------------------
-atcaacaccatgagcctcagggaccttaaacaggtagagaaca----------------

>gnl|SRA|SRR2638781.70511302.2 HWI-D00731:29:HGNNYADXX:1:2204:6266:77692.

------------------------------cagttgcagaagt----caatgc-------
ccagcactaccagcaggagtcctccaaactgcgccaacaaatcagtagcttacagaacgc
aaacagtaggaccatagtgggggattct--------------------------------
-atcaacaccatgagcctcagggaccttaaacaggtagagaaca----------------

>gnl|SRA|SRR2638781.54211580.2 HWI-D00731:29:HGNNYADXX:1:2109:15433:10503.

------------------------------cagttgcagaagt----caatgc-------
ccagcactaccagcaggagtcctccaaactgcgccaacaaatcagtagcttacagaacgc
aaacagtaggaccatagtgggggattct--------------------------------
-atcaacaccatgagcctcagggaccttaaacaggtagagaaca----------------

>_R_gnl|SRA|SRR2638781.2123982.1 HWI-D00731:29:HGNNYADXX:1:1102:14770:67032.

-----------------------------acagttgcagaagt----caatgc-------
ccagcactaccagcaggagtcctccaaactgcgccaacaaatcagtagcttacagaacgc
aaacagtaggaccatagtgggggattct--------------------------------
-atcaacaccatgagcctcagggaccttaaacaggtagagaac-----------------

>gnl|SRA|SRR2638781.56631455.2 HWI-D00731:29:HGNNYADXX:1:2110:10899:80483.

----------------------------cacagttgcagaagt----caatgc-------
ccagcactaccagcaggagtcctccaaactgcgccaacaaatcagtagcttacagaacgc
aaacagtaggaccatagtgggggattct--------------------------------
-atcaacaccatgagcctcagggaccttaaacaggtagagaa------------------

```
------------------------------------------------------------
------------------
>_R_gnl|SRA|SRR2638781.86803219.1 HWI-D00731:29:HGNNYADXX:1:2216:10077:38272.
------------------------------------------------------------
-----------------------------------gcagaagt----caatgc-------
ccagcactaccagcaggagtcctccaaactgcgccaacaaatcagtagcttacagaacgc
aaacagtaggaccatagtgggggattct--------------------------------
-atcaacaccatgagcctcagggaccttaaacaggtagagaacaggctg-----------
------------------------------------------------------------
------------------
>_R_gnl|SRA|SRR2638781.79693933.1 HWI-D00731:29:HGNNYADXX:1:2211:15476:52344.
------------------------------------------------------------
-----------------------------------gcagaagt----caatgc-------
ccagcactaccagcaggagtcctccaaactgcgccaacaaatcagtagcttacagaacgc
aaacagtaggaccatagtgggggattct--------------------------------
-atcaacaccatgagcctcagggaccttaaacaggtagagaacaggctg-----------
------------------------------------------------------------
------------------
>_R_gnl|SRA|SRR2638781.11983721.1 HWI-D00731:29:HGNNYADXX:1:1110:3571:12785.
------------------------------------------------------------
-----------------------------------gcagaagt----caatgc-------
ccagcactaccagcaggagtcctccaaactgcgccaacaaatcagtagcttacagaacgc
aaacagtaggaccatagtgggggattct--------------------------------
-atcaacaccatgagcctcagggaccttaaacaggtagagaacaggctg-----------
------------------------------------------------------------
------------------
>_R_gnl|SRA|SRR2638781.7171278.1 HWI-D00731:29:HGNNYADXX:1:1106:10873:57694.
------------------------------------------------------------
-----------------------------------gcagaagt----caatgc-------
ccagcactaccagcaggagtcctccaaactgcgccaacaaatcagtagcttacagaacgc
aaacagtaggaccatagtgggggattct--------------------------------
-atcaacaccatgagcctcagggaccttaaacaggtagagaacaggctg-----------
------------------------------------------------------------
------------------
>gnl|SRA|SRR2638781.85356639.2 HWI-D00731:29:HGNNYADXX:1:2215:10307:41604.
------------------------------------------------------------
----------------------------------tgcagaagt----caatgc-------
ccagcactaccggcaggagtcctccaaactgcgccaacaaatcagtagcttacagaacgc
aaacagtaggaccatagtgggggattct--------------------------------
-atcaacaccatgagcctcagggaccttaaacaggtagagaacaggct------------
------------------------------------------------------------
------------------
>gnl|SRA|SRR2638781.64251598.2 HWI-D00731:29:HGNNYADXX:1:2115:21109:95773.
------------------------------------------------------------
----------------------------------tgcagaagt----caatgc-------
ccagcactaccagcaggagtcctccaaactgcgccaacaaatcagtagcttacagaacgc
aaacagtaggaccatagtgggggattct--------------------------------
-atcaacaccatgagcctcagggaccttaaacaggtagagaacaggct------------
------------------------------------------------------------
------------------
>gnl|SRA|SRR2638781.52866280.2 HWI-D00731:29:HGNNYADXX:1:2108:8129:14589.
```

----------------------------------tgcagaagt----caatgc-------
ccagcactaccagcaggagtcctccaaactgcgccaacaaatcagtagcttacagaacgc
aaacagtaggaccatagtgggggattct--------------------------------
-atcaacaccatgagcctcagggaccgtaaacaggtagagaacaggct------------

>gnl|SRA|SRR2638781.82158614.2 HWI-D00731:29:HGNNYADXX:1:2213:17617:24397.

------------------------ctggcacagttgcagaagt----caatgc-------
ccagcactaccagcaggagtcctccaaactgcgccaacaaatcagtagcttacagaacgc
aaacagtaggaccatagtgggggattct--------------------------------
-atcaacaccatgagcctcagggaccttaaacaggtag----------------------

>gnl|SRA|SRR2638781.65224375.2 HWI-D00731:29:HGNNYADXX:1:2116:20834:60307.

------------------------ctggcacagttgcagaagt----caatgc-------
ccagcactaccagcaggagtcctccaaactgcgccaacaaatcagtagcttacagaacgc
aaacagtaggaccatagtgggggattct--------------------------------
-atcaacaccatgagcctcagggaccttaaacaggtag----------------------

>gnl|SRA|SRR2638781.50130875.2 HWI-D00731:29:HGNNYADXX:1:2106:10777:14907.

------------------------ctggcacagttgcagaagt----caatgc-------
ccagcactaccagcaggagtcctccaaactgcgccaacaaatcagtagcttacagaacgc
aaacagtaggaccatagtgggggattct--------------------------------
-atcaacaccatgagcctcagggaccttaaacaggtag----------------------

>_R_gnl|SRA|SRR2638781.27459705.1 HWI-D00731:29:HGNNYADXX:1:1205:10311:61177.

------------------------ctggcacagttgcagaagt----caatgc-------
ccagcactaccagcaggagtcctccaaactgcgccaacaaatcagtagcttacagaacgc
aaacagtaggaccatagtgggggattct--------------------------------
-atcaacaccatgagcctcagggaccttaaacaggtag----------------------

>_R_gnl|SRA|SRR2638781.10281006.1 HWI-D00731:29:HGNNYADXX:1:1108:6993:89133.

------------------------ctggcacagttgcagaagt----caatgc-------
ccagcactaccagcaggagtcctccaaactgcgccaacaaatcagtagcttacagaacgc
aaacagtaggaccatagtgggggattct--------------------------------
-atcaacaccatgagcctcagggaccttaaacaggtag----------------------

>_R_gnl|SRA|SRR2638781.47895815.1 HWI-D00731:29:HGNNYADXX:1:2104:5826:49882.

--------------------------ggcacagttgcagaagt----caatgc-------
ccagcactaccagcaggagtcctccaaactgcgccaacaaatcagtagcttacagaacgc

aaacagtaggaccatagtgggggattct--------------------------------
-atcaacaccatgagcctcagggaccttaaacaggtagag--------------------

>_R_gnl|SRA|SRR2638781.45851397.1 HWI-D00731:29:HGNNYADXX:1:2102:10276:90010.

--------------------------ggcacagttgcagaagt----caatgc-------
ccagcactaccagcaggagtcctccaaactgcgtcaacaaatcagtagcttacagaacgc
aaacagtaggaccatagtgggggattca--------------------------------
-atcaacaccatgagcctcagggaccttaaacaggtagag--------------------

>gnl|SRA|SRR2638781.71190002.2 HWI-D00731:29:HGNNYADXX:1:2205:6193:29658.

----------------------ctctggcacagttgcagaagt----caatgc-------
ccaccactaccagcaggagtcctccaaactgcgccaacaaatcagtagcttacagaacgc
aaacagtaggaccatagtgggggattct--------------------------------
-atcaacaccatgagcctcagggaccttaaacaggt------------------------

>_R_gnl|SRA|SRR2638781.47155884.1 HWI-D00731:29:HGNNYADXX:1:2103:10460:94334.

----------------------ctctggcacagttgcagaagt----caatgc-------
ccagcactaccagcaggagtcctccaaactgcgccaacaaatcagtagcttacagaacgc
aaacagtaggaccatagtgggggattct--------------------------------
-atcaacaccatgagcctcagggaccttaaacaggt------------------------

>_R_gnl|SRA|SRR2638781.21350717.1 HWI-D00731:29:HGNNYADXX:1:1116:17454:69589.

----------------------ctctggcacagttgcagaagt----caatgc-------
ccagcactaccagcaggagtcctccaaactgcgccaacaaatcagtagcttacagaacgc
aaacagtaggaccatagtgggggattct--------------------------------
-atcaacaccatgagcctcagggaccttaaacaggt------------------------

>_R_gnl|SRA|SRR2638781.45877415.1 HWI-D00731:29:HGNNYADXX:1:2102:5560:92204.

---------------------actctggcacagttgcagaagt----caatgc-------
ccagcactaccagcaggagtcctccaaactgcgccaacaaatcagtagcttacagaacgc
aaacagtaggaccatagtgggggattct--------------------------------
-atcaacaccatgagcctcagggaccttaaacagg-------------------------

>_R_gnl|SRA|SRR2638781.6671508.1 HWI-D00731:29:HGNNYADXX:1:1106:11103:20582.

---------------------actctggcacagttgcagaagt----caatgt-------
ccagcactaccagcaggagtcctccaaactgcgccaacaaatcagtagcttacagaacgc
aaacagtaggaccatagtgggggattct--------------------------------
-atcaacaccatgagcctcagggaccttaaacagg-------------------------

>gnl|SRA|SRR2638781.83349300.2 HWI-D00731:29:HGNNYADXX:1:2214:19927:6181.

----------------ctccaactctggcacagttgcagaagt----caatgc-------
ccagcactaccagcaggagtcctccaaactgcgccaacaaatcagtagcttacagaacgc
aaacagtaggaccatagtgggggattct--------------------------------
-atcaacaccatgagcctcagggaccttaa------------------------------

>gnl|SRA|SRR2638781.81002554.2 HWI-D00731:29:HGNNYADXX:1:2212:3186:44297.

----------------ctccaactctggcacagttgcagaagt----caatgc-------
ccagcactaccagcaggagtcctccaaactgcgccaacaaatcagtagcttacagaacgc
aaacagtaggaccatagtgggggattct--------------------------------
-atcaacaccatgagcctcagggaccttaa------------------------------

>gnl|SRA|SRR2638781.58575663.2 HWI-D00731:29:HGNNYADXX:1:2112:19178:15451.

----------------ctccaactctggcacagttgcagaagt----caatgc-------
ccagcactaccagcaggagtcctccaaactgcgccaacaaatcagtagcttacagaacgc
aaacagtaggaccatagtgggggattct--------------------------------
-atcaacaccatgagcctcagggaccttaa------------------------------

>gnl|SRA|SRR2638781.29959722.2 HWI-D00731:29:HGNNYADXX:1:1207:10388:51386.

----------------ctccaactctggcacagttgcagaagt----caatgc-------
ccagcactaccagcaggagtcctccaaactgcgccaacaaatcagtagcttacagaacgc
aaacagtaggaccatagtgggggattct--------------------------------
-atcaacaccatgagcctcagggaccttaa------------------------------

>gnl|SRA|SRR2638781.25061578.2 HWI-D00731:29:HGNNYADXX:1:1203:14756:74865.

----------------ctccaactctggcacagttgcagaagt----caatgc-------
ccagcactaccagcaggagtcctccaaactgcgccaacaaatcagtagcttacagaacgc
aaacagtaggaccatagtgggggattct--------------------------------
-atcaacaccatgagcctcagggaccttaa------------------------------

>gnl|SRA|SRR2638781.15109636.2 HWI-D00731:29:HGNNYADXX:1:1112:18463:35992.

----------------ctccaactctggcacagttgcagaagt----caatgc-------
ccagcactaccagcaggagtcctccaaactgcgccaacaaatcagtagcttacagaacgc
aaacagtaggaccatagtgggggattct--------------------------------
-atcaacaccatgagcctcagggaccttaa------------------------------

>gnl|SRA|SRR2638781.14613459.2 HWI-D00731:29:HGNNYADXX:1:1111:8619:100478.

----------------ctccaactctggcacagttgcagaagt----caatgc-------
ccagcactaccagcaggagtcctccaaactgcgccaacaaatcagtagcttacagaacgc
aaacagtaggaccatagtgggggattct--------------------------------
-atcaacaccatgagcctcagggaccttaa------------------------------

>gnl|SRA|SRR2638781.13505416.2 HWI-D00731:29:HGNNYADXX:1:1111:6204:22087.

----------------ctccaactctggcacagttgcagaagt----caatgc-------
ccagcactaccagcaggagtcctccaaactgcgccaacaaatcagtagcttacagaacgc
aaacagtaggaccatagtgggggattct--------------------------------
-atcaacaccatgagcctcagggaccttaa------------------------------

>gnl|SRA|SRR2638781.3730889.2 HWI-D00731:29:HGNNYADXX:1:1103:4451:98525.

----------------ctccaactctggcacagttgcagaagt----caatgc-------
ccagcactaccagcaggagtcctccaaactgcgccaacaaatcagtagcttacagaacgc
aaacagtaggaccatagtgggggattct--------------------------------
-atcaacaccatgagcctcagggaccttaa------------------------------

>gnl|SRA|SRR2638781.64030891.2 HWI-D00731:29:HGNNYADXX:1:2115:8728:81597.

----------------ctccaactctggcacagttgcagaagt----caatgc-------
ccagcactaccagcaggagtcctccaaactgcgccaacaaatcagtagcttacataacgc
aaacagtaggaccatagtgggggattct--------------------------------
-atcaacaccatgagcctcagggaccttaa------------------------------

>_R_gnl|SRA|SRR2638781.75822938.1 HWI-D00731:29:HGNNYADXX:1:2208:14788:74974.

------------------ccaactctggcacagttgcagaagt----caatgc-------
ccagcactaccagcaggagtcctccaaactgcgccaacaaatcagtagcttacagaacgc
aaacagtaggaccatagtgggggattct--------------------------------
-atcaacaccatgagcctcagggaccttaaac----------------------------

>gnl|SRA|SRR2638781.45523948.2 HWI-D00731:29:HGNNYADXX:1:2102:6821:65442.

-------------------caactctggcacagttgcagaagt----caatgc-------
ccagcactaccagcaggagtcctccaaactgcgccaacaaatcagtagcttacagaacgc
aaacagtaggaccatagtgggggattct--------------------------------
-atcaacaccatgagcctcagggaccttaaaca---------------------------

>gnl|SRA|SRR2638781.25865011.2 HWI-D00731:29:HGNNYADXX:1:1204:4155:38267.

-------------------caactctggcacagttgcagaagt----caatgc-------
ccagcactaccagcaggagtcctccaaactgcgccaacaaatcagtagcttacagaacgc
aaacagtaggaccatagtgggggattct--------------------------------

-atcaacaccatgagcctcagggaccttaaaca---------------------------

>gnl|SRA|SRR2638781.25111838.2 HWI-D00731:29:HGNNYADXX:1:1203:19733:78771.

-----------------tccaactctggcacagttgcagaagt----caatgc-------
ccagcactaccagcaggagtcctccaaactgcgccaacaaatcagtagcttacagaacgc
aaacagtaggaccatagtgggggattct--------------------------------
-atcaacaccatgagcctcagggaccttaaa----------------------------

>gnl|SRA|SRR2638781.22447396.2 HWI-D00731:29:HGNNYADXX:1:1201:3920:52119.

-----------------tccaactctggcacagttgcagaagt----caatgc-------
ccagcactaccagcaggagtcctccaaactgcgccaacaaatcagtagcttacagaacgc
aaacagtaggaccatagtgggggattct--------------------------------
-atcaacaccatgagcctcagggaccttaaa----------------------------

>gnl|SRA|SRR2638781.13113875.2 HWI-D00731:29:HGNNYADXX:1:1110:1650:93712.

-----------------tccaactctggcacagttgcagaagt----caatgc-------
ccagcactaccagcaggagtcctccaaactgcgccaacaaatcagtagcttacagaacgc
aaacagtaggaccatagtgggggattct--------------------------------
-atcaacaccatgagcctcagggaccttaaa----------------------------

>gnl|SRA|SRR2638781.19274266.2 HWI-D00731:29:HGNNYADXX:1:1115:6710:26604.

-----------------tccaactctggcacagttgcagaagt----caatgc-------
ccagcactaccagcaggagtcctccaaactgcgccaacaaatcagtagcttacagaacgc
aaacagtaggaccatagtgggggattct--------------------------------
-atcaaca-catgagcctcagggaccttaaac---------------------------

>_R_gnl|SRA|SRR2638781.82794303.1 HWI-D00731:29:HGNNYADXX:1:2213:15787:67851.

-----------gacacctccaactctggcacagttgcagaagt----caatgc-------
ccagcactaccagcaggagtcctccaaactgcgccaacaaatcagtagcttacagaacgc
aaacagtaggaccatagtgggggattct--------------------------------
-atcaacaccatgagcctcagggac-----------------------------------

>_R_gnl|SRA|SRR2638781.64519189.1 HWI-D00731:29:HGNNYADXX:1:2116:7397:14062.

-----------gacacctccaactctggcacagttgcagaagt----caatgc-------
ccagcactaccagcaggagtcctccaaactgcgccaacaaatcagtagcttacagaacgc
aaacagtaggaccatagtgggggattct--------------------------------
-atcaacaccatgagcctcagggac-----------------------------------

>_R_gnl|SRA|SRR2638781.58986223.1 HWI-D00731:29:HGNNYADXX:1:2112:14592:43414.

-----------gacacctccaactctggcacagttgcagaagt----caatgc-------
ccagcactaccagcaggagtcctccaaactgcgccaacaaatcagtagcttacagaacgc
aaacagtaggaccatagtgggggattct--------------------------------
-atcaacaccatgagcctcagggac-----------------------------------

>_R_gnl|SRA|SRR2638781.8488896.1 HWI-D00731:29:HGNNYADXX:1:1107:10931:57373.

-----------gacacctccaactctggcacagttgcagaagt----caatgc-------
ccagcactaccagcaggagtcctccaaactgcgccaacaaatcagtagcttacagaacgc
aaacagtaggaccatagtgggggattct--------------------------------
-atcaacaccatgagcctcagggac-----------------------------------

>_R_gnl|SRA|SRR2638781.80077344.1 HWI-D00731:29:HGNNYADXX:1:2211:5999:79458.

-----------gacgcctccaactctggcacagttgcagaagt----caatgc-------
ccagcactaccagcaggagtcctccaaactgcgccaacaaatcagtagcttacagaacgc
aaacagtaggaccatagtgggggattct--------------------------------
-atcaacaccatgagcctcagggac-----------------------------------

>_R_gnl|SRA|SRR2638781.73927226.1 HWI-D00731:29:HGNNYADXX:1:2207:4167:35157.

------------acacctccaactctggcacagttgcagaagt----caatgc-------
ccagcactaccagcaggagtcctccaaactgcgccaacaaatcagtagcttacagaacgc
aaacagtaggaccatagtgggggattct--------------------------------
-atcaacaccatgagcctcagggacc----------------------------------

>gnl|SRA|SRR2638781.27564969.2 HWI-D00731:29:HGNNYADXX:1:1205:11556:69170.

------------acacctccaactctggcacagttgcagaagt----caatgc-------
ccagcactaccagcaggagtcctccaaactgcgccaacaaatcagtagcttacagaacgc
aaacagtaggaccatagtgggggattct--------------------------------
-atcaacaccatgagcctcagggacc----------------------------------

>_R_gnl|SRA|SRR2638781.48449793.1 HWI-D00731:29:HGNNYADXX:1:2104:13598:90626.

------------acacctccaactctggcacagttgcagaagt----caatgc-------
ccagcactaccagcaggagtcctccaaactgcgccaacaaatcagtagcttacagaacgc
aaacagtaggaccatagtgggggattct--------------------------------
-atcaacaccatgagcctcagggacg----------------------------------

>_R_gnl|SRA|SRR2638781.16065904.1 HWI-D00731:29:HGNNYADXX:1:1113:17111:3585.

--------------acctccaactctggcacagttgcagaagt----caatgc-------

ccagcactaccagcaggagtcctccaaactgcgccaacaaatcagtagcttacagaacgc
aaacagtaggaccatagtgggggattct--------------------------------
-atcaacaccatgagcctcagggacctt--------------------------------

>_R_gnl|SRA|SRR2638781.66301328.1 HWI-D00731:29:HGNNYADXX:1:2201:19607:36906.

--------agtgacacctccaactctggcacagttgcagaagt----caatgc-------
ccagcactaccagcaggagtcctccaaactgcgccaacaaatcagtagcttacagaacgc
aaacagtaggaccatagtgggggattct--------------------------------
-atcaacaccatgagcctcagg--------------------------------------

>gnl|SRA|SRR2638781.49756360.2 HWI-D00731:29:HGNNYADXX:1:2105:19997:86851.

--------agtgacacctccaactctggcacagttgcagaagt----caatgc-------
ccagcactaccagcaggagtcctccaaactgcgccaacaaatcagtagcttacagaacgc
aaacagtaggaccatagtgggggattct--------------------------------
-atcaacaccatgagcctcagg--------------------------------------

>_R_gnl|SRA|SRR2638781.43264402.1 HWI-D00731:29:HGNNYADXX:1:1216:1891:92921.

--------agtgacacctccaactctggcacagttgcagaagt----caatgc-------
ccagcactaccagcaggagtcctccaaactgcgccaacaaatcagtagcttacagaacgc
aaacagtaggaccatagtgggggattct--------------------------------
-atcaacaccatgagcctcagg--------------------------------------

>_R_gnl|SRA|SRR2638781.29684992.1 HWI-D00731:29:HGNNYADXX:1:1207:1328:30399.

--------agtgacacctccaactctggcacagttgcagaagt----caatgc-------
ccagcactaccagcaggagtcctccaaactgcgccaacaaatcagtagcttacagaacgc
aaacagtaggaccatagtgggggattct--------------------------------
-atcaacaccatgagcctcagg--------------------------------------

>gnl|SRA|SRR2638781.26464312.2 HWI-D00731:29:HGNNYADXX:1:1204:15198:84342.

--------agtgacacctccaactctggcacagttgcagaagt----caatgc-------
ccagcactaccagcaggagtcctccaaactgcgccaacaaatcagtagcttacagaacgc
aaacagtaggaccatagtgggggattct--------------------------------
-atcaacaccatgagcctcagg--------------------------------------

>_R_gnl|SRA|SRR2638781.4111215.1 HWI-D00731:29:HGNNYADXX:1:1104:16000:27879.

--------agtgacacctccaactctggcacagttgcagaagt----caatgc-------
ccagcactaccagcaggagtcctccaaactgcgccaacaaatcagtagcttacagaacgc
aaacagtaggaccatagtgggggattct--------------------------------
-atcaacaccatgagcctcagg--------------------------------------

>_R_gnl|SRA|SRR2638781.25973134.1 HWI-D00731:29:HGNNYADXX:1:1204:11635:46527.

-------cagtgacacctccaactctggcacagttgcagaagt----caatgc-------
ccagcactaccagcaggagtcctccaaactgcgccaacaaatcagtagcttacagaacgc
aaacagtaggaccatagtgggggattct--------------------------------
-atcaacaccatgagcctcag--

>_R_gnl|SRA|SRR2638781.24905799.1 HWI-D00731:29:HGNNYADXX:1:1203:14592:59410.

-------cagtgacacctccaactctggcacagttgcagaagt----caatgc-------
ccagcactaccagcaggagtcctccaaactgcgccaacaaatcagtagcttacagaacgc
aaacagtaggaccatagtgggggattct--------------------------------
-atcaacaccatgagcctcag--

>_R_gnl|SRA|SRR2638781.39644448.1 HWI-D00731:29:HGNNYADXX:1:1214:11251:47485.

---------gtgacacctccaactctggcacagttgcagaagt----caatgc-------
ccagcactaccagcaggagtcctccaaactgcgccaacaaatcagtagcttacagaacgc
aaacagtaggaccatagtgggggattct--------------------------------
-atcaacaccatgagcctcaggg--------------------------------------
----

>gnl|SRA|SRR2638781.31472563.2 HWI-D00731:29:HGNNYADXX:1:1208:8728:64584.

---------gtgacacctccaactctggcacagttgcagaagt----caatgc-------
ccagcactaccagcaggagtcctccaaactgcgccaacaaatcagtagcttacagaacgc
aaacagtaggaccatagtgggggattct--------------------------------
-atcaacaccatgagcctcaggg--------------------------------------
----

>_R_gnl|SRA|SRR2638781.6544257.1 HWI-D00731:29:HGNNYADXX:1:1106:16896:11239.

---------gtgacacctccaactctggcacagttgcagaagt----caatgc-------
ccagcactaccagcaggagtcctccaaactgcgccaacaaatcagtagcttacagaacgc
aaacagtaggaccatagtgggggattct--------------------------------
-atcaacaccatgagcctcaggg--------------------------------------
----

>_R_gnl|SRA|SRR2638781.2793789.1 HWI-D00731:29:HGNNYADXX:1:1103:19456:18761.

---------gtgacacctccaactctggcacagttgcagaagt----caatac-------
ccagcactaccagcaggagtcctccaaactgcgccaacaaatcagtagcttacagaacgc
aaacagtaggaccatagtgggggattct--------------------------------
-atcaacaccatgagcctcaggg--------------------------------------
----

>gnl|SRA|SRR2638781.87350985.2 HWI-D00731:29:HGNNYADXX:1:2216:4497:76037.

----------------ctccaactctggcacagttgcagaagt----caatgc-------
ccagcactaccagcaggagtcctccaaactgcgccaacaaatcagtagcttacagaacgc
aaac---aggaccatagtgggggattct--------------------------------
-atcaacaccatgagcctcagggaccttaaaca---------------------------

>gnl|SRA|SRR2638781.80419452.2 HWI-D00731:29:HGNNYADXX:1:2212:11863:3934.

----------------ctccaactctggcacagttgcagaagt----caatgc-------
ccagcactaccagcaggagtcctccaaactgcgccaacaaatcagtagcttacagaacgc
aaac---aggaccatagtgggggattct--------------------------------
-atcaacaccatgagcctcagggaccttaaaca---------------------------

>gnl|SRA|SRR2638781.66979479.2 HWI-D00731:29:HGNNYADXX:1:2201:3769:90406.

----------------ctccaactctggcacagttgcagaagt----caatgc-------
ccagcactaccagcaggagtcctccaaactgcgccaacaaatcagtagcttacagaacgc
aaac---aggaccatagtgggggattct--------------------------------
-atcaacaccatgagcctcagggaccttaaaca---------------------------

>gnl|SRA|SRR2638781.61143440.2 HWI-D00731:29:HGNNYADXX:1:2113:20559:89590.

----------------ctccaactctggcacagttgcagaagt----caatgc-------
ccagcactaccagcaggagtcctccaaactgcgccaacaaatcagtagcttacagaacgc
aaac---aggaccatagtgggggattct--------------------------------
-atcaacaccatgagcctcagggaccttaaaca---------------------------

>gnl|SRA|SRR2638781.32581926.2 HWI-D00731:29:HGNNYADXX:1:1209:15571:46018.

----------------ctccaactctggcacagttgcagaagt----caatgc-------
ccagcactaccagcaggagtcctccaaactgcgccaacaaatcagtagcttacagaacgc
aaac---aggaccatagtgggggattct--------------------------------
-atcaacaccatgagcctcagggaccttaaaca---------------------------

>gnl|SRA|SRR2638781.56840925.2 HWI-D00731:29:HGNNYADXX:1:2110:3571:94945.

----------------------ctctggcacagttgcagaagt----caatgc-------
ccagcactaccagcaggagtcctccaaactgcgccaacaaatcagtagcttacagaacgc
aaac---aggaccatagtgggggattct--------------------------------
-atcaacaccatgagcctcagggaccttaaacaggtaga---------------------

>gnl|SRA|SRR2638781.56705257.2 HWI-D00731:29:HGNNYADXX:1:2110:17340:85356.

----------------------ctctggcacagttgcagaagt----caatgc-------
ccagcactaccagcaggagtcctccaaactgcgccaacaaatcagtagcttacagaacgc

aaac---aggaccatagtgggggattct--------------------------------
-atcaacaccatgagcctcagggaccttaaacaggtaga---------------------

>gnl|SRA|SRR2638781.3808453.2 HWI-D00731:29:HGNNYADXX:1:1104:11126:5189.

----------------------ctctggaacagctgcagaagg----caaagc-------
ccagcactaccagcaggagtcctccaaactgcgccaacaaatcagtagcttacagaacgc
aaac---aggaccatagtgggggattct--------------------------------
-atcaacaccatgagcctcagggaccttaaacaggtaga---------------------

>_R_gnl|SRA|SRR2638781.51709033.1 HWI-D00731:29:HGNNYADXX:1:2107:16650:29915.

--------------------aactctggcacagttgcagaagt----caatgc-------
ccagcactaccagcaggagtcctccaaactgcgccaacaaatcagtagcttacagaacgc
aaac---aggaccatagtgggggattct--------------------------------
-atcaacaccatgagcctcagggaccttaaacaggtg-----------------------

>_R_gnl|SRA|SRR2638781.72683716.1 HWI-D00731:29:HGNNYADXX:1:2206:8494:42154.

--t----caatgc-------
ccagcactaccagcaggagtcctccaaactgcgccaacaaatcagtagcttacagaacgc
aaacagtaggaccatagtgggggattct--------------------------------
-atcaacaccatgagcctcagggaccttaaacaggtagagaacaggctggagaaag----

>_R_gnl|SRA|SRR2638781.62683417.1 HWI-D00731:29:HGNNYADXX:1:2114:16175:92304.

--t----caatgc-------
ccagcactaccagcaggagtcctccaaactgcgccaacaaatcagtagcttacagaacgc
aaacagtaggaccatagtgggggattct--------------------------------
-atcaacaccatgagcctcagggaccttaaacaggtagagaacaggctggagaaag----

>gnl|SRA|SRR2638781.58436708.2 HWI-D00731:29:HGNNYADXX:1:2112:17610:5968.

---gt----caatgc-------
ccagcactaccagcaggagtcctccaaactgcgccaacaaatcagtagcttacagaacgc
aaacagtaggaccatagtgggggattct--------------------------------
-atcaacaccatgagcctcagggaccttaaacaggtagagaacaggctggagaga-----

>gnl|SRA|SRR2638781.57637567.2 HWI-D00731:29:HGNNYADXX:1:2111:16464:50424.

---gt----caatgc-------
ccagcactaccagcaggagtcctccaaactgcgccaacaaatcagtagcttacagaacgc
aaacagtaggaccatagtgggggattct--------------------------------
-atcaacaccatgagcctcagggaccttaaacaggtagagaacaggctggagaaa-----

>gnl|SRA|SRR2638781.20477557.2 HWI-D00731:29:HGNNYADXX:1:1116:2622:10143.

---gt----caatgc-------
ccagcactaccagcaggagtcctccaaactgcgccaacaaatcagtagcttacagaacgc
aaacagtaggaccatagtgggggattct--------------------------------
-atcaacaccatgagcctcagggaccttaaacaggtagagaacaggctggagaaa-----

>gnl|SRA|SRR2638781.45893357.2 HWI-D00731:29:HGNNYADXX:1:2102:19297:93245.

--agt----caatgc-------
ccagcactaccagcaggagtcctccaaactgcgccaacaaatcagtagcttacagaacgc
aaacagtaggaccatagtgggggattct--------------------------------
-atcaacaccatgagcctcagggaccttaaacaggtagagaacaggctggagaa------

>gnl|SRA|SRR2638781.25769483.2 HWI-D00731:29:HGNNYADXX:1:1204:9615:30764.

--agt----caatgc-------
ccagcactaccagcaggagtcctccaaactgcgccaacaaatcagtagcttacagaacgc
aaacagtaggaccatagtgggggattct--------------------------------
-atcaacaccatgagcctcagggaccttaaacaggtagagaacaggctggagaa------

>gnl|SRA|SRR2638781.1383772.2 HWI-D00731:29:HGNNYADXX:1:1102:2644:10100.

--agt----caatgc-------
ccagcactaccagcaggagtcctccaaactgcgccaacaaatcagtagcttacagaacgc
aaacagtaagaccatagtgggggattct--------------------------------
-atcaacaccatgagcctcagggaccttaaacaggtagagaacaggctggagag------

>_R_gnl|SRA|SRR2638781.66177437.1 HWI-D00731:29:HGNNYADXX:1:2201:13642:27245.

---ctcttc-------
cgatctctaccagcaggagtcctccaaactgcgccaacaaatcagtagcttacagaacgc
aaacagtaggaccatagtgggggattct--------------------------------
-atcaacaccatgagcctcagggaccttaaacaggtagagaacaggctggagaaagg---

>_R_gnl|SRA|SRR2638781.53687691.1 HWI-D00731:29:HGNNYADXX:1:2108:6949:73205.

---caatgc-------
ccagcactaccagcaggagtcctccaaactgcgccaacaaatcagtagcttacagaacgc
aaacagtaggaccatagtgggggattct--------------------------------
-atcaacaccatgagcctcagggaccttaaacaggtagagaacaggctggagaaagg---

>_R_gnl|SRA|SRR2638781.32049489.1 HWI-D00731:29:HGNNYADXX:1:1209:12824:7383.

---caatgc-------
ccagcactaccagcaggagtcctccaaactgcgccaacaaatcagtagcttacagaacgc
aaacagtaggaccatagtgggggattct--------------------------------
-atcaacaccatgagcctcagggaccttaaacaggtagagaacaggctggagaaagg---

>_R_gnl|SRA|SRR2638781.63786640.1 HWI-D00731:29:HGNNYADXX:1:2115:16618:65267.

--aatgc-------
ccagcactaccagcaggagtcctccaaactgcgccaacaaatcagtagcttacagaacgc
aaacagtaggaccatagtgggggattct--------------------------------
-atcaacaccatgagcctcagggaccttaaacaggtagagaacaggctggagaaagg---
-----c--

>_R_gnl|SRA|SRR2638781.60140639.1 HWI-D00731:29:HGNNYADXX:1:2113:6098:22361.

--aatgc-------
ccagcactaccagcaggagtcctccaaactgcgccaacaaatcagtagcttacagaacgc
aaacagtaggaccatagtgggggattct--------------------------------
-atcaacaccatgagcctcagggaccttaaacaggtagagaacaggctggagaaagg---
-----c--

>gnl|SRA|SRR2638781.33108575.2 HWI-D00731:29:HGNNYADXX:1:1209:9212:84468.

--aatgc-------
ccagcactaccagcaggagtcctccaaactgcgccaacaaatcagtagcttacagaacgc
aaacagtaggaccatagtgggggattct--------------------------------
-atcaacaccatgagcctcagggaccttaaacaggtagagaacaggctggagaaagg---
-----c--

>_R_gnl|SRA|SRR2638781.7341248.1 HWI-D00731:29:HGNNYADXX:1:1106:14929:70184.

--aatgc-------
ccagcactaccagcaggagtcctccaaactgcgccaacaaatcagtagcttacagaacgc
aaacagtaggaccatagtgggggattct--------------------------------
-atcaacaccatgagcctcagggaccttaaacaggtagagaacaggctggagaaagg---
-----c--

>_R_gnl|SRA|SRR2638781.63011206.1 HWI-D00731:29:HGNNYADXX:1:2115:15042:14616.

---atgc-------
ccagcactaccagcaggagtcctccaaactgcgccaacaaatcagtagcttacagaacgc
aaacagtaggaccatagtgggggattct--------------------------------
-atcaacaccatgagcctcagggaccttaaacaggtagagaacaggctggagaaagg---
-----ca---

>_R_gnl|SRA|SRR2638781.27540563.1 HWI-D00731:29:HGNNYADXX:1:1205:3651:67273.

---atgc-------
ccagcactaccagcaggagtcctccaaactgcgccaacaaatcagtagcttacagaacgc
aaacagtaggaccatagtgggggattct--------------------------------

-atcaacaccatgagcctcagggaccttaaacaggtagagaacaggctggagaaagg---
-----ca---

>_R_gnl|SRA|SRR2638781.10838607.1 HWI-D00731:29:HGNNYADXX:1:1109:17383:29933.

---cttc-------
cgatctctaccagcaggagtcctccaaactgcgccaacaaatcagtagcttacagaacgc
aaacagtaggaccatagtgggggattct--------------------------------
-atcaacaccatgagcctcagggaccttaaacaggtagagaacaggctggagaaagg---
-----cc---

>_R_gnl|SRA|SRR2638781.45623925.1 HWI-D00731:29:HGNNYADXX:1:2102:19378:72909.

--tgc-------
ccagcactaccagcaggagtcctccaaactgcgccaacaaatcagtagcttacagaacgc
aaacagtaggaccatagtgggggattct--------------------------------
-atcaacaccatgagcctcagggaccttaaacaggtagagaacaggctggagaaagg---
-----ccg--

>_R_gnl|SRA|SRR2638781.37766200.1 HWI-D00731:29:HGNNYADXX:1:1213:1877:17574.

--tgc-------
ccagcactaccagcaggagtcctccaaactgcgccaacaaatcagtagcttacagaacgc
aaacagtaggaccatagtgggggattct--------------------------------
-atcaacaccatgagcctcagggaccttaaacaggtagagaacaggctggagaaagg---
-----cat--

>_R_gnl|SRA|SRR2638781.13678178.1 HWI-D00731:29:HGNNYADXX:1:1111:8297:34277.

--tgc-------
ccagcactaccagcaggagtcctccaaactgcgccaacaaatcagtagcttacagaacgc
aaacagtaggaccatagtgggggattct--------------------------------
-atcaacaccatgagcctcagggaccttaaacaggtagagaacaggctggagaaagg---
-----cat--

>_R_gnl|SRA|SRR2638781.12798354.1 HWI-D00731:29:HGNNYADXX:1:1110:5123:71004.

--tgc-------
ccagcactaccagcaggagtcctccaaactgcgccaacaaatcagtagcttacagaacgc
aaacagtaggaccatagtggaggattct--------------------------------
-atcaacaccatgagcctcagggaccttaaacaggtagagaacaggctggagaaagg---
-----cat--

>gnl|SRA|SRR2638781.63727963.2 HWI-D00731:29:HGNNYADXX:1:2115:10125:61553.

--ctg----caaact-------
gcagcactaccagcaggagtcctccaaactgcgccaacaaatcagtagcttacagaacgc
aaacagtaggaccatagtgggggattct--------------------------------
-atcaacaccatgagcctcagggaccttaaacaggtagagaacaggctggagaa------

>_R_gnl|SRA|SRR2638781.68345745.1 HWI-D00731:29:HGNNYADXX:1:2202:1375:96756.

--------------------------------------gaagt----caatgc-------
ccagcactaccagcaggagtcctccaaactgcgccaacaaatcagtagcttacagaacgc
aaacagtaggaccatagtgggggattct--------------------------------
-atcaacaccatgagcctcagggaccttaaacaggtagagaacaggctggag--------

>_R_gnl|SRA|SRR2638781.63170969.1 HWI-D00731:29:HGNNYADXX:1:2115:14888:25172.

--------------------------------------gaagt----caatgc-------
ccagcactaccagcaggagtcctccaaactgcgccaacaaatcagtagcttacagaacgc
aaacagtaggaccatagtgggggattct--------------------------------
-atcaacaccatgagcctcagggaccttaaacaggtagagaacaggctggag--------

>_R_gnl|SRA|SRR2638781.41367253.1 HWI-D00731:29:HGNNYADXX:1:1215:4793:64776.

--------------------------------------gaagt----caatgc-------
ccagcactaccagcaggagtcctccaaactgcgccaacaaatcagtagcttacagaacgc
aaacagtaggaccatagtgggggattct--------------------------------
-atcaacaccatgagcctcagggaccttaaacaggtagagaacaggctggag--------

>_R_gnl|SRA|SRR2638781.36773188.1 HWI-D00731:29:HGNNYADXX:1:1212:18124:47478.

--------------------------------------gaagt----caatgc-------
ccagcactaccagcaggagtcctccaaactgcgccaacaaatcagtagcttacagaacgc
aaacagtaggaccatagtgggggattct--------------------------------
-atcaacaccatgagcctcagggaccttaaacaggtagagaacaggctggag--------

>_R_gnl|SRA|SRR2638781.16780099.1 HWI-D00731:29:HGNNYADXX:1:1113:11840:53435.

--------------------------------------gaagt----caatgc-------
ccagcactaccagcaggagtcctccaaactgcgccaacaaatcagtagcttacagaacgc
aaacagtaggaccatagtgggggattct--------------------------------
-atcaacaccatgagcctcagggaccttaaacaggtagagaacaggctggag--------

>_R_gnl|SRA|SRR2638781.9365576.1 HWI-D00731:29:HGNNYADXX:1:1108:13304:22585.

--------------------------------------gaagt----caatgc-------
ccagcactaccagcaggagtcctccaaactgcgccaacaaatcagtagcttacagaacgc
aaacagtaggaccatagtgggggattct--------------------------------
-atcaacaccatgagcctcagggaccttaaacaggtagagaacaggctggag--------

>_R_gnl|SRA|SRR2638781.2325079.1 HWI-D00731:29:HGNNYADXX:1:1102:10179:82540.

--------------------------------------gaagt----caatgc-------

ccagcactaccagcaggagtcctccaaactgcgccaacaaatcagtagcttacagaacgc
aaacagtaggaccatagtgggggattct--------------------------------
-atcaacaccatgagcctcagggaccttaaacaggtagagaacaggctggag--------

>gnl|SRA|SRR2638781.78363199.2 HWI-D00731:29:HGNNYADXX:1:2210:10832:58220.

--------------------------------------gaagt----caatgc-------
ccagcactaccagcaggagtcctccaaactgcgccaacaaatcagtagcttgcagaacgc
aaacagtaggaccatagtgggggattct--------------------------------
-atcaacaccatgagcctcagggaccttaaacaggtagagaacaggctggag--------

>_R_gnl|SRA|SRR2638781.58436708.1 HWI-D00731:29:HGNNYADXX:1:2112:17610:5968.

--------------------------------------tctgt----caatgc-------
ccagcactaccagcaggagtcctccaaactgcgccaacaaatcagtagcttacagaacgc
aaacagtaggaccatagtgggggattct--------------------------------
-atcaacaccatgagcctcagggaccttaaacaggtagagaacaggctggag--------

>_R_gnl|SRA|SRR2638781.1383772.1 HWI-D00731:29:HGNNYADXX:1:1102:2644:10100.

--------------------------------------ctagt----caatgc-------
ccagcactaccagcaggagtcctccaaactgcgccaacaaatcagtagcttacagaacgc
aaacagtaagaccatagtgggggattct--------------------------------
-atcaacaccatgagcctcagggaccttaaacaggtagagaacaggctggag--------

>gnl|SRA|SRR2638781.87701962.2 HWI-D00731:29:HGNNYADXX:1:2216:7000:99234.

------------------------------cagttgcagaagt----caatgc-------
ccagcactaccagcaggagtcctccaaactgcgccaacaaatcagtagcttacagaacgc
aaacag---gaccatagtgggggattct--------------------------------
-atcaacaccatgagcctcagggaccttaaacaggtagagaacaggc-------------

>gnl|SRA|SRR2638781.70770312.2 HWI-D00731:29:HGNNYADXX:1:2204:12141:97205.

------------------------------cagttgcagaagt----caatgc-------
ccagcactaccagcaggagtcctccaaactgcgccaacaaatcagtagcttacagaacgc
aaacag---gaccatagtgggggattct--------------------------------
-atcaacaccatgagcctcagggaccttaaacaggtagagaacaggc-------------

>gnl|SRA|SRR2638781.41367499.2 HWI-D00731:29:HGNNYADXX:1:1215:6137:64888.

------------------------------cagttgcagaagt----caatgc-------
ccagcactaccagcaggagtcctccaaactgcgccaacaaatcagtagcttacagaacgc
aaacag---gaccatagtgggggattct--------------------------------
-atcaacaccatgagcctcagggaccttaaacaggtagagaacaggc-------------

>_R_gnl|SRA|SRR2638781.41367499.1 HWI-D00731:29:HGNNYADXX:1:1215:6137:64888.

-------------------------------agttgcagaagt----caatgc-------
ccagcactaccagcaggagtcctccaaactgcgccaacaaatcagtagcttacagaacgc
aaacag---gaccatagtgggggattct--------------------------------
-atcaacaccatgagcctcagggaccttaaacaggtagagaacaggca------------

>gnl|SRA|SRR2638781.15862939.2 HWI-D00731:29:HGNNYADXX:1:1112:1476:88962.

-------------------------------agttgcagaagt----caatgc-------
ccagcactaccagcaggagtcctccaaactgcgccaacaaatcagtagcttacagaacgc
aaacag---gaccatagtgggggattct--------------------------------
-atcaacaccatgagcctcagggaccttaaacaggtagagaacaggct------------

>_R_gnl|SRA|SRR2638781.71039080.1 HWI-D00731:29:HGNNYADXX:1:2205:14808:18006.

--------------------------------gttgcagaagt----caatgc-------
ccagcactaccagcaggagtcctccaaactgcgccaacaaatcagtagcttacagaacgc
aaacag---gaccatagtgggggattct--------------------------------
-atcaacaccatgagcctcagggaccttaaacaggtagagaacaggctg-----------

>gnl|SRA|SRR2638781.66407583.2 HWI-D00731:29:HGNNYADXX:1:2201:13432:45274.

--------------------------------gttgcagaagt----caatgc-------
ccagcactaccagcaggagtcctccaaactgcgccaacaaatcagtagcttacagaacgc
aaacag---gaccatagtgggggattct--------------------------------
-atcaacaccatgagcctcagggaccttaaacaggtagagaacaggctg-----------

>_R_gnl|SRA|SRR2638781.58927574.1 HWI-D00731:29:HGNNYADXX:1:2112:13716:39358.

--------------------------------gttgcagaagt----caatgc-------
ccagcactaccagcaggagtcctccaaactgcgccaacaaatcagtagcttacagaacgc
aaacag---gaccatagtgggggattct--------------------------------
-atcaacaccatgagcctcagggaccttaaacaggtagagaacaggctg-----------

>_R_gnl|SRA|SRR2638781.27979551.1 HWI-D00731:29:HGNNYADXX:1:1205:17687:100302.

--------------------------------gttgcagaagt----caatgc-------
ccagcactaccagcaggagtcctccaaactgcgccaacaaatcagtagcttacagaacgc
aaacag---gaccatagtgggggattct--------------------------------
-atcaacaccatgagcctcagggaccttaaacaggtagagaacaggctg-----------

>_R_gnl|SRA|SRR2638781.11070793.1 HWI-D00731:29:HGNNYADXX:1:1109:12911:46643.

--------------------------------gttgcagaagt----caatgc-------
ccagcactaccagcaggagtcctccaaactgcgccaacaaatcagtagcttacagaacgc
aaacag---gaccatagtgggggattct--------------------------------
-atcaacaccatgagcctcagggaccttaaacaggtagagaacaggctg-----------

>gnl|SRA|SRR2638781.10156888.2 HWI-D00731:29:HGNNYADXX:1:1108:8809:80207.

--------------------------------gttgcagaagt----caatgc-------
ccagcactaccagcaggagtcctccaaactgcgccaacaaatcagtagcttacagaacgc
aaacag---gaccatagtgggggattct--------------------------------
-atcaacaccatgagcctcagggaccttaaacaggtagagaacaggctg-----------

>_R_gnl|SRA|SRR2638781.65163917.1 HWI-D00731:29:HGNNYADXX:1:2116:2764:56629.

----------------------------cacagttgcagaagt----caatgc-------
ccagcactaccagcaggagtcctccaaactgcgccaacaaatcagtagcttacagaacgc
aaacag---gaccatagtgggggattct--------------------------------
-atcaacaccatgagcctcagggaccttaaacaggtagagaacag---------------

>_R_gnl|SRA|SRR2638781.61197995.1 HWI-D00731:29:HGNNYADXX:1:2113:10051:93301.

----------------------------cacagttgcagaagt----caatgc-------
ccagcactaccagcaggagtcctccaaactgcgccaacaaatcagtagcttacagaacgc
aaacag---gaccatagtgggggattct--------------------------------
-atcaacaccatgagcctcagggaccttaaacaggtagagaacag---------------

>_R_gnl|SRA|SRR2638781.79525875.1 HWI-D00731:29:HGNNYADXX:1:2211:12862:40715.

-----------------------------------gcagaagt----caatgc-------
ccagcactaccagcaggagtcctccaaactgcgccaacaaatcagtagcttacagaacgc
aaacag---gaccatagtgggggattct--------------------------------
-atcaacaccatgagcctcagggaccttaaacaggtagagaacaggctggag--------

>_R_gnl|SRA|SRR2638781.56705257.1 HWI-D00731:29:HGNNYADXX:1:2110:17340:85356.

-----------------------------------gcagaagt----caatgc-------
ccagcactaccagcaggagtcctccaaactgcgccaacaaatcagtagcttacagaacgc
aaacag---gaccatagtgggggattct--------------------------------
-atcaacaccatgagcctcagggaccttaaacaggtagagaacaggctggag--------

>_R_gnl|SRA|SRR2638781.45993419.1 HWI-D00731:29:HGNNYADXX:1:2102:9873:100671.

-----------------------------------gcagaagt----caatgc-------
ccagcactaccagcaggagtcctccaaactgcgccaacaaatcagtagcttacagaacgc

aaacag---gaccatagtgggggattct--------------------------------
-atcaacaccatgagcctcagggaccttaaacaggtagagaacaggctggag--------

>_R_gnl|SRA|SRR2638781.20410389.1 HWI-D00731:29:HGNNYADXX:1:1116:3359:5616.

-----------------------------------gcagaagt----caatgc-------
ccagcactaccagcaggagtcctccaaactgcgccaacaaatcagtagcttacagaacgc
aaacag---gaccatagtgggggattct--------------------------------
-atcaacaccatgagcctcagggaccttaaacaggtagagaacaggctggag--------

>_R_gnl|SRA|SRR2638781.45123092.1 HWI-D00731:29:HGNNYADXX:1:2102:18814:34531.

-----------------------------------gcagaagt----caatgc-------
ccagcactaccagcaggagtcctccaaactgcgccaccaaatcagtagcttacagaacgc
aaacag---gaccatagtgggggattct--------------------------------
-atcaacaccatgagcctcagggaccttaaacaggtagagaacaggctggag--------

>_R_gnl|SRA|SRR2638781.82843316.1 HWI-D00731:29:HGNNYADXX:1:2213:3008:71255.

---------------------------------------aagt----caatgc-------
ccagcactaccagcaggagtcctccaaactgcgccaacaaatcagtagcttacagaacgc
aaacag---gaccatagtgggggattct--------------------------------
-atcaacaccatgagcctcagggaccttaaacaggtagagaacaggctggagagag----

>_R_gnl|SRA|SRR2638781.37903203.1 HWI-D00731:29:HGNNYADXX:1:1213:3215:27110.

---------------------------------------aagt----caatgc-------
ccagcactaccagcaggagtcctccaaactgcgccaacaaatcagtagcttacagaacgc
aaacag---gaccatagtgggggattct--------------------------------
-atcaacaccatgagcctcagggaccttaaacaggtagagaacaggctggagagag----

>_R_gnl|SRA|SRR2638781.81732048.1 HWI-D00731:29:HGNNYADXX:1:2212:4345:94691.

---------------------------------------aagt----caatgc-------
ccagcactaccagcaggagtcctccaaactgcgccaacaaatcagtagcttacagaacgc
aaacag---gaccatagtgggggattct--------------------------------
-atcaacaccatgagcctcagggaccttaaacaggtagagaacaggctggagaaag----

>_R_gnl|SRA|SRR2638781.81743907.1 HWI-D00731:29:HGNNYADXX:1:2212:10182:95352.

--agt----caatgc-------
ccagcactaccagcaggagtcctccaaactgcgccaacaaatcagtagcttacagaacgc
aaacag---gaccatagtgggggattct--------------------------------
-atcaacaccatgagcctcagggaccttaaacaggtagagaacaggctggagaaagg---

>gnl|SRA|SRR2638781.36683744.2 HWI-D00731:29:HGNNYADXX:1:1212:16531:41220.

--agt----caatgc-------
ccagcactaccagcaggagtcctccaaactgcgccaacaaatcagtagcttacagaacgc
aaacag---gaccatagtgggggattct--------------------------------
-atcaacaccatgagcctcagggaccttaaacaggtagagaacaggctggagaaagg---

>_R_gnl|SRA|SRR2638781.32705237.1 HWI-D00731:29:HGNNYADXX:1:1209:10798:55036.

--agt----caatgc-------
ccagcactaccagcaggagtcctccaaactgcgccaacaaatcagtagcttacagaacgc
aaacag---gaccatagtgggggattct--------------------------------
-atcaacaccatgagcctcagggaccttaaacaggtagagaacaggctggagaaagg---

>gnl|SRA|SRR2638781.32592200.2 HWI-D00731:29:HGNNYADXX:1:1209:14681:46890.

--agt----caatgc-------
ccagcactaccagcaggagtcctccaaactgcgccaacaaatcagtagcttacagaacgc
aaacag---gaccatagtgggggattct--------------------------------
-atcaacaccatgagcctcagggaccttaaacaggtagagaacaggctggagaaagg---

>gnl|SRA|SRR2638781.46269187.2 HWI-D00731:29:HGNNYADXX:1:2103:4575:21968.

--agt----caatgt-------
ccatcactaccagcaggagtccttcaaaccgcgccaacaaatcagtagcttacagaacgc
aaacag---gaccatagtgggggattct--------------------------------
-atcaacaccatgagcttcagggaccttaaacaggtagagaacaggctggagaaagg---

>_R_gnl|SRA|SRR2638781.80421726.1 HWI-D00731:29:HGNNYADXX:1:2212:4687:4179.

---gt----caatgc-------
ccagcactaccagcaggagtcctccaaactgcgccaacaaatcagtagcttacagaacgc
aaacag---gaccatagtgggggattct--------------------------------
-atcaacaccatgagcctcagggaccttaaacaggtagagaacaggctggagaaagg---
-----c--

>gnl|SRA|SRR2638781.50260215.2 HWI-D00731:29:HGNNYADXX:1:2106:3595:24486.

---gt----caatgc-------
ccagcactaccagcaggagtcctccaaactgcgccaacaaatcagtagcttacagaacgc
aaacag---gaccatagtgggggattct--------------------------------
-atcaacaccatgagcctcagggaccttaaacaggtagagaacaggctggagaaagg---
-----c--

>_R_gnl|SRA|SRR2638781.42144395.1 HWI-D00731:29:HGNNYADXX:1:1216:8124:17466.

---gt----caatgc-------
ccagcactaccagcaggagtcctccaaactgcgccaacaaatcagtagcttacagaacgc
aaacag---gaccatagtgggggattct--------------------------------
-atcaacaccatgagcctcagggaccttaaacaggtagagaacaggctggagaaagg---
-----c--

>_R_gnl|SRA|SRR2638781.30464122.1 HWI-D00731:29:HGNNYADXX:1:1207:19070:89270.

---gt----caatgc-------
ccagcactaccagcaggagtcctccaaactgcgccaacaaatcagtagcttacagaacgc
aaacag---gaccatagtgggggattct--------------------------------
-atcaacaccatgagcctcagggaccttaaacaggtagagaacaggctggagaaagg---
-----c--

>gnl|SRA|SRR2638781.49124565.2 HWI-D00731:29:HGNNYADXX:1:2105:13408:40805.

----------------ctccaactctggcacagttgcagaagt----caatgc-------
ccagcactaccagcaggagtcctccaaactgcgccaacaaatcagtagcttacagaacgc
aaacag---gaccatagtgggggattct--------------------------------
-atcaacaccatgagcctcagggacc------------agatcgg---------------

>gnl|SRA|SRR2638781.38024121.2 HWI-D00731:29:HGNNYADXX:1:1213:17843:35290.

----------------ctccaactctggcacagttgcagaagt----caatgc-------
ccagcactaccagcaggagtcctccaaactgcgccaacaaatcagtagcttacagaacgc
aaacag---gaccatagtgggggattct--------------------------------
-atcaacaccatgagcctcagggacc------------agatcgg---------------

>gnl|SRA|SRR2638781.85927066.2 HWI-D00731:29:HGNNYADXX:1:2215:8181:79512.

----aaacagtgacacctccaactctggcacagttgcagaagt----caatgc-------
ccagcactaccagcaggagtcctccaaactgcgccaacaaatcagtagcttacagaacgc
aaacagtaggaccatagtgggggattct--------------------------------
-atcaacaccatgagcct--

>_R_gnl|SRA|SRR2638781.84293756.1 HWI-D00731:29:HGNNYADXX:1:2214:1716:70141.

---caaacagtgacacctccaactctggcacagttgcagaagt----caatgc-------
ccagcactaccagcaggagtcctccaaactgcgccaacaaatcagtagcttacagaacgc
aaacagtaggaccatagtgggggattct--------------------------------
-atcaacaccatgagcc---

>_R_gnl|SRA|SRR2638781.66088400.1 HWI-D00731:29:HGNNYADXX:1:2201:14466:20098.

---caaacagtgacacctccaactctggcacagttgcagaagt----caatgc-------
ccagcactaccagcaggagtcctccaaactgcgccaacaaatcagtagcttacagaacgc
aaacagtaggaccatagtgggggattct--------------------------------

-atcaacaccatgagcc---

>_R_gnl|SRA|SRR2638781.17029223.1 HWI-D00731:29:HGNNYADXX:1:1113:5721:70558.

---caaacagtgacacctccaactctggcacagttgcagaagt----caatgc-------
ccagcactaccagcaggagtcctccaaactgcgccaacaaatcagtagcttacagaacgc
aaacagtaggaccatagtgggggattct--------------------------------
-atcaacaccatgagcc---

>_R_gnl|SRA|SRR2638781.64999960.1 HWI-D00731:29:HGNNYADXX:1:2116:1344:45926.

-ggcaaacagtgacacctccaactctggcacagttgcagaagt----caatgc-------
ccagcactaccagcaggagtcctccaaactgcgccaacaaatcagtagcttacagaacgc
aaacagtaggaccatagtgggggattct--------------------------------
-atcaacaccatgag---

>gnl|SRA|SRR2638781.64950426.2 HWI-D00731:29:HGNNYADXX:1:2116:18761:42459.

-ggcaaacagtgacacctccaactctggcacagttgcagaagt----caatgc-------
ccagcactaccagcaggagtcctccaaactgcgccaacaaatcagtagcttacagaacgc
aaacagtaggaccatagtgggggattct--------------------------------
-atcaacaccatgag---

>_R_gnl|SRA|SRR2638781.60352150.1 HWI-D00731:29:HGNNYADXX:1:2113:11393:36694.

-ggcaaacagtgacacctccaactctggcacagttgcagaagt----caatgc-------
ccagcactaccagcaggagtcctccaaactgcgccaacaaatcagtagcttacagaacgc
aaacagtaggaccatagtgggggattct--------------------------------
-atcaacaccatgag---

>_R_gnl|SRA|SRR2638781.50529323.1 HWI-D00731:29:HGNNYADXX:1:2106:9077:43980.

-ggcaaacagtgacacctccaactctggcacagttgcagaagt----caatgc-------
ccagcactaccagcaggagtcctccaaactgcgccaacaaatcagtagcttacagaacgc
aaacagtaggaccatagtgggggattct--------------------------------
-atcaacaccatgag---

>_R_gnl|SRA|SRR2638781.87306390.1 HWI-D00731:29:HGNNYADXX:1:2216:5754:73170.

----aaacagtgacacctccaactctggcacagttgcagaagt----caatgc-------
ccagcactaccagcaggagtcctccaaactgcgccaacaaatcagtagcttacagaacgc
aaac---aggaccatagtgggggattct--------------------------------
-atcaacaccatgagcctcag---------------------------------------

>_R_gnl|SRA|SRR2638781.13029014.1 HWI-D00731:29:HGNNYADXX:1:1110:19607:87298.

----aaacagtgacacctccaactctggcacagttgcagaagt----caatgc-------
ccagcactaccagcaggagtcctccaaactgcgccaacaaatcagtagcttacagaacgc
aaac---aggaccatagtgggggattct--------------------------------
-atcaacaccatgagcctcag---------------------------------------

>_R_gnl|SRA|SRR2638781.9032289.1 HWI-D00731:29:HGNNYADXX:1:1107:3775:97604.

----aaacagtgacacctccaactctggcacagttgcagaagt----caatgc-------
ccagcactaccagcaggagtcctccaaactgcgccaacaaatcagtagcttacagaacgc
aaac---aggaccatagtgggggattct--------------------------------
-atcaacaccatgagcctcag---------------------------------------

>gnl|SRA|SRR2638781.44563291.2 HWI-D00731:29:HGNNYADXX:1:2101:18940:91321.

---caaacagtgacacctccaactctggcacagttgcagaagt----caatgc-------
ccagcactaccagcaggagtcctccaaactgcgccaacaaatcagtagcttacagaacgc
aaac---aggaccatagtgggggattct--------------------------------
-atcaacaccatgagcctca--

>gnl|SRA|SRR2638781.35757925.2 HWI-D00731:29:HGNNYADXX:1:1211:11129:75086.

---caaacagtgacacctccaactctggcacagttgcagaagt----caatgc-------
ccagcactaccagcaggagtcctccaaactgcgccaacaaatcagtagcttacagaacgc
aaac---aggaccatagtgggggattct--------------------------------
-atcaacaccatgagcctca--

>gnl|SRA|SRR2638781.27041229.2 HWI-D00731:29:HGNNYADXX:1:1205:20198:29248.

---caaacagtgacacctccaactctggcacagttgcagaagt----caatgc-------
ccagcactaccagcaggagtcctccaaactgcgccaacaaatcagtagcttacagaacgc
aaac---aggaccatagtgggggattct--------------------------------
-atcaacaccatgagcctca--

>gnl|SRA|SRR2638781.3286325.2 HWI-D00731:29:HGNNYADXX:1:1103:16745:64175.

---caaacagtgacacctccaactctggcacagttgcagaagt----caatgc-------
ccagcactaccagcaggagtcctccaaactgcgccaacaaatcagtagcttacagaacgc
aaac---aggaccatagtgggggattct--------------------------------
-atcaacaccatgagcctca--

>_R_gnl|SRA|SRR2638781.2326419.1 HWI-D00731:29:HGNNYADXX:1:1102:18426:82732.

---caaacagtgacacctccaactctggcacagttgcagaagt----caatgc-------

ccagcactaccagcaggagtcctccaaactgcgccaacaaatcagtagcttacagaacgc
aaac---aggaccatagtgggggattct--------------------------------
-atcaacaccatgagcctca--

>_R_gnl|SRA|SRR2638781.78605824.1 HWI-D00731:29:HGNNYADXX:1:2210:3590:75385.

-----aacagtgacacctccaactctggcacagttgcggaagt----caatgc-------
ccagcactaccagcaggagtcctccaaactgcgccaacaaatcagtagcttacagaacgc
aaac---aggaccatagtgggggattct--------------------------------
-atcaacaccatgagcctcagg--------------------------------------

>_R_gnl|SRA|SRR2638781.53039585.1 HWI-D00731:29:HGNNYADXX:1:2108:18861:26811.

-----aacagtgacacctccaactctggcacagttgcagaagt----caatgc-------
ccagcactaccagcaggagtcctccaaactgcgccaacaaatcagtagcttacagaacgc
aaac---aggaccatagtgggggattct--------------------------------
-atcaacaccatgagcctcagg--------------------------------------

>_R_gnl|SRA|SRR2638781.5355089.1 HWI-D00731:29:HGNNYADXX:1:1105:11742:21827.

-----aacagtgacacctccaactctggcacagttgcagaagt----caatgc-------
ccagcactaccagcaggagtcctccaaactgcgccaacaaatcagtagcttacagaacgc
aaac---aggaccatagtgggggattct--------------------------------
-atcaacaccatgagcctcagg--------------------------------------

>_R_gnl|SRA|SRR2638781.569730.1 HWI-D00731:29:HGNNYADXX:1:1101:10951:46324.

-----aacagtgacacctccaactctggcacagttgcagaagt----caatgc-------
ccagcactaccagcaggagtcctccaaactgcgccaacaaatcagtagcttacagaacgc
aaac---aggaccatagtgggggattct--------------------------------
-atcaacaccatgagcctcagg--------------------------------------

>_R_gnl|SRA|SRR2638781.12919029.1 HWI-D00731:29:HGNNYADXX:1:1110:15354:79648.

-----aacagtgacacctccaactctggcacagttgcagaagt----caatgc-------
ccagcactaccagcaggagtcctccaaactgcgccaacaaatcagtagcttacagaacgc
aaac---aggaccatagtgggggattct--------------------------------
-atcaacaccatgagcctcaag--------------------------------------

>_R_gnl|SRA|SRR2638781.1765340.1 HWI-D00731:29:HGNNYADXX:1:1102:12354:39352.

-----aacagtgacacctccaactctggcacagttgcagaagt----caatgc-------
ccagcactaccagcaggagtcctccaaactgcgccaacaaatcagtagcttacagaacgc
aaac---aggaccatagtgggggattct--------------------------------
-atcaacaccatgagcctcaag--------------------------------------

>_R_gnl|SRA|SRR2638781.55178739.1 HWI-D00731:29:HGNNYADXX:1:2109:3660:78768.

------acagtgacacctccaactctggcacagttgcagaagt----caatgc-------
ccagcactaccagcaggagtcctccaaactgcgccaacaaatcagtagcttacagaacgc
aaac---aggaccatagtgggggattct-------------------------------
-atcaacaccatgagcctcaggg-----------------------------------

>gnl|SRA|SRR2638781.37533135.2 HWI-D00731:29:HGNNYADXX:1:1212:7345:100624.

-------cagtgacacctccaactctggcacagttgcagaagt----caatgc-------
ccagcactaccagcaggagtcctccaaactgcgccaacaaatcagtagcttacagaacgc
aaac---aggaccatagtgggggattct-------------------------------
-atcaacaccatgagcctcaggga----------------------------------

>_R_gnl|SRA|SRR2638781.29336212.1 HWI-D00731:29:HGNNYADXX:1:1207:7713:4227.

--------agtgacacctccaactctggcacagttgcagaagt----caatgc-------
ccagcactaccagcaggagtcctccaaactgcgccaacaaatcagtagcttacagaacgc
aaac---aggaccatagtgggggattct-------------------------------
-atcaacaccatgagcctcagggac---------------------------------

>gnl|SRA|SRR2638781.65918258.2 HWI-D00731:29:HGNNYADXX:1:2201:2765:6928.

---------gtgacacctccaactctggcacagttgcagaagt----caatgc-------
ccagcactaccagcaggagtcctccaaactgcgccaacaaatcagtagcttacagaacgc
aaac---aggaccatagtgggggattct-------------------------------
-atcaacaccatgagcctcagggacc--------------------------------

>_R_gnl|SRA|SRR2638781.49124565.1 HWI-D00731:29:HGNNYADXX:1:2105:13408:40805.

---------ccgatctctccaactctggcacagttgcagaagt----caatgc-------
ccagcactaccagcaggagtcctccaaactgcgccaacaaatcagtagcttacagaacgc
aaac---aggaccatagtgggggattct-------------------------------
-atcaacaccatgagcctcagggacc--------------------------------

>_R_gnl|SRA|SRR2638781.38024121.1 HWI-D00731:29:HGNNYADXX:1:1213:17843:35290.

---------ccgatctctccaactctggcacagttgcagaagt----caatgc-------
ccagcactaccagcaggagtcctccaaactgcgccaacaaatcagtagcttacagaacgc
aaac---aggaccatagtgggggattct-------------------------------
-atcaacaccatgagcctcagggacc--------------------------------

>_R_gnl|SRA|SRR2638781.82337138.1 HWI-D00731:29:HGNNYADXX:1:2213:16638:36527.

------------------------------aaga--------------------------
aggcaaacagtgacacctccaactctggcacagttgcagaagt----caatgc-------
ccagcactaccagcaggagtcctccaaactgcgccaacaaatcagtagcttacagaacgc
aaac---aggaccatagtgggggattct--------------------------------
-atcaacaccatg---

>gnl|SRA|SRR2638781.6605491.2 HWI-D00731:29:HGNNYADXX:1:1106:20794:15595.
------------------------------aaga--------------------------
aggcaaacagtgacacctccaactctggcacagttgcagaagt----caatgc-------
ccagcactaccagcaggagtcctccaaactgcgccaacaaatcagtagcttacagaacgc
aaac---aggaccatagtgggggattct--------------------------------
-atcaacaccatg---

>gnl|SRA|SRR2638781.56973598.2 HWI-D00731:29:HGNNYADXX:1:2111:20903:4542.
-------------------------------aga--------------------------
aggcaaacagtgacacctccaactctggcacagttgcagaagt----caatgc-------
ccagcactaccagcaggagtcctccaaactgcgccaacaaatcagtagcttacagaacgc
aaac---aggaccatagtgggggattct--------------------------------
-atcaacaccatga--

>_R_gnl|SRA|SRR2638781.31574996.1 HWI-D00731:29:HGNNYADXX:1:1208:6946:72230.
--------------------------------ga--------------------------
aggcaaacagtgacacctccaactctggcacagttgcagaagt----caatgc-------
ccagcactaccagcaggagtcctccaaactgcgccaacaaatcagtagcttacagaacgc
aaac---aggaccatagtgggggattct--------------------------------
-atcaacaccatgac---

>_R_gnl|SRA|SRR2638781.686399.1 HWI-D00731:29:HGNNYADXX:1:1101:20754:55390.
--------------------------------ga--------------------------
aggcaaacagtgacacctccaactctggcacagttgcagaagt----caatgc-------
ccagcactaccagcaggagtcctccaaactgcgccaacaaatcagtagcttacagaacgc
aaac---aggaccatagtgggggattct--------------------------------
-atcaacaccatgag---

>gnl|SRA|SRR2638781.26306717.2 HWI-D00731:29:HGNNYADXX:1:1204:5162:72498.
----
aggcaaacagtgacacctccaactctggcacagttgcagaagt----caatgc-------
ccagcactaccagcaggagtcctccaaactgcgccaacaaatcagtagcttacagaacgc
aaac---aggaccatagtgggggattct--------------------------------
-atcaacaccatgagcc---

>gnl|SRA|SRR2638781.15653075.2 HWI-D00731:29:HGNNYADXX:1:1112:3828:74040.
----
aggcaaacagtgacacctccaactctggcacagttgcagaagt----caatgc-------
ccagcactaccagcaggagtcctccaaactgcgccaacaaatcagtagcttacagaacgc

aaac---aggaccatagtgggggattct--------------------------------
-atcaacaccatgagcc---

>gnl|SRA|SRR2638781.16037220.2 HWI-D00731:29:HGNNYADXX:1:1112:13511:100985.

-ggcaaacagtgacacctccaactctggcacagttgcagaagt----caatgc-------
ccagcactaccagcaggagtcctccaaactgcgccaacaaatcagtagcttacagaacgc
aaac---aggaccatagtgggggattct--------------------------------
-atcaacaccatgagcct--

>gnl|SRA|SRR2638781.81097144.2 HWI-D00731:29:HGNNYADXX:1:2212:3462:50998.

-ggcaaacagtgacacctccaactctggcacagttgcagaagt----caatgc-------
ccagcactaccagcaggagtcctccaaactgcgccaacaaatcagtagcttacagaacgc
aaac---aggaccatagtgagggattct--------------------------------
-atcaacaccatgagcct--

>_R_gnl|SRA|SRR2638781.84037034.1 HWI-D00731:29:HGNNYADXX:1:2214:3414:52808.
--------------------------------ga--------------------------
aggcaaacagtgacacctccaactctggcacagttgcagaagt----caatgc-------
ccagcactaccagcaggagtcctccaaactgcgccaacaaatcagtagcttacagaacgc
aaacagtaggaccatagtgggggattct--------------------------------
-atcaacaccat--

>_R_gnl|SRA|SRR2638781.39833132.1 HWI-D00731:29:HGNNYADXX:1:1214:20752:60231.
--------------------------------ga--------------------------
aggcaaacagtgacacctccaactctggcacagttgcagaagt----caatgc-------
ccagcactaccagcaggagtcctccaaactgcgccaacaaatcagtagcttacagaacgc
aaacagtaggaccatagtgggggattct--------------------------------
-atcaacaccat--

>_R_gnl|SRA|SRR2638781.73970452.1 HWI-D00731:29:HGNNYADXX:1:2207:4890:38469.
--------------------------------ga--------------------------
aggcaaacagtgacacctccaactctggcacagttgcagaagt----caatgc-------
ccagcactaccagcaggagtcctccaaactgcgccaacaaatcagtagcttacagaacgc
aaacagtaggaccatagtgggggattct--------------------------------
-atcaacaccag--

>gnl|SRA|SRR2638781.41634129.2 HWI-D00731:29:HGNNYADXX:1:1215:10640:82952.
-------------------------------aga--------------------------
aggcaaacagtgacacctccaactctggcacagttgcagaagt----caatgc-------
ccagcactaccagcaggagtcctccaaactgcgccaacaaatcagtagcttacagaacgc
aaacagtaggaccatagtgggggattct--------------------------------
-atcaacacca---

>_R_gnl|SRA|SRR2638781.82735777.1 HWI-D00731:29:HGNNYADXX:1:2213:17254:63840.
---------------------------tacaaga--------------------------
aggcaaacagtgacacctccaactctggcacagttgcagaagt----caatgc-------
ccagcactaccagcaggagtcctccaaactgcgccaacaaatcagtagcttacagaacgc
aaacagtaggaccatagtgggggattct--------------------------------
-atcaac---

>_R_gnl|SRA|SRR2638781.74112435.1 HWI-D00731:29:HGNNYADXX:1:2207:6429:49065.
---------------------------tacaaga--------------------------
aggcaaacagtgacacctccaactctggcacagttgcagaagt----caatgc-------
ccagcactaccagcaggagtcctccaaactgcgccaacaaatcagtagcttacagaacgc
aaacagtaggaccatagtgggggattct--------------------------------
-atcaac---

>_R_gnl|SRA|SRR2638781.81104132.1 HWI-D00731:29:HGNNYADXX:1:2212:1952:51491.
----------------------------acaaga--------------------------
aggcaaacagtgacacctccaactctggcacagttgcagaagt----caatgc-------
ccagcactaccagcaggagtcctccaaactgcgccaacaaatcagtagcttacagaacgc
aaacagtaggaccatagtgggggattct--------------------------------
-atcaaca--

>gnl|SRA|SRR2638781.14079708.2 HWI-D00731:29:HGNNYADXX:1:1111:1856:62858.
----------------------------acaaga--------------------------
aggcaaacagtgacacctccaactctggcacagttgcagaagt----caatgc-------
ccagcactaccagcaggagtcctccaaactgcgccaacaaatcagtagcttacagaacgc
aaacagtaggaccatagtgggggattct--------------------------------
-atcaaca--

>_R_gnl|SRA|SRR2638781.77735769.1 HWI-D00731:29:HGNNYADXX:1:2210:10017:13536.
-----------------------------caaga--------------------------
aggcaaacagtgacacctccaactctggcacagttgcagaagt----caatgc-------
ccagcactaccagcaggagtcctccaaactgcgccaacaaatcagtagcttacagaacgc
aaacagtaggaccatagtgggggattct--------------------------------
-atcaacac---

>gnl|SRA|SRR2638781.71712006.2 HWI-D00731:29:HGNNYADXX:1:2205:9310:68825.
-----------------------------caaga--------------------------
aggcaaacagtgacacctccaactctggcacagttgcagaagt----caatgc-------
ccagcactaccagcaggagtcctccaaactgcgccaacaaatcagtagcttacagaacgc
aaacagtaggaccatagtgggggattct--------------------------------
-atcaacac---

>_R_gnl|SRA|SRR2638781.17147066.1 HWI-D00731:29:HGNNYADXX:1:1113:13427:78719.
-----------------------------caaga--------------------------

aggcaaacagtgacacctccaactctggcacagttgcagaagt----caatgc-------
ccagcactaccagcaggagtcctccaaactgcgccaacaaatcagtagcttacagaacgc
aaacagtaggaccatagtgggggattct--------------------------------
-atcaacac---

>gnl|SRA|SRR2638781.1691291.2 HWI-D00731:29:HGNNYADXX:1:1102:15102:33723.
-----------------------------caaga--------------------------
aggcaaacagtgacacctccaactctggcacagttgcagaagt----caatgc-------
ccagcactaccagcaggagtcctccaaactgcgccaacaaatcagtagcttacagaacgc
aaacagtaggaccatagtgggggattct--------------------------------
-atcaacac---

>_R_gnl|SRA|SRR2638781.74419365.1 HWI-D00731:29:HGNNYADXX:1:2207:7298:71866.
-------------------------ggtacaaga--------------------------
aggcaaacagtgacacctccaactctggcacagttgcagaagt----caatgc-------
ccagcactaccagcaggagtcctccaaactgcgccaacaaatcagtagcttacagaacgc
aaacagtaggaccatagtgggggattct--------------------------------
-atca---

>_R_gnl|SRA|SRR2638781.28560689.1 HWI-D00731:29:HGNNYADXX:1:1206:4571:45109.
-------------------------ggtacaaga--------------------------
aggcaaacagtgacacctccaactctggcacagttgcagaagt----caatgc-------
ccagcactaccagcaggagtcctccaaactgcgccaacaaatcagtagcttacagaacgc
aaacagtaggaccatagtgggggattct--------------------------------
-atca---

>gnl|SRA|SRR2638781.13798241.2 HWI-D00731:29:HGNNYADXX:1:1111:4721:42798.
-------------------------ggtacaaga--------------------------
aggcaaacagtgacacctccaactctggcacagttgcagaagt----caatgc-------
ccagcactaccagcaggagtcctccaaactgcgccaacaaatcagtagcttacagaacgc
aaacagtaggaccatagtgggggattct--------------------------------
-atca---

>gnl|SRA|SRR2638781.19641045.2 HWI-D00731:29:HGNNYADXX:1:1115:1633:52325.
--------------------------gtacaaga--------------------------
aggcaaacagtgacacctccaactctggcacagttgcagaagt----caatgc-------
ccagcactaccagcaggagtcctccaaactgcgccaacaaatcagtagcttacagaacgc
aaacagtaggaccatagtgggggattct--------------------------------
-atcaa--

>gnl|SRA|SRR2638781.73370767.2 HWI-D00731:29:HGNNYADXX:1:2206:5077:93191.
---------------------gagaggtacaaga--------------------------
aggcaaacagtgacacctccaactctggcacagttgcagaagt----caatgc-------
ccagcactaccagcaggagtcctccaaactgcgccaacaaatcagtagcttacagaacgc
aaacagtaggaccatagtgggggattct--------------------------------

>_R_gnl|SRA|SRR2638781.40109306.1 HWI-D00731:29:HGNNYADXX:1:1214:8585:79210.
----------------------agaggtacaaga--------------------------
aggcaaacagtgacacctccaactctggcacagttgcagaagt----caatgc-------
ccagcactaccagcaggagtcctccaaactgcgccaacaaatcagtagcttacagaacgc
aaacagtaggaccatagtgggggattct--------------------------------
-a--

>_R_gnl|SRA|SRR2638781.63650976.1 HWI-D00731:29:HGNNYADXX:1:2115:7981:56646.
-----------------------gaggtacaaga--------------------------
aggcaaacagtgacacctccaactctggcacagttgcagaagt----caatgc-------
ccagcactaccagcaggagtcctccaaactgcgccaacaaatcagtagcttacagaacgc
aaacagtaggaccatagtgggggattct--------------------------------
-at---

>_R_gnl|SRA|SRR2638781.52478927.1 HWI-D00731:29:HGNNYADXX:1:2107:3586:86461.
------------------------aggtacaaga--------------------------
aggcaaacagtgacacctccaactctggcacagttgcagaagt----caatgc-------
ccagcactaccagcaggagtcctccaaactgcgccaacaaatcagtagcttacagaacgc
aaacagtaggaccatagtgggggattct--------------------------------
-atc--

>_R_gnl|SRA|SRR2638781.41423220.1 HWI-D00731:29:HGNNYADXX:1:1215:7804:68685.
------------------------aggtacaaga--------------------------
aggcaaacagtgacacctccaactctggcacagttgcagaagt----caatgc-------
ccagcactaccagcaggagtcctccaaactgcgccaacaaatcagtagcttacagaacgc
aaacagtaggaccatagtgggggattct--------------------------------
-atc--

>_R_gnl|SRA|SRR2638781.8614533.1 HWI-D00731:29:HGNNYADXX:1:1107:3547:66901.
------------------------aggtacaaga--------------------------
aggcaaacagtgacacctccaactctggcacagttgcagaagt----caatgc-------
ccagcactaccagcaggagtcctccaaactgcgccaacaaatcagtagcttacagaacgc
aaacagtaggaccatagtgggggattct--------------------------------
-atc--

>_R_gnl|SRA|SRR2638781.2793349.1 HWI-D00731:29:HGNNYADXX:1:1103:16484:18890.
------------------------aggtacaaga--------------------------
aggcaaacagtgacacctccaactctggctgagttgcagaagt----caatgc-------
ccagcactaccagcaggagtcctccaaactgcgccaacaaatcagtagcttacagaacgc
aaacagtaggaccatagtgggggattct--------------------------------
-atc--

>_R_gnl|SRA|SRR2638781.73328864.1 HWI-D00731:29:HGNNYADXX:1:2206:17056:89921.
--------------------tgagaggtacaaga--------------------------
aggcaaacagtgacacctccaactctggcacagttgcagaagt----caatgc-------
ccagcactaccagcaggagtcctccaaactgcgccaacaaatcagtagcttacagaacgc
aaac---aggaccatagtgggggattct--------------------------------
-at---

>_R_gnl|SRA|SRR2638781.50947698.1 HWI-D00731:29:HGNNYADXX:1:2106:12230:74051.
---------------------gagaggtacaaga--------------------------
aggcaaacagtgacacctccaactctggcacagttgcagaagt----caatgc-------
ccagcactaccagcaggagtcctccaaactgcgccaacaaatcagtagcttacagaacgc
aaac---aggaccatagtgggggattct--------------------------------
-atc--

>_R_gnl|SRA|SRR2638781.49835172.1 HWI-D00731:29:HGNNYADXX:1:2105:14889:92595.
---------------------gagaggtacaaga--------------------------
aggcaaacagtgacacctccaactctggcacagttgcagaagt----caatgc-------
ccagcactaccagcaggagtcctccaaactgcgccaacaaatcagtagcttacagaacgc
aaac---aggaccatagtgggggattct--------------------------------
-atc--

>_R_gnl|SRA|SRR2638781.48471543.1 HWI-D00731:29:HGNNYADXX:1:2104:1764:92496.
---------------------gagaggtacaaga--------------------------
aggcaaacagtgacacctccaactctggcacagttgcagaagt----caatgc-------
ccagcactaccagcaggagtcctccaaactgcgccaacaaatcagtagcttacagaacgc
aaac---aggaccatagtgggggattct--------------------------------
-atc--

>_R_gnl|SRA|SRR2638781.56953050.1 HWI-D00731:29:HGNNYADXX:1:2111:7308:3362.
----------------------agaggtacaaga--------------------------
aggcaaacagtgacacctccaactctggcacagttgcagaagt----caatgc-------
ccagcactaccagcaggagtcctccaaactgcgccaacaaatcagtagcttacagaacgc
aaac---aggaccatagtgggggattct--------------------------------
-atca---

>_R_gnl|SRA|SRR2638781.53739897.1 HWI-D00731:29:HGNNYADXX:1:2108:4420:76920.
----------------------agaggtacaaga--------------------------
aggcaaacagtgacacctccaactctggcacagttgcagaagt----caatgc-------
ccagcactaccagcaggagtcctccaaactgcgccaacaaatcagtagcttacagaacgc
aaac---aggaccatagtgggggattct--------------------------------
-atca---

>_R_gnl|SRA|SRR2638781.43705967.1 HWI-D00731:29:HGNNYADXX:1:2101:5271:26165.
----------------------agaggtacaaga--------------------------
aggcaaacagtgacacctccaactctggcacagttgcagaagt----caatgc-------

ccagcactaccagcaggagtcctccaaactgcgccaacaaatcagtagcttacagaacgc
aaac---aggaccatagtgggggattct--------------------------------
-atca---

>_R_gnl|SRA|SRR2638781.47616702.1 HWI-D00731:29:HGNNYADXX:1:2104:13354:29164.
------------------------aggtacaaga--------------------------
aggcaaacagtgacacctccaactctggcacagttgcagaagt----caatgc-------
ccagcactaccagcaggagtcctccaaactgcgccaacaaatcagtagcttacagaacgc
aaac---aggaccatagtgggggattct--------------------------------
-atcaac---

>gnl|SRA|SRR2638781.37692687.2 HWI-D00731:29:HGNNYADXX:1:1213:13202:12432.
--------------------------gtacaaga--------------------------
aggcaaacagtgacacctccaactctggcacagttgcagaagt----caatgc-------
ccagcactaccagcaggagtcctccaaactgcgccaacaaatcagtagcttacagaacgc
aaac---aggaccatagtgggggattct--------------------------------
-atcaacac---

>gnl|SRA|SRR2638781.24492243.2 HWI-D00731:29:HGNNYADXX:1:1203:3988:13480.
--------------------------gtacaaga--------------------------
aggcaaacagtgacacctccaactctggcacagttgcagaagt----caatgc-------
ccagcactaccagcaggagtcctccaaactgcgccaacaaatcagtagcttacagaacgc
aaac---aggaccatagtgggggattct--------------------------------
-atcaacac---

>gnl|SRA|SRR2638781.63786640.2 HWI-D00731:29:HGNNYADXX:1:2115:16618:65267.
-----------------------gaggtacaaga--------------------------
aggcaaacagtgacacctccaactctggcacagttggagaaga----caatgc-------
ccagcactaccagcaggcgtcctccaaactgcgactacaaatcagtagcttacagaacgc
aagcagtaggaccatagtgggggcttct--------------------------------
-at---

>gnl|SRA|SRR2638781.39979017.2 HWI-D00731:29:HGNNYADXX:1:1214:19466:70150.
------------------gttgagaggtacaaga--------------------------
aggcaaacagtgacacctccaactctggcacagttgcagaagt----caatgc-------
ccagcactaccagcaggagtcctccaaactgcgccaacaaatcagtagcttacagaacgc
aaacagtaggaccatagtgggggat-----------------------------------

>_R_gnl|SRA|SRR2638781.72239995.1 HWI-D00731:29:HGNNYADXX:1:2206:1952:9008.
----------------ccgttgagaggtacaaga--------------------------
aggcaaacagtgacacctccaactctggcacagttgcagaagt----caatgc-------
ccagcactaccagcaggagtcctccaaactgcgccaacaaatcagtagcttacagaacgc
aaacagtaggaccatagtggggg-------------------------------------

>_R_gnl|SRA|SRR2638781.47325708.1 HWI-D00731:29:HGNNYADXX:1:2104:10175:7550.
----------------ccgttgagaggtacaaga--------------------------
aggcaaacagtgacacctccaactctggcacagttgcagaagt----caatgc-------
ccagcactaccagcaggagtcctccaaactgcgccaacaaatcagtagcttacagaacgc
aaacagtaggaccatagtggggg-------------------------------------

>_R_gnl|SRA|SRR2638781.26052062.1 HWI-D00731:29:HGNNYADXX:1:1204:17557:52674.
----------------ccgttgagaggtacaaga--------------------------
aggcaaacagtgacacctccaactctggcacagttgcagaagt----caatgc-------
ccagcactaccagcaggagtcctccaaactgcgccaacaaatcagtagcttacagaacgc
aaacagtaggaccatagtggggg-------------------------------------

>_R_gnl|SRA|SRR2638781.10921768.1 HWI-D00731:29:HGNNYADXX:1:1109:15674:35889.
----------------ccgttgagaggtacaaga--------------------------
aggcaaacagtgacacctccaactctggcacagttgcagaagt----caatgc-------
ccagcactaccagcaggagtcctccaaactgcgccaacaaatcagtagcttacagaacgc
aaacagtaggaccatagtggggg-------------------------------------

>_R_gnl|SRA|SRR2638781.69165444.1 HWI-D00731:29:HGNNYADXX:1:2203:7958:74209.
----------------ccgttgagaggtacaaga--------------------------
aggcaaacagtgacacctccaactctggcacagttgcagaagt----caatgc-------
ccagcactaccagcaggagtcctccaaactgcgccaacaaatcagtagcttacagaacgc
aaacagtaggaccatagcagggg-------------------------------------

>_R_gnl|SRA|SRR2638781.44386627.1 HWI-D00731:29:HGNNYADXX:1:2101:1960:78174.
----------------ccgttgagaggtacaaga--------------------------
aggcaaacagtgacacctccaactctggcacagttgcagaagt----caatgc-------
ccagcactaccagcaggagtcctccaaactgcgccaacaaatcagtagcttacagaacgc
aaacagtaggaccatagtgcggg-------------------------------------

>_R_gnl|SRA|SRR2638781.18601316.1 HWI-D00731:29:HGNNYADXX:1:1114:10466:79984.
-----------------cgttgagaggtacaaga--------------------------
aggcaaacagtgacacctccaactctggcacagttgcagaagt----caatgc-------
ccagcactaccagcaggagtcctccaaactgcgccaacaaatcagtagcttacagaacgc
aaacagtaggaccatagtgggggg------------------------------------

>_R_gnl|SRA|SRR2638781.49136063.1 HWI-D00731:29:HGNNYADXX:1:2105:21044:41514.

---------------accgttgagaggtacaaga--------------------------
aggcaaacagtgacacctccaactctggcacagttgcagaagt----caatgc-------
ccagcactaccagcaggagtcctccaaactgcgccaacaaatcagtagcttacagaacgc
aaacagtaggaccatagtgggg--------------------------------------

>_R_gnl|SRA|SRR2638781.9241463.1 HWI-D00731:29:HGNNYADXX:1:1108:8492:13583.
---------------accgttgagaggtacaaga--------------------------
aggcaaacagtgacacctccaactctggcacagttgcagaagt----caatgc-------
ccagcactaccagcaggagtcctccaaactgcgccaacaaatcagtagcttacagaacgc
aaacagtaggaccatagtgggg--------------------------------------

>_R_gnl|SRA|SRR2638781.84711822.1 HWI-D00731:29:HGNNYADXX:1:2214:18683:97963.
---------------accgttgagaggtacaaga--------------------------
aggcaaacagtgacacctccaactctggcacagttgcagaagt----caatgc-------
ccagcactaccagcaggagtcctccaaactgcgccaacaaatcagtagcttacagaacgc
aaacagtaggaccatagcgggg--------------------------------------

>_R_gnl|SRA|SRR2638781.18759801.1 HWI-D00731:29:HGNNYADXX:1:1114:18250:90672.
---------------accgttgagaggtacaaga--------------------------
aggcaaacagtgacacctccaactctggcacagttgcagaagt----caatgc-------
ccagcactaccagcaggagtcctccaaactgcgccaacaaatcagtagcttacagaacgc
aaacagtaggaccatagcgggg--------------------------------------

>_R_gnl|SRA|SRR2638781.10298868.1 HWI-D00731:29:HGNNYADXX:1:1108:9746:90327.
---------------accgttgagaggtacaaga--------------------------
aggcaaacagtgacacctccaactctggcacagttgcagaagt----caatgc-------
ccagcactaccagcaggagtcctccaaactgcgccaacaaatcagtagcttacagaacgc
aaacagtaggaccatagcgggg--------------------------------------

>_R_gnl|SRA|SRR2638781.60118381.1 HWI-D00731:29:HGNNYADXX:1:2113:7116:20827.
---------------accgttgagaggtacaaga--------------------------
aggcaaacagtgccacctccaactctggcacagttgcagaagt----caatgc-------
ccagcactaccagcaggagtcctccaaactgcgccaacaaatcagtagcttacagaacgc
aaacagtaggaccatagtgcgg--------------------------------------

>gnl|SRA|SRR2638781.60625520.2 HWI-D00731:29:HGNNYADXX:1:2113:6120:55166.
--------------caccgttgagaggtacaaga--------------------------
aggcaaacagtgacacctccaactctggcacagttgcagaagt----caatgc-------
ccagcactaccagcaggagtcctccaaactgcgccaacaaatcagtagcttacagaacgc

aaacagtaggaccatagtggg---------------------------------------

>_R_gnl|SRA|SRR2638781.53983322.1 HWI-D00731:29:HGNNYADXX:1:2108:18641:93755.
--------------caccgttgagaggtacaaga--------------------------
aggcaaacagtgacacctccaactctggcacagttgcagaagt----caatgc-------
ccagcactaccagcaggagtcctccaaactgcgccaacaaatcagtagcttacagaacgc
aaacagtaggaccatagtggg---------------------------------------

>gnl|SRA|SRR2638781.10874647.2 HWI-D00731:29:HGNNYADXX:1:1109:4157:32611.
--------------caccgttgagaggtacaaga--------------------------
aggcaaacagtgacacctccaactctggcacagttgcagaagt----caatgc-------
ccagcactaccagcaggagtcctccaaactgcgccaacaaatcagtagcttacagaacgc
aaacagtaggaccatagtggg---------------------------------------

>gnl|SRA|SRR2638781.27459705.2 HWI-D00731:29:HGNNYADXX:1:1205:10311:61177.
-------------ccaccgttgagaggtacaaga--------------------------
aggcaaacagtgacacctccaactctggcacagttgcagaagt----caatgc-------
ccagcactaccagcaggagtcctccaaactgcgccaacaaatcagtagcttacagaacgc
aaacagtaggaccatagtgg--

>gnl|SRA|SRR2638781.10281006.2 HWI-D00731:29:HGNNYADXX:1:1108:6993:89133.
-------------ccaccgttgagaggtacaaga--------------------------
aggcaaacagtgacacctccaactctggcacagttgcagaagt----caatgc-------
ccagcactaccagcaggagtcctccaaactgcgccaacaaatcagtagcttacagaacgc
aaacagtaggaccatagtgg--

>gnl|SRA|SRR2638781.18759801.2 HWI-D00731:29:HGNNYADXX:1:1114:18250:90672.
-------------ccaccgttgagaggtacaaga--------------------------
aggcaaacagtgacacctccaactctggcacagttgcagaagt----caatgc-------
ccagcactaccagcaggagtcctccaaactgcgccaacaaatcagtagcttacagaacgc
aaacagtaggaccatagcgg--

>_R_gnl|SRA|SRR2638781.84017022.1 HWI-D00731:29:HGNNYADXX:1:2214:14008:51317.
-------------ccaccgttgagaggtacaaga--------------------------
aggcaaacagtgacacctccaactctggcacagttgcagaagt----caatgc-------
ccagcactaccagcaggagtcctccaaactgcgccaacaaatcagtagcttacagaacgc
aaacagtaggaccatagggg--

>_R_gnl|SRA|SRR2638781.12348820.1 HWI-D00731:29:HGNNYADXX:1:1110:9099:38769.
------------tccaccgttgagaggtacaaga--------------------------
aggcaaacagtgacacctccaactctggcacagttgcagaagt----caatgc-------
ccagcactaccagcaggagtcctccaaactgcgccaacaaatcagtagcttacagaacgc
aaacagtaggaccatagtg---

>gnl|SRA|SRR2638781.75974191.2 HWI-D00731:29:HGNNYADXX:1:2208:4395:85760.
-----------atccaccgttgagaggtacaaga--------------------------
aggcaaacagtgacacctccaactctggcacagttgcagaagt----caatgc-------
ccagcactaccagcaggagtcctccaaactgcgccaacaaatcagtagcttacagaacgc
aaacagtaggaccatagt--

>gnl|SRA|SRR2638781.6788696.2 HWI-D00731:29:HGNNYADXX:1:1106:5109:29307.
-----------atccaccgttgagaggtacaaga--------------------------
aggcaaacagtgacacctccaactctggcacagttgcagaagt----caatgc-------
ccagcactaccagcaggagtcctccaaactgcgccaacaaatcagtagcttacagaacgc
aaacagtaggaccatagt--

>_R_gnl|SRA|SRR2638781.27338972.1 HWI-D00731:29:HGNNYADXX:1:1205:16822:51966.
---------aaatccaccgttgagaggtacaaga--------------------------
aggcaaacagtgacacctccaactctggcacagttgcagaagt----caatgc-------
ccagcactaccagcaggagtcctccaaactgcgccaacaaatcagtagcttacagaacgc
aaacagtaggaccata--

>gnl|SRA|SRR2638781.58927574.2 HWI-D00731:29:HGNNYADXX:1:2112:13716:39358.
--------------caccgttgagaggtacaaga--------------------------
aggcaaacagtgacacctccaactctggcacagttgcagaagt----caatgc-------
ccagcactaccagcaggagtcctccaaactgcgccaacaaatcagtagcttacagaacgc
aaac---aggaccatagtggggga------------------------------------

>gnl|SRA|SRR2638781.50947698.2 HWI-D00731:29:HGNNYADXX:1:2106:12230:74051.
--------------caccgttgagaggtacaaga--------------------------
aggcaaacagtgacacctccaactctggcacagttgcagaagt----caatgc-------
ccagcactaccagcaggagtcctccaaactgcgccaacaaatcagtagcttacagaacgc
aaac---aggaccatagtggggga------------------------------------

>gnl|SRA|SRR2638781.27979551.2 HWI-D00731:29:HGNNYADXX:1:1205:17687:100302.
--------------caccgttgagaggtacaaga--------------------------

aggcaaacagtgacacctccaactctggcacagttgcagaagt----caatgc-------
ccagcactaccagcaggagtcctccaaactgcgccaacaaatcagtagcttacagaacgc
aaac---aggaccatagtggggga--

>gnl|SRA|SRR2638781.11070793.2 HWI-D00731:29:HGNNYADXX:1:1109:12911:46643.
----------------caccgttgagaggtacaaga------------------------------
aggcaaacagtgacacctccaactctggcacagttgcagaagt----caatgc-------
ccagcactaccagcaggagtcctccaaactgcgccaacaaatcagtagcttacagaacgc
aaac---aggaccatagtggggga--

>_R_gnl|SRA|SRR2638781.48534493.1 HWI-D00731:29:HGNNYADXX:1:2104:12502:96966.
---------------ccaccgttgagaggtacaaga------------------------------
aggcaaacagtgacacctccaactctggcacagttgcagaagt----caatgc-------
ccagcactaccagcaggagtcctccaaactgcgccaacaaatcagtagcttacagaacgc
aaac---aggaccatagtggggg---

>_R_gnl|SRA|SRR2638781.17074190.1 HWI-D00731:29:HGNNYADXX:1:1113:11272:73720.
---------------ccaccgttgagaggtacaaga------------------------------
aggcaaacagtgacacctccaactctggcacagttgcagaagt----caatgc-------
ccagcactaccagcaggagtcctccaaactgcgccaacaaatcagtagcttacagaacgc
aaac---aggaccatagtggggg---

>gnl|SRA|SRR2638781.1514671.2 HWI-D00731:29:HGNNYADXX:1:1102:3012:20168.
---------------ccaccgttgagaggtacaaga------------------------------
aggcaaacagtgacacctccaactctggcacagttgcagaagt----caatgc-------
ccagcactaccagcaggagtcctccaaactgcgccaacaaatcagtagcttacagaacgc
aaac---aggaccatagtggggg---

>gnl|SRA|SRR2638781.1042207.2 HWI-D00731:29:HGNNYADXX:1:1101:3978:83123.
---------------ccaccgttgagaggtacaaga------------------------------
aggcaaacagtgacacctccaactctggcacagttgcagaagt----caatgc-------
ccagcactaccagcaggagtcctccaaactgcgccaacaaatcagtagcttacagaacgc
aaac---aggaccatagtggggg---

>_R_gnl|SRA|SRR2638781.64680084.1 HWI-D00731:29:HGNNYADXX:1:2116:5991:24633.
---------------ccaccgttgagaggtacaaga------------------------------
aggcaaacagtgacacctccaactctggcacagttgcagaagt----caatgc-------
ccagcactaccagcaggagtcctccaaactgcgccaacaaatcagtagcttacagaacgc
aaac---aggaccatagtgggtg---

>_R_gnl|SRA|SRR2638781.83699998.1 HWI-D00731:29:HGNNYADXX:1:2214:19565:29913.
-----------atccaccgttgagaggtacaaga--------------------------
aggcaaacagtgacacctccaactctggcacagttgcagaagt----caatgc-------
ccagcactaccagcaggagtcctccaaactgcgccaacaaatcagtagcttacagaacgc
aaac---aggaccatagtggg---------------------------------------

>_R_gnl|SRA|SRR2638781.65441322.1 HWI-D00731:29:HGNNYADXX:1:2116:12154:74683.
-----------atccaccgttgagaggtacaaga--------------------------
aggcaaacagtgacacctccaactctggcacagttgcagaagt----caatgc-------
ccagcactaccagcaggagtcctccaaactgcgccaacaaatcagtagcttacagaacgc
aaac---aggaccatagtggg---------------------------------------

>_R_gnl|SRA|SRR2638781.53747514.1 HWI-D00731:29:HGNNYADXX:1:2108:6930:77460.
-----------atccaccgttgagaggtacaaga--------------------------
aggcaaacagtgacacctccaactctggcacagttgcagaagt----caatgc-------
ccagcactaccagcaggagtcctccaaactgcgccaacaaatcagtagcttacagaacgc
aaac---aggaccatagtggg---------------------------------------

>_R_gnl|SRA|SRR2638781.25590072.1 HWI-D00731:29:HGNNYADXX:1:1204:15708:16841.
-----------atccaccgttgagaggtacaaga--------------------------
aggcaaacagtgacacctccaactctggcacagttgcagaagt----caatgc-------
ccagcactaccagcaggagtcctccaaactgcgccaacaaatcagtagcttacagaacgc
aaac---aggaccatagtggg---------------------------------------

>_R_gnl|SRA|SRR2638781.6504179.1 HWI-D00731:29:HGNNYADXX:1:1106:17530:8223.
-----------atccaccgttgagaggtacaaga--------------------------
aggcaaacagtgacacctccaactctggcacagttgcagaagt----caatgc-------
ccagcactaccagcaggagtcctccaaactgcgccaacaaatcagtagcttacagaacgc
aaac---aggaccatagtggg---------------------------------------

>gnl|SRA|SRR2638781.76477549.2 HWI-D00731:29:HGNNYADXX:1:2209:16656:22744.
------gtgaaatccaccgttgagaggtacaaga--------------------------
aggcaaacagtgacacctccaactctggcacagttgcagaagt----caatgc-------
ccagcactaccagcaggagtcctccaaactgcgccaacaaatcagtagcttacagaacgc
aaacagtaggacc---

>gnl|SRA|SRR2638781.66088400.2 HWI-D00731:29:HGNNYADXX:1:2201:14466:20098.
------gtgaaatccaccgttgagaggtacaaga--------------------------
aggcaaacagtgacacctccaactctggcacagttgcagaagt----caatgc-------
ccagcactaccagcaggagtcctccaaactgcgccaacaaatcagtagcttacagaacgc
aaacagtaggacc---

>gnl|SRA|SRR2638781.65201609.2 HWI-D00731:29:HGNNYADXX:1:2116:19113:58873.
------gtgaaatccaccgttgagaggtacaaga--------------------------
aggcaaacagtgacacctccaactctggcacagttgcagaagt----caatgc-------
ccagcactaccagcaggagtcctccaaactgcgccaacaaatcagtagcttacagaacgc
aaacagtaggacc---

>gnl|SRA|SRR2638781.62683417.2 HWI-D00731:29:HGNNYADXX:1:2114:16175:92304.
------gtgaaatccaccgttgagaggtacaaga--------------------------
aggcaaacagtgacacctccaactctggcacagttgcagaagt----caatgc-------
ccagcactaccagcaggagtcctccaaactgcgccaacaaatcagtagcttacagaacgc
aaacagtaggacc---

>gnl|SRA|SRR2638781.50529323.2 HWI-D00731:29:HGNNYADXX:1:2106:9077:43980.
------gtgaaatccaccgttgagaggtacaaga--------------------------
aggcaaacagtgacacctccaactctggcacagttgcagaagt----caatgc-------
ccagcactaccagcaggagtcctccaaactgcgccaacaaatcagtagcttacagaacgc
aaacagtaggacc---

>gnl|SRA|SRR2638781.34446666.2 HWI-D00731:29:HGNNYADXX:1:1210:13568:81100.
------gtgaaatccaccgttgagaggtacaaga--------------------------
aggcaaacagtgacacctccaactctggcacagttgcagaagt----caatgc-------
ccagcactaccagcaggagtcctccaaactgcgccaacaaatcagtagcttacagaacgc
aaacagtaggacc---

>gnl|SRA|SRR2638781.18410222.2 HWI-D00731:29:HGNNYADXX:1:1114:14503:66656.
------gtgaaatccaccgttgagaggtacaaga--------------------------
aggcaaacagtgacacctccaactctggcacagttgcagaagt----caatgc-------
ccagcactaccagcaggagtcctccaaactgcgccaacaaatcagtagcttacagaacgc
aaacagtaggacc---

>gnl|SRA|SRR2638781.17029223.2 HWI-D00731:29:HGNNYADXX:1:1113:5721:70558.
------gtgaaatccaccgttgagaggtacaaga--------------------------
aggcaaacagtgacacctccaactctggcacagttgcagaagt----caatgc-------

ccagcactaccagcaggagtcctccaaactgcgccaacaaatcagtagcttacagaacgc
aaacagtaggacc---

>gnl|SRA|SRR2638781.6544257.2 HWI-D00731:29:HGNNYADXX:1:1106:16896:11239.
------gtgaaatccaccgttgagaggtacaaga--------------------------
aggcaaacagtgacacctccaactctggcacagttgcagaagt----caatgc-------
ccagcactaccagcaggagtcctccaaactgcgccaacaaatcagtagcttacagaacgc
aaacagtaggacc---

>gnl|SRA|SRR2638781.53538542.2 HWI-D00731:29:HGNNYADXX:1:2108:18147:62312.
------gtgaaatccaccgttgagaggtacaaga--------------------------
aggcaaacagtgacacctccaactctggcacagttgcagaagt----caatgc-------
ccagcactaccagcaggagtcctccaaactgcgccaacgaatcagtagcttacagaacgc
aaacagtaggacc---

>gnl|SRA|SRR2638781.75049397.2 HWI-D00731:29:HGNNYADXX:1:2208:12968:18585.
------gtgaaatccaccgttgagaggtacaaga--------------------------
aggcaaacagtgacacctccaactctggcacagttgcagaagt----caatgc-------
ccagcactaccagcaggagtcctccaaactgcgccaacaaatcagtagcttacagaacgc
aaacagtaagatc---

>gnl|SRA|SRR2638781.52391632.2 HWI-D00731:29:HGNNYADXX:1:2107:18400:79795.
------gtgaaatccaccgttgagaggtacaaga--------------------------
aggcaaacagtgacacctccaactctggcacagttgcagaagt----caatgc-------
ccagcactaccagcaggagtcctccaaactgcgtcaacaaatcagtagcttacagaacgc
aaacagtaagatc---

>gnl|SRA|SRR2638781.61440537.2 HWI-D00731:29:HGNNYADXX:1:2114:19644:10175.
------gtgaaatccaccgttgagaggtacaaga--------------------------
aggcaaacagtgacacctccaactctggcacagttgcagaagt----caatgc-------
ccagcactaccagcaggagtcctccaaactgcgccaacaaatcagtagcttacagaacgc
aaacagtaggaga---

>_R_gnl|SRA|SRR2638781.38016434.1 HWI-D00731:29:HGNNYADXX:1:1213:15309:34901.
-----tgtgaaatccaccgttgagaggtacaaga--------------------------
aggcaaacagtgacacctccaactctggcacagttgcagaagt----caatgc-------
ccagcactaccagcaggagtcctccaaactgcgccaacaaatcagtagcttacagaacgc
aaacagtaggac--

>_R_gnl|SRA|SRR2638781.29397614.1 HWI-D00731:29:HGNNYADXX:1:1207:10585:8547.
-----tgtgaaatccaccgttgagaggtacaaga-------------------------
aggcaaacagtgacacctccaactctggcacagttgcagaagt----caatgc-------
ccagcactaccagcaggagtcctccaaactgcgccaacaaatcagtagcttacagaacgc
aaacagtaggac--

>_R_gnl|SRA|SRR2638781.50360769.1 HWI-D00731:29:HGNNYADXX:1:2106:8868:31542.
----gtgtgaaatccaccgttgagaggtacaaga-------------------------
aggcaaacagtgacacctccaactctggcacagttgcagaagt----caatgc-------
ccagcactaccagcaggagtcctccaaactgcgccaacaaatcagtagcttacagaacgc
aaacagtagga---

>_R_gnl|SRA|SRR2638781.86297531.1 HWI-D00731:29:HGNNYADXX:1:2216:2861:4960.
---agtgtgaaatccaccgttgagaggtacaaga-------------------------
aggcaaacagtgacacctccaactctggcacagttgcagaagt----caatgc-------
ccagcactaccagcaggagtcctccaaactgcgccaacaaatcagtagcttacagaacgc
aaacagtagg--

>_R_gnl|SRA|SRR2638781.77964019.1 HWI-D00731:29:HGNNYADXX:1:2210:6024:29866.
---agtgtgaaatccaccgttgagaggtacaaga-------------------------
aggcaaacagtgacacctccaactctggcacagttgcagaagt----caatgc-------
ccagcactaccagcaggagtcctccaaactgcgccaacaaatcagtagcttacagaacgc
aaacagtagg--

>_R_gnl|SRA|SRR2638781.69905911.1 HWI-D00731:29:HGNNYADXX:1:2204:14333:31369.
---agtgtgaaatccaccgttgagaggtacaaga-------------------------
aggcaaacagtgacacctccaactctggcacagttgcagaagt----caatgc-------
ccagcactaccagcaggagtcctccaaactgcgccaacaaatcagtagcttacagaacgc
aaacagtagg--

>_R_gnl|SRA|SRR2638781.53486786.1 HWI-D00731:29:HGNNYADXX:1:2108:2395:58799.
---agtgtgaaatccaccgttgagaggtacaaga-------------------------
aggcaaacagtgacacctccaactctggcacagttgcagaagt----caatgc-------
ccagcactaccagcaggagtcctccaaactgcgccaacaaatcagtagcttacagaacgc
aaacagtagg--

>_R_gnl|SRA|SRR2638781.61440537.1 HWI-D00731:29:HGNNYADXX:1:2114:19644:10175.

---tctgtgaaatccaccgttgagaggtacaaga-------------------------
aggcaaacagtgacacctccaactctggcacagttgcagaagt----caatgc-------
ccagcactaccagcaggagtcctccaaactgcgccaacaaatcagtagcttacagaacgc
aaacagtagg--

>_R_gnl|SRA|SRR2638781.86669517.1 HWI-D00731:29:HGNNYADXX:1:2216:4281:29480.
--cagtgtgaaatccaccgttgagaggtacaaga--------------------------
aggcaaacagtgacgcctccaactctggcacagttgcagaagt----caatgc-------
ccagcactaccagcaggagtcctccaaactgcgccaacaaatcagtagcttacagaacgc
aaacagtag---

>_R_gnl|SRA|SRR2638781.78077784.1 HWI-D00731:29:HGNNYADXX:1:2210:10900:37997.
--cagtgtgaaatccaccgttgagaggtacaaga--------------------------
aggcaaacagtgacacctccaactctggcacagttgcagaagt----caatgc-------
ccagcactaccagcaggagtcctccaaactgcgccaacaaatcagtagcttacagaacgc
aaacagtag---

>_R_gnl|SRA|SRR2638781.71070040.1 HWI-D00731:29:HGNNYADXX:1:2205:2626:20719.
--cagtgtgaaatccaccgttgagaggtacaaga--------------------------
aggcaaacagtgacacctccaactctggcacagttgcagaagt----caatgc-------
ccagcactaccagcaggagtcctccaaactgcgccaacaaatcagtagcttacagaacgc
aaacagtag---

>_R_gnl|SRA|SRR2638781.68427801.1 HWI-D00731:29:HGNNYADXX:1:2203:14260:3695.
--cagtgtgaaatccaccgttgagaggtacaaga--------------------------
aggcaaacagtgacacctccaactctggcacagttgcagaagt----caatgc-------
ccagcactaccagcaggagtcctccaaactgcgccaacaaatcagtagcttacagaacgc
aaacagtag---

>_R_gnl|SRA|SRR2638781.68318999.1 HWI-D00731:29:HGNNYADXX:1:2202:19791:94685.
--cagtgtgaaatccaccgttgagaggtacaaga--------------------------
aggcaaacagtgacacctccaactctggcacagttgcagaagt----caatgc-------
ccagcactaccagcaggagtcctccaaactgcgccaacaaatcagtagcttacagaacgc
aaacagtag---

>_R_gnl|SRA|SRR2638781.66972882.1 HWI-D00731:29:HGNNYADXX:1:2201:3147:89842.
--cagtgtgaaatccaccgttgagaggtacaaga--------------------------
aggcaaacagtgacacctccaactctggcacagttgcagaagt----caatgc-------
ccagcactaccagcaggagtcctccaaactgcgccaacaaatcagtagcttacagaacgc

aaacagtag---

>_R_gnl|SRA|SRR2638781.64044264.1 HWI-D00731:29:HGNNYADXX:1:2115:19087:82314.
--cagtgtgaaatccaccgttgagaggtacaaga--------------------------
aggcaaacagtgacacctccaactctggcacagttgcagaagt----caatgc-------
ccagcactaccagcaggagtcctccaaactgcgccaacaaatcagtagcttacagaacgc
aaacagtag---

>_R_gnl|SRA|SRR2638781.59429532.1 HWI-D00731:29:HGNNYADXX:1:2112:17633:73279.
--cagtgtgaaatccaccgttgagaggtacaaga--------------------------
aggcaaacagtgacacctccaactctggcacagttgcagaagt----caatgc-------
ccagcactaccagcaggagtcctccaaactgcgccaacaaatcagtagcttacagaacgc
aaacagtag---

>_R_gnl|SRA|SRR2638781.56698267.1 HWI-D00731:29:HGNNYADXX:1:2110:18994:84950.
--cagtgtgaaatccaccgttgagaggtacaaga--------------------------
aggcaaacagtgacacctccaactctggcacagttgcagaagt----caatgc-------
ccagcactaccagcaggagtcctccaaactgcgccaacaaatcagtagcttacagaacgc
aaacagtag---

>_R_gnl|SRA|SRR2638781.48164056.1 HWI-D00731:29:HGNNYADXX:1:2104:15909:69537.
--cagtgtgaaatccaccgttgagaggtacaaga--------------------------
aggcaaacagtgacacctccaactctggcacagttgcagaagt----caatgc-------
ccagcactaccagcaggagtcctccaaactgcgccaacaaatcagtagcttacagaacgc
aaacagtag---

>_R_gnl|SRA|SRR2638781.38554277.1 HWI-D00731:29:HGNNYADXX:1:1213:5161:72201.
--cagtgtgaaatccaccgttgagaggtacaaga--------------------------
aggcaaacagtgacacctccaactctggcacagttgcagaagt----caatgc-------
ccagcactaccagcaggagtcctccaaactgcgccaacaaatcagtagcttacagaacgc
aaacagtag---

>_R_gnl|SRA|SRR2638781.29320660.1 HWI-D00731:29:HGNNYADXX:1:1207:15818:2844.
--cagtgtgaaatccaccgttgagaggtacaaga--------------------------
aggcaaacagtgacacctccaactctggcacagttgcagaagt----caatgc-------
ccagcactaccagcaggagtcctccaaactgcgccaacaaatcagtagcttacagaacgc
aaacagtag---

>_R_gnl|SRA|SRR2638781.27840944.1 HWI-D00731:29:HGNNYADXX:1:1205:2018:90233.
--cagtgtgaaatccaccgttgagaggtacaaga---------------------------
aggcaaacagtgacacctccaactctggcacagttgcagaagt----caatgc-------
ccagcactaccagcaggagtcctccaaactgcgccaacaaatcagtagcttacagaacgc
aaacagtag--

>_R_gnl|SRA|SRR2638781.16597200.1 HWI-D00731:29:HGNNYADXX:1:1113:14515:40673.
--cagtgtgaaatccaccgttgagaggtacaaga---------------------------
aggcaaacagtgacacctccaactctggcacagttgcagaagt----caatgc-------
ccagcactaccagcaggagtcctccaaactgcgccaacaaatcagtagcttacagaacgc
aaacagtag--

>_R_gnl|SRA|SRR2638781.5727152.1 HWI-D00731:29:HGNNYADXX:1:1105:14756:49501.
--cagtgtgaaatccaccgttgagaggtacaaga---------------------------
aggcaaacagtgacacctccaactctggcacagttgcagaagt----caatgc-------
ccagcactaccagcaggagtcctccaaactgcgccaacaaatcagtagcttacagaacgc
aaacagtag--

>_R_gnl|SRA|SRR2638781.5607143.1 HWI-D00731:29:HGNNYADXX:1:1105:19557:40544.
--cagtgtgaaatccaccgttgagaggtacaaga---------------------------
aggcaaacagtgacacctccaactctggcacagttgcagaagt----caatgc-------
ccagcactaccagcaggagtcctccaaactgcgccaacaaatcagtagcttacagaacgc
aaacagtag--

>_R_gnl|SRA|SRR2638781.26015197.1 HWI-D00731:29:HGNNYADXX:1:1204:10912:49853.
--cagtgtgaaatccaccgttgagaggtacaaga---------------------------
aggcaaacagtgacacctccaactctggcacagttgcagaagt----caattc-------
ccagcactaccagcaggagtcctccaaactgcgccaacaaatcagtagcttacagaacgc
aaacagtag--

>gnl|SRA|SRR2638781.59321892.2 HWI-D00731:29:HGNNYADXX:1:2112:14917:66121.
aacagtgtgaaatccaccgttgagaggtacaaga---------------------------
aggcaaacagtgacacctccaactctggcacagttgcagaagt----caatgc-------
ccagcactaccagcaggagtcctccaaactgcgccaacaaatcagtagcttacagaacgc
aaacagt--

>gnl|SRA|SRR2638781.37157972.2 HWI-D00731:29:HGNNYADXX:1:1212:9028:74476.
aacagtgtgaaatccaccgttgagaggtacaaga---------------------------

aggcaaacagtgacacctccaactctggcacagttgcagaagt----caatgc-------
ccagcactaccagcaggagtcctccaaactgcgccaacaaatcagtagcttacagaacgc
aaacagt---

>_R_gnl|SRA|SRR2638781.75049397.1 HWI-D00731:29:HGNNYADXX:1:2208:12968:18585.
-gatctgtgaaatccaccgttgagaggtacaaga--------------------------
aggcaaacagtgacacctccaactctggcacagttgcagaagt----caatgc-------
ccagcactaccagcaggagtcctccaaactgcgccaacaaatcagtagcttacagaacgc
aaacagta--

>_R_gnl|SRA|SRR2638781.52391632.1 HWI-D00731:29:HGNNYADXX:1:2107:18400:79795.
-gatctgtgaaatccaccgttgagaggtacaaga--------------------------
aggcaaacagtgacacctccaactctggcacagttgcagaagt----caatgc-------
ccagcactaccagcaggagtcctccaaactgcgccaacaaatcagtagcttacagaacgc
aaacagta--

>gnl|SRA|SRR2638781.85031470.2 HWI-D00731:29:HGNNYADXX:1:2215:11370:19894.

----------------------------------caacaaatcagtagcttacagaacgc
aaacagtaggaccatagtgggggattct--------------------------------
-atcaacaccatgagcctcagggaccttaaacaggtagagaacaggctggagaaagg---
-----catagctaagataag--ggctagaaagaatgagctgttatat-------------

>gnl|SRA|SRR2638781.2766622.2 HWI-D00731:29:HGNNYADXX:1:1103:8277:16762.

----------------------------------caacaaatcagtagcttacagaacgc
aaacagtaggaccatagtgggggattct--------------------------------
-atcaacaccatgagcctcagggaccttaaacaggtagagaacaggctggagaaagg---
-----catagctaagataag--ggctagaaagaatgagctgttatat-------------

>gnl|SRA|SRR2638781.85021018.2 HWI-D00731:29:HGNNYADXX:1:2215:16784:19139.

------------------------------------acaaatcagtagcttacagaacgc
aaacagtaggaccatagtgggggattct--------------------------------
-atcaacaccatgagcctcagggaccttaaacaggtagagaacaggctggagaaagg---
-----catagctaagataag--ggctagaaagaatgagctgttatatgc-----------

>_R_gnl|SRA|SRR2638781.39518020.1 HWI-D00731:29:HGNNYADXX:1:1214:20316:38605.

------------------------------------acaaatcagtagcttacagaacgc
aaacagtaggaccatagtgggggattct--------------------------------

-atcaacaccatgagcctcagggaccttaaacaggtagagaacaggctggagaaagg---
-----aatagctaagataag--ggctagaaagaatgagctgttatatgc-----------

>gnl|SRA|SRR2638781.73529171.2 HWI-D00731:29:HGNNYADXX:1:2207:19434:5272.

-------------------------------------caaatcagtagcttacagaacgc
aaacagtaggaccatagtgggggattct--------------------------------
-atcaacaccatgagcctcagggaccttaaacaggtagagaacaggctggagaaagg---
-----catagctaagataag--ggctagaaagaatgagctgttatatgct----------

>gnl|SRA|SRR2638781.62546165.2 HWI-D00731:29:HGNNYADXX:1:2114:17910:83272.

-------------------------------------caaatcagtagcttacagaacgc
aaacagtaggaccatagtgggggattct--------------------------------
-atcaacaccatgagcctcagggaccttaaacaggtagagaacaggctggagaaagg---
-----catagctaagataag--ggctagaaagaatgagctgttatatgct----------

>gnl|SRA|SRR2638781.30552934.2 HWI-D00731:29:HGNNYADXX:1:1207:3498:96166.

-------------------------------------caaatcagtagcttacagaacgc
aaacagtaggaccatagtgggggattct--------------------------------
-atcaacaccatgagcctcagggaccttaaacaggtagagaacaggctggagaaagg---
-----catagctaagataag--ggctagaaagaatgagctgttatatgct----------

>gnl|SRA|SRR2638781.19546171.2 HWI-D00731:29:HGNNYADXX:1:1115:20777:45486.

-------------------------------------caaatcagtagcttacagaacgc
aaacagtaggaccatagtgggggattct--------------------------------
-atcaacaccatgagcctcagggaccttaaacaggtagagaacaggctggagaaagg---
-----catagctaagataag--ggctagaaagaatgagctgttatatgct----------

>gnl|SRA|SRR2638781.16365629.2 HWI-D00731:29:HGNNYADXX:1:1113:5194:24632.

-------------------------------------caaatcagtagcttacagaacgc
aaacagtaggaccatagtgggggattct--------------------------------
-atcaacaccatgagcctcagggaccttaaacaggtagagaacaggctggagaaagg---
-----catagctaagataag--ggctagaaagaatgagctgttatatgct----------

>gnl|SRA|SRR2638781.24933986.2 HWI-D00731:29:HGNNYADXX:1:1203:19740:62950.

-------------------------------------caaatcagtagcttacagaacgc
aaacagtaggaccatagtgggggattct--------------------------------
-atcaacaccatgagcctcagggaccttaaacaggtagagaacaggctggagaaagg---
-----catagctaagatagg--ggctagaaagaatgagctgttatatgct----------

>gnl|SRA|SRR2638781.78976253.2 HWI-D00731:29:HGNNYADXX:1:2211:3607:2088.

------------------------------gcgccaacaaatcagtagcttacagaacgc
aaac---aggaccatagtgggggattct--------------------------------
-atcaacaccatgagcctcagggaccttaaacaggtagagaacaggctggagaaagg---
-----catagctaagataag--ggctagaaagaatgagctgttata--------------

>gnl|SRA|SRR2638781.15498692.2 HWI-D00731:29:HGNNYADXX:1:1112:4994:63414.

------------------------------gcgccaacaaatcagtagcttacagaacgc
aaac---aggaccatagtgggggattct--------------------------------
-atcaacaccatgagcctcagggaccttaaacaggtagagaacaggctggagaaagg---
-----catagctaagataag--ggctagaaagaatgagctgttata--------------

>gnl|SRA|SRR2638781.29691758.2 HWI-D00731:29:HGNNYADXX:1:1207:2863:30811.

-------------------------------cgccaacaaatcagtagcttacagaacgc
aaac---aggaccatagtgggggattct--------------------------------
-atcaacaccatgagcctcagggaccttaaacaggtagagaacaggctggagaaagg---
-----catagctaagataag--ggctagaaagaatgagctgttatat-------------

>_R_gnl|SRA|SRR2638781.85965871.1 HWI-D00731:29:HGNNYADXX:1:2215:12909:82056.

---agtagcttacagaacgc
aaac---aggaccatagtgggggattct--------------------------------
-atcaacaccatgagcctcagggaccttaaacaggtagagaacaggctggagaaagg---
-----catagctaagataag--ggctagaaagaatgagctgttatatgctgaagttgag-

>_R_gnl|SRA|SRR2638781.65918258.1 HWI-D00731:29:HGNNYADXX:1:2201:2765:6928.

---agtagcttacagaacgc
aaac---aggaccatagtgggggattct--------------------------------
-atcaacaccatgagcctcagggaccttaaacaggtagagaacaggctggagaaagg---
-----catagctaagataag--ggctagaaagaatgagctgttatatgctgaagttgag-

>_R_gnl|SRA|SRR2638781.63515167.1 HWI-D00731:29:HGNNYADXX:1:2115:17020:47537.

---agtagcttacagaacgc
aaac---aggaccatagtgggggattct--------------------------------
-atcaacaccatgagcctcagggaccttaaacaggtagagaacaggctggagaaagg---
-----catagctaagataag--ggctagaaagaatgagctgttatatgctgaagttgag-

>_R_gnl|SRA|SRR2638781.56453345.1 HWI-D00731:29:HGNNYADXX:1:2110:7019:68156.

---agtagcttacagaacgc
aaac---aggaccatagtgggggattct--------------------------------
-atcaacaccatgagcctcagggaccttaaacaggtagagaacaggctggagaaagg---
-----catagctaagataag--ggctagaaagaatgagctgttatatgctgaagttgag-

>gnl|SRA|SRR2638781.51839134.2 HWI-D00731:29:HGNNYADXX:1:2107:5913:39650.

---agtagcttacagaacgc
aaac---aggaccatagtgggggattct--------------------------------
-atcaacaccatgagcctcagggaccttaaacaggtagagaacaggctggagaaagg---
-----catagctaagataag--ggctagaaagaatgagctgttatatgctgaagttgag-

>_R_gnl|SRA|SRR2638781.17170798.1 HWI-D00731:29:HGNNYADXX:1:1113:6703:80326.

---agtagcttacagaacgc
aaac---aggaccatagtgggggattct--------------------------------
-atcaacaccatgagcctcagggaccttaaacaggtagagaataggctggagaaagg---
-----catagctaagataag--ggctagaaagaatgagctgttatatgctgaagttgag-

>gnl|SRA|SRR2638781.71547589.2 HWI-D00731:29:HGNNYADXX:1:2205:18085:56484.

--cagtagcttacagaacgc
aaac---aggaccatagtgggggattct--------------------------------
-atcaacaccatgagcctcagggaccttaaacaggtagagaacaggctggagaaagg---
-----catagctaagataag--ggctagaaagaatgagctgttatatgctgaagttga--

>gnl|SRA|SRR2638781.46025726.2 HWI-D00731:29:HGNNYADXX:1:2103:19464:3542.

--cagtagcttacagaacgc
aaac---aggaccatagtgggggattct--------------------------------
-atcaacaccatgagcctcagggaccttaaacaggtagagaacaggctggagaaagg---
-----catagctaagataag--ggctagaaagaatgagctgttatatgctgaagttga--

>_R_gnl|SRA|SRR2638781.69253220.1 HWI-D00731:29:HGNNYADXX:1:2203:11030:80766.

--gtagcttacagaacgc
aaac---aggaccatagtgggggattct--------------------------------
-atcaacaccatgagcctcagggaccttaaacaggtagagaacaggctggagaaagg---
-----catagctaagataag--ggctagaaagaatgagctgttatatgctgaagttgagt

>gnl|SRA|SRR2638781.4967934.2 HWI-D00731:29:HGNNYADXX:1:1104:20811:92152.

--gtagcttacagaacgc
aaac---aggaccatagtgggggattct--------------------------------
-atcaacaccatgagcctcagggaccttaaacaggtagagaacaggctggagaaagg---

-----catagctaagataag--ggctagaaagaatgagctgttatatgctgaagttgagt

>gnl|SRA|SRR2638781.80067217.2 HWI-D00731:29:HGNNYADXX:1:2211:8100:78540.

-----------------------------------aacaaatcagtagcttacagaacgc
aaac---aggaccatagtgggggattct--------------------------------
-atcaacaccatgagcctcagggaccttaaacaggtagagaacaggctggagaaagg---
-----catagctaagataag--ggctagaaagaatgagctgttatatgctg---------

>gnl|SRA|SRR2638781.71185702.2 HWI-D00731:29:HGNNYADXX:1:2205:19926:29246.

-----------------------------------aacaaatcagtagcttacagaacgc
aaac---aggaccatagtgggggattct--------------------------------
-atcaacaccatgagcctcagggaccttaaacaggtagagaacaggctggagaaagg---
-----catagctaagataag--ggctagaaagaatgagctgttatatgctg---------

>gnl|SRA|SRR2638781.70833781.2 HWI-D00731:29:HGNNYADXX:1:2205:15219:2551.

-----------------------------------aacaaatcagtagcttacagaacgc
aaac---aggaccatagtgggggattct--------------------------------
-atcaacaccatgagcctcagggaccttaaacaggtagagaacaggctggagaaagg---
-----catagctaagataag--ggctagaaagaatgagctgttatatgctg---------

>_R_gnl|SRA|SRR2638781.66979479.1 HWI-D00731:29:HGNNYADXX:1:2201:3769:90406.

-----------------------------------aacaaatcagtagcttacagaacgc
aaac---aggaccatagtgggggattct--------------------------------
-atcaacaccatgagcctcagggaccttaaacaggtagagaacaggctggagaaagg---
-----catagctaagataag--ggctagaaagaatgagctgttatatgctg---------

>gnl|SRA|SRR2638781.52647415.2 HWI-D00731:29:HGNNYADXX:1:2107:6953:98263.

----------------------------------caacaaatcagtagcttacagaacgc
aaac---aggaccatagtgggggattct--------------------------------
-atcaacaccatgagcctcagggaccttaaacaggtagagaacaggctggagaaagg---
-----catagctaagataag--ggctagaaagaatgagctgttatatgct----------

>gnl|SRA|SRR2638781.48656021.2 HWI-D00731:29:HGNNYADXX:1:2105:3898:6552.

-------------------------------------caaatcagtagcttacagaacgc
aaac---aggaccatagtgggggattct--------------------------------
-atcaacaccatgagcctcagggaccttaaacaggtagagaacaggctggagaaagg---
-----catagctaagataag--ggctagaaagaatgagctgttatatgctgaa-------

>_R_gnl|SRA|SRR2638781.15006728.1 HWI-D00731:29:HGNNYADXX:1:1112:4085:28999.

--------------------------------------aaatcagtagcttacagaacgc
aaac---aggaccatagtgggggattct--------------------------------
-atcaacaccatgagcctcagggaccttaaacaggtagagaacaggctggagaaagg---
-----catagctaagataag--ggctagaaagaatgagctgttatatgctgaag------

>_R_gnl|SRA|SRR2638781.44563291.1 HWI-D00731:29:HGNNYADXX:1:2101:18940:91321.

--atcagtagcttacagaacgc
aaac---aggaccatagtgggggattct--------------------------------
-atcaacaccatgagcctcagggaccttaaacaggtagagaacaggctggagaaagg---
-----catagctaagataag--ggctagaaagaatgagctgttatatgctgaagtt----

>_R_gnl|SRA|SRR2638781.35757925.1 HWI-D00731:29:HGNNYADXX:1:1211:11129:75086.

--atcagtagcttacagaacgc
aaac---aggaccatagtgggggattct--------------------------------
-atcaacaccatgagcctcagggaccttaaacaggtagagaacaggctggagaaagg---
-----catagctaagataag--ggctagaaagaatgagctgttatatgctgaagtt----

>gnl|SRA|SRR2638781.71637996.2 HWI-D00731:29:HGNNYADXX:1:2205:2084:63483.

--cagtagcttacagaacgc
aaacagtaggaccatagtgggggattct--------------------------------
-atcaacaccatgagcctcagggaccttaaacaggtagagaacaggctggagaaagg---
-----catagctaagataag--ggctagaaagaatgagctgttatatgctggaga-----

>gnl|SRA|SRR2638781.57091739.2 HWI-D00731:29:HGNNYADXX:1:2111:9324:12965.

--cagtagcttacagaacgc
aaacagtaggaccatagtgggggattct--------------------------------
-atcaacaccatgagcctcagggaccttaaacaggtagagaacaggctggagaaagg---
-----catagctaagataag--ggctagaaagaatgagctgttatatgctggaga-----

>gnl|SRA|SRR2638781.18698774.2 HWI-D00731:29:HGNNYADXX:1:1114:5032:86564.

--cagtagcttacagaacgc
aaacagtaggaccatagtgggggattct--------------------------------
-atcaacaccatgagcctcagggaccttaaacaggtagagaacaggctggagaaagg---
-----catagctaagataag--ggctagaaagaatgagctgttatatgctgaagt-----

>_R_gnl|SRA|SRR2638781.71190002.1 HWI-D00731:29:HGNNYADXX:1:2205:6193:29658.

---tcagtagcttacagaacgc

aaacagtaggaccatagtgggggattct--------------------------------
-atcaacaccatgagcctcagggaccttaaacaggtagagaacaggctggagaaagg---
-----catagctaagataag--ggctagaaagaatgagctgttatatgctgaag------

>_R_gnl|SRA|SRR2638781.30313796.1 HWI-D00731:29:HGNNYADXX:1:1207:16107:78213.

--gtagcttacagaacgc
aaacagtaggaccatagtgggggattct--------------------------------
-atcaacaccatgagcctcagggaccttaaacaggtagagaacaggctggagaaagg---
-----catagctaagataag--ggctagaaagaatgagctgttatatgctgaagttg---

>_R_gnl|SRA|SRR2638781.75151838.1 HWI-D00731:29:HGNNYADXX:1:2208:8282:26099.

--agcttacagaacgc
aaacagtaggaccatagtgggggattct--------------------------------
-atcaacaccatgagcctcagggaccttaaacaggtagagaacaggctggagaaagg---
-----catagctaagataag--ggctagaaagaatgagctgttatatgctgaagttgag-

>_R_gnl|SRA|SRR2638781.65201609.1 HWI-D00731:29:HGNNYADXX:1:2116:19113:58873.

--agcttacagaacgc
aaacagtaggaccatagtgggggattct--------------------------------
-atcaacaccatgagcctcagggaccttaaacaggtagagaacaggctggagaaagg---
-----catagctaagataag--ggctagaaagaatgagctgttatatgctgaagttgag-

>_R_gnl|SRA|SRR2638781.63561559.1 HWI-D00731:29:HGNNYADXX:1:2115:20356:50557.

--agcttacagaacgc
aaacagtaggaccatagtgggggattct--------------------------------
-atcaacaccatgagcctcagggaccttaaacaggtagagaacaggctggagaaagg---
-----catagctaagataag--ggctagaaagaatgagctgttatatgctgaagttgag-

>_R_gnl|SRA|SRR2638781.59512891.1 HWI-D00731:29:HGNNYADXX:1:2112:7746:79174.

--agcttacagaacgc
aaacagtaggaccatagtgggggattct--------------------------------
-atcaacaccatgagcctcagggaccttaaacaggtagagaacaggctggagaaagg---
-----catagctaagataag--ggctagaaagaatgagctgttatatgctgaagttgag-

>_R_gnl|SRA|SRR2638781.34446666.1 HWI-D00731:29:HGNNYADXX:1:1210:13568:81100.

--agcttacagaacgc
aaacagtaggaccatagtgggggattct--------------------------------
-atcaacaccatgagcctcagggaccttaaacaggtagagaacaggctggagaaagg---
-----catagctaagataag--ggctagaaagaatgagctgttatatgctgaagttgag-

>_R_gnl|SRA|SRR2638781.2843176.1 HWI-D00731:29:HGNNYADXX:1:1103:5437:23226.

--agcttacagaacgc
aaacagtaggaccatagtgggggattct--------------------------------
-atcaacaccatgagcctcagggaccttaaacaggtagagaacaggctggagaaagg---
-----catagctaagataag--ggctagaaagaatgagctgttatatgctgaagttgag-

>_R_gnl|SRA|SRR2638781.87518837.1 HWI-D00731:29:HGNNYADXX:1:2216:9210:87224.

---gcttacagaacgc
aaacagtaggaccatagtgggggattct--------------------------------
-atcaacaccatgagcctcagggaccttaaacaggtagagaacaggctggagaaagg---
-----catagctaagataag--ggctagaaagaatgagctgttatatgctgaagttgagt

>_R_gnl|SRA|SRR2638781.62574394.1 HWI-D00731:29:HGNNYADXX:1:2114:6854:85286.

---gcttacagaacgc
aaacagtaggaccatagtgggggattct--------------------------------
-atcaacaccatgagcctcagggaccttaaacaggtagagaacaggctggagaaagg---
-----catagctaagataag--ggctagaaagaatgagctgttatatgctgaagttgagt

>gnl|SRA|SRR2638781.40104400.2 HWI-D00731:29:HGNNYADXX:1:1214:1961:78877.

---gcttacagaacgc
aaacagtaggaccatagtgggggattct--------------------------------
-atcaacaccatgagcctcagggaccttaaacaggtagagaacaggctggagaaagg---
-----catagctaagataag--ggctagaaagaatgagctgttatatgctgaagttgagt

>_R_gnl|SRA|SRR2638781.25018539.1 HWI-D00731:29:HGNNYADXX:1:1203:7150:71350.

---gcttacagaacgc
aaacagtaggaccatagtgggggattct--------------------------------
-atcaacaccatgagcctcagggaccttaaacaggtagagaacaggctggagaaagg---
-----catagctaagataag--ggctagaaagaatgagctgttatatgctgaagttgagt

>_R_gnl|SRA|SRR2638781.23577451.1 HWI-D00731:29:HGNNYADXX:1:1202:11657:41555.

---gcttacagaacgc
aaacagtaggaccatagtgggggattct--------------------------------
-atcaacaccatgagcctcagggaccttaaacaggtagagaacaggctggagaaagg---
-----catagctaagataag--ggctagaaagaatgagctgttatatgctgaagttgagt

>gnl|SRA|SRR2638781.23237925.2 HWI-D00731:29:HGNNYADXX:1:1202:4070:15127.

---gcttacagaacgc
aaacagtaggaccatagtgggggattct--------------------------------
-atcaacaccatgagcctcagggaccttaaacaggtagagaacaggctggagaaagg---
-----catagctaagataag--ggctagaaagaatgagctgttatatgctgaagttgagt

>_R_gnl|SRA|SRR2638781.11774577.1 HWI-D00731:29:HGNNYADXX:1:1109:6891:97037.

---gcttacagaacgc
aaacagtaggaccatagtgggggattct--------------------------------
-atcaacaccatgagcctcagggaccttaaacaggtagagaacaggctggagaaagg---
-----catagctaagataag--ggctagaaagaatgagctgttatatgctgaagttgagt

>_R_gnl|SRA|SRR2638781.10813935.1 HWI-D00731:29:HGNNYADXX:1:1109:15029:28140.

---gcttacagaacgc
aaacagtaggaccatagtgggggattct--------------------------------
-atcaacaccatgagcctcagggaccttaaacaggtagagaacaggctggagaaagg---
-----catagctaagataag--ggctagaaagaatgagctgttatatgctgaagttgagt

>_R_gnl|SRA|SRR2638781.71637996.1 HWI-D00731:29:HGNNYADXX:1:2205:2084:63483.

---------------------------------------tctcagtagcttacagaacgc
aaacagtaggaccatagtgggggattct--------------------------------
-atcaacaccatgagcctcagggaccttaaacaggtagagaacaggctggagaaagg---
-----catagctaagataag--ggctagaaagaatgagctgttatatgctgg--------

>_R_gnl|SRA|SRR2638781.57091739.1 HWI-D00731:29:HGNNYADXX:1:2111:9324:12965.

---------------------------------------tctcagtagcttacagaacgc
aaacagtaggaccatagtgggggattct--------------------------------
-atcaacaccatgagcctcagggaccttaaacaggtagagaacaggctggagaaagg---
-----catagctaagataag--ggctagaaagaatgagctgttatatgctgg--------

>gnl|SRA|SRR2638781.53639617.2 HWI-D00731:29:HGNNYADXX:1:2108:13532:69682.

---agtagcttacagaacgc
aaacagtaggaccatagtgggggattct--------------------------------
-atcaacaccatgagcctcagggatcttaaacaggtagagaacaggatggagaaagg---
-----catagctaagataag--ggctagaaagaatgagctgttatatgctgaagtt----

>_R_gnl|SRA|SRR2638781.70511302.1 HWI-D00731:29:HGNNYADXX:1:2204:6266:77692.

--cagaacgc
aaacagtaggaccatagtgggggattct--------------------------------

-atcaacaccatgagcctcagggaccttaaacaggtagagaacaggctggagaaagg---
-----catagctaagataag--ggctagaaagaatgagctgttatatgctgaagttgagt
acatg-------------
>_R_gnl|SRA|SRR2638781.67308445.1 HWI-D00731:29:HGNNYADXX:1:2202:19795:16449.

--cagaacgc
aaacagtaggaccatagtgggggattct--------------------------------
-atcaacaccatgagcctcagggaccttaaacaggtagagaacaggctggagaaagg---
-----catagctaagataag--ggctagaaagaatgagctgttatatgctgaagttgagt
acatg-------------
>_R_gnl|SRA|SRR2638781.57637567.1 HWI-D00731:29:HGNNYADXX:1:2111:16464:50424.

--cagaacgc
aaacagtaggaccatagtgggggattct--------------------------------
-atcaacaccatgagcctcagggaccttaaacaggtagagaacaggctggagaaagg---
-----catagctaagataag--ggctagaaagaatgagctgttatatgctgaagttgagt
acatg-------------
>_R_gnl|SRA|SRR2638781.41634129.1 HWI-D00731:29:HGNNYADXX:1:1215:10640:82952.

--cagaacgc
aaacagtaggaccatagtgggggattct--------------------------------
-atcaacaccatgagcctcagggaccttaaacaggtagagaacaggctggagaaagg---
-----catagctaagataag--ggctagaaagaatgagctgttatatgctgaagttgagt
acatg-------------
>_R_gnl|SRA|SRR2638781.34449761.1 HWI-D00731:29:HGNNYADXX:1:1210:10772:81385.

--cagaacgc
aaacagtaggaccatagtgggggattct--------------------------------
-atcaacaccatgagcctcagggaccttaaacaggtagagaacaggctggagaaagg---
-----catagctaagataag--ggctagaaagaatgagctgttatatgctgaagttgagt
acatg-------------
>_R_gnl|SRA|SRR2638781.4425454.1 HWI-D00731:29:HGNNYADXX:1:1104:9285:51571.

--cagaacgc
aaacagtaggaccatagtgggggattct--------------------------------
-atcaacaccatgagcctcagggaccttaaacaggtagagaacaggctggagaaagg---
-----catagctaagataag--ggctagaaagaatgagctgttatatgctgaagttgagt
acatg-------------
>_R_gnl|SRA|SRR2638781.83349300.1 HWI-D00731:29:HGNNYADXX:1:2214:19927:6181.

---agaacgc
aaacagtaggaccatagtgggggattct--------------------------------
-atcaacaccatgagcctcagggaccttaaacaggtagagaacaggctggagaaagg---
-----catagctaagataag--ggctagaaagaatgagctgttatatgctgaagttgagt
acatgc------------

>gnl|SRA|SRR2638781.75299963.2 HWI-D00731:29:HGNNYADXX:1:2208:12278:36947.

---agaacgc
aaacagtaggaccatagtgggggattct--------------------------------
-atcaacaccatgagcctcagggaccttaaacaggtagagaacaggctggagaaagg---
-----catagctaagataag--ggctagaaagaatgagctgttatatgctgaagttgagt
acatgc------------
>_R_gnl|SRA|SRR2638781.3730889.1 HWI-D00731:29:HGNNYADXX:1:1103:4451:98525.

---agaacgc
aaacagtaggaccatagtgggggattct--------------------------------
-atcaacaccatgagcctcagggaccttaaacaggtagagaacaggctggagaaagg---
-----catagctaagataag--ggctagaaagaatgagctgttatatgctgaagttgagt
acatgc------------
>_R_gnl|SRA|SRR2638781.65989590.1 HWI-D00731:29:HGNNYADXX:1:2201:11499:12327.

--gaacgc
aaacagtaggaccatagtgggggattct--------------------------------
-atcaacaccatgagcctcagggaccttaaacaggtagagaacaggctggagaaagg---
-----catagctaagataag--ggctagaaagaatgagctgttatatgctgaagttgagt
acatgcg-----------
>_R_gnl|SRA|SRR2638781.18571917.1 HWI-D00731:29:HGNNYADXX:1:1114:8643:77898.

--gaacgc
aaacagtaggaccatagtgggggattct--------------------------------
-atcaacaccatgagcctcagggaccttaaacaggtagagaacaggctggagaaagg---
-----catagctaagataag--ggctagaaagaatgagctgttatatgctgaagttgagt
acatgca-----------
>_R_gnl|SRA|SRR2638781.10090580.1 HWI-D00731:29:HGNNYADXX:1:1108:5736:75368.

--gaacgc
aaacagtaggaccatagtgggggattct--------------------------------
-atcaacaccatgagcctcagggaccttaaacaggtagagaacaggctggagaaagg---
-----catagctaagataag--ggctagaaagaatgagctgttatatgctgaagttgagt
acatgca-----------
>_R_gnl|SRA|SRR2638781.7169974.1 HWI-D00731:29:HGNNYADXX:1:1106:3371:57749.

--gaacgc
aaacagtaggaccatagtgggggattct--------------------------------
-atcaacaccatgagcctcagggaccttaaacaggtagagaacaggctggagaaagg---
-----catagctaagataag--ggctagaaagaatgagctgttatatgctgaagttgagt
acatgca-----------
>_R_gnl|SRA|SRR2638781.5587296.1 HWI-D00731:29:HGNNYADXX:1:1105:20837:39038.

--gaacgc
aaacagtaggaccatagtgggggattct--------------------------------
-atcaacaccatgagcctcagggaccttaaacaggtagagaacaggctggagaaagg---
-----catagctaagataag--ggctagaaagaatgagctgttatatgctgaagttgagt
acatgca-----------
>_R_gnl|SRA|SRR2638781.57223058.1 HWI-D00731:29:HGNNYADXX:1:2111:11359:21783.

--gaacgc
aaacagtaggaccatagtgggggattct--------------------------------
-atcaacaccatgagcctcagggaccttaaacaggtagagaacaggctggagaaagg---
-----catagctaagataag--ggctagaaagaatgagctgttatatgctgaagttgagt
acatgct-----------
>_R_gnl|SRA|SRR2638781.30763599.1 HWI-D00731:29:HGNNYADXX:1:1208:9014:12347.

---aacgc
aaacagtaggaccatagtgggggattct--------------------------------
-atcaacaccatgagcctcagggaccttaaacaggtagagaacaggctggagaaagg---
-----catagctaagataag--ggctagaaagaatgagctgttatatgctgaagttgagt
acatgcag----------
>_R_gnl|SRA|SRR2638781.6231714.1 HWI-D00731:29:HGNNYADXX:1:1105:1505:87475.

---aacgc
aaacagtaggaccatagtgggggattct--------------------------------
-atcaacaccatgagcctcagggaccttaaacaggtagagaacaggctggagaaagg---
-----catagctaagataag--ggctagaaagaatgagctgttatatgctgaagttgagt
acatgcag----------
>_R_gnl|SRA|SRR2638781.55791893.1 HWI-D00731:29:HGNNYADXX:1:2110:17108:22103.

--acgc
aaacagtaggaccatagtgggggattct--------------------------------
-atcaacaccatgagcctcagggaccttaaacaggtagagaacaggctggagaaagg---
-----catagctaagataag--ggctagaaagaatgagctgttatatgctgaagttgagt
acatgcagg---------
>_R_gnl|SRA|SRR2638781.13191693.1 HWI-D00731:29:HGNNYADXX:1:1110:2734:99021.

--acgc
aaacagtaggaccatagtgggggattct--------------------------------
-atcaacaccatgagcctcagggaccttaaacaggtagagaacaggctggagaaagg---
-----catagctaagataag--ggctagaaagaatgagctgttatatgctgaagttgagt
acatgcagg---------
>_R_gnl|SRA|SRR2638781.8720914.1 HWI-D00731:29:HGNNYADXX:1:1107:12802:74580.

--acgc
aaacagtaggaccatagtgggggattct--------------------------------
-atcaacaccatgagcctcagggaccttaaacaggtagagaacaggctggagaaagg---

-----catagctaagataag--ggctagaaagaatgagctgttatatgctgaagttgagt
acatgcaga---------
>_R_gnl|SRA|SRR2638781.40446197.1 HWI-D00731:29:HGNNYADXX:1:1215:10018:2552.

--accc
aaacagtaggaccatagtgggggattct--------------------------------
-atcaacaccatgagcctcagggaccttaaacaggtagagaacaggctggagaaagg---
-----catagctaagataag--ggctagaaagaatgagctgttatatgctgaagttgagt
acatgcagt---------
>gnl|SRA|SRR2638781.56884474.2 HWI-D00731:29:HGNNYADXX:1:2110:5305:97968.

---cgc
aaacagtaggaccatagtgggggattct--------------------------------
-atcaacaccatgagcctcagggaccttaaacaggtagagaacaggctggagaaagg---
-----catagctaagataag--ggctagaaagaatgagctgttatatgctgaagttgagt
acatgcagaa--------
>gnl|SRA|SRR2638781.46070197.2 HWI-D00731:29:HGNNYADXX:1:2103:20849:6903.

---cgc
aaacagtaggaccatagtgggggattct--------------------------------
-atcaacaccatgagcctcagggaccttaaacaggtagagaacaggctggagaaagg---
-----catagctaagataag--ggctagaaagaatgagctgttatatgctgaagttgagt
acatgcagaa--------
>gnl|SRA|SRR2638781.3148748.2 HWI-D00731:29:HGNNYADXX:1:1103:3909:51117.

---cgc
aaacagtaggaccatagtgggggattct--------------------------------
-atcaacaccatgagcctcagggaccttaaacaggtagagaacaggctggagaaagg---
-----catagctaagataag--ggctagaaagaatgagctgttatatgctgaagttgagt
acatgcagaa--------
>gnl|SRA|SRR2638781.75385702.2 HWI-D00731:29:HGNNYADXX:1:2208:14693:43206.

---c
aaacagtaggaccatagtgggggattct--------------------------------
-atcaacaccatgagcctcagggaccttaaacaggtagagaacaggctggagaaagg---
-----catagctaagataag--ggctagaaagaatgagctgttatatgctgaagttgagt
acatgcagaaaa------
>gnl|SRA|SRR2638781.10391038.2 HWI-D00731:29:HGNNYADXX:1:1108:3693:97228.

---c
aaacagtaggaccatagtgggggattct--------------------------------
-atcaacaccatgagcctcagggatcttaaacaggtagagaacaggctggagaaagg---
-----catagctaagataag--ggctagaaagaatgagctgttatatgctgaagttgagt
acatgcagaaaa------
>_R_gnl|SRA|SRR2638781.77821264.1 HWI-D00731:29:HGNNYADXX:1:2210:15231:19697.

----
aaacagtaggaccatagtgggggattct--------------------------------
-atcaacaccatgagcctcagggaccttaaacaggtagagaacaggctggagaaagg---
-----catagctaaggtaag--ggctagaaagaatgagctgttatatgctgaagttgagt
acatgcagaaaag-----
>_R_gnl|SRA|SRR2638781.21651966.1 HWI-D00731:29:HGNNYADXX:1:1116:5920:89851.

----
aaacagtaggaccatagtgggggattct--------------------------------
-atcaacaccatgagcctcagggaccttaaacaggtagagaacaggctggagaaagg---
-----catagctaagataag--ggctagaaagaatgagctgttatatgctgaagttgagt
acatgcagaaaag-----
>gnl|SRA|SRR2638781.80155012.2 HWI-D00731:29:HGNNYADXX:1:2211:1565:84898.

-aacagtaggaccatagtgggggattct--------------------------------
-atcaacaccatgagcctcagggaccttaaacaggtagagaacaggctggagaaagg---
-----catagctaagataag--ggctagaaagaatgagctgttatatgctgaagttgagt
acatgcagaaaagg----
>_R_gnl|SRA|SRR2638781.83962210.1 HWI-D00731:29:HGNNYADXX:1:2214:17209:47649.

--acagtaggaccatagtgggggattct--------------------------------
-atcaacaccatgagcctcagggaccttaaacaggtagagaacaggctggagaaagg---
-----catagctaagataag--ggctagaaagaatgagctgttatatgctgaagttgagt
acatgcagaaaaggg---
>_R_gnl|SRA|SRR2638781.28297334.1 HWI-D00731:29:HGNNYADXX:1:1206:14498:25033.

--acagtaggaccatagtgggggattct--------------------------------
-atcaacaccatgagcctcagggaccttaaacaggtagagaacaggctggagaaagg---
-----catagctaagataag--ggctagaaagaatgagctgttatatgctgaagttgagt
acatgcagaaaaggg---
>_R_gnl|SRA|SRR2638781.66078080.1 HWI-D00731:29:HGNNYADXX:1:2201:9186:19498.

--tcagtaggaccatagtgggggattct--------------------------------
-atcaacaccatgagcctcagggaccttaaacaggtagagaacaggctggagaaagg---
-----catagctaagataag--ggctagaaagaatgagctgttatatgctgaagttgagt
acatgcagaaaaggg---
>_R_gnl|SRA|SRR2638781.86824819.1 HWI-D00731:29:HGNNYADXX:1:2216:8227:39881.

---cagtaggaccatagtgggggattct--------------------------------
-atcaacaccatgagcctcagggaccttaaacaggtagagaacaggctggagaaagg---
-----catagctaagataag--ggctagaaagaatgagctgttatatgctgaagttgagt
acatgcagaaaaggga--
>gnl|SRA|SRR2638781.66078080.2 HWI-D00731:29:HGNNYADXX:1:2201:9186:19498.

---cagtaggaccatagtgggggattct--------------------------------
-atcaacaccatgagcctcagggaccttaaacaggtagagaacaggctggagaaagg---
-----catagctaagataag--ggctagaaagaatgagctgttatatgctgaagttgagt
acatgcagaaaaggga--
>_R_gnl|SRA|SRR2638781.61015784.1 HWI-D00731:29:HGNNYADXX:1:2113:3112:81282.

---cagtaggaccatagtgggggattct--------------------------------
-atcaacaccatgagcctcagggaccttaaacaggtagagaacaggctggagaaagg---
-----catagctaagataag--ggctagaaagaatgagctgttatatgctgaagttgagt
acatgcagaaaaggga--
>_R_gnl|SRA|SRR2638781.54211580.1 HWI-D00731:29:HGNNYADXX:1:2109:15433:10503.

---cagtaggaccatagtgggggattct--------------------------------
-atcaacaccatgagcctcagggaccttaaacaggtagagaacaggctggagaaagg---
-----catagctaagataag--ggctagaaagaatgagctgttatatgctgaagttgagt
acatgcagaaaagggg--
>_R_gnl|SRA|SRR2638781.76477549.1 HWI-D00731:29:HGNNYADXX:1:2209:16656:22744.

----agtaggaccatagtgggggattct--------------------------------
-atcaacaccatgagcctcagggaccttaaacaggtagagaacaggctggagaaagg---
-----catagctaagataag--ggctagaaagaatgagctgttatatgctgaagttgagt
acatgcagaaaagggga-
>_R_gnl|SRA|SRR2638781.53538542.1 HWI-D00731:29:HGNNYADXX:1:2108:18147:62312.

----agtaggaccatagtgggggattct--------------------------------
-atcaacaccatgagcctcagggaccttaaacaggtagagaacaggctggagaaagg---
-----catagctaagataag--ggctagaaagaatgagctgttatatgctgaagttgagt
acatgcagaaaagggga-
>_R_gnl|SRA|SRR2638781.85927066.1 HWI-D00731:29:HGNNYADXX:1:2215:8181:79512.

-----gtaggaccatagtgggggattct--------------------------------
-atcaacaccatgagcctcagggaccttaaacaggtagagaacaggctggagaaagg---
-----catagctaagataag--ggctagaaagaatgagctgttatatgctgaagttgagt

acatgcagaaaagggaag
>_R_gnl|SRA|SRR2638781.57431223.1 HWI-D00731:29:HGNNYADXX:1:2111:20137:36135.

-----gtaggaccatagtgggggattct--------------------------------
-atcaacaccatgagcctcagggaccttaaacaggtagagaacaggctggagaaagg---
-----catagctaagataag--ggctagaaagaatgagctgttatatgctgaagttgagt
acatgcagaaaagggaag
>_R_gnl|SRA|SRR2638781.45523948.1 HWI-D00731:29:HGNNYADXX:1:2102:6821:65442.

-----gtaggaccatagtgggggattct--------------------------------
-atcaacaccatgagcctcagggaccttaaacaggtagagaacaggctggagaaagg---
-----catagctaagataag--ggctagaaagaatgagctgttatatgctgaagttgagt
acatgcagaaaagggaag
>_R_gnl|SRA|SRR2638781.37159584.1 HWI-D00731:29:HGNNYADXX:1:1212:17774:74419.

-----gtaggaccatagtgggggattct--------------------------------
-atcaacaccatgagcctcagggaccttaaacaggtagagaacaggctggagaaagg---
-----catagctaagataag--ggctagaaagaatgagctgttatatgctgaagttgagt
acatgcagaaaagggaag
>_R_gnl|SRA|SRR2638781.25865011.1 HWI-D00731:29:HGNNYADXX:1:1204:4155:38267.

-----gtaggaccatagtgggggattct--------------------------------
-atcaacaccatgagcctcagggaccttaaacaggtagagaacaggctggagaaagg---
-----catagctaagataag--ggctagaaagaatgagctgttatatgctgaagttgagt
acatgcagaaaagggaag
>gnl|SRA|SRR2638781.9189635.2 HWI-D00731:29:HGNNYADXX:1:1108:5343:9816.

-----gtaggaccatagtgggggattct--------------------------------
-atcaacaccatgagcctcagggaccttaaacaggtagagaacaggctggagaaagg---
-----catagctaagataag--ggctagaaagaatgagctgttatatgctgaagttgagt
acatgcagaaaagggaag
>gnl|SRA|SRR2638781.8207064.2 HWI-D00731:29:HGNNYADXX:1:1107:10311:35419.

-----gtaggaccatagtgggggattct--------------------------------
-atcaacaccatgagcctcagggaccttaaacaggtagagaacaggctggagaaagg---
-----catagctaagataag--ggctagaaagaatgagctgttatatgctgaagttgagt
acatgcagaaaagggaag
>gnl|SRA|SRR2638781.7864239.2 HWI-D00731:29:HGNNYADXX:1:1107:6167:9574.

-----gtaggaccatagtgggggattct--------------------------------
-atcaacaccatgagcctcagggaccttaaacaggtagagaacaggctggagaaagg---
-----catagctaagataag--ggctagaaagaatgagctgttatatgctgaagttgagt
acatgcagaaaagggaag
>gnl|SRA|SRR2638781.44913244.2 HWI-D00731:29:HGNNYADXX:1:2102:18512:18626.

----agtaggaccatagtgggggattct--------------------------------
-atcaacaccatgagcctcagggaccttaaacaggtagagaacaggctggagaaagg---
-----catagctaagataag--ggctagaaagaatgagctgttatatgctgaagttgagt
acatagatcggaagagc-
>gnl|SRA|SRR2638781.38096919.2 HWI-D00731:29:HGNNYADXX:1:1213:18461:40375.

----agtaggaccatagtgggggattct--------------------------------
-atcaacaccatgagcctcagggaccttaaacaggtagagaacaggctggagaaagg---
-----catagctaagataag--ggctagaaagaatgagctgttatatgctgaagttgagt
acatagatcggaagagc-
>gnl|SRA|SRR2638781.40446197.2 HWI-D00731:29:HGNNYADXX:1:1215:10018:2552.

---acagaaccc
aaacagtaggaccatagtgggggattct--------------------------------
-atcaacaccatgagcctcagggaccttaaacaggtagagaacaggctggagaaagg---
-----catagctaagataag--ggctagaaagaatgagctgttatatgctgaagttgagt
acat--------------
>_R_gnl|SRA|SRR2638781.44913244.1 HWI-D00731:29:HGNNYADXX:1:2102:18512:18626.

---gctcttccg
atctagtaggaccatagtgggggattct--------------------------------
-atcaacaccatgagcctcagggaccttaaacaggtagagaacaggctggagaaagg---
-----catagctaagataag--ggctagaaagaatgagctgttatatgctgaagttgagt
acat--------------
>_R_gnl|SRA|SRR2638781.38096919.1 HWI-D00731:29:HGNNYADXX:1:1213:18461:40375.

---gctcttccg
atctagtaggaccatagtgggggattct--------------------------------
-atcaacaccatgagcctcagggaccttaaacaggtagagaacaggctggagaaagg---
-----catagctaagataag--ggctagaaagaatgagctgttatatgctgaagttgagt
acat--------------
>_R_gnl|SRA|SRR2638781.83171697.1 HWI-D00731:29:HGNNYADXX:1:2213:20463:93476.

--cttacagaacgc
a---aacaggaccatagtgggggattct--------------------------------

-atcaacaccatgagcctcagggaccttaaacaggtagagaacaggctggagaaagg---
-----catagctaagataag--ggctagaaagaatgagctgttatatgctgaagttgagt
acat-------------
>gnl|SRA|SRR2638781.71048330.2 HWI-D00731:29:HGNNYADXX:1:2205:10541:18929.

--cttacagaacgc
a---aacaggaccatagtgggggattct--------------------------------
-atcaacaccatgagcctcagggaccttaaacaggtagagaacaggctggagaaagg---
-----catagctaagataag--ggctagaaagaatgagctgttatatgctgaagttgagt
acat-------------
>gnl|SRA|SRR2638781.79800753.2 HWI-D00731:29:HGNNYADXX:1:2211:18586:59868.

--cttacagaacgc
a---aacaggaccatagtgggggattct--------------------------------
-atcaacaccatgagcctcagggaccttaaacaggaagagaacaggctggagaaagg---
-----catagctaagataag--ggctagaaagaatgagctgttatatgctgaagttgagt
acat-------------
>_R_gnl|SRA|SRR2638781.87350985.1 HWI-D00731:29:HGNNYADXX:1:2216:4497:76037.

---ttacagaacgc
a---aacaggaccatagtgggggattct--------------------------------
-atcaacaccatgagcctcagggaccttaaacaggtagagaacaggctggagaaagg---
-----catagctaatataag--ggctagaaagaatgagctgttatatgctgaagttgagt
acatg------------
>_R_gnl|SRA|SRR2638781.74835176.1 HWI-D00731:29:HGNNYADXX:1:2208:2204:3115.

---ttacagaacgc
a---aacaggaccatagtgggggattct--------------------------------
-atcaacaccatgagcctcagggaccttaaacaggtagagaacaggctggagaaagg---
-----catagctaagataag--ggctagaaagaatgagctgttatatgctgaagttgagt
acatg------------
>_R_gnl|SRA|SRR2638781.65618262.1 HWI-D00731:29:HGNNYADXX:1:2116:15478:85956.

---ttacagaacgc
a---aacaggaccatagtgggggattct--------------------------------
-atcaacaccatgagcctcagggaccttaaacaggtagagaacaggctggagaaagg---
-----catagctaagataag--ggctagaaagaatgagctgttatatgctgaagttgagt
acatg------------
>_R_gnl|SRA|SRR2638781.13176145.1 HWI-D00731:29:HGNNYADXX:1:1110:13764:97915.

---ttacagaacgc
a---aacaggaccatagtgggggattct--------------------------------
-atcaacaccatgagcctcagggaccttaaacaggtagagaacaggctggagaaagg---
-----catagctaagataag--ggctagaaagaatgagctgttatatgctgaagttgagt
acatg------------

>gnl|SRA|SRR2638781.7742262.2 HWI-D00731:29:HGNNYADXX:1:1106:3968:99935.

---acagaacgc
a---aacaggaccatagtgggggattct--------------------------------
-atcaacaccatgagcctcagggaccttaaacaggtagagaacaggctggagaaagg---
-----catagctaagataag--ggctagaaagaatgagctgttatatgctgaagttgagt
acatgca-----------
>gnl|SRA|SRR2638781.81245838.2 HWI-D00731:29:HGNNYADXX:1:2212:19959:60997.

-------------caggagtcctccaaactgcgccaacaaatcagtagcttacagaacgc
aaacagtaggaccatagtgggggattct--------------------------------
-atcaacaccatgagcctcagggaccttaaacaggtagagaacaggctggagaaagg---
-----catagctaagataag--ggct----------------------------------

>gnl|SRA|SRR2638781.30994467.2 HWI-D00731:29:HGNNYADXX:1:1208:10025:29353.

-------------caggagtcctccaaactgcgccaacaaatcagtagcttacagaacgc
aaacagtaggaccatagtgggggattct--------------------------------
-atcaacaccatgagcctcagggaccttaaacaggtagagaacaggctggagaaagg---
-----catagctaagataag--ggct----------------------------------

>gnl|SRA|SRR2638781.23935215.2 HWI-D00731:29:HGNNYADXX:1:1202:18060:69634.

-------------caggagtcctccaaactgcgccaacaaatcagtagcttacagaacgc
aaacagtaggaccatagtgggggattct--------------------------------
-atcaacaccatgagcctcagggaccttaaacaggtagagaacaggctggagaaagg---
-----catagctaagataag--ggct----------------------------------

>_R_gnl|SRA|SRR2638781.60231399.1 HWI-D00731:29:HGNNYADXX:1:2113:16955:28323.

------------gcaggagtcctccaaactgcgccaacaaatcagtagcttacagaacgc
aaacagtaggaccatagtgggggattct--------------------------------
-atcaacaccatgagcctcagggaccttaaacaggtagagaacaggctggagaaagg---
-----catagctaagataag--gac-----------------------------------

>gnl|SRA|SRR2638781.70248708.2 HWI-D00731:29:HGNNYADXX:1:2204:8439:57716.

----------cagcaggagtcctccaaactgcgccaacaaatcagtagcttacagaacgc
aaacagtaggaccatagtgggggattct--------------------------------
-atcaacaccatgagcctcagggaccttaaacaggtagagaacaggctggagaaagg---
-----catagctaagataag--g-------------------------------------

>_R_gnl|SRA|SRR2638781.66605906.1 HWI-D00731:29:HGNNYADXX:1:2201:2467:61069.

----------cagcaggagtcctccaaactgcgccaacaaatcagtagcttacagaacgc
aaacagtaggaccatagtgggggattct--------------------------------
-atcaacaccatgagcctcagggaccttaaacaggtagagaacaggctggagaaagg---
-----catagctaagataag--g-------------------------------------

>_R_gnl|SRA|SRR2638781.20477557.1 HWI-D00731:29:HGNNYADXX:1:1116:2622:10143.

----------cagcaggagtcctccaaactgcgccaacaaatcagtagcttacagaacgc
aaacagtaggaccatagtgggggattct--------------------------------
-atcaacaccatgagcctcagggaccttaaacaggtagagaacaggctggagaaagg---
-----catagctaagataag--g-------------------------------------

>_R_gnl|SRA|SRR2638781.9484945.1 HWI-D00731:29:HGNNYADXX:1:1108:9447:31286.

----------cagcaggagtcctccaaactgcgccaacaaatcagtagcttacagaacgc
aaacagtaggaccatagtgggggattct--------------------------------
-atcaacaccatgagcctcagggaccttaaacaggtagagaacaggctggagaaagg---
-----catagctaagataag--g-------------------------------------

>_R_gnl|SRA|SRR2638781.1691291.1 HWI-D00731:29:HGNNYADXX:1:1102:15102:33723.

----------cagcaggagtcctccaaactgcgccaacaaatcagtagcttacagaacgc
aaacagtaggaccatagtgggggattct--------------------------------
-atcaacaccatgagcctcagggaccttaaacaggtagagaacaggctggagaaagg---
-----catagctaagataag--g-------------------------------------

>gnl|SRA|SRR2638781.18379.2 HWI-D00731:29:HGNNYADXX:1:1101:8022:3420.

----------cagcaggagtcctccaaactgcgccaacaaatcagtagcttacagaacgc
aaacagtaggaccatagtgggggattct--------------------------------
-atcaacaccatgagcctcagggaccttaaacaggtagagaacaggctggagaaagg---
-----catagctaagataag--g-------------------------------------

>_R_gnl|SRA|SRR2638781.20340041.1 HWI-D00731:29:HGNNYADXX:1:1115:19333:99992.

----------cagcaggagtcctccaaactgcgccaacaaatcagtagcttacagaacgc
aaacagtaggaccatagtgggggattct--------------------------------
-atcaacgccatgagcctcagggaccttaaacaggtagagaacaggctggagaaagg---
-----catagctaagataag--g-------------------------------------

>gnl|SRA|SRR2638781.60231399.2 HWI-D00731:29:HGNNYADXX:1:2113:16955:28323.

---------ccagcaggagtcctccaaactgcgccaacaaatcagtagcttacagaacgc
aaacagtaggaccatagtgggggattct--------------------------------
-atcaacaccatgagcctcagggaccttaaacaggtagagaacaggctggagaaagg---

-----catagctaagataag--

>_R_gnl|SRA|SRR2638781.68881782.1 HWI-D00731:29:HGNNYADXX:1:2203:16623:43439.

-----------agcaggagtcctccaaactgcgccaacaaatcagtagcttacagaacgc
aaacagtaggaccatagtgggggattct--------------------------------
-atcaacaccatgagcctcagggaccttaaacaggtagagaacaggctggagaaagg---
-----catagctaagataag--gg------------------------------------

>gnl|SRA|SRR2638781.65938576.2 HWI-D00731:29:HGNNYADXX:1:2201:11490:8456.

-----------agcaggagtcctccaaactgcgccaacaaatcagtagcttacagaacgc
aaacagtaggaccatagtgggggattct--------------------------------
-atcaacaccatgagcctcagggaccttaaacaggtagagaacaggctggagaaagg---
-----catagctaagataag--gg------------------------------------

>_R_gnl|SRA|SRR2638781.58575663.1 HWI-D00731:29:HGNNYADXX:1:2112:19178:15451.

-----------agcaggagtcctccaaactgcgccaacaaatcagtagcttacagaacgc
aaacagtaggaccatagtgggggattct--------------------------------
-atcaacaccatgagcctcagggaccttaaacaggtagagaacaggctggagaaagg---
-----catagctaagataag--gg------------------------------------

>_R_gnl|SRA|SRR2638781.39345105.1 HWI-D00731:29:HGNNYADXX:1:1214:17195:26930.

-----------agcaggagtcctccaaactgcgccaacaaatcagtagcttacagaacgc
aaacagtaggaccatagtgggggattct--------------------------------
-atcaacaccatgagcctcagggaccttaaacaggtagagaacaggctggagaaagg---
-----catagctaagataag--gg------------------------------------

>_R_gnl|SRA|SRR2638781.29959722.1 HWI-D00731:29:HGNNYADXX:1:1207:10388:51386.

-----------agcaggagtcctccaaactgcgccaacaaatcagtagcttacagaacgc
aaacagtaggaccatagtgggggattct--------------------------------
-atcaacaccatgagcctcagggaccttaaacaggtagagaacaggctggagaaagg---
-----catagctaagataag--gg------------------------------------

>_R_gnl|SRA|SRR2638781.15109636.1 HWI-D00731:29:HGNNYADXX:1:1112:18463:35992.

-----------agcaggagtcctccaaactgcgccaacaaatcagtagcttacagaacgc
aaacagtaggaccatagtgggggattct--------------------------------
-atcaacaccatgagcctcagggaccttaaacaggtagagaacaggctggagaaagg---
-----catagctaagataag--gg------------------------------------

>_R_gnl|SRA|SRR2638781.3683366.1 HWI-D00731:29:HGNNYADXX:1:1103:17257:94935.

-----------agcaggagtcctccaaactgcgccaacaaatcagtagcttacagaacgc
aaacagtaggaccatagtgggggattct--------------------------------
-atcaacaccatgagcctcagggaccttaaacaggtagagaacaggctggagaaagg---
-----catagctaagataag--gg------------------------------------

>gnl|SRA|SRR2638781.3364689.2 HWI-D00731:29:HGNNYADXX:1:1103:8277:70644.

-----------agcaggagtcctccaaactgcgccaacaaatcagtagcttacagaacgc
aaacagtaggaccatagtgggggattct--------------------------------
-atcaacaacatgagcctcagggaccttaaacaggtagagaacaggctggagaaagg---
-----catagctaagataag--gg------------------------------------

>_R_gnl|SRA|SRR2638781.81002554.1 HWI-D00731:29:HGNNYADXX:1:2212:3186:44297.

--------------------------aactgcgccaacaaatcagtagcttacagaacgc
aaacagtaggaccatagtgggggattct--------------------------------
-atcaacaccatgagcctcagggaccttaaacaggtagagaacaggctggagaaagg---
-----catagctaagataag--ggctagaaagaatgagc---------------------

>_R_gnl|SRA|SRR2638781.72949870.1 HWI-D00731:29:HGNNYADXX:1:2206:13146:61983.

--------------------------aactgcgccaacaaatcagtagcttacagaacgc
aaacagtaggaccatagtgggggattct--------------------------------
-atcaacaccatgagcctcagggaccttaaacaggtagagaacaggctggagaaagg---
-----catagctaagataag--ggctagaaagaatgagc---------------------

>_R_gnl|SRA|SRR2638781.71712006.1 HWI-D00731:29:HGNNYADXX:1:2205:9310:68825.

--------------------------aactgcgccaacaaatcagtagcttacagaacgc
aaacagtaggaccatagtgggggattct--------------------------------
-atcaacaccatgagcctcagggaccttaaacaggtagagaacaggctggagaaagg---
-----catagctaagataag--ggctagaaagaatgagc---------------------

>_R_gnl|SRA|SRR2638781.61785021.1 HWI-D00731:29:HGNNYADXX:1:2114:6692:33035.

--------------------------aactgcgccaacaaatcagtagcttacagaacgc
aaacagtaggaccatagtgggggattct--------------------------------
-atcaacaccatgagcctcagggaccttaaacaggtagagaacaggctggagaaagg---
-----catagctaagataag--ggctagaaagaatgagc---------------------

>_R_gnl|SRA|SRR2638781.59321892.1 HWI-D00731:29:HGNNYADXX:1:2112:14917:66121.

--------------------------aactgcgccaacaaatcagtagcttacagaacgc

aaacagtaggaccatagtgggggattct--------------------------------
-atcaacaccatgagcctcagggaccttaaacaggtagagaacaggctggagaaagg---
-----catagctaagataag--ggctagaaagaatgagc---------------------

>_R_gnl|SRA|SRR2638781.49674845.1 HWI-D00731:29:HGNNYADXX:1:2105:6668:81180.

--------------------------aactgcgccaacaaatcagtagcttacagaacgc
aaacagtaggaccatagtgggggattct--------------------------------
-atcaacaccatgagcctcagggaccttaaacaggtagagaacaggctggagaaagg---
-----catagctaagataag--ggctagaaagaatgagc---------------------

>_R_gnl|SRA|SRR2638781.37157972.1 HWI-D00731:29:HGNNYADXX:1:1212:9028:74476.

--------------------------aactgcgccaacaaatcagtagcttacagaacgc
aaacagtaggaccatagtgggggattct--------------------------------
-atcaacaccatgagcctcagggaccttaaacaggtagagaacaggctggagaaagg---
-----catagctaagataag--ggctagaaagaatgagc---------------------

>_R_gnl|SRA|SRR2638781.4452946.1 HWI-D00731:29:HGNNYADXX:1:1104:14954:53672.

--------------------------aactgcgccaacaaatcagtagcttacagaacgc
aaacagtaggaccatagtgggggattct--------------------------------
-atcaacaccatgagcctcagggaccttaaacaggtagagaacaggctggagaaagg---
-----catagctaagataag--ggctagaaagaatgagc---------------------

>_R_gnl|SRA|SRR2638781.72441268.1 HWI-D00731:29:HGNNYADXX:1:2206:16183:23782.

---------------------------actgcgccaacaaatcagtagcttacagaacgc
aaacagtaggaccatagtgggggattct--------------------------------
-atcaacaccatgagcctcagggaccttaaacaggtagagaacaggctggagaaagg---
-----catagctaagataag--ggctagaaagaatgagcc--------------------

>_R_gnl|SRA|SRR2638781.31163255.1 HWI-D00731:29:HGNNYADXX:1:1208:12042:41825.

---------------------------actgcgccaacaaatcagtagcttacagaacgc
aaacagtaggaccatagtgggggattct--------------------------------
-atcaacaccatgagcctcagggaccttaaacaggtagagaacaggctggagaaagg---
-----catagctaagataag--ggctagaaagaatgagcc--------------------

>_R_gnl|SRA|SRR2638781.60625520.1 HWI-D00731:29:HGNNYADXX:1:2113:6120:55166.

---------------------------actgcgccaacaaatcagtagcttacagaacgc
aaacagtaggaccatagtgggggattct--------------------------------
-atcaacaccatgagcctcagggaccttaaacaggtagagaacaggctggagaaagg---
-----catagctaagataag--ggctagaaagaatgagca--------------------

>_R_gnl|SRA|SRR2638781.70990392.1 HWI-D00731:29:HGNNYADXX:1:2205:19616:14477.

-------------------------aaactgcgccaacaaatcagtagcttacagaacgc
aaacagtaggaccatagtgggggattct--------------------------------
-atcaacaccatgagcctcagggaccttaaacaggtagagaacaggctggagaaagg---
-----catagctaagataag--ggctagaaagaatgag----------------------

>_R_gnl|SRA|SRR2638781.68971127.1 HWI-D00731:29:HGNNYADXX:1:2203:16975:54918.

-------------------------aaactgcgccaacaaatcagtagcttacagaacgc
aaacagtaggaccatagtgggggattct--------------------------------
-atcaacaccatgagcctcagggaccttaaacaggtagagaacaggctggagaaagg---
-----catagctaagataag--ggctagaaagaatgag----------------------

>_R_gnl|SRA|SRR2638781.60506947.1 HWI-D00731:29:HGNNYADXX:1:2113:6533:47239.

-------------------------aaactgcgccaacaaatcagtagcttacagaacgc
aaacagtaggaccatagtgggggattct--------------------------------
-atcaacaccatgagcctcagggaccttaaacaggtagagaacaggctggagaaagg---
-----catagctaagataag--ggctagaaagaatgag----------------------

>_R_gnl|SRA|SRR2638781.34490850.1 HWI-D00731:29:HGNNYADXX:1:1210:7015:84354.

-------------------------aaactgcgccaacaaatcagtagcttacagaacgc
aaacagtaggaccatagtgggggattct--------------------------------
-atcaacaccatgagcctcagggaccttaaacaggtagagaacaggctggagaaagg---
-----catagctaagataag--ggctagaaagaatgag----------------------

>_R_gnl|SRA|SRR2638781.19431769.1 HWI-D00731:29:HGNNYADXX:1:1115:15363:37485.

-------------------------aaactgcgccaacaaatcagtagcttacagaacgc
aaacagtaggaccatagtgggggattct--------------------------------
-atcaacaccatgagcctcagggaccttaaacaggtagagaacaggctggagaaagg---
-----catagctaagataag--ggctagaaagaatgag----------------------

>_R_gnl|SRA|SRR2638781.2293712.1 HWI-D00731:29:HGNNYADXX:1:1102:17990:80033.

-------------------------aaactgcgccaacaaatcagtagcttacagaacgc
aaacagtaggaccatagtgggggattct--------------------------------
-atcaacaccatgagcctcagggaccttaaacaggtagagaacaggctggagaaagg---
-----catagctaagataag--ggctagaaagaatgag----------------------

>gnl|SRA|SRR2638781.64341684.2 HWI-D00731:29:HGNNYADXX:1:2116:8253:2538.

------------------------caaactgcgccaacaaatcagtagcttacagaacgc
aaacagtaggaccatagtgggggattct--------------------------------
-atcaacaccatgagcctcagggaccttaaacaggtagagaacaggctggagaaagg---
-----catagctaagataag--ggctagaaagaatga-----------------------

>gnl|SRA|SRR2638781.44794196.2 HWI-D00731:29:HGNNYADXX:1:2102:11691:9689.

------------------------caaactgcgccaacaaatcagtagcttacagaacgc
aaacagtaggaccatagtgggggattct--------------------------------
-atcaacaccatgagcctcagggaccttaaacaggtagagaacaggctggagaaagg---
-----catagctaagataag--ggctagaaagaatga-----------------------

>gnl|SRA|SRR2638781.8231078.2 HWI-D00731:29:HGNNYADXX:1:1107:20279:37056.

-----------------------ccaaactgcgccaacaaatcagtagcttacagaacgc
aaacagtaggaccatagtgggggattct--------------------------------
-atcaacaccatgagcctcagggaccttaaacaggtagagaacaggctggagaaagg---
-----catagctaagataag--ggctagaaagaatg------------------------

>_R_gnl|SRA|SRR2638781.77848125.1 HWI-D00731:29:HGNNYADXX:1:2210:8089:21675.

----------------------------ctgcgccaacaaatcagtagcttacagaacgc
aaacagtaggaccatagtgggggattct--------------------------------
-atcaacaccatgagcctcagggaccttaaacaggtagagaacaggctggagaaagg---
-----catagctaagataag--ggctagaaagaatgagctg-------------------

>_R_gnl|SRA|SRR2638781.18204765.1 HWI-D00731:29:HGNNYADXX:1:1114:14353:52257.

----------------------------ctgcgccaacaaatcagtagcttacagaacgc
aaacagtaggaccatagtgggggattct--------------------------------
-atcaacaccatgagcctcagggaccttaaacaggtagagaacaggctggagaaagg---
-----catagctaagataag--ggctagaaagaatgagctg-------------------

>_R_gnl|SRA|SRR2638781.66374670.1 HWI-D00731:29:HGNNYADXX:1:2201:3050:42906.

----------------------------ctgcgccaacaaatcagtagcttacagaacgc
aaacagtaggaccatagtgggggattct--------------------------------
-atcaacaccatgagcctcagggaccttaaacaggtagagaacaggctggagaaagg---
-----catagctaagataag--ggctagaaagaatgagccg-------------------

>_R_gnl|SRA|SRR2638781.46875946.1 HWI-D00731:29:HGNNYADXX:1:2103:12369:73354.

----------------------------ctgcgccaacaaatcagtagcttacagaacgc
aaacagtaggaccatagtgggggattct--------------------------------

-atcaacaccatgagcctcagggaccttaaacaggtagagaacaggctggagaaagg---
-----catagctaagataag--ggctagaaagaatgagccg-------------------

>_R_gnl|SRA|SRR2638781.75410784.1 HWI-D00731:29:HGNNYADXX:1:2208:4245:45081.

----------------------------ctgcgccaacaaatcagtagcttacagaacgc
aaacagtaggaccatagtgggggattct--------------------------------
-atcaacaccatgagcctcagggaccttaaacaggtagagaacaggctggagaaagg---
-----catagctaagataac--ggctagaaagaatgagctg-------------------

>gnl|SRA|SRR2638781.49114740.2 HWI-D00731:29:HGNNYADXX:1:2105:15612:40059.

---------------------ctccaaactgcgccaacaaatcagtagcttacagaacgc
aaacagtaggaccatagtgggggattct--------------------------------
-atcaacaccatgagcctcagggaccttaaacaggtagagaacaggctggagaaagg---
-----catagctaagataag--ggctagaaagaa---------------------------
----
>gnl|SRA|SRR2638781.36873200.2 HWI-D00731:29:HGNNYADXX:1:1212:18923:54486.

---------------------ctccaaactgcgccaacaaatcagtagcttacagaacgc
aaacagtaggaccatagtgggggattct--------------------------------
-atcaacaccatgagcctcagggaccttaaacaggtagagaacaggctggagaaagg---
-----catagctaagataag--ggctagaaagaa---------------------------
----
>gnl|SRA|SRR2638781.35985204.2 HWI-D00731:29:HGNNYADXX:1:1211:7079:91463.

---------------------ctccaaactgcgccaacaaatcagtagcttacagaacgc
aaacagtaggaccatagtgggggattct--------------------------------
-atcaacaccatgagcctcagggaccttaaacaggtagagaacaggctggagaaagg---
-----catagctaagataag--ggctagaaagaa---------------------------
----
>gnl|SRA|SRR2638781.16487161.2 HWI-D00731:29:HGNNYADXX:1:1113:18428:32821.

---------------------ctccaaactgcgccaacaaatcagtagcttacagaacgc
aaacagtaggaccatagtgggggattct--------------------------------
-atcaacaccatgagcctcagggaccttaaacaggtagagaacaggctggagaaagg---
-----catagctaagataag--ggctagaaagaa---------------------------
----
>_R_gnl|SRA|SRR2638781.26464312.1 HWI-D00731:29:HGNNYADXX:1:1204:15198:84342.

----------------------tccaaactgcgccaacaaatcagtagcttacagaacgc
aaacagtaggaccatagtgggggattct--------------------------------
-atcaacaccatgagcctcagggaccttaaacaggtagagaacaggctggagaaagg---
-----catagctaagataag--ggctagaaagaat--------------------------
----

>gnl|SRA|SRR2638781.48825335.2 HWI-D00731:29:HGNNYADXX:1:2105:14012:18879.

----------------------tccaaactgcgccaacaaatcagtagcttacagaacgc
aaacagtagcaccatagtgggggattct--------------------------------
-atcaacaccatgagcctcagggaccttaaacagggagagaacaggctggagaaagg---
-----catagctaagataag--ggctagaaagaat-------------------------

>gnl|SRA|SRR2638781.76909230.2 HWI-D00731:29:HGNNYADXX:1:2209:3267:53861.

----------------gagtcctccaaactgcgccaacaaatcagtagcttacagaacgc
aaacagtaggaccatagtgggggattct--------------------------------
-atcaacaccatgagcctcagggaccttaaacaggtagagaacaggctggagaaagg---
-----catagctaagataag--ggctaga-------------------------------

>_R_gnl|SRA|SRR2638781.12107325.1 HWI-D00731:29:HGNNYADXX:1:1110:10282:21728.

----------------gagtcctccaaactgcgccaacaaatcagtagcttacagaacgc
aaacagtaggaccatagtgggggattct--------------------------------
-atcaacaccatgagcctcagggaccttaaacaggtagagaacaggctggagaaagg---
-----catagctaagataag--ggctaga-------------------------------

>gnl|SRA|SRR2638781.72949870.2 HWI-D00731:29:HGNNYADXX:1:2206:13146:61983.

-----------------agtcctccaaactgcgccaacaaatcagtagcttacagaacgc
aaacagtaggaccatagtgggggattct--------------------------------
-atcaacaccatgagcctcagggaccttaaacaggtagagaacaggctggagaaagg---
-----catagctaagataag--ggctagaa------------------------------

>gnl|SRA|SRR2638781.49674845.2 HWI-D00731:29:HGNNYADXX:1:2105:6668:81180.

-----------------agtcctccaaactgcgccaacaaatcagtagcttacagaacgc
aaacagtaggaccatagtgggggattct--------------------------------
-atcaacaccatgagcctcagggaccttaaacaggtagagaacaggctggagaaagg---
-----catagctaagataag--ggctagaa------------------------------

>gnl|SRA|SRR2638781.13191693.2 HWI-D00731:29:HGNNYADXX:1:1110:2734:99021.

---------------ggagtcctccaaactgcgccaacaaatcagtagcttacagaacgc
aaacagtaggaccatagtgggggattct--------------------------------
-atcaacaccatgagcctcagggaccttaaacaggtagagaacaggctggagaaagg---
-----catagctaagataag--ggctag--------------------------------

>gnl|SRA|SRR2638781.59383474.2 HWI-D00731:29:HGNNYADXX:1:2112:7826:70483.

------------------gtcctccaaactgcgccaacaaatcagtagcttacagaacgc
aaacagtaggaccatagtgggggattct--------------------------------
-atcaacaccatgagcctcagggaccttaaacaggtagagaacaggctggagaaagg---
-----catagctaagataag--ggctagaaa-----------------------------

>gnl|SRA|SRR2638781.15406428.2 HWI-D00731:29:HGNNYADXX:1:1112:8257:56986.

------------------gtcctccaaactgcgccaacaaatcagtagcttacagaacgc
aaacagtaggaccatagtgggggattct--------------------------------
-atcaacaccatgagcctcagggaccttaaacaggtagagaacaggctggagaaagg---
-----catagctaagataag--ggctagaaa-----------------------------

>_R_gnl|SRA|SRR2638781.6694474.1 HWI-D00731:29:HGNNYADXX:1:1106:6657:22428.

-------------------tcctccaaactgcgccaacaaatcagtagcttacagaacgc
aaacagtaggaccatagtgggggattct--------------------------------
-atcaacaccatgagcctcagggaccttaaacaggtagagaacaggctggagaaagg---
-----catagctaagataag--ggctagaaag----------------------------

>gnl|SRA|SRR2638781.76449451.2 HWI-D00731:29:HGNNYADXX:1:2209:13641:20569.

-------------caggagtcctccaaactgcgccaacaaatcagtagcttacagaacgc
aaac---aggaccatagtgggggattct--------------------------------
-atcaacaccatgagcctcagggaccttaaacaggtagagaacaggctggagaaagg---
-----catagctaagataag--ggctaga-------------------------------

>_R_gnl|SRA|SRR2638781.27041229.1 HWI-D00731:29:HGNNYADXX:1:1205:20198:29248.

------------gcaggagtcctccaaactgcgccaacaaatcagtagcttacagaacgc
aaac---aggaccatagtgggggattct--------------------------------
-atcaacaccatgagcctcagggaccttaaacaggtagagaacaggctggagaaagg---
-----catagctaagataag--ggctag--------------------------------

>_R_gnl|SRA|SRR2638781.73115415.1 HWI-D00731:29:HGNNYADXX:1:2206:15197:74198.

------ctaccagcaggagtcctccaaactgcgccaacaaatcagtagcttacagaacgc
aaac---aggaccatagtgggggattct--------------------------------
-atcaacaccatgagcctcagggaccttaaacaggtagagaacaggctggagaaagg---
-----catagctaagataag--

>gnl|SRA|SRR2638781.62765139.2 HWI-D00731:29:HGNNYADXX:1:2114:2802:97851.

------ctaccagcaggagtcctccaaactgcgccaacaaatcagtagcttacagaacgc
aaac---aggaccatagtgggggattct--------------------------------
-atcaacaccatgagcctcagggaccttaaacaggtagagaacaggctggagaaagg---

-----catagctaagataag--

>gnl|SRA|SRR2638781.52655877.2 HWI-D00731:29:HGNNYADXX:1:2107:15150:98786.

------ctaccagcaggagtcctccaaactgcgccaacaaatcagtagcttacagaacgc
aaac---aggaccatagtgggggattct--------------------------------
-atcaacaccatgagcctcagggaccttaaacaggtagagaacaggctggagaaagg---
-----catagctaagataag--

>_R_gnl|SRA|SRR2638781.64471244.1 HWI-D00731:29:HGNNYADXX:1:2116:19443:10899.

-------taccagcaggagtcctccaaactgcgccaacaaatcagtagcttacagaacgc
aaac---aggaccatagtgggggattct--------------------------------
-atcaacaccatgagcctcagggaccttaaacaggtagagaacaggctggagaaagg---
-----catagctaagataag--g-------------------------------------

>_R_gnl|SRA|SRR2638781.37533135.1 HWI-D00731:29:HGNNYADXX:1:1212:7345:100624.

-------taccagcaggagtcctccaaactgcgccaacaaatcagtagcttacagaacgc
aaac---aggaccatagtgggggattct--------------------------------
-atcaacaccatgagcctcagggaccttaaacaggtagagaacaggctggagaaagg---
-----catagctaagataag--g-------------------------------------

>_R_gnl|SRA|SRR2638781.58119279.1 HWI-D00731:29:HGNNYADXX:1:2111:6000:83527.

--------accagcaggagtcctccaaactgcgccaacaaatcagtagcttacagaacgc
aaac---aggaccatagtgggggattct--------------------------------
-atcaacaccatgagcctcagggaccttaaacaggtagagaacaggctggagaaagg---
-----catagctaagataag--gg------------------------------------

>_R_gnl|SRA|SRR2638781.58049945.1 HWI-D00731:29:HGNNYADXX:1:2111:1957:78894.

--------accagcaggagtcctccaaactgcgccaacaaatcagtagcttacagaacgc
aaac---aggaccatagtgggggattct--------------------------------
-atcaacaccatgagcctcagggaccttaaacaggtagagaacaggctggagaaagg---
-----catagctaagataag--gg------------------------------------

>_R_gnl|SRA|SRR2638781.19249613.1 HWI-D00731:29:HGNNYADXX:1:1115:12964:24934.

--------accagcaggagtcctccaaactgcgccaacaaatcagtagcttacagaacgc
aaac---aggaccatagtgggggattct--------------------------------
-atcaacaccatgagcctcagggaccttaaacaggtagagaacaggctggagaaagg---
-----catagctaagataag--gg------------------------------------

>_R_gnl|SRA|SRR2638781.15661915.1 HWI-D00731:29:HGNNYADXX:1:1112:13436:74600.

--------accagcaggagtcctccaaactgcgccaacaaatcagtagcttacagaacgc
aaac---aggaccatagtgggggattct--------------------------------
-atcaacaccatgagcctcagggaccttaaacaggtagagaacaggctggagaaagg---
-----catagctaagataag--gg------------------------------------

>_R_gnl|SRA|SRR2638781.15653075.1 HWI-D00731:29:HGNNYADXX:1:1112:3828:74040.

--------accagcaggagtcctccaaactgcgccaacaaatcagtagcttacagaacgc
aaac---aggaccatagtgggggattct--------------------------------
-atcaacaccatgagcctcagggaccttaaacaggtagagaacaggctggagaaagg---
-----catagctaagataag--gg------------------------------------

>_R_gnl|SRA|SRR2638781.15862939.1 HWI-D00731:29:HGNNYADXX:1:1112:1476:88962.

---------ccagcaggagtcctccaaactgcgccaacaaatcagtagcttacagaacgc
aaac---aggaccatagtgggggattct--------------------------------
-atcaacaccatgagcctcagggaccttaaacaggtagagaacaggctggagaaagg---
-----catagctaagataag--ggc-----------------------------------

>gnl|SRA|SRR2638781.7312591.2 HWI-D00731:29:HGNNYADXX:1:1106:7490:68041.

----------cagcaggagtcctccaaactgcgccaacaaatcagtagcttacagaacgc
aaac---aggaccatagtgggggattct--------------------------------
-atcaacaccatgagcctcagggaccttaaacaggtagagaacaggctggagaaagg---
-----catagctaagataag--ggct----------------------------------

>gnl|SRA|SRR2638781.73260103.2 HWI-D00731:29:HGNNYADXX:1:2206:10571:84987.

-cagcactaccagcaggagtcctccaaactgcgccaacaaatcagtagcttacagaacgc
aaacagtaggaccatagtgggggattct--------------------------------
-atcaacaccatgagcctcagggaccttaaacaggtagagaacaggctggagaaagg---
-----catagct--

>gnl|SRA|SRR2638781.25018539.2 HWI-D00731:29:HGNNYADXX:1:1203:7150:71350.

-cagcactaccagcaggagtcctccaaactgcgccaacaaatcagtagcttacagaacgc
aaacagtaggaccatagtgggggattct--------------------------------
-atcaacaccatgagcctcagggaccttaaacaggtagagaacaggctggagaaagg---
-----catagct--

>gnl|SRA|SRR2638781.24718910.2 HWI-D00731:29:HGNNYADXX:1:1203:12840:33517.

-cagcactaccagcaggagtcctccaaactgcgccaacaaatcagtagcttacagaacgc

aaacagtaggaccatagtgggggattct--------------------------------
-atcaacaccatgagcctcagggaccttaaacaggtagagaacaggctggagaaagg---
-----catagct--

>gnl|SRA|SRR2638781.66374670.2 HWI-D00731:29:HGNNYADXX:1:2201:3050:42906.

--agcactaccagcaggagtcctccaaactgcgccaacaaatcagtagcttacagaacgc
aaacagtaggaccatagtgggggattct--------------------------------
-atcaacaccatgagcctcagggaccttaaacaggtagagaacaggctggagaaagg---
-----catagcta---

>gnl|SRA|SRR2638781.46875946.2 HWI-D00731:29:HGNNYADXX:1:2103:12369:73354.

--agcactaccagcaggagtcctccaaactgcgccaacaaatcagtagcttacagaacgc
aaacagtaggaccatagtgggggattct--------------------------------
-atcaacaccatgagcctcagggaccttaaacaggtagagaacaggctggagaaagg---
-----catagcta---

>gnl|SRA|SRR2638781.34776412.2 HWI-D00731:29:HGNNYADXX:1:1211:16539:5405.

---gcactaccagcaggagtcctccaaactgcgccaacaaatcagtagcttacagaacgc
aaacagtaggaccatagtgggggattct--------------------------------
-atcaacaccatgagcctcagggaccttaaacaggtagagaacaggctggagaaagg---
-----catagctaa--

>gnl|SRA|SRR2638781.66806440.2 HWI-D00731:29:HGNNYADXX:1:2201:19067:76518.

----cactaccagcaggagtcctccaaactgcgccaacaaatcagtagcttacagaacgc
aaacagtaggaccatagtgggggattct--------------------------------
-atcaacaccatgagcctcagggaccttaaacaggtagagaacaggctggagaaagg---
-----catagctaag---

>_R_gnl|SRA|SRR2638781.56235053.1 HWI-D00731:29:HGNNYADXX:1:2110:19470:52992.

----cactaccagcaggagtcctccaaactgcgccaacaaatcagtagcttacagaacgc
aaacagtaggaccatagtgggggattct--------------------------------
-atcaacaccatgagcctcagggaccttaaacaggtagagaacaggctggagaaagg---
-----catagctaag---

>_R_gnl|SRA|SRR2638781.45893357.1 HWI-D00731:29:HGNNYADXX:1:2102:19297:93245.

----cactaccagcaggagtcctccaaactgcgccaacaaatcagtagcttacagaacgc
aaacagtaggaccatagtgggggattct--------------------------------
-atcaacaccatgagcctcagggaccttaaacaggtagagaacaggctggagaaagg---
-----catagctaag---

>_R_gnl|SRA|SRR2638781.39979017.1 HWI-D00731:29:HGNNYADXX:1:1214:19466:70150.

----cactaccagcaggagtcctccaaactgcgccaacaaatcagtagcttacagaacgc
aaacagtaggaccatagtgggggattct--------------------------------
-atcaacaccatgagcctcagggaccttaaacaggtagagaacaggctggagaaagg---
-----catagctaag---

>_R_gnl|SRA|SRR2638781.11637233.1 HWI-D00731:29:HGNNYADXX:1:1109:2928:87380.

----cactaccagcaggagtcctccaaactgcgccaacaaatcagtagcttacagaacgc
aaacagtaggaccatagtgggggattct--------------------------------
-atcaacaccatgagcctcagggaccttaaacaggtagagaacaggctggagaaagg---
-----catagctaag---

>_R_gnl|SRA|SRR2638781.10043642.1 HWI-D00731:29:HGNNYADXX:1:1108:13586:71978.

----cactaccagcaggagtcctccaaactgcgccaacaaatcagtagcttacagaacgc
aaacagtaggaccatagtgggggattct--------------------------------
-atcaacaccatgagcctcagggaccttaaacaggtagagaacaggctggagaaagg---
-----catagctaag---

>_R_gnl|SRA|SRR2638781.65798071.1 HWI-D00731:29:HGNNYADXX:1:2116:9579:97446.

----ctctaccagcaggagtcctccaaactgcgccaacaaatcagtagcttacagaacgc
aaacagtaggaccatagtgggggattct--------------------------------
-atcaacaccatgagcctcagggaccttaaacaggtagagaacaggctggagaaagg---
-----catagctaag---

>gnl|SRA|SRR2638781.3858794.2 HWI-D00731:29:HGNNYADXX:1:1104:13277:8787.

----
ccagcactaccagcaggagtcctccaaactgcgccaacaaatcagtagcttacagaacgc
aaacagtaggaccatagtgggggattct--------------------------------
-atcaacaccatgagcctcagggaccttaaacaggtagagaacaggctggagaaagg---
-----catagc---

>gnl|SRA|SRR2638781.66177437.2 HWI-D00731:29:HGNNYADXX:1:2201:13642:27245.

------ctaccagcaggagtcctccaaactgcgccaacaaatcagtagcttacagaacgc
aaacagtaggaccatagtgggggattct--------------------------------
-atcaacaccatgagcctcagggaccttaaacaggtagagaacaggctggagaaagg---
-------agatcggaagag---

>gnl|SRA|SRR2638781.65989590.2 HWI-D00731:29:HGNNYADXX:1:2201:11499:12327.

------ctaccagcaggagtcctccaaactgcgccaacaaatcagtagcttacagaacgc
aaacagtaggaccatagtgggggattct--------------------------------
-atcaacaccatgagcctcagggaccttaaacaggtagagaacaggctggagaaagg---
-----catagctaagat---

>gnl|SRA|SRR2638781.26853770.2 HWI-D00731:29:HGNNYADXX:1:1205:15576:14864.

------ctaccagcaggagtcctccaaactgcgccaacaaatcagtagcttacagaacgc
aaacagtaggaccatagtgggggattct--------------------------------
-atcaacaccatgagcctcagggaccttaaacaggtagagaacaggctggagaaagg---
-----catagctaagat---

>gnl|SRA|SRR2638781.65798071.2 HWI-D00731:29:HGNNYADXX:1:2116:9579:97446.

------ctaccagcaggagtcctccaaactgcgccaacaaatcagtagcttacagaacgc
aaacagtaggaccatagtgggggattct--------------------------------
-atcaacaccatgagcctcagggaccttaaacaggtagagaacaggctggagaaagg---
-----catagctaagag---

>gnl|SRA|SRR2638781.10838607.2 HWI-D00731:29:HGNNYADXX:1:1109:17383:29933.

------ctaccagcaggagtcctccaaactgcgccaacaaatcagtagcttacagaacgc
aaacagtaggaccatagtgggggattct--------------------------------
-atcaacaccatgagcctcagggaccttaaacaggtagagaacaggctggagaaagg---
-----ccagatcggaag---

>gnl|SRA|SRR2638781.86457566.2 HWI-D00731:29:HGNNYADXX:1:2216:16138:15073.

--agcactaccagcaggagtcctccaaactgcgccaacaaatcagtagcttacagaacgc
aaac---aggaccatagtgggggattct--------------------------------
-atcaacaccatgagcctcagggaccttaaacaggtagagaacaggctggagaaagg---
-----catagctaaga--

>_R_gnl|SRA|SRR2638781.30128333.1 HWI-D00731:29:HGNNYADXX:1:1207:14069:64223.

---gcactaccagcaggagtcctccaaactgcgccaacaaatcagtagcttacagaacgc
aaac---aggaccatagtgggggattct--------------------------------
-atcaacaccatgagcctcagggaccttaaacaggtagagaacaggctggagaaagg---
-----catagctaagat---

>_R_gnl|SRA|SRR2638781.16037220.1 HWI-D00731:29:HGNNYADXX:1:1112:13511:100985.

-cagcactaccagcaggagtcctccaaactgcgccaacaaatcagtagcttacagaacgc
aaac---aggaccatagtgggggattct--------------------------------

-atcaacaccatgagcctcagggaccttaaacaggtagagaacaggctggagaaagg---
-----catagctaag---

>_R_gnl|SRA|SRR2638781.83013949.1 HWI-D00731:29:HGNNYADXX:1:2213:8578:82931.

-------------------tcctccaaactgcgccaacaaatcagtagcttacagaacgc
aaac---aggaccatagtgggggattct--------------------------------
-atcaacaccatgagcctcagggaccttaaacaggtagagaacaggctggagaaagg---
-----catagctaagataag--ggctagaaagaat-------------------------

>gnl|SRA|SRR2638781.54544316.2 HWI-D00731:29:HGNNYADXX:1:2109:7052:34163.

--------------------cctccaaactgcgccaacaaatcagtagcttacagaacgc
aaac---aggaccatagtgggggattct--------------------------------
-atcaacaccatgagcctcagggaccttaaacaggtagagaacaggctggagaaagg---
-----catagctaagataag--ggctagaaagaatg------------------------

>gnl|SRA|SRR2638781.17864095.2 HWI-D00731:29:HGNNYADXX:1:1114:12551:28945.

--------------------cctccaaactgcgccaacaaatcagtagcttacagaacgc
aaac---aggaccatagtgggggattct--------------------------------
-atcaacaccatgagcctcagggaccttaaacaggtagagaacaggctggagaaagg---
-----catagctaagataag--ggctagaaagaatg------------------------

>gnl|SRA|SRR2638781.54102331.2 HWI-D00731:29:HGNNYADXX:1:2109:5653:3126.

------------------gtcctccaaactgcgccaacaaatcagtagcttacagaacgc
aaac---aggaccatagtgggggattct--------------------------------
-atcaacaccatgagcctcagggaccttaaacaggtagagaacaggctggagaaagg---
-----catagctaagataag--ggctagaaagaa--------------------------

>gnl|SRA|SRR2638781.62492182.2 HWI-D00731:29:HGNNYADXX:1:2114:13944:79926.

---------------------ctccaaactgcgccaacaaatcagtagcttacagaacgc
aaac---aggaccatagtgggggattct--------------------------------
-atcaacaccatgagcctcagggaccttaaacaggtagagaacaggctggagaaagg---
-----catagctaagataag--ggctagaaagaatga-----------------------

>gnl|SRA|SRR2638781.32110104.2 HWI-D00731:29:HGNNYADXX:1:1209:6129:11981.

---------------------ctccaaactgcgccaacaaatcagtagcttacagaacgc
aaac---aggaccatagtgggggattct--------------------------------
-atcaacaccatgagcctcagggaccttaaacaggtagagaacaggctggagaaagg---
-----catagctaagataag--ggctagaaagaatga-----------------------

>gnl|SRA|SRR2638781.17194189.2 HWI-D00731:29:HGNNYADXX:1:1113:15949:81967.

---------------------ctccaaactgcgccaacaaatcagtagcttacagaacgc
aaac---aggaccatagtgggggattct--------------------------------
-atcaacaccatgagcctcagggaccttaaacaggtagagaacaggctggagaaagg---
-----catagctaagataag--ggctagaaagaatga-----------------------

>gnl|SRA|SRR2638781.22528503.2 HWI-D00731:29:HGNNYADXX:1:1201:18836:58253.

---------------------ctccaaactgcgccaacaaatcagtagcttacagaacgc
aaac---aggaccatagcgggggattct--------------------------------
-atcaacaccatgagcctcaggtaccttaaacaggtagagaacaggccggagaaagg---
-----catagctaagataag--ggctagaaagaatga-----------------------

>_R_gnl|SRA|SRR2638781.56973598.1 HWI-D00731:29:HGNNYADXX:1:2111:20903:4542.

-----------------------ccaaactgcgccaacaaatcagtagcttacagaacgc
aaac---aggaccatagtgggggattct--------------------------------
-atcaacaccatgagcctcagggaccttaaacaggtagagaacaggctggagaaagg---
-----catagctaagataag--ggctagaaagaatgagc---------------------

>gnl|SRA|SRR2638781.55657072.2 HWI-D00731:29:HGNNYADXX:1:2110:7254:12935.

-----------------------ccaaactgcgccaacaaatcagtagcttacagaacgc
aaac---aggaccatagtgggggattct--------------------------------
-atcaacaccatgagcctcagggaccttaaacaggtagagaacaggctggagaaagg---
-----catagctaagataag--ggctagaaagaatgagc---------------------

>_R_gnl|SRA|SRR2638781.35748915.1 HWI-D00731:29:HGNNYADXX:1:1211:18889:74453.

-----------------------ccaaactgcgccaacaaatcagtagcttacagaacgc
aaac---aggaccatagtgggggattct--------------------------------
-atcaacaccatgagcctcagggaccttaaacaggtagagaacaggctggagaaagg---
-----catagctaagataag--ggctagaaagaatgagc---------------------

>gnl|SRA|SRR2638781.7984283.2 HWI-D00731:29:HGNNYADXX:1:1107:21310:18317.

-----------------------ccaaactgcgccaacaaatcagtagcttacagaacgc
aaac---aggaccatagtgggggattct--------------------------------
-atcaacaccatgagcctcagggaccttaaacaggtagagaacaggctggagaaagg---
-----catagctaagataag--ggctagaaagaatgagc---------------------

>_R_gnl|SRA|SRR2638781.1514671.1 HWI-D00731:29:HGNNYADXX:1:1102:3012:20168.

-----------------------ccaaactgcgccaacaaatcagtagcttacagaacgc
aaac---aggaccatagtgggggattct--------------------------------
-atcaacaccatgagcctcagggaccttaaacaggtagagaacaggctggagaaagg---
-----catagctaagataag--ggctagaaagaatgagc---------------------

>_R_gnl|SRA|SRR2638781.1042207.1 HWI-D00731:29:HGNNYADXX:1:1101:3978:83123.

-----------------------ccaaactgcgccaacaaatcagtagcttacagaacgc
aaac---aggaccatagtgggggattct--------------------------------
-atcaacaccatgagcctcagggaccttaaacaggtagagaacaggctggagaaagg---
-----catagctaagataag--ggctagaaagaatgagc---------------------

>gnl|SRA|SRR2638781.14456949.2 HWI-D00731:29:HGNNYADXX:1:1111:12487:89349.

------------------------caaactgcgccaacaaatcagtagcttacagaacgc
aaac---aggaccatagtgggggattct--------------------------------
-atcaacaccatgagcctcagggaccttaaacaggtagagaacaggctggagaaagg---
-----catagctaagataag--ggctagaaagaatgagct--------------------

>_R_gnl|SRA|SRR2638781.25564843.1 HWI-D00731:29:HGNNYADXX:1:1204:19024:14972.

-------------------------aaactgcgccaacaaatcagtagcttacagaacgc
aaac---aggaccatagtgggggattct--------------------------------
-atcaacaccatgagcctcagggaccttaaacaggtagagaacaggctggagaaagg---
-----catagctaagataag--ggctagaaagaatgagctg-------------------

>_R_gnl|SRA|SRR2638781.64939304.1 HWI-D00731:29:HGNNYADXX:1:2116:19422:41702.

----------------------tccaaactgcgccaacaaatcagtagcttacagaacgc
aaac---aggaccatagtgggggattct--------------------------------
-atcaaccccacgagcctcagggaccttaaacaggtagagaacaggctggagaaagg---
-----catagctaagataag--ggctagaaagaatgag----------------------

>_R_gnl|SRA|SRR2638781.63462417.1 HWI-D00731:29:HGNNYADXX:1:2115:18705:44198.

---------ttccgatctgtcctccaaactgcgccaacaaatcagtagcttacagaacgc
aaacagtaggaccatagtgggggattct--------------------------------
-atcaacaccatgagcctcagggaccttaaacaggtagagaacaggctggagaaagg---
-----catagctaagataag--

>_R_gnl|SRA|SRR2638781.28761846.1 HWI-D00731:29:HGNNYADXX:1:1206:4993:60314.

---------ttccgatctgtcctccaaactgcgccaacaaatcagtagcttacagaacgc
aaacagtaggaccatagtgggggattct--------------------------------
-atcaacaccatgagcctcagggaccttaaacaggtagagaacaggctggagaaagg---

-----catagctaagataag--

>gnl|SRA|SRR2638781.48756639.2 HWI-D00731:29:HGNNYADXX:1:2105:11909:13869.

--c-------
ccagcactaccagaaggagtcctccaaactgcgccaacaaatcagtggcttacagaacgc
aaac---aggaccatagtgggggattct--------------------------------
-atcaacaccatgagcctcagggaccttaaacaggtagagaacaggctggagaaagg---
-----catagcta---

>gnl|SRA|SRR2638781.65346736.2 HWI-D00731:29:HGNNYADXX:1:2116:4231:68586.

-------------------------aaactgcgccaacaaatcagtagcttacagaacgc
aaacagtaggaccatagtgggggattct--------------------------------
-atcaacaccatgagcctcagggaccttaaacaggtagagaacaggctggagaaagg---
-----catagctaagataa---ggagatcggaagagcgt---------------------

>gnl|SRA|SRR2638781.8942936.2 HWI-D00731:29:HGNNYADXX:1:1107:2473:91006.

-----------------------ccaaactgcgccaacaaatcagtagcttacagaacgc
aaacagtaggaccatagtgggggattct--------------------------------
-atcaacaccatgagcctcagggaccttaaacaggtagagaacaggctggagaaagg---
-----catagctaagataag--ggagatcggaagag------------------------

>gnl|SRA|SRR2638781.63462417.2 HWI-D00731:29:HGNNYADXX:1:2115:18705:44198.

------------------gtcctccaaactgcgccaacaaatcagtagcttacagaacgc
aaacagtaggaccatagtgggggattct--------------------------------
-atcaacaccatgagcctcagggaccttaaacaggtagagaacaggctggagaaagg---
-----catagctaagataag--agatcggaa-----------------------------

>gnl|SRA|SRR2638781.28761846.2 HWI-D00731:29:HGNNYADXX:1:1206:4993:60314.

------------------gtcctccaaactgcgccaacaaatcagtagcttacagaacgc
aaacagtaggaccatagtgggggattct--------------------------------
-atcaacaccatgagcctcagggaccttaaacaggtagagaacaggctggagaaagg---
-----catagctaagataag--agatcggaa-----------------------------

>_R_gnl|SRA|SRR2638781.65346736.1 HWI-D00731:29:HGNNYADXX:1:2116:4231:68586.

-gtgctcttcc-------gatct--aaactgcgccaacaaatcagtagcttacagaacgc
aaacagtaggaccatagtgggggattct--------------------------------
-atcaacaccatgagcctcagggaccttaaacaggtagagaacaggctggagaaagg---
-----catagctaagataag--g-------------------------------------

>_R_gnl|SRA|SRR2638781.8942936.1 HWI-D00731:29:HGNNYADXX:1:1107:2473:91006.

----ctcttcc-------gatctccaaactgcgccaacaaatcagtagcttacagaacgc
aaacagtaggaccatagtgggggattct--------------------------------
-atcaacaccatgagcctcagggaccttaaacaggtagagaacaggctggagaaagg---
-----catagctaagataag--gg------------------------------------

>_R_gnl|SRA|SRR2638781.43464319.1 HWI-D00731:29:HGNNYADXX:1:2101:8560:7630.
---------------------------gactgga--------------------------
gttcagacgtgtgctcttccgatcta------gttgcagaagt----caatgc-------
ccagcactaccagcaggagtcctccaaactgcgccaacaaatcagtagcttacagaacgc
aaacagtaggaccatagtgggggattct--------------------------------
-atcaacaccatg---

>_R_gnl|SRA|SRR2638781.25356923.1 HWI-D00731:29:HGNNYADXX:1:1203:11562:98140.
----------------------------actgga--------------------------
gttcagacgtgtgctcttccgatct-------gttgcagaagt----caatgc-------
ccagcactaccagcaggagtcctccaaactgcgccaacaaatcagtagcttacagaacgc
aaacagtaggaccatagtgggggattct--------------------------------
-atcaacaccatgag---

>_R_gnl|SRA|SRR2638781.17621657.1 HWI-D00731:29:HGNNYADXX:1:1114:9249:12221.
----
gttcagacgtgtgctcttccgatct-------gttgcagaagt----caatgc-------
ccagcactaccagcaggagtcctccaaactgcgccaacaaatcagtagcttacagaacgc
aaacagtaggaccatagtgggggattct--------------------------------
-atcaacaccatgagcctcag---------------------------------------

>_R_gnl|SRA|SRR2638781.12656820.1 HWI-D00731:29:HGNNYADXX:1:1110:14491:60877.

--tcagacgtgtgctcttccgatct---------tgcagaagt----caatgc-------
ccagcactaccagcaggagtcctccaaactgcgccaacaaatcagtagcttacagaacgc
aaacagtaggaccatagtgggggattct--------------------------------
-atcaacaccatgagcctcagggac-----------------------------------

>_R_gnl|SRA|SRR2638781.11040750.1 HWI-D00731:29:HGNNYADXX:1:1109:20879:44378.
---------------agattacaaggtgactgga--------------------------
gttcagacgtgtgctcttccgatct--------------------------gc-------
ccagcactaccagcaggagtcctccaaactgcgccaacaaatcagtagcttacagaacgc
aaacagtaggaccatagtgggggattct--------------------------------
-atcaacaccatgagcc---

>_R_gnl|SRA|SRR2638781.9615475.1 HWI-D00731:29:HGNNYADXX:1:1108:16844:40753.
---------------agattacaaggtgactgga--------------------------
gttcagacgtgtgctcttccgatct--------------------------gc-------
ccagcactaccagcaggagtcctccaaactgcgccaacaaatcagtagcttacagaacgc

aaacagtaggaccatagtgggggattct--------------------------------
-atcaacaccatgagcc---

>_R_gnl|SRA|SRR2638781.82119918.1 HWI-D00731:29:HGNNYADXX:1:2213:4627:21935.

---------------------------------tgactggagt----taagacgtgtgct
cttccgatctcagcaggagtcctccaaactgcgccaacaaatcagtagcttacagaacgc
aaacagtaggaccatagtgggggattct--------------------------------
-atcaacaccatgagcctcagggaccttaaacaggtagag--------------------

>gnl|SRA|SRR2638781.4720308.2 HWI-D00731:29:HGNNYADXX:1:1104:14734:73513.

--cagtagcttacagaacgc
aaacagtaggaccatagtgggggattct--------------------------------
-atcaacaccatgagcctcagggaccttaaacaggtagagaacaggctggagaaagg---
-----catagctaagataag--agatcggaagagcg-----tcgtgtagggaaagagtgt

>gnl|SRA|SRR2638781.13391535.2 HWI-D00731:29:HGNNYADXX:1:1111:18895:13942.

---cgc
aaacagtaggaccatagtgggggattct--------------------------------
-atcaacaccatgagcctcagggaccttaaacaggtagagaacaggctggagaaagg---
-----catagctaagataaggaagatcggaagagcg-----tcgtgtagggaaagagtgt
agatctcggtggt-----
>_R_gnl|SRA|SRR2638781.4720308.1 HWI-D00731:29:HGNNYADXX:1:1104:14734:73513.

---------------------------------tgactggagt----tcagacgtgtgc-
-------------------tcttccgatctc------------agtagcttacagaacgc
aaacagtaggaccatagtgggggattct--------------------------------
-atcaacaccatgagcctcagggaccttaaacaggtagagaacaggctggagaaagg---
-----catagctaagataag--

>_R_gnl|SRA|SRR2638781.13391535.1 HWI-D00731:29:HGNNYADXX:1:1111:18895:13942.
-----------------------------cgaga--------------------------
-------------------------ttacaaggtgactggagt----tcagacgtgtgc-
-------------------tcttccgatct---------------------------cgc
aaacagtaggaccatagtgggggattct--------------------------------
-atcaacaccatgagcctcagggaccttaaacaggtagagaacaggctggagaaagg---
-----catagctaagataag--ga------------------------------------

>gnl|SRA|SRR2638781.20924601.2 HWI-D00731:29:HGNNYADXX:1:1116:13440:40239.

----------------------------tgctatcatatatgct--gcaaact-------
gcagcactaccagcaggagtcctccaaactgcgccaacaaatcagtagcttacagaacgc
aaac---agtaggaccatagcgg-------------------------------------
---------------------------gagatcggaagagcgtcgtgtagggaaaga---
----------------------gtgtagatctcggtggt---------------------

>gnl|SRA|SRR2638781.65803504.2 HWI-D00731:29:HGNNYADXX:1:2116:17862:97663.

------------------------------cagttgcagaagt----caatgc-------
ccagcactaccagcaggagtcctccaaactgcgccaacaaatcagtagcttacagaacgc
aaac---agtaggac---
----------------------------agatcggaagagcgtcgtgtagggaaaga---
----------------------gtgtagatctcggtggtcgccgtatcatta--------

>gnl|SRA|SRR2638781.34602250.2 HWI-D00731:29:HGNNYADXX:1:1210:20914:92050.

---------------cctccaactctggcacagttgcagaagt----caatgc-------
ccagcactaccagcaggagtcctccaaactgcgccaacaaatcagtagcttacagaacgc
aaac---aggaccatagtgggggattct---------------------------------
-at-------------------------agatcggaagagcgtcgtgtagggaaaga---

>gnl|SRA|SRR2638781.67095521.2 HWI-D00731:29:HGNNYADXX:1:2201:3474:99429.

----------------ctccaactctggcacagttgcagaagt----caatgc-------
ccagcactaccagcaggagtcctccaaactgcgccaacaaatcagtagcttacagaacgc
aaac---aggaccatagtggggg-------------------------------------
----------------------------agatcggaagagcgtcgtgtagggaaaga---
----------------------gtgtagat------------------------------

>gnl|SRA|SRR2638781.31293651.2 HWI-D00731:29:HGNNYADXX:1:1208:2095:51676.

----------------ctccaactctggcacagttgcagaagt----caatgc-------
ccagcactaccagcaggagtcctccaaactgcgccaacaaatcagtagcttacagaacgc
aaac---aggaccatagtggggg-------------------------------------
----------------------------agatcggaagagcgtcgtgtagggaaaga---
----------------------gtgtagat------------------------------

>_R_gnl|SRA|SRR2638781.34602250.1 HWI-D00731:29:HGNNYADXX:1:1210:20914:92050.
------------------------------tggagttcagacgtgtgctcttccgatct-
---------------cctccaactctggcacagttgcagaagt----caatgc-------
ccagcactaccagcaggagtcctccaaactgcgccaacaaatcagtagcttacagaacgc
aaac---aggaccatagtgggggattct---------------------------------
-at---

>_R_gnl|SRA|SRR2638781.67095521.1 HWI-D00731:29:HGNNYADXX:1:2201:3474:99429.
----------------------aaggtgactggagttcagacgtgtgctcttccgatct-
----------------ctccaactctggcacagttgcagaagt----caatgc-------
ccagcactaccagcaggagtcctccaaactgcgccaacaaatcagtagcttacagaacgc
aaac---aggaccatagtggggg-------------------------------------

>_R_gnl|SRA|SRR2638781.31293651.1 HWI-D00731:29:HGNNYADXX:1:1208:2095:51676.
----------------------aaggtgactggagttcagacgtgtgctcttccgatct-

----------------ctccaactctggcacagttgcagaagt----caatgc-------
ccagcactaccagcaggagtcctccaaactgcgccaacaaatcagtagcttacagaacgc
aaac---aggaccatagtggggg-------------------------------------

>gnl|SRA|SRR2638781.26653566.2 HWI-D00731:29:HGNNYADXX:1:1204:12311:98964.

--gcaaacagtgacacctccaactctggcacagttgcagaagt----caatgc-------
ccagcactaccagcaggagtcctccaaactgcgccaacaaatcagtagcttacagaacgc
aaac---aggaccatagtgggggattct-------------------------------
-atc----------------------aagatcggaagagcg------------------

>_R_gnl|SRA|SRR2638781.26653566.1 HWI-D00731:29:HGNNYADXX:1:1204:12311:98964.
---tgctcttccgatct-
--gcaaacagtgacacctccaactctggcacagttgcagaagt----caatgc-------
ccagcactaccagcaggagtcctccaaactgcgccaacaaatcagtagcttacagaacgc
aaac---aggaccatagtgggggattct-------------------------------
-atc----------------------a--------------------------------

>gnl|SRA|SRR2638781.74760922.2 HWI-D00731:29:HGNNYADXX:1:2207:11831:96786.
----------------------aagg---------------------------------
---caaacagtgacacctccaactctggcacagttgcagaagt----caatgc-------
ccagcactaccagcaggagtcctccaaactgcgccaacaaatcagtagcttacagaacgc
aaac---agta---
---------------------------gagatcggaagagcgtcgtgtagggaaaga---
----------------------gt-----------------------------------

>_R_gnl|SRA|SRR2638781.74760922.1 HWI-D00731:29:HGNNYADXX:1:2207:11831:96786.
----------------------------actggagttcagacgtgtgctcttccgatcta
aggcaaacagtgacacctccaactctggcacagttgcagaagt----caatgc-------
ccagcactaccagcaggagtcctccaaactgcgccaacaaatcagtagcttacagaacgc
aaac---agtag---

>_R_gnl|SRA|SRR2638781.20924601.1 HWI-D00731:29:HGNNYADXX:1:1116:13440:40239.
---------------------cgagattacaaggtgactgga------------------
gttcagacgtgtgctcttccgatct-----tgctatcatatatgctgcaaact-------
gcagcactaccagcaggagtcctccaaactgcgccaacaaatcagtagcttacagaacgc
aaacagtaggaccatagcggg---------------------------------------

>_R_gnl|SRA|SRR2638781.65803504.1 HWI-D00731:29:HGNNYADXX:1:2116:17862:97663.
---------gaagacggcatacgagattacaaggtgactggag-----------------
gttcagacgtgtgctcttccgatct-----cagttgcagaagt----caatgc-------
ccagcactaccagcaggagtcctccaaactgcgccaacaaatcagtagcttacagaacgc
aaacagtaggac--

>_R_gnl|SRA|SRR2638781.19354158.1 HWI-D00731:29:HGNNYADXX:1:1115:5653:32092.

-aacagtaggaccatagtgggggctcttggagtgaagcagatgatctgctgctgcaacaa
gatggatgccaccactcccaagtactccaaggcccgttatgatgaaatcgtgaaggaagt
ctcatcctacctgaagaaggtcggctacaat-----------------------------

SRX507920
>control .

-------------------tagcttac-agaacgcaaacagtaggaccatagtgggggat
tc--

>_R_gnl|SRA|SRR1213690.37706776.1 HISEQ1:355:D1Y5LACXX:3:2315:5467:40830.

----------------cagtagcttac-agaacgcaaacagtaggaccatagtgggggat
tctatcaacaccatgagcctcagggaccttaaacaggtagagaacaggctggagaaag--

>_R_gnl|SRA|SRR1213690.22352241.1 HISEQ1:355:D1Y5LACXX:3:2110:20986:45250.

----------------cagtagcttac-agaacgcaaacagtaggaccatagtgggggat
tctatcaacaccatgagcctcagggaccttaaacaggtagagaacaggctggagaaag--

>_R_gnl|SRA|SRR1213690.21396680.1 HISEQ1:355:D1Y5LACXX:3:2108:13173:13710.

----------------cagtagcttac-agaacgcaaacagtaggaccatagtgggggat
tctatcaacaccatgagcctcagggaccttaaacaggtagagaacaggctggagaaag--

>_R_gnl|SRA|SRR1213690.17445131.1 HISEQ1:355:D1Y5LACXX:3:1313:9307:43049.

----------------cagtagcttac-agaacgcaaacagtaggaccatagtgggggat
tctatcaacaccatgagcctcagggaccttaaacaggtagagaacaggctggagaaag--

>_R_gnl|SRA|SRR1213690.6413046.1 HISEQ1:355:D1Y5LACXX:3:1201:18701:11027.

----------------cagtagcttac-agaacgcaaacagtaggaccatagtgggggat
tctatcaacaccatgagcctcagggaccttaaacaggtagagaacaggctggagaaag--

>_R_gnl|SRA|SRR1213690.4869819.1 HISEQ1:355:D1Y5LACXX:3:1113:12611:91645.

----------------cagtagcttac-agaacgcaaacagtaggaccatagtgggggat
tctatcaacaccatgagcctcagggaccttaaacaggtagagaacaggctggagaaag--

>_R_gnl|SRA|SRR1213690.3836387.1 HISEQ1:355:D1Y5LACXX:3:1111:5287:65257.

----------------cagtagcttac-agaacgcaaacagtaggaccatagtgggggat
tctatcaacaccatgagcctcagggaccttaaacaggtagagaacaggctggagaaag--

>_R_gnl|SRA|SRR1213690.30319704.1 HISEQ1:355:D1Y5LACXX:3:2213:16400:40632.

----------------cagtagcttac-agaacgcaaacagtaggaccatagtggggnat
tctntcaacaccatgagcctcagggaccttaaacaggtagagaacaggctggagaaag--

>_R_gnl|SRA|SRR1213690.22677588.1 HISEQ1:355:D1Y5LACXX:3:2111:10531:20467.

----------------cagtagcttac-agaacgcaaacagtaggatcatagtgggggat
tctatcaacaccatgagcctcagggaccttaaacaggtagagaacaggctggagaaag--

>_R_gnl|SRA|SRR1213690.3113028.1 HISEQ1:355:D1Y5LACXX:3:1109:11594:96593.

---------------tcagtagcttac-agaacgcaaacagtaggaccatagtgggggat
tctatcaacaccatgagcctcagggaccttaaacaggtagagaacaggctggagaaa---

>_R_gnl|SRA|SRR1213690.788815.1 HISEQ1:355:D1Y5LACXX:3:1103:17424:66190.

---------------tcagtagcttac-agaacgcaaacagtaggaccatagtgggggat
tctatcaacaccatgagcctcagggaccttaaacaggtagagaacaggctggagaaa---

>_R_gnl|SRA|SRR1213690.28901172.1 HISEQ1:355:D1Y5LACXX:3:2210:7236:18344.

---------------tcagtagcttac-agaacgcaaacagtaggaccatagtgggngat
tcnatcaacaccatgagcctcagggaccttaaacaggtagagaacaggctggagaag---

>_R_gnl|SRA|SRR1213690.37450197.1 HISEQ1:355:D1Y5LACXX:3:2314:14317:84463.

---------------tcagtagcttac-agaacgcaaacagtaggaccatagtggtggat
tctatcaacaccatgagcctcagggaccttaaacaggtagagaacaggctggagaaa---

>_R_gnl|SRA|SRR1213690.23294985.1 HISEQ1:355:D1Y5LACXX:3:2112:2615:56481.

-----------------agtagcttac-agaacgcaaacagtaggaccatagtgggggat
tctatcaacaccatgagcctcagggaccttaaacaggtagagagcaggctggagaaagg-

>_R_gnl|SRA|SRR1213690.21667203.1 HISEQ1:355:D1Y5LACXX:3:2108:4841:80595.

-----------------agtagcttac-agaacgcaaacagtaggaccatagtgggggat
tctatcaacaccatgagcctcagggaccttaaacaggtagagaacaggctggagaaagg-

>_R_gnl|SRA|SRR1213690.12940329.1 HISEQ1:355:D1Y5LACXX:3:1301:3251:35547.

-----------------agtagcttac-agaacgcaaacagtaggaccatagtgggggat
tctatcaacaccatgagcctcagggaccttaaacaggtagagaacaggctggagaaagg-

>_R_gnl|SRA|SRR1213690.9663367.1 HISEQ1:355:D1Y5LACXX:3:1210:4570:10013.

-----------------agtagcttac-agaacgcaaacagtaggaccatagtgggggat
tctatcaacaccatgagcctcagggaccttaaacaggtagagaacaggctggagaaagg-

>_R_gnl|SRA|SRR1213690.9609312.1 HISEQ1:355:D1Y5LACXX:3:1209:6316:95931.

-----------------agtagcttac-agaacgcaaacagtaggaccatagtgggggat
tctatcaacaccatgagcctcagggaccttaaacaggtagagaacaggctggagaaagg-

>_R_gnl|SRA|SRR1213690.7212402.1 HISEQ1:355:D1Y5LACXX:3:1203:5287:54999.

-----------------agtagcttac-agaacgcaaacagtaggaccatagtgggggat
tctatcaacaccatgagcctcagggaccttaaacaggtagagaacaggctggagaaagg-

>_R_gnl|SRA|SRR1213690.6362048.1 HISEQ1:355:D1Y5LACXX:3:1116:4829:96279.

-----------------agtagcttac-agaacgcaaacagtaggaccatagtgggggat
tctatcaacaccatgagcctcagggaccttaaacaggtagagaacaggctggagaaagg-

>_R_gnl|SRA|SRR1213690.18093590.1 HISEQ1:355:D1Y5LACXX:3:1314:10524:97051.

-----------------agtagcttac-agaacgcaaacagtaggaccatagtgggggat
tctatcaacaccatgagcctcagggaccttaaacaggtagagaacaggctggagaagggg-
----
>_R_gnl|SRA|SRR1213690.11290573.1 HISEQ1:355:D1Y5LACXX:3:1213:15448:81159.

-----------------agtagcttac-agaacgcaaacagtaggaccatagtgggggat
tctatcaacaccatgagcctcagggaccttaaacaggtagagaacaggctggagaagggg-
----
>_R_gnl|SRA|SRR1213690.27548735.1 HISEQ1:355:D1Y5LACXX:3:2206:17102:73646.

-----------------agtagcttac-agaacgcaaacagtaggaccatagtggggggnt
tctaccaacaccatgagcctcagggaccttaaacaggtagagaacaggctggagaaagg-

>_R_gnl|SRA|SRR1213690.18319475.1 HISEQ1:355:D1Y5LACXX:3:1315:18699:51272.

-----------------agtagcttac-agatcgcaaacagtaggaccatagtgggggat
tctatcaacaccatgagcctcagagaccttaaacaggtatagaacaggctggagaaagg-

>_R_gnl|SRA|SRR1213690.7226712.1 HISEQ1:355:D1Y5LACXX:3:1203:1408:59030.

------------------gtagcttac-agaacgcaaacagtaggaccatagtgggggat
tctatcaacaccatgagcctcagggaccttaaacaggtagagaacaggctggagaaaggc

>_R_gnl|SRA|SRR1213690.30268147.1 HISEQ1:355:D1Y5LACXX:3:2213:3689:29897.

------------------gtagcttac-agaacgcaaacagtaggaccatagtggggggan
tctatnaacaccatgagcctcagggaccttaaacaggtagagaacaggctggagaaaggc

>_R_gnl|SRA|SRR1213690.26736362.1 HISEQ1:355:D1Y5LACXX:3:2204:9684:47935.

------------------gtagcttac-agaacgcaaacagtaggaccatagtggggggan

tctattaacaccatgagcctcagggaccttaaacaggtagagaacaggctggagaaaggc

>_R_gnl|SRA|SRR1213690.33833230.1 HISEQ1:355:D1Y5LACXX:3:2306:14170:2748.

------------------gtggcttac-agaacgcaaacagtaggaccatagtgggggat
tctatcaacaccatgagcctcagggaccttaaacaggtagagaacaggctggagaaaggc

>_R_gnl|SRA|SRR1213690.31150227.1 HISEQ1:355:D1Y5LACXX:3:2215:4880:12438.

------------------gtagcttac-agaacgcaaacagtaggaccatagtgggggan
tctatnaacaccatgagcctcagggaccttaaacaggtagagaacaggctggagaaaagc

>_R_gnl|SRA|SRR1213690.26727752.1 HISEQ1:355:D1Y5LACXX:3:2204:12828:45382.

------------------gtagcttac-agaacgcaaacagtaggaccatagtgggggan
tctatgaaccccatgagcctcagggaccttaaacaggtagagaacaggctggagaaaggc

>_R_gnl|SRA|SRR1213690.2709788.1 HISEQ1:355:D1Y5LACXX:3:1108:14177:98329.

-------------------tagcttac-agaacgcaaacagtaggaccatagtgggggat
tctatcaacaccatgagcctcagggaccttaaacaggtagagaacaggctggagaaaggc
g---------------
>_R_gnl|SRA|SRR1213690.31231416.1 HISEQ1:355:D1Y5LACXX:3:2215:8066:28132.

-------------------tagcttac-agaacgcaaacagtaggaccatagtgggggat
nctatcnacaccatgagcctcagggaccttaaacaggtagagaacaggctggagaaaggc
a---------------
>_R_gnl|SRA|SRR1213690.25914687.1 HISEQ1:355:D1Y5LACXX:3:2202:15858:3255.

-------------------tagcttac-agaacgcaaacagtaggaccatagtgggggat
cctatccacaccatgagcctcagggaccttaaacaggtagagaacaggctggagaaaggc
g---------------
>_R_gnl|SRA|SRR1213690.6244382.1 HISEQ1:355:D1Y5LACXX:3:1116:16252:73287.

---------------------gcttac-agaacgcaaacagtaggaccatagtgggggat
tctatcaacaccatgagcctcagggaccttaaacaggtagagaacaggctggagaaaggc
ata-------------
>_R_gnl|SRA|SRR1213690.14163692.1 HISEQ1:355:D1Y5LACXX:3:1305:12024:3421.

----------------------cttac-agaacgcaaacagtaggaccatagtgggggat
tctatcaacaccatgagcctcagggaccttaaacaggtagagaacaggctggagaaaggc
atag------------
>_R_gnl|SRA|SRR1213690.35213266.1 HISEQ1:355:D1Y5LACXX:3:2309:5403:65792.

-----------------------ttac-agaacgcaaacagtaggaccatagtgggggat
tctatcaacaccatgagcctcagggaccttaaacaggtagagaacaggctggagaaaggc
atagc-----------
>_R_gnl|SRA|SRR1213690.37464826.1 HISEQ1:355:D1Y5LACXX:3:2314:4135:87610.

-------------aatcagtagcttac-agaacgcaaac---aggaccatagtgggggat
tctatcaacaccatgagcctcagggaccttaaacaggtagagaacaggctggagaaag--

>_R_gnl|SRA|SRR1213690.33863506.1 HISEQ1:355:D1Y5LACXX:3:2306:3009:11190.

-------------aatcagtagcttac-agaacgcaaac---aggaccatagtgggggat
tctatcaacaccatgagcctcagggaccttaaacaggtagagaacaggctggagaaag--

>_R_gnl|SRA|SRR1213690.25572960.1 HISEQ1:355:D1Y5LACXX:3:2116:1861:97522.

-------------aatcagtagcttac-agaacgcaaac---aggaccatagtgggggat
tctatcaacaccatgagcctcagggaccttaaacaggtagagaacaggctggagaaag--

>_R_gnl|SRA|SRR1213690.20710563.1 HISEQ1:355:D1Y5LACXX:3:2106:15540:33533.

-------------aatcagtagcttac-agaacgcaaac---aggaccatagtgggggat
tctatcaacaccatgagcctcagggaccttaaacaggtagagaacaggctggagaaag--

>_R_gnl|SRA|SRR1213690.18042493.1 HISEQ1:355:D1Y5LACXX:3:1314:11815:85134.

-------------aatcagtagcttac-agaacgcaaac---aggaccatagtgggggat
tctatcaacaccatgagcctcagggaccttaaacaggtagagaacaggctggagaaag--

>_R_gnl|SRA|SRR1213690.16136710.1 HISEQ1:355:D1Y5LACXX:3:1310:13023:26254.

-------------aatcagtagcttac-agaacgcaaac---aggaccatagtgggggat
tctatcaacaccatgagcctcagggaccttaaacaggtagagaacaggctggagaaag--

>_R_gnl|SRA|SRR1213690.13236653.1 HISEQ1:355:D1Y5LACXX:3:1302:19901:28035.

-------------aatcagtagcttac-agaacgcaaac---aggaccatagtgggggat
tctatcaacaccatgagcctcagggaccttaaacaggtagagaacaggctggagaaag--

>_R_gnl|SRA|SRR1213690.194083.1 HISEQ1:355:D1Y5LACXX:3:1101:16795:69676.

-------------aatcagtagcttac-agaacgcaaac---aggaccatagtgggggat
tctatcaacaccatgagcctcagggaccttaaacaggtagagaacaggctggagaaag--

>_R_gnl|SRA|SRR1213690.29786788.1 HISEQ1:355:D1Y5LACXX:3:2212:18733:23610.

-------------aatcagtagcttac-agaacgcaaac---aggaccatagtggggnat
tctntcaacaccatgagcctcagggaccttaaacaggtagagaacaggctggagaaag--

>_R_gnl|SRA|SRR1213690.26141876.1 HISEQ1:355:D1Y5LACXX:3:2202:18079:72222.

-------------aatcagtagcttac-agaacgcaaac---aggaccatagtggggtat
tctatcaacaccatgagcctcagggaccttaaacaggtagagaacaggctggagaaag--

>_R_gnl|SRA|SRR1213690.29854816.1 HISEQ1:355:D1Y5LACXX:3:2212:20048:38693.

-------------aatcagtagcttac-agaacgcaaac---aggaccatagtggtgnat
tctntcaacaccatgagcctcagggaccttaaacaggtagagaacaggttggagaaag--

>_R_gnl|SRA|SRR1213690.32938745.1 HISEQ1:355:D1Y5LACXX:3:2303:10651:44219.

--------------atcagtagcttac-agaacgcaaac---aggaccatagtgggggat
tctatcaacaccatgagcctcagggaccttaaacaggtagagaacaggctggagaaagg-

>_R_gnl|SRA|SRR1213690.5529356.1 HISEQ1:355:D1Y5LACXX:3:1115:5375:29957.

--------------atcagtagcttac-agaacgcaaac---aggaccatagtgggggat
tctatcaacaccatgagcctcagggaccttaaacaggtagagaacaggctggagaaagg-

>_R_gnl|SRA|SRR1213690.23287318.1 HISEQ1:355:D1Y5LACXX:3:2112:12149:54712.

--------------atcagtagcttac-agaacgcaaac---aggaccatagtgggggat
tctatcaacaccatgagcctcagggaccttaaacaggtagagaacaggctggagaaggg-

>_R_gnl|SRA|SRR1213690.18924721.1 HISEQ1:355:D1Y5LACXX:3:1316:17386:94233.

--------------atcagtagcttac-agaacgcgaac---aggaccatagtgggggat
tctatcaactccatgagcctcagggaccttaaacaggtagagaacaggctggagaaagg-

>_R_gnl|SRA|SRR1213690.33440387.1 HISEQ1:355:D1Y5LACXX:3:2304:9056:90471.

---------------tcagtagcttac-agaacgcaaac---aggaccatagtgggggat
tctatcaacaccatgagcctcagggaccttaaacaggtagagaacaggctggagaaaggc

>_R_gnl|SRA|SRR1213690.24606737.1 HISEQ1:355:D1Y5LACXX:3:2115:1594:23196.

---------------tcagtagcttac-agaacgcaaac---aggaccatagtgggggat
tctatcaacaccatgagcctcagggaccttaaacaggtagagaacaggctggagaaaggc

>_R_gnl|SRA|SRR1213690.22056098.1 HISEQ1:355:D1Y5LACXX:3:2109:14930:75203.

---------------tcagtagcttac-agaacgcaaac---aggaccatagtgggggat
tctatcaacaccatgagcctcagggaccttaaacaggtagagaacaggctggagaaaggc

>_R_gnl|SRA|SRR1213690.19273564.1 HISEQ1:355:D1Y5LACXX:3:2102:10782:9464.

---------------tcagtagcttac-agaacgcaaac---aggaccatagtgggggat
tctatcaacaccatgagcctcagggaccttaaacaggtagagaacaggctggagaaaggc

>_R_gnl|SRA|SRR1213690.5181496.1 HISEQ1:355:D1Y5LACXX:3:1114:10637:57460.

---------------tcagtagcttac-agaacgcaaac---aggaccatagtgggggat
tctatcaacaccatgagcctcagggaccttaaacaggtagagaacaggctggagaaaggc

>_R_gnl|SRA|SRR1213690.1925131.1 HISEQ1:355:D1Y5LACXX:3:1106:18854:96405.

---------------tcagtagcttac-agaacgcaaac---aggaccatagtgggggat
tctatcaacaccatgagcctcagggaccttaaacaggtagagaacaggctggagaaaggc

>_R_gnl|SRA|SRR1213690.20406278.1 HISEQ1:355:D1Y5LACXX:3:2105:21317:49252.

---------------tcagtagcttac-agaacgcaaac---aggacnatagtgggggat
tctatcaacaccatgagcctcagggaccttaaacaggtagagaacaggctggagaaaggc

>_R_gnl|SRA|SRR1213690.17882662.1 HISEQ1:355:D1Y5LACXX:3:1314:9298:47748.

---------------tcagtagcttac-agaacgcaaac---aggaccatagtgggggat
tctatcaacaccatgagcctaagggaccttaaacaggtagagaacaggctggagaaaggc

>_R_gnl|SRA|SRR1213690.17289656.1 HISEQ1:355:D1Y5LACXX:3:1313:14443:6737.

---------------tcagtagcttac-agaacgcaaac---aggaccatagtgggggat
tctatcaacaccatgagcctcagggaccttaaacaggtagagaacaggctggagaaagtc

>_R_gnl|SRA|SRR1213690.33122288.1 HISEQ1:355:D1Y5LACXX:3:2303:7510:97958.

----------------cagtagcttac-agaacgcaaac---aggaccatagtgggggat
tctatcaacaccatgagcctcagggaccttaaacaggtagagaacaggctggagaaaggc
g---------------
>_R_gnl|SRA|SRR1213690.23722347.1 HISEQ1:355:D1Y5LACXX:3:2113:16900:46256.

----------------cagtagcttac-agaacgcaaac---aggaccatagtgggggat
tctatcaacaccatgagcctcagggaccttaaacaggtagagaacaggctggagaaaggc
g---------------
>_R_gnl|SRA|SRR1213690.22052036.1 HISEQ1:355:D1Y5LACXX:3:2109:16660:74019.

----------------cagtagcttac-agaacgcaaac---aggaccatagtgggggat
tctatcaacaccatgagcctcagggaccttaaacaggtagagaacaggctggagaaaggc
g---------------
>_R_gnl|SRA|SRR1213690.17912013.1 HISEQ1:355:D1Y5LACXX:3:1314:8739:54354.

-----------ccgatc--tagcttac-agaacgcaaac---aggaccatagtgggggat
tctatcaacaccatgagcctcagggaccttaaacaggtagagaacaggctggagaaag--

>_R_gnl|SRA|SRR1213690.25419568.1 HISEQ1:355:D1Y5LACXX:3:2116:7923:71143.

-actgcgccaacaaatcagtagcttac-agaacgcaaacagtaggaccatagtgggggat
tctatcaacaccatgagcctcagggaccttaaacaggtagaga-----------------

>_R_gnl|SRA|SRR1213690.26658863.1 HISEQ1:355:D1Y5LACXX:3:2204:8187:25849.

-actgcgccaacaaatcagtagcttac-agaacgcaaacagtnggaccttagtgggggat
tctatcaacaccatgagcctcagggaccttaaacaggtagaga-----------------

>_R_gnl|SRA|SRR1213690.3300680.1 HISEQ1:355:D1Y5LACXX:3:1110:15179:41970.
----
aactgcgccaacaaatcagtagcttac-agaacgcaaacagtaggaccatagtgggggat
tctatcaacaccatgagcctcagggaccttaaacaggtagag------------------

>_R_gnl|SRA|SRR1213690.31426883.1 HISEQ1:355:D1Y5LACXX:3:2215:18838:65850.
----

aactgcgccaacaaatcagtagcttac-agaacgcaaacagnaggacnatagtgggggat
tctatcaacaccatgagcctcagggaccttaaacaggcagag------------------

>_R_gnl|SRA|SRR1213690.21462108.1 HISEQ1:355:D1Y5LACXX:3:2108:14324:29760.

--ctgcgccaacaaatcagtagcttac-agaacgcaaacagtaggaccatagtgggggat
tctatcaacaccatgagcctcagggaccttaaacaggtagagaa----------------

>_R_gnl|SRA|SRR1213690.10694215.1 HISEQ1:355:D1Y5LACXX:3:1212:13194:50075.

---tgcgccaacaaatcagtagcttac-agaacgcaaacagtaggaccatagtgggggat
tctatcaacaccatgagcctcagggaccttaaacaggtagagaac---------------

>_R_gnl|SRA|SRR1213690.4792138.1 HISEQ1:355:D1Y5LACXX:3:1113:8985:75339.

---tgcgccaacaaatcagtagcttac-agaacgcaaacagtaggaccatagtgggggat
tctatcaacaccatgagcctcagggaccttaaacaggtagagaac---------------

>_R_gnl|SRA|SRR1213690.4086222.1 HISEQ1:355:D1Y5LACXX:3:1112:14848:22186.

---tgcgccaacaaatcagtagcttac-agaacgcaaacagtaggaccatagtgggggat
tctatcaacaccatgagcctcagggaccttaaacaggtagagaac---------------

>_R_gnl|SRA|SRR1213690.38379820.1 HISEQ1:355:D1Y5LACXX:3:2316:3711:80819.

------------aaatcagtagcttac-agaacgcaaacagtaggaccatagtgggggat
tctatcaacaccatgagcctcagggaccttaaacaggtagagaacaggctggag------

>_R_gnl|SRA|SRR1213690.22798814.1 HISEQ1:355:D1Y5LACXX:3:2111:4883:47485.

------------aaatcagtagcttac-agaacgcaaacagtaggaccatagtgggggat
tctatcaacaccatgagcctcagggaccttaaacaggtagagaacaggctggag------

>_R_gnl|SRA|SRR1213690.18354643.1 HISEQ1:355:D1Y5LACXX:3:1315:16424:59728.

------------aaatcagtagcttac-agaacgcaaacagtaggaccatagtgggggat
tctatcaacaccatgagcctcagggaccttaaacaggtagagaacaggctggag------

>_R_gnl|SRA|SRR1213690.15604243.1 HISEQ1:355:D1Y5LACXX:3:1308:7967:91419.

------------aaatcagtagcttac-agaacgcaaacagtaggaccatagtgggggat
tctatcaacaccatgagcctcagggaccttaaacaggtagagaacaggctggag------

>_R_gnl|SRA|SRR1213690.12042568.1 HISEQ1:355:D1Y5LACXX:3:1215:14788:40536.

------------aaatcagtagcttac-agaacgcaaacagtaggaccatagtgggggat
tctatcaacaccatgagcctcagggaccttaaacaggtagagaacaggctggag------

>_R_gnl|SRA|SRR1213690.29078728.1 HISEQ1:355:D1Y5LACXX:3:2210:19145:60102.

------------aaatcagtagcttac-agaacgcaaacagtaggaccatagtngggggan

tctatcaacaccatgagcctcagggaccttaaacaggtagagaacaggctagag------

>_R_gnl|SRA|SRR1213690.29312165.1 HISEQ1:355:D1Y5LACXX:3:2211:16638:15281.

------------aaatcagtagcttac-agaacgcaaacagtaggaccatagtnggggan
tctatcaacaccatgagcctcagggaccttaaacaggtacagaacaggctggag------

>_R_gnl|SRA|SRR1213690.36969857.1 HISEQ1:355:D1Y5LACXX:3:2313:9331:78725.

---------aacaaatcagtagcttac-agaacgcaaacagtaggaccatagtgggggat
tctatcaacaccatgagcctcagggaccttaaacaggtagagaacaggctg---------

>_R_gnl|SRA|SRR1213690.35018971.1 HISEQ1:355:D1Y5LACXX:3:2309:16999:17441.

---------aacaaatcagtagcttac-agaacgcaaacagtaggaccatagtgggggat
tctatcaacaccatgagcctcagggaccttaaacaggtagagaacaggctg---------

>_R_gnl|SRA|SRR1213690.27788.1 HISEQ1:355:D1Y5LACXX:3:1101:15555:11958.

---------aacaaatcag-agcttac-agaacgcaaacagtaggaccatagtgggggat
tctatcaacaccatgagcctcagggaccttaaacaggtagagaacaggctgg--------

>_R_gnl|SRA|SRR1213690.23142825.1 HISEQ1:355:D1Y5LACXX:3:2112:13323:23799.

----------acaaatcagtagcttac-agaacgcaaacagtaggaccatagtgggggat
tctatcaacaccatgagcctcagggaccttaaacaggtagagaacaggctgg--------

>_R_gnl|SRA|SRR1213690.15671927.1 HISEQ1:355:D1Y5LACXX:3:1309:19616:9567.

----------acaaatcagtagcttac-agaacgcaaacagtaggaccatagtgggggat
tctatcaacaccatgagcctcagggaccttaaacaggtagagaacaggctgg--------

>_R_gnl|SRA|SRR1213690.9222998.1 HISEQ1:355:D1Y5LACXX:3:1208:19025:100535.

----------acaaatcagtagcttac-agaacgcaaacagtaggaccatagtgggggat
tctatcaacaccatgagcctcagggaccttaaacaggtagagaacaggctgg--------

>_R_gnl|SRA|SRR1213690.11213157.1 HISEQ1:355:D1Y5LACXX:3:1213:10713:64579.

----------acaaatcagtagcttac-agaacgcaaacagtaggaccatagtgggggat
tctatcaacaccatgagcctcagggaccttaaacaggtagagaacatgctgg--------

>_R_gnl|SRA|SRR1213690.22974285.1 HISEQ1:355:D1Y5LACXX:3:2111:17951:86226.

-----------caaatcagtagcttac-agaacgcaaacagtaggaccatagtgggggat
tctatcaacaccatgagcctcagggaccttaaacaggtagagaacaggctgga-------

>_R_gnl|SRA|SRR1213690.18598501.1 HISEQ1:355:D1Y5LACXX:3:1316:2958:18203.

-----------caaatcagtagcttac-agaacgcaaacagtaggaccatagtgggggat
tctatcaacaccatgagcctcagggaccttaaacaggtagagaacaggctgga-------

>_R_gnl|SRA|SRR1213690.18200079.1 HISEQ1:355:D1Y5LACXX:3:1315:11207:23633.

-----------caaatcagtagcttac-agaacgcaaacagtaggaccatagtgggggat
tctatcaacaccatgagcctcagggaccttaaacaggtagagaacaggctgga-------

>_R_gnl|SRA|SRR1213690.15885112.1 HISEQ1:355:D1Y5LACXX:3:1309:20499:62776.

-----------caaatcagtagcttac-agaacgcaaacagtaggaccatagtgggggat
tctatcaacaccatgagcctcagggaccttaaacaggtagagaacaggctgga-------

>_R_gnl|SRA|SRR1213690.34242426.1 HISEQ1:355:D1Y5LACXX:3:2307:18702:14415.

-----cgccaacaaatcagtagcttac-agaacgcaaacagtaggaccatagtgggggat
tctatcaacaccatgagcctcagggaccttaaacaggtagagaacag-------------

>_R_gnl|SRA|SRR1213690.24828434.1 HISEQ1:355:D1Y5LACXX:3:2115:15545:63835.

-----cgccaacaaatcagtagcttac-agaacgcaaacagtaggaccatagtgggggat
tctatcaacaccatgagcctcagggaccttaaacaggtagagaacag-------------

>_R_gnl|SRA|SRR1213690.23138577.1 HISEQ1:355:D1Y5LACXX:3:2112:1283:23093.

-----cgccaacaaatcagtagcttac-agaacgcaaacagtaggaccatagtgggggat
tctatcaacaccatgagcctcagggaccttaaacaggtagagaacag-------------

>_R_gnl|SRA|SRR1213690.16689769.1 HISEQ1:355:D1Y5LACXX:3:1311:4316:61869.

-----cgccaacaaatcagtagcttac-agaacgcaaacagtaggaccatagtgggggat
tctatcaacaccatgagcctcagggaccttaaacaggtagagaacag-------------

>_R_gnl|SRA|SRR1213690.15358550.1 HISEQ1:355:D1Y5LACXX:3:1308:12012:28647.

-----cgccaacaaatcagtagcttac-agaacgcaaacagtaggaccatagtgggggat
tctatcaacaccatgagcctcagggaccttaaacaggtagagaacag-------------

>_R_gnl|SRA|SRR1213690.9016781.1 HISEQ1:355:D1Y5LACXX:3:1208:19489:49320.

-----cgccaacaaatcagtagcttac-agaacgcaaacagtaggaccatagtgggggat
tctatcaacaccatgagcctcagggaccttaaacaggtagagaacag-------------

>_R_gnl|SRA|SRR1213690.6230665.1 HISEQ1:355:D1Y5LACXX:3:1116:17743:70554.

-----cgccaacaaatcagtagcttac-agaacgcaaacagtaggaccatagtgggggat
tctatcaacaccatgagcctcagggaccttaaacaggtagagaacag-------------

>_R_gnl|SRA|SRR1213690.25606247.1 HISEQ1:355:D1Y5LACXX:3:2201:11170:6787.

-----cgccaacaaatcagtagcttac-agaacgcaaacagtaggatcatagtgggggat
tctatcaacaccatgagcctcagggaccttaaacaggtagagaacag-------------

>_R_gnl|SRA|SRR1213690.18942619.1 HISEQ1:355:D1Y5LACXX:3:1316:8203:98640.

------gccaacaaatcagtagcttacaagaacgcaaacagtaggaccatagtgggggat
tctatcaacaccatgagcctcagggaccttaaacaggtagagaacag-------------

>_R_gnl|SRA|SRR1213690.24063997.1 HISEQ1:355:D1Y5LACXX:3:2114:1586:17069.

------gccaacaaatcagtagcttac-agaacgcaaacagtaggaccatagtgggggat
tctatcaacaccatgagcctcagggaccttaaacaggtagagaacagg------------

>_R_gnl|SRA|SRR1213690.15335103.1 HISEQ1:355:D1Y5LACXX:3:1308:5732:22636.

------gccaacaaatcagtagcttac-agaacgcaaacagtaggaccatagtgggggat
tctatcaacaccatgagcctcagggaccttaaacaggtagagaacagg------------

>_R_gnl|SRA|SRR1213690.27825754.1 HISEQ1:355:D1Y5LACXX:3:2207:17291:47655.

------gccaacaaatcagtagcttac-agaacgcaaacagtaggacnatagtnggggat
tctatcaacaccatgagcctcagggaccttaaacaggtagagaacagg------------

>_R_gnl|SRA|SRR1213690.15490200.1 HISEQ1:355:D1Y5LACXX:3:1308:14561:62494.

------gccaacaaatcagtagcttac-agaacgcaaacagtaggaccatagtgggggat
tctatcaacaccatgagtctcagggaccttaaacaggtagagaacagg------------

>_R_gnl|SRA|SRR1213690.25364717.1 HISEQ1:355:D1Y5LACXX:3:2116:19855:61345.

-------ccaacaaatcagtagcttac-agaacgcaaacagtaggaccatagtgggggat
tctatcaacaccatgagcctcagggaccttaaacaggtagagaactggc-----------

>_R_gnl|SRA|SRR1213690.14748311.1 HISEQ1:355:D1Y5LACXX:3:1306:9087:65509.

-------ccaacaaatcagtagcttac-agaacgcaaacagtaggaccatagtgggggat
tctatcaacaccatgagcctcagggaccttaaacaggtagagaacaggc-----------

>_R_gnl|SRA|SRR1213690.7778525.1 HISEQ1:355:D1Y5LACXX:3:1205:18759:18373.

-------ccaacaaatcagtagcttac-agaacgcaaacagtaggaccatagtgggggat
tctatcaacaccatgagcctcagggaccttaaacaggtagagaacaggc-----------

>_R_gnl|SRA|SRR1213690.2022612.1 HISEQ1:355:D1Y5LACXX:3:1107:9081:23329.

-------ccaacaaatcagtagcttac-agaacgcaaacagtaggaccatagtgggggat
tctatcaacaccatgagcctcagggacctttaacaggtagagaacaggc-----------

>_R_gnl|SRA|SRR1213690.20272072.1 HISEQ1:355:D1Y5LACXX:3:2105:15191:11905.

--------caacaaatcagtagcttac-agaacgcaaacagtaggaccatagtgggggat
tctatcaacaccatgagcctcagggaccttaaacaggtagagaacaggcc----------

>_R_gnl|SRA|SRR1213690.31573141.1 HISEQ1:355:D1Y5LACXX:3:2215:19794:93640.

--------caacaaatcagtagcttac-agaacgcaaacagtaggaccanagtggnggat
tctatcaacaccatgagcctcagggaccttaaacaggtagagaacaggcc----------

>_R_gnl|SRA|SRR1213690.27265197.1 HISEQ1:355:D1Y5LACXX:3:2205:2825:96250.

--------catcaaatcagtagcttac-agaacgcaaacagtaggaccanagtggcggat
tctatcaacaccatgagcctcagggaccttaaacaggtagagaacaggcc----------

>_R_gnl|SRA|SRR1213690.37993072.1 HISEQ1:355:D1Y5LACXX:3:2316:13092:2983.
----
accggcgccgacaaatcagtagcttac-agaacgcaaacag---gaccatagtgggggat
tctatcaacaccatgagcctcagggaccttaaacaggtagagaac---------------

>_R_gnl|SRA|SRR1213690.18127261.1 HISEQ1:355:D1Y5LACXX:3:1315:11661:6455.
----
aactgcgccaacaaatcagtagcttac-agaacgcaaacag---gaccatagtgggggat
tctatcaacaccatgagcctcagggaccttaaacaggtagagaac---------------

>_R_gnl|SRA|SRR1213690.34757546.1 HISEQ1:355:D1Y5LACXX:3:2308:12658:50016.

----gcgccaacaaatcagtagcttac-agaacgcaaacag---gaccatagtgggggat
tctatcaacaccatgagcctcagggaccttaaacaggtagagaacaggc-----------

>_R_gnl|SRA|SRR1213690.21406412.1 HISEQ1:355:D1Y5LACXX:3:2108:9047:16008.

----gcgccaacaaatcagtagcttac-agaacgcaaacag---gaccatagtgggggat
tctatcaacaccatgagcctcagggaccttaaacaggtagagaacaggc-----------

>_R_gnl|SRA|SRR1213690.18859420.1 HISEQ1:355:D1Y5LACXX:3:1316:8854:79174.

----gcgccaacaaatcagtagcttac-agaacgcaaacag---gaccatagtgggggat
tctatcaacaccatgagcctcagggaccttaaacaggtagagaacaggc-----------

>_R_gnl|SRA|SRR1213690.30267353.1 HISEQ1:355:D1Y5LACXX:3:2213:10795:29582.

----gcgccaacaaatcagtagcttac-agaacgcaaacag---gaccntagtgngggat
tctatcaacaccatgagcctcagggaccttaaacaggtagagaacaggc-----------

>_R_gnl|SRA|SRR1213690.20312705.1 HISEQ1:355:D1Y5LACXX:3:2105:11654:23280.

----gcgccaacaaatcagtagcttac-agaacgcaaacag---gaccatagtgggggat
tctatcaacaccatgagccccagggaccttaaacaggtagagaacaggc-----------

>_R_gnl|SRA|SRR1213690.7001700.1 HISEQ1:355:D1Y5LACXX:3:1202:17241:91575.

----gcgccaacaaatcagtagcttac-agaacgcaaacag---gaccatagtgggggat
tctatcaacaccatgagcctcagggaccttaaacaggtagagaacaggg-----------

>_R_gnl|SRA|SRR1213690.14460928.1 HISEQ1:355:D1Y5LACXX:3:1305:1510:86137.

---tgcgccaacaaatcagtagcttac-agaacgcaaacag---gaccatagtgggggat
tctatcaacaccatgagcctcagggaccttaaacaggtagagaacagg------------

>_R_gnl|SRA|SRR1213690.1699154.1 HISEQ1:355:D1Y5LACXX:3:1106:4366:35546.

---tgcgccaacaaatcagtagcttac-agaacgcaaacag---gaccatagtgggggat
tctatcaacaccatgagcctcagggaccttaaacaggtagagaacagg------------

>_R_gnl|SRA|SRR1213690.25033269.1 HISEQ1:355:D1Y5LACXX:3:2116:4723:2588.

--ctgcgccaacaaatcagtagcttac-agaacgcaaacag---gaccatagtgggggat
tctatcaacaccatgagcctcagggaccttaaacaggtagagaacag-------------

>_R_gnl|SRA|SRR1213690.3544332.1 HISEQ1:355:D1Y5LACXX:3:1110:6570:97830.

--ctgcgccaacaaatcagtagcttac-agaacgcaaacag---gaccatagtggggaat
tctatcaacaccatgagcctcagggaccttaaacaggtagagaacag-------------

>_R_gnl|SRA|SRR1213690.35265633.1 HISEQ1:355:D1Y5LACXX:3:2309:13794:78608.

-----cgccaacaaatcagtagcttac-agaacgcaaacag---gaccatagtgggggat
tctatcaacaccatgagcctcagggaccttaaacaggtagcgaacaggcc----------

>_R_gnl|SRA|SRR1213690.34011139.1 HISEQ1:355:D1Y5LACXX:3:2306:1301:51388.

---------aacaaatcagtagcttac-agaacgcaaac---aggaccatagtgggggat
tctatcaacaccatgagcctcagggaccttaaacaggtagagaacaggctggag------

>_R_gnl|SRA|SRR1213690.20564400.1 HISEQ1:355:D1Y5LACXX:3:2105:6672:92813.

---------aacaaatcagtagcttac-agaacgcaaac---aggaccatagtgggggat
tctatcaacaccatgagcctcagggaccttaaacaggtagagaacaggctggag------

>_R_gnl|SRA|SRR1213690.1468277.1 HISEQ1:355:D1Y5LACXX:3:1105:9174:69878.

---------aacaaatcagtagcttac-agaacgcaaac---aggaccatagtgggggat
tctatcaacaccatgagcctcagggaccttaaacaggtagagaacaggctggag------

>_R_gnl|SRA|SRR1213690.17169010.1 HISEQ1:355:D1Y5LACXX:3:1312:12072:76922.

---------aacaaatcagtagcttac-agaacgcaaac---aggaccatagtgggggat
tctatcaacaccatgagcctcagggaccttaaacaggtagagaacaggccggag------

>_R_gnl|SRA|SRR1213690.25271022.1 HISEQ1:355:D1Y5LACXX:3:2116:16896:44877.

---------aacaaatcactagcttac-agaacgcaaac---aggaccatagtgggggat
tctatcaacaccatgagcctcagggaccttaaacaggtagagaacaggccggag------

>_R_gnl|SRA|SRR1213690.35717728.1 HISEQ1:355:D1Y5LACXX:3:2310:21003:88773.

---------aacaaatcagtagcttac-agaacgcaaac---acgaccatagtgggggat

tctatcaacgccatgagcctcagggaccttaaacaggtagagaacagcctggag------

>_R_gnl|SRA|SRR1213690.35759548.1 HISEQ1:355:D1Y5LACXX:3:2310:12429:98886.
--gtcctcca
aactgcgccaacaaatcagtagcttac-agaacgcaaacagtaggaccatagtgggggat
tctatcaacaccatgagcctcagggaccttaaac--------------------------

>_R_gnl|SRA|SRR1213690.3536485.1 HISEQ1:355:D1Y5LACXX:3:1110:4237:96236.
--gtcctcca
aactgcgccaacaaatcagtagcttac-agaacgcaaacagtaggaccatagtgggggat
tctatcaacaccatgagcctcagggaccttaaac--------------------------

>_R_gnl|SRA|SRR1213690.32612937.1 HISEQ1:355:D1Y5LACXX:3:2302:14103:45227.
---agtcttcca
aactgcgccaacaaatcagtagcttac-agaacgcaaacagtaggaccatagtgggggat
tctatcaacaccatgagcctcagggaccttaaa---------------------------

>_R_gnl|SRA|SRR1213690.10685232.1 HISEQ1:355:D1Y5LACXX:3:1212:14281:48159.
--gagtcctcca
aactgcgccaacaaatcagtagcttac-agaacgcaaacagtaggaccatagtgggggat
tctatcaacaccatgagcctcagggaccttaa----------------------------

>_R_gnl|SRA|SRR1213690.15461398.1 HISEQ1:355:D1Y5LACXX:3:1308:9329:55230.
--gcaggagtcctcca
aactgcgccaacaaatcagtagcttac-agaacgcaaacagtaggaccatagtgggggat
tctatcaacaccatgagcctcagggacc--------------------------------

>_R_gnl|SRA|SRR1213690.11710500.1 HISEQ1:355:D1Y5LACXX:3:1214:6242:70841.
--gcaggagtcctcca
aactgcgccaacaaatcagtagcttac-agaacgcaaacagtaggaccatagtgggggat
tctatcaacaccatgagcctcagggacc--------------------------------

>_R_gnl|SRA|SRR1213690.5284293.1 HISEQ1:355:D1Y5LACXX:3:1114:12851:78461.
--gcaggagtcctcca
aactgcgccaacaaatcagtagcttac-agaacgcaaacagtaggaccatagtgggggat
tctatcaacaccatgagcctcagggacc--------------------------------

>_R_gnl|SRA|SRR1213690.5260359.1 HISEQ1:355:D1Y5LACXX:3:1114:5955:73735.
--gcaggagtcctcca
aactgcgccaacaaatcagtagcttac-agaacgcaaacagtaggaccatagtgggggat
tctatcaacaccatgagcctcagggacc--------------------------------

>_R_gnl|SRA|SRR1213690.23659570.1 HISEQ1:355:D1Y5LACXX:3:2113:11778:33722.
--gcaggagacctcca
aacaaagccaacaaagcagtagcttac-agaacgcaaacagtagggccatagtgggggat
tgtatcaacactatgagcctcagggacc--------------------------------

>_R_gnl|SRA|SRR1213690.10869079.1 HISEQ1:355:D1Y5LACXX:3:1212:6628:88795.
---caggagtcctcca
aactgcgccaacaaatcagtagcttac-agaacgcaaacagtaggaccatagtgggggat
tctatcaacaccatgagcctcagggacct-------------------------------

>_R_gnl|SRA|SRR1213690.973316.1 HISEQ1:355:D1Y5LACXX:3:1104:7150:24148.
---caggagtcctcca
aactgcgccaacaaatcagtagcttac-agaacgcaaacagtaggaccatagtgggggat
tctatcaacaccatgagcctcagggaccc-------------------------------

>_R_gnl|SRA|SRR1213690.32876119.1 HISEQ1:355:D1Y5LACXX:3:2303:8088:25622.
---cca
aactgcgccaacaaatcagtagcttac-agaacgcaaacagtaggaccatagtgggggat
tctatcaacaccatgagcctcagggaccttaaacaggta---------------------

>_R_gnl|SRA|SRR1213690.23638155.1 HISEQ1:355:D1Y5LACXX:3:2113:2574:29492.
---ctcca
aactgcgccaacaaatcagtagcttac-agaacgcaaacagtaggaccatagtgggggat
tctatcaacaccatgagcctcagggaccttaaacagg----------------------

>_R_gnl|SRA|SRR1213690.11920204.1 HISEQ1:355:D1Y5LACXX:3:1215:5251:15630.
---ctcca
aactgcgccaacaaatcagtagcttac-agaacgcaaacagtaggaccatagtgggggat
tctatcaacaccatgagcctcagggaccttaaacagg----------------------

>_R_gnl|SRA|SRR1213690.21419113.1 HISEQ1:355:D1Y5LACXX:3:2108:2464:19356.
--tcca
aactgcgccaacaaatcagtagcttac-agaacgcaaacagtaggaccatagtgggggat
tctatcaacaccatgagcctcagggaccttaaacaggc---------------------

>_R_gnl|SRA|SRR1213690.633513.1 HISEQ1:355:D1Y5LACXX:3:1103:20438:17582.
--tcca
aactgcgccaacaaatcagtagcttac-agaacgcaaacagtaggaccatagtgggggat
tctatcaacaccatgagcctcagggaccttaaacaggc---------------------

>_R_gnl|SRA|SRR1213690.9476619.1 HISEQ1:355:D1Y5LACXX:3:1209:20399:63925.
--tcca
aactgcgccaacaaatcagtagcttac-agaacgcaaacagtaggaccatagtgggggat
tctatcaacaccatgagcctcagggaccttaaacaggt---------------------

>_R_gnl|SRA|SRR1213690.31852671.1 HISEQ1:355:D1Y5LACXX:3:2216:21015:46528.
--tcca
aactgggccaacaaatcagtagcttac-agaacgcaancagtangaccatagtgggggat
tctatcaacaccatgagcctcagggaccttaaacaggc---------------------

>_R_gnl|SRA|SRR1213690.15816150.1 HISEQ1:355:D1Y5LACXX:3:1309:6735:45971.
---ggagtcctcca
aactgcgccaacaaatcagtagcttac-agaacgcaaacag---gaccatagtgggggat
tctatcaacaccatgagcctcagggaccttaaac--------------------------

>_R_gnl|SRA|SRR1213690.5568587.1 HISEQ1:355:D1Y5LACXX:3:1115:11409:37726.
---tcctcca
aactgcgccaacaaatcagtagcttac-agaacgcaaacag---gaccatagtgggggat
tctatcaacaccatgagcctcagggaccttaaacaggt---------------------

>_R_gnl|SRA|SRR1213690.29099817.1 HISEQ1:355:D1Y5LACXX:3:2210:8086:65218.
---gcctcca
aactgcgccaacaaagcagtagcttac-agaacgnaaacan---gaccatagtgggggat
tctatcaaccccatgagcctcagggaccttaaacaggt----------------------

>_R_gnl|SRA|SRR1213690.26153071.1 HISEQ1:355:D1Y5LACXX:3:2202:5637:75682.
--cctcca
aactgcgccaacaaatcagtagcttac-agaacgctaacag---caccatagtgggggat
tctatcaacaccatgagcctcagggaccttaaacaggta---------------------

>_R_gnl|SRA|SRR1213690.25597636.1 HISEQ1:355:D1Y5LACXX:3:2201:16310:4119.
--ca
aactgcgccaacaaatcagtagcttac-agaacgcaaactg---gaccctagtgggggat
tctatcaacaccatgagcctcagggaccttaaacaggtagaga-----------------

>_R_gnl|SRA|SRR1213690.18793454.1 HISEQ1:355:D1Y5LACXX:3:1316:5369:63558.

----------------------------agaacgcaaacagtaggaccatagtgggggat
tctatcaacaccatgagcctcagggaccttaaacaggtagagaacaggctggagaaaggc
atagctaag-------
>_R_gnl|SRA|SRR1213690.15822888.1 HISEQ1:355:D1Y5LACXX:3:1309:20127:47297.

----------------------------agaacgcaaacagtaggaccatagtgggggat
tctatcaacaccatgagcctcagggaccttaaacaggtagagaacaggctggagaaaggc
atagctaag-------
>_R_gnl|SRA|SRR1213690.35140191.1 HISEQ1:355:D1Y5LACXX:3:2309:13054:47705.

----------------------------agaacgcaaacagtcggaccatagtgggggat
tctatcaacaccatgagcctcagggaccttaaacaggtagagaacaggctggagaaaggc
atagctaag-------
>_R_gnl|SRA|SRR1213690.34574374.1 HISEQ1:355:D1Y5LACXX:3:2308:13377:3021.

----------------------------atctcgcaaacagtaggaccatagtgggggat
tctatcaacaccatgagcctcagggaccttaaacaggtagagaacaggctggagaaaggc
atagctaag-------
>_R_gnl|SRA|SRR1213690.23581623.1 HISEQ1:355:D1Y5LACXX:3:2113:13398:17521.

-------------------------------acgcaaacagtaggaccatagtgggggat
tctatcaacaccatgagcctcagggaccttaaacaggtagagaacaggctggagaaaggc
atagctaagata----
>_R_gnl|SRA|SRR1213690.37124458.1 HISEQ1:355:D1Y5LACXX:3:2314:8884:13968.

---------------------------------gcaaacagtaggaccatagtgggggat
tctatcaacaccatgagcctcagggaccttaaacaggtagagaacaggctggagaaaggc
atagctaagataag--
>_R_gnl|SRA|SRR1213690.32911161.1 HISEQ1:355:D1Y5LACXX:3:2303:15231:35873.

---------------------------------gcaaacagtaggaccatagtgggggat
tctatcaacaccatgagcctcagggaccttaaacaggtagagaacaggctggagaaaggc
atagctaagataag--
>_R_gnl|SRA|SRR1213690.30594413.1 HISEQ1:355:D1Y5LACXX:3:2213:19089:97919.

---------------------------------gcaaacagtaggaccatagtgggggat
tctatcaacaccangagccncagggaccttaaacaggtagagaacaggctggagaaaggc
atagctaagataag--
>_R_gnl|SRA|SRR1213690.27729240.1 HISEQ1:355:D1Y5LACXX:3:2207:3158:22630.

---------------------------------gcaaacagtaggaccatagtgggggat
tctatcaacaccangagccncagggaccttaaacaggtagagaacaggctggagaaaggc
atagctaagataag--
>_R_gnl|SRA|SRR1213690.20676857.1 HISEQ1:355:D1Y5LACXX:3:2106:10766:24504.

---------------------------------gcaaacagtaggaccatagtgggggat
tctatcaacaccatgagcctcagggaccttaaacaggtagagaacaggctggagaaaggc
atagctaagataag--
>_R_gnl|SRA|SRR1213690.18895949.1 HISEQ1:355:D1Y5LACXX:3:1316:13675:87483.

---------------------------------gcaaacagtaggaccatagtgggggat
tctatcaacaccatgagcctcagggaccttaaacaggtagagaacaggctggagaaaggc
atagctaagataag--
>_R_gnl|SRA|SRR1213690.16668709.1 HISEQ1:355:D1Y5LACXX:3:1311:14832:56502.

---------------------------------gcaaacagtaggaccatagtgggggat
tctatcaacaccatgagcctcagggaccttaaacaggtagagaacaggctggagaaaggc
atagctaagataag--
>_R_gnl|SRA|SRR1213690.12152281.1 HISEQ1:355:D1Y5LACXX:3:1215:6173:63434.

---------------------------------gcaaacagtaggaccatagtgggggat
tctatcaacaccatgagcctcagggaccttaaacaggtagagaacaggctggagaaaggc
atagctaagataag--
>_R_gnl|SRA|SRR1213690.9100006.1 HISEQ1:355:D1Y5LACXX:3:1208:10855:70349.

---------------------------------gcaaacagtaggaccatagtgggggat
tctatcaacaccatgagcctcagggaccttaaacaggtagagaacaggctggagaaaggc
atagctaagataag--
>_R_gnl|SRA|SRR1213690.32204313.1 HISEQ1:355:D1Y5LACXX:3:2301:20744:17675.

---------------------------------gcaaacagtaggaccatagtgggggat
tctatcaacaccatgagcctcagggaccttaaacaggtagagaacaggctggagaaatgc
atagctaagataag--
>_R_gnl|SRA|SRR1213690.37990421.1 HISEQ1:355:D1Y5LACXX:3:2316:10726:2297.

----------------------------------caaacagtaggaccatagtgggggat
tctatcaacaccatgagcctcagggaccttaaacaggtagagaacaggctggagaaaggc
atagctaagataagg-
>_R_gnl|SRA|SRR1213690.37454219.1 HISEQ1:355:D1Y5LACXX:3:2314:3637:85497.

----------------------------------caaacagtaggaccatagtgggggat
tctatcaacaccatgagcctcagggaccttaaacaggtagagaacaggctggagaaaggc
atagctaagataagg-
>_R_gnl|SRA|SRR1213690.32915264.1 HISEQ1:355:D1Y5LACXX:3:2303:11301:37089.

---------------------------------caaacagtaggaccatagtgggggat
tctatcaacaccatgagcctcagggaccttaaacaggtagagaacaggctggagaaaggc
atagctaagataagg-
>_R_gnl|SRA|SRR1213690.30012449.1 HISEQ1:355:D1Y5LACXX:3:2212:16335:73186.

---------------------------------caaacagtaggaccatagtgggggat
tctatcaacaccatnagcctnagggaccttaaacaggtagagaacaggctggagaaaggc
atagctaagataagg-
>_R_gnl|SRA|SRR1213690.28900351.1 HISEQ1:355:D1Y5LACXX:3:2210:11924:18020.

---------------------------------caaacagtaggaccatagtgggggat
tctatcaacaccatnagcctnagggaccttaaacaggtagagaacaggctggagaaaggc
atagctaagataagg-
>_R_gnl|SRA|SRR1213690.28084225.1 HISEQ1:355:D1Y5LACXX:3:2208:9914:15456.

---------------------------------caaacagtaggaccatagtgggggat
tctatcaacaccatnagcctcagggaccttaaacaggtagagaacaggctggagaaaggc
atagctaagataagg-
>_R_gnl|SRA|SRR1213690.27967766.1 HISEQ1:355:D1Y5LACXX:3:2207:6938:84251.

---------------------------------caaacagtaggaccatagtgggggat
tctatcaacaccatnagcctnagggaccttaaacaggtagagaacaggctggagaaaggc
atagctaagataagg-
>_R_gnl|SRA|SRR1213690.22712305.1 HISEQ1:355:D1Y5LACXX:3:2111:5000:28017.

---------------------------------caaacagtaggaccatagtgggggat
tctatcaacaccatgagcctcagggaccttaaacaggtagagaacaggctggagaaaggc
atagctaagataagg-
>_R_gnl|SRA|SRR1213690.20561217.1 HISEQ1:355:D1Y5LACXX:3:2105:16107:91842.

---------------------------------caaacagtaggaccatagtgggggat
tctatcaacaccatgagcctcagggaccttaaacaggtagagaacaggctggagaaaggc
atagctaagataagg-
>_R_gnl|SRA|SRR1213690.19527445.1 HISEQ1:355:D1Y5LACXX:3:2102:6842:89474.

---------------------------------caaacagtaggaccatagtgggggat
tctatcaacaccatgagcctcagggaccttaaacaggtagagaacaggctggagaaaggc
atagctaagataagg-
>_R_gnl|SRA|SRR1213690.19038207.1 HISEQ1:355:D1Y5LACXX:3:2101:7058:31392.

---------------------------------caaacagtaggaccatagtgggggat
tctatcaacaccatgagcctcagggaccttaaacaggtagagaacaggctggagaaaggc
atagctaagataagg-
>_R_gnl|SRA|SRR1213690.14028467.1 HISEQ1:355:D1Y5LACXX:3:1304:5680:63867.

---------------------------------caaacagtaggaccatagtgggggat
tctatcaacaccatgagcctcagggaccttaaacaggtagagaacaggctggagaaaggc
atagctaagataagg-
>_R_gnl|SRA|SRR1213690.13816447.1 HISEQ1:355:D1Y5LACXX:3:1304:10233:2981.

---------------------------------caaacagtaggaccatagtgggggat

tctatcaacaccatgagcctcagggaccttaaacaggtagagaacaggctggagaaaggc
atagctaagataagg-
>_R_gnl|SRA|SRR1213690.13332724.1 HISEQ1:355:D1Y5LACXX:3:1302:3341:57739.

----------------------------------caaacagtaggaccatagtgggggat
tctatcaacaccatgagcctcagggaccttaaacaggtagagaacaggctggagaaaggc
atagctaagataagg-
>_R_gnl|SRA|SRR1213690.13134822.1 HISEQ1:355:D1Y5LACXX:3:1301:8205:95831.

----------------------------------caaacagtaggaccatagtgggggat
tctatcaacaccatgagcctcagggaccttaaacaggtagagaacaggctggagaaaggc
atagctaagataagg-
>_R_gnl|SRA|SRR1213690.12374065.1 HISEQ1:355:D1Y5LACXX:3:1216:2403:9314.

----------------------------------caaacagtaggaccatagtgggggat
tctatcaacaccatgagcctcagggaccttaaacaggtagagaacaggctggagaaaggc
atagctaagataagg-
>_R_gnl|SRA|SRR1213690.12304869.1 HISEQ1:355:D1Y5LACXX:3:1215:3910:94022.

----------------------------------caaacagtaggaccatagtgggggat
tctatcaacaccatgagcctcagggaccttaaacaggtagagaacaggctggagaaaggc
atagctaagataagg-
>_R_gnl|SRA|SRR1213690.12276354.1 HISEQ1:355:D1Y5LACXX:3:1215:4585:88413.

----------------------------------caaacagtaggaccatagtgggggat
tctatcaacaccatgagcctcagggaccttaaacaggtagagaacaggctggagaaaggc
atagctaagataagg-
>_R_gnl|SRA|SRR1213690.5525004.1 HISEQ1:355:D1Y5LACXX:3:1115:16105:28803.

----------------------------------caaacagtaggaccatagtgggggat
tctatcaacaccatgagcctcagggaccttaaacaggtagagaacaggctggagaaaggc
atagctaagataagg-
>_R_gnl|SRA|SRR1213690.9961938.1 HISEQ1:355:D1Y5LACXX:3:1210:21407:80142.

----------------------------------caaacagtaggaccatagtggggnat
tctnnnnnnnnnnnnngcctcanngaccttaaacaggtagagaacaggctggagaaaggc
atagctaagataagn-
>_R_gnl|SRA|SRR1213690.1826367.1 HISEQ1:355:D1Y5LACXX:3:1106:9437:70153.

----------------------------------caaacagtaggaccatagtgggggat
tctatccacaccatgagtctcagggaccttaaacaggtagagaacaggctggagaaaggc
atagctaagataagg-
>_R_gnl|SRA|SRR1213690.33015809.1 HISEQ1:355:D1Y5LACXX:3:2303:19226:66563.

-----------------------------------aaacagtaggaccatagtgggggat
tctatcaacaccatgagcctcagggaccttaaacaggtagagaacaggctggagaaaggc
atagctaagataaggg
>_R_gnl|SRA|SRR1213690.31284683.1 HISEQ1:355:D1Y5LACXX:3:2215:6478:38319.

-----------------------------------aaacagtaggaccatagtgggggat
tctatcaacaccatgngcctcngggaccttaaacaggtagagaacaggctggagaaaggc

atagctaagataaggg
>_R_gnl|SRA|SRR1213690.29508211.1 HISEQ1:355:D1Y5LACXX:3:2211:18395:60022.

-----------------------------------aaacagtaggaccatagtgggggat
tctatcaacaccatgngcctcngggaccttaaacaggtagagaacaggctggagaaaggc
atagctaagataaggg
>_R_gnl|SRA|SRR1213690.24308888.1 HISEQ1:355:D1Y5LACXX:3:2114:2896:65218.

-----------------------------------aaacagtaggaccatagtgggggat
tctatcaacaccatgagcctcagggaccttaaacaggtagagaacaggctggagaaaggc
atagctaagataaggg
>_R_gnl|SRA|SRR1213690.21901200.1 HISEQ1:355:D1Y5LACXX:3:2109:12518:38273.

-----------------------------------aaacagtaggaccatagtgggggat
tctatcaacaccatgagcctcagggaccttaaacaggtagagaacaggctggagaaaggc
atagctaagataaggg
>_R_gnl|SRA|SRR1213690.15602940.1 HISEQ1:355:D1Y5LACXX:3:1308:21219:90804.

-----------------------------------aaacagtaggaccatagtgggggat
tctatcaacaccatgagcctcagggaccttaaacaggtagagaacaggctggagaaaggc
atagctaagataaggg
>_R_gnl|SRA|SRR1213690.12240802.1 HISEQ1:355:D1Y5LACXX:3:1215:17664:81017.

-----------------------------------aaacagtaggaccatagtgggggat
tctatcaacaccatgagcctcagggaccttaaacaggtagagaacaggctggagaaaggc
atagctaagataaggg
>_R_gnl|SRA|SRR1213690.7217458.1 HISEQ1:355:D1Y5LACXX:3:1203:3351:56463.

-----------------------------------aaacagtaggaccatagtgggggat
tctatcaacaccatgagcctcagggaccttaaacaggtagagaacaggctggagaaaggc
atagctaagataaggg
>_R_gnl|SRA|SRR1213690.6647819.1 HISEQ1:355:D1Y5LACXX:3:1201:14219:83910.

-----------------------------------aaacagtaggaccatagtgggggat
tctatcaacaccatgagcctcagggaccttaaacaggtagagaacaggctggagaaaggc
atagctaagataaggg
>_R_gnl|SRA|SRR1213690.2860791.1 HISEQ1:355:D1Y5LACXX:3:1109:11444:36259.

-----------------------------------aaacagtaggaccatagtgggggat
tctatcaacaccatgagcctcagggaccttaaacaggtagagaacaggctggagaaaggc
atagctaagataaggg
>_R_gnl|SRA|SRR1213690.998587.1 HISEQ1:355:D1Y5LACXX:3:1104:17226:31574.

-----------------------------------aaacagtaggaccatagtgggggat
tctatcaacaccatgagcctcagggaccttaaacaggtagagaacaggctggagaaaggc
atagctaagataaggg
>_R_gnl|SRA|SRR1213690.752953.1 HISEQ1:355:D1Y5LACXX:3:1103:18644:55112.

-----------------------------------aaacagtaggaccatagtgggggat
tctatcaacaccatgagcctcagggaccttaaacaggtagagaacaggctggagaaaggc
atagctaagataaggg

>_R_gnl|SRA|SRR1213690.26842994.1 HISEQ1:355:D1Y5LACXX:3:2204:15344:78075.

-----------------------------------aaacagtaggaccatagtgggggat
tctatcaacaccatgngcctctgggaccttaaacaggtagagaacaggctggagaaaggc
atagctaagataaggg
>_R_gnl|SRA|SRR1213690.26366385.1 HISEQ1:355:D1Y5LACXX:3:2203:6575:40146.

-----------------------------------aaacagtaggaccatagtgggggat
tctatcaacaccatgngcctctgggaccttaaacaggtagagaacaggctggagaaaggc
atagctaagataaggg
>_R_gnl|SRA|SRR1213690.32164781.1 HISEQ1:355:D1Y5LACXX:3:2301:6605:5255.

-----------------------------------aaacagtaggaccatagtgggggat
tctatcaacaccatgagcctcagggaccttaaacaggtagagaacaggttggagaaaggc
atagctaagataaggg
>_R_gnl|SRA|SRR1213690.15595123.1 HISEQ1:355:D1Y5LACXX:3:1308:2869:89168.

-----------------------------------aaacagtaggaccatagtgggggat
tctatcaacaccatgagcctcagggaccttaaacaggtagagaacaggctggagaaaggc
atagctaagatagggg
>_R_gnl|SRA|SRR1213690.8586278.1 HISEQ1:355:D1Y5LACXX:3:1207:18124:38407.

-----------------------------------aaacagtaggaccatagtgggggat
tctatcaacaccatgagcctcagggaccttaaacaggtagagaacaggctggagaaaggc
atagctaagatagggg
>_R_gnl|SRA|SRR1213690.11149716.1 HISEQ1:355:D1Y5LACXX:3:1213:4658:51037.

-----------------------------------aaacagtaggaccatagtgggggat
tctatcaacaccatgagcctcagggaccttaaacaggtagagaacaggctggagaaaggc
atagctaagataagga
>_R_gnl|SRA|SRR1213690.26599631.1 HISEQ1:355:D1Y5LACXX:3:2204:7085:8942.

-----------------------------------aaacagtaggaccatagtgggggat
tctatcaacaccatangcctccgggaccttaaacaggtagagaacaggctggagaaaggc
atagctaagataaggg
>_R_gnl|SRA|SRR1213690.2729136.1 HISEQ1:355:D1Y5LACXX:3:1109:13886:4430.

-----------------------------------aaacagtaggaccatagtgggggat
tctatcaaccccatgagcctcagggaccttaaacaggtagagaacaggctggagaaaggc
atagctaagataaggg
>_R_gnl|SRA|SRR1213690.6132837.1 HISEQ1:355:D1Y5LACXX:3:1116:12392:51436.

-----------------------------------aaacagtaggaccatagtggtggat
tctatcaacaccatgagcctcagggaccttaaacaggtagagaacaggctggagaaaggc
atagctaagataaggg
>_R_gnl|SRA|SRR1213690.6018905.1 HISEQ1:355:D1Y5LACXX:3:1116:7252:29117.

-----------------------------------ctctagtaggaccatagtgggggat
tctatcaacaccatgagcctcagggaccttaaacaggtagagaacaggctggagaaaggc
atagctaagataaggg
>_R_gnl|SRA|SRR1213690.36589173.1 HISEQ1:355:D1Y5LACXX:3:2312:7615:92792.

------------------------------aacgcaaaca---ggaccatagtgggggat
tctatcaacaccatgagcctcagggaccttaaacaggtagagaacaggctggagaaaggc
atagctaagataag--
>_R_gnl|SRA|SRR1213690.34585659.1 HISEQ1:355:D1Y5LACXX:3:2308:6011:6113.

------------------------------aacgcaaaca---ggaccatagtgggggat
tctatcaacaccatgagcctcagggaccttaaacaggtagagaacaggctggagaaaggc
atagctaagataag--
>_R_gnl|SRA|SRR1213690.33627417.1 HISEQ1:355:D1Y5LACXX:3:2305:4085:44344.

------------------------------aacgcaaaca---ggaccatagtgggggat
tctatcaacaccatgagcctcagggaccttaaacaggtagagaacaggctggagaaaggc
atagctaagataag--
>_R_gnl|SRA|SRR1213690.20144149.1 HISEQ1:355:D1Y5LACXX:3:2104:14169:74309.

------------------------------aacgcaaaca---ggaccatagtgggggat
tctatcaacaccatgagcctcagggaccttaaacaggtagagaacaggctggagaaaggc
atagctaagataag--
>_R_gnl|SRA|SRR1213690.19089989.1 HISEQ1:355:D1Y5LACXX:3:2101:6462:48693.

------------------------------ctcgcaaaca---ggaccatagtgggggat
tctatcaacaccatgagcctcagggaccttaaacaggtagagaacaggctggagaaaggc
atagctaagataag--
>_R_gnl|SRA|SRR1213690.34135288.1 HISEQ1:355:D1Y5LACXX:3:2306:1309:84740.

-------------------------------acgcaaaca---ggaccatagtgggggat
tctatcaacaccatgagcctcagggaccttaaacaggtagagaacaggctggagaaaggc
atagctaagataagg-
>_R_gnl|SRA|SRR1213690.22338396.1 HISEQ1:355:D1Y5LACXX:3:2110:5880:42223.

-------------------------------acgcaaaca---ggaccatagtgggggat
tctatcaacaccatgagcctcagggaccttaaacaggtagagaacaggctggagaaaggc
atagctaagataagg-
>_R_gnl|SRA|SRR1213690.15818616.1 HISEQ1:355:D1Y5LACXX:3:1309:17313:46499.

-------------------------------acgcaaaca---ggaccatagtgggggat
tctatcaacaccatgagcctcagggaccttaaacaggtagagaacaggctggagaaaggc
atagctaagataagg-
>_R_gnl|SRA|SRR1213690.13900033.1 HISEQ1:355:D1Y5LACXX:3:1304:1680:27057.

-------------------------------acgcaaaca---ggaccatagtgggggat
tctatcaacaccatgagcctcagggaccttaaacaggtagagaacaggctggagaaaggc
atagctaagataagg-
>_R_gnl|SRA|SRR1213690.3582826.1 HISEQ1:355:D1Y5LACXX:3:1111:16032:7985.

-------------------------------acgcaaaca---ggaccatagtgggggat
tctatcaacaccatgagcctcagggaccttaaacaggtagagaacaggctggagaaaggc
atagctaagataagg-
>_R_gnl|SRA|SRR1213690.16667017.1 HISEQ1:355:D1Y5LACXX:3:1311:1659:56261.

-------------------------------acgcaaaca---ggaccatagtgggggat
tctatcaacaccatgagcctcagggaccttaaacaggtagagaacaggctggagaaaggc
atagctaagataaga-
>_R_gnl|SRA|SRR1213690.14883259.1 HISEQ1:355:D1Y5LACXX:3:1307:2926:3126.

-------------------------------acgcaaaca---ggaccatagtgggggat
tctatcaacaccatgagcctcagggaccttaaacagggagagaacaggctggagaaaggc
atagctaagataagg-
>_R_gnl|SRA|SRR1213690.26431501.1 HISEQ1:355:D1Y5LACXX:3:2203:9398:59190.

-------------------------------acgcaaaca---ggaccatagtgggggat
tctatcaacaccatnagcctgagggaccttaaacaggtagagaacaggctggagaaaggc
atagctaagataagg-
>_R_gnl|SRA|SRR1213690.32167359.1 HISEQ1:355:D1Y5LACXX:3:2301:10415:6064.

-------------------------------tcgcaaaca---ggaccatagtgggggat
tctatcaacaccatgagcctcagggaccttaaacaggtagagaacagcctggagaaaggc
atagctaagataagg-
>_R_gnl|SRA|SRR1213690.37278997.1 HISEQ1:355:D1Y5LACXX:3:2314:2980:47884.

--------------------------------cgcaaaca---ggaccatagtgggggat
tctatcaacaccatgagcctcagggaccttaaacaggtagagaacaggctggagaaaggc
atagctaagataaggg
>_R_gnl|SRA|SRR1213690.16976657.1 HISEQ1:355:D1Y5LACXX:3:1312:15804:31496.

--------------------------------cgcaaaca---ggaccatagtgggggat
tctatcaacaccatgagcctcagggaccttaaacaggtagagaacaggctggagaaaggc
atagctaagataaggg
>_R_gnl|SRA|SRR1213690.15744174.1 HISEQ1:355:D1Y5LACXX:3:1309:7771:27841.

--------------------------------cgcaaaca---ggaccatagtgggggat
tctatcaacaccatgagcctcagggaccttaaacaggtagagaacaggctggagaaaggc
atagctaagataaggg
>_R_gnl|SRA|SRR1213690.12925646.1 HISEQ1:355:D1Y5LACXX:3:1301:19540:30986.

--------------------------------cgcaaaca---ggaccatagtgggggat
tctatcaacaccatgagcctcagggaccttaaacaggtagagaacaggctggagaaaggc
atagctaagataaggg
>_R_gnl|SRA|SRR1213690.38351711.1 HISEQ1:355:D1Y5LACXX:3:2316:7357:75479.
--accagcaggagtcctcca
aactgcgccaacaaatcagtagcttac-agaacgcaaacagtaggaccatagtgggggat
tctatcaacaccatgagcctcagg------------------------------------

>_R_gnl|SRA|SRR1213690.34409749.1 HISEQ1:355:D1Y5LACXX:3:2307:20884:58581.
--accagcaggagtcctcca
aactgcgccaacaaatcagtagcttac-agaacgcaaacagtaggaccatagtgggggat
tctatcaacaccatgagcctcagg------------------------------------

>_R_gnl|SRA|SRR1213690.20404662.1 HISEQ1:355:D1Y5LACXX:3:2105:7276:49034.
--accagcaggagtcctcca
aactgcgccaacaaatcagtagcttac-agaacgcaaacagtaggaccatagtgggggat

tctatcaacaccatgagcctcagg------------------------------------

>_R_gnl|SRA|SRR1213690.17610433.1 HISEQ1:355:D1Y5LACXX:3:1313:18792:82098.
--accagcaggagtcctcca
aactgcgccaacaaatcagtagcttac-agaacgcaaacagtaggaccatagtgggggat
tctatcaacaccatgagcctcagg------------------------------------

>_R_gnl|SRA|SRR1213690.16960590.1 HISEQ1:355:D1Y5LACXX:3:1312:5647:27510.
--accagcaggagtcctcca
aactgcgccaacaaatcagtagcttac-agaacgcaaacagtaggaccatagtgggggat
tctatcaacaccatgagcctcagg------------------------------------

>_R_gnl|SRA|SRR1213690.9849260.1 HISEQ1:355:D1Y5LACXX:3:1210:17113:53868.
--accagcaggagtcctcca
aactgcgccaacaaatcagtagcttac-agaacgcaaacagtaggaccatagtgggggat
tctatcaacaccatgagcctcagg------------------------------------

>_R_gnl|SRA|SRR1213690.31987597.1 HISEQ1:355:D1Y5LACXX:3:2216:7078:71238.
--accagcaggagtcctcca
aactgcgccaacaaatcagtagnttac-anaacgcaaacagtaggaccatagtgggggat
tctatcaacaccatgagcctcagg------------------------------------

>_R_gnl|SRA|SRR1213690.20382442.1 HISEQ1:355:D1Y5LACXX:3:2105:12700:42763.
--accagcaggagtcctcca
aactgcgccaacaaatcagtagcttac-agaacgcaaacagtaggaccatagtgggggat
tctatcaataccatgagcctcagg------------------------------------

>_R_gnl|SRA|SRR1213690.17727037.1 HISEQ1:355:D1Y5LACXX:3:1314:18079:10875.
--atcagcaggggtcctcca
aactgcgccaacaaatcagtagcttac-agaacgcaaacagtaggaccatagtgggggat
tctatcaacaccatgagcctcagg------------------------------------

>_R_gnl|SRA|SRR1213690.32611056.1 HISEQ1:355:D1Y5LACXX:3:2302:7994:44528.
--accaccaggagtcctcca
aactgcgccaacaaatcagtagcttac-agaacgcaaacagtaggaccatagtgggggat
tctatcaacaccatgagcctcagg------------------------------------

>_R_gnl|SRA|SRR1213690.1786320.1 HISEQ1:355:D1Y5LACXX:3:1106:20126:59024.
--accagcaggagtcctcca
aactgcgccaacaaatcagtagcttac-agaacgcaaacagtaggaccatagtgggggat
tctatcaacaccatgagcctcacg------------------------------------

>_R_gnl|SRA|SRR1213690.32862555.1 HISEQ1:355:D1Y5LACXX:3:2303:4335:21727.
---ccagcaggagtcctcca
aactgcgccaacaaatcagtagcttac-agaacgcaaacagtaggaccatagtgggggat
tctatcaacaccatgagcctcaggg-----------------------------------

>_R_gnl|SRA|SRR1213690.11542354.1 HISEQ1:355:D1Y5LACXX:3:1214:13288:35576.
---ccagcaggagtcctcca
aactgcgccaacaaatcagtagcttac-agaacgcaaacagtaggaccatagtgggggat
tctatcaacaccatgagcctcaggg-----------------------------------

>_R_gnl|SRA|SRR1213690.37641213.1 HISEQ1:355:D1Y5LACXX:3:2315:14504:26777.
--ctaccagcaggagtcctcca
aactgcgccaacaaatcagtagcttac-agaacgcaaacagtaggaccatagtgggggat
tctatcaacaccatgagcctca--------------------------------------

>_R_gnl|SRA|SRR1213690.8119558.1 HISEQ1:355:D1Y5LACXX:3:1206:11776:13477.
--ctaccagcaggagtcctcca
aactgcgccaacaaatcagtagcttac-agaacgcaaacagtaggaccatagtgggggat
tctatcaacaccatgagcctca--------------------------------------

>_R_gnl|SRA|SRR1213690.325480.1 HISEQ1:355:D1Y5LACXX:3:1102:11017:15379.
--ctaccagcaggagtcctcca
aactgcgccaacaaatcagtagcttac-agaacgcaaacagtaggaccatagtgggggat
tctatcaacaccatgagcctca--------------------------------------

>_R_gnl|SRA|SRR1213690.30135264.1 HISEQ1:355:D1Y5LACXX:3:2212:18739:99812.
--ctaccagcaggagtcctcca
aactgcgccaacaaatcagtngcttan-agaacgcaaacagtaggaccatagtgggggat
tctatcaacaccatgagcctca--------------------------------------

>_R_gnl|SRA|SRR1213690.28149554.1 HISEQ1:355:D1Y5LACXX:3:2208:10314:31802.
--ctaccagcaggagtcctcca
aactgcgccaacaaatcagtngcttat-agaacgcaaacagtaggaccatagtgggggat
tctatcaacaccatgagcctca--------------------------------------

>_R_gnl|SRA|SRR1213690.826418.1 HISEQ1:355:D1Y5LACXX:3:1103:15686:77902.
--ctaccagcaggagtcctcca
aactgcgccaacaaatcagtagcttac-agaacgcaaacagtaggaccatagtgggggat
tctatcaacaccatgagcctcc--------------------------------------

>_R_gnl|SRA|SRR1213690.36903066.1 HISEQ1:355:D1Y5LACXX:3:2313:9308:63830.
---taccagcaggagtcctcca
aactgcgccaacaaatcagtagcttac-agaacgcaaacagtaggaccatagtgggggat
tctatcaacaccatgagcctcag-------------------------------------

>_R_gnl|SRA|SRR1213690.22163002.1 HISEQ1:355:D1Y5LACXX:3:2109:18588:100250.
---taccagcaggagtcctcca
aactgcgccaacaaatcagtagcttac-agaacgcaaacagtaggaccatagtgggggat
tctatcaacaccatgagcctcag-------------------------------------

>_R_gnl|SRA|SRR1213690.14443540.1 HISEQ1:355:D1Y5LACXX:3:1305:17795:81008.
---taccagcaggagtcctcca
aactgcgccaacaaatcagtagcttac-agaacgcaaacagtaggaccatagtgggggat
tctatcaacaccatgagcctcag-------------------------------------

>_R_gnl|SRA|SRR1213690.5874786.1 HISEQ1:355:D1Y5LACXX:3:1115:10306:99186.
---taccagcaggagtcctcca
aactgcgccaacaaatcagtagcttac-agaacgcaaacagtaggaccatagtgggggat
tctatcaacaccatgagcctcag-------------------------------------

>_R_gnl|SRA|SRR1213690.5613678.1 HISEQ1:355:D1Y5LACXX:3:1115:9074:46933.
--taccagcaggagtcctcca
aactgcgccaacaaatcagtagcttac-agaacgcaaacagtaggaccatagtgggggat
tctatcaacaccatgagcctcag------------------------------------

>_R_gnl|SRA|SRR1213690.3284571.1 HISEQ1:355:D1Y5LACXX:3:1110:13948:38158.
--taccagcaggagtcctcca
aactgcgccaacaaatcagtagcttac-agaacgcaaacagtaggaccatagtgggggat
tctatcaacaccatgagcctcag------------------------------------

>_R_gnl|SRA|SRR1213690.29663041.1 HISEQ1:355:D1Y5LACXX:3:2211:19509:94757.
--taccagcaggagtcctcca
aactgcgccaacaaatcagtancttac-ngaacgcaaacagtaggaccatagtgggggat
tctatcaacaccatgagcctcag------------------------------------

>_R_gnl|SRA|SRR1213690.27404757.1 HISEQ1:355:D1Y5LACXX:3:2206:9173:35377.
--taccagcaggagtcctcca
aactgcgccaacaaatcagtancttac-ggaacgcaaacagtaggaccatagtgggggat
tctatcaacaccatgagcctcag------------------------------------

>_R_gnl|SRA|SRR1213690.33826066.1 HISEQ1:355:D1Y5LACXX:3:2305:14747:99481.
--tatcagcaggagtcctcca
aactgcgccaacaaatcagtagcttac-agaacgcaaacagtaggaccatagtgggggat
tctatcaacaccatgagcctcag------------------------------------

>_R_gnl|SRA|SRR1213690.5458835.1 HISEQ1:355:D1Y5LACXX:3:1115:18840:15480.
--taccagcaggagtcctcca
aactgcgccaacaaatcagtagcttac-agaacgcaaacagtaggaccatagtgggggat
tctatcaacaccatgagccccag------------------------------------

>_R_gnl|SRA|SRR1213690.22779193.1 HISEQ1:355:D1Y5LACXX:3:2111:14731:42928.
--taccagcaggagtcctcca
aactgcgccaacaaatcagtagcttac-agaacgcgaacagtaggaccatagtgggggat
tctatcaacaccatgagcctcag------------------------------------

>_R_gnl|SRA|SRR1213690.7855775.1 HISEQ1:355:D1Y5LACXX:3:1205:20644:39867.
--taccagcaggagtcctcca
aactgcgccaagaaatcagtagcttac-agaacgcaaacagtaggaccatagtgggggat
tctatccacgccatgagcctcag------------------------------------

>_R_gnl|SRA|SRR1213690.38335721.1 HISEQ1:355:D1Y5LACXX:3:2316:14402:72042.
---------------------------------------ctaccagcaggagtcctcca
aactgcgccaacaaatcagtagcttac-agaacgcaaac---aggaccatagtgggggat
tctatcaacaccatgagcctcaggg----------------------------------

>_R_gnl|SRA|SRR1213690.36519951.1 HISEQ1:355:D1Y5LACXX:3:2312:14085:77058.
---------------------------------------ctaccagcaggagtcctcca
aactgcgccaacaaatcagtagcttac-agaacgcaaac---aggaccatagtgggggat
tctatcaacaccatgagcctcaggg----------------------------------

>_R_gnl|SRA|SRR1213690.38052134.1 HISEQ1:355:D1Y5LACXX:3:2316:18345:14999.

--ctaccagcaggagtcctcca
aactgcgccaacaaatcagtagcttac-agaacgcaaac---cggaccatagtgggggat
tctatcaacaccatgagcctcaggg-----------------------------------

>_R_gnl|SRA|SRR1213690.16074861.1 HISEQ1:355:D1Y5LACXX:3:1310:12165:11123.
---------------------------------------actaccagcaggagtcctcca
aactgcgccaacaaatcagtagcttac-agaacgcaaac---aggaccatagtgggggat
tctatcaacaccatgagcctcagg------------------------------------

>_R_gnl|SRA|SRR1213690.15932987.1 HISEQ1:355:D1Y5LACXX:3:1309:1539:75200.
---------------------------------------actaccagcaggagtcctcca
aactgcgccaacaaatcagtagcttac-agaacgcaaac---aggaccatagtgggggat
tctatcaacaccatgagcctcagg------------------------------------

>_R_gnl|SRA|SRR1213690.31177469.1 HISEQ1:355:D1Y5LACXX:3:2215:5726:17608.
---------------------------------------actaccagcaggagtcctcca
aactgcgccaacaaatcagnagcttnc-agaacgcaaac---aggaccatagtgggggat
tctatcaacaccatgagcctcggg------------------------------------

>_R_gnl|SRA|SRR1213690.36466392.1 HISEQ1:355:D1Y5LACXX:3:2312:18187:64909.
-------------------------------------gcactaccagcaggagtcctcca
aactgcgccaacaaatcagtagcttac-agaacgcaaac---aggaccatagtgggggat
tctatcaacaccatgagcctca--------------------------------------

>_R_gnl|SRA|SRR1213690.23906563.1 HISEQ1:355:D1Y5LACXX:3:2113:6934:84222.
-------------------------------------gcactaccagcaggagtcctcca
aactgcgccaacaaatcagtagcttac-agaacgcaaac---aggaccatagtgggggat
tctatcaacaccatgagcctca--------------------------------------

>_R_gnl|SRA|SRR1213690.10801430.1 HISEQ1:355:D1Y5LACXX:3:1212:14314:73866.
-------------------------------------gcactaccagcaggagtcctcca
aactgcgccaacaaatcagtagcttac-agaacgcaaac---aggaccatagtgggggat
tctatcaacaccatgagcctca--------------------------------------

>_R_gnl|SRA|SRR1213690.24107208.1 HISEQ1:355:D1Y5LACXX:3:2114:9325:25501.
--------------------------------------cactaccagcaggagtcctcca
aactgcgccaacaaatcagtagcttac-agaacgcaaac---aggaccatagtgggggat
tctatcaacaccatgagcctcag-------------------------------------

>_R_gnl|SRA|SRR1213690.22291618.1 HISEQ1:355:D1Y5LACXX:3:2110:3368:31485.
--------------------------------------cactaccagcaggagtcctcca
aactgcgccaacaaatcagtagcttac-agaacgcaaac---aggaccatagtgggggat
tctatcaacaccatgagcctcag-------------------------------------

>_R_gnl|SRA|SRR1213690.15509980.1 HISEQ1:355:D1Y5LACXX:3:1308:19272:67449.
--------------------------------------cactaccagcaggagtcctcca
aactgcgccaacaaatcagtagcttac-agaacgcaaac---aggaccatagtgggggat
tctatcaacaccatgagcctcag-------------------------------------

>_R_gnl|SRA|SRR1213690.13265140.1 HISEQ1:355:D1Y5LACXX:3:1302:18532:36802.
--------------------------------------cactaccagcaggagtcctcca

aactgcgccaacaaatcagtagcttac-agaacgcaaac---aggaccatagtgggggat
tctatcaacaccatgagcctcag-------------------------------------

>_R_gnl|SRA|SRR1213690.5445637.1 HISEQ1:355:D1Y5LACXX:3:1115:3788:12789.
--------------------------------------cactaccagcaggagtcctcca
aactgcgccaacaaatcagtagcttac-agaacgcaaac---aggaccatagtgggggat
tctatcaacaccatgagcctcag-------------------------------------

>_R_gnl|SRA|SRR1213690.3632027.1 HISEQ1:355:D1Y5LACXX:3:1111:7026:19187.
--------------------------------------cactaccagcaggagtcctcca
aactgcgccaacaaatcagtagcttac-agaacgcaaac---aggaccatagtgggggat
tctatcaacaccatgagcctcag-------------------------------------

>_R_gnl|SRA|SRR1213690.603269.1 HISEQ1:355:D1Y5LACXX:3:1103:9783:7917.
--------------------------------------cactaccagcaggagtcctcca
aactgcgccaacaaatcagtagcttac-agaacgcaaac---aggaccatagtgggggat
tctatcaacaccatgagcctcag-------------------------------------

>_R_gnl|SRA|SRR1213690.15932703.1 HISEQ1:355:D1Y5LACXX:3:1309:15763:74893.
--------------------------------------cactaccagcaggagtcctcca
aactgcgccaacaaatcagtagcttac-agaacgcaaac---aggaccatagtgggggat
tctatcaacaccatgagccccag-------------------------------------

>_R_gnl|SRA|SRR1213690.26387347.1 HISEQ1:355:D1Y5LACXX:3:2203:15599:46113.
--------------------------------------cactaccagcaggagtcctcca
aactgcgccaacaaatcantagctcac-agaacgcaaac---aggaccatagtgggggat
tctatcaacaccatgagcctcag-------------------------------------

>_R_gnl|SRA|SRR1213690.26397849.1 HISEQ1:355:D1Y5LACXX:3:2203:19842:49195.
--------------------------------------cactaccagcaggagtcctcca
aactgcgccaacaaatcantagctgac-agaacgcaaac---aggaccatagtgggggat
tctatcaacaccatgagcctcag-------------------------------------

>_R_gnl|SRA|SRR1213690.36314631.1 HISEQ1:355:D1Y5LACXX:3:2312:7963:30373.
---ccagcaggagtcctcca
aactgcgccaacaaatcagtagcttac-agaacgcaaac---aggaccatagtgggggat
tctatcaacaccatgagcctcagggacc--------------------------------

>_R_gnl|SRA|SRR1213690.32633980.1 HISEQ1:355:D1Y5LACXX:3:2302:2422:51642.
---ccagcaggagtcctcca
aactgcgccaacaaatcagtagcttac-agaacgcaaac---aggaccatagtgggggat
tctatcaacaccatgagcctcagggacc--------------------------------

>_R_gnl|SRA|SRR1213690.31484584.1 HISEQ1:355:D1Y5LACXX:3:2215:21050:76995.
---ccagcaggagtcctcca
aactgcgccaacaaatcagtagcntac-agnacgcaaac---aggaccatagtgggggat
tctatcaacaccatgagcctcagggacc--------------------------------

>_R_gnl|SRA|SRR1213690.32810193.1 HISEQ1:355:D1Y5LACXX:3:2303:2758:6099.
----------------------------------ccagcactaccagcaggagtcctcca
aactgcgccaacaaatcagtagcttac-agaacgcaaacagtaggaccatagtgggggat

tctatcaacaccatga--

>_R_gnl|SRA|SRR1213690.11109781.1 HISEQ1:355:D1Y5LACXX:3:1213:15691:42306.
--------------------------------ccagcactaccagcaggagtcctcca
aactgcgccaacaaatcagtagcttac-agaacgcaaacagtaggaccatagtgggggat
tctatcaacaccatga--

>_R_gnl|SRA|SRR1213690.21898340.1 HISEQ1:355:D1Y5LACXX:3:2109:19200:37589.
----------------------------------agcactaccagcaggagtcctcca
aactgcgccaacaaatcagtagcttac-agaacgcaaacagtaggaccatagtgggggat
tctatcaacaccatgagc--

>_R_gnl|SRA|SRR1213690.14608391.1 HISEQ1:355:D1Y5LACXX:3:1306:5453:27702.
-----------------------------------gcactaccagcaggagtcctcca
aactgcgccaacaaatcagtagcttac-agaacgcaaacagtaggaccatagtgggggat
tctatcaacaccatgagcc---------------------------------------

>_R_gnl|SRA|SRR1213690.13120549.1 HISEQ1:355:D1Y5LACXX:3:1301:14400:91282.
-----------------------------------gcactaccagcaggagtcctcca
aactgcgccaacaaatcagtagcttac-agaacgcaaacagtaggaccatagtgggggat
tctatcaacaccatgagcc---------------------------------------

>_R_gnl|SRA|SRR1213690.25552084.1 HISEQ1:355:D1Y5LACXX:3:2116:16315:93852.
---------------------------------cagcactaccagcaggagtcctcca
acctgcgccaacaaatcagtagcttac-agaacgcaaacagtaggaccatagtgggggat
tctatcaacaccatggg---

>_R_gnl|SRA|SRR1213690.548279.1 HISEQ1:355:D1Y5LACXX:3:1102:4892:88956.
-------------------------------cccagcactaccagcaggagtcctcca
aactgcgccaacaaatcagtagcttac-agaacgcaaacagtaggaccatagtgggggat
tctatcaacaccgag---

>_R_gnl|SRA|SRR1213690.38031861.1 HISEQ1:355:D1Y5LACXX:3:2316:5957:10954.
----------------------------atgcccagctctaccagcaggagtcctcca
aactgcgccaacaaatcagtagcttac-agaacgcaaacagtaggaccatagtgggggat
tctatcaacacc--

>_R_gnl|SRA|SRR1213690.29738498.1 HISEQ1:355:D1Y5LACXX:3:2212:8803:13170.
----------------------------atgcccagcactaccagcaggagtcctcca
aactgcgccancaaatnagtagcttac-agaacgcaaacagtaggaccatagtgggggat
tctatcaacacc--

>_R_gnl|SRA|SRR1213690.37161753.1 HISEQ1:355:D1Y5LACXX:3:2314:8848:22167.
-----------------------------tgcccagcactaccagcaggagtcctcca
aactgcgccaacaaatcagtagcttac-agaacgcaaacagtaggaccatagtgggggat
tctatcaacacca---

>_R_gnl|SRA|SRR1213690.19102433.1 HISEQ1:355:D1Y5LACXX:3:2101:11188:52657.
------------------------------gcccagcactaccagcaggagtcctcca
aactgcgccaacaaatcagtagcttac-agaacgcaaacagtaggaccatagtgggggat
tctatcaacaccac--

>_R_gnl|SRA|SRR1213690.37400356.1 HISEQ1:355:D1Y5LACXX:3:2314:11844:73808.
----------------------------caatgcccagcactaccagcaggagtcctcca
aactgcgccaacaaatcagtagcttac-agaacgcaaacagtaggaccatagtgggggat
tctatcaaca--

>_R_gnl|SRA|SRR1213690.36829878.1 HISEQ1:355:D1Y5LACXX:3:2313:11317:47618.
----------------------------caatgcccagcactaccagcaggagtcctcca
aactgcgccaacaaatcagtagcttac-agaacgcaaacagtaggaccatagtgggggat
tctatcaaca--

>_R_gnl|SRA|SRR1213690.36682236.1 HISEQ1:355:D1Y5LACXX:3:2313:14531:14776.
----------------------------caatgcccagcactaccagcaggagtcctcca
aactgcgccaacaaatcagtagcttac-agaacgcaaacagtaggaccatagtgggggat
tctatcaaca--

>_R_gnl|SRA|SRR1213690.10027614.1 HISEQ1:355:D1Y5LACXX:3:1210:5551:95519.
----------------------------caatgcccagcactaccagcaggagtcctcca
aactgcgccaacaaatcagtagcttac-agaacgcaaacagtaggaccatagtgggggat
tctatcaaca--

>_R_gnl|SRA|SRR1213690.2674683.1 HISEQ1:355:D1Y5LACXX:3:1108:14966:89802.
----------------------------caatgcccagcactaccagcaggagtcctcca
aactgcgccaacaaatcagtagcttac-agaacgcaaacagtaggaccatagtgggggat
tctatcaaca--

>_R_gnl|SRA|SRR1213690.27213748.1 HISEQ1:355:D1Y5LACXX:3:2205:4746:82358.
----------------------------caatgcccagcactaccagcaggagtcctcca
aactgcgcnaacaattcagtagcttac-agaacgcaaacagtaggaccatagtgggggat
tctatcaaca--

>_R_gnl|SRA|SRR1213690.12995301.1 HISEQ1:355:D1Y5LACXX:3:1301:6296:52573.
-----------------------------aatgcccagcactaccagcaggagtcctcca
aactgcgccaacaaatcagtagcttac-agaacgcaaacagtaggaccatagtgggggat
tctatcaacac---

>_R_gnl|SRA|SRR1213690.25262463.1 HISEQ1:355:D1Y5LACXX:3:2116:15082:43425.
---------------------------tcaatgccctgcactaccagcaggagtcctcca
aactgcgccaacaaatcagtagcttac-agaacgcaaacagtaggaccatagtgggggat
tctatcaac---

>_R_gnl|SRA|SRR1213690.24864060.1 HISEQ1:355:D1Y5LACXX:3:2115:15370:70251.
---------------------------tcaatgcccagcactaccagcaggagtcctcca
aactgcgccaacaaatcagtagcttac-agaacgcaaacagtaggaccatagtgggggat
tctatcaac---

>_R_gnl|SRA|SRR1213690.22350725.1 HISEQ1:355:D1Y5LACXX:3:2110:12435:44874.
---------------------------tcaatgcccagcactaccagcaggagtcctcca
aactgcgccaacaaatcagtagcttac-agaacgcaaacagtaggaccatagtgggggat
tctatcaac---

>_R_gnl|SRA|SRR1213690.17905273.1 HISEQ1:355:D1Y5LACXX:3:1314:4187:52935.
---------------------------tcaatgcccagcactaccagcaggagtcctcca
aactgcgccaacaaatcagtagcttac-agaacgcaaacagtaggaccatagtgggggat
tctatcaac---

>_R_gnl|SRA|SRR1213690.36887753.1 HISEQ1:355:D1Y5LACXX:3:2313:17141:60389.
-----------------------gaagtcaatgcccagcactaccagcaggagtcctcca
aactgcgccaacaaatcagtagcttac-agaacgcaaacagtaggaccatagtgggggat
tctat---

>_R_gnl|SRA|SRR1213690.33632525.1 HISEQ1:355:D1Y5LACXX:3:2305:19444:45731.
-----------------------gaagtcaatgcccagcactaccagcaggagtcctcca
aactgcgccaacaaatcagtagcttac-agaacgcaaacagtaggaccatagtgggggat
tctat---

>_R_gnl|SRA|SRR1213690.9502646.1 HISEQ1:355:D1Y5LACXX:3:1209:16539:70110.
-----------------------gaagtcaatgcccagcactaccagcaggagtcctcca
aactgcgccaacaaatcagtagcttac-agaacgcaaacagtaggaccatagtgggggat
tctat---

>_R_gnl|SRA|SRR1213690.4640874.1 HISEQ1:355:D1Y5LACXX:3:1113:12146:43322.
-----------------------gaagtcaatgcccagcactaccagcaggagtcctcca
aactgcgccaacaaatcagtagcttac-agaacgcaaacagtaggaccatagtgggggat
tctat---

>_R_gnl|SRA|SRR1213690.1274789.1 HISEQ1:355:D1Y5LACXX:3:1105:18750:14447.
-----------------------gaagtcaatgcccagcactaccagcaggagtcctcca
aactgcgccaacaaatcagtagcttac-agaacgcaaacagtaggaccatagtgggggat
tctat---

>_R_gnl|SRA|SRR1213690.28629448.1 HISEQ1:355:D1Y5LACXX:3:2209:3513:52094.
-----------------------gaagtcaatgcccagcactaccagcaggagtcctcca
aacngcgcccacaaatcagtagcttac-agaacgcaaacagtaggaccatagtgggggat
tctat---

>_R_gnl|SRA|SRR1213690.26356123.1 HISEQ1:355:D1Y5LACXX:3:2203:6404:37013.
-----------------------gaagtcaatgcccagcactaccagcaggagtcctcca
aacngcgcccacaaatcagtagcttac-agaacgcaaacagtaggaccatagtgggggat
tctat---

>_R_gnl|SRA|SRR1213690.23389516.1 HISEQ1:355:D1Y5LACXX:3:2112:11865:76043.
-------------------------agtcaatgcccagcactaccagcaggagtcctcca
aactgcgccaacaaatcagtagcttac-agaacgcaaacagtaggaccatagtgggggat
tctatca---

>_R_gnl|SRA|SRR1213690.15690307.1 HISEQ1:355:D1Y5LACXX:3:1309:6058:14362.
------------------------aagtcaatgcccagcactaccagcaggagtcctcca
aactgcgccaacaaatcagtagcttac-agaacgcaaacagtaggaccatagtgggggat
tctatc--

>_R_gnl|SRA|SRR1213690.34153007.1 HISEQ1:355:D1Y5LACXX:3:2306:4747:89284.

--------------------gcagaagtcaatgcccagcactaccagcaggagtcctcca
aactgcgccaacaaatcagtagcttac-agaacgcaaacagtaggaccatagtgggggat
tc---

>_R_gnl|SRA|SRR1213690.20642112.1 HISEQ1:355:D1Y5LACXX:3:2106:20263:15248.
---------------------cagaagtcaatgcccagcactaccagcaggagtcctcca
aactgcgccaacaaatcagtagcttac-agaacgcaaacagtaggaccatagtgggggat
tct--

>_R_gnl|SRA|SRR1213690.2272255.1 HISEQ1:355:D1Y5LACXX:3:1107:19272:87814.
---------------------cagaagtcaatgcccagcactaccagcaggagtcctcca
aactgcgccaacaaatcagtagcttac-agaacgcaaacagtaggaccatagtgggggat
tct--

>_R_gnl|SRA|SRR1213690.31471261.1 HISEQ1:355:D1Y5LACXX:3:2215:21064:74274.
----------------------agaagtcaatgcccagcactaccagcaggagtcctcca
aantgcgcnaacaaatcagtagcttac-agaacgcaaacagtaggaccatagtgggggat
tcta---

>_R_gnl|SRA|SRR1213690.16046488.1 HISEQ1:355:D1Y5LACXX:3:1310:1398:4402.
----------------------agaagtcaatgcccagcactaccagcaggagtcctcca
aactgcgccaacaaatcagtagcttac-agaacgcaaacagtaggaccatagtgggggat
tcta---

>_R_gnl|SRA|SRR1213690.38367969.1 HISEQ1:355:D1Y5LACXX:3:2316:19042:78397.
----------------------------------ccagcactaccagcaggagtcctcca
aactgcgccaacaaatcagtagcttac-agaacgcaaac---aggaccatagtgggggat
tctatcaacaccatgagcc--

>_R_gnl|SRA|SRR1213690.16002266.1 HISEQ1:355:D1Y5LACXX:3:1309:8174:92064.
----------------------------------ccagcactaccagcaggagtcctcca
aactgcgccaacaaatcagtagcttac-agaacgcaaac---aggaccatagtgggggat
tctatcaacaccatgagcc--

>_R_gnl|SRA|SRR1213690.26902476.1 HISEQ1:355:D1Y5LACXX:3:2204:13933:94904.
----------------------------------ccagcactaccagcaggagtcctcca
aactgcgccaacaantcagtttcttac-agaacgcaaac---aggaccatagtgggggat
tctatcaacaccatgagcc--

>_R_gnl|SRA|SRR1213690.30404035.1 HISEQ1:355:D1Y5LACXX:3:2213:12503:58379.
-------------------------------tgcccagcactaccagcaggagtcctcca
aactgcgccaanaaatcngtagcttac-agaacgcaaac---aggaccatagtgggggat
tctatcaacaccatga---

>_R_gnl|SRA|SRR1213690.22751047.1 HISEQ1:355:D1Y5LACXX:3:2111:14983:36677.
--------------------------------gcccagcactaccagcaggagtcctcca
aactgcgccaacaaatcagtagcttac-agaacgcaaac---aggaccatagtgggggat
tctatcaacaccatgag--

>_R_gnl|SRA|SRR1213690.1437427.1 HISEQ1:355:D1Y5LACXX:3:1105:11152:61044.
--------------------------------gcccagcactaccagcaggagtcctcca

aactgcgccaacaaatcagtagcttac-agaacgcaaac---aggaccatagtgggggat
tctatcaacaccatgag---

>_R_gnl|SRA|SRR1213690.37411813.1 HISEQ1:355:D1Y5LACXX:3:2314:3785:76370.
--------------------------gtcaatgcccagcactaccagcaggagtcctcca
aactgcgccaacaaatcagtagcttac-agaacgcaaacag---gaccatagtgggggat
tctatcaacac---

>_R_gnl|SRA|SRR1213690.14560454.1 HISEQ1:355:D1Y5LACXX:3:1306:20990:14305.
---------------------------tcaatgcccagcactaccagcaggagtcctcca
aactgcgccaacaaatcagtagcttac-agaacgcaaacag---gaccatagtgggggat
tctatcaacacc--

>_R_gnl|SRA|SRR1213690.13637424.1 HISEQ1:355:D1Y5LACXX:3:1303:8432:49478.
---------------------------tcaatgcccagcactaccagcaggagtcctcca
aactgcgccaacaaatcagtagcttac-agaacgcaaacag---gaccatagtgggggat
tctatcaacacc--

>_R_gnl|SRA|SRR1213690.36205520.1 HISEQ1:355:D1Y5LACXX:3:2312:6654:5385.
-------------------------agtcaatgcccagcactaccagcaggagtcctcca
aactgcgccaacaaatcagtagcttac-agaacgcaaacag---gaccatagtgggggat
tctatcaaca--

>_R_gnl|SRA|SRR1213690.15514149.1 HISEQ1:355:D1Y5LACXX:3:1308:5840:68694.
-------------------------agtcaatgcccagcactaccagcaggagtcctcca
aactgcgccaacaaatcagtagcttac-agaacgcaaacag---gaccatagtgggggat
tctatcaaca--

>_R_gnl|SRA|SRR1213690.13596265.1 HISEQ1:355:D1Y5LACXX:3:1303:4064:37422.
-------------------------agtcaatgcccagcactaccagcaggagtcctcca
aactgcgccaacaaatcagtagcttac-agaacgcaaacag---gaccatagtgggggat
tctatcaaca--

>_R_gnl|SRA|SRR1213690.9154495.1 HISEQ1:355:D1Y5LACXX:3:1208:6217:83864.
-------------------------agtcaatgcccagcactaccagcaggagtcctcca
aactgcgccaacaaatcagtagcttac-agaacgcaaacag---gaccatagtgggggat
tctatcaaca--

>_R_gnl|SRA|SRR1213690.27139505.1 HISEQ1:355:D1Y5LACXX:3:2205:17419:61825.
-------------------------agtcaatgcccagcactaccagcaggagtcctcca
aactgngccaataaatcagtagcttac-agaacgcaaacag---gaccatagtgggggat
tctatcaaca--

>_R_gnl|SRA|SRR1213690.35695098.1 HISEQ1:355:D1Y5LACXX:3:2310:14042:83750.
------------------------aagtcaatgcccagcactaccagcaggagtcctcca
aactgcgccaacaaatcagtagcttac-agaacgcaaacag---gaccatagtgggggat
tctttcaac---

>_R_gnl|SRA|SRR1213690.21662961.1 HISEQ1:355:D1Y5LACXX:3:2108:3544:79639.
------------------------aagtcaatgcccagcactaccagcaggagtcctcca
aactgcgccaacaaatcagtagcttac-agaacgcaaacag---gaccatagtgggggat

tctatcaac---

>_R_gnl|SRA|SRR1213690.15179783.1 HISEQ1:355:D1Y5LACXX:3:1307:21292:80798.
------------------------aagtcaatgcccagcactaccagcaggagtcctcca
aactnngccaacaaatcagtagcttac-agaacgcaaacag---gaccatagtgggggat
tctatcaac---

>_R_gnl|SRA|SRR1213690.14837743.1 HISEQ1:355:D1Y5LACXX:3:1306:8513:89654.
------------------------aagtcaatgcccagcactaccagcaggagtcctcca
aactgcgccaacaaatcagtagcttac-agaacgcaaacag---gaccatagtgggggat
tctatcaac---

>_R_gnl|SRA|SRR1213690.2714284.1 HISEQ1:355:D1Y5LACXX:3:1108:20102:99462.
------------------------aagtcaatgcccagcactaccagcaggagtcctcca
aactgcgccaacaaatcagtagcttac-agaacgcaaacag---gaccatagtgggggat
tctatcaac---

>_R_gnl|SRA|SRR1213690.30521049.1 HISEQ1:355:D1Y5LACXX:3:2213:2121:82792.
------------------------aagtcaatgcccagcactaccagcaggagtcctcca
aactncgccancaaatcagtagcttac-agaacgcaagcag---gaccatagtgggggat
tctatcaac---

>_R_gnl|SRA|SRR1213690.5485425.1 HISEQ1:355:D1Y5LACXX:3:1115:11869:20902.
-----------------------------aatgcccagcactaccagcaggagtcctcca
aactgcgccaacaaatcagtagcttac-agaacgcaaacag---gaccatagtgggggat
tctatcaacaccag--

>_R_gnl|SRA|SRR1213690.34847400.1 HISEQ1:355:D1Y5LACXX:3:2308:2473:73086.
---------------------cagaagtcaatgcccagcactaccagcaggagtcctcca
aactgcgccaacaaatcagtagcttac-agaacgcaaacag---gaccatagtgggggat
tctatc--

>_R_gnl|SRA|SRR1213690.33882205.1 HISEQ1:355:D1Y5LACXX:3:2306:13510:16181.
----------------------agaagtcaatgcccagcactaccagcaggagtcctcca
aactgcgccaacaaatcagtagcttac-agaacgcaaacag---gaccatagtgggggat
tctatca---

>_R_gnl|SRA|SRR1213690.20562498.1 HISEQ1:355:D1Y5LACXX:3:2105:3589:92363.
----------------------agaagtcaatgcccagcactaccagcaggagtcctcca
aactgcgccaacaaatcagtagcttac-agaacgcaaacag---gaccatagtgggggat
tctatca---

>_R_gnl|SRA|SRR1213690.18199527.1 HISEQ1:355:D1Y5LACXX:3:1315:2205:23728.
----------------------agaagtcaatgcccagcactaccagcaggagtcctcca
aactgcgccaacaaatcagtagcttac-agaacgcaaacag---gaccatagtgggggat
tctatca---

>_R_gnl|SRA|SRR1213690.1550164.1 HISEQ1:355:D1Y5LACXX:3:1105:19464:92824.
----------------------agaagtcaatgcccagcactaccagcaggagtcctcca
aactgcgccaacaaatcagtagcttac-agaacgcaaacag---gaccatagtgggggat
tctatca---

>_R_gnl|SRA|SRR1213690.30025821.1 HISEQ1:355:D1Y5LACXX:3:2212:13543:76230.
--------------------gcagaagtcaatgcccagcactaccagcaggagtcctcca
nactgcnccaacaaatcagtagcttac-agaacgcaaacag---gaccatagtgggggat
tctat--

>_R_gnl|SRA|SRR1213690.25296984.1 HISEQ1:355:D1Y5LACXX:3:2116:4868:49508.
--------------------gcagaagtcaatgcccagcactaccagcaggagtcctcca
aactgcgccaacaaatcagtagcttac-agaacgcaaacag---gaccatagtgggggat
tctat--

>_R_gnl|SRA|SRR1213690.24741359.1 HISEQ1:355:D1Y5LACXX:3:2115:14806:47794.
--------------------gcagaagtcaatgcccagcactaccagcaggagtcctcca
aactgcgccaacaaatcagtagcttac-agaacgcaaacag---gaccatagtgggggat
tctat--

>_R_gnl|SRA|SRR1213690.16871829.1 HISEQ1:355:D1Y5LACXX:3:1312:15471:6283.
--------------------gcagaagtcaatgcccagcactaccagcaggagtcctcca
aactgcgccaacaaatcagtagcttac-agaacgcaaacag---gaccatagtgggggat
tctat--

>_R_gnl|SRA|SRR1213690.5343926.1 HISEQ1:355:D1Y5LACXX:3:1114:10316:90613.
--------------------gcagaagtcaatgcccagcactaccagcaggagtcctcca
aactgcgccaacaaatcagtagcttac-agaacgcaaacag---gaccatagtgggggat
tctat--

>_R_gnl|SRA|SRR1213690.29396718.1 HISEQ1:355:D1Y5LACXX:3:2211:19828:34704.
--------------------gcagaagtcaatgcccagcactaccagcaggagtcctcca
nactgcnccaacaaatcagtagcttac-agaacgcaaacag---gaccattgtgggggat
tctat--

>_R_gnl|SRA|SRR1213690.22389626.1 HISEQ1:355:D1Y5LACXX:3:2110:6644:53799.
------------------ttgcagaagtcaatgcccagcactaccagcaggagtcctcca
aactgcgccaacaaatcagtagcttac-agaacgcaaacag---gaccatagtgggggat
tct--

>_R_gnl|SRA|SRR1213690.16530413.1 HISEQ1:355:D1Y5LACXX:3:1311:17944:23429.
------------------ttgcagaagtcaatgcccagcactaccagcaggagtcctcca
aactgcgccaacaaatcagtagcttac-agaacgcaaacag---gaccatagtgggggat
tct--

>_R_gnl|SRA|SRR1213690.19683973.1 HISEQ1:355:D1Y5LACXX:3:2103:6458:38327.
-----------------------gaagtcaatgcccagcactaccagcaggagtcctcca
aactgcgccaacaaatcagtagcttac-agaacgcaaacag---gaccaaagtgggggat
tctatcaa---

>_R_gnl|SRA|SRR1213690.25751848.1 HISEQ1:355:D1Y5LACXX:3:2201:17165:52185.
------------------------------atgcccagcactaccagcaggagtcctcga
aactgcgccaccaaattagtagcttac-agaacgcaaacag---gaccatagtgggggat
tctatcaacaccatg--

>_R_gnl|SRA|SRR1213690.17542043.1 HISEQ1:355:D1Y5LACXX:3:1313:1827:66298.
-----------------gttgcagaagtcaatgcccagcactaccagcaggagtcctcca
aactgcgccaacaaatcagtagcttac-agaacgcaaacagtaggaccatagtggggga-

>_R_gnl|SRA|SRR1213690.18478719.1 HISEQ1:355:D1Y5LACXX:3:1315:15618:88617.
-----------------gttgcagaagtcaatgcccagcactaccagcaggagtcctcca
aactgcgccaacaaatcagtagcttac-ataacgcaaacagtaggaccatagtgagggt-

>_R_gnl|SRA|SRR1213690.38153980.1 HISEQ1:355:D1Y5LACXX:3:2316:14287:35525.
----------------agttgcagaggtcaatgcccagcactaccagcaggagtcctcca
aactgcgccaacaaatcagtagcttac-agaacgcaaacagtaggaccatagtggggg--

>_R_gnl|SRA|SRR1213690.24302470.1 HISEQ1:355:D1Y5LACXX:3:2114:2950:63853.
----------------agttgcagaagtcaatgcccagcactaccagcaggagtcctcca
aactgcgccaacaaatcagtagcttac-agaacgcaaacagtaggaccatagtggggg--

>_R_gnl|SRA|SRR1213690.26258454.1 HISEQ1:355:D1Y5LACXX:3:2203:10315:8145.
----------------agttgcagaagtcaatgcccagcactaccagcaggagtccncca
aactgcgccaacaaatcagtagcttac-agaacgcaaacagtaggaccatagtgggaa--

>_R_gnl|SRA|SRR1213690.1212840.1 HISEQ1:355:D1Y5LACXX:3:1104:11241:95038.
----------------agttgcagaagtcaatgcccagcactaccagcaggagtcctcca
aactgcgccaacaaatcagtagcttac-agaacgcaaacagtaggaccatagtgggcg--

>_R_gnl|SRA|SRR1213690.25935819.1 HISEQ1:355:D1Y5LACXX:3:2202:16206:9888.
----------------agttgcagaagtcaatgcccagcactaccagcaggagtcctcca
aagtgcgccaacaaatcagtagcttac-agaacgcaaacagtaggaccatagtggggg--

>_R_gnl|SRA|SRR1213690.38099463.1 HISEQ1:355:D1Y5LACXX:3:2316:9314:24637.
---------------cagttgcagtagtcaatgcccagcactaccagcaggagtcctcca
aactgcgccaacaaatcagtagcttac-agaacgcaaacagtaggaccatagtgggg---

>_R_gnl|SRA|SRR1213690.35651249.1 HISEQ1:355:D1Y5LACXX:3:2310:20532:73142.
---------------cagttgcagaagtcaatgctcagcactaccagcaggagtcctcca
aactgcgccaacaaatcaatagcttac-agaacgcaaacagtaggaccatagtgggg---

>_R_gnl|SRA|SRR1213690.31943052.1 HISEQ1:355:D1Y5LACXX:3:2216:8888:63207.
---------------cagttgcagaagtcaatgcccagcactaccagcaggagtcntcca
anctgcgccaacaaatcagtagcttac-agaacgcaaacagtaggaccatagtgggg---

>_R_gnl|SRA|SRR1213690.28292860.1 HISEQ1:355:D1Y5LACXX:3:2208:6822:67828.

---------------cagttgcagaagtcaatgcccagcactaccagcaggagtcntcca
agctgcgccaacaaatcagtagcttac-agaacgcaaacagtaggaccatagtgggg---

>_R_gnl|SRA|SRR1213690.22950725.1 HISEQ1:355:D1Y5LACXX:3:2111:3392:81226.
---------------cagttgcagaagtcaatgcccagcactaccagcaggagtcctcca
aactgcgccaacaaatcagtagcttac-agaacgcaaacagtaggaccatagtgggg---

>_R_gnl|SRA|SRR1213690.13841096.1 HISEQ1:355:D1Y5LACXX:3:1304:4941:10068.
---------------cagttgcagaagtcaatgcccagcactaccagcaggagtcctcca
aactgcgccaacaaatcagtagcttac-agaacgcaaacagtaggaccatagtgggg---

>_R_gnl|SRA|SRR1213690.13710881.1 HISEQ1:355:D1Y5LACXX:3:1303:16631:70970.
---------------cagttgcagaagtcaatgcccagcactaccagcaggagtcctcca
aactgcgccaacaaatcagtagcttac-agaacgcaaacagtaggaccatagtgggg---

>_R_gnl|SRA|SRR1213690.9632122.1 HISEQ1:355:D1Y5LACXX:3:1210:7898:2630.
---------------cagttgcagaagtcaatgcccagcactaccagcaggagtcctcca
aactgcgccaacaaatcagtagcttac-agaacgcaaacagtaggaccatagtgggg---

>_R_gnl|SRA|SRR1213690.7844816.1 HISEQ1:355:D1Y5LACXX:3:1205:18220:36869.
---------------cagttgcagaagtcaatgcccagcactaccagcaggagtcctcca
aactgcgccaacaaatcagtagcttac-agaacgcaaacagtaggaccatagtgggg---

>_R_gnl|SRA|SRR1213690.7489222.1 HISEQ1:355:D1Y5LACXX:3:1204:9886:35776.
---------------cagttgcagaagtcaatgcccagcactaccagcaggagtcctcca
aactgcgccaacaaatcagtagcttac-agaacgcaaacagtaggaccatagtgggg---

>_R_gnl|SRA|SRR1213690.5047917.1 HISEQ1:355:D1Y5LACXX:3:1114:12626:29844.
---------------cagttgcagaagtcaatgcccagcactaccagcaggagtcctcca
aactgcgccaacaaatcagtagcttac-agaacgcaaacagtaggaccatagtgggg---

>_R_gnl|SRA|SRR1213690.4859140.1 HISEQ1:355:D1Y5LACXX:3:1113:15190:89391.
---------------cagttgcagaagtcaatgcccagcactaccagcaggagtcctcca
aactgcgccaacaaatcagtagcttac-agaacgcaaacagtaggaccatagtgggg---

>_R_gnl|SRA|SRR1213690.4369694.1 HISEQ1:355:D1Y5LACXX:3:1112:9197:84155.
---------------cagttgcagaagtcaatgcccagcactaccagcaggagtcctcca
aactgcgccaacaaatcagtagcttac-agaacgcaaacagtaggaccatagtgggg---

>_R_gnl|SRA|SRR1213690.1568041.1 HISEQ1:355:D1Y5LACXX:3:1105:17472:97933.
---------------cagttgcagaagtcaatgcccagcactaccagcaggagtcctcca

aactgcgccaacaaatcagtagcttac-agaacgcaaacagtaggaccatagtgggg---

>_R_gnl|SRA|SRR1213690.27644074.1 HISEQ1:355:D1Y5LACXX:3:2206:15116:98896.
---------------cagttgcagaagtcaatgcccagcactaccagcaggagtcntcca
acctgcgccaacaaatcagtagcttac-agaacgcaaacagtaggaccatagtgggg---

>_R_gnl|SRA|SRR1213690.27624158.1 HISEQ1:355:D1Y5LACXX:3:2206:16666:93719.
---------------cagttgcagaagtcaatgcccagcactaccagcaggagtcntcca
acctgcgccaacaaatcagtagcttac-agaacgcaaacagtaggaccatagtgggg---

>_R_gnl|SRA|SRR1213690.33610841.1 HISEQ1:355:D1Y5LACXX:3:2305:17612:39661.
---------------cagttgcagaagtcaatgcccagcactaccagcaggagtcctcca
aactgcgccaacaaatcagtagcttac-agaacgcaaacagtaggaccatagcgggg---

>_R_gnl|SRA|SRR1213690.29191801.1 HISEQ1:355:D1Y5LACXX:3:2210:12952:86354.
---------------cagttgcagaagtcaatgcccagcactaccagcaggagtcntcca
anctgcgccaacaaatcagtagcttac-agaacgcaaacagtaggaccatagcgggg---

>_R_gnl|SRA|SRR1213690.34579258.1 HISEQ1:355:D1Y5LACXX:3:2308:13387:4342.
---------------cagttgcagaagtcaatgcccagcactaccagcaggagtcctcca
aactgcgccaacaaatcagtagcttac-agaacgcaaacagtaggaccatagtgagg---

>_R_gnl|SRA|SRR1213690.35061516.1 HISEQ1:355:D1Y5LACXX:3:2309:13090:28093.
---------------cagttgcagaagtctatgcccagcactaccagcaggagtcctcca
aactgcgccaacaaatcagtagcttac-agaacgcaaacagtaggaccatagtgggg---

>_R_gnl|SRA|SRR1213690.22047596.1 HISEQ1:355:D1Y5LACXX:3:2109:14228:73146.
---------------cagttgcagaagtcaatgcccagcactaccagcaggagtcctcca
aactgcgccaacaaatcagtagcttac-agaacgcaaacagtaggaccatagtgggt---

>_R_gnl|SRA|SRR1213690.35957498.1 HISEQ1:355:D1Y5LACXX:3:2311:1424:46849.
---------------cagttgcagaagtcaatgcccagcactaccagcaggagtcctcca
aactgcgccaacaaatcagtagcttac-agaacgcaaacagttggaccatagtgggg---

>_R_gnl|SRA|SRR1213690.16971912.1 HISEQ1:355:D1Y5LACXX:3:1312:4122:30366.
---------------cagttgcagaagtcaatgcccagcactaccagcaggagtcatcca
aactgcgccaccaaatcagtagcttac-agaacgcaaacagtaggaccatagtgggg---

>_R_gnl|SRA|SRR1213690.37971850.1 HISEQ1:355:D1Y5LACXX:3:2315:9744:97201.
---------------aagttgcaaaagtcaatgcccagcactaccagcaggagtcctcca
aactgcgccaacaaatcagtagcttac-agaacgcaaacagtaggaccatagtgggg---

>_R_gnl|SRA|SRR1213690.29345791.1 HISEQ1:355:D1Y5LACXX:3:2211:15813:23201.
---------------cagttgcagaagtcaatgcccagctctaccagcaggagtcntcca
anctgcgccaacaaatcagtagcttac-agaacggaaacagttggaccatagtgggg---

>_R_gnl|SRA|SRR1213690.7318196.1 HISEQ1:355:D1Y5LACXX:3:1203:6612:85664.
---------------cagttgcagaagtcaatgcccagcactaccagcacgagtcctcca
aactgcgccaacaaatcagtagcttag-agaacgcaaacagtaggaccatagtgggg---

>_R_gnl|SRA|SRR1213690.33302379.1 HISEQ1:355:D1Y5LACXX:3:2304:14980:51211.
--------------acagttgcagaagtcaatgcccagcactaccagcaggagtcctcca
aactgcgccaacaaatcagtagcttac-agaacgcaaacagtaggaccatagtggg----

>_R_gnl|SRA|SRR1213690.23340479.1 HISEQ1:355:D1Y5LACXX:3:2112:8723:65979.
--------------acagttgcagaagtcaatgcccagcactaccagcaggagtcctcca
aactgcgccaacaaatcagtagcttac-agaacgcaaacagtaggaccatagtggg----

>_R_gnl|SRA|SRR1213690.1597591.1 HISEQ1:355:D1Y5LACXX:3:1106:15093:7429.
--------------acagttgcagaagtcaatgcccagcactaccagcaggagtcctcca
aactgcgccaacaaatcagtagcttac-agaacgcaaacagtaggaccatagtggg----

>_R_gnl|SRA|SRR1213690.16393259.1 HISEQ1:355:D1Y5LACXX:3:1310:13686:88884.
--------------acagttgcagaagtcaatgcccagcactaccagcaggagtcctcca
aactgcgccaacaaatcagtagcttac-agaacgcaaacagtaggaccatagtgag----

>_R_gnl|SRA|SRR1213690.1542923.1 HISEQ1:355:D1Y5LACXX:3:1105:17776:90959.
--------------acagttgcagaagtcaatgcccagcactaccagcaggagtcctcca
acccgcgccaacaaatcagtagcttac-agaacgcaaacagtaggaccatagtggg----

>_R_gnl|SRA|SRR1213690.33172774.1 HISEQ1:355:D1Y5LACXX:3:2304:16933:13607.
-------------cacagttgcagaagtcaatgcccagcactaccagcaggagtcctcca
aactgcgccaacaaatcagtagcttac-agaacgcaaacagtaggaccatagtgg-----

>_R_gnl|SRA|SRR1213690.25386302.1 HISEQ1:355:D1Y5LACXX:3:2116:19326:65087.
-------------cacagttgcagaagtcaatgcccagcactaccagcaggagtcctcca
aactgcgccaacaaatcagtagcttac-agaacgcaaacagtaggaccatagtgg-----

>_R_gnl|SRA|SRR1213690.17969488.1 HISEQ1:355:D1Y5LACXX:3:1314:17292:67672.
-------------cacagttgcagaagtcaatgcccagcactaccagcaggagtcctcca
aactgcgccaacaaatcagtagcttac-agaacgcaaacagtaggaccatagtgg-----

>_R_gnl|SRA|SRR1213690.1009711.1 HISEQ1:355:D1Y5LACXX:3:1104:20326:34947.
-------------cacagttgcagaagtcaatgcccagcactaccagcaggagtcctcca
aactgcgccaacaaatcagtagcttac-agaacgcaaacagtaggaccatagtgg-----

>_R_gnl|SRA|SRR1213690.22408255.1 HISEQ1:355:D1Y5LACXX:3:2110:4139:58200.
------------gcacagttgcagaagtcaatgcccagcactaccagcaggagtcctcca
aactgcgccaacaaatcagtagcttac-agaacgcaaacagtaggaccatagtg------

>_R_gnl|SRA|SRR1213690.11747982.1 HISEQ1:355:D1Y5LACXX:3:1214:8649:78648.
------------gcacagttgcagaagtcaatgcccagcactaccagcaggagtcctcca
aactgcgccaacaaatcagtagcttac-agaacgcaaacagtaggaccatagtg------

>_R_gnl|SRA|SRR1213690.6522667.1 HISEQ1:355:D1Y5LACXX:3:1201:13836:45375.
------------gcacagttgcagaagtcaatgcccagcactaccagcaggagtcctcca
aactgcgccaacaaatcagtagcttac-agaacgcaaacagtaggaccatagtg------

>_R_gnl|SRA|SRR1213690.34986829.1 HISEQ1:355:D1Y5LACXX:3:2309:15311:9437.
----------tggcacagttgcagaagtcaatgcccagcactaccagcaggagtcctcca
aactgcgccaacaaatcagtagcttac-agaacgcaaacagtaggaccatag--------

>_R_gnl|SRA|SRR1213690.32179857.1 HISEQ1:355:D1Y5LACXX:3:2301:14339:10232.
---------------cagttgcagaagtcaatgcccagcactaccagcaggagtcctcca
aactgcgccaacaaatcagtagcttac-agaacgcaaacag---gaccatagtgggggac

>_R_gnl|SRA|SRR1213690.3260025.1 HISEQ1:355:D1Y5LACXX:3:1110:15855:32450.
---------------cagttgcagaagtcaatgcccagcactaccagcaggagtcctcca
aactgcgccaacaaatcagtagcttac-agaacgcaaacag---gaccatagtgggggac

>_R_gnl|SRA|SRR1213690.33754495.1 HISEQ1:355:D1Y5LACXX:3:2305:9258:79720.
-------------cacagttgcagaagtcaatgcccagcactaccagcaggagtcctcca
aactgcgccaacaaatcagtagcttac-agaacgcaaacag---gaccatagtggggg--

>_R_gnl|SRA|SRR1213690.29781973.1 HISEQ1:355:D1Y5LACXX:3:2212:11598:22657.
-------------cacagttgcagaagtcaatgcccagcactaccagcaggagncctccn
aactgcgccaacaaatcagtagcttac-agaacgcaaacag---gaccatagtggggg--

>_R_gnl|SRA|SRR1213690.14625551.1 HISEQ1:355:D1Y5LACXX:3:1306:20865:32200.
-------------cacagttgcagaagtcaatgcccagcactaccagcaggagtcctcca
aactgcgccaacaaatcagtagcttac-agaacgcaaacag---gaccatagtggggg--

>_R_gnl|SRA|SRR1213690.26768862.1 HISEQ1:355:D1Y5LACXX:3:2204:14201:57064.
-------------cacagttgcagaagtcaatgcccagcactaccagcaggagncctccg
aactgcgccaacaaatcagtagcttac-agaacgcaaacag---gaccatagtggggg--

>_R_gnl|SRA|SRR1213690.784900.1 HISEQ1:355:D1Y5LACXX:3:1103:20069:64955.
-------------cacagttgcagaagtcaatgccccgcactaccagcaggagtcctcca
aactgcgccaacaaatcagtagcttac-agaacgcaaacag---gaccatagtggggg--

>_R_gnl|SRA|SRR1213690.37280994.1 HISEQ1:355:D1Y5LACXX:3:2314:16013:48077.
------------gcacagttgcagaagtcaatgcccagcattaccagcaggagtcctcca
aactgcgccaacaaatcagtagcttac-agaacgcaaacag---gaccatagtgggg---

>_R_gnl|SRA|SRR1213690.36539427.1 HISEQ1:355:D1Y5LACXX:3:2312:5486:81587.
------------gcacagttgcagaagtcaatgcccagcactaccagcaggagtcctcca
aactgcgccaacaaatcagtagcttac-agaacgcaaacag---gaccatagtgggg---

>_R_gnl|SRA|SRR1213690.34633482.1 HISEQ1:355:D1Y5LACXX:3:2308:15059:18449.
------------gcacagttgcagaagtcaatgcccagcactaccagcaggagtcctcca
aactgcgccaacaaatcagtagcttac-agaacgcaaacag---gaccatagtgggg---

>_R_gnl|SRA|SRR1213690.20438071.1 HISEQ1:355:D1Y5LACXX:3:2105:4957:58461.
------------gcacagttgcagaagtcaatgcccagcactaccagcaggagtcctcca
aactgcgccaacaaatcagtagcttac-agaacgcaaacag---gaccatagtgggg---

>_R_gnl|SRA|SRR1213690.20292635.1 HISEQ1:355:D1Y5LACXX:3:2105:3014:17963.
------------gcacagttgcagaagtcaatgcccagcactaccagcaggagtcctcca
aactgcgccaacaaatcagtagcttac-agaacgcaaacag---gaccatagtgggg---

>_R_gnl|SRA|SRR1213690.18509759.1 HISEQ1:355:D1Y5LACXX:3:1315:7441:95923.
------------gcacagttgcagaagtcaatgcccagcactaccagcaggagtcctcca
aactgcgccaacaaatcagtagcttac-agaacgcaaacag---gaccatagtgggg---

>_R_gnl|SRA|SRR1213690.16180725.1 HISEQ1:355:D1Y5LACXX:3:1310:8537:37120.
------------gcacagttgcagaagtcaatgcccagcactaccagcaggagtcctcca
aactgcgccaacaaatcagtagcttac-agaacgcaaacag---gaccatagtgggg---

>_R_gnl|SRA|SRR1213690.14127654.1 HISEQ1:355:D1Y5LACXX:3:1304:20741:91758.
------------gcacagttgcagaagtcaatgcccagcactaccagcaggagtcctcca
aactgcgccaacaaatcagtagcttac-agaacgcaaacag---gaccatagtgggg---

>_R_gnl|SRA|SRR1213690.17890923.1 HISEQ1:355:D1Y5LACXX:3:1314:18010:49480.

------------gcacagttgcagaagtcaatgcccagcactaccagcaggagtcctcca
aactgcgccaacaaatcagtagcttac-agaacgcaaacag---gaccatagtggag---

>_R_gnl|SRA|SRR1213690.9601529.1 HISEQ1:355:D1Y5LACXX:3:1209:15617:93895.
------------gcacagttgcagaagtcaatgcccagcactaccagcaggagtcctcca
aactgcgccaacaaatcagtagcttac-agaacgcaaacag---gaccatagtggag---

>_R_gnl|SRA|SRR1213690.15604823.1 HISEQ1:355:D1Y5LACXX:3:1308:19931:91489.
------------gcacagttgcagaagtcaatgcccagcactaccagcaggagtcctcca
aactgcgccaacaaatcagtagcttac-agaacgcaaacag---gaccatagcgggg---

>_R_gnl|SRA|SRR1213690.14226897.1 HISEQ1:355:D1Y5LACXX:3:1305:15301:21213.
------------gcacagttgcagaagtcaatgcccagcactaccagcaggagtcctcca
aactgcgccaacaaatcagtagcttac-agaacgcaaacag---gaccatagcgggg---

>_R_gnl|SRA|SRR1213690.10761192.1 HISEQ1:355:D1Y5LACXX:3:1212:2521:65198.
------------gcacagttgcagaagtcaatgcccagcactaccagcaggagtcctcca
aactgcgccaacaaatcagtagcttac-agaacgcaaacag---gaccatagcgggg---

>_R_gnl|SRA|SRR1213690.4424149.1 HISEQ1:355:D1Y5LACXX:3:1112:4570:95888.
------------gcacagttgcagaagtcaatgcccagcactaccagcaggagtcctcca
aactgcgccaacaaatcagtagcttac-agaacgcaaacag---gaccatagcgggg---

>_R_gnl|SRA|SRR1213690.1220386.1 HISEQ1:355:D1Y5LACXX:3:1104:5997:97355.
------------gcacagttgcagaagttaatgcccagcactaccagcaggagtcctcca
aactgcgccaacaaatcagtagcttac-agaacgcaaacag---gaccatagtaggg---

>_R_gnl|SRA|SRR1213690.15143032.1 HISEQ1:355:D1Y5LACXX:3:1307:20412:71305.
-----------ggcacagttgcagaagtcaatgcccagcactaccagcaggagtcctcca
aactgcgccaacaaatcagtagcttac-agaacgcaaacag---gaccatagtggg----

>_R_gnl|SRA|SRR1213690.13864602.1 HISEQ1:355:D1Y5LACXX:3:1304:9124:16782.
-----------ggcacagttgcagaagtcaatgcccagcactaccagcaggagtcctcca
aactgcgccaacaaatcagtagcttac-agaacgcaaacag---gaccatagtggg----

>_R_gnl|SRA|SRR1213690.11463447.1 HISEQ1:355:D1Y5LACXX:3:1214:20380:19044.
-----------ggcacagttgcagaagtcaatgcccagcactaccagcaggagtcctcca
aactgcgccaacaaatcagtagcttac-agaacgcaaacag---gaccatagtggg----

>_R_gnl|SRA|SRR1213690.4166719.1 HISEQ1:355:D1Y5LACXX:3:1112:14283:39755.
-----------ggcacagttgcagaagtcaatgcccagcactaccagcaggagtcctcca

aactgcgccaacaaatcagtagcttac-agaacgcaaacag---gaccatagtggg----

>_R_gnl|SRA|SRR1213690.1201083.1 HISEQ1:355:D1Y5LACXX:3:1104:16055:91738.
-----------ggcacagttgcagaagtcaatgcccagcactaccagcaggagtcctcca
aactgcgccaacaaatcagtagcttac-agaacgcaaacag---gaccatagtggg----

>_R_gnl|SRA|SRR1213690.23834304.1 HISEQ1:355:D1Y5LACXX:3:2113:7917:69267.
-----------ggcacagttgcagaagtcaatgcccagcactaccagcaggagtcctcca
aactgcgccaacaaatcagtagcttac-agaacgcaaacag---gaccatagcggg----

>_R_gnl|SRA|SRR1213690.1918180.1 HISEQ1:355:D1Y5LACXX:3:1106:11246:94611.
-----------ggcacagttgcagaagtcaatgcccagcactaccagcaggagtcctcca
aactgcgccaacaaatcagtagcttac-agaacgcaaacag---gaccatagtgag----

>_R_gnl|SRA|SRR1213690.34968901.1 HISEQ1:355:D1Y5LACXX:3:2309:17145:4848.
----------tggcacagttgcagaagtcaatgcccagcactaccagcaggagtcctcca
aactgcgccaacaaatcagtagcttac-agaacgcaaacag---gaccatagtgg-----

>_R_gnl|SRA|SRR1213690.6959351.1 HISEQ1:355:D1Y5LACXX:3:1202:12250:79164.
---------ctggcacagttgcagaagtcaatgcccagcactaccagcaggagtcctcca
aactgcgccaacaaatcagtagcttac-agaacgcaaacag---gaccatagtg------

>_R_gnl|SRA|SRR1213690.4450768.1 HISEQ1:355:D1Y5LACXX:3:1113:6951:2855.
---------ctggcacagttgcagaagtcaatgcccagcactaccagcaggagtcctcca
aactgcgccaacaaatcagtagcttac-agaacgcaaacag---gaccatagtg------

>_R_gnl|SRA|SRR1213690.7678694.1 HISEQ1:355:D1Y5LACXX:3:1204:11642:89271.
-------ctctggcacagttgcagaagtcaatgcccagcactaccagcaggagtcctcca
aactgcgccaacaaatcagtagcttac-agaacgcaaacag---gaccatag--------

>_R_gnl|SRA|SRR1213690.3726719.1 HISEQ1:355:D1Y5LACXX:3:1111:13042:40276.
-------ctctggcacagttgcagaagtcaatgcccagcactaccagcaggagtcctcca
aactgcgccaacaaatcagtagcttac-agaacgcaaacag---gaccatag--------

>_R_gnl|SRA|SRR1213690.13769869.1 HISEQ1:355:D1Y5LACXX:3:1303:5893:88153.
-------ctctggcacagttgcagaagtcaatgcccagcactaccagcaggagtcctcca
aactgcgccaacaaatcagtagcttac-agaacgcaaacagtaggacca-----------

>_R_gnl|SRA|SRR1213690.34727445.1 HISEQ1:355:D1Y5LACXX:3:2308:18940:42362.
-----aactctggcacagttgcagaagtcaatgcccagcactaccagcaggagtcctcca
aactgcgccaacaaatcagtagcttac-agaacgcaaacagtaggac-------------

>_R_gnl|SRA|SRR1213690.24988402.1 HISEQ1:355:D1Y5LACXX:3:2115:19044:92889.
-----aactctggcacagttgcagaagtcaatgcccagcactaccagcaggagtcctcca
aactgcgccaacaaatcagtagcttac-agaacgcaaacagtaggac-------------

>_R_gnl|SRA|SRR1213690.20087478.1 HISEQ1:355:D1Y5LACXX:3:2104:15889:58085.
-----aactctggcacagttgcagaagtcaatgcccagcactaccagcaggagtcctcca
aactgcgccaacaaatcagtagcttac-agaacgcaaacagtaggac-------------

>_R_gnl|SRA|SRR1213690.8355859.1 HISEQ1:355:D1Y5LACXX:3:1206:7927:76781.
-----aactctggcacagttgcagaagtcaatgcccagcactaccagcaggagtcctcca
aactgcgccaacaaatcagtagcttac-agaacgcaaacagtaggac-------------

>_R_gnl|SRA|SRR1213690.1102743.1 HISEQ1:355:D1Y5LACXX:3:1104:13605:62740.
-----aactctggcacagttgcagaagtcaatgcccagcactaccagcaggagtcctcca
aactgcgccaacaaatcagtagcttac-agaacgcaaacagtaggac-------------

>_R_gnl|SRA|SRR1213690.25258281.1 HISEQ1:355:D1Y5LACXX:3:2116:16201:42730.
-----aactctggctcagttgcagaagtcaatgcccagcactaccagcaggagtcctcca
aactgcgccaacaaatcagtagcttac-agaacgcaaacagtaggac-------------

>_R_gnl|SRA|SRR1213690.14008403.1 HISEQ1:355:D1Y5LACXX:3:1304:7460:58201.
----caactctggcacagttgcagaagtcaatgcccagcactaccagcaggagtcctcca
aactgcgccaacaaatcagtagcttac-agaacgcaaacagtagga--------------

>_R_gnl|SRA|SRR1213690.33287698.1 HISEQ1:355:D1Y5LACXX:3:2304:17291:46777.
---ccaactctggcacagttgcagaagtcaatgcccagcactaccagcaggagtcctcca
aactgcgccaacaaatcagtagcttac-agaacgcaaacagtagg---------------

>_R_gnl|SRA|SRR1213690.19516661.1 HISEQ1:355:D1Y5LACXX:3:2102:18370:85943.
---ccaactctggcacagttgcagaagtcaatgcccagcactaccagcaggagtcctcca
aactgcgccaacaaatcagtagcttac-agaacgcaaacagtagg---------------

>_R_gnl|SRA|SRR1213690.18375734.1 HISEQ1:355:D1Y5LACXX:3:1315:13834:64583.
---ccaactctggcacagttgcagaagtcaatgcccagcactaccagcaggagtcctcca
aactgcgccaacaaatcagtagcttac-agaacgcaaacagtagg---------------

>_R_gnl|SRA|SRR1213690.14006177.1 HISEQ1:355:D1Y5LACXX:3:1304:14910:57476.
---ccaactctggcacagttgcagaagtcaatgcccagcactaccagcaggagtcctcca
aactgcgccaacaaatcagtagcttac-agaacgcaaacagtagg---------------

>_R_gnl|SRA|SRR1213690.6905334.1 HISEQ1:355:D1Y5LACXX:3:1202:4244:63131.
---ccaactctggcacagttgcagaagtcaatgcccagcactaccagcaggagtcctcca
aactgcgccaacaaatcagtagcttac-agaacgcaaacagtagg---------------

>_R_gnl|SRA|SRR1213690.3281463.1 HISEQ1:355:D1Y5LACXX:3:1110:14943:37337.
---ccaactctggcacagttgcagaagtcaatgcccagcactaccagcaggagtcctcca
aactgcgccaacaaatcagtagcttac-agaacgcaaacaggacc---------------

>_R_gnl|SRA|SRR1213690.33346923.1 HISEQ1:355:D1Y5LACXX:3:2304:3284:63845.
--tccaactctggcacagttgcagaagtcaatgcccagcactaccagcaggagtcctcca
aactgcgccaacaaatcagtagcttac-agaacgcaaacagtag----------------

>_R_gnl|SRA|SRR1213690.20029785.1 HISEQ1:355:D1Y5LACXX:3:2104:16769:41647.
--tccaactctggcacagttgcagaagtcaatgcccagcactaccagcaggagtcctcca
aactgcgccaacaaatcagtagcttac-agaacgcaaacagtag----------------

>_R_gnl|SRA|SRR1213690.19265744.1 HISEQ1:355:D1Y5LACXX:3:2102:12601:6751.
--tccaactctggcacagttgcagaagtcaatgcccagcactaccagcaggagtcctcca
aactgcgccaacaaatcagtagcttac-agaacgcaaacagtag----------------

>_R_gnl|SRA|SRR1213690.18352478.1 HISEQ1:355:D1Y5LACXX:3:1315:16616:59001.
--tccaactctggcacagttgcagaagtcaatgcccagcactaccagcaggagtcctcca
aactgcgccaacaaatcagtagcttac-agaacgcaaacagtag----------------

>_R_gnl|SRA|SRR1213690.15003566.1 HISEQ1:355:D1Y5LACXX:3:1307:9018:34931.
--tccaactctggcacagttgcagaagtcaatgcccagcactaccagcaggagtcctcca
aactgcgccaacaaatcagtagcttac-agaacgcaaacagtag----------------

>_R_gnl|SRA|SRR1213690.9907404.1 HISEQ1:355:D1Y5LACXX:3:1210:2019:67643.
--tccaactctggcacagttgcagaagtcaatgcccagcactaccagcaggagtcctcca
aactgcgccaacaaatcagtagcttac-agaacgcaaacagtag----------------

>_R_gnl|SRA|SRR1213690.8909500.1 HISEQ1:355:D1Y5LACXX:3:1208:5290:22584.
--tccaactctggcacagttgcagaagtcaatgcccagcactaccagcaggagtcctcca
aactgcgccaacaaatcagtagcttac-agaacgcaaacagtag----------------

>_R_gnl|SRA|SRR1213690.8863977.1 HISEQ1:355:D1Y5LACXX:3:1208:13801:10954.
--tccaactctggcacagttgcagaagtcaatgcccagcactaccagcaggagtcctcca
aactgcgccaacaaatcagtagcttac-agaacgcaaacagtag----------------

>_R_gnl|SRA|SRR1213690.6491542.1 HISEQ1:355:D1Y5LACXX:3:1201:19328:35572.
--tccaactctggcacagttgcagaagtcaatgcccagcactaccagcaggagtcctcca
aactgcgccaacaaatcagtagcttac-agaacgcaaacagtag----------------

>_R_gnl|SRA|SRR1213690.29737806.1 HISEQ1:355:D1Y5LACXX:3:2212:16972:12800.
--tccaactctggcacagttgcagaagtcaatgcccagcactnccagcnggagtcctcca
aactgcgccaacaaatcagtagcttac-agaacgcaaacaggac----------------

>_R_gnl|SRA|SRR1213690.29569191.1 HISEQ1:355:D1Y5LACXX:3:2211:2667:74187.
--tccaactctggcacagttgcagaagtcaatgcccagcactnccagcnggagtcctcca
aactgcgccaacaaatcagtagcttac-agaacgcaaacaggac----------------

>_R_gnl|SRA|SRR1213690.24936635.1 HISEQ1:355:D1Y5LACXX:3:2115:7443:83576.
--tccaactctggcacagttgcagaagtcaatgcccagcactaccagcaggagtcctcca
aactgcgccaacaaatcagtagcttac-agaacgcaaacaggac----------------

>_R_gnl|SRA|SRR1213690.22469370.1 HISEQ1:355:D1Y5LACXX:3:2110:16302:71909.
--tccaactctggcacagttgcagaagtcaatgcccagcactaccagcaggagtcctcca
aactgcgccaacaaatcagtagcttac-agaacgcaaacaggac----------------

>_R_gnl|SRA|SRR1213690.20103246.1 HISEQ1:355:D1Y5LACXX:3:2104:19653:62675.
--tccaactctggcacagttgcagaagtcaatgcccagcactaccagcaggagtcctcca
aactgcgccaacaaatcagtagcttac-agaacgcaaacaggac----------------

>_R_gnl|SRA|SRR1213690.18806978.1 HISEQ1:355:D1Y5LACXX:3:1316:4472:66938.
--tccaactctggcacagttgcagaagtcaatgcccagcactaccagcaggagtcctcca
aactgcgccaacaaatcagtagcttac-agaacgcaaacaggac----------------

>_R_gnl|SRA|SRR1213690.17514797.1 HISEQ1:355:D1Y5LACXX:3:1313:9613:59964.
--tccaactctggcacagttgcagaagtcaatgcccagcactaccagcaggagtcctcca
aactgcgccaacaaatcagtagcttac-agaacgcaaacaggac----------------

>_R_gnl|SRA|SRR1213690.16275728.1 HISEQ1:355:D1Y5LACXX:3:1310:10166:60489.
--tccaactctggcacagttgcagaagtcaatgcccagcactaccagcaggagtcctcca
aactgcgccaacaaatcagtagcttac-agaacgcaaacaggac----------------

>_R_gnl|SRA|SRR1213690.14532251.1 HISEQ1:355:D1Y5LACXX:3:1306:5560:6965.
--tccaactctggcacagttgcagaagtcaatgcccagcactaccagcaggagtcctcca
aactgcgccaacaaatcagtagcttac-agaacgcaaacaggac----------------

>_R_gnl|SRA|SRR1213690.14376976.1 HISEQ1:355:D1Y5LACXX:3:1305:2026:63148.

--tccaactctggcacagttgcagaagtcaatgcccagcactaccagcaggagtcctcca
aactgcgccaacaaatcagtagcttac-agaacgcaaacaggac----------------

>_R_gnl|SRA|SRR1213690.10916914.1 HISEQ1:355:D1Y5LACXX:3:1212:5953:99466.
--tccaactctggcacagttgcagaagtcaatgcccagcactaccagcaggagtcctcca
aactgcgccaacaaatcagtagcttac-agaacgcaaacaggac----------------

>_R_gnl|SRA|SRR1213690.3325537.1 HISEQ1:355:D1Y5LACXX:3:1110:12594:47659.
--tccaactctggcacagttgcagaagtcaatgcccagcactaccagcaggagtcctcca
aactgcgccaacaaatcagtagcttac-agaacgcaaacaggac----------------

>_R_gnl|SRA|SRR1213690.35545782.1 HISEQ1:355:D1Y5LACXX:3:2310:20016:47785.
tctccaactctggcacagttgcagaagtcaatgcccagcactaccagcaggagtcctcca
aactgcgccaacaaatcagtagcttac-agaacgcaaacagg------------------

>_R_gnl|SRA|SRR1213690.34840050.1 HISEQ1:355:D1Y5LACXX:3:2308:9526:71030.
cctccaactctggcacagttgcagaagtcaatgcccagcactaccagcaggagtcctcca
aactgcgccaacaaatcagtagcttac-agaacgcaaacagg------------------

>_R_gnl|SRA|SRR1213690.28513387.1 HISEQ1:355:D1Y5LACXX:3:2209:13215:23716.
cctccaactctggctcagttgcagaagtcaatgcccagcantaccaccaggagtcctcca
aactgcgccaacaaatcagtagcttac-agaacgcaaacagg------------------

>_R_gnl|SRA|SRR1213690.25647558.1 HISEQ1:355:D1Y5LACXX:3:2201:7509:19866.
cctccaactctggcacagttgcagaagtcaatgcccagcattaccatcaggagtcctcca
aactgcgccaacaaatcagtagcttac-agaacgcaaacagg------------------

DRX000335
>control ..
--------------tagcttacagaac-----------gcaaacagtaggaccatagtgg
ggg----attc----------------
>_R_gnl|SRA|DRR000630.2464660.1 HWUSI-EAS1632R_0006_FC61F2Y:1:79:9342:8163..
cgccaacaaatcagt-------------------------acaaacaggaccatagtgg
ggg----attctatc------------
>gnl|SRA|DRR000630.9310868.2 HWUSI-EAS1632R_0006_FC61F2Y:3:45:6049:6044..
---caaacaggaccatagtgg
ggg----attctatc------------
>gnl|SRA|DRR000630.8659660.1 HWUSI-EAS1632R_0006_FC61F2Y:3:26:1658:20640..
---caaacaggaccatagtgg
ggg----attctatc------------
>gnl|SRA|DRR000630.486496.1 HWUSI-EAS1632R_0006_FC61F2Y:1:15:1779:8099..
---caaacaggaccatagtgg

ggg----attctatc------------
>_R_gnl|SRA|DRR000630.881789.2 HWUSI-EAS1632R_0006_FC61F2Y:1:26:16080:11755..
cgccaacgaatcagtagcttacagaac--------------gcaaacaggaccatagtgg
ggg----attctatc------------
>_R_gnl|SRA|DRR000630.4306558.1 HWUSI-EAS1632R_0006_FC61F2Y:2:15:2628:2341..
cgccaacaaatcaatagcttacagaac--------------gcaaacaggaccatagtgg
ggg----attctatc------------
>_R_gnl|SRA|DRR000630.9677119.2 HWUSI-EAS1632R_0006_FC61F2Y:3:57:3851:3737..
-----acaaatcagtagcttacagaac--------------gcaaacaggaccatagtgg
ggg----attctatc------------
>_R_gnl|SRA|DRR000630.2206491.2 HWUSI-EAS1632R_0006_FC61F2Y:1:71:3140:9856..
---caacaaatcagtagcttacagaac--------------gcaaacaggaccatagtgg
ggg----attctatc------------
>gnl|SRA|DRR000630.8661656.2 HWUSI-EAS1632R_0006_FC61F2Y:3:26:2844:17345..
------caaatcagtagcttacagaac--------------gcaaacaggaccatagtgg
ggg----attctatc------------
>_R_gnl|SRA|DRR000630.11502459.2 HWUSI-EAS1632R_0006_FC61F2Y:3:112:14370:12288..
---------ctcagtagcttacagaac--------------gcaaacaggaccatagtgg
ggg----attctatc------------
>_R_gnl|SRA|DRR000630.10666948.2 HWUSI-EAS1632R_0006_FC61F2Y:3:88:7773:13064..
---------atcagtagcttacagaac--------------gcaaacaggaccatagtgg
ggg----attctatc------------
>gnl|SRA|DRR000630.9713609.1 HWUSI-EAS1632R_0006_FC61F2Y:3:58:6578:14752..
---------atcagtagcttacagaac--------------gcaaacaggaccatagtgg
ggg----attctatc------------
>_R_gnl|SRA|DRR000630.9102171.2 HWUSI-EAS1632R_0006_FC61F2Y:3:38:19443:5469..
---------atcagtagcttacagaac--------------gcaaacaggaccatagtgg
ggg----attctatc------------
>gnl|SRA|DRR000630.11502459.1 HWUSI-EAS1632R_0006_FC61F2Y:3:112:14370:12288..
-----------cagtagcttacagaac--------------gcaaacaggaccatagtgg
ggg----attctatc------------
>_R_gnl|SRA|DRR000630.1964366.2 HWUSI-EAS1632R_0006_FC61F2Y:1:63:3181:18463..
-----------cagtagcttacagaac--------------gcaaacaggaccatagtgg
ggg----attctatc------------
>_R_gnl|SRA|DRR000630.6015746.1 HWUSI-EAS1632R_0006_FC61F2Y:2:67:18080:1772..
----------tcagtagcttacagaac--------------gcaaacaggaccatagtgg
ggg----attctatc------------
>_R_gnl|SRA|DRR000630.4637084.2 HWUSI-EAS1632R_0006_FC61F2Y:2:24:13570:15591..
----------tcagtagcttacagaac--------------gcaaacaggaccatagtgg
ggg----attctatc------------
>_R_gnl|SRA|DRR000630.4237094.2 HWUSI-EAS1632R_0006_FC61F2Y:2:13:2653:5027..
----------gcagtagctgacagaac--------------gcaaacaggaccatagtgg
ggg----attctatc------------
>_R_gnl|SRA|DRR000630.3105349.2 HWUSI-EAS1632R_0006_FC61F2Y:1:100:9640:4243..
------------agtagcttacagaac--------------gcaaacaggaccatagtgg
ggg----attctatc------------
>_R_gnl|SRA|DRR000630.4639988.2 HWUSI-EAS1632R_0006_FC61F2Y:2:24:15134:16942..
------------agtagcttacagaac--------------gcaaacagggccatagtgg
ggg----attctatc------------
>gnl|SRA|DRR000630.1071440.1 HWUSI-EAS1632R_0006_FC61F2Y:1:32:13572:9676..
--------------tagcttacagaac--------------gcaaacaggaccatagtgg

ggg----attctatc------------
>_R_gnl|SRA|DRR000630.9713609.2 HWUSI-EAS1632R_0006_FC61F2Y:3:58:6578:14752..
--------------------acagaac--------------gcaaacaggaccatagtgg
ggg----attctatc------------
>_R_gnl|SRA|DRR000630.4635277.1 HWUSI-EAS1632R_0006_FC61F2Y:2:24:12555:9101..
--------------------acagaac--------------gcaaacaggaccatagtgg
ggg----attctatc------------
>_R_gnl|SRA|DRR000630.1433901.2 HWUSI-EAS1632R_0006_FC61F2Y:1:45:9221:4925..
--------------------acagaac--------------gcaaacaggaccatagtgg
ggg----attctatc------------
>_R_gnl|SRA|DRR000630.5933618.2 HWUSI-EAS1632R_0006_FC61F2Y:2:65:6148:15922..
--cgcaaacaggaccatagtgg
ggg----attctatc------------
>gnl|SRA|DRR000630.2812035.2 HWUSI-EAS1632R_0006_FC61F2Y:1:91:11841:7649..
--cgcaaacaggaccatagtgg
ggg----attctatc------------
>gnl|SRA|DRR000630.7816258.1 HWUSI-EAS1632R_0006_FC61F2Y:3:2:2972:10625..
--cgcaaacaggaccatagtgg
ggg----attctatc------------
>_R_gnl|SRA|DRR000630.1097210.2 HWUSI-EAS1632R_0006_FC61F2Y:1:33:9779:18975..
--cgcaaacaggaccatagtgg
ggg----attctatc------------
>gnl|SRA|DRR000630.7167816.1 HWUSI-EAS1632R_0006_FC61F2Y:2:103:12185:14128..
--cgcaaacaggaccatagtgg
ggg----attctatc------------
>_R_gnl|SRA|DRR000630.7816258.2 HWUSI-EAS1632R_0006_FC61F2Y:3:2:2972:10625..
--------------------------cgctcttccgatctcgcaaacaggaccatagtgg
ggg----attctatc------------
>_R_gnl|SRA|DRR000630.7167816.2 HWUSI-EAS1632R_0006_FC61F2Y:2:103:12185:14128..
--------------ttccctacacgacgctcttccgatctcgcaaacaggaccatagtgg
ggg----attctatc------------
>_R_gnl|SRA|DRR000630.10177345.2 HWUSI-EAS1632R_0006_FC61F2Y:3:72:17877:10594..
---gcaaacaggaccatagtgg
ggg----attctatc------------
>_R_gnl|SRA|DRR000630.10997334.1 HWUSI-EAS1632R_0006_FC61F2Y:3:98:3291:4046..
-gccaacaaatcagtagcttacagaac-----------gcaaacagtaggaccatagtgg
ggg----attctatc------------
>_R_gnl|SRA|DRR000630.10249753.1 HWUSI-EAS1632R_0006_FC61F2Y:3:75:9482:5986..
-gccaacaaatcagtagcttacagaac-----------gcaaacagtaggaccatagtgg
ggg----attctatc------------
>_R_gnl|SRA|DRR000630.9317893.1 HWUSI-EAS1632R_0006_FC61F2Y:3:45:10651:12657..
-gccaacaaatcagtagcttacagaac-----------gcaaacagtaggaccatagtgg
ggg----attctatc------------
>_R_gnl|SRA|DRR000630.5242852.2 HWUSI-EAS1632R_0006_FC61F2Y:2:42:14806:4159..
-gccaacaaatcagtagcttacagaac-----------gcaaacagtaggaccatagtgg
ggg----attctatc------------
>_R_gnl|SRA|DRR000630.4888081.1 HWUSI-EAS1632R_0006_FC61F2Y:2:32:2481:11984..
-gccaacaaatcagtagcttacagaac-----------gcaaacagtaggaccatagtgg
ggg----attctatc------------
>_R_gnl|SRA|DRR000630.4363567.1 HWUSI-EAS1632R_0006_FC61F2Y:2:16:14449:9114..
-gccaacaaatcagtagcttacagaac-----------gcaaacagtaggaccatagtgg

ggg----attctatc------------
>_R_gnl|SRA|DRR000630.31978.2 HWUSI-EAS1632R_0006_FC61F2Y:1:1:17584:15109..
-gccaacaaatcagtagcttacagaac-----------gcaaacagtaggaccatagtgg
ggg----attctatc------------
>_R_gnl|SRA|DRR000630.7507400.1 HWUSI-EAS1632R_0006_FC61F2Y:2:113:9345:1979..
-gccaacaaatcagtagcttacagaac-----------gcaaacagtaggaccatagtgg
ggg----attctatc------------
>_R_gnl|SRA|DRR000630.292458.2 HWUSI-EAS1632R_0006_FC61F2Y:1:9:7609:17891..
-gccaacaaatcagtagcttacagaac-----------gcaagtagtaggaccatagtgg
ggg----attctatc------------
>_R_gnl|SRA|DRR000630.1932007.1 HWUSI-EAS1632R_0006_FC61F2Y:1:62:1290:1672..
-gcaaacaaatcagtagcttacagaac-----------gcaaacagtaggaccatagtgg
ggg----attctatc------------
>gnl|SRA|DRR000630.8724457.1 HWUSI-EAS1632R_0006_FC61F2Y:3:27:17960:15475..
--ccaacaaatcagtagcttacagaac-----------gcaaacagtaggaccatagtgg
ggg----attctatc------------
>_R_gnl|SRA|DRR000630.4902330.1 HWUSI-EAS1632R_0006_FC61F2Y:2:32:10118:3099..
--ccaacaaatcagtagcttacagaac-----------gcaaacagtaggaccatagtgg
ggg----attctatc------------
>_R_gnl|SRA|DRR000630.8364371.2 HWUSI-EAS1632R_0006_FC61F2Y:3:17:10582:3034..
--ccaacaaatcagtagcttacagaac-----------gcaaacagtaggaccatagtgg
ggg----attctatc------------
>_R_gnl|SRA|DRR000630.7720330.1 HWUSI-EAS1632R_0006_FC61F2Y:2:119:9343:8259..
---caacaaatcagtagcttacagaac-----------gcaaacagtaggaccatagtgg
ggg----attctatc------------
>_R_gnl|SRA|DRR000630.5490781.2 HWUSI-EAS1632R_0006_FC61F2Y:2:50:19756:5795..
-----acaaatcagtagcttacagaac-----------gcaaacagtaggaccatagtgg
ggg----attctatc------------
>_R_gnl|SRA|DRR000630.10778815.2 HWUSI-EAS1632R_0006_FC61F2Y:3:91:13880:3984..
------caaatcagtagcttacagaac-----------gcaaacagtaggaccatagtgg
ggg----attctatc------------
>_R_gnl|SRA|DRR000630.8817742.2 HWUSI-EAS1632R_0006_FC61F2Y:3:30:12467:17763..
--------aatcagtagcttacagaac-----------gcaaacagtaggaccatagtgg
ggg----attctatc------------
>_R_gnl|SRA|DRR000630.6237572.1 HWUSI-EAS1632R_0006_FC61F2Y:2:75:3717:2080..
--------aatcagtagcttacagaac-----------gcaaacagtaggaccatagtgg
ggg----attctatc------------
>_R_gnl|SRA|DRR000630.9122084.2 HWUSI-EAS1632R_0006_FC61F2Y:3:39:11845:11437..
---------atcagtagcttacagaac-----------gcaaacagtaggaccatagtgg
ggg----attctatc------------
>_R_gnl|SRA|DRR000630.2615961.2 HWUSI-EAS1632R_0006_FC61F2Y:1:85:11514:6993..
-----------cagtagcttacagaac-----------gcaaacagtaggaccatagtgg
ggg----attctatc------------
>_R_gnl|SRA|DRR000630.8657894.1 HWUSI-EAS1632R_0006_FC61F2Y:3:25:19297:10430..
------------agtagcttacagaac-----------gcaaacagtaggaccatagtgg
ggg----attctatc------------
>_R_gnl|SRA|DRR000630.6580040.2 HWUSI-EAS1632R_0006_FC61F2Y:2:86:2571:16996..
------------agtagcttacagaac-----------gcaaacagtaggaccatagtgg
ggg----attctatc------------
>_R_gnl|SRA|DRR000630.2098650.2 HWUSI-EAS1632R_0006_FC61F2Y:1:67:11730:19315..
------------agtagcttacagaac-----------gcaaacagtaggaccatagtgg

ggg----attctatc------------
>_R_gnl|SRA|DRR000630.1228161.1 HWUSI-EAS1632R_0006_FC61F2Y:1:37:10783:10876..
------------agtagcttacataac-----------gcaaacagtaggaccatagtgg
ggg----attctatc------------
>_R_gnl|SRA|DRR000630.8197537.1 HWUSI-EAS1632R_0006_FC61F2Y:3:12:15444:18464..
-------------gtagcttacagaac-----------gcaaacagtaggaccatagtgg
ggg----attctatc------------
>_R_gnl|SRA|DRR000630.5484559.2 HWUSI-EAS1632R_0006_FC61F2Y:2:50:16093:10341..
-------------gtagcttacagaac-----------gcaaacagtaggaccatagtgg
ggg----attctatc------------
>_R_gnl|SRA|DRR000630.3574593.2 HWUSI-EAS1632R_0006_FC61F2Y:1:114:6870:11999..
-------------gtagcttacagaac-----------gcaaacagtaggaccatagtgg
ggg----attctatc------------
>_R_gnl|SRA|DRR000630.10868929.2 HWUSI-EAS1632R_0006_FC61F2Y:3:94:7751:15428..
-------------ggagcttacagaac-----------gcaaacagtaggaccatagtgg
ggg----attctatc------------
>_R_gnl|SRA|DRR000630.7112462.2 HWUSI-EAS1632R_0006_FC61F2Y:2:101:19403:1795..
-------------gtgacttacagaac-----------acaaacattaggaccatagtgg
ggg----attctatc------------
>_R_gnl|SRA|DRR000630.10977299.1 HWUSI-EAS1632R_0006_FC61F2Y:3:97:10832:12057..
---------------agcttacagaac-----------gcaaacagtaggaccatagtgg
ggg----attctatc------------
>gnl|SRA|DRR000630.2499889.2 HWUSI-EAS1632R_0006_FC61F2Y:1:80:17511:18614..
---------------agcttacagaac-----------gcaaacagtaggaccatagtgg
ggg----attctatc------------
>gnl|SRA|DRR000630.7442198.2 HWUSI-EAS1632R_0006_FC61F2Y:2:111:11483:11588..
---------------agcttacagaac-----------gcaaacagtaggaccatagtgg
ggg----attctatc------------
>_R_gnl|SRA|DRR000630.7442198.1 HWUSI-EAS1632R_0006_FC61F2Y:2:111:11483:11588..
gctcttccgatc--tagcttacagaac-----------gcaaacagtaggaccatagtgg
ggg----attctatc------------
>_R_gnl|SRA|DRR000630.2296658.1 HWUSI-EAS1632R_0006_FC61F2Y:1:74:1882:14512..
-------------------tacagaac-----------gcaaacagtaggaccatagtgg
ggg----attctatc------------
>gnl|SRA|DRR000630.9894501.2 HWUSI-EAS1632R_0006_FC61F2Y:3:64:1367:3026..
---------------------cagaac-----------gcaaacagtaggaccatagtgg
ggg----attctatc------------
>gnl|SRA|DRR000630.4307832.2 HWUSI-EAS1632R_0006_FC61F2Y:2:15:3332:19751..
-----------------------gaac-----------gcaaacagtaggaccatagtgg
ggg----attctatc------------
>_R_gnl|SRA|DRR000630.4307832.1 HWUSI-EAS1632R_0006_FC61F2Y:2:15:3332:19751..
----------------ccgatctgaac-----------gcaaacagtaggaccatagtgg
ggg----attctatc------------
>_R_gnl|SRA|DRR000630.8652881.1 HWUSI-EAS1632R_0006_FC61F2Y:3:25:16573:13823..
------------------------aac-----------gcaaacagtaggaccatagtgg
ggg----attctatc------------
>gnl|SRA|DRR000630.7758024.1 HWUSI-EAS1632R_0006_FC61F2Y:2:120:10047:8020..
------------------------aac-----------gcaaacagtaggaccatagtgg
ggg----attctatc------------
>_R_gnl|SRA|DRR000630.11261515.1 HWUSI-EAS1632R_0006_FC61F2Y:3:105:16051:17964..
-------------------------ac-----------gcaaacagtaggaccatagtgg

ggg----attctatc------------
>gnl|SRA|DRR000630.11260559.1 HWUSI-EAS1632R_0006_FC61F2Y:3:105:15533:8853..
--------------------------c-----------gcaaacagtaggaccatagtgg
ggg----attctatc------------
>gnl|SRA|DRR000630.9293986.1 HWUSI-EAS1632R_0006_FC61F2Y:3:44:14712:2762..
--------------------------c-----------gcaaacagtaggaccatagtgg
ggg----attctatc------------
>gnl|SRA|DRR000630.6181687.2 HWUSI-EAS1632R_0006_FC61F2Y:2:73:4549:4309..
--------------------------c-----------gcaaacagtaggaccatagtgg
ggg----attctatc------------
>gnl|SRA|DRR000630.5690503.1 HWUSI-EAS1632R_0006_FC61F2Y:2:57:7323:12780..
--------------------------c-----------gcaaacagtaggaccatagtgg
ggg----attctatc------------
>gnl|SRA|DRR000630.1484795.2 HWUSI-EAS1632R_0006_FC61F2Y:1:47:2429:19064..
--------------------------c-----------gcaaacagtaggaccatagtgg
ggg----attctatc------------
>_R_gnl|SRA|DRR000630.10173023.1 HWUSI-EAS1632R_0006_FC61F2Y:3:72:15301:17685..
--------------------------------------gcaaacagtaggaccatagtgg
ggg----attctatc------------
>gnl|SRA|DRR000630.8375516.2 HWUSI-EAS1632R_0006_FC61F2Y:3:17:16391:4080..
--------------------------------------gcaaacagtaggaccatagtgg
ggg----attctatc------------
>gnl|SRA|DRR000630.7156504.2 HWUSI-EAS1632R_0006_FC61F2Y:2:103:6130:2566..
--------------------------------------gcaaacagtaggaccatagtgg
ggg----attctatc------------
>_R_gnl|SRA|DRR000630.1484795.1 HWUSI-EAS1632R_0006_FC61F2Y:1:47:2429:19064..
------cagacgtgtgctcttccgatct---------cgcaaacagtaggaccatagtgg
ggg----attctatc------------
>_R_gnl|SRA|DRR000630.8375516.1 HWUSI-EAS1632R_0006_FC61F2Y:3:17:16391:4080..
------cagacgtgtgctcttccgatct----------gcaaacagtaggaccatagtgg
ggg----attctatc------------
>_R_gnl|SRA|DRR000630.7156504.1 HWUSI-EAS1632R_0006_FC61F2Y:2:103:6130:2566..
------cagacgtgtgctcttccgatct----------gcaaacagtaggaccatagtgg
ggg----attctatc------------
>_R_gnl|SRA|DRR000630.11260559.2 HWUSI-EAS1632R_0006_FC61F2Y:3:105:15533:8853..
---------------------------------------caaacagtaggaccatagtgg
ggg----attctatc------------
>_R_gnl|SRA|DRR000630.9790309.2 HWUSI-EAS1632R_0006_FC61F2Y:3:60:14322:19538..
---------------------------------------caaacagtaggaccatagtgg
ggg----attctatc------------
>gnl|SRA|DRR000630.8469353.2 HWUSI-EAS1632R_0006_FC61F2Y:3:20:10965:8730..
---------------------------------------caaacagtaggaccatagtgg
ggg----attctatc------------
>_R_gnl|SRA|DRR000630.7316653.2 HWUSI-EAS1632R_0006_FC61F2Y:2:107:18416:19317..
---------------------------------------caaacagtaggaccatagtgg
ggg----attctatc------------
>gnl|SRA|DRR000630.7245722.2 HWUSI-EAS1632R_0006_FC61F2Y:2:105:17366:4459..
---------------------------------------caaacagtaggaccatagtgg
ggg----attctatc------------
>_R_gnl|SRA|DRR000630.6842364.2 HWUSI-EAS1632R_0006_FC61F2Y:2:93:19725:12499..
---------------------------------------caaacagtaggaccatagtgg

ggg----attctatc------------
>gnl|SRA|DRR000630.4705142.1 HWUSI-EAS1632R_0006_FC61F2Y:2:26:13424:14213..
--caaacagtaggaccatagtgg
ggg----attctatc------------
>gnl|SRA|DRR000630.3711011.2 HWUSI-EAS1632R_0006_FC61F2Y:1:118:5367:5407..
--caaacagtaggaccatagtgg
ggg----attctatc------------
>_R_gnl|SRA|DRR000630.3291247.1 HWUSI-EAS1632R_0006_FC61F2Y:1:105:19671:16732..
--caaacagtaggaccatagtgg
ggg----attctatc------------
>_R_gnl|SRA|DRR000630.2683846.2 HWUSI-EAS1632R_0006_FC61F2Y:1:87:13165:17997..
--caaacagtaggaccatagtgg
ggg----attctatc------------
>gnl|SRA|DRR000630.356974.1 HWUSI-EAS1632R_0006_FC61F2Y:1:11:5467:12555..
--caaacagtaggaccatagtgg
ggg----attctatc------------
>_R_gnl|SRA|DRR000630.16066.2 HWUSI-EAS1632R_0006_FC61F2Y:1:1:9646:20356..
--caaacagtaggaccatagtgg
ggg----attctatc------------
>_R_gnl|SRA|DRR000630.10508003.2 HWUSI-EAS1632R_0006_FC61F2Y:3:83:12115:16436..
---aaacagtaggaccatagtgg
ggg----attctatc------------
>_R_gnl|SRA|DRR000630.9894501.1 HWUSI-EAS1632R_0006_FC61F2Y:3:64:1367:3026..
---aaacagtaggaccatagtgg
ggg----attctatc------------
>_R_gnl|SRA|DRR000630.2785448.1 HWUSI-EAS1632R_0006_FC61F2Y:1:90:15090:6985..
---aaacagtaggaccatagtgg
ggg----attctatc------------
>_R_gnl|SRA|DRR000630.2499889.1 HWUSI-EAS1632R_0006_FC61F2Y:1:80:17511:18614..
---aaacagtaggaccatagtgg
ggg----attctatc------------
>_R_gnl|SRA|DRR000630.1590056.1 HWUSI-EAS1632R_0006_FC61F2Y:1:50:10670:4180..
--aacagtaggaccatagtgg
ggg----attctatc------------
>_R_gnl|SRA|DRR000630.618668.1 HWUSI-EAS1632R_0006_FC61F2Y:1:18:18645:14548..
--aacagtaggaccatagtgg
ggg----attctatc------------
>_R_gnl|SRA|DRR000630.4941571.1 HWUSI-EAS1632R_0006_FC61F2Y:2:33:13172:10646..
--aacagtaggaccatagtgg
ggg----attctatc------------
>_R_gnl|SRA|DRR000630.9862621.2 HWUSI-EAS1632R_0006_FC61F2Y:3:63:1309:6796..
--cagtaggaccatagtgg
ggg----attctatc------------
>_R_gnl|SRA|DRR000630.10764883.2 HWUSI-EAS1632R_0006_FC61F2Y:3:91:6313:2561..
---agtaggaccatagtgg
ggg----attctatc------------
>gnl|SRA|DRR000630.5225074.2 HWUSI-EAS1632R_0006_FC61F2Y:2:42:4893:5620..
---agtaggaccatagtgg
ggg----attctatc------------
>gnl|SRA|DRR000630.5991282.2 HWUSI-EAS1632R_0006_FC61F2Y:2:67:3556:11658..
---agtaggaccatagtgg

ggg----attctatc------------
>gnl|SRA|DRR000630.1751916.2 HWUSI-EAS1632R_0006_FC61F2Y:1:55:16931:4737..
---aacaggaccatagtgg
ggg----attctatc------------
>_R_gnl|SRA|DRR000630.5225074.1 HWUSI-EAS1632R_0006_FC61F2Y:2:42:4893:5620..
-----------------------gatc-----------------tagtaggaccatagtgg
ggg----attctatc------------
>_R_gnl|SRA|DRR000630.5991282.1 HWUSI-EAS1632R_0006_FC61F2Y:2:67:3556:11658..
-------agacgtgtgctcttccgatc-----------------tagtaggaccatagtgg
ggg----attctatc------------
>gnl|SRA|DRR000630.10612157.2 HWUSI-EAS1632R_0006_FC61F2Y:3:86:14352:13740..
--gtaggaccatagtgg
ggg----attctatc------------
>_R_gnl|SRA|DRR000630.9627587.1 HWUSI-EAS1632R_0006_FC61F2Y:3:55:11836:11252..
--gtaggaccatagtgg
ggg----attctatc------------
>_R_gnl|SRA|DRR000630.8469353.1 HWUSI-EAS1632R_0006_FC61F2Y:3:20:10965:8730..
--gtaggaccatagtgg
ggg----attctatc------------
>gnl|SRA|DRR000630.3051020.1 HWUSI-EAS1632R_0006_FC61F2Y:1:98:16372:10414..
--gtaggaccatagtgg
ggg----attctatc------------
>gnl|SRA|DRR000630.373846.2 HWUSI-EAS1632R_0006_FC61F2Y:1:11:14472:12349..
--gtaggaccatagtgg
ggg----attctatc------------
>_R_gnl|SRA|DRR000630.11388249.1 HWUSI-EAS1632R_0006_FC61F2Y:3:109:9533:12000..
--acaggaccatagtgg
ggg----attctatc------------
>_R_gnl|SRA|DRR000630.4802901.1 HWUSI-EAS1632R_0006_FC61F2Y:2:29:11482:2461..
--gtaggaccatagtgg
ggg----atactatc------------
>_R_gnl|SRA|DRR000630.7674136.2 HWUSI-EAS1632R_0006_FC61F2Y:2:118:4182:17750..
--tctggaccatagtgg
ggg----attctatc------------
>_R_gnl|SRA|DRR000630.10612157.1 HWUSI-EAS1632R_0006_FC61F2Y:3:86:14352:13740..
--------------------------tct---------tccgatctgtaggaccatagtgg
ggg----attctatc------------
>gnl|SRA|DRR000630.3000649.1 HWUSI-EAS1632R_0006_FC61F2Y:1:97:6750:13043..
--aggaccatagtgg
ggg----attctatc------------
>gnl|SRA|DRR000630.10697745.2 HWUSI-EAS1632R_0006_FC61F2Y:3:89:6327:2129..
---caggaccatagtgg
ggg----attctatc------------
>gnl|SRA|DRR000630.9468395.2 HWUSI-EAS1632R_0006_FC61F2Y:3:50:13195:9529..
---caggaccatagtgg
ggg----attctatc------------
>gnl|SRA|DRR000630.6075671.1 HWUSI-EAS1632R_0006_FC61F2Y:2:69:16198:6939..
---caggaccatagtgg
ggg----attctatc------------
>gnl|SRA|DRR000630.6856086.1 HWUSI-EAS1632R_0006_FC61F2Y:2:94:8408:2136..
---caggaccatagtgg

ggg----attctatc------------
>gnl|SRA|DRR000630.3361208.1 HWUSI-EAS1632R_0006_FC61F2Y:1:108:2408:14237..
--catgaccatagtgg
ggg----attctatc------------
>_R_gnl|SRA|DRR000630.6075671.2 HWUSI-EAS1632R_0006_FC61F2Y:2:69:16198:6939..
----------------------------ctc------ttccgatctcaggaccatagtgg
ggg----attctatc------------
>_R_gnl|SRA|DRR000630.6856086.2 HWUSI-EAS1632R_0006_FC61F2Y:2:94:8408:2136..
------------ctttccctacacgacgctc------ttccgatctcaggaccatagtgg
ggg----attctatc------------
>gnl|SRA|DRR000630.10424744.1 HWUSI-EAS1632R_0006_FC61F2Y:3:81:2962:8372..
--ggaccatagtgg
ggg----attctatc------------
>gnl|SRA|DRR000630.7674136.1 HWUSI-EAS1632R_0006_FC61F2Y:2:118:4182:17750..
--ggaccatagtgg
ggg----attctatc------------
>gnl|SRA|DRR000630.6605819.1 HWUSI-EAS1632R_0006_FC61F2Y:2:86:16929:14034..
--ggaccatagtgg
ggg----attctatc------------
>gnl|SRA|DRR000630.4415216.1 HWUSI-EAS1632R_0006_FC61F2Y:2:18:5168:7130..
--ggaccatagtgg
ggg----attctatc------------
>gnl|SRA|DRR000630.4160505.1 HWUSI-EAS1632R_0006_FC61F2Y:2:10:17643:12148..
--ggaccatagtgg
ggg----attctatc------------
>_R_gnl|SRA|DRR000630.3000649.2 HWUSI-EAS1632R_0006_FC61F2Y:1:97:6750:13043..
----------------ccctacacgacgctc-------ttccgatctaggaccatagtgg
ggg----attctatc------------
>_R_gnl|SRA|DRR000630.10072475.1 HWUSI-EAS1632R_0006_FC61F2Y:3:69:13012:20313..
---gaccatagtgg
ggg----attctatc------------
>gnl|SRA|DRR000630.4993803.1 HWUSI-EAS1632R_0006_FC61F2Y:2:35:5084:15427..
--accatagtgg
ggg----attctatc------------
>gnl|SRA|DRR000630.10177345.1 HWUSI-EAS1632R_0006_FC61F2Y:3:72:17877:10594..
---gcaaacaggaccatagtgg
ggg----attctatcaacaccatgagc
>_R_gnl|SRA|DRR000630.5417645.1 HWUSI-EAS1632R_0006_FC61F2Y:2:48:14457:15363..
cgccaacaaatcagtagcttacagaac-----------gcaaacagtaggaccatagtgg
ggg----attc----------------
>_R_gnl|SRA|DRR000630.5790900.2 HWUSI-EAS1632R_0006_FC61F2Y:2:60:12390:15430..
cgccaacaaatcagtaggttacagaac-----------gcaaacagtaggaccatagtgg
ggg----attc----------------
>_R_gnl|SRA|DRR000630.9746823.1 HWUSI-EAS1632R_0006_FC61F2Y:3:59:7310:7275..
cgccaacaaatcagtagcttacagaac-----------gcaaacagtaggaccatagtgg
ggg----attctat-------------
>_R_gnl|SRA|DRR000630.4936788.2 HWUSI-EAS1632R_0006_FC61F2Y:2:33:10496:18829..
cgccaacaaatcagtagcttacagaac-----------gcaaacagtaggaccatagtgg
ggg----attctat-------------
>_R_gnl|SRA|DRR000630.99774.1 HWUSI-EAS1632R_0006_FC61F2Y:1:3:15958:5543..
cgccaacaaatcagtagcttacagaac-----------gcaaacagtaggaccatagtgg

ggg----attctat-------------
>_R_gnl|SRA|DRR000630.2917864.1 HWUSI-EAS1632R_0006_FC61F2Y:1:94:15839:18494..
cgccaacaaatcagtagcttacagaac-----------gcaaacagtaggaccatagtgg
ggg----attcta--------------
>gnl|SRA|DRR000630.8817742.1 HWUSI-EAS1632R_0006_FC61F2Y:3:30:12467:17763..
cgccaacaaatcagtagcttacagaac-----------gcaaacagtaggaccatagtgg
ggg----attctatc------------
>_R_gnl|SRA|DRR000630.8716753.1 HWUSI-EAS1632R_0006_FC61F2Y:3:27:13813:7161..
cgccaacaaatcagtagcttacagaac-----------gcaaacagtaggaccatagtgg
ggg----attctatc------------
>gnl|SRA|DRR000630.4941571.2 HWUSI-EAS1632R_0006_FC61F2Y:2:33:13172:10646..
cgccaacaaatcagtagcttacagaac-----------gcaaacagtaggaccatagtgg
ggg----attctatc------------
>_R_gnl|SRA|DRR000630.11739233.1 HWUSI-EAS1632R_0006_FC61F2Y:3:119:7469:6512..
cgccaacaaatcagtagcttacagaac-----------gcaaacagtaggaccatagtgg
ggg----attctatc------------
>_R_gnl|SRA|DRR000630.11690585.1 HWUSI-EAS1632R_0006_FC61F2Y:3:118:1433:8968..
cgccaacaaatcagtagcttacagaac-----------gcaaacagtaggaccatagtgg
ggg----attctatc------------
>_R_gnl|SRA|DRR000630.11006598.2 HWUSI-EAS1632R_0006_FC61F2Y:3:98:8111:6715..
cgccaacaaatcagtagcttacagaac-----------gcaaacagtaggaccatagtgg
ggg----attctatc------------
>_R_gnl|SRA|DRR000630.5695937.1 HWUSI-EAS1632R_0006_FC61F2Y:2:57:10490:1974..
cgccaacaaatcagtagcttacagaac-----------gcaaacagtaggaccatagtgg
ggg----attctatc------------
>_R_gnl|SRA|DRR000630.2665441.2 HWUSI-EAS1632R_0006_FC61F2Y:1:87:2766:8935..
cgccaacaaatcagtagcttacagaac-----------gcaaacagtaggaccatagtgg
ggg----attctatc------------
>gnl|SRA|DRR000630.11690585.2 HWUSI-EAS1632R_0006_FC61F2Y:3:118:1433:8968..
cgccaacaaatcagtagcttacagaac-----------gcaaacagtaggaccatagtgg
ggg----attctatc------------
>gnl|SRA|DRR000630.10977299.2 HWUSI-EAS1632R_0006_FC61F2Y:3:97:10832:12057..
cgccaacaaatcagtagcttacagaac-----------gcaaacagtaggaccatagtgg
ggg----attctatc------------
>_R_gnl|SRA|DRR000630.6978719.1 HWUSI-EAS1632R_0006_FC61F2Y:2:98:1937:13491..
cgccaacaaatcagtagcttacagaac-----------gcaaacagtaggaccatagtgg
ggg----attctatc------------
>_R_gnl|SRA|DRR000630.2662431.2 HWUSI-EAS1632R_0006_FC61F2Y:1:87:1077:19380..
cgccaacaaatcagtagcttacagaac-----------gcaaacagtaggaccatagtgg
ggg----attctatc------------
>_R_gnl|SRA|DRR000630.8274105.2 HWUSI-EAS1632R_0006_FC61F2Y:3:14:18600:11363..
cgccaacaaatcagtagcttacagaac-----------gcaaacagtaggaccatagtgg
ggg----attctatc------------
>gnl|SRA|DRR000630.3945647.2 HWUSI-EAS1632R_0006_FC61F2Y:2:4:14595:9363..
cgccaacaaatcagtagcttacagaac-----------gcaaacagtaggaccatagtgg
ggg----attctatc------------
>_R_gnl|SRA|DRR000630.3558249.2 HWUSI-EAS1632R_0006_FC61F2Y:1:113:16338:16724..
cgccaacaaatcagtagcttacagaac-----------gcaaacagtaggaccatagtgg
ggg----attctatc------------
>gnl|SRA|DRR000630.9627587.2 HWUSI-EAS1632R_0006_FC61F2Y:3:55:11836:11252..
cgccaacaaatcagtagcttacagaac-----------gcaaacagtaggaccatagtgg

ggg----attctatc------------
>gnl|SRA|DRR000630.4902330.2 HWUSI-EAS1632R_0006_FC61F2Y:2:32:10118:3099..
cgccaacaaatcagtagcttacagaac-----------gcaaacagtaggaccatagtgg
ggg----attctatc------------
>gnl|SRA|DRR000630.9317893.2 HWUSI-EAS1632R_0006_FC61F2Y:3:45:10651:12657..
cgccaacaaatcagtagcttacagaac-----------gcaaacagtaggaccatagtgg
ggg----attctatc------------
>gnl|SRA|DRR000630.4185074.1 HWUSI-EAS1632R_0006_FC61F2Y:2:11:11924:11626..
cgccaacaaatcagtagcttacagaac-----------gcaaacagtaggaccatagtgg
ggg----attctatc------------
>gnl|SRA|DRR000630.4888081.2 HWUSI-EAS1632R_0006_FC61F2Y:2:32:2481:11984..
cgccaacaaatcagtagcttacagaac-----------gcaaacagtaggaccatagtgg
ggg----attctatc------------
>_R_gnl|SRA|DRR000630.8094791.2 HWUSI-EAS1632R_0006_FC61F2Y:3:9:16919:16645..
cgccaacaaatcagtagcttacagaac-----------gcaaacagtaggaccatagtgg
ggg----attctatc------------
>_R_gnl|SRA|DRR000630.1410535.1 HWUSI-EAS1632R_0006_FC61F2Y:1:44:13761:3601..
cgccaacaaatcagtagcttacagaac-----------gcaaacagtaggaccatagtgg
ggg----attctatc------------
>gnl|SRA|DRR000630.6405229.2 HWUSI-EAS1632R_0006_FC61F2Y:2:80:14279:5249..
cgccaacaaatcagtagcttacagaac-----------gcaaacagtaggaccatagtgg
ggg----attctatc------------
>gnl|SRA|DRR000630.1877555.2 HWUSI-EAS1632R_0006_FC61F2Y:1:60:3684:4865..
cgccaacaaatcagtagcttacagaac-----------gcaaacagtaggaccatagtgg
ggg----attctatc------------
>gnl|SRA|DRR000630.617843.1 HWUSI-EAS1632R_0006_FC61F2Y:1:18:18207:4155..
cgccaacaaatcagtagcttacagaac-----------gcaaacagtaggaccatagtgg
ggg----attctatc------------
>_R_gnl|SRA|DRR000630.5380665.1 HWUSI-EAS1632R_0006_FC61F2Y:2:47:9801:13003..
cgccaacaaatcagtagcttacagaac-----------gcaaacagtaggaccatagtgg
ggg----attctatc------------
>gnl|SRA|DRR000630.11624595.1 HWUSI-EAS1632R_0006_FC61F2Y:3:116:4234:5908..
cgccaacaaatcagtagcttacagaac-----------gcaaacagtaggaccatagtgg
ggg----attctatc------------
>_R_gnl|SRA|DRR000630.646898.1 HWUSI-EAS1632R_0006_FC61F2Y:1:19:15586:7862..
cgccaacaaatcagtagcttacagaac-----------gcaaacagtaggaccatagtgg
ggg----attctatc------------
>gnl|SRA|DRR000630.9945922.2 HWUSI-EAS1632R_0006_FC61F2Y:3:65:13022:15898..
cgccaacaaatcagtagcttacagaac-----------gcaaacagtaggaccatagtgg
ggg----attctatc------------
>gnl|SRA|DRR000630.7720330.2 HWUSI-EAS1632R_0006_FC61F2Y:2:119:9343:8259..
cgccaacaaatcagtagcttacagaac-----------gcaaacagtaggaccatagtgg
ggg----attctatc------------
>gnl|SRA|DRR000630.7564150.2 HWUSI-EAS1632R_0006_FC61F2Y:2:115:2325:14891..
cgccaacaaatcagtagcttacagaac-----------gcaaacagtaggaccatagtgg
ggg----attctatc------------
>gnl|SRA|DRR000630.5430607.1 HWUSI-EAS1632R_0006_FC61F2Y:2:49:3656:3259..
cgccaacaaatcagtagcttacagaac-----------gcaaacagtaggaccatagtgg
ggg----attctatc------------
>gnl|SRA|DRR000630.1228161.2 HWUSI-EAS1632R_0006_FC61F2Y:1:37:10783:10876..
cgccaacaaatcagtagcttacagaac-----------gcaaacagtaggaccatagtgg

ggg----attctatc------------
>gnl|SRA|DRR000630.9862621.1 HWUSI-EAS1632R_0006_FC61F2Y:3:63:1309:6796..
cgccaacaaatcagtagcttacagaac-----------gcaaacagtaggaccatagtgg
ggg----attctatc------------
>_R_gnl|SRA|DRR000630.3667325.2 HWUSI-EAS1632R_0006_FC61F2Y:1:116:19714:7508..
cgccaacaaatcagtagcttacagaac-----------gcaaacagtaggaccatagtgg
ggg----attctatc------------
>_R_gnl|SRA|DRR000630.2067573.2 HWUSI-EAS1632R_0006_FC61F2Y:1:66:11296:1786..
cgccaacaaatcagtagcttacagaac-----------gcaaacagtaggaccatagtgg
ggg----attctatc------------
>_R_gnl|SRA|DRR000630.9384554.1 HWUSI-EAS1632R_0006_FC61F2Y:3:48:2476:15348..
cgccaacaaatcagtagcttacagaac-----------gcaaacagtaggaccatagtgg
ggg----attctatc------------
>_R_gnl|SRA|DRR000630.3129176.2 HWUSI-EAS1632R_0006_FC61F2Y:1:101:4326:15177..
cgccaacaaatcagtagcttacagaac-----------gcaaacagtaggaccatagtgg
ggg----attctatc------------
>gnl|SRA|DRR000630.8960348.1 HWUSI-EAS1632R_0006_FC61F2Y:3:34:15877:3491..
cgccaacaaatcagtagcttacagaac-----------gcaaacagtaggaccatagtgg
ggg----attctatc------------
>gnl|SRA|DRR000630.6122173.2 HWUSI-EAS1632R_0006_FC61F2Y:2:71:6593:9216..
cgccaacaaatcagtagcttacagaac-----------gcaaacagtaggaccatagtgg
ggg----attctatc------------
>gnl|SRA|DRR000630.497313.1 HWUSI-EAS1632R_0006_FC61F2Y:1:15:7750:19619..
cgccaacaaatcagtagcttacagaac-----------gcaaacagtaggaccatagtgg
ggg----attctatc------------
>gnl|SRA|DRR000630.7316653.1 HWUSI-EAS1632R_0006_FC61F2Y:2:107:18416:19317..
cgccaacaaatcagtagcttacagaac-----------gcaaacagtaggaccatagtgg
ggg----attctatc------------
>gnl|SRA|DRR000630.3558249.1 HWUSI-EAS1632R_0006_FC61F2Y:1:113:16338:16724..
cgccaacaaatcagtagcttacagaac-----------gcaaacagtaggaccatagtgg
ggg----attctatc------------
>gnl|SRA|DRR000630.2665441.1 HWUSI-EAS1632R_0006_FC61F2Y:1:87:2766:8935..
cgccaacaaatcagtagcttacagaac-----------gcaaacagtaggaccatagtgg
ggg----attctatc------------
>gnl|SRA|DRR000630.9122084.1 HWUSI-EAS1632R_0006_FC61F2Y:3:39:11845:11437..
cgccaacaaatcagtagcttacagaac-----------gcaaacagtaggaccatagtgg
ggg----attctatc------------
>_R_gnl|SRA|DRR000630.5166315.2 HWUSI-EAS1632R_0006_FC61F2Y:2:40:8681:2585..
cgccaacaaatcagtagcttacagaac-----------gcaaacagtaggaccatagtgg
ggg----attctatc------------
>_R_gnl|SRA|DRR000630.1922478.2 HWUSI-EAS1632R_0006_FC61F2Y:1:61:13651:16779..
cgccaacaaatcagtagcttacagaac-----------gcaaacagtaggaccatagtgg
ggg----attctatc------------
>gnl|SRA|DRR000630.5166315.1 HWUSI-EAS1632R_0006_FC61F2Y:2:40:8681:2585..
cgccaacaaatcagtagcttacagaac-----------gcaaacagtaggaccatagtgg
ggg----attctatc------------
>gnl|SRA|DRR000630.1922478.1 HWUSI-EAS1632R_0006_FC61F2Y:1:61:13651:16779..
cgccaacaaatcagtagcttacagaac-----------gcaaacagtaggaccatagtgg
ggg----attctatc------------
>gnl|SRA|DRR000630.10072475.2 HWUSI-EAS1632R_0006_FC61F2Y:3:69:13012:20313..
cgccaacaaatcagtagcttacagaac-----------gcaaacagtaggaccatagtgg

ggg----attctatc------------
>_R_gnl|SRA|DRR000630.8189386.2 HWUSI-EAS1632R_0006_FC61F2Y:3:12:11241:20767..
cgccaacaaatcagtagcttacagaac-----------gcaaacagtaggaccatagtgg
ggg----attctatc------------
>_R_gnl|SRA|DRR000630.6846013.1 HWUSI-EAS1632R_0006_FC61F2Y:2:94:3007:3848..
cgccaacaaatcagtagcttacagaac-----------gcaaacagtaggaccatagtgg
ggg----attctatc------------
>_R_gnl|SRA|DRR000630.5430607.2 HWUSI-EAS1632R_0006_FC61F2Y:2:49:3656:3259..
cgccaacaaatcagtagcttacagaac-----------gcaaacagtaggaccatagtgg
ggg----attctatc------------
>_R_gnl|SRA|DRR000630.5350039.1 HWUSI-EAS1632R_0006_FC61F2Y:2:46:6108:8061..
cgccaacaaatcagtagcttacagaac-----------gcaaacagtaggaccatagtgg
ggg----attctatc------------
>_R_gnl|SRA|DRR000630.585279.2 HWUSI-EAS1632R_0006_FC61F2Y:1:17:18741:3139..
cgccaacaaatcagtagcttacagaac-----------gcaaacagtaggaccatagtgg
ggg----attctatc------------
>gnl|SRA|DRR000630.8197537.2 HWUSI-EAS1632R_0006_FC61F2Y:3:12:15444:18464..
cgccaacaaatcagtagcttacagaac-----------gcaaacagtaggaccatagtgg
ggg----attctatc------------
>_R_gnl|SRA|DRR000630.8960348.2 HWUSI-EAS1632R_0006_FC61F2Y:3:34:15877:3491..
cgccaacaaatcagtagcttacagaac-----------gcaaacagtaggaccatagtgg
ggg----attctatc------------
>_R_gnl|SRA|DRR000630.6505175.1 HWUSI-EAS1632R_0006_FC61F2Y:2:83:15613:16690..
cgccaacaaatcagtagcttacagaac-----------gcaaacagtaggaccatagtgg
ggg----attctatc------------
>_R_gnl|SRA|DRR000630.408996.2 HWUSI-EAS1632R_0006_FC61F2Y:1:12:14918:16632..
cgccaacaagtcagtagcttacagaac-----------gcaaacagtaggaccatagtgg
ggg----attctatc------------
>_R_gnl|SRA|DRR000630.9390296.2 HWUSI-EAS1632R_0006_FC61F2Y:3:48:5802:19426..
cgccaacaaatcagtagcttacagaac-----------gcaaacagtaggactatagtgg
ggg----attctatc------------
>_R_gnl|SRA|DRR000630.12923.1 HWUSI-EAS1632R_0006_FC61F2Y:1:1:8027:11762..
cgccaacaaatcagtagcttacagaac-----------gcaaacagtaggaccatagtgg
ggc----attctatc------------
>gnl|SRA|DRR000630.5680643.1 HWUSI-EAS1632R_0006_FC61F2Y:2:57:1218:9487..
cgccaacaaatcagtagcttacagaac-----------gcaaacagtaggaccatagtgg
ggg----cttctctc------------
>_R_gnl|SRA|DRR000630.497313.2 HWUSI-EAS1632R_0006_FC61F2Y:1:15:7750:19619..
cgccaacaaatcagtagcttacagaac-----------gcaaactgtaggaccatagtgg
ggg----cttctatc------------
>gnl|SRA|DRR000630.935944.2 HWUSI-EAS1632R_0006_FC61F2Y:1:28:9595:17939..
cgccaacaaatcagtagcttacagaac-----------gcaaacagtaggaccatagtgg
ggg----att-----------------
>gnl|SRA|DRR000630.5350039.2 HWUSI-EAS1632R_0006_FC61F2Y:2:46:6108:8061..
cgccaacaaatcagtagcttacagaac-----------gcaaaccgtaggaccatagtgg
ggg----att-----------------
>_R_gnl|SRA|DRR000630.8874348.2 HWUSI-EAS1632R_0006_FC61F2Y:3:32:6245:16338..
cgccaacaaatcagtagcttacagaac-----------gcaaacagtaggaccatagtgg
ggg----a-------------------
>_R_gnl|SRA|DRR000630.4445532.1 HWUSI-EAS1632R_0006_FC61F2Y:2:19:3027:5027..
cgccaacaaatcagtagcttacagaac-----------gcaaacagtaggaccatagtgg

ggg----a-------------------
>_R_gnl|SRA|DRR000630.1834632.1 HWUSI-EAS1632R_0006_FC61F2Y:1:58:13168:15128..
cgccaacaaatcagtagcttggagaac-----------gcaaacagtagtaccatagtgg
ggg----a-------------------
>_R_gnl|SRA|DRR000630.6990277.2 HWUSI-EAS1632R_0006_FC61F2Y:2:98:8099:14489..
cgccaacaaatcagtagcttacagaac-----------gcaaacagtaggaccatagtgg
gg-------------------------
>_R_gnl|SRA|DRR000630.4898225.1 HWUSI-EAS1632R_0006_FC61F2Y:2:32:7971:20086..
cgccaacaaatcagtagcttacagaac-----------gcaaacagtaggaccatagtgg
gg-------------------------
>_R_gnl|SRA|DRR000630.3920478.2 HWUSI-EAS1632R_0006_FC61F2Y:2:4:1582:14370..
cgccaacaaatcagtagcttacagaac-----------gcaaacagtaggaccatagtgg
gg-------------------------
>_R_gnl|SRA|DRR000630.3013601.2 HWUSI-EAS1632R_0006_FC61F2Y:1:97:13910:4017..
cgccaacaaatcagtagcttacagaac-----------gcaaacagtaggaccatagtgg
gg-------------------------
>_R_gnl|SRA|DRR000630.1332552.2 HWUSI-EAS1632R_0006_FC61F2Y:1:42:3568:12504..
cgccaacaaatcagtagcttacagaac-----------gcaaacagtaggaccatagtgg
gg-------------------------
>_R_gnl|SRA|DRR000630.4795939.1 HWUSI-EAS1632R_0006_FC61F2Y:2:29:7750:13794..
cgccaacaaatcagtagcttacagaac-----------gcaaacagtaggaccatagtgg
gg-------------------------
>_R_gnl|SRA|DRR000630.9973022.1 HWUSI-EAS1632R_0006_FC61F2Y:3:66:10600:4726..
cgccaacaaatcagtagcttacagaac-----------gcaaacagtaggaccatagcgg
gg-------------------------
>_R_gnl|SRA|DRR000630.43380.2 HWUSI-EAS1632R_0006_FC61F2Y:1:2:4987:13982..
cgccaacaaatcagtagcttacagaac-----------gcaaacagtaggaccatagcgg
gg-------------------------
>gnl|SRA|DRR000630.9746823.2 HWUSI-EAS1632R_0006_FC61F2Y:3:59:7310:7275..
cgccaacaaatcagtagcttacagaac-----------gcaaacagtaggaccatagtgg
ggg------------------------
>gnl|SRA|DRR000630.4073304.1 HWUSI-EAS1632R_0006_FC61F2Y:2:8:8214:19860..
tgccaacaaatcagtagcttacagaac-----------gcaaacagtaggaccatagtgg
ggg------------------------
>_R_gnl|SRA|DRR000630.4073304.2 HWUSI-EAS1632R_0006_FC61F2Y:2:8:8214:19860..
tgccaacaaatcagtagcttacagaac-----------gcaaacagtaggaccatagtgg
ggg------------------------
>gnl|SRA|DRR000630.4445532.2 HWUSI-EAS1632R_0006_FC61F2Y:2:19:3027:5027..
cgccaacaaatcagtagcttacagaac-----------gcaaacagtaggaccatagtgg
ggg-aagatcggaag------------
>gnl|SRA|DRR000630.11707539.2 HWUSI-EAS1632R_0006_FC61F2Y:3:118:9850:1152..
cgccaacaaatcagtagcttacagaac-----------gcaaacagtaggaccatagtgg
gag----atcggaag------------
>gnl|SRA|DRR000630.261123.2 HWUSI-EAS1632R_0006_FC61F2Y:1:8:9391:20393..
cgccaacaaatcagtagcttacagaac-----------gcaaacagtaggaccatagtgg
gag----atcggaag------------
>_R_gnl|SRA|DRR000630.64891.2 HWUSI-EAS1632R_0006_FC61F2Y:1:2:15960:10287..
cgccaacaaatcagtagcttacagaac-----------gcaaacagtaggaccatagtgg
g--------------------------
>_R_gnl|SRA|DRR000630.11707539.1 HWUSI-EAS1632R_0006_FC61F2Y:3:118:9850:1152..
cgccaacaaatcagtagcttacagaac-----------gcaaacagtaggaccatagtgg

g--------------------------
>_R_gnl|SRA|DRR000630.261123.1 HWUSI-EAS1632R_0006_FC61F2Y:1:8:9391:20393..
cgccaacaaatcagtagcttacagaac-----------gcaaacagtaggaccatagtgg
g--------------------------
>_R_gnl|SRA|DRR000630.9505861.1 HWUSI-EAS1632R_0006_FC61F2Y:3:51:16050:12174..
cgccaacaaatcagtagcttacagaac-----------gcaaacagtatgaccatagtgg
g--------------------------
>gnl|SRA|DRR000630.10778815.1 HWUSI-EAS1632R_0006_FC61F2Y:3:91:13880:3984..
cgccaacaaatcagtagcttacagaac-----------gcaaacagtaggaccatagt--

>_R_gnl|SRA|DRR000630.10713801.1 HWUSI-EAS1632R_0006_FC61F2Y:3:89:15104:17602..
cgccaacaaatcagtagcttacagaac-----------gcaaacagtaggaccatagt--

>_R_gnl|SRA|DRR000630.9978925.1 HWUSI-EAS1632R_0006_FC61F2Y:3:66:14041:10942..
cgccaacaaatcagtagcttacagaac-----------gcaaacagtaggaccatagcg-

>_R_gnl|SRA|DRR000630.9357819.2 HWUSI-EAS1632R_0006_FC61F2Y:3:47:3678:1017..
cgccaacaaatcagtagcttacagaac-----------gcaaacagtaggaccatag---

>gnl|SRA|DRR000630.3927361.2 HWUSI-EAS1632R_0006_FC61F2Y:2:4:5184:8871..
cgccaacaaatcagtagcttacagaac-----------gcaaacagtaggaccataa---
------gatc-----------------
>_R_gnl|SRA|DRR000630.3927361.1 HWUSI-EAS1632R_0006_FC61F2Y:2:4:5184:8871..
cgccaacaaatcagtagcttacagaac-----------gcaaacagtaggaccata----

>gnl|SRA|DRR000630.1590056.2 HWUSI-EAS1632R_0006_FC61F2Y:1:50:10670:4180..
cgccaacaaatcagtagcttacagaac-----------gcaaacagtaggaccata----

>gnl|SRA|DRR000630.4795939.2 HWUSI-EAS1632R_0006_FC61F2Y:2:29:7750:13794..
cgccaacaaatcagtagcttacagaac-----------gcaaacagtaggaccat-----

>gnl|SRA|DRR000630.585279.1 HWUSI-EAS1632R_0006_FC61F2Y:1:17:18741:3139..
cgccaacaaatcngtagcttacagaac-----------gcaaacagtaggaccat-----

>gnl|SRA|DRR000630.2917864.2 HWUSI-EAS1632R_0006_FC61F2Y:1:94:15839:18494..
cgccaacaaatcagtagcttacagaac-----------gcaaacagtaggacc-------

>gnl|SRA|DRR000630.99774.2 HWUSI-EAS1632R_0006_FC61F2Y:1:3:15958:5543..
cgccaacaaatcagtagcttacagaac-----------gcaaacagtaggacc-------

>_R_gnl|SRA|DRR000630.5072850.2 HWUSI-EAS1632R_0006_FC61F2Y:2:37:11845:6502..
cgccaacaaatcagtagcttacagaac-----------gcaaacagtaggac--------

>_R_gnl|SRA|DRR000630.4737965.1 HWUSI-EAS1632R_0006_FC61F2Y:2:27:12850:13335..
cgccaacaaatcagtagcttacagaac-----------gcaaacagtaggac--------

>_R_gnl|SRA|DRR000630.10033590.1 HWUSI-EAS1632R_0006_FC61F2Y:3:68:9038:1072..
cgccaacaaatcagtagcttacagaac-----------gcaaacagtaggacca------

>_R_gnl|SRA|DRR000630.9106811.1 HWUSI-EAS1632R_0006_FC61F2Y:3:39:3553:12464..
cgccaacaaatcagtagcttacagaac-----------gcaaacagtaggacca------

>_R_gnl|SRA|DRR000630.997595.2 HWUSI-EAS1632R_0006_FC61F2Y:1:30:9058:2385..
cgctaacaaatcagtagcttacagagc-----------gcaaacagtaggacca------

>_R_gnl|SRA|DRR000630.728631.1 HWUSI-EAS1632R_0006_FC61F2Y:1:22:5373:17110..
cgccaacaaatcagtagcttacagaac-----------gcaaacagtaggctca------

>_R_gnl|SRA|DRR000630.5427966.2 HWUSI-EAS1632R_0006_FC61F2Y:2:49:2004:6453..
cgccaacaaatcagtagcttacagaac-----------gcaaacagtaggctcatggtgt
tgatagaatcc----------------
>_R_gnl|SRA|DRR000630.2182258.2 HWUSI-EAS1632R_0006_FC61F2Y:1:70:7064:7883..
cgccaacaaatcagtagcttacagaac-----------gcaaacagtaggctcatggtgt
tgatagaatcccc--------------
>gnl|SRA|DRR000630.5427966.1 HWUSI-EAS1632R_0006_FC61F2Y:2:49:2004:6453..
cgccaacaaatcagtagcttacagaac-----------gcaaacagtaggctcatggtgt
tgatagaatccagat------------
>_R_gnl|SRA|DRR000630.7831909.1 HWUSI-EAS1632R_0006_FC61F2Y:3:2:10852:11757..
cgccaacaaatcagtagcttacagaac-----------gcaaacagtagga---------

>gnl|SRA|DRR000630.3129176.1 HWUSI-EAS1632R_0006_FC61F2Y:1:101:4326:15177..
cgccaacaaatcagtagcttacagaac-----------gcaaacagtagga---------

>_R_gnl|SRA|DRR000630.11740116.1 HWUSI-EAS1632R_0006_FC61F2Y:3:119:7883:17196..
cgccaacaaatcagtagcttacagaac-----------gcaaacagtag-----------

>gnl|SRA|DRR000630.8094791.1 HWUSI-EAS1632R_0006_FC61F2Y:3:9:16919:16645..
cgccaacaaatcagtagcttacagaac-----------gcaaacagta------------

>gnl|SRA|DRR000630.4802901.2 HWUSI-EAS1632R_0006_FC61F2Y:2:29:11482:2461..
cgccaacaaatcagtagcttacagaac-----------gcaaacagta------------

>gnl|SRA|DRR000630.2296658.2 HWUSI-EAS1632R_0006_FC61F2Y:1:74:1882:14512..
cgccaacaaatcagtagcttacagaac-----------gcaaacagta------------

>gnl|SRA|DRR000630.6580040.1 HWUSI-EAS1632R_0006_FC61F2Y:2:86:2571:16996..
cgccaacaaatcagtagcttacagaac-----------gcaaacagca------------

>gnl|SRA|DRR000630.9102514.1 HWUSI-EAS1632R_0006_FC61F2Y:3:38:19630:12908..
cgccaacaaatcagtagcttacagaaa-----------gcaaacagca------------

>gnl|SRA|DRR000630.3291247.2 HWUSI-EAS1632R_0006_FC61F2Y:1:105:19671:16732..
cgccaacaaatcagtagcttacagaac-----------gcaaacagccggcacatagcgg
ggg----att-----------------
>gnl|SRA|DRR000630.4898225.2 HWUSI-EAS1632R_0006_FC61F2Y:2:32:7971:20086..
cgccaacaaatcagtagcttacagaac-----------gcaaacag-------------

>gnl|SRA|DRR000630.3667325.1 HWUSI-EAS1632R_0006_FC61F2Y:1:116:19714:7508..
cgccaacaaatcagtagcttacagaac-----------gcaaacag-------------

>gnl|SRA|DRR000630.9893859.2 HWUSI-EAS1632R_0006_FC61F2Y:3:63:19770:5363..
cgccaacaaatcagtagcttacagaac-----------gcaaaaag-------------

>gnl|SRA|DRR000630.8273797.1 HWUSI-EAS1632R_0006_FC61F2Y:3:14:18415:15917..
cgccaacaaatcagtagcttacagaac-------------gcaaacagatc-------------
----
>gnl|SRA|DRR000630.10841675.1 HWUSI-EAS1632R_0006_FC61F2Y:3:93:11312:1501..
cgccaacaaatcagtagcttacagaac-------------gcaaacagatcg------------
----
>_R_gnl|SRA|DRR000630.6963990.1 HWUSI-EAS1632R_0006_FC61F2Y:2:97:12215:17974..
cgccaacaaatcagtagcttacagaac-------------gcaaaca-----------------
----
>_R_gnl|SRA|DRR000630.2116418.1 HWUSI-EAS1632R_0006_FC61F2Y:1:68:4137:2331..
cgccaacaaatcagtagcttacagaac-------------gcaaaca-----------------
----
>_R_gnl|SRA|DRR000630.11041853.1 HWUSI-EAS1632R_0006_FC61F2Y:3:99:8594:6566..
cgccaacaaatcagtagcttacagaac-------------gcaaacag---gacca------
----
>_R_gnl|SRA|DRR000630.9555265.2 HWUSI-EAS1632R_0006_FC61F2Y:3:53:7372:12536..
cgccaacaaatcagtagcttacagaac-------------gcaaacag---gaccatagtgg
ggg----attc----------------
>_R_gnl|SRA|DRR000630.6492236.2 HWUSI-EAS1632R_0006_FC61F2Y:2:83:8363:17393..
cgccaacaaatcagtagcttacagaac-------------gcaaacag---gaccatagtgg
ggg----attc----------------
>_R_gnl|SRA|DRR000630.5108496.2 HWUSI-EAS1632R_0006_FC61F2Y:2:38:13264:2828..
cgccaacaaatcagtagcttacagaac-------------gcaaacag---gaccatagtgg
ggg----attc----------------
>gnl|SRA|DRR000630.4457478.2 HWUSI-EAS1632R_0006_FC61F2Y:2:19:9191:5000..
cgccaacaaatcagtagcttacagaac-------------gcaaacag---gaccatagtgg
ggg----attcta--------------
>gnl|SRA|DRR000630.1097210.1 HWUSI-EAS1632R_0006_FC61F2Y:1:33:9779:18975..
cgccaacaaatcagtagcttacagaac-------------gcaaacag---gaccatagtgg
ggg----attcta--------------
>_R_gnl|SRA|DRR000630.8668045.1 HWUSI-EAS1632R_0006_FC61F2Y:3:26:6337:19560..
cgccaacaaatcagtagcttacagaac-------------gcaaacag---gaccatagtgg
ggg----attctatc------------
>_R_gnl|SRA|DRR000630.4860338.1 HWUSI-EAS1632R_0006_FC61F2Y:2:31:5893:13442..
cgccaacaaatcagtagcttacagaac-------------gcaaacag---gaccatagtgg
ggg----attctatc------------
>_R_gnl|SRA|DRR000630.2777341.1 HWUSI-EAS1632R_0006_FC61F2Y:1:90:10613:18618..
cgccaacaaatcagtagcttacagaac-------------gcaaacag---gaccatagtgg
ggg----attctatc------------
>_R_gnl|SRA|DRR000630.8128395.2 HWUSI-EAS1632R_0006_FC61F2Y:3:10:16070:8752..
cgccaacaaatcagtagcttacagaac-------------gcaaacag---gaccatagtgg
ggg----attctatc------------
>_R_gnl|SRA|DRR000630.6006472.1 HWUSI-EAS1632R_0006_FC61F2Y:2:67:12558:6652..
cgccaacaaatcagtagcttacagaac-------------gcaaacag---gaccatagtgg
ggg----attctatc------------
>_R_gnl|SRA|DRR000630.7564588.2 HWUSI-EAS1632R_0006_FC61F2Y:2:115:2563:3146..
cgccaacaaatcagtagcttacagaac-------------gcaaacag---gaccatagtgg
ggg----attctatc------------
>_R_gnl|SRA|DRR000630.6574586.1 HWUSI-EAS1632R_0006_FC61F2Y:2:85:17841:12847..
cgccaacaaatcagtagcttacagaac-------------gcaaacag---gaccatagtgg

ggg----attctatc------------
>_R_gnl|SRA|DRR000630.109887.2 HWUSI-EAS1632R_0006_FC61F2Y:1:4:3006:13656..
cgccaacaaatcagtagcttacagaac-----------gcaaacag---gaccatagtgg
ggg----attctatc------------
>_R_gnl|SRA|DRR000630.3161464.1 HWUSI-EAS1632R_0006_FC61F2Y:1:102:3471:2817..
cgccaacaaatcagtagcttacagaac-----------gcaaacag---gaccatagtgg
ggg----attctatc------------
>gnl|SRA|DRR000630.1391280.2 HWUSI-EAS1632R_0006_FC61F2Y:1:44:2456:12853..
cgccaacaaatcagtagcttacagaac-----------gcaaacag---gaccatagtgg
ggg----attctatc------------
>_R_gnl|SRA|DRR000630.6582909.2 HWUSI-EAS1632R_0006_FC61F2Y:2:86:4208:11910..
cgccaacaaatcagtagcttacagaac-----------gcaaacag---gaccatagtgg
ggg----attctatc------------
>_R_gnl|SRA|DRR000630.3859075.1 HWUSI-EAS1632R_0006_FC61F2Y:2:2:6804:9181..
cgccaacaaatcagtagcttacagaac-----------gcaaacag---gaccatagtgg
ggg----attctatc------------
>gnl|SRA|DRR000630.6947986.2 HWUSI-EAS1632R_0006_FC61F2Y:2:97:3505:11999..
cgccaacaaatcagtagcttacagaac-----------gcaaacag---gaccatagtgg
ggg----attctatc------------
>gnl|SRA|DRR000630.1964366.1 HWUSI-EAS1632R_0006_FC61F2Y:1:63:3181:18463..
cgccaacaaatcagtagcttacagaac-----------gcaaacag---gaccatagtgg
ggg----attctatc------------
>gnl|SRA|DRR000630.1825621.2 HWUSI-EAS1632R_0006_FC61F2Y:1:58:7266:12301..
cgccaacaaatcagtagcttacagaac-----------gcaaacag---gaccatagtgg
ggg----attctatc------------
>gnl|SRA|DRR000630.3859075.2 HWUSI-EAS1632R_0006_FC61F2Y:2:2:6804:9181..
cgccaacaaatcagtagcttacagaac-----------gcaaacag---gaccatagtgg
ggg----attctatc------------
>gnl|SRA|DRR000630.11263498.2 HWUSI-EAS1632R_0006_FC61F2Y:3:105:17160:11751..
cgccaacaaatcagtagcttacagaac-----------gcaaacag---gaccatagtgg
ggg----attctatc------------
>gnl|SRA|DRR000630.3668477.2 HWUSI-EAS1632R_0006_FC61F2Y:1:117:1641:18712..
cgccaacaaatcagtagcttacagaac-----------gcaaacag---gaccatagtgg
ggg----attctatc------------
>gnl|SRA|DRR000630.2384146.2 HWUSI-EAS1632R_0006_FC61F2Y:1:76:16551:14646..
cgccaacaaatcagtagcttacagaac-----------gcaaacag---gaccatagtgg
ggg----attctatc------------
>_R_gnl|SRA|DRR000630.9199648.2 HWUSI-EAS1632R_0006_FC61F2Y:3:41:17531:4962..
cgccaacaaatcagtagcttacagaac-----------gcaaacag---gaccatagtgg
ggg----attctatc------------
>_R_gnl|SRA|DRR000630.5162752.1 HWUSI-EAS1632R_0006_FC61F2Y:2:40:6712:7718..
cgccaacaaatcagtagcttacagaac-----------gcaaacag---gaccatagtgg
ggg----attctatc------------
>_R_gnl|SRA|DRR000630.1191499.1 HWUSI-EAS1632R_0006_FC61F2Y:1:36:7974:16800..
cgccaacaaatcagtagcttacagaac-----------gcaaacag---gaccatagtgg
ggg----attctatc------------
>_R_gnl|SRA|DRR000630.2641976.2 HWUSI-EAS1632R_0006_FC61F2Y:1:86:7935:2922..
cgccaacaaatcagtagcttacagaac-----------gcaaacag---gaccatagtgg
ggg----attctatc------------
>gnl|SRA|DRR000630.4664482.2 HWUSI-EAS1632R_0006_FC61F2Y:2:25:9822:1733..
cgccaacaaatcagtagcttacagaac-----------gcaaacag---gaccatagtgg

ggg----attctatc------------
>_R_gnl|SRA|DRR000630.4664482.1 HWUSI-EAS1632R_0006_FC61F2Y:2:25:9822:1733..
cgccaacaaatcagtagcttacagaac-----------gcaaacag---gaccatagtgg
ggg----attctatc------------
>_R_gnl|SRA|DRR000630.7374521.2 HWUSI-EAS1632R_0006_FC61F2Y:2:109:12351:4590..
cgccaacaaatcagtagcttacagaac-----------gcaaacag---gaccatagtgg
ggg----attctatc------------
>_R_gnl|SRA|DRR000630.11263498.1 HWUSI-EAS1632R_0006_FC61F2Y:3:105:17160:11751..
cgccaacaaatcagtagcttacagaac-----------gcaaacag---gaccatagtgg
ggg----attctatc------------
>_R_gnl|SRA|DRR000630.5028146.1 HWUSI-EAS1632R_0006_FC61F2Y:2:36:5619:9117..
cgccaacaaatcagtagcttacagaac-----------gcaaacag---gaccatagtgg
ggg----attctatc------------
>_R_gnl|SRA|DRR000630.4884122.1 HWUSI-EAS1632R_0006_FC61F2Y:2:31:18848:2296..
cgccaacaaatcagtagcttacagaac-----------gcaaacag---gaccatagtgg
ggg----attctatc------------
>_R_gnl|SRA|DRR000630.4457478.1 HWUSI-EAS1632R_0006_FC61F2Y:2:19:9191:5000..
cgccaacaaatcagtagcttacagaac-----------gcaaacag---gaccatagtgg
ggg----attctatc------------
>_R_gnl|SRA|DRR000630.3668477.1 HWUSI-EAS1632R_0006_FC61F2Y:1:117:1641:18712..
cgccaacaaatcagtagcttacagaac-----------gcaaacag---gaccatagtgg
ggg----attctatc------------
>_R_gnl|SRA|DRR000630.7893060.2 HWUSI-EAS1632R_0006_FC61F2Y:3:4:4733:1727..
cgccaacaaatcagtagcttacagaac-----------gcaaacag---gaccatagtgg
ggg----attctatc------------
>_R_gnl|SRA|DRR000630.3310602.1 HWUSI-EAS1632R_0006_FC61F2Y:1:106:11531:13014..
cgccaacaaatcagtagcttacagaac-----------gcaaacag---gaccatagtgg
ggg----attctatc------------
>_R_gnl|SRA|DRR000630.3296338.1 HWUSI-EAS1632R_0006_FC61F2Y:1:106:3839:17028..
cgccaacaaatcagtagcttacagaac-----------gcaaacag---gaccatagtgg
ggg----attctatc------------
>_R_gnl|SRA|DRR000630.2630341.2 HWUSI-EAS1632R_0006_FC61F2Y:1:86:1387:13630..
cgccaacaaatcagtagcttacagaac-----------gcaaacag---gaccatagtgg
ggg----attctatc------------
>_R_gnl|SRA|DRR000630.4130451.2 HWUSI-EAS1632R_0006_FC61F2Y:2:10:1663:16748..
cgccaacaaatcagtagcttacagaac-----------gcaaacag---gaccatagtgg
ggg----attctatc------------
>_R_gnl|SRA|DRR000630.1825621.1 HWUSI-EAS1632R_0006_FC61F2Y:1:58:7266:12301..
cgccaacaaatcagtagcttacagaac-----------gcaaacag---gaccatagtgg
ggg----attctatc------------
>gnl|SRA|DRR000630.1208842.2 HWUSI-EAS1632R_0006_FC61F2Y:1:36:17994:8571..
cgccaacaaatcagtagcttacagaac-----------gcaaacag---gaccatagtcg
ggg----attctatc------------
>_R_gnl|SRA|DRR000630.1391280.1 HWUSI-EAS1632R_0006_FC61F2Y:1:44:2456:12853..
cgccaacaaatcagtagcttacagaac-----------gcaaacag---gaccatagtgg
ggg----attctata------------
>_R_gnl|SRA|DRR000630.2455875.1 HWUSI-EAS1632R_0006_FC61F2Y:1:79:3803:12268..
cgccaacaaatcagtagcttacagaac-----------gcaaacag---gaccatagtgg
ggg----attctat-------------
>gnl|SRA|DRR000630.1586209.1 HWUSI-EAS1632R_0006_FC61F2Y:1:50:8384:4306..
cgccaacaaatcagtagcttacagaac-----------gcaaacag---gaccatagtgg

ggg----attctat-------------
>_R_gnl|SRA|DRR000630.1586209.2 HWUSI-EAS1632R_0006_FC61F2Y:1:50:8384:4306..
cgccaacaaatcagtagcttacagcac-----------gcaaacag---gaccatagtgg
ggg----attctat-------------
>_R_gnl|SRA|DRR000630.5463199.1 HWUSI-EAS1632R_0006_FC61F2Y:2:50:3833:4811..
cgccaacaaatcagtagcttacagaac-----------gcaaacag---gaccatagtgg
ggg----atttc---------------
>_R_gnl|SRA|DRR000630.9880328.2 HWUSI-EAS1632R_0006_FC61F2Y:3:63:11515:7418..
cgccaacaaatcagtagcttacagaac-----------gcaaacag---gaccatagtgg
ggg----at------------------
>_R_gnl|SRA|DRR000630.9079835.2 HWUSI-EAS1632R_0006_FC61F2Y:3:38:7331:2557..
cgccaacaaatcagtagcttacagaac-----------gcaaacag---gaccatagtgg
ggg------------------------
>_R_gnl|SRA|DRR000630.2992474.2 HWUSI-EAS1632R_0006_FC61F2Y:1:97:2201:16105..
cgccaacaaatcagtagcttacagaac-----------gcaaacag---gaccatagtgg
ggg------------------------
>_R_gnl|SRA|DRR000630.9670370.1 HWUSI-EAS1632R_0006_FC61F2Y:3:56:18260:4248..
cgccaacaaatcagtagcttacagaac-----------gcaaacag---gaccatagtgg
gg-------------------------
>_R_gnl|SRA|DRR000630.4872119.2 HWUSI-EAS1632R_0006_FC61F2Y:2:31:12162:7997..
cgccaacaaatcagtagcttacagaac-----------gcaaacag---gaccatagtgg
gg-------------------------
>_R_gnl|SRA|DRR000630.124080.1 HWUSI-EAS1632R_0006_FC61F2Y:1:4:10453:3856..
cgccaacaaatcagtagcttacagaac-----------gcaaacag---gaccatagtgg
gg-------------------------
>_R_gnl|SRA|DRR000630.8242451.1 HWUSI-EAS1632R_0006_FC61F2Y:3:14:1934:19887..
cgccaacaaatcagtagcttacagaac-----------gcaaacag---gaccatagtgg
gg-------------------------
>gnl|SRA|DRR000630.5717911.1 HWUSI-EAS1632R_0006_FC61F2Y:2:58:5166:11027..
cgccaacaaatcagtagcttacagaac-----------gcaaacag---gaccatagtgg
gag----gtgtca--------------
>_R_gnl|SRA|DRR000630.5717911.2 HWUSI-EAS1632R_0006_FC61F2Y:2:58:5166:11027..
cgccaacaaatcagtagcttacagaac-----------gcaaacag---gaccatagtgg
gag----gtgtcactgttt--------
>_R_gnl|SRA|DRR000630.8270288.2 HWUSI-EAS1632R_0006_FC61F2Y:3:14:16575:16595..
cgccaacaaatcagtagcttacagaac-----------gcaaacag---gaccatagtgg
g--------------------------
>_R_gnl|SRA|DRR000630.4951492.2 HWUSI-EAS1632R_0006_FC61F2Y:2:33:18744:20177..
cgccaacaaatcagtagcttacagaac-----------gcaaacag---gaccatagtgg

>_R_gnl|SRA|DRR000630.4568294.1 HWUSI-EAS1632R_0006_FC61F2Y:2:22:13262:5303..
cgccaacaaatcagtagcttacagaac-----------gcaaacag---gaccatagtgg

>_R_gnl|SRA|DRR000630.9808439.2 HWUSI-EAS1632R_0006_FC61F2Y:3:61:6154:5658..
cgccaacaaatcagtagcttacagaac-----------gcaaacag---gaccatagtg-

>_R_gnl|SRA|DRR000630.5553381.2 HWUSI-EAS1632R_0006_FC61F2Y:2:52:18970:10247..
cgccaacaaatcagtagcttacagaac-----------gcaaacag---gaccatagtg-

>_R_gnl|SRA|DRR000630.1756479.1 HWUSI-EAS1632R_0006_FC61F2Y:1:56:1087:3634..
cgccaacaaatcagtagcttacagaac-----------gcaaacag---gaccatag---

>_R_gnl|SRA|DRR000630.11735356.1 HWUSI-EAS1632R_0006_FC61F2Y:3:119:5553:2548..
cgccaacaaatcagtagcttacagaac-----------gcaaacag---gaccatag---

>gnl|SRA|DRR000630.10666948.1 HWUSI-EAS1632R_0006_FC61F2Y:3:88:7773:13064..
cgccaacaaatcagtagcttacagaac-----------gcaaacag---gacc-------

>gnl|SRA|DRR000630.9996741.2 HWUSI-EAS1632R_0006_FC61F2Y:3:67:5995:8139..
cgccaacaaatcagtagcttacagaac-----------gcaaacag---gacc-------

>gnl|SRA|DRR000630.559043.2 HWUSI-EAS1632R_0006_FC61F2Y:1:17:4403:11514..
cgccaacaaatcagtagcttacagaac-----------gcaaacag---gacc-------

>_R_gnl|SRA|DRR000630.11125744.2 HWUSI-EAS1632R_0006_FC61F2Y:3:101:16758:12364..
cgccaacaaatcagtagcttacagaac-----------gcaaacag---gac--------

>_R_gnl|SRA|DRR000630.3060080.2 HWUSI-EAS1632R_0006_FC61F2Y:1:99:3147:19796..
cgccaacaaatcagtagcttacagaac-----------gcaaacag---gac--------

>_R_gnl|SRA|DRR000630.879879.1 HWUSI-EAS1632R_0006_FC61F2Y:1:26:15037:10906..
cgccaacaaatcagtagcttacagaac-----------gcaaacag---gac--------

>gnl|SRA|DRR000630.8852675.1 HWUSI-EAS1632R_0006_FC61F2Y:3:31:12900:6424..
cgccaacaaatcagtagcttacagaac-----------gcaaacag---gac--------

>_R_gnl|SRA|DRR000630.8852675.2 HWUSI-EAS1632R_0006_FC61F2Y:3:31:12900:6424..
cgccaacaaatcagtagcttacagaac-----------gcaaacag---gac--------

>gnl|SRA|DRR000630.5553381.1 HWUSI-EAS1632R_0006_FC61F2Y:2:52:18970:10247..
cgccaacaaatcagtagcttacagaac-----------gcaaacag---ga---------

>gnl|SRA|DRR000630.5108496.1 HWUSI-EAS1632R_0006_FC61F2Y:2:38:13264:2828..
cgccaacaaatcagtagcttacagaac-----------gcaaacag---ga---------

>gnl|SRA|DRR000630.6582909.1 HWUSI-EAS1632R_0006_FC61F2Y:2:86:4208:11910..
cgccaacaaatcagtagcttacagaac-----------gcaaacag---g----------

>_R_gnl|SRA|DRR000630.5794620.1 HWUSI-EAS1632R_0006_FC61F2Y:2:60:14653:3574..
cgccaacaaatcagtagcttacagaac-----------gcaaacag---g----------

>gnl|SRA|DRR000630.5758574.1 HWUSI-EAS1632R_0006_FC61F2Y:2:59:10802:11850..
cgccaacaaatcagtagcttacagaac-----------gcaaacag---g----------

>_R_gnl|SRA|DRR000630.5250530.1 HWUSI-EAS1632R_0006_FC61F2Y:2:42:19217:13403..
tgccaacaaatcagtagcttacagaac-----------gcaaacag---g----------

>_R_gnl|SRA|DRR000630.10841675.2 HWUSI-EAS1632R_0006_FC61F2Y:3:93:11312:1501..
cgccaacaaatcagtagcttacagaac-----------gcaaac----------------

>_R_gnl|SRA|DRR000630.9893859.1 HWUSI-EAS1632R_0006_FC61F2Y:3:63:19770:5363..
cgccaacaaatcagtagcttacagaac-----------gcaaac----------------

>_R_gnl|SRA|DRR000630.8336684.1 HWUSI-EAS1632R_0006_FC61F2Y:3:16:14646:18415..
cgccaacaaatcagtagcttacagaac-----------gcaaac----------------

>_R_gnl|SRA|DRR000630.7155045.1 HWUSI-EAS1632R_0006_FC61F2Y:2:103:5381:15384..
cgccaacaaatcagtagcttacagaac-----------gcaaac----------------

>_R_gnl|SRA|DRR000630.6078446.2 HWUSI-EAS1632R_0006_FC61F2Y:2:69:17885:16046..
cgccaacaaatcagtagcttacagaac-----------gcaaac----------------

>_R_gnl|SRA|DRR000630.5478860.2 HWUSI-EAS1632R_0006_FC61F2Y:2:50:12726:16383..
cgccaacaaatcagtagcttacagaac-----------gcaaac----------------

>_R_gnl|SRA|DRR000630.5200434.2 HWUSI-EAS1632R_0006_FC61F2Y:2:41:9392:8047..
cgccaacaaatcagtagcttacagaac-----------gcaaac----------------

>_R_gnl|SRA|DRR000630.3928885.2 HWUSI-EAS1632R_0006_FC61F2Y:2:4:5957:6459..
cgccaacaaatcagtagcttacagaac-----------gcaaac----------------

>_R_gnl|SRA|DRR000630.2259882.2 HWUSI-EAS1632R_0006_FC61F2Y:1:72:16740:9496..
cgccaacaaatcagtagcttacagaac-----------gcaaac----------------

>_R_gnl|SRA|DRR000630.2248077.2 HWUSI-EAS1632R_0006_FC61F2Y:1:72:9647:14687..
cgccaacaaatcagtagcttacagaac-----------gcaaac----------------

>_R_gnl|SRA|DRR000630.1662667.1 HWUSI-EAS1632R_0006_FC61F2Y:1:52:17431:9294..
cgccaacaaatcagtagcttacagaac-----------gcaaac----------------

>_R_gnl|SRA|DRR000630.8273797.2 HWUSI-EAS1632R_0006_FC61F2Y:3:14:18415:15917..
cgccaacaaatcagtagcttacagaac-----------gcaaac----------------

>_R_gnl|SRA|DRR000630.5834772.1 HWUSI-EAS1632R_0006_FC61F2Y:2:62:2056:9191..
cgccaacaaatcagtagcttacagaac-----------gcaaac----------------

>_R_gnl|SRA|DRR000630.2206396.1 HWUSI-EAS1632R_0006_FC61F2Y:1:71:3088:10171..
cgccaacaaatcagtagcttacagaac-----------gcaaac----------------

>gnl|SRA|DRR000630.4233603.2 HWUSI-EAS1632R_0006_FC61F2Y:2:12:19290:15630..
cgccaacaaatcagtagcttacagaac-----------gcaaaa----------------

>gnl|SRA|DRR000630.11739233.2 HWUSI-EAS1632R_0006_FC61F2Y:3:119:7469:6512..
cgccaacaaatcagtagcttacagaac-----------gcaaacagt-------------

>gnl|SRA|DRR000630.7258341.1 HWUSI-EAS1632R_0006_FC61F2Y:2:106:5698:16905..
cgccaacaaatcagtagcttacagaac-----------gcaaacagt-------------

>gnl|SRA|DRR000630.6505175.2 HWUSI-EAS1632R_0006_FC61F2Y:2:83:15613:16690..
cgccaacaaatcagtagcttacagaac-----------gcaaacagt-------------

>gnl|SRA|DRR000630.4906167.2 HWUSI-EAS1632R_0006_FC61F2Y:2:32:12139:14370..
cgccaacaaatcagtagcttacagaac-----------gcaaacagt-------------

>gnl|SRA|DRR000630.4363567.2 HWUSI-EAS1632R_0006_FC61F2Y:2:16:14449:9114..
cgccaacaaatcagtagcttacagaac-----------gcaaacagt-------------

>gnl|SRA|DRR000630.2831297.2 HWUSI-EAS1632R_0006_FC61F2Y:1:92:4255:7483..
cgccaacaaatcagtagcttacagaac-----------gcaaaagat-------------

>_R_gnl|SRA|DRR000630.1643125.2 HWUSI-EAS1632R_0006_FC61F2Y:1:52:5778:13384..
cgccagcaaatcagtagcttacagaac-----------gcaaaccgg-------------

>_R_gnl|SRA|DRR000630.11799848.1 HWUSI-EAS1632R_0006_FC61F2Y:3:120:19636:4450..
cgccaacaaatcagtagcttacagaac-----------gcaaa-----------------

>_R_gnl|SRA|DRR000630.10694658.2 HWUSI-EAS1632R_0006_FC61F2Y:3:89:4654:2016..
cgccaacaaatcagtagcttacagaac-----------gcaaa-----------------

>_R_gnl|SRA|DRR000630.7802790.1 HWUSI-EAS1632R_0006_FC61F2Y:3:1:14710:10231..
cgccaacaaatcagtagcttacagaac-----------gcaaa-----------------

>_R_gnl|SRA|DRR000630.6958851.1 HWUSI-EAS1632R_0006_FC61F2Y:2:97:9425:9435..
cgccaacaaatcagtagcttacagaac-----------gcaaa-----------------

>_R_gnl|SRA|DRR000630.6086899.1 HWUSI-EAS1632R_0006_FC61F2Y:2:70:4331:11051..
cgccaacaaatcagtagcttacagaac-----------gcaaa-----------------

>_R_gnl|SRA|DRR000630.3083805.2 HWUSI-EAS1632R_0006_FC61F2Y:1:99:16003:12986..
cgccaacaaatcagtagcttacagaac-----------gcaaa-----------------

>_R_gnl|SRA|DRR000630.4233603.1 HWUSI-EAS1632R_0006_FC61F2Y:2:12:19290:15630..
cgccaacaaatcagtagcttacagaac-----------gcaaa-----------------

>_R_gnl|SRA|DRR000630.2831297.1 HWUSI-EAS1632R_0006_FC61F2Y:1:92:4255:7483..
cgccaacaaatcagtagcttacagaac-----------gcaaa-----------------

>_R_gnl|SRA|DRR000630.11793435.2 HWUSI-EAS1632R_0006_FC61F2Y:3:120:16063:3847..
cgccaacaaatcagtagcttacagaac-----------gcaa------------------

>_R_gnl|SRA|DRR000630.10548904.2 HWUSI-EAS1632R_0006_FC61F2Y:3:84:16282:9134..
cgccaacaaatcagtagcttacagaac-----------gcaa------------------

>gnl|SRA|DRR000630.8652881.2 HWUSI-EAS1632R_0006_FC61F2Y:3:25:16573:13823..
cgccaacaaatcagtagcttacagaac-----------gcaa------------------

>gnl|SRA|DRR000630.8128395.1 HWUSI-EAS1632R_0006_FC61F2Y:3:10:16070:8752..
cgccaacaaatcagtagcttacagaac-----------gcaa------------------

>gnl|SRA|DRR000630.7831909.2 HWUSI-EAS1632R_0006_FC61F2Y:3:2:10852:11757..
cgccaacaaatcagtagcttacagaac-----------gcaa------------------

>gnl|SRA|DRR000630.4568294.2 HWUSI-EAS1632R_0006_FC61F2Y:2:22:13262:5303..
cgccaacaaatcagtagcttacagaac-----------gcaa------------------

>gnl|SRA|DRR000630.2992474.1 HWUSI-EAS1632R_0006_FC61F2Y:1:97:2201:16105..
cgccaacaaatcagtagcttacagaac------------gcaa-------------------
----
>_R_gnl|SRA|DRR000630.2702862.1 HWUSI-EAS1632R_0006_FC61F2Y:1:88:5614:2736..
cgccaacaaatcagtagcttacagaac------------gcaa-------------------
----
>gnl|SRA|DRR000630.1576720.2 HWUSI-EAS1632R_0006_FC61F2Y:1:50:2617:8042..
cgccaacaaatcagtagcttacagaac------------gcaa-------------------
----
>_R_gnl|SRA|DRR000630.126959.2 HWUSI-EAS1632R_0006_FC61F2Y:1:4:11912:16755..
cgccaacaaatcagtagcttacagaac------------gcaa-------------------
----
>gnl|SRA|DRR000630.11735356.2 HWUSI-EAS1632R_0006_FC61F2Y:3:119:5553:2548..
cgcccacaaatcagtagcttacagaac------------gcaaacag---gaccatagaga
ccgg--------------------------

DRX000334
>control ..
acagaa---------------cgcaaacagtaggaccatagtgggggattc---
>_R_gnl|SRA|DRR000628.8179316.1 HWUSI-EAS1632R_0006_FC61F2Y:3:21:7280:6124.
acagaa---------------cgcaaacagtaggaccatagtgggggattc---
>_R_gnl|SRA|DRR000628.7114115.2 HWUSI-EAS1632R_0006_FC61F2Y:2:109:14679:7759.
acagaa---------------cgcaaacagtaggaccatagtgggggattc---
>_R_gnl|SRA|DRR000628.6615980.2 HWUSI-EAS1632R_0006_FC61F2Y:2:94:10771:11727.
acagaa---------------cgcaaacagtaggaccatagtgggggattc---
>_R_gnl|SRA|DRR000628.6130562.1 HWUSI-EAS1632R_0006_FC61F2Y:2:79:6100:1326.
acagaa---------------cgcaaacagtaggaccatagtgggggattc---
>_R_gnl|SRA|DRR000627.5279894.2 HWUSI-EAS1632R_0006_FC61F2Y:2:51:5315:11801.
acagaa---------------cgcaaacagtaggaccatagtgggggattc---
>gnl|SRA|DRR000628.7377961.2 HWUSI-EAS1632R_0006_FC61F2Y:2:117:12323:3055.
acagaa---------------cgcaaacagtaggaccatcgtgggggattc---
>gnl|SRA|DRR000628.9272937.2 HWUSI-EAS1632R_0006_FC61F2Y:3:55:14751:3050.
acagaa---------------cgcaaacagtaggaccatagtgggggatt----
>gnl|SRA|DRR000628.5553257.2 HWUSI-EAS1632R_0006_FC61F2Y:2:59:13355:10604.
acagaa---------------cgcaaacagtaggaccatagtgggggatt----
>_R_gnl|SRA|DRR000629.9187620.1 HWUSI-EAS1632R_0006_FC61F2Y:2:73:13397:1339.
acagaa---------------cgcaaacagtaggaccatagtgggggattcta-
>_R_gnl|SRA|DRR000629.8126056.1 HWUSI-EAS1632R_0006_FC61F2Y:2:49:15381:11986.
acagaa---------------cgcaaacagtaggaccatagtgggggattctat
>_R_gnl|SRA|DRR000628.2969110.1 HWUSI-EAS1632R_0006_FC61F2Y:1:99:11581:1476.
acagaa---------------cgcaaacagtaggaccatagtgggggattctat
>_R_gnl|SRA|DRR000628.7377961.1 HWUSI-EAS1632R_0006_FC61F2Y:2:117:12323:3055.
acagaa---------------cgcaaacagtaggaccatagtgggggattctat
>_R_gnl|SRA|DRR000628.2671540.1 HWUSI-EAS1632R_0006_FC61F2Y:1:90:5257:7290.
acagaa---------------cgcaaacagtaggaccatagtgggggattctat
>_R_gnl|SRA|DRR000628.10063549.2 HWUSI-EAS1632R_0006_FC61F2Y:3:81:16151:4654.
acagaa---------------cgcaaacagtaggaccatagtgggggattctat

>_R_gnl|SRA|DRR000628.9272937.1 HWUSI-EAS1632R_0006_FC61F2Y:3:55:14751:3050.
acagaa--------------cgcaaacagtaggaccatagtgggggattctat
>_R_gnl|SRA|DRR000628.8595896.1 HWUSI-EAS1632R_0006_FC61F2Y:3:33:18386:6024.
acagaa--------------cgcaaacagtaggaccatagtgggggattctat
>_R_gnl|SRA|DRR000628.4160646.1 HWUSI-EAS1632R_0006_FC61F2Y:2:15:9859:9963.
acagaa--------------cgcaaacagtaggaccatagtgggggattctat
>_R_gnl|SRA|DRR000628.810578.1 HWUSI-EAS1632R_0006_FC61F2Y:1:25:15056:11853.
acagaa--------------cgcaaacagtaggaccatagtgggtgattctat
>_R_gnl|SRA|DRR000628.5553257.1 HWUSI-EAS1632R_0006_FC61F2Y:2:59:13355:10604.
acagaa--------------cgcaaacagtaggaccatagtgggggattctat
>gnl|SRA|DRR000627.6077609.2 HWUSI-EAS1632R_0006_FC61F2Y:2:78:14396:6875.
acagaa--------------cgcaaacagtaggaccatagtgggggattctat
>_R_gnl|SRA|DRR000628.9564830.2 HWUSI-EAS1632R_0006_FC61F2Y:3:65:5957:18559.
acagaa--------------cgcaaacagtaggaccatagtgggggattctat
>_R_gnl|SRA|DRR000628.10506932.1 HWUSI-EAS1632R_0006_FC61F2Y:3:95:10898:8444.
acagaa--------------cgcaaacagtaggaccatagtgggggattctat
>_R_gnl|SRA|DRR000628.9416645.1 HWUSI-EAS1632R_0006_FC61F2Y:3:60:8819:12632.
acagaa--------------cgcaaacagtaggaccatagtgggggattctat
>_R_gnl|SRA|DRR000628.4465691.1 HWUSI-EAS1632R_0006_FC61F2Y:2:24:13731:1654.
acagaa--------------cgcaaacagtaggaccatagtgggggattctat
>_R_gnl|SRA|DRR000627.10938398.1 HWUSI-EAS1632R_0006_FC61F2Y:3:110:9145:1919.
acagaa--------------cgcaaacagtaggaccatagtgggggattctat
>_R_gnl|SRA|DRR000627.8861293.2 HWUSI-EAS1632R_0006_FC61F2Y:3:42:17722:13599.
acagaa--------------cgcaaacagtaggaccatagtgggggattctat
>_R_gnl|SRA|DRR000627.9399832.2 HWUSI-EAS1632R_0006_FC61F2Y:3:61:3219:2291.
gcagaa--------------cgcaaacagtaggaccatagtgggggattctat
>gnl|SRA|DRR000628.7210129.2 HWUSI-EAS1632R_0006_FC61F2Y:2:112:12819:20058.
acagaa--------------cgcaaacagtaggaccatagtgggggattctat
>_R_gnl|SRA|DRR000628.7210129.1 HWUSI-EAS1632R_0006_FC61F2Y:2:112:12819:20058.
acagaa--------------cgcaaacagtaggaccatagtgggggattctat
>_R_gnl|SRA|DRR000628.3791787.1 HWUSI-EAS1632R_0006_FC61F2Y:2:4:9113:7722.
acagaa--------------cgcaaacagtaggaccatagtgggggattctat
>_R_gnl|SRA|DRR000628.7937415.1 HWUSI-EAS1632R_0006_FC61F2Y:3:14:2147:17622.
acagaa--------------cgcaaacagtaggaccatagtgggggattctat
>_R_gnl|SRA|DRR000628.3171852.1 HWUSI-EAS1632R_0006_FC61F2Y:1:105:16955:16023.
acagaa--------------cgcaaacagtaggaccatagtgggggattctat
>_R_gnl|SRA|DRR000627.525937.1 HWUSI-EAS1632R_0006_FC61F2Y:1:16:12409:18482.
acagca--------------cgcaaacagtaggaccatagtgggggattctat
>_R_gnl|SRA|DRR000627.2206647.2 HWUSI-EAS1632R_0006_FC61F2Y:1:74:6090:1028.
acagaa--------------cgcaaacagtaggaccatagtgggggattctat
>_R_gnl|SRA|DRR000628.10088490.2 HWUSI-EAS1632R_0006_FC61F2Y:3:82:12094:5517.
acagaa--------------cgcaaacagtaggaccatagtgggggattcta-
>_R_gnl|SRA|DRR000628.3830370.1 HWUSI-EAS1632R_0006_FC61F2Y:2:5:11704:15054.
acagaa--------------cgcaaacagtaggaccatagtgggggattcta-
>_R_gnl|SRA|DRR000628.4620930.1 HWUSI-EAS1632R_0006_FC61F2Y:2:29:9045:8452.
acagaa--------------cgcaaacagtaggaccatagtgggggattcta-
>gnl|SRA|DRR000628.7937415.2 HWUSI-EAS1632R_0006_FC61F2Y:3:14:2147:17622.
acagaa--------------cgcaaacagtaggaccatagtgggggattcta-
>gnl|SRA|DRR000628.3171852.2 HWUSI-EAS1632R_0006_FC61F2Y:1:105:16955:16023.
acagaa--------------cgcaaacagtaggaccatagtgggggattcta-
>gnl|SRA|DRR000627.525937.2 HWUSI-EAS1632R_0006_FC61F2Y:1:16:12409:18482.

acagaa--------------cgcaaacagtaggaccatagtgggggattcta-
>gnl|SRA|DRR000628.3276617.2 HWUSI-EAS1632R_0006_FC61F2Y:1:109:3041:5869.
acagaa--------------cgcaaacagtaggaccatagtgggggattcta-
>_R_gnl|SRA|DRR000628.6148544.1 HWUSI-EAS1632R_0006_FC61F2Y:2:79:16811:9779.
acagaa--------------cgcaaacagtaggaccatagtgggggat-----
>_R_gnl|SRA|DRR000628.6059666.1 HWUSI-EAS1632R_0006_FC61F2Y:2:76:18431:8033.
acagaa--------------cgcaaacagtaggaccatagtgggggat-----
>_R_gnl|SRA|DRR000628.1041187.2 HWUSI-EAS1632R_0006_FC61F2Y:1:33:3649:3873.
acagaa--------------cgcaaacagtaggaccatagcgggggat-----
>_R_gnl|SRA|DRR000628.1401789.2 HWUSI-EAS1632R_0006_FC61F2Y:1:46:5870:8848.
acagaa--------------cgcaaacagtaggaccatagtgggggga------
>_R_gnl|SRA|DRR000628.8356518.2 HWUSI-EAS1632R_0006_FC61F2Y:3:26:13366:18278.
acagaa--------------cgcaaacagtaggaccatagtggggg-------
>_R_gnl|SRA|DRR000628.5069961.1 HWUSI-EAS1632R_0006_FC61F2Y:2:43:8262:2805.
acagaa--------------cgcaaacagtaggaccatagtggggg-------
>_R_gnl|SRA|DRR000628.3692265.2 HWUSI-EAS1632R_0006_FC61F2Y:2:1:10677:15589.
acagaa--------------cgcaaacagtaggaccatagtggggg-------
>_R_gnl|SRA|DRR000627.7411885.1 HWUSI-EAS1632R_0006_FC61F2Y:2:119:10742:13199.
acagaa--------------cgcaaacagtaggaccatagtggggg-------
>_R_gnl|SRA|DRR000627.2021568.2 HWUSI-EAS1632R_0006_FC61F2Y:1:67:17108:14354.
acagaa--------------cgcaaacagtaggaccatagtggggg-------
>_R_gnl|SRA|DRR000628.10789840.2 HWUSI-EAS1632R_0006_FC61F2Y:3:104:3587:20599.
acagaa--------------cgcaaacagtaggaccatagtgggg--------
>_R_gnl|SRA|DRR000628.9349739.1 HWUSI-EAS1632R_0006_FC61F2Y:3:58:5348:7801.
acagaa--------------cgcaaacagtaggaccatagtgggg--------
>_R_gnl|SRA|DRR000628.9232860.2 HWUSI-EAS1632R_0006_FC61F2Y:3:54:9519:7291.
acagaa--------------cgcaaacagtaggaccatagtgggg--------
>_R_gnl|SRA|DRR000628.7884651.2 HWUSI-EAS1632R_0006_FC61F2Y:3:12:10046:6544.
acagaa--------------cgcaaacagtaggaccatagtgggg--------
>_R_gnl|SRA|DRR000628.5835868.1 HWUSI-EAS1632R_0006_FC61F2Y:2:69:4127:1081.
acagaa--------------cgcaaacagtaggaccatagtgggg--------
>_R_gnl|SRA|DRR000628.4610805.1 HWUSI-EAS1632R_0006_FC61F2Y:2:29:3476:19488.
acagaa--------------cgcaaacagtaggaccatagtgggg--------
>_R_gnl|SRA|DRR000628.3430854.1 HWUSI-EAS1632R_0006_FC61F2Y:1:113:15899:9314.
acagaa--------------cgcaaacagtaggaccatagtgggg--------
>_R_gnl|SRA|DRR000628.2954469.2 HWUSI-EAS1632R_0006_FC61F2Y:1:99:3373:7768.
acagaa--------------cgcaaacagtaggaccatagtgggg--------
>_R_gnl|SRA|DRR000628.1200070.1 HWUSI-EAS1632R_0006_FC61F2Y:1:38:5738:12628.
acagaa--------------cgcaaacagtaggaccatagtgggg--------
>_R_gnl|SRA|DRR000627.9000363.1 HWUSI-EAS1632R_0006_FC61F2Y:3:47:18049:17336.
acagaa--------------cgcaaacagtaggaccatagtgggg--------
>_R_gnl|SRA|DRR000627.9000354.1 HWUSI-EAS1632R_0006_FC61F2Y:3:47:18043:6802.
acagaa--------------cgcaaacagtaggaccatagtgggg--------
>_R_gnl|SRA|DRR000627.6693290.1 HWUSI-EAS1632R_0006_FC61F2Y:2:98:4320:5616.
acagaa--------------cgcaaacagtaggaccatagtgggg--------
>_R_gnl|SRA|DRR000627.2089745.2 HWUSI-EAS1632R_0006_FC61F2Y:1:70:5822:16406.
acagaa--------------cgcaaacagtaggaccatagtgggg--------
>_R_gnl|SRA|DRR000628.1668207.1 HWUSI-EAS1632R_0006_FC61F2Y:1:55:5766:6580.
acagaa--------------cgcagacagtaggaccatagtgggg--------
>_R_gnl|SRA|DRR000628.1059231.2 HWUSI-EAS1632R_0006_FC61F2Y:1:33:14063:6460.
acagaa--------------cgcaaacagtaggaccagagtgggg--------

>_R_gnl|SRA|DRR000628.4629815.2 HWUSI-EAS1632R_0006_FC61F2Y:2:29:14052:17715.
acagaa--------------cgcaaacagtaggaccatagtgggg--------
>_R_gnl|SRA|DRR000628.3007706.2 HWUSI-EAS1632R_0006_FC61F2Y:1:100:15221:20771.
acagaa--------------cgcaaacagtaggaccatagtgggg--------
>_R_gnl|SRA|DRR000628.5322799.2 HWUSI-EAS1632R_0006_FC61F2Y:2:52:2831:10816.
acagaa--------------cgcaaacagtaggaccatagtggg---------
>_R_gnl|SRA|DRR000628.1597233.1 HWUSI-EAS1632R_0006_FC61F2Y:1:52:16362:8705.
acagaa--------------cgcaaacagtaggaccatagtggg---------
>_R_gnl|SRA|DRR000628.9696385.2 HWUSI-EAS1632R_0006_FC61F2Y:3:69:10944:6239.
acagaa--------------cgcaaacagtaggaccatagtgg----------
>_R_gnl|SRA|DRR000628.6235599.2 HWUSI-EAS1632R_0006_FC61F2Y:2:82:12346:11038.
acagaa--------------cgcaaacagtaggaccatagtgg----------
>_R_gnl|SRA|DRR000628.5793914.1 HWUSI-EAS1632R_0006_FC61F2Y:2:67:15458:8380.
acagaa--------------cgcaaacagtaggaccatagtgg----------
>_R_gnl|SRA|DRR000628.5656232.2 HWUSI-EAS1632R_0006_FC61F2Y:2:63:4746:4705.
acagaa--------------cgcaaacagtaggaccatagtgg----------
>_R_gnl|SRA|DRR000628.4909095.2 HWUSI-EAS1632R_0006_FC61F2Y:2:38:6875:19087.
acagaa--------------cgcaaacagtaggaccatagtgg----------
>_R_gnl|SRA|DRR000628.391919.1 HWUSI-EAS1632R_0006_FC61F2Y:1:12:15349:20805.
acagaa--------------cgcaaacagtaggaccatagtgg----------
>_R_gnl|SRA|DRR000628.10072734.2 HWUSI-EAS1632R_0006_FC61F2Y:3:82:3021:18746.
acagaa--------------cgcaaacagtaggaccatagtg-----------
>_R_gnl|SRA|DRR000628.6459056.2 HWUSI-EAS1632R_0006_FC61F2Y:2:89:12797:9309.
acagaa--------------cgcaaacagtaggaccatagtg-----------
>_R_gnl|SRA|DRR000627.7504558.1 HWUSI-EAS1632R_0006_FC61F2Y:3:2:3240:5088.
acagaa--------------cgcaaacagtaggaccatagtg-----------
>_R_gnl|SRA|DRR000627.2580444.1 HWUSI-EAS1632R_0006_FC61F2Y:1:88:1543:10007.
acagaa--------------cgcaaacagtaggaccatagtg-----------
>gnl|SRA|DRR000629.14878844.2 HWUSI-EAS1632R_0006_FC61F2Y:3:67:9842:7913.
acagaa--------------cgcaaacagtaggaccatag-------------
>_R_gnl|SRA|DRR000628.5677037.1 HWUSI-EAS1632R_0006_FC61F2Y:2:63:17687:19453.
acagaa--------------cgcaaacagtaggaccatag-------------
>_R_gnl|SRA|DRR000628.4720782.2 HWUSI-EAS1632R_0006_FC61F2Y:2:32:10382:8026.
acagaa--------------cgcaaacagtaggaccatag-------------
>_R_gnl|SRA|DRR000628.4066803.2 HWUSI-EAS1632R_0006_FC61F2Y:2:12:13211:17812.
acagaa--------------cgcaaacagtaggaccatag-------------
>_R_gnl|SRA|DRR000628.3135180.2 HWUSI-EAS1632R_0006_FC61F2Y:1:104:14581:9065.
acagaa--------------cgcaaacagtaggaccatag-------------
>_R_gnl|SRA|DRR000628.632838.1 HWUSI-EAS1632R_0006_FC61F2Y:1:20:5573:2721.
acagaa--------------cgcaaacagtaggaccatag-------------
>_R_gnl|SRA|DRR000628.11142342.1 HWUSI-EAS1632R_0006_FC61F2Y:3:114:13518:3881.
acagaa--------------cgcaaac---aggaccatagtgggggattc---
>_R_gnl|SRA|DRR000628.10622620.1 HWUSI-EAS1632R_0006_FC61F2Y:3:99:2155:17991.
acagaa--------------cgcaaac---aggaccatagtgggggattc---
>_R_gnl|SRA|DRR000628.4126693.1 HWUSI-EAS1632R_0006_FC61F2Y:2:14:9514:12147.
acagaa--------------cgcaaac---aggaccatagtgggggattc---
>_R_gnl|SRA|DRR000628.575540.1 HWUSI-EAS1632R_0006_FC61F2Y:1:18:9733:10733.
acagaa--------------cgcaaac---aggaccatagtgggggattc---
>_R_gnl|SRA|DRR000628.10705556.2 HWUSI-EAS1632R_0006_FC61F2Y:3:101:11830:16538.
acagaa--------------cgcaaac---aggaccatagtgggggattct--
>_R_gnl|SRA|DRR000628.4786519.1 HWUSI-EAS1632R_0006_FC61F2Y:2:34:10770:11515.

acagaa--------------cgcaaac---aggaccatagtgggggattcta-
>_R_gnl|SRA|DRR000628.4692877.2 HWUSI-EAS1632R_0006_FC61F2Y:2:31:12971:14250.
acagaa--------------cgcaaac---aggaccatagtgggggattcta-
>_R_gnl|SRA|DRR000628.6283286.1 HWUSI-EAS1632R_0006_FC61F2Y:2:84:3238:3850.
acagaa--------------cgcaaac---aggaccatagtgggggattctat
>_R_gnl|SRA|DRR000628.4096496.1 HWUSI-EAS1632R_0006_FC61F2Y:2:13:11239:2876.
acagaa--------------cgcaaac---aggaccatagtgggggattctat
>_R_gnl|SRA|DRR000628.2263869.1 HWUSI-EAS1632R_0006_FC61F2Y:1:75:12284:9922.
acagaa--------------cgcaaac---aggaccatagtgggggattctat
>_R_gnl|SRA|DRR000627.9817641.2 HWUSI-EAS1632R_0006_FC61F2Y:3:75:8181:15300.
acagaa--------------cgcaaac---aggaccatagtgggggattctat
>_R_gnl|SRA|DRR000627.8061630.2 HWUSI-EAS1632R_0006_FC61F2Y:3:18:7716:1102.
acagaa--------------cgcaaac---aggaccatagtgggggattctat
>_R_gnl|SRA|DRR000628.1648044.1 HWUSI-EAS1632R_0006_FC61F2Y:1:54:11467:10541.
acagaa--------------cgcaaac---aggaccatagtggggcattctat
>gnl|SRA|DRR000627.7472887.2 HWUSI-EAS1632R_0006_FC61F2Y:3:1:5530:1056.
acagaa--------------cgcaaac---aggaccatagtgggggattctat
>_R_gnl|SRA|DRR000628.6530698.1 HWUSI-EAS1632R_0006_FC61F2Y:2:91:17260:4710.
acagaa--------------cgcaaac---aggaccatagtgggggat-----
>_R_gnl|SRA|DRR000628.6399443.2 HWUSI-EAS1632R_0006_FC61F2Y:2:87:15278:20046.
acagaa--------------cgcaaac---aggaccatagtgggggat-----
>_R_gnl|SRA|DRR000628.401615.2 HWUSI-EAS1632R_0006_FC61F2Y:1:13:2404:16860.
acagaa--------------cgcaaac---aggaccatagtgggggat-----
>_R_gnl|SRA|DRR000627.353062.2 HWUSI-EAS1632R_0006_FC61F2Y:1:11:8097:20323.
acagaa--------------cgcaaac---aggaccatagtgggggat-----
>_R_gnl|SRA|DRR000628.10540595.2 HWUSI-EAS1632R_0006_FC61F2Y:3:96:11283:6205.
acagaa--------------cgcaaac---aggaccatagtgggggaa-----
>_R_gnl|SRA|DRR000628.8622799.1 HWUSI-EAS1632R_0006_FC61F2Y:3:34:15168:13212.
acagaa--------------cgcaaac---aggaccatagtggggg-------
>_R_gnl|SRA|DRR000628.1666936.1 HWUSI-EAS1632R_0006_FC61F2Y:1:55:4928:12742.
acagaa--------------cgcaaac---aggaccatagtggggg-------
>_R_gnl|SRA|DRR000627.7961870.1 HWUSI-EAS1632R_0006_FC61F2Y:3:15:8150:19011.
acagaa--------------cgcaaac---aggaccatagtggggg-------
>_R_gnl|SRA|DRR000628.10569510.2 HWUSI-EAS1632R_0006_FC61F2Y:3:97:9057:14120.
acagaa--------------cgcaaac---aggaccatagtgggg--------
>_R_gnl|SRA|DRR000628.10220269.2 HWUSI-EAS1632R_0006_FC61F2Y:3:86:14187:9279.
acagaa--------------cgcaaac---aggaccatagtgggg--------
>_R_gnl|SRA|DRR000628.9430109.1 HWUSI-EAS1632R_0006_FC61F2Y:3:60:16982:14539.
acagaa--------------cgcaaac---aggaccatagtgggg--------
>_R_gnl|SRA|DRR000628.8080595.1 HWUSI-EAS1632R_0006_FC61F2Y:3:18:7599:13261.
acagaa--------------cgcaaac---aggaccatagtgggg--------
>_R_gnl|SRA|DRR000628.7316637.1 HWUSI-EAS1632R_0006_FC61F2Y:2:115:15581:17633.
acagaa--------------cgcaaac---aggaccatagtgggg--------
>_R_gnl|SRA|DRR000628.5825944.2 HWUSI-EAS1632R_0006_FC61F2Y:2:68:16430:17102.
acagaa--------------cgcaaac---aggaccatagtgggg--------
>_R_gnl|SRA|DRR000628.4638735.2 HWUSI-EAS1632R_0006_FC61F2Y:2:29:19259:8789.
acagaa--------------cgcaaac---aggaccatagtgggg--------
>_R_gnl|SRA|DRR000628.4549517.1 HWUSI-EAS1632R_0006_FC61F2Y:2:27:5790:12530.
acagaa--------------cgcaaac---aggaccatagtgggg--------
>_R_gnl|SRA|DRR000628.4493021.1 HWUSI-EAS1632R_0006_FC61F2Y:2:25:10529:2680.
acagaa--------------cgcaaac---aggaccatagtgggg--------

>_R_gnl|SRA|DRR000628.4252647.1 HWUSI-EAS1632R_0006_FC61F2Y:2:18:5538:7289.
acagaa--------------cgcaaac---aggaccatagtgggg--------
>_R_gnl|SRA|DRR000628.3250363.1 HWUSI-EAS1632R_0006_FC61F2Y:1:108:6539:8285.
acagaa--------------cgcaaac---aggaccatagtgggg--------
>_R_gnl|SRA|DRR000627.6588257.1 HWUSI-EAS1632R_0006_FC61F2Y:2:94:18557:2141.
acagaa--------------cgcaaac---aggaccatagtgggg--------
>_R_gnl|SRA|DRR000627.2746689.2 HWUSI-EAS1632R_0006_FC61F2Y:1:93:7958:13350.
acagaa--------------cgcaaac---aggaccatagtgggg--------
>_R_gnl|SRA|DRR000628.8744288.1 HWUSI-EAS1632R_0006_FC61F2Y:3:38:10343:15241.
acagaa--------------cgcaaac---aggaccatagtggga--------
>_R_gnl|SRA|DRR000628.10099157.2 HWUSI-EAS1632R_0006_FC61F2Y:3:82:18381:10501.
acagaa--------------cgcaaac---aggaccatagtgggg--------
>_R_gnl|SRA|DRR000628.6782680.1 HWUSI-EAS1632R_0006_FC61F2Y:2:99:13513:18233.
acagaa--------------cgcaaac---aggaccatagtggg---------
>_R_gnl|SRA|DRR000628.5757492.1 HWUSI-EAS1632R_0006_FC61F2Y:2:66:11598:3830.
acagaa--------------cgcaaac---aggaccatagtggg---------
>_R_gnl|SRA|DRR000628.10146306.2 HWUSI-EAS1632R_0006_FC61F2Y:3:84:8468:14904.
acagaa--------------cgcaaac---aggaccatagtgg----------
>_R_gnl|SRA|DRR000628.5829808.1 HWUSI-EAS1632R_0006_FC61F2Y:2:68:19025:19055.
acagaa--------------cgcaaac---aggaccatagtgg----------
>_R_gnl|SRA|DRR000628.5248005.1 HWUSI-EAS1632R_0006_FC61F2Y:2:49:13239:3115.
acagaa--------------cgcaaac---aggaccatagtgg----------
>_R_gnl|SRA|DRR000628.3402420.1 HWUSI-EAS1632R_0006_FC61F2Y:1:112:18600:6534.
acagaa--------------cgcaaac---aggaccatagtgg----------
>_R_gnl|SRA|DRR000628.5810812.1 HWUSI-EAS1632R_0006_FC61F2Y:2:68:7331:16737.
acagaa--------------cgcaaac---aggaccatagtag----------
>gnl|SRA|DRR000628.3484701.2 HWUSI-EAS1632R_0006_FC61F2Y:1:115:9200:8383.
acagaa--------------cgcaaac---aggaccatagtg-----------
>_R_gnl|SRA|DRR000627.7218911.1 HWUSI-EAS1632R_0006_FC61F2Y:2:113:18990:3000.
acagaa--------------cgcaaac---aggaccatagtg-----------
>_R_gnl|SRA|DRR000628.6775445.1 HWUSI-EAS1632R_0006_FC61F2Y:2:99:9467:15403.
acagaa--------------cgcaaac---aggaccatagtc-----------
>gnl|SRA|DRR000627.979163.1 HWUSI-EAS1632R_0006_FC61F2Y:1:30:16251:2745.
agagaa--------------cgcaaacagtaggaccatagtgggggatt----
>gnl|SRA|DRR000629.9187620.2 HWUSI-EAS1632R_0006_FC61F2Y:2:73:13397:1339.
acagaa--------------cgcaaacagtaggaccatagtgggggattctaa
>_R_gnl|SRA|DRR000627.6632739.1 HWUSI-EAS1632R_0006_FC61F2Y:2:96:6820:13400.
acagaa--------------cgcaaacagtaggaccatagtgggggc------
>gnl|SRA|DRR000629.6346495.2 HWUSI-EAS1632R_0006_FC61F2Y:2:13:1755:12016.
acagaa--------------cgcaaacagtaggaccatagtgggggattctat
>gnl|SRA|DRR000628.4776875.1 HWUSI-EAS1632R_0006_FC61F2Y:2:34:5332:14140.
acagaa--------------cgcaaacagtaggaccatagtgggggattctat
>_R_gnl|SRA|DRR000628.3706181.2 HWUSI-EAS1632R_0006_FC61F2Y:2:1:18177:10544.
acagaa--------------cgcaaacagtaggaccatagtgggggattctat
>_R_gnl|SRA|DRR000627.8929418.2 HWUSI-EAS1632R_0006_FC61F2Y:3:45:2428:6422.
acagaa--------------cgcaaacagtaggaccatagtgggggattctat
>_R_gnl|SRA|DRR000628.3013240.1 HWUSI-EAS1632R_0006_FC61F2Y:1:100:18528:12377.
acagaa--------------cgcaaacagtaggaccatagtgggggattctat
>_R_gnl|SRA|DRR000628.9268944.1 HWUSI-EAS1632R_0006_FC61F2Y:3:55:12394:4884.
acagaa--------------cgcaaacagtaggaccatagtgggggattctat
>_R_gnl|SRA|DRR000627.11206469.1 HWUSI-EAS1632R_0006_FC61F2Y:3:118:5627:8051.

acagaa--------------cgcaaacagtaggaccatagtgggggattctat
>gnl|SRA|DRR000627.9734326.1 HWUSI-EAS1632R_0006_FC61F2Y:3:72:8504:18022.
acagaa--------------cgcaaacagtaggaccatagtgggggattctat
>_R_gnl|SRA|DRR000627.3512843.1 HWUSI-EAS1632R_0006_FC61F2Y:1:116:17611:17990.
acagaa--------------cgcaaacagtaggaccatagtgggggattctat
>_R_gnl|SRA|DRR000628.5337735.2 HWUSI-EAS1632R_0006_FC61F2Y:2:52:11620:8175.
acagaa--------------cgcaaacagtaggaccatagtgggggattctat
>_R_gnl|SRA|DRR000628.5010207.2 HWUSI-EAS1632R_0006_FC61F2Y:2:41:10018:19218.
acagaa--------------cgcaaacagtaggaccatagtgggggattctat
>_R_gnl|SRA|DRR000628.3474629.2 HWUSI-EAS1632R_0006_FC61F2Y:1:115:3580:2103.
acagaa--------------cgcaaacagtaggaccatagtgggggattctat
>gnl|SRA|DRR000628.2487233.2 HWUSI-EAS1632R_0006_FC61F2Y:1:84:7458:9007.
acagaa--------------cgcaaacagtaggaccatagtgggggattctat
>_R_gnl|SRA|DRR000628.10306032.1 HWUSI-EAS1632R_0006_FC61F2Y:3:89:8000:19780.
acagaa--------------cgcaaacagtaggaccatagtgggggattctat
>_R_gnl|SRA|DRR000627.6437462.2 HWUSI-EAS1632R_0006_FC61F2Y:2:90:5348:2191.
acagaa--------------cgcaaacagtaggaccatagtgggggattctat
>gnl|SRA|DRR000628.10759233.1 HWUSI-EAS1632R_0006_FC61F2Y:3:103:4939:15943.
acagaa--------------cgcaaacagtaggaccatagtgggggattctat
>gnl|SRA|DRR000628.7435307.1 HWUSI-EAS1632R_0006_FC61F2Y:2:119:6473:10827.
acagaa--------------cgcaaacagtaggaccatagtgggggattctat
>gnl|SRA|DRR000628.6721747.2 HWUSI-EAS1632R_0006_FC61F2Y:2:97:15915:10439.
acagaa--------------cgcaaacagtaggaccatagtgggggattctat
>_R_gnl|SRA|DRR000628.3642414.1 HWUSI-EAS1632R_0006_FC61F2Y:1:120:2855:20370.
acagaa--------------cgcaaacagtaggaccatagtgggggattctat
>_R_gnl|SRA|DRR000628.2614335.2 HWUSI-EAS1632R_0006_FC61F2Y:1:88:8421:2362.
acagaa--------------cgcaaacagtaggaccatagtgggggattctat
>_R_gnl|SRA|DRR000627.9208645.1 HWUSI-EAS1632R_0006_FC61F2Y:3:54:14782:13752.
acagaa--------------cgcaaacagtaggaccatagtgggggattctat
>gnl|SRA|DRR000627.6437462.1 HWUSI-EAS1632R_0006_FC61F2Y:2:90:5348:2191.
acagaa--------------cgcaaacagtaggaccatagtgggggattctat
>_R_gnl|SRA|DRR000628.5852540.1 HWUSI-EAS1632R_0006_FC61F2Y:2:69:14185:5817.
acataa--------------cgcaaacagtaggaccatagtgggggattctat
>_R_gnl|SRA|DRR000628.8819357.2 HWUSI-EAS1632R_0006_FC61F2Y:3:40:16148:7279.
acagaa--------------cgcaaacag---gaccatagtgggggattctat
>_R_gnl|SRA|DRR000628.8425712.2 HWUSI-EAS1632R_0006_FC61F2Y:3:28:15162:2355.
acagaa--------------cgcaaacag---gaccatagtgggggattctat
>_R_gnl|SRA|DRR000628.2269870.2 HWUSI-EAS1632R_0006_FC61F2Y:1:75:15930:11160.
acagaa--------------cgcaaacag---gaccatagtgggggattctat
>_R_gnl|SRA|DRR000628.3037970.2 HWUSI-EAS1632R_0006_FC61F2Y:1:101:14200:12779.
acagaa--------------cgcaaacag---gaccatagtgggggattctat
>_R_gnl|SRA|DRR000628.2572349.1 HWUSI-EAS1632R_0006_FC61F2Y:1:87:2208:10865.
acagaa--------------cgcaaacag---gaccatagtgggggattctat
>gnl|SRA|DRR000627.6824552.1 HWUSI-EAS1632R_0006_FC61F2Y:2:102:3962:3226.
acagaa--------------cgcaaacag---gaccatagtgggggattctat
>_R_gnl|SRA|DRR000627.5628536.2 HWUSI-EAS1632R_0006_FC61F2Y:2:63:3635:18407.
acagaa--------------cgcaaacag---gaccatagtgggggattctat
>_R_gnl|SRA|DRR000627.8991589.1 HWUSI-EAS1632R_0006_FC61F2Y:3:47:12072:14412.
acagaa--------------cgcaaacag---gaccatagtgggggattctat
>_R_gnl|SRA|DRR000627.4191955.1 HWUSI-EAS1632R_0006_FC61F2Y:2:16:13305:20398.
acagaa--------------cgcaaacag---gaccatagtgggggattctat

>_R_gnl|SRA|DRR000627.4081444.1 HWUSI-EAS1632R_0006_FC61F2Y:2:13:8061:2559.
acagaa--------------cgcaaacag---gaccatagtgggggattctat
>_R_gnl|SRA|DRR000628.5073573.2 HWUSI-EAS1632R_0006_FC61F2Y:2:43:10367:18535.
acagaa--------------cgcaaacag---gaccatagtgggggattctat
>_R_gnl|SRA|DRR000628.3669317.2 HWUSI-EAS1632R_0006_FC61F2Y:1:120:16655:3618.
acagaa--------------cgcaaacag---gaccatagtgggggattctat
>_R_gnl|SRA|DRR000628.4528290.1 HWUSI-EAS1632R_0006_FC61F2Y:2:26:12090:16633.
acagaa--------------cgcaaacag---gaccatagtgggggattctat
>_R_gnl|SRA|DRR000628.11235505.1 HWUSI-EAS1632R_0006_FC61F2Y:3:117:8537:2210.
acagaa--------------cgcaaacagtaggaccatagtgggggattctat
>_R_gnl|SRA|DRR000628.9779596.2 HWUSI-EAS1632R_0006_FC61F2Y:3:72:4819:18528.
acagaa--------------cgcaaacagtaggaccatagtgggggattctat
>_R_gnl|SRA|DRR000628.7486293.1 HWUSI-EAS1632R_0006_FC61F2Y:2:120:15003:2923.
acagaa--------------cgcaaacagtaggaccatagtgggggattctat
>_R_gnl|SRA|DRR000627.2103461.1 HWUSI-EAS1632R_0006_FC61F2Y:1:70:14457:5845.
acagaa--------------cgcaaacagtaggaccatagtgggggattctat
>_R_gnl|SRA|DRR000628.11257143.2 HWUSI-EAS1632R_0006_FC61F2Y:3:118:1938:15906.
acagaa--------------cgcaaacagtaggaccatagtgggggattctat
>_R_gnl|SRA|DRR000628.1627330.2 HWUSI-EAS1632R_0006_FC61F2Y:1:53:16847:19568.
acagaa--------------cgcaaacagtcggaccatagtgggggattctat
>_R_gnl|SRA|DRR000628.4891958.2 HWUSI-EAS1632R_0006_FC61F2Y:2:37:15548:11464.
acagaa--------------cgcaaacagtaggaccatagtgggggattctat
>gnl|SRA|DRR000627.10468609.1 HWUSI-EAS1632R_0006_FC61F2Y:3:96:5697:13199.
acagaa--------------cgcaaacagtaggaccatagtgggggattctat
>gnl|SRA|DRR000627.28797.1 HWUSI-EAS1632R_0006_FC61F2Y:1:1:16443:20335.
acagaa--------------cgcaaacagtaggaccatagtgggggattctat
>gnl|SRA|DRR000627.4142644.2 HWUSI-EAS1632R_0006_FC61F2Y:2:15:4615:17241.
acagaa--------------cgcaaacagtaggaccatagtgggggattctat
>gnl|SRA|DRR000627.8981841.2 HWUSI-EAS1632R_0006_FC61F2Y:3:47:5727:20444.
acagaa--------------cgcaaacagtaggaccatagtgggggattctat
>_R_gnl|SRA|DRR000628.11004822.1 HWUSI-EAS1632R_0006_FC61F2Y:3:110:11868:11732.
acagaa--------------cgcaaacag---gaccatagtgggggattctat
>_R_gnl|SRA|DRR000628.3487863.1 HWUSI-EAS1632R_0006_FC61F2Y:1:115:10886:4289.
acagaa--------------cgcaaacag---gaccatagtgggggattctat
>_R_gnl|SRA|DRR000628.10683708.1 HWUSI-EAS1632R_0006_FC61F2Y:3:100:18101:11975.
acagaa--------------cgcaaacag---gaccatagtgggggattctat
>_R_gnl|SRA|DRR000628.10326486.1 HWUSI-EAS1632R_0006_FC61F2Y:3:90:1375:4202.
acagaa--------------cgcaaacag---gaccatagtgggggattctat
>_R_gnl|SRA|DRR000628.5208811.2 HWUSI-EAS1632R_0006_FC61F2Y:2:48:8558:10345.
acagaa--------------cgcaaacag---gaccatagtgggggattctat
>gnl|SRA|DRR000627.10664205.1 HWUSI-EAS1632R_0006_FC61F2Y:3:102:5245:9036.
acagaa--------------cgcaaacag---gaccatagtgggggattctat
>_R_gnl|SRA|DRR000627.9185399.1 HWUSI-EAS1632R_0006_FC61F2Y:3:53:19113:20079.
acagaa--------------cgcaaacag---gaccatagtgggggattctat
>gnl|SRA|DRR000627.8115268.2 HWUSI-EAS1632R_0006_FC61F2Y:3:19:18848:17629.
acagaa--------------cgcaaacag---gaccatagtgggggattctat
>_R_gnl|SRA|DRR000627.519462.1 HWUSI-EAS1632R_0006_FC61F2Y:1:16:8808:2658.
acagaa--------------cgcaaacag---gaccatagtgcgggattctat
>_R_gnl|SRA|DRR000628.1948441.2 HWUSI-EAS1632R_0006_FC61F2Y:1:65:1103:16537.
acagaa--------------cgcaaacag---gaccatagtgggggattctat
>gnl|SRA|DRR000627.4081444.2 HWUSI-EAS1632R_0006_FC61F2Y:2:13:8061:2559.

acagaa--------------cgcaaacag---gaccatagtgggggattctat
>gnl|SRA|DRR000628.349244.2 HWUSI-EAS1632R_0006_FC61F2Y:1:11:10002:3350.
acagca--------------cgcaaacag---gaccatagtgggggattctat
>_R_gnl|SRA|DRR000627.1577131.2 HWUSI-EAS1632R_0006_FC61F2Y:1:52:2901:20697.
acagaa--------------cgcaaacag---gaccatagcgggggattctat
>gnl|SRA|DRR000627.1577131.1 HWUSI-EAS1632R_0006_FC61F2Y:1:52:2901:20697.
acagaa--------------cgcaaacag---gaccatagggggggattctat
>gnl|SRA|DRR000628.4822221.2 HWUSI-EAS1632R_0006_FC61F2Y:2:35:12544:19246.
acagaa--------------cgcaaacag---gaccatagtgggggattctat
>_R_gnl|SRA|DRR000628.4822221.1 HWUSI-EAS1632R_0006_FC61F2Y:2:35:12544:19246.
acagaa--------------cgcaaacag---gaccatagtgggggattctat
>gnl|SRA|DRR000628.2340128.2 HWUSI-EAS1632R_0006_FC61F2Y:1:78:3520:19108.
acagaa--------------cgcaaacag---gaccatagtgggggattctat
>gnl|SRA|DRR000627.7033068.2 HWUSI-EAS1632R_0006_FC61F2Y:2:108:9566:5473.
acagaa--------------cgcaaacagtaggaccatagtgggggattctat
>_R_gnl|SRA|DRR000628.376097.1 HWUSI-EAS1632R_0006_FC61F2Y:1:12:6593:1185.
acagaa--------------cgcaaacag---gaacatagtgggggattctat
>gnl|SRA|DRR000628.1927208.2 HWUSI-EAS1632R_0006_FC61F2Y:1:64:6163:20449.
acagaa--------------cgcaaacagtaggaccatagtgggggattctat
>_R_gnl|SRA|DRR000628.678004.2 HWUSI-EAS1632R_0006_FC61F2Y:1:21:12809:1258.
acagaa--------------cgcaaacag---gaccatagtggggga------
>_R_gnl|SRA|DRR000628.9031724.2 HWUSI-EAS1632R_0006_FC61F2Y:3:48:1785:5454.
acagaa--------------cgcaaacag---gaccatagtggggg-------
>_R_gnl|SRA|DRR000628.1020939.2 HWUSI-EAS1632R_0006_FC61F2Y:1:32:10325:8866.
acagaa--------------cgcaaacag---gaccatagtag----------
>gnl|SRA|DRR000627.3232384.1 HWUSI-EAS1632R_0006_FC61F2Y:1:108:10136:13988.
acagaa--------------cgcaaacag---gaccatagtgggggattctat
>_R_gnl|SRA|DRR000628.3885176.2 HWUSI-EAS1632R_0006_FC61F2Y:2:7:5179:9421.
acagaa--------------cgcaaacag---gaccatagtgggggattctat
>_R_gnl|SRA|DRR000628.9477144.1 HWUSI-EAS1632R_0006_FC61F2Y:3:62:8351:6142.
acagaa--------------cgcaaacagtaggacca----------------
>_R_gnl|SRA|DRR000628.3092504.1 HWUSI-EAS1632R_0006_FC61F2Y:1:103:8756:18942.
acagaa--------------cgcaaacagtaggacca----------------
>_R_gnl|SRA|DRR000627.10517347.1 HWUSI-EAS1632R_0006_FC61F2Y:3:97:14840:11120.
acagaa--------------cgcaaacagtaggacca----------------
>_R_gnl|SRA|DRR000628.11360657.1 HWUSI-EAS1632R_0006_FC61F2Y:3:120:19827:11523.
acagaa--------------cgcaaac---aggaccatag-------------
>_R_gnl|SRA|DRR000628.10963174.2 HWUSI-EAS1632R_0006_FC61F2Y:3:109:7431:10335.
acagaa--------------cgcaaac---aggaccatag-------------
>_R_gnl|SRA|DRR000628.10319422.1 HWUSI-EAS1632R_0006_FC61F2Y:3:89:15633:13758.
acagaa--------------cgcaaac---aggaccatag-------------
>_R_gnl|SRA|DRR000628.7010997.2 HWUSI-EAS1632R_0006_FC61F2Y:2:106:12639:19161.
acagaa--------------cgcaaac---aggaccatag-------------
>_R_gnl|SRA|DRR000628.5047343.1 HWUSI-EAS1632R_0006_FC61F2Y:2:42:13280:19129.
acagaa--------------cgcaaac---aggaccatag-------------
>_R_gnl|SRA|DRR000628.1091940.1 HWUSI-EAS1632R_0006_FC61F2Y:1:34:14870:16606.
acagaa--------------cgcaaac---aggaccatag-------------
>_R_gnl|SRA|DRR000627.8062514.1 HWUSI-EAS1632R_0006_FC61F2Y:3:18:8206:2788.
acagaa--------------cgcaaac---aggaccatag-------------
>_R_gnl|SRA|DRR000627.951117.2 HWUSI-EAS1632R_0006_FC61F2Y:1:29:18373:19332.
acagaa--------------cgcaaac---aggaccatag-------------

>_R_gnl|SRA|DRR000627.5952484.2 HWUSI-EAS1632R_0006_FC61F2Y:2:74:2980:16054.
acagaa--------------cgcaaac---aggaccatag-------------
>_R_gnl|SRA|DRR000628.3083714.2 HWUSI-EAS1632R_0006_FC61F2Y:1:103:3686:20583.
acagaa--------------cgcaaac---aggaccatgg-------------
>_R_gnl|SRA|DRR000627.10126191.2 HWUSI-EAS1632R_0006_FC61F2Y:3:85:12453:6797.
acagaa--------------cgcaaacagtaggacc-----------------
>_R_gnl|SRA|DRR000628.6976719.2 HWUSI-EAS1632R_0006_FC61F2Y:2:105:12105:12216.
acagaa--------------cgcaaacagtaggac------------------
>_R_gnl|SRA|DRR000628.3895986.1 HWUSI-EAS1632R_0006_FC61F2Y:2:7:10957:5082.
acagaa--------------cgcaaacagtaggac------------------
>_R_gnl|SRA|DRR000629.13027728.1 HWUSI-EAS1632R_0006_FC61F2Y:3:28:2943:3938.
acagaa--------------cgcaaacaggaccat------------------
>_R_gnl|SRA|DRR000628.5550552.1 HWUSI-EAS1632R_0006_FC61F2Y:2:59:11582:8460.
acagaa--------------cgcaaacagtagga-------------------
>_R_gnl|SRA|DRR000628.10608209.2 HWUSI-EAS1632R_0006_FC61F2Y:3:98:12319:15095.
acagaa--------------cgcaaacaggacca-------------------
>_R_gnl|SRA|DRR000628.7941626.1 HWUSI-EAS1632R_0006_FC61F2Y:3:14:4602:20651.
acagaa--------------cgcaaacaggacca-------------------
>_R_gnl|SRA|DRR000628.5413655.1 HWUSI-EAS1632R_0006_FC61F2Y:2:55:1678:7184.
acagaa--------------cgcaaacaggacca-------------------
>_R_gnl|SRA|DRR000629.14212609.1 HWUSI-EAS1632R_0006_FC61F2Y:3:52:16347:14425.
acagaa--------------cgcaaacagtagg--------------------
>_R_gnl|SRA|DRR000628.11110855.1 HWUSI-EAS1632R_0006_FC61F2Y:3:113:14783:4693.
acagaa--------------cgcaaacagtagg--------------------
>_R_gnl|SRA|DRR000628.4754311.1 HWUSI-EAS1632R_0006_FC61F2Y:2:33:10746:1713.
acagaa--------------cgcaaacagtagg--------------------
>gnl|SRA|DRR000627.9641416.2 HWUSI-EAS1632R_0006_FC61F2Y:3:69:7124:12981.
acagaa--------------cgcaaacagtagg--------------------
>gnl|SRA|DRR000628.10326486.2 HWUSI-EAS1632R_0006_FC61F2Y:3:90:1375:4202.
acagaa--------------cgcaaacaggacc--------------------
>_R_gnl|SRA|DRR000628.10186070.1 HWUSI-EAS1632R_0006_FC61F2Y:3:85:12813:5004.
acagaa--------------cgcaaacaggacc--------------------
>_R_gnl|SRA|DRR000628.3699101.2 HWUSI-EAS1632R_0006_FC61F2Y:2:1:14372:14448.
acagaa--------------cgcaaacaggacc--------------------
>gnl|SRA|DRR000627.5135220.2 HWUSI-EAS1632R_0006_FC61F2Y:2:46:4559:3532.
acagaa--------------cgcaaacaggacc--------------------
>gnl|SRA|DRR000628.10072734.1 HWUSI-EAS1632R_0006_FC61F2Y:3:82:3021:18746.
acagaa--------------cgcaaacagtag---------------------
>_R_gnl|SRA|DRR000628.8599481.2 HWUSI-EAS1632R_0006_FC61F2Y:3:34:1887:10600.
acagaa--------------cgcaaacagtag---------------------
>_R_gnl|SRA|DRR000628.6588242.2 HWUSI-EAS1632R_0006_FC61F2Y:2:93:13445:18383.
acagaa--------------cgcaaacagtag---------------------
>gnl|SRA|DRR000627.5694582.2 HWUSI-EAS1632R_0006_FC61F2Y:2:65:8549:4170.
acagaa--------------cgcaaacagtag---------------------
>gnl|SRA|DRR000628.10146306.1 HWUSI-EAS1632R_0006_FC61F2Y:3:84:8468:14904.
acagaa--------------cgcaaacaggac---------------------
>_R_gnl|SRA|DRR000628.8513201.2 HWUSI-EAS1632R_0006_FC61F2Y:3:31:8976:6188.
acagaa--------------cgcaaacaggac---------------------
>_R_gnl|SRA|DRR000628.5523204.2 HWUSI-EAS1632R_0006_FC61F2Y:2:58:13070:16905.
acagaa--------------cgcaaacaggac---------------------
>_R_gnl|SRA|DRR000628.4969391.2 HWUSI-EAS1632R_0006_FC61F2Y:2:40:4841:13445.

acagaa--------------cgcaaacaggac---------------------
>gnl|SRA|DRR000628.4254299.1 HWUSI-EAS1632R_0006_FC61F2Y:2:18:6396:7767.
acagaa--------------cgcaaacaggac---------------------
>_R_gnl|SRA|DRR000628.1438015.2 HWUSI-EAS1632R_0006_FC61F2Y:1:47:9514:17769.
acagaa--------------cgcaaacaggac---------------------
>_R_gnl|SRA|DRR000627.4719662.2 HWUSI-EAS1632R_0006_FC61F2Y:2:32:14107:3132.
acagaa--------------cgcaaacaggac---------------------
>gnl|SRA|DRR000628.9942557.1 HWUSI-EAS1632R_0006_FC61F2Y:3:78:1375:14016.
acagaa--------------cgcaaacagta----------------------
>gnl|SRA|DRR000627.5716946.1 HWUSI-EAS1632R_0006_FC61F2Y:2:66:4308:13850.
acagaa--------------cgcaaacagta----------------------
>_R_gnl|SRA|DRR000628.4056811.2 HWUSI-EAS1632R_0006_FC61F2Y:2:12:7637:6678.
acagaa--------------cgcaaacagga----------------------
>gnl|SRA|DRR000627.5628536.1 HWUSI-EAS1632R_0006_FC61F2Y:2:63:3635:18407.
acagaa--------------cgcaaacagga----------------------
>gnl|SRA|DRR000627.2458539.2 HWUSI-EAS1632R_0006_FC61F2Y:1:84:2441:13259.
acagaa--------------cgcaaacagga----------------------
>gnl|SRA|DRR000627.8962315.2 HWUSI-EAS1632R_0006_FC61F2Y:3:46:8786:7395.
acagaa--------------cgcaaacagt-----------------------
>gnl|SRA|DRR000627.3172104.1 HWUSI-EAS1632R_0006_FC61F2Y:1:106:13038:5561.
acagaa--------------cgcaaacagt-----------------------
>_R_gnl|SRA|DRR000628.11002236.2 HWUSI-EAS1632R_0006_FC61F2Y:3:110:10422:14200.
acagaa--------------cgcaaacagg-----------------------
>_R_gnl|SRA|DRR000628.9935173.1 HWUSI-EAS1632R_0006_FC61F2Y:3:77:15513:4441.
acagaa--------------cgcaaacagg-----------------------
>gnl|SRA|DRR000628.5208811.1 HWUSI-EAS1632R_0006_FC61F2Y:2:48:8558:10345.
acagaa--------------cgcaaacagg-----------------------
>_R_gnl|SRA|DRR000628.3303327.2 HWUSI-EAS1632R_0006_FC61F2Y:1:109:18045:13695.
acagaa--------------cgcaaacagg-----------------------
>_R_gnl|SRA|DRR000628.5468.2 HWUSI-EAS1632R_0006_FC61F2Y:1:1:4380:11002.
acagaa--------------cgcaaacagg-----------------------
>_R_gnl|SRA|DRR000627.1931011.1 HWUSI-EAS1632R_0006_FC61F2Y:1:64:14053:14079.
acagaa--------------cgcaaacagg-----------------------
>gnl|SRA|DRR000628.10171271.2 HWUSI-EAS1632R_0006_FC61F2Y:3:85:4501:7743.
acagaa--------------cgcaaacaga-----------------------
>_R_gnl|SRA|DRR000628.10871681.2 HWUSI-EAS1632R_0006_FC61F2Y:3:106:12181:20110.
acagaa--------------cgcaaacagg-----------------------
>_R_gnl|SRA|DRR000628.10171271.1 HWUSI-EAS1632R_0006_FC61F2Y:3:85:4501:7743.
acagaa--------------cgcaaacag------------------------
>gnl|SRA|DRR000628.3923114.2 HWUSI-EAS1632R_0006_FC61F2Y:2:8:7604:2282.
acagaa--------------cgcaaacag------------------------
>_R_gnl|SRA|DRR000628.3912465.1 HWUSI-EAS1632R_0006_FC61F2Y:2:8:1652:12225.
acagaa--------------cgcaaaca-------------------------
>_R_gnl|SRA|DRR000628.1106343.2 HWUSI-EAS1632R_0006_FC61F2Y:1:35:5086:1707.
acagaa--------------cgcaaaca-------------------------
>gnl|SRA|DRR000627.2637451.2 HWUSI-EAS1632R_0006_FC61F2Y:1:89:17058:10903.
acagaa--------------cgcaaaca-------------------------
>_R_gnl|SRA|DRR000629.11761820.1 HWUSI-EAS1632R_0006_FC61F2Y:3:4:13630:10399.
acagaa--------------cgcaaac--------------------------
>_R_gnl|SRA|DRR000629.2890540.1 HWUSI-EAS1632R_0006_FC61F2Y:1:62:9804:4316.
acagaa--------------cgcaaac--------------------------

>_R_gnl|SRA|DRR000628.11207825.1 HWUSI-EAS1632R_0006_FC61F2Y:3:116:12049:20590.
acagaa--------------cgcaaac--------------------------
>_R_gnl|SRA|DRR000628.10934734.1 HWUSI-EAS1632R_0006_FC61F2Y:3:108:10223:8574.
acagaa--------------cgcaaac--------------------------
>_R_gnl|SRA|DRR000628.9652138.1 HWUSI-EAS1632R_0006_FC61F2Y:3:68:3055:7275.
acagaa--------------cgcaaac--------------------------
>_R_gnl|SRA|DRR000628.7872303.1 HWUSI-EAS1632R_0006_FC61F2Y:3:12:3269:4048.
acagaa--------------cgcaaac--------------------------
>gnl|SRA|DRR000628.7316637.2 HWUSI-EAS1632R_0006_FC61F2Y:2:115:15581:17633.
acagaa--------------cgcaaac--------------------------
>gnl|SRA|DRR000628.4126693.2 HWUSI-EAS1632R_0006_FC61F2Y:2:14:9514:12147.
acagaa--------------cgcaaac--------------------------
>_R_gnl|SRA|DRR000628.3416412.1 HWUSI-EAS1632R_0006_FC61F2Y:1:113:8069:18583.
acagaa--------------cgcaaac--------------------------
>_R_gnl|SRA|DRR000628.1965313.1 HWUSI-EAS1632R_0006_FC61F2Y:1:65:11734:13993.
acagaa--------------cgcaaac--------------------------
>_R_gnl|SRA|DRR000628.1671126.1 HWUSI-EAS1632R_0006_FC61F2Y:1:55:7692:7480.
acagaa--------------cgcaaac--------------------------
>_R_gnl|SRA|DRR000628.1602444.2 HWUSI-EAS1632R_0006_FC61F2Y:1:52:19602:15230.
acagaa--------------cgcaaac--------------------------
>_R_gnl|SRA|DRR000627.10930491.1 HWUSI-EAS1632R_0006_FC61F2Y:3:110:4868:14564.
acagaa--------------cgcaaac--------------------------
>_R_gnl|SRA|DRR000627.10407966.1 HWUSI-EAS1632R_0006_FC61F2Y:3:94:8146:20648.
acagaa--------------cgcaaac--------------------------
>_R_gnl|SRA|DRR000627.9516089.2 HWUSI-EAS1632R_0006_FC61F2Y:3:65:3032:2121.
acagaa--------------cgcaaac--------------------------
>gnl|SRA|DRR000627.4276203.2 HWUSI-EAS1632R_0006_FC61F2Y:2:19:4342:17357.
acagaa--------------cgcaaac--------------------------
>_R_gnl|SRA|DRR000628.8371646.2 HWUSI-EAS1632R_0006_FC61F2Y:3:27:3468:1906.
acagaa--------------cgcaaac--------------------------
>gnl|SRA|DRR000628.4603480.2 HWUSI-EAS1632R_0006_FC61F2Y:2:28:17756:1106.
acagaa--------------cgcaaac--------------------------
>_R_gnl|SRA|DRR000628.4205926.1 HWUSI-EAS1632R_0006_FC61F2Y:2:16:16489:7737.
acagaa--------------cgcaaac--------------------------
>_R_gnl|SRA|DRR000627.1846787.1 HWUSI-EAS1632R_0006_FC61F2Y:1:61:14165:9479.
acagaa--------------cgcaaac--------------------------
>_R_gnl|SRA|DRR000628.1495597.1 HWUSI-EAS1632R_0006_FC61F2Y:1:49:8397:12136.
acagaa--------------cgcaaac--------------------------
>_R_gnl|SRA|DRR000627.10873044.1 HWUSI-EAS1632R_0006_FC61F2Y:3:108:10326:18536.
acagaa--------------cgcaaac--------------------------
>_R_gnl|SRA|DRR000628.4358853.2 HWUSI-EAS1632R_0006_FC61F2Y:2:21:9261:17574.
acagaa--------------cgcaaac--------------------------
>_R_gnl|SRA|DRR000628.9461084.1 HWUSI-EAS1632R_0006_FC61F2Y:3:61:16998:2501.
acagaa--------------cgcaaac--------------------------
>_R_gnl|SRA|DRR000628.11154774.2 HWUSI-EAS1632R_0006_FC61F2Y:3:115:1954:6012.
acagaa--------------cgcaaa---------------------------
>_R_gnl|SRA|DRR000628.9662477.1 HWUSI-EAS1632R_0006_FC61F2Y:3:68:9061:5041.
acagaa--------------cgcaaa---------------------------
>_R_gnl|SRA|DRR000628.9150741.2 HWUSI-EAS1632R_0006_FC61F2Y:3:51:16308:4180.
acagaa--------------cgcaaa---------------------------
>_R_gnl|SRA|DRR000628.7955862.2 HWUSI-EAS1632R_0006_FC61F2Y:3:14:12361:13637.

acagaa--------------cgcaaa---------------------------
>_R_gnl|SRA|DRR000628.7659926.1 HWUSI-EAS1632R_0006_FC61F2Y:3:5:16259:10625.
acagaa--------------cgcaaa---------------------------
>_R_gnl|SRA|DRR000628.6789803.1 HWUSI-EAS1632R_0006_FC61F2Y:2:99:17604:14231.
acagaa--------------cgcaaa---------------------------
>_R_gnl|SRA|DRR000628.6663738.1 HWUSI-EAS1632R_0006_FC61F2Y:2:96:1279:17799.
acagaa--------------cgcaaa---------------------------
>_R_gnl|SRA|DRR000628.3568756.2 HWUSI-EAS1632R_0006_FC61F2Y:1:117:18627:10980.
acagaa--------------cgcaaa---------------------------
>_R_gnl|SRA|DRR000628.3551703.1 HWUSI-EAS1632R_0006_FC61F2Y:1:117:9256:9587.
acagaa--------------cgcaaa---------------------------
>_R_gnl|SRA|DRR000628.2469275.1 HWUSI-EAS1632R_0006_FC61F2Y:1:83:14350:18081.
acagaa--------------cgcaaa---------------------------
>_R_gnl|SRA|DRR000627.9354446.2 HWUSI-EAS1632R_0006_FC61F2Y:3:59:12041:16722.
acagaa--------------cgcaaa---------------------------
>_R_gnl|SRA|DRR000627.1357471.2 HWUSI-EAS1632R_0006_FC61F2Y:1:44:11744:3469.
acagaa--------------cgcaaa---------------------------
>_R_gnl|SRA|DRR000628.2473993.2 HWUSI-EAS1632R_0006_FC61F2Y:1:83:17804:14496.
acagaa--------------cgcaaa---------------------------
>gnl|SRA|DRR000627.8123219.2 HWUSI-EAS1632R_0006_FC61F2Y:3:20:4816:5578.
acagaa--------------cgcaaa---------------------------
>_R_gnl|SRA|DRR000627.663659.2 HWUSI-EAS1632R_0006_FC61F2Y:1:20:16659:18162.
acagaa--------------cgcaaacagt-----------------------
>_R_gnl|SRA|DRR000628.7775009.2 HWUSI-EAS1632R_0006_FC61F2Y:3:9:5486:2383.
acagaa--------------cgcaaacagg-----------------------
>gnl|SRA|DRR000628.1648044.2 HWUSI-EAS1632R_0006_FC61F2Y:1:54:11467:10541.
acagaa--------------cgcaaacagg-----------------------
>_R_gnl|SRA|DRR000628.5770846.2 HWUSI-EAS1632R_0006_FC61F2Y:2:67:1425:10689.
acagaa--------------cgcaaacagtagga-------------------
>_R_gnl|SRA|DRR000628.4574149.2 HWUSI-EAS1632R_0006_FC61F2Y:2:27:19772:20597.
acagaa--------------cgcaaacagtagg--------------------
>_R_gnl|SRA|DRR000628.683830.2 HWUSI-EAS1632R_0006_FC61F2Y:1:21:16235:19734.
acagaa--------------cgcaaacagtagg--------------------
>_R_gnl|SRA|DRR000628.8584325.1 HWUSI-EAS1632R_0006_FC61F2Y:3:33:11821:18339.
acagaa--------------cgcaaacagtag---------------------
>_R_gnl|SRA|DRR000628.4638226.1 HWUSI-EAS1632R_0006_FC61F2Y:2:29:18957:3821.
acagaa--------------cgcaaacagtag---------------------
>gnl|SRA|DRR000629.14941990.2 HWUSI-EAS1632R_0006_FC61F2Y:3:68:16773:14053.
acagaa--------------cgcaaacaggac---------------------
>gnl|SRA|DRR000627.3960612.2 HWUSI-EAS1632R_0006_FC61F2Y:2:9:15872:3034.
acagaa--------------cgcaaacaggac---------------------
>_R_gnl|SRA|DRR000628.1561847.2 HWUSI-EAS1632R_0006_FC61F2Y:1:51:12793:13754.
gcagaa--------------cgcaaacaggac---------------------
>_R_gnl|SRA|DRR000628.6716055.2 HWUSI-EAS1632R_0006_FC61F2Y:2:97:12698:14105.
acagaa--------------cgcaaacaggac---------------------
>_R_gnl|SRA|DRR000628.824925.1 HWUSI-EAS1632R_0006_FC61F2Y:1:26:5036:15312.
acagaa--------------cgcaaacagtaggac------------------
>gnl|SRA|DRR000627.8879796.1 HWUSI-EAS1632R_0006_FC61F2Y:3:43:10215:3998.
acagaa--------------cgcaaacaggac---------------------
>gnl|SRA|DRR000627.5814621.2 HWUSI-EAS1632R_0006_FC61F2Y:2:69:9904:2308.
acagaa--------------cgcaaacagcaggaccatagtgggg--------

>gnl|SRA|DRR000627.3184748.2 HWUSI-EAS1632R_0006_FC61F2Y:1:107:1805:14510.
acagaa--------------cgcaaacagtaggaccatagtg-----------
>gnl|SRA|DRR000627.3512843.2 HWUSI-EAS1632R_0006_FC61F2Y:1:116:17611:17990.
acagaa--------------cgcaaacagtaggaccatagtg-----------
>gnl|SRA|DRR000627.4573577.2 HWUSI-EAS1632R_0006_FC61F2Y:2:28:5060:15698.
acagaa--------------cgcaaacag---gaccatagtgggggagtctct
>gnl|SRA|DRR000628.10814246.2 HWUSI-EAS1632R_0006_FC61F2Y:3:104:17113:7260.
acagaa--------------cgcaaacag---gaccatagtgggggattct--
>gnl|SRA|DRR000628.9157017.2 HWUSI-EAS1632R_0006_FC61F2Y:3:52:1242:11024.
acagaa--------------cgcaaacag---gaccatagtgggggattctat
>gnl|SRA|DRR000628.3019049.1 HWUSI-EAS1632R_0006_FC61F2Y:1:101:3496:9853.
acagaa--------------cgcaaacag---gaccatagtgggggattctat
>_R_gnl|SRA|DRR000628.1244703.1 HWUSI-EAS1632R_0006_FC61F2Y:1:40:9623:9917.
acagaa--------------cgcaaacagtagtaccatagtggggg-------
>gnl|SRA|DRR000627.10991537.2 HWUSI-EAS1632R_0006_FC61F2Y:3:112:1419:19592.
acagaa--------------cgcaaacag---gaccatagt------------
>_R_gnl|SRA|DRR000628.9868750.2 HWUSI-EAS1632R_0006_FC61F2Y:3:75:6529:5980.
acagaa--------------cgcaaacag---gaccatagtgggggat-----
>_R_gnl|SRA|DRR000627.5785737.2 HWUSI-EAS1632R_0006_FC61F2Y:2:68:10463:6306.
acagaa--------------cgcaaacag---gaccatagtgggggattctat
>gnl|SRA|DRR000629.12021183.1 HWUSI-EAS1632R_0006_FC61F2Y:3:9:5029:18970.
acagaa--------------cgcaaacagtaggaccatagtgggggattctat
>_R_gnl|SRA|DRR000627.5216174.1 HWUSI-EAS1632R_0006_FC61F2Y:2:49:3768:3362.
acagaa--------------cgcaaacagtaggaccatagtgggggattctat
>gnl|SRA|DRR000628.4960146.2 HWUSI-EAS1632R_0006_FC61F2Y:2:39:17845:6548.
acagaa--------------cgcaaacagtaggaccatagtgggggattctat
>_R_gnl|SRA|DRR000628.11156562.1 HWUSI-EAS1632R_0006_FC61F2Y:3:115:2951:10312.
acagaa--------------cgcaaacagtaggaccatagtgggggattctat
>_R_gnl|SRA|DRR000627.11093763.2 HWUSI-EAS1632R_0006_FC61F2Y:3:115:1344:11235.
acagaa--------------cgcaaacagtaggaccatagtgggggattctat
>_R_gnl|SRA|DRR000627.10558276.2 HWUSI-EAS1632R_0006_FC61F2Y:3:99:1200:5719.
acagaa--------------cgcaaacagtaggaccatagtgggggattctat
>_R_gnl|SRA|DRR000627.8553088.2 HWUSI-EAS1632R_0006_FC61F2Y:3:33:5546:6567.
acagaa--------------cgcaaacagtaggaccatagtgggggattctat
>_R_gnl|SRA|DRR000628.5114524.2 HWUSI-EAS1632R_0006_FC61F2Y:2:44:16965:3603.
acagaa--------------ctcaaacagtaggaccatagtgggggattctat
>_R_gnl|SRA|DRR000627.10516793.1 HWUSI-EAS1632R_0006_FC61F2Y:3:97:14511:4037.
acagaa--------------cgcaaacagtaggaccatagtgggggattctat
>_R_gnl|SRA|DRR000628.7041745.2 HWUSI-EAS1632R_0006_FC61F2Y:2:107:11454:5916.
acacac--------------ttcaaacagtaggaccatagtgggggattctat
>_R_gnl|SRA|DRR000628.6263274.1 HWUSI-EAS1632R_0006_FC61F2Y:2:83:9926:4993.
acagaa--------------cgcaaacagtaggaccatagtgggggattctat
>_R_gnl|SRA|DRR000628.9677193.2 HWUSI-EAS1632R_0006_FC61F2Y:3:68:17925:10756.
acagaa--------------cgcaaacagtaggaccatagtgggggattctat
>_R_gnl|SRA|DRR000628.6906411.2 HWUSI-EAS1632R_0006_FC61F2Y:2:103:9690:18666.
acagaa--------------cgcaaacagtaggaccatagtgggggattctat
>_R_gnl|SRA|DRR000628.3342376.2 HWUSI-EAS1632R_0006_FC61F2Y:1:111:3208:19790.
acagaa--------------cgcaaacagtaggaccatagtgggggattctat
>_R_gnl|SRA|DRR000627.6594073.2 HWUSI-EAS1632R_0006_FC61F2Y:2:95:3335:13694.
acagaa--------------cgcaaaaagtaggaccatagtgggggattctat
>_R_gnl|SRA|DRR000627.6593944.2 HWUSI-EAS1632R_0006_FC61F2Y:2:95:3258:12823.

acagaa--------------cgcaaaaagtaggaccatagtgggggattctat
>_R_gnl|SRA|DRR000627.10468609.2 HWUSI-EAS1632R_0006_FC61F2Y:3:96:5697:13199.
acagaa--------------cgcaaacagtaggaccatagtgggggattctat
>_R_gnl|SRA|DRR000627.28797.2 HWUSI-EAS1632R_0006_FC61F2Y:1:1:16443:20335.
acagaa--------------cgcaaacagtaggaccatagtgggggattctat
>gnl|SRA|DRR000627.875871.1 HWUSI-EAS1632R_0006_FC61F2Y:1:27:9341:18323.
acagaa--------------cgcaaacagtaagaccatagtgggggattctat
>gnl|SRA|DRR000628.9539261.2 HWUSI-EAS1632R_0006_FC61F2Y:3:64:8977:15904.
acagaa--------------cgcaaacagtaggaccatagtgggggattctat
>gnl|SRA|DRR000627.5360598.1 HWUSI-EAS1632R_0006_FC61F2Y:2:53:17909:7850.
acagaa--------------cgcaaacagtaggaccatagtgggggattctat
>gnl|SRA|DRR000627.3436446.2 HWUSI-EAS1632R_0006_FC61F2Y:1:114:12838:13380.
acagaa--------------cgcaaacagtaggaccatagtgggggattctat
>_R_gnl|SRA|DRR000628.4849271.2 HWUSI-EAS1632R_0006_FC61F2Y:2:36:9499:13926.
acagaa--------------cgcaaacagtaggaccatagtgggggattctat
>gnl|SRA|DRR000627.6826272.1 HWUSI-EAS1632R_0006_FC61F2Y:2:102:4908:14403.
acagaa--------------cgcaaacagtaggaccatagtgggggattctat
>_R_gnl|SRA|DRR000628.7435307.2 HWUSI-EAS1632R_0006_FC61F2Y:2:119:6473:10827.
acagaa--------------cgcaaacagtaggaccatagtgggggattctat
>_R_gnl|SRA|DRR000627.1635414.1 HWUSI-EAS1632R_0006_FC61F2Y:1:54:3332:4587.
acagaa--------------cgcaaacagtaggaccatagtgggggattctat
>_R_gnl|SRA|DRR000628.6451234.2 HWUSI-EAS1632R_0006_FC61F2Y:2:89:8236:5185.
acagaa--------------cgcaaacagtaggaccatagtgggggattctat
>_R_gnl|SRA|DRR000628.6193722.2 HWUSI-EAS1632R_0006_FC61F2Y:2:81:6464:15175.
acagaa--------------cgcaaacagtaggaccatagtgggggattctat
>_R_gnl|SRA|DRR000628.3315841.1 HWUSI-EAS1632R_0006_FC61F2Y:1:110:6671:2044.
acagaa--------------cgcaaacagtaggaccatagtgggggattctat
>gnl|SRA|DRR000628.1029467.1 HWUSI-EAS1632R_0006_FC61F2Y:1:32:15233:13413.
acagaa--------------cgcaaacagtaggaccatagtgggggattctat
>gnl|SRA|DRR000628.5578816.1 HWUSI-EAS1632R_0006_FC61F2Y:2:60:11436:16547.
acagaa--------------cgcaaacagtaggaccatagtgggggattctat
>gnl|SRA|DRR000628.5497256.2 HWUSI-EAS1632R_0006_FC61F2Y:2:57:15709:20611.
acagaa--------------cgcaaacagtaggaccatagtgggggattctat
>gnl|SRA|DRR000628.4698958.2 HWUSI-EAS1632R_0006_FC61F2Y:2:31:16451:3492.
acagaa--------------cgcaaacagtaggaccatagtgggggattctat
>_R_gnl|SRA|DRR000628.2637919.1 HWUSI-EAS1632R_0006_FC61F2Y:1:89:3884:14296.
acagaa--------------cgcaaacagtaggaccatagtgggggattctat
>gnl|SRA|DRR000627.9244615.1 HWUSI-EAS1632R_0006_FC61F2Y:3:55:18486:16322.
acagaa--------------cgcaaacagtaggaccatagtgggggattctat
>gnl|SRA|DRR000628.1479595.2 HWUSI-EAS1632R_0006_FC61F2Y:1:48:16823:14974.
acagaa--------------cgcaaacagtaggaccatagtgggggattctat
>_R_gnl|SRA|DRR000629.10089389.1 HWUSI-EAS1632R_0006_FC61F2Y:2:93:2056:19842.
acagaa--------------cgcaaacag---gaccatagtgggggattctat
>_R_gnl|SRA|DRR000629.9791967.1 HWUSI-EAS1632R_0006_FC61F2Y:2:86:17564:11621.
acagaa--------------cgcaaacag---gaccatagtgggggattctat
>_R_gnl|SRA|DRR000627.6824552.2 HWUSI-EAS1632R_0006_FC61F2Y:2:102:3962:3226.
acagaa--------------cgcaaacag---gaccatagtgggggattctat
>gnl|SRA|DRR000628.9884268.1 HWUSI-EAS1632R_0006_FC61F2Y:3:76:1088:16828.
acagaa--------------cgcaaacag---gaccatagtgggggattctat
>gnl|SRA|DRR000628.4526859.1 HWUSI-EAS1632R_0006_FC61F2Y:2:26:11233:9314.
acagaa--------------cgcaaacag---gaccatagtgggggattctat

>gnl|SRA|DRR000627.1036755.1 HWUSI-EAS1632R_0006_FC61F2Y:1:32:13230:14721.
acagaa--------------cgcaaacag---gaccatagtgggggattctat
>gnl|SRA|DRR000627.8063793.1 HWUSI-EAS1632R_0006_FC61F2Y:3:18:8900:17623.
acagaa--------------cgcaaacag---gaccatagtgggggattctat
>_R_gnl|SRA|DRR000628.7600311.1 HWUSI-EAS1632R_0006_FC61F2Y:3:4:2454:12522.
acagaa--------------cgcaaacag---gaccatagtgggggattctat
>_R_gnl|SRA|DRR000628.4603480.1 HWUSI-EAS1632R_0006_FC61F2Y:2:28:17756:1106.
acagaa--------------cgcaaacag---gaccatagtgggggattctat
>_R_gnl|SRA|DRR000628.5640463.2 HWUSI-EAS1632R_0006_FC61F2Y:2:62:13189:4038.
acagaa--------------cgcaaacag---gaccatagtgggggattctat
>_R_gnl|SRA|DRR000628.2951107.1 HWUSI-EAS1632R_0006_FC61F2Y:1:99:1389:8198.
acagaa--------------cgcaaacag---gaccatagtgggggattctat
>gnl|SRA|DRR000627.7804284.2 HWUSI-EAS1632R_0006_FC61F2Y:3:10:14824:5205.
acagaa--------------cgcaaacag---gaccatagtgggggattctat
>gnl|SRA|DRR000627.1646364.1 HWUSI-EAS1632R_0006_FC61F2Y:1:54:10478:5647.
acagaa--------------cgcaaacag---gaccatagtgggggattctat
>_R_gnl|SRA|DRR000628.7839024.2 HWUSI-EAS1632R_0006_FC61F2Y:3:11:3572:5823.
acagaa--------------cgcaaacag---gaccatagtgggggattctat
>_R_gnl|SRA|DRR000628.7208000.2 HWUSI-EAS1632R_0006_FC61F2Y:2:112:11620:5956.
acagaa--------------cgcaaacag---gaccatagtgggggattctat
>_R_gnl|SRA|DRR000628.2757285.1 HWUSI-EAS1632R_0006_FC61F2Y:1:92:18319:12058.
acagaa--------------cgcaaacag---gacgatagtgggggattctat
>_R_gnl|SRA|DRR000628.795308.2 HWUSI-EAS1632R_0006_FC61F2Y:1:25:6269:5185.
acagaa--------------cgcaaactg---gaccatagtgggggattctat
>_R_gnl|SRA|DRR000628.8252256.1 HWUSI-EAS1632R_0006_FC61F2Y:3:23:10723:10077.
acagaa--------------cgcaaacag---gaccatagtgggggattctat
>_R_gnl|SRA|DRR000628.166147.2 HWUSI-EAS1632R_0006_FC61F2Y:1:5:18739:10258.
acagaa--------------cgcaaacag---gaccatagtgggggattctat
>_R_gnl|SRA|DRR000628.10924888.1 HWUSI-EAS1632R_0006_FC61F2Y:3:108:4904:3981.
acagaa--------------cgcaaacag---gaccatagtgggggattctat
>_R_gnl|SRA|DRR000628.8645807.1 HWUSI-EAS1632R_0006_FC61F2Y:3:35:9830:7813.
acagaa--------------cgcaaacag---gaccatagtgggggattctat
>_R_gnl|SRA|DRR000628.7530576.2 HWUSI-EAS1632R_0006_FC61F2Y:3:2:1916:12872.
acagaa--------------cgcaaacag---gaccatagtgggggattctat
>_R_gnl|SRA|DRR000628.1846775.1 HWUSI-EAS1632R_0006_FC61F2Y:1:61:10117:18813.
acagaa--------------cgcaaacag---gaccatagtgggggattctat
>_R_gnl|SRA|DRR000627.2458539.1 HWUSI-EAS1632R_0006_FC61F2Y:1:84:2441:13259.
acagaa--------------cgcaaacag---gaccatagtgggggattctat
>_R_gnl|SRA|DRR000628.4079096.2 HWUSI-EAS1632R_0006_FC61F2Y:2:13:1570:16101.
acagag--------------cgcaaacag---gaccatagtgggggattctat
>_R_gnl|SRA|DRR000628.7630452.1 HWUSI-EAS1632R_0006_FC61F2Y:3:4:18700:3612.
acagaa--------------cgcaaacag---gaccatagtgggggattctat
>_R_gnl|SRA|DRR000627.5135220.1 HWUSI-EAS1632R_0006_FC61F2Y:2:46:4559:3532.
acagaa--------------cgcaaacag---gaccatagtgggggattctat
>_R_gnl|SRA|DRR000627.10576986.1 HWUSI-EAS1632R_0006_FC61F2Y:3:99:11655:20810.
acagaa--------------cgcaaacag---gaccatagtgggggattctat
>gnl|SRA|DRR000628.936520.2 HWUSI-EAS1632R_0006_FC61F2Y:1:29:16249:2955.
acagaa--------------cgcaaacag---gaccgtagtgggggattctat
>_R_gnl|SRA|DRR000628.8877610.1 HWUSI-EAS1632R_0006_FC61F2Y:3:42:12881:14841.
acagaa--------------cgcaaacag---gaccatagtgggggattctat
>_R_gnl|SRA|DRR000628.1678562.2 HWUSI-EAS1632R_0006_FC61F2Y:1:55:12234:8680.

acagaa--------------cgcaaacag---gaccatagtgggggattctat
>_R_gnl|SRA|DRR000627.10991537.1 HWUSI-EAS1632R_0006_FC61F2Y:3:112:1419:19592.
acagaa--------------cgcaaacag---gaccatagtgggggattctat
>_R_gnl|SRA|DRR000627.10917453.1 HWUSI-EAS1632R_0006_FC61F2Y:3:109:16279:7454.
acagaa--------------cgcaaacag---gaccatagtgggggattctat
>_R_gnl|SRA|DRR000628.286011.2 HWUSI-EAS1632R_0006_FC61F2Y:1:9:11201:18343.
acagaa--------------cgcaaactg---gaccatagtgggggattctat
>_R_gnl|SRA|DRR000628.9884268.2 HWUSI-EAS1632R_0006_FC61F2Y:3:76:1088:16828.
acagaa--------------cgcaaacag---gaccatagtgggggattctat
>_R_gnl|SRA|DRR000628.7756279.2 HWUSI-EAS1632R_0006_FC61F2Y:3:8:13579:15922.
acagaa--------------cgcaaacag---gaccatagtgggggattctat
>_R_gnl|SRA|DRR000628.7422112.1 HWUSI-EAS1632R_0006_FC61F2Y:2:118:17825:3871.
acagaa--------------cgcaaacag---gaccatagtgggggattctat
>_R_gnl|SRA|DRR000627.8115268.1 HWUSI-EAS1632R_0006_FC61F2Y:3:19:18848:17629.
acagaa--------------cgcaaacag---gaccatagtgggggattctat
>_R_gnl|SRA|DRR000627.3232384.2 HWUSI-EAS1632R_0006_FC61F2Y:1:108:10136:13988.
acagaa--------------cgcaaacag---gaccatagtgggggattctat
>_R_gnl|SRA|DRR000627.1710202.2 HWUSI-EAS1632R_0006_FC61F2Y:1:56:14848:2808.
acagaa--------------cgcaaacag---gaccatagtgggggattctat
>_R_gnl|SRA|DRR000628.3212838.1 HWUSI-EAS1632R_0006_FC61F2Y:1:107:3596:1438.
acagaa--------------cgcaaacag---gaccatagtgggggattctat
>_R_gnl|SRA|DRR000628.1765062.1 HWUSI-EAS1632R_0006_FC61F2Y:1:58:11907:10572.
acagaa--------------cgcaaacag---gaccatagtgggggattctat
>_R_gnl|SRA|DRR000628.7116128.2 HWUSI-EAS1632R_0006_FC61F2Y:2:109:15833:14047.
acagaa--------------cgcaaacag---gaccatagtgggggattctat
>gnl|SRA|DRR000627.2855102.2 HWUSI-EAS1632R_0006_FC61F2Y:1:96:15710:10036.
acagaa--------------cgcaaacag---gaccatagtgggggattctat
>_R_gnl|SRA|DRR000628.9679033.1 HWUSI-EAS1632R_0006_FC61F2Y:3:68:19221:8200.
acagaa--------------cgcaaacag---gaccatagtgggggattctat
>_R_gnl|SRA|DRR000628.7822695.1 HWUSI-EAS1632R_0006_FC61F2Y:3:10:13051:8399.
acagaa--------------cgcaaacag---gaccatagtgggggattctat
>_R_gnl|SRA|DRR000627.8934456.2 HWUSI-EAS1632R_0006_FC61F2Y:3:45:5895:19712.
acagaa--------------cgcaaacag---gaccatagtgggggattctat
>_R_gnl|SRA|DRR000628.8738150.2 HWUSI-EAS1632R_0006_FC61F2Y:3:38:6832:20396.
acagaa--------------cgcaaacag---gaccatagtgggggattctat
>_R_gnl|SRA|DRR000628.672327.2 HWUSI-EAS1632R_0006_FC61F2Y:1:21:9571:3891.
actgaa--------------cgcaaacag---gaccatagtgggggattctat
>_R_gnl|SRA|DRR000628.9058020.1 HWUSI-EAS1632R_0006_FC61F2Y:3:48:17465:12982.
acagaa--------------cgcaaacag---gaccatagtgggggattctat
>_R_gnl|SRA|DRR000628.151828.1 HWUSI-EAS1632R_0006_FC61F2Y:1:5:10758:13867.
acagaa--------------cgcaaacag---gaccatagtgggggattctat
>_R_gnl|SRA|DRR000627.4697629.1 HWUSI-EAS1632R_0006_FC61F2Y:2:32:1572:11101.
acagaa--------------cgcaaacag---gaccatagtgggggattctat
>gnl|SRA|DRR000627.5409398.2 HWUSI-EAS1632R_0006_FC61F2Y:2:55:11012:4208.
acagaa--------------cgcaaacag---gaccatagtgggggattctat
>gnl|SRA|DRR000627.10963987.2 HWUSI-EAS1632R_0006_FC61F2Y:3:111:4695:3344.
acagaa--------------cgcaaacag---gaccatagtgggggattctat
>_R_gnl|SRA|DRR000627.8879796.2 HWUSI-EAS1632R_0006_FC61F2Y:3:43:10215:3998.
acagaa--------------cgcaaacag---gaccatagtgggggattctat
>_R_gnl|SRA|DRR000627.5242225.1 HWUSI-EAS1632R_0006_FC61F2Y:2:49:19504:3714.
acagaa--------------cgcaaacag---gaccatagtgggggattctat

>gnl|SRA|DRR000627.8329858.2 HWUSI-EAS1632R_0006_FC61F2Y:3:26:9094:1785.
acagaa--------------cgcaaacag---gaccatagtgggggattctat
>_R_gnl|SRA|DRR000628.489375.1 HWUSI-EAS1632R_0006_FC61F2Y:1:15:15733:18441.
tcaaaa--------------cgcaaacaggagggccatagtgggggattctat
>gnl|SRA|DRR000627.3494030.2 HWUSI-EAS1632R_0006_FC61F2Y:1:116:7393:10804.
acagaa--------------cgcaaacag---gaccatagtgggggattctat
>gnl|SRA|DRR000627.1553792.2 HWUSI-EAS1632R_0006_FC61F2Y:1:51:6749:5097.
acagta--------------cgcaaacag---gaccatagtgggggattctat
>_R_gnl|SRA|DRR000627.10055067.2 HWUSI-EAS1632R_0006_FC61F2Y:3:83:7866:14248.
acagaa--------------cgcaaacagtaggaccatagtgggggattctat
>_R_gnl|SRA|DRR000628.11120162.2 HWUSI-EAS1632R_0006_FC61F2Y:3:114:1523:9622.
acagaa--------------cgcaaacagtaggaccatagtgggggattctat
>gnl|SRA|DRR000627.6292285.2 HWUSI-EAS1632R_0006_FC61F2Y:2:85:12985:13063.
acagaa--------------cgcaaacagtaggaccatagtgggggattctat
>_R_gnl|SRA|DRR000627.4276203.1 HWUSI-EAS1632R_0006_FC61F2Y:2:19:4342:17357.
acagaa--------------cgcaaacagtaggaccatagtgggggattctat
>_R_gnl|SRA|DRR000627.979163.2 HWUSI-EAS1632R_0006_FC61F2Y:1:30:16251:2745.
acagaa--------------cgcaaacagtaggaccatagtgggggattctat
>gnl|SRA|DRR000627.10055067.1 HWUSI-EAS1632R_0006_FC61F2Y:3:83:7866:14248.
acagaa--------------cgcaaacagtaggaccatagtgggggattctat
>_R_gnl|SRA|DRR000628.3577121.2 HWUSI-EAS1632R_0006_FC61F2Y:1:118:4828:19114.
acagaa--------------cgcaaacagtaggaccatagtgggggattctat
>_R_gnl|SRA|DRR000627.9641416.1 HWUSI-EAS1632R_0006_FC61F2Y:3:69:7124:12981.
acagaa--------------cgcaaacagtaggaccatagtgggggattctat
>_R_gnl|SRA|DRR000627.9481157.1 HWUSI-EAS1632R_0006_FC61F2Y:3:63:17953:7755.
acagaa--------------cgcaaacagtaggaccatagtgggggattctat
>_R_gnl|SRA|DRR000627.8767375.2 HWUSI-EAS1632R_0006_FC61F2Y:3:39:18337:18940.
acagaa--------------cgcaaacagtaggaccatagtgggggattctat
>_R_gnl|SRA|DRR000627.8301717.2 HWUSI-EAS1632R_0006_FC61F2Y:3:25:11839:6758.
acagaa--------------cgcaaacagtaggaccatagtgggggattctat
>_R_gnl|SRA|DRR000627.8123219.1 HWUSI-EAS1632R_0006_FC61F2Y:3:20:4816:5578.
acagaa--------------cgcaaacagtaggaccatagtgggggattctat
>_R_gnl|SRA|DRR000627.4700507.2 HWUSI-EAS1632R_0006_FC61F2Y:2:32:3324:6679.
acagaa--------------cgcaaacagtaggaccatagtgggggattctat
>_R_gnl|SRA|DRR000628.1152398.2 HWUSI-EAS1632R_0006_FC61F2Y:1:36:13687:16048.
acagaa--------------cgcaaacagtaggaccatagttggggattctat
>_R_gnl|SRA|DRR000627.8767369.2 HWUSI-EAS1632R_0006_FC61F2Y:3:39:18334:18961.
acagac--------------cgcaaccagtaggaccatagtgggggattctat
>_R_gnl|SRA|DRR000628.11057320.1 HWUSI-EAS1632R_0006_FC61F2Y:3:112:3962:6255.
acagaa--------------cgcaaacagtaggaccatagtgggggattctat
>_R_gnl|SRA|DRR000628.6503101.1 HWUSI-EAS1632R_0006_FC61F2Y:2:91:1504:2231.
acagaa--------------cgcaaacagtaggaccatagtgggggattctat
>_R_gnl|SRA|DRR000628.5920687.1 HWUSI-EAS1632R_0006_FC61F2Y:2:71:18770:18352.
acagaa--------------cgcaaacagtaggaccatagtgggggattctat
>_R_gnl|SRA|DRR000628.4202462.1 HWUSI-EAS1632R_0006_FC61F2Y:2:16:14539:5509.
acagaa--------------cgcaaacagtaggaccatagtgggggattctat
>_R_gnl|SRA|DRR000628.4091666.1 HWUSI-EAS1632R_0006_FC61F2Y:2:13:8619:6895.
acagaa--------------cgcaaacagtaggaccatagtgggggattctat
>_R_gnl|SRA|DRR000627.4233132.2 HWUSI-EAS1632R_0006_FC61F2Y:2:17:17446:5017.
acagaa--------------cgcaaacagtaggaccatagtgggggattctat
>_R_gnl|SRA|DRR000628.8169444.1 HWUSI-EAS1632R_0006_FC61F2Y:3:21:1642:1366.

acagaa--------------cgcaaacagtaggaccatagtgggggattctat
>gnl|SRA|DRR000627.4700507.1 HWUSI-EAS1632R_0006_FC61F2Y:2:32:3324:6679.
acagaa--------------cgcaaacagtaggaccatagtgggggattctat
>gnl|SRA|DRR000627.1341930.2 HWUSI-EAS1632R_0006_FC61F2Y:1:44:2115:10421.
acagaa--------------cgcaaacagtaggaccatagtgggggattctat
>_R_gnl|SRA|DRR000628.4299005.2 HWUSI-EAS1632R_0006_FC61F2Y:2:19:12451:1804.
acagaa--------------cgcaaacagtaggaccatagtgggggattctat
>gnl|SRA|DRR000628.4133926.1 HWUSI-EAS1632R_0006_FC61F2Y:2:14:13464:2467.
acagaa--------------cgcaaacagtaggaccatagtgggggattctat
>gnl|SRA|DRR000628.4802637.1 HWUSI-EAS1632R_0006_FC61F2Y:2:35:1456:1806.
acagaa--------------cgcaaacagtaggaccatagtgggggattctat
>_R_gnl|SRA|DRR000628.2293530.2 HWUSI-EAS1632R_0006_FC61F2Y:1:76:11929:9206.
acagaa--------------cgcaaacagtaggaccatagtgggggattctat
>_R_gnl|SRA|DRR000627.1489879.1 HWUSI-EAS1632R_0006_FC61F2Y:1:49:2700:4986.
acagaa--------------cgcaaacagtaggaccatagtgggggattccat
>_R_gnl|SRA|DRR000628.10124290.1 HWUSI-EAS1632R_0006_FC61F2Y:3:83:14075:1563.
acagaa--------------cgcaaacagtaggaccatagtgggggattctat
>_R_gnl|SRA|DRR000628.7851138.2 HWUSI-EAS1632R_0006_FC61F2Y:3:11:10188:18736.
acagaa--------------cgcaaacagtaggaccatagtgggggattctat
>_R_gnl|SRA|DRR000628.7522280.1 HWUSI-EAS1632R_0006_FC61F2Y:3:1:16037:8701.
acagaa--------------cgcaaacagtaggaccatagtgggggattctat
>_R_gnl|SRA|DRR000628.5785410.1 HWUSI-EAS1632R_0006_FC61F2Y:2:67:10295:18672.
acagaa--------------cgcaaacagtaggaccatagtgggggattctat
>_R_gnl|SRA|DRR000628.5583127.1 HWUSI-EAS1632R_0006_FC61F2Y:2:60:14085:12338.
acagaa--------------cgcaaacagtaggaccatagtgggggattctat
>_R_gnl|SRA|DRR000628.4960146.1 HWUSI-EAS1632R_0006_FC61F2Y:2:39:17845:6548.
acagaa--------------cgcaaacagtaggaccatagtgggggattctat
>_R_gnl|SRA|DRR000627.9734326.2 HWUSI-EAS1632R_0006_FC61F2Y:3:72:8504:18022.
acagaa--------------cgcaaacagtaggaccatagtgggggattctat
>_R_gnl|SRA|DRR000627.7101350.1 HWUSI-EAS1632R_0006_FC61F2Y:2:110:10326:1543.
acagaa--------------cgcaaacagtaggaccatagtgggggattctat
>_R_gnl|SRA|DRR000627.7033068.1 HWUSI-EAS1632R_0006_FC61F2Y:2:108:9566:5473.
acagaa--------------cgcaaacagtaggaccatagtgggggattctat
>_R_gnl|SRA|DRR000628.3763978.2 HWUSI-EAS1632R_0006_FC61F2Y:2:3:12361:6454.
acagaa--------------cgcaaacagtaggaccatagtgggggattctat
>_R_gnl|SRA|DRR000628.1302833.1 HWUSI-EAS1632R_0006_FC61F2Y:1:42:19389:14861.
acagaa--------------cgcaaacagtaggaccatagtgggggattctat
>_R_gnl|SRA|DRR000627.5694582.1 HWUSI-EAS1632R_0006_FC61F2Y:2:65:8549:4170.
acagaa--------------cgcaaacagtaggaccatagtgggggattctat
>_R_gnl|SRA|DRR000627.4951192.1 HWUSI-EAS1632R_0006_FC61F2Y:2:39:19209:7060.
acagaa--------------cgcaaacagtaggaccatagtgggggattctat
>gnl|SRA|DRR000628.5500928.1 HWUSI-EAS1632R_0006_FC61F2Y:2:57:18030:6021.
acagaa--------------cgcaaacagtaggaccatagtgggggattctat
>_R_gnl|SRA|DRR000627.6826272.2 HWUSI-EAS1632R_0006_FC61F2Y:2:102:4908:14403.
acagaa--------------cgcaaacagtaggaccatagtgggggattctat
>_R_gnl|SRA|DRR000628.7466569.1 HWUSI-EAS1632R_0006_FC61F2Y:2:120:4666:1472.
acagag--------------cgcaaacagtagggccatagtgggggattctat
>_R_gnl|SRA|DRR000629.17107303.1 HWUSI-EAS1632R_0006_FC61F2Y:3:112:8192:11704.
acagaa--------------cgcaaacagtaggaccatagtgggggattctat
>_R_gnl|SRA|DRR000629.12894225.1 HWUSI-EAS1632R_0006_FC61F2Y:3:25:10467:17383.
acagaa--------------cgcaaacagtaggaccatagtgggggattctat

>_R_gnl|SRA|DRR000629.7246692.1 HWUSI-EAS1632R_0006_FC61F2Y:2:30:15322:17459.
acagaa--------------cgcaaacagtcggaccatagtgggggattctat
>gnl|SRA|DRR000628.761043.1 HWUSI-EAS1632R_0006_FC61F2Y:1:24:5189:20069.
acagaa--------------cgcaaacagtaggaccatagtgggggattctat
>gnl|SRA|DRR000628.10468454.1 HWUSI-EAS1632R_0006_FC61F2Y:3:94:7634:17439.
acagaa--------------cgcaaacagtaggaccatagtgggggattctat
>gnl|SRA|DRR000627.5253225.1 HWUSI-EAS1632R_0006_FC61F2Y:2:50:7544:9913.
acagaa--------------cgcaaacagtaggaccatagtgggggattctat
>gnl|SRA|DRR000627.6433283.2 HWUSI-EAS1632R_0006_FC61F2Y:2:90:3002:1897.
acagaa--------------cgcaaacagtaggaccatagtgggggattctat
>_R_gnl|SRA|DRR000629.12021183.2 HWUSI-EAS1632R_0006_FC61F2Y:3:9:5029:18970.
-cagaa--------------cgcaaacagtaggaccatagtgggggattctat
>_R_gnl|SRA|DRR000629.6346495.1 HWUSI-EAS1632R_0006_FC61F2Y:2:13:1755:12016.
-cagaa--------------cgcaaacagtaggaccatagtgggggattctat
>_R_gnl|SRA|DRR000628.10407611.1 HWUSI-EAS1632R_0006_FC61F2Y:3:92:10307:10153.
---gaa--------------cgcaaacagtaggaccatagtgggggattctat
>_R_gnl|SRA|DRR000628.5188657.1 HWUSI-EAS1632R_0006_FC61F2Y:2:47:13811:11604.
---gaa--------------cgcaaacagtaggaccatagtgggggattctat
>_R_gnl|SRA|DRR000628.4698958.1 HWUSI-EAS1632R_0006_FC61F2Y:2:31:16451:3492.
---gaa--------------cgcaaacagtaggaccatagtgggggattctat
>_R_gnl|SRA|DRR000628.3276617.1 HWUSI-EAS1632R_0006_FC61F2Y:1:109:3041:5869.
---gaa--------------cgcaaacagtaggaccatagtgggggattctat
>_R_gnl|SRA|DRR000627.8962315.1 HWUSI-EAS1632R_0006_FC61F2Y:3:46:8786:7395.
---gaa--------------cgcaaacagtaggaccatagtgggggattctat
>_R_gnl|SRA|DRR000627.5716946.2 HWUSI-EAS1632R_0006_FC61F2Y:2:66:4308:13850.
---gaa--------------cgcaaacagtaggaccatagtgggggattctat
>_R_gnl|SRA|DRR000627.2040337.2 HWUSI-EAS1632R_0006_FC61F2Y:1:68:10853:7299.
---gaa--------------cgcaaacagtaggaccatagtgggggattctat
>_R_gnl|SRA|DRR000627.5312172.1 HWUSI-EAS1632R_0006_FC61F2Y:2:52:6529:7946.
---gaa--------------cgcagacagtaggaccatagtgggggattctat
>_R_gnl|SRA|DRR000628.11088596.2 HWUSI-EAS1632R_0006_FC61F2Y:3:113:2676:2296.
acagaa--------------cgcaaaca---ggaccatagtgggggattctat
>_R_gnl|SRA|DRR000628.9675533.2 HWUSI-EAS1632R_0006_FC61F2Y:3:68:16921:6937.
acagaa--------------cgcaaaca---ggaccatagtgggggattctat
>gnl|SRA|DRR000628.2283416.2 HWUSI-EAS1632R_0006_FC61F2Y:1:76:5855:5747.
acagaa--------------cgcaaaca---ggaccatagtgggggattctat
>gnl|SRA|DRR000627.8405851.1 HWUSI-EAS1632R_0006_FC61F2Y:3:28:14628:2286.
acagaa--------------cgcaaaca---ggaccatagtgggggattctat
>_R_gnl|SRA|DRR000628.9465137.2 HWUSI-EAS1632R_0006_FC61F2Y:3:62:1128:6076.
acagaa--------------cgcaaaca---ggaccatagtgggggattctat
>_R_gnl|SRA|DRR000627.10963987.1 HWUSI-EAS1632R_0006_FC61F2Y:3:111:4695:3344.
acagaa--------------cgcaaaca---ggaccatagtgggggattctat
>_R_gnl|SRA|DRR000627.5523880.2 HWUSI-EAS1632R_0006_FC61F2Y:2:59:9045:12306.
acagaa--------------cgcaaaca---ggaccatagtgggggattctat
>_R_gnl|SRA|DRR000627.3960612.1 HWUSI-EAS1632R_0006_FC61F2Y:2:9:15872:3034.
acagaa--------------cgcaaaca---ggaccatagtgggggattctat
>_R_gnl|SRA|DRR000628.6970828.2 HWUSI-EAS1632R_0006_FC61F2Y:2:105:8771:2994.
acagaa--------------cgcaaaca---ggaccatagtgggggattctat
>_R_gnl|SRA|DRR000627.9689373.1 HWUSI-EAS1632R_0006_FC61F2Y:3:70:18038:2736.
acagaa--------------cgcaaaca---ggaccatagtgggggattctat
>_R_gnl|SRA|DRR000628.2358727.2 HWUSI-EAS1632R_0006_FC61F2Y:1:78:14676:16491.

acagaa--------------cgcaaaca---ggaccatagtgggggattctat
>_R_gnl|SRA|DRR000627.9280460.1 HWUSI-EAS1632R_0006_FC61F2Y:3:57:3328:10225.
acagaa--------------cgcaaaca---ggaccatagtgggggattctat
>_R_gnl|SRA|DRR000627.8068521.1 HWUSI-EAS1632R_0006_FC61F2Y:3:18:11485:1457.
acagaa--------------cgcaaaca---ggaccatagtgggggattctat
>_R_gnl|SRA|DRR000627.7472887.1 HWUSI-EAS1632R_0006_FC61F2Y:3:1:5530:1056.
acagaa--------------cgcaaaca---ggaccatagtgggggattctat
>_R_gnl|SRA|DRR000627.2187078.2 HWUSI-EAS1632R_0006_FC61F2Y:1:73:12314:7320.
acagaa--------------cgcaaaca---ggaccatagtgggggattctat
>_R_gnl|SRA|DRR000628.10516785.2 HWUSI-EAS1632R_0006_FC61F2Y:3:95:16568:18038.
acagaa--------------cgcaaaca---ggaccatagtgggggattctat
>gnl|SRA|DRR000627.11195070.2 HWUSI-EAS1632R_0006_FC61F2Y:3:117:18277:8153.
acagaa--------------cgcaaaca---ggaccatagtgggggattctat
>_R_gnl|SRA|DRR000628.9157017.1 HWUSI-EAS1632R_0006_FC61F2Y:3:52:1242:11024.
acagaa--------------cgcaaaca---ggaccatagtgggggattctat
>_R_gnl|SRA|DRR000627.6696035.2 HWUSI-EAS1632R_0006_FC61F2Y:2:98:5877:19021.
acagaa--------------cgcaaaca---ggaccatagtgggggattctat
>_R_gnl|SRA|DRR000628.8356522.1 HWUSI-EAS1632R_0006_FC61F2Y:3:26:13369:14852.
acagaa--------------cgcaaaca---ggaccatagtgggggattctat
>gnl|SRA|DRR000627.4471102.1 HWUSI-EAS1632R_0006_FC61F2Y:2:25:2601:5277.
acagaa--------------cgcaaaca---ggaccatagtgggggattctat
>gnl|SRA|DRR000627.2405591.1 HWUSI-EAS1632R_0006_FC61F2Y:1:81:10493:2516.
acagaa--------------cgcaaaca---ggaccatagtgggggattctat
>gnl|SRA|DRR000627.5310877.2 HWUSI-EAS1632R_0006_FC61F2Y:2:52:5772:9935.
acagaa--------------cgcaaaca---ggaccatagtgggggattctat
>gnl|SRA|DRR000627.2143872.2 HWUSI-EAS1632R_0006_FC61F2Y:1:72:3429:3379.
acagaa--------------cgcaaaca---ggaccatagtgggggattctat
>_R_gnl|SRA|DRR000629.9662081.1 HWUSI-EAS1632R_0006_FC61F2Y:2:84:4008:20796.
acagaa--------------cgcaaaca---ggaccatagtgggggattctat
>_R_gnl|SRA|DRR000628.6233256.2 HWUSI-EAS1632R_0006_FC61F2Y:2:82:10951:10732.
acagaa--------------cgcaaaca---ggaccatagtgggggattctat
>_R_gnl|SRA|DRR000628.2340128.1 HWUSI-EAS1632R_0006_FC61F2Y:1:78:3520:19108.
acagaa--------------cgcaaaca---ggaccatagtgggggattctat
>_R_gnl|SRA|DRR000627.8405851.2 HWUSI-EAS1632R_0006_FC61F2Y:3:28:14628:2286.
acagaa--------------cgcaaaca---ggaccatagtgggggattctat
>_R_gnl|SRA|DRR000627.7804284.1 HWUSI-EAS1632R_0006_FC61F2Y:3:10:14824:5205.
acagaa--------------cgcaaaca---ggaccatagtgggggattctat
>_R_gnl|SRA|DRR000627.2405591.2 HWUSI-EAS1632R_0006_FC61F2Y:1:81:10493:2516.
acagaa--------------cgcaaaca---ggaccatagtgggggattctat
>gnl|SRA|DRR000628.10201876.1 HWUSI-EAS1632R_0006_FC61F2Y:3:86:3675:19617.
-cagaa--------------cgcaaaca---ggaccatagtgggggattctat
>_R_gnl|SRA|DRR000627.9700422.2 HWUSI-EAS1632R_0006_FC61F2Y:3:71:6314:4715.
-cagaa--------------cgcaaaca---ggaccatagtgggggattctat
>gnl|SRA|DRR000627.7246139.2 HWUSI-EAS1632R_0006_FC61F2Y:2:114:15064:11649.
-cagaa--------------cgcaaaca---ggaccatagtgggggattctat
>_R_gnl|SRA|DRR000628.1752524.1 HWUSI-EAS1632R_0006_FC61F2Y:1:58:3302:3947.
acagaa--------------cgcaaaca---ggaccatagtgggggattctat
>_R_gnl|SRA|DRR000629.5234208.1 HWUSI-EAS1632R_0006_FC61F2Y:1:112:13547:5396.
--------------------cgcaaacagtaggaccatagtgggggattctat
>_R_gnl|SRA|DRR000629.2779008.1 HWUSI-EAS1632R_0006_FC61F2Y:1:59:16463:8619.
--------------------cgcaaacagtaggaccatagtgggggattctat

>gnl|SRA|DRR000628.8571706.2 HWUSI-EAS1632R_0006_FC61F2Y:3:33:4818:4094.
--------------------cgcaaacagtaggaccatagtgggggattctat
>gnl|SRA|DRR000628.2138502.1 HWUSI-EAS1632R_0006_FC61F2Y:1:71:8907:6424.
--------------------cgcaaacagtaggaccatagtgggggattctat
>gnl|SRA|DRR000627.3340217.1 HWUSI-EAS1632R_0006_FC61F2Y:1:111:14960:19879.
--------------------cgcaaacagtaggaccatagtgggggattctat
>_R_gnl|SRA|DRR000627.6077609.1 HWUSI-EAS1632R_0006_FC61F2Y:2:78:14396:6875.
---------------------gcaaacagtaggaccatagtgggggattctat
>_R_gnl|SRA|DRR000627.3436446.1 HWUSI-EAS1632R_0006_FC61F2Y:1:114:12838:13380.
---------------------gcaaacagtaggaccatagtgggggattctat
>gnl|SRA|DRR000628.8208241.2 HWUSI-EAS1632R_0006_FC61F2Y:3:22:4818:8957.
----------------------caaacagtaggaccatagtgggggattctat
>gnl|SRA|DRR000628.6578417.1 HWUSI-EAS1632R_0006_FC61F2Y:2:93:7911:6701.
----------------------caaacagtaggaccatagtgggggattctat
>_R_gnl|SRA|DRR000628.5364064.1 HWUSI-EAS1632R_0006_FC61F2Y:2:53:8889:13980.
----------------------caaacagtaggaccatagtgggggattctat
>_R_gnl|SRA|DRR000628.1767014.1 HWUSI-EAS1632R_0006_FC61F2Y:1:58:13264:7424.
----------------------caaacagtaggaccatagtgggggattctat
>gnl|SRA|DRR000627.6109399.1 HWUSI-EAS1632R_0006_FC61F2Y:2:79:15003:17054.
----------------------caaacagtaggaccatagtgggggattctat
>_R_gnl|SRA|DRR000627.3184748.1 HWUSI-EAS1632R_0006_FC61F2Y:1:107:1805:14510.
----------------------caaacagtaggaccatagtgggggattctat
>_R_gnl|SRA|DRR000627.2482978.2 HWUSI-EAS1632R_0006_FC61F2Y:1:84:17082:3958.
----------------------caaacagtaggaccatagtgggggattctat
>gnl|SRA|DRR000627.1103832.1 HWUSI-EAS1632R_0006_FC61F2Y:1:34:15997:16919.
----------------------caaacagtaggaccatagtgggggattctat
>_R_gnl|SRA|DRR000627.111441.1 HWUSI-EAS1632R_0006_FC61F2Y:1:4:5171:19127.
----------------------caaacagtaggaccatagtgggggattctat
>_R_gnl|SRA|DRR000628.1205493.1 HWUSI-EAS1632R_0006_FC61F2Y:1:38:9839:20040.
----------------------caaacagtaggaccatagtgggggattctat
>gnl|SRA|DRR000627.879476.2 HWUSI-EAS1632R_0006_FC61F2Y:1:27:11392:8012.
----------------------caaacagtaggaccatagtgggggattctat
>_R_gnl|SRA|DRR000627.271360.1 HWUSI-EAS1632R_0006_FC61F2Y:1:8:18312:17807.
----------------------tctctagtaggaccatagtgggggattctat
>_R_gnl|SRA|DRR000628.8339759.1 HWUSI-EAS1632R_0006_FC61F2Y:3:26:4154:11133.
----------------------acgcaaacaggaccatagtgggggattctat
>_R_gnl|SRA|DRR000628.9611735.1 HWUSI-EAS1632R_0006_FC61F2Y:3:66:15677:13671.
-----------------------aaacagtaggaccatagtgggggattctat
>_R_gnl|SRA|DRR000628.2958052.1 HWUSI-EAS1632R_0006_FC61F2Y:1:99:5406:4617.
-----------------------aaacagtaggaccatagtgggggattctat
>_R_gnl|SRA|DRR000628.2138502.2 HWUSI-EAS1632R_0006_FC61F2Y:1:71:8907:6424.
-----------------------aaacagtaggaccatagtgggggattctat
>_R_gnl|SRA|DRR000627.8981841.1 HWUSI-EAS1632R_0006_FC61F2Y:3:47:5727:20444.
-----------------------aaacagtaggaccatagtgggggattctat
>_R_gnl|SRA|DRR000627.6433283.1 HWUSI-EAS1632R_0006_FC61F2Y:2:90:3002:1897.
-----------------------aaacagtaggaccatagtgggggattctat
>_R_gnl|SRA|DRR000628.7322279.1 HWUSI-EAS1632R_0006_FC61F2Y:2:115:18929:20597.
-----------------------cgcaaacaggaccatagtgggggattctat
>_R_gnl|SRA|DRR000628.6118764.1 HWUSI-EAS1632R_0006_FC61F2Y:2:78:17680:12348.
-----------------------cgcaaacaggaccatagtgggggattctat
>_R_gnl|SRA|DRR000627.911912.2 HWUSI-EAS1632R_0006_FC61F2Y:1:28:12049:9618.

-----------------------cgcaaacaggaccatagtgggggattctat
>_R_gnl|SRA|DRR000628.9539261.1 HWUSI-EAS1632R_0006_FC61F2Y:3:64:8977:15904.
------------------------aacagtaggaccatagtgggggattctat
>_R_gnl|SRA|DRR000627.5814621.1 HWUSI-EAS1632R_0006_FC61F2Y:2:69:9904:2308.
------------------------aacagtaggaccatagtgggggattctat
>_R_gnl|SRA|DRR000627.97929.1 HWUSI-EAS1632R_0006_FC61F2Y:1:3:16326:15574.
-------------------------acagtaggaccatagtgggggattctat
>gnl|SRA|DRR000628.11312435.1 HWUSI-EAS1632R_0006_FC61F2Y:3:119:12621:4814.
-------------------------caaacaggaccatagtgggggattctat
>_R_gnl|SRA|DRR000627.4471102.2 HWUSI-EAS1632R_0006_FC61F2Y:2:25:2601:5277.
-------------------------caaacaggaccatagtgggggattctat
>_R_gnl|SRA|DRR000628.4621149.1 HWUSI-EAS1632R_0006_FC61F2Y:2:29:9178:5072.
--------------------------cagtaggaccatagtggggtattctat
>_R_gnl|SRA|DRR000628.10814246.1 HWUSI-EAS1632R_0006_FC61F2Y:3:104:17113:7260.
--------------------------aaacaggaccatagtgggggattctat
>gnl|SRA|DRR000628.7600348.1 HWUSI-EAS1632R_0006_FC61F2Y:3:4:2473:15960.
--------------------------aaacaggaccatagtgggggattctat
>_R_gnl|SRA|DRR000628.3019049.2 HWUSI-EAS1632R_0006_FC61F2Y:1:101:3496:9853.
--------------------------aaacaggaccatagtgggggattctat
>_R_gnl|SRA|DRR000627.8063793.2 HWUSI-EAS1632R_0006_FC61F2Y:3:18:8900:17623.
--------------------------aaacaggaccatagtgggggattctat
>_R_gnl|SRA|DRR000628.10115869.1 HWUSI-EAS1632R_0006_FC61F2Y:3:83:9333:20673.
----------------------------gtaggaccatagtgggggattctat
>gnl|SRA|DRR000627.833632.1 HWUSI-EAS1632R_0006_FC61F2Y:1:26:3472:15792.
----------------------------ccaggaccatagtgggggattctat
>_R_gnl|SRA|DRR000629.2250601.1 HWUSI-EAS1632R_0006_FC61F2Y:1:47:10351:3948.
------------------------------aggaccatagtgggggattctat
>gnl|SRA|DRR000627.10596286.1 HWUSI-EAS1632R_0006_FC61F2Y:3:100:4111:11883.
------------------------------aggaccatagtgggggattctat
>gnl|SRA|DRR000628.10595090.1 HWUSI-EAS1632R_0006_FC61F2Y:3:98:5066:20150.
-----------------------------caggaccatagtgggggattctat
>gnl|SRA|DRR000628.6141496.1 HWUSI-EAS1632R_0006_FC61F2Y:2:79:12562:10176.
-----------------------------caggaccatagtgggggattctat
>gnl|SRA|DRR000628.1181239.2 HWUSI-EAS1632R_0006_FC61F2Y:1:37:12313:10194.
-----------------------------caggaccatagtgggggattctat
>gnl|SRA|DRR000627.8568931.1 HWUSI-EAS1632R_0006_FC61F2Y:3:33:14568:6726.
-----------------------------caggaccatagtgggggattctat
>gnl|SRA|DRR000627.1217258.1 HWUSI-EAS1632R_0006_FC61F2Y:1:38:12593:14363.
-----------------------------caggaccatagtgggggattctat
>gnl|SRA|DRR000627.305828.1 HWUSI-EAS1632R_0006_FC61F2Y:1:9:18920:12513.
-----------------------------caggaccatagtgggggattctat
>gnl|SRA|DRR000628.8555988.2 HWUSI-EAS1632R_0006_FC61F2Y:3:32:14361:2305.
-------------------------------ggaccatagtgggggattctat
>gnl|SRA|DRR000627.8010130.1 HWUSI-EAS1632R_0006_FC61F2Y:3:16:16179:5927.
-------------------------------ggaccatagtgggggattctat
>gnl|SRA|DRR000627.6706767.1 HWUSI-EAS1632R_0006_FC61F2Y:2:98:11957:15842.
-------------------------------ggaccatagtgggggattctat
>gnl|SRA|DRR000627.4077812.2 HWUSI-EAS1632R_0006_FC61F2Y:2:13:6145:6102.
-------------------------------ggaccatagtgggggattctat
>gnl|SRA|DRR000627.1428004.1 HWUSI-EAS1632R_0006_FC61F2Y:1:46:18719:12730.
-------------------------------ggaccatagtgggggattctat

>gnl|SRA|DRR000627.2892672.1 HWUSI-EAS1632R_0006_FC61F2Y:1:97:19046:1563.
--------------------------------gaccatagtgggggattctat
>gnl|SRA|DRR000627.4918780.2 HWUSI-EAS1632R_0006_FC61F2Y:2:38:18664:18266.
-----a--------------cgcaaaca---ggaccatagtgggggattctat
>_R_gnl|SRA|DRR000628.9312572.1 HWUSI-EAS1632R_0006_FC61F2Y:3:57:1332:13827.
-----a--------------cgcaagca---tgaccatagtgggggattctat
>gnl|SRA|DRR000628.4119950.2 HWUSI-EAS1632R_0006_FC61F2Y:2:14:5893:4483.
-----a--------------tgtactcaacttgaccatagtgggggattctat
>gnl|SRA|DRR000627.1481428.1 HWUSI-EAS1632R_0006_FC61F2Y:1:48:15773:20662.
--------------------cgcaaaca---ggaacatagtgggggattcnat
>_R_gnl|SRA|DRR000627.9413329.2 HWUSI-EAS1632R_0006_FC61F2Y:3:61:11619:19432.
tccgat--------------ctcaaacagtaggaccatagtgggggattctat
>_R_gnl|SRA|DRR000628.527754.2 HWUSI-EAS1632R_0006_FC61F2Y:1:16:19406:16657.
acacgacgctcttccgatctcgcaaacagtaggaccatagtgggggattctat
>gnl|SRA|DRR000628.4621149.2 HWUSI-EAS1632R_0006_FC61F2Y:2:29:9178:5072.
--agaa--------------cgcaaacagtaggaccatagtgggggattctat
>gnl|SRA|DRR000627.5327087.2 HWUSI-EAS1632R_0006_FC61F2Y:2:52:15702:11444.
-----------------------------caggaccatagtgggggattctat
>gnl|SRA|DRR000628.527754.1 HWUSI-EAS1632R_0006_FC61F2Y:1:16:19406:16657.
--------------------cgcaaacagtaggaccatagtgggggattctat
>gnl|SRA|DRR000627.9413329.1 HWUSI-EAS1632R_0006_FC61F2Y:3:61:11619:19432.
----------------------caaacagtaggaccatagtgggggattctat
>_R_gnl|SRA|DRR000627.5327087.1 HWUSI-EAS1632R_0006_FC61F2Y:2:52:15702:11444.
tccgatctc---------------------aggaccatagtgggggattctat
>_R_gnl|SRA|DRR000628.7333.1 HWUSI-EAS1632R_0006_FC61F2Y:1:1:5387:1349.
acataa--------------cgcaaacag---gaccatagtgggggattctat
>_R_gnl|SRA|DRR000627.605228.2 HWUSI-EAS1632R_0006_FC61F2Y:1:19:1996:9585.
acagaa--------------cgcaaacagtaggaccatagtgggggattctat
>_R_gnl|SRA|DRR000627.2856664.1 HWUSI-EAS1632R_0006_FC61F2Y:1:96:16611:4859.
gcctaa-------------atatctctagtaggaccatagtgggggattctat
>gnl|SRA|DRR000627.271360.2 HWUSI-EAS1632R_0006_FC61F2Y:1:8:18312:17807.
gcctaa-------------atatctctagtaggaccatagtgggggattctat
>_R_gnl|SRA|DRR000627.2970696.1 HWUSI-EAS1632R_0006_FC61F2Y:1:100:8885:3257.
acagaa--------------cgcaaacagtaggaccatagtgggggattctat
>gnl|SRA|DRR000628.5530199.1 HWUSI-EAS1632R_0006_FC61F2Y:2:58:17412:14336.
-----------------------------caggaccatagtgggggattctat
>_R_gnl|SRA|DRR000628.1512607.2 HWUSI-EAS1632R_0006_FC61F2Y:1:49:19039:1241.
gccgatc-----------------------tggaccatagtgggggattctat
>gnl|SRA|DRR000628.1512607.1 HWUSI-EAS1632R_0006_FC61F2Y:1:49:19039:1241.
-------------------------------ggaccatagtgggggattctat
>gnl|SRA|DRR000628.1644143.1 HWUSI-EAS1632R_0006_FC61F2Y:1:54:9019:11653.
acagaa--------------cgcaaacagtaggaccatagtgggggattctat
>gnl|SRA|DRR000627.6356549.1 HWUSI-EAS1632R_0006_FC61F2Y:2:87:13637:18684.
---gaa--------------agcaaacag---gaccatagtgggggattctat
>_R_gnl|SRA|DRR000627.8513186.1 HWUSI-EAS1632R_0006_FC61F2Y:3:31:19757:13896.
--cgaa--------------cgcaaacag---gaccatagtgggggattctat
>gnl|SRA|DRR000627.10516793.2 HWUSI-EAS1632R_0006_FC61F2Y:3:97:14511:4037.
acagaa--------------cgcaaacagtaggaccatagtgggggattctat
>_R_gnl|SRA|DRR000628.1066210.2 HWUSI-EAS1632R_0006_FC61F2Y:1:33:18176:13791.
acagaa--------------cgcaaacagtaggaccatagtgggggattgtat
>_R_gnl|SRA|DRR000628.3629483.2 HWUSI-EAS1632R_0006_FC61F2Y:1:119:14236:15704.

acagaa--------------cgcaaacagtaggaccatagtgggg--------
>_R_gnl|SRA|DRR000628.7010567.1 HWUSI-EAS1632R_0006_FC61F2Y:2:106:12400:11722.
acagaa--------------cgcaaacagtaggaccatagt------------
>_R_gnl|SRA|DRR000627.827242.2 HWUSI-EAS1632R_0006_FC61F2Y:1:25:18248:17128.
acagaa--------------cgcaa----------------------------
>_R_gnl|SRA|DRR000627.3892751.1 HWUSI-EAS1632R_0006_FC61F2Y:2:7:15963:15780.
acagaa--------------cgcaaacagtaggaccatagcgg----------
>gnl|SRA|DRR000628.9492826.2 HWUSI-EAS1632R_0006_FC61F2Y:3:62:17963:20148.
acagaa--------------cgcaaacagtaggaccatagtgag---------
>_R_gnl|SRA|DRR000628.9492826.1 HWUSI-EAS1632R_0006_FC61F2Y:3:62:17963:20148.
acagaa--------------cgcaaacagtaggaccatagtg-----------
>_R_gnl|SRA|DRR000629.4935579.2 HWUSI-EAS1632R_0006_FC61F2Y:1:106:19255:13038.
acagaa--------------cgcaaac---aggaccatagtgggggattctgt
>_R_gnl|SRA|DRR000627.5479024.2 HWUSI-EAS1632R_0006_FC61F2Y:2:57:17569:15333.
acagaa--------------cgcaaac---aggaccatagtgggggattctat
>_R_gnl|SRA|DRR000627.5635896.2 HWUSI-EAS1632R_0006_FC61F2Y:2:63:8271:6890.
acagaa--------------cgcaaac---aggaccatagtgggggattctat
>_R_gnl|SRA|DRR000627.8111259.1 HWUSI-EAS1632R_0006_FC61F2Y:3:19:16612:19124.
acagaa--------------cgcaaac---aggaccatagtgggggattctat
>gnl|SRA|DRR000627.8111259.2 HWUSI-EAS1632R_0006_FC61F2Y:3:19:16612:19124.
acagaa--------------cgcaaac---aggaccatagtgggggattctat
>gnl|SRA|DRR000627.5635896.1 HWUSI-EAS1632R_0006_FC61F2Y:2:63:8271:6890.
acagaa--------------cgcaaac---aggaccatagtgggggattctat
>gnl|SRA|DRR000627.5479024.1 HWUSI-EAS1632R_0006_FC61F2Y:2:57:17569:15333.
acagaa--------------cgcaaac---aggaccatagtgggggattctat
>gnl|SRA|DRR000629.4935579.1 HWUSI-EAS1632R_0006_FC61F2Y:1:106:19255:13038.
acagaa--------------cgcaaac---aggaccatagtgggggattatgt
>gnl|SRA|DRR000628.5226776.2 HWUSI-EAS1632R_0006_FC61F2Y:2:48:19342:4714.
acagaa--------------cgcaaac---aggaccatagag-----------
>_R_gnl|SRA|DRR000628.5226776.1 HWUSI-EAS1632R_0006_FC61F2Y:2:48:19342:4714.
acagaa--------------cgcaaac---aggaccatag-------------
>_R_gnl|SRA|DRR000627.5804780.2 HWUSI-EAS1632R_0006_FC61F2Y:2:69:3813:4207.
acagaa--------------cgcaaac---agga-------------------
>_R_gnl|SRA|DRR000628.1795780.1 HWUSI-EAS1632R_0006_FC61F2Y:1:59:13505:13614.
acagaa--------------cgcaaac---agg--------------------
>gnl|SRA|DRR000627.5804780.1 HWUSI-EAS1632R_0006_FC61F2Y:2:69:3813:4207.
acagaa--------------cgcaaac---aggaagatcg-------------
>_R_gnl|SRA|DRR000627.10136106.2 HWUSI-EAS1632R_0006_FC61F2Y:3:85:18282:3529.
acagaa--------------cgcaaacagtaggacc-----------------
>gnl|SRA|DRR000627.10136106.1 HWUSI-EAS1632R_0006_FC61F2Y:3:85:18282:3529.
acagaa--------------cgcaaacagtagg--------------------
>_R_gnl|SRA|DRR000628.5503386.2 HWUSI-EAS1632R_0006_FC61F2Y:2:57:19566:10756.
acagaa--------------cgcaaacagtaggac------------------
>_R_gnl|SRA|DRR000627.4150692.1 HWUSI-EAS1632R_0006_FC61F2Y:2:15:8972:11898.
acagaa--------------cgcaaac--------------------------
>_R_gnl|SRA|DRR000628.5557789.1 HWUSI-EAS1632R_0006_FC61F2Y:2:59:16465:7747.
acagaa--------------cgcaaac--------------------------
>gnl|SRA|DRR000627.1489879.2 HWUSI-EAS1632R_0006_FC61F2Y:1:49:2700:4986.
accgaa--------------cgcaaacagtaggaccatagcgggggattctat
>gnl|SRA|DRR000629.14084952.2 HWUSI-EAS1632R_0006_FC61F2Y:3:50:2550:9610.
acagaa--------------cgcaaacag---gaccacagtgggggatcctat

>gnl|SRA|DRR000629.9863389.2 HWUSI-EAS1632R_0006_FC61F2Y:2:88:8123:4581.
acagaa-------------cgcaaaca-----------------------
>gnl|SRA|DRR000627.7353702.2 HWUSI-EAS1632R_0006_FC61F2Y:2:117:17172:9165.
gcaaac---------------caaacagtaggaccatagtgggggattctat
>_R_gnl|SRA|DRR000627.7353702.1 HWUSI-EAS1632R_0006_FC61F2Y:2:117:17172:9165.
gcaaac---------------caaacagtaggaccatagtgggggattctat
>gnl|SRA|DRR000627.6269205.1 HWUSI-EAS1632R_0006_FC61F2Y:2:84:17909:19453.
-------------------cgcaaacagtaggaccatagtgggggattctat
>_R_gnl|SRA|DRR000627.10346114.1 HWUSI-EAS1632R_0006_FC61F2Y:3:92:9878:4126.
acagaa-------------cgcaaacag----------------------
>gnl|SRA|DRR000627.5242225.2 HWUSI-EAS1632R_0006_FC61F2Y:2:49:19504:3714.
acagaa-------------cgcaaacagg----------------------
>gnl|SRA|DRR000627.8942341.1 HWUSI-EAS1632R_0006_FC61F2Y:3:45:11620:17516.
acagaa-------------cgcaaacagtagg-------------------
>gnl|SRA|DRR000627.605228.1 HWUSI-EAS1632R_0006_FC61F2Y:1:19:1996:9585.
acagaa-------------cgcaaacagtagg-------------------
>_R_gnl|SRA|DRR000629.12021130.2 HWUSI-EAS1632R_0006_FC61F2Y:3:9:5012:18983.
-ctgaa-------------cgcaaacagtaggaccatagtggtttattcttt
>_R_gnl|SRA|DRR000629.2356449.2 HWUSI-EAS1632R_0006_FC61F2Y:1:49:17840:19991.
acagaa-------------cgcaaacattag--------------tttttt
>gnl|SRA|DRR000628.4592714.2 HWUSI-EAS1632R_0006_FC61F2Y:2:28:11563:10244.
agggta-------ggagccctgaggcgggtaggaccatagtgggggattctat
>_R_gnl|SRA|DRR000627.1264659.1 HWUSI-EAS1632R_0006_FC61F2Y:1:41:2338:14751.
acagaa-------------cgcaaac------------------------
>gnl|SRA|DRR000629.5756702.1 HWUSI-EAS1632R_0006_FC61F2Y:2:2:4326:3115.
acagaa-------------cgcaaacagcaagaccaca-------------

DRX000333
>control ...
--------------------tagcttacagaacgcaaacagtaggaccata---gtggg
------------ggattc------------
>_R_gnl|SRA|DRR000626.12424240.1 HWUSI-EAS1632R_0006_FC61F2Y:3:116:15304:2721..
caaactgcgccaacaaatcagtagcttacagaacgcaaac---aggaccata---gtggg
------------ggattctatcaacaccat--
>_R_gnl|SRA|DRR000626.12031644.2 HWUSI-EAS1632R_0006_FC61F2Y:3:106:5067:3215..
caaactgcgccaacaaatcagtagcttacagaacgcaaac---aggaccata---gtggg
------------ggattctatcaacaccat--
>_R_gnl|SRA|DRR000626.9931385.1 HWUSI-EAS1632R_0006_FC61F2Y:3:45:4571:17869..
caaactgcgccaacaaatcagtagcttacagaacgcaaac---aggaccata---gtggg
------------ggattctatcaacaccat--
>_R_gnl|SRA|DRR000626.1959760.2 HWUSI-EAS1632R_0006_FC61F2Y:1:58:11589:10127..
caaactgcgccaacaaatcagtagcttacagaacgcaaac---aggaccata---gtggg
------------ggattctatcaacaccat--
>_R_gnl|SRA|DRR000626.9522364.1 HWUSI-EAS1632R_0006_FC61F2Y:3:33:14795:8654..
caaactgcgccaacaaatcagtagcttacagaacgcaaac---aggaccata---gtggg
------------ggattctatcaacaccat--
>_R_gnl|SRA|DRR000626.10108261.1 HWUSI-EAS1632R_0006_FC61F2Y:3:50:15394:14015..
caaactgcgccaacaaatcagtagcttacagaacgcaaac---aggaccata---gtggg
------------ggattctatcaacaccat--

>_R_gnl|SRA|DRR000626.3511541.1 HWUSI-EAS1632R_0006_FC61F2Y:1:105:13575:11648..
caaactgcgccaacaaatcagtagcttacagaacgcaaac---aggaccata---gtggg
-------------ggattctatcaacaccat--
>_R_gnl|SRA|DRR000626.3960455.2 HWUSI-EAS1632R_0006_FC61F2Y:1:118:1394:19432..
caaactgcgccaacaaatcagtagcttacagaacgcaaac---aggaccata---gtggg
-------------ggattctatcaacaccat--
>_R_gnl|SRA|DRR000626.12279553.2 HWUSI-EAS1632R_0006_FC61F2Y:3:112:18252:13755..
caaactgcgccaacaaatcagtagcttacagaacgcaaac---aggaccata---gtggg
-------------ggattctatcaacaccat--
>_R_gnl|SRA|DRR000626.8747374.1 HWUSI-EAS1632R_0006_FC61F2Y:3:12:13583:17650..
caaactgcgccaacaaatcagtagcttacagaacgcaaac---aggaccata---gtggg
-------------ggattctatcaacaccat--
>_R_gnl|SRA|DRR000626.5696761.1 HWUSI-EAS1632R_0006_FC61F2Y:2:45:16597:19407..
caaactgcgccaacaaatcagtagcttacagaacgcaaac---aggaccata---gtggg
-------------ggattctatcaacaccat--
>_R_gnl|SRA|DRR000626.4104773.1 HWUSI-EAS1632R_0006_FC61F2Y:2:1:16020:6534..
caaactgcgccaacaaatcagtagcttacagaacgcaaac---aggaccata---gtggg
-------------ggattctatcaacaccat--
>_R_gnl|SRA|DRR000626.2699602.2 HWUSI-EAS1632R_0006_FC61F2Y:1:81:16728:6441..
caaactgcgccaacaaatcagtagcttacagaacgcaaac---aggaccata---gtggg
-------------ggattctatcaacaccat--
>_R_gnl|SRA|DRR000626.2603416.2 HWUSI-EAS1632R_0006_FC61F2Y:1:78:6788:17163..
caaactgcgccaacaaatcagtagcttacagaacgcaaac---aggaccata---gtggg
-------------ggattctatcaacaccat--
>gnl|SRA|DRR000626.6751059.2 HWUSI-EAS1632R_0006_FC61F2Y:2:77:19598:18501..
caaactgcgccaacaaatcagtagcttacagaacgcaaac---aggaccata---gtggg
-------------ggattctatcaacaccat--
>_R_gnl|SRA|DRR000626.4962748.1 HWUSI-EAS1632R_0006_FC61F2Y:2:24:19636:19654..
caaactgcgccaacaaatcagtagcttacagaacgcaaac---aggaccata---gtggg
-------------ggattctatcaacaccat--
>gnl|SRA|DRR000626.8747374.2 HWUSI-EAS1632R_0006_FC61F2Y:3:12:13583:17650..
caaactgcgccaacaaatcagtagcttacagaacgcaaac---aggaccata---gtggg
-------------ggattctatcaacaccat--
>_R_gnl|SRA|DRR000626.12050219.1 HWUSI-EAS1632R_0006_FC61F2Y:3:106:14395:14455..
caaactgcgccaacaaatcagtagcttacagaacgcaaac---aggaccata---gtggg
-------------ggattctatcaacaccat--
>_R_gnl|SRA|DRR000626.8284636.2 HWUSI-EAS1632R_0006_FC61F2Y:2:120:9237:11039..
caaactgcgccaacaaatcagtagcttacagaacgcaaac---aggaccata---gtggg
-------------ggattctatcaacaccat--
>_R_gnl|SRA|DRR000626.8251522.1 HWUSI-EAS1632R_0006_FC61F2Y:2:119:11925:11808..
caaactgcgccaacaaatcagtagcttacagaacgcaaac---aggaccata---gtggg
-------------ggattctatcaacaccat--
>_R_gnl|SRA|DRR000626.3508004.2 HWUSI-EAS1632R_0006_FC61F2Y:1:105:11795:19520..
caaactgcgccaacaaatcagtagcttacagaacgcaaac---aggaccata---gtggg
-------------ggattctatcaacaccat--
>_R_gnl|SRA|DRR000626.6570265.2 HWUSI-EAS1632R_0006_FC61F2Y:2:72:5431:13836..
caaactgcgccaacaaatcagtagcttacagaacgcaaac---aggaccata---gtggg
-------------ggattctatcaacaccat--
>gnl|SRA|DRR000626.1351401.2 HWUSI-EAS1632R_0006_FC61F2Y:1:38:19633:9708..
caaactgcgccaacaaatcagtagcttacagaacgcaaac---aggaccata---gtggg
-------------ggattctatcaacaccat--

>gnl|SRA|DRR000626.4962748.2 HWUSI-EAS1632R_0006_FC61F2Y:2:24:19636:19654..
caaactgcgccaacaaatcagtagcttacagaacgcaaac---aggaccata---gtggg
-------------ggattctatcaacaccat--
>gnl|SRA|DRR000626.8120419.2 HWUSI-EAS1632R_0006_FC61F2Y:2:116:3570:1814..
caaactgcgccaacaaatcagtagcttacagaacgcaaac---aggaccata---gtggg
-------------ggattctatcaacaccat--
>gnl|SRA|DRR000626.3409113.2 HWUSI-EAS1632R_0006_FC61F2Y:1:102:16139:3182..
caaactgcgccaacaaatcagtagcttacagaacgcaaac---aggaccata---gtggg
-------------ggattctatcaacaccat--
>_R_gnl|SRA|DRR000626.2992442.2 HWUSI-EAS1632R_0006_FC61F2Y:1:91:1288:16450..
caaactgcgccaacaaatcagtagcttacagaacgcaaac---aggaccata---gtggg
-------------ggattctatcaacaccat--
>gnl|SRA|DRR000626.4119003.2 HWUSI-EAS1632R_0006_FC61F2Y:2:2:4447:16412..
caaactgcgccaacaaatcagtagcttacagaacgcaaac---aggaccata---gtggg
-------------ggattctatcaacaccat--
>_R_gnl|SRA|DRR000626.1612891.1 HWUSI-EAS1632R_0006_FC61F2Y:1:47:17748:11799..
caaactgcgccaacaaatcagtagcttacagaacgcaaac---aggaccata---gtggg
-------------ggattctatcaacaccac--
>_R_gnl|SRA|DRR000626.1339521.2 HWUSI-EAS1632R_0006_FC61F2Y:1:38:10715:16611..
caaactgcgccaacaaatcagtagcatacagaacgcaaac---aggaccata---gtggg
-------------ggattctatcaacaccat--
>_R_gnl|SRA|DRR000626.5119819.2 HWUSI-EAS1632R_0006_FC61F2Y:2:29:7407:5247..
caaactgcgccaacaaatcagtagcgtacagaacgcaaac---aggaccata---gtggg
-------------ggattctatcaacaccat--
>_R_gnl|SRA|DRR000626.11804924.1 HWUSI-EAS1632R_0006_FC61F2Y:3:100:1411:18506..
caaactgcgccaaaaaatcagtagcttacagaacgcaaac---aggaccata---gtggg
-------------ggattctatcaacaccat--
>gnl|SRA|DRR000626.1762494.2 HWUSI-EAS1632R_0006_FC61F2Y:1:52:9640:9245..
taaactgcgccaacaaatcagaagcttacagaacgcaaac---aggaccata---gtggg
-------------ggattctatcaacaccat--
>gnl|SRA|DRR000626.7609877.1 HWUSI-EAS1632R_0006_FC61F2Y:2:102:5884:4902..
-aaactgcgccaacaaatcagtagcttacagaacgcaaac---aggaccata---gtggg
-------------ggattctatcaacaccat--
>_R_gnl|SRA|DRR000626.11344496.1 HWUSI-EAS1632R_0006_FC61F2Y:3:87:5833:11679..
-----tgcgccaacaaatcagtagcttacagaacgcaaac---aggaccata---gtggg
-------------ggattctatcaacaccat--
>_R_gnl|SRA|DRR000626.6751059.1 HWUSI-EAS1632R_0006_FC61F2Y:2:77:19598:18501..
-----tgcgccaacaaatcagtagcttacagaacgcaaac---aggaccata---gtggg
-------------ggattctatcaacaccat--
>_R_gnl|SRA|DRR000626.5536285.1 HWUSI-EAS1632R_0006_FC61F2Y:2:41:1090:17176..
-----tgcgccaacaaatcagtagcttacagaacgcaaac---aggaccata---gtggg
-------------ggattctatcaacaccat--
>_R_gnl|SRA|DRR000626.4781662.2 HWUSI-EAS1632R_0006_FC61F2Y:2:20:1742:17954..
-----tgcgccaacaaatcagtagcttacagaacgcaaac---aggaccata---gtggg
-------------ggattctatcaacaccat--
>gnl|SRA|DRR000626.7790369.2 HWUSI-EAS1632R_0006_FC61F2Y:2:107:4888:15090..
------gcgccaacaaatcagtagcttacagaacgcaaac---aggaccata---gtggg
-------------ggattctatcaacaccat--
>gnl|SRA|DRR000626.2742528.2 HWUSI-EAS1632R_0006_FC61F2Y:1:83:14528:12183..
------gcgccaacaaatcagtagcttacagaacgcaaac---aggaccata---gtggg
-------------ggattctatcaacaccat--

>_R_gnl|SRA|DRR000626.5191447.1 HWUSI-EAS1632R_0006_FC61F2Y:2:31:6884:8237..
ccgatctcgccaacaaatcagtagcttacagaacgcaaac---aggaccata---gtggg
-------------ggattctatcaacaccat--
>_R_gnl|SRA|DRR000626.3424839.2 HWUSI-EAS1632R_0006_FC61F2Y:1:103:5946:13754..
ccgatctcgccaacaaatcagtagcttacagaacgcaaac---aggaccata---gtggg
-------------ggattctatcaacaccat--
>gnl|SRA|DRR000626.5191447.2 HWUSI-EAS1632R_0006_FC61F2Y:2:31:6884:8237..
-------cgccaacaaatcagtagcttacagaacgcaaac---aggaccata---gtggg
-------------ggattctatcaacaccat--
>gnl|SRA|DRR000626.3424839.1 HWUSI-EAS1632R_0006_FC61F2Y:1:103:5946:13754..
-------cgccaacaaatcagtagcttacagaacgcaaac---aggaccata---gtggg
-------------ggattctatcaacaccat--
>gnl|SRA|DRR000626.8314861.2 HWUSI-EAS1632R_0006_FC61F2Y:3:1:5509:17320..
---------ccaacaaatcagtagcttacagaacgcaaac---aggaccata---gtggg
-------------ggattctatcaacaccat--
>_R_gnl|SRA|DRR000626.2097183.1 HWUSI-EAS1632R_0006_FC61F2Y:1:62:18334:8869..
---------ccaacaaatcagtagcttacagaacgcaaac---aggaccata---gtggg
-------------ggattctatcaacaccat--
>_R_gnl|SRA|DRR000626.1427321.1 HWUSI-EAS1632R_0006_FC61F2Y:1:42:6639:19873..
----------gaacaaatcagtagcttacagaacgcaaac---aggaccata---gtggg
-------------ggattctatcaacaccat--
>gnl|SRA|DRR000626.1372539.1 HWUSI-EAS1632R_0006_FC61F2Y:1:39:19267:20608..
----------caacaaatcagtagcttacagaacgcaaac---aggaccata---gtggg
-------------ggattctatcaacaccat--
>_R_gnl|SRA|DRR000626.11185227.1 HWUSI-EAS1632R_0006_FC61F2Y:3:82:15271:3661..
------------acaaatcagtagcttacagaacgcaaac---aggaccata---gtggg
-------------ggattctatcaacaccat--
>_R_gnl|SRA|DRR000626.8650858.1 HWUSI-EAS1632R_0006_FC61F2Y:3:10:2853:10945..
------------acaaatcagtagcttacagaacgcaaac---aggaccata---gtggg
-------------gaattctatcaacaccat--
>_R_gnl|SRA|DRR000626.1351401.1 HWUSI-EAS1632R_0006_FC61F2Y:1:38:19633:9708..
----------------atcagtagcttacagaacgcaaac---aggaccata---gtggg
-------------ggattctatcaacaccat--
>_R_gnl|SRA|DRR000626.4376516.1 HWUSI-EAS1632R_0006_FC61F2Y:2:9:2102:14032..
---------------aatcagtagcttacagaacgcaaac---aggaccata---gtggg
-------------ggattctatcaacaccat--
>gnl|SRA|DRR000626.3200706.2 HWUSI-EAS1632R_0006_FC61F2Y:1:96:18430:4755..
---------------catcagtagcttacagaacgcaaac---aggaccata---gtggg
-------------ggattctatcaacaccat--
>_R_gnl|SRA|DRR000626.12127643.2 HWUSI-EAS1632R_0006_FC61F2Y:3:108:16398:10149..
-------------caagtcagtagcttacagaacgcaaac---aggaccata---gtggg
-------------ggattctatcaacaccat--
>gnl|SRA|DRR000626.4742290.2 HWUSI-EAS1632R_0006_FC61F2Y:2:18:18973:16364..
-------------cttatcagtagcttacagaacgcaaac---aggaccata---gtggg
-------------ggattctatcaacaccat--
>_R_gnl|SRA|DRR000626.11927796.2 HWUSI-EAS1632R_0006_FC61F2Y:3:103:8150:16396..
-----------------tcagtagcttacagaacgcaaac---aggaccata---gtggg
-------------ggattctatcaacaccat--
>_R_gnl|SRA|DRR000626.11917831.2 HWUSI-EAS1632R_0006_FC61F2Y:3:103:3229:19376..
-----------------gcagtagcttacagaacgcaaac---aggaccata---gtggg
-------------ggattctatcaacaccat--

>_R_gnl|SRA|DRR000626.1762494.1 HWUSI-EAS1632R_0006_FC61F2Y:1:52:9640:9245..
-----------------------gcttacagaacgcaaac---aggaccata---gtggg
-------------ggattctatcaacaccat--
>_R_gnl|SRA|DRR000626.7943164.1 HWUSI-EAS1632R_0006_FC61F2Y:2:111:7373:20763..
------------------cagtagcttacagaacgcaaac---agggccata---gtggg
-------------ggattctatcaacaccat--
>_R_gnl|SRA|DRR000626.8118018.1 HWUSI-EAS1632R_0006_FC61F2Y:2:116:2374:6614..
---------------------------acagaacgcaaac---aggaccata---gtggg
-------------ggattctatcaacaccat--
>gnl|SRA|DRR000626.10903423.1 HWUSI-EAS1632R_0006_FC61F2Y:3:74:4156:19689..
----------------------------cagaacgcaaac---aggaccata---gtggg
-------------ggattctatcaacaccat--
>_R_gnl|SRA|DRR000626.8403812.2 HWUSI-EAS1632R_0006_FC61F2Y:3:3:11112:14101..
-------------------agtagcatacagaacgcaaac---agggccata---gtggg
-------------ggattctatcaacaccat--
>_R_gnl|SRA|DRR000626.12399224.1 HWUSI-EAS1632R_0006_FC61F2Y:3:116:3227:14444..
--------------------------------acgcaaac---aggaccata---gtggg
-------------ggattctatcaacaccat--
>_R_gnl|SRA|DRR000626.4742290.1 HWUSI-EAS1632R_0006_FC61F2Y:2:18:18973:16364..
--------------------------------acgcaaac---aggaccata---gtggg
-------------ggattctatcaacaccat--
>_R_gnl|SRA|DRR000626.6261998.2 HWUSI-EAS1632R_0006_FC61F2Y:2:63:1340:1122..
--------------------------------gcgcgaac---atgaccata---gtggg
-------------ggattctatcaacgccat--
>gnl|SRA|DRR000626.10364971.2 HWUSI-EAS1632R_0006_FC61F2Y:3:58:5701:1975..
---------------------------------cgcaaac---aggaccata---gtggg
-------------ggattctatcaacaccat--
>_R_gnl|SRA|DRR000626.8314861.1 HWUSI-EAS1632R_0006_FC61F2Y:3:1:5509:17320..
---------------------------------cgcaaac---aggaccata---gtggg
-------------ggattctatcaacaccat--
>gnl|SRA|DRR000626.7762827.2 HWUSI-EAS1632R_0006_FC61F2Y:2:106:9489:10720..
---------------------------------cgcaaac---aggaccata---gtggg
-------------ggattctatcaacaccat--
>_R_gnl|SRA|DRR000626.7762827.1 HWUSI-EAS1632R_0006_FC61F2Y:2:106:9489:10720..
---------------------------------cgcaaac---aggaccata---gtggg
-------------ggattctatcaacaccat--
>gnl|SRA|DRR000626.8772069.2 HWUSI-EAS1632R_0006_FC61F2Y:3:13:7359:6704..
----------------------ttctgcatgaacgcaaac---aggaccata---gtggg
-------------ggattctatcaacaccat--
>_R_gnl|SRA|DRR000626.4119003.1 HWUSI-EAS1632R_0006_FC61F2Y:2:2:4447:16412..
----------------------------------gcaaac---aggaccata---gtggg
-------------ggattctatcaacaccat--
>gnl|SRA|DRR000626.2558745.1 HWUSI-EAS1632R_0006_FC61F2Y:1:76:19583:10743..
-----------------------------------caaac---aggaccata---gtggg
-------------ggattctatcaacaccat--
>gnl|SRA|DRR000626.5766128.1 HWUSI-EAS1632R_0006_FC61F2Y:2:48:4715:20799..
---------------------------------------c---aggaccata---gtggg
-------------ggattctatcaacaccat--
>gnl|SRA|DRR000626.9204803.2 HWUSI-EAS1632R_0006_FC61F2Y:3:25:2036:4371..
--ggaccata---gtggg
-------------ggattctatcaacaccat--

>gnl|SRA|DRR000626.438903.1 HWUSI-EAS1632R_0006_FC61F2Y:1:12:17484:6591..
--ggaccata---gtggg
-------------ggattctatcaacaccat--
>_R_gnl|SRA|DRR000626.12351092.2 HWUSI-EAS1632R_0006_FC61F2Y:3:114:16585:20326..
--------------aaatcagtagcttacagaacgcaaacagtaggaccata---gtggg
-------------ggattctatcaacaccat--
>_R_gnl|SRA|DRR000626.11531304.1 HWUSI-EAS1632R_0006_FC61F2Y:3:92:9613:10424..
-------------caaatcagtagcttacagaacgcaaacagtaggaccata---gtggg
-------------ggattctatcaacaccat--
>gnl|SRA|DRR000626.10262585.1 HWUSI-EAS1632R_0006_FC61F2Y:3:55:5748:13155..
-------------caaatcagtagcttacagaacgcaaacagtaggaccata---gtggg
-------------ggattctatcaacaccat--
>_R_gnl|SRA|DRR000626.9722855.2 HWUSI-EAS1632R_0006_FC61F2Y:3:39:6916:15767..
-------------caaatcagtagcttacagaacgcaaacagtaggaccata---gtggg
-------------ggattctatcaacaccat--
>_R_gnl|SRA|DRR000626.7299569.1 HWUSI-EAS1632R_0006_FC61F2Y:2:93:13035:10946..
-------------caaatcagtagcttacagaacgcaaacagtaggaccata---gtggg
-------------ggattctatcaacaccat--
>_R_gnl|SRA|DRR000626.4307191.2 HWUSI-EAS1632R_0006_FC61F2Y:2:7:4684:15239..
-------------caaatcagtagcttacagaacgcaaacagtaggaccata---gtggg
-------------ggattctatcaacaccat--
>_R_gnl|SRA|DRR000626.3083027.1 HWUSI-EAS1632R_0006_FC61F2Y:1:93:11972:19845..
-------------caaatcagtagcttacagaacgcaaacagtaggaccata---gtggg
-------------ggattctatcaacaccat--
>_R_gnl|SRA|DRR000626.1679074.2 HWUSI-EAS1632R_0006_FC61F2Y:1:49:17820:19976..
-------------caaatcagtagcttacagaaagcaaacagtaggaccata---gtggg
-------------ggattctatcaacaccat--
>_R_gnl|SRA|DRR000626.8511047.1 HWUSI-EAS1632R_0006_FC61F2Y:3:6:7689:16339..
------------acaaatcagtagcttacagaacgcaaacagtaggaccata---gtggg
-------------ggattctatcaacaccat--
>_R_gnl|SRA|DRR000626.12241937.1 HWUSI-EAS1632R_0006_FC61F2Y:3:111:17970:16798..
----------caacaaatcagtagcttacagaacgcaaacagtaggaccata---gtggg
-------------ggattctatcaacaccat--
>_R_gnl|SRA|DRR000626.11837789.2 HWUSI-EAS1632R_0006_FC61F2Y:3:100:17973:11823..
--------gccaacaaatcagtagcttacagaacgcaaacagtaggaccata---gtggg
-------------ggattctatcaacaccat--
>_R_gnl|SRA|DRR000626.3670496.2 HWUSI-EAS1632R_0006_FC61F2Y:1:110:2916:1820..
--------gccaacaaatcagtagcttacagaacgcaaacagtaggaccata---gtggg
-------------ggattctatcaacaccat--
>_R_gnl|SRA|DRR000626.3104507.1 HWUSI-EAS1632R_0006_FC61F2Y:1:94:4947:8845..
--------gccaacaaatcagtagcttacagaacgcaaacagtaggaccata---gtggg
-------------ggattctatcaacaccat--
>_R_gnl|SRA|DRR000626.4637988.2 HWUSI-EAS1632R_0006_FC61F2Y:2:16:3821:10500..
--------gccaacaaatcagtagcttacagaacgcaaacagtaggaccata---gtggg
-------------ggattctatcaacaccat--
>_R_gnl|SRA|DRR000626.9858221.2 HWUSI-EAS1632R_0006_FC61F2Y:3:43:2657:14040..
------gcgccaacaaatcagtagcttacagaacgcaaacagtaggaccata---gtggg
-------------ggattctatcaacaccat--
>_R_gnl|SRA|DRR000626.8839032.1 HWUSI-EAS1632R_0006_FC61F2Y:3:15:3641:2810..
------gcgccaacaaatcagtagcttacagaacgcaaacagtaggaccata---gtggg
-------------ggattctatcaacaccat--

>_R_gnl|SRA|DRR000626.5647801.1 HWUSI-EAS1632R_0006_FC61F2Y:2:44:6656:17824..
------gcgccaacaaatcagtagcttacagaacgcaaacagtaggaccata---gtggg
-------------ggattctatcaacaccat--
>_R_gnl|SRA|DRR000626.992047.2 HWUSI-EAS1632R_0006_FC61F2Y:1:28:7705:18885..
------gcgccaacaaatcagtagcttacagaacgcaaacagtaggaccata---gtggg
-------------ggattctatcaacaccat--
>_R_gnl|SRA|DRR000626.11886682.1 HWUSI-EAS1632R_0006_FC61F2Y:3:102:5820:17810..
----ctgcgccaacaaatcagtagcttacagaacgcaaacagtaggaccata---gtggg
-------------ggattctatcaacaccat--
>_R_gnl|SRA|DRR000626.1919508.1 HWUSI-EAS1632R_0006_FC61F2Y:1:57:6406:19139..
----ctgcgccaacaaatcagtagcttacagaacgcaaacagtaggaccgta---gtggg
-------------ggattctatcaacaccat--
>_R_gnl|SRA|DRR000626.8439325.1 HWUSI-EAS1632R_0006_FC61F2Y:3:4:9750:11316..
---actgcgccaacaaatcagtagcttacagaacgcaaacagtaggaccata---gtggg
-------------ggattctatcaacaccat--
>_R_gnl|SRA|DRR000626.85939.1 HWUSI-EAS1632R_0006_FC61F2Y:1:3:6234:7662..
---actgcgccaacaaatcagtagcttacagaacgcaaacagtaggaccata---gtggg
-------------ggattctatcaacaccat--
>gnl|SRA|DRR000626.1950215.2 HWUSI-EAS1632R_0006_FC61F2Y:1:58:5642:10446..
-------cgccaacaaatcagtagcttacagaacgcaaacagtaggaccata---gtggg
-------------ggattctatcaacaccat--
>_R_gnl|SRA|DRR000626.2369177.1 HWUSI-EAS1632R_0006_FC61F2Y:1:71:6158:12315..
-------cgccaacaaatcagtagcttacagaacgcagacagtaggaccata---gtggg
-------------ggattctatcaacaccat--
>_R_gnl|SRA|DRR000626.3388163.2 HWUSI-EAS1632R_0006_FC61F2Y:1:102:5494:15244..
-------cgccaacaaatcagtagcgtacagaacgcaaacagtaggaccata---gtggg
-------------ggattctatcaacaccat--
>_R_gnl|SRA|DRR000626.10367988.1 HWUSI-EAS1632R_0006_FC61F2Y:3:58:7391:8963..
---------ccaacaaatcagtagcttacagaacgcaaacagtaggaccata---gtggg
-------------ggattctatcaacaccat--
>_R_gnl|SRA|DRR000626.631717.1 HWUSI-EAS1632R_0006_FC61F2Y:1:18:5720:8903..
---------ccaacaaatcagtagcttacagaacgcaaacagtaggaccata---gtggg
-------------agattctatcaacaccat--
>_R_gnl|SRA|DRR000626.3467895.2 HWUSI-EAS1632R_0006_FC61F2Y:1:104:9636:1587..
---------ccaacaaatcaggagcttacagaacgcaaacagtaggaccata---gtggg
-------------ggattctatcaacaccat--
>gnl|SRA|DRR000626.1138282.1 HWUSI-EAS1632R_0006_FC61F2Y:1:32:12759:13552..
-----------aacaaatcagtagcttacagaacgcaaacagtaggaccata---gtggg
-------------ggattctatcaacaccat--
>_R_gnl|SRA|DRR000626.11161775.1 HWUSI-EAS1632R_0006_FC61F2Y:3:82:3190:16446..
---------------aatcagtagcttacagaacgcaaacagtaggaccata---gtggg
-------------ggattctatcaacaccat--
>_R_gnl|SRA|DRR000626.10262585.2 HWUSI-EAS1632R_0006_FC61F2Y:3:55:5748:13155..
---------------aatcagtagcttacagaacgcaaacagtaggaccata---gtggg
-------------ggattctatcaacaccat--
>_R_gnl|SRA|DRR000626.2826057.1 HWUSI-EAS1632R_0006_FC61F2Y:1:86:4747:18903..
---------------aatcagtagcttacagaacgcaaacagtaggaccata---gtggg
-------------ggattctatcaacaccat--
>_R_gnl|SRA|DRR000626.3858577.2 HWUSI-EAS1632R_0006_FC61F2Y:1:115:5756:17677..
---------------aatcagtagcgtacagaacgcaaacagtaggaccata---gtggg
-------------ggattctatcaacaccat--

>_R_gnl|SRA|DRR000626.3624401.1 HWUSI-EAS1632R_0006_FC61F2Y:1:108:16291:14922..
-----------------tcagtagcttacagaacgcaaacagtaggaccata---gtggg
-------------ggattcgatcaacaccat--
>_R_gnl|SRA|DRR000626.332404.2 HWUSI-EAS1632R_0006_FC61F2Y:1:9:18981:8305..
-----------------tcagtagcttacagaacgcaaacagtaggaccata---gtggg
-------------ggattctatcaacaccat--
>_R_gnl|SRA|DRR000626.3911633.2 HWUSI-EAS1632R_0006_FC61F2Y:1:116:13745:3095..
----------------agcagtagcttacagaacgcaaacagtaggaccata---gtggg
-------------ggattctatcaacaccat--
>_R_gnl|SRA|DRR000626.3296004.1 HWUSI-EAS1632R_0006_FC61F2Y:1:99:12615:19248..
------------------cagtagcttacagaacgcaaacagtaggaccata---gtggg
-------------ggattctatcaacaccat--
>_R_gnl|SRA|DRR000626.4696504.2 HWUSI-EAS1632R_0006_FC61F2Y:2:17:14319:18697..
--------------------gtagcttacagaacgcaaacagtaggaccata---gtggg
-------------ggattctatcaacaccat--
>_R_gnl|SRA|DRR000626.1196119.1 HWUSI-EAS1632R_0006_FC61F2Y:1:34:6482:8890..
--------------------gtagcttacagaacgcaaacagtaggaccata---gtggg
-------------ggattctatcaacaccat--
>_R_gnl|SRA|DRR000626.11789738.1 HWUSI-EAS1632R_0006_FC61F2Y:3:99:11958:11301..
--------------------atctcttacagaacgcaaacagtaggaccata---gtggg
-------------ggattctatcaacaccat--
>_R_gnl|SRA|DRR000626.1580913.2 HWUSI-EAS1632R_0006_FC61F2Y:1:46:18402:16740..
--------------------atctcttacagaacgcaaacagtaggaccata---gtggg
-------------ggattctatcaacaccat--
>_R_gnl|SRA|DRR000626.11159645.1 HWUSI-EAS1632R_0006_FC61F2Y:3:82:2107:14151..
-----------------------gcttacagaacgcaaacagtaggaccata---gtggg
-------------ggattctatcaacaccat--
>_R_gnl|SRA|DRR000626.5812128.1 HWUSI-EAS1632R_0006_FC61F2Y:2:49:11054:4785..
----------------------agcttacagaacgcaaacagtaggaccata---gtggg
-------------ggattctatcaacaccat--
>_R_gnl|SRA|DRR000626.2298860.2 HWUSI-EAS1632R_0006_FC61F2Y:1:69:3667:4364..
----------------------agcttacagaacgcaaacagtaggaccata---gtggg
-------------ggattctatcaacaccat--
>_R_gnl|SRA|DRR000626.1170541.2 HWUSI-EAS1632R_0006_FC61F2Y:1:33:11277:18001..
----------------------atcttacagaacgcaaacagtaggaccata---gtggg
-------------ggattctatcaacaccat--
>gnl|SRA|DRR000626.11789738.2 HWUSI-EAS1632R_0006_FC61F2Y:3:99:11958:11301..
------------------------cttacagaacgcaaacagtaggaccata---gtggg
-------------ggattctatcaacaccat--
>gnl|SRA|DRR000626.1580913.1 HWUSI-EAS1632R_0006_FC61F2Y:1:46:18402:16740..
------------------------cttacagaacgcaaacagtaggaccata---gtggg
-------------ggattctatcaacaccat--
>gnl|SRA|DRR000626.10015791.1 HWUSI-EAS1632R_0006_FC61F2Y:3:48:3392:12029..
--------------------------tacagaacgcaaacagtaggaccata---gtggg
-------------ggattctatcaacaccat--
>gnl|SRA|DRR000626.7318191.2 HWUSI-EAS1632R_0006_FC61F2Y:2:94:4282:18172..
--------------------------tacagaacgcaaacagtaggaccata---gtggg
-------------ggattctatcaacaccat--
>_R_gnl|SRA|DRR000626.5305892.1 HWUSI-EAS1632R_0006_FC61F2Y:2:34:10366:5616..
--------------------------tacagaacgcaaacagtaggaccata---gtggg
-------------ggattctatcaacaccat--

>_R_gnl|SRA|DRR000626.11353526.2 HWUSI-EAS1632R_0006_FC61F2Y:3:87:10435:16178..
---------------------------acagaacgcaaacagtaggaccata---gtggg
-------------ggattctatcaacaccat--
>_R_gnl|SRA|DRR000626.4006681.2 HWUSI-EAS1632R_0006_FC61F2Y:1:119:5752:10558..
----------------------------cagaacgcaaacagtaggaccata---gtggg
-------------ggattctatcaacaccat--
>gnl|SRA|DRR000626.11793167.2 HWUSI-EAS1632R_0006_FC61F2Y:3:99:13720:8378..
----------------------------cagaacgcaaacagtaggaccata---gtggg
-------------gtattctatcaacaccat--
>_R_gnl|SRA|DRR000626.5629485.2 HWUSI-EAS1632R_0006_FC61F2Y:2:43:14738:3857..
-----------------------------agaacgcaaacagtaggaccata---gtggg
-------------ggattctatcaacaccat--
>_R_gnl|SRA|DRR000626.11023550.1 HWUSI-EAS1632R_0006_FC61F2Y:3:78:4607:17311..
------------------------------gaacgcaaacagtaggaccata---gtggg
-------------ggattctatcaacaccat--
>_R_gnl|SRA|DRR000626.10040134.1 HWUSI-EAS1632R_0006_FC61F2Y:3:48:16260:16600..
------------------------------gaacgcaaacagtaggaccata---gtggg
-------------ggattctatcaacaccat--
>_R_gnl|SRA|DRR000626.8190734.2 HWUSI-EAS1632R_0006_FC61F2Y:2:117:19766:6264..
------------------------------gaacgcaaacagtaggaccata---gtggg
-------------ggattctatcaacaccat--
>_R_gnl|SRA|DRR000626.268561.1 HWUSI-EAS1632R_0006_FC61F2Y:1:8:5255:15202..
------------------------------gaacgcaaacagtaggaccata---gtggg
-------------ggattctatcaacaccat--
>gnl|SRA|DRR000626.9928135.2 HWUSI-EAS1632R_0006_FC61F2Y:3:45:2684:12206..
-------------------------------aacgcaaacagtaggaccata---gtggg
-------------ggattctatcaacaccat--
>gnl|SRA|DRR000626.8856459.1 HWUSI-EAS1632R_0006_FC61F2Y:3:15:12310:14597..
---------------------------------cgcaaacagtaggaccata---gtggg
-------------ggattctatcaacaccat--
>gnl|SRA|DRR000626.8755441.2 HWUSI-EAS1632R_0006_FC61F2Y:3:12:17521:13657..
---------------------------------cgcaaacagtaggaccata---gtggg
-------------ggattctatcaacaccat--
>gnl|SRA|DRR000626.6813907.1 HWUSI-EAS1632R_0006_FC61F2Y:2:79:17022:17356..
---------------------------------cgcaaacagtaggaccata---gtggg
-------------ggattctatcaacaccat--
>gnl|SRA|DRR000626.11180751.2 HWUSI-EAS1632R_0006_FC61F2Y:3:82:12900:14706..
----------------------------------gcaaacagtaggaccata---gtggg
-------------ggattctatcaacaccat--
>gnl|SRA|DRR000626.8320220.1 HWUSI-EAS1632R_0006_FC61F2Y:3:1:8085:7828..
----------------------------------gcaaacagtaggaccata---gtggg
-------------ggattctatcaacaccat--
>gnl|SRA|DRR000626.316876.2 HWUSI-EAS1632R_0006_FC61F2Y:1:9:11089:4026..
----------------------------------gcaaacagtaggaccata---gtggg
-------------ggattctatcaacaccat--
>_R_gnl|SRA|DRR000626.316876.1 HWUSI-EAS1632R_0006_FC61F2Y:1:9:11089:4026..
------------------tgtgctcttccgatctgcaaacagtaggaccata---gtggg
-------------ggattctatcaacaccat--
>_R_gnl|SRA|DRR000626.8612884.1 HWUSI-EAS1632R_0006_FC61F2Y:3:9:2541:10013..
-----------------------------------caaacagtaggaccata---gtggg
-------------ggattctatcaacaccat--

>gnl|SRA|DRR000626.3990800.1 HWUSI-EAS1632R_0006_FC61F2Y:1:118:16322:19022..
-----------------------------------caaacagtaggaccata---gtggg
-------------ggattctatcaacaccat--
>gnl|SRA|DRR000626.467615.1 HWUSI-EAS1632R_0006_FC61F2Y:1:13:13641:18215..
-----------------------------------caaacagtaggaccata---gtggg
-------------ggattctatcaacaccat--
>_R_gnl|SRA|DRR000626.633482.1 HWUSI-EAS1632R_0006_FC61F2Y:1:18:6615:10134..
-----------------------------------caaacagtaggaccata---gtggg
-------------ggattctatcaacaccat--
>_R_gnl|SRA|DRR000626.11800101.1 HWUSI-EAS1632R_0006_FC61F2Y:3:99:17407:5756..
------------------------------------aaacagtaggaccata---gtggg
-------------ggattctatcaacaccat--
>_R_gnl|SRA|DRR000626.10769484.1 HWUSI-EAS1632R_0006_FC61F2Y:3:70:5520:5723..
------------------------------------aaacagtaggaccata---gtggg
-------------ggattctatcaacaccat--
>_R_gnl|SRA|DRR000626.6439939.1 HWUSI-EAS1632R_0006_FC61F2Y:2:68:7937:13517..
------------------------------------aaacagtaggaccata---gtggg
-------------ggattctatcaacaccat--
>gnl|SRA|DRR000626.70958.1 HWUSI-EAS1632R_0006_FC61F2Y:1:2:17196:8372..
-------------------------------------aacagtaggaccata---gtggg
-------------ggattctatcaacaccat--
>gnl|SRA|DRR000626.5880699.2 HWUSI-EAS1632R_0006_FC61F2Y:2:51:10930:11176..
-------------------------ttacagaacgcaaaaagtaggaccata---gtggg
-------------ggattctatcaacaccat--
>gnl|SRA|DRR000626.9648190.1 HWUSI-EAS1632R_0006_FC61F2Y:3:37:5434:6746..
---gtaggaccata---gtggg
-------------ggattctatcaacaccat--
>gnl|SRA|DRR000626.8062718.1 HWUSI-EAS1632R_0006_FC61F2Y:2:114:11738:11870..
---gtaggaccata---gtggg
-------------ggattctatcaacaccat--
>_R_gnl|SRA|DRR000626.3604983.1 HWUSI-EAS1632R_0006_FC61F2Y:1:108:6508:13484..
---gtaggaccata---gtggg
-------------ggattctatcaacaccat--
>_R_gnl|SRA|DRR000626.3069503.1 HWUSI-EAS1632R_0006_FC61F2Y:1:93:5026:7158..
---gtaggaccata---gtggg
-------------ggattctatcaacaccat--
>gnl|SRA|DRR000626.1981431.2 HWUSI-EAS1632R_0006_FC61F2Y:1:59:6129:15138..
---gtaggaccata---gtggg
-------------ggattctatcaacaccat--
>gnl|SRA|DRR000626.766030.1 HWUSI-EAS1632R_0006_FC61F2Y:1:21:19279:3205..
---gtaggaccata---gtggg
-------------ggattctatcaacaccat--
>_R_gnl|SRA|DRR000626.11765495.2 HWUSI-EAS1632R_0006_FC61F2Y:3:98:18303:2369..
----ccgcgccaacaaagcaggagcatacagaacgcaaacagtaggacccta---gtggg
-------------ggattctatcaacaccat--
>gnl|SRA|DRR000626.3808595.1 HWUSI-EAS1632R_0006_FC61F2Y:1:113:17309:19819..
--taggaccata---gtggg
-------------ggattctatcaacaccat--
>_R_gnl|SRA|DRR000626.5404408.2 HWUSI-EAS1632R_0006_FC61F2Y:2:37:6145:20679..
--tctaggaccata---gtggg
-------------ggattctatcaacaccat--

>gnl|SRA|DRR000626.5404408.1 HWUSI-EAS1632R_0006_FC61F2Y:2:37:6145:20679..
---aggaccata---gtggg
-------------ggattctatcaacaccat--
>_R_gnl|SRA|DRR000626.4168105.1 HWUSI-EAS1632R_0006_FC61F2Y:2:3:10143:17196..
---aggaccata---gtggg
-------------ggattctatcaacaccat--
>_R_gnl|SRA|DRR000626.3200706.1 HWUSI-EAS1632R_0006_FC61F2Y:1:96:18430:4755..
--------------------------------acgctaac---aggcccata---gtggg
-------------ggattctatcaactccat--
>_R_gnl|SRA|DRR000626.12249421.2 HWUSI-EAS1632R_0006_FC61F2Y:3:112:3205:12367..
--------------aacacagtagcttacagagcgcaagcactatgaccata---gtggg
-------------ggattctatcaacaccat--
>_R_gnl|SRA|DRR000626.11506160.1 HWUSI-EAS1632R_0006_FC61F2Y:3:91:15273:17556..
caaactgcgccaacaaatcagtagcttacagaacgcaaacagtaggaccata---gtggg
-------------ggattc--------------
>_R_gnl|SRA|DRR000626.6880380.2 HWUSI-EAS1632R_0006_FC61F2Y:2:81:15523:3739..
caaactgcgccaacaaatcagtagcttacagaacgcaaacagtaggaccata---gtggg
-------------ggattc--------------
>_R_gnl|SRA|DRR000626.2944669.1 HWUSI-EAS1632R_0006_FC61F2Y:1:89:12682:3182..
caaactgcgccaacaaatcagtagcttacagaacgcaaacagtaggaccata---gtggg
-------------ggattct-------------
>_R_gnl|SRA|DRR000626.974090.1 HWUSI-EAS1632R_0006_FC61F2Y:1:27:16498:6907..
caaactgcgcgaacaaatcagtagcttacagaacgcaaacagtaggaccata---gtggg
-------------ggattct-------------
>gnl|SRA|DRR000626.11837789.1 HWUSI-EAS1632R_0006_FC61F2Y:3:100:17973:11823..
caaactgcgccaacaaatcagtagcttacagaacgcaaacagtaggaccata---gtggg
-------------ggattctatcaac-------
>_R_gnl|SRA|DRR000626.6187802.1 HWUSI-EAS1632R_0006_FC61F2Y:2:60:14435:13817..
caaactgcgccaacaaatcagtagcttacagaacgcaaacagtaggaccata---gtggg
-------------ggattctatcaac-------
>gnl|SRA|DRR000626.12423953.2 HWUSI-EAS1632R_0006_FC61F2Y:3:116:15166:13045..
caaactgcgccaacaaatcagtagcttacagaacgcaaacagtaggaccata---gtggg
-------------ggattctatcaacaccat--
>gnl|SRA|DRR000626.3104507.2 HWUSI-EAS1632R_0006_FC61F2Y:1:94:4947:8845..
caaactgcgccaacaaatcagtagcttacagaacgcaaacagtaggaccata---gtggg
-------------ggattctatcaacaccat--
>gnl|SRA|DRR000626.10562084.2 HWUSI-EAS1632R_0006_FC61F2Y:3:64:3390:6494..
caaactgcgccaacaaatcagtagcttacagaacgcaaacagtaggaccata---gtggg
-------------ggattctatcaacaccat--
>_R_gnl|SRA|DRR000626.2156005.1 HWUSI-EAS1632R_0006_FC61F2Y:1:64:15013:20110..
caaactgcgccaacaaatcagtagcttacagaacgcaaacagtaggaccata---gtggg
-------------ggattctatcaacaccat--
>_R_gnl|SRA|DRR000626.7079939.1 HWUSI-EAS1632R_0006_FC61F2Y:2:87:9949:20331..
caaactgcgccaacaaatcagtagcttacagaacgcaaacagtaggaccata---gtggg
-------------ggattctatcaacaccat--
>gnl|SRA|DRR000626.1679074.1 HWUSI-EAS1632R_0006_FC61F2Y:1:49:17820:19976..
caaactgcgccaacaaatcagtagcttacagaacgcaaacagtaggaccata---gtggg
-------------ggattctatcaacaccat--
>_R_gnl|SRA|DRR000626.9947128.1 HWUSI-EAS1632R_0006_FC61F2Y:3:45:14747:8830..
caaactgcgccaacaaatcagtagcttacagaacgcaaacagtaggaccata---gtggg
-------------ggattctatcaacaccat--

>_R_gnl|SRA|DRR000626.9136575.1 HWUSI-EAS1632R_0006_FC61F2Y:3:23:4767:13111..
caaactgcgccaacaaatcagtagcttacagaacgcaaacagtaggaccata---gtggg
-------------ggattctatcaacaccat--
>_R_gnl|SRA|DRR000626.6085870.1 HWUSI-EAS1632R_0006_FC61F2Y:2:57:12207:13902..
caaactgcgccaacaaatcagtagcttacagaacgcaaacagtaggaccata---gtggg
-------------ggattctatcaacaccat--
>_R_gnl|SRA|DRR000626.8508617.1 HWUSI-EAS1632R_0006_FC61F2Y:3:6:6511:18689..
caaactgcgccaacaaatcagtagcttacagaacgcaaacagtaggaccata---gtggg
-------------ggattctatcaacaccat--
>_R_gnl|SRA|DRR000626.3156797.2 HWUSI-EAS1632R_0006_FC61F2Y:1:95:13836:10506..
caaactgcgccaacaaatcagtagcttacagaacgcaaacagtaggaccata---gtggg
-------------ggattctatcaacaccat--
>gnl|SRA|DRR000626.992047.1 HWUSI-EAS1632R_0006_FC61F2Y:1:28:7705:18885..
caaactgcgccaacaaatcagtagcttacagaacgcaaacagtaggaccata---gtggg
-------------ggattctatcaacaccan--
>gnl|SRA|DRR000626.8508617.2 HWUSI-EAS1632R_0006_FC61F2Y:3:6:6511:18689..
caaactgcgccaacaaatcagtagcttacagaacgcaaacagtaggaccata---gtggg
-------------ggattctatcaacaccat--
>gnl|SRA|DRR000626.7422414.2 HWUSI-EAS1632R_0006_FC61F2Y:2:97:2746:11402..
caaactgcgccaacaaatcagtagcttacagaacgcaaacagtaggaccata---gtggg
-------------ggattctatcaacaccat--
>_R_gnl|SRA|DRR000626.7315693.1 HWUSI-EAS1632R_0006_FC61F2Y:2:94:3028:6975..
caaactgcgccaacaaatcagtagcttacagaacgcaaacagtaggaccata---gtggg
-------------ggattctatcaacaccat--
>_R_gnl|SRA|DRR000626.4140301.1 HWUSI-EAS1632R_0006_FC61F2Y:2:2:14910:14699..
caaactgcgccaacaaatcagtagcttacagaacgcaaacagtaggaccata---gtggg
-------------ggattctatcaacaccat--
>_R_gnl|SRA|DRR000626.1112341.1 HWUSI-EAS1632R_0006_FC61F2Y:1:31:17474:5808..
caaactgcgccaacaaatcagtagcttacagaacgcaaacagtaggaccata---gtggg
-------------ggattctatcaacaccat--
>_R_gnl|SRA|DRR000626.768619.1 HWUSI-EAS1632R_0006_FC61F2Y:1:22:2168:1742..
caaactgcgccaacaaatcagtagcttacagaacgcaaacagtaggaccata---gtggg
-------------ggattctatcaacaccat--
>_R_gnl|SRA|DRR000626.452493.1 HWUSI-EAS1632R_0006_FC61F2Y:1:13:6039:14386..
caaactgcgccaacaaatcagtagcttacagaacgcaaacagtaggaccata---gtggg
-------------ggattctatcaacaccat--
>_R_gnl|SRA|DRR000626.5834509.1 HWUSI-EAS1632R_0006_FC61F2Y:2:50:4699:14139..
caaactgcgccaacaaatcagtagcttacagaacgcaaacagtaggaccata---gtggg
-------------ggattctatcaacaccat--
>_R_gnl|SRA|DRR000626.3878567.2 HWUSI-EAS1632R_0006_FC61F2Y:1:115:15771:19514..
caaactgcgccaacaaatcagtagcttacagaacgcaaacagtaggaccata---gtggg
-------------ggattctatcaacaccat--
>gnl|SRA|DRR000626.2045430.1 HWUSI-EAS1632R_0006_FC61F2Y:1:61:6869:15150..
caaactgcgccaacaaatcagtagcttacagaacgcaaacagtaggaccata---gtggg
-------------ggattctatcaacaccat--
>gnl|SRA|DRR000626.11800101.2 HWUSI-EAS1632R_0006_FC61F2Y:3:99:17407:5756..
caaactgcgccaacaaatcagtagcttacagaacgcaaacagtaggaccata---gtggg
-------------ggattctatcaacaccat--
>_R_gnl|SRA|DRR000626.3185389.2 HWUSI-EAS1632R_0006_FC61F2Y:1:96:10406:8817..
caaactgcgccaacaaatcagtagcttacagaacgcaaacagtaggaccata---gtggg
-------------ggattctatcaacaccat--

>_R_gnl|SRA|DRR000626.9268918.1 HWUSI-EAS1632R_0006_FC61F2Y:3:26:15897:16597..
caaactgcgccaacaaatcagtagcttacagaacgcaaacagtaggaccata---gtggg
-------------ggattctatcaacaccat--
>_R_gnl|SRA|DRR000626.1756603.1 HWUSI-EAS1632R_0006_FC61F2Y:1:52:6311:8679..
caaactgcgccaacaaatcagtagcttacagaacgcaaacagtaggaccata---gtggg
-------------ggattctatcaacaccat--
>_R_gnl|SRA|DRR000626.7026181.1 HWUSI-EAS1632R_0006_FC61F2Y:2:85:18743:14369..
caaactgcgccaacaaatcagtagcttacagaacgcaaacagtaggaccata---gtggg
-------------ggattctatcaacaccat--
>_R_gnl|SRA|DRR000626.6801575.1 HWUSI-EAS1632R_0006_FC61F2Y:2:79:10427:14484..
caaactgcgccaacaaatcagtagcttacagaacgcaaacagtaggaccata---gtggg
-------------ggattctatcaacaccat--
>_R_gnl|SRA|DRR000626.4527283.2 HWUSI-EAS1632R_0006_FC61F2Y:2:13:3869:6697..
caaactgcgccaacaaatcagtagcttacagaacgcaaacagtaggaccata---gtggg
-------------ggattctatcaacaccat--
>gnl|SRA|DRR000626.3604983.2 HWUSI-EAS1632R_0006_FC61F2Y:1:108:6508:13484..
caaactgcgccaacaaatcagtagcttacagaacgcaaacagtaggaccata---gtggg
-------------ggattctatcaacaccat--
>_R_gnl|SRA|DRR000626.2858185.1 HWUSI-EAS1632R_0006_FC61F2Y:1:87:3526:2946..
caaactgcgccaacaaatcagtagcttacagaacgcaaacagtaggaccata---gtggg
-------------ggattctatcaacaccat--
>_R_gnl|SRA|DRR000626.3826337.2 HWUSI-EAS1632R_0006_FC61F2Y:1:114:7950:5689..
caaactgcgccaacaaatcagtagcttacagaacgcaaacagtaggaccata---gtggg
-------------ggattctatcaacaccat--
>_R_gnl|SRA|DRR000626.3243908.1 HWUSI-EAS1632R_0006_FC61F2Y:1:98:4081:17716..
caaactgcgccaacaaatcagtagcttacagaacgcaaacagtaggaccata---gtggg
-------------ggattctatcaacaccat--
>_R_gnl|SRA|DRR000626.7862427.1 HWUSI-EAS1632R_0006_FC61F2Y:2:109:4049:11266..
caaactgcgccaacaaatcagtagcttacagaacgcagacagtaggaccata---gtggg
-------------ggattctatcaacaccat--
>_R_gnl|SRA|DRR000626.8349518.2 HWUSI-EAS1632R_0006_FC61F2Y:3:2:3490:10807..
caaactgcgccaacaaatcagtagcttacagaactcaaacagtaggaccata---gtggg
-------------ggattctatcaacaccat--
>gnl|SRA|DRR000626.5246410.2 HWUSI-EAS1632R_0006_FC61F2Y:2:32:16536:16590..
caaactgcgccaacaaatcagtagcttacagaacgcaaacagtaggaccata---gtggg
-------------ggattctatcaacac-----
>gnl|SRA|DRR000626.631717.2 HWUSI-EAS1632R_0006_FC61F2Y:1:18:5720:8903..
caaactgcgccaacaaatcagtagcttacagaacgcaaacagtaggaccata---gtggg
-------------ggattctatcaacacca---
>gnl|SRA|DRR000626.9722855.1 HWUSI-EAS1632R_0006_FC61F2Y:3:39:6916:15767..
caaactgcgccaacaaatcagtagcttacagaacgcaaacagtaggaccata---gtggg
-------------ggattctatcaa--------
>gnl|SRA|DRR000626.6439939.2 HWUSI-EAS1632R_0006_FC61F2Y:2:68:7937:13517..
caaactgcgccaacaaatcagtagcttacagaacgcaaacagtaggaccata---gtggg
-------------ggattctatcaa--------
>gnl|SRA|DRR000626.11353526.1 HWUSI-EAS1632R_0006_FC61F2Y:3:87:10435:16178..
caaactgcgccaacaaatcagtagcttacagaacgcaaacagtaggaccata---gtggg
-------------ggattctatca---------
>_R_gnl|SRA|DRR000626.5924976.1 HWUSI-EAS1632R_0006_FC61F2Y:2:52:16622:2070..
caaactgcgccaacaaatcagtagcttacagaacgcaaacagtaggaccata---gtggg
-------------ggattctat-----------

>gnl|SRA|DRR000626.10040134.2 HWUSI-EAS1632R_0006_FC61F2Y:3:48:16260:16600..
caaactgcgccaacaaatcagtagcttacagaacgcaaacagtaggaccata---gtggg
-------------ggattcta------------
>_R_gnl|SRA|DRR000626.2028444.2 HWUSI-EAS1632R_0006_FC61F2Y:1:60:15670:17801..
caaactgcgccaacaaatcagtagcttacagaacgcaaacagtaggaccata---gtggg
-------------ggattcta------------
>_R_gnl|SRA|DRR000626.9670603.1 HWUSI-EAS1632R_0006_FC61F2Y:3:37:16943:14974..
caaactgcgccaacaaatcagtagcttacagaacgcaaacagtaggaccata---gtggg
-------------gg------------------
>_R_gnl|SRA|DRR000626.3156625.2 HWUSI-EAS1632R_0006_FC61F2Y:1:95:13747:16004..
caaactgcgccaacaaatcagtagcttacagaacgcaaacagtaggaccata---gtggg
-------------gg------------------
>_R_gnl|SRA|DRR000626.8237533.1 HWUSI-EAS1632R_0006_FC61F2Y:2:119:5361:17301..
caaactgcgccaacaaatcagtagcttacagaacgcaaacagtaggaccata---gtggg
-------------g-------------------
>_R_gnl|SRA|DRR000626.7014999.1 HWUSI-EAS1632R_0006_FC61F2Y:2:85:12775:11210..
caaactgcgccaacaaatcagtagcttacagaacgcaaacagtaggaccata---gtggg

>_R_gnl|SRA|DRR000626.3943094.1 HWUSI-EAS1632R_0006_FC61F2Y:1:117:11088:9655..
caaactgcgccaacaaatcagtagcttacagaacgcaaacagtaggaccata---gtggg

>_R_gnl|SRA|DRR000626.10640388.1 HWUSI-EAS1632R_0006_FC61F2Y:3:66:9396:19766..
caaactgcgccaacaaatcagtagcttacagaacgcaaacagtaggaccata---gtgg-

>_R_gnl|SRA|DRR000626.7190600.1 HWUSI-EAS1632R_0006_FC61F2Y:2:90:12016:1836..
caaactgcgccaacaaatcagtagcttacagaacgcaaacagtaggaccata---gtgg-

>_R_gnl|SRA|DRR000626.8715756.1 HWUSI-EAS1632R_0006_FC61F2Y:3:11:16305:7842..
caaactgcgccaacaaatcagtagcttacagaacgcaaacagtaggaccata---gtg--

>_R_gnl|SRA|DRR000626.3608895.2 HWUSI-EAS1632R_0006_FC61F2Y:1:108:8497:5779..
caaactgcgccaacaaatcagtagcttacagaacgcaaacagtaggaccata---gtg--

>_R_gnl|SRA|DRR000626.4289202.1 HWUSI-EAS1632R_0006_FC61F2Y:2:6:14167:19919..
caaactgcgccaacaaatcagtagcttacagaacgcaaacagtaggaccata---g----

>_R_gnl|SRA|DRR000626.2160202.1 HWUSI-EAS1632R_0006_FC61F2Y:1:64:17480:4622..
caaactgcgccaacaaatcagtagcttacagaacgcaaacagtaggaccata---g----

>gnl|SRA|DRR000626.3069503.2 HWUSI-EAS1632R_0006_FC61F2Y:1:93:5026:7158..
caaactgcgccaacaaatcagtagcttacagaacgcaaacagtaggaccata---g----

>_R_gnl|SRA|DRR000626.6759932.1 HWUSI-EAS1632R_0006_FC61F2Y:2:78:5933:14190..
caaactgcgccaacaaatcagtagcttacagaacgcaaacagtaggaccata--------

>_R_gnl|SRA|DRR000626.2186607.1 HWUSI-EAS1632R_0006_FC61F2Y:1:65:13911:6652..
caaactgcgccaacaaatcagtagcttacagaacgcaaacagtaggaccata--------

>_R_gnl|SRA|DRR000626.4641817.1 HWUSI-EAS1632R_0006_FC61F2Y:2:16:5756:14628..
caaactgcgccaacaaatcagtagcttacagaacgcaaacagtaggaccata--------

>gnl|SRA|DRR000626.4641817.2 HWUSI-EAS1632R_0006_FC61F2Y:2:16:5756:14628..
caaactgcgccaacaaatcagtagcttacagaacgcaaacagtaggaccataagatcgga
-------------agagcgt-------------
>gnl|SRA|DRR000626.3296004.2 HWUSI-EAS1632R_0006_FC61F2Y:1:99:12615:19248..
caaactgcgccaacaaatcagtagcttacagaacgcaaacagtaggaccat---------

>_R_gnl|SRA|DRR000626.10957830.1 HWUSI-EAS1632R_0006_FC61F2Y:3:76:3721:16409..
caaactgcgccaacaaatcagtagcttacagaacgcaaacagtaggacca----------

>_R_gnl|SRA|DRR000626.994128.2 HWUSI-EAS1632R_0006_FC61F2Y:1:28:8807:20397..
caaactgcgccaacaaatcagtagcttacagaacgcaaacagtaggacca----------

>_R_gnl|SRA|DRR000626.850456.2 HWUSI-EAS1632R_0006_FC61F2Y:1:24:7825:2484..
caaactgcgccaacaaatcagtagcttacagaacgtaaacagtaggacca----------

>_R_gnl|SRA|DRR000626.6425075.1 HWUSI-EAS1632R_0006_FC61F2Y:2:67:18317:8624..
caaactgcgccaacaaatcagtagcttacagaacgcaaacagtaggac------------

>_R_gnl|SRA|DRR000626.5462050.1 HWUSI-EAS1632R_0006_FC61F2Y:2:38:17751:11817..
caaactgcgccaacaaatcagtagcttacagaacgcaaacagtaggac------------

>_R_gnl|SRA|DRR000626.1211616.1 HWUSI-EAS1632R_0006_FC61F2Y:1:34:14675:8646..
caaactgcgccaacaaatcagtggcttacagaacgcaaacagtaggac------------

>_R_gnl|SRA|DRR000626.2178797.2 HWUSI-EAS1632R_0006_FC61F2Y:1:65:9515:7211..
caaactgcgccaacaaatcagtagcttacagaacgcaaacagtaggactcct---g----

>gnl|SRA|DRR000626.6880380.1 HWUSI-EAS1632R_0006_FC61F2Y:2:81:15523:3739..
caaactgcgccaacaaatcagtagcttacagaacgcaaacagtaggacc-----------

>_R_gnl|SRA|DRR000626.4210080.1 HWUSI-EAS1632R_0006_FC61F2Y:2:4:12143:15607..
caaactgcgccaacaaatcagtagcttacagaacgcaaacagtaggacc-----------

>gnl|SRA|DRR000626.633482.2 HWUSI-EAS1632R_0006_FC61F2Y:1:18:6615:10134..
caaactgcgccaacaaatcagtagcttacagaacgcaaacagtaggacc-----------

>gnl|SRA|DRR000626.768619.2 HWUSI-EAS1632R_0006_FC61F2Y:1:22:2168:1742..
caaactgcgccaacaaatcagtagcttacagaacgcaaacagtaggcac-----------

>_R_gnl|SRA|DRR000626.1594363.2 HWUSI-EAS1632R_0006_FC61F2Y:1:47:7526:19302..
caaactgcgccaacaaatcagtagcttacagaacgcaaacagtagga-------------

>gnl|SRA|DRR000626.3083027.2 HWUSI-EAS1632R_0006_FC61F2Y:1:93:11972:19845..
caaactgcgccaacaaatcagtagcttacagaacgcaaacagtagg--------------

>gnl|SRA|DRR000626.1200534.2 HWUSI-EAS1632R_0006_FC61F2Y:1:34:8857:10560..
caaactgcgccaacaaatcagtagcttacagaacgcaaacagtagg--------------

>_R_gnl|SRA|DRR000626.6738810.1 HWUSI-EAS1632R_0006_FC61F2Y:2:77:12582:20331..
caaactgcgccaacaaatcagtagcttacagaacgcaaacagtaggctcatggtgttgat
-------------agaatccc------------

>_R_gnl|SRA|DRR000626.1200534.1 HWUSI-EAS1632R_0006_FC61F2Y:1:34:8857:10560..
caaactgcgccaacaaatcagtagcttacagaacgcaaacagtagtactata---gtggg
-------------ggattctatcaaca------
>gnl|SRA|DRR000626.2446995.2 HWUSI-EAS1632R_0006_FC61F2Y:1:73:12560:7844..
caaactgcgccaacaaatcagtagcttacagaacgcaaacagtag--------------

>gnl|SRA|DRR000626.2298860.1 HWUSI-EAS1632R_0006_FC61F2Y:1:69:3667:4364..
caaactgcgccaacaaatcagtagcttacagaacgcaaacagtag--------------

>gnl|SRA|DRR000626.11023550.2 HWUSI-EAS1632R_0006_FC61F2Y:3:78:4607:17311..
caaactgcgccaacaaatcagtagcttacagaacgcaaacagta---------------

>_R_gnl|SRA|DRR000626.7931508.1 HWUSI-EAS1632R_0006_FC61F2Y:2:111:1580:14081..
caaactgcgccaacaaatcagtagcttacagaacgcaaacagta---------------

>gnl|SRA|DRR000626.2156005.2 HWUSI-EAS1632R_0006_FC61F2Y:1:64:15013:20110..
caaactgcgccaacaaatcagtagcttacagaacgcaaacagta---------------

>gnl|SRA|DRR000626.1594363.1 HWUSI-EAS1632R_0006_FC61F2Y:1:47:7526:19302..
caaactgcgccaacaaatcagtagcttacagaacgcaaacagta---------------

>gnl|SRA|DRR000626.8907018.1 HWUSI-EAS1632R_0006_FC61F2Y:3:16:18966:7966..
caaactgcgccaacaaatcagtagcttacagaacgcaaacagt----------------

>gnl|SRA|DRR000626.8190734.1 HWUSI-EAS1632R_0006_FC61F2Y:2:117:19766:6264..
caaactgcgccaacaaatcagtagcttacagaacgcaaacagt----------------

>gnl|SRA|DRR000626.3416980.1 HWUSI-EAS1632R_0006_FC61F2Y:1:103:1845:3681..
caaactgcgccaacaaatcagtagcttacagaacgcaaacagt----------------

>gnl|SRA|DRR000626.2402579.2 HWUSI-EAS1632R_0006_FC61F2Y:1:72:6414:18741..
caaactgcgccaacaaatcagtagcttacagaacgcaaacagt----------------

>gnl|SRA|DRR000626.452493.2 HWUSI-EAS1632R_0006_FC61F2Y:1:13:6039:14386..
caaactgcgccaacaaatcagtagcttacagaacgcaaacagt----------------

>gnl|SRA|DRR000626.85939.2 HWUSI-EAS1632R_0006_FC61F2Y:1:3:6234:7662..
caaactgcgccaacaaatcagtagcttacagaacgcaaacagt----------------

>_R_gnl|SRA|DRR000626.63870.1 HWUSI-EAS1632R_0006_FC61F2Y:1:2:13701:17809..
caaactgcgccaacaaatcagtagcttacagaacgcaaacaag----------------

>_R_gnl|SRA|DRR000626.11434295.2 HWUSI-EAS1632R_0006_FC61F2Y:3:89:15234:10859..
caaactgcgccaacaaatcagtagcttacagaacgcaaacag-----------------

>gnl|SRA|DRR000626.11631874.2 HWUSI-EAS1632R_0006_FC61F2Y:3:95:5639:1259..
caaactgcgccaacaaatcagtagcttacagaacgcaaaaag-----------------

>_R_gnl|SRA|DRR000626.4579102.2 HWUSI-EAS1632R_0006_FC61F2Y:2:14:11156:8454..
caaactgcgccaacaaatcagtagcttacagaacgcaaaca------------------

>_R_gnl|SRA|DRR000626.1412202.1 HWUSI-EAS1632R_0006_FC61F2Y:1:41:14823:3552..
caaactgcgccaacaaatcagtagcttacagaacgcaaacag---gaccata---gtggg
-------------ggattctttcaacaccat--
>_R_gnl|SRA|DRR000626.9035560.1 HWUSI-EAS1632R_0006_FC61F2Y:3:20:9721:9099..
caaactgcgccaacaaatcagtagcttacagaacgcaaacag---gagcata---gtggg
-------------ggattctatcaacaccat--
>gnl|SRA|DRR000626.560348.1 HWUSI-EAS1632R_0006_FC61F2Y:1:16:5943:16606..
caaactgcgccaacaaatcagtagcttacagaacgcgaacag---gaccata---gtggg
-------------ggattctatcaacatcat--
>_R_gnl|SRA|DRR000626.12159895.1 HWUSI-EAS1632R_0006_FC61F2Y:3:109:13926:5636..
caaactgcgccaacaaatcagtagcttacagaacgcaaacag---gaccata---gtggg
-------------ggattcta------------
>_R_gnl|SRA|DRR000626.1637897.2 HWUSI-EAS1632R_0006_FC61F2Y:1:48:13250:15873..
caaactgcgccaacaaatcagtagcttacagaacgcaaacag---gaccata---gtggg
-------------ggattcta------------
>gnl|SRA|DRR000626.7943164.2 HWUSI-EAS1632R_0006_FC61F2Y:2:111:7373:20763..
caaactgcgccaacaaatcagtagcttacagaacgcaaacag---gaccata---gtggg
-------------ggattcta------------
>gnl|SRA|DRR000626.11941519.1 HWUSI-EAS1632R_0006_FC61F2Y:3:103:15097:1031..
caaactgcgccaacaaatcagtagcttacagaacgcaaacag---gaccata---gtggg
-------------ggattctat-----------
>_R_gnl|SRA|DRR000626.9979192.1 HWUSI-EAS1632R_0006_FC61F2Y:3:46:18659:14468..
caaactgcgccaacaaatcagtagcttacagaacgcaaacag---gaccata---gtggg
-------------ggattctatc----------
>gnl|SRA|DRR000626.11185227.2 HWUSI-EAS1632R_0006_FC61F2Y:3:82:15271:3661..
caaactgcgccaacaaatcagtagcttacagaacgcaaacag---gaccata---gtggg
-------------ggattctatcaaca------
>_R_gnl|SRA|DRR000626.11579531.1 HWUSI-EAS1632R_0006_FC61F2Y:3:93:15793:5959..
caaactgcgccaacaaatcagtagcttacagaacgcaaacag---gaccata---gtggg
-------------ggattctatcaaca------
>gnl|SRA|DRR000626.11579531.2 HWUSI-EAS1632R_0006_FC61F2Y:3:93:15793:5959..
caaactgcgccaacaaatcagtagcttacagaacgcaaacag---gaccata---gtggg
-------------ggattctatcaacaagatcg
>gnl|SRA|DRR000626.8412968.2 HWUSI-EAS1632R_0006_FC61F2Y:3:3:15369:12564..
caaactgcgccaacaaatcagtagcttacagaacgcaaacag---gaccata---gtggg
-------------ggattctatcaac-------
>_R_gnl|SRA|DRR000626.7978482.1 HWUSI-EAS1632R_0006_FC61F2Y:2:112:6604:12464..
caaactgcgccaacaaatcagtagcttacagaacgcaaacag---gaccata---gtggg
-------------ggattctatcaac-------
>_R_gnl|SRA|DRR000626.10663088.2 HWUSI-EAS1632R_0006_FC61F2Y:3:67:3468:9219..
caaactgcgccaacaaatcagtagcttacagaacgcaaacag---gaccata---gtggg
-------------ggattctatcaacac-----
>gnl|SRA|DRR000626.1772764.1 HWUSI-EAS1632R_0006_FC61F2Y:1:52:15348:7939..
caaactgcgccaacaaatcagtagcttacagaacgcaaacag---gaccata---gtggg
-------------ggattctatcaacac-----
>_R_gnl|SRA|DRR000626.1648742.2 HWUSI-EAS1632R_0006_FC61F2Y:1:48:19313:5655..
caaactgcgccaacaaatcagtagcttacagaacgcaaacag---gaccata---gtggg
-------------ggattctatcaacacc----
>gnl|SRA|DRR000626.2097183.2 HWUSI-EAS1632R_0006_FC61F2Y:1:62:18334:8869..
caaactgcgccaacaaatcagtagcttacagaacgcaaacag---gaccata---gtggg
-------------ggcttctatcaacacca---

>gnl|SRA|DRR000626.3508004.1 HWUSI-EAS1632R_0006_FC61F2Y:1:105:11795:19520..
caaactgcgccaacaaatcagtagcttacagaacgcaaacag---gaccata---gtggg
-------------ggattctatcaacacca---
>_R_gnl|SRA|DRR000626.9999341.2 HWUSI-EAS1632R_0006_FC61F2Y:3:47:12053:4029..
caaactgcgccaacaaatcagtagcttacagaacgcaaacag---gaccata---gtggg
-------------ggattct-------------
>_R_gnl|SRA|DRR000626.8258085.1 HWUSI-EAS1632R_0006_FC61F2Y:2:119:15053:19637..
caaactgcgccaacaaatcagtagcttacagaacgcaaacag---gaccata---gtggg
-------------ggattc--------------
>_R_gnl|SRA|DRR000626.6744678.2 HWUSI-EAS1632R_0006_FC61F2Y:2:77:15880:4365..
caaactgcgccaacaaatcagtagcttacagaacgcaaacag---gaccata---gtggg
-------------ggattc--------------
>gnl|SRA|DRR000626.4781662.1 HWUSI-EAS1632R_0006_FC61F2Y:2:20:1742:17954..
caaactgcgccaacaaatcagtagcttacagaacgcaaacag---gaccata---gtggg
-------------ggatt---------------
>_R_gnl|SRA|DRR000626.6915475.1 HWUSI-EAS1632R_0006_FC61F2Y:2:82:15572:10798..
caaactgcgccaacaaatcagtagcttacagaacgcaaacag---gaccata---gtggg
-------------ggat----------------
>_R_gnl|SRA|DRR000626.6833449.1 HWUSI-EAS1632R_0006_FC61F2Y:2:80:9111:19977..
caaactgcgccaacaaatcagtagcttacagaacgcaaacag---gaccata---gtggg
-------------ggat----------------
>_R_gnl|SRA|DRR000626.11654089.1 HWUSI-EAS1632R_0006_FC61F2Y:3:95:17003:14352..
caaactgcgccaacaaatcagtagcttacagaacgcaaacag---gaccata---gtggg
-------------ggat----------------
>_R_gnl|SRA|DRR000626.2084078.2 HWUSI-EAS1632R_0006_FC61F2Y:1:62:10681:16723..
caaactgcgccaacaaatcagtagcttacagaacgcaaacag---gaccata---gtggg
-------------gg------------------
>_R_gnl|SRA|DRR000626.3881606.1 HWUSI-EAS1632R_0006_FC61F2Y:1:115:17285:9523..
caaactgcgccaacaaatcagtagcttacagaacgcaaacag---gaccata---gtggg
-------------g-------------------
>_R_gnl|SRA|DRR000626.1169327.2 HWUSI-EAS1632R_0006_FC61F2Y:1:33:10643:7513..
caaactgcgccaacaaatcagtagcttacagaacgcaaacag---gaccaga---gtggg
-------------g-------------------
>_R_gnl|SRA|DRR000626.9305634.2 HWUSI-EAS1632R_0006_FC61F2Y:3:27:15847:16434..
caaactgcgccaacaaatcagtagcttacagaacgcaaacag---gaccata---gtggg

>_R_gnl|SRA|DRR000626.5234202.1 HWUSI-EAS1632R_0006_FC61F2Y:2:32:10241:19702..
caaactgcgccaacaaatcagtagcttacagaacgcaaacag---gaccata---gtggg

>_R_gnl|SRA|DRR000626.1503453.1 HWUSI-EAS1632R_0006_FC61F2Y:1:44:12642:11088..
caaactgcgccaacaaatcagtagcttacagaacgcaaacag---gaccata---gtggg

>_R_gnl|SRA|DRR000626.6635277.1 HWUSI-EAS1632R_0006_FC61F2Y:2:74:4295:9570..
caaactgcgccaacaaatcagtagcttacagaacgcaaacag---gaccata---gtggg

>gnl|SRA|DRR000626.11654089.2 HWUSI-EAS1632R_0006_FC61F2Y:3:95:17003:14352..
caaactgcgccaacaaatcagtagcttacagaacgcaaacag---gaccata---gtggg
gg---------atagatcggaagagcgtcgtgt
>gnl|SRA|DRR000626.6635277.2 HWUSI-EAS1632R_0006_FC61F2Y:2:74:4295:9570..
caaactgcgccaacaaatcagtagcttacagaacgcaaacag---gaccata---gtggg
-------------agatcggaagagcgtcgtg-

```
>_R_gnl|SRA|DRR000626.9964599.1 HWUSI-EAS1632R_0006_FC61F2Y:3:46:8162:16518..
caaactgcgccaacaaatcagtagcttacagaacgcaaacag---gaccata---gtg--
---------------------------------
>_R_gnl|SRA|DRR000626.5966441.1 HWUSI-EAS1632R_0006_FC61F2Y:2:54:2065:14017..
caaactgcgccaacaaatcagtagcttacagaacgcaaacag---gaccata---gtgg-
---------------------------------
>_R_gnl|SRA|DRR000626.5802208.2 HWUSI-EAS1632R_0006_FC61F2Y:2:49:5743:3133..
caaactgcgccaacaaatcagtagcttacagaacgcaaacag---gaccata---gtgg-
---------------------------------
>_R_gnl|SRA|DRR000626.3224767.2 HWUSI-EAS1632R_0006_FC61F2Y:1:97:12433:1877..
caaactgcgccaacaaatcagtagcttacagaacgcaaacag---gaccata---gtgg-
---------------------------------
>_R_gnl|SRA|DRR000626.2657678.1 HWUSI-EAS1632R_0006_FC61F2Y:1:79:19382:20628..
caagctgcgccaacaaatcagtagcttacagaacgcaaacag---taccata---gtgg-
---------------------------------
>_R_gnl|SRA|DRR000626.10039372.1 HWUSI-EAS1632R_0006_FC61F2Y:3:48:15837:4377..
caaactgcgccaacaaatcagtagcttacagaacgcaaacag---gaccata---g----
---------------------------------
>_R_gnl|SRA|DRR000626.2187062.1 HWUSI-EAS1632R_0006_FC61F2Y:1:65:14160:2021..
caaactgcgccaacaaatcagtagcttacagaacgcaaacag---gaccata---g----
---------------------------------
>gnl|SRA|DRR000626.9931385.2 HWUSI-EAS1632R_0006_FC61F2Y:3:45:4571:17869..
caaactgcgccaacaaatcagtagcttacagaacgcaaacag---gaccata--------
---------------------------------
>gnl|SRA|DRR000626.3150051.1 HWUSI-EAS1632R_0006_FC61F2Y:1:95:10383:19966..
caaactgcgccaacaaatcagtagcttacagaacgcaaacag---gaccata--------
---------------------------------
>_R_gnl|SRA|DRR000626.2584965.2 HWUSI-EAS1632R_0006_FC61F2Y:1:77:15195:1037..
caaactgcgccaacaaatcagtagcttacagaacgcaaacag---gaccata--------
---------------------------------
>gnl|SRA|DRR000626.4755912.2 HWUSI-EAS1632R_0006_FC61F2Y:2:19:7296:11640..
caaactgcgccaacaaatcagtagcttacagaacgcaaacag---gaccata---gtggt
agtgctgggcattgacttctgccactgtgcca-
>gnl|SRA|DRR000626.3995292.1 HWUSI-EAS1632R_0006_FC61F2Y:1:118:18683:17675..
caaactgcgccaacaaatcagtagcttacagaacgcaaacag---gaccata---gtggt
agtgctgggcattgacttctgcaactgtgcca-
>_R_gnl|SRA|DRR000626.3995292.2 HWUSI-EAS1632R_0006_FC61F2Y:1:118:18683:17675..
--aactgcgccaacaaatcagtagcttacagaacgcaaacag---gaccata---gtggt
agtgctgggcattgacttctgcaactgtgcca-
>_R_gnl|SRA|DRR000626.9886556.2 HWUSI-EAS1632R_0006_FC61F2Y:3:43:17411:19834..
caaactgcgccaacaaatcagtagcttacagaacgcaaacag---gacc-----------
---------------------------------
>gnl|SRA|DRR000626.10646787.2 HWUSI-EAS1632R_0006_FC61F2Y:3:66:12927:16144..
caaactgcgccaacaaatcagtagcttacagaacgcaaacag---ga-------------
---------------------------------
>_R_gnl|SRA|DRR000626.12417240.2 HWUSI-EAS1632R_0006_FC61F2Y:3:116:11877:8995..
caaactgcgccaacaaatcagtagcttacagaacgcaaacag---g--------------
---------------------------------
>gnl|SRA|DRR000626.11344496.2 HWUSI-EAS1632R_0006_FC61F2Y:3:87:5833:11679..
caaactgcgccaacaaatcagtagcttacagaacgcaaacag---g--------------
---------------------------------
```

>gnl|SRA|DRR000626.7354646.2 HWUSI-EAS1632R_0006_FC61F2Y:2:95:4595:5100..
caaactgcgccaacaaatcagtagcttacagaacgcaaacag---g--------------

>gnl|SRA|DRR000626.7318204.1 HWUSI-EAS1632R_0006_FC61F2Y:2:94:4288:6778..
caaactgcgccaacaaatcagtagcttacagaacgcaaacag---g--------------

>_R_gnl|SRA|DRR000626.7995589.2 HWUSI-EAS1632R_0006_FC61F2Y:2:112:15136:3696..
caaactgcgccaacaaatcagtagcttacagaacgcaaacag---gac------------

>_R_gnl|SRA|DRR000626.7188566.2 HWUSI-EAS1632R_0006_FC61F2Y:2:90:10931:11288..
caaactgcgccaacaaatcagtagcttacagaacgcaaacag---gac------------

>_R_gnl|SRA|DRR000626.3262660.1 HWUSI-EAS1632R_0006_FC61F2Y:1:98:13774:9782..
caaactgcgccaacaaatcagtagcttacagaacgcaaacag---gac------------

>_R_gnl|SRA|DRR000626.1218706.2 HWUSI-EAS1632R_0006_FC61F2Y:1:34:18465:18233..
caaactgcgccaacaaatcagtagcttacagaacgcaaacag---gac------------

>_R_gnl|SRA|DRR000626.7247153.1 HWUSI-EAS1632R_0006_FC61F2Y:2:92:4548:20385..
caaactgcgccaacaaatcagtagcttacagaacgcaaacag---gac------------

>_R_gnl|SRA|DRR000626.888442.2 HWUSI-EAS1632R_0006_FC61F2Y:1:25:9151:7191..
caaactgcgccaacaaatcagtagcttacagaacgcaaacag---gac------------

>_R_gnl|SRA|DRR000626.562912.2 HWUSI-EAS1632R_0006_FC61F2Y:1:16:7311:2539..
caaacttcgccaacaaatcagtagcttgcagaacgcaaacag---gac------------

>_R_gnl|SRA|DRR000626.8703550.2 HWUSI-EAS1632R_0006_FC61F2Y:3:11:10363:2263..
caaactgcgccatcaaatcagtagctaacagaacgcaaacag---gac------------

>_R_gnl|SRA|DRR000626.11345408.1 HWUSI-EAS1632R_0006_FC61F2Y:3:87:6298:10512..
caaactgcgccaacaaatcagtagcttacagaacgcaaac--------------------

>_R_gnl|SRA|DRR000626.6649144.1 HWUSI-EAS1632R_0006_FC61F2Y:2:74:13053:18185..
caaactgcgccaacaaatcagtagcttacagaacgcaaac--------------------

>_R_gnl|SRA|DRR000626.6469110.1 HWUSI-EAS1632R_0006_FC61F2Y:2:69:5539:14884..
caaactgcgccaacaaatcagtagcttacagaacgcaaac--------------------

>_R_gnl|SRA|DRR000626.6174371.1 HWUSI-EAS1632R_0006_FC61F2Y:2:60:7019:7207..
caaactgcgccaacaaatcagtagcttacagaacgcaaac--------------------

>_R_gnl|SRA|DRR000626.3353923.1 HWUSI-EAS1632R_0006_FC61F2Y:1:101:5823:1627..
caaactgcgccaacaaatcagtagcttacagaacgcaaac--------------------

>_R_gnl|SRA|DRR000626.2795089.2 HWUSI-EAS1632R_0006_FC61F2Y:1:85:6651:2378..
caaactgcgccaacaaatcagtagcttacagaacgcaaac--------------------

>_R_gnl|SRA|DRR000626.637324.2 HWUSI-EAS1632R_0006_FC61F2Y:1:18:8572:9854..
caaactgcgccaacaaatcagtagcttacagaacgcaaac--------------------

>_R_gnl|SRA|DRR000626.1630893.2 HWUSI-EAS1632R_0006_FC61F2Y:1:48:9411:14410..
caaactgcgccaacaaatcagtagcttacagaacgcaaac--------------------

>_R_gnl|SRA|DRR000626.4853426.2 HWUSI-EAS1632R_0006_FC61F2Y:2:22:1163:12240..
caaactgcgccaacaaatcagtagcttacagaacgcaaac--------------------

>_R_gnl|SRA|DRR000626.3488426.2 HWUSI-EAS1632R_0006_FC61F2Y:1:105:1775:9312..
caaactgcgccaacaaatcagtagcttacagaacgcaaac--------------------

>_R_gnl|SRA|DRR000626.10745292.1 HWUSI-EAS1632R_0006_FC61F2Y:3:69:10816:6563..
caaactgcgccaacaaatcagtagcttacagaacgcaaa---------------------

>_R_gnl|SRA|DRR000626.9509319.2 HWUSI-EAS1632R_0006_FC61F2Y:3:33:8203:7696..
caaactgcgccaacaaatcagtagcttacagaacgcaaa---------------------

>_R_gnl|SRA|DRR000626.7460796.1 HWUSI-EAS1632R_0006_FC61F2Y:2:98:4032:12277..
caaactgcgccaacaaatcagtagcttacagaacgcaaa---------------------

>_R_gnl|SRA|DRR000626.3298787.1 HWUSI-EAS1632R_0006_FC61F2Y:1:99:14077:15238..
caaactgcgccaacaaatcagtagcttacagaacgcaaa---------------------

>_R_gnl|SRA|DRR000626.2618480.2 HWUSI-EAS1632R_0006_FC61F2Y:1:78:15092:9222..
caaactgcgccaacaaatcagtagcttacagaacgcaaa---------------------

>_R_gnl|SRA|DRR000626.504245.2 HWUSI-EAS1632R_0006_FC61F2Y:1:14:14005:15571..
caaactgcgccaacaaatcagtagcttacagaacgcaaa---------------------

>_R_gnl|SRA|DRR000626.5216819.2 HWUSI-EAS1632R_0006_FC61F2Y:2:32:1137:1437..
caaactgcgccaacaaatcagtagcttacagaacgcaaa---------------------

>_R_gnl|SRA|DRR000626.5287032.1 HWUSI-EAS1632R_0006_FC61F2Y:2:33:19264:1817..
caaactgcgccaacaaatcagtagcttacagaacgcaa----------------------

>_R_gnl|SRA|DRR000626.3139235.1 HWUSI-EAS1632R_0006_FC61F2Y:1:95:4722:10238..
caaactgcgccaacaaatcagtagcttacagaacgcaa----------------------

>_R_gnl|SRA|DRR000626.788191.2 HWUSI-EAS1632R_0006_FC61F2Y:1:22:12305:14320..
caaactgcgccaacaaatcagtagcttacagaacgcaa----------------------

>gnl|SRA|DRR000626.788191.1 HWUSI-EAS1632R_0006_FC61F2Y:1:22:12305:14320..
caaactgcgccaacaaatcagtagcttacagaacgcaa----------------------

>_R_gnl|SRA|DRR000626.490728.1 HWUSI-EAS1632R_0006_FC61F2Y:1:14:7159:2384..
caaactgcgccaacaaatcagtagcttacagaacgcaa----------------------

>gnl|SRA|DRR000626.1339521.1 HWUSI-EAS1632R_0006_FC61F2Y:1:38:10715:16611..
caaactgcgccaacaaatcagtagcttacagaacgcaa----------------------

>_R_gnl|SRA|DRR000626.227542.2 HWUSI-EAS1632R_0006_FC61F2Y:1:7:3106:1635..
caaactgcgccagcaaatcagtagcttgcagaacgcaaacag---gctcatggt-gttga
-----------tagaatccc-------------

>gnl|SRA|DRR000626.12246293.1 HWUSI-EAS1632R_0006_FC61F2Y:3:112:1668:9700..
-------------caaatcagtagcttacagaacgcaaacag---gaccata---gtggg
-------------ggattctatagatcggaa--
>_R_gnl|SRA|DRR000626.12246293.2 HWUSI-EAS1632R_0006_FC61F2Y:3:112:1668:9700..
-------------caaatcagtagcttacagaacgcaaacag---gaccata---gtggg
-------------ggattctat-----------

SRX100746
>control ..
tagcttacagaacgcaaacagtaggaccatagtgggggattc
>gnl|SRA|SRR352190.13545071.1:1-35 HWI-EAS216_0001:8:56:18551:14502.
-------cagaacgcaaacagtaggaccatagtgggggattc
>gnl|SRA|SRR352190.9714329.1:1-35 HWI-EAS216_0001:8:40:18766:2470.
-------cagaacgcaaacagtaggaccatagtgggggattc
>gnl|SRA|SRR352190.23393514.1:1-34 HWI-EAS216_0001:8:99:14671:13892.
--------agaacgcaaacagtaggaccatagtgggggattc
>_R_gnl|SRA|SRR352190.6694164.1:2-35 HWI-EAS216_0001:8:28:8248:18897.
--------agaacgcaaacagtaggaccatagtgggggattc
>gnl|SRA|SRR352190.21355397.1:1-33 HWI-EAS216_0001:8:90:18564:5310.
---------gaacgcaaacagtaggaccatagtgggggattc
>_R_gnl|SRA|SRR352190.6772159.1:3-35 HWI-EAS216_0001:8:28:14083:4596.
---------gaacgcaaacagtaggaccatagtgggggattc
>gnl|SRA|SRR352190.6213364.1:1-32 HWI-EAS216_0001:8:26:8810:20401.
----------aacgcaaacagtaggaccatagtgggggattc
>_R_gnl|SRA|SRR352190.5400558.1:4-35 HWI-EAS216_0001:8:23:3101:6227.
----------aacgcaaacagtaggaccatagtgggggattc
>gnl|SRA|SRR352190.26005983.1:1-30 HWI-EAS216_0001:8:111:2599:11489.
------------cgcaaacagtaggaccatagtgggggattc
>gnl|SRA|SRR352190.24053576.1:1-30 HWI-EAS216_0001:8:102:11621:15513.
------------cgcaaacagtaggaccatagtgggggattc
>gnl|SRA|SRR352190.19456265.1:1-30 HWI-EAS216_0001:8:82:12999:7596.
------------cgcaaacagtaggaccatagtgggggattc
>gnl|SRA|SRR352190.14273248.1:1-30 HWI-EAS216_0001:8:59:19736:13482.
------------cgcaaacagtaggaccatagtgggggattc
>gnl|SRA|SRR352190.13728946.1:1-30 HWI-EAS216_0001:8:57:14368:16206.
------------cgcaaacagtaggaccatagtgggggattc
>gnl|SRA|SRR352190.8992227.1:1-30 HWI-EAS216_0001:8:37:19516:19517.
------------cgcaaacagtaggaccatagtgggggattc
>gnl|SRA|SRR352190.23468965.1:1-28 HWI-EAS216_0001:8:100:2754:14760.
--------------caaacagtaggaccatagtgggggattc
>gnl|SRA|SRR352190.23122753.1:1-28 HWI-EAS216_0001:8:98:11557:5707.
--------------caaacagtaggaccatagtgggggattc
>gnl|SRA|SRR352190.21292869.1:1-28 HWI-EAS216_0001:8:90:13348:2273.
--------------caaacagtaggaccatagtgggggattc
>gnl|SRA|SRR352190.18678097.1:1-28 HWI-EAS216_0001:8:79:6246:17191.
--------------caaacagtaggaccatagtgggggattc
>gnl|SRA|SRR352190.16573947.1:1-28 HWI-EAS216_0001:8:70:2027:16440.
--------------caaacagtaggaccatagtgggggattc

>gnl|SRA|SRR352190.7113642.1:1-28 HWI-EAS216_0001:8:30:3184:17856.
--------------caaacagtaggaccatagtgggggattc
>gnl|SRA|SRR352190.3917106.1:1-28 HWI-EAS216_0001:8:17:2346:21246.
--------------caaacagtaggaccatagtgggggattc
>gnl|SRA|SRR352190.25495062.1:1-29 HWI-EAS216_0001:8:108:16761:8596.
-------------gcaaacagtaggaccatagtgggggattc
>gnl|SRA|SRR352190.19505666.1:1-29 HWI-EAS216_0001:8:82:16997:20893.
-------------gcaaacagtaggaccatagtgggggattc
>gnl|SRA|SRR352190.19084482.1:1-29 HWI-EAS216_0001:8:81:2240:20927.
-------------gcaaacagtaggaccatagtgggggattc
>gnl|SRA|SRR352190.16578748.1:1-29 HWI-EAS216_0001:8:70:2394:7023.
-------------gcaaacagtaggaccatagtgggggattc
>gnl|SRA|SRR352190.13052827.1:1-29 HWI-EAS216_0001:8:54:17303:16075.
-------------gcaaacagtaggaccatagtgggggattc
>gnl|SRA|SRR352190.6120725.1:1-29 HWI-EAS216_0001:8:26:1907:17959.
-------------gcaaggagtagggccatagtgggggattc
>gnl|SRA|SRR352190.20217304.1:1-27 HWI-EAS216_0001:8:85:19056:1316.
---------------aaacagtaggaccatagtgggggattc
>_R_gnl|SRA|SRR352190.15006487.1:9-35 HWI-EAS216_0001:8:63:3797:7337.
---------------aaacagtaggaccatagtgggggattc
>gnl|SRA|SRR352190.12928024.1:1-27 HWI-EAS216_0001:8:54:7663:5676.
---------------aaacagtaggaccatagtgggggattc
>_R_gnl|SRA|SRR352190.10876119.1:10-35 HWI-EAS216_0001:8:45:15305:19391.
----------------aacagtaggaccatagtgggggattc
>_R_gnl|SRA|SRR352190.23356237.1:14-35 HWI-EAS216_0001:8:99:11744:4308.
--------------------gtaggaccatagtgggggattc
>_R_gnl|SRA|SRR352190.22302728.1:14-35 HWI-EAS216_0001:8:95:2577:3601.
--------------------gtaggaccatagtgggggattc
>_R_gnl|SRA|SRR352190.14940283.1:15-35 HWI-EAS216_0001:8:62:16565:1388.
---------------------taggaccatagtgggggattc
>_R_gnl|SRA|SRR352190.2800656.1:15-35 HWI-EAS216_0001:8:12:10178:13230.
---------------------taggaccatagtgggggattc
>gnl|SRA|SRR352190.28221772.1:2-21 HWI-EAS216_0001:8:120:12075:7548.
----------------------aggaccatagtgggggattc
>gnl|SRA|SRR352190.26879612.1:1-20 HWI-EAS216_0001:8:114:15947:6683.
----------------------aggaccatagtgggggattc
>gnl|SRA|SRR352190.21430577.1:6-25 HWI-EAS216_0001:8:91:6139:10363.
----------------------aggaccatagtgggggattc
>_R_gnl|SRA|SRR352190.20802186.1:13-32 HWI-EAS216_0001:8:88:11076:3073.
----------------------aggaccatagtgggggattc
>gnl|SRA|SRR352190.17122791.1:2-21 HWI-EAS216_0001:8:72:9311:17120.
----------------------aggaccatagtgggggattc
>gnl|SRA|SRR352190.15962997.1:2-21 HWI-EAS216_0001:8:67:7759:18559.
----------------------aggaccatagtgggggattc
>_R_gnl|SRA|SRR352190.14945945.1:15-34 HWI-EAS216_0001:8:62:17035:16483.
----------------------aggaccatagtgggggattc
>_R_gnl|SRA|SRR352190.13212908.1:15-34 HWI-EAS216_0001:8:55:11244:17918.
----------------------aggaccatagtgggggattc
>gnl|SRA|SRR352190.11794581.1:2-21 HWI-EAS216_0001:8:49:12349:12060.
----------------------aggaccatagtgggggattc
>_R_gnl|SRA|SRR352190.9675449.1:16-35 HWI-EAS216_0001:8:40:15766:6693.

----------------------aggaccatagtgggggattc
>gnl|SRA|SRR352190.6832637.1:2-21 HWI-EAS216_0001:8:28:18782:9367.
----------------------aggaccatagtgggggattc
>gnl|SRA|SRR352190.3014789.1:2-21 HWI-EAS216_0001:8:13:7943:17712.
----------------------aggaccatagtgggggattc
>gnl|SRA|SRR352190.1936167.1:2-21 HWI-EAS216_0001:8:8:18975:6765.
----------------------aggaccatagtgggggattc
>gnl|SRA|SRR352190.695185.1:6-25 HWI-EAS216_0001:8:3:16850:15960.
----------------------aggaccatagtgggggattc
>gnl|SRA|SRR352190.20468315.1:1-35 HWI-EAS216_0001:8:87:2421:7437.
----ttacagaacgcaaac---aggaccatagtgggggattc
>_R_gnl|SRA|SRR352190.16023794.1:1-35 HWI-EAS216_0001:8:67:12576:12549.
----ttacagaacgcaaac---aggaccatagtgggggattc
>_R_gnl|SRA|SRR352190.1332186.1:1-35 HWI-EAS216_0001:8:6:9860:21436.
----ttanagaacgcaaac---aggaccatagtgggggattc
>_R_gnl|SRA|SRR352190.15409447.1:3-35 HWI-EAS216_0001:8:64:18101:5051.
------acagaacgcaaac---aggaccatagtgggggattc
>gnl|SRA|SRR352190.26646519.1:1-32 HWI-EAS216_0001:8:113:16018:10592.
-------cagaacgcaaac---aggaccatagtgggggattc
>gnl|SRA|SRR352190.26355746.1:1-32 HWI-EAS216_0001:8:112:11461:17653.
-------cagaacgcaaac---aggaccatagtgggggattc
>gnl|SRA|SRR352190.23961887.1:1-32 HWI-EAS216_0001:8:102:4563:17923.
-------cagaacgcaaac---aggaccatagtgggggattc
>gnl|SRA|SRR352190.21621445.1:1-32 HWI-EAS216_0001:8:92:3222:2953.
-------cagaacgcaaac---aggaccatagtgggggattc
>gnl|SRA|SRR352190.17893334.1:1-32 HWI-EAS216_0001:8:75:16350:10482.
-------cagaacgcaaac---aggaccatagtgggggattc
>gnl|SRA|SRR352190.16384813.1:1-32 HWI-EAS216_0001:8:69:5052:20869.
-------cagaacgcaaac---aggaccatagtgggggattc
>_R_gnl|SRA|SRR352190.16676803.1:6-35 HWI-EAS216_0001:8:70:10032:2169.
---------gaacgcaaac---aggaccatagtgggggattc
>gnl|SRA|SRR352190.11817291.1:1-30 HWI-EAS216_0001:8:49:14089:8707.
---------gaacgcaaac---aggaccatagtgggggattc
>gnl|SRA|SRR352190.12371534.1:1-29 HWI-EAS216_0001:8:52:1439:3033.
----------aacgcaaac---aggaccatagtgggggattc
>gnl|SRA|SRR352190.12423931.1:1-28 HWI-EAS216_0001:8:52:5591:13947.
-----------acgcaaac---aggaccatagtgggggattc
>_R_gnl|SRA|SRR352190.20204016.1:9-35 HWI-EAS216_0001:8:85:17736:13454.
------------cgcaaac---aggaccatagtgggggattc
>gnl|SRA|SRR352190.20098238.1:1-27 HWI-EAS216_0001:8:85:9282:7180.
------------cgcaaac---aggaccatagtgggggattc
>_R_gnl|SRA|SRR352190.19887738.1:9-35 HWI-EAS216_0001:8:84:10807:14174.
------------cgcaaac---aggaccatagtgggggattc
>gnl|SRA|SRR352190.19549367.1:1-27 HWI-EAS216_0001:8:83:2459:4977.
------------cgcaaac---aggaccatagtgggggattc
>gnl|SRA|SRR352190.18213172.1:1-27 HWI-EAS216_0001:8:77:5638:15388.
------------cgcaaac---aggaccatagtgggggattc
>_R_gnl|SRA|SRR352190.16917747.1:9-35 HWI-EAS216_0001:8:71:11064:17688.
------------cgcaaac---aggaccatagtgggggattc
>gnl|SRA|SRR352190.10938319.1:1-27 HWI-EAS216_0001:8:46:1649:14526.
------------cgcaaac---aggaccatagtgggggattc

>gnl|SRA|SRR352190.4503236.1:1-27 HWI-EAS216_0001:8:19:9333:12762.
------------cgcaaac---aggaccatagtgggggattc
>_R_gnl|SRA|SRR352190.4436750.1:9-35 HWI-EAS216_0001:8:19:4462:19401.
------------cgcaaac---aggaccatagtgggggattc
>_R_gnl|SRA|SRR352190.308771.1:9-35 HWI-EAS216_0001:8:2:5959:13574.
------------cgcaaac---aggaccatagtgggggattc
>_R_gnl|SRA|SRR352190.15717582.1:9-35 HWI-EAS216_0001:8:66:6345:8924.
------------cgcaaac---aggaccatagtggggggttc
>_R_gnl|SRA|SRR352190.7533803.1:10-35 HWI-EAS216_0001:8:31:16366:2178.
-------------gcaaac---aggaccatagtgggggattc
>gnl|SRA|SRR352190.1881065.1:1-26 HWI-EAS216_0001:8:8:14657:11163.
-------------gcaaac---aggaccatagtgggggattc
>_R_gnl|SRA|SRR352190.329714.1:10-35 HWI-EAS216_0001:8:2:7508:15405.
-------------gcaaac---aggaccatagtgggggattc
>gnl|SRA|SRR352190.25235354.1:1-19 HWI-EAS216_0001:8:107:14016:17056.
-----------------------ggaccatagtgggggattc
>gnl|SRA|SRR352190.22141040.1:1-19 HWI-EAS216_0001:8:94:7693:19986.
-----------------------ggaccatagtgggggattc
>gnl|SRA|SRR352190.10244026.1:3-21 HWI-EAS216_0001:8:43:3937:13049.
-----------------------ggaccatagtgggggattc
>gnl|SRA|SRR352190.2475160.1:1-19 HWI-EAS216_0001:8:11:4149:11685.
-----------------------ggaccatagtgggggattc
>gnl|SRA|SRR352190.867508.1:3-21 HWI-EAS216_0001:8:4:11532:2172.
-----------------------ggaccatagtgggggattc
>_R_gnl|SRA|SRR352190.24744545.1:18-35 HWI-EAS216_0001:8:105:11233:10550.
------------------------gaccatagtgggggattc
>_R_gnl|SRA|SRR352190.23542846.1:18-35 HWI-EAS216_0001:8:100:8320:11253.
------------------------gaccatagtgggggattc
>_R_gnl|SRA|SRR352190.17836229.1:18-35 HWI-EAS216_0001:8:75:11704:9798.
------------------------gaccatagtgggggattc
>_R_gnl|SRA|SRR352190.13700753.1:18-35 HWI-EAS216_0001:8:57:12177:13428.
------------------------gaccatagtgggggattc
>gnl|SRA|SRR352190.13296138.1:1-18 HWI-EAS216_0001:8:55:17674:1633.
------------------------gaccatagtgggggattc
>_R_gnl|SRA|SRR352190.9378878.1:18-35 HWI-EAS216_0001:8:39:11798:9249.
------------------------gaccatagtgggggattc
>gnl|SRA|SRR352190.6074872.1:1-17 HWI-EAS216_0001:8:25:16833:18922.
-------------------------accatagtgggggattc
>_R_gnl|SRA|SRR352190.25799618.1:20-35 HWI-EAS216_0001:8:110:4556:19185.
--------------------------ccatagtgggggattc
>gnl|SRA|SRR352190.2617685.1:1-16 HWI-EAS216_0001:8:11:14780:5696.
--------------------------ccatagtgggggattc
>gnl|SRA|SRR352190.26014470.1:1-35 HWI-EAS216_0001:8:111:3229:18536.
---cttacagaacgcaaacagtaggaccatagtggggg----
>_R_gnl|SRA|SRR352190.16227020.1:1-35 HWI-EAS216_0001:8:68:10604:4128.
---cttacagaacgcaaacagtaggaccatagtggggg----
>gnl|SRA|SRR352190.14437877.1:1-35 HWI-EAS216_0001:8:60:13844:18424.
---cttacagaacgcaaacagtaggaccatagtggggg----
>_R_gnl|SRA|SRR352190.5972938.1:1-35 HWI-EAS216_0001:8:25:9207:17789.
---cttacagaacgcaaacagtaggaccatagtggggg----
>gnl|SRA|SRR352190.1662929.1:1-35 HWI-EAS216_0001:8:7:16586:9505.

---cttacagaacgcaaacagtaggaccatagtggggg----
>gnl|SRA|SRR352190.1075107.1:1-35 HWI-EAS216_0001:8:5:8850:8632.
---cttacagaacgcaaacagtaggaccatagtggggg----
>_R_gnl|SRA|SRR352190.8008.1:1-35 HWI-EAS216_0001:8:1:1803:2097.
---cttacagaacgcaaacagtaggaccatagtagggg----
>gnl|SRA|SRR352190.267842.1:1-31 HWI-EAS216_0001:8:2:2896:17982.
---cttacagaacgcaaacagtaggaccatagtg--------
>_R_gnl|SRA|SRR352190.18203939.1:7-35 HWI-EAS216_0001:8:77:4918:18238.
---------gaacgcaaacagtaggaccatagtggggg----
>gnl|SRA|SRR352190.23568202.1:1-35 HWI-EAS216_0001:8:100:10260:18935.
--gcttacagaacgcaaacagtaggaccatagtgggg-----
>_R_gnl|SRA|SRR352190.19823095.1:1-35 HWI-EAS216_0001:8:84:5831:19926.
--gcttacagaacgcaaacagtaggaccatagtgggg-----
>gnl|SRA|SRR352190.18890293.1:1-35 HWI-EAS216_0001:8:80:5001:2358.
--gcttacagaacgcaaacagtaggaccatagtgggg-----
>gnl|SRA|SRR352190.16988189.1:1-35 HWI-EAS216_0001:8:71:16784:15841.
--gcttacagaacgcaaacagtaggaccatagtgggg-----
>_R_gnl|SRA|SRR352190.14441149.1:1-35 HWI-EAS216_0001:8:60:14091:20390.
--gcttacagaacgcaaacagtaggaccatagtgggg-----
>gnl|SRA|SRR352190.12046489.1:1-35 HWI-EAS216_0001:8:50:13299:2121.
--gcttacagaacgcaaacagtaggaccatagtgggg-----
>_R_gnl|SRA|SRR352190.4441679.1:1-35 HWI-EAS216_0001:8:19:4826:8759.
--gcttacagaacgcaaacagtaggaccatagtgggg-----
>_R_gnl|SRA|SRR352190.10429146.1:1-35 HWI-EAS216_0001:8:43:18050:14809.
--gcttgcagaacgcaaacagtaggaccatagtgggg-----
>gnl|SRA|SRR352190.18673205.1:1-35 HWI-EAS216_0001:8:79:5868:5609.
-agcttacagaacgcaaacagtaggaccatagtggg------
>gnl|SRA|SRR352190.18098673.1:1-35 HWI-EAS216_0001:8:76:14562:4559.
-agcttacagaacgcaaacagtaggaccatagtggg------
>_R_gnl|SRA|SRR352190.10676529.1:1-35 HWI-EAS216_0001:8:44:18584:17394.
-agcttacagaacgcaaacagtaggaccatagtggg------
>_R_gnl|SRA|SRR352190.8600847.1:1-35 HWI-EAS216_0001:8:36:8247:18552.
-agcttacagaacgcaaacagtaggaccatagtggg------
>gnl|SRA|SRR352190.206969.1:1-35 HWI-EAS216_0001:8:1:16626:7082.
-agcttacagaacgcaaacagtaggaccatagtggg------
>_R_gnl|SRA|SRR352190.2105447.1:1-35 HWI-EAS216_0001:8:9:13191:21193.
-agcttacagaacgcaaacagtaggaccatagtcgg------
>gnl|SRA|SRR352190.13975615.1:1-35 HWI-EAS216_0001:8:58:15075:4027.
------acagaacgcaaacagtaggaccatagtgggggatt-
>gnl|SRA|SRR352190.12275032.1:1-35 HWI-EAS216_0001:8:51:12482:7503.
------acagaacgcaaacagtaggaccatagtgggggatt-
>gnl|SRA|SRR352190.10186805.1:1-35 HWI-EAS216_0001:8:42:17978:15445.
------acagaacgcaaacagtaggaccatagtgggggatt-
>_R_gnl|SRA|SRR352190.15643982.1:2-35 HWI-EAS216_0001:8:65:18831:18014.
--gcttacagaacgcaaac---aggaccatagtggggga---
>_R_gnl|SRA|SRR352190.19793052.1:1-35 HWI-EAS216_0001:8:84:3523:3964.
---cttacagaacgcaaac---aggaccttagtgggggatt-
>_R_gnl|SRA|SRR352190.27641059.1:1-35 HWI-EAS216_0001:8:118:2692:17915.
tagcttacagaacgcaaacagtaggaccatagtgg-------
>_R_gnl|SRA|SRR352190.26513219.1:1-35 HWI-EAS216_0001:8:113:5769:17326.
tagcttacagaacgcaaacagtaggaccatagtgg-------

>_R_gnl|SRA|SRR352190.25037017.1:1-35 HWI-EAS216_0001:8:106:16080:17063.
tagcttacagaacgcaaacagtaggaccatagtgg-------
>_R_gnl|SRA|SRR352190.23115731.1:1-35 HWI-EAS216_0001:8:98:11004:6689.
tagcttacagaacgcaaacagtaggaccatagtgg-------
>_R_gnl|SRA|SRR352190.2177321.1:1-35 HWI-EAS216_0001:8:9:18705:17581.
tagcttacagaacgcaaacagtaggaccatagtgg-------
>_R_gnl|SRA|SRR352190.202144.1:1-35 HWI-EAS216_0001:8:1:16257:7136.
tagcttacagaacgcaaacagtaggaccatagcgg-------
>_R_gnl|SRA|SRR352190.23322281.1:1-34 HWI-EAS216_0001:8:99:9051:4059.
tagcttacagaacgcaaacagtaggaccatagtg--------
>gnl|SRA|SRR352190.22804403.1:2-35 HWI-EAS216_0001:8:97:4996:19050.
tagcttacagaacgcaaacagtaggaccatagtg--------
>_R_gnl|SRA|SRR352190.16109556.1:1-34 HWI-EAS216_0001:8:68:1488:11730.
tagcttacagaacgcaaacagtaggaccatagtg--------
>gnl|SRA|SRR352190.15347244.1:2-35 HWI-EAS216_0001:8:64:12911:9433.
tagcttacagaacgcaaacagtaggaccatagtg--------
>gnl|SRA|SRR352190.3685187.1:2-35 HWI-EAS216_0001:8:16:3389:6570.
tagcttacagaacgcaaacagtaggaccatagtg--------
>gnl|SRA|SRR352190.449083.1:2-35 HWI-EAS216_0001:8:2:16488:4183.
tagcttacagaacgcaaacagtaggaccatagtg--------
>_R_gnl|SRA|SRR352190.9787324.1:3-35 HWI-EAS216_0001:8:41:5867:17408.
tagcttacagaacgcaaacagtaggaccatagt---------
>_R_gnl|SRA|SRR352190.27255708.1:1-32 HWI-EAS216_0001:8:116:8792:2015.
tagcttacagaacgcaaacagtaggaccatag----------
>_R_gnl|SRA|SRR352190.16722831.1:1-32 HWI-EAS216_0001:8:70:13696:20772.
tagcttacagaacgcaaacagtaggaccatag----------
>gnl|SRA|SRR352190.16615972.1:4-35 HWI-EAS216_0001:8:70:5294:10951.
tagcttacagaacgcaaacagtaggaccatag----------
>_R_gnl|SRA|SRR352190.13254299.1:1-32 HWI-EAS216_0001:8:55:14416:9658.
tagcttacagaacgcaaacagtaggaccatag----------
>_R_gnl|SRA|SRR352190.10074757.1:1-32 HWI-EAS216_0001:8:42:9368:19205.
tagcttacagaacgcaaacagtaggaccatag----------
>gnl|SRA|SRR352190.4532367.1:4-35 HWI-EAS216_0001:8:19:11495:5200.
tagcttacagaacgcaaacagtaggaccatag----------
>gnl|SRA|SRR352190.4149533.1:4-35 HWI-EAS216_0001:8:18:1169:15587.
tagcttacagaacgcaaacagtaggaccatag----------
>_R_gnl|SRA|SRR352190.6696791.1:1-31 HWI-EAS216_0001:8:28:8443:7376.
tagcttacagaacgcaaacagtaggaccata-----------
>_R_gnl|SRA|SRR352190.25144397.1:1-29 HWI-EAS216_0001:8:107:6606:11398.
tagcttacagaacgcaaacagtaggacca-------------
>gnl|SRA|SRR352190.21883857.1:7-35 HWI-EAS216_0001:8:93:5652:2025.
tagcttacagaacgcaaacagtaggacca-------------
>gnl|SRA|SRR352190.12515317.1:7-35 HWI-EAS216_0001:8:52:12607:9442.
tagcttacagaacgcaaacagtaggacca-------------
>_R_gnl|SRA|SRR352190.27417179.1:2-29 HWI-EAS216_0001:8:117:3356:14829.
tagcttacagaacgcaaacagtaggacc--------------
>_R_gnl|SRA|SRR352190.23493738.1:2-29 HWI-EAS216_0001:8:100:4614:11524.
tagcttacagaacgcaaacagtaggacc--------------
>_R_gnl|SRA|SRR352190.13495091.1:2-29 HWI-EAS216_0001:8:56:14629:4150.
tagcttacagaacgcaaacagtaggacc--------------
>gnl|SRA|SRR352190.11776482.1:8-35 HWI-EAS216_0001:8:49:10977:18350.

tagcttacagaacgcaaacagtaggacc--------------
>gnl|SRA|SRR352190.22545263.1:9-35 HWI-EAS216_0001:8:96:3243:9264.
tagcttacagaacgcaaacagtaggac---------------
>_R_gnl|SRA|SRR352190.21707384.1:1-27 HWI-EAS216_0001:8:92:9817:17893.
tagcttacagaacgcaaacagtaggac---------------
>gnl|SRA|SRR352190.13869641.1:9-35 HWI-EAS216_0001:8:58:6873:8765.
tagcttacagaacgcaaacagtaggac---------------
>_R_gnl|SRA|SRR352190.11530356.1:1-27 HWI-EAS216_0001:8:48:10406:17010.
tagcttacagaacgcaaacagtaggac---------------
>gnl|SRA|SRR352190.8845319.1:9-35 HWI-EAS216_0001:8:37:8279:14170.
tagcttacagaacgcaaacagtaggac---------------
>gnl|SRA|SRR352190.1713164.1:9-35 HWI-EAS216_0001:8:8:2090:10849.
tagcttacagaacgcaaacagtaggac---------------
>_R_gnl|SRA|SRR352190.17904763.1:1-24 HWI-EAS216_0001:8:75:17323:20170.
tagcttacagaacgcaaacagtag------------------
>_R_gnl|SRA|SRR352190.12930000.1:1-24 HWI-EAS216_0001:8:54:7812:18124.
tagcttacagaacgcaaacagtag------------------
>_R_gnl|SRA|SRR352190.9481128.1:1-24 HWI-EAS216_0001:8:39:19737:9803.
tagcttacagaacgcaaacagtag------------------
>_R_gnl|SRA|SRR352190.9218316.1:1-24 HWI-EAS216_0001:8:38:18090:16767.
tagcttacagaacgcaaacagtag------------------
>_R_gnl|SRA|SRR352190.7735715.1:1-24 HWI-EAS216_0001:8:32:13193:20319.
tagcttacagaacgcaaacagtag------------------
>_R_gnl|SRA|SRR352190.1713392.1:1-24 HWI-EAS216_0001:8:8:2107:16049.
tagcttacagaacgcaaacagtag------------------
>_R_gnl|SRA|SRR352190.24980552.1:1-23 HWI-EAS216_0001:8:106:11674:18306.
tagcttacagaacgcaaacagta-------------------
>_R_gnl|SRA|SRR352190.22642958.1:1-23 HWI-EAS216_0001:8:96:10579:19944.
tagcttacagaacgcaaacagta-------------------
>_R_gnl|SRA|SRR352190.145334.1:1-23 HWI-EAS216_0001:8:1:12019:15051.
tagcttacagaacgcaaacagta-------------------
>_R_gnl|SRA|SRR352190.18114589.1:6-27 HWI-EAS216_0001:8:76:15856:20837.
tagcttacagaacgcaaacagt--------------------
>gnl|SRA|SRR352190.6197140.1:14-35 HWI-EAS216_0001:8:26:7616:8097.
tagcttacagaacgcaaacagt--------------------
>gnl|SRA|SRR352190.24576259.1:12-32 HWI-EAS216_0001:8:104:16229:4958.
tagcttacagaacgcaaacag---------------------
>_R_gnl|SRA|SRR352190.23359167.1:9-29 HWI-EAS216_0001:8:99:11970:9537.
tagcttacagaacgcaaacag---------------------
>gnl|SRA|SRR352190.21941012.1:11-31 HWI-EAS216_0001:8:93:10063:18123.
tagcttacagaacgcaaacag---------------------
>_R_gnl|SRA|SRR352190.21099269.1:1-21 HWI-EAS216_0001:8:89:16323:16816.
tagcttacagaacgcaaacag---------------------
>_R_gnl|SRA|SRR352190.17327621.1:1-21 HWI-EAS216_0001:8:73:7529:12136.
tagcttacagaacgcaaacag---------------------
>_R_gnl|SRA|SRR352190.13704991.1:2-22 HWI-EAS216_0001:8:57:12503:13505.
tagcttacagaacgcaaacag---------------------
>gnl|SRA|SRR352190.12221077.1:15-35 HWI-EAS216_0001:8:51:8335:8842.
tagcttacagaacgcaaacag---------------------
>gnl|SRA|SRR352190.11496774.1:10-30 HWI-EAS216_0001:8:48:7832:8224.
tagcttacagaacgcaaacag---------------------

>_R_gnl|SRA|SRR352190.9620644.1:2-22 HWI-EAS216_0001:8:40:11647:15199.
tagcttacagaacgcaaacag---------------------
>_R_gnl|SRA|SRR352190.7401399.1:4-24 HWI-EAS216_0001:8:31:6462:3338.
tagcttacagaacgcaaacag---------------------
>gnl|SRA|SRR352190.7307831.1:15-35 HWI-EAS216_0001:8:30:17782:2012.
tagcttacagaacgcaaacag---------------------
>_R_gnl|SRA|SRR352190.7178825.1:4-24 HWI-EAS216_0001:8:30:8028:1361.
tagcttacagaacgcaaacag---------------------
>_R_gnl|SRA|SRR352190.6907061.1:4-24 HWI-EAS216_0001:8:29:5966:1886.
tagcttacagaacgcaaacag---------------------
>gnl|SRA|SRR352190.5760600.1:15-35 HWI-EAS216_0001:8:24:11678:1111.
tagcttacagaacgcaaacag---------------------
>gnl|SRA|SRR352190.5212429.1:14-34 HWI-EAS216_0001:8:22:7341:7938.
tagcttacagaacgcaaacag---------------------
>gnl|SRA|SRR352190.4798680.1:8-28 HWI-EAS216_0001:8:20:13071:10923.
tagcttacagaacgcaaacag---------------------
>gnl|SRA|SRR352190.4282874.1:15-35 HWI-EAS216_0001:8:18:11229:11087.
tagcttacagaacgcaaacag---------------------
>_R_gnl|SRA|SRR352190.4129861.1:9-29 HWI-EAS216_0001:8:17:18241:19250.
tagcttacagaacgcaaacag---------------------
>gnl|SRA|SRR352190.3848328.1:15-35 HWI-EAS216_0001:8:16:15497:14100.
tagcttacagaacgcaaacag---------------------
>gnl|SRA|SRR352190.2944166.1:15-35 HWI-EAS216_0001:8:13:2699:17101.
tagcttacagaacgcaaacag---------------------
>_R_gnl|SRA|SRR352190.20508748.1:1-35 HWI-EAS216_0001:8:87:5788:4886.
tagcttacagaacgcaaacag---gaccatagtggggg----
>_R_gnl|SRA|SRR352190.1920930.1:1-35 HWI-EAS216_0001:8:8:17745:13842.
tagcttacagaacgcaaacag---gaccatagtggggg----
>gnl|SRA|SRR352190.27645048.1:2-35 HWI-EAS216_0001:8:118:3001:2150.
tagcttacagaacgcaaacag---gaccatagtgggg-----
>_R_gnl|SRA|SRR352190.27185878.1:1-34 HWI-EAS216_0001:8:116:3509:17343.
tagcttacagaacgcaaacag---gaccatagtgggg-----
>_R_gnl|SRA|SRR352190.26026445.1:1-34 HWI-EAS216_0001:8:111:4116:7425.
tagcttacagaacgcaaacag---gaccatagtgggg-----
>_R_gnl|SRA|SRR352190.23339576.1:1-34 HWI-EAS216_0001:8:99:10416:4767.
tagcttacagaacgcaaacag---gaccatagtgggg-----
>_R_gnl|SRA|SRR352190.18282774.1:1-34 HWI-EAS216_0001:8:77:11066:11565.
tagcttacagaacgcaaacag---gaccatagtgggg-----
>_R_gnl|SRA|SRR352190.15835983.1:1-34 HWI-EAS216_0001:8:66:15758:7287.
tagcttacagaacgcaaacag---gaccatagtgggg-----
>gnl|SRA|SRR352190.5945748.1:2-35 HWI-EAS216_0001:8:25:7192:3901.
tagcttacagaacgcaaacag---gaccatagtgggg-----
>_R_gnl|SRA|SRR352190.540963.1:1-34 HWI-EAS216_0001:8:3:5160:11710.
tagcttacagaacgcaaacag---gaccatagtgggg-----
>_R_gnl|SRA|SRR352190.26475761.1:1-32 HWI-EAS216_0001:8:113:2929:7046.
tagcttacagaacgcaaacag---gaccatagtgg-------
>_R_gnl|SRA|SRR352190.10503347.1:1-32 HWI-EAS216_0001:8:44:5304:18440.
tagcttacagaacgcaaacag---gaccatagtgg-------
>_R_gnl|SRA|SRR352190.8771084.1:1-32 HWI-EAS216_0001:8:37:2705:1307.
tagcttacagaacgcaaacag---gaccatagtgg-------
>_R_gnl|SRA|SRR352190.18660390.1:1-32 HWI-EAS216_0001:8:79:4882:9658.

tagcttacagaacgcaaacag---gaccatagcgg-------
>_R_gnl|SRA|SRR352190.6515228.1:1-31 HWI-EAS216_0001:8:27:13115:4962.
tagcttacagaacgcaaacag---gaccatagtg--------
>gnl|SRA|SRR352190.16553539.1:6-35 HWI-EAS216_0001:8:69:18658:7859.
tagcttacagaacgcaaacag---gaccatagt---------
>gnl|SRA|SRR352190.15046883.1:6-35 HWI-EAS216_0001:8:63:6983:11103.
tagcttacagaacgcaaacag---gaccatagt---------
>gnl|SRA|SRR352190.10963242.1:6-35 HWI-EAS216_0001:8:46:3646:7809.
tagcttacagaacgcaaacag---gaccatagt---------
>_R_gnl|SRA|SRR352190.8789228.1:1-30 HWI-EAS216_0001:8:37:4088:16634.
tagcttacagaacgcaaacag---gaccatagt---------
>_R_gnl|SRA|SRR352190.4150830.1:1-30 HWI-EAS216_0001:8:18:1310:18634.
tagcttacagaacgcaaacag---gaccatagt---------
>_R_gnl|SRA|SRR352190.27865243.1:1-29 HWI-EAS216_0001:8:119:2249:6253.
tagcttacagaacgcaaacag---gaccatag----------
>_R_gnl|SRA|SRR352190.21873637.1:1-29 HWI-EAS216_0001:8:93:4859:15375.
tagcttacagaacgcaaacag---gaccatag----------
>_R_gnl|SRA|SRR352190.19421555.1:1-29 HWI-EAS216_0001:8:82:10289:6036.
tagcttacagaacgcaaacag---gaccatag----------
>_R_gnl|SRA|SRR352190.16883181.1:1-29 HWI-EAS216_0001:8:71:8326:20846.
tagcttacagaacgcaaacag---gaccatag----------
>gnl|SRA|SRR352190.6516763.1:7-35 HWI-EAS216_0001:8:27:13235:17604.
tagcttacagaacgcaaacag---gaccatag----------
>gnl|SRA|SRR352190.3695597.1:7-35 HWI-EAS216_0001:8:16:4169:1461.
tagcttacagaacgcaaacag---gaccatag----------
>gnl|SRA|SRR352190.3458110.1:7-35 HWI-EAS216_0001:8:15:4740:6744.
tagcttacagaacgcaaacag---gaccatag----------
>_R_gnl|SRA|SRR352190.3443633.1:2-30 HWI-EAS216_0001:8:15:3661:7958.
tagcttacagaacgcaaacag---gaccatag----------
>gnl|SRA|SRR352190.3204558.1:7-35 HWI-EAS216_0001:8:14:3968:18774.
tagcttacagaacgcaaacag---gaccatag----------
>_R_gnl|SRA|SRR352190.2189948.1:3-31 HWI-EAS216_0001:8:9:19742:6256.
tagcttacagaacgcaaacag---gaccatag----------
>gnl|SRA|SRR352190.26161007.1:8-35 HWI-EAS216_0001:8:111:14388:5581.
tagcttacagaacgcaaacag---gaccata-----------
>gnl|SRA|SRR352190.23821469.1:8-35 HWI-EAS216_0001:8:101:11671:3397.
tagcttacagaacgcaaacag---gaccata-----------
>gnl|SRA|SRR352190.10629383.1:9-35 HWI-EAS216_0001:8:44:14907:5718.
tagcttacagaacgcaaacag---gaccat------------
>_R_gnl|SRA|SRR352190.28126068.1:1-19 HWI-EAS216_0001:8:120:4717:11740.
tagcttacagaacgcaaac-----------------------
>_R_gnl|SRA|SRR352190.27359203.1:1-19 HWI-EAS216_0001:8:116:16887:20500.
tagcttacagaacgcaaac-----------------------
>_R_gnl|SRA|SRR352190.25116967.1:1-19 HWI-EAS216_0001:8:107:4405:5401.
tagcttacagaacgcaaac-----------------------
>_R_gnl|SRA|SRR352190.23662007.1:1-19 HWI-EAS216_0001:8:100:17525:17132.
tagcttacagaacgcaaac-----------------------
>_R_gnl|SRA|SRR352190.22447647.1:1-19 HWI-EAS216_0001:8:95:13671:18136.
tagcttacagaacgcaaac-----------------------
>_R_gnl|SRA|SRR352190.22222183.1:1-19 HWI-EAS216_0001:8:94:14036:8955.
tagcttacagaacgcaaac-----------------------

>_R_gnl|SRA|SRR352190.15268011.1:1-19 HWI-EAS216_0001:8:64:6611:7779.
tagcttacagaacgcaaac-----------------------
>_R_gnl|SRA|SRR352190.13744033.1:1-19 HWI-EAS216_0001:8:57:15533:4215.
tagcttacagaacgcaaac-----------------------
>_R_gnl|SRA|SRR352190.13689879.1:1-19 HWI-EAS216_0001:8:57:11351:1600.
tagcttacagaacgcaaac-----------------------
>_R_gnl|SRA|SRR352190.7469452.1:2-20 HWI-EAS216_0001:8:31:11528:9962.
tagcttacagaacgcaaac-----------------------
>_R_gnl|SRA|SRR352190.6183650.1:1-19 HWI-EAS216_0001:8:26:6618:8528.
tagcttacagaacgcaaac-----------------------
>_R_gnl|SRA|SRR352190.5978959.1:1-19 HWI-EAS216_0001:8:25:9658:3438.
tagcttacagaacgcaaac-----------------------
>_R_gnl|SRA|SRR352190.5547502.1:1-19 HWI-EAS216_0001:8:23:14016:1214.
tagcttacagaacgcaaac-----------------------
>_R_gnl|SRA|SRR352190.4712099.1:1-19 HWI-EAS216_0001:8:20:6651:9237.
tagcttacagaacgcaaac-----------------------
>_R_gnl|SRA|SRR352190.3425667.1:1-19 HWI-EAS216_0001:8:15:2291:12952.
tagcttacagaacgcaaac-----------------------
>_R_gnl|SRA|SRR352190.2568806.1:1-19 HWI-EAS216_0001:8:11:11092:11877.
tagcttacagaacgcaaac-----------------------
>_R_gnl|SRA|SRR352190.1761245.1:1-19 HWI-EAS216_0001:8:8:5723:9785.
tagcttacagaacgcaaac-----------------------
>_R_gnl|SRA|SRR352190.545889.1:1-19 HWI-EAS216_0001:8:3:5528:7239.
tagcttacagaacgcaaac-----------------------
>_R_gnl|SRA|SRR352190.25898818.1:1-18 HWI-EAS216_0001:8:110:12172:18875.
tagcttacagaacgcaaa------------------------
>_R_gnl|SRA|SRR352190.25595496.1:1-18 HWI-EAS216_0001:8:109:6523:21224.
tagcttacagaacgcaaa------------------------
>_R_gnl|SRA|SRR352190.22392630.1:1-18 HWI-EAS216_0001:8:95:9400:11829.
tagcttacagaacgcaaa------------------------
>_R_gnl|SRA|SRR352190.19358781.1:1-18 HWI-EAS216_0001:8:82:5450:6839.
tagcttacagaacgcaaa------------------------
>_R_gnl|SRA|SRR352190.18828804.1:1-18 HWI-EAS216_0001:8:79:18371:16630.
tagcttacagaacgcaaa------------------------
>_R_gnl|SRA|SRR352190.18212937.1:1-18 HWI-EAS216_0001:8:77:5619:11093.
tagcttacagaacgcaaa------------------------
>_R_gnl|SRA|SRR352190.16538182.1:1-18 HWI-EAS216_0001:8:69:17264:10656.
tagcttacagaacgcaaa------------------------
>_R_gnl|SRA|SRR352190.15613372.1:2-19 HWI-EAS216_0001:8:65:16123:7020.
tagcttacagaacgcaaa------------------------
>gnl|SRA|SRR352190.14473442.1:18-35 HWI-EAS216_0001:8:60:16583:2360.
tagcttacagaacgcaaa------------------------
>_R_gnl|SRA|SRR352190.14061110.1:1-18 HWI-EAS216_0001:8:59:3326:13197.
tagcttacagaacgcaaa------------------------
>_R_gnl|SRA|SRR352190.13865299.1:1-18 HWI-EAS216_0001:8:58:6539:21066.
tagcttacagaacgcaaa------------------------
>gnl|SRA|SRR352190.12756231.1:18-35 HWI-EAS216_0001:8:53:12803:2157.
tagcttacagaacgcaaa------------------------
>_R_gnl|SRA|SRR352190.11753065.1:1-18 HWI-EAS216_0001:8:49:9187:18272.
tagcttacagaacgcaaa------------------------
>_R_gnl|SRA|SRR352190.11644957.1:1-18 HWI-EAS216_0001:8:48:19378:2171.

tagcttacagaacgcaaa------------------------
>_R_gnl|SRA|SRR352190.10701098.1:1-18 HWI-EAS216_0001:8:45:2006:2621.
tagcttacagaacgcaaa------------------------
>gnl|SRA|SRR352190.1183575.1:18-35 HWI-EAS216_0001:8:5:17012:14305.
tagcttacagaacgcaaa------------------------
>_R_gnl|SRA|SRR352190.19099428.1:1-17 HWI-EAS216_0001:8:81:3363:21071.
tagcttacagaacgcaa-------------------------
>_R_gnl|SRA|SRR352190.17768479.1:1-17 HWI-EAS216_0001:8:75:6385:17748.
tagcttacagaacgcaa-------------------------
>_R_gnl|SRA|SRR352190.17763098.1:1-17 HWI-EAS216_0001:8:75:5965:5534.
tagcttacagaacgcaa-------------------------
>_R_gnl|SRA|SRR352190.17079143.1:1-17 HWI-EAS216_0001:8:72:5912:13197.
tagcttacagaacgcaa-------------------------
>_R_gnl|SRA|SRR352190.17017885.1:1-17 HWI-EAS216_0001:8:72:1167:11445.
tagcttacagaacgcaa-------------------------
>_R_gnl|SRA|SRR352190.10614017.1:1-17 HWI-EAS216_0001:8:44:13717:3351.
tagcttacagaacgcaa-------------------------
>_R_gnl|SRA|SRR352190.4445203.1:1-17 HWI-EAS216_0001:8:19:5088:17806.
tagcttacagaacgcaa-------------------------
>_R_gnl|SRA|SRR352190.4370077.1:1-17 HWI-EAS216_0001:8:18:17808:10809.
tagcttacagaacgcaa-------------------------
>_R_gnl|SRA|SRR352190.3762095.1:1-17 HWI-EAS216_0001:8:16:9083:15249.
tagcttacagaacgcaa-------------------------
>_R_gnl|SRA|SRR352190.11484779.1:1-16 HWI-EAS216_0001:8:48:6921:19853.
tagcttacagaacgca--------------------------
>gnl|SRA|SRR352190.9702775.1:20-35 HWI-EAS216_0001:8:40:17864:17957.
tagcttacagaacgca--------------------------
>_R_gnl|SRA|SRR352190.8187948.1:7-22 HWI-EAS216_0001:8:34:12842:17141.
tagcttacagaacgca--------------------------
>_R_gnl|SRA|SRR352190.1816395.1:1-16 HWI-EAS216_0001:8:8:9802:3029.
tagcttacagaacgca--------------------------
>_R_gnl|SRA|SRR352190.8974487.1:18-35 HWI-EAS216_0001:8:37:18090:1450.
----ttacagaacgcaaacagt--------------------
>_R_gnl|SRA|SRR352190.8345534.1:15-35 HWI-EAS216_0001:8:35:7318:2699.
-----tacagaacgcaaacagcagga----------------

SRX472914
>control .
cagtagcttacagaacgcaaacaa---gaccatagtgggggattc
>_R_>gnl|SRA|SRR1171870.209162769.1:24-65 HWI-ST967_77:2:2307:13801:35600.
cagtagcttacagaacgcaaacaa---gaccatagtgggggattc
>_R_gnl|SRA|SRR1171870.208887161.1:17-58 HWI-ST967_77:2:2306:17299:196311.
cagtagcttacagaacgcaaacaa---gaccatagtgggggattc
>gnl|SRA|SRR1171870.208590524.1:23-64 HWI-ST967_77:2:2306:6535:184150.
cagtagcttacagaacgcaaacaa---gaccatagtgggggattc
>_R_gnl|SRA|SRR1171870.208507651.1:33-74 HWI-ST967_77:2:2306:15039:180755.
cagtagcttacagaacgcaaacaa---gaccatagtgggggattc
>_R_gnl|SRA|SRR1171870.208010968.1:36-77 HWI-ST967_77:2:2306:4810:162380.
cagtagcttacagaacgcaaacaa---gaccatagtgggggattc

>gnl|SRA|SRR1171870.206235599.1:2-43 HWI-ST967_77:2:2306:12057:94929.
cagtagcttacagaacgcaaacaa---gaccatagtgggggattc
>_R_gnl|SRA|SRR1171870.205346690.1:48-89 HWI-ST967_77:2:2306:16049:61601.
cagtagcttacagaacgcaaacaa---gaccatagtgggggattc
>_R_gnl|SRA|SRR1171870.205338656.1:17-58 HWI-ST967_77:2:2306:12338:61421.
cagtagcttacagaacgcaaacaa---gaccatagtgggggattc
>_R_gnl|SRA|SRR1171870.204317832.1:4-45 HWI-ST967_77:2:2306:5019:24022.
cagtagcttacagaacgcaaacaa---gaccatagtgggggattc
>_R_gnl|SRA|SRR1171870.204104037.1:11-52 HWI-ST967_77:2:2306:19933:16186.
cagtagcttacagaacgcaaacaa---gaccatagtgggggattc
>gnl|SRA|SRR1171870.203442183.2:57-98 HWI-ST967_77:2:2305:17531:190472.
cagtagcttacagaacgcaaacaa---gaccatagtgggggattc
>_R_gnl|SRA|SRR1171870.203442183.1:53-94 HWI-ST967_77:2:2305:17531:190472.
cagtagcttacagaacgcaaacaa---gaccatagtgggggattc
>_R_gnl|SRA|SRR1171870.203150787.1:36-77 HWI-ST967_77:2:2305:13905:180050.
cagtagcttacagaacgcaaacaa---gaccatagtgggggattc
>_R_gnl|SRA|SRR1171870.202593909.2:28-69 HWI-ST967_77:2:2305:2184:160369.
cagtagcttacagaacgcaaacaa---gaccatagtgggggattc
>gnl|SRA|SRR1171870.202593909.1:52-93 HWI-ST967_77:2:2305:2184:160369.
cagtagcttacagaacgcaaacaa---gaccatagtgggggattc
>gnl|SRA|SRR1171870.202102594.1:12-53 HWI-ST967_77:2:2305:1095:142588.
cagtagcttacagaacgcaaacaa---gaccatagtgggggattc
>_R_gnl|SRA|SRR1171870.201658364.1:41-82 HWI-ST967_77:2:2305:15432:126404.
cagtagcttacagaacgcaaacaa---gaccatagtgggggattc
>gnl|SRA|SRR1171870.201539353.2:57-98 HWI-ST967_77:2:2305:2348:122249.
cagtagcttacagaacgcaaacaa---gaccatagtgggggattc
>_R_gnl|SRA|SRR1171870.201539353.1:59-100 HWI-ST967_77:2:2305:2348:122249.
cagtagcttacagaacgcaaacaa---gaccatagtgggggattc
>_R_gnl|SRA|SRR1171870.200040599.1:2-43 HWI-ST967_77:2:2305:8209:64459.
cagtagcttacagaacgcaaacaa---gaccatagtgggggattc
>gnl|SRA|SRR1171870.199706672.2:20-61 HWI-ST967_77:2:2305:18092:51783.
cagtagcttacagaacgcaaacaa---gaccatagtgggggattc
>_R_gnl|SRA|SRR1171870.199271209.1:37-78 HWI-ST967_77:2:2305:18739:35887.
cagtagcttacagaacgcaaacaa---gaccatagtgggggattc
>_R_gnl|SRA|SRR1171870.199215941.1:53-94 HWI-ST967_77:2:2305:16848:33818.
cagtagcttacagaacgcaaacaa---gaccatagtgggggattc
>_R_gnl|SRA|SRR1171870.199136221.1:30-71 HWI-ST967_77:2:2305:3933:31213.
cagtagcttacagaacgcaaacaa---gaccatagtgggggattc
>_R_gnl|SRA|SRR1171870.198303906.1:33-74 HWI-ST967_77:2:2304:4826:199877.
cagtagcttacagaacgcaaacaa---gaccatagtgggggattc
>gnl|SRA|SRR1171870.197043897.2:1-42 HWI-ST967_77:2:2304:5628:155031.
cagtagcttacagaacgcaaacaa---gaccatagtgggggattc
>gnl|SRA|SRR1171870.194607875.2:38-79 HWI-ST967_77:2:2304:5392:67286.
cagtagcttacagaacgcaaacaa---gaccatagtgggggattc
>gnl|SRA|SRR1171870.193543941.1:22-63 HWI-ST967_77:2:2304:14990:29089.
cagtagcttacagaacgcaaacaa---gaccatagtgggggattc
>_R_gnl|SRA|SRR1171870.193503996.2:25-66 HWI-ST967_77:2:2304:20394:27553.
cagtagcttacagaacgcaaacaa---gaccatagtgggggattc
>_R_gnl|SRA|SRR1171870.193093228.1:53-94 HWI-ST967_77:2:2304:8840:13067.
cagtagcttacagaacgcaaacaa---gaccatagtgggggattc
>gnl|SRA|SRR1171870.192275539.2:5-46 HWI-ST967_77:2:2303:3269:180934.

cagtagcttacagaacgcaaacaa---gaccatagtgggggattc
>gnl|SRA|SRR1171870.192228233.2:49-90 HWI-ST967_77:2:2303:14853:178779.
cagtagcttacagaacgcaaacaa---gaccatagtgggggattc
>_R_gnl|SRA|SRR1171870.192228233.1:56-97 HWI-ST967_77:2:2303:14853:178779.
cagtagcttacagaacgcaaacaa---gaccatagtgggggattc
>_R_gnl|SRA|SRR1171870.191658249.1:24-65 HWI-ST967_77:2:2303:15414:157990.
cagtagcttacagaacgcaaacaa---gaccatagtgggggattc
>_R_gnl|SRA|SRR1171870.191536765.2:27-68 HWI-ST967_77:2:2303:3453:153742.
cagtagcttacagaacgcaaacaa---gaccatagtgggggattc
>gnl|SRA|SRR1171870.190926496.1:9-50 HWI-ST967_77:2:2303:8002:131347.
cagtagcttacagaacgcaaacaa---gaccatagtgggggattc
>gnl|SRA|SRR1171870.190683697.2:39-80 HWI-ST967_77:2:2303:3417:122593.
cagtagcttacagaacgcaaacaa---gaccatagtgggggattc
>gnl|SRA|SRR1171870.190599379.2:20-61 HWI-ST967_77:2:2303:16608:119327.
cagtagcttacagaacgcaaacaa---gaccatagtgggggattc
>_R_gnl|SRA|SRR1171870.190157346.1:2-43 HWI-ST967_77:2:2303:4772:103478.
cagtagcttacagaacgcaaacaa---gaccatagtgggggattc
>gnl|SRA|SRR1171870.189985344.2:51-92 HWI-ST967_77:2:2303:16206:96978.
cagtagcttacagaacgcaaacaa---gaccatagtgggggattc
>gnl|SRA|SRR1171870.188827379.2:23-64 HWI-ST967_77:2:2303:15752:54370.
cagtagcttacagaacgcaaacaa---gaccatagtgggggattc
>_R_gnl|SRA|SRR1171870.188812376.2:41-82 HWI-ST967_77:2:2303:12209:53843.
cagtagcttacagaacgcaaacaa---gaccatagtgggggattc
>_R_gnl|SRA|SRR1171870.188245221.1:33-74 HWI-ST967_77:2:2303:11201:33085.
cagtagcttacagaacgcaaacaa---gaccatagtgggggattc
>gnl|SRA|SRR1171870.187917657.1:22-63 HWI-ST967_77:2:2303:11661:21216.
cagtagcttacagaacgcaaacaa---gaccatagtgggggattc
>_R_gnl|SRA|SRR1171870.186579703.1:48-89 HWI-ST967_77:2:2302:10941:170367.
cagtagcttacagaacgcaaacaa---gaccatagtgggggattc
>gnl|SRA|SRR1171870.186405936.2:57-98 HWI-ST967_77:2:2302:7833:163800.
cagtagcttacagaacgcaaacaa---gaccatagtgggggattc
>_R_gnl|SRA|SRR1171870.186405936.1:27-68 HWI-ST967_77:2:2302:7833:163800.
cagtagcttacagaacgcaaacaa---gaccatagtgggggattc
>gnl|SRA|SRR1171870.185401921.1:40-81 HWI-ST967_77:2:2302:11459:125388.
cagtagcttacagaacgcaaacaa---gaccatagtgggggattc
>_R_gnl|SRA|SRR1171870.184188997.1:43-84 HWI-ST967_77:2:2302:20140:78084.
cagtagcttacagaacgcaaacaa---gaccatagtgggggattc
>_R_gnl|SRA|SRR1171870.183797961.2:18-59 HWI-ST967_77:2:2302:4331:63381.
cagtagcttacagaacgcaaacaa---gaccatagtgggggattc
>_R_gnl|SRA|SRR1171870.182539072.1:51-92 HWI-ST967_77:2:2302:18557:15915.
cagtagcttacagaacgcaaacaa---gaccatagtgggggattc
>gnl|SRA|SRR1171870.182393223.2:6-47 HWI-ST967_77:2:2302:20133:10384.
cagtagcttacagaacgcaaacaa---gaccatagtgggggattc
>_R_gnl|SRA|SRR1171870.182261777.1:46-87 HWI-ST967_77:2:2302:7037:5671.
cagtagcttacagaacgcaaacaa---gaccatagtgggggattc
>_R_gnl|SRA|SRR1171870.181788096.2:12-53 HWI-ST967_77:2:2301:10530:185846.
cagtagcttacagaacgcaaacaa---gaccatagtgggggattc
>_R_gnl|SRA|SRR1171870.181701062.2:50-91 HWI-ST967_77:2:2301:7652:182617.
cagtagcttacagaacgcaaacaa---gaccatagtgggggattc
>gnl|SRA|SRR1171870.181111688.2:50-91 HWI-ST967_77:2:2301:16366:160068.
cagtagcttacagaacgcaaacaa---gaccatagtgggggattc

>_R_gnl|SRA|SRR1171870.181111688.1:50-91 HWI-ST967_77:2:2301:16366:160068.
cagtagcttacagaacgcaaacaa---gaccatagtgggggattc
>_R_gnl|SRA|SRR1171870.180907757.2:53-94 HWI-ST967_77:2:2301:13784:152350.
cagtagcttacagaacgcaaacaa---gaccatagtgggggattc
>_R_gnl|SRA|SRR1171870.180536928.2:46-87 HWI-ST967_77:2:2301:8851:137878.
cagtagcttacagaacgcaaacaa---gaccatagtgggggattc
>gnl|SRA|SRR1171870.180536928.1:56-97 HWI-ST967_77:2:2301:8851:137878.
cagtagcttacagaacgcaaacaa---gaccatagtgggggattc
>gnl|SRA|SRR1171870.180000543.2:6-47 HWI-ST967_77:2:2301:6122:116657.
cagtagcttacagaacgcaaacaa---gaccatagtgggggattc
>_R_gnl|SRA|SRR1171870.179650569.2:41-82 HWI-ST967_77:2:2301:17221:102841.
cagtagcttacagaacgcaaacaa---gaccatagtgggggattc
>gnl|SRA|SRR1171870.178844957.2:1-42 HWI-ST967_77:2:2301:6044:71818.
cagtagcttacagaacgcaaacaa---gaccatagtgggggattc
>_R_gnl|SRA|SRR1171870.178684217.2:43-84 HWI-ST967_77:2:2301:8755:65637.
cagtagcttacagaacgcaaacaa---gaccatagtgggggattc
>gnl|SRA|SRR1171870.178484665.2:22-63 HWI-ST967_77:2:2301:10071:57922.
cagtagcttacagaacgcaaacaa---gaccatagtgggggattc
>_R_gnl|SRA|SRR1171870.174918935.1:53-94 HWI-ST967_77:2:2206:14378:172021.
cagtagcttacagaacgcaaacaa---gaccatagtgggggattc
>gnl|SRA|SRR1171870.174585631.1:56-97 HWI-ST967_77:2:2206:7498:156017.
cagtagcttacagaacgcaaacaa---gaccatagtgggggattc
>gnl|SRA|SRR1171870.174532332.1:20-61 HWI-ST967_77:2:2206:11639:153585.
cagtagcttacagaacgcaaacaa---gaccatagtgggggattc
>gnl|SRA|SRR1171870.173618235.2:51-92 HWI-ST967_77:2:2206:8256:112230.
cagtagcttacagaacgcaaacaa---gaccatagtgggggattc
>_R_gnl|SRA|SRR1171870.173618235.1:43-84 HWI-ST967_77:2:2206:8256:112230.
cagtagcttacagaacgcaaacaa---gaccatagtgggggattc
>_R_gnl|SRA|SRR1171870.172852583.1:13-54 HWI-ST967_77:2:2206:18747:79217.
cagtagcttacagaacgcaaacaa---gaccatagtgggggattc
>_R_gnl|SRA|SRR1171870.170963488.1:28-69 HWI-ST967_77:2:2205:2663:199855.
cagtagcttacagaacgcaaacaa---gaccatagtgggggattc
>gnl|SRA|SRR1171870.170844275.2:22-63 HWI-ST967_77:2:2205:5921:195623.
cagtagcttacagaacgcaaacaa---gaccatagtgggggattc
>gnl|SRA|SRR1171870.170291507.2:46-87 HWI-ST967_77:2:2205:13285:176030.
cagtagcttacagaacgcaaacaa---gaccatagtgggggattc
>_R_gnl|SRA|SRR1171870.170291507.1:35-76 HWI-ST967_77:2:2205:13285:176030.
cagtagcttacagaacgcaaacaa---gaccatagtgggggattc
>_R_gnl|SRA|SRR1171870.169955808.1:44-85 HWI-ST967_77:2:2205:19031:164184.
cagtagcttacagaacgcaaacaa---gaccatagtgggggattc
>_R_gnl|SRA|SRR1171870.169309506.2:19-60 HWI-ST967_77:2:2205:13140:141045.
cagtagcttacagaacgcaaacaa---gaccatagtgggggattc
>gnl|SRA|SRR1171870.167649987.1:5-46 HWI-ST967_77:2:2205:3189:80738.
cagtagcttacagaacgcaaacaa---gaccatagtgggggattc
>gnl|SRA|SRR1171870.167154484.2:51-92 HWI-ST967_77:2:2205:7075:62741.
cagtagcttacagaacgcaaacaa---gaccatagtgggggattc
>gnl|SRA|SRR1171870.166387687.1:50-91 HWI-ST967_77:2:2205:19140:35160.
cagtagcttacagaacgcaaacaa---gaccatagtgggggattc
>gnl|SRA|SRR1171870.166245548.1:20-61 HWI-ST967_77:2:2205:19115:30210.
cagtagcttacagaacgcaaacaa---gaccatagtgggggattc
>_R_gnl|SRA|SRR1171870.165098965.1:18-59 HWI-ST967_77:2:2204:13864:188258.

cagtagcttacagaacgcaaacaa---gaccatagtgggggattc
>gnl|SRA|SRR1171870.163360343.2:44-85 HWI-ST967_77:2:2204:13291:126592.
cagtagcttacagaacgcaaacaa---gaccatagtgggggattc
>_R_gnl|SRA|SRR1171870.163189703.2:8-49 HWI-ST967_77:2:2204:4434:120674.
cagtagcttacagaacgcaaacaa---gaccatagtgggggattc
>_R_gnl|SRA|SRR1171870.162970392.1:16-57 HWI-ST967_77:2:2204:15436:112516.
cagtagcttacagaacgcaaacaa---gaccatagtgggggattc
>_R_gnl|SRA|SRR1171870.161140589.2:18-59 HWI-ST967_77:2:2204:4041:47661.
cagtagcttacagaacgcaaacaa---gaccatagtgggggattc
>_R_gnl|SRA|SRR1171870.160659668.2:26-67 HWI-ST967_77:2:2204:7328:30668.
cagtagcttacagaacgcaaacaa---gaccatagtgggggattc
>_R_gnl|SRA|SRR1171870.160086837.1:12-53 HWI-ST967_77:2:2204:5995:10389.
cagtagcttacagaacgcaaacaa---gaccatagtgggggattc
>_R_gnl|SRA|SRR1171870.159481220.1:33-74 HWI-ST967_77:2:2203:19986:186905.
cagtagcttacagaacgcaaacaa---gaccatagtgggggattc
>_R_gnl|SRA|SRR1171870.158537186.1:38-79 HWI-ST967_77:2:2203:17609:152819.
cagtagcttacagaacgcaaacaa---gaccatagtgggggattc
>_R_gnl|SRA|SRR1171870.158255402.2:7-48 HWI-ST967_77:2:2203:10303:142773.
cagtagcttacagaacgcaaacaa---gaccatagtgggggattc
>_R_gnl|SRA|SRR1171870.157513323.1:24-65 HWI-ST967_77:2:2203:5775:116254.
cagtagcttacagaacgcaaacaa---gaccatagtgggggattc
>_R_gnl|SRA|SRR1171870.157414559.1:6-47 HWI-ST967_77:2:2203:1328:112997.
cagtagcttacagaacgcaaacaa---gaccatagtgggggattc
>gnl|SRA|SRR1171870.157088706.2:13-54 HWI-ST967_77:2:2203:16511:100908.
cagtagcttacagaacgcaaacaa---gaccatagtgggggattc
>gnl|SRA|SRR1171870.156611734.1:51-92 HWI-ST967_77:2:2203:13855:83535.
cagtagcttacagaacgcaaacaa---gaccatagtgggggattc
>_R_gnl|SRA|SRR1171870.155698339.1:13-54 HWI-ST967_77:2:2203:13124:50574.
cagtagcttacagaacgcaaacaa---gaccatagtgggggattc
>_R_gnl|SRA|SRR1171870.155299725.1:17-58 HWI-ST967_77:2:2203:7059:36395.
cagtagcttacagaacgcaaacaa---gaccatagtgggggattc
>_R_gnl|SRA|SRR1171870.153932243.2:50-91 HWI-ST967_77:2:2202:9153:185519.
cagtagcttacagaacgcaaacaa---gaccatagtgggggattc
>gnl|SRA|SRR1171870.153932243.1:57-98 HWI-ST967_77:2:2202:9153:185519.
cagtagcttacagaacgcaaacaa---gaccatagtgggggattc
>_R_gnl|SRA|SRR1171870.153373287.1:8-49 HWI-ST967_77:2:2202:16347:165496.
cagtagcttacagaacgcaaacaa---gaccatagtgggggattc
>gnl|SRA|SRR1171870.153320180.2:52-93 HWI-ST967_77:2:2202:3127:163679.
cagtagcttacagaacgcaaacaa---gaccatagtgggggattc
>_R_gnl|SRA|SRR1171870.153030859.2:46-87 HWI-ST967_77:2:2202:13012:152939.
cagtagcttacagaacgcaaacaa---gaccatagtgggggattc
>_R_gnl|SRA|SRR1171870.152667682.1:37-78 HWI-ST967_77:2:2202:17522:139460.
cagtagcttacagaacgcaaacaa---gaccatagtgggggattc
>gnl|SRA|SRR1171870.152207770.1:10-51 HWI-ST967_77:2:2202:13648:122040.
cagtagcttacagaacgcaaacaa---gaccatagtgggggattc
>gnl|SRA|SRR1171870.151925519.2:20-61 HWI-ST967_77:2:2202:7782:111667.
cagtagcttacagaacgcaaacaa---gaccatagtgggggattc
>_R_gnl|SRA|SRR1171870.151450249.1:43-84 HWI-ST967_77:2:2202:11618:93525.
cagtagcttacagaacgcaaacaa---gaccatagtgggggattc
>_R_gnl|SRA|SRR1171870.151237523.1:48-89 HWI-ST967_77:2:2202:6042:85735.
cagtagcttacagaacgcaaacaa---gaccatagtgggggattc

>_R_gnl|SRA|SRR1171870.150772921.2:53-94 HWI-ST967_77:2:2202:17563:68178.
cagtagcttacagaacgcaaacaa---gaccatagtgggggattc
>_R_gnl|SRA|SRR1171870.149702377.1:31-72 HWI-ST967_77:2:2202:7330:28739.
cagtagcttacagaacgcaaacaa---gaccatagtgggggattc
>gnl|SRA|SRR1171870.149193493.2:40-81 HWI-ST967_77:2:2202:1629:9932.
cagtagcttacagaacgcaaacaa---gaccatagtgggggattc
>_R_gnl|SRA|SRR1171870.149177954.2:50-91 HWI-ST967_77:2:2202:15795:9183.
cagtagcttacagaacgcaaacaa---gaccatagtgggggattc
>_R_gnl|SRA|SRR1171870.149150944.1:25-66 HWI-ST967_77:2:2202:16988:8051.
cagtagcttacagaacgcaaacaa---gaccatagtgggggattc
>_R_gnl|SRA|SRR1171870.148842200.1:33-74 HWI-ST967_77:2:2201:5784:195217.
cagtagcttacagaacgcaaacaa---gaccatagtgggggattc
>_R_gnl|SRA|SRR1171870.146944424.1:15-56 HWI-ST967_77:2:2201:11840:123050.
cagtagcttacagaacgcaaacaa---gaccatagtgggggattc
>_R_gnl|SRA|SRR1171870.146211223.2:19-60 HWI-ST967_77:2:2201:9992:94847.
cagtagcttacagaacgcaaacaa---gaccatagtgggggattc
>_R_gnl|SRA|SRR1171870.146207591.1:35-76 HWI-ST967_77:2:2201:18497:94523.
cagtagcttacagaacgcaaacaa---gaccatagtgggggattc
>gnl|SRA|SRR1171870.145605316.2:57-98 HWI-ST967_77:2:2201:1949:71867.
cagtagcttacagaacgcaaacaa---gaccatagtgggggattc
>_R_gnl|SRA|SRR1171870.145605316.1:48-89 HWI-ST967_77:2:2201:1949:71867.
cagtagcttacagaacgcaaacaa---gaccatagtgggggattc
>gnl|SRA|SRR1171870.145507813.2:51-92 HWI-ST967_77:2:2201:6556:68111.
cagtagcttacagaacgcaaacaa---gaccatagtgggggattc
>_R_gnl|SRA|SRR1171870.145368976.1:13-54 HWI-ST967_77:2:2201:5453:62778.
cagtagcttacagaacgcaaacaa---gaccatagtgggggattc
>gnl|SRA|SRR1171870.145318500.1:57-98 HWI-ST967_77:2:2201:11635:60953.
cagtagcttacagaacgcaaacaa---gaccatagtgggggattc
>_R_gnl|SRA|SRR1171870.145128247.1:15-56 HWI-ST967_77:2:2201:13526:53503.
cagtagcttacagaacgcaaacaa---gaccatagtgggggattc
>_R_gnl|SRA|SRR1171870.144582684.1:4-45 HWI-ST967_77:2:2201:6688:32854.
cagtagcttacagaacgcaaacaa---gaccatagtgggggattc
>_R_gnl|SRA|SRR1171870.144102502.2:39-80 HWI-ST967_77:2:2201:8772:14375.
cagtagcttacagaacgcaaacaa---gaccatagtgggggattc
>gnl|SRA|SRR1171870.144102502.1:49-90 HWI-ST967_77:2:2201:8772:14375.
cagtagcttacagaacgcaaacaa---gaccatagtgggggattc
>_R_gnl|SRA|SRR1171870.143960262.2:7-48 HWI-ST967_77:2:2201:9699:8856.
cagtagcttacagaacgcaaacaa---gaccatagtgggggattc
>_R_gnl|SRA|SRR1171870.143889275.1:48-89 HWI-ST967_77:2:2201:9829:6200.
cagtagcttacagaacgcaaacaa---gaccatagtgggggattc
>_R_gnl|SRA|SRR1171870.142638069.1:53-94 HWI-ST967_77:2:2107:7385:30838.
cagtagcttacagaacgcaaacaa---gaccatagtgggggattc
>gnl|SRA|SRR1171870.142379648.2:42-83 HWI-ST967_77:2:2106:6644:199171.
cagtagcttacagaacgcaaacaa---gaccatagtgggggattc
>_R_gnl|SRA|SRR1171870.141550610.1:2-43 HWI-ST967_77:2:2106:11809:167879.
cagtagcttacagaacgcaaacaa---gaccatagtgggggattc
>gnl|SRA|SRR1171870.141347227.1:51-92 HWI-ST967_77:2:2106:11804:160277.
cagtagcttacagaacgcaaacaa---gaccatagtgggggattc
>_R_gnl|SRA|SRR1171870.138868440.1:26-67 HWI-ST967_77:2:2106:14008:68353.
cagtagcttacagaacgcaaacaa---gaccatagtgggggattc
>gnl|SRA|SRR1171870.138189720.1:20-61 HWI-ST967_77:2:2106:4026:43869.

cagtagcttacagaacgcaaacaa---gaccatagtgggggattc
>gnl|SRA|SRR1171870.137659987.1:3-44 HWI-ST967_77:2:2106:4962:24577.
cagtagcttacagaacgcaaacaa---gaccatagtgggggattc
>gnl|SRA|SRR1171870.137214274.2:27-68 HWI-ST967_77:2:2106:20641:8209.
cagtagcttacagaacgcaaacaa---gaccatagtgggggattc
>_R_gnl|SRA|SRR1171870.137214274.1:27-68 HWI-ST967_77:2:2106:20641:8209.
cagtagcttacagaacgcaaacaa---gaccatagtgggggattc
>_R_gnl|SRA|SRR1171870.137183070.1:2-43 HWI-ST967_77:2:2106:11394:7025.
cagtagcttacagaacgcaaacaa---gaccatagtgggggattc
>_R_gnl|SRA|SRR1171870.136828907.1:36-77 HWI-ST967_77:2:2105:10736:192314.
cagtagcttacagaacgcaaacaa---gaccatagtgggggattc
>_R_gnl|SRA|SRR1171870.136139009.2:23-64 HWI-ST967_77:2:2105:14913:167214.
cagtagcttacagaacgcaaacaa---gaccatagtgggggattc
>_R_gnl|SRA|SRR1171870.136037704.2:51-92 HWI-ST967_77:2:2105:20327:163437.
cagtagcttacagaacgcaaacaa---gaccatagtgggggattc
>gnl|SRA|SRR1171870.135500136.2:57-98 HWI-ST967_77:2:2105:4081:144046.
cagtagcttacagaacgcaaacaa---gaccatagtgggggattc
>_R_gnl|SRA|SRR1171870.135500136.1:53-94 HWI-ST967_77:2:2105:4081:144046.
cagtagcttacagaacgcaaacaa---gaccatagtgggggattc
>gnl|SRA|SRR1171870.135369792.1:56-97 HWI-ST967_77:2:2105:1857:139421.
cagtagcttacagaacgcaaacaa---gaccatagtgggggattc
>_R_gnl|SRA|SRR1171870.135346043.1:53-94 HWI-ST967_77:2:2105:11384:138297.
cagtagcttacagaacgcaaacaa---gaccatagtgggggattc
>_R_gnl|SRA|SRR1171870.134463784.1:36-77 HWI-ST967_77:2:2105:7476:105727.
cagtagcttacagaacgcaaacaa---gaccatagtgggggattc
>_R_gnl|SRA|SRR1171870.134391033.1:30-71 HWI-ST967_77:2:2105:11494:102839.
cagtagcttacagaacgcaaacaa---gaccatagtgggggattc
>gnl|SRA|SRR1171870.134047130.1:45-86 HWI-ST967_77:2:2105:19962:89901.
cagtagcttacagaacgcaaacaa---gaccatagtgggggattc
>_R_gnl|SRA|SRR1171870.133990400.1:35-76 HWI-ST967_77:2:2105:9758:87971.
cagtagcttacagaacgcaaacaa---gaccatagtgggggattc
>_R_gnl|SRA|SRR1171870.133804811.2:19-60 HWI-ST967_77:2:2105:11505:80771.
cagtagcttacagaacgcaaacaa---gaccatagtgggggattc
>_R_gnl|SRA|SRR1171870.133633430.2:17-58 HWI-ST967_77:2:2105:7073:74300.
cagtagcttacagaacgcaaacaa---gaccatagtgggggattc
>gnl|SRA|SRR1171870.133507690.1:51-92 HWI-ST967_77:2:2105:5652:69561.
cagtagcttacagaacgcaaacaa---gaccatagtgggggattc
>gnl|SRA|SRR1171870.132561792.2:30-71 HWI-ST967_77:2:2105:18971:34183.
cagtagcttacagaacgcaaacaa---gaccatagtgggggattc
>_R_gnl|SRA|SRR1171870.131243216.1:50-91 HWI-ST967_77:2:2104:16022:184764.
cagtagcttacagaacgcaaacaa---gaccatagtgggggattc
>gnl|SRA|SRR1171870.129268155.2:20-61 HWI-ST967_77:2:2104:11338:112691.
cagtagcttacagaacgcaaacaa---gaccatagtgggggattc
>_R_gnl|SRA|SRR1171870.128346665.1:10-51 HWI-ST967_77:2:2104:6338:78748.
cagtagcttacagaacgcaaacaa---gaccatagtgggggattc
>_R_gnl|SRA|SRR1171870.128189142.1:4-45 HWI-ST967_77:2:2104:4835:72977.
cagtagcttacagaacgcaaacaa---gaccatagtgggggattc
>_R_gnl|SRA|SRR1171870.128075150.1:50-91 HWI-ST967_77:2:2104:10367:68577.
cagtagcttacagaacgcaaacaa---gaccatagtgggggattc
>_R_gnl|SRA|SRR1171870.126815105.1:50-91 HWI-ST967_77:2:2104:13660:22495.
cagtagcttacagaacgcaaacaa---gaccatagtgggggattc

>_R_gnl|SRA|SRR1171870.126428319.2:25-66 HWI-ST967_77:2:2104:10166:8459.
cagtagcttacagaacgcaaacaa---gaccatagtgggggattc
>gnl|SRA|SRR1171870.126132747.2:57-98 HWI-ST967_77:2:2103:12333:195749.
cagtagcttacagaacgcaaacaa---gaccatagtgggggattc
>_R_gnl|SRA|SRR1171870.126132747.1:43-84 HWI-ST967_77:2:2103:12333:195749.
cagtagcttacagaacgcaaacaa---gaccatagtgggggattc
>_R_gnl|SRA|SRR1171870.125455867.2:38-79 HWI-ST967_77:2:2103:17187:170694.
cagtagcttacagaacgcaaacaa---gaccatagtgggggattc
>gnl|SRA|SRR1171870.125455867.1:45-86 HWI-ST967_77:2:2103:17187:170694.
cagtagcttacagaacgcaaacaa---gaccatagtgggggattc
>_R_gnl|SRA|SRR1171870.125439016.1:19-60 HWI-ST967_77:2:2103:6905:170147.
cagtagcttacagaacgcaaacaa---gaccatagtgggggattc
>_R_gnl|SRA|SRR1171870.124298260.2:25-66 HWI-ST967_77:2:2103:10957:127774.
cagtagcttacagaacgcaaacaa---gaccatagtgggggattc
>_R_gnl|SRA|SRR1171870.123676763.1:6-47 HWI-ST967_77:2:2103:9195:104969.
cagtagcttacagaacgcaaacaa---gaccatagtgggggattc
>_R_gnl|SRA|SRR1171870.123032116.2:52-93 HWI-ST967_77:2:2103:11382:80977.
cagtagcttacagaacgcaaacaa---gaccatagtgggggattc
>_R_gnl|SRA|SRR1171870.121982614.1:32-73 HWI-ST967_77:2:2103:15812:41509.
cagtagcttacagaacgcaaacaa---gaccatagtgggggattc
>_R_gnl|SRA|SRR1171870.121014715.2:2-43 HWI-ST967_77:2:2103:20753:5584.
cagtagcttacagaacgcaaacaa---gaccatagtgggggattc
>_R_gnl|SRA|SRR1171870.121012604.2:28-69 HWI-ST967_77:2:2103:14528:5687.
cagtagcttacagaacgcaaacaa---gaccatagtgggggattc
>_R_gnl|SRA|SRR1171870.120785395.2:25-66 HWI-ST967_77:2:2102:14558:195354.
cagtagcttacagaacgcaaacaa---gaccatagtgggggattc
>gnl|SRA|SRR1171870.119965346.1:1-42 HWI-ST967_77:2:2102:13975:164624.
cagtagcttacagaacgcaaacaa---gaccatagtgggggattc
>_R_gnl|SRA|SRR1171870.119714612.1:2-43 HWI-ST967_77:2:2102:10950:155050.
cagtagcttacagaacgcaaacaa---gaccatagtgggggattc
>_R_gnl|SRA|SRR1171870.119526809.1:43-84 HWI-ST967_77:2:2102:18325:147850.
cagtagcttacagaacgcaaacaa---gaccatagtgggggattc
>_R_gnl|SRA|SRR1171870.119409026.1:18-59 HWI-ST967_77:2:2102:16292:143469.
cagtagcttacagaacgcaaacaa---gaccatagtgggggattc
>gnl|SRA|SRR1171870.118829001.1:30-71 HWI-ST967_77:2:2102:4377:121136.
cagtagcttacagaacgcaaacaa---gaccatagtgggggattc
>gnl|SRA|SRR1171870.118629985.1:5-46 HWI-ST967_77:2:2102:11391:113426.
cagtagcttacagaacgcaaacaa---gaccatagtgggggattc
>_R_gnl|SRA|SRR1171870.117868376.2:8-49 HWI-ST967_77:2:2102:6764:83955.
cagtagcttacagaacgcaaacaa---gaccatagtgggggattc
>_R_gnl|SRA|SRR1171870.117832902.2:30-71 HWI-ST967_77:2:2102:16976:82296.
cagtagcttacagaacgcaaacaa---gaccatagtgggggattc
>_R_gnl|SRA|SRR1171870.117819223.2:44-85 HWI-ST967_77:2:2102:14312:81924.
cagtagcttacagaacgcaaacaa---gaccatagtgggggattc
>gnl|SRA|SRR1171870.117069943.2:57-98 HWI-ST967_77:2:2102:1984:53485.
cagtagcttacagaacgcaaacaa---gaccatagtgggggattc
>_R_gnl|SRA|SRR1171870.117069943.1:28-69 HWI-ST967_77:2:2102:1984:53485.
cagtagcttacagaacgcaaacaa---gaccatagtgggggattc
>gnl|SRA|SRR1171870.116523908.2:22-63 HWI-ST967_77:2:2102:21385:32261.
cagtagcttacagaacgcaaacaa---gaccatagtgggggattc
>_R_gnl|SRA|SRR1171870.116513819.1:28-69 HWI-ST967_77:2:2102:10296:32224.

cagtagcttacagaacgcaaacaa---gaccatagtgggggattc
>gnl|SRA|SRR1171870.115023088.2:57-98 HWI-ST967_77:2:2101:12919:173415.
cagtagcttacagaacgcaaacaa---gaccatagtgggggattc
>_R_gnl|SRA|SRR1171870.113934912.1:14-55 HWI-ST967_77:2:2101:3932:131412.
cagtagcttacagaacgcaaacaa---gaccatagtgggggattc
>_R_gnl|SRA|SRR1171870.111953481.2:28-69 HWI-ST967_77:2:2101:14778:53639.
cagtagcttacagaacgcaaacaa---gaccatagtgggggattc
>_R_gnl|SRA|SRR1171870.111823588.1:16-57 HWI-ST967_77:2:2101:7458:48737.
cagtagcttacagaacgcaaacaa---gaccatagtgggggattc
>gnl|SRA|SRR1171870.110770199.2:44-85 HWI-ST967_77:2:2101:8382:7220.
cagtagcttacagaacgcaaacaa---gaccatagtgggggattc
>_R_gnl|SRA|SRR1171870.110770199.1:31-72 HWI-ST967_77:2:2101:8382:7220.
cagtagcttacagaacgcaaacaa---gaccatagtgggggattc
>gnl|SRA|SRR1171870.109679954.1:3-44 HWI-ST967_77:2:1308:14225:24305.
cagtagcttacagaacgcaaacaa---gaccatagtgggggattc
>_R_gnl|SRA|SRR1171870.109349042.1:18-59 HWI-ST967_77:2:1308:2456:6476.
cagtagcttacagaacgcaaacaa---gaccatagtgggggattc
>_R_gnl|SRA|SRR1171870.108137699.1:56-97 HWI-ST967_77:2:1307:3886:126410.
cagtagcttacagaacgcaaacaa---gaccatagtgggggattc
>gnl|SRA|SRR1171870.107368071.1:49-90 HWI-ST967_77:2:1307:16675:79914.
cagtagcttacagaacgcaaacaa---gaccatagtgggggattc
>_R_gnl|SRA|SRR1171870.104090745.1:18-59 HWI-ST967_77:2:1306:2765:132320.
cagtagcttacagaacgcaaacaa---gaccatagtgggggattc
>_R_gnl|SRA|SRR1171870.102813751.2:56-97 HWI-ST967_77:2:1306:4859:85927.
cagtagcttacagaacgcaaacaa---gaccatagtgggggattc
>_R_gnl|SRA|SRR1171870.101275254.1:6-47 HWI-ST967_77:2:1306:9849:31200.
cagtagcttacagaacgcaaacaa---gaccatagtgggggattc
>_R_gnl|SRA|SRR1171870.98251771.1:27-68 HWI-ST967_77:2:1305:4750:123582.
cagtagcttacagaacgcaaacaa---gaccatagtgggggattc
>_R_gnl|SRA|SRR1171870.97232829.1:5-46 HWI-ST967_77:2:1305:4981:86043.
cagtagcttacagaacgcaaacaa---gaccatagtgggggattc
>gnl|SRA|SRR1171870.97073470.2:47-88 HWI-ST967_77:2:1305:15167:79842.
cagtagcttacagaacgcaaacaa---gaccatagtgggggattc
>_R_gnl|SRA|SRR1171870.96785362.1:51-92 HWI-ST967_77:2:1305:12619:69448.
cagtagcttacagaacgcaaacaa---gaccatagtgggggattc
>_R_gnl|SRA|SRR1171870.95868151.2:35-76 HWI-ST967_77:2:1305:11175:37089.
cagtagcttacagaacgcaaacaa---gaccatagtgggggattc
>gnl|SRA|SRR1171870.95864054.1:19-60 HWI-ST967_77:2:1305:19306:36850.
cagtagcttacagaacgcaaacaa---gaccatagtgggggattc
>_R_gnl|SRA|SRR1171870.95658312.2:57-98 HWI-ST967_77:2:1305:4328:29803.
cagtagcttacagaacgcaaacaa---gaccatagtgggggattc
>_R_gnl|SRA|SRR1171870.95445156.2:41-82 HWI-ST967_77:2:1305:10418:22328.
cagtagcttacagaacgcaaacaa---gaccatagtgggggattc
>_R_gnl|SRA|SRR1171870.94455370.2:25-66 HWI-ST967_77:2:1304:19361:186311.
cagtagcttacagaacgcaaacaa---gaccatagtgggggattc
>gnl|SRA|SRR1171870.92441231.1:50-91 HWI-ST967_77:2:1304:15331:115710.
cagtagcttacagaacgcaaacaa---gaccatagtgggggattc
>_R_gnl|SRA|SRR1171870.91322494.1:53-94 HWI-ST967_77:2:1304:11817:75552.
cagtagcttacagaacgcaaacaa---gaccatagtgggggattc
>gnl|SRA|SRR1171870.91144959.1:30-71 HWI-ST967_77:2:1304:2302:69456.
cagtagcttacagaacgcaaacaa---gaccatagtgggggattc

>gnl|SRA|SRR1171870.89614722.2:52-93 HWI-ST967_77:2:1304:6132:15156.
cagtagcttacagaacgcaaacaa---gaccatagtgggggattc
>_R_gnl|SRA|SRR1171870.89614722.1:40-81 HWI-ST967_77:2:1304:6132:15156.
cagtagcttacagaacgcaaacaa---gaccatagtgggggattc
>gnl|SRA|SRR1171870.88320825.2:38-79 HWI-ST967_77:2:1303:3866:167727.
cagtagcttacagaacgcaaacaa---gaccatagtgggggattc
>_R_gnl|SRA|SRR1171870.87813442.1:25-66 HWI-ST967_77:2:1303:18943:149446.
cagtagcttacagaacgcaaacaa---gaccatagtgggggattc
>gnl|SRA|SRR1171870.86124640.2:53-94 HWI-ST967_77:2:1303:14353:88544.
cagtagcttacagaacgcaaacaa---gaccatagtgggggattc
>_R_gnl|SRA|SRR1171870.86124640.1:24-65 HWI-ST967_77:2:1303:14353:88544.
cagtagcttacagaacgcaaacaa---gaccatagtgggggattc
>_R_gnl|SRA|SRR1171870.85799972.2:32-73 HWI-ST967_77:2:1303:3551:76929.
cagtagcttacagaacgcaaacaa---gaccatagtgggggattc
>gnl|SRA|SRR1171870.85799972.1:52-93 HWI-ST967_77:2:1303:3551:76929.
cagtagcttacagaacgcaaacaa---gaccatagtgggggattc
>_R_gnl|SRA|SRR1171870.85559008.2:16-57 HWI-ST967_77:2:1303:3406:68194.
cagtagcttacagaacgcaaacaa---gaccatagtgggggattc
>gnl|SRA|SRR1171870.84384294.2:34-75 HWI-ST967_77:2:1303:7428:25603.
cagtagcttacagaacgcaaacaa---gaccatagtgggggattc
>_R_gnl|SRA|SRR1171870.84384294.1:43-84 HWI-ST967_77:2:1303:7428:25603.
cagtagcttacagaacgcaaacaa---gaccatagtgggggattc
>gnl|SRA|SRR1171870.83164218.1:14-55 HWI-ST967_77:2:1302:12555:179931.
cagtagcttacagaacgcaaacaa---gaccatagtgggggattc
>gnl|SRA|SRR1171870.82763169.2:50-91 HWI-ST967_77:2:1302:19591:165101.
cagtagcttacagaacgcaaacaa---gaccatagtgggggattc
>_R_gnl|SRA|SRR1171870.82497201.2:24-65 HWI-ST967_77:2:1302:9180:155434.
cagtagcttacagaacgcaaacaa---gaccatagtgggggattc
>_R_gnl|SRA|SRR1171870.81838563.2:2-43 HWI-ST967_77:2:1302:17439:129853.
cagtagcttacagaacgcaaacaa---gaccatagtgggggattc
>_R_gnl|SRA|SRR1171870.80406613.1:58-99 HWI-ST967_77:2:1302:12919:74030.
cagtagcttacagaacgcaaacaa---gaccatagtgggggattc
>_R_gnl|SRA|SRR1171870.80187396.1:37-78 HWI-ST967_77:2:1302:2421:65976.
cagtagcttacagaacgcaaacaa---gaccatagtgggggattc
>_R_gnl|SRA|SRR1171870.79469834.1:33-74 HWI-ST967_77:2:1302:15587:38756.
cagtagcttacagaacgcaaacaa---gaccatagtgggggattc
>gnl|SRA|SRR1171870.79436261.1:1-42 HWI-ST967_77:2:1302:14881:37606.
cagtagcttacagaacgcaaacaa---gaccatagtgggggattc
>_R_gnl|SRA|SRR1171870.79209496.1:41-82 HWI-ST967_77:2:1302:16016:29199.
cagtagcttacagaacgcaaacaa---gaccatagtgggggattc
>_R_gnl|SRA|SRR1171870.79198189.1:46-87 HWI-ST967_77:2:1302:3118:28837.
cagtagcttacagaacgcaaacaa---gaccatagtgggggattc
>gnl|SRA|SRR1171870.78946979.1:1-42 HWI-ST967_77:2:1302:14972:19345.
cagtagcttacagaacgcaaacaa---gaccatagtgggggattc
>gnl|SRA|SRR1171870.77803337.1:39-80 HWI-ST967_77:2:1301:8892:175241.
cagtagcttacagaacgcaaacaa---gaccatagtgggggattc
>_R_gnl|SRA|SRR1171870.76797583.2:28-69 HWI-ST967_77:2:1301:11139:137247.
cagtagcttacagaacgcaaacaa---gaccatagtgggggattc
>_R_gnl|SRA|SRR1171870.76278630.1:8-49 HWI-ST967_77:2:1301:11911:117339.
cagtagcttacagaacgcaaacaa---gaccatagtgggggattc
>gnl|SRA|SRR1171870.76203663.2:46-87 HWI-ST967_77:2:1301:2616:114640.

cagtagcttacagaacgcaaacaa---gaccatagtgggggattc
>_R_gnl|SRA|SRR1171870.76203663.1:25-66 HWI-ST967_77:2:1301:2616:114640.
cagtagcttacagaacgcaaacaa---gaccatagtgggggattc
>_R_gnl|SRA|SRR1171870.76147807.2:13-54 HWI-ST967_77:2:1301:12239:112365.
cagtagcttacagaacgcaaacaa---gaccatagtgggggattc
>gnl|SRA|SRR1171870.75940051.1:40-81 HWI-ST967_77:2:1301:16988:104320.
cagtagcttacagaacgcaaacaa---gaccatagtgggggattc
>gnl|SRA|SRR1171870.74870968.1:48-89 HWI-ST967_77:2:1301:12763:63457.
cagtagcttacagaacgcaaacaa---gaccatagtgggggattc
>_R_gnl|SRA|SRR1171870.74816829.1:19-60 HWI-ST967_77:2:1301:8381:61404.
cagtagcttacagaacgcaaacaa---gaccatagtgggggattc
>_R_gnl|SRA|SRR1171870.71189192.1:52-93 HWI-ST967_77:2:1207:6487:111254.
cagtagcttacagaacgcaaacaa---gaccatagtgggggattc
>_R_gnl|SRA|SRR1171870.68618995.1:50-91 HWI-ST967_77:2:1206:19030:145128.
cagtagcttacagaacgcaaacaa---gaccatagtgggggattc
>gnl|SRA|SRR1171870.68464449.2:45-86 HWI-ST967_77:2:1206:4816:139668.
cagtagcttacagaacgcaaacaa---gaccatagtgggggattc
>_R_gnl|SRA|SRR1171870.68464449.1:46-87 HWI-ST967_77:2:1206:4816:139668.
cagtagcttacagaacgcaaacaa---gaccatagtgggggattc
>_R_gnl|SRA|SRR1171870.66719409.1:30-71 HWI-ST967_77:2:1206:10309:75064.
cagtagcttacagaacgcaaacaa---gaccatagtgggggattc
>gnl|SRA|SRR1171870.66710869.2:58-99 HWI-ST967_77:2:1206:6149:74770.
cagtagcttacagaacgcaaacaa---gaccatagtgggggattc
>_R_gnl|SRA|SRR1171870.66710869.1:36-77 HWI-ST967_77:2:1206:6149:74770.
cagtagcttacagaacgcaaacaa---gaccatagtgggggattc
>gnl|SRA|SRR1171870.66615096.2:20-61 HWI-ST967_77:2:1206:5673:71351.
cagtagcttacagaacgcaaacaa---gaccatagtgggggattc
>gnl|SRA|SRR1171870.66548084.2:54-95 HWI-ST967_77:2:1206:6981:68799.
cagtagcttacagaacgcaaacaa---gaccatagtgggggattc
>_R_gnl|SRA|SRR1171870.66548084.1:12-53 HWI-ST967_77:2:1206:6981:68799.
cagtagcttacagaacgcaaacaa---gaccatagtgggggattc
>_R_gnl|SRA|SRR1171870.65188690.1:31-72 HWI-ST967_77:2:1206:14345:20032.
cagtagcttacagaacgcaaacaa---gaccatagtgggggattc
>_R_gnl|SRA|SRR1171870.64786712.2:43-84 HWI-ST967_77:2:1206:21210:5693.
cagtagcttacagaacgcaaacaa---gaccatagtgggggattc
>gnl|SRA|SRR1171870.64786712.1:50-91 HWI-ST967_77:2:1206:21210:5693.
cagtagcttacagaacgcaaacaa---gaccatagtgggggattc
>gnl|SRA|SRR1171870.64329318.1:22-63 HWI-ST967_77:2:1205:19743:187556.
cagtagcttacagaacgcaaacaa---gaccatagtgggggattc
>_R_gnl|SRA|SRR1171870.64186593.2:42-83 HWI-ST967_77:2:1205:12381:182659.
cagtagcttacagaacgcaaacaa---gaccatagtgggggattc
>gnl|SRA|SRR1171870.64186593.1:13-54 HWI-ST967_77:2:1205:12381:182659.
cagtagcttacagaacgcaaacaa---gaccatagtgggggattc
>gnl|SRA|SRR1171870.63520714.2:20-61 HWI-ST967_77:2:1205:17198:158539.
cagtagcttacagaacgcaaacaa---gaccatagtgggggattc
>_R_gnl|SRA|SRR1171870.62580343.1:3-44 HWI-ST967_77:2:1205:14876:124923.
cagtagcttacagaacgcaaacaa---gaccatagtgggggattc
>_R_gnl|SRA|SRR1171870.62036211.1:2-43 HWI-ST967_77:2:1205:13937:105408.
cagtagcttacagaacgcaaacaa---gaccatagtgggggattc
>_R_gnl|SRA|SRR1171870.61613125.1:23-64 HWI-ST967_77:2:1205:18840:89733.
cagtagcttacagaacgcaaacaa---gaccatagtgggggattc

>gnl|SRA|SRR1171870.61424044.2:34-75 HWI-ST967_77:2:1205:10053:82598.
cagtagcttacagaacgcaaacaa---gaccatagtgggggattc
>_R_gnl|SRA|SRR1171870.61336656.2:41-82 HWI-ST967_77:2:1205:9303:79316.
cagtagcttacagaacgcaaacaa---gaccatagtgggggattc
>_R_gnl|SRA|SRR1171870.60965497.1:4-45 HWI-ST967_77:2:1205:10984:65808.
cagtagcttacagaacgcaaacaa---gaccatagtgggggattc
>gnl|SRA|SRR1171870.59064262.2:54-95 HWI-ST967_77:2:1204:14118:196724.
cagtagcttacagaacgcaaacaa---gaccatagtgggggattc
>_R_gnl|SRA|SRR1171870.59064262.1:12-53 HWI-ST967_77:2:1204:14118:196724.
cagtagcttacagaacgcaaacaa---gaccatagtgggggattc
>gnl|SRA|SRR1171870.55337139.2:50-91 HWI-ST967_77:2:1204:8462:62944.
cagtagcttacagaacgcaaacaa---gaccatagtgggggattc
>gnl|SRA|SRR1171870.55246219.2:47-88 HWI-ST967_77:2:1204:7273:59592.
cagtagcttacagaacgcaaacaa---gaccatagtgggggattc
>gnl|SRA|SRR1171870.55031254.2:52-93 HWI-ST967_77:2:1204:3543:51869.
cagtagcttacagaacgcaaacaa---gaccatagtgggggattc
>_R_gnl|SRA|SRR1171870.54568742.1:23-64 HWI-ST967_77:2:1204:18454:35124.
cagtagcttacagaacgcaaacaa---gaccatagtgggggattc
>_R_gnl|SRA|SRR1171870.52962243.1:37-78 HWI-ST967_77:2:1203:17710:176334.
cagtagcttacagaacgcaaacaa---gaccatagtgggggattc
>_R_gnl|SRA|SRR1171870.52499875.2:27-68 HWI-ST967_77:2:1203:5270:160130.
cagtagcttacagaacgcaaacaa---gaccatagtgggggattc
>_R_gnl|SRA|SRR1171870.51494398.2:8-49 HWI-ST967_77:2:1203:21151:124018.
cagtagcttacagaacgcaaacaa---gaccatagtgggggattc
>gnl|SRA|SRR1171870.49788128.2:47-88 HWI-ST967_77:2:1203:8278:62693.
cagtagcttacagaacgcaaacaa---gaccatagtgggggattc
>_R_gnl|SRA|SRR1171870.49788128.1:36-77 HWI-ST967_77:2:1203:8278:62693.
cagtagcttacagaacgcaaacaa---gaccatagtgggggattc
>_R_gnl|SRA|SRR1171870.49481787.1:31-72 HWI-ST967_77:2:1203:19761:51373.
cagtagcttacagaacgcaaacaa---gaccatagtgggggattc
>_R_gnl|SRA|SRR1171870.48715089.2:13-54 HWI-ST967_77:2:1203:11946:23751.
cagtagcttacagaacgcaaacaa---gaccatagtgggggattc
>_R_gnl|SRA|SRR1171870.48068650.2:38-79 HWI-ST967_77:2:1202:18555:198725.
cagtagcttacagaacgcaaacaa---gaccatagtgggggattc
>_R_gnl|SRA|SRR1171870.47673565.1:33-74 HWI-ST967_77:2:1202:4948:184625.
cagtagcttacagaacgcaaacaa---gaccatagtgggggattc
>_R_gnl|SRA|SRR1171870.47642898.1:53-94 HWI-ST967_77:2:1202:16103:183299.
cagtagcttacagaacgcaaacaa---gaccatagtgggggattc
>_R_gnl|SRA|SRR1171870.47429366.2:51-92 HWI-ST967_77:2:1202:20660:175694.
cagtagcttacagaacgcaaacaa---gaccatagtgggggattc
>_R_gnl|SRA|SRR1171870.47259541.2:36-77 HWI-ST967_77:2:1202:6918:169522.
cagtagcttacagaacgcaaacaa---gaccatagtgggggattc
>_R_gnl|SRA|SRR1171870.45159149.1:18-59 HWI-ST967_77:2:1202:6382:91026.
cagtagcttacagaacgcaaacaa---gaccatagtgggggattc
>_R_gnl|SRA|SRR1171870.44430488.2:24-65 HWI-ST967_77:2:1202:18220:63612.
cagtagcttacagaacgcaaacaa---gaccatagtgggggattc
>gnl|SRA|SRR1171870.44079028.1:55-96 HWI-ST967_77:2:1202:17392:50683.
cagtagcttacagaacgcaaacaa---gaccatagtgggggattc
>gnl|SRA|SRR1171870.43239947.2:57-98 HWI-ST967_77:2:1202:5542:19775.
cagtagcttacagaacgcaaacaa---gaccatagtgggggattc
>_R_gnl|SRA|SRR1171870.43239947.1:46-87 HWI-ST967_77:2:1202:5542:19775.

cagtagcttacagaacgcaaacaa---gaccatagtgggggattc
>_R_gnl|SRA|SRR1171870.42532724.1:26-67 HWI-ST967_77:2:1201:21360:191865.
cagtagcttacagaacgcaaacaa---gaccatagtgggggattc
>_R_gnl|SRA|SRR1171870.41305310.1:42-83 HWI-ST967_77:2:1201:11250:145955.
cagtagcttacagaacgcaaacaa---gaccatagtgggggattc
>_R_gnl|SRA|SRR1171870.41159378.1:25-66 HWI-ST967_77:2:1201:8254:140344.
cagtagcttacagaacgcaaacaa---gaccatagtgggggattc
>gnl|SRA|SRR1171870.39921623.1:57-98 HWI-ST967_77:2:1201:12262:92787.
cagtagcttacagaacgcaaacaa---gaccatagtgggggattc
>gnl|SRA|SRR1171870.39737750.2:6-47 HWI-ST967_77:2:1201:9036:85821.
cagtagcttacagaacgcaaacaa---gaccatagtgggggattc
>_R_gnl|SRA|SRR1171870.38897647.1:8-49 HWI-ST967_77:2:1201:9704:53957.
cagtagcttacagaacgcaaacaa---gaccatagtgggggattc
>_R_gnl|SRA|SRR1171870.38364770.1:53-94 HWI-ST967_77:2:1201:3430:33621.
cagtagcttacagaacgcaaacaa---gaccatagtgggggattc
>_R_gnl|SRA|SRR1171870.37636341.2:30-71 HWI-ST967_77:2:1201:5128:5665.
cagtagcttacagaacgcaaacaa---gaccatagtgggggattc
>gnl|SRA|SRR1171870.36944316.1:56-97 HWI-ST967_77:2:1108:2510:86169.
cagtagcttacagaacgcaaacaa---gaccatagtgggggattc
>_R_gnl|SRA|SRR1171870.34695540.1:35-76 HWI-ST967_77:2:1107:3092:115627.
cagtagcttacagaacgcaaacaa---gaccatagtgggggattc
>_R_gnl|SRA|SRR1171870.32860903.1:17-58 HWI-ST967_77:2:1107:9964:33163.
cagtagcttacagaacgcaaacaa---gaccatagtgggggattc
>gnl|SRA|SRR1171870.32440559.1:50-91 HWI-ST967_77:2:1107:9359:14943.
cagtagcttacagaacgcaaacaa---gaccatagtgggggattc
>_R_gnl|SRA|SRR1171870.32190490.1:59-100 HWI-ST967_77:2:1107:12028:4042.
cagtagcttacagaacgcaaacaa---gaccatagtgggggattc
>_R_gnl|SRA|SRR1171870.32078586.1:36-77 HWI-ST967_77:2:1106:17264:197993.
cagtagcttacagaacgcaaacaa---gaccatagtgggggattc
>gnl|SRA|SRR1171870.32056669.1:46-87 HWI-ST967_77:2:1106:13915:197010.
cagtagcttacagaacgcaaacaa---gaccatagtgggggattc
>_R_gnl|SRA|SRR1171870.30028368.1:32-73 HWI-ST967_77:2:1106:14031:124003.
cagtagcttacagaacgcaaacaa---gaccatagtgggggattc
>_R_gnl|SRA|SRR1171870.29961077.1:26-67 HWI-ST967_77:2:1106:19800:121743.
cagtagcttacagaacgcaaacaa---gaccatagtgggggattc
>_R_gnl|SRA|SRR1171870.29786586.1:32-73 HWI-ST967_77:2:1106:15672:115265.
cagtagcttacagaacgcaaacaa---gaccatagtgggggattc
>gnl|SRA|SRR1171870.29060989.1:1-42 HWI-ST967_77:2:1106:2847:89350.
cagtagcttacagaacgcaaacaa---gaccatagtgggggattc
>gnl|SRA|SRR1171870.28687537.2:50-91 HWI-ST967_77:2:1106:9812:75881.
cagtagcttacagaacgcaaacaa---gaccatagtgggggattc
>_R_gnl|SRA|SRR1171870.28687537.1:31-72 HWI-ST967_77:2:1106:9812:75881.
cagtagcttacagaacgcaaacaa---gaccatagtgggggattc
>_R_gnl|SRA|SRR1171870.28294188.1:30-71 HWI-ST967_77:2:1106:14613:61564.
cagtagcttacagaacgcaaacaa---gaccatagtgggggattc
>gnl|SRA|SRR1171870.27613232.1:1-42 HWI-ST967_77:2:1106:10070:37594.
cagtagcttacagaacgcaaacaa---gaccatagtgggggattc
>gnl|SRA|SRR1171870.27408472.1:30-71 HWI-ST967_77:2:1106:16837:30289.
cagtagcttacagaacgcaaacaa---gaccatagtgggggattc
>gnl|SRA|SRR1171870.27098930.1:17-58 HWI-ST967_77:2:1106:10371:19718.
cagtagcttacagaacgcaaacaa---gaccatagtgggggattc

>gnl|SRA|SRR1171870.26985339.2:6-47 HWI-ST967_77:2:1106:11639:15728.
cagtagcttacagaacgcaaacaa---gaccatagtgggggattc
>_R_gnl|SRA|SRR1171870.26895166.1:11-52 HWI-ST967_77:2:1106:17525:12417.
cagtagcttacagaacgcaaacaa---gaccatagtgggggattc
>gnl|SRA|SRR1171870.25617232.2:57-98 HWI-ST967_77:2:1105:2619:165980.
cagtagcttacagaacgcaaacaa---gaccatagtgggggattc
>_R_gnl|SRA|SRR1171870.25048260.2:23-64 HWI-ST967_77:2:1105:3096:145509.
cagtagcttacagaacgcaaacaa---gaccatagtgggggattc
>_R_gnl|SRA|SRR1171870.24793270.2:30-71 HWI-ST967_77:2:1105:11971:136388.
cagtagcttacagaacgcaaacaa---gaccatagtgggggattc
>gnl|SRA|SRR1171870.23781052.2:20-61 HWI-ST967_77:2:1105:5852:99867.
cagtagcttacagaacgcaaacaa---gaccatagtgggggattc
>_R_gnl|SRA|SRR1171870.23569782.1:17-58 HWI-ST967_77:2:1105:1801:92326.
cagtagcttacagaacgcaaacaa---gaccatagtgggggattc
>gnl|SRA|SRR1171870.23160943.2:2-43 HWI-ST967_77:2:1105:8227:77564.
cagtagcttacagaacgcaaacaa---gaccatagtgggggattc
>gnl|SRA|SRR1171870.22332000.2:40-81 HWI-ST967_77:2:1105:2976:47988.
cagtagcttacagaacgcaaacaa---gaccatagtgggggattc
>gnl|SRA|SRR1171870.21407734.1:51-92 HWI-ST967_77:2:1105:13291:14763.
cagtagcttacagaacgcaaacaa---gaccatagtgggggattc
>_R_gnl|SRA|SRR1171870.20323195.1:41-82 HWI-ST967_77:2:1104:4815:173737.
cagtagcttacagaacgcaaacaa---gaccatagtgggggattc
>_R_gnl|SRA|SRR1171870.19218320.1:3-44 HWI-ST967_77:2:1104:7705:131868.
cagtagcttacagaacgcaaacaa---gaccatagtgggggattc
>_R_gnl|SRA|SRR1171870.19192627.2:43-84 HWI-ST967_77:2:1104:10062:130759.
cagtagcttacagaacgcaaacaa---gaccatagtgggggattc
>gnl|SRA|SRR1171870.19192627.1:30-71 HWI-ST967_77:2:1104:10062:130759.
cagtagcttacagaacgcaaacaa---gaccatagtgggggattc
>gnl|SRA|SRR1171870.18991850.2:20-61 HWI-ST967_77:2:1104:16832:123152.
cagtagcttacagaacgcaaacaa---gaccatagtgggggattc
>gnl|SRA|SRR1171870.18780837.2:40-81 HWI-ST967_77:2:1104:15680:115019.
cagtagcttacagaacgcaaacaa---gaccatagtgggggattc
>gnl|SRA|SRR1171870.18291537.1:10-51 HWI-ST967_77:2:1104:9740:96525.
cagtagcttacagaacgcaaacaa---gaccatagtgggggattc
>_R_gnl|SRA|SRR1171870.17424553.1:3-44 HWI-ST967_77:2:1104:10279:64488.
cagtagcttacagaacgcaaacaa---gaccatagtgggggattc
>gnl|SRA|SRR1171870.16380370.1:45-86 HWI-ST967_77:2:1104:10806:26378.
cagtagcttacagaacgcaaacaa---gaccatagtgggggattc
>_R_gnl|SRA|SRR1171870.15423432.1:50-91 HWI-ST967_77:2:1103:7721:190071.
cagtagcttacagaacgcaaacaa---gaccatagtgggggattc
>_R_gnl|SRA|SRR1171870.15267027.1:25-66 HWI-ST967_77:2:1103:6326:184266.
cagtagcttacagaacgcaaacaa---gaccatagtgggggattc
>_R_gnl|SRA|SRR1171870.15115721.2:49-90 HWI-ST967_77:2:1103:16415:178519.
cagtagcttacagaacgcaaacaa---gaccatagtgggggattc
>gnl|SRA|SRR1171870.14736878.2:57-98 HWI-ST967_77:2:1103:13030:164657.
cagtagcttacagaacgcaaacaa---gaccatagtgggggattc
>_R_gnl|SRA|SRR1171870.14736878.1:53-94 HWI-ST967_77:2:1103:13030:164657.
cagtagcttacagaacgcaaacaa---gaccatagtgggggattc
>gnl|SRA|SRR1171870.13315723.1:56-97 HWI-ST967_77:2:1103:4672:111776.
cagtagcttacagaacgcaaacaa---gaccatagtgggggattc
>_R_gnl|SRA|SRR1171870.11707830.2:16-57 HWI-ST967_77:2:1103:15694:51865.

cagtagcttacagaacgcaaacaa---gaccatagtgggggattc
>_R_gnl|SRA|SRR1171870.10343092.2:17-58 HWI-ST967_77:2:1102:10791:199609.
cagtagcttacagaacgcaaacaa---gaccatagtgggggattc
>gnl|SRA|SRR1171870.10343092.1:41-82 HWI-ST967_77:2:1102:10791:199609.
cagtagcttacagaacgcaaacaa---gaccatagtgggggattc
>_R_gnl|SRA|SRR1171870.10204609.2:36-77 HWI-ST967_77:2:1102:13558:194379.
cagtagcttacagaacgcaaacaa---gaccatagtgggggattc
>_R_gnl|SRA|SRR1171870.9962863.1:12-53 HWI-ST967_77:2:1102:2936:185250.
cagtagcttacagaacgcaaacaa---gaccatagtgggggattc
>_R_gnl|SRA|SRR1171870.8832758.1:32-73 HWI-ST967_77:2:1102:13252:141603.
cagtagcttacagaacgcaaacaa---gaccatagtgggggattc
>_R_gnl|SRA|SRR1171870.8381776.1:23-64 HWI-ST967_77:2:1102:5823:123800.
cagtagcttacagaacgcaaacaa---gaccatagtgggggattc
>_R_gnl|SRA|SRR1171870.7901730.2:5-46 HWI-ST967_77:2:1102:2412:105049.
cagtagcttacagaacgcaaacaa---gaccatagtgggggattc
>_R_gnl|SRA|SRR1171870.7424683.2:27-68 HWI-ST967_77:2:1102:18211:86282.
cagtagcttacagaacgcaaacaa---gaccatagtgggggattc
>_R_gnl|SRA|SRR1171870.6957244.1:27-68 HWI-ST967_77:2:1102:13808:68435.
cagtagcttacagaacgcaaacaa---gaccatagtgggggattc
>gnl|SRA|SRR1171870.6947663.2:9-50 HWI-ST967_77:2:1102:5416:68167.
cagtagcttacagaacgcaaacaa---gaccatagtgggggattc
>gnl|SRA|SRR1171870.6946151.2:57-98 HWI-ST967_77:2:1102:20551:67852.
cagtagcttacagaacgcaaacaa---gaccatagtgggggattc
>_R_gnl|SRA|SRR1171870.4788066.1:2-43 HWI-ST967_77:2:1101:13490:184736.
cagtagcttacagaacgcaaacaa---gaccatagtgggggattc
>_R_gnl|SRA|SRR1171870.4742488.1:21-62 HWI-ST967_77:2:1101:16992:182893.
cagtagcttacagaacgcaaacaa---gaccatagtgggggattc
>_R_gnl|SRA|SRR1171870.4629821.1:21-62 HWI-ST967_77:2:1101:19875:178569.
cagtagcttacagaacgcaaacaa---gaccatagtgggggattc
>_R_gnl|SRA|SRR1171870.3247638.2:52-93 HWI-ST967_77:2:1101:9313:126528.
cagtagcttacagaacgcaaacaa---gaccatagtgggggattc
>_R_gnl|SRA|SRR1171870.3134235.1:24-65 HWI-ST967_77:2:1101:4366:122276.
cagtagcttacagaacgcaaacaa---gaccatagtgggggattc
>_R_gnl|SRA|SRR1171870.2721942.2:50-91 HWI-ST967_77:2:1101:4061:106723.
cagtagcttacagaacgcaaacaa---gaccatagtgggggattc
>gnl|SRA|SRR1171870.2721942.1:51-92 HWI-ST967_77:2:1101:4061:106723.
cagtagcttacagaacgcaaacaa---gaccatagtgggggattc
>_R_gnl|SRA|SRR1171870.1135010.1:14-55 HWI-ST967_77:2:1101:2452:45821.
cagtagcttacagaacgcaaacaa---gaccatagtgggggattc
>gnl|SRA|SRR1171870.590494.2:43-84 HWI-ST967_77:2:1101:8962:24914.
cagtagcttacagaacgcaaacaa---gaccatagtgggggattc
>_R_gnl|SRA|SRR1171870.71929185.2:48-89 HWI-ST967_77:2:1208:7871:8292.
cagtagcttacagaacgcaaacaa---gaccatngtnggggattc
>gnl|SRA|SRR1171870.110576660.1:29-70 HWI-ST967_77:2:1308:7101:182588.
cagtagcttacagaacgcaaacaa---gaccattgtgggggattc
>_R_gnl|SRA|SRR1171870.196729546.1:36-77 HWI-ST967_77:2:2304:17072:143570.
cagtagcttacagaacgcaaacaa---gaccatagtgggggactc
>_R_gnl|SRA|SRR1171870.176271108.1:43-84 HWI-ST967_77:2:2208:2567:45232.
cagtagcttacagaacgcaaacaa---gaccatagtgggagattc
>_R_gnl|SRA|SRR1171870.142379648.1:43-84 HWI-ST967_77:2:2106:6644:199171.
cagtagcttacagaacgcaaacaa---ggccatagtgggggattc

>_R_gnl|SRA|SRR1171870.98033608.2:57-98 HWI-ST967_77:2:1305:7198:115931.
cagtagcttacagaacgcaaacaa---gaccatagtggggaattc
>_R_gnl|SRA|SRR1171870.46166025.1:29-70 HWI-ST967_77:2:1202:19441:128939.
cagtagcctacagaacgcaaacaa---gaccatagtgggggattc
>gnl|SRA|SRR1171870.30028368.2:57-98 HWI-ST967_77:2:1106:14031:124003.
cagtagcttacagaacgcaaacaa---gaccatagtggggggttc
>_R_gnl|SRA|SRR1171870.25617232.1:22-63 HWI-ST967_77:2:1105:2619:165980.
cagtagcttacagaacgtaaacaa---gaccatagtgggggattc
>gnl|SRA|SRR1171870.103617968.2:57-98 HWI-ST967_77:2:1306:8332:115097.
cagtagcttacagaacgcaaacaa---gaccatagtgtgggattc
>_R_gnl|SRA|SRR1171870.109325959.2:5-46 HWI-ST967_77:2:1308:3249:5261.
cagtancttacanaacgcaaacnn---gaccgtagtgnggnantc
>_R_gnl|SRA|SRR1171870.38195428.1:53-94 HWI-ST967_77:2:1201:3051:27030.
cagtagcttacagaacgcaaacac---gaccatagtgggggattc
>_R_gnl|SRA|SRR1171870.4099281.2:27-68 HWI-ST967_77:2:1101:8532:158819.
cagtatcttacagaacgcaaacaa---gaccatagtgggggattc
>gnl|SRA|SRR1171870.149490882.2:14-55 HWI-ST967_77:2:2202:1262:20940.
cagtagcttacagaacgaaaacaa---gaccatagtgggggattc
>_R_gnl|SRA|SRR1171870.142995336.1:32-73 HWI-ST967_77:2:2107:6755:79656.
cagtagcatacagaacgcaaacaa---gaccatagtgggggattc
>_R_gnl|SRA|SRR1171870.55031254.1:51-92 HWI-ST967_77:2:1204:3543:51869.
cagtagcatacagaacgcaaacaa---gaccatagtgggggattc
>_R_gnl|SRA|SRR1171870.32440559.2:54-95 HWI-ST967_77:2:1107:9359:14943.
cagcagcttacagaacgcaaacaa---gaccatcgtgggggattc
>gnl|SRA|SRR1171870.179650569.1:57-98 HWI-ST967_77:2:2301:17221:102841.
cagtagcttacagaacgcaaacaa---gaccatagtgggggcttc
>gnl|SRA|SRR1171870.130070616.1:35-76 HWI-ST967_77:2:2104:3931:142410.
cagtagcttacagaacgcaaacaa---gaccaaagtgggggcttc
>gnl|SRA|SRR1171870.142807511.1:56-97 HWI-ST967_77:2:2107:2918:54949.
cagtagcttacagaacgcaaacaa---gaccagagtgggggattc
>_R_gnl|SRA|SRR1171870.74084488.2:36-77 HWI-ST967_77:2:1301:11655:33033.
cagtagcttacagaacgcaaacaa---gaccaaagtgggggattc
>_R_gnl|SRA|SRR1171870.180327725.1:31-72 HWI-ST967_77:2:2301:19380:129440.
cagtagcttacagaacgcaaacaa---gcccatagtgggggattc
>_R_gnl|SRA|SRR1171870.171901016.2:24-65 HWI-ST967_77:2:2206:12534:39880.
cagtagcttacagaacgcaaacaa---gcccatagtgggggattc
>_R_gnl|SRA|SRR1171870.89838854.1:50-91 HWI-ST967_77:2:1304:2165:23104.
cagtagcttacagaacgcaaacaa---gcccatagtgggggattc
>_R_gnl|SRA|SRR1171870.22395852.2:16-57 HWI-ST967_77:2:1105:5742:50178.
cagtagcttacagaacgcaaacaa---gcccatagtgggggattc
>_R_gnl|SRA|SRR1171870.153320180.1:58-99 HWI-ST967_77:2:2202:3127:163679.
cagtagcttacagaacgcaaacaa---gtccatagtgggggattc
>_R_gnl|SRA|SRR1171870.86807360.1:18-59 HWI-ST967_77:2:1303:15131:113475.
cagtagcttacagaacgcaaacaa---gtccatagtgggggattc
>_R_gnl|SRA|SRR1171870.70054719.1:17-58 HWI-ST967_77:2:1206:9986:199337.
cagtagcttacagaacgcaaacaa---gtccatagtgggggattc
>_R_gnl|SRA|SRR1171870.206739321.1:51-92 HWI-ST967_77:2:2306:2001:113988.
cagtagcttacagaacgcaaacaa---gaccatagtgggtgattc
>_R_gnl|SRA|SRR1171870.6248690.2:31-72 HWI-ST967_77:2:1102:19296:41260.
cagtagcttacagaacgcaaacaa---gaccatagtgggtgattc
>_R_gnl|SRA|SRR1171870.22664053.2:26-67 HWI-ST967_77:2:1105:14823:59555.

cagtagctaacagaacgcaaacaa---gaccatagtgggggattc
>gnl|SRA|SRR1171870.70054719.2:56-97 HWI-ST967_77:2:1206:9986:199337.
cagtagctgacagaacgcaaacaa---gaccatagtggggggttc
>_R_gnl|SRA|SRR1171870.204558795.1:20-61 HWI-ST967_77:2:2306:10689:32753.
cagtagcttaaagaacgcaaacaa---gaccatagtgggggattc
>_R_gnl|SRA|SRR1171870.198288473.2:44-85 HWI-ST967_77:2:2304:21242:199204.
cagtagcttacagcacgcaaacaa---gaccatagtgggggattc
>gnl|SRA|SRR1171870.70901329.1:5-46 HWI-ST967_77:2:1207:7239:79804.
cagtcgcttacagaacgcaaacaa---gaccatagtgggggattc
>_R_gnl|SRA|SRR1171870.61072272.1:52-93 HWI-ST967_77:2:1205:18713:69718.
cagtagcttacagaactcaaacaa---gaccatagtgggggattc
>_R_gnl|SRA|SRR1171870.35034497.1:17-58 HWI-ST967_77:2:1107:3453:131678.
cagtaggttacagaacgcaaacaa---gaccatagtgggggattc
>_R_gnl|SRA|SRR1171870.108369655.1:33-74 HWI-ST967_77:2:1307:14821:140907.
cagtagcttacagaacgcaaacaa---gaccatagtggcggattc
>gnl|SRA|SRR1171870.69671865.1:57-98 HWI-ST967_77:2:1206:15044:184544.
cagtagcttacagaacgcaaacaa---gaccatagtggtggattc
>gnl|SRA|SRR1171870.102351169.2:51-92 HWI-ST967_77:2:1306:6963:69148.
cagtagcgtacagaacgcaaacaa---gaccatagtggtggattc
>gnl|SRA|SRR1171870.184188997.2:55-96 HWI-ST967_77:2:2302:20140:78084.
cagtagcttacagaacgccaacaa---gaccatagtgggggattc
>_R_gnl|SRA|SRR1171870.108972363.1:20-61 HWI-ST967_77:2:1307:20697:181085.
cagtagcttacagaacgccaacca---gaccatagtgggggattc
>_R_gnl|SRA|SRR1171870.25332499.1:50-91 HWI-ST967_77:2:1105:14144:155569.
cagtagcttacagaacgccatcaa---gaccatagtgggagattc
>gnl|SRA|SRR1171870.20323195.2:49-90 HWI-ST967_77:2:1104:4815:173737.
caggagcttacagtacgcaagcaa---gaccatagtgggggattc
>_R_gnl|SRA|SRR1171870.1648117.2:53-94 HWI-ST967_77:2:1101:1768:65562.
cagtagctcacagaacgcaaaaaa---gaccaaagtgggggattc
>gnl|SRA|SRR1171870.138314896.2:1-42 HWI-ST967_77:2:2106:6846:48481.
cagtagcttacggaccgcaaacaa---gaccatagtgggggtttc
>_R_gnl|SRA|SRR1171870.129043071.1:28-69 HWI-ST967_77:2:2104:6648:104436.
cagtagcctacagaacgcaaacaa---gaccattgggggggattc
>gnl|SRA|SRR1171870.104722961.2:20-61 HWI-ST967_77:2:1306:8218:155120.
caggatcttacataacgcaaacaa---gaccatagtgggggattc
>gnl|SRA|SRR1171870.1648117.1:44-85 HWI-ST967_77:2:1101:1768:65562.
caggagcttacataacgcaaacaa---taccatagtggcgggttc
>gnl|SRA|SRR1171870.172852583.2:52-93 HWI-ST967_77:2:2206:18747:79217.
cagtagcttacagaacgcacacaa---gaccatggtgtgtgattc
>gnl|SRA|SRR1171870.174455635.2:48-92 HWI-ST967_77:2:2206:9798:150101.
cagtagcttacagaacgcaaacaagaggaccatagtgggggattc
>_R_gnl|SRA|SRR1171870.78532254.1:32-76 HWI-ST967_77:2:1302:8675:4196.
cagtagcttacagaacgcaaacaagaggaccatagtgggggattc
>_R_gnl|SRA|SRR1171870.63776958.2:22-63 HWI-ST967_77:2:1205:15968:167939.
cagtagcttacagaactcaaacaa---gtccacaggggggggttc
>gnl|SRA|SRR1171870.104987552.2:45-86 HWI-ST967_77:2:1306:10625:164613.
cattatcttacagaagtcaaacaa---gaccatagtgggggattc
>_R_gnl|SRA|SRR1171870.189985344.1:60-100 HWI-ST967_77:2:2303:16206:96978.
-agtagcttacagaacgcaaacaa---gaccatagtgggggattc
>_R_gnl|SRA|SRR1171870.101262376.1:60-100 HWI-ST967_77:2:1306:13830:30641.
-agtagcttacagaacgcaaacaa---gaccatagtgggggattc

>_R_gnl|SRA|SRR1171870.75940051.2:60-100 HWI-ST967_77:2:1301:16988:104320.
-agtagcttacagaacgcaaacaa---gaccatagtgggggattc
>_R_gnl|SRA|SRR1171870.60858668.2:60-100 HWI-ST967_77:2:1205:6195:62076.
-agtagcttacagaacgcaaacaa---gaccatagtgggggattc
>_R_gnl|SRA|SRR1171870.43711638.1:49-89 HWI-ST967_77:2:1202:15419:37248.
-agtagcttacagaacgcaaacaa---gaccatagtgggggattc
>_R_gnl|SRA|SRR1171870.11942882.1:35-75 HWI-ST967_77:2:1103:14769:60568.
-agtagcttacagaacgcaaacaa---gaccatagtgggggattc
>gnl|SRA|SRR1171870.12580093.2:57-97 HWI-ST967_77:2:1103:12445:84319.
-agcagcttacagaacgcaaacaa---gaccatagtgggggattc
>_R_gnl|SRA|SRR1171870.103855064.1:60-100 HWI-ST967_77:2:1306:13666:123637.
-agtagcttacagaacgcaaacaa---gaccatagtgggtgattc
>_R_gnl|SRA|SRR1171870.99531668.2:60-100 HWI-ST967_77:2:1305:15245:168429.
-agtagcttacagaacgcaaacaa---gacaatagtgggggattc
>_R_gnl|SRA|SRR1171870.74870968.2:43-83 HWI-ST967_77:2:1301:12763:63457.
-agtagttgacagaacgcaaacaa---gacaatagtgggggattc
>_R_gnl|SRA|SRR1171870.157640193.1:49-89 HWI-ST967_77:2:2203:11139:120754.
-agtacctaacaaaacgcaaacaa---gaccatagtgggggattc
>gnl|SRA|SRR1171870.48948155.2:1-41 HWI-ST967_77:2:1203:18751:32228.
-agtggcttacagaacgcaaacaa---gaccattgagggggattc
>_R_gnl|SRA|SRR1171870.27069344.2:22-62 HWI-ST967_77:2:1106:7690:18571.
-agtatcttacagaacgcaatcaa---gaccatagtgggggaatc
>_R_gnl|SRA|SRR1171870.156611734.2:61-100 HWI-ST967_77:2:2203:13855:83535.
--gtagcttacagaacgcaaacaa---gaccatagtgggggattc
>gnl|SRA|SRR1171870.151273966.1:58-97 HWI-ST967_77:2:2202:15592:86839.
--gtagcttacagaacgcaaacaa---gaccatagtgggggattc
>_R_gnl|SRA|SRR1171870.135047467.2:47-86 HWI-ST967_77:2:2105:15460:127036.
--gtagcttacagaacgcaaacaa---gaccatagtgggggattc
>gnl|SRA|SRR1171870.103518229.1:1-40 HWI-ST967_77:2:1306:17009:111323.
--gtagcttacagaacgcaaacaa---gaccatagtgggggattc
>gnl|SRA|SRR1171870.23336340.2:1-40 HWI-ST967_77:2:1105:13770:83974.
--gtagcttacagaacgcaaacaa---gaccatagtgggggattc
>_R_gnl|SRA|SRR1171870.71910921.2:19-58 HWI-ST967_77:2:1208:8979:7746.
--gtngcttacagaacgcaaacaa---gacnatagtgnggnnttc
>_R_gnl|SRA|SRR1171870.130070616.2:61-100 HWI-ST967_77:2:2104:3931:142410.
--gtagcttacagaacgcaaacaa---gaccatcgtgggggattc
>gnl|SRA|SRR1171870.55264812.2:1-40 HWI-ST967_77:2:1204:20606:60041.
--gtagcttacagaacgcaaacaa---gaccataggggggggattc
>_R_gnl|SRA|SRR1171870.75866389.2:62-100 HWI-ST967_77:2:1301:11582:101561.
---tagcttacagaacgcaaacaa---gaccatagtgggggattc
>gnl|SRA|SRR1171870.171469791.2:34-72 HWI-ST967_77:2:2206:14606:22012.
---tagcttacagaacgcaaacaa---gaccagagtgggggattc
>_R_gnl|SRA|SRR1171870.67363022.2:64-100 HWI-ST967_77:2:1206:11669:98550.
-----gcttacagaacgcaaacaa---gaccatagtgggggattc
>gnl|SRA|SRR1171870.56042973.2:1-37 HWI-ST967_77:2:1204:12690:88411.
-----gcttacagaacgcaaacaa---gaccatagtgggggattc
>gnl|SRA|SRR1171870.23184052.2:1-37 HWI-ST967_77:2:1105:15679:78282.
-----gcttacagaacgcaaacaa---gaccatagtgggggattc
>gnl|SRA|SRR1171870.204725373.1:1-36 HWI-ST967_77:2:2306:18280:38804.
------cttacagaacgcaaacaa---gaccatagtgggggattc
>gnl|SRA|SRR1171870.197052543.1:1-36 HWI-ST967_77:2:2304:10477:155370.

------cttacagaacgcaaacaa---gaccatagtgggggattc
>gnl|SRA|SRR1171870.193039737.1:1-36 HWI-ST967_77:2:2304:15730:11191.
------cttacagaacgcaaacaa---gaccatagtgggggattc
>_R_gnl|SRA|SRR1171870.186319210.2:65-100 HWI-ST967_77:2:2302:7071:160602.
------cttacagaacgcaaacaa---gaccatagtgggggattc
>gnl|SRA|SRR1171870.177893229.1:1-36 HWI-ST967_77:2:2301:16953:34913.
------cttacagaacgcaaacaa---gaccatagtgggggattc
>gnl|SRA|SRR1171870.168143875.1:1-36 HWI-ST967_77:2:2205:13992:99022.
------cttacagaacgcaaacaa---gaccatagtgggggattc
>gnl|SRA|SRR1171870.117811618.2:1-36 HWI-ST967_77:2:2102:11245:81517.
------cttacagaacgcaaacaa---gaccatagtgggggattc
>gnl|SRA|SRR1171870.81429243.1:1-36 HWI-ST967_77:2:1302:18045:113923.
------cttacagaacgcaaacaa---gaccatagtgggggattc
>gnl|SRA|SRR1171870.77981971.2:1-36 HWI-ST967_77:2:1301:7881:181799.
------cttacagaacgcaaacaa---gaccatagtgggggattc
>gnl|SRA|SRR1171870.202026307.1:1-35 HWI-ST967_77:2:2305:20035:139501.
-------ttacagaacgcaaacaa---gaccatagtgggggattc
>gnl|SRA|SRR1171870.178194071.1:1-35 HWI-ST967_77:2:2301:12753:46572.
-------ttacagaacgcaaacaa---gaccatagtgggggattc
>_R_gnl|SRA|SRR1171870.170802391.1:66-100 HWI-ST967_77:2:2205:8418:194220.
-------ttacagaacgcaaacaa---gaccatagtgggggattc
>gnl|SRA|SRR1171870.130619261.2:1-35 HWI-ST967_77:2:2104:11815:162332.
-------ttacagaacgcaaacaa---gaccatagtgggggattc
>gnl|SRA|SRR1171870.57444059.1:1-35 HWI-ST967_77:2:1204:2914:139481.
-------ttacagaacgcaaacaa---gaccatagtgggggattc
>gnl|SRA|SRR1171870.20300209.1:1-35 HWI-ST967_77:2:1104:15166:172607.
-------ttacagaacgcaaacaa---gaccatagtgggggattc
>_R_gnl|SRA|SRR1171870.11945217.1:66-100 HWI-ST967_77:2:1103:2648:60871.
-------ttacagagcgcaaacaa---gaccatagtgggggattc
>gnl|SRA|SRR1171870.144755000.2:1-34 HWI-ST967_77:2:2201:13866:39342.
--------tacagaacgcaaacaa---gaccatagtgggggattc
>_R_gnl|SRA|SRR1171870.93020207.1:67-100 HWI-ST967_77:2:1304:10672:136175.
--------tacagaacgcaaacaa---gaccatagtgggggattc
>gnl|SRA|SRR1171870.78243770.1:1-34 HWI-ST967_77:2:1301:9946:191715.
--------tacagaacgcaaacaa---gaccatagtgggggattc
>_R_gnl|SRA|SRR1171870.71448266.1:67-100 HWI-ST967_77:2:1207:8174:144750.
--------tacagaacgcaaacaa---gaccatagtgggggattc
>_R_gnl|SRA|SRR1171870.57118905.2:67-100 HWI-ST967_77:2:1204:6037:127620.
--------tacagaacgcaaacaa---gaccatagtgggggattc
>gnl|SRA|SRR1171870.150977131.1:1-32 HWI-ST967_77:2:2202:5613:75949.
----------cagaacgcaaacaa---gaccatagtgggggattc
>gnl|SRA|SRR1171870.125846562.2:1-32 HWI-ST967_77:2:2103:8534:185225.
----------cagaacgcaaacaa---gaccatagtgggggattc
>gnl|SRA|SRR1171870.53784595.1:1-32 HWI-ST967_77:2:1204:9628:7276.
----------cagaacgcaaacaa---gaccatagtgggggattc
>gnl|SRA|SRR1171870.22724026.1:1-32 HWI-ST967_77:2:1105:8088:61779.
----------cagaacgcaaacaa---gaccatagtgggggattc
>_R_gnl|SRA|SRR1171870.191666868.1:68-100 HWI-ST967_77:2:2303:20427:158079.
---------acagaacgcaaacaa---gaccatagtgggggattc
>_R_gnl|SRA|SRR1171870.170844275.1:68-100 HWI-ST967_77:2:2205:5921:195623.
---------acagaacgcaaacaa---gaccatagtgggggattc

>gnl|SRA|SRR1171870.162528296.1:41-73 HWI-ST967_77:2:2204:19896:96855.
---------acagaacgcaaacaa---gaccatagtgggggattc
>gnl|SRA|SRR1171870.77824671.2:41-73 HWI-ST967_77:2:1301:13228:175905.
---------acagaacgcaaacaa---gaccatagtgggggattc
>_R_gnl|SRA|SRR1171870.61424044.1:68-100 HWI-ST967_77:2:1205:10053:82598.
---------acagaacgcaaacaa---gaccatagtgggggattc
>_R_gnl|SRA|SRR1171870.50411049.1:68-100 HWI-ST967_77:2:1203:10558:85068.
---------acagaacgcaaacaa---gaccatagtgggggattc
>_R_gnl|SRA|SRR1171870.16558742.2:68-100 HWI-ST967_77:2:1104:6542:32920.
---------acagaacgcaaacaa---gaccatagtgggggattc
>_R_gnl|SRA|SRR1171870.12580093.1:68-100 HWI-ST967_77:2:1103:12445:84319.
---------acagaacgcaaacaa---gaccatagtgggggattc
>_R_gnl|SRA|SRR1171870.67971674.2:68-100 HWI-ST967_77:2:1206:17496:121034.
---------acagaacacaaacaa---gaccatagtgggggattc
>_R_gnl|SRA|SRR1171870.11462242.2:68-100 HWI-ST967_77:2:1103:5706:42759.
---------acagaacgcaaacaa---gaccagagaggggggattc
>gnl|SRA|SRR1171870.167517033.2:1-31 HWI-ST967_77:2:2205:10028:75704.
-----------agaacgcaaacaa---gaccatagtgggggattc
>_R_gnl|SRA|SRR1171870.154189321.2:70-100 HWI-ST967_77:2:2202:5618:194921.
-----------agaacgcaaacaa---gaccatagtgggggattc
>_R_gnl|SRA|SRR1171870.152383026.2:70-100 HWI-ST967_77:2:2202:1304:128793.
-----------agaacgcaaacaa---gaccatagtgggggattc
>_R_gnl|SRA|SRR1171870.145318500.2:70-100 HWI-ST967_77:2:2201:11635:60953.
-----------agaacgcaaacaa---gaccatagtgggggattc
>_R_gnl|SRA|SRR1171870.133507690.2:70-100 HWI-ST967_77:2:2105:5652:69561.
-----------agaacgcaaacaa---gaccatagtgggggattc
>_R_gnl|SRA|SRR1171870.81460217.2:70-100 HWI-ST967_77:2:1302:14617:115082.
-----------agaacgcaaacaa---gaccatagtgggggattc
>gnl|SRA|SRR1171870.73737659.1:1-31 HWI-ST967_77:2:1301:4749:19909.
-----------agaacgcaaacaa---gaccatagtgggggattc
>_R_gnl|SRA|SRR1171870.50592355.2:70-100 HWI-ST967_77:2:1203:18224:91602.
-----------agaacgcaaacaa---gaccatagtgggggattc
>_R_gnl|SRA|SRR1171870.39921623.2:70-100 HWI-ST967_77:2:1201:12262:92787.
-----------agaacgcaaacaa---gaccatagtgggggattc
>gnl|SRA|SRR1171870.90057250.2:1-30 HWI-ST967_77:2:1304:16338:30604.
------------gaacgcaaacaa---gaccatagtgggggattc
>gnl|SRA|SRR1171870.74765275.2:1-30 HWI-ST967_77:2:1301:10821:59372.
------------gaacgcaaacaa---gaccatagtgggggattc
>gnl|SRA|SRR1171870.140875191.2:1-30 HWI-ST967_77:2:2106:14663:142809.
------------gaacggaaacaa---gaccatagtgggggattc
>gnl|SRA|SRR1171870.95490363.2:1-29 HWI-ST967_77:2:1305:15485:23842.
-------------aacgcaaacaa---gaccatagtgggggattc
>_R_gnl|SRA|SRR1171870.82763169.1:72-100 HWI-ST967_77:2:1302:19591:165101.
-------------aacgcaaacaa---gaccatagtgggggattc
>_R_gnl|SRA|SRR1171870.31860207.2:72-100 HWI-ST967_77:2:1106:11635:190174.
-------------aacgcaaacaa---gaccatagtgggggattc
>gnl|SRA|SRR1171870.27193998.1:1-29 HWI-ST967_77:2:1106:16714:22911.
-------------aacgcaaacaa---gaccatagtgggggattc
>_R_gnl|SRA|SRR1171870.167504877.1:73-100 HWI-ST967_77:2:2205:14872:75226.
--------------acgcaaacaa---gaccatagtgggggattc
>_R_gnl|SRA|SRR1171870.157088706.1:73-100 HWI-ST967_77:2:2203:16511:100908.

--------------acgcaaacaa---gaccatagtgggggattc
>gnl|SRA|SRR1171870.128526359.2:1-28 HWI-ST967_77:2:2104:15598:85188.
--------------acgcaaacaa---gaccatagtgggggattc
>_R_gnl|SRA|SRR1171870.66615096.1:73-100 HWI-ST967_77:2:1206:5673:71351.
--------------acgcaaacaa---gaccatagtgggggattc
>_R_gnl|SRA|SRR1171870.28102105.2:73-100 HWI-ST967_77:2:1106:9393:54885.
--------------acgcaaacaa---gaccatagtgggggattc
>_R_gnl|SRA|SRR1171870.22724026.2:73-100 HWI-ST967_77:2:1105:8088:61779.
--------------acgcaaacaa---gaccatagtgggggattc
>_R_gnl|SRA|SRR1171870.18780837.1:73-100 HWI-ST967_77:2:1104:15680:115019.
--------------acgcaaacaa---gaccatagtgggggattc
>_R_gnl|SRA|SRR1171870.206872260.1:73-100 HWI-ST967_77:2:2306:4391:118826.
--------------acgcaaacaa---gaccaaagtgggggattc
>gnl|SRA|SRR1171870.121068709.2:1-25 HWI-ST967_77:2:2103:19113:7740.
-----------------caaacaa---gaccatagtgggggattc
>gnl|SRA|SRR1171870.19195439.1:1-25 HWI-ST967_77:2:1104:17795:130912.
-----------------caaacaa---gaccatagtgggggattc
>gnl|SRA|SRR1171870.206482441.1:1-27 HWI-ST967_77:2:2306:9873:104155.
---------------cgcaaacaa---gaccatagtgggggattc
>_R_gnl|SRA|SRR1171870.203587746.1:74-100 HWI-ST967_77:2:2305:13953:195684.
---------------cgcaaacaa---gaccatagtgggggattc
>gnl|SRA|SRR1171870.189488638.1:1-27 HWI-ST967_77:2:2303:8574:78537.
---------------cgcaaacaa---gaccatagtgggggattc
>_R_gnl|SRA|SRR1171870.154050326.1:74-100 HWI-ST967_77:2:2202:7828:189842.
---------------cgcaaacaa---gaccatagtgggggattc
>_R_gnl|SRA|SRR1171870.149193493.1:74-100 HWI-ST967_77:2:2202:1629:9932.
---------------cgcaaacaa---gaccatagtgggggattc
>gnl|SRA|SRR1171870.127277338.2:1-27 HWI-ST967_77:2:2104:7442:39269.
---------------cgcaaacaa---gaccatagtgggggattc
>gnl|SRA|SRR1171870.127205584.2:1-27 HWI-ST967_77:2:2104:15705:36524.
---------------cgcaaacaa---gaccatagtgggggattc
>gnl|SRA|SRR1171870.125276974.2:1-27 HWI-ST967_77:2:2103:10904:163913.
---------------cgcaaacaa---gaccatagtgggggattc
>_R_gnl|SRA|SRR1171870.112564429.1:74-100 HWI-ST967_77:2:2101:11076:77517.
---------------cgcaaacaa---gaccatagtgggggattc
>gnl|SRA|SRR1171870.106193371.1:1-27 HWI-ST967_77:2:1307:19711:13643.
---------------cgcaaacaa---gaccatagtgggggattc
>gnl|SRA|SRR1171870.99692732.2:1-27 HWI-ST967_77:2:1305:7539:174048.
---------------cgcaaacaa---gaccatagtgggggattc
>gnl|SRA|SRR1171870.79026414.2:1-27 HWI-ST967_77:2:1302:10757:22498.
---------------cgcaaacaa---gaccatagtgggggattc
>gnl|SRA|SRR1171870.52591664.2:1-27 HWI-ST967_77:2:1203:5610:163293.
---------------cgcaaacaa---gaccatagtgggggattc
>gnl|SRA|SRR1171870.42005215.2:1-27 HWI-ST967_77:2:1201:3776:172315.
---------------cgcaaacaa---gaccatagtgggggattc
>gnl|SRA|SRR1171870.24369139.2:1-27 HWI-ST967_77:2:1105:15177:120880.
---------------cgcaaacaa---gaccatagtgggggattc
>gnl|SRA|SRR1171870.20153646.1:1-27 HWI-ST967_77:2:1104:14688:167221.
---------------cgcaaacaa---gaccatagtgggggattc
>_R_gnl|SRA|SRR1171870.18128797.1:74-100 HWI-ST967_77:2:1104:17303:90433.
---------------cgcaaacaa---gaccatagtgggggattc

>gnl|SRA|SRR1171870.7741135.2:1-27 HWI-ST967_77:2:1102:17503:98514.
---------------cgcaaacaa---gaccatagtgggggattc
>gnl|SRA|SRR1171870.204849449.1:1-27 HWI-ST967_77:2:2306:20462:43494.
---------------cgcaaacaa---gaccatggtgggggattc
>gnl|SRA|SRR1171870.140985685.2:1-27 HWI-ST967_77:2:2106:4122:147124.
---------------cgcaaacaa---gaccacagtgggggattc
>gnl|SRA|SRR1171870.132712909.2:1-27 HWI-ST967_77:2:2105:4997:39940.
---------------cgcaaacaa---gactatagtgggggattc
>gnl|SRA|SRR1171870.36539229.2:1-27 HWI-ST967_77:2:1108:10075:20159.
---------------cgcaaacaa---gaccatagtgggggcttc
>gnl|SRA|SRR1171870.71375838.1:1-27 HWI-ST967_77:2:1207:5672:134542.
---------------cgcaaacaa---gacaagagtgggggattc
>gnl|SRA|SRR1171870.200776226.2:1-26 HWI-ST967_77:2:2305:5329:93101.
----------------gcaaacaa---gaccatagtgggggattc
>gnl|SRA|SRR1171870.200457116.2:1-26 HWI-ST967_77:2:2305:3369:80314.
----------------gcaaacaa---gaccatagtgggggattc
>_R_gnl|SRA|SRR1171870.198602001.1:75-100 HWI-ST967_77:2:2305:3208:12180.
----------------gcaaacaa---gaccatagtgggggattc
>gnl|SRA|SRR1171870.193880753.2:1-26 HWI-ST967_77:2:2304:18746:41077.
----------------gcaaacaa---gaccatagtgggggattc
>gnl|SRA|SRR1171870.190741588.2:1-26 HWI-ST967_77:2:2303:11429:124667.
----------------gcaaacaa---gaccatagtgggggattc
>gnl|SRA|SRR1171870.187451312.1:1-26 HWI-ST967_77:2:2303:18377:4081.
----------------gcaaacaa---gaccatagtgggggattc
>gnl|SRA|SRR1171870.180684588.2:1-26 HWI-ST967_77:2:2301:15450:143653.
----------------gcaaacaa---gaccatagtgggggattc
>_R_gnl|SRA|SRR1171870.178484665.1:75-100 HWI-ST967_77:2:2301:10071:57922.
----------------gcaaacaa---gaccatagtgggggattc
>gnl|SRA|SRR1171870.176059303.1:1-26 HWI-ST967_77:2:2208:8629:18227.
----------------gcaaacaa---gaccatagtgggggattc
>gnl|SRA|SRR1171870.160359740.2:1-26 HWI-ST967_77:2:2204:21132:19967.
----------------gcaaacaa---gaccatagtgggggattc
>gnl|SRA|SRR1171870.153608848.2:1-26 HWI-ST967_77:2:2202:19728:173771.
----------------gcaaacaa---gaccatagtgggggattc
>gnl|SRA|SRR1171870.145608605.1:1-26 HWI-ST967_77:2:2201:11725:71897.
----------------gcaaacaa---gaccatagtgggggattc
>_R_gnl|SRA|SRR1171870.135992093.1:75-100 HWI-ST967_77:2:2105:9052:161805.
----------------gcaaacaa---gaccatagtgggggattc
>gnl|SRA|SRR1171870.125389645.2:1-26 HWI-ST967_77:2:2103:13973:168138.
----------------gcaaacaa---gaccatagtgggggattc
>gnl|SRA|SRR1171870.95173126.1:1-26 HWI-ST967_77:2:1305:19325:12782.
----------------gcaaacaa---gaccatagtgggggattc
>_R_gnl|SRA|SRR1171870.92441231.2:75-100 HWI-ST967_77:2:1304:15331:115710.
----------------gcaaacaa---gaccatagtgggggattc
>gnl|SRA|SRR1171870.88584464.1:1-26 HWI-ST967_77:2:1303:11281:176933.
----------------gcaaacaa---gaccatagtgggggattc
>gnl|SRA|SRR1171870.84018906.2:1-26 HWI-ST967_77:2:1303:12837:12482.
----------------gcaaacaa---gaccatagtgggggattc
>gnl|SRA|SRR1171870.83865791.2:1-26 HWI-ST967_77:2:1303:12780:6905.
----------------gcaaacaa---gaccatagtgggggattc
>_R_gnl|SRA|SRR1171870.82270827.2:75-100 HWI-ST967_77:2:1302:4596:146927.

----------------gcaaacaa---gaccatagtgggggattc
>_R_gnl|SRA|SRR1171870.44079028.2:75-100 HWI-ST967_77:2:1202:17392:50683.
----------------gcaaacaa---gaccatagtgggggattc
>_R_gnl|SRA|SRR1171870.36818017.1:75-100 HWI-ST967_77:2:1108:12066:66568.
----------------gcaaacaa---gaccatagtgggggattc
>_R_gnl|SRA|SRR1171870.590494.1:75-100 HWI-ST967_77:2:1101:8962:24914.
----------------gcaaacaa---gaccatagtgggggattc
>gnl|SRA|SRR1171870.195401133.1:1-26 HWI-ST967_77:2:2304:12734:96216.
----------------gcaaacaa---gaccatagtgggggtttc
>gnl|SRA|SRR1171870.92837738.2:1-26 HWI-ST967_77:2:1304:17788:129613.
----------------gcaaacaa---gaccatagtgtgggattc
>_R_gnl|SRA|SRR1171870.47814277.1:75-100 HWI-ST967_77:2:1202:9276:189667.
----------------gcaaacaa---gaccagagtgggggattc
>gnl|SRA|SRR1171870.195435814.1:1-24 HWI-ST967_77:2:2304:12132:97357.
------------------aaacaa---gaccatagtgggggattc
>gnl|SRA|SRR1171870.187094650.1:1-24 HWI-ST967_77:2:2302:13950:189452.
------------------aaacaa---gaccatagtgggggattc
>gnl|SRA|SRR1171870.170686034.2:1-24 HWI-ST967_77:2:2205:2300:190156.
------------------aaacaa---gaccatagtgggggattc
>gnl|SRA|SRR1171870.154731420.2:1-24 HWI-ST967_77:2:2203:16531:15901.
------------------aaacaa---gaccatagtgggggattc
>gnl|SRA|SRR1171870.112972445.1:1-24 HWI-ST967_77:2:2101:11411:93613.
------------------aaacaa---gaccatagtgggggattc
>gnl|SRA|SRR1171870.109505391.1:1-24 HWI-ST967_77:2:1308:5574:13474.
------------------aaacaa---gaccatagtgggggattc
>gnl|SRA|SRR1171870.105154047.1:1-24 HWI-ST967_77:2:1306:9663:170611.
------------------aaacaa---gaccatagtgggggattc
>gnl|SRA|SRR1171870.88811330.2:1-24 HWI-ST967_77:2:1303:12070:184967.
------------------aaacaa---gaccatagtgggggattc
>gnl|SRA|SRR1171870.87615029.1:1-24 HWI-ST967_77:2:1303:8783:142390.
------------------aaacaa---gaccatagtgggggattc
>gnl|SRA|SRR1171870.85139474.1:1-24 HWI-ST967_77:2:1303:12796:52848.
------------------aaacaa---gaccatagtgggggattc
>gnl|SRA|SRR1171870.59603559.2:1-24 HWI-ST967_77:2:1205:10480:17377.
------------------aaacaa---gaccatagtgggggattc
>gnl|SRA|SRR1171870.53574707.2:1-24 HWI-ST967_77:2:1203:10660:198136.
------------------aaacaa---gaccatagtgggggattc
>_R_gnl|SRA|SRR1171870.43290289.1:35-58 HWI-ST967_77:2:1202:13663:21505.
------------------aaacaa---gaccatagtgggggattc
>gnl|SRA|SRR1171870.4242945.2:1-24 HWI-ST967_77:2:1101:20790:164032.
------------------aaacaa---gaccatagtgggggattc
>_R_gnl|SRA|SRR1171870.64973181.2:9-32 HWI-ST967_77:2:1206:15512:12331.
------------------aaacaa---gaccagagtgggggattc
>gnl|SRA|SRR1171870.60538229.2:44-67 HWI-ST967_77:2:1205:10503:50596.
------------------aaacca---gaccagagtgggggattc
>gnl|SRA|SRR1171870.139412348.1:1-23 HWI-ST967_77:2:2106:9519:88473.
-------------------aacaa---gaccatagtgggggattc
>gnl|SRA|SRR1171870.43290289.2:1-23 HWI-ST967_77:2:1202:13663:21505.
-------------------aacaa---gaccatagtgggggattc
>_R_gnl|SRA|SRR1171870.6946151.1:78-100 HWI-ST967_77:2:1102:20551:67852.
-------------------aacaa---gaccatagtgggggattc

>gnl|SRA|SRR1171870.187094650.1:65-86 HWI-ST967_77:2:2302:13950:189452.
--------------------acaa---gaccatagtgggggattc
>_R_gnl|SRA|SRR1171870.187094650.2:25-46 HWI-ST967_77:2:2302:13950:189452.
--------------------acaa---gaccatagtgggggattc
>_R_gnl|SRA|SRR1171870.104987552.1:79-100 HWI-ST967_77:2:1306:10625:164613.
--------------------acaa---gaccatagtgggggattc
>_R_gnl|SRA|SRR1171870.95497796.2:79-100 HWI-ST967_77:2:1305:16101:24232.
--------------------acaa---gaccatagtgggggattc
>gnl|SRA|SRR1171870.14507487.2:1-22 HWI-ST967_77:2:1103:11312:156137.
--------------------acaa---gaccatagcgggggattc
>gnl|SRA|SRR1171870.197962529.2:1-21 HWI-ST967_77:2:2304:1820:187806.
---------------------caa---gaccatagtgggggattc
>gnl|SRA|SRR1171870.195998873.1:1-21 HWI-ST967_77:2:2304:3119:117639.
---------------------caa---gaccatagtgggggattc
>gnl|SRA|SRR1171870.160248334.2:1-21 HWI-ST967_77:2:2204:4748:16096.
---------------------caa---gaccatagtgggggattc
>_R_gnl|SRA|SRR1171870.147105640.2:80-100 HWI-ST967_77:2:2201:15398:129459.
---------------------caa---gaccatagtgggggattc
>gnl|SRA|SRR1171870.138641016.1:1-21 HWI-ST967_77:2:2106:14781:60102.
---------------------caa---gaccatagtgggggattc
>gnl|SRA|SRR1171870.118662079.1:1-21 HWI-ST967_77:2:2102:10485:114566.
---------------------caa---gaccatagtgggggattc
>gnl|SRA|SRR1171870.113439005.2:1-21 HWI-ST967_77:2:2101:12170:111778.
---------------------caa---gaccatagtgggggattc
>gnl|SRA|SRR1171870.111692379.1:1-21 HWI-ST967_77:2:2101:14698:43370.
---------------------caa---gaccatagtgggggattc
>_R_gnl|SRA|SRR1171870.102351169.1:80-100 HWI-ST967_77:2:1306:6963:69148.
---------------------caa---gaccatagtgggggattc
>_R_gnl|SRA|SRR1171870.91599568.1:80-100 HWI-ST967_77:2:1304:13743:85572.
---------------------caa---gaccatagtgggggattc
>gnl|SRA|SRR1171870.81193744.2:1-21 HWI-ST967_77:2:1302:3076:104985.
---------------------caa---gaccatagtgggggattc
>gnl|SRA|SRR1171870.75464433.2:1-21 HWI-ST967_77:2:1301:15700:86149.
---------------------caa---gaccatagtgggggattc
>gnl|SRA|SRR1171870.75460164.2:1-21 HWI-ST967_77:2:1301:2984:86044.
---------------------caa---gaccatagtgggggattc
>_R_gnl|SRA|SRR1171870.74098690.2:80-100 HWI-ST967_77:2:1301:14900:33595.
---------------------caa---gaccatagtgggggattc
>gnl|SRA|SRR1171870.61286995.2:1-21 HWI-ST967_77:2:1205:2103:77711.
---------------------caa---gaccatagtgggggattc
>_R_gnl|SRA|SRR1171870.55246219.1:80-100 HWI-ST967_77:2:1204:7273:59592.
---------------------caa---gaccatagtgggggattc
>gnl|SRA|SRR1171870.40328331.1:1-21 HWI-ST967_77:2:1201:9777:108726.
---------------------caa---gaccatagtgggggattc
>gnl|SRA|SRR1171870.35929959.1:1-21 HWI-ST967_77:2:1107:1241:175519.
---------------------caa---gaccatagtgggggattc
>gnl|SRA|SRR1171870.30776773.1:1-21 HWI-ST967_77:2:1106:15970:151158.
---------------------caa---gaccatagtgggggattc
>gnl|SRA|SRR1171870.16079971.2:1-21 HWI-ST967_77:2:1104:9819:15718.
---------------------caa---gaccatagtgggggattc
>gnl|SRA|SRR1171870.9081385.2:1-21 HWI-ST967_77:2:1102:11575:151423.

---------------------caa---gaccatagtgggggattc
>gnl|SRA|SRR1171870.4656745.2:1-21 HWI-ST967_77:2:1101:20715:179685.
---------------------caa---gaccatagtgggggattc
>_R_gnl|SRA|SRR1171870.75712489.1:81-100 HWI-ST967_77:2:1301:18506:95685.
----------------------aa---gaccatagtgggggattc
>gnl|SRA|SRR1171870.202863241.1:1-19 HWI-ST967_77:2:2305:18217:169849.
-----------------------a---gaccatagtgggggattc
>_R_gnl|SRA|SRR1171870.156887713.1:82-100 HWI-ST967_77:2:2203:13257:93537.
-----------------------a---gaccatagtgggggattc
>gnl|SRA|SRR1171870.155702469.2:1-19 HWI-ST967_77:2:2203:5329:50956.
-----------------------a---gaccatagtgggggattc
>gnl|SRA|SRR1171870.152341074.2:1-19 HWI-ST967_77:2:2202:14146:127178.
-----------------------a---gaccatagtgggggattc
>gnl|SRA|SRR1171870.151308024.2:1-19 HWI-ST967_77:2:2202:17992:88099.
-----------------------a---gaccatagtgggggattc
>gnl|SRA|SRR1171870.117002818.1:1-19 HWI-ST967_77:2:2102:18254:50571.
-----------------------a---gaccatagtgggggattc
>gnl|SRA|SRR1171870.82512250.1:1-19 HWI-ST967_77:2:1302:14249:155778.
-----------------------a---gaccatagtgggggattc
>gnl|SRA|SRR1171870.80978963.1:1-19 HWI-ST967_77:2:1302:12666:96389.
-----------------------a---gaccatagtgggggattc
>_R_gnl|SRA|SRR1171870.647339.2:82-100 HWI-ST967_77:2:1101:4735:27169.
-----------------------a---gaccatagtgggggattc
>_R_gnl|SRA|SRR1171870.96276769.2:31-72 HWI-ST967_77:2:1305:15836:51346.
cagtagcgtcaagaacccaaccaa---gaccatagtgggggagtc
>gnl|SRA|SRR1171870.206708997.2:58-99 HWI-ST967_77:2:2306:10317:112557.
cagtgtctgacaggacgcaagcaa---gaccatagttgggtattc
>_R_gnl|SRA|SRR1171870.174585631.2:33-74 HWI-ST967_77:2:2206:7498:156017.
cagtagtccatagcacgccaacaa---gacaatcgtgggggattc
>gnl|SRA|SRR1171870.159720957.1:60-100 HWI-ST967_77:2:2203:14368:195662.
cagtagcttacagaacgcaaacaa---gaccatagtgggggatt-
>_R_gnl|SRA|SRR1171870.123060995.1:1-41 HWI-ST967_77:2:2103:17410:81759.
cagtagcttacagaacgcaaacaa---gaccatagtgggggatt-
>_R_gnl|SRA|SRR1171870.109068191.1:32-72 HWI-ST967_77:2:1307:5877:187904.
cagtagcttacagaacgcaaacaa---gaccatagtgggggatt-
>gnl|SRA|SRR1171870.38364770.2:60-100 HWI-ST967_77:2:1201:3430:33621.
cagtagcttacagaacgcaaacaa---gaccatagtgggggatt-
>gnl|SRA|SRR1171870.166642512.2:44-84 HWI-ST967_77:2:2205:2087:44380.
cagtagcttacagaacgcaaacaa---gacaatagtgggggatt-
>_R_gnl|SRA|SRR1171870.29059898.2:18-58 HWI-ST967_77:2:1106:18828:89108.
cagtagcttacagaacgcaaccaa---gaccatagtgggggatt-
>gnl|SRA|SRR1171870.67971674.1:51-91 HWI-ST967_77:2:1206:17496:121034.
cagaagcttacagaacgcaaccaa---gaccatagtgggggatt-
>_R_gnl|SRA|SRR1171870.104466291.2:14-54 HWI-ST967_77:2:1306:7768:145777.
cagtagattacagaacgcaaaaaa---gacaatagtgggggatt-
>_R_gnl|SRA|SRR1171870.203160762.1:1-40 HWI-ST967_77:2:2305:2595:180585.
cagtagcttacagaacgcaaacaa---gaccatagtgggggat--
>gnl|SRA|SRR1171870.136828907.2:47-86 HWI-ST967_77:2:2105:10736:192314.
cagtagcttacagaacgcaaacaa---gaccatagtgggggat--
>_R_gnl|SRA|SRR1171870.81042408.1:1-40 HWI-ST967_77:2:1302:11785:98927.
cagtagcttacagaacgcaaacaa---gaccatagtgggggat--

>gnl|SRA|SRR1171870.33236182.1:40-79 HWI-ST967_77:2:1107:6797:49549.
cagtagcttacagaacgcaaacaa---gaccatagtgggggat--
>_R_gnl|SRA|SRR1171870.13666435.2:1-40 HWI-ST967_77:2:1103:8825:124957.
cagtagcttacagaacgcaaacaa---gaccatagtgggggat--
>gnl|SRA|SRR1171870.8832758.2:52-91 HWI-ST967_77:2:1102:13252:141603.
cagtagcttacagaacgcaaacaa---gaccatagtgggggat--
>gnl|SRA|SRR1171870.191958599.1:62-100 HWI-ST967_77:2:2303:4888:168879.
cagtagcttacagaacgcaaacaa---gaccatagtgggggga---
>_R_gnl|SRA|SRR1171870.52946705.2:1-39 HWI-ST967_77:2:1203:13591:175978.
cagtagcttacagaacgcaaacaa---gaccatagtgggggga---
>_R_gnl|SRA|SRR1171870.675156.1:1-39 HWI-ST967_77:2:1101:10151:28075.
cagtagcttacagaacgcaaacaa---gaccatagtgggggga---
>_R_gnl|SRA|SRR1171870.146751530.2:1-39 HWI-ST967_77:2:2201:14098:115579.
cagtagcttacagaacgcaaacaa---gaccatagtgggaga---
>gnl|SRA|SRR1171870.22395852.1:63-100 HWI-ST967_77:2:1105:5742:50178.
cagtagcttacagaacgcaaacaa---gaccatagtgggggg----
>gnl|SRA|SRR1171870.152998608.2:64-100 HWI-ST967_77:2:2202:18491:151704.
cagtagcttacagaacgcaaacaa---gaccatagtgggg-----
>_R_gnl|SRA|SRR1171870.122807145.2:1-37 HWI-ST967_77:2:2103:2802:72743.
cagtagcttacagaacgcaaacaa---gaccatagtgggg-----
>_R_gnl|SRA|SRR1171870.120148102.2:1-37 HWI-ST967_77:2:2102:9680:171596.
cagtagcttacagaacgcaaacaa---gaccatagtgggg-----
>_R_gnl|SRA|SRR1171870.93995728.2:1-37 HWI-ST967_77:2:1304:1169:170744.
cagtagcttacagaacgcaaacaa---gaccatagtgggg-----
>_R_gnl|SRA|SRR1171870.88976165.1:1-37 HWI-ST967_77:2:1303:16388:190609.
cagtagcttacagaacgcaaacaa---gaccatagtgggg-----
>gnl|SRA|SRR1171870.50890396.2:64-100 HWI-ST967_77:2:1203:5728:102629.
cagtagcttacagaacgcaaacaa---gaccatagtgggg-----
>_R_gnl|SRA|SRR1171870.176398222.1:1-37 HWI-ST967_77:2:2208:13131:69889.
cagtaccttacagaacgcaaacaa---gaccatagtgggg-----
>gnl|SRA|SRR1171870.101262376.2:54-90 HWI-ST967_77:2:1306:13830:30641.
cagtagcttacataacgcaaacaa---gaccatagtgggg-----
>gnl|SRA|SRR1171870.203150787.2:65-100 HWI-ST967_77:2:2305:13905:180050.
cagtagcttacagaacgcaaacaa---gaccatagtggg------
>_R_gnl|SRA|SRR1171870.178586414.1:1-36 HWI-ST967_77:2:2301:3911:61946.
cagtagcttacagaacgcaaacaa---gaccatagtggg------
>_R_gnl|SRA|SRR1171870.142807511.2:49-84 HWI-ST967_77:2:2107:2918:54949.
cagtagcttacagaacgccaacac---gaccatagtggg------
>gnl|SRA|SRR1171870.191536765.1:67-100 HWI-ST967_77:2:2303:3453:153742.
cagtagcttacagaacgcaaacaa---gaccatagtg--------
>gnl|SRA|SRR1171870.149177954.1:67-100 HWI-ST967_77:2:2202:15795:9183.
cagtagcttacagaacgcaaacaa---gaccatagtg--------
>_R_gnl|SRA|SRR1171870.127016674.2:1-35 HWI-ST967_77:2:2104:20492:29535.
cagtagcttacagaacgcaaacaa---gaccatagtgg-------
>gnl|SRA|SRR1171870.114531769.1:66-100 HWI-ST967_77:2:2101:4795:154688.
cagtagcttacagaacgcaaacaa---gaccatagtgg-------
>gnl|SRA|SRR1171870.91599568.2:66-100 HWI-ST967_77:2:1304:13743:85572.
cagtagcttacagaacgcaaacaa---gaccatagtgg-------
>_R_gnl|SRA|SRR1171870.87562206.1:1-35 HWI-ST967_77:2:1303:17357:140490.
cagtagcttacagaacgcaaacaa---gaccatagtgg-------
>gnl|SRA|SRR1171870.182261777.2:66-100 HWI-ST967_77:2:2302:7037:5671.

cagtagcatacagaacgcaaacaa---gaccatagtgg-------
>gnl|SRA|SRR1171870.11462242.1:66-100 HWI-ST967_77:2:1103:5706:42759.
cagtagcttacagaacgcaaacaa---gaccatagagg-------
>_R_gnl|SRA|SRR1171870.206863108.1:1-32 HWI-ST967_77:2:2306:15760:118477.
cagtagcttacagaacgcaaacaa---gaccatag----------
>gnl|SRA|SRR1171870.199215941.2:69-100 HWI-ST967_77:2:2305:16848:33818.
cagtagcttacagaacgcaaacaa---gaccatag----------
>_R_gnl|SRA|SRR1171870.194760041.2:41-72 HWI-ST967_77:2:2304:3641:72975.
cagtagcttacagaacgcaaacaa---gaccatag----------
>_R_gnl|SRA|SRR1171870.191408678.1:1-32 HWI-ST967_77:2:2303:7923:148986.
cagtagcttacagaacgcaaacaa---gaccatag----------
>_R_gnl|SRA|SRR1171870.127864260.2:1-32 HWI-ST967_77:2:2104:12209:60794.
cagtagcttacagaacgcaaacaa---gaccatag----------
>_R_gnl|SRA|SRR1171870.113990189.1:1-32 HWI-ST967_77:2:2101:15461:133324.
cagtagcttacagaacgcaaacaa---gaccatag----------
>_R_gnl|SRA|SRR1171870.90828803.2:1-32 HWI-ST967_77:2:1304:5832:58070.
cagtagcttacagaacgcaaacaa---gaccatag----------
>_R_gnl|SRA|SRR1171870.57982762.1:1-32 HWI-ST967_77:2:1204:7893:158688.
cagtagcttacagaacgcaaacaa---gaccatag----------
>_R_gnl|SRA|SRR1171870.32541302.1:1-32 HWI-ST967_77:2:1107:15370:19051.
cagtagcttacagaacgcaaacaa---gaccatag----------
>_R_gnl|SRA|SRR1171870.4858992.2:1-32 HWI-ST967_77:2:1101:5954:187429.
cagtagcttacagaacgcaaacaa---gaccatag----------
>gnl|SRA|SRR1171870.139143583.2:68-100 HWI-ST967_77:2:2106:19497:78382.
cagtagcttacagaacgcaaacaa---gaccatagt---------
>gnl|SRA|SRR1171870.91322494.2:68-100 HWI-ST967_77:2:1304:11817:75552.
cagtagcttacagaacgcaaacaa---gaccatagt---------
>gnl|SRA|SRR1171870.13797218.1:68-100 HWI-ST967_77:2:1103:17963:129577.
cagtagcttacagaacgcaaacaa---gaccatagt---------
>_R_gnl|SRA|SRR1171870.9831089.1:1-33 HWI-ST967_77:2:1102:2587:180401.
cagtagcttacagaacgcaaacaa---gaccatagt---------
>gnl|SRA|SRR1171870.33665610.2:68-100 HWI-ST967_77:2:1107:8067:68514.
caggagcttacagaacgcacacaa---gaccatagt---------
>_R_gnl|SRA|SRR1171870.161679482.2:1-30 HWI-ST967_77:2:2204:16556:66563.
cagtagcttacagaacgcaaacaa---gaccat------------
>gnl|SRA|SRR1171870.126815105.2:71-100 HWI-ST967_77:2:2104:13660:22495.
cagtagcttacagaacgcaaacaa---gaccat------------
>_R_gnl|SRA|SRR1171870.32331594.1:1-30 HWI-ST967_77:2:1107:13617:10034.
cagtagcttacagaacgcaaacaa---gaccat------------
>gnl|SRA|SRR1171870.204773304.1:70-100 HWI-ST967_77:2:2306:17631:40665.
cagtagcttacagaacgcaaacaa---gaccata-----------
>gnl|SRA|SRR1171870.186579703.2:70-100 HWI-ST967_77:2:2302:10941:170367.
cagtagcttacagaacgcaaacaa---gaccata-----------
>gnl|SRA|SRR1171870.181701062.1:70-100 HWI-ST967_77:2:2301:7652:182617.
cagtagcttacagaacgcaaacaa---gaccata-----------
>gnl|SRA|SRR1171870.179014895.1:70-100 HWI-ST967_77:2:2301:11080:78421.
cagtagcttacagaacgcaaacaa---gaccata-----------
>gnl|SRA|SRR1171870.178684217.1:70-100 HWI-ST967_77:2:2301:8755:65637.
cagtagcttacagaacgcaaacaa---gaccata-----------
>gnl|SRA|SRR1171870.170802391.2:70-100 HWI-ST967_77:2:2205:8418:194220.
cagtagcttacagaacgcaaacaa---gaccata-----------

>gnl|SRA|SRR1171870.156887713.2:70-100 HWI-ST967_77:2:2203:13257:93537.
cagtagcttacagaacgcaaacaa---gaccata-----------
>gnl|SRA|SRR1171870.154050326.2:70-100 HWI-ST967_77:2:2202:7828:189842.
cagtagcttacagaacgcaaacaa---gaccata-----------
>gnl|SRA|SRR1171870.152272206.2:70-100 HWI-ST967_77:2:2202:7828:124550.
cagtagcttacagaacgcaaacaa---gaccata-----------
>gnl|SRA|SRR1171870.150772921.1:70-100 HWI-ST967_77:2:2202:17563:68178.
cagtagcttacagaacgcaaacaa---gaccata-----------
>gnl|SRA|SRR1171870.148842200.2:70-100 HWI-ST967_77:2:2201:5784:195217.
cagtagcttacagaacgcaaacaa---gaccata-----------
>gnl|SRA|SRR1171870.123032116.1:70-100 HWI-ST967_77:2:2103:11382:80977.
cagtagcttacagaacgcaaacaa---gaccata-----------
>gnl|SRA|SRR1171870.110817504.1:70-100 HWI-ST967_77:2:2101:16613:8874.
cagtagcttacagaacgcaaacaa---gaccata-----------
>gnl|SRA|SRR1171870.99869049.2:70-100 HWI-ST967_77:2:1305:20533:180068.
cagtagcttacagaacgcaaacaa---gaccata-----------
>gnl|SRA|SRR1171870.66719409.2:70-100 HWI-ST967_77:2:1206:10309:75064.
cagtagcttacagaacgcaaacaa---gaccata-----------
>gnl|SRA|SRR1171870.47642898.2:70-100 HWI-ST967_77:2:1202:16103:183299.
cagtagcttacagaacgcaaacaa---gaccata-----------
>gnl|SRA|SRR1171870.4788066.2:70-100 HWI-ST967_77:2:1101:13490:184736.
cagtagcttacagaacgcaaacaa---gaccata-----------
>gnl|SRA|SRR1171870.1135010.2:70-100 HWI-ST967_77:2:1101:2452:45821.
cagtagcttacagaacgcaaacaa---gaccata-----------
>gnl|SRA|SRR1171870.112564429.2:68-98 HWI-ST967_77:2:2101:11076:77517.
cagtagcttacagaacgcacacaa---gaccata-----------
>gnl|SRA|SRR1171870.61072272.2:70-100 HWI-ST967_77:2:1205:18713:69718.
cagtatcttaccgaacgcaaacaa---gaccata-----------
>gnl|SRA|SRR1171870.65188690.2:72-100 HWI-ST967_77:2:1206:14345:20032.
cagtagcttacagaacgcaaacaa---gacca-------------
>gnl|SRA|SRR1171870.25048260.1:72-100 HWI-ST967_77:2:1105:3096:145509.
cagtagcttacagaacgcaaacaa---gacca-------------
>_R_gnl|SRA|SRR1171870.20806930.1:1-29 HWI-ST967_77:2:1104:9078:191666.
cagtagcttacagaacgcaaacaa---gacca-------------
>_R_gnl|SRA|SRR1171870.133833032.1:1-28 HWI-ST967_77:2:2105:18050:81994.
cagtagcttacagaacgcaaacaa---gacc--------------
>gnl|SRA|SRR1171870.72387694.1:73-100 HWI-ST967_77:2:1208:4640:32580.
cagtagcttacagaacgcaaacaa---gacc--------------
>gnl|SRA|SRR1171870.47673565.2:73-100 HWI-ST967_77:2:1202:4948:184625.
cagtagcttacagaacgcaaacaa---gacc--------------
>_R_gnl|SRA|SRR1171870.32905836.1:1-28 HWI-ST967_77:2:1107:6937:35042.
cagtagcttacagaacgcaaacaa---gacc--------------
>gnl|SRA|SRR1171870.207121187.1:74-100 HWI-ST967_77:2:2306:17895:128054.
cagtagcttacagaacgcaaacaa---gac---------------
>_R_gnl|SRA|SRR1171870.149792300.1:1-27 HWI-ST967_77:2:2202:11345:31982.
cagtagcttacagaacgcaaacaa---gac---------------
>_R_gnl|SRA|SRR1171870.145089058.1:1-27 HWI-ST967_77:2:2201:14053:52070.
cagtagcttacagaacgcaaacaa---gac---------------
>_R_gnl|SRA|SRR1171870.128385638.1:1-27 HWI-ST967_77:2:2104:19836:79778.
cagtagcttacagaacgcaaacaa---gac---------------
>_R_gnl|SRA|SRR1171870.118519632.2:1-27 HWI-ST967_77:2:2102:9561:109212.

cagtagcttacagaacgcaaacaa---gac----------------
>_R_gnl|SRA|SRR1171870.75051350.1:1-27 HWI-ST967_77:2:1301:5756:70288.
cagtagcttacagaacgcaaacaa---gac----------------
>_R_gnl|SRA|SRR1171870.73800570.1:1-27 HWI-ST967_77:2:1301:18710:22095.
cagtagcttacagaacgcaaacaa---gac----------------
>_R_gnl|SRA|SRR1171870.49642752.1:1-27 HWI-ST967_77:2:1203:6309:57490.
cagtagcttacagaacgcaaacaa---gac----------------
>_R_gnl|SRA|SRR1171870.26371255.2:1-27 HWI-ST967_77:2:1105:11079:192385.
cagtagcttacagaacgcaaacaa---gac----------------
>_R_gnl|SRA|SRR1171870.14806154.2:1-27 HWI-ST967_77:2:1103:18611:167005.
cagtagcttacagaacgcaaacaa---gac----------------
>_R_gnl|SRA|SRR1171870.2612922.2:1-27 HWI-ST967_77:2:1101:12394:102286.
cagtagcttacagaacgcaaacaa---gac----------------
>_R_gnl|SRA|SRR1171870.184208961.2:1-26 HWI-ST967_77:2:2302:1778:79222.
cagtagcttacagaacgcaaacaa---ga-----------------
>gnl|SRA|SRR1171870.98251771.2:75-100 HWI-ST967_77:2:1305:4750:123582.
cagtagcttacagaacgcaaacaa---ga-----------------
>gnl|SRA|SRR1171870.93020207.2:75-100 HWI-ST967_77:2:1304:10672:136175.
cagtagcttacagaacgcaaacaa---ga-----------------
>_R_gnl|SRA|SRR1171870.202223260.2:1-25 HWI-ST967_77:2:2305:11305:146864.
cagtagcttacagaacgcaaacaa---g------------------
>gnl|SRA|SRR1171870.201604750.1:66-90 HWI-ST967_77:2:2305:16871:124340.
cagtagcttacagaacgcaaacaa---g------------------
>_R_gnl|SRA|SRR1171870.199704536.2:1-25 HWI-ST967_77:2:2305:12097:51936.
cagtagcttacagaacgcaaacaa---g------------------
>gnl|SRA|SRR1171870.198303906.2:76-100 HWI-ST967_77:2:2304:4826:199877.
cagtagcttacagaacgcaaacaa---g------------------
>gnl|SRA|SRR1171870.198173129.1:50-74 HWI-ST967_77:2:2304:15256:195235.
cagtagcttacagaacgcaaacaa---g------------------
>gnl|SRA|SRR1171870.193502749.2:58-82 HWI-ST967_77:2:2304:16526:27653.
cagtagcttacagaacgcaaacaa---g------------------
>_R_gnl|SRA|SRR1171870.191727240.1:1-25 HWI-ST967_77:2:2303:14324:160343.
cagtagcttacagaacgcaaacaa---g------------------
>gnl|SRA|SRR1171870.188812376.1:76-100 HWI-ST967_77:2:2303:12209:53843.
cagtagcttacagaacgcaaacaa---g------------------
>gnl|SRA|SRR1171870.186319210.1:76-100 HWI-ST967_77:2:2302:7071:160602.
cagtagcttacagaacgcaaacaa---g------------------
>_R_gnl|SRA|SRR1171870.169433468.1:1-25 HWI-ST967_77:2:2205:11402:145695.
cagtagcttacagaacgcaaacaa---g------------------
>_R_gnl|SRA|SRR1171870.168871575.2:1-25 HWI-ST967_77:2:2205:17036:125397.
cagtagcttacagaacgcaaacaa---g------------------
>_R_gnl|SRA|SRR1171870.163035932.1:1-25 HWI-ST967_77:2:2204:2952:115137.
cagtagcttacagaacgcaaacaa---g------------------
>gnl|SRA|SRR1171870.162837570.1:30-54 HWI-ST967_77:2:2204:18093:107981.
cagtagcttacagaacgcaaacaa---g------------------
>_R_gnl|SRA|SRR1171870.157406513.1:1-25 HWI-ST967_77:2:2203:18078:112274.
cagtagcttacagaacgcaaacaa---g------------------
>gnl|SRA|SRR1171870.153030859.1:76-100 HWI-ST967_77:2:2202:13012:152939.
cagtagcttacagaacgcaaacaa---g------------------
>gnl|SRA|SRR1171870.147105640.1:76-100 HWI-ST967_77:2:2201:15398:129459.
cagtagcttacagaacgcaaacaa---g------------------

>_R_gnl|SRA|SRR1171870.143866227.1:1-25 HWI-ST967_77:2:2201:18376:5152.
cagtagcttacagaacgcaaacaa---g-----------------
>_R_gnl|SRA|SRR1171870.138878462.1:1-25 HWI-ST967_77:2:2106:4570:68811.
cagtagcttacagaacgcaaacaa---g-----------------
>_R_gnl|SRA|SRR1171870.136057086.1:1-25 HWI-ST967_77:2:2105:15991:164217.
cagtagcttacagaacgcaaacaa---g-----------------
>gnl|SRA|SRR1171870.133990400.2:76-100 HWI-ST967_77:2:2105:9758:87971.
cagtagcttacagaacgcaaacaa---g-----------------
>_R_gnl|SRA|SRR1171870.127588606.1:1-25 HWI-ST967_77:2:2104:19203:50533.
cagtagcttacagaacgcaaacaa---g-----------------
>gnl|SRA|SRR1171870.116546986.1:51-75 HWI-ST967_77:2:2102:11527:33361.
cagtagcttacagaacgcaaacaa---g-----------------
>_R_gnl|SRA|SRR1171870.111741203.1:1-25 HWI-ST967_77:2:2101:9969:45407.
cagtagcttacagaacgcaaacaa---g-----------------
>gnl|SRA|SRR1171870.109606648.2:50-74 HWI-ST967_77:2:1308:12916:19244.
cagtagcttacagaacgcaaacaa---g-----------------
>_R_gnl|SRA|SRR1171870.109606648.1:60-84 HWI-ST967_77:2:1308:12916:19244.
cagtagcttacagaacgcaaacaa---g-----------------
>gnl|SRA|SRR1171870.104090745.2:76-100 HWI-ST967_77:2:1306:2765:132320.
cagtagcttacagaacgcaaacaa---g-----------------
>gnl|SRA|SRR1171870.100414257.1:50-74 HWI-ST967_77:2:1305:4592:199465.
cagtagcttacagaacgcaaacaa---g-----------------
>_R_gnl|SRA|SRR1171870.96255756.1:1-25 HWI-ST967_77:2:1305:17158:50621.
cagtagcttacagaacgcaaacaa---g-----------------
>_R_gnl|SRA|SRR1171870.90150268.2:1-25 HWI-ST967_77:2:1304:18350:33974.
cagtagcttacagaacgcaaacaa---g-----------------
>_R_gnl|SRA|SRR1171870.89645895.1:1-25 HWI-ST967_77:2:1304:14503:16249.
cagtagcttacagaacgcaaacaa---g-----------------
>gnl|SRA|SRR1171870.84058970.1:76-100 HWI-ST967_77:2:1303:9505:13817.
cagtagcttacagaacgcaaacaa---g-----------------
>gnl|SRA|SRR1171870.79198189.2:76-100 HWI-ST967_77:2:1302:3118:28837.
cagtagcttacagaacgcaaacaa---g-----------------
>_R_gnl|SRA|SRR1171870.79130503.1:1-25 HWI-ST967_77:2:1302:21227:26161.
cagtagcttacagaacgcaaacaa---g-----------------
>gnl|SRA|SRR1171870.75712489.2:76-100 HWI-ST967_77:2:1301:18506:95685.
cagtagcttacagaacgcaaacaa---g-----------------
>_R_gnl|SRA|SRR1171870.60583997.2:51-75 HWI-ST967_77:2:1205:1476:52479.
cagtagcttacagaacgcaaacaa---g-----------------
>_R_gnl|SRA|SRR1171870.57715470.2:1-25 HWI-ST967_77:2:1204:3339:149152.
cagtagcttacagaacgcaaacaa---g-----------------
>gnl|SRA|SRR1171870.47429366.1:76-100 HWI-ST967_77:2:1202:20660:175694.
cagtagcttacagaacgcaaacaa---g-----------------
>gnl|SRA|SRR1171870.37636341.1:76-100 HWI-ST967_77:2:1201:5128:5665.
cagtagcttacagaacgcaaacaa---g-----------------
>gnl|SRA|SRR1171870.28294188.2:76-100 HWI-ST967_77:2:1106:14613:61564.
cagtagcttacagaacgcaaacaa---g-----------------
>gnl|SRA|SRR1171870.15555040.2:76-100 HWI-ST967_77:2:1103:13114:194955.
cagtagcttacagaacgcaaacaa---g-----------------
>gnl|SRA|SRR1171870.15537287.2:47-71 HWI-ST967_77:2:1103:1305:194377.
cagtagcttacagaacgcaaacaa---g-----------------
>gnl|SRA|SRR1171870.15115721.1:76-100 HWI-ST967_77:2:1103:16415:178519.

cagtagcttacagaacgcaaacaa---g-----------------
>gnl|SRA|SRR1171870.13079679.2:55-79 HWI-ST967_77:2:1103:20608:102891.
cagtagcttacagaacgcaaacaa---g-----------------
>_R_gnl|SRA|SRR1171870.13079679.1:76-100 HWI-ST967_77:2:1103:20608:102891.
cagtagcttacagaacgcaaacaa---g-----------------
>_R_gnl|SRA|SRR1171870.12366796.1:1-25 HWI-ST967_77:2:1103:13669:76395.
cagtagcttacagaacgcaaacaa---g-----------------
>_R_gnl|SRA|SRR1171870.10527784.1:20-44 HWI-ST967_77:2:1103:20092:8229.
cagtagcttacagaacgcaaacaa---g-----------------
>gnl|SRA|SRR1171870.7882101.2:22-46 HWI-ST967_77:2:1102:19644:104182.
cagtagcttacagaacgcaaacaa---g-----------------
>_R_gnl|SRA|SRR1171870.4918020.1:1-25 HWI-ST967_77:2:1101:1850:189574.
cagtagcttacagaacgcaaacaa---g-----------------
>_R_gnl|SRA|SRR1171870.353770.1:1-25 HWI-ST967_77:2:1101:15870:15592.
cagtagcttacagaacgcaaacaa---g-----------------
>gnl|SRA|SRR1171870.146207591.2:76-100 HWI-ST967_77:2:2201:18497:94523.
cagtagcatacagaacgcaaacaa---g-----------------
>gnl|SRA|SRR1171870.76797583.1:76-100 HWI-ST967_77:2:1301:11139:137247.
cagtagcttaaagaacgcaaacaa---g-----------------
>gnl|SRA|SRR1171870.32905836.2:76-100 HWI-ST967_77:2:1107:6937:35042.
cagtagcttacagaactcaaacaa---g-----------------
>_R_gnl|SRA|SRR1171870.104367801.2:49-89 HWI-ST967_77:2:1306:4407:142487.
cagtagcttaaagaacgcaaacaa---gacccgcgggggtgatt-
>_R_gnl|SRA|SRR1171870.143778413.1:47-86 HWI-ST967_77:2:2108:3987:185742.
caggagcttacagcacgcaaacaa---gaccatagggggggat--
>gnl|SRA|SRR1171870.647339.1:58-99 HWI-ST967_77:2:1101:4735:27169.
cagtagcttacagaacgcaaacaaaa---ccatagtgggggattc
>gnl|SRA|SRR1171870.68332648.2:3-44 HWI-ST967_77:2:1206:15375:134640.
caggagcttacagaacgcaaacaata---ccatagttggggattc
>_R_gnl|SRA|SRR1171870.130137955.1:67-100 HWI-ST967_77:2:2104:20955:144558.
--------tacagaacgcaaacaaaa---ccatagtgggggattc
>_R_gnl|SRA|SRR1171870.209104001.1:36-80 HWI-ST967_77:2:2307:11332:25649.
cagtagcttacagaacgcaaacaataggaccatagtgggggattc
>_R_gnl|SRA|SRR1171870.208843823.1:51-95 HWI-ST967_77:2:2306:2289:194626.
cagtagcttacagaacgcaaacaataggaccatagtgggggattc
>_R_gnl|SRA|SRR1171870.207578116.1:13-57 HWI-ST967_77:2:2306:20650:145946.
cagtagcttacagaacgcaaacaataggaccatagtgggggattc
>_R_gnl|SRA|SRR1171870.206575226.1:6-50 HWI-ST967_77:2:2306:9115:107676.
cagtagcttacagaacgcaaacaataggaccatagtgggggattc
>_R_gnl|SRA|SRR1171870.206086462.1:6-50 HWI-ST967_77:2:2306:7568:89476.
cagtagcttacagaacgcaaacaataggaccatagtgggggattc
>gnl|SRA|SRR1171870.205103564.1:45-89 HWI-ST967_77:2:2306:1625:52913.
cagtagcttacagaacgcaaacaataggaccatagtgggggattc
>gnl|SRA|SRR1171870.205098982.1:39-83 HWI-ST967_77:2:2306:8001:52642.
cagtagcttacagaacgcaaacaataggaccatagtgggggattc
>_R_gnl|SRA|SRR1171870.204567363.1:26-70 HWI-ST967_77:2:2306:15133:33103.
cagtagcttacagaacgcaaacaataggaccatagtgggggattc
>_R_gnl|SRA|SRR1171870.203879537.1:24-68 HWI-ST967_77:2:2306:17606:7965.
cagtagcttacagaacgcaaacaataggaccatagtgggggattc
>_R_gnl|SRA|SRR1171870.203753478.1:24-68 HWI-ST967_77:2:2306:8060:3441.
cagtagcttacagaacgcaaacaataggaccatagtgggggattc

>_R_gnl|SRA|SRR1171870.203370021.2:46-90 HWI-ST967_77:2:2305:14289:187940.
cagtagcttacagaacgcaaacaataggaccatagtgggggattc
>_R_gnl|SRA|SRR1171870.203273507.2:52-96 HWI-ST967_77:2:2305:1188:184609.
cagtagcttacagaacgcaaacaataggaccatagtgggggattc
>_R_gnl|SRA|SRR1171870.202614788.1:33-77 HWI-ST967_77:2:2305:2475:161070.
cagtagcttacagaacgcaaacaataggaccatagtgggggattc
>_R_gnl|SRA|SRR1171870.202253147.1:46-90 HWI-ST967_77:2:2305:17407:147921.
cagtagcttacagaacgcaaacaataggaccatagtgggggattc
>gnl|SRA|SRR1171870.201382991.2:43-87 HWI-ST967_77:2:2305:18703:116064.
cagtagcttacagaacgcaaacaataggaccatagtgggggattc
>_R_gnl|SRA|SRR1171870.200970201.1:4-48 HWI-ST967_77:2:2305:3112:100920.
cagtagcttacagaacgcaaacaataggaccatagtgggggattc
>gnl|SRA|SRR1171870.200586995.2:40-84 HWI-ST967_77:2:2305:17362:85406.
cagtagcttacagaacgcaaacaataggaccatagtgggggattc
>gnl|SRA|SRR1171870.199381965.2:51-95 HWI-ST967_77:2:2305:2342:40117.
cagtagcttacagaacgcaaacaataggaccatagtgggggattc
>_R_gnl|SRA|SRR1171870.198972425.2:8-52 HWI-ST967_77:2:2305:12359:25116.
cagtagcttacagaacgcaaacaataggaccatagtgggggattc
>gnl|SRA|SRR1171870.198169221.2:50-94 HWI-ST967_77:2:2304:5097:195206.
cagtagcttacagaacgcaaacaataggaccatagtgggggattc
>_R_gnl|SRA|SRR1171870.198169221.1:35-79 HWI-ST967_77:2:2304:5097:195206.
cagtagcttacagaacgcaaacaataggaccatagtgggggattc
>_R_gnl|SRA|SRR1171870.197268179.1:3-47 HWI-ST967_77:2:2304:12087:163157.
cagtagcttacagaacgcaaacaataggaccatagtgggggattc
>gnl|SRA|SRR1171870.194571896.1:4-48 HWI-ST967_77:2:2304:2994:66110.
cagtagcttacagaacgcaaacaataggaccatagtgggggattc
>_R_gnl|SRA|SRR1171870.192092005.1:37-81 HWI-ST967_77:2:2303:12526:173631.
cagtagcttacagaacgcaaacaataggaccatagtgggggattc
>gnl|SRA|SRR1171870.192046162.1:9-53 HWI-ST967_77:2:2303:18341:171809.
cagtagcttacagaacgcaaacaataggaccatagtgggggattc
>gnl|SRA|SRR1171870.191086624.2:35-79 HWI-ST967_77:2:2303:12526:137167.
cagtagcttacagaacgcaaacaataggaccatagtgggggattc
>_R_gnl|SRA|SRR1171870.190969561.1:37-81 HWI-ST967_77:2:2303:13111:132813.
cagtagcttacagaacgcaaacaataggaccatagtgggggattc
>_R_gnl|SRA|SRR1171870.190361193.2:37-81 HWI-ST967_77:2:2303:1557:110843.
cagtagcttacagaacgcaaacaataggaccatagtgggggattc
>_R_gnl|SRA|SRR1171870.190347037.2:1-45 HWI-ST967_77:2:2303:19292:110122.
cagtagcttacagaacgcaaacaataggaccatagtgggggattc
>_R_gnl|SRA|SRR1171870.188289019.2:51-95 HWI-ST967_77:2:2303:18403:34714.
cagtagcttacagaacgcaaacaataggaccatagtgggggattc
>gnl|SRA|SRR1171870.188289019.1:39-83 HWI-ST967_77:2:2303:18403:34714.
cagtagcttacagaacgcaaacaataggaccatagtgggggattc
>_R_gnl|SRA|SRR1171870.187184390.1:33-77 HWI-ST967_77:2:2302:16567:192682.
cagtagcttacagaacgcaaacaataggaccatagtgggggattc
>_R_gnl|SRA|SRR1171870.186822931.2:3-47 HWI-ST967_77:2:2302:16082:179423.
cagtagcttacagaacgcaaacaataggaccatagtgggggattc
>_R_gnl|SRA|SRR1171870.186756559.1:37-81 HWI-ST967_77:2:2302:19494:176777.
cagtagcttacagaacgcaaacaataggaccatagtgggggattc
>_R_gnl|SRA|SRR1171870.186670517.1:25-69 HWI-ST967_77:2:2302:2942:173993.
cagtagcttacagaacgcaaacaataggaccatagtgggggattc
>_R_gnl|SRA|SRR1171870.183975202.2:11-55 HWI-ST967_77:2:2302:4311:70165.

cagtagcttacagaacgcaaacaataggaccatagtgggggattc
>_R_gnl|SRA|SRR1171870.183917239.1:10-54 HWI-ST967_77:2:2302:8849:67970.
cagtagcttacagaacgcaaacaataggaccatagtgggggattc
>_R_gnl|SRA|SRR1171870.183888956.1:46-90 HWI-ST967_77:2:2302:1625:66873.
cagtagcttacagaacgcaaacaataggaccatagtgggggattc
>_R_gnl|SRA|SRR1171870.181900396.1:38-82 HWI-ST967_77:2:2301:14159:190243.
cagtagcttacagaacgcaaacaataggaccatagtgggggattc
>gnl|SRA|SRR1171870.181046011.1:52-96 HWI-ST967_77:2:2301:16133:157596.
cagtagcttacagaacgcaaacaataggaccatagtgggggattc
>_R_gnl|SRA|SRR1171870.180713113.2:23-67 HWI-ST967_77:2:2301:5548:144780.
cagtagcttacagaacgcaaacaataggaccatagtgggggattc
>gnl|SRA|SRR1171870.180617919.1:13-57 HWI-ST967_77:2:2301:5178:141179.
cagtagcttacagaacgcaaacaataggaccatagtgggggattc
>_R_gnl|SRA|SRR1171870.180140487.2:36-80 HWI-ST967_77:2:2301:3794:122040.
cagtagcttacagaacgcaaacaataggaccatagtgggggattc
>_R_gnl|SRA|SRR1171870.178519139.2:17-61 HWI-ST967_77:2:2301:16257:59175.
cagtagcttacagaacgcaaacaataggaccatagtgggggattc
>gnl|SRA|SRR1171870.178291993.2:23-67 HWI-ST967_77:2:2301:15318:50356.
cagtagcttacagaacgcaaacaataggaccatagtgggggattc
>gnl|SRA|SRR1171870.178072245.1:39-83 HWI-ST967_77:2:2301:14915:41967.
cagtagcttacagaacgcaaacaataggaccatagtgggggattc
>_R_gnl|SRA|SRR1171870.177879648.1:53-97 HWI-ST967_77:2:2301:15075:34280.
cagtagcttacagaacgcaaacaataggaccatagtgggggattc
>gnl|SRA|SRR1171870.177500943.1:20-64 HWI-ST967_77:2:2301:16558:19592.
cagtagcttacagaacgcaaacaataggaccatagtgggggattc
>gnl|SRA|SRR1171870.175971850.1:51-95 HWI-ST967_77:2:2208:13798:13033.
cagtagcttacagaacgcaaacaataggaccatagtgggggattc
>_R_gnl|SRA|SRR1171870.175304527.1:53-97 HWI-ST967_77:2:2206:2845:190845.
cagtagcttacagaacgcaaacaataggaccatagtgggggattc
>gnl|SRA|SRR1171870.175039950.1:6-50 HWI-ST967_77:2:2206:19926:177899.
cagtagcttacagaacgcaaacaataggaccatagtgggggattc
>gnl|SRA|SRR1171870.174849188.2:50-94 HWI-ST967_77:2:2206:4356:168982.
cagtagcttacagaacgcaaacaataggaccatagtgggggattc
>_R_gnl|SRA|SRR1171870.173820300.1:36-80 HWI-ST967_77:2:2206:3577:121193.
cagtagcttacagaacgcaaacaataggaccatagtgggggattc
>_R_gnl|SRA|SRR1171870.172961409.1:25-69 HWI-ST967_77:2:2206:8762:83772.
cagtagcttacagaacgcaaacaataggaccatagtgggggattc
>gnl|SRA|SRR1171870.172163719.2:15-59 HWI-ST967_77:2:2206:11616:50602.
cagtagcttacagaacgcaaacaataggaccatagtgggggattc
>_R_gnl|SRA|SRR1171870.171623725.1:46-90 HWI-ST967_77:2:2206:6752:28548.
cagtagcttacagaacgcaaacaataggaccatagtgggggattc
>gnl|SRA|SRR1171870.170839357.1:44-88 HWI-ST967_77:2:2205:11726:195493.
cagtagcttacagaacgcaaacaataggaccatagtgggggattc
>gnl|SRA|SRR1171870.170345743.2:9-53 HWI-ST967_77:2:2205:6598:178194.
cagtagcttacagaacgcaaacaataggaccatagtgggggattc
>_R_gnl|SRA|SRR1171870.170006303.1:8-52 HWI-ST967_77:2:2205:3847:166217.
cagtagcttacagaacgcaaacaataggaccatagtgggggattc
>gnl|SRA|SRR1171870.169870692.2:17-61 HWI-ST967_77:2:2205:17820:161077.
cagtagcttacagaacgcaaacaataggaccatagtgggggattc
>_R_gnl|SRA|SRR1171870.169640807.1:5-49 HWI-ST967_77:2:2205:3013:153169.
cagtagcttacagaacgcaaacaataggaccatagtgggggattc

>_R_gnl|SRA|SRR1171870.169268646.1:32-76 HWI-ST967_77:2:2205:16067:139640.
cagtagcttacagaacgcaaacaataggaccatagtgggggattc
>gnl|SRA|SRR1171870.168940517.1:50-94 HWI-ST967_77:2:2205:16447:127976.
cagtagcttacagaacgcaaacaataggaccatagtgggggattc
>_R_gnl|SRA|SRR1171870.167532240.1:26-70 HWI-ST967_77:2:2205:14016:76002.
cagtagcttacagaacgcaaacaataggaccatagtgggggattc
>_R_gnl|SRA|SRR1171870.165932902.2:3-47 HWI-ST967_77:2:2205:20359:19015.
cagtagcttacagaacgcaaacaataggaccatagtgggggattc
>_R_gnl|SRA|SRR1171870.164604790.1:20-64 HWI-ST967_77:2:2204:13090:171234.
cagtagcttacagaacgcaaacaataggaccatagtgggggattc
>gnl|SRA|SRR1171870.163266146.2:37-81 HWI-ST967_77:2:2204:2763:123444.
cagtagcttacagaacgcaaacaataggaccatagtgggggattc
>gnl|SRA|SRR1171870.162559036.1:23-67 HWI-ST967_77:2:2204:7630:98200.
cagtagcttacagaacgcaaacaataggaccatagtgggggattc
>gnl|SRA|SRR1171870.162384676.2:19-63 HWI-ST967_77:2:2204:11526:91954.
cagtagcttacagaacgcaaacaataggaccatagtgggggattc
>gnl|SRA|SRR1171870.160872617.2:38-82 HWI-ST967_77:2:2204:7678:38121.
cagtagcttacagaacgcaaacaataggaccatagtgggggattc
>_R_gnl|SRA|SRR1171870.160872617.1:51-95 HWI-ST967_77:2:2204:7678:38121.
cagtagcttacagaacgcaaacaataggaccatagtgggggattc
>_R_gnl|SRA|SRR1171870.160614058.2:16-60 HWI-ST967_77:2:2204:18694:28757.
cagtagcttacagaacgcaaacaataggaccatagtgggggattc
>gnl|SRA|SRR1171870.158564923.2:20-64 HWI-ST967_77:2:2203:16940:153878.
cagtagcttacagaacgcaaacaataggaccatagtgggggattc
>gnl|SRA|SRR1171870.157625930.2:17-61 HWI-ST967_77:2:2203:9918:120391.
cagtagcttacagaacgcaaacaataggaccatagtgggggattc
>gnl|SRA|SRR1171870.157499448.2:49-93 HWI-ST967_77:2:2203:5567:115978.
cagtagcttacagaacgcaaacaataggaccatagtgggggattc
>_R_gnl|SRA|SRR1171870.157499448.1:41-85 HWI-ST967_77:2:2203:5567:115978.
cagtagcttacagaacgcaaacaataggaccatagtgggggattc
>gnl|SRA|SRR1171870.157031923.2:31-75 HWI-ST967_77:2:2203:9833:98959.
cagtagcttacagaacgcaaacaataggaccatagtgggggattc
>_R_gnl|SRA|SRR1171870.156400113.2:53-97 HWI-ST967_77:2:2203:2824:76132.
cagtagcttacagaacgcaaacaataggaccatagtgggggattc
>_R_gnl|SRA|SRR1171870.156065255.2:35-79 HWI-ST967_77:2:2203:15479:63881.
cagtagcttacagaacgcaaacaataggaccatagtgggggattc
>gnl|SRA|SRR1171870.155815245.2:1-45 HWI-ST967_77:2:2203:12532:54950.
cagtagcttacagaacgcaaacaataggaccatagtgggggattc
>gnl|SRA|SRR1171870.155202928.2:1-45 HWI-ST967_77:2:2203:9953:32958.
cagtagcttacagaacgcaaacaataggaccatagtgggggattc
>_R_gnl|SRA|SRR1171870.154569153.1:2-46 HWI-ST967_77:2:2203:11452:10191.
cagtagcttacagaacgcaaacaataggaccatagtgggggattc
>_R_gnl|SRA|SRR1171870.154385351.1:50-94 HWI-ST967_77:2:2203:7010:3521.
cagtagcttacagaacgcaaacaataggaccatagtgggggattc
>_R_gnl|SRA|SRR1171870.154290215.1:11-55 HWI-ST967_77:2:2202:12399:198336.
cagtagcttacagaacgcaaacaataggaccatagtgggggattc
>_R_gnl|SRA|SRR1171870.154227410.2:36-80 HWI-ST967_77:2:2202:13739:196094.
cagtagcttacagaacgcaaacaataggaccatagtgggggattc
>_R_gnl|SRA|SRR1171870.154220017.1:50-94 HWI-ST967_77:2:2202:12542:195906.
cagtagcttacagaacgcaaacaataggaccatagtgggggattc
>_R_gnl|SRA|SRR1171870.153110165.1:18-62 HWI-ST967_77:2:2202:7331:155784.

cagtagcttacagaacgcaaacaataggaccatagtgggggattc
>gnl|SRA|SRR1171870.152941758.2:23-67 HWI-ST967_77:2:2202:11408:149506.
cagtagcttacagaacgcaaacaataggaccatagtgggggattc
>gnl|SRA|SRR1171870.151815836.2:45-89 HWI-ST967_77:2:2202:16392:107489.
cagtagcttacagaacgcaaacaataggaccatagtgggggattc
>_R_gnl|SRA|SRR1171870.151618809.2:38-82 HWI-ST967_77:2:2202:2150:100056.
cagtagcttacagaacgcaaacaataggaccatagtgggggattc
>_R_gnl|SRA|SRR1171870.151156518.2:23-67 HWI-ST967_77:2:2202:2748:82617.
cagtagcttacagaacgcaaacaataggaccatagtgggggattc
>_R_gnl|SRA|SRR1171870.150998331.1:18-62 HWI-ST967_77:2:2202:9059:76624.
cagtagcttacagaacgcaaacaataggaccatagtgggggattc
>_R_gnl|SRA|SRR1171870.150744783.1:43-87 HWI-ST967_77:2:2202:14464:67217.
cagtagcttacagaacgcaaacaataggaccatagtgggggattc
>_R_gnl|SRA|SRR1171870.149838263.1:23-67 HWI-ST967_77:2:2202:7025:33637.
cagtagcttacagaacgcaaacaataggaccatagtgggggattc
>_R_gnl|SRA|SRR1171870.149242379.1:18-62 HWI-ST967_77:2:2202:5383:11585.
cagtagcttacagaacgcaaacaataggaccatagtgggggattc
>_R_gnl|SRA|SRR1171870.148265674.2:32-76 HWI-ST967_77:2:2201:14258:173576.
cagtagcttacagaacgcaaacaataggaccatagtgggggattc
>_R_gnl|SRA|SRR1171870.147384429.1:51-95 HWI-ST967_77:2:2201:6305:140326.
cagtagcttacagaacgcaaacaataggaccatagtgggggattc
>_R_gnl|SRA|SRR1171870.147228819.2:43-87 HWI-ST967_77:2:2201:3092:134385.
cagtagcttacagaacgcaaacaataggaccatagtgggggattc
>_R_gnl|SRA|SRR1171870.146292888.2:51-95 HWI-ST967_77:2:2201:1638:98046.
cagtagcttacagaacgcaaacaataggaccatagtgggggattc
>gnl|SRA|SRR1171870.145864398.2:34-78 HWI-ST967_77:2:2201:7419:81518.
cagtagcttacagaacgcaaacaataggaccatagtgggggattc
>_R_gnl|SRA|SRR1171870.145864398.1:50-94 HWI-ST967_77:2:2201:7419:81518.
cagtagcttacagaacgcaaacaataggaccatagtgggggattc
>_R_gnl|SRA|SRR1171870.144859104.2:5-49 HWI-ST967_77:2:2201:12926:43263.
cagtagcttacagaacgcaaacaataggaccatagtgggggattc
>_R_gnl|SRA|SRR1171870.144422063.2:10-54 HWI-ST967_77:2:2201:11364:26733.
cagtagcttacagaacgcaaacaataggaccatagtgggggattc
>gnl|SRA|SRR1171870.143921409.1:45-89 HWI-ST967_77:2:2201:8855:7284.
cagtagcttacagaacgcaaacaataggaccatagtgggggattc
>gnl|SRA|SRR1171870.142272315.2:30-74 HWI-ST967_77:2:2106:4424:195192.
cagtagcttacagaacgcaaacaataggaccatagtgggggattc
>gnl|SRA|SRR1171870.141001410.1:25-69 HWI-ST967_77:2:2106:10230:147527.
cagtagcttacagaacgcaaacaataggaccatagtgggggattc
>_R_gnl|SRA|SRR1171870.140858069.1:50-94 HWI-ST967_77:2:2106:5062:142365.
cagtagcttacagaacgcaaacaataggaccatagtgggggattc
>_R_gnl|SRA|SRR1171870.140171125.2:51-95 HWI-ST967_77:2:2106:9920:116650.
cagtagcttacagaacgcaaacaataggaccatagtgggggattc
>_R_gnl|SRA|SRR1171870.139755521.1:14-58 HWI-ST967_77:2:2106:7393:101140.
cagtagcttacagaacgcaaacaataggaccatagtgggggattc
>_R_gnl|SRA|SRR1171870.139227403.1:51-95 HWI-ST967_77:2:2106:4742:81653.
cagtagcttacagaacgcaaacaataggaccatagtgggggattc
>gnl|SRA|SRR1171870.139180055.1:14-58 HWI-ST967_77:2:2106:6730:79835.
cagtagcttacagaacgcaaacaataggaccatagtgggggattc
>_R_gnl|SRA|SRR1171870.138494975.1:9-53 HWI-ST967_77:2:2106:10481:54969.
cagtagcttacagaacgcaaacaataggaccatagtgggggattc

>_R_gnl|SRA|SRR1171870.138256137.1:46-90 HWI-ST967_77:2:2106:15661:46007.
cagtagcttacagaacgcaaacaataggaccatagtgggggattc
>_R_gnl|SRA|SRR1171870.138009472.1:26-70 HWI-ST967_77:2:2106:19754:37032.
cagtagcttacagaacgcaaacaataggaccatagtgggggattc
>gnl|SRA|SRR1171870.137462635.2:20-64 HWI-ST967_77:2:2106:1352:17361.
cagtagcttacagaacgcaaacaataggaccatagtgggggattc
>_R_gnl|SRA|SRR1171870.137245389.1:13-57 HWI-ST967_77:2:2106:11204:9274.
cagtagcttacagaacgcaaacaataggaccatagtgggggattc
>_R_gnl|SRA|SRR1171870.136000042.2:43-87 HWI-ST967_77:2:2105:11836:162152.
cagtagcttacagaacgcaaacaataggaccatagtgggggattc
>_R_gnl|SRA|SRR1171870.135178068.1:48-92 HWI-ST967_77:2:2105:15167:132051.
cagtagcttacagaacgcaaacaataggaccatagtgggggattc
>gnl|SRA|SRR1171870.135086881.2:56-100 HWI-ST967_77:2:2105:16731:128535.
cagtagcttacagaacgcaaacaataggaccatagtgggggattc
>_R_gnl|SRA|SRR1171870.135086881.1:56-100 HWI-ST967_77:2:2105:16731:128535.
cagtagcttacagaacgcaaacaataggaccatagtgggggattc
>_R_gnl|SRA|SRR1171870.134712299.2:5-49 HWI-ST967_77:2:2105:14811:114598.
cagtagcttacagaacgcaaacaataggaccatagtgggggattc
>_R_gnl|SRA|SRR1171870.132180270.2:18-62 HWI-ST967_77:2:2105:9191:20425.
cagtagcttacagaacgcaaacaataggaccatagtgggggattc
>_R_gnl|SRA|SRR1171870.130377618.1:35-79 HWI-ST967_77:2:2104:2528:153618.
cagtagcttacagaacgcaaacaataggaccatagtgggggattc
>gnl|SRA|SRR1171870.130306031.2:30-74 HWI-ST967_77:2:2104:11613:150951.
cagtagcttacagaacgcaaacaataggaccatagtgggggattc
>_R_gnl|SRA|SRR1171870.129735915.1:7-51 HWI-ST967_77:2:2104:11487:129891.
cagtagcttacagaacgcaaacaataggaccatagtgggggattc
>gnl|SRA|SRR1171870.129654912.1:4-48 HWI-ST967_77:2:2104:13063:126765.
cagtagcttacagaacgcaaacaataggaccatagtgggggattc
>_R_gnl|SRA|SRR1171870.129357468.1:53-97 HWI-ST967_77:2:2104:13769:115963.
cagtagcttacagaacgcaaacaataggaccatagtgggggattc
>_R_gnl|SRA|SRR1171870.128463075.1:36-80 HWI-ST967_77:2:2104:7911:82801.
cagtagcttacagaacgcaaacaataggaccatagtgggggattc
>gnl|SRA|SRR1171870.127327589.1:38-82 HWI-ST967_77:2:2104:14870:41044.
cagtagcttacagaacgcaaacaataggaccatagtgggggattc
>_R_gnl|SRA|SRR1171870.127209504.2:4-48 HWI-ST967_77:2:2104:7566:36961.
cagtagcttacagaacgcaaacaataggaccatagtgggggattc
>_R_gnl|SRA|SRR1171870.126971410.2:2-46 HWI-ST967_77:2:2104:8832:28144.
cagtagcttacagaacgcaaacaataggaccatagtgggggattc
>_R_gnl|SRA|SRR1171870.125290451.2:21-65 HWI-ST967_77:2:2103:11240:164279.
cagtagcttacagaacgcaaacaataggaccatagtgggggattc
>_R_gnl|SRA|SRR1171870.125261975.2:17-61 HWI-ST967_77:2:2103:6665:163369.
cagtagcttacagaacgcaaacaataggaccatagtgggggattc
>gnl|SRA|SRR1171870.125241614.2:37-81 HWI-ST967_77:2:2103:6296:162589.
cagtagcttacagaacgcaaacaataggaccatagtgggggattc
>gnl|SRA|SRR1171870.125143727.2:19-63 HWI-ST967_77:2:2103:16466:158859.
cagtagcttacagaacgcaaacaataggaccatagtgggggattc
>_R_gnl|SRA|SRR1171870.124449581.2:17-61 HWI-ST967_77:2:2103:16906:133287.
cagtagcttacagaacgcaaacaataggaccatagtgggggattc
>_R_gnl|SRA|SRR1171870.124399872.2:51-95 HWI-ST967_77:2:2103:10428:131574.
cagtagcttacagaacgcaaacaataggaccatagtgggggattc
>_R_gnl|SRA|SRR1171870.123585113.1:21-65 HWI-ST967_77:2:2103:16282:101474.

cagtagcttacagaacgcaaacaataggaccatagtgggggattc
>_R_gnl|SRA|SRR1171870.122845774.2:24-68 HWI-ST967_77:2:2103:16982:73904.
cagtagcttacagaacgcaaacaataggaccatagtgggggattc
>_R_gnl|SRA|SRR1171870.121923027.1:26-70 HWI-ST967_77:2:2103:18232:39316.
cagtagcttacagaacgcaaacaataggaccatagtgggggattc
>_R_gnl|SRA|SRR1171870.120423622.1:2-46 HWI-ST967_77:2:2102:19769:181808.
cagtagcttacagaacgcaaacaataggaccatagtgggggattc
>_R_gnl|SRA|SRR1171870.119619854.2:2-46 HWI-ST967_77:2:2102:3051:151700.
cagtagcttacagaacgcaaacaataggaccatagtgggggattc
>_R_gnl|SRA|SRR1171870.119169875.2:52-96 HWI-ST967_77:2:2102:17867:134088.
cagtagcttacagaacgcaaacaataggaccatagtgggggattc
>_R_gnl|SRA|SRR1171870.118353837.2:41-85 HWI-ST967_77:2:2102:17657:102508.
cagtagcttacagaacgcaaacaataggaccatagtgggggattc
>_R_gnl|SRA|SRR1171870.117894238.1:22-66 HWI-ST967_77:2:2102:7136:84953.
cagtagcttacagaacgcaaacaataggaccatagtgggggattc
>_R_gnl|SRA|SRR1171870.117726468.1:22-66 HWI-ST967_77:2:2102:7780:78437.
cagtagcttacagaacgcaaacaataggaccatagtgggggattc
>gnl|SRA|SRR1171870.117367790.1:23-67 HWI-ST967_77:2:2102:6682:64744.
cagtagcttacagaacgcaaacaataggaccatagtgggggattc
>_R_gnl|SRA|SRR1171870.117210120.2:51-95 HWI-ST967_77:2:2102:6331:58614.
cagtagcttacagaacgcaaacaataggaccatagtgggggattc
>_R_gnl|SRA|SRR1171870.117183071.1:53-97 HWI-ST967_77:2:2102:4261:57576.
cagtagcttacagaacgcaaacaataggaccatagtgggggattc
>_R_gnl|SRA|SRR1171870.117061892.2:2-46 HWI-ST967_77:2:2102:17109:52937.
cagtagcttacagaacgcaaacaataggaccatagtgggggattc
>_R_gnl|SRA|SRR1171870.117026834.1:33-77 HWI-ST967_77:2:2102:11597:51666.
cagtagcttacagaacgcaaacaataggaccatagtgggggattc
>_R_gnl|SRA|SRR1171870.116536053.1:26-70 HWI-ST967_77:2:2102:17754:32878.
cagtagcttacagaacgcaaacaataggaccatagtgggggattc
>gnl|SRA|SRR1171870.115907739.1:56-100 HWI-ST967_77:2:2102:5929:9153.
cagtagcttacagaacgcaaacaataggaccatagtgggggattc
>gnl|SRA|SRR1171870.115842309.1:6-50 HWI-ST967_77:2:2102:8144:6717.
cagtagcttacagaacgcaaacaataggaccatagtgggggattc
>_R_gnl|SRA|SRR1171870.115208058.2:20-64 HWI-ST967_77:2:2101:5328:180630.
cagtagcttacagaacgcaaacaataggaccatagtgggggattc
>gnl|SRA|SRR1171870.115208058.1:40-84 HWI-ST967_77:2:2101:5328:180630.
cagtagcttacagaacgcaaacaataggaccatagtgggggattc
>_R_gnl|SRA|SRR1171870.113832844.2:18-62 HWI-ST967_77:2:2101:4836:127272.
cagtagcttacagaacgcaaacaataggaccatagtgggggattc
>gnl|SRA|SRR1171870.113741314.1:50-94 HWI-ST967_77:2:2101:16956:123673.
cagtagcttacagaacgcaaacaataggaccatagtgggggattc
>gnl|SRA|SRR1171870.112929456.1:54-98 HWI-ST967_77:2:2101:16831:91880.
cagtagcttacagaacgcaaacaataggaccatagtgggggattc
>gnl|SRA|SRR1171870.112691160.2:50-94 HWI-ST967_77:2:2101:10301:82705.
cagtagcttacagaacgcaaacaataggaccatagtgggggattc
>_R_gnl|SRA|SRR1171870.112691160.1:13-57 HWI-ST967_77:2:2101:10301:82705.
cagtagcttacagaacgcaaacaataggaccatagtgggggattc
>gnl|SRA|SRR1171870.112134554.2:19-63 HWI-ST967_77:2:2101:4348:60982.
cagtagcttacagaacgcaaacaataggaccatagtgggggattc
>_R_gnl|SRA|SRR1171870.112134554.1:53-97 HWI-ST967_77:2:2101:4348:60982.
cagtagcttacagaacgcaaacaataggaccatagtgggggattc

>_R_gnl|SRA|SRR1171870.111882763.1:53-97 HWI-ST967_77:2:2101:13307:50969.
cagtagcttacagaacgcaaacaataggaccatagtgggggattc
>_R_gnl|SRA|SRR1171870.111519157.2:52-96 HWI-ST967_77:2:2101:9020:36675.
cagtagcttacagaacgcaaacaataggaccatagtgggggattc
>_R_gnl|SRA|SRR1171870.111108465.1:22-66 HWI-ST967_77:2:2101:13714:20334.
cagtagcttacagaacgcaaacaataggaccatagtgggggattc
>_R_gnl|SRA|SRR1171870.110933497.1:4-48 HWI-ST967_77:2:2101:2109:13588.
cagtagcttacagaacgcaaacaataggaccatagtgggggattc
>_R_gnl|SRA|SRR1171870.107273024.1:50-94 HWI-ST967_77:2:1307:16629:74409.
cagtagcttacagaacgcaaacaataggaccatagtgggggattc
>gnl|SRA|SRR1171870.104344140.1:1-45 HWI-ST967_77:2:1306:15766:141303.
cagtagcttacagaacgcaaacaataggaccatagtgggggattc
>_R_gnl|SRA|SRR1171870.103846135.1:52-96 HWI-ST967_77:2:1306:8027:123302.
cagtagcttacagaacgcaaacaataggaccatagtgggggattc
>gnl|SRA|SRR1171870.102651050.1:34-78 HWI-ST967_77:2:1306:10834:79871.
cagtagcttacagaacgcaaacaataggaccatagtgggggattc
>gnl|SRA|SRR1171870.101301672.1:30-74 HWI-ST967_77:2:1306:3873:32026.
cagtagcttacagaacgcaaacaataggaccatagtgggggattc
>_R_gnl|SRA|SRR1171870.100759245.1:37-81 HWI-ST967_77:2:1306:11417:13152.
cagtagcttacagaacgcaaacaataggaccatagtgggggattc
>_R_gnl|SRA|SRR1171870.100655267.1:26-70 HWI-ST967_77:2:1306:21019:9495.
cagtagcttacagaacgcaaacaataggaccatagtgggggattc
>_R_gnl|SRA|SRR1171870.100207666.2:51-95 HWI-ST967_77:2:1305:8433:192105.
cagtagcttacagaacgcaaacaataggaccatagtgggggattc
>_R_gnl|SRA|SRR1171870.99785228.1:6-50 HWI-ST967_77:2:1305:7670:177382.
cagtagcttacagaacgcaaacaataggaccatagtgggggattc
>_R_gnl|SRA|SRR1171870.99722352.1:54-98 HWI-ST967_77:2:1305:10510:175096.
cagtagcttacagaacgcaaacaataggaccatagtgggggattc
>_R_gnl|SRA|SRR1171870.99478527.1:25-69 HWI-ST967_77:2:1305:7180:166662.
cagtagcttacagaacgcaaacaataggaccatagtgggggattc
>_R_gnl|SRA|SRR1171870.98217704.1:22-66 HWI-ST967_77:2:1305:9195:122312.
cagtagcttacagaacgcaaacaataggaccatagtgggggattc
>_R_gnl|SRA|SRR1171870.97872138.2:32-76 HWI-ST967_77:2:1305:14177:110166.
cagtagcttacagaacgcaaacaataggaccatagtgggggattc
>_R_gnl|SRA|SRR1171870.97537932.2:18-62 HWI-ST967_77:2:1305:8580:97884.
cagtagcttacagaacgcaaacaataggaccatagtgggggattc
>_R_gnl|SRA|SRR1171870.96883938.1:2-46 HWI-ST967_77:2:1305:9406:72930.
cagtagcttacagaacgcaaacaataggaccatagtgggggattc
>_R_gnl|SRA|SRR1171870.95778078.1:19-63 HWI-ST967_77:2:1305:19630:33767.
cagtagcttacagaacgcaaacaataggaccatagtgggggattc
>gnl|SRA|SRR1171870.95562645.1:52-96 HWI-ST967_77:2:1305:17000:26405.
cagtagcttacagaacgcaaacaataggaccatagtgggggattc
>gnl|SRA|SRR1171870.95366759.2:56-100 HWI-ST967_77:2:1305:13842:19709.
cagtagcttacagaacgcaaacaataggaccatagtgggggattc
>_R_gnl|SRA|SRR1171870.95138215.1:50-94 HWI-ST967_77:2:1305:3857:11765.
cagtagcttacagaacgcaaacaataggaccatagtgggggattc
>gnl|SRA|SRR1171870.92755629.1:51-95 HWI-ST967_77:2:1304:6029:126863.
cagtagcttacagaacgcaaacaataggaccatagtgggggattc
>gnl|SRA|SRR1171870.92059474.2:15-59 HWI-ST967_77:2:1304:15136:102238.
cagtagcttacagaacgcaaacaataggaccatagtgggggattc
>gnl|SRA|SRR1171870.91640557.1:17-61 HWI-ST967_77:2:1304:12432:87010.

cagtagcttacagaacgcaaacaataggaccatagtgggggattc
>_R_gnl|SRA|SRR1171870.91605628.2:33-77 HWI-ST967_77:2:1304:11179:86000.
cagtagcttacagaacgcaaacaataggaccatagtgggggattc
>_R_gnl|SRA|SRR1171870.89455124.2:5-49 HWI-ST967_77:2:1304:11297:9383.
cagtagcttacagaacgcaaacaataggaccatagtgggggattc
>_R_gnl|SRA|SRR1171870.89413523.2:13-57 HWI-ST967_77:2:1304:13070:7872.
cagtagcttacagaacgcaaacaataggaccatagtgggggattc
>_R_gnl|SRA|SRR1171870.89260364.1:54-98 HWI-ST967_77:2:1304:19748:2472.
cagtagcttacagaacgcaaacaataggaccatagtgggggattc
>gnl|SRA|SRR1171870.88307183.1:1-45 HWI-ST967_77:2:1303:5308:167063.
cagtagcttacagaacgcaaacaataggaccatagtgggggattc
>gnl|SRA|SRR1171870.87334903.2:21-65 HWI-ST967_77:2:1303:11111:132378.
cagtagcttacagaacgcaaacaataggaccatagtgggggattc
>gnl|SRA|SRR1171870.87121296.2:23-67 HWI-ST967_77:2:1303:20220:124630.
cagtagcttacagaacgcaaacaataggaccatagtgggggattc
>_R_gnl|SRA|SRR1171870.85454718.2:32-76 HWI-ST967_77:2:1303:3091:64463.
cagtagcttacagaacgcaaacaataggaccatagtgggggattc
>gnl|SRA|SRR1171870.85416799.2:3-47 HWI-ST967_77:2:1303:13765:62918.
cagtagcttacagaacgcaaacaataggaccatagtgggggattc
>gnl|SRA|SRR1171870.84734927.2:54-98 HWI-ST967_77:2:1303:1532:38292.
cagtagcttacagaacgcaaacaataggaccatagtgggggattc
>_R_gnl|SRA|SRR1171870.84158447.1:51-95 HWI-ST967_77:2:1303:15376:17319.
cagtagcttacagaacgcaaacaataggaccatagtgggggattc
>gnl|SRA|SRR1171870.83894039.2:44-88 HWI-ST967_77:2:1303:13502:7776.
cagtagcttacagaacgcaaacaataggaccatagtgggggattc
>_R_gnl|SRA|SRR1171870.83894039.1:48-92 HWI-ST967_77:2:1303:13502:7776.
cagtagcttacagaacgcaaacaataggaccatagtgggggattc
>_R_gnl|SRA|SRR1171870.83789822.2:16-60 HWI-ST967_77:2:1303:15478:4234.
cagtagcttacagaacgcaaacaataggaccatagtgggggattc
>_R_gnl|SRA|SRR1171870.83633854.1:43-87 HWI-ST967_77:2:1302:5429:196839.
cagtagcttacagaacgcaaacaataggaccatagtgggggattc
>gnl|SRA|SRR1171870.81804648.2:54-98 HWI-ST967_77:2:1302:12219:128603.
cagtagcttacagaacgcaaacaataggaccatagtgggggattc
>_R_gnl|SRA|SRR1171870.81756680.2:37-81 HWI-ST967_77:2:1302:2422:126831.
cagtagcttacagaacgcaaacaataggaccatagtgggggattc
>gnl|SRA|SRR1171870.81756680.1:52-96 HWI-ST967_77:2:1302:2422:126831.
cagtagcttacagaacgcaaacaataggaccatagtgggggattc
>gnl|SRA|SRR1171870.81478203.1:56-100 HWI-ST967_77:2:1302:10302:115965.
cagtagcttacagaacgcaaacaataggaccatagtgggggattc
>_R_gnl|SRA|SRR1171870.81063060.1:27-71 HWI-ST967_77:2:1302:15860:99513.
cagtagcttacagaacgcaaacaataggaccatagtgggggattc
>gnl|SRA|SRR1171870.80920879.2:5-49 HWI-ST967_77:2:1302:10686:94129.
cagtagcttacagaacgcaaacaataggaccatagtgggggattc
>_R_gnl|SRA|SRR1171870.80845507.1:2-46 HWI-ST967_77:2:1302:14335:91218.
cagtagcttacagaacgcaaacaataggaccatagtgggggattc
>_R_gnl|SRA|SRR1171870.80040396.1:44-88 HWI-ST967_77:2:1302:17172:60221.
cagtagcttacagaacgcaaacaataggaccatagtgggggattc
>_R_gnl|SRA|SRR1171870.80031788.1:41-85 HWI-ST967_77:2:1302:11392:59887.
cagtagcttacagaacgcaaacaataggaccatagtgggggattc
>gnl|SRA|SRR1171870.79077998.1:44-88 HWI-ST967_77:2:1302:5099:24465.
cagtagcttacagaacgcaaacaataggaccatagtgggggattc

>_R_gnl|SRA|SRR1171870.77644702.1:50-94 HWI-ST967_77:2:1301:9348:169077.
cagtagcttacagaacgcaaacaataggaccatagtgggggattc
>gnl|SRA|SRR1171870.77615905.1:1-45 HWI-ST967_77:2:1301:2484:168143.
cagtagcttacagaacgcaaacaataggaccatagtgggggattc
>_R_gnl|SRA|SRR1171870.76298113.1:33-77 HWI-ST967_77:2:1301:11935:118117.
cagtagcttacagaacgcaaacaataggaccatagtgggggattc
>_R_gnl|SRA|SRR1171870.76213805.1:52-96 HWI-ST967_77:2:1301:13077:114904.
cagtagcttacagaacgcaaacaataggaccatagtgggggattc
>_R_gnl|SRA|SRR1171870.75155362.1:35-79 HWI-ST967_77:2:1301:5009:74289.
cagtagcttacagaacgcaaacaataggaccatagtgggggattc
>_R_gnl|SRA|SRR1171870.74462155.1:6-50 HWI-ST967_77:2:1301:19207:47677.
cagtagcttacagaacgcaaacaataggaccatagtgggggattc
>gnl|SRA|SRR1171870.74051702.2:52-96 HWI-ST967_77:2:1301:10746:31966.
cagtagcttacagaacgcaaacaataggaccatagtgggggattc
>_R_gnl|SRA|SRR1171870.74021530.2:35-79 HWI-ST967_77:2:1301:17884:30590.
cagtagcttacagaacgcaaacaataggaccatagtgggggattc
>_R_gnl|SRA|SRR1171870.73879927.2:10-54 HWI-ST967_77:2:1301:2469:25482.
cagtagcttacagaacgcaaacaataggaccatagtgggggattc
>_R_gnl|SRA|SRR1171870.70032711.2:51-95 HWI-ST967_77:2:1206:3142:198534.
cagtagcttacagaacgcaaacaataggaccatagtgggggattc
>gnl|SRA|SRR1171870.69965458.2:56-100 HWI-ST967_77:2:1206:15090:195863.
cagtagcttacagaacgcaaacaataggaccatagtgggggattc
>gnl|SRA|SRR1171870.68856591.2:49-93 HWI-ST967_77:2:1206:3373:154047.
cagtagcttacagaacgcaaacaataggaccatagtgggggattc
>_R_gnl|SRA|SRR1171870.68856591.1:52-96 HWI-ST967_77:2:1206:3373:154047.
cagtagcttacagaacgcaaacaataggaccatagtgggggattc
>gnl|SRA|SRR1171870.65043225.2:56-100 HWI-ST967_77:2:1206:18354:14991.
cagtagcttacagaacgcaaacaataggaccatagtgggggattc
>_R_gnl|SRA|SRR1171870.64445817.2:13-57 HWI-ST967_77:2:1205:12104:191750.
cagtagcttacagaacgcaaacaataggaccatagtgggggattc
>_R_gnl|SRA|SRR1171870.64251036.1:13-57 HWI-ST967_77:2:1205:16102:184910.
cagtagcttacagaacgcaaacaataggaccatagtgggggattc
>_R_gnl|SRA|SRR1171870.63928075.1:3-47 HWI-ST967_77:2:1205:9893:173302.
cagtagcttacagaacgcaaacaataggaccatagtgggggattc
>_R_gnl|SRA|SRR1171870.63722463.1:52-96 HWI-ST967_77:2:1205:19402:165906.
cagtagcttacagaacgcaaacaataggaccatagtgggggattc
>gnl|SRA|SRR1171870.63111663.2:40-84 HWI-ST967_77:2:1205:18779:143873.
cagtagcttacagaacgcaaacaataggaccatagtgggggattc
>_R_gnl|SRA|SRR1171870.63111663.1:52-96 HWI-ST967_77:2:1205:18779:143873.
cagtagcttacagaacgcaaacaataggaccatagtgggggattc
>_R_gnl|SRA|SRR1171870.63046459.1:6-50 HWI-ST967_77:2:1205:11181:141603.
cagtagcttacagaacgcaaacaataggaccatagtgggggattc
>gnl|SRA|SRR1171870.62524556.2:22-66 HWI-ST967_77:2:1205:14010:122860.
cagtagcttacagaacgcaaacaataggaccatagtgggggattc
>_R_gnl|SRA|SRR1171870.61519464.1:19-63 HWI-ST967_77:2:1205:15842:86123.
cagtagcttacagaacgcaaacaataggaccatagtgggggattc
>gnl|SRA|SRR1171870.59697069.2:56-100 HWI-ST967_77:2:1205:14752:20726.
cagtagcttacagaacgcaaacaataggaccatagtgggggattc
>_R_gnl|SRA|SRR1171870.59697069.1:51-95 HWI-ST967_77:2:1205:14752:20726.
cagtagcttacagaacgcaaacaataggaccatagtgggggattc
>_R_gnl|SRA|SRR1171870.59307224.1:8-52 HWI-ST967_77:2:1205:15363:6894.

cagtagcttacagaacgcaaacaataggaccatagtgggggattc
>gnl|SRA|SRR1171870.57714850.2:20-64 HWI-ST967_77:2:1204:1487:149234.
cagtagcttacagaacgcaaacaataggaccatagtgggggattc
>_R_gnl|SRA|SRR1171870.57180067.1:23-67 HWI-ST967_77:2:1204:3514:129758.
cagtagcttacagaacgcaaacaataggaccatagtgggggattc
>_R_gnl|SRA|SRR1171870.56986881.1:53-97 HWI-ST967_77:2:1204:2742:122880.
cagtagcttacagaacgcaaacaataggaccatagtgggggattc
>_R_gnl|SRA|SRR1171870.56915040.1:5-49 HWI-ST967_77:2:1204:12729:120141.
cagtagcttacagaacgcaaacaataggaccatagtgggggattc
>_R_gnl|SRA|SRR1171870.56818816.2:52-96 HWI-ST967_77:2:1204:12502:116620.
cagtagcttacagaacgcaaacaataggaccatagtgggggattc
>_R_gnl|SRA|SRR1171870.55945352.1:53-97 HWI-ST967_77:2:1204:4222:84985.
cagtagcttacagaacgcaaacaataggaccatagtgggggattc
>gnl|SRA|SRR1171870.55793257.1:52-96 HWI-ST967_77:2:1204:15733:79119.
cagtagcttacagaacgcaaacaataggaccatagtgggggattc
>gnl|SRA|SRR1171870.54673505.1:52-96 HWI-ST967_77:2:1204:15502:38884.
cagtagcttacagaacgcaaacaataggaccatagtgggggattc
>gnl|SRA|SRR1171870.54438821.2:48-92 HWI-ST967_77:2:1204:9436:30687.
cagtagcttacagaacgcaaacaataggaccatagtgggggattc
>_R_gnl|SRA|SRR1171870.53748088.1:50-94 HWI-ST967_77:2:1204:5288:6210.
cagtagcttacagaacgcaaacaataggaccatagtgggggattc
>_R_gnl|SRA|SRR1171870.53719371.1:22-66 HWI-ST967_77:2:1204:4732:5009.
cagtagcttacagaacgcaaacaataggaccatagtgggggattc
>gnl|SRA|SRR1171870.53578562.2:51-95 HWI-ST967_77:2:1203:1863:198264.
cagtagcttacagaacgcaaacaataggaccatagtgggggattc
>_R_gnl|SRA|SRR1171870.53092234.2:2-46 HWI-ST967_77:2:1203:4675:181034.
cagtagcttacagaacgcaaacaataggaccatagtgggggattc
>_R_gnl|SRA|SRR1171870.52458991.1:39-83 HWI-ST967_77:2:1203:9066:158714.
cagtagcttacagaacgcaaacaataggaccatagtgggggattc
>_R_gnl|SRA|SRR1171870.51969488.1:7-51 HWI-ST967_77:2:1203:12027:141097.
cagtagcttacagaacgcaaacaataggaccatagtgggggattc
>_R_gnl|SRA|SRR1171870.51945881.2:50-94 HWI-ST967_77:2:1203:5678:140343.
cagtagcttacagaacgcaaacaataggaccatagtgggggattc
>gnl|SRA|SRR1171870.51559801.1:37-81 HWI-ST967_77:2:1203:7391:126557.
cagtagcttacagaacgcaaacaataggaccatagtgggggattc
>_R_gnl|SRA|SRR1171870.50376909.1:52-96 HWI-ST967_77:2:1203:11388:83794.
cagtagcttacagaacgcaaacaataggaccatagtgggggattc
>gnl|SRA|SRR1171870.50322359.2:23-67 HWI-ST967_77:2:1203:12065:81796.
cagtagcttacagaacgcaaacaataggaccatagtgggggattc
>_R_gnl|SRA|SRR1171870.48731076.2:11-55 HWI-ST967_77:2:1203:17449:24477.
cagtagcttacagaacgcaaacaataggaccatagtgggggattc
>_R_gnl|SRA|SRR1171870.48630740.2:43-87 HWI-ST967_77:2:1203:9038:20901.
cagtagcttacagaacgcaaacaataggaccatagtgggggattc
>gnl|SRA|SRR1171870.48126295.1:35-79 HWI-ST967_77:2:1203:8697:2700.
cagtagcttacagaacgcaaacaataggaccatagtgggggattc
>_R_gnl|SRA|SRR1171870.46852111.1:22-66 HWI-ST967_77:2:1202:9283:154559.
cagtagcttacagaacgcaaacaataggaccatagtgggggattc
>gnl|SRA|SRR1171870.46373663.2:4-48 HWI-ST967_77:2:1202:1691:136772.
cagtagcttacagaacgcaaacaataggaccatagtgggggattc
>_R_gnl|SRA|SRR1171870.46105850.2:27-71 HWI-ST967_77:2:1202:19130:126747.
cagtagcttacagaacgcaaacaataggaccatagtgggggattc

>_R_gnl|SRA|SRR1171870.45835341.1:4-48 HWI-ST967_77:2:1202:3757:116511.
cagtagcttacagaacgcaaacaataggaccatagtgggggattc
>gnl|SRA|SRR1171870.45510351.2:34-78 HWI-ST967_77:2:1202:8108:104476.
cagtagcttacagaacgcaaacaataggaccatagtgggggattc
>_R_gnl|SRA|SRR1171870.45393125.1:13-57 HWI-ST967_77:2:1202:15940:99905.
cagtagcttacagaacgcaaacaataggaccatagtgggggattc
>gnl|SRA|SRR1171870.44083037.2:23-67 HWI-ST967_77:2:1202:9745:50783.
cagtagcttacagaacgcaaacaataggaccatagtgggggattc
>_R_gnl|SRA|SRR1171870.43436901.2:12-56 HWI-ST967_77:2:1202:4425:27069.
cagtagcttacagaacgcaaacaataggaccatagtgggggattc
>gnl|SRA|SRR1171870.42802632.1:20-64 HWI-ST967_77:2:1202:19407:3713.
cagtagcttacagaacgcaaacaataggaccatagtgggggattc
>_R_gnl|SRA|SRR1171870.42257053.1:10-54 HWI-ST967_77:2:1201:20461:181531.
cagtagcttacagaacgcaaacaataggaccatagtgggggattc
>_R_gnl|SRA|SRR1171870.41821231.2:50-94 HWI-ST967_77:2:1201:8699:165414.
cagtagcttacagaacgcaaacaataggaccatagtgggggattc
>gnl|SRA|SRR1171870.41821231.1:53-97 HWI-ST967_77:2:1201:8699:165414.
cagtagcttacagaacgcaaacaataggaccatagtgggggattc
>_R_gnl|SRA|SRR1171870.40789418.2:6-50 HWI-ST967_77:2:1201:3862:126387.
cagtagcttacagaacgcaaacaataggaccatagtgggggattc
>_R_gnl|SRA|SRR1171870.40598315.2:17-61 HWI-ST967_77:2:1201:18592:118820.
cagtagcttacagaacgcaaacaataggaccatagtgggggattc
>gnl|SRA|SRR1171870.40377519.2:22-66 HWI-ST967_77:2:1201:2163:110658.
cagtagcttacagaacgcaaacaataggaccatagtgggggattc
>gnl|SRA|SRR1171870.40163157.1:54-98 HWI-ST967_77:2:1201:20616:102167.
cagtagcttacagaacgcaaacaataggaccatagtgggggattc
>gnl|SRA|SRR1171870.38793394.2:34-78 HWI-ST967_77:2:1201:11862:49933.
cagtagcttacagaacgcaaacaataggaccatagtgggggattc
>_R_gnl|SRA|SRR1171870.38245916.2:36-80 HWI-ST967_77:2:1201:18472:28865.
cagtagcttacagaacgcaaacaataggaccatagtgggggattc
>gnl|SRA|SRR1171870.38245916.1:50-94 HWI-ST967_77:2:1201:18472:28865.
cagtagcttacagaacgcaaacaataggaccatagtgggggattc
>_R_gnl|SRA|SRR1171870.38054233.1:46-90 HWI-ST967_77:2:1201:5761:21612.
cagtagcttacagaacgcaaacaataggaccatagtgggggattc
>_R_gnl|SRA|SRR1171870.37619260.1:23-67 HWI-ST967_77:2:1201:12645:4904.
cagtagcttacagaacgcaaacaataggaccatagtgggggattc
>_R_gnl|SRA|SRR1171870.34676298.1:13-57 HWI-ST967_77:2:1107:8652:114518.
cagtagcttacagaacgcaaacaataggaccatagtgggggattc
>_R_gnl|SRA|SRR1171870.34608248.1:31-75 HWI-ST967_77:2:1107:13576:111311.
cagtagcttacagaacgcaaacaataggaccatagtgggggattc
>gnl|SRA|SRR1171870.32726351.1:1-45 HWI-ST967_77:2:1107:1133:27256.
cagtagcttacagaacgcaaacaataggaccatagtgggggattc
>_R_gnl|SRA|SRR1171870.31836103.2:23-67 HWI-ST967_77:2:1106:3418:189362.
cagtagcttacagaacgcaaacaataggaccatagtgggggattc
>_R_gnl|SRA|SRR1171870.31582166.1:25-69 HWI-ST967_77:2:1106:11657:180248.
cagtagcttacagaacgcaaacaataggaccatagtgggggattc
>gnl|SRA|SRR1171870.31509025.2:34-78 HWI-ST967_77:2:1106:2893:177607.
cagtagcttacagaacgcaaacaataggaccatagtgggggattc
>_R_gnl|SRA|SRR1171870.28668163.1:33-77 HWI-ST967_77:2:1106:13747:75239.
cagtagcttacagaacgcaaacaataggaccatagtgggggattc
>gnl|SRA|SRR1171870.28216397.2:50-94 HWI-ST967_77:2:1106:14934:58787.

cagtagcttacagaacgcaaacaataggaccatagtgggggattc
>_R_gnl|SRA|SRR1171870.28216397.1:13-57 HWI-ST967_77:2:1106:14934:58787.
cagtagcttacagaacgcaaacaataggaccatagtgggggattc
>_R_gnl|SRA|SRR1171870.28174187.1:48-92 HWI-ST967_77:2:1106:14657:57491.
cagtagcttacagaacgcaaacaataggaccatagtgggggattc
>gnl|SRA|SRR1171870.26952953.1:2-46 HWI-ST967_77:2:1106:20742:14386.
cagtagcttacagaacgcaaacaataggaccatagtgggggattc
>gnl|SRA|SRR1171870.26728885.2:11-55 HWI-ST967_77:2:1106:13463:6693.
cagtagcttacagaacgcaaacaataggaccatagtgggggattc
>gnl|SRA|SRR1171870.25587042.2:20-64 HWI-ST967_77:2:1105:15919:164702.
cagtagcttacagaacgcaaacaataggaccatagtgggggattc
>_R_gnl|SRA|SRR1171870.24167965.2:34-78 HWI-ST967_77:2:1105:9851:113532.
cagtagcttacagaacgcaaacaataggaccatagtgggggattc
>gnl|SRA|SRR1171870.24167965.1:56-100 HWI-ST967_77:2:1105:9851:113532.
cagtagcttacagaacgcaaacaataggaccatagtgggggattc
>gnl|SRA|SRR1171870.23893331.2:51-95 HWI-ST967_77:2:1105:7655:103808.
cagtagcttacagaacgcaaacaataggaccatagtgggggattc
>_R_gnl|SRA|SRR1171870.23712920.1:25-69 HWI-ST967_77:2:1105:10022:97309.
cagtagcttacagaacgcaaacaataggaccatagtgggggattc
>_R_gnl|SRA|SRR1171870.23702825.2:52-96 HWI-ST967_77:2:1105:1363:97196.
cagtagcttacagaacgcaaacaataggaccatagtgggggattc
>gnl|SRA|SRR1171870.23453436.1:30-74 HWI-ST967_77:2:1105:8023:88249.
cagtagcttacagaacgcaaacaataggaccatagtgggggattc
>gnl|SRA|SRR1171870.22409376.1:50-94 HWI-ST967_77:2:1105:4428:50729.
cagtagcttacagaacgcaaacaataggaccatagtgggggattc
>_R_gnl|SRA|SRR1171870.22313046.1:43-87 HWI-ST967_77:2:1105:8282:47116.
cagtagcttacagaacgcaaacaataggaccatagtgggggattc
>_R_gnl|SRA|SRR1171870.22043979.1:37-81 HWI-ST967_77:2:1105:12706:37340.
cagtagcttacagaacgcaaacaataggaccatagtgggggattc
>gnl|SRA|SRR1171870.20304948.1:14-58 HWI-ST967_77:2:1104:9675:172983.
cagtagcttacagaacgcaaacaataggaccatagtgggggattc
>_R_gnl|SRA|SRR1171870.19835518.2:32-76 HWI-ST967_77:2:1104:14301:155024.
cagtagcttacagaacgcaaacaataggaccatagtgggggattc
>_R_gnl|SRA|SRR1171870.19685345.1:5-49 HWI-ST967_77:2:1104:19249:149433.
cagtagcttacagaacgcaaacaataggaccatagtgggggattc
>_R_gnl|SRA|SRR1171870.18715801.2:52-96 HWI-ST967_77:2:1104:17099:112577.
cagtagcttacagaacgcaaacaataggaccatagtgggggattc
>gnl|SRA|SRR1171870.18715801.1:55-99 HWI-ST967_77:2:1104:17099:112577.
cagtagcttacagaacgcaaacaataggaccatagtgggggattc
>_R_gnl|SRA|SRR1171870.18628171.1:14-58 HWI-ST967_77:2:1104:12337:109342.
cagtagcttacagaacgcaaacaataggaccatagtgggggattc
>_R_gnl|SRA|SRR1171870.18302045.1:43-87 HWI-ST967_77:2:1104:1718:97085.
cagtagcttacagaacgcaaacaataggaccatagtgggggattc
>_R_gnl|SRA|SRR1171870.18103870.2:18-62 HWI-ST967_77:2:1104:4339:89601.
cagtagcttacagaacgcaaacaataggaccatagtgggggattc
>_R_gnl|SRA|SRR1171870.17940281.2:17-61 HWI-ST967_77:2:1104:11418:83370.
cagtagcttacagaacgcaaacaataggaccatagtgggggattc
>_R_gnl|SRA|SRR1171870.16990646.2:16-60 HWI-ST967_77:2:1104:4284:48578.
cagtagcttacagaacgcaaacaataggaccatagtgggggattc
>gnl|SRA|SRR1171870.15691879.2:50-94 HWI-ST967_77:2:1103:17477:199772.
cagtagcttacagaacgcaaacaataggaccatagtgggggattc

>_R_gnl|SRA|SRR1171870.15691879.1:28-72 HWI-ST967_77:2:1103:17477:199772.
cagtagcttacagaacgcaaacaataggaccatagtgggggattc
>_R_gnl|SRA|SRR1171870.15033717.2:53-97 HWI-ST967_77:2:1103:13498:175623.
cagtagcttacagaacgcaaacaataggaccatagtgggggattc
>_R_gnl|SRA|SRR1171870.14875467.1:23-67 HWI-ST967_77:2:1103:5036:169855.
cagtagcttacagaacgcaaacaataggaccatagtgggggattc
>gnl|SRA|SRR1171870.14451802.2:4-48 HWI-ST967_77:2:1103:6058:154223.
cagtagcttacagaacgcaaacaataggaccatagtgggggattc
>gnl|SRA|SRR1171870.14168500.2:31-75 HWI-ST967_77:2:1103:2380:143684.
cagtagcttacagaacgcaaacaataggaccatagtgggggattc
>_R_gnl|SRA|SRR1171870.14118639.2:49-93 HWI-ST967_77:2:1103:13422:141669.
cagtagcttacagaacgcaaacaataggaccatagtgggggattc
>gnl|SRA|SRR1171870.13999414.1:38-82 HWI-ST967_77:2:1103:19727:137149.
cagtagcttacagaacgcaaacaataggaccatagtgggggattc
>_R_gnl|SRA|SRR1171870.13721073.2:8-52 HWI-ST967_77:2:1103:11922:126844.
cagtagcttacagaacgcaaacaataggaccatagtgggggattc
>gnl|SRA|SRR1171870.13383704.2:17-61 HWI-ST967_77:2:1103:6221:114351.
cagtagcttacagaacgcaaacaataggaccatagtgggggattc
>_R_gnl|SRA|SRR1171870.13180895.1:53-97 HWI-ST967_77:2:1103:3568:106945.
cagtagcttacagaacgcaaacaataggaccatagtgggggattc
>gnl|SRA|SRR1171870.10841052.1:20-64 HWI-ST967_77:2:1103:7029:19943.
cagtagcttacagaacgcaaacaataggaccatagtgggggattc
>_R_gnl|SRA|SRR1171870.10439538.1:37-81 HWI-ST967_77:2:1103:2036:5048.
cagtagcttacagaacgcaaacaataggaccatagtgggggattc
>_R_gnl|SRA|SRR1171870.9615341.2:19-63 HWI-ST967_77:2:1102:16175:171833.
cagtagcttacagaacgcaaacaataggaccatagtgggggattc
>_R_gnl|SRA|SRR1171870.8908062.1:23-67 HWI-ST967_77:2:1102:10091:144668.
cagtagcttacagaacgcaaacaataggaccatagtgggggattc
>gnl|SRA|SRR1171870.8687667.1:34-78 HWI-ST967_77:2:1102:12759:135909.
cagtagcttacagaacgcaaacaataggaccatagtgggggattc
>_R_gnl|SRA|SRR1171870.6801716.1:53-97 HWI-ST967_77:2:1102:1700:62720.
cagtagcttacagaacgcaaacaataggaccatagtgggggattc
>_R_gnl|SRA|SRR1171870.6611074.1:18-62 HWI-ST967_77:2:1102:19578:55208.
cagtagcttacagaacgcaaacaataggaccatagtgggggattc
>_R_gnl|SRA|SRR1171870.5504973.1:26-70 HWI-ST967_77:2:1102:8928:13324.
cagtagcttacagaacgcaaacaataggaccatagtgggggattc
>_R_gnl|SRA|SRR1171870.5003781.2:38-82 HWI-ST967_77:2:1101:16841:192600.
cagtagcttacagaacgcaaacaataggaccatagtgggggattc
>gnl|SRA|SRR1171870.4266162.2:23-67 HWI-ST967_77:2:1101:10984:165020.
cagtagcttacagaacgcaaacaataggaccatagtgggggattc
>_R_gnl|SRA|SRR1171870.4208205.1:46-90 HWI-ST967_77:2:1101:16200:162958.
cagtagcttacagaacgcaaacaataggaccatagtgggggattc
>_R_gnl|SRA|SRR1171870.3761826.1:13-57 HWI-ST967_77:2:1101:9950:146148.
cagtagcttacagaacgcaaacaataggaccatagtgggggattc
>_R_gnl|SRA|SRR1171870.2821175.1:43-87 HWI-ST967_77:2:1101:5977:110285.
cagtagcttacagaacgcaaacaataggaccatagtgggggattc
>_R_gnl|SRA|SRR1171870.2056309.1:6-50 HWI-ST967_77:2:1101:12437:81048.
cagtagcttacagaacgcaaacaataggaccatagtgggggattc
>gnl|SRA|SRR1171870.2048427.1:32-76 HWI-ST967_77:2:1101:8179:80930.
cagtagcttacagaacgcaaacaataggaccatagtgggggattc
>_R_gnl|SRA|SRR1171870.1138958.2:13-57 HWI-ST967_77:2:1101:14197:45827.

cagtagcttacagaacgcaaacaataggaccatagtgggggattc
>_R_gnl|SRA|SRR1171870.1006158.2:44-88 HWI-ST967_77:2:1101:7012:40948.
cagtagcttacagaacgcaaacaataggaccatagtgggggattc
>gnl|SRA|SRR1171870.846071.1:19-63 HWI-ST967_77:2:1101:15165:34646.
cagtagcttacagaacgcaaacaataggaccatagtgggggattc
>_R_gnl|SRA|SRR1171870.510235.1:14-58 HWI-ST967_77:2:1101:1695:21766.
cagtagcttacagaacgcaaacaataggaccatagtgggggattc
>gnl|SRA|SRR1171870.186756559.2:48-92 HWI-ST967_77:2:2302:19494:176777.
cagtagcttacagaacgcaaacaatgggaccatagtgggggattc
>gnl|SRA|SRR1171870.192092005.2:49-93 HWI-ST967_77:2:2303:12526:173631.
cagtagcttacagaacgcaaacaataggaccatagtggggggttc
>gnl|SRA|SRR1171870.181900396.2:40-84 HWI-ST967_77:2:2301:14159:190243.
cagtagcttacagaacgcaaacaataggaccatagtggggggttc
>gnl|SRA|SRR1171870.156065255.1:50-94 HWI-ST967_77:2:2203:15479:63881.
cagtagcttacagaacgcaaacaataggaccatagtggggggttc
>gnl|SRA|SRR1171870.145192522.2:47-91 HWI-ST967_77:2:2201:8926:56211.
cagtagcttacagaacgcaaacaataggaccatagtggggggttc
>gnl|SRA|SRR1171870.173820300.2:51-95 HWI-ST967_77:2:2206:3577:121193.
cagtagcttgcagaacgcaaacaataggaccatagtgggggattc
>gnl|SRA|SRR1171870.70976111.1:22-66 HWI-ST967_77:2:1207:17059:87723.
cagtagcttgcagaacgcaaacaataggaccatagtgggggattc
>gnl|SRA|SRR1171870.156476286.2:52-96 HWI-ST967_77:2:2203:2131:78827.
cagtagcttacagaacgcaaacaatagggccatagtgggggattc
>gnl|SRA|SRR1171870.147311377.2:24-68 HWI-ST967_77:2:2201:19537:137362.
cagtagcttacaggacgcaaacaataggaccatagtgggggattc
>_R_gnl|SRA|SRR1171870.127409055.2:37-81 HWI-ST967_77:2:2104:13105:44123.
cagtagcttacagaacgcaaacaataggaccataatgggggattc
>gnl|SRA|SRR1171870.51626834.1:20-64 HWI-ST967_77:2:1203:17611:128799.
cagtagcttacagaacgcaaacaataggaccatagcgggggattc
>_R_gnl|SRA|SRR1171870.42802632.2:53-97 HWI-ST967_77:2:1202:19407:3713.
cagtagctcacagaacgcaaacaataggaccatagtgggggattc
>gnl|SRA|SRR1171870.20763653.2:50-94 HWI-ST967_77:2:1104:20220:189915.
cagtagcttacagaacgcaaacgataggaccatagtgggggattc
>_R_gnl|SRA|SRR1171870.185569147.2:37-81 HWI-ST967_77:2:2302:12956:131983.
cagtagctgacagaacgcaaacaataggaccatagtgggggattc
>_R_gnl|SRA|SRR1171870.84283762.2:50-94 HWI-ST967_77:2:1303:16601:21878.
cagtagcttacagaacgcaaacgttaggaccatagtgggggattc
>_R_gnl|SRA|SRR1171870.55909812.1:43-87 HWI-ST967_77:2:1204:17519:83317.
cagtagcttacagaacgcaaacattaggaccatagtgggggattc
>gnl|SRA|SRR1171870.179638580.1:4-48 HWI-ST967_77:2:2301:20220:102491.
cagtagcttacagaacgcaaacaataggaccatagagggggattc
>gnl|SRA|SRR1171870.118744176.2:5-49 HWI-ST967_77:2:2102:3070:117813.
cagtagcttacagaacgcaaacaataggaccataggggggggattc
>_R_gnl|SRA|SRR1171870.173676904.1:51-95 HWI-ST967_77:2:2206:15759:114716.
cagtagcttacagaacgcaaacaataggcccatagtgggggattc
>_R_gnl|SRA|SRR1171870.153549322.1:4-48 HWI-ST967_77:2:2202:6768:171965.
cagtagcttacagaacgcaaacaataggcccatagtgggggattc
>_R_gnl|SRA|SRR1171870.110193.1:50-94 HWI-ST967_77:2:1101:20962:6052.
cagtagcttacagaacgcaaacaataggcccatagtgggggattc
>_R_gnl|SRA|SRR1171870.73040388.2:28-72 HWI-ST967_77:2:1208:12534:138536.
cagtagcttacagnacncaaacaataggtccatagtgggngattc

>_R_gnl|SRA|SRR1171870.87009418.1:49-93 HWI-ST967_77:2:1303:20000:120738.
cagtagcttacagaacgcaaacgataggtccatagtgggggattc
>_R_gnl|SRA|SRR1171870.38446933.2:2-46 HWI-ST967_77:2:1201:13780:36539.
cagtaacttacagaacgcaaacaataggtccatagtgggggattc
>_R_gnl|SRA|SRR1171870.90774602.2:17-61 HWI-ST967_77:2:1304:11672:56070.
cagtagcttacagaacgcaaacaataggaccatagtgggcgattc
>gnl|SRA|SRR1171870.171623725.2:23-67 HWI-ST967_77:2:2206:6752:28548.
cagtagcttacagaactcaaacaataggaccatagtgggggattc
>_R_gnl|SRA|SRR1171870.9938235.1:31-75 HWI-ST967_77:2:1102:7996:184363.
cagtagcttacagaactcaaacaataggaccatagtgggggattc
>gnl|SRA|SRR1171870.46964905.2:28-72 HWI-ST967_77:2:1202:2004:158961.
cagtagcttacaggactcaaacaataggaccatggtgggggattc
>_R_gnl|SRA|SRR1171870.4011107.1:51-95 HWI-ST967_77:2:1101:2428:155677.
cagcagcttacagaacgcaaacaacaggaccatagtgggggattc
>_R_gnl|SRA|SRR1171870.80169492.2:33-77 HWI-ST967_77:2:1302:8061:65091.
cagtagcttacagaacgcaaacaataggaccattgtgggggattc
>_R_gnl|SRA|SRR1171870.167434507.1:48-92 HWI-ST967_77:2:2205:13155:72720.
cagtagcttacagaacgcaaacaatatgaccatagtgggggattc
>_R_gnl|SRA|SRR1171870.204867349.1:49-93 HWI-ST967_77:2:2306:12464:44231.
cagtagcttacagaacgcaaacaataggaccagagtgggggattc
>_R_gnl|SRA|SRR1171870.183308552.2:33-77 HWI-ST967_77:2:2302:12007:44970.
cagtcgcttacagaacgcaaacaataggaccatagtgggggattc
>gnl|SRA|SRR1171870.98266395.1:43-87 HWI-ST967_77:2:1305:5651:124238.
cagtagcttacagaacgcaaacaataggagcatagtgggggattc
>_R_gnl|SRA|SRR1171870.68148985.1:17-61 HWI-ST967_77:2:1206:8417:127896.
cagtagcttacagaacgcaaacaataggaacatagtgggggattc
>gnl|SRA|SRR1171870.96109413.1:38-82 HWI-ST967_77:2:1305:6760:45708.
cagtagcttacagaacgcaaacaataggaccatagtggcggattc
>_R_gnl|SRA|SRR1171870.40163157.2:52-96 HWI-ST967_77:2:1201:20616:102167.
cagtagcttacagaacgaaaacaataggaccatagtgggggattc
>_R_gnl|SRA|SRR1171870.3893293.2:43-87 HWI-ST967_77:2:1101:8320:151106.
cagtagcttacagaacgaaaacaataggaccatagtgggggattc
>_R_gnl|SRA|SRR1171870.32573838.2:18-62 HWI-ST967_77:2:1107:9394:20573.
cagtagcatacagaacgcaaacaataggaccatagtgggggattc
>gnl|SRA|SRR1171870.14118639.1:43-87 HWI-ST967_77:2:1103:13422:141669.
cagtagcttacagaccgcaaacaataggaccatagtgggggattc
>_R_gnl|SRA|SRR1171870.151815836.1:48-92 HWI-ST967_77:2:2202:16392:107489.
cagtagcttacagaacgcaaaaaagaggaccatagtgggggattc
>_R_gnl|SRA|SRR1171870.102575743.2:51-95 HWI-ST967_77:2:1306:14461:77119.
cagtagcttacagaacgcaaaaaataggaccatagtgggggattc
>_R_gnl|SRA|SRR1171870.102374482.2:18-62 HWI-ST967_77:2:1306:14187:69997.
cagtagcttacagaacgcaaaaaataggaccatagtgggggattc
>gnl|SRA|SRR1171870.189467794.2:22-66 HWI-ST967_77:2:2303:6611:77991.
cagtagcttacagaacgcaaacaataggacaatagtgggggattc
>gnl|SRA|SRR1171870.56986881.2:40-84 HWI-ST967_77:2:1204:2742:122880.
cagtagcttacagaacgcatacaataggacgatagtgggggattc
>_R_gnl|SRA|SRR1171870.127813971.2:37-81 HWI-ST967_77:2:2104:4574:59226.
cagtatcttacagaacgcaaacaataggaccatagtgggggattc
>gnl|SRA|SRR1171870.143666147.1:47-91 HWI-ST967_77:2:2108:4522:62147.
cagtatcttaccgaacgcaaacaataggaccatagtgggggattc
>gnl|SRA|SRR1171870.38915329.1:56-100 HWI-ST967_77:2:1201:3783:54730.

cagtagcttaccgaacgcaaacaataggaccatagtgggggattc
>gnl|SRA|SRR1171870.139725965.2:4-48 HWI-ST967_77:2:2106:17445:99996.
cattagcttactgaacgcaaacaataggaccatagtgggggattc
>_R_gnl|SRA|SRR1171870.16322622.2:25-69 HWI-ST967_77:2:1104:5226:24428.
caggagcttacagggcgcaaacaataggaccatagtgggggattc
>gnl|SRA|SRR1171870.66524243.2:52-96 HWI-ST967_77:2:1206:16965:67856.
cagaagcttacataacgcaaacaataggaccatagtgggggattc
>_R_gnl|SRA|SRR1171870.108335037.1:36-80 HWI-ST967_77:2:1307:17724:138687.
cagtagcttacagaacgctaacaataggaccatagtgggggcttc
>_R_gnl|SRA|SRR1171870.44503358.2:16-60 HWI-ST967_77:2:1202:14929:66403.
cagtagcttacagaacgcaaacaatgggaccataatgggggtttc
>gnl|SRA|SRR1171870.141412118.2:34-78 HWI-ST967_77:2:2106:21109:162613.
cagtagcttacagaacgcaaccaataggcccatagtgggggactc
>_R_gnl|SRA|SRR1171870.33338024.2:14-58 HWI-ST967_77:2:1107:6632:54238.
cagtagcttacagaacgcaaacaataggaccatcgggggcattc
>_R_gnl|SRA|SRR1171870.205386658.1:17-60 HWI-ST967_77:2:2306:13708:63235.
cagtagcttacagaacgcaaacaataggaccatagtgggggatt-
>gnl|SRA|SRR1171870.180853566.2:57-100 HWI-ST967_77:2:2301:7038:150424.
cagtagcttacagaacgcaaacaataggaccatagtgggggatt-
>gnl|SRA|SRR1171870.177879648.2:57-100 HWI-ST967_77:2:2301:15075:34280.
cagtagcttacagaacgcaaacaataggaccatagtgggggatt-
>_R_gnl|SRA|SRR1171870.150707554.1:1-44 HWI-ST967_77:2:2202:4623:65813.
cagtagcttacagaacgcaaacaataggaccatagtgggggatt-
>gnl|SRA|SRR1171870.119169875.1:50-93 HWI-ST967_77:2:2102:17867:134088.
cagtagcttacagaacgcaaacaataggaccatagtgggggatt-
>gnl|SRA|SRR1171870.117183071.2:57-100 HWI-ST967_77:2:2102:4261:57576.
cagtagcttacagaacgcaaacaataggaccatagtgggggatt-
>_R_gnl|SRA|SRR1171870.103985341.2:49-92 HWI-ST967_77:2:1306:12367:128413.
cagtagcttacagaacgcaaacaataggaccatagtgggggatt-
>gnl|SRA|SRR1171870.100632960.1:57-100 HWI-ST967_77:2:1306:18623:8750.
cagtagcttacagaacgcaaacaataggaccatagtgggggatt-
>gnl|SRA|SRR1171870.99478527.2:57-100 HWI-ST967_77:2:1305:7180:166662.
cagtagcttacagaacgcaaacaataggaccatagtgggggatt-
>gnl|SRA|SRR1171870.95138215.2:57-100 HWI-ST967_77:2:1305:3857:11765.
cagtagcttacagaacgcaaacaataggaccatagtgggggatt-
>gnl|SRA|SRR1171870.89589159.1:57-100 HWI-ST967_77:2:1304:13058:14045.
cagtagcttacagaacgcaaacaataggaccatagtgggggatt-
>gnl|SRA|SRR1171870.74903358.1:57-100 HWI-ST967_77:2:1301:12049:64713.
cagtagcttacagaacgcaaacaataggaccatagtgggggatt-
>gnl|SRA|SRR1171870.58508153.2:57-100 HWI-ST967_77:2:1204:15308:177056.
cagtagcttacagaacgcaaacaataggaccatagtgggggatt-
>gnl|SRA|SRR1171870.51945881.1:57-100 HWI-ST967_77:2:1203:5678:140343.
cagtagcttacagaacgcaaacaataggaccatagtgggggatt-
>_R_gnl|SRA|SRR1171870.50287793.1:1-44 HWI-ST967_77:2:1203:11571:80699.
cagtagcttacagaacgcaaacaataggaccatagtgggggatt-
>_R_gnl|SRA|SRR1171870.33990566.1:13-56 HWI-ST967_77:2:1107:20355:83044.
cagtagcttacagaacgcaaacaataggaccatagtgggggatt-
>gnl|SRA|SRR1171870.510235.2:57-100 HWI-ST967_77:2:1101:1695:21766.
cagtagcttacagaacgcaaacaataggaccatagtgggggatt-
>gnl|SRA|SRR1171870.80031788.2:50-93 HWI-ST967_77:2:1302:11392:59887.
cagtagcttacagaacgcaaacaataggacaatagtggggggtt-

>gnl|SRA|SRR1171870.180140487.1:57-100 HWI-ST967_77:2:2301:3794:122040.
cagtagcttccagcacgcaaacaataggaccatagtgggggatt-
>_R_gnl|SRA|SRR1171870.103554078.2:48-91 HWI-ST967_77:2:1306:2565:112822.
cagtaccttacagaacgaaaacaataggacaatagtgggggatt-
>_R_gnl|SRA|SRR1171870.104523754.2:18-61 HWI-ST967_77:2:1306:13332:147864.
cagtagcttaaagaaagcaaacaacaggacaatagtgggggatt-
>gnl|SRA|SRR1171870.203273507.1:58-100 HWI-ST967_77:2:2305:1188:184609.
cagtagcttacagaacgcaaacaataggaccatagtgggggat--
>_R_gnl|SRA|SRR1171870.183920398.2:1-43 HWI-ST967_77:2:2302:18122:67974.
cagtagcttacagaacgcaaacaataggaccatagtgggggat--
>gnl|SRA|SRR1171870.149838263.2:58-100 HWI-ST967_77:2:2202:7025:33637.
cagtagcttacagaacgcaaacaataggaccatagtgggggat--
>gnl|SRA|SRR1171870.135178068.2:58-100 HWI-ST967_77:2:2105:15167:132051.
cagtagcttacagaacgcaaacaataggaccatagtgggggat--
>_R_gnl|SRA|SRR1171870.121618212.2:1-43 HWI-ST967_77:2:2103:13534:28158.
cagtagcttacagaacgcaaacaataggaccatagtgggggat--
>gnl|SRA|SRR1171870.89413523.1:58-100 HWI-ST967_77:2:1304:13070:7872.
cagtagcttacagaacgcaaacaataggaccatagtgggggat--
>gnl|SRA|SRR1171870.89266828.2:58-100 HWI-ST967_77:2:1304:17755:2676.
cagtagcttacagaacgcaaacaataggaccatagtgggggat--
>gnl|SRA|SRR1171870.27648582.1:52-94 HWI-ST967_77:2:1106:9119:38927.
cagtagcttacagaacgcaaacaataggaccatagtgggggat--
>gnl|SRA|SRR1171870.171473786.1:56-98 HWI-ST967_77:2:2206:8506:22441.
cagcagcttacagaacgcaaacaataggaccatagtgggggat--
>gnl|SRA|SRR1171870.36665651.1:50-92 HWI-ST967_77:2:1108:2175:41871.
cagtagcttaccgaacgcaaacaataggaccatagtggaggat--
>gnl|SRA|SRR1171870.29892159.2:15-57 HWI-ST967_77:2:1106:20827:119100.
caggagcttacagaacgcaaacagtaggaccagagtgggggat--
>_R_gnl|SRA|SRR1171870.196341822.2:1-42 HWI-ST967_77:2:2304:4449:129878.
cagtagcttacagaacgcaaacaataggaccatagtgggggga---
>_R_gnl|SRA|SRR1171870.189873150.1:1-42 HWI-ST967_77:2:2303:5964:92868.
cagtagcttacagaacgcaaacaataggaccatagtgggggga---
>_R_gnl|SRA|SRR1171870.140709507.2:16-57 HWI-ST967_77:2:2106:5726:136889.
cagtagcttacagaacgcaaacaataggaccatagtgggggga---
>gnl|SRA|SRR1171870.122845774.1:59-100 HWI-ST967_77:2:2103:16982:73904.
cagtagcttacagaacgcaaacaataggaccatagtgggggga---
>gnl|SRA|SRR1171870.38043510.1:59-100 HWI-ST967_77:2:1201:12583:21158.
cagtagcttacagaacgcaaacaataggaccatagtgggggga---
>gnl|SRA|SRR1171870.186670517.2:59-100 HWI-ST967_77:2:2302:2942:173993.
cagtagcttacagagcgcaaacagtaggaccatagtgggggga---
>_R_gnl|SRA|SRR1171870.36090423.1:1-42 HWI-ST967_77:2:1107:15786:183459.
cagtagcttacagaacgcaaacaataggacaatagtgggggga---
>gnl|SRA|SRR1171870.4011107.2:59-100 HWI-ST967_77:2:1101:2428:155677.
cagtagcttacagaacgcaaacaataggaccatagtgcggga---
>_R_gnl|SRA|SRR1171870.161927991.1:1-41 HWI-ST967_77:2:2204:3201:75612.
cagtagcttacagaacgcaaacaataggaccatagtgggggg----
>gnl|SRA|SRR1171870.116073549.2:57-97 HWI-ST967_77:2:2102:8073:15459.
cagtagcttacagaacgcaaacaataggaccatagtgggggg----
>_R_gnl|SRA|SRR1171870.113378689.2:1-41 HWI-ST967_77:2:2101:4249:109651.
cagtagcttacagaacgcaaacaataggaccatagtgggggg----
>_R_gnl|SRA|SRR1171870.85431487.1:1-41 HWI-ST967_77:2:1303:16023:63447.

cagtagcttacagaacgcaaacaataggaccatagtggggg----
>gnl|SRA|SRR1171870.81063060.2:60-100 HWI-ST967_77:2:1302:15860:99513.
cagtagcttacagaacgcaaacaataggaccatagtggggg----
>gnl|SRA|SRR1171870.80040396.2:60-100 HWI-ST967_77:2:1302:17172:60221.
cagtagcttacagaacgcaaacaataggaccatagtggggg----
>gnl|SRA|SRR1171870.28174187.2:60-100 HWI-ST967_77:2:1106:14657:57491.
cagtagcttacagaacgcaaacaataggaccatagtggggg----
>_R_gnl|SRA|SRR1171870.3278163.1:1-41 HWI-ST967_77:2:1101:2230:127830.
cagtagcttacagaacgcaaacaataggaccatagtggggg----
>_R_gnl|SRA|SRR1171870.52632503.1:1-41 HWI-ST967_77:2:1203:1185:164878.
cagtaacttacagaacgcaaacaataggaccatagtggggg----
>_R_gnl|SRA|SRR1171870.160534318.1:1-41 HWI-ST967_77:2:2204:13361:26157.
cagtagcttacagaacgcaaacaataggaccataggggggg----
>gnl|SRA|SRR1171870.68697158.1:52-92 HWI-ST967_77:2:1206:10995:148213.
cagtagcttacagaacgcaaacaataggcccatagtggggg----
>_R_gnl|SRA|SRR1171870.152091014.1:1-40 HWI-ST967_77:2:2202:3813:117773.
cagtagcttacagaacgcaaacaataggaccatagtgggg-----
>_R_gnl|SRA|SRR1171870.111086816.2:1-40 HWI-ST967_77:2:2101:5563:19674.
cagtagcttacagaacgcaaacaataggaccatagtgggg-----
>gnl|SRA|SRR1171870.100942902.1:51-90 HWI-ST967_77:2:1306:4857:19510.
cagtagcttacagaacgcaaacaataggaccatagtgggg-----
>_R_gnl|SRA|SRR1171870.95996372.2:1-40 HWI-ST967_77:2:1305:9539:41734.
cagtagcttacagaacgcaaacaataggaccatagtgggg-----
>gnl|SRA|SRR1171870.85325668.1:61-100 HWI-ST967_77:2:1303:11350:59512.
cagtagcttacagaacgcaaacaataggaccatagtgggg-----
>_R_gnl|SRA|SRR1171870.84392454.2:1-40 HWI-ST967_77:2:1303:10760:25773.
cagtagcttacagaacgcaaacaataggaccatagtgggg-----
>_R_gnl|SRA|SRR1171870.19289949.1:1-40 HWI-ST967_77:2:1104:3614:134575.
cagtagcttacagaacgcaaacaataggaccatagtgggg-----
>gnl|SRA|SRR1171870.154385351.2:62-100 HWI-ST967_77:2:2203:7010:3521.
cagtagcttacagaacgcaaacaataggaccatagtggg------
>_R_gnl|SRA|SRR1171870.66877546.1:1-39 HWI-ST967_77:2:1206:7634:80922.
cagtagcttacagaacgcaaacaataggaccatagtggg------
>gnl|SRA|SRR1171870.53748088.2:62-100 HWI-ST967_77:2:1204:5288:6210.
cagtagcttacagaacgcaaacaataggaccatagtggg------
>_R_gnl|SRA|SRR1171870.24043769.1:1-39 HWI-ST967_77:2:1105:14779:109246.
cagtagcttacagaacgcaaacaataggaccatagtggg------
>_R_gnl|SRA|SRR1171870.181619599.1:3-40 HWI-ST967_77:2:2301:19511:179284.
cagtagcttacagaacgcaaacaataggaccatagtgg-------
>gnl|SRA|SRR1171870.110870223.2:63-100 HWI-ST967_77:2:2101:2696:11224.
cagtagcttacagaacgcaaacaataggaccatagtgg-------
>gnl|SRA|SRR1171870.23712920.2:63-100 HWI-ST967_77:2:1105:10022:97309.
cagtagcttacagaacgcaaacaataggaccatagtgg-------
>_R_gnl|SRA|SRR1171870.8639364.1:1-38 HWI-ST967_77:2:1102:20807:133951.
cagtagcttacagaacgcaaacaataggaccatagtgg-------
>gnl|SRA|SRR1171870.181563782.2:64-100 HWI-ST967_77:2:2301:10382:177297.
cagtagcttacagaacgcaaacaataggaccatagtg--------
>_R_gnl|SRA|SRR1171870.115944598.1:1-37 HWI-ST967_77:2:2102:16531:10446.
cagtagcttacagaacgcaaacaataggaccatagtg--------
>gnl|SRA|SRR1171870.97537932.1:64-100 HWI-ST967_77:2:1305:8580:97884.
cagtagcttacagaacgcaaacaataggaccatagtg--------

>gnl|SRA|SRR1171870.10439538.2:64-100 HWI-ST967_77:2:1103:2036:5048.
cagtagcttacagaacgcaaacaataggaccatagtg--------
>_R_gnl|SRA|SRR1171870.180914700.2:1-36 HWI-ST967_77:2:2301:15198:152579.
cagtagcttacagaacgcaaacaataggaccatagt---------
>gnl|SRA|SRR1171870.50613992.1:65-100 HWI-ST967_77:2:1203:2301:92678.
cagaagcttacagaacgcaaacaataggaccatagt---------
>gnl|SRA|SRR1171870.183975202.1:66-100 HWI-ST967_77:2:2302:4311:70165.
cagtagcttacagaacgcaaacaataggaccatag----------
>_R_gnl|SRA|SRR1171870.167626118.2:1-35 HWI-ST967_77:2:2205:11169:79739.
cagtagcttacagaacgcaaacaataggaccatag----------
>gnl|SRA|SRR1171870.140851749.1:66-100 HWI-ST967_77:2:2106:6406:142150.
cagtagcttacagaacgcaaacaataggaccatag----------
>_R_gnl|SRA|SRR1171870.92018026.2:1-35 HWI-ST967_77:2:1304:17416:100636.
cagtagcttacagaacgcaaacaataggaccatag----------
>_R_gnl|SRA|SRR1171870.72305389.1:1-35 HWI-ST967_77:2:1208:4339:26705.
cagtagcttacagaacgcaaacaataggaccatag----------
>_R_gnl|SRA|SRR1171870.65349643.1:1-34 HWI-ST967_77:2:1206:11893:25848.
cagtagcttacagaacgcaaacaataggaccata-----------
>gnl|SRA|SRR1171870.54396708.2:67-100 HWI-ST967_77:2:1204:10266:29024.
cagtagcttacagaacgcaaacaataggaccata-----------
>gnl|SRA|SRR1171870.18628171.2:67-100 HWI-ST967_77:2:1104:12337:109342.
cagtagcttacagaacgcaaacaataggaccata-----------
>gnl|SRA|SRR1171870.99722352.2:68-100 HWI-ST967_77:2:1305:10510:175096.
cagtagcttacagaacgcaaacaataggaccat------------
>_R_gnl|SRA|SRR1171870.67625842.1:1-33 HWI-ST967_77:2:1206:6677:108486.
cagtagcttacagaacgcaaacaataggaccat------------
>_R_gnl|SRA|SRR1171870.65455255.1:1-33 HWI-ST967_77:2:1206:12454:29546.
cagtagcttacagaacgcaaacaataggaccat------------
>_R_gnl|SRA|SRR1171870.46199562.1:1-33 HWI-ST967_77:2:1202:19916:130016.
cagtagcttacagaacgcaaacaataggaccat------------
>_R_gnl|SRA|SRR1171870.208180948.2:23-67 HWI-ST967_77:2:2306:16798:168711.
cagtagattaaagaacgaaaccaataggaccatagtgggggattc
>_R_gnl|SRA|SRR1171870.139291441.2:2-46 HWI-ST967_77:2:2106:12276:83988.
cagtagcttacagaacgaaaacaatagcaccctactgggggattc
>_R_gnl|SRA|SRR1171870.143921409.2:57-100 HWI-ST967_77:2:2201:8855:7284.
-agtagcttacagaacgcaaacaataggaccatagtgggggattc
>_R_gnl|SRA|SRR1171870.14089819.2:57-100 HWI-ST967_77:2:1103:8255:140635.
-agtagcttacagaacgcaaacaataggaccatagtgggggattc
>_R_gnl|SRA|SRR1171870.13833041.2:17-60 HWI-ST967_77:2:1103:5298:131003.
-agtagcttacagaacgcaaacaagaggaccgtagcgggggattc
>gnl|SRA|SRR1171870.138701296.2:16-58 HWI-ST967_77:2:2106:9573:62331.
--gtagcttacagaacgcaaacaataggaccatagtgggggattc
>_R_gnl|SRA|SRR1171870.85325668.2:58-100 HWI-ST967_77:2:1303:11350:59512.
--gtagcttacagaacgcaaacaataggaccatagtgggggattc
>gnl|SRA|SRR1171870.75789154.1:1-43 HWI-ST967_77:2:1301:13778:98613.
--gtagcttacagaacgcaaacaataggaccatagtgggggattc
>_R_gnl|SRA|SRR1171870.71403725.1:58-100 HWI-ST967_77:2:1207:7915:138287.
--gtagcttacagaacgcaaacaataggaccatagtgggggattc
>gnl|SRA|SRR1171870.53439046.1:1-43 HWI-ST967_77:2:1203:6175:193319.
--gtagcttacagaacgcaaacaataggaccatagtgggggattc
>_R_gnl|SRA|SRR1171870.35750614.1:21-63 HWI-ST967_77:2:1107:2570:166632.

--gtagcttacagaacgcaaacaataggaccatagtgggggattc
>gnl|SRA|SRR1171870.6801716.2:32-74 HWI-ST967_77:2:1102:1700:62720.
--gtagcttacagaacgcaaacaataggaccatagtgggggattc
>gnl|SRA|SRR1171870.142380438.2:1-43 HWI-ST967_77:2:2106:8791:199072.
--gtagcttacagaactcaaacaataggaccatagtgggggattc
>_R_gnl|SRA|SRR1171870.198664272.2:60-100 HWI-ST967_77:2:2305:18177:14098.
----agcttacagaacgcaaacaataggaccatagtgggggattc
>_R_gnl|SRA|SRR1171870.193785656.1:60-100 HWI-ST967_77:2:2304:7286:37889.
----agcttacagaacgcaaacaataggaccatagtgggggattc
>_R_gnl|SRA|SRR1171870.181563782.1:60-100 HWI-ST967_77:2:2301:10382:177297.
----agcttacagaacgcaaacaataggaccatagtgggggattc
>_R_gnl|SRA|SRR1171870.174849188.1:60-100 HWI-ST967_77:2:2206:4356:168982.
----agcttacagaacgcaaacaataggaccatagtgggggattc
>_R_gnl|SRA|SRR1171870.156349729.1:60-100 HWI-ST967_77:2:2203:16005:74075.
----agcttacagaacgcaaacaataggaccatagtgggggattc
>_R_gnl|SRA|SRR1171870.74903358.2:60-100 HWI-ST967_77:2:1301:12049:64713.
----agcttacagaacgcaaacaataggaccatagtgggggattc
>_R_gnl|SRA|SRR1171870.70723746.1:60-100 HWI-ST967_77:2:1207:3939:61859.
----agcttacagaacgcaaacaataggaccatagtgggggattc
>_R_gnl|SRA|SRR1171870.51626834.2:60-100 HWI-ST967_77:2:1203:17611:128799.
----agcttacagaacgcaaacaataggaccatagtgggggattc
>_R_gnl|SRA|SRR1171870.23893331.1:60-100 HWI-ST967_77:2:1105:7655:103808.
----agcttacagaacgcaaacaataggaccatagtgggggattc
>gnl|SRA|SRR1171870.131309252.2:1-40 HWI-ST967_77:2:2104:7083:187373.
-----gcttacagaacgcaaacaataggaccatagtgggggattc
>gnl|SRA|SRR1171870.194883980.2:1-39 HWI-ST967_77:2:2304:4869:77348.
------cttacagaacgcaaacaataggaccatagtgggggattc
>gnl|SRA|SRR1171870.54895476.1:1-38 HWI-ST967_77:2:1204:10459:46890.
-------ttacagaacgcaaacaataggaccatagtgggggattc
>gnl|SRA|SRR1171870.15178342.2:1-38 HWI-ST967_77:2:1103:3368:181209.
-------ttacagaacgcaaacaataggaccatagtgggggattc
>_R_gnl|SRA|SRR1171870.13207896.2:63-100 HWI-ST967_77:2:1103:4157:107797.
-------ttacagaacgcaaacaataggaccatagtgggggattc
>_R_gnl|SRA|SRR1171870.76822257.2:64-100 HWI-ST967_77:2:1301:5918:138063.
--------tacagaacgcaaacaataggaccatagtgggggattc
>_R_gnl|SRA|SRR1171870.38915329.2:63-99 HWI-ST967_77:2:1201:3783:54730.
--------tacagaacgcaaacaataggaccatagtgggagattc
>gnl|SRA|SRR1171870.184264788.1:37-72 HWI-ST967_77:2:2302:13328:81129.
---------acagaacgcaaacaataggaccatagtgggggattc
>gnl|SRA|SRR1171870.155433297.1:1-36 HWI-ST967_77:2:2203:8710:41124.
---------acagaacgcaaacaataggaccatagtgggggattc
>_R_gnl|SRA|SRR1171870.141060156.1:65-100 HWI-ST967_77:2:2106:3883:149891.
---------acagaacgcaaacaataggaccatagtgggggattc
>_R_gnl|SRA|SRR1171870.99207901.1:65-100 HWI-ST967_77:2:1305:12147:157232.
---------acagaacgcaaacaataggaccatagtgggggattc
>_R_gnl|SRA|SRR1171870.46725015.1:65-100 HWI-ST967_77:2:1202:12439:149957.
---------acagaacgcaaacaataggaccatagtgggggattc
>_R_gnl|SRA|SRR1171870.24650180.1:65-100 HWI-ST967_77:2:1105:2402:131419.
---------acagaacgcaaacaataggaccatagtgggggattc
>_R_gnl|SRA|SRR1171870.11707770.1:65-100 HWI-ST967_77:2:1103:15288:51955.
---------acagaacgcaagcaatagggccatagtgggggattc

>_R_gnl|SRA|SRR1171870.69149303.2:4-39 HWI-ST967_77:2:1206:15467:164951.
---------acagaacgcaaaaaataggaccatagtgggtgattc
>gnl|SRA|SRR1171870.195975960.1:24-67 HWI-ST967_77:2:2304:16845:116565.
-cagcagctacagaacgcaaacaataggaccatagtgggggattc
>_R_gnl|SRA|SRR1171870.100140699.1:66-100 HWI-ST967_77:2:1305:20975:189626.
----------cagaacgcaaacaataggaccatagtgggggattc
>gnl|SRA|SRR1171870.98888444.2:1-35 HWI-ST967_77:2:1305:6786:145792.
----------cagaacgcaaacaataggaccatagtgggggattc
>_R_gnl|SRA|SRR1171870.92548423.1:66-100 HWI-ST967_77:2:1304:18353:119326.
----------cagaacgcaaacaataggaccatagtgggggattc
>_R_gnl|SRA|SRR1171870.50613992.2:66-100 HWI-ST967_77:2:1203:2301:92678.
----------cagaacgcaaacaataggaccagagtgggggattc
>_R_gnl|SRA|SRR1171870.89589159.2:67-100 HWI-ST967_77:2:1304:13058:14045.
-----------agaacgcaaacaataggaccatagtgggggattc
>_R_gnl|SRA|SRR1171870.64897532.2:67-100 HWI-ST967_77:2:1206:17166:9585.
-----------agaacgcaaacaataggaccatagtgggggattc
>_R_gnl|SRA|SRR1171870.64819155.1:67-100 HWI-ST967_77:2:1206:12924:6802.
-----------agaacgcaaacaataggaccatagtgggggattc
>gnl|SRA|SRR1171870.53342541.1:1-34 HWI-ST967_77:2:1203:12145:189909.
-----------agaacgcaaacaataggaccatagtgggggattc
>_R_gnl|SRA|SRR1171870.100512010.1:69-100 HWI-ST967_77:2:1306:20249:4451.
-------------aacgcaaacaataggaccatagtgggggattc
>gnl|SRA|SRR1171870.167063966.1:37-68 HWI-ST967_77:2:2205:7800:59427.
-------------aactcaaacaataggaccatagtgggggattc
>_R_gnl|SRA|SRR1171870.161296060.1:68-99 HWI-ST967_77:2:2204:8890:53126.
-------------aacgcaaacaataggaccagagtgggggattc
>_R_gnl|SRA|SRR1171870.199381965.1:70-100 HWI-ST967_77:2:2305:2342:40117.
--------------acgcaaacaataggaccatagtgggggattc
>_R_gnl|SRA|SRR1171870.129097320.1:70-100 HWI-ST967_77:2:2104:7585:106417.
--------------acgcaaacaataggaccatagtgggggattc
>_R_gnl|SRA|SRR1171870.25587042.1:70-100 HWI-ST967_77:2:1105:15919:164702.
--------------acgcaaacaataggaccatagtgggggattc
>_R_gnl|SRA|SRR1171870.175971850.2:66-98 HWI-ST967_77:2:2208:13798:13033.
------------gaacgcaaacaataggaccatagtgggggattc
>_R_gnl|SRA|SRR1171870.170839357.2:68-100 HWI-ST967_77:2:2205:11726:195493.
------------gaacgcaaacaataggaccatagtgggggattc
>_R_gnl|SRA|SRR1171870.157031923.1:68-100 HWI-ST967_77:2:2203:9833:98959.
------------gaacgcaaacaataggaccatagtgggggattc
>_R_gnl|SRA|SRR1171870.14168500.1:68-100 HWI-ST967_77:2:1103:2380:143684.
------------gaacgcaaacaataggaccatagtgggggattc
>_R_gnl|SRA|SRR1171870.164114107.1:68-100 HWI-ST967_77:2:2204:17278:153514.
------------gaacgcaaacaataggaccatagtgggggagtc
>_R_gnl|SRA|SRR1171870.152941758.1:68-100 HWI-ST967_77:2:2202:11408:149506.
------------gaacgcaaacaataggcccatagtgggggattc
>_R_gnl|SRA|SRR1171870.36793850.1:68-100 HWI-ST967_77:2:1108:10140:62840.
------------gatcgcaaacaataggaccatagtgggggattc
>_R_gnl|SRA|SRR1171870.34541136.2:68-100 HWI-ST967_77:2:1107:8081:108480.
------------gaacgtaaacaataggagcatagtgggggattc
>gnl|SRA|SRR1171870.178567599.1:1-30 HWI-ST967_77:2:2301:5855:61162.
---------------cgcaaacaataggaccatagtgggggattc
>gnl|SRA|SRR1171870.148306883.2:1-30 HWI-ST967_77:2:2201:16676:175189.

---------------cgcaaacaataggaccatagtgggggattc
>gnl|SRA|SRR1171870.141660289.1:1-30 HWI-ST967_77:2:2106:16272:171997.
---------------cgcaaacaataggaccatagtgggggattc
>gnl|SRA|SRR1171870.124732050.2:1-30 HWI-ST967_77:2:2103:8110:143906.
---------------cgcaaacaataggaccatagtgggggattc
>gnl|SRA|SRR1171870.31990411.2:1-30 HWI-ST967_77:2:1106:4187:194822.
---------------cgcaaacaataggaccatagtgggggattc
>gnl|SRA|SRR1171870.138127180.1:1-29 HWI-ST967_77:2:2106:2938:41640.
----------------gcaaacaataggaccatagtgggggattc
>_R_gnl|SRA|SRR1171870.137341649.1:72-100 HWI-ST967_77:2:2106:9327:12881.
----------------gcaaacaataggaccatagtgggggattc
>gnl|SRA|SRR1171870.53779771.1:1-29 HWI-ST967_77:2:1204:15726:7164.
----------------gcaaacaataggaccatagtgggggattc
>_R_gnl|SRA|SRR1171870.201382991.1:73-100 HWI-ST967_77:2:2305:18703:116064.
-----------------caaacaataggaccatagtgggggattc
>_R_gnl|SRA|SRR1171870.174455635.1:73-100 HWI-ST967_77:2:2206:9798:150101.
-----------------caaacaataggaccatagtgggggattc
>_R_gnl|SRA|SRR1171870.170345743.1:73-100 HWI-ST967_77:2:2205:6598:178194.
-----------------caaacaataggaccatagtgggggattc
>_R_gnl|SRA|SRR1171870.129654912.2:73-100 HWI-ST967_77:2:2104:13063:126765.
-----------------caaacaataggaccatagtgggggattc
>_R_gnl|SRA|SRR1171870.113786400.1:73-100 HWI-ST967_77:2:2101:18106:125344.
-----------------caaacaataggaccatagtgggggattc
>_R_gnl|SRA|SRR1171870.95158797.1:73-100 HWI-ST967_77:2:1305:19973:12436.
-----------------caaacaataggaccatagtgggggattc
>_R_gnl|SRA|SRR1171870.74051702.1:73-100 HWI-ST967_77:2:1301:10746:31966.
-----------------caaacaataggaccatagtgggggattc
>_R_gnl|SRA|SRR1171870.66524243.1:73-100 HWI-ST967_77:2:1206:16965:67856.
-----------------caaacaataggaccatagtgggggattc
>_R_gnl|SRA|SRR1171870.54673505.2:73-100 HWI-ST967_77:2:1204:15502:38884.
-----------------caaacaataggaccatagtgggggattc
>gnl|SRA|SRR1171870.18025679.2:1-28 HWI-ST967_77:2:1104:8099:86625.
-----------------caaacaataggaccatagtgggggattc
>_R_gnl|SRA|SRR1171870.14451802.1:73-100 HWI-ST967_77:2:1103:6058:154223.
-----------------caaacaataggaccatagtgggggattc
>_R_gnl|SRA|SRR1171870.10662392.1:73-100 HWI-ST967_77:2:1103:17549:13184.
-----------------caaacaataggaccatagtgggggattc
>_R_gnl|SRA|SRR1171870.12726469.1:47-91 HWI-ST967_77:2:1103:6760:89791.
cagtagcttacagaacgcaagtaatagttcgatagtggtggattc
>_R_gnl|SRA|SRR1171870.52082590.1:53-94 HWI-ST967_77:2:1203:14462:145092.
cagtagcttacagaacgcaaacaagg---ccacagtgggccattc
>_R_gnl|SRA|SRR1171870.186944606.2:1-23 HWI-ST967_77:2:2302:13026:183995.
cagtagcttacagaacgcaaaca----------------------
>_R_gnl|SRA|SRR1171870.185316740.1:1-23 HWI-ST967_77:2:2302:5300:122027.
cagtagcttacagaacgcaaaca----------------------
>_R_gnl|SRA|SRR1171870.170065934.1:1-23 HWI-ST967_77:2:2205:13363:168194.
cagtagcttacagaacgcaaaca----------------------
>_R_gnl|SRA|SRR1171870.166756599.1:1-23 HWI-ST967_77:2:2205:8128:48352.
cagtagcttacagaacgcaaaca----------------------
>_R_gnl|SRA|SRR1171870.163776170.1:1-23 HWI-ST967_77:2:2204:8442:141709.
cagtagcttacagaacgcaaaca----------------------

>_R_gnl|SRA|SRR1171870.131845691.2:1-23 HWI-ST967_77:2:2105:7004:8443.
cagtagcttacagaacgcaaaca----------------------
>gnl|SRA|SRR1171870.117026834.2:78-100 HWI-ST967_77:2:2102:11597:51666.
cagtagcttacagaacgcaaaca----------------------
>gnl|SRA|SRR1171870.102575743.1:78-100 HWI-ST967_77:2:1306:14461:77119.
cagtagcttacagaacgcaaaca----------------------
>gnl|SRA|SRR1171870.87009418.2:70-92 HWI-ST967_77:2:1303:20000:120738.
cagtagcttacagaacgcaaaca----------------------
>_R_gnl|SRA|SRR1171870.86617853.1:1-23 HWI-ST967_77:2:1303:7744:106622.
cagtagcttacagaacgcaaaca----------------------
>_R_gnl|SRA|SRR1171870.82350733.1:1-23 HWI-ST967_77:2:1302:8221:149902.
cagtagcttacagaacgcaaaca----------------------
>gnl|SRA|SRR1171870.60583997.1:78-100 HWI-ST967_77:2:1205:1476:52479.
cagtagcttacagaacgcaaaca----------------------
>_R_gnl|SRA|SRR1171870.58444871.1:1-23 HWI-ST967_77:2:1204:16553:174981.
cagtagcttacagaacgcaaaca----------------------
>gnl|SRA|SRR1171870.47814277.2:78-100 HWI-ST967_77:2:1202:9276:189667.
cagtagcttacagaacgcaaaca----------------------
>_R_gnl|SRA|SRR1171870.38739772.1:1-23 HWI-ST967_77:2:1201:8808:47909.
cagtagcttacagaacgcaaaca----------------------
>_R_gnl|SRA|SRR1171870.14929150.1:1-23 HWI-ST967_77:2:1103:3516:171848.
cagtagcttacagaacgcaaaca----------------------
>_R_gnl|SRA|SRR1171870.143687990.1:74-96 HWI-ST967_77:2:2108:6570:78559.
cagtagcttacataacgcaaaca----------------------
>gnl|SRA|SRR1171870.32541302.2:68-99 HWI-ST967_77:2:1107:15370:19051.
cagtagcttacagaacgcaaacaata---ccatag----------
>gnl|SRA|SRR1171870.200970201.2:76-99 HWI-ST967_77:2:2305:3112:100920.
cagtagcttacagaacgcaaacaa---------------------
>_R_gnl|SRA|SRR1171870.199049095.1:2-25 HWI-ST967_77:2:2305:12349:27859.
cagtagcttacagaacgcaaacaa---------------------
>gnl|SRA|SRR1171870.193636979.2:76-99 HWI-ST967_77:2:2304:19437:32339.
cagtagcttacagaacgcaaacaa---------------------
>_R_gnl|SRA|SRR1171870.187192518.1:10-33 HWI-ST967_77:2:2302:21095:192921.
cagtagcttacagaacgcaaacaa---------------------
>gnl|SRA|SRR1171870.186465294.2:14-37 HWI-ST967_77:2:2302:5705:166153.
cagtagcttacagaacgcaaacaa---------------------
>_R_gnl|SRA|SRR1171870.182525866.1:1-24 HWI-ST967_77:2:2302:18961:15320.
cagtagcttacagaacgcaaacaa---------------------
>_R_gnl|SRA|SRR1171870.179598466.1:7-30 HWI-ST967_77:2:2301:15050:100895.
cagtagcttacagaacgcaaacaa---------------------
>gnl|SRA|SRR1171870.178519139.1:70-93 HWI-ST967_77:2:2301:16257:59175.
cagtagcttacagaacgcaaacaa---------------------
>gnl|SRA|SRR1171870.167532240.2:76-99 HWI-ST967_77:2:2205:14016:76002.
cagtagcttacagaacgcaaacaa---------------------
>gnl|SRA|SRR1171870.164327647.2:76-99 HWI-ST967_77:2:2204:10353:161310.
cagtagcttacagaacgcaaacaa---------------------
>_R_gnl|SRA|SRR1171870.164214224.2:1-24 HWI-ST967_77:2:2204:5511:157410.
cagtagcttacagaacgcaaacaa---------------------
>_R_gnl|SRA|SRR1171870.154548880.2:4-27 HWI-ST967_77:2:2203:13110:9484.
cagtagcttacagaacgcaaacaa---------------------
>gnl|SRA|SRR1171870.150998331.2:75-98 HWI-ST967_77:2:2202:9059:76624.

cagtagcttacagaacgcaaacaa---------------------
>gnl|SRA|SRR1171870.147228819.1:76-99 HWI-ST967_77:2:2201:3092:134385.
cagtagcttacagaacgcaaacaa---------------------
>_R_gnl|SRA|SRR1171870.146637310.2:7-30 HWI-ST967_77:2:2201:2788:111441.
cagtagcttacagaacgcaaacaa---------------------
>_R_gnl|SRA|SRR1171870.143921077.2:9-32 HWI-ST967_77:2:2201:7796:7290.
cagtagcttacagaacgcaaacaa---------------------
>_R_gnl|SRA|SRR1171870.143001577.1:2-25 HWI-ST967_77:2:2107:2472:80640.
cagtagcttacagaacgcaaacaa---------------------
>gnl|SRA|SRR1171870.141714152.2:76-99 HWI-ST967_77:2:2106:14862:173963.
cagtagcttacagaacgcaaacaa---------------------
>_R_gnl|SRA|SRR1171870.141517375.1:1-24 HWI-ST967_77:2:2106:12387:166748.
cagtagcttacagaacgcaaacaa---------------------
>gnl|SRA|SRR1171870.138256137.2:70-93 HWI-ST967_77:2:2106:15661:46007.
cagtagcttacagaacgcaaacaa---------------------
>_R_gnl|SRA|SRR1171870.136738763.2:10-33 HWI-ST967_77:2:2105:11192:189211.
cagtagcttacagaacgcaaacaa---------------------
>gnl|SRA|SRR1171870.136000042.1:76-99 HWI-ST967_77:2:2105:11836:162152.
cagtagcttacagaacgcaaacaa---------------------
>gnl|SRA|SRR1171870.135579285.1:69-92 HWI-ST967_77:2:2105:13000:146862.
cagtagcttacagaacgcaaacaa---------------------
>gnl|SRA|SRR1171870.134712299.1:71-94 HWI-ST967_77:2:2105:14811:114598.
cagtagcttacagaacgcaaacaa---------------------
>gnl|SRA|SRR1171870.132180270.1:76-99 HWI-ST967_77:2:2105:9191:20425.
cagtagcttacagaacgcaaacaa---------------------
>gnl|SRA|SRR1171870.130377618.2:70-93 HWI-ST967_77:2:2104:2528:153618.
cagtagcttacagaacgcaaacaa---------------------
>_R_gnl|SRA|SRR1171870.128263694.1:9-32 HWI-ST967_77:2:2104:3576:75663.
cagtagcttacagaacgcaaacaa---------------------
>gnl|SRA|SRR1171870.127409055.1:70-93 HWI-ST967_77:2:2104:13105:44123.
cagtagcttacagaacgcaaacaa---------------------
>_R_gnl|SRA|SRR1171870.125316688.1:2-25 HWI-ST967_77:2:2103:10730:165316.
cagtagcttacagaacgcaaacaa---------------------
>gnl|SRA|SRR1171870.125261975.1:76-99 HWI-ST967_77:2:2103:6665:163369.
cagtagcttacagaacgcaaacaa---------------------
>gnl|SRA|SRR1171870.124609894.2:76-99 HWI-ST967_77:2:2103:9795:139269.
cagtagcttacagaacgcaaacaa---------------------
>gnl|SRA|SRR1171870.124399872.1:69-92 HWI-ST967_77:2:2103:10428:131574.
cagtagcttacagaacgcaaacaa---------------------
>gnl|SRA|SRR1171870.124298260.1:77-100 HWI-ST967_77:2:2103:10957:127774.
cagtagcttacagaacgcaaacaa---------------------
>_R_gnl|SRA|SRR1171870.118494847.1:7-30 HWI-ST967_77:2:2102:13316:108087.
cagtagcttacagaacgcaaacaa---------------------
>gnl|SRA|SRR1171870.118353837.1:76-99 HWI-ST967_77:2:2102:17657:102508.
cagtagcttacagaacgcaaacaa---------------------
>gnl|SRA|SRR1171870.117894238.2:76-99 HWI-ST967_77:2:2102:7136:84953.
cagtagcttacagaacgcaaacaa---------------------
>gnl|SRA|SRR1171870.117061892.1:75-98 HWI-ST967_77:2:2102:17109:52937.
cagtagcttacagaacgcaaacaa---------------------
>gnl|SRA|SRR1171870.116536053.2:70-93 HWI-ST967_77:2:2102:17754:32878.
cagtagcttacagaacgcaaacaa---------------------

>gnl|SRA|SRR1171870.116329066.2:70-93 HWI-ST967_77:2:2102:7970:25219.
cagtagcttacagaacgcaaacaa---------------------
>_R_gnl|SRA|SRR1171870.114763236.1:1-24 HWI-ST967_77:2:2101:18749:163391.
cagtagcttacagaacgcaaacaa---------------------
>_R_gnl|SRA|SRR1171870.114164964.1:5-28 HWI-ST967_77:2:2101:19624:140058.
cagtagcttacagaacgcaaacaa---------------------
>gnl|SRA|SRR1171870.113786400.2:76-99 HWI-ST967_77:2:2101:18106:125344.
cagtagcttacagaacgcaaacaa---------------------
>_R_gnl|SRA|SRR1171870.112390138.1:7-30 HWI-ST967_77:2:2101:3655:70863.
cagtagcttacagaacgcaaacaa---------------------
>gnl|SRA|SRR1171870.104523754.1:76-99 HWI-ST967_77:2:1306:13332:147864.
cagtagcttacagaacgcaaacaa---------------------
>gnl|SRA|SRR1171870.104356301.1:76-99 HWI-ST967_77:2:1306:10884:141898.
cagtagcttacagaacgcaaacaa---------------------
>gnl|SRA|SRR1171870.103985341.1:70-93 HWI-ST967_77:2:1306:12367:128413.
cagtagcttacagaacgcaaacaa---------------------
>gnl|SRA|SRR1171870.102564917.1:75-98 HWI-ST967_77:2:1306:4093:76831.
cagtagcttacagaacgcaaacaa---------------------
>gnl|SRA|SRR1171870.100655267.2:70-93 HWI-ST967_77:2:1306:21019:9495.
cagtagcttacagaacgcaaacaa---------------------
>gnl|SRA|SRR1171870.97232829.2:77-100 HWI-ST967_77:2:1305:4981:86043.
cagtagcttacagaacgcaaacaa---------------------
>gnl|SRA|SRR1171870.96785362.2:76-99 HWI-ST967_77:2:1305:12619:69448.
cagtagcttacagaacgcaaacaa---------------------
>gnl|SRA|SRR1171870.93593725.1:73-96 HWI-ST967_77:2:1304:11321:156412.
cagtagcttacagaacgcaaacaa---------------------
>_R_gnl|SRA|SRR1171870.92449148.1:1-24 HWI-ST967_77:2:1304:17803:115752.
cagtagcttacagaacgcaaacaa---------------------
>_R_gnl|SRA|SRR1171870.92334549.1:4-27 HWI-ST967_77:2:1304:13957:111764.
cagtagcttacagaacgcaaacaa---------------------
>_R_gnl|SRA|SRR1171870.91315053.1:5-28 HWI-ST967_77:2:1304:10278:75309.
cagtagcttacagaacgcaaacaa---------------------
>gnl|SRA|SRR1171870.91108968.1:75-98 HWI-ST967_77:2:1304:19763:67876.
cagtagcttacagaacgcaaacaa---------------------
>gnl|SRA|SRR1171870.89455124.1:73-96 HWI-ST967_77:2:1304:11297:9383.
cagtagcttacagaacgcaaacaa---------------------
>gnl|SRA|SRR1171870.88599640.2:76-99 HWI-ST967_77:2:1303:14564:177423.
cagtagcttacagaacgcaaacaa---------------------
>_R_gnl|SRA|SRR1171870.84863956.1:2-25 HWI-ST967_77:2:1303:16771:42810.
cagtagcttacagaacgcaaacaa---------------------
>_R_gnl|SRA|SRR1171870.83017490.1:7-30 HWI-ST967_77:2:1302:5352:174539.
cagtagcttacagaacgcaaacaa---------------------
>_R_gnl|SRA|SRR1171870.82665422.2:1-24 HWI-ST967_77:2:1302:10714:161579.
cagtagcttacagaacgcaaacaa---------------------
>_R_gnl|SRA|SRR1171870.82306496.1:7-30 HWI-ST967_77:2:1302:13492:148140.
cagtagcttacagaacgcaaacaa---------------------
>gnl|SRA|SRR1171870.80169492.1:76-99 HWI-ST967_77:2:1302:8061:65091.
cagtagcttacagaacgcaaacaa---------------------
>_R_gnl|SRA|SRR1171870.79262160.1:1-24 HWI-ST967_77:2:1302:12814:31173.
cagtagcttacagaacgcaaacaa---------------------
>gnl|SRA|SRR1171870.76822257.1:70-93 HWI-ST967_77:2:1301:5918:138063.

cagtagcttacagaacgcaaacaa---------------------
>gnl|SRA|SRR1171870.76213805.2:70-93 HWI-ST967_77:2:1301:13077:114904.
cagtagcttacagaacgcaaacaa---------------------
>_R_gnl|SRA|SRR1171870.71782342.1:4-27 HWI-ST967_77:2:1208:12982:2430.
cagtagcttacagaacgcaaacaa---------------------
>gnl|SRA|SRR1171870.71290545.1:70-93 HWI-ST967_77:2:1207:16112:123530.
cagtagcttacagaacgcaaacaa---------------------
>gnl|SRA|SRR1171870.64445817.1:77-100 HWI-ST967_77:2:1205:12104:191750.
cagtagcttacagaacgcaaacaa---------------------
>_R_gnl|SRA|SRR1171870.55922778.1:4-27 HWI-ST967_77:2:1204:15979:83889.
cagtagcttacagaacgcaaacaa---------------------
>gnl|SRA|SRR1171870.55909812.2:70-93 HWI-ST967_77:2:1204:17519:83317.
cagtagcttacagaacgcaaacaa---------------------
>_R_gnl|SRA|SRR1171870.55732770.1:7-30 HWI-ST967_77:2:1204:19713:76839.
cagtagcttacagaacgcaaacaa---------------------
>gnl|SRA|SRR1171870.53719371.2:70-93 HWI-ST967_77:2:1204:4732:5009.
cagtagcttacagaacgcaaacaa---------------------
>gnl|SRA|SRR1171870.53092234.1:76-99 HWI-ST967_77:2:1203:4675:181034.
cagtagcttacagaacgcaaacaa---------------------
>gnl|SRA|SRR1171870.51395187.2:77-100 HWI-ST967_77:2:1203:17765:120632.
cagtagcttacagaacgcaaacaa---------------------
>_R_gnl|SRA|SRR1171870.46986197.2:2-25 HWI-ST967_77:2:1202:5024:159582.
cagtagcttacagaacgcaaacaa---------------------
>gnl|SRA|SRR1171870.45393125.2:74-97 HWI-ST967_77:2:1202:15940:99905.
cagtagcttacagaacgcaaacaa---------------------
>gnl|SRA|SRR1171870.43604027.2:75-98 HWI-ST967_77:2:1202:16164:33175.
cagtagcttacagaacgcaaacaa---------------------
>gnl|SRA|SRR1171870.42609302.1:76-99 HWI-ST967_77:2:1201:7369:194767.
cagtagcttacagaacgcaaacaa---------------------
>_R_gnl|SRA|SRR1171870.40539825.1:4-27 HWI-ST967_77:2:1201:19296:116608.
cagtagcttacagaacgcaaacaa---------------------
>_R_gnl|SRA|SRR1171870.39290101.1:5-28 HWI-ST967_77:2:1201:3812:68896.
cagtagcttacagaacgcaaacaa---------------------
>_R_gnl|SRA|SRR1171870.38760214.1:1-24 HWI-ST967_77:2:1201:10946:48560.
cagtagcttacagaacgcaaacaa---------------------
>_R_gnl|SRA|SRR1171870.27676856.1:9-32 HWI-ST967_77:2:1106:8306:39818.
cagtagcttacagaacgcaaacaa---------------------
>_R_gnl|SRA|SRR1171870.27670807.1:1-24 HWI-ST967_77:2:1106:11191:39553.
cagtagcttacagaacgcaaacaa---------------------
>_R_gnl|SRA|SRR1171870.22763729.1:1-24 HWI-ST967_77:2:1105:2116:63467.
cagtagcttacagaacgcaaacaa---------------------
>gnl|SRA|SRR1171870.18302045.2:70-93 HWI-ST967_77:2:1104:1718:97085.
cagtagcttacagaacgcaaacaa---------------------
>gnl|SRA|SRR1171870.17816989.2:70-93 HWI-ST967_77:2:1104:5300:78956.
cagtagcttacagaacgcaaacaa---------------------
>gnl|SRA|SRR1171870.13180895.2:76-99 HWI-ST967_77:2:1103:3568:106945.
cagtagcttacagaacgcaaacaa---------------------
>_R_gnl|SRA|SRR1171870.12158153.2:4-27 HWI-ST967_77:2:1103:13642:68659.
cagtagcttacagaacgcaaacaa---------------------
>gnl|SRA|SRR1171870.11707770.2:77-100 HWI-ST967_77:2:1103:15288:51955.
cagtagcttacagaacgcaaacaa---------------------

>_R_gnl|SRA|SRR1171870.10552543.1:1-24 HWI-ST967_77:2:1103:12892:9010.
cagtagcttacagaacgcaaacaa---------------------
>gnl|SRA|SRR1171870.8732742.2:69-92 HWI-ST967_77:2:1102:15242:137698.
cagtagcttacagaacgcaaacaa---------------------
>_R_gnl|SRA|SRR1171870.8732742.1:4-27 HWI-ST967_77:2:1102:15242:137698.
cagtagcttacagaacgcaaacaa---------------------
>gnl|SRA|SRR1171870.5003781.1:75-98 HWI-ST967_77:2:1101:16841:192600.
cagtagcttacagaacgcaaacaa---------------------
>_R_gnl|SRA|SRR1171870.3519554.1:1-24 HWI-ST967_77:2:1101:14678:136907.
cagtagcttacagaacgcaaacaa---------------------
>gnl|SRA|SRR1171870.2056309.2:70-93 HWI-ST967_77:2:1101:12437:81048.
cagtagcttacagaacgcaaacaa---------------------
>_R_gnl|SRA|SRR1171870.203295069.1:3-26 HWI-ST967_77:2:2305:2631:185317.
cagtagcttacagagcgcaaacaa---------------------
>gnl|SRA|SRR1171870.203753478.2:70-93 HWI-ST967_77:2:2306:8060:3441.
cagtagcttacagaacgcgagcaa---------------------
>_R_gnl|SRA|SRR1171870.207018350.1:8-31 HWI-ST967_77:2:2306:6385:124440.
cagtagcttacagaaagcaaacaa---------------------
>gnl|SRA|SRR1171870.183888956.2:70-93 HWI-ST967_77:2:2302:1625:66873.
caggagcttacagaacgcaaacaa---------------------
>_R_gnl|SRA|SRR1171870.72412664.1:9-32 HWI-ST967_77:2:1208:12494:35245.
cactagcttacagaacgcaaacaa---------------------
>gnl|SRA|SRR1171870.40789418.1:70-93 HWI-ST967_77:2:1201:3862:126387.
cattagcttacagaacgcaaacaa---------------------
>gnl|SRA|SRR1171870.22043979.2:70-93 HWI-ST967_77:2:1105:12706:37340.
cagtagcgtacagaacgcaaacaa---------------------
>_R_gnl|SRA|SRR1171870.206258002.1:1-22 HWI-ST967_77:2:2306:18323:95605.
cagtagcttacagaacgcaaac-----------------------
>gnl|SRA|SRR1171870.201003106.2:79-100 HWI-ST967_77:2:2305:7180:102012.
cagtagcttacagaacgcaaac-----------------------
>_R_gnl|SRA|SRR1171870.190570907.1:1-22 HWI-ST967_77:2:2303:13798:118270.
cagtagcttacagaacgcaaac-----------------------
>_R_gnl|SRA|SRR1171870.187544210.1:1-22 HWI-ST967_77:2:2303:8261:7527.
cagtagcttacagaacgcaaac-----------------------
>gnl|SRA|SRR1171870.177610894.2:79-100 HWI-ST967_77:2:2301:20517:23840.
cagtagcttacagaacgcaaac-----------------------
>_R_gnl|SRA|SRR1171870.169183202.1:2-23 HWI-ST967_77:2:2205:11875:136662.
cagtagcttacagaacgcaaac-----------------------
>gnl|SRA|SRR1171870.159613475.2:79-100 HWI-ST967_77:2:2203:7012:191998.
cagtagcttacagaacgcaaac-----------------------
>gnl|SRA|SRR1171870.155299725.2:79-100 HWI-ST967_77:2:2203:7059:36395.
cagtagcttacagaacgcaaac-----------------------
>_R_gnl|SRA|SRR1171870.145814589.1:1-22 HWI-ST967_77:2:2201:15644:79748.
cagtagcttacagaacgcaaac-----------------------
>gnl|SRA|SRR1171870.143889275.2:79-100 HWI-ST967_77:2:2201:9829:6200.
cagtagcttacagaacgcaaac-----------------------
>gnl|SRA|SRR1171870.135992093.2:79-100 HWI-ST967_77:2:2105:9052:161805.
cagtagcttacagaacgcaaac-----------------------
>_R_gnl|SRA|SRR1171870.132597027.1:1-22 HWI-ST967_77:2:2105:3686:35664.
cagtagcttacagaacgcaaac-----------------------
>_R_gnl|SRA|SRR1171870.131377129.1:1-22 HWI-ST967_77:2:2104:2811:189820.

cagtagcttacagaacgcaaac-----------------------
>gnl|SRA|SRR1171870.119619854.1:79-100 HWI-ST967_77:2:2102:3051:151700.
cagtagcttacagaacgcaaac-----------------------
>_R_gnl|SRA|SRR1171870.113060456.2:1-22 HWI-ST967_77:2:2101:5948:97181.
cagtagcttacagaacgcaaac-----------------------
>_R_gnl|SRA|SRR1171870.112535914.1:1-22 HWI-ST967_77:2:2101:20453:76312.
cagtagcttacagaacgcaaac-----------------------
>_R_gnl|SRA|SRR1171870.92329496.2:1-22 HWI-ST967_77:2:1304:19731:111539.
cagtagcttacagaacgcaaac-----------------------
>_R_gnl|SRA|SRR1171870.85095161.1:1-22 HWI-ST967_77:2:1303:4450:51499.
cagtagcttacagaacgcaaac-----------------------
>_R_gnl|SRA|SRR1171870.84157090.1:1-22 HWI-ST967_77:2:1303:11513:17390.
cagtagcttacagaacgcaaac-----------------------
>_R_gnl|SRA|SRR1171870.82298985.1:1-22 HWI-ST967_77:2:1302:10606:147806.
cagtagcttacagaacgcaaac-----------------------
>_R_gnl|SRA|SRR1171870.76890486.1:1-22 HWI-ST967_77:2:1301:11544:140551.
cagtagcttacagaacgcaaac-----------------------
>_R_gnl|SRA|SRR1171870.52511645.2:1-22 HWI-ST967_77:2:1203:18619:160357.
cagtagcttacagaacgcaaac-----------------------
>gnl|SRA|SRR1171870.52458991.2:79-100 HWI-ST967_77:2:1203:9066:158714.
cagtagcttacagaacgcaaac-----------------------
>_R_gnl|SRA|SRR1171870.46309954.1:1-22 HWI-ST967_77:2:1202:9698:134280.
cagtagcttacagaacgcaaac-----------------------
>_R_gnl|SRA|SRR1171870.42920607.2:1-22 HWI-ST967_77:2:1202:5048:8085.
cagtagcttacagaacgcaaac-----------------------
>_R_gnl|SRA|SRR1171870.34653071.1:1-22 HWI-ST967_77:2:1107:2153:113742.
cagtagcttacagaacgcaaac-----------------------
>gnl|SRA|SRR1171870.24650180.2:79-100 HWI-ST967_77:2:1105:2402:131419.
cagtagcttacagaacgcaaac-----------------------
>_R_gnl|SRA|SRR1171870.23239420.1:1-22 HWI-ST967_77:2:1105:16104:80320.
cagtagcttacagaacgcaaac-----------------------
>gnl|SRA|SRR1171870.19835518.1:79-100 HWI-ST967_77:2:1104:14301:155024.
cagtagcttacagaacgcaaac-----------------------
>_R_gnl|SRA|SRR1171870.17649976.1:1-22 HWI-ST967_77:2:1104:11886:72594.
cagtagcttacagaacgcaaac-----------------------
>_R_gnl|SRA|SRR1171870.16822499.1:1-22 HWI-ST967_77:2:1104:12076:42424.
cagtagcttacagaacgcaaac-----------------------
>gnl|SRA|SRR1171870.15267027.2:79-100 HWI-ST967_77:2:1103:6326:184266.
cagtagcttacagaacgcaaac-----------------------
>_R_gnl|SRA|SRR1171870.15118043.1:1-22 HWI-ST967_77:2:1103:4193:178863.
cagtagcttacagaacgcaaac-----------------------
>gnl|SRA|SRR1171870.1006158.1:79-100 HWI-ST967_77:2:1101:7012:40948.
cagtagcttacagaacgcaaac-----------------------
>gnl|SRA|SRR1171870.52632503.2:79-100 HWI-ST967_77:2:1203:1185:164878.
cagtaacttacagaacgcaaac-----------------------
>gnl|SRA|SRR1171870.149150944.2:79-100 HWI-ST967_77:2:2202:16988:8051.
caggagcttacagaacgcaaac-----------------------
>_R_gnl|SRA|SRR1171870.6788502.2:1-22 HWI-ST967_77:2:1102:20145:61890.
cattagcttacagaacgcaaac-----------------------
>_R_gnl|SRA|SRR1171870.204973720.1:1-21 HWI-ST967_77:2:2306:1322:48095.
cagtagcttacagaacgcaaa------------------------

>gnl|SRA|SRR1171870.200040599.2:80-100 HWI-ST967_77:2:2305:8209:64459.
cagtagcttacagaacgcaaa------------------------
>_R_gnl|SRA|SRR1171870.189045194.1:1-21 HWI-ST967_77:2:2303:12984:62498.
cagtagcttacagaacgcaaa------------------------
>_R_gnl|SRA|SRR1171870.188326706.1:1-21 HWI-ST967_77:2:2303:8755:36207.
cagtagcttacagaacgcaaa------------------------
>gnl|SRA|SRR1171870.183308552.1:80-100 HWI-ST967_77:2:2302:12007:44970.
cagtagcttacagaacgcaaa------------------------
>_R_gnl|SRA|SRR1171870.180909251.1:1-21 HWI-ST967_77:2:2301:18023:152415.
cagtagcttacagaacgcaaa------------------------
>_R_gnl|SRA|SRR1171870.166287458.1:1-21 HWI-ST967_77:2:2205:16791:31582.
cagtagcttacagaacgcaaa------------------------
>gnl|SRA|SRR1171870.141060156.2:80-100 HWI-ST967_77:2:2106:3883:149891.
cagtagcttacagaacgcaaa------------------------
>_R_gnl|SRA|SRR1171870.49224217.1:1-21 HWI-ST967_77:2:1203:14413:42206.
cagtagcttacagaacgcaaa------------------------
>gnl|SRA|SRR1171870.193785656.2:81-100 HWI-ST967_77:2:2304:7286:37889.
cagtagcttacagaacgcaa-------------------------
>gnl|SRA|SRR1171870.183920398.1:81-100 HWI-ST967_77:2:2302:18122:67974.
cagtagcttacagaacgcaa-------------------------
>gnl|SRA|SRR1171870.174499527.1:81-100 HWI-ST967_77:2:2206:11850:152010.
cagtagcttacagaacgcaa-------------------------
>gnl|SRA|SRR1171870.157640193.2:81-100 HWI-ST967_77:2:2203:11139:120754.
cagtagcttacagaacgcaa-------------------------
>gnl|SRA|SRR1171870.152667682.2:81-100 HWI-ST967_77:2:2202:17522:139460.
cagtagcttacagaacgcaa-------------------------
>gnl|SRA|SRR1171870.137245389.2:81-100 HWI-ST967_77:2:2106:11204:9274.
cagtagcttacagaacgcaa-------------------------
>gnl|SRA|SRR1171870.128463075.2:81-100 HWI-ST967_77:2:2104:7911:82801.
cagtagcttacagaacgcaa-------------------------
>gnl|SRA|SRR1171870.128346665.2:81-100 HWI-ST967_77:2:2104:6338:78748.
cagtagcttacagaacgcaa-------------------------
>gnl|SRA|SRR1171870.128075150.2:81-100 HWI-ST967_77:2:2104:10367:68577.
cagtagcttacagaacgcaa-------------------------
>_R_gnl|SRA|SRR1171870.127345517.2:2-21 HWI-ST967_77:2:2104:7832:41811.
cagtagcttacagaacgcaa-------------------------
>gnl|SRA|SRR1171870.117868376.1:81-100 HWI-ST967_77:2:2102:6764:83955.
cagtagcttacagaacgcaa-------------------------
>gnl|SRA|SRR1171870.112846226.1:81-100 HWI-ST967_77:2:2101:17379:88512.
cagtagcttacagaacgcaa-------------------------
>gnl|SRA|SRR1171870.102374482.1:81-100 HWI-ST967_77:2:1306:14187:69997.
cagtagcttacagaacgcaa-------------------------
>gnl|SRA|SRR1171870.80187396.2:81-100 HWI-ST967_77:2:1302:2421:65976.
cagtagcttacagaacgcaa-------------------------
>gnl|SRA|SRR1171870.79469834.2:81-100 HWI-ST967_77:2:1302:15587:38756.
cagtagcttacagaacgcaa-------------------------
>gnl|SRA|SRR1171870.63722463.2:81-100 HWI-ST967_77:2:1205:19402:165906.
cagtagcttacagaacgcaa-------------------------
>_R_gnl|SRA|SRR1171870.59672787.2:1-20 HWI-ST967_77:2:1205:6968:19941.
cagtagcttacagaacgcaa-------------------------
>_R_gnl|SRA|SRR1171870.58143332.1:1-20 HWI-ST967_77:2:1204:4718:164301.

cagtagcttacagaacgcaa-------------------------
>gnl|SRA|SRR1171870.50592355.1:81-100 HWI-ST967_77:2:1203:18224:91602.
cagtagcttacagaacgcaa-------------------------
>gnl|SRA|SRR1171870.47259541.1:81-100 HWI-ST967_77:2:1202:6918:169522.
cagtagcttacagaacgcaa-------------------------
>gnl|SRA|SRR1171870.46105850.1:81-100 HWI-ST967_77:2:1202:19130:126747.
cagtagcttacagaacgcaa-------------------------
>gnl|SRA|SRR1171870.40125786.2:81-100 HWI-ST967_77:2:1201:4803:100947.
cagtagcttacagaacgcaa-------------------------
>gnl|SRA|SRR1171870.16990646.1:81-100 HWI-ST967_77:2:1104:4284:48578.
cagtagcttacagaacgcaa-------------------------
>gnl|SRA|SRR1171870.15033717.1:81-100 HWI-ST967_77:2:1103:13498:175623.
cagtagcttacagaacgcaa-------------------------
>gnl|SRA|SRR1171870.11942882.2:81-100 HWI-ST967_77:2:1103:14769:60568.
cagtagcttacagaacgcaa-------------------------
>gnl|SRA|SRR1171870.6788502.1:81-100 HWI-ST967_77:2:1102:20145:61890.
cagtagcttacagaacgcaa-------------------------
>gnl|SRA|SRR1171870.6611074.2:81-100 HWI-ST967_77:2:1102:19578:55208.
cagtagcttacagaacgcaa-------------------------
>gnl|SRA|SRR1171870.4208205.2:81-100 HWI-ST967_77:2:1101:16200:162958.
cagtagcttacagaacgcaa-------------------------
>gnl|SRA|SRR1171870.2067406.1:81-100 HWI-ST967_77:2:1101:6572:81707.
cagtagcttacagaacgcaa-------------------------
>gnl|SRA|SRR1171870.73212461.1:56-97 HWI-ST967_77:2:1208:4235:176508.
cagttgcttacagaacgcaaacaa---gaccatacttggtgattc
>gnl|SRA|SRR1171870.201658364.2:82-100 HWI-ST967_77:2:2305:15432:126404.
cagtagcttacagaacgca--------------------------
>_R_gnl|SRA|SRR1171870.179514408.1:1-19 HWI-ST967_77:2:2301:13340:97739.
cagtagcttacagaacgca--------------------------
>gnl|SRA|SRR1171870.160614058.1:82-100 HWI-ST967_77:2:2204:18694:28757.
cagtagcttacagaacgca--------------------------
>gnl|SRA|SRR1171870.146211223.1:82-100 HWI-ST967_77:2:2201:9992:94847.
cagtagcttacagaacgca--------------------------
>_R_gnl|SRA|SRR1171870.145550475.2:1-19 HWI-ST967_77:2:2201:15500:69656.
cagtagcttacagaacgca--------------------------
>gnl|SRA|SRR1171870.145128247.2:82-100 HWI-ST967_77:2:2201:13526:53503.
cagtagcttacagaacgca--------------------------
>gnl|SRA|SRR1171870.127192664.2:82-100 HWI-ST967_77:2:2104:17438:36034.
cagtagcttacagaacgca--------------------------
>gnl|SRA|SRR1171870.123585113.2:82-100 HWI-ST967_77:2:2103:16282:101474.
cagtagcttacagaacgca--------------------------
>_R_gnl|SRA|SRR1171870.118331495.1:1-19 HWI-ST967_77:2:2102:8592:101888.
cagtagcttacagaacgca--------------------------
>_R_gnl|SRA|SRR1171870.117786393.1:1-19 HWI-ST967_77:2:2102:12582:80650.
cagtagcttacagaacgca--------------------------
>gnl|SRA|SRR1171870.115944598.2:82-100 HWI-ST967_77:2:2102:16531:10446.
cagtagcttacagaacgca--------------------------
>gnl|SRA|SRR1171870.102722425.1:82-100 HWI-ST967_77:2:1306:17147:82275.
cagtagcttacagaacgca--------------------------
>_R_gnl|SRA|SRR1171870.76510157.2:1-19 HWI-ST967_77:2:1301:19598:126236.
cagtagcttacagaacgca--------------------------

>_R_gnl|SRA|SRR1171870.74249193.1:1-19 HWI-ST967_77:2:1301:20726:39442.
cagtagcttacagaacgca--------------------------
>gnl|SRA|SRR1171870.28668163.2:82-100 HWI-ST967_77:2:1106:13747:75239.
cagtagcttacagaacgca--------------------------
>_R_gnl|SRA|SRR1171870.20644308.2:1-19 HWI-ST967_77:2:1104:4194:185618.
cagtagcttacagaacgca--------------------------
>gnl|SRA|SRR1171870.17940281.1:82-100 HWI-ST967_77:2:1104:11418:83370.
cagtagcttacagaacgca--------------------------
>_R_gnl|SRA|SRR1171870.10624340.1:1-19 HWI-ST967_77:2:1103:5987:11802.
cagtagcttacagaacgca--------------------------
>gnl|SRA|SRR1171870.6957244.2:82-100 HWI-ST967_77:2:1102:13808:68435.
cagtagcttacagaacgca--------------------------
>gnl|SRA|SRR1171870.110489699.1:70-100 HWI-ST967_77:2:1308:9279:164051.
cagtagcttacagaatgcaaacaa---gaccata-----------
>gnl|SRA|SRR1171870.181788096.1:76-100 HWI-ST967_77:2:2301:10530:185846.
cagtagcttacagaaagcaaacaa---g-----------------
>gnl|SRA|SRR1171870.32190490.2:76-100 HWI-ST967_77:2:1107:12028:4042.
cagtagcttacagaacccaaacaa---g-----------------
>_R_gnl|SRA|SRR1171870.132561792.1:62-100 HWI-ST967_77:2:2105:18971:34183.
-agtagcttacagagcgcaaacaa---gaccatagtgggggat--
>_R_gnl|SRA|SRR1171870.108934811.1:1-42 HWI-ST967_77:2:1307:10690:178557.
-agtagcttacagaacgcaaacaatcggaacatagtgggggat--
>gnl|SRA|SRR1171870.32573838.1:63-100 HWI-ST967_77:2:1107:9394:20573.
-----gcttacagaacgcaaacaataggaccatagtgggggat--
>_R_gnl|SRA|SRR1171870.71925006.2:18-58 HWI-ST967_77:2:1208:13721:8165.
--gtngcttacagaacgcaaacaatagnaccatantgnnggat--
>_R_gnl|SRA|SRR1171870.114936713.1:63-100 HWI-ST967_77:2:2101:8793:170152.
--gtagcttacagaacgcaaacaaga---ccgtagtgggggat--
>_R_gnl|SRA|SRR1171870.115517433.2:78-100 HWI-ST967_77:2:2101:13191:192354.
--gtagcttacagaacgcaaacaag--------------------
>gnl|SRA|SRR1171870.19212494.2:1-23 HWI-ST967_77:2:1104:9646:131741.
--gtagcttacagaacgcaaacaag--------------------
>gnl|SRA|SRR1171870.63046459.2:78-99 HWI-ST967_77:2:1205:11181:141603.
--gtagcttacagaacgcaaacaa---------------------
>_R_gnl|SRA|SRR1171870.108051400.2:1-22 HWI-ST967_77:2:1307:13524:120990.
--gtagcttacagaccgcaaacaa---------------------
>gnl|SRA|SRR1171870.1151918.2:23-46 HWI-ST967_77:2:1101:13770:46377.
-agtagcttacagaacgcaaacaag--------------------
>gnl|SRA|SRR1171870.198856771.2:77-100 HWI-ST967_77:2:2305:3089:21245.
-agtagcgtacaggacgcaaacaag--------------------
>gnl|SRA|SRR1171870.208843823.2:80-100 HWI-ST967_77:2:2306:2289:194626.
----agcttacagaacgcaaacaag--------------------
>_R_gnl|SRA|SRR1171870.127680697.1:1-20 HWI-ST967_77:2:2104:10699:54163.
--gtagcttacagaacgcaaac-----------------------
>_R_gnl|SRA|SRR1171870.72387694.2:34-74 HWI-ST967_77:2:1208:4640:32580.
cagtagattacttaangcnaacaa---gaccatagtgggtgatt-
>gnl|SRA|SRR1171870.204511149.2:58-98 HWI-ST967_77:2:2306:10253:31131.
cagtgacgtacataacgcaaacaa---gaccatagtagggcatt-
>_R_gnl|SRA|SRR1171870.47235413.1:64-100 HWI-ST967_77:2:1202:15366:168635.
-----gcttacagaacgcaaaaaa---gcccatagtgggacattc
>gnl|SRA|SRR1171870.32970992.2:24-63 HWI-ST967_77:2:1107:11668:37881.

--gtaggttacataacgcgaccaa---gaccatagtgttggattc
>gnl|SRA|SRR1171870.100759245.2:33-76 HWI-ST967_77:2:1306:11417:13152.
-aggagcttacagaactcaaacaatatgaccatattgggggattc
>_R_gnl|SRA|SRR1171870.103682737.2:14-57 HWI-ST967_77:2:1306:15211:117310.
-agtagcccagagaacgcaaacaataggtccatactgggggattc
>_R_gnl|SRA|SRR1171870.102722425.2:59-100 HWI-ST967_77:2:1306:17147:82275.
---tagcttacagaacgaaaacaataggaccatagtgccggattc
>_R_gnl|SRA|SRR1171870.2067406.2:55-94 HWI-ST967_77:2:1101:6572:81707.
cagtagcttacagcgcctaaacaagaggaccatagtgggg-----
>_R_gnl|SRA|SRR1171870.100632960.2:1-44 HWI-ST967_77:2:1306:18623:8750.
cagtagcttaccgaccgcaaaccataggagcctagtgggggatt-
>_R_gnl|SRA|SRR1171870.37365661.1:7-33 HWI-ST967_77:2:1108:9289:155116.
cagtagctgacagaacgcaaacaatac------------------
>_R_gnl|SRA|SRR1171870.174532332.2:67-100 HWI-ST967_77:2:2206:11639:153585.
-------ttacagaacgaaaacaa---gacaatagtgggggatt-
>gnl|SRA|SRR1171870.103855064.2:24-63 HWI-ST967_77:2:1306:13666:123637.
-aggagctttcagaacgcaaacaa---gacgatagtggtgtatt-
>gnl|SRA|SRR1171870.208530793.2:24-46 HWI-ST967_77:2:2306:5854:181865.
-------ttacagaacgcaaacaa---gaacat------------
>_R_gnl|SRA|SRR1171870.205487236.2:59-80 HWI-ST967_77:2:2306:13185:66856.
--------tacataacgcaaacaa---gaccat------------
>gnl|SRA|SRR1171870.72520087.1:60-81 HWI-ST967_77:2:1208:15455:59005.
--------tacataacgcaaacaa---gaccat------------
>gnl|SRA|SRR1171870.176653988.2:75-96 HWI-ST967_77:2:2208:4375:109309.
--------tacagaacgcaaacaa---gaacat------------
>gnl|SRA|SRR1171870.175308111.2:30-51 HWI-ST967_77:2:2206:16062:190969.
--------tacagaacgcaaacaa---gaacat------------
>gnl|SRA|SRR1171870.172038716.2:62-83 HWI-ST967_77:2:2206:3369:45549.
--------tacagaacgcaaacaa---gaacat------------
>gnl|SRA|SRR1171870.110038127.1:70-91 HWI-ST967_77:2:1308:19039:86303.
--------tacagaacgcaaacaa---gaacat------------
>gnl|SRA|SRR1171870.106145884.1:23-44 HWI-ST967_77:2:1307:11201:11205.
--------tacagaacgcaaacaa---gaacat------------
>gnl|SRA|SRR1171870.105185983.2:70-91 HWI-ST967_77:2:1306:1434:171930.
--------tacagaacgcaaacaa---gaacat------------
>gnl|SRA|SRR1171870.104425211.2:63-84 HWI-ST967_77:2:1306:9183:144423.
--------tacagaacgcaaacaa---gaacat------------
>gnl|SRA|SRR1171870.73253522.1:26-47 HWI-ST967_77:2:1208:16770:188543.
--------tacagaacgcaaacaa---gaacat------------
>gnl|SRA|SRR1171870.65112301.2:23-44 HWI-ST967_77:2:1206:16492:17272.
--------tacagaacgcaaacaa---gaacat------------
>_R_gnl|SRA|SRR1171870.40774898.1:66-87 HWI-ST967_77:2:1201:18698:125653.
--------tacagaacgcaaacaa---gaacat------------
>gnl|SRA|SRR1171870.35233606.2:64-85 HWI-ST967_77:2:1107:14910:140800.
--------tacagaacgcaaacaa---gaacat------------
>gnl|SRA|SRR1171870.108666710.2:47-68 HWI-ST967_77:2:1307:8666:160409.
--------tacagaacgcaaacac---gaccat------------
>gnl|SRA|SRR1171870.34971586.2:39-61 HWI-ST967_77:2:1107:6351:128637.
--------tacagaacgcaaacaa---gaacata-----------
>_R_gnl|SRA|SRR1171870.206312355.2:74-97 HWI-ST967_77:2:2306:20470:97508.
-------ttacagtacgtaaacaa---gaccata-----------

>gnl|SRA|SRR1171870.175094762.2:63-86 HWI-ST967_77:2:2206:11026:180607.
-------ttacgtaacgcaaacaa---gaccata-----------
>gnl|SRA|SRR1171870.59178525.2:62-85 HWI-ST967_77:2:1205:16002:2317.
-------ttacagaacgcaaataa---gaacata-----------
>gnl|SRA|SRR1171870.27552202.2:46-70 HWI-ST967_77:2:1106:18773:35410.
--------tacataacgcaaataa---gaccatagt---------
>_R_gnl|SRA|SRR1171870.176433378.1:6-34 HWI-ST967_77:2:2208:16421:74742.
-agtagcgtacataacgcaaacaa---caacat------------
>_R_gnl|SRA|SRR1171870.104951027.2:63-86 HWI-ST967_77:2:1306:6391:163314.
------cttacataacgcaaacaa---caccat------------
>_R_gnl|SRA|SRR1171870.13797218.2:38-79 HWI-ST967_77:2:1103:17963:129577.
cagtagcttacagaacaaaaacaa---gcccatggtgggggattc
>gnl|SRA|SRR1171870.27069344.1:70-98 HWI-ST967_77:2:1106:7690:18571.
cagtagcttacagctggcaaaaaa---gacca-------------
>gnl|SRA|SRR1171870.171315039.2:67-95 HWI-ST967_77:2:2206:9609:15898.
----------cagaactcaaacaa---gaccctagtaggtga---
>gnl|SRA|SRR1171870.110272097.2:62-80 HWI-ST967_77:2:1308:5630:123018.
-----------------caaacaa---gaccatagtggg------
>gnl|SRA|SRR1171870.176909644.1:65-87 HWI-ST967_77:2:2208:5859:158644.
--------tacagaacgcaaacaa---caccata-----------
>_R_gnl|SRA|SRR1171870.99293209.1:68-90 HWI-ST967_77:2:1305:10199:160044.
--------tacagaacgcaaacaa---caccata-----------
>_R_gnl|SRA|SRR1171870.143684883.1:67-88 HWI-ST967_77:2:2108:14072:76129.
--------tacagaacgcaaacaa---caccat------------
>gnl|SRA|SRR1171870.142819363.2:78-99 HWI-ST967_77:2:2107:5183:56498.
--------tacagaacgcaaacaa---caccat------------
>gnl|SRA|SRR1171870.142553232.2:78-99 HWI-ST967_77:2:2107:7525:19426.
--------tacagaacgcaaacaa---caccat------------
>gnl|SRA|SRR1171870.108792610.2:47-68 HWI-ST967_77:2:1307:11916:168950.
--------tacagaacgcaaacaa---caccat------------
>gnl|SRA|SRR1171870.105708104.2:63-84 HWI-ST967_77:2:1306:13577:190267.
--------tacagaacgcaaacaa---caccat------------
>gnl|SRA|SRR1171870.100997500.2:62-83 HWI-ST967_77:2:1306:15607:21450.
--------tacagaacgcaaacaa---caccat------------
>_R_gnl|SRA|SRR1171870.93662306.2:59-80 HWI-ST967_77:2:1304:3798:158896.
--------tacagaacgcaaacaa---caccat------------
>_R_gnl|SRA|SRR1171870.77544536.2:59-80 HWI-ST967_77:2:1301:7085:165415.
--------tacagaacgcaaacaa---caccat------------
>_R_gnl|SRA|SRR1171870.67014034.1:58-79 HWI-ST967_77:2:1206:6037:85966.
--------tacagaacgcaaacaa---caccat------------
>gnl|SRA|SRR1171870.66426640.2:21-42 HWI-ST967_77:2:1206:14661:64314.
--------tacagaacgcaaacaa---caccat------------
>gnl|SRA|SRR1171870.65757166.2:26-47 HWI-ST967_77:2:1206:12873:40373.
--------tacagaacgcaaacaa---caccat------------
>gnl|SRA|SRR1171870.33513685.2:24-45 HWI-ST967_77:2:1107:8469:61770.
--------tacagaacgcaaacaa---caccat------------
>gnl|SRA|SRR1171870.32586925.2:24-45 HWI-ST967_77:2:1107:13503:21014.
--------tacagaacgcaaacaa---caccat------------
>_R_gnl|SRA|SRR1171870.28674803.2:64-85 HWI-ST967_77:2:1106:12979:75467.
--------tacagaacgcaaacaa---caccat------------
>_R_gnl|SRA|SRR1171870.5944826.2:65-86 HWI-ST967_77:2:1102:2374:30103.

--------tacagaacgcaaacaa---caccat------------
>_R_gnl|SRA|SRR1171870.145550920.2:53-75 HWI-ST967_77:2:2201:16788:69716.
-------ttacagaacgcaaacaa---caccat------------
>_R_gnl|SRA|SRR1171870.176699173.2:60-81 HWI-ST967_77:2:2208:13877:117289.
-------ttacagaacgcaaacaa---cacca-------------
>gnl|SRA|SRR1171870.107637712.2:45-69 HWI-ST967_77:2:1307:2413:95809.
------cttacagaacgcaaacaa---caccgta-----------
>gnl|SRA|SRR1171870.208103657.2:26-49 HWI-ST967_77:2:2306:6217:165842.
-------ttacagaacgcaaacaa---caacata-----------
>_R_gnl|SRA|SRR1171870.146197167.1:62-85 HWI-ST967_77:2:2201:6690:94492.
-------ttacagaacgcaaacaa---caacata-----------
>gnl|SRA|SRR1171870.33465145.2:64-87 HWI-ST967_77:2:1107:15416:59609.
-------ttacagaacgcaaacaa---caacata-----------
>gnl|SRA|SRR1171870.143164191.1:25-51 HWI-ST967_77:2:2107:14385:104663.
----agattacagaacgcaaacaa---caacata-----------
>gnl|SRA|SRR1171870.32726337.2:52-75 HWI-ST967_77:2:1107:21029:27080.
------cttacagaacgcaaacaa---caacat------------
>gnl|SRA|SRR1171870.105436933.2:20-43 HWI-ST967_77:2:1306:16097:180674.
---------acagaacgcaaacaa---caacatagt---------
>_R_gnl|SRA|SRR1171870.70885642.2:70-93 HWI-ST967_77:2:1207:17879:78147.
------cttacagaacgaaaacac---gaccat------------
>gnl|SRA|SRR1171870.72516021.2:66-88 HWI-ST967_77:2:1208:16717:58094.
-------------aacgcaaacag---gaccatagtggg------
>gnl|SRA|SRR1171870.70954877.2:30-53 HWI-ST967_77:2:1207:15161:85339.
-----------agaacgcaagaca--agaccatagtg--------

SRX2342718
>control .
agcctgcagaac-tcaaacagtaggtccttagtgaag
>gnl|SRA|SRR5012160.16015436.1:103-139 16015436.
agcctgcagaac-tcaaacagtaggtccttagtgaag
>gnl|SRA|SRR5012160.15877169.1:68-104 15877169.
agcctgcagaac-tcaaacagtaggtccttagtgaag
>gnl|SRA|SRR5012160.15358334.1:68-104 15358334.
agcctgcagaac-tcaaacagtaggtccttagtgaag
>gnl|SRA|SRR5012160.12989578.1:99-135 12989578.
agcctgcagaac-tcaaacagtaggtccttagtgaag
>gnl|SRA|SRR5012160.12710636.1:22-58 12710636.
agcctgcagaac-tcaaacagtaggtccttagtgaag
>gnl|SRA|SRR5012160.12409757.1:59-95 12409757.
agcctgcagaac-tcaaacagtaggtccttagtgaag
>gnl|SRA|SRR5012160.11042335.1:103-139 11042335.
agcctgcagaac-tcaaacagtaggtccttagtgaag
>gnl|SRA|SRR5012160.9615244.1:91-127 9615244.
agcctgcagaac-tcaaacagtaggtccttagtgaag
>gnl|SRA|SRR5012160.7802423.1:62-98 7802423.
agcctgcagaac-tcaaacagtaggtccttagtgaag
>gnl|SRA|SRR5012160.5394596.1:67-103 5394596.
agcctgcagaac-tcaaacagtaggtccttagtgaag
>gnl|SRA|SRR5012160.4377524.1:45-81 4377524.

agcctgcagaac-tcaaacagtaggtccttagtgaag
>gnl|SRA|SRR5012160.3992010.1:76-112 3992010.
agcctgcagaac-tcaaacagtaggtccttagtgaag
>gnl|SRA|SRR5012160.1674303.1:62-98 1674303.
agcctgcagaac-tcaaacagtaggtccttagtgaag
>gnl|SRA|SRR5012160.1636070.1:106-142 1636070.
agcctgcagaac-tcaaacagtaggtccttagtgaag
>gnl|SRA|SRR5012160.5545825.1:79-114 5545825.
agcctgcagaac-tcaaacagtaggtccttagtgaag
>gnl|SRA|SRR5012160.15475861.1:144-177 15475861.
agcctgcagaac-tcaaacagtaggtccttagtga--
>gnl|SRA|SRR5012160.3179582.1:1-30 3179582.
-------agaac-tcaaacagtaggtccttagtgaag
>gnl|SRA|SRR5012160.1326489.1:1-30 1326489.
-------agaac-tcaaacagtaggtccttagtgaag
>gnl|SRA|SRR5012160.9293594.1:1-29 9293594.
--------gaac-tcaaacagtaggtccttagtgaag
>gnl|SRA|SRR5012160.3631493.1:1-29 3631493.
--------gaac-tcaaacagtaggtccttagtgaag
>gnl|SRA|SRR5012160.9852278.1:1-19 9852278.
-------------------agtaggtccttagtgaag
>gnl|SRA|SRR5012160.6518239.1:1-19 6518239.
-------------------agtaggtccttagtgaag
>gnl|SRA|SRR5012160.8680416.1:59-92 8680416.
agcctgcagaac-tcaaacag---gtccttagtgaag
>gnl|SRA|SRR5012160.6522379.1:60-93 6522379.
agcctgcagaac-tcaaacag---gtccttagtgaag
>gnl|SRA|SRR5012160.5449764.1:22-55 5449764.
agcctgcagaac-tcaaacag---gtccttagtgaag
>gnl|SRA|SRR5012160.4455717.1:28-61 4455717.
agcctgcagaac-tcaaacag---gtccttagtgaag
>gnl|SRA|SRR5012160.6053381.1:103-135 6053381.
agcctgcagaac-tcaaacag---gtcctta-tgaag
>gnl|SRA|SRR5012160.11802706.1:1-22 11802706.
a-------------cagtgag---gtccttagtgaag
>gnl|SRA|SRR5012160.15572341.1:103-137 15572341.
agcctgcagaacatcaaacagtaggtccttagtga--

SRX2342716
>control .
agcctgcag-aactcaaacagtaggtccttagtgaagg
>gnl|SRA|SRR5012158.14895539.1:99-135 14895539.
agcctgcag-aactcaaacagtaggtccttagtgaagg
>gnl|SRA|SRR5012158.14586666.1:116-152 14586666.
agcctgcag-aactcaaacagtaggtccttagtgaagg
>gnl|SRA|SRR5012158.13626365.1:33-69 13626365.
agcctgcag-aactcaaacagtaggtccttagtgaagg
>gnl|SRA|SRR5012158.12180898.1:67-103 12180898.
agcctgcag-aactcaaacagtaggtccttagtgaagg

>gnl|SRA|SRR5012158.11662049.1:103-139 11662049.
agcctgcag-aactcaaacagtaggtccttagtgaagg
>gnl|SRA|SRR5012158.10974111.1:22-58 10974111.
agcctgcag-aactcaaacagtaggtccttagtgaagg
>gnl|SRA|SRR5012158.10605540.1:36-72 10605540.
agcctgcag-aactcaaacagtaggtccttagtgaagg
>gnl|SRA|SRR5012158.10597476.1:93-129 10597476.
agcctgcag-aactcaaacagtaggtccttagtgaagg
>gnl|SRA|SRR5012158.10431720.1:103-139 10431720.
agcctgcag-aactcaaacagtaggtccttagtgaagg
>gnl|SRA|SRR5012158.9791132.1:45-81 9791132.
agcctgcag-aactcaaacagtaggtccttagtgaagg
>gnl|SRA|SRR5012158.9663777.1:21-57 9663777.
agcctgcag-aactcaaacagtaggtccttagtgaagg
>gnl|SRA|SRR5012158.9260576.1:88-124 9260576.
agcctgcag-aactcaaacagtaggtccttagtgaagg
>gnl|SRA|SRR5012158.9025592.1:36-72 9025592.
agcctgcag-aactcaaacagtaggtccttagtgaagg
>gnl|SRA|SRR5012158.8769480.1:36-72 8769480.
agcctgcag-aactcaaacagtaggtccttagtgaagg
>gnl|SRA|SRR5012158.6731540.1:99-135 6731540.
agcctgcag-aactcaaacagtaggtccttagtgaagg
>gnl|SRA|SRR5012158.6029242.1:99-135 6029242.
agcctgcag-aactcaaacagtaggtccttagtgaagg
>gnl|SRA|SRR5012158.5996242.1:90-126 5996242.
agcctgcag-aactcaaacagtaggtccttagtgaagg
>gnl|SRA|SRR5012158.5042829.1:13-49 5042829.
agcctgcag-aactcaaacagtaggtccttagtgaagg
>gnl|SRA|SRR5012158.4870464.1:67-103 4870464.
agcctgcag-aactcaaacagtaggtccttagtgaagg
>gnl|SRA|SRR5012158.4761681.1:36-72 4761681.
agcctgcag-aactcaaacagtaggtccttagtgaagg
>gnl|SRA|SRR5012158.4282042.1:38-74 4282042.
agcctgcag-aactcaaacagtaggtccttagtgaagg
>gnl|SRA|SRR5012158.3021290.1:59-95 3021290.
agcctgcag-aactcaaacagtaggtccttagtgaagg
>gnl|SRA|SRR5012158.2977451.1:3-39 2977451.
agcctgcag-aactcaaacagtaggtccttagtgaagg
>gnl|SRA|SRR5012158.2650116.1:78-114 2650116.
agcctgcag-aactcaaacagtaggtccttagtgaagg
>gnl|SRA|SRR5012158.2621831.1:91-127 2621831.
agcctgcag-aactcaaacagtaggtccttagtgaagg
>gnl|SRA|SRR5012158.2533025.1:76-112 2533025.
agcctgcag-aactcaaacagtaggtccttagtgaagg
>gnl|SRA|SRR5012158.2259403.1:32-68 2259403.
agcctgcag-aactcaaacagtaggtccttagtgaagg
>gnl|SRA|SRR5012158.1767514.1:32-68 1767514.
agcctgcag-aactcaaacagtaggtccttagtgaagg
>gnl|SRA|SRR5012158.1656017.1:23-59 1656017.
agcctgcag-aactcaaacagtaggtccttagtgaagg
>gnl|SRA|SRR5012158.1525356.1:111-147 1525356.

agcctgcag-aactcaaacagtaggtccttagtgaagg
>gnl|SRA|SRR5012158.734176.1:99-135 734176.
agcctgcag-aactcaaacagtaggtccttagtgaagg
>gnl|SRA|SRR5012158.543927.1:93-129 543927.
agcctgcag-aactcaaacagtaggtccttagtgaagg
>gnl|SRA|SRR5012158.529892.1:59-95 529892.
agcctgcag-aactcaaacagtaggtccttagtgaagg
>gnl|SRA|SRR5012158.5883021.1:133-166 5883021.
agcctgcag-aactcaaacagtaggtccttagtga---
>gnl|SRA|SRR5012158.7970182.1:193-224 7970182.
agcctgcag-aactcaaacagtaggtccttagt-----
>gnl|SRA|SRR5012158.13028042.1:103-119 13028042.
agcctgcag-aactcaaa--------------------
>gnl|SRA|SRR5012158.15330419.1:36-69 15330419.
agcctgcag-aactcaaacag---gtccttagtgaagg
>gnl|SRA|SRR5012158.15159552.1:13-46 15159552.
agcctgcag-aactcaaacag---gtccttagtgaagg
>gnl|SRA|SRR5012158.15144500.1:22-55 15144500.
agcctgcag-aactcaaacag---gtccttagtgaagg
>gnl|SRA|SRR5012158.15082613.1:22-55 15082613.
agcctgcag-aactcaaacag---gtccttagtgaagg
>gnl|SRA|SRR5012158.10580727.1:22-55 10580727.
agcctgcag-aactcaaacag---gtccttagtgaagg
>gnl|SRA|SRR5012158.3757887.1:30-63 3757887.
agcctgcag-aactcaaacag---gtccttagtgaagg
>gnl|SRA|SRR5012158.2445832.1:14-47 2445832.
agcctgcag-aactcaaacag---gtccttagtgaagg
>gnl|SRA|SRR5012158.3114520.1:103-133 3114520.
agcctgcag-aactcaaacag---gtccttagtga---
>gnl|SRA|SRR5012158.2753734.1:1-27 2753734.
-------ag-aactcaaacag---gtccttagtgaagg
>gnl|SRA|SRR5012158.11394882.1:25-58 11394882.
agcctgcag-agctcaaacag---gtccttagtgaagg
>gnl|SRA|SRR5012158.7653481.1:25-58 7653481.
agcctgcag-agctcaaacag---gtccttagtgaagg
>gnl|SRA|SRR5012158.8651956.1:12-47 8651956.
agcctgcag-aactc-aacagtaggtccttagtgaagg
>gnl|SRA|SRR5012158.12122513.1:1-22 12122513.
----------------aacagtaggtccttagtgaagg
>gnl|SRA|SRR5012158.14980124.1:1-30 14980124.
-------ag-aactcaaacagtaggtccttagtgaagg
>gnl|SRA|SRR5012158.3869872.1:1-30 3869872.
-------ag-aactcaaacagtaggtccttagtgaagg
>gnl|SRA|SRR5012158.8449437.1:1-29 8449437.
--------g-aactcaaacagtaggtccttagtgaagg
>gnl|SRA|SRR5012158.14780043.1:1-24 14780043.
--------------caaacagtaggtccttagtgaagg
>gnl|SRA|SRR5012158.8747123.1:1-24 8747123.
--------------caaacagtaggtccttagtgaagg
>gnl|SRA|SRR5012158.2569946.1:1-24 2569946.
--------------caaacagtaggtccttagtgaagg

>gnl|SRA|SRR5012158.11794649.1:33-67 11794649.
agcctgcagaactcaaacag---gtccttagtgaagg

SRX1583837
>control .
agcctgcagaactcaaacagtaggtccttagtgaagg
>_R_gnl|SRA|SRR3170527.35036219.1:6-42 35036219.
agcctgcagaactcaaacagtaggtccttagtgaagg
>_R_gnl|SRA|SRR3170527.23836711.2:6-42 23836711.
agcctgcagaactcaaacagtaggtccttagtgaagg
>_R_gnl|SRA|SRR3170527.22981671.2:6-42 22981671.
agcctgcagaactcaaacagtaggtccttagtgaagg
>_R_gnl|SRA|SRR3170527.7067741.2:56-92 7067741.
agcctgcagaactcaaacagtaggtccttagtgaagg
>gnl|SRA|SRR3170527.1453289.2:4-40 1453289.
agcctgcagaactcaaacagtaggtccttagtgaagg
>_R_gnl|SRA|SRR3170527.1453289.1:55-91 1453289.
agcctgcagaactcaaacagtaggtccttagtgaagg
>gnl|SRA|SRR3170527.6670922.2:67-96 6670922.
-------agaactcaaacagtaggtccttagtgaagg
>_R_gnl|SRA|SRR3170527.35450473.1:5-38 35450473.
agcctgcagaactcaaacag---gtccttagtgaagg
>_R_gnl|SRA|SRR3170527.37901239.1:6-24 37901239.
agcctgcagaactcaaaca------------------
>_R_gnl|SRA|SRR3170527.33914612.1:1-28 33914612.
agcctgcagaactcaaacagtaggtcct---------
>_R_gnl|SRA|SRR3170527.29663759.2:1-21 29663759.
agcctgcagaactcaaacagt----------------

SRX1583838
>control .
agcctgcagaactcaaacagtaggtccttagtgaagg
>gnl|SRA|SRR3166985.33906380.2:1-37 33906380.
agcctgcagaactcaaacagtaggtccttagtgaagg
>gnl|SRA|SRR3166985.29731249.1:24-60 29731249.
agcctgcagaactcaaacagtaggtccttagtgaagg
>gnl|SRA|SRR3166985.27805695.2:58-94 27805695.
agcctgcagaactcaaacagtaggtccttagtgaagg
>_R_gnl|SRA|SRR3166985.27805695.1:23-59 27805695.
agcctgcagaactcaaacagtaggtccttagtgaagg
>_R_gnl|SRA|SRR3166985.23718779.1:9-45 23718779.
agcctgcagaactcaaacagtaggtccttagtgaagg
>_R_gnl|SRA|SRR3166985.16846519.2:18-54 16846519.
agcctgcagaactcaaacagtaggtccttagtgaagg
>gnl|SRA|SRR3166985.7433022.2:1-37 7433022.
agcctgcagaactcaaacagtaggtccttagtgaagg

>_R_gnl|SRA|SRR3166985.5970296.2:9-45 5970296.
agcctgcagaactcaaacagtaggtccttagtgaagg
>gnl|SRA|SRR3166985.5970296.1:5-41 5970296.
agcctgcagaactcaaacagtaggtccttagtgaagg
>gnl|SRA|SRR3166985.5694623.2:60-96 5694623.
agcctgcagaactcaaacagtaggtccttagtgaagg
>_R_gnl|SRA|SRR3166985.5694623.1:8-44 5694623.
agcctgcagaactcaaacagtaggtccttagtgaagg
>gnl|SRA|SRR3166985.3439598.2:1-37 3439598.
agcctgcagaactcaaacagtaggtccttagtgaagg
>gnl|SRA|SRR3166985.1830665.2:53-89 1830665.
agcctgcagaactcaaacagtaggtccttagtgaagg
>_R_gnl|SRA|SRR3166985.13511916.1:1-32 13511916.
agcctgcagaactcaaacagtaggtccttagt-----
>gnl|SRA|SRR3166985.37171849.2:72-100 37171849.
agcctgcagaactcaaacagtaggtcctt--------
>_R_gnl|SRA|SRR3166985.36051828.1:1-23 36051828.
agcctgcagaactcaaacagtag--------------
>gnl|SRA|SRR3166985.16846519.1:78-100 16846519.
agcctgcagaactcaaacagtag--------------
>gnl|SRA|SRR3166985.3681086.2:80-99 3681086.
agcctgcagaactcaaacag-----------------
>_R_gnl|SRA|SRR3166985.31284651.2:6-23 31284651.
-gcctgcagaactcaaaca------------------
>gnl|SRA|SRR3166985.28948016.2:8-41 28948016.
agcctgcagaactcaaacag---gtccttagtgaagg
>_R_gnl|SRA|SRR3166985.26190018.2:54-87 26190018.
agcctgcagaactcaaacag---gtccttagtgaagg
>_R_gnl|SRA|SRR3166985.23652897.1:41-74 23652897.
agcctgcagaactcaaacag---gtccttagtgaagg
>_R_gnl|SRA|SRR3166985.18796436.2:34-67 18796436.
agcctgcagaactcaaacag---gtccttagtgaagg
>_R_gnl|SRA|SRR3166985.9644269.1:21-54 9644269.
agcctgcagaactcaaacag---gtccttagtgaagg
>_R_gnl|SRA|SRR3166985.4590977.2:65-98 4590977.
agcctgcagaactcaaacag---gtccttagtgaagg
>_R_gnl|SRA|SRR3166985.671216.1:21-54 671216.
agcctgcagaactcaaacag---gtccttagtgaagg
>_R_gnl|SRA|SRR3166985.17969896.1:1-33 17969896.
agcctgcagaactcaaacag---gtccttagtgaag-
>_R_gnl|SRA|SRR3166985.5715826.1:1-27 5715826.
agcctgcagaactcaaacag---gtcctta-------
>_R_gnl|SRA|SRR3166985.28948016.1:77-100 28948016.
----------actcaaacag---gtccttagtgaagg
>gnl|SRA|SRR3166985.10658800.2:1-32 10658800.
-----gcagaactcaaacagtaggtccttagtgaagg
>_R_gnl|SRA|SRR3166985.33906380.1:70-100 33906380.
------cagaactcaaacagtaggtccttagtgaagg
>gnl|SRA|SRR3166985.7252084.1:1-31 7252084.
------cagaactcaaacagtaggtccttagtgaagg
>_R_gnl|SRA|SRR3166985.1830665.1:77-100 1830665.

-------------caaacagtaggtccttagtgaagg
>gnl|SRA|SRR3166985.21784207.1:2-24 21784207.
--------------aaacagtaggtccttagtgaagg
>_R_gnl|SRA|SRR3166985.31706717.2:5-26 31706717.
---ctacagaactcaaacagtaggt------------
>gnl|SRA|SRR3166985.31706717.1:72-93 31706717.
---ctacagaactcaaacagtaggt------------
>_R_gnl|SRA|SRR3166985.26350522.2:4-25 26350522.
---ctacagaactcaaacagtaggt------------
>_R_gnl|SRA|SRR3166985.37171849.1:63-93 37171849.
------caaaacaaaaaaagtagctccttagtgaagg

SRX1583839
>control .
agcctgcagaactcaaacagtaggtccttagtgaagg
>_R_gnl|SRA|SRR3170528.41269942.1:52-88 41269942.
agcctgcagaactcaaacagtaggtccttagtgaagg
>_R_gnl|SRA|SRR3170528.35538436.1:36-72 35538436.
agcctgcagaactcaaacagtaggtccttagtgaagg
>gnl|SRA|SRR3170528.35194465.1:22-58 35194465.
agcctgcagaactcaaacagtaggtccttagtgaagg
>gnl|SRA|SRR3170528.29559831.1:3-39 29559831.
agcctgcagaactcaaacagtaggtccttagtgaagg
>gnl|SRA|SRR3170528.20232525.1:42-78 20232525.
agcctgcagaactcaaacagtaggtccttagtgaagg
>gnl|SRA|SRR3170528.12399264.2:8-44 12399264.
agcctgcagaactcaaacagtaggtccttagtgaagg
>gnl|SRA|SRR3170528.2205555.2:8-44 2205555.
agcctgcagaactcaaacagtaggtccttagtgaagg
>_R_gnl|SRA|SRR3170528.207774.1:17-53 207774.
agcctgcagaactcaaacagtaggtccttagtgaagg
>_R_gnl|SRA|SRR3170528.23645008.1:1-28 23645008.
agcctgcagaactcaaacagtaggtcct---------
>_R_gnl|SRA|SRR3170528.22405488.2:23-41 22405488.
agcctgcagaactcaaaca------------------
>_R_gnl|SRA|SRR3170528.10820673.2:1-18 10820673.
-------agaactcaaacagtaggt------------
>gnl|SRA|SRR3170528.10820673.1:72-89 10820673.
-------agaactcaaacagtaggt------------
>_R_gnl|SRA|SRR3170528.12399264.1:74-100 12399264.
----------actcaaacagtaggtccttagtgaagg
>gnl|SRA|SRR3170528.9747952.1:1-27 9747952.
----------actcaaacagtaggtccttagtgaagg
>_R_gnl|SRA|SRR3170528.2205555.1:74-100 2205555.
----------actcaaacagtaggtccttagtgaagg
>gnl|SRA|SRR3170528.19414913.1:1-24 19414913.
-------------caaacagtaggtccttagtgaagg
>_R_gnl|SRA|SRR3170528.35886264.2:34-56 35886264.

--------------aaacagtaggtccttagtgaagg
>gnl|SRA|SRR3170528.35886264.1:1-23 35886264.
--------------aaacagtaggtccttagtgaagg
>_R_gnl|SRA|SRR3170528.19414913.2:12-40 19414913.
--------gatctcaaacagtaggtccttagtgaagg

SRX100693
>gnl|SRA|SRR352139.13615748.1:10-35 HWI-EAS216_0001:1:65:5189:4421.
agcctgcagcactcaaacagtaggtc--------------
>gnl|SRA|SRR352139.20523144.1:3-35 HWI-EAS216_0001:1:96:9751:6660.
agcctgcagaactcaaacagtaggtccttagtg-------
>control .
agcctgcagaactcaaacagtaggtccttagtgaagg---
>_R_gnl|SRA|SRR352139.25645288.1:1-35 HWI-EAS216_0001:1:119:12701:18819.
--cctgcagaactcaaacagtaggtccttagtgaagg---
>_R_gnl|SRA|SRR352139.9916411.1:1-35 HWI-EAS216_0001:1:47:11230:17188.
--cctgcagaactcaaacagtaggtccttagtgaagg---
>_R_gnl|SRA|SRR352139.3550001.1:1-35 HWI-EAS216_0001:1:18:15212:19083.
--cctgcagaactcaaacagtaggtccttagtgaagg---
>gnl|SRA|SRR352139.11608015.1:1-32 HWI-EAS216_0001:1:55:10141:6660.
-----gcagaactcaaacagtaggtccttagtgaagg---
>gnl|SRA|SRR352139.1559167.1:1-32 HWI-EAS216_0001:1:9:11526:12670.
-----gcagaactcaaacagtaggtccttagtgaagg---
>_R_gnl|SRA|SRR352139.23392452.1:5-35 HWI-EAS216_0001:1:109:5386:18751.
------cagaactcaaacagtaggtccttagtgaagg---
>gnl|SRA|SRR352139.11077501.1:1-31 HWI-EAS216_0001:1:52:18607:19845.
------cagaactcaaacagtaggtccttagtgaagg---
>_R_gnl|SRA|SRR352139.12570435.1:6-35 HWI-EAS216_0001:1:60:5171:16336.
-------agaactcaaacagtaggtccttagtgaagg---
>_R_gnl|SRA|SRR352139.6833401.1:6-35 HWI-EAS216_0001:1:33:11391:9131.
-------agaactcaaacagtaggtccttagtgaagg---
>_R_gnl|SRA|SRR352139.5393159.1:6-35 HWI-EAS216_0001:1:27:2669:1621.
-------agaactcaaacagtaggtccttagtgaagg---
>_R_gnl|SRA|SRR352139.4981581.1:6-35 HWI-EAS216_0001:1:25:5488:8592.
-------agaactcaaacagtaggtccttagtgaagg---
>_R_gnl|SRA|SRR352139.23470316.1:7-35 HWI-EAS216_0001:1:109:11696:5249.
--------gaactcaaacagtaggtccttagtgaagg---
>_R_gnl|SRA|SRR352139.6895808.1:7-35 HWI-EAS216_0001:1:33:16487:3926.
--------gaactcaaacagtaggtccttagtgaagg---
>_R_gnl|SRA|SRR352139.5821304.1:9-35 HWI-EAS216_0001:1:28:19440:3577.
----------actcaaacagtaggtccttagtgaagg---
>gnl|SRA|SRR352139.21068768.1:1-26 HWI-EAS216_0001:1:98:17187:13121.
-----------ctcaaacagtaggtccttagtgaagg---
>gnl|SRA|SRR352139.15259324.1:1-26 HWI-EAS216_0001:1:72:17182:20380.
-----------ctcaaacagtaggtccttagtgaagg---
>gnl|SRA|SRR352139.11226406.1:1-26 HWI-EAS216_0001:1:53:13343:8977.
-----------ctcaaacagtaggtccttagtgaagg---
>gnl|SRA|SRR352139.2468530.1:1-26 HWI-EAS216_0001:1:13:16412:7907.
-----------ctcaaacagtaggtccttagtgaagg---

>_R_gnl|SRA|SRR352139.9305530.1:11-35 HWI-EAS216_0001:1:44:14932:19618.
------------tcaaacagtaggtccttagtgaagg---
>gnl|SRA|SRR352139.22371273.1:1-24 HWI-EAS216_0001:1:104:13085:1373.
-------------caaacagtaggtccttagtgaagg---
>gnl|SRA|SRR352139.11197497.1:1-24 HWI-EAS216_0001:1:53:10841:14525.
-------------caaacagtaggtccttagtgaagg---
>gnl|SRA|SRR352139.10852822.1:1-24 HWI-EAS216_0001:1:51:17439:7935.
-------------caaacagtaggtccttagtgaagg---
>_R_gnl|SRA|SRR352139.14011867.1:13-35 HWI-EAS216_0001:1:67:2993:20181.
--------------aaacagtaggtccttagtgaagg---
>gnl|SRA|SRR352139.11731805.1:1-18 HWI-EAS216_0001:1:56:2570:6269.
-------------------gtaggtccttagtgaagg---
>gnl|SRA|SRR352139.10175205.1:1-18 HWI-EAS216_0001:1:48:14729:11649.
-------------------gtaggtccttagtgaagg---
>gnl|SRA|SRR352139.20571419.1:6-21 HWI-EAS216_0001:1:96:13596:16955.
---------------------aggtccttagtgaagg---
>gnl|SRA|SRR352139.8005208.1:2-17 HWI-EAS216_0001:1:38:16684:2311.
---------------------aggtccttagtgaagg---
>_R_gnl|SRA|SRR352139.6168027.1:3-35 HWI-EAS216_0001:1:30:11406:6641.
-gcctgcagaactcaaacag---gtccttagtgaagg---
>_R_gnl|SRA|SRR352139.6726069.1:6-35 HWI-EAS216_0001:1:33:2526:19852.
----tgcagaactcaaacag---gtccttagtgaagg---
>_R_gnl|SRA|SRR352139.21990983.1:8-35 HWI-EAS216_0001:1:102:18814:15538.
------cagaactcaaacag---gtccttagtgaagg---
>gnl|SRA|SRR352139.7329962.1:1-23 HWI-EAS216_0001:1:35:15843:4147.
-----------ctcaaacag---gtccttagtgaagg---
>gnl|SRA|SRR352139.4387700.1:1-23 HWI-EAS216_0001:1:22:11337:17184.
-----------ctcaaacag---gtccttagtgaagg---
>gnl|SRA|SRR352139.3002114.1:1-23 HWI-EAS216_0001:1:16:6438:9531.
-----------ctcaaacag---gtccttagtgaagg---
>_R_gnl|SRA|SRR352139.10304348.1:14-35 HWI-EAS216_0001:1:49:7393:1733.
------------tcaaacag---gtccttagtgaagg---
>_R_gnl|SRA|SRR352139.7463771.1:5-24 HWI-EAS216_0001:1:36:8698:1527.
agcctgcagaactcaaacag--------------------
>_R_gnl|SRA|SRR352139.15007443.1:1-18 HWI-EAS216_0001:1:71:14365:13924.
agcctgcagaactcaaac----------------------
>_R_gnl|SRA|SRR352139.10228599.1:1-18 HWI-EAS216_0001:1:48:19217:11996.
agcctgcagaactcaaac----------------------
>_R_gnl|SRA|SRR352139.2333425.1:1-18 HWI-EAS216_0001:1:13:4876:12174.
agcctgcagaactcaaac----------------------
>_R_gnl|SRA|SRR352139.3659427.1:1-16 HWI-EAS216_0001:1:19:6053:11105.
agcctgcagaactcaa------------------------
>gnl|SRA|SRR352139.25668577.1:5-35 HWI-EAS216_0001:1:119:14731:11637.
agcctgcagaactcaaacag---gtccttagtga------

SRX100694
>control .
agcctgcagaactcaaacagtaggtccttagtgaagg

>_R_gnl|SRA|SRR352140.18170628.1:1-35 HWI-EAS216_0001:2:85:8950:2322.
--cctgcagaactcaaacagtaggtccttagtgaagg
>gnl|SRA|SRR352140.24069788.1:1-34 HWI-EAS216_0001:2:111:14808:20168.
---ctgcagaactcaaacagtaggtccttagtgaagg
>gnl|SRA|SRR352140.3970271.1:1-34 HWI-EAS216_0001:2:19:15822:1271.
---ctgcagaactcaaacagtaggtccttagtgaagg
>gnl|SRA|SRR352140.21568537.1:1-32 HWI-EAS216_0001:2:100:11726:19167.
-----gcagaactcaaacagtaggtccttagtgaagg
>gnl|SRA|SRR352140.18208641.1:1-32 HWI-EAS216_0001:2:85:12027:17431.
-----gcagaactcaaacagtaggtccttagtgaagg
>gnl|SRA|SRR352140.17350522.1:1-32 HWI-EAS216_0001:2:81:14535:14754.
-----gcagaactcaaacagtaggtccttagtgaagg
>gnl|SRA|SRR352140.15686733.1:1-32 HWI-EAS216_0001:2:74:4376:4417.
-----gcagaactcaaacagtaggtccttagtgaagg
>gnl|SRA|SRR352140.13082233.1:1-32 HWI-EAS216_0001:2:61:14765:2522.
-----gcagaactcaaacagtaggtccttagtgaagg
>gnl|SRA|SRR352140.11527406.1:1-32 HWI-EAS216_0001:2:54:1401:9894.
-----gcagaactcaaacagtaggtccttagtgaagg
>gnl|SRA|SRR352140.6823381.1:1-32 HWI-EAS216_0001:2:32:11191:13779.
-----gcagaactcaaacagtaggtccttagtgaagg
>gnl|SRA|SRR352140.5107358.1:1-32 HWI-EAS216_0001:2:24:17225:7837.
-----gcagaactcaaacagtaggtccttagtgaagg
>gnl|SRA|SRR352140.2918166.1:1-32 HWI-EAS216_0001:2:15:2422:11331.
-----gcagaactcaaacagtaggtccttagtgaagg
>gnl|SRA|SRR352140.2891940.1:1-32 HWI-EAS216_0001:2:14:18465:17467.
-----gcagaactcaaacagtaggtccttagtgaagg
>gnl|SRA|SRR352140.24673942.1:1-31 HWI-EAS216_0001:2:114:10224:8569.
------cagaactcaaacagtaggtccttagtgaagg
>_R_gnl|SRA|SRR352140.21757900.1:5-35 HWI-EAS216_0001:2:101:8898:10012.
------cagaactcaaacagtaggtccttagtgaagg
>gnl|SRA|SRR352140.21633920.1:1-31 HWI-EAS216_0001:2:100:17022:3064.
------cagaactcaaacagtaggtccttagtgaagg
>gnl|SRA|SRR352140.20869053.1:1-31 HWI-EAS216_0001:2:97:9801:1649.
------cagaactcaaacagtaggtccttagtgaagg
>gnl|SRA|SRR352140.20038378.1:1-31 HWI-EAS216_0001:2:93:15273:11101.
------cagaactcaaacagtaggtccttagtgaagg
>gnl|SRA|SRR352140.18287282.1:1-31 HWI-EAS216_0001:2:85:18552:11452.
------cagaactcaaacagtaggtccttagtgaagg
>gnl|SRA|SRR352140.16805329.1:1-31 HWI-EAS216_0001:2:79:6165:13008.
------cagaactcaaacagtaggtccttagtgaagg
>gnl|SRA|SRR352140.15408137.1:1-31 HWI-EAS216_0001:2:72:17507:9412.
------cagaactcaaacagtaggtccttagtgaagg
>gnl|SRA|SRR352140.14681356.1:1-31 HWI-EAS216_0001:2:69:10561:16415.
------cagaactcaaacagtaggtccttagtgaagg
>gnl|SRA|SRR352140.12298910.1:1-31 HWI-EAS216_0001:2:57:15255:1487.
------cagaactcaaacagtaggtccttagtgaagg
>gnl|SRA|SRR352140.8680607.1:1-31 HWI-EAS216_0001:2:40:17290:6182.
------cagaactcaaacagtaggtccttagtgaagg
>gnl|SRA|SRR352140.8014296.1:1-31 HWI-EAS216_0001:2:37:17318:14610.
------cagaactcaaacagtaggtccttagtgaagg
>gnl|SRA|SRR352140.7976742.1:1-31 HWI-EAS216_0001:2:37:14234:17478.

------cagaactcaaacagtaggtccttagtgaagg
>gnl|SRA|SRR352140.3859971.1:1-31 HWI-EAS216_0001:2:19:6865:19699.
------cagaactcaaacagtaggtccttagtgaagg
>gnl|SRA|SRR352140.2865011.1:1-31 HWI-EAS216_0001:2:14:16235:17597.
------cagaactcaaacagtaggtccttagtgaagg
>gnl|SRA|SRR352140.2016370.1:1-31 HWI-EAS216_0001:2:10:18674:4361.
------cagaactcaaacagtaggtccttagtgaagg
>gnl|SRA|SRR352140.1560422.1:1-31 HWI-EAS216_0001:2:8:16246:19408.
------cagaactcaaacagtaggtccttagtgaagg
>gnl|SRA|SRR352140.21937432.1:1-30 HWI-EAS216_0001:2:102:5347:6960.
-------agaactcaaacagtaggtccttagtgaagg
>_R_gnl|SRA|SRR352140.13956248.1:6-35 HWI-EAS216_0001:2:66:2890:7967.
-------agaactcaaacagtaggtccttagtgaagg
>_R_gnl|SRA|SRR352140.6580561.1:6-35 HWI-EAS216_0001:2:31:9673:15894.
-------agaactcaaacagtaggtccttagtgaagg
>_R_gnl|SRA|SRR352140.25594638.1:7-35 HWI-EAS216_0001:2:118:15058:18659.
--------gaactcaaacagtaggtccttagtgaagg
>_R_gnl|SRA|SRR352140.25429943.1:7-35 HWI-EAS216_0001:2:117:19130:14226.
--------gaactcaaacagtaggtccttagtgaagg
>_R_gnl|SRA|SRR352140.23415767.1:7-35 HWI-EAS216_0001:2:108:15973:15196.
--------gaactcaaacagtaggtccttagtgaagg
>_R_gnl|SRA|SRR352140.21878286.1:7-35 HWI-EAS216_0001:2:101:18773:1202.
--------gaactcaaacagtaggtccttagtgaagg
>_R_gnl|SRA|SRR352140.20642015.1:7-35 HWI-EAS216_0001:2:96:9631:2125.
--------gaactcaaacagtaggtccttagtgaagg
>_R_gnl|SRA|SRR352140.20071691.1:7-35 HWI-EAS216_0001:2:93:18044:3467.
--------gaactcaaacagtaggtccttagtgaagg
>_R_gnl|SRA|SRR352140.19552476.1:7-35 HWI-EAS216_0001:2:91:12190:18037.
--------gaactcaaacagtaggtccttagtgaagg
>_R_gnl|SRA|SRR352140.19552183.1:7-35 HWI-EAS216_0001:2:91:12167:18033.
--------gaactcaaacagtaggtccttagtgaagg
>gnl|SRA|SRR352140.19304269.1:1-29 HWI-EAS216_0001:2:90:10259:19306.
--------gaactcaaacagtaggtccttagtgaagg
>_R_gnl|SRA|SRR352140.17828526.1:7-35 HWI-EAS216_0001:2:83:17364:10568.
--------gaactcaaacagtaggtccttagtgaagg
>gnl|SRA|SRR352140.16907933.1:1-29 HWI-EAS216_0001:2:79:14569:1291.
--------gaactcaaacagtaggtccttagtgaagg
>_R_gnl|SRA|SRR352140.16801507.1:7-35 HWI-EAS216_0001:2:79:5852:4627.
--------gaactcaaacagtaggtccttagtgaagg
>_R_gnl|SRA|SRR352140.13690123.1:7-35 HWI-EAS216_0001:2:64:16090:8518.
--------gaactcaaacagtaggtccttagtgaagg
>_R_gnl|SRA|SRR352140.11544738.1:7-35 HWI-EAS216_0001:2:54:3165:8358.
--------gaactcaaacagtaggtccttagtgaagg
>_R_gnl|SRA|SRR352140.10239298.1:7-35 HWI-EAS216_0001:2:47:18895:7454.
--------gaactcaaacagtaggtccttagtgaagg
>_R_gnl|SRA|SRR352140.9957817.1:7-35 HWI-EAS216_0001:2:46:13600:8845.
--------gaactcaaacagtaggtccttagtgaagg
>_R_gnl|SRA|SRR352140.7573147.1:7-35 HWI-EAS216_0001:2:35:17734:8063.
--------gaactcaaacagtaggtccttagtgaagg
>_R_gnl|SRA|SRR352140.6950463.1:7-35 HWI-EAS216_0001:2:33:3333:17312.
--------gaactcaaacagtaggtccttagtgaagg

>_R_gnl|SRA|SRR352140.5628863.1:7-35 HWI-EAS216_0001:2:27:5127:12123.
--------gaactcaaacagtaggtccttagtgaagg
>_R_gnl|SRA|SRR352140.26013545.1:9-35 HWI-EAS216_0001:2:120:15439:13850.
----------actcaaacagtaggtccttagtgaagg
>_R_gnl|SRA|SRR352140.25914628.1:9-35 HWI-EAS216_0001:2:120:6745:3939.
----------actcaaacagtaggtccttagtgaagg
>_R_gnl|SRA|SRR352140.12151598.1:9-35 HWI-EAS216_0001:2:57:1816:9625.
----------actcaaacagtaggtccttagtgaagg
>_R_gnl|SRA|SRR352140.1515970.1:9-35 HWI-EAS216_0001:2:8:12459:3135.
----------actcaaacagtaggtccttagtgaagg
>_R_gnl|SRA|SRR352140.6969399.1:9-35 HWI-EAS216_0001:2:33:4910:14939.
----------acgcaaacagtaggtccttagtgaagg
>gnl|SRA|SRR352140.26031148.1:1-26 HWI-EAS216_0001:2:120:16979:16039.
-----------ctcaaacagtaggtccttagtgaagg
>_R_gnl|SRA|SRR352140.25939901.1:10-35 HWI-EAS216_0001:2:120:8995:20412.
-----------ctcaaacagtaggtccttagtgaagg
>gnl|SRA|SRR352140.19919982.1:1-26 HWI-EAS216_0001:2:93:5735:15866.
-----------ctcaaacagtaggtccttagtgaagg
>_R_gnl|SRA|SRR352140.18324798.1:10-35 HWI-EAS216_0001:2:86:3356:8794.
-----------ctcaaacagtaggtccttagtgaagg
>gnl|SRA|SRR352140.16430476.1:1-26 HWI-EAS216_0001:2:77:11552:19356.
-----------ctcaaacagtaggtccttagtgaagg
>_R_gnl|SRA|SRR352140.14735131.1:10-35 HWI-EAS216_0001:2:69:15158:4928.
-----------ctcaaacagtaggtccttagtgaagg
>gnl|SRA|SRR352140.14513107.1:1-26 HWI-EAS216_0001:2:68:14269:15067.
-----------ctcaaacagtaggtccttagtgaagg
>gnl|SRA|SRR352140.13801412.1:1-26 HWI-EAS216_0001:2:65:7613:9314.
-----------ctcaaacagtaggtccttagtgaagg
>gnl|SRA|SRR352140.13768718.1:1-26 HWI-EAS216_0001:2:65:4809:15613.
-----------ctcaaacagtaggtccttagtgaagg
>gnl|SRA|SRR352140.13418108.1:1-26 HWI-EAS216_0001:2:63:9146:17538.
-----------ctcaaacagtaggtccttagtgaagg
>gnl|SRA|SRR352140.12889294.1:6-31 HWI-EAS216_0001:2:60:15994:2128.
-----------ctcaaacagtaggtccttagtgaagg
>gnl|SRA|SRR352140.11898537.1:1-26 HWI-EAS216_0001:2:55:16087:10479.
-----------ctcaaacagtaggtccttagtgaagg
>gnl|SRA|SRR352140.11417900.1:1-26 HWI-EAS216_0001:2:53:10285:5636.
-----------ctcaaacagtaggtccttagtgaagg
>_R_gnl|SRA|SRR352140.10577487.1:10-35 HWI-EAS216_0001:2:49:10980:15768.
-----------ctcaaacagtaggtccttagtgaagg
>gnl|SRA|SRR352140.10480309.1:1-26 HWI-EAS216_0001:2:49:2744:20088.
-----------ctcaaacagtaggtccttagtgaagg
>gnl|SRA|SRR352140.10426950.1:1-26 HWI-EAS216_0001:2:48:16469:19306.
-----------ctcaaacagtaggtccttagtgaagg
>gnl|SRA|SRR352140.10240342.1:6-31 HWI-EAS216_0001:2:47:18983:19224.
-----------ctcaaacagtaggtccttagtgaagg
>gnl|SRA|SRR352140.10118982.1:1-26 HWI-EAS216_0001:2:47:8853:8176.
-----------ctcaaacagtaggtccttagtgaagg
>gnl|SRA|SRR352140.9189276.1:6-31 HWI-EAS216_0001:2:43:4545:2821.
-----------ctcaaacagtaggtccttagtgaagg
>gnl|SRA|SRR352140.6579650.1:1-26 HWI-EAS216_0001:2:31:9597:2827.

-----------ctcaaacagtaggtccttagtgaagg
>_R_gnl|SRA|SRR352140.2972447.1:10-35 HWI-EAS216_0001:2:15:6949:19222.
-----------ctcaaacagtaggtccttagtgaagg
>gnl|SRA|SRR352140.743646.1:1-26 HWI-EAS216_0001:2:4:15596:12366.
-----------ctcaaacagtaggtccttagtgaagg
>gnl|SRA|SRR352140.649049.1:1-26 HWI-EAS216_0001:2:4:6916:12767.
-----------ctcaaacagtaggtccttagtgaagg
>gnl|SRA|SRR352140.277755.1:1-26 HWI-EAS216_0001:2:2:8498:16263.
-----------ctcaaacagtaggtccttagtgaagg
>_R_gnl|SRA|SRR352140.9130032.1:11-35 HWI-EAS216_0001:2:42:17852:8629.
------------tcaaacagtaggtccttagtgaagg
>gnl|SRA|SRR352140.25473118.1:1-24 HWI-EAS216_0001:2:118:4742:12224.
-------------caaacagtaggtccttagtgaagg
>gnl|SRA|SRR352140.24966047.1:1-24 HWI-EAS216_0001:2:115:16325:10122.
-------------caaacagtaggtccttagtgaagg
>gnl|SRA|SRR352140.24936635.1:1-24 HWI-EAS216_0001:2:115:13880:12624.
-------------caaacagtaggtccttagtgaagg
>gnl|SRA|SRR352140.23654970.1:1-24 HWI-EAS216_0001:2:109:17267:3797.
-------------caaacagtaggtccttagtgaagg
>gnl|SRA|SRR352140.23336063.1:1-24 HWI-EAS216_0001:2:108:9563:12370.
-------------caaacagtaggtccttagtgaagg
>gnl|SRA|SRR352140.21697772.1:1-24 HWI-EAS216_0001:2:101:4076:15773.
-------------caaacagtaggtccttagtgaagg
>gnl|SRA|SRR352140.18593125.1:1-24 HWI-EAS216_0001:2:87:7011:3293.
-------------caaacagtaggtccttagtgaagg
>gnl|SRA|SRR352140.18314071.1:1-24 HWI-EAS216_0001:2:86:2489:12472.
-------------caaacagtaggtccttagtgaagg
>gnl|SRA|SRR352140.15760136.1:1-24 HWI-EAS216_0001:2:74:10459:11185.
-------------caaacagtaggtccttagtgaagg
>gnl|SRA|SRR352140.13525740.1:1-24 HWI-EAS216_0001:2:64:1242:10608.
-------------caaacagtaggtccttagtgaagg
>gnl|SRA|SRR352140.8179498.1:1-24 HWI-EAS216_0001:2:38:12597:13585.
-------------caaacagtaggtccttagtgaagg
>gnl|SRA|SRR352140.6883415.1:1-24 HWI-EAS216_0001:2:32:16071:14007.
-------------caaacagtaggtccttagtgaagg
>gnl|SRA|SRR352140.1157737.1:1-24 HWI-EAS216_0001:2:6:16999:16196.
-------------caaacagtaggtccttagtgaagg
>gnl|SRA|SRR352140.23859889.1:1-23 HWI-EAS216_0001:2:110:15800:20515.
--------------aaacagtaggtccttagtgaagg
>gnl|SRA|SRR352140.23800707.1:1-23 HWI-EAS216_0001:2:110:11019:5171.
--------------aaacagtaggtccttagtgaagg
>_R_gnl|SRA|SRR352140.23130262.1:13-35 HWI-EAS216_0001:2:107:10993:17711.
--------------aaacagtaggtccttagtgaagg
>_R_gnl|SRA|SRR352140.21047980.1:13-35 HWI-EAS216_0001:2:98:6117:2444.
--------------aaacagtaggtccttagtgaagg
>_R_gnl|SRA|SRR352140.20201434.1:13-35 HWI-EAS216_0001:2:94:10289:18823.
--------------aaacagtaggtccttagtgaagg
>gnl|SRA|SRR352140.18867095.1:1-23 HWI-EAS216_0001:2:88:11074:8869.
--------------aaacagtaggtccttagtgaagg
>_R_gnl|SRA|SRR352140.14932159.1:13-35 HWI-EAS216_0001:2:70:13715:19182.
--------------aaacagtaggtccttagtgaagg

>gnl|SRA|SRR352140.6111168.1:1-23 HWI-EAS216_0001:2:29:7888:12668.
--------------aaacagtaggtccttagtgaagg
>_R_gnl|SRA|SRR352140.25179949.1:10-31 HWI-EAS216_0001:2:116:16079:6895.
---------------aacagtaggtccttagtgaagg
>_R_gnl|SRA|SRR352140.20274086.1:10-31 HWI-EAS216_0001:2:94:16179:5911.
---------------aacagtaggtccttagtgaagg
>_R_gnl|SRA|SRR352140.5645261.1:10-31 HWI-EAS216_0001:2:27:6454:19225.
---------------aacagtaggtccttagtgaagg
>gnl|SRA|SRR352140.22618904.1:1-21 HWI-EAS216_0001:2:105:5926:8580.
----------------acagtaggtccttagtgaagg
>gnl|SRA|SRR352140.10641913.1:1-21 HWI-EAS216_0001:2:49:16379:17053.
----------------acagtaggtccttagtgaagg
>_R_gnl|SRA|SRR352140.11981698.1:16-35 HWI-EAS216_0001:2:56:5177:20388.
-----------------cagtaggtccttagtgaagg
>gnl|SRA|SRR352140.17731835.1:1-19 HWI-EAS216_0001:2:83:9433:20483.
------------------agtaggtccttagtgaagg
>gnl|SRA|SRR352140.5180565.1:1-19 HWI-EAS216_0001:2:25:5036:20508.
------------------agtaggtccttagtgaagg
>gnl|SRA|SRR352140.3771664.1:1-19 HWI-EAS216_0001:2:18:17817:13662.
------------------agtaggtccttagtgaagg
>_R_gnl|SRA|SRR352140.14354160.1:18-35 HWI-EAS216_0001:2:67:19026:10815.
-------------------gtaggtccttagtgaagg
>gnl|SRA|SRR352140.14695318.1:1-17 HWI-EAS216_0001:2:69:11747:8424.
--------------------taggtccttagtgaagg
>_R_gnl|SRA|SRR352140.25620115.1:20-35 HWI-EAS216_0001:2:118:17221:15936.
---------------------aggtccttagtgaagg
>_R_gnl|SRA|SRR352140.25360785.1:20-35 HWI-EAS216_0001:2:117:13235:14984.
---------------------aggtccttagtgaagg
>gnl|SRA|SRR352140.24282655.1:6-21 HWI-EAS216_0001:2:112:14131:19878.
---------------------aggtccttagtgaagg
>gnl|SRA|SRR352140.19842406.1:6-21 HWI-EAS216_0001:2:92:17633:5624.
---------------------aggtccttagtgaagg
>gnl|SRA|SRR352140.15534524.1:2-17 HWI-EAS216_0001:2:73:9861:14413.
---------------------aggtccttagtgaagg
>gnl|SRA|SRR352140.15125030.1:2-17 HWI-EAS216_0001:2:71:11908:5796.
---------------------aggtccttagtgaagg
>gnl|SRA|SRR352140.12845573.1:2-17 HWI-EAS216_0001:2:60:11891:14839.
---------------------aggtccttagtgaagg
>gnl|SRA|SRR352140.11097061.1:6-21 HWI-EAS216_0001:2:51:18682:11604.
---------------------aggtccttagtgaagg
>gnl|SRA|SRR352140.10410984.1:6-21 HWI-EAS216_0001:2:48:15123:13233.
---------------------aggtccttagtgaagg
>gnl|SRA|SRR352140.7225942.1:6-21 HWI-EAS216_0001:2:34:7629:2440.
---------------------aggtccttagtgaagg
>gnl|SRA|SRR352140.6552036.1:2-17 HWI-EAS216_0001:2:31:7352:14766.
---------------------aggtccttagtgaagg
>_R_gnl|SRA|SRR352140.5049406.1:20-35 HWI-EAS216_0001:2:24:12531:19254.
---------------------aggtccttagtgaagg
>_R_gnl|SRA|SRR352140.4248486.1:20-35 HWI-EAS216_0001:2:21:2017:17958.
---------------------aggtccttagtgaagg
>gnl|SRA|SRR352140.3937136.1:2-17 HWI-EAS216_0001:2:19:13111:12038.

---------------------aggtccttagtgaagg
>_R_gnl|SRA|SRR352140.2515309.1:20-35 HWI-EAS216_0001:2:13:5669:21058.
---------------------aggtccttagtgaagg
>gnl|SRA|SRR352140.807344.1:6-21 HWI-EAS216_0001:2:5:3471:17923.
---------------------aggtccttagtgaagg
>_R_gnl|SRA|SRR352140.4147579.1:3-35 HWI-EAS216_0001:2:20:12034:15430.
-gcctgcagaactcaaacag---gtccttagtgaagg
>_R_gnl|SRA|SRR352140.3142950.1:3-35 HWI-EAS216_0001:2:16:2741:6835.
-gcctgcagaactcaaacag---gtccttagtgaagg
>gnl|SRA|SRR352140.22782476.1:1-30 HWI-EAS216_0001:2:105:19318:11874.
----tgcagaactcaaacag---gtccttagtgaagg
>gnl|SRA|SRR352140.19601946.1:1-30 HWI-EAS216_0001:2:91:16260:7902.
----tgcagaactcaaacag---gtccttagtgaagg
>gnl|SRA|SRR352140.19347382.1:1-30 HWI-EAS216_0001:2:90:13748:19298.
----tgcagaactcaaacag---gtccttagtgaagg
>gnl|SRA|SRR352140.24936292.1:1-29 HWI-EAS216_0001:2:115:13853:2699.
-----gcagaactcaaacag---gtccttagtgaagg
>gnl|SRA|SRR352140.20960601.1:1-29 HWI-EAS216_0001:2:97:17221:9408.
-----gcagaactcaaacag---gtccttagtgaagg
>_R_gnl|SRA|SRR352140.20292093.1:7-35 HWI-EAS216_0001:2:94:17682:17234.
-----gcagaactcaaacag---gtccttagtgaagg
>gnl|SRA|SRR352140.19741121.1:1-29 HWI-EAS216_0001:2:92:9398:4888.
-----gcagaactcaaacag---gtccttagtgaagg
>gnl|SRA|SRR352140.16393827.1:1-29 HWI-EAS216_0001:2:77:8545:15212.
-----gcagaactcaaacag---gtccttagtgaagg
>gnl|SRA|SRR352140.7957810.1:1-29 HWI-EAS216_0001:2:37:12699:16635.
-----gcagaactcaaacag---gtccttagtgaagg
>_R_gnl|SRA|SRR352140.7317632.1:7-35 HWI-EAS216_0001:2:34:15081:7645.
-----gcagaactcaaacag---gtccttagtgaagg
>gnl|SRA|SRR352140.6754923.1:1-29 HWI-EAS216_0001:2:32:5635:15175.
-----gcagaactcaaacag---gtccttagtgaagg
>_R_gnl|SRA|SRR352140.3379897.1:7-35 HWI-EAS216_0001:2:17:3999:12350.
-----gcagaactcaaacag---gtccttagtgaagg
>_R_gnl|SRA|SRR352140.9473034.1:7-35 HWI-EAS216_0001:2:44:9751:3925.
-----gcagagctcaaacag---gtccttagtgaagg
>_R_gnl|SRA|SRR352140.4969751.1:10-35 HWI-EAS216_0001:2:24:6127:6267.
--------gaactcaaacag---gtccttagtgaagg
>gnl|SRA|SRR352140.19901524.1:1-23 HWI-EAS216_0001:2:93:4262:2754.
-----------ctcaaacag---gtccttagtgaagg
>gnl|SRA|SRR352140.11488556.1:1-23 HWI-EAS216_0001:2:53:16467:9212.
-----------ctcaaacag---gtccttagtgaagg
>gnl|SRA|SRR352140.5991401.1:1-23 HWI-EAS216_0001:2:28:16301:16244.
-----------ctcaaacag---gtccttagtgaagg
>_R_gnl|SRA|SRR352140.25322633.1:1-35 HWI-EAS216_0001:2:117:10085:15653.
-gcctgcagaactcaaacagtaggtccttagtgaag-
>_R_gnl|SRA|SRR352140.25073749.1:1-35 HWI-EAS216_0001:2:116:7279:19517.
-gcctgcagaactcaaacagtaggtccttagtgaag-
>gnl|SRA|SRR352140.24398235.1:1-35 HWI-EAS216_0001:2:113:5625:7277.
-gcctgcagaactcaaacagtaggtccttagtgaag-
>_R_gnl|SRA|SRR352140.23276968.1:1-35 HWI-EAS216_0001:2:108:4830:15212.
-gcctgcagaactcaaacagtaggtccttagtgaag-

>_R_gnl|SRA|SRR352140.21338529.1:1-35 HWI-EAS216_0001:2:99:11322:6163.
-gcctgcagaactcaaacagtaggtccttagtgaag-
>gnl|SRA|SRR352140.18830192.1:1-35 HWI-EAS216_0001:2:88:8099:10040.
-gcctgcagaactcaaacagtaggtccttagtgaag-
>gnl|SRA|SRR352140.18646951.1:1-35 HWI-EAS216_0001:2:87:11366:15706.
-gcctgcagaactcaaacagtaggtccttagtgaag-
>gnl|SRA|SRR352140.16454629.1:1-35 HWI-EAS216_0001:2:77:13546:18745.
-gcctgcagaactcaaacagtaggtccttagtgaag-
>_R_gnl|SRA|SRR352140.15010026.1:1-35 HWI-EAS216_0001:2:71:2256:2737.
-gcctgcagaactcaaacagtaggtccttagtgaag-
>gnl|SRA|SRR352140.14005174.1:1-35 HWI-EAS216_0001:2:66:7083:2462.
-gcctgcagaactcaaacagtaggtccttagtgaag-
>gnl|SRA|SRR352140.13426905.1:1-35 HWI-EAS216_0001:2:63:10030:1401.
-gcctgcagaactcaaacagtaggtccttagtgaag-
>gnl|SRA|SRR352140.9958604.1:1-35 HWI-EAS216_0001:2:46:13665:14131.
-gcctgcagaactcaaacagtaggtccttagtgaag-
>_R_gnl|SRA|SRR352140.9738497.1:1-35 HWI-EAS216_0001:2:45:13589:13266.
-gcctgcagaactcaaacagtaggtccttagtgaag-
>_R_gnl|SRA|SRR352140.6774174.1:1-35 HWI-EAS216_0001:2:32:7197:2176.
-gcctgcagaactcaaacagtaggtccttagtgaag-
>gnl|SRA|SRR352140.6770639.1:1-35 HWI-EAS216_0001:2:32:6912:12199.
-gcctgcagaactcaaacagtaggtccttagtgaag-
>_R_gnl|SRA|SRR352140.5097761.1:1-35 HWI-EAS216_0001:2:24:16445:11919.
-gcctgcagaactcaaacagtaggtccttagtgaag-
>gnl|SRA|SRR352140.4272226.1:1-35 HWI-EAS216_0001:2:21:4052:19702.
-gcctgcagaactcaaacagtaggtccttagtgaag-
>_R_gnl|SRA|SRR352140.3006951.1:1-35 HWI-EAS216_0001:2:15:9771:20395.
-gcctgcagaactcaaacagtaggtccttagtgaag-
>gnl|SRA|SRR352140.2889134.1:1-35 HWI-EAS216_0001:2:14:18231:17960.
-gcctgcagaactcaaacagtaggtccttagtgaag-
>_R_gnl|SRA|SRR352140.2851014.1:1-35 HWI-EAS216_0001:2:14:15079:21028.
-gcctgcagaactcaaacagtaggtccttagtgaag-
>gnl|SRA|SRR352140.2044765.1:1-35 HWI-EAS216_0001:2:11:2917:8874.
-gcctgcagaactcaaacagtaggtccttagtgaag-
>gnl|SRA|SRR352140.1924393.1:1-35 HWI-EAS216_0001:2:10:10922:14985.
-gcctgcagaactcaaacagtaggtccttagtgaag-
>gnl|SRA|SRR352140.15084605.1:1-35 HWI-EAS216_0001:2:71:8535:7299.
agcctgcagaactcaaacagtaggtccttagtgaa--
>gnl|SRA|SRR352140.6702087.1:1-35 HWI-EAS216_0001:2:31:19569:2458.
agcctgcagaactcaaacagtaggtccttagtgaa--
>gnl|SRA|SRR352140.947729.1:1-35 HWI-EAS216_0001:2:5:16013:14316.
agcctgcagaactcaaacagtaggtccttagtgaa--
>_R_gnl|SRA|SRR352140.16329997.1:1-34 HWI-EAS216_0001:2:77:3273:20280.
agcctgcagaactcaaacagtaggtccttagtga---
>_R_gnl|SRA|SRR352140.20927893.1:1-31 HWI-EAS216_0001:2:97:14527:6656.
agcctgcagaactcaaacagtaggtccttag------
>_R_gnl|SRA|SRR352140.2338843.1:1-31 HWI-EAS216_0001:2:12:9232:3233.
agcctgcagaactcaaacagtaggtccttag------
>_R_gnl|SRA|SRR352140.24632970.1:1-30 HWI-EAS216_0001:2:114:6869:16964.
agcctgcagaactcaaacagtaggtcctta-------
>_R_gnl|SRA|SRR352140.4843802.1:1-30 HWI-EAS216_0001:2:23:14045:11474.

agcctgcagaactcaaacagtaggtcctta-------
>gnl|SRA|SRR352140.18929687.1:7-35 HWI-EAS216_0001:2:88:16184:15012.
agcctgcagaactcaaacagtaggtcctt--------
>gnl|SRA|SRR352140.13449640.1:7-35 HWI-EAS216_0001:2:63:12279:16865.
agcctgcagaactcaaacagtaggtcctt--------
>gnl|SRA|SRR352140.5702698.1:7-35 HWI-EAS216_0001:2:27:11080:13587.
agcctgcagaactcaaacagtaggtcctt--------
>gnl|SRA|SRR352140.17026195.1:8-35 HWI-EAS216_0001:2:80:6126:9699.
agcctgcagaactcaaacagtaggtcct---------
>gnl|SRA|SRR352140.16867058.1:8-35 HWI-EAS216_0001:2:79:11213:2208.
agcctgcagaactcaaacagtaggtcct---------
>gnl|SRA|SRR352140.8742388.1:8-35 HWI-EAS216_0001:2:41:4174:8709.
agcctgcagaactcaaacagtaggtcct---------
>gnl|SRA|SRR352140.6358338.1:8-35 HWI-EAS216_0001:2:30:9781:5090.
agcctgcagaactcaaacagtaggtcct---------
>gnl|SRA|SRR352140.4954068.1:9-35 HWI-EAS216_0001:2:24:4854:11377.
agcctgcagaactcaaacagtaggtcc----------
>gnl|SRA|SRR352140.19078723.1:10-35 HWI-EAS216_0001:2:89:10102:7286.
agcctgcagaactcaaacagtaggtc-----------
>gnl|SRA|SRR352140.18547602.1:10-35 HWI-EAS216_0001:2:87:3331:16532.
agcctgcagaactcaaacagtaggtc-----------
>gnl|SRA|SRR352140.16824025.1:10-35 HWI-EAS216_0001:2:79:7684:6760.
agcctgcagaactcaaacagtaggtc-----------
>gnl|SRA|SRR352140.12491550.1:10-35 HWI-EAS216_0001:2:58:14750:17158.
agcctgcagaactcaaacagtaggtc-----------
>gnl|SRA|SRR352140.12306608.1:10-35 HWI-EAS216_0001:2:57:15956:16821.
agcctgcagaactcaaacagtaggtc-----------
>gnl|SRA|SRR352140.3980852.1:10-35 HWI-EAS216_0001:2:19:16693:9513.
agcctgcagaactcaaacagtaggtc-----------
>gnl|SRA|SRR352140.1247244.1:10-35 HWI-EAS216_0001:2:7:6951:15075.
agcctgcagaactcaaacagtaggtc-----------
>gnl|SRA|SRR352140.20782994.1:11-35 HWI-EAS216_0001:2:97:2907:4645.
agcctgcagaactcaaacagtaggt------------
>gnl|SRA|SRR352140.19609327.1:11-35 HWI-EAS216_0001:2:91:16881:5126.
agcctgcagaactcaaacagtaggt------------
>gnl|SRA|SRR352140.14531512.1:11-35 HWI-EAS216_0001:2:68:15873:5696.
agcctgcagaactcaaacagtaggt------------
>gnl|SRA|SRR352140.14171923.1:11-35 HWI-EAS216_0001:2:67:3255:15460.
agcctgcagaactcaaacagtaggt------------
>gnl|SRA|SRR352140.11399271.1:11-35 HWI-EAS216_0001:2:53:8652:13917.
agcctgcagaactcaaacagtaggt------------
>gnl|SRA|SRR352140.10909508.1:11-35 HWI-EAS216_0001:2:51:2642:4470.
agcctgcagaactcaaacagtaggt------------
>gnl|SRA|SRR352140.9079307.1:11-35 HWI-EAS216_0001:2:42:13649:15169.
agcctgcagaactcaaacagtaggt------------
>gnl|SRA|SRR352140.8682992.1:11-35 HWI-EAS216_0001:2:40:17495:16431.
agcctgcagaactcaaacagtaggt------------
>gnl|SRA|SRR352140.5008426.1:11-35 HWI-EAS216_0001:2:24:9245:12685.
agcctgcagaactcaaacagtaggt------------
>gnl|SRA|SRR352140.4398694.1:12-35 HWI-EAS216_0001:2:21:14290:15219.
agcctgcagaactcaaacagtagg-------------

>gnl|SRA|SRR352140.3346525.1:12-35 HWI-EAS216_0001:2:16:19426:13049.
agcctgcagaactcaaacagtagg-------------
>gnl|SRA|SRR352140.20710598.1:13-35 HWI-EAS216_0001:2:96:15185:17714.
agcctgcagaactcaaacagtag--------------
>gnl|SRA|SRR352140.17036064.1:13-35 HWI-EAS216_0001:2:80:6931:1694.
agcctgcagaactcaaacagtag--------------
>gnl|SRA|SRR352140.6113188.1:15-35 HWI-EAS216_0001:2:29:8051:11920.
agcctgcagaactcaaacagt----------------
>gnl|SRA|SRR352140.893578.1:15-35 HWI-EAS216_0001:2:5:11211:13239.
agcctgcagaactcaaacagt----------------
>gnl|SRA|SRR352140.25253614.1:13-32 HWI-EAS216_0001:2:117:4237:13067.
agcctgcagaactcaaacag-----------------
>_R_gnl|SRA|SRR352140.21898044.1:2-21 HWI-EAS216_0001:2:102:2172:15183.
agcctgcagaactcaaacag-----------------
>_R_gnl|SRA|SRR352140.21189308.1:2-21 HWI-EAS216_0001:2:98:17501:16719.
agcctgcagaactcaaacag-----------------
>_R_gnl|SRA|SRR352140.19934399.1:2-21 HWI-EAS216_0001:2:93:6905:16831.
agcctgcagaactcaaacag-----------------
>gnl|SRA|SRR352140.12854581.1:16-35 HWI-EAS216_0001:2:60:12739:20497.
agcctgcagaactcaaacag-----------------
>gnl|SRA|SRR352140.10456032.1:11-30 HWI-EAS216_0001:2:48:18939:7803.
agcctgcagaactcaaacag-----------------
>gnl|SRA|SRR352140.4578854.1:16-35 HWI-EAS216_0001:2:22:10751:16954.
agcctgcagaactcaaacag-----------------
>gnl|SRA|SRR352140.3688561.1:16-35 HWI-EAS216_0001:2:18:11050:17823.
agcctgcagaactcaaacag-----------------
>_R_gnl|SRA|SRR352140.21467073.1:1-33 HWI-EAS216_0001:2:100:3600:14239.
agcctgcagaactcaaacag---gtccttagtgaag-
>_R_gnl|SRA|SRR352140.4565102.1:1-33 HWI-EAS216_0001:2:22:9656:8226.
agcctgcagaactcaaacag---gtccttagtgaag-
>_R_gnl|SRA|SRR352140.1785858.1:1-33 HWI-EAS216_0001:2:9:17350:8957.
agcctgcagaactcaaacag---gtccttagtgaag-
>gnl|SRA|SRR352140.22833847.1:9-35 HWI-EAS216_0001:2:106:5154:15528.
agcctgcagaactcaaacag---gtcctta-------
>gnl|SRA|SRR352140.16230878.1:9-35 HWI-EAS216_0001:2:76:13213:9606.
agcctgcagaactcaaacag---gtcctta-------
>gnl|SRA|SRR352140.10142972.1:9-35 HWI-EAS216_0001:2:47:10852:7414.
agcctgcagaactcaaacag---gtcctta-------
>gnl|SRA|SRR352140.6222283.1:9-35 HWI-EAS216_0001:2:29:16896:14198.
agcctgcagaactcaaacag---gtcctta-------
>gnl|SRA|SRR352140.25992194.1:18-35 HWI-EAS216_0001:2:120:13579:17050.
agcctgcagaactcaaac-------------------
>_R_gnl|SRA|SRR352140.18610319.1:5-22 HWI-EAS216_0001:2:87:8399:8185.
agcctgcagaactcaaac-------------------
>_R_gnl|SRA|SRR352140.13816776.1:1-18 HWI-EAS216_0001:2:65:8928:10982.
agcctgcagaactcaaac-------------------
>_R_gnl|SRA|SRR352140.2969820.1:1-18 HWI-EAS216_0001:2:15:6734:15441.
agcctgcagaactcaaac-------------------
>_R_gnl|SRA|SRR352140.21114445.1:1-17 HWI-EAS216_0001:2:98:11422:14065.
agcctgcagaactcaaa--------------------
>_R_gnl|SRA|SRR352140.21039295.1:1-17 HWI-EAS216_0001:2:98:5414:20555.

agcctgcagaactcaaa--------------------
>_R_gnl|SRA|SRR352140.15358199.1:1-17 HWI-EAS216_0001:2:72:13263:3268.
agcctgcagaactcaaa--------------------
>_R_gnl|SRA|SRR352140.14683334.1:1-17 HWI-EAS216_0001:2:69:10729:10186.
agcctgcagaactcaaa--------------------
>_R_gnl|SRA|SRR352140.10663441.1:1-17 HWI-EAS216_0001:2:49:18194:8186.
agcctgcagaactcaaa--------------------

SRX100691
>control .
agcctgcagaactcaaacagtaggtccttagtgaagg
>_R_gnl|SRA|SRR352137.12382241.1:1-35 HWI-EAS216_0001:7:86:12234:13020.
--cctgcagaactcaaacagtaggtccttagtgaagg
>_R_gnl|SRA|SRR352137.10731917.1:1-35 HWI-EAS216_0001:7:75:4107:21304.
--cctgcagaactcaaacagtaggtccttagtgaagg
>gnl|SRA|SRR352137.12695642.1:1-32 HWI-EAS216_0001:7:88:13539:12137.
-----gcagaactcaaacagtaggtccttagtgaagg
>gnl|SRA|SRR352137.11922209.1:1-32 HWI-EAS216_0001:7:83:11463:14387.
-----gcagaactcaaacagtaggtccttagtgaagg
>gnl|SRA|SRR352137.8695483.1:1-32 HWI-EAS216_0001:7:57:13133:20281.
-----gcagaactcaaacagtaggtccttagtgaagg
>gnl|SRA|SRR352137.5012887.1:1-32 HWI-EAS216_0001:7:32:11174:5201.
-----gcagaactcaaacagtaggtccttagtgaagg
>gnl|SRA|SRR352137.16370872.1:1-31 HWI-EAS216_0001:7:112:9371:19636.
------cagaactcaaacagtaggtccttagtgaagg
>gnl|SRA|SRR352137.15374754.1:1-29 HWI-EAS216_0001:7:106:2003:1501.
--------gaactcaaacagtaggtccttagtgaagg
>gnl|SRA|SRR352137.15160681.1:1-29 HWI-EAS216_0001:7:104:13699:8435.
--------gaactcaaacagtaggtccttagtgaagg
>gnl|SRA|SRR352137.11895423.1:1-29 HWI-EAS216_0001:7:83:8150:16318.
--------gaactcaaacagtaggtccttagtgaagg
>gnl|SRA|SRR352137.6604580.1:1-27 HWI-EAS216_0001:7:43:1761:18444.
----------actcaaacagtaggtccttagtgaagg
>gnl|SRA|SRR352137.3168090.1:1-27 HWI-EAS216_0001:7:20:13052:12438.
----------actcaaacagtaggtccttagtgaagg
>gnl|SRA|SRR352137.335823.1:1-27 HWI-EAS216_0001:7:3:1128:12039.
----------actcaaacagtaggtccttagtgaagg
>gnl|SRA|SRR352137.13935074.1:1-26 HWI-EAS216_0001:7:96:15518:14694.
-----------ctcaaacagtaggtccttagtgaagg
>gnl|SRA|SRR352137.10397543.1:1-26 HWI-EAS216_0001:7:72:14265:19604.
-----------ctcaaacagtaggtccttagtgaagg
>gnl|SRA|SRR352137.8308962.1:1-26 HWI-EAS216_0001:7:54:18791:16631.
-----------ctcaaacagtaggtccttagtgaagg
>gnl|SRA|SRR352137.4205876.1:1-26 HWI-EAS216_0001:7:27:7031:12526.
-----------ctcaaacagtaggtccttagtgaagg
>_R_gnl|SRA|SRR352137.6229764.1:13-35 HWI-EAS216_0001:7:40:11187:17081.
--------------aaacagtaggtccttagtgaagg
>_R_gnl|SRA|SRR352137.321650.1:13-35 HWI-EAS216_0001:7:2:18230:8506.

--------------aaacagtaggtccttagtgaagg
>_R_gnl|SRA|SRR352137.16404766.1:1-21 HWI-EAS216_0001:7:112:13359:15324.
---------------aacagtaggtccttagtgaag-
>_R_gnl|SRA|SRR352137.12965292.1:1-21 HWI-EAS216_0001:7:90:9358:2392.
---------------aacagtaggtccttagtgaag-
>_R_gnl|SRA|SRR352137.3075129.1:1-21 HWI-EAS216_0001:7:20:2055:9162.
---------------aacagtaggtccttagtgaag-
>_R_gnl|SRA|SRR352137.2452673.1:1-21 HWI-EAS216_0001:7:16:3146:10428.
---------------aacagtaggtccttagtgaag-
>_R_gnl|SRA|SRR352137.11688473.1:16-35 HWI-EAS216_0001:7:81:19768:3928.
-----------------cagtaggtccttagtgaagg
>_R_gnl|SRA|SRR352137.7469201.1:16-35 HWI-EAS216_0001:7:49:4321:10938.
-----------------cagtaggtccttagtgaagg
>_R_gnl|SRA|SRR352137.3831630.1:1-35 HWI-EAS216_0001:7:24:17768:15257.
agcctgcagaactcaaacagtaggtccttagtgaa--
>_R_gnl|SRA|SRR352137.1160788.1:1-35 HWI-EAS216_0001:7:8:1803:11160.
agcctgcagaactcaaacagtaggtccttagtgaa--
>_R_gnl|SRA|SRR352137.15839992.1:1-30 HWI-EAS216_0001:7:109:1858:6921.
agcctgcagaactcaaacagtaggtcctta-------
>_R_gnl|SRA|SRR352137.471597.1:1-30 HWI-EAS216_0001:7:3:16348:5300.
agcctgcagaactcaaacagtaggtcctta-------
>gnl|SRA|SRR352137.5666040.1:7-35 HWI-EAS216_0001:7:36:16340:11372.
agcctgcagaactcaaacagtaggtcctt--------
>_R_gnl|SRA|SRR352137.14486651.1:2-28 HWI-EAS216_0001:7:100:7618:10808.
agcctgcagaactcaaacagtaggtcc----------
>gnl|SRA|SRR352137.6327783.1:9-35 HWI-EAS216_0001:7:41:4665:14143.
agcctgcagaactcaaacagtaggtcc----------
>_R_gnl|SRA|SRR352137.8504503.1:2-27 HWI-EAS216_0001:7:56:7050:2885.
agcctgcagaactcaaacagtaggtc-----------
>gnl|SRA|SRR352137.17657280.1:11-35 HWI-EAS216_0001:7:120:13359:17654.
agcctgcagaactcaaacagtaggt------------
>gnl|SRA|SRR352137.11067039.1:11-35 HWI-EAS216_0001:7:77:16484:12147.
agcctgcagaactcaaacagtaggt------------
>gnl|SRA|SRR352137.10604555.1:13-35 HWI-EAS216_0001:7:74:3737:19286.
agcctgcagaactcaaacagtag--------------
>gnl|SRA|SRR352137.1975593.1:13-35 HWI-EAS216_0001:7:13:3064:14367.
agcctgcagaactcaaacagtag--------------
>gnl|SRA|SRR352137.9339078.1:15-35 HWI-EAS216_0001:7:65:8679:3291.
agcctgcagaactcaaacagt----------------
>_R_gnl|SRA|SRR352137.14030416.1:3-22 HWI-EAS216_0001:7:97:8565:17679.
agcctgcagaactcaaacag-----------------
>_R_gnl|SRA|SRR352137.4204653.1:3-22 HWI-EAS216_0001:7:27:6877:3008.
agcctgcagaactcaaacag-----------------
>_R_gnl|SRA|SRR352137.8077513.1:1-34 HWI-EAS216_0001:7:53:7495:2246.
agcctgcagaactcaaacag---gtccttagtgaagg
>_R_gnl|SRA|SRR352137.16376327.1:1-33 HWI-EAS216_0001:7:112:10025:20802.
agcctgcagaactcaaacag---gtccttagtgaag-
>_R_gnl|SRA|SRR352137.2598568.1:1-33 HWI-EAS216_0001:7:17:1605:10903.
agcctgcagaactcaaacag---gtccttagtgaag-
>gnl|SRA|SRR352137.15319989.1:10-35 HWI-EAS216_0001:7:105:14132:2022.
agcctgcagaactcaaacag---gtcctt--------

>gnl|SRA|SRR352137.3439687.1:10-35 HWI-EAS216_0001:7:22:8310:15318.
agcctgcagaactcaaacag---gtcctt--------
>_R_gnl|SRA|SRR352137.1405419.1:7-35 HWI-EAS216_0001:7:9:11336:1206.
-----gcagaactcaaacag---gtccttagtgaagg
>gnl|SRA|SRR352137.16351184.1:15-32 HWI-EAS216_0001:7:112:6985:19744.
--cctgcagaactcgaacag-----------------
>_R_gnl|SRA|SRR352137.12608305.1:3-20 HWI-EAS216_0001:7:88:2753:10807.
--cctgcagaactcgaacag-----------------
>_R_gnl|SRA|SRR352137.12595043.1:3-20 HWI-EAS216_0001:7:88:1082:7458.
--cctgcagaactcgaacag-----------------
>gnl|SRA|SRR352137.11936758.1:16-33 HWI-EAS216_0001:7:83:13222:10859.
--cctgcagaactcgaacag-----------------
>gnl|SRA|SRR352137.11469575.1:14-31 HWI-EAS216_0001:7:80:11077:17078.
--cctgcagaactcgaacag-----------------
>gnl|SRA|SRR352137.7213088.1:14-31 HWI-EAS216_0001:7:47:8308:12260.
--cctgcagaactcgaacag-----------------
>gnl|SRA|SRR352137.6353868.1:14-31 HWI-EAS216_0001:7:41:7869:4785.
--cctgcagaactcgaacag-----------------
>gnl|SRA|SRR352137.6155586.1:16-33 HWI-EAS216_0001:7:40:2058:6723.
--cctgcagaactcgaacag-----------------
>_R_gnl|SRA|SRR352137.1789854.1:11-28 HWI-EAS216_0001:7:11:18597:12524.
--cctgcagaactcgaacag-----------------
>gnl|SRA|SRR352137.50726.1:17-34 HWI-EAS216_0001:7:1:6851:10108.
--cctgcagaactcgaacag-----------------
>gnl|SRA|SRR352137.1127047.1:5-22 HWI-EAS216_0001:7:7:16494:12322.
--cctgcagaactccaacag-----------------

SRX375649; SRX378862
>control .
cttgcaaaactcaaacagtaggtcactggtgagagatt------
>gnl|SRA|SRR1032036.5745293.1:28-65 5745293.
cttgcaaaactcaaacagtaggtcactggtgagagatt------
>gnl|SRA|SRR1032036.5555573.2:28-65 5555573.
cttgcaaaactcaaacagtaggtcactggtgagagatt------
>gnl|SRA|SRR1032036.5007230.1:28-65 5007230.
cttgcaaaactcaaacagtaggtcactggtgagagatt------
>gnl|SRA|SRR1032036.4935301.1:28-65 4935301.
cttgcaaaactcaaacagtaggtcactggtgagagatt------
>gnl|SRA|SRR1032036.3959680.2:28-65 3959680.
cttgcaaaactcaaacagtaggtcactggtgagagatt------
>gnl|SRA|SRR1032036.3837994.2:28-65 3837994.
cttgcaaaactcaaacagtaggtcactggtgagagatt------
>gnl|SRA|SRR1032036.3702521.2:16-53 3702521.
cttgcaaaactcaaacagtaggtcactggtgagagatt------
>gnl|SRA|SRR1032036.3216849.2:3-40 3216849.
cttgcaaaactcaaacagtaggtcactggtgagagatt------
>_R_gnl|SRA|SRR1032036.2332968.2:25-62 2332968.
cttgcaaaactcaaacagtaggtcactggtgagagatt------

>gnl|SRA|SRR1032036.1502148.1:5-42 1502148.
cttgcaaaactcaaacagtaggtcactggtgagagatt------
>gnl|SRA|SRR1032036.1429973.1:39-76 1429973.
cttgcaaaactcaaacagtaggtcactggtgagagatt------
>gnl|SRA|SRR1032036.1313769.2:28-65 1313769.
cttgcaaaactcaaacagtaggtcactggtgagagatt------
>_R_gnl|SRA|SRR1032036.824553.1:45-82 824553.
cttgcaaaactcaaacagtaggtcactggtgagagatt------
>gnl|SRA|SRR1032035.5309896.1:37-74 5309896.
cttgcaaaactcaaacagtaggtcactggtgagagatt------
>_R_gnl|SRA|SRR1032035.4894614.2:11-48 4894614.
cttgcaaaactcaaacagtaggtcactggtgagagatt------
>gnl|SRA|SRR1032035.1063244.2:16-53 1063244.
cttgcaaaactcaaacagtaggtcactggtgagagatt------
>gnl|SRA|SRR1032036.4605798.1:55-90 4605798.
cttgcaaaactcaaacagtaggtcactggtgagaga--------
>gnl|SRA|SRR1032036.4879608.1:1-31 4879608.
-------aactcaaacagtaggtcactggtgagagatt------
>gnl|SRA|SRR1032036.5236159.2:1-27 5236159.
-----------caaacagtaggtcactggtgagagatt------
>gnl|SRA|SRR1032036.4758333.1:1-27 4758333.
-----------caaacagtaggtcactggtgagagatt------
>gnl|SRA|SRR1032036.4299937.2:1-27 4299937.
-----------caaacagtaggtcactggtgagagatt------
>gnl|SRA|SRR1032036.4250327.1:1-27 4250327.
-----------caaacagtaggtcactggtgagagatt------
>_R_gnl|SRA|SRR1032036.4483539.2:69-90 4483539.
----------------agtaggtcactggtgagagatt------
>_R_gnl|SRA|SRR1032036.1321147.1:69-90 1321147.
----------------agtaggtcactggtgagagatt------
>_R_gnl|SRA|SRR1032036.3022214.2:70-90 3022214.
-----------------gtaggtcactggtgagagatt------
>gnl|SRA|SRR1032036.4136576.1:4-22 4136576.
-------------------aggtcactggtgagagatt------
>gnl|SRA|SRR1032036.1949383.2:4-22 1949383.
-------------------aggtcactggtgagagatt------
>_R_gnl|SRA|SRR1032036.1043816.1:70-88 1043816.
-------------------aggtcactggtgagagatt------
>gnl|SRA|SRR1032036.5645161.1:1-18 5645161.
--------------------ggtcactggtgagagatt------
>_R_gnl|SRA|SRR1032036.6111209.1:74-90 6111209.
---------------------gtcactggtgagagatt------
>gnl|SRA|SRR1032036.590245.1:1-28 590245.
-------aactcaaacag---gtcactggtgagagatt------
>gnl|SRA|SRR1032036.6058905.1:48-82 6058905.
cttgcaaaactcaaacag---gtcactggtgagagatt------
>gnl|SRA|SRR1032036.5867131.2:40-74 5867131.
cttgcaaaactcaaacag---gtcactggtgagagatt------
>gnl|SRA|SRR1032036.4767729.1:41-75 4767729.
cttgcaaaactcaaacag---gtcactggtgagagatt------
>gnl|SRA|SRR1032036.4412091.1:16-50 4412091.

cttgcaaaactcaaacag---gtcactggtgagagatt------
>gnl|SRA|SRR1032036.3707201.2:15-49 3707201.
cttgcaaaactcaaacag---gtcactggtgagagatt------
>gnl|SRA|SRR1032036.3069259.1:40-74 3069259.
cttgcaaaactcaaacag---gtcactggtgagagatt------
>gnl|SRA|SRR1032036.2257093.2:40-74 2257093.
cttgcaaaactcaaacag---gtcactggtgagagatt------
>gnl|SRA|SRR1032036.1335367.2:39-73 1335367.
cttgcaaaactcaaacag---gtcactggtgagagatt------
>gnl|SRA|SRR1032036.719022.1:31-65 719022.
cttgcaaaactcaaacag---gtcactggtgagagatt------
>gnl|SRA|SRR1032035.6313425.2:2-36 6313425.
cttgcaaaactcaaacag---gtcactggtgagagatt------
>gnl|SRA|SRR1032035.965005.1:37-71 965005.
cttgcaaaactcaaacag---gtcactggtgagagatt------
>gnl|SRA|SRR1032036.272677.2:58-90 272677.
cttgcaaaactcaaacag---gtcactggtgagaga-------
>gnl|SRA|SRR1032035.1508684.2:51-76 1508684.
cttgcaaaactcaaacag---gtcactgg---------------
>gnl|SRA|SRR1032035.1201119.2:51-76 1201119.
cttgcaaaactcaaacag---gtcactgg---------------
>gnl|SRA|SRR1032036.497056.2:28-62 497056.
cttgcaaaactcaaacag---ggcactggtgagagatt------

SRX099185
CLUSTAL W(1.60) multiple sequence alignment

control gcttgcaaaacgcaaacagtaggaccatagtgggagat-----
_R_gnl|SRA|SRR349768.17046803.1:1-30 gcttgcaaaacgcaaacagtaggaccatag-------------
gnl|SRA|SRR349768.16503247.1:10-35 gcttgcaaaacgcaaacagtaggacc-----------------
_R_gnl|SRA|SRR349768.9937425.1:1-23 gcttgcaaaacgcaaacagtagg--------------------
_R_gnl|SRA|SRR349768.20037715.1:4-22 gcttgcaaaacgcaaacag------------------------
_R_gnl|SRA|SRR349768.8714895.1:4-22 gcttgcaaaacgcaaacag------------------------
_R_gnl|SRA|SRR349768.5532076.1:1-17 gcttgcaaaacgcaaac--------------------------
_R_gnl|SRA|SRR349768.8711755.1:1-16 gcttgcaaaacgcaaa---------------------------
_R_gnl|SRA|SRR349768.8358473.1:1-16 gcttgcaaaacgcaaa---------------------------
_R_gnl|SRA|SRR349768.1897105.1:1-16 gcttgcaaaacgcaaa---------------------------
_R_gnl|SRA|SRR349768.20072115.1:1-15 gcttgcaaaacgcaa----------------------------
_R_gnl|SRA|SRR349768.16514850.1:1-15 gcttgcaaaacgcaa----------------------------
_R_gnl|SRA|SRR349768.13268680.1:1-30 gcttgcaaaacgcaaacag---gaccatagtgg----------
_R_gnl|SRA|SRR349768.9496473.1:1-29 gcttgcaaaacgcaaacag---gaccatagtg-----------
gnl|SRA|SRR349768.8652267.1:10-35 gcttgcaaaacgcaaacag---gaccata--------------
gnl|SRA|SRR349768.1663449.1:17-34 gcttgcaaaacacaaaca-------------------------
_R_gnl|SRA|SRR349768.12938454.1:4-22 gcttgcataacgcaaacag------------------------
gnl|SRA|SRR349768.205710.1:1-28 ----------cgcaaacagtaggaccatagtgggagat-----
_R_gnl|SRA|SRR349768.19396153.1:10-35 ------------caaacagtaggaccatagtgggagat-----
_R_gnl|SRA|SRR349768.5354797.1:10-35 ------------caaacagtaggaccatagtgggagat-----

_R_gnl|SRA|SRR349768.14316331.1:16-35 ---------------gtag---gaccatagtgggagat-----
gnl|SRA|SRR349768.15272433.1:2-19 -----------------ag---gaccatagtgggagat-----
gnl|SRA|SRR349768.13942263.1:6-23 -----------------ag---gaccatagtgggagat-----
gnl|SRA|SRR349768.9523738.1:2-19 -----------------ag---gaccatagtgggagat-----
gnl|SRA|SRR349768.5376949.1:4-21 -----------------ag---gaccatagtgggagat-----
gnl|SRA|SRR349768.4458748.1:2-19 -----------------ag---gaccatagtgggagat-----
_R_gnl|SRA|SRR349768.18290109.1:20-35 ----------------------gaccatagtgggagat-----
gnl|SRA|SRR349768.3924048.1:1-16 ----------------------gaccatagtgggagat-----
gnl|SRA|SRR349768.7050873.1:1-33 --ttgcaaaacgcaaacag---gaccatagtgggagat-----
gnl|SRA|SRR349768.9230090.1:1-32 ---tgcaaaacgcaaacag---gaccatagtgggagat-----
gnl|SRA|SRR349768.18202966.1:1-31 ----gcaaaacgcaaacag---gaccatagtgggagat-----
gnl|SRA|SRR349768.14306986.1:1-31 ----gcaaaacgcaaacag---gaccatagtgggagat-----
_R_gnl|SRA|SRR349768.8148045.1:9-35 --------aacgcaaacag---gaccatagtgggagat-----
gnl|SRA|SRR349768.11044246.1:1-25 ----------cgcaaacag---gaccatagtgggagat-----
_R_gnl|SRA|SRR349768.8817615.1:11-35 ----------cgcaaacag---gaccatagtgggagat-----
_R_gnl|SRA|SRR349768.7230622.1:11-35 ----------cgcaaacag---gaccatagtgggagat-----
_R_gnl|SRA|SRR349768.3206310.1:11-35 ----------cgcaaacag---gaccatagtgggagat-----
_R_gnl|SRA|SRR349768.1869095.1:11-35 ----------cgcaaacag---gaccatagtgggagat-----
gnl|SRA|SRR349768.8120004.1:1-24 -----------gcaaacag---gaccatagtgggagat-----
_R_gnl|SRA|SRR349768.13177496.1:6-35 -----caaaacgcaaacag---gaccatagtgggacat-----
gnl|SRA|SRR349768.15505982.1:17-34 ----------------cagtaggaccaaagtggg---------
gnl|SRA|SRR349768.10384073.1:12-29 ----------------cagtaggaccaaagtggg---------
_R_gnl|SRA|SRR349768.8331184.1:14-31 ----------------cagtaggaccaaagtggg---------
_R_gnl|SRA|SRR349768.7704725.1:14-31 ----------------cagtaggaccaaagtggg---------

SRX099141
>control .
--gcttgcaaaacgcaaacagtaggaccatagtgggagat------
>_R_gnl|SRA|SRR349754.9855210.1:1-35 HWI-EAS216_0001:6:40:10905:17009.
---cttgcaaaacgcaaacagtaggaccatagtgggag--------
>_R_gnl|SRA|SRR349754.11706967.1:1-30 HWI-EAS216_0001:6:48:2003:15633.
--gcttgcaaaacgcaaacagtaggaccatag--------------
>_R_gnl|SRA|SRR349754.7967767.1:1-34 HWI-EAS216_0001:6:32:18399:9457.
--gcttgcaaaacgcaaacag---gaccatagtgggaga-------
>gnl|SRA|SRR349754.19488278.1:3-35 HWI-EAS216_0001:6:80:11926:6551.
--gcttgcaaaacgcaaacag---gaccatagtgggag--------
>_R_gnl|SRA|SRR349754.2770060.1:1-33 HWI-EAS216_0001:6:12:4272:15875.
--gcttgcaaaacgcaaacag---gaccatagtgggag--------
>gnl|SRA|SRR349754.25012041.1:4-35 HWI-EAS216_0001:6:103:10908:12580.
--gcttgcaaaacgcaaacag---gaccatagtggga---------
>gnl|SRA|SRR349754.2603629.1:4-35 HWI-EAS216_0001:6:11:10404:11263.
--gcttgcaaaacgcaaacag---gaccatagtggga---------
>_R_gnl|SRA|SRR349754.24353444.1:1-30 HWI-EAS216_0001:6:100:15596:12749.
--gcttgcaaaacgcaaacag---gaccatagtgg-----------
>gnl|SRA|SRR349754.20115172.1:6-35 HWI-EAS216_0001:6:83:5020:10458.
--gcttgcaaaacgcaaacag---gaccatagtgg-----------
>gnl|SRA|SRR349754.5512492.1:6-35 HWI-EAS216_0001:6:23:3133:8346.
--gcttgcaaaacgcaaacag---gaccatagtgg-----------
>_R_gnl|SRA|SRR349754.2166315.1:1-30 HWI-EAS216_0001:6:9:15084:15371.

--gcttgcaaaacgcaaacag---gaccatagtgg-----------
>_R_gnl|SRA|SRR349754.11303458.1:1-29 HWI-EAS216_0001:6:46:8618:1632.
--gcttgcaaaacgcaaacag---gaccatagtg------------
>_R_gnl|SRA|SRR349754.15808649.1:1-27 HWI-EAS216_0001:6:65:2209:3194.
--gcttgcaaaacgcaaacag---gaccatag--------------
>_R_gnl|SRA|SRR349754.14385194.1:1-27 HWI-EAS216_0001:6:59:2204:11990.
--gcttgcaaaacgcaaacag---gaccatag--------------
>gnl|SRA|SRR349754.9437114.1:9-35 HWI-EAS216_0001:6:38:16761:18433.
--gcttgcaaaacgcaaacag---gaccatag--------------
>_R_gnl|SRA|SRR349754.1023630.1:1-27 HWI-EAS216_0001:6:5:4114:20604.
--gcttgcaaaacgcaaacag---gaccatag--------------
>gnl|SRA|SRR349754.27742576.1:10-35 HWI-EAS216_0001:6:114:15778:16480.
--gcttgcaaaacgcaaacag---gaccata---------------
>gnl|SRA|SRR349754.23825876.1:10-35 HWI-EAS216_0001:6:98:12137:5602.
--gcttgcaaaacgcaaacag---gaccata---------------
>_R_gnl|SRA|SRR349754.16812513.1:1-26 HWI-EAS216_0001:6:69:7086:1575.
--gcttgcaaaacgcaaacag---gaccata---------------
>_R_gnl|SRA|SRR349754.5826911.1:1-26 HWI-EAS216_0001:6:24:7930:10132.
--gcttgcaaaacgcaaacag---gaccata---------------
>gnl|SRA|SRR349754.4621285.1:10-35 HWI-EAS216_0001:6:19:11456:9825.
--gcttgcaaaacgcaaacag---gaccata---------------
>gnl|SRA|SRR349754.3381525.1:10-35 HWI-EAS216_0001:6:14:12477:7546.
--gcttgcaaaacgcaaacag---gaccata---------------
>gnl|SRA|SRR349754.1573519.1:10-35 HWI-EAS216_0001:6:7:7999:5621.
--gcttgcaaaacgcaaacag---gaccata---------------
>gnl|SRA|SRR349754.1034516.1:17-35 HWI-EAS216_0001:6:5:4927:14225.
--gcttgcaaaacgcaaatag-------------------------
>gnl|SRA|SRR349754.25094040.1:1-34 HWI-EAS216_0001:6:103:17018:21400.
------gcaaaacgcaaacagtaggaccatagtgggagat------
>gnl|SRA|SRR349754.9543159.1:1-34 HWI-EAS216_0001:6:39:6222:8187.
------gcaaaacgcaaacagtaggaccatagtgggagat------
>gnl|SRA|SRR349754.8745252.1:1-32 HWI-EAS216_0001:6:36:2342:8538.
--------aaaacgcaaacagtaggaccatagtgggagat------
>gnl|SRA|SRR349754.28995275.1:1-28 HWI-EAS216_0001:6:120:1203:18853.
------------cgcaaacagtaggaccatagtgggagat------
>gnl|SRA|SRR349754.16433898.1:1-28 HWI-EAS216_0001:6:67:14011:6420.
------------cgcaaacagtaggaccatagtgggagat------
>gnl|SRA|SRR349754.10288044.1:1-28 HWI-EAS216_0001:6:42:6335:2740.
------------cgcaaacagtaggaccatagtgggagat------
>gnl|SRA|SRR349754.24853655.1:1-27 HWI-EAS216_0001:6:102:17069:6745.
-------------gcaaacagtaggaccatagtgggagat------
>gnl|SRA|SRR349754.6010256.1:1-26 HWI-EAS216_0001:6:25:3003:17762.
--------------caaacagtaggaccatagtgggagat------
>_R_gnl|SRA|SRR349754.20294725.1:11-35 HWI-EAS216_0001:6:83:18616:12980.
---------------aaacagtaggaccatagtgggagat------
>gnl|SRA|SRR349754.10802162.1:1-25 HWI-EAS216_0001:6:44:7881:20366.
---------------aaacagtaggaccatagtgggagat------
>gnl|SRA|SRR349754.5862039.1:1-25 HWI-EAS216_0001:6:24:10519:6518.
---------------aaacagtaggaccatagtgggagat------
>gnl|SRA|SRR349754.11980431.1:1-34 HWI-EAS216_0001:6:49:4317:2506.
---cttgcaaaacgcaaacag---gaccatagtgggagat------

>gnl|SRA|SRR349754.24760990.1:1-33 HWI-EAS216_0001:6:102:10212:4013.
----ttgcaaaacgcaaacag---gaccatagtgggagat------
>gnl|SRA|SRR349754.19747071.1:1-33 HWI-EAS216_0001:6:81:13229:3749.
----ttgcaaaacgcaaacag---gaccatagtgggagat------
>gnl|SRA|SRR349754.4369478.1:1-33 HWI-EAS216_0001:6:18:11385:9963.
----ttgcaaaacgcaaacag---gaccatagtgggagat------
>gnl|SRA|SRR349754.22105662.1:1-32 HWI-EAS216_0001:6:91:10154:1460.
-----tgcaaaacgcaaacag---gaccatagtgggagat------
>_R_gnl|SRA|SRR349754.19388521.1:4-35 HWI-EAS216_0001:6:80:4551:6068.
-----tgcaaaacgcaaacag---gaccatagtgggagat------
>gnl|SRA|SRR349754.17627970.1:1-32 HWI-EAS216_0001:6:72:14959:17832.
-----tgcaaaacgcaaacag---gaccatagtgggagat------
>_R_gnl|SRA|SRR349754.15050744.1:4-35 HWI-EAS216_0001:6:61:15991:16291.
-----tgcaaaacgcaaacag---gaccatagtgggagat------
>_R_gnl|SRA|SRR349754.8773022.1:4-35 HWI-EAS216_0001:6:36:4440:19341.
-----tgcaaaacgcaaacag---gaccatagtgggagat------
>_R_gnl|SRA|SRR349754.2066416.1:4-35 HWI-EAS216_0001:6:9:7749:20477.
-----tgcaaaacgcaaacag---gaccatagtgggagat------
>gnl|SRA|SRR349754.24837332.1:1-31 HWI-EAS216_0001:6:102:15788:9960.
------gcaaaacgcaaacag---gaccatagtgggagat------
>gnl|SRA|SRR349754.22042206.1:1-31 HWI-EAS216_0001:6:91:5529:7739.
------gcaaaacgcaaacag---gaccatagtgggagat------
>gnl|SRA|SRR349754.21883509.1:1-31 HWI-EAS216_0001:6:90:11592:15454.
------gcaaaacgcaaacag---gaccatagtgggagat------
>gnl|SRA|SRR349754.20819864.1:1-31 HWI-EAS216_0001:6:86:3993:6861.
------gcaaaacgcaaacag---gaccatagtgggagat------
>gnl|SRA|SRR349754.18481553.1:1-31 HWI-EAS216_0001:6:76:7783:6619.
------gcaaaacgcaaacag---gaccatagtgggagat------
>gnl|SRA|SRR349754.15154436.1:1-31 HWI-EAS216_0001:6:62:6046:8236.
------gcaaaacgcaaacag---gaccatagtgggagat------
>gnl|SRA|SRR349754.8519440.1:1-31 HWI-EAS216_0001:6:35:4042:3066.
------gcaaaacgcaaacag---gaccatagtgggagat------
>gnl|SRA|SRR349754.4156594.1:1-31 HWI-EAS216_0001:6:17:14121:11232.
------gcaaaacgcaaacag---gaccatagtgggagat------
>gnl|SRA|SRR349754.2976887.1:1-31 HWI-EAS216_0001:6:12:19571:21228.
------gcaaaacgcaaacag---gaccatagtgggagat------
>gnl|SRA|SRR349754.790075.1:1-31 HWI-EAS216_0001:6:4:5101:11825.
------gcaaaacgcaaacag---gaccatagtgggagat------
>_R_gnl|SRA|SRR349754.27200944.1:6-35 HWI-EAS216_0001:6:112:11620:6844.
-------caaaacgcaaacag---gaccatagtgggagat------
>gnl|SRA|SRR349754.25943492.1:1-30 HWI-EAS216_0001:6:107:8270:9217.
-------caaaacgcaaacag---gaccatagtgggagat------
>_R_gnl|SRA|SRR349754.25152965.1:6-35 HWI-EAS216_0001:6:104:3617:6208.
-------caaaacgcaaacag---gaccatagtgggagat------
>_R_gnl|SRA|SRR349754.22010036.1:6-35 HWI-EAS216_0001:6:91:3143:16125.
-------caaaacgcaaacag---gaccatagtgggagat------
>gnl|SRA|SRR349754.14239189.1:1-30 HWI-EAS216_0001:6:58:9561:3710.
-------caaaacgcaaacag---gaccatagtgggagat------
>gnl|SRA|SRR349754.9157578.1:1-30 HWI-EAS216_0001:6:37:14445:7491.
-------caaaacgcaaacag---gaccatagtgggagat------
>_R_gnl|SRA|SRR349754.8506672.1:6-35 HWI-EAS216_0001:6:35:3085:5131.

-------caaaacgcaaacag---gaccatagtgggagat------
>_R_gnl|SRA|SRR349754.8362108.1:6-35 HWI-EAS216_0001:6:34:10833:16601.
-------caaaacgcaaacag---gaccatagtgggagat------
>gnl|SRA|SRR349754.14512309.1:1-29 HWI-EAS216_0001:6:59:12003:15102.
--------aaaacgcaaacag---gaccatagtgggagat------
>_R_gnl|SRA|SRR349754.28218957.1:8-35 HWI-EAS216_0001:6:116:15232:3373.
---------aaacgcaaacag---gaccatagtgggagat------
>gnl|SRA|SRR349754.28007848.1:1-28 HWI-EAS216_0001:6:115:17573:12191.
---------aaacgcaaacag---gaccatagtgggagat------
>_R_gnl|SRA|SRR349754.7449868.1:8-35 HWI-EAS216_0001:6:30:16925:3916.
---------aaacgcaaacag---gaccatagtgggagat------
>_R_gnl|SRA|SRR349754.20350411.1:9-35 HWI-EAS216_0001:6:84:4755:19724.
----------aacgcaaacag---gaccatagtgggagat------
>_R_gnl|SRA|SRR349754.96270.1:9-35 HWI-EAS216_0001:6:1:8369:12922.
----------aacgcaaacag---gaccatagtgggagat------
>_R_gnl|SRA|SRR349754.29014160.1:10-35 HWI-EAS216_0001:6:120:2619:4113.
-----------acgcaaacag---gaccatagtgggagat------
>_R_gnl|SRA|SRR349754.21302922.1:10-35 HWI-EAS216_0001:6:88:4380:5469.
-----------acgcaaacag---gaccatagtgggagat------
>_R_gnl|SRA|SRR349754.17965265.1:10-35 HWI-EAS216_0001:6:74:4726:17633.
-----------acgcaaacag---gaccatagtgggagat------
>_R_gnl|SRA|SRR349754.14566285.1:10-35 HWI-EAS216_0001:6:59:16103:9662.
-----------acgcaaacag---gaccatagtgggagat------
>_R_gnl|SRA|SRR349754.28122582.1:11-35 HWI-EAS216_0001:6:116:8210:1126.
------------cgcaaacag---gaccatagtgggagat------
>_R_gnl|SRA|SRR349754.21427515.1:11-35 HWI-EAS216_0001:6:88:13463:18892.
------------cgcaaacag---gaccatagtgggagat------
>_R_gnl|SRA|SRR349754.20287277.1:11-35 HWI-EAS216_0001:6:83:17910:12951.
------------cgcaaacag---gaccatagtgggagat------
>gnl|SRA|SRR349754.18652025.1:1-25 HWI-EAS216_0001:6:77:2822:15514.
------------cgcaaacag---gaccatagtgggagat------
>gnl|SRA|SRR349754.18640719.1:1-25 HWI-EAS216_0001:6:77:1980:6775.
------------cgcaaacag---gaccatagtgggagat------
>_R_gnl|SRA|SRR349754.17768107.1:11-35 HWI-EAS216_0001:6:73:7701:5335.
------------cgcaaacag---gaccatagtgggagat------
>_R_gnl|SRA|SRR349754.12234173.1:11-35 HWI-EAS216_0001:6:50:5005:6332.
------------cgcaaacag---gaccatagtgggagat------
>gnl|SRA|SRR349754.11865586.1:1-25 HWI-EAS216_0001:6:48:14022:7774.
------------cgcaaacag---gaccatagtgggagat------
>gnl|SRA|SRR349754.11860474.1:1-25 HWI-EAS216_0001:6:48:13639:5246.
------------cgcaaacag---gaccatagtgggagat------
>_R_gnl|SRA|SRR349754.7993300.1:11-35 HWI-EAS216_0001:6:33:1811:18041.
------------cgcaaacag---gaccatagtgggagat------
>gnl|SRA|SRR349754.6791743.1:1-25 HWI-EAS216_0001:6:28:5253:12467.
------------cgcaaacag---gaccatagtgggagat------
>gnl|SRA|SRR349754.3841864.1:1-25 HWI-EAS216_0001:6:16:9423:6907.
------------cgcaaacag---gaccatagtgggagat------
>gnl|SRA|SRR349754.27893288.1:1-24 HWI-EAS216_0001:6:115:9187:20740.
-------------gcaaacag---gaccatagtgggagat------
>gnl|SRA|SRR349754.27524634.1:1-24 HWI-EAS216_0001:6:113:17637:8459.
-------------gcaaacag---gaccatagtgggagat------

>gnl|SRA|SRR349754.27459915.1:1-24 HWI-EAS216_0001:6:113:12834:11882.
-------------gcaaacag---gaccatagtgggagat------
>gnl|SRA|SRR349754.23743767.1:1-24 HWI-EAS216_0001:6:98:6108:12597.
-------------gcaaacag---gaccatagtgggagat------
>gnl|SRA|SRR349754.14719850.1:1-24 HWI-EAS216_0001:6:60:9496:11143.
-------------gcaaacag---gaccatagtgggagat------
>gnl|SRA|SRR349754.7059640.1:1-24 HWI-EAS216_0001:6:29:6560:5402.
-------------gcaaacag---gaccatagtgggagat------
>_R_gnl|SRA|SRR349754.5631848.1:12-35 HWI-EAS216_0001:6:23:11950:15890.
-------------gcaaacag---gaccatagtgggagat------

SRX099021
>control .
gcttgcaaaacgcaaacagtaggaccatagtgggagat---
>gnl|SRA|SRR349645.13998569.1:1-38 HWI-EAS397_0017:4:69:12496:17821.
gcttgcaaaacgcaaacagtaggaccatagtgggagat---
>gnl|SRA|SRR349645.6075703.1:1-37 HWI-EAS397_0017:4:30:3517:7123.
-cttgcaaaacgcaaacagtaggaccatagtgggagat---
>gnl|SRA|SRR349645.15925442.1:1-35 HWI-EAS397_0017:4:79:9304:19116.
---tgcaaaacgcaaacagtaggaccatagtgggagat---
>gnl|SRA|SRR349645.2787102.1:1-34 HWI-EAS397_0017:4:14:7699:10363.
----gcaaaacgcaaacagtaggaccatagtgggagat---
>_R_gnl|SRA|SRR349645.8339160.1:10-40 HWI-EAS397_0017:4:41:4610:3267.
-------aaacgcaaacagtaggaccatagtgggagat---
>gnl|SRA|SRR349645.23138606.1:1-28 HWI-EAS397_0017:4:114:16047:15462.
----------cgcaaacagtaggaccatagtgggagat---
>gnl|SRA|SRR349645.14998797.1:1-28 HWI-EAS397_0017:4:74:13892:9312.
----------cgcaaacagtaggaccatagtgggagat---
>_R_gnl|SRA|SRR349645.10401737.1:13-40 HWI-EAS397_0017:4:51:12880:20876.
----------cgcaaacagtaggaccatagtgggagat---
>gnl|SRA|SRR349645.6457799.1:1-28 HWI-EAS397_0017:4:31:17778:3589.
----------cgcaaacagtaggaccatagtgggagat---
>_R_gnl|SRA|SRR349645.19848254.1:1-36 HWI-EAS397_0017:4:98:17620:11162.
gcttgcaaaacgcaaacagtaggaccatagtgggag-----
>_R_gnl|SRA|SRR349645.13462972.1:1-36 HWI-EAS397_0017:4:66:18127:4058.
gcttgcaaaacgcaaacagtaggaccatagtgggag-----
>gnl|SRA|SRR349645.1902459.1:7-40 HWI-EAS397_0017:4:10:1928:16215.
gcttgcaaaacgcaaacagtaggaccatagtggg-------
>gnl|SRA|SRR349645.16404522.1:10-40 HWI-EAS397_0017:4:81:16992:20625.
gcttgcaaaacgcaaacagtaggaccatagt----------
>_R_gnl|SRA|SRR349645.989878.1:1-30 HWI-EAS397_0017:4:5:12179:19841.
gcttgcaaaacgcaaacagtaggaccatag-----------
>_R_gnl|SRA|SRR349645.4217957.1:1-35 HWI-EAS397_0017:4:21:6801:15793.
gcttgcaaaacgcaaacagtaggaccatagcggga------
>_R_gnl|SRA|SRR349645.21015580.1:1-33 HWI-EAS397_0017:4:104:9271:6768.
gcttgcaaaacgcaaacag---gaccatagtgggag-----
>_R_gnl|SRA|SRR349645.14360605.1:1-33 HWI-EAS397_0017:4:71:9505:11900.
gcttgcaaaacgcaaacag---gaccatagtgggag-----
>_R_gnl|SRA|SRR349645.11583241.1:1-33 HWI-EAS397_0017:4:57:11787:21088.

gcttgcaaaacgcaaacag---gaccatagtgggag-----
>_R_gnl|SRA|SRR349645.9994260.1:1-33 HWI-EAS397_0017:4:49:11463:16703.
gcttgcaaaacgcaaacag---gaccatagtgggag-----
>_R_gnl|SRA|SRR349645.5326673.1:1-33 HWI-EAS397_0017:4:26:11522:19463.
gcttgcaaaacgcaaacag---gaccatagtgggag-----
>gnl|SRA|SRR349645.23683630.1:9-40 HWI-EAS397_0017:4:117:14482:8097.
gcttgcaaaacgcaaacag---gaccatagtggga------
>gnl|SRA|SRR349645.19372723.1:10-40 HWI-EAS397_0017:4:96:12646:20556.
gcttgcaaaacgcaaacag---gaccatagtggg-------
>_R_gnl|SRA|SRR349645.6007256.1:2-32 HWI-EAS397_0017:4:29:15389:17490.
gcttgcaaaacgcaaacag---gaccatagtggg-------
>gnl|SRA|SRR349645.2892755.1:10-40 HWI-EAS397_0017:4:14:16792:11465.
gcttgcaaaacgcaaacag---gaccatagtggg-------
>_R_gnl|SRA|SRR349645.18795281.1:3-29 HWI-EAS397_0017:4:93:16058:19561.
gcttgcaaaacgcaaacag---gaccatag-----------
>gnl|SRA|SRR349645.18367622.1:14-40 HWI-EAS397_0017:4:91:14539:9456.
gcttgcaaaacgcaaacag---gaccatag-----------
>gnl|SRA|SRR349645.17510428.1:14-40 HWI-EAS397_0017:4:87:10271:15143.
gcttgcaaaacgcaaacag---gaccatag-----------
>_R_gnl|SRA|SRR349645.16108797.1:1-27 HWI-EAS397_0017:4:80:8145:14459.
gcttgcaaaacgcaaacag---gaccatag-----------
>_R_gnl|SRA|SRR349645.2836729.1:1-30 HWI-EAS397_0017:4:14:11899:11328.
gcttgcaaaacgcaaacag---gaccatagcgg--------
>gnl|SRA|SRR349645.13890426.1:15-40 HWI-EAS397_0017:4:69:2860:19796.
gcttgcaaaacgcaaacag---gaccata------------
>gnl|SRA|SRR349645.24304669.1:16-40 HWI-EAS397_0017:4:120:13350:9192.
gcttgcaaaacgcaaacag---gaccat-------------
>gnl|SRA|SRR349645.14695290.1:16-40 HWI-EAS397_0017:4:73:4257:16420.
gcttgcaaaacgcaaacag---gaccat-------------
>_R_gnl|SRA|SRR349645.22924183.1:3-37 HWI-EAS397_0017:4:113:15349:10820.
gcttgcaaaacgcaaacag---gaccatagtgggagat---
>_R_gnl|SRA|SRR349645.21669970.1:6-40 HWI-EAS397_0017:4:107:12374:8136.
gcttgcaaaacgcaaacag---gaccatagtgggagat---
>gnl|SRA|SRR349645.19712099.1:4-38 HWI-EAS397_0017:4:98:6358:16603.
gcttgcaaaacgcaaacag---gaccatagtgggagat---
>_R_gnl|SRA|SRR349645.17221010.1:6-40 HWI-EAS397_0017:4:86:2042:11021.
gcttgcaaaacgcaaacag---gaccatagtgggagat---
>gnl|SRA|SRR349645.16817448.1:1-35 HWI-EAS397_0017:4:84:1143:8692.
gcttgcaaaacgcaaacag---gaccatagtgggagat---
>gnl|SRA|SRR349645.16590846.1:2-36 HWI-EAS397_0017:4:82:16094:6498.
gcttgcaaaacgcaaacag---gaccatagtgggagat---
>gnl|SRA|SRR349645.14769148.1:6-40 HWI-EAS397_0017:4:73:10889:20022.
gcttgcaaaacgcaaacag---gaccatagtgggagat---
>gnl|SRA|SRR349645.13742105.1:4-38 HWI-EAS397_0017:4:68:7386:3748.
gcttgcaaaacgcaaacag---gaccatagtgggagat---
>_R_gnl|SRA|SRR349645.13494878.1:6-40 HWI-EAS397_0017:4:67:3270:3624.
gcttgcaaaacgcaaacag---gaccatagtgggagat---
>gnl|SRA|SRR349645.13313664.1:4-38 HWI-EAS397_0017:4:66:4978:19987.
gcttgcaaaacgcaaacag---gaccatagtgggagat---
>gnl|SRA|SRR349645.12116561.1:4-38 HWI-EAS397_0017:4:60:5479:14204.
gcttgcaaaacgcaaacag---gaccatagtgggagat---

>_R_gnl|SRA|SRR349645.11532187.1:6-40 HWI-EAS397_0017:4:57:7364:8594.
gcttgcaaaacgcaaacag---gaccatagtgggagat---
>_R_gnl|SRA|SRR349645.9299452.1:6-40 HWI-EAS397_0017:4:46:2044:6791.
gcttgcaaaacgcaaacag---gaccatagtgggagat---
>_R_gnl|SRA|SRR349645.6989770.1:3-37 HWI-EAS397_0017:4:34:9883:21409.
gcttgcaaaacgcaaacag---gaccatagtgggagat---
>gnl|SRA|SRR349645.3630112.1:6-40 HWI-EAS397_0017:4:18:9689:5242.
gcttgcaaaacgcaaacag---gaccatagtgggagat---
>_R_gnl|SRA|SRR349645.23437009.1:6-40 HWI-EAS397_0017:4:116:11463:9735.
gcgtgcaaaacgcaaacag---gaccatagtgggagat---
>gnl|SRA|SRR349645.23600463.1:1-34 HWI-EAS397_0017:4:117:7586:15952.
-cttgcaaaacgcaaacag---gaccatagtgggagat---
>_R_gnl|SRA|SRR349645.13753443.1:7-40 HWI-EAS397_0017:4:68:8390:15685.
-cttgcaaaacgcaaacag---gaccatagtgggagat---
>gnl|SRA|SRR349645.2295917.1:1-32 HWI-EAS397_0017:4:11:18636:12986.
---tgcaaaacgcaaacag---gaccatagtgggagat---
>gnl|SRA|SRR349645.23737426.1:1-31 HWI-EAS397_0017:4:118:1359:20350.
----gcaaaacgcaaacag---gaccatagtgggagat---
>gnl|SRA|SRR349645.23275908.1:1-31 HWI-EAS397_0017:4:115:13849:1426.
----gcaaaacgcaaacag---gaccatagtgggagat---
>_R_gnl|SRA|SRR349645.21436017.1:10-40 HWI-EAS397_0017:4:106:9764:1940.
----gcaaaacgcaaacag---gaccatagtgggagat---
>gnl|SRA|SRR349645.18544290.1:1-31 HWI-EAS397_0017:4:92:12365:18473.
----gcaaaacgcaaacag---gaccatagtgggagat---
>gnl|SRA|SRR349645.16098388.1:1-31 HWI-EAS397_0017:4:80:7221:1487.
----gcaaaacgcaaacag---gaccatagtgggagat---
>gnl|SRA|SRR349645.14502986.1:1-31 HWI-EAS397_0017:4:72:4647:13543.
----gcaaaacgcaaacag---gaccatagtgggagat---
>gnl|SRA|SRR349645.10252819.1:1-31 HWI-EAS397_0017:4:50:17202:15759.
----gcaaaacgcaaacag---gaccatagtgggagat---
>gnl|SRA|SRR349645.7991084.1:1-31 HWI-EAS397_0017:4:39:8468:19362.
----gcaaaacgcaaacag---gaccatagtgggagat---
>_R_gnl|SRA|SRR349645.3974842.1:10-40 HWI-EAS397_0017:4:20:3975:10006.
----gcaaaacgcaaacag---gaccatagtgggagat---
>gnl|SRA|SRR349645.2533805.1:1-31 HWI-EAS397_0017:4:13:3611:8406.
----gcaaaacgcaaacag---gaccatagtgggagat---
>gnl|SRA|SRR349645.2024113.1:1-31 HWI-EAS397_0017:4:10:12504:8249.
----gcaaaacgcaaacag---gaccatagtgggagat---
>_R_gnl|SRA|SRR349645.22867563.1:11-40 HWI-EAS397_0017:4:113:10572:8306.
-----caaaacgcaaacag---gaccatagtgggagat---
>_R_gnl|SRA|SRR349645.21736269.1:11-40 HWI-EAS397_0017:4:107:18320:4136.
-----caaaacgcaaacag---gaccatagtgggagat---
>gnl|SRA|SRR349645.21244535.1:1-30 HWI-EAS397_0017:4:105:11129:17085.
-----caaaacgcaaacag---gaccatagtgggagat---
>gnl|SRA|SRR349645.21223471.1:1-30 HWI-EAS397_0017:4:105:9351:13311.
-----caaaacgcaaacag---gaccatagtgggagat---
>_R_gnl|SRA|SRR349645.14434343.1:11-40 HWI-EAS397_0017:4:71:16039:2518.
-----caaaacgcaaacag---gaccatagtgggagat---
>gnl|SRA|SRR349645.12596170.1:1-30 HWI-EAS397_0017:4:62:12305:18940.
-----caaaacgcaaacag---gaccatagtgggagat---
>_R_gnl|SRA|SRR349645.11170935.1:11-40 HWI-EAS397_0017:4:55:10738:14548.

-----caaaacgcaaacag---gaccatagtgggagat---
>_R_gnl|SRA|SRR349645.10821533.1:11-40 HWI-EAS397_0017:4:53:15147:5344.
-----caaaacgcaaacag---gaccatagtgggagat---
>gnl|SRA|SRR349645.8409610.1:1-30 HWI-EAS397_0017:4:41:10744:6325.
-----caaaacgcaaacag---gaccatagtgggagat---
>gnl|SRA|SRR349645.5412726.1:1-30 HWI-EAS397_0017:4:26:18866:11787.
-----caaaacgcaaacag---gaccatagtgggagat---
>_R_gnl|SRA|SRR349645.3070841.1:11-40 HWI-EAS397_0017:4:15:14431:4982.
-----caaaacgcaaacag---gaccatagtgggagat---
>_R_gnl|SRA|SRR349645.2837586.1:11-40 HWI-EAS397_0017:4:14:11971:14144.
-----caaaacgcaaacag---gaccatagtgggagat---
>gnl|SRA|SRR349645.9551041.1:1-29 HWI-EAS397_0017:4:47:6898:18027.
------aaaacgcaaacag---gaccatagtgggagat---
>gnl|SRA|SRR349645.21105821.1:1-28 HWI-EAS397_0017:4:104:17040:1881.
-------aaacgcaaacag---gaccatagtgggagat---
>gnl|SRA|SRR349645.6558450.1:1-28 HWI-EAS397_0017:4:32:8500:6149.
-------aaacgcaaacag---gaccatagtgggagat---
>_R_gnl|SRA|SRR349645.21651852.1:15-40 HWI-EAS397_0017:4:107:10804:18482.
---------acgcaaacag---gaccatagtgggagat---
>_R_gnl|SRA|SRR349645.331058.1:15-40 HWI-EAS397_0017:4:2:10066:8337.
---------acgcaaacag---gaccatagtgggagat---
>gnl|SRA|SRR349645.20979157.1:1-25 HWI-EAS397_0017:4:104:6147:20765.
----------cgcaaacag---gaccatagtgggagat---
>gnl|SRA|SRR349645.18591710.1:1-25 HWI-EAS397_0017:4:92:16482:8722.
----------cgcaaacag---gaccatagtgggagat---
>gnl|SRA|SRR349645.17734238.1:1-25 HWI-EAS397_0017:4:88:12374:18695.
----------cgcaaacag---gaccatagtgggagat---
>gnl|SRA|SRR349645.17217073.1:1-25 HWI-EAS397_0017:4:86:1672:19183.
----------cgcaaacag---gaccatagtgggagat---
>gnl|SRA|SRR349645.12788350.1:1-25 HWI-EAS397_0017:4:63:11638:4041.
----------cgcaaacag---gaccatagtgggagat---
>gnl|SRA|SRR349645.12748713.1:1-25 HWI-EAS397_0017:4:63:8155:21085.
----------cgcaaacag---gaccatagtgggagat---
>gnl|SRA|SRR349645.12232219.1:1-25 HWI-EAS397_0017:4:60:15489:11392.
----------cgcaaacag---gaccatagtgggagat---
>gnl|SRA|SRR349645.9135116.1:1-25 HWI-EAS397_0017:4:45:4958:2620.
----------cgcaaacag---gaccatagtgggagat---
>gnl|SRA|SRR349645.8430923.1:1-25 HWI-EAS397_0017:4:41:12636:4861.
----------cgcaaacag---gaccatagtgggagat---
>gnl|SRA|SRR349645.8262954.1:1-25 HWI-EAS397_0017:4:40:15245:3294.
----------cgcaaacag---gaccatagtgggagat---
>gnl|SRA|SRR349645.8054038.1:1-25 HWI-EAS397_0017:4:39:14002:2663.
----------cgcaaacag---gaccatagtgggagat---
>gnl|SRA|SRR349645.7759203.1:1-25 HWI-EAS397_0017:4:38:5816:5150.
----------cgcaaacag---gaccatagtgggagat---
>gnl|SRA|SRR349645.7335171.1:1-25 HWI-EAS397_0017:4:36:4172:6970.
----------cgcaaacag---gaccatagtgggagat---
>gnl|SRA|SRR349645.5649153.1:1-25 HWI-EAS397_0017:4:28:3220:19249.
----------cgcaaacag---gaccatagtgggagat---
>gnl|SRA|SRR349645.4844100.1:1-25 HWI-EAS397_0017:4:24:6184:8704.
----------cgcaaacag---gaccatagtgggagat---

>gnl|SRA|SRR349645.4323068.1:1-25 HWI-EAS397_0017:4:21:15540:7096.
----------cgcaaacag---gaccatagtgggagat---
>gnl|SRA|SRR349645.3947443.1:1-25 HWI-EAS397_0017:4:20:1623:14965.
----------cgcaaacag---gaccatagtgggagat---
>gnl|SRA|SRR349645.3631251.1:1-25 HWI-EAS397_0017:4:18:9785:7451.
----------cgcaaacag---gaccatagtgggagat---
>gnl|SRA|SRR349645.3549908.1:1-25 HWI-EAS397_0017:4:18:2931:18206.
----------cgcaaacag---gaccatagtgggagat---
>gnl|SRA|SRR349645.3235093.1:1-25 HWI-EAS397_0017:4:16:11009:5933.
----------cgcaaacag---gaccatagtgggagat---
>gnl|SRA|SRR349645.1167617.1:1-25 HWI-EAS397_0017:4:6:9587:13006.
----------cgcaaacag---gaccatagtgggagat---
>gnl|SRA|SRR349645.18172224.1:1-24 HWI-EAS397_0017:4:90:15358:15795.
-----------gcaaacag---gaccatagtgggagat---
>gnl|SRA|SRR349645.17950621.1:1-24 HWI-EAS397_0017:4:89:13714:14624.
-----------gcaaacag---gaccatagtgggagat---
>gnl|SRA|SRR349645.16691449.1:1-24 HWI-EAS397_0017:4:83:7574:5829.
-----------gcaaacag---gaccatagtgggagat---
>gnl|SRA|SRR349645.8730920.1:1-24 HWI-EAS397_0017:4:43:4085:15076.
-----------gcaaacag---gaccatagtgggagat---
>gnl|SRA|SRR349645.5189490.1:1-24 HWI-EAS397_0017:4:25:17799:14275.
-----------gcaaacag---gaccatagtgggagat---
>_R_gnl|SRA|SRR349645.19011374.1:18-40 HWI-EAS397_0017:4:94:16757:6116.
---------------acagtaggaccatagtgggagat---
>_R_gnl|SRA|SRR349645.1232496.1:18-40 HWI-EAS397_0017:4:6:15060:11944.
---------------acagtaggaccatagtgggagat---
>_R_gnl|SRA|SRR349645.13281158.1:19-40 HWI-EAS397_0017:4:66:2067:5423.
----------------cagtaggaccatagtgggagat---

SRX058598 ZMM2, ZMM23
>contrZMM2 .
cagcttgcaaaacgcaaacactaggaacatagtgggagattc---
>contrZMM23 .
tagcttgcaaaacgcaaacagtaggaccatagtgggagattc---
>gnl|SRA|SRR189762.14576720.1:1-38 HWI-EAS397_0015:3:90:12625:8685.
----ttgcaaaacgcaaacactaggaacatagtgggagattc---
>gnl|SRA|SRR189762.7841199.1:1-38 HWI-EAS397_0015:3:49:1993:10054.
----ttgcaaaacgcaaacactaggaacatagtgggagattc---
>gnl|SRA|SRR189762.18447216.1:1-37 HWI-EAS397_0015:3:113:11994:14145.
-----tgcaaaacgcaaacactaggaacatagtgggagattc---
>_R_gnl|SRA|SRR189762.18177954.1:2-38 HWI-EAS397_0015:3:112:2268:5696.
-----tgcaaaacgcaaacactaggaacatagtgggagattc---
>_R_gnl|SRA|SRR189762.17294289.1:2-38 HWI-EAS397_0015:3:106:17302:6253.
-----tgcaaaacgcaaacactaggaacatagtgggagattc---
>_R_gnl|SRA|SRR189762.14363326.1:2-38 HWI-EAS397_0015:3:89:7471:12043.
-----tgcaaaacgcaaacactaggaacatagtgggagattc---
>_R_gnl|SRA|SRR189762.8047152.1:2-38 HWI-EAS397_0015:3:50:7480:19405.
-----tgcaaaacgcaaacactaggaacatagtgggagattc---
>_R_gnl|SRA|SRR189762.4034389.1:2-38 HWI-EAS397_0015:3:25:6694:6066.

```
-----tgcaaaacgcaaacactaggaacatagtgggagattc---
>_R_gnl|SRA|SRR189762.3510422.1:2-38 HWI-EAS397_0015:3:22:3068:1856.
-----tgcaaaacgcaaacactaggaacatagtgggagattc---
>_R_gnl|SRA|SRR189762.2565695.1:2-38 HWI-EAS397_0015:3:16:6419:15305.
-----tgcaaaacgcaaacactaggaacatagtgggagattc---
>_R_gnl|SRA|SRR189762.1653404.1:2-38 HWI-EAS397_0015:3:10:12766:13776.
-----tgcaaaacgcaaacactaggaacatagtgggagattc---
>_R_gnl|SRA|SRR189762.16101257.1:4-38 HWI-EAS397_0015:3:99:14528:16013.
-------caaaacgcaaacactaggaacatagtgggagattc---
>gnl|SRA|SRR189762.14666398.1:1-35 HWI-EAS397_0015:3:91:4921:1235.
-------caaaacgcaaacactaggaacatagtgggagattc---
>_R_gnl|SRA|SRR189762.14633624.1:4-38 HWI-EAS397_0015:3:91:1425:12955.
-------caaaacgcaaacactaggaacatagtgggagattc---
>gnl|SRA|SRR189762.14466409.1:1-35 HWI-EAS397_0015:3:89:18786:3880.
-------caaaacgcaaacactaggaacatagtgggagattc---
>gnl|SRA|SRR189762.10760144.1:1-35 HWI-EAS397_0015:3:67:14300:4069.
-------caaaacgcaaacactaggaacatagtgggagattc---
>gnl|SRA|SRR189762.10213686.1:1-35 HWI-EAS397_0015:3:64:6807:16018.
-------caaaacgcaaacactaggaacatagtgggagattc---
>gnl|SRA|SRR189762.9720783.1:1-35 HWI-EAS397_0015:3:61:4479:4113.
-------caaaacgcaaacactaggaacatagtgggagattc---
>gnl|SRA|SRR189762.19634686.1:1-36 HWI-EAS397_0015:3:120:5008:21021.
------gcaaaacgcaaacactaggaacatagtgggagattc---
>gnl|SRA|SRR189762.18929071.1:1-36 HWI-EAS397_0015:3:116:7283:4865.
------gcaaaacgcaaacactaggaacatagtgggagattc---
>gnl|SRA|SRR189762.18587273.1:1-36 HWI-EAS397_0015:3:114:8526:1915.
------gcaaaacgcaaacactaggaacatagtgggagattc---
>gnl|SRA|SRR189762.17883033.1:1-36 HWI-EAS397_0015:3:110:7312:8558.
------gcaaaacgcaaacactaggaacatagtgggagattc---
>gnl|SRA|SRR189762.17108652.1:1-36 HWI-EAS397_0015:3:105:15486:3810.
------gcaaaacgcaaacactaggaacatagtgggagattc---
>gnl|SRA|SRR189762.17095046.1:1-36 HWI-EAS397_0015:3:105:14030:3133.
------gcaaaacgcaaacactaggaacatagtgggagattc---
>gnl|SRA|SRR189762.16820530.1:1-36 HWI-EAS397_0015:3:104:3114:20688.
------gcaaaacgcaaacactaggaacatagtgggagattc---
>gnl|SRA|SRR189762.16009192.1:1-36 HWI-EAS397_0015:3:99:4974:19201.
------gcaaaacgcaaacactaggaacatagtgggagattc---
>gnl|SRA|SRR189762.15375557.1:1-36 HWI-EAS397_0015:3:95:9889:6780.
------gcaaaacgcaaacactaggaacatagtgggagattc---
>gnl|SRA|SRR189762.15301510.1:1-36 HWI-EAS397_0015:3:95:2229:11772.
------gcaaaacgcaaacactaggaacatagtgggagattc---
>gnl|SRA|SRR189762.14304123.1:1-36 HWI-EAS397_0015:3:89:1193:3882.
------gcaaaacgcaaacactaggaacatagtgggagattc---
>gnl|SRA|SRR189762.13447350.1:1-36 HWI-EAS397_0015:3:83:16665:2701.
------gcaaaacgcaaacactaggaacatagtgggagattc---
>_R_gnl|SRA|SRR189762.12393525.1:3-38 HWI-EAS397_0015:3:77:12288:10870.
------gcaaaacgcaaacactaggaacatagtgggagattc---
>gnl|SRA|SRR189762.11426231.1:1-36 HWI-EAS397_0015:3:71:15731:20697.
------gcaaaacgcaaacactaggaacatagtgggagattc---
>gnl|SRA|SRR189762.7773227.1:1-36 HWI-EAS397_0015:3:48:12348:3007.
------gcaaaacgcaaacactaggaacatagtgggagattc---
```

>gnl|SRA|SRR189762.6963494.1:1-36 HWI-EAS397_0015:3:43:11163:18159.
------gcaaaacgcaaacactaggaacatagtgggagattc---
>gnl|SRA|SRR189762.6432295.1:1-36 HWI-EAS397_0015:3:40:6536:7120.
------gcaaaacgcaaacactaggaacatagtgggagattc---
>gnl|SRA|SRR189762.3198483.1:1-36 HWI-EAS397_0015:3:20:4561:8398.
------gcaaaacgcaaacactaggaacatagtgggagattc---
>gnl|SRA|SRR189762.2344162.1:1-36 HWI-EAS397_0015:3:14:17446:6603.
------gcaaaacgcaaacactaggaacatagtgggagattc---
>gnl|SRA|SRR189762.1401384.1:1-36 HWI-EAS397_0015:3:9:3274:18256.
------gcaaaacgcaaacactaggaacatagtgggagattc---
>gnl|SRA|SRR189762.6863221.1:1-36 HWI-EAS397_0015:3:42:17508:8348.
------gcaaaacgcaaacagtaggaccatagtgggagattc---
>gnl|SRA|SRR189762.4578905.1:2-34 HWI-EAS397_0015:3:28:12777:19406.
---------aaacgcaaacactaggaacatagtgggagattc---
>gnl|SRA|SRR189762.11942394.1:1-34 HWI-EAS397_0015:3:74:17719:21079.
--------aaaacgcaaacactaggaacatagtgggagattc---
>gnl|SRA|SRR189762.6523374.1:1-34 HWI-EAS397_0015:3:40:15931:12204.
--------aaaacgcaaacactaggaacatagtgggagattc---
>gnl|SRA|SRR189762.6281999.1:1-34 HWI-EAS397_0015:3:39:7823:1504.
--------aaaacgcaaacagtaggaccatagtgggagattc---
>gnl|SRA|SRR189762.6971096.1:1-32 HWI-EAS397_0015:3:43:11932:12015.
----------aacgcaaacactaggaacatagtgggagattc---
>_R_gnl|SRA|SRR189762.17105148.1:9-38 HWI-EAS397_0015:3:105:15102:11964.
------------cgcaaacactaggaacatagtgggagattc---
>gnl|SRA|SRR189762.17098746.1:1-30 HWI-EAS397_0015:3:105:14418:9126.
------------cgcaaacactaggaacatagtgggagattc---
>gnl|SRA|SRR189762.11396861.1:1-30 HWI-EAS397_0015:3:71:12586:8655.
------------cgcaaacactaggaacatagtgggagattc---
>gnl|SRA|SRR189762.5741024.1:1-30 HWI-EAS397_0015:3:35:18533:10459.
------------cgcaaacactaggaacatagtgggagattc---
>gnl|SRA|SRR189762.3861998.1:1-30 HWI-EAS397_0015:3:24:5777:12095.
------------cgcaaacactaggaacatagtgggagattc---
>gnl|SRA|SRR189762.3310853.1:1-30 HWI-EAS397_0015:3:20:16426:18460.
------------cgcaaacactaggaacatagtgggagattc---
>_R_gnl|SRA|SRR189762.3184501.1:9-38 HWI-EAS397_0015:3:20:2888:17802.
------------cgcaaacactaggaacatagtgggagattc---
>gnl|SRA|SRR189762.370162.1:1-30 HWI-EAS397_0015:3:3:2787:19327.
------------cgcaaacactaggaacatagtgggagattc---
>_R_gnl|SRA|SRR189762.14902152.1:2-38 HWI-EAS397_0015:3:92:12655:17790.
--gcttgcaaaacgcaaacag---gaccatagtgggagattc---
>_R_gnl|SRA|SRR189762.10769339.1:2-38 HWI-EAS397_0015:3:67:15295:6569.
--gcttgcaaaacgcaaacag---gaccatagtgggagattc---
>_R_gnl|SRA|SRR189762.3124276.1:2-38 HWI-EAS397_0015:3:19:14016:14795.
--gcttgcaaaacgcaaacag---gaccatagtgggagattc---
>_R_gnl|SRA|SRR189762.17641214.1:2-38 HWI-EAS397_0015:3:108:17792:17096.
--gcttgcaaaacgcaaacac---gaacatagtgggagattc---
>_R_gnl|SRA|SRR189762.17124159.1:2-38 HWI-EAS397_0015:3:105:17183:18257.
--gcttgcaaaacgcaaacac---gaacatagtgggagattc---
>_R_gnl|SRA|SRR189762.12221646.1:2-38 HWI-EAS397_0015:3:76:11974:19582.
--gcttgcaaaacgcaaacac---gaacatagtgggagattc---
>_R_gnl|SRA|SRR189762.5195711.1:2-38 HWI-EAS397_0015:3:32:11655:2035.

--gcttgcaaaacgcaaacac---gaacatagtgggagattc---
>_R_gnl|SRA|SRR189762.4376049.1:2-38 HWI-EAS397_0015:3:27:8471:16758.
--gcttgcaaaacgcaaacac---gaacatagtgggagattc---
>gnl|SRA|SRR189762.5039406.1:1-36 HWI-EAS397_0015:3:31:11486:11030.
---cttgcaaaacgcaaacag---gaccatagtgggagattc---
>_R_gnl|SRA|SRR189762.16673396.1:4-38 HWI-EAS397_0015:3:103:4591:20477.
----ttgcaaaacgcaaacag---gaccatagtgggagattc---
>_R_gnl|SRA|SRR189762.13690055.1:4-38 HWI-EAS397_0015:3:85:6631:20558.
----ttgcaaaacgcaaacag---gaccatagtgggagattc---
>_R_gnl|SRA|SRR189762.900352.1:4-38 HWI-EAS397_0015:3:6:3889:16843.
----ttgcaaaacgcaaacag---gaccatagtgggagattc---
>gnl|SRA|SRR189762.16079302.1:1-33 HWI-EAS397_0015:3:99:12241:20396.
------gcaaaacgcaaacag---gaccatagtgggagattc---
>gnl|SRA|SRR189762.12455574.1:1-33 HWI-EAS397_0015:3:77:18826:20848.
------gcaaaacgcaaacag---gaccatagtgggagattc---
>gnl|SRA|SRR189762.5972529.1:1-33 HWI-EAS397_0015:3:37:9254:11417.
------gcaaaacgcaaacag---gaccatagtgggagattc---
>gnl|SRA|SRR189762.7728039.1:1-33 HWI-EAS397_0015:3:48:7539:9636.
------gcaaaacgcaaacag---gaccatagtgggacattc---
>gnl|SRA|SRR189762.15352218.1:1-33 HWI-EAS397_0015:3:95:7508:16042.
------gcaaaacgcaaacac---gaacatagtgggagattc---
>gnl|SRA|SRR189762.11631280.1:1-33 HWI-EAS397_0015:3:73:2248:15402.
------gcaaaacgcaaacac---gaacatagtgggagattc---
>gnl|SRA|SRR189762.6386528.1:1-33 HWI-EAS397_0015:3:39:18531:11562.
------gcaaaacgcaaacac---gaacatagtgggagattc---
>gnl|SRA|SRR189762.8384893.1:1-32 HWI-EAS397_0015:3:52:9933:1136.
-------caaaacgcaaacag---gaccatagtgggagattc---
>gnl|SRA|SRR189762.12939366.1:1-32 HWI-EAS397_0015:3:80:16567:18528.
-------caaaacgcaaacac---gaacatagtgggagattc---
>gnl|SRA|SRR189762.12492591.1:1-32 HWI-EAS397_0015:3:78:5050:2178.
-------caaaacgcaaacac---gaacatagtgggagattc---
>gnl|SRA|SRR189762.4378799.1:1-32 HWI-EAS397_0015:3:27:8762:19896.
-------caaaacgcaaacac---gaacatagtgggagattc---
>gnl|SRA|SRR189762.1072520.1:1-32 HWI-EAS397_0015:3:7:3947:10741.
-------caaaacgcaaacac---gaacatagtgggagattc---
>_R_gnl|SRA|SRR189762.12687397.1:8-38 HWI-EAS397_0015:3:79:7938:3781.
--------aaaacgcaaacag---gaccatagtgggagattc---
>gnl|SRA|SRR189762.11983367.1:1-31 HWI-EAS397_0015:3:75:4393:10837.
--------aaaacgcaaacag---gaccatagtgggagattc---
>gnl|SRA|SRR189762.3927523.1:1-31 HWI-EAS397_0015:3:24:12699:5938.
--------aaaacgcaaacag---gaccatagtgggagattc---
>gnl|SRA|SRR189762.13514278.1:1-31 HWI-EAS397_0015:3:84:6061:4690.
--------aaaacgcaaacac---gaacatagtgggagattc---
>gnl|SRA|SRR189762.5514890.1:1-31 HWI-EAS397_0015:3:34:11918:15772.
--------aaaacgcaaacac---gaacatagtgggagattc---
>gnl|SRA|SRR189762.5602012.1:1-27 HWI-EAS397_0015:3:35:3943:4282.
------------cgcaaacag---gaccatagtgggagattc---
>gnl|SRA|SRR189762.18319577.1:1-27 HWI-EAS397_0015:3:112:16740:16050.
------------cgcaaacac---gaacatagtgggagattc---
>gnl|SRA|SRR189762.13168148.1:1-27 HWI-EAS397_0015:3:82:5097:13323.
------------cgcaaacac---gaacatagtgggagattc---

>gnl|SRA|SRR189762.15706649.1:1-26 HWI-EAS397_0015:3:97:8940:15474.
-------------gcaaacac---gaacatagtgggagattc---
>gnl|SRA|SRR189762.8406150.1:1-25 HWI-EAS397_0015:3:52:12236:9228.
--------------caaacac---gaacatagtgggagattc---
>gnl|SRA|SRR189762.15685311.1:1-36 HWI-EAS397_0015:3:97:6671:5529.
---cttgcaaaacgcaaacac---gaacatagtggcagagtc---
>_R_gnl|SRA|SRR189762.18954789.1:11-38 HWI-EAS397_0015:3:116:9800:5584.
---cttgcaaaacgcaaacac---gaacatggtg-----------
>_R_gnl|SRA|SRR189762.7190950.1:1-38 HWI-EAS397_0015:3:44:17958:1721.
--gcttgcaaaacgcaaacactaggaacatagtgggagat-----
>_R_gnl|SRA|SRR189762.7190739.1:2-38 HWI-EAS397_0015:3:44:17938:1719.
--gcttgcaaaacgcaaacactaggaacatagtgggaga------
>_R_gnl|SRA|SRR189762.16102770.1:1-38 HWI-EAS397_0015:3:99:14689:20024.
cagcttgcaaaacgcaaacactaggaacatagtgggag-------
>_R_gnl|SRA|SRR189762.13993582.1:1-38 HWI-EAS397_0015:3:87:3334:20636.
cagcttgcaaaacgcaaacactaggaacatagtgggag-------
>_R_gnl|SRA|SRR189762.10454893.1:1-38 HWI-EAS397_0015:3:65:15908:4947.
cagcttgcaaaacgcaaacactaggaacatagtgggag-------
>_R_gnl|SRA|SRR189762.9751521.1:1-38 HWI-EAS397_0015:3:61:8002:13412.
cagcttgcaaaacgcaaacactaggaacatagtgggag-------
>_R_gnl|SRA|SRR189762.9681392.1:1-38 HWI-EAS397_0015:3:60:17803:2086.
cagcttgcaaaacgcaaacactaggaacatagtgggag-------
>_R_gnl|SRA|SRR189762.6725088.1:1-38 HWI-EAS397_0015:3:42:2801:13573.
cagcttgcaaaacgcaaacactaggaacatagtgggag-------
>_R_gnl|SRA|SRR189762.5999247.1:1-38 HWI-EAS397_0015:3:37:12013:12823.
cagcttgcaaaacgcaaacactaggaacatagtgggag-------
>_R_gnl|SRA|SRR189762.5989619.1:1-38 HWI-EAS397_0015:3:37:11039:4437.
cagcttgcaaaacgcaaacactaggaacatagtgggag-------
>gnl|SRA|SRR189762.5398005.1:1-38 HWI-EAS397_0015:3:33:16287:6703.
cagcttgcaaaacgcaaacactaggaacatagtgggag-------
>_R_gnl|SRA|SRR189762.4468579.1:1-38 HWI-EAS397_0015:3:27:17872:15852.
cagcttgcaaaacgcaaacactaggaacatagtgggag-------
>_R_gnl|SRA|SRR189762.2355167.1:1-38 HWI-EAS397_0015:3:14:18555:19651.
cagcttgcaaaacgcaaacactaggaacatagtgggag-------
>_R_gnl|SRA|SRR189762.2002623.1:1-38 HWI-EAS397_0015:3:12:15449:3488.
cagcttgcaaaacgcaaacactaggaacatagtgggag-------
>_R_gnl|SRA|SRR189762.1098297.1:1-38 HWI-EAS397_0015:3:7:6725:1984.
cagcttgcaaaacgcaaacactaggaacatagtgggag-------
>_R_gnl|SRA|SRR189762.16102770.1:1-37 HWI-EAS397_0015:3:99:14689:20024.
-agcttgcaaaacgcaaacactaggaacatagtgggag-------
>_R_gnl|SRA|SRR189762.13993582.1:1-37 HWI-EAS397_0015:3:87:3334:20636.
-agcttgcaaaacgcaaacactaggaacatagtgggag-------
>_R_gnl|SRA|SRR189762.10454893.1:1-37 HWI-EAS397_0015:3:65:15908:4947.
-agcttgcaaaacgcaaacactaggaacatagtgggag-------
>_R_gnl|SRA|SRR189762.9751521.1:1-37 HWI-EAS397_0015:3:61:8002:13412.
-agcttgcaaaacgcaaacactaggaacatagtgggag-------
>_R_gnl|SRA|SRR189762.9681392.1:1-37 HWI-EAS397_0015:3:60:17803:2086.
-agcttgcaaaacgcaaacactaggaacatagtgggag-------
>_R_gnl|SRA|SRR189762.6725088.1:1-37 HWI-EAS397_0015:3:42:2801:13573.
-agcttgcaaaacgcaaacactaggaacatagtgggag-------
>_R_gnl|SRA|SRR189762.5999247.1:1-37 HWI-EAS397_0015:3:37:12013:12823.

-agcttgcaaaacgcaaacactaggaacatagtgggag-------
>_R_gnl|SRA|SRR189762.5989619.1:1-37 HWI-EAS397_0015:3:37:11039:4437.
-agcttgcaaaacgcaaacactaggaacatagtgggag-------
>gnl|SRA|SRR189762.5398005.1:2-38 HWI-EAS397_0015:3:33:16287:6703.
-agcttgcaaaacgcaaacactaggaacatagtgggag-------
>_R_gnl|SRA|SRR189762.4468579.1:1-37 HWI-EAS397_0015:3:27:17872:15852.
-agcttgcaaaacgcaaacactaggaacatagtgggag-------
>_R_gnl|SRA|SRR189762.2355167.1:1-37 HWI-EAS397_0015:3:14:18555:19651.
-agcttgcaaaacgcaaacactaggaacatagtgggag-------
>_R_gnl|SRA|SRR189762.2002623.1:1-37 HWI-EAS397_0015:3:12:15449:3488.
-agcttgcaaaacgcaaacactaggaacatagtgggag-------
>_R_gnl|SRA|SRR189762.1098297.1:1-37 HWI-EAS397_0015:3:7:6725:1984.
-agcttgcaaaacgcaaacactaggaacatagtgggag-------
>_R_gnl|SRA|SRR189762.4643474.1:1-38 HWI-EAS397_0015:3:29:2018:18654.
-agcttgcaaaacgcaaacactaggaacatagtgggaga------
>_R_gnl|SRA|SRR189762.4263469.1:1-38 HWI-EAS397_0015:3:26:13607:2173.
-agcttgcaaaacgcaaacactaggaacatagtgggaga------
>_R_gnl|SRA|SRR189762.11644237.1:1-36 HWI-EAS397_0015:3:73:3670:15997.
-agcttgcaaaacgcaaacactaggaacatagtggga--------
>_R_gnl|SRA|SRR189762.4302292.1:1-36 HWI-EAS397_0015:3:26:17567:11612.
-agcttgcaaaacgcaaacactaggaacatagtggga--------
>gnl|SRA|SRR189762.19552681.1:4-38 HWI-EAS397_0015:3:119:14727:19921.
-agcttgcaaaacgcaaacactaggaacatagtggg---------
>gnl|SRA|SRR189762.11350704.1:4-38 HWI-EAS397_0015:3:71:7645:5957.
-agcttgcaaaacgcaaacactaggaacatagtggg---------
>_R_gnl|SRA|SRR189762.11644237.1:1-37 HWI-EAS397_0015:3:73:3670:15997.
cagcttgcaaaacgcaaacactaggaacatagtggga--------
>_R_gnl|SRA|SRR189762.4302292.1:1-37 HWI-EAS397_0015:3:26:17567:11612.
cagcttgcaaaacgcaaacactaggaacatagtggga--------
>gnl|SRA|SRR189762.19552681.1:3-38 HWI-EAS397_0015:3:119:14727:19921.
cagcttgcaaaacgcaaacactaggaacatagtggg---------
>gnl|SRA|SRR189762.11350704.1:3-38 HWI-EAS397_0015:3:71:7645:5957.
cagcttgcaaaacgcaaacactaggaacatagtggg---------
>_R_gnl|SRA|SRR189762.18031688.1:1-35 HWI-EAS397_0015:3:111:4923:5727.
cagcttgcaaaacgcaaacactaggaacatagtgg----------
>_R_gnl|SRA|SRR189762.16474456.1:1-35 HWI-EAS397_0015:3:102:1113:5763.
cagcttgcaaaacgcaaacactaggaacatagtgg----------
>_R_gnl|SRA|SRR189762.16200592.1:1-35 HWI-EAS397_0015:3:100:7376:2818.
cagcttgcaaaacgcaaacactaggaacatagtgg----------
>_R_gnl|SRA|SRR189762.12364043.1:1-35 HWI-EAS397_0015:3:77:9223:12816.
cagcttgcaaaacgcaaacactaggaacatagtgg----------
>_R_gnl|SRA|SRR189762.9617689.1:1-35 HWI-EAS397_0015:3:60:10975:20237.
cagcttgcaaaacgcaaacactaggaacatagtgg----------
>gnl|SRA|SRR189762.8649506.1:4-38 HWI-EAS397_0015:3:54:4470:12518.
cagcttgcaaaacgcaaacactaggaacatagtgg----------
>gnl|SRA|SRR189762.5062528.1:4-38 HWI-EAS397_0015:3:31:13821:11876.
cagcttgcaaaacgcaaacactaggaacatagtgg----------
>_R_gnl|SRA|SRR189762.4235174.1:1-35 HWI-EAS397_0015:3:26:10701:4465.
cagcttgcaaaacgcaaacactaggaacatagtgg----------
>_R_gnl|SRA|SRR189762.2639464.1:1-35 HWI-EAS397_0015:3:16:14142:16647.
cagcttgcaaaacgcaaacactaggaacatagtgg----------

>_R_gnl|SRA|SRR189762.14763382.1:1-35 HWI-EAS397_0015:3:91:15225:13728.
cagcttgcaaaacgcaaacactaggagcatagtgg----------
>_R_gnl|SRA|SRR189762.18031688.1:1-34 HWI-EAS397_0015:3:111:4923:5727.
-agcttgcaaaacgcaaacactaggaacatagtgg----------
>_R_gnl|SRA|SRR189762.16474456.1:1-34 HWI-EAS397_0015:3:102:1113:5763.
-agcttgcaaaacgcaaacactaggaacatagtgg----------
>_R_gnl|SRA|SRR189762.16200592.1:1-34 HWI-EAS397_0015:3:100:7376:2818.
-agcttgcaaaacgcaaacactaggaacatagtgg----------
>_R_gnl|SRA|SRR189762.12364043.1:1-34 HWI-EAS397_0015:3:77:9223:12816.
-agcttgcaaaacgcaaacactaggaacatagtgg----------
>_R_gnl|SRA|SRR189762.9617689.1:1-34 HWI-EAS397_0015:3:60:10975:20237.
-agcttgcaaaacgcaaacactaggaacatagtgg----------
>gnl|SRA|SRR189762.8649506.1:5-38 HWI-EAS397_0015:3:54:4470:12518.
-agcttgcaaaacgcaaacactaggaacatagtgg----------
>gnl|SRA|SRR189762.5062528.1:5-38 HWI-EAS397_0015:3:31:13821:11876.
-agcttgcaaaacgcaaacactaggaacatagtgg----------
>_R_gnl|SRA|SRR189762.4235174.1:1-34 HWI-EAS397_0015:3:26:10701:4465.
-agcttgcaaaacgcaaacactaggaacatagtgg----------
>_R_gnl|SRA|SRR189762.2639464.1:1-34 HWI-EAS397_0015:3:16:14142:16647.
-agcttgcaaaacgcaaacactaggaacatagtgg----------
>_R_gnl|SRA|SRR189762.14763382.1:1-34 HWI-EAS397_0015:3:91:15225:13728.
-agcttgcaaaacgcaaacactaggagcatagtgg----------
>_R_gnl|SRA|SRR189762.18693940.1:1-32 HWI-EAS397_0015:3:115:1547:12246.
cagcttgcaaaacgcaaacactaggaacatag-------------
>_R_gnl|SRA|SRR189762.12506607.1:1-32 HWI-EAS397_0015:3:78:6560:15535.
cagcttgcaaaacgcaaacactaggaacatag-------------
>_R_gnl|SRA|SRR189762.9980601.1:1-32 HWI-EAS397_0015:3:62:16316:17794.
cagcttgcaaaacgcaaacactaggaacatag-------------
>_R_gnl|SRA|SRR189762.9230971.1:1-32 HWI-EAS397_0015:3:57:18463:11319.
cagcttgcaaaacgcaaacactaggaacatag-------------
>_R_gnl|SRA|SRR189762.7600820.1:1-32 HWI-EAS397_0015:3:47:10952:4905.
cagcttgcaaaacgcaaacactaggaacatag-------------
>_R_gnl|SRA|SRR189762.3676453.1:1-32 HWI-EAS397_0015:3:23:3341:10271.
cagcttgcaaaacgcaaacactaggaacatag-------------
>_R_gnl|SRA|SRR189762.18693940.1:1-31 HWI-EAS397_0015:3:115:1547:12246.
-agcttgcaaaacgcaaacactaggaacatag-------------
>_R_gnl|SRA|SRR189762.12506607.1:1-31 HWI-EAS397_0015:3:78:6560:15535.
-agcttgcaaaacgcaaacactaggaacatag-------------
>_R_gnl|SRA|SRR189762.9980601.1:1-31 HWI-EAS397_0015:3:62:16316:17794.
-agcttgcaaaacgcaaacactaggaacatag-------------
>_R_gnl|SRA|SRR189762.9230971.1:1-31 HWI-EAS397_0015:3:57:18463:11319.
-agcttgcaaaacgcaaacactaggaacatag-------------
>_R_gnl|SRA|SRR189762.7600820.1:1-31 HWI-EAS397_0015:3:47:10952:4905.
-agcttgcaaaacgcaaacactaggaacatag-------------
>_R_gnl|SRA|SRR189762.3676453.1:1-31 HWI-EAS397_0015:3:23:3341:10271.
-agcttgcaaaacgcaaacactaggaacatag-------------
>gnl|SRA|SRR189762.9659516.1:8-38 HWI-EAS397_0015:3:60:15502:13202.
cagcttgcaaaacgcaaacactaggaacata--------------
>gnl|SRA|SRR189762.9641127.1:8-38 HWI-EAS397_0015:3:60:13519:11014.
cagcttgcaaaacgcaaacactaggaacata--------------
>_R_gnl|SRA|SRR189762.3868140.1:1-31 HWI-EAS397_0015:3:24:6474:16023.

cagcttgcaaaacgcaaacactaggaacata--------------
>gnl|SRA|SRR189762.9659516.1:9-38 HWI-EAS397_0015:3:60:15502:13202.
-agcttgcaaaacgcaaacactaggaacata--------------
>gnl|SRA|SRR189762.9641127.1:9-38 HWI-EAS397_0015:3:60:13519:11014.
-agcttgcaaaacgcaaacactaggaacata--------------
>_R_gnl|SRA|SRR189762.3868140.1:1-30 HWI-EAS397_0015:3:24:6474:16023.
-agcttgcaaaacgcaaacactaggaacata--------------
>gnl|SRA|SRR189762.12355591.1:9-38 HWI-EAS397_0015:3:77:8341:11288.
cagcttgcaaaacgcaaacactaggaacat---------------
>gnl|SRA|SRR189762.9318884.1:9-38 HWI-EAS397_0015:3:58:11336:18149.
cagcttgcaaaacgcaaacactaggaacat---------------
>gnl|SRA|SRR189762.3842574.1:9-38 HWI-EAS397_0015:3:24:3523:18010.
cagcttgcaaaacgcaaacactaggaacat---------------
>_R_gnl|SRA|SRR189762.2746508.1:2-31 HWI-EAS397_0015:3:17:8452:7009.
cagcttgcaaaacgcaaacactaggaacat---------------
>gnl|SRA|SRR189762.12355591.1:10-38 HWI-EAS397_0015:3:77:8341:11288.
-agcttgcaaaacgcaaacactaggaacat---------------
>gnl|SRA|SRR189762.9318884.1:10-38 HWI-EAS397_0015:3:58:11336:18149.
-agcttgcaaaacgcaaacactaggaacat---------------
>gnl|SRA|SRR189762.3842574.1:10-38 HWI-EAS397_0015:3:24:3523:18010.
-agcttgcaaaacgcaaacactaggaacat---------------
>_R_gnl|SRA|SRR189762.2746508.1:2-30 HWI-EAS397_0015:3:17:8452:7009.
-agcttgcaaaacgcaaacactaggaacat---------------
>gnl|SRA|SRR189762.3294379.1:10-38 HWI-EAS397_0015:3:20:14731:5197.
cagcttgcaaaacgcaaacactaggaaca----------------
>gnl|SRA|SRR189762.2967556.1:10-38 HWI-EAS397_0015:3:18:14612:19080.
tagcttgcaaaacgcaaacagtaggacca----------------
>gnl|SRA|SRR189762.8995709.1:3-38 HWI-EAS397_0015:3:56:8649:7612.
cagcttgcaaaacgcaaacac---gaacatagtgggaga------
>_R_gnl|SRA|SRR189762.4597652.1:1-36 HWI-EAS397_0015:3:28:14648:4583.
cagcttgcaaaacgcaaacac---gaacatagtgggaga------
>_R_gnl|SRA|SRR189762.9163245.1:1-35 HWI-EAS397_0015:3:57:10853:19355.
cagcttgcaaaacgcaaacac---gaacatagtgggag-------
>gnl|SRA|SRR189762.6731989.1:4-38 HWI-EAS397_0015:3:42:3676:14975.
cagcttgcaaaacgcaaacac---gaacatagtgggag-------
>_R_gnl|SRA|SRR189762.4636610.1:1-35 HWI-EAS397_0015:3:28:18623:7556.
cagcttgcaaaacgcaaacac---gaacatagtgggag-------
>_R_gnl|SRA|SRR189762.9163245.1:1-34 HWI-EAS397_0015:3:57:10853:19355.
-agcttgcaaaacgcaaacac---gaacatagtgggag-------
>gnl|SRA|SRR189762.6731989.1:5-38 HWI-EAS397_0015:3:42:3676:14975.
-agcttgcaaaacgcaaacac---gaacatagtgggag-------
>_R_gnl|SRA|SRR189762.4636610.1:1-34 HWI-EAS397_0015:3:28:18623:7556.
-agcttgcaaaacgcaaacac---gaacatagtgggag-------
>gnl|SRA|SRR189762.16698934.1:1-38 HWI-EAS397_0015:3:103:7385:10003.
---cttgcaaaacgcaaacactaggaacatagtgggagatt----
>gnl|SRA|SRR189762.9178660.1:1-38 HWI-EAS397_0015:3:57:12637:20570.
---cttgcaaaacgcaaacactaggaacatagtgggagatt----
>gnl|SRA|SRR189762.6760182.1:1-38 HWI-EAS397_0015:3:42:6855:8447.
---cttgcaaaacgcaaacactaggaacatagtgggagatt----
>_R_gnl|SRA|SRR189762.11472555.1:1-34 HWI-EAS397_0015:3:72:3002:12866.
tagcttgcaaaacgcaaacag---gaccatagtggga--------

>_R_gnl|SRA|SRR189762.5713477.1:1-34 HWI-EAS397_0015:3:35:15708:5339.
tagcttgcaaaacgcaaacag---gaccatagtggga--------
>_R_gnl|SRA|SRR189762.17931074.1:1-35 HWI-EAS397_0015:3:110:12177:9100.
tagcttgcaaaacgcaaacag---gaccatagtgggag-------
>gnl|SRA|SRR189762.2535213.1:4-38 HWI-EAS397_0015:3:16:2713:3093.
tagcttgccaaacgcaaacag---gaccatagtgggag-------
>gnl|SRA|SRR189762.3783963.1:6-38 HWI-EAS397_0015:3:23:14764:18099.
tagcttgcaaaacgcaaacag---gaccatagtggg---------
>gnl|SRA|SRR189762.18910998.1:7-38 HWI-EAS397_0015:3:116:5475:8623.
tagcttgcaaaacgcaaacag---gaccatagtgg----------
>gnl|SRA|SRR189762.5772669.1:7-38 HWI-EAS397_0015:3:36:5060:18880.
tagcttgcaaaacgcaaacag---gaccatagtgg----------
>gnl|SRA|SRR189762.128846.1:7-38 HWI-EAS397_0015:3:1:14097:2179.
tagcttgcaaaacgcaaacag---gaccatagtgg----------
>_R_gnl|SRA|SRR189762.15131859.1:1-31 HWI-EAS397_0015:3:94:2052:18634.
tagcttgcaaaacgcaaacag---gaccatagtg-----------
>_R_gnl|SRA|SRR189762.10103832.1:1-31 HWI-EAS397_0015:3:63:12353:20896.
tagcttgcaaaacgcaaacag---gaccatagtg-----------
>gnl|SRA|SRR189762.18027780.1:9-38 HWI-EAS397_0015:3:111:4517:12580.
tagcttgcaaaacgcaaacag---gaccatagt------------
>gnl|SRA|SRR189762.16533809.1:9-38 HWI-EAS397_0015:3:102:7459:14738.
tagcttgcaaaacgcaaacag---gaccatagt------------
>gnl|SRA|SRR189762.11595077.1:9-38 HWI-EAS397_0015:3:72:16146:4413.
tagcttgcaaaacgcaaacag---gaccatagt------------
>gnl|SRA|SRR189762.5853270.1:9-38 HWI-EAS397_0015:3:36:13523:4244.
tagcttgcaaaacgcaaacag---gaccatagt------------
>_R_gnl|SRA|SRR189762.9473470.1:1-29 HWI-EAS397_0015:3:59:11792:12930.
tagcttgcaaaacgcaaacag---gaccatag-------------
>gnl|SRA|SRR189762.13582472.1:11-38 HWI-EAS397_0015:3:84:13066:10742.
tagcttgcaaaacgcaaacag---gaccata--------------
>_R_gnl|SRA|SRR189762.17931074.1:1-34 HWI-EAS397_0015:3:110:12177:9100.
-agcttgcaaaacgcaaacag---gaccatagtgggag-------
>gnl|SRA|SRR189762.2535213.1:5-38 HWI-EAS397_0015:3:16:2713:3093.
-agcttgccaaacgcaaacag---gaccatagtgggag-------
>_R_gnl|SRA|SRR189762.11472555.1:1-33 HWI-EAS397_0015:3:72:3002:12866.
-agcttgcaaaacgcaaacag---gaccatagtggga--------
>_R_gnl|SRA|SRR189762.5713477.1:1-33 HWI-EAS397_0015:3:35:15708:5339.
-agcttgcaaaacgcaaacag---gaccatagtggga--------
>gnl|SRA|SRR189762.3783963.1:7-38 HWI-EAS397_0015:3:23:14764:18099.
-agcttgcaaaacgcaaacag---gaccatagtggg---------
>gnl|SRA|SRR189762.18910998.1:8-38 HWI-EAS397_0015:3:116:5475:8623.
-agcttgcaaaacgcaaacag---gaccatagtgg----------
>gnl|SRA|SRR189762.5772669.1:8-38 HWI-EAS397_0015:3:36:5060:18880.
-agcttgcaaaacgcaaacag---gaccatagtgg----------
>gnl|SRA|SRR189762.128846.1:8-38 HWI-EAS397_0015:3:1:14097:2179.
-agcttgcaaaacgcaaacag---gaccatagtgg----------
>_R_gnl|SRA|SRR189762.15131859.1:1-30 HWI-EAS397_0015:3:94:2052:18634.
-agcttgcaaaacgcaaacag---gaccatagtg-----------
>_R_gnl|SRA|SRR189762.10103832.1:1-30 HWI-EAS397_0015:3:63:12353:20896.
-agcttgcaaaacgcaaacag---gaccatagtg-----------
>gnl|SRA|SRR189762.18027780.1:10-38 HWI-EAS397_0015:3:111:4517:12580.

-agcttgcaaaacgcaaacag---gaccatagt------------
>gnl|SRA|SRR189762.16533809.1:10-38 HWI-EAS397_0015:3:102:7459:14738.
-agcttgcaaaacgcaaacag---gaccatagt------------
>gnl|SRA|SRR189762.11595077.1:10-38 HWI-EAS397_0015:3:72:16146:4413.
-agcttgcaaaacgcaaacag---gaccatagt------------
>gnl|SRA|SRR189762.5853270.1:10-38 HWI-EAS397_0015:3:36:13523:4244.
-agcttgcaaaacgcaaacag---gaccatagt------------
>_R_gnl|SRA|SRR189762.9473470.1:1-28 HWI-EAS397_0015:3:59:11792:12930.
-agcttgcaaaacgcaaacag---gaccatag-------------
>_R_gnl|SRA|SRR189762.17327038.1:9-38 HWI-EAS397_0015:3:107:3086:5987.
----ttgcaaaacgcaaacactaggaacatggtg-----------

SRX058600 ZMM2

>contrZMM2 .
cagcttgcaaaacgcaaacactaggaacatagtgggagattc--
>_R_gnl|SRA|SRR393016.17670278.1:1-38 HWI-EAS397_0015:6:115:7656:12048.
cagcttgcaaaacgcaaacactaggaacatagtgggag------
>gnl|SRA|SRR393016.17395933.1:1-38 HWI-EAS397_0015:6:113:11721:7601.
cagcttgcaaaacgcaaacactaggaacatagtgggag------
>_R_gnl|SRA|SRR393016.14502039.1:1-38 HWI-EAS397_0015:6:95:16651:2979.
cagcttgcaaaacgcaaacactaggaacatagtgggag------
>_R_gnl|SRA|SRR393016.9795169.1:1-38 HWI-EAS397_0015:6:66:3130:9169.
cagcttgcaaaacgcaaacactaggaacatagtgggag------
>_R_gnl|SRA|SRR393016.4204405.1:1-38 HWI-EAS397_0015:6:28:5830:10923.
cagcttgcaaaacgcaaacactaggaacatagtgggag------
>_R_gnl|SRA|SRR393016.14208964.1:1-37 HWI-EAS397_0015:6:94:2304:18726.
cagcttgcaaaacgcaaacactaggaacatagtggga-------
>_R_gnl|SRA|SRR393016.13132953.1:1-37 HWI-EAS397_0015:6:87:7799:9394.
cagcttgcaaaacgcaaacactaggaacatagtggga-------
>_R_gnl|SRA|SRR393016.13614939.1:1-36 HWI-EAS397_0015:6:90:7858:4819.
cagcttgcaaaacgcaaacactaggaacatagtggg--------
>_R_gnl|SRA|SRR393016.1152116.1:1-36 HWI-EAS397_0015:6:8:8632:3300.
cagcttgcaaaacgcaaacactaggaacatagtggg--------
>_R_gnl|SRA|SRR393016.16785507.1:1-35 HWI-EAS397_0015:6:109:14352:8818.
cagcttgcaaaacgcaaacactaggaacatagtgg---------
>_R_gnl|SRA|SRR393016.13427738.1:1-35 HWI-EAS397_0015:6:89:4911:4122.
cagcttgcaaaacgcaaacactaggaacatagtgg---------
>_R_gnl|SRA|SRR393016.7715491.1:1-35 HWI-EAS397_0015:6:52:5584:3561.
cagcttgcaaaacgcaaacactaggaacatagtgg---------
>_R_gnl|SRA|SRR393016.4926585.1:1-35 HWI-EAS397_0015:6:33:7601:20931.
cagcttgcaaaacgcaaacactaggaacatagtgg---------
>_R_gnl|SRA|SRR393016.4098055.1:1-35 HWI-EAS397_0015:6:27:10626:8311.
cagcttgcaaaacgcaaacactaggaacatagtgg---------
>gnl|SRA|SRR393016.2354721.1:4-38 HWI-EAS397_0015:6:16:5230:9519.
cagcttgcaaaacgcaaacactaggaacatagtgg---------
>gnl|SRA|SRR393016.13230869.1:5-38 HWI-EAS397_0015:6:87:18796:9831.
cagcttgcaaaacgcaaacactaggaacatagtg----------

>_R_gnl|SRA|SRR393016.10633817.1:1-34 HWI-EAS397_0015:6:71:10788:11077.
cagcttgcaaaacgcaaacactaggaacatagtg----------
>gnl|SRA|SRR393016.12836800.1:6-38 HWI-EAS397_0015:6:85:10535:7439.
cagcttgcaaaacgcaaacactaggaacatagt-----------
>_R_gnl|SRA|SRR393016.9280546.1:1-32 HWI-EAS397_0015:6:62:14917:11240.
cagcttgcaaaacgcaaacactaggaacatag------------
>_R_gnl|SRA|SRR393016.3733541.1:1-32 HWI-EAS397_0015:6:25:2468:5290.
cagcttgcaaaacggaaacactaggaacatag------------
>_R_gnl|SRA|SRR393016.14092747.1:1-31 HWI-EAS397_0015:6:93:7319:12901.
cagcttgcaaaacgcaaacactaggaacata-------------
>gnl|SRA|SRR393016.3322761.1:9-38 HWI-EAS397_0015:6:22:8725:18162.
cagcttgcaaaacgcaaacactaggaacat--------------
>gnl|SRA|SRR393016.2042269.1:9-38 HWI-EAS397_0015:6:14:5901:12114.
cagcttgcaaaacgcaaacactaggaacat--------------
>gnl|SRA|SRR393016.16302695.1:11-38 HWI-EAS397_0015:6:106:13459:7709.
cagcttgcaaaacgcaaacactaggaac----------------
>gnl|SRA|SRR393016.12695529.1:11-38 HWI-EAS397_0015:6:84:12505:20556.
cagcttgcaaaacgcaaacactaggaac----------------
>_R_gnl|SRA|SRR393016.8602105.1:1-28 HWI-EAS397_0015:6:58:5845:10416.
cagcttgcaaaacgcaaacactaggaac----------------
>gnl|SRA|SRR393016.6993393.1:11-38 HWI-EAS397_0015:6:47:7158:2193.
cagcttgcaaaacgcaaacactaggaac----------------
>_R_gnl|SRA|SRR393016.2333016.1:1-27 HWI-EAS397_0015:6:16:2618:10513.
cagcttgcaaaacgcaaacactaggaa-----------------
>_R_gnl|SRA|SRR393016.17722735.1:1-25 HWI-EAS397_0015:6:115:13519:20326.
cagcttgcaaaacgcaaacactagg-------------------
>_R_gnl|SRA|SRR393016.10722541.1:1-25 HWI-EAS397_0015:6:72:3310:19854.
cagcttgcaaaacgcaaacactagg-------------------
>gnl|SRA|SRR393016.8534592.1:14-38 HWI-EAS397_0015:6:57:14690:18439.
cagcttgcaaaacgcaaacactagg-------------------
>gnl|SRA|SRR393016.3571127.1:14-38 HWI-EAS397_0015:6:23:18662:1254.
cagcttgcaaaacgcaaacactagg-------------------
>gnl|SRA|SRR393016.3048956.1:14-38 HWI-EAS397_0015:6:20:12336:15652.
cagcttgcaaaacgcaaacactagg-------------------
>gnl|SRA|SRR393016.9615528.1:15-38 HWI-EAS397_0015:6:64:18030:4052.
cagcttgcaaaacgcaaacactag--------------------
>gnl|SRA|SRR393016.9582101.1:15-38 HWI-EAS397_0015:6:64:14110:7320.
cagcttgcaaaacgcaaacactag--------------------
>_R_gnl|SRA|SRR393016.6778293.1:1-24 HWI-EAS397_0015:6:45:16090:4917.
cagcttgcaaaacgcaaacactag--------------------
>_R_gnl|SRA|SRR393016.4269760.1:1-24 HWI-EAS397_0015:6:28:12990:12012.
cagcttgcaaaacgcaaacactag--------------------
>gnl|SRA|SRR393016.4161737.1:15-38 HWI-EAS397_0015:6:27:17424:21234.
cagcttgcaaaacgcaaacactag--------------------
>gnl|SRA|SRR393016.3923490.1:15-38 HWI-EAS397_0015:6:26:7511:6223.
cagcttgcaaaacgcaaacactag--------------------
>_R_gnl|SRA|SRR393016.2826973.1:1-24 HWI-EAS397_0015:6:19:4833:15439.
cagcttgcaaaacgcaaacactag--------------------
>gnl|SRA|SRR393016.595960.1:15-38 HWI-EAS397_0015:6:4:15281:14212.
cagcttgcaaaacgcaaacactag--------------------
>_R_gnl|SRA|SRR393016.181821.1:1-24 HWI-EAS397_0015:6:2:4167:10507.

cagcttgcaaaacgcaaacactag--------------------
>gnl|SRA|SRR393016.15190163.1:17-38 HWI-EAS397_0015:6:100:1850:1228.
cagcttgcaaaacgcaaacact----------------------
>gnl|SRA|SRR393016.11427525.1:17-38 HWI-EAS397_0015:6:76:12652:16527.
cagcttgcaaaacgcaaacact----------------------
>gnl|SRA|SRR393016.8418739.1:17-38 HWI-EAS397_0015:6:56:18207:15675.
cagcttgcaaaacgcaaacact----------------------
>_R_gnl|SRA|SRR393016.15423426.1:5-25 HWI-EAS397_0015:6:101:8762:6831.
cagcttgcaaaacgcaaacac-----------------------
>_R_gnl|SRA|SRR393016.13751536.1:5-25 HWI-EAS397_0015:6:91:5347:9132.
cagcttgcaaaacgcaaacac-----------------------
>gnl|SRA|SRR393016.11666427.1:15-35 HWI-EAS397_0015:6:78:4134:1725.
cagcttgcaaaacgcaaacac-----------------------
>gnl|SRA|SRR393016.10556228.1:18-38 HWI-EAS397_0015:6:71:1902:4357.
cagcttgcaaaacgcaaacac-----------------------
>_R_gnl|SRA|SRR393016.9753378.1:5-25 HWI-EAS397_0015:6:65:16046:16226.
cagcttgcaaaacgcaaacac-----------------------
>gnl|SRA|SRR393016.9570560.1:18-38 HWI-EAS397_0015:6:64:12784:7647.
cagcttgcaaaacgcaaacac-----------------------
>_R_gnl|SRA|SRR393016.4635465.1:5-25 HWI-EAS397_0015:6:31:6500:20853.
cagcttgcaaaacgcaaacac-----------------------
>gnl|SRA|SRR393016.725362.1:18-38 HWI-EAS397_0015:6:5:12544:16208.
cagcttgcaaaacgcaaacac-----------------------
>_R_gnl|SRA|SRR393016.18361777.1:1-35 HWI-EAS397_0015:6:119:14972:1577.
cagcttgcaaaacgcaaacac---gaacatagtgggag------
>_R_gnl|SRA|SRR393016.13773964.1:1-35 HWI-EAS397_0015:6:91:7770:19856.
cagcttgcaaaacgcaaacac---gaacatagtgggag------
>_R_gnl|SRA|SRR393016.10160399.1:1-35 HWI-EAS397_0015:6:68:9754:2409.
cagcttgcaaaacgcaaacac---gaacatagtgggag------
>_R_gnl|SRA|SRR393016.4405832.1:1-35 HWI-EAS397_0015:6:29:12211:15584.
cagcttgcaaaacgcaaatac---gaacatagtgggag------
>_R_gnl|SRA|SRR393016.2254844.1:1-34 HWI-EAS397_0015:6:15:11735:7206.
cagcttgcaaaacgcaaacac---gaacatagtggga-------
>_R_gnl|SRA|SRR393016.4771682.1:1-33 HWI-EAS397_0015:6:32:6036:16559.
cagcttgcaaaacgcaaacac---gaacatagtggg--------
>_R_gnl|SRA|SRR393016.17663582.1:1-32 HWI-EAS397_0015:6:115:6915:12126.
cagcttgcaaaacgcaaacac---gaacatagtgg---------
>_R_gnl|SRA|SRR393016.13798889.1:1-32 HWI-EAS397_0015:6:91:10460:1049.
cagcttgcaaaacgcaaacac---gaacatagtgg---------
>_R_gnl|SRA|SRR393016.9494800.1:1-32 HWI-EAS397_0015:6:64:4108:18094.
cagcttgcaaaacgcaaacac---gaacatagtgg---------
>gnl|SRA|SRR393016.6156144.1:7-38 HWI-EAS397_0015:6:41:13692:12011.
cagcttgcaaaacgcaaacac---gaacatagtgg---------
>_R_gnl|SRA|SRR393016.4604036.1:1-32 HWI-EAS397_0015:6:30:18423:12812.
cagcttgcaaaacgcaaacac---gaacatagtgg---------
>_R_gnl|SRA|SRR393016.3072631.1:1-32 HWI-EAS397_0015:6:20:14847:16945.
cagcttgcaaaacgcaaacac---gaacatagtgg---------
>_R_gnl|SRA|SRR393016.1932062.1:1-32 HWI-EAS397_0015:6:13:11212:21108.
cagcttgcaaaacgcaaacac---gaacatagtgg---------
>_R_gnl|SRA|SRR393016.1053270.1:1-32 HWI-EAS397_0015:6:7:14663:8763.
cagcttgcaaaacgcaaacac---gaacatagtgg---------

>_R_gnl|SRA|SRR393016.10998061.1:1-31 HWI-EAS397_0015:6:73:16781:18966.
cagcttgcaaaacgcaaacac---gaacatagtg----------
>_R_gnl|SRA|SRR393016.12244391.1:1-29 HWI-EAS397_0015:6:81:15083:15998.
cagcttgcaaaacgcaaacac---gaacatag------------
>_R_gnl|SRA|SRR393016.8477363.1:1-29 HWI-EAS397_0015:6:57:8306:17436.
cagcttgcaaaacgcaaacac---gaacatag------------
>_R_gnl|SRA|SRR393016.7232615.1:1-29 HWI-EAS397_0015:6:48:17223:10688.
cagcttgcaaaacgcaaacac---gaacatag------------
>gnl|SRA|SRR393016.5127927.1:11-38 HWI-EAS397_0015:6:34:13847:15826.
cagcttgcaaaacgcaaacac---gaacata-------------
>gnl|SRA|SRR393016.15406013.1:1-38 HWI-EAS397_0015:6:101:6946:14398.
---cttgcaaaacgcaaacactaggaacatagtgggagatt---
>gnl|SRA|SRR393016.10660464.1:1-38 HWI-EAS397_0015:6:71:13844:14941.
---cttgcaaaacgcaaacactaggaacatagtgggagatt---
>gnl|SRA|SRR393016.5407354.1:1-38 HWI-EAS397_0015:6:36:12379:11917.
---cttgcaaaacgcaaacaccaggaacatagtgggagatt---
>gnl|SRA|SRR393016.12413445.1:1-38 HWI-EAS397_0015:6:82:16242:15832.
--gcttgcaaaacgcaaacactaggaacatagtgggagat----
>gnl|SRA|SRR393016.11604740.1:1-38 HWI-EAS397_0015:6:77:14858:13646.
--gcttgcaaaacgcaaacactaggaacatagtgggagat----
>gnl|SRA|SRR393016.9495682.1:1-38 HWI-EAS397_0015:6:64:4216:13163.
--gcttgcaaaacgcaaacactaggaacatagtgggagat----
>_R_gnl|SRA|SRR393016.1355236.1:1-38 HWI-EAS397_0015:6:9:13940:2638.
--gcttgcaaaacgcaaacactaggaacatagtgggagat----
>gnl|SRA|SRR393016.15080493.1:1-36 HWI-EAS397_0015:6:99:7648:12176.
------gcaaaacgcaaacactaggaacatagtgggagattc--
>gnl|SRA|SRR393016.13027693.1:1-36 HWI-EAS397_0015:6:86:13766:14247.
------gcaaaacgcaaacactaggaacatagtgggagattc--
>gnl|SRA|SRR393016.10775212.1:1-36 HWI-EAS397_0015:6:72:9233:3428.
------gcaaaacgcaaacactaggaacatagtgggagattc--
>gnl|SRA|SRR393016.8361615.1:1-36 HWI-EAS397_0015:6:56:11878:16554.
------gcaaaacgcaaacactaggaacatagtgggagattc--
>gnl|SRA|SRR393016.7882229.1:1-36 HWI-EAS397_0015:6:53:7638:8153.
------gcaaaacgcaaacactaggaacatagtgggagattc--
>gnl|SRA|SRR393016.7882169.1:1-36 HWI-EAS397_0015:6:53:7632:8133.
------gcaaaacgcaaacactaggaacatagtgggagattc--
>gnl|SRA|SRR393016.7421736.1:1-36 HWI-EAS397_0015:6:50:6056:16037.
------gcaaaacgcaaacactaggaacatagtgggagattc--
>gnl|SRA|SRR393016.4724869.1:1-36 HWI-EAS397_0015:6:31:16446:9543.
------gcaaaacgcaaacactaggaacatagtgggagattc--
>gnl|SRA|SRR393016.3491646.1:1-36 HWI-EAS397_0015:6:23:10216:17269.
------gcaaaacgcaaacactaggaacatagtgggagattc--
>gnl|SRA|SRR393016.3408515.1:1-36 HWI-EAS397_0015:6:22:17892:3612.
------gcaaaacgcaaacactaggaacatagtgggagattc--
>gnl|SRA|SRR393016.2868134.1:1-36 HWI-EAS397_0015:6:19:9504:4700.
------gcaaaacgcaaacactaggaacatagtgggagattc--
>gnl|SRA|SRR393016.1397139.1:1-36 HWI-EAS397_0015:6:9:18396:20067.
------gcaaaacgcaaacactaggaacatagtgggagattc--
>gnl|SRA|SRR393016.2684136.1:1-36 HWI-EAS397_0015:6:18:6368:13914.
------gcaaaacgcaaacagtaggaccatagtgggagattc--
>_R_gnl|SRA|SRR393016.18098867.1:4-38 HWI-EAS397_0015:6:118:3170:19701.

-------caaaacgcaaacactaggaacatagtgggagattc--
>_R_gnl|SRA|SRR393016.10223968.1:4-38 HWI-EAS397_0015:6:68:17022:10609.
-------caaaacgcaaacactaggaacatagtgggagattc--
>gnl|SRA|SRR393016.8396020.1:1-35 HWI-EAS397_0015:6:56:15689:2175.
-------caaaacgcaaacactaggaacatagtgggagattc--
>gnl|SRA|SRR393016.3300475.1:1-35 HWI-EAS397_0015:6:22:6230:14989.
-------caaaacgcaaacactaggaacatagtgggagattc--
>gnl|SRA|SRR393016.2623823.1:1-35 HWI-EAS397_0015:6:17:16837:2693.
-------caaaacgcaaacactaggaacatagtgggagattc--
>_R_gnl|SRA|SRR393016.1492063.1:4-38 HWI-EAS397_0015:6:10:12318:21152.
-------caaaacgcaaacactaggaacatagtgggagattc--
>gnl|SRA|SRR393016.1213442.1:1-35 HWI-EAS397_0015:6:8:15200:3958.
-------caaaacgcaaacactaggaacatagtgggagattc--
>gnl|SRA|SRR393016.11510001.1:1-35 HWI-EAS397_0015:6:77:4329:2049.
-------caaaacgcaaacagtaggaccatagtgggagattc--
>gnl|SRA|SRR393016.6677855.1:1-34 HWI-EAS397_0015:6:45:4753:14011.
--------aaaacgcaaacactaggaacatagtgggagattc--
>gnl|SRA|SRR393016.17938925.1:1-30 HWI-EAS397_0015:6:117:2780:18925.
------------cgcaaacactaggaacatagtgggagattc--
>gnl|SRA|SRR393016.10423590.1:1-30 HWI-EAS397_0015:6:70:4388:7876.
------------cgcaaacactaggaacatagtgggagattc--
>gnl|SRA|SRR393016.792941.1:1-30 HWI-EAS397_0015:6:6:2583:11690.
------------cgcaaacactaggaacatcgtgggagattc--
>gnl|SRA|SRR393016.14806821.1:1-30 HWI-EAS397_0015:6:97:13796:11192.
------------cgcaaacactaggaacataggggaagattc--
>_R_gnl|SRA|SRR393016.15442576.1:10-38 HWI-EAS397_0015:6:101:10749:21160.
-------------gcaaacactaggaacatagtgggagattc--
>gnl|SRA|SRR393016.10820508.1:1-29 HWI-EAS397_0015:6:72:14310:2148.
-------------gcaaacactaggaacatagtgggagattc--
>gnl|SRA|SRR393016.6424948.1:1-29 HWI-EAS397_0015:6:43:10208:2942.
-------------gcaaacactaggaacatagtgggagattc--
>gnl|SRA|SRR393016.3995063.1:1-29 HWI-EAS397_0015:6:26:15372:19177.
-------------gcaaacactaggaacatagtgggagattc--
>gnl|SRA|SRR393016.9611452.1:1-29 HWI-EAS397_0015:6:64:17541:11212.
-------------gcaaacagtaggaccatagtgggagattc--
>gnl|SRA|SRR393016.13239511.1:1-28 HWI-EAS397_0015:6:88:1912:19574.
--------------caaacactaggaacatagtgggagattc--
>_R_gnl|SRA|SRR393016.376357.1:12-38 HWI-EAS397_0015:6:3:8416:15523.
---------------aaacactaggaacatagtgggagattc--
>gnl|SRA|SRR393016.3724747.1:1-27 HWI-EAS397_0015:6:24:18660:2988.
---------------aaacactaggcacatagtgggagattc--
>_R_gnl|SRA|SRR393016.3962449.1:2-38 HWI-EAS397_0015:6:26:11844:9969.
--gcttgcaaaacgcaaacac---gaacatagtgggagattc--
>gnl|SRA|SRR393016.2952311.1:1-37 HWI-EAS397_0015:6:19:18589:8397.
--gcttgcaaaacgcaaacac---gaacatagtgggagattc--
>_R_gnl|SRA|SRR393016.1411189.1:2-38 HWI-EAS397_0015:6:10:2929:13414.
--gcttgcaaaacgcaaacag---gaccatagtgggagattc--
>gnl|SRA|SRR393016.16149623.1:1-36 HWI-EAS397_0015:6:105:14852:21086.
---cttgcaaaacgcaaacac---gaacatagtgggagattc--
>gnl|SRA|SRR393016.15813926.1:1-36 HWI-EAS397_0015:6:103:15054:12068.
---cttgcaaaacgcaaacag---gaccatagtgggagattc--

>_R_gnl|SRA|SRR393016.2709528.1:3-38 HWI-EAS397_0015:6:18:9128:3032.
---cttgcaaaacgcaaacag---gaccatagtgggagattc--
>_R_gnl|SRA|SRR393016.17889978.1:4-38 HWI-EAS397_0015:6:116:14743:14808.
----ttgcaaaacgcaaacac---gaacatagtgggagattc--
>_R_gnl|SRA|SRR393016.9850390.1:4-38 HWI-EAS397_0015:6:66:9562:15263.
----ttgcaaaacgcaaacac---gaacatagtgggagattc--
>_R_gnl|SRA|SRR393016.258400.1:4-38 HWI-EAS397_0015:6:2:12507:6596.
----ttgcaaaacgcaaacac---gaacatagtgggagattc--
>_R_gnl|SRA|SRR393016.11591830.1:4-38 HWI-EAS397_0015:6:77:13398:18424.
----ttgcaaaacgcaaacag---gaccatagtgggagattc--
>_R_gnl|SRA|SRR393016.8980936.1:4-38 HWI-EAS397_0015:6:60:16130:7347.
----ttgcaaaacgcaaacag---gaccatagtgggagattc--
>gnl|SRA|SRR393016.17135061.1:1-33 HWI-EAS397_0015:6:111:17920:14997.
------gcaaaacgcaaacac---gaacatagtgggagattc--
>gnl|SRA|SRR393016.13368934.1:1-33 HWI-EAS397_0015:6:88:16046:15954.
------gcaaaacgcaaacac---gaacatagtgggagattc--
>gnl|SRA|SRR393016.10832798.1:1-33 HWI-EAS397_0015:6:72:15709:8089.
------gcaaaacgcaaacac---gaacatagtgggagattc--
>gnl|SRA|SRR393016.6112609.1:1-33 HWI-EAS397_0015:6:41:8927:10894.
------gcaaaacgcaaacac---gaacatagtgggagattc--
>gnl|SRA|SRR393016.4230240.1:1-33 HWI-EAS397_0015:6:28:8740:5592.
------gcaaaacgcaaacac---gaacatagtgggagattc--
>gnl|SRA|SRR393016.2625022.1:1-33 HWI-EAS397_0015:6:17:16957:11538.
------gcaaaacgcaaacac---gaacatagtgggagattc--
>gnl|SRA|SRR393016.2394349.1:1-33 HWI-EAS397_0015:6:16:9567:11349.
------gcaaaacgcaaacac---gaacatagtgggagattc--
>gnl|SRA|SRR393016.16140798.1:1-33 HWI-EAS397_0015:6:105:13906:1090.
------gcaaaacgcaaacag---gaccatagtgggagattc--
>gnl|SRA|SRR393016.15908418.1:1-33 HWI-EAS397_0015:6:104:7288:6655.
------gcaaaacgcaaacag---gaccatagtgggagattc--
>_R_gnl|SRA|SRR393016.15097156.1:6-38 HWI-EAS397_0015:6:99:9424:17449.
------gcaaaacgcaaacag---gaccatagtgggagattc--
>gnl|SRA|SRR393016.13951627.1:1-33 HWI-EAS397_0015:6:92:9557:7552.
------gcaaaacgcaaacag---gaccatagtgggagattc--
>gnl|SRA|SRR393016.12879348.1:1-33 HWI-EAS397_0015:6:85:15148:11170.
------gcaaaacgcaaacag---gaccatagtgggagattc--
>gnl|SRA|SRR393016.12415091.1:1-33 HWI-EAS397_0015:6:82:16428:2010.
------gcaaaacgcaaacag---gaccatagtgggagattc--
>gnl|SRA|SRR393016.4727676.1:1-33 HWI-EAS397_0015:6:31:16748:3830.
------gcaaaacgcaaacag---gaccatagtgggagattc--
>gnl|SRA|SRR393016.3577088.1:1-33 HWI-EAS397_0015:6:24:1939:5701.
------gcaaaacgcaaacag---gaccatagtgggagattc--
>gnl|SRA|SRR393016.3718647.1:1-32 HWI-EAS397_0015:6:24:18010:18105.
-------caaaacgcaaacac---gaacatagtgggagattc--
>gnl|SRA|SRR393016.13779682.1:1-32 HWI-EAS397_0015:6:91:8393:5446.
-------caaaacgcaaacag---gaccatagtgggagattc--
>_R_gnl|SRA|SRR393016.16140268.1:8-38 HWI-EAS397_0015:6:105:13850:3736.
--------aaaacgcaaacac---gaacatagtgggagattc--
>gnl|SRA|SRR393016.9479956.1:1-31 HWI-EAS397_0015:6:64:2353:9267.
--------aaaacgcaaacac---gaacatagtgggagattc--
>gnl|SRA|SRR393016.9868638.1:1-31 HWI-EAS397_0015:6:66:11638:7170.

--------aaaacgcaaacag---gaccatagtgggagattc--
>_R_gnl|SRA|SRR393016.7795987.1:8-38 HWI-EAS397_0015:6:52:14721:15553.
--------aaaacgcaaacag---gaccatagtgggagattc--
>_R_gnl|SRA|SRR393016.18020464.1:9-38 HWI-EAS397_0015:6:117:11820:12753.
---------aaacgcaaacac---gaacatagtgggagattc--
>_R_gnl|SRA|SRR393016.17690124.1:9-38 HWI-EAS397_0015:6:115:9850:14177.
---------aaacgcaaacag---gaccatagtgggagattc--
>_R_gnl|SRA|SRR393016.12055382.1:9-38 HWI-EAS397_0015:6:80:11915:9693.
---------aaacgcaaacag---gaccatagtgggagattc--
>_R_gnl|SRA|SRR393016.11340506.1:10-38 HWI-EAS397_0015:6:76:2748:9828.
----------aacgcaaacac---gaacatagtgggagattc--
>gnl|SRA|SRR393016.8471984.1:1-29 HWI-EAS397_0015:6:57:7695:8163.
----------aacgcaaacac---gaacatagtgggagattc--
>gnl|SRA|SRR393016.15970551.1:1-29 HWI-EAS397_0015:6:104:13699:3671.
----------aacgcaaacag---gaccatagtgggagattc--
>_R_gnl|SRA|SRR393016.16531342.1:11-38 HWI-EAS397_0015:6:108:3866:5548.
-----------acgcaaacag---gaccatagtgggagattc--
>gnl|SRA|SRR393016.18376305.1:1-27 HWI-EAS397_0015:6:119:16667:6057.
------------cgcaaacac---gaacatagtgggagattc--
>gnl|SRA|SRR393016.6572535.1:1-27 HWI-EAS397_0015:6:44:9861:18341.
------------cgcaaacac---gaacatagtgggagattc--
>gnl|SRA|SRR393016.4253687.1:1-27 HWI-EAS397_0015:6:28:11280:1086.
------------cgcaaacac---gaacatagtgggagattc--
>gnl|SRA|SRR393016.7504075.1:1-26 HWI-EAS397_0015:6:50:15093:3159.
-------------gcaaacac---gaacatagtgggagattc--
>gnl|SRA|SRR393016.6790533.1:1-26 HWI-EAS397_0015:6:45:17421:18684.
-------------gcaaacag---gaccatagtgggagattc--
>_R_gnl|SRA|SRR393016.14636767.1:15-38 HWI-EAS397_0015:6:96:13366:10587.
---------------aaacac---gaacatagtgggagattc--
>_R_gnl|SRA|SRR393016.13987573.1:15-38 HWI-EAS397_0015:6:92:13473:11624.
---------------aaacac---gaacatagtgggagattc--
>_R_gnl|SRA|SRR393016.13273728.1:15-38 HWI-EAS397_0015:6:88:5647:13069.
----------aacgcaaacac---taggcccatggtg-------

SRX058600 ZMM23

>contrZMM23 .
tagcttgcaaaacgcaaacagtaggaccatagtgggagattc-
>_R_gnl|SRA|SRR393016.9506646.1:1-38 HWI-EAS397_0015:6:64:5491:17685.
tagcttgcaaaacgcaaacagtaggaccatagtgggag-----
>_R_gnl|SRA|SRR393016.17670278.1:1-38 HWI-EAS397_0015:6:115:7656:12048.
cagcttgcaaaacgcaaacactaggaacatagtgggag-----
>gnl|SRA|SRR393016.17395933.1:1-38 HWI-EAS397_0015:6:113:11721:7601.
cagcttgcaaaacgcaaacactaggaacatagtgggag-----
>_R_gnl|SRA|SRR393016.14502039.1:1-38 HWI-EAS397_0015:6:95:16651:2979.
cagcttgcaaaacgcaaacactaggaacatagtgggag-----
>_R_gnl|SRA|SRR393016.9795169.1:1-38 HWI-EAS397_0015:6:66:3130:9169.
cagcttgcaaaacgcaaacactaggaacatagtgggag-----
>_R_gnl|SRA|SRR393016.4204405.1:1-38 HWI-EAS397_0015:6:28:5830:10923.
cagcttgcaaaacgcaaacactaggaacatagtgggag-----
>_R_gnl|SRA|SRR393016.14208964.1:1-37 HWI-EAS397_0015:6:94:2304:18726.

cagcttgcaaaacgcaaacactaggaacatagtggga------
>_R_gnl|SRA|SRR393016.13132953.1:1-37 HWI-EAS397_0015:6:87:7799:9394.
cagcttgcaaaacgcaaacactaggaacatagtggga------
>_R_gnl|SRA|SRR393016.13614939.1:1-36 HWI-EAS397_0015:6:90:7858:4819.
cagcttgcaaaacgcaaacactaggaacatagtggg-------
>_R_gnl|SRA|SRR393016.1152116.1:1-36 HWI-EAS397_0015:6:8:8632:3300.
cagcttgcaaaacgcaaacactaggaacatagtggg-------
>gnl|SRA|SRR393016.13230869.1:5-38 HWI-EAS397_0015:6:87:18796:9831.
cagcttgcaaaacgcaaacactaggaacatagtg---------
>_R_gnl|SRA|SRR393016.10633817.1:1-34 HWI-EAS397_0015:6:71:10788:11077.
cagcttgcaaaacgcaaacactaggaacatagtg---------
>gnl|SRA|SRR393016.14370299.1:4-38 HWI-EAS397_0015:6:95:2510:7379.
tagcttgcaaaacgcaaacagtaggaccatagtgg--------
>gnl|SRA|SRR393016.11036782.1:4-38 HWI-EAS397_0015:6:74:3486:18890.
tagcttgcaaaacgcaaacagtaggaccatagtgg--------
>_R_gnl|SRA|SRR393016.16785507.1:1-35 HWI-EAS397_0015:6:109:14352:8818.
cagcttgcaaaacgcaaacactaggaacatagtgg--------
>_R_gnl|SRA|SRR393016.13427738.1:1-35 HWI-EAS397_0015:6:89:4911:4122.
cagcttgcaaaacgcaaacactaggaacatagtgg--------
>_R_gnl|SRA|SRR393016.7715491.1:1-35 HWI-EAS397_0015:6:52:5584:3561.
cagcttgcaaaacgcaaacactaggaacatagtgg--------
>_R_gnl|SRA|SRR393016.4926585.1:1-35 HWI-EAS397_0015:6:33:7601:20931.
cagcttgcaaaacgcaaacactaggaacatagtgg--------
>_R_gnl|SRA|SRR393016.4098055.1:1-35 HWI-EAS397_0015:6:27:10626:8311.
cagcttgcaaaacgcaaacactaggaacatagtgg--------
>gnl|SRA|SRR393016.2354721.1:4-38 HWI-EAS397_0015:6:16:5230:9519.
cagcttgcaaaacgcaaacactaggaacatagtgg--------
>gnl|SRA|SRR393016.12836800.1:6-38 HWI-EAS397_0015:6:85:10535:7439.
cagcttgcaaaacgcaaacactaggaacatagt----------
>_R_gnl|SRA|SRR393016.9280546.1:1-32 HWI-EAS397_0015:6:62:14917:11240.
cagcttgcaaaacgcaaacactaggaacatag-----------
>_R_gnl|SRA|SRR393016.3733541.1:1-32 HWI-EAS397_0015:6:25:2468:5290.
cagcttgcaaaacggaaacactaggaacatag-----------
>_R_gnl|SRA|SRR393016.14092747.1:1-31 HWI-EAS397_0015:6:93:7319:12901.
cagcttgcaaaacgcaaacactaggaacata------------
>gnl|SRA|SRR393016.3322761.1:9-38 HWI-EAS397_0015:6:22:8725:18162.
cagcttgcaaaacgcaaacactaggaacat-------------
>gnl|SRA|SRR393016.2042269.1:9-38 HWI-EAS397_0015:6:14:5901:12114.
cagcttgcaaaacgcaaacactaggaacat-------------
>gnl|SRA|SRR393016.16302695.1:11-38 HWI-EAS397_0015:6:106:13459:7709.
cagcttgcaaaacgcaaacactaggaac---------------
>gnl|SRA|SRR393016.12695529.1:11-38 HWI-EAS397_0015:6:84:12505:20556.
cagcttgcaaaacgcaaacactaggaac---------------
>_R_gnl|SRA|SRR393016.8602105.1:1-28 HWI-EAS397_0015:6:58:5845:10416.
cagcttgcaaaacgcaaacactaggaac---------------
>gnl|SRA|SRR393016.6993393.1:11-38 HWI-EAS397_0015:6:47:7158:2193.
cagcttgcaaaacgcaaacactaggaac---------------
>_R_gnl|SRA|SRR393016.2333016.1:1-27 HWI-EAS397_0015:6:16:2618:10513.
cagcttgcaaaacgcaaacactaggaa----------------
>_R_gnl|SRA|SRR393016.17722735.1:1-25 HWI-EAS397_0015:6:115:13519:20326.
cagcttgcaaaacgcaaacactagg------------------

>_R_gnl|SRA|SRR393016.10722541.1:1-25 HWI-EAS397_0015:6:72:3310:19854.
cagcttgcaaaacgcaaacactagg------------------
>gnl|SRA|SRR393016.8534592.1:14-38 HWI-EAS397_0015:6:57:14690:18439.
cagcttgcaaaacgcaaacactagg------------------
>gnl|SRA|SRR393016.3571127.1:14-38 HWI-EAS397_0015:6:23:18662:1254.
cagcttgcaaaacgcaaacactagg------------------
>gnl|SRA|SRR393016.3048956.1:14-38 HWI-EAS397_0015:6:20:12336:15652.
cagcttgcaaaacgcaaacactagg------------------
>gnl|SRA|SRR393016.9615528.1:15-38 HWI-EAS397_0015:6:64:18030:4052.
cagcttgcaaaacgcaaacactag-------------------
>gnl|SRA|SRR393016.9582101.1:15-38 HWI-EAS397_0015:6:64:14110:7320.
cagcttgcaaaacgcaaacactag-------------------
>_R_gnl|SRA|SRR393016.6778293.1:1-24 HWI-EAS397_0015:6:45:16090:4917.
cagcttgcaaaacgcaaacactag-------------------
>_R_gnl|SRA|SRR393016.4269760.1:1-24 HWI-EAS397_0015:6:28:12990:12012.
cagcttgcaaaacgcaaacactag-------------------
>gnl|SRA|SRR393016.4161737.1:15-38 HWI-EAS397_0015:6:27:17424:21234.
cagcttgcaaaacgcaaacactag-------------------
>gnl|SRA|SRR393016.3923490.1:15-38 HWI-EAS397_0015:6:26:7511:6223.
cagcttgcaaaacgcaaacactag-------------------
>_R_gnl|SRA|SRR393016.2826973.1:1-24 HWI-EAS397_0015:6:19:4833:15439.
cagcttgcaaaacgcaaacactag-------------------
>gnl|SRA|SRR393016.595960.1:15-38 HWI-EAS397_0015:6:4:15281:14212.
cagcttgcaaaacgcaaacactag-------------------
>_R_gnl|SRA|SRR393016.181821.1:1-24 HWI-EAS397_0015:6:2:4167:10507.
cagcttgcaaaacgcaaacactag-------------------
>gnl|SRA|SRR393016.15190163.1:17-38 HWI-EAS397_0015:6:100:1850:1228.
cagcttgcaaaacgcaaacact---------------------
>gnl|SRA|SRR393016.11427525.1:17-38 HWI-EAS397_0015:6:76:12652:16527.
cagcttgcaaaacgcaaacact---------------------
>gnl|SRA|SRR393016.8418739.1:17-38 HWI-EAS397_0015:6:56:18207:15675.
cagcttgcaaaacgcaaacact---------------------
>_R_gnl|SRA|SRR393016.15423426.1:5-25 HWI-EAS397_0015:6:101:8762:6831.
cagcttgcaaaacgcaaacac----------------------
>_R_gnl|SRA|SRR393016.13751536.1:5-25 HWI-EAS397_0015:6:91:5347:9132.
cagcttgcaaaacgcaaacac----------------------
>gnl|SRA|SRR393016.11666427.1:15-35 HWI-EAS397_0015:6:78:4134:1725.
cagcttgcaaaacgcaaacac----------------------
>gnl|SRA|SRR393016.10556228.1:18-38 HWI-EAS397_0015:6:71:1902:4357.
cagcttgcaaaacgcaaacac----------------------
>_R_gnl|SRA|SRR393016.9753378.1:5-25 HWI-EAS397_0015:6:65:16046:16226.
cagcttgcaaaacgcaaacac----------------------
>gnl|SRA|SRR393016.9570560.1:18-38 HWI-EAS397_0015:6:64:12784:7647.
cagcttgcaaaacgcaaacac----------------------
>_R_gnl|SRA|SRR393016.4635465.1:5-25 HWI-EAS397_0015:6:31:6500:20853.
cagcttgcaaaacgcaaacac----------------------
>gnl|SRA|SRR393016.725362.1:18-38 HWI-EAS397_0015:6:5:12544:16208.
cagcttgcaaaacgcaaacac----------------------
>_R_gnl|SRA|SRR393016.18361777.1:1-35 HWI-EAS397_0015:6:119:14972:1577.
cagcttgcaaaacgcaaacac---gaacatagtgggag-----
>_R_gnl|SRA|SRR393016.13773964.1:1-35 HWI-EAS397_0015:6:91:7770:19856.

cagcttgcaaaacgcaaacac---gaacatagtgggag-----
>_R_gnl|SRA|SRR393016.10160399.1:1-35 HWI-EAS397_0015:6:68:9754:2409.
cagcttgcaaaacgcaaacac---gaacatagtgggag-----
>_R_gnl|SRA|SRR393016.4405832.1:1-35 HWI-EAS397_0015:6:29:12211:15584.
cagcttgcaaaacgcaaatac---gaacatagtgggag-----
>_R_gnl|SRA|SRR393016.2254844.1:1-34 HWI-EAS397_0015:6:15:11735:7206.
cagcttgcaaaacgcaaacac---gaacatagtggga------
>_R_gnl|SRA|SRR393016.4771682.1:1-33 HWI-EAS397_0015:6:32:6036:16559.
cagcttgcaaaacgcaaacac---gaacatagtggg-------
>_R_gnl|SRA|SRR393016.17663582.1:1-32 HWI-EAS397_0015:6:115:6915:12126.
cagcttgcaaaacgcaaacac---gaacatagtgg--------
>_R_gnl|SRA|SRR393016.13798889.1:1-32 HWI-EAS397_0015:6:91:10460:1049.
cagcttgcaaaacgcaaacac---gaacatagtgg--------
>_R_gnl|SRA|SRR393016.9494800.1:1-32 HWI-EAS397_0015:6:64:4108:18094.
cagcttgcaaaacgcaaacac---gaacatagtgg--------
>gnl|SRA|SRR393016.6156144.1:7-38 HWI-EAS397_0015:6:41:13692:12011.
cagcttgcaaaacgcaaacac---gaacatagtgg--------
>_R_gnl|SRA|SRR393016.4604036.1:1-32 HWI-EAS397_0015:6:30:18423:12812.
cagcttgcaaaacgcaaacac---gaacatagtgg--------
>_R_gnl|SRA|SRR393016.3072631.1:1-32 HWI-EAS397_0015:6:20:14847:16945.
cagcttgcaaaacgcaaacac---gaacatagtgg--------
>_R_gnl|SRA|SRR393016.1932062.1:1-32 HWI-EAS397_0015:6:13:11212:21108.
cagcttgcaaaacgcaaacac---gaacatagtgg--------
>_R_gnl|SRA|SRR393016.1053270.1:1-32 HWI-EAS397_0015:6:7:14663:8763.
cagcttgcaaaacgcaaacac---gaacatagtgg--------
>_R_gnl|SRA|SRR393016.10998061.1:1-31 HWI-EAS397_0015:6:73:16781:18966.
cagcttgcaaaacgcaaacac---gaacatagtg---------
>_R_gnl|SRA|SRR393016.12244391.1:1-29 HWI-EAS397_0015:6:81:15083:15998.
cagcttgcaaaacgcaaacac---gaacatag-----------
>_R_gnl|SRA|SRR393016.8477363.1:1-29 HWI-EAS397_0015:6:57:8306:17436.
cagcttgcaaaacgcaaacac---gaacatag-----------
>_R_gnl|SRA|SRR393016.7232615.1:1-29 HWI-EAS397_0015:6:48:17223:10688.
cagcttgcaaaacgcaaacac---gaacatag-----------
>gnl|SRA|SRR393016.5127927.1:11-38 HWI-EAS397_0015:6:34:13847:15826.
cagcttgcaaaacgcaaacac---gaacata------------
>gnl|SRA|SRR393016.11990107.1:19-38 HWI-EAS397_0015:6:80:4671:19821.
cagcttgcaaaacgcaaaca-----------------------
>gnl|SRA|SRR393016.8010427.1:19-38 HWI-EAS397_0015:6:54:5138:15830.
cagcttgcaaaacgcaaaca-----------------------
>_R_gnl|SRA|SRR393016.2668472.1:1-19 HWI-EAS397_0015:6:18:4563:2097.
cagcttgcaaaacgcaaac------------------------
>_R_gnl|SRA|SRR393016.17938098.1:1-18 HWI-EAS397_0015:6:117:2686:9677.
cagcttgcaaaacgcaaa-------------------------
>gnl|SRA|SRR393016.14929369.1:21-38 HWI-EAS397_0015:6:98:9133:17753.
cagcttgcaaaacgcaaa-------------------------
>gnl|SRA|SRR393016.6293783.1:21-38 HWI-EAS397_0015:6:42:12348:2263.
cagcttgcaaaacgcaaa-------------------------
>_R_gnl|SRA|SRR393016.4341024.1:1-18 HWI-EAS397_0015:6:29:4693:19355.
cagcttgcaaaacgcaaa-------------------------
>_R_gnl|SRA|SRR393016.4009543.1:1-18 HWI-EAS397_0015:6:26:16935:3713.
cagcttgcaaaacgcaaa-------------------------

>gnl|SRA|SRR393016.17276434.1:22-38 HWI-EAS397_0015:6:112:15948:5372.
cagcttgcaaaacgcaa--------------------------
>gnl|SRA|SRR393016.7935418.1:22-38 HWI-EAS397_0015:6:53:13536:15988.
cagcttgcaaaacgcaa--------------------------
>_R_gnl|SRA|SRR393016.1269675.1:1-17 HWI-EAS397_0015:6:9:4452:20103.
cagcttgcaaaacgcaa--------------------------
>gnl|SRA|SRR393016.11601065.1:9-38 HWI-EAS397_0015:6:77:14444:11341.
-agcttgcaaaacgcaaacagtaggaccata------------
>gnl|SRA|SRR393016.16301381.1:12-30 HWI-EAS397_0015:6:106:13317:11219.
-agcttgcaaaacgcaaaca-----------------------
>gnl|SRA|SRR393016.11314563.1:12-30 HWI-EAS397_0015:6:75:17518:1744.
-agcttgcaaaacgcaaaca-----------------------
>gnl|SRA|SRR393016.7077009.1:12-30 HWI-EAS397_0015:6:47:16433:4683.
-agcttgcaaaacgcaaaca-----------------------
>gnl|SRA|SRR393016.17891139.1:19-37 HWI-EAS397_0015:6:116:14879:11786.
-agcttgcaaaacgcaaaca-----------------------
>gnl|SRA|SRR393016.16804142.1:15-33 HWI-EAS397_0015:6:109:16449:18454.
-agcttgcaaaacgcaaaca-----------------------
>gnl|SRA|SRR393016.12699309.1:16-34 HWI-EAS397_0015:6:84:12945:13010.
-agcttgcaaaacgcaaaca-----------------------
>gnl|SRA|SRR393016.11659437.1:15-33 HWI-EAS397_0015:6:78:3351:5585.
-agcttgcaaaacgcaaaca-----------------------
>gnl|SRA|SRR393016.10997752.1:16-34 HWI-EAS397_0015:6:73:16746:19028.
-agcttgcaaaacgcaaaca-----------------------
>gnl|SRA|SRR393016.7573504.1:19-37 HWI-EAS397_0015:6:51:6291:11144.
-agcttgcaaaacgcaaaca-----------------------
>gnl|SRA|SRR393016.4462835.1:13-31 HWI-EAS397_0015:6:29:18252:15185.
-agcttgcaaaacgcaaaca-----------------------
>gnl|SRA|SRR393016.470801.1:19-37 HWI-EAS397_0015:6:3:18499:8060.
-agcttgcaaaacgcaaaca-----------------------
>gnl|SRA|SRR393016.2057811.1:20-38 HWI-EAS397_0015:6:14:7597:1247.
-agcttgcaaaacgcaaaca-----------------------
>gnl|SRA|SRR393016.6915642.1:3-38 HWI-EAS397_0015:6:46:14951:13053.
-agcttgcaaaacgcaaacag---gaccatagtgggagat---
>_R_gnl|SRA|SRR393016.13964636.1:1-34 HWI-EAS397_0015:6:92:10957:3027.
-agcttgcaaaacgcaaacag---gaccatagtgggag-----
>gnl|SRA|SRR393016.9838572.1:9-38 HWI-EAS397_0015:6:66:8202:14244.
-agcttgcaaaacgcaaacag---gaccatagtg---------
>gnl|SRA|SRR393016.7641388.1:9-38 HWI-EAS397_0015:6:51:14007:16683.
-agcttgcaaaacgcaaacag---gaccatagtg---------
>gnl|SRA|SRR393016.7583694.1:9-38 HWI-EAS397_0015:6:51:7518:4295.
-agcttgcaaaacgcaaacag---gaccatagtg---------
>gnl|SRA|SRR393016.2614513.1:9-38 HWI-EAS397_0015:6:17:15861:9092.
-agcttgcaaaacgcaaacag---gaccatagtg---------
>gnl|SRA|SRR393016.2995766.1:23-38 HWI-EAS397_0015:6:20:6596:17210.
-agcttgcaaaacgcaa-------------------------
>gnl|SRA|SRR393016.2684136.1:1-36 HWI-EAS397_0015:6:18:6368:13914.
------gcaaaacgcaaacagtaggaccatagtgggagattc-
>gnl|SRA|SRR393016.15080493.1:1-36 HWI-EAS397_0015:6:99:7648:12176.
------gcaaaacgcaaacactaggaacatagtgggagattc-
>gnl|SRA|SRR393016.13027693.1:1-36 HWI-EAS397_0015:6:86:13766:14247.

------gcaaaacgcaaacactaggaacatagtgggagattc-
>gnl|SRA|SRR393016.10775212.1:1-36 HWI-EAS397_0015:6:72:9233:3428.
------gcaaaacgcaaacactaggaacatagtgggagattc-
>gnl|SRA|SRR393016.8361615.1:1-36 HWI-EAS397_0015:6:56:11878:16554.
------gcaaaacgcaaacactaggaacatagtgggagattc-
>gnl|SRA|SRR393016.7882229.1:1-36 HWI-EAS397_0015:6:53:7638:8153.
------gcaaaacgcaaacactaggaacatagtgggagattc-
>gnl|SRA|SRR393016.7882169.1:1-36 HWI-EAS397_0015:6:53:7632:8133.
------gcaaaacgcaaacactaggaacatagtgggagattc-
>gnl|SRA|SRR393016.7421736.1:1-36 HWI-EAS397_0015:6:50:6056:16037.
------gcaaaacgcaaacactaggaacatagtgggagattc-
>gnl|SRA|SRR393016.4724869.1:1-36 HWI-EAS397_0015:6:31:16446:9543.
------gcaaaacgcaaacactaggaacatagtgggagattc-
>gnl|SRA|SRR393016.3491646.1:1-36 HWI-EAS397_0015:6:23:10216:17269.
------gcaaaacgcaaacactaggaacatagtgggagattc-
>gnl|SRA|SRR393016.3408515.1:1-36 HWI-EAS397_0015:6:22:17892:3612.
------gcaaaacgcaaacactaggaacatagtgggagattc-
>gnl|SRA|SRR393016.2868134.1:1-36 HWI-EAS397_0015:6:19:9504:4700.
------gcaaaacgcaaacactaggaacatagtgggagattc-
>gnl|SRA|SRR393016.1397139.1:1-36 HWI-EAS397_0015:6:9:18396:20067.
------gcaaaacgcaaacactaggaacatagtgggagattc-
>gnl|SRA|SRR393016.11510001.1:1-35 HWI-EAS397_0015:6:77:4329:2049.
-------caaaacgcaaacagtaggaccatagtgggagattc-
>_R_gnl|SRA|SRR393016.18098867.1:4-38 HWI-EAS397_0015:6:118:3170:19701.
-------caaaacgcaaacactaggaacatagtgggagattc-
>_R_gnl|SRA|SRR393016.10223968.1:4-38 HWI-EAS397_0015:6:68:17022:10609.
-------caaaacgcaaacactaggaacatagtgggagattc-
>gnl|SRA|SRR393016.8396020.1:1-35 HWI-EAS397_0015:6:56:15689:2175.
-------caaaacgcaaacactaggaacatagtgggagattc-
>gnl|SRA|SRR393016.3300475.1:1-35 HWI-EAS397_0015:6:22:6230:14989.
-------caaaacgcaaacactaggaacatagtgggagattc-
>gnl|SRA|SRR393016.2623823.1:1-35 HWI-EAS397_0015:6:17:16837:2693.
-------caaaacgcaaacactaggaacatagtgggagattc-
>_R_gnl|SRA|SRR393016.1492063.1:4-38 HWI-EAS397_0015:6:10:12318:21152.
-------caaaacgcaaacactaggaacatagtgggagattc-
>gnl|SRA|SRR393016.1213442.1:1-35 HWI-EAS397_0015:6:8:15200:3958.
-------caaaacgcaaacactaggaacatagtgggagattc-
>gnl|SRA|SRR393016.6677855.1:1-34 HWI-EAS397_0015:6:45:4753:14011.
--------aaaacgcaaacactaggaacatagtgggagattc-
>gnl|SRA|SRR393016.17938925.1:1-30 HWI-EAS397_0015:6:117:2780:18925.
------------cgcaaacactaggaacatagtgggagattc-
>gnl|SRA|SRR393016.10423590.1:1-30 HWI-EAS397_0015:6:70:4388:7876.
------------cgcaaacactaggaacatagtgggagattc-
>gnl|SRA|SRR393016.792941.1:1-30 HWI-EAS397_0015:6:6:2583:11690.
------------cgcaaacactaggaacatcgtgggagattc-
>gnl|SRA|SRR393016.9611452.1:1-29 HWI-EAS397_0015:6:64:17541:11212.
-------------gcaaacagtaggaccatagtgggagattc-
>_R_gnl|SRA|SRR393016.15442576.1:10-38 HWI-EAS397_0015:6:101:10749:21160.
-------------gcaaacactaggaacatagtgggagattc-
>gnl|SRA|SRR393016.10820508.1:1-29 HWI-EAS397_0015:6:72:14310:2148.
-------------gcaaacactaggaacatagtgggagattc-

>gnl|SRA|SRR393016.6424948.1:1-29 HWI-EAS397_0015:6:43:10208:2942.
-------------gcaaacactaggaacatagtgggagattc-
>gnl|SRA|SRR393016.3995063.1:1-29 HWI-EAS397_0015:6:26:15372:19177.
-------------gcaaacactaggaacatagtgggagattc-
>gnl|SRA|SRR393016.13239511.1:1-28 HWI-EAS397_0015:6:88:1912:19574.
--------------caaacactaggaacatagtgggagattc-
>_R_gnl|SRA|SRR393016.376357.1:12-38 HWI-EAS397_0015:6:3:8416:15523.
---------------aaacactaggaacatagtgggagattc-
>gnl|SRA|SRR393016.3724747.1:1-27 HWI-EAS397_0015:6:24:18660:2988.
---------------aaacactaggcacatagtgggagattc-
>_R_gnl|SRA|SRR393016.10470100.1:15-38 HWI-EAS397_0015:6:70:9673:9670.
------------------cactaggaacatagtgggagattc-
>gnl|SRA|SRR393016.8880748.1:1-24 HWI-EAS397_0015:6:60:4598:10115.
------------------cactaggaacatagtgggagattc-
>gnl|SRA|SRR393016.13074698.1:16-36 HWI-EAS397_0015:6:87:1379:16463.
---------------------taggaacatagtgggagattc-
>gnl|SRA|SRR393016.5665382.1:16-36 HWI-EAS397_0015:6:38:8839:1067.
---------------------taggaacatagtgggagattc-
>_R_gnl|SRA|SRR393016.4603189.1:18-38 HWI-EAS397_0015:6:30:18331:16731.
---------------------taggaacatagtgggagattc-
>_R_gnl|SRA|SRR393016.2488562.1:18-38 HWI-EAS397_0015:6:17:1835:20060.
---------------------taggaacatagtgggagattc-
>gnl|SRA|SRR393016.16666149.1:1-19 HWI-EAS397_0015:6:109:1403:9819.
-----------------------ggaacatagtgggagattc-
>gnl|SRA|SRR393016.16056912.1:1-19 HWI-EAS397_0015:6:105:5093:6224.
-----------------------ggaacatagtgggagattc-
>gnl|SRA|SRR393016.9695111.1:1-19 HWI-EAS397_0015:6:65:9362:19073.
-----------------------ggaacatagtgggagattc-
>gnl|SRA|SRR393016.9676148.1:1-19 HWI-EAS397_0015:6:65:7217:3390.
-----------------------ggaacatagtgggagattc-
>gnl|SRA|SRR393016.7151619.1:1-19 HWI-EAS397_0015:6:48:8309:17229.
-----------------------ggaacatagtgggagattc-
>gnl|SRA|SRR393016.3788638.1:1-19 HWI-EAS397_0015:6:25:9170:7312.
-----------------------ggaacatagtgggagattc-
>gnl|SRA|SRR393016.1904743.1:1-19 HWI-EAS397_0015:6:13:8270:2173.
-----------------------ggaacatagtgggagattc-
>_R_gnl|SRA|SRR393016.1411189.1:2-38 HWI-EAS397_0015:6:10:2929:13414.
--gcttgcaaaacgcaaacag---gaccatagtgggagattc-
>_R_gnl|SRA|SRR393016.3962449.1:2-38 HWI-EAS397_0015:6:26:11844:9969.
--gcttgcaaaacgcaaacac---gaacatagtgggagattc-
>gnl|SRA|SRR393016.2952311.1:1-37 HWI-EAS397_0015:6:19:18589:8397.
--gcttgcaaaacgcaaacac---gaacatagtgggagattc-
>gnl|SRA|SRR393016.15813926.1:1-36 HWI-EAS397_0015:6:103:15054:12068.
---cttgcaaaacgcaaacag---gaccatagtgggagattc-
>_R_gnl|SRA|SRR393016.2709528.1:3-38 HWI-EAS397_0015:6:18:9128:3032.
---cttgcaaaacgcaaacag---gaccatagtgggagattc-
>gnl|SRA|SRR393016.16149623.1:1-36 HWI-EAS397_0015:6:105:14852:21086.
---cttgcaaaacgcaaacac---gaacatagtgggagattc-
>_R_gnl|SRA|SRR393016.11591830.1:4-38 HWI-EAS397_0015:6:77:13398:18424.
----ttgcaaaacgcaaacag---gaccatagtgggagattc-
>_R_gnl|SRA|SRR393016.8980936.1:4-38 HWI-EAS397_0015:6:60:16130:7347.

----ttgcaaaacgcaaacag---gaccatagtgggagattc-
>_R_gnl|SRA|SRR393016.17889978.1:4-38 HWI-EAS397_0015:6:116:14743:14808.
----ttgcaaaacgcaaacac---gaacatagtgggagattc-
>_R_gnl|SRA|SRR393016.9850390.1:4-38 HWI-EAS397_0015:6:66:9562:15263.
----ttgcaaaacgcaaacac---gaacatagtgggagattc-
>_R_gnl|SRA|SRR393016.258400.1:4-38 HWI-EAS397_0015:6:2:12507:6596.
----ttgcaaaacgcaaacac---gaacatagtgggagattc-
>gnl|SRA|SRR393016.16140798.1:1-33 HWI-EAS397_0015:6:105:13906:1090.
------gcaaaacgcaaacag---gaccatagtgggagattc-
>gnl|SRA|SRR393016.15908418.1:1-33 HWI-EAS397_0015:6:104:7288:6655.
------gcaaaacgcaaacag---gaccatagtgggagattc-
>_R_gnl|SRA|SRR393016.15097156.1:6-38 HWI-EAS397_0015:6:99:9424:17449.
------gcaaaacgcaaacag---gaccatagtgggagattc-
>gnl|SRA|SRR393016.13951627.1:1-33 HWI-EAS397_0015:6:92:9557:7552.
------gcaaaacgcaaacag---gaccatagtgggagattc-
>gnl|SRA|SRR393016.12879348.1:1-33 HWI-EAS397_0015:6:85:15148:11170.
------gcaaaacgcaaacag---gaccatagtgggagattc-
>gnl|SRA|SRR393016.12415091.1:1-33 HWI-EAS397_0015:6:82:16428:2010.
------gcaaaacgcaaacag---gaccatagtgggagattc-
>gnl|SRA|SRR393016.4727676.1:1-33 HWI-EAS397_0015:6:31:16748:3830.
------gcaaaacgcaaacag---gaccatagtgggagattc-
>gnl|SRA|SRR393016.3577088.1:1-33 HWI-EAS397_0015:6:24:1939:5701.
------gcaaaacgcaaacag---gaccatagtgggagattc-
>gnl|SRA|SRR393016.17135061.1:1-33 HWI-EAS397_0015:6:111:17920:14997.
------gcaaaacgcaaacac---gaacatagtgggagattc-
>gnl|SRA|SRR393016.13368934.1:1-33 HWI-EAS397_0015:6:88:16046:15954.
------gcaaaacgcaaacac---gaacatagtgggagattc-
>gnl|SRA|SRR393016.10832798.1:1-33 HWI-EAS397_0015:6:72:15709:8089.
------gcaaaacgcaaacac---gaacatagtgggagattc-
>gnl|SRA|SRR393016.6112609.1:1-33 HWI-EAS397_0015:6:41:8927:10894.
------gcaaaacgcaaacac---gaacatagtgggagattc-
>gnl|SRA|SRR393016.4230240.1:1-33 HWI-EAS397_0015:6:28:8740:5592.
------gcaaaacgcaaacac---gaacatagtgggagattc-
>gnl|SRA|SRR393016.2625022.1:1-33 HWI-EAS397_0015:6:17:16957:11538.
------gcaaaacgcaaacac---gaacatagtgggagattc-
>gnl|SRA|SRR393016.2394349.1:1-33 HWI-EAS397_0015:6:16:9567:11349.
------gcaaaacgcaaacac---gaacatagtgggagattc-
>gnl|SRA|SRR393016.13779682.1:1-32 HWI-EAS397_0015:6:91:8393:5446.
-------caaaacgcaaacag---gaccatagtgggagattc-
>gnl|SRA|SRR393016.3718647.1:1-32 HWI-EAS397_0015:6:24:18010:18105.
-------caaaacgcaaacac---gaacatagtgggagattc-
>gnl|SRA|SRR393016.9868638.1:1-31 HWI-EAS397_0015:6:66:11638:7170.
--------aaaacgcaaacag---gaccatagtgggagattc-
>_R_gnl|SRA|SRR393016.7795987.1:8-38 HWI-EAS397_0015:6:52:14721:15553.
--------aaaacgcaaacag---gaccatagtgggagattc-
>_R_gnl|SRA|SRR393016.16140268.1:8-38 HWI-EAS397_0015:6:105:13850:3736.
--------aaaacgcaaacac---gaacatagtgggagattc-
>gnl|SRA|SRR393016.9479956.1:1-31 HWI-EAS397_0015:6:64:2353:9267.
--------aaaacgcaaacac---gaacatagtgggagattc-
>_R_gnl|SRA|SRR393016.17690124.1:9-38 HWI-EAS397_0015:6:115:9850:14177.
---------aaacgcaaacag---gaccatagtgggagattc-

>_R_gnl|SRA|SRR393016.12055382.1:9-38 HWI-EAS397_0015:6:80:11915:9693.
---------aaacgcaaacag---gaccatagtgggagattc-
>_R_gnl|SRA|SRR393016.18020464.1:9-38 HWI-EAS397_0015:6:117:11820:12753.
---------aaacgcaaacac---gaacatagtgggagattc-
>gnl|SRA|SRR393016.15970551.1:1-29 HWI-EAS397_0015:6:104:13699:3671.
----------aacgcaaacag---gaccatagtgggagattc-
>_R_gnl|SRA|SRR393016.11340506.1:10-38 HWI-EAS397_0015:6:76:2748:9828.
----------aacgcaaacac---gaacatagtgggagattc-
>gnl|SRA|SRR393016.8471984.1:1-29 HWI-EAS397_0015:6:57:7695:8163.
----------aacgcaaacac---gaacatagtgggagattc-
>_R_gnl|SRA|SRR393016.16531342.1:11-38 HWI-EAS397_0015:6:108:3866:5548.
-----------acgcaaacag---gaccatagtgggagattc-
>gnl|SRA|SRR393016.6790533.1:1-26 HWI-EAS397_0015:6:45:17421:18684.
-------------gcaaacag---gaccatagtgggagattc-
>gnl|SRA|SRR393016.18376305.1:1-27 HWI-EAS397_0015:6:119:16667:6057.
------------cgcaaacac---gaacatagtgggagattc-
>gnl|SRA|SRR393016.6572535.1:1-27 HWI-EAS397_0015:6:44:9861:18341.
------------cgcaaacac---gaacatagtgggagattc-
>gnl|SRA|SRR393016.4253687.1:1-27 HWI-EAS397_0015:6:28:11280:1086.
------------cgcaaacac---gaacatagtgggagattc-
>gnl|SRA|SRR393016.13961220.1:5-24 HWI-EAS397_0015:6:92:10594:2531.
----------------------aggaccatagtgggagattc-
>_R_gnl|SRA|SRR393016.13842418.1:17-36 HWI-EAS397_0015:6:91:15286:11531.
----------------------aggaccatagtgggagattc-
>gnl|SRA|SRR393016.12142442.1:2-21 HWI-EAS397_0015:6:81:3975:17403.
----------------------aggaccatagtgggagattc-
>gnl|SRA|SRR393016.11396509.1:6-25 HWI-EAS397_0015:6:76:9148:11571.
----------------------aggaccatagtgggagattc-
>gnl|SRA|SRR393016.9506324.1:2-21 HWI-EAS397_0015:6:64:5453:11707.
----------------------aggaccatagtgggagattc-
>gnl|SRA|SRR393016.1157883.1:5-24 HWI-EAS397_0015:6:8:9250:19575.
----------------------aggaccatagtgggagattc-
>gnl|SRA|SRR393016.15406013.1:1-38 HWI-EAS397_0015:6:101:6946:14398.
---cttgcaaaacgcaaacactaggaacatagtgggagatt--
>gnl|SRA|SRR393016.10660464.1:1-38 HWI-EAS397_0015:6:71:13844:14941.
---cttgcaaaacgcaaacactaggaacatagtgggagatt--
>gnl|SRA|SRR393016.5407354.1:1-38 HWI-EAS397_0015:6:36:12379:11917.
---cttgcaaaacgcaaacaccaggaacatagtgggagatt--
>gnl|SRA|SRR393016.12413445.1:1-38 HWI-EAS397_0015:6:82:16242:15832.
--gcttgcaaaacgcaaacactaggaacatagtgggagat---
>gnl|SRA|SRR393016.11604740.1:1-38 HWI-EAS397_0015:6:77:14858:13646.
--gcttgcaaaacgcaaacactaggaacatagtgggagat---
>gnl|SRA|SRR393016.9495682.1:1-38 HWI-EAS397_0015:6:64:4216:13163.
--gcttgcaaaacgcaaacactaggaacatagtgggagat---
>_R_gnl|SRA|SRR393016.1355236.1:1-38 HWI-EAS397_0015:6:9:13940:2638.
--gcttgcaaaacgcaaacactaggaacatagtgggagat---

SRX058607 ZMM2, ZMM23
>contrZMM2 .
cagcttgcaaaacgcaaacactaggaacatagtgggagattc
>_R_gnl|SRA|SRR189772.8144473.1:1-35 HWUSI-EAS1599_0024:1:56:10794:18854.
-------caaaacgcaaacactaggaacatagtgggagattc
>gnl|SRA|SRR189772.15028636.1:1-35 HWUSI-EAS1599_0024:1:97:16730:15625.
-------caaaacgcaaacactaggaacatagtgggagattc
>gnl|SRA|SRR189772.13907063.1:1-35 HWUSI-EAS1599_0024:1:91:7429:13293.
-------caaaacgcaaacactaggaacatagtgggagattc
>gnl|SRA|SRR189772.515889.1:1-35 HWUSI-EAS1599_0024:1:3:18237:12895.
-------caaaacgcaaacactaggaacatagtgggagattc
>_R_gnl|SRA|SRR189772.14589194.1:3-35 HWUSI-EAS1599_0024:1:95:6530:9349.
---------aaacgcaaacactaggaacatagtgggagattc
>_R_gnl|SRA|SRR189772.11038260.1:3-35 HWUSI-EAS1599_0024:1:73:14913:1049.
---------aaacgcaaacactaggaacatagtgggagattc
>_R_gnl|SRA|SRR189772.9488994.1:3-35 HWUSI-EAS1599_0024:1:64:6339:11312.
---------aaacgcaaacactaggaacatagtgggagattc
>_R_gnl|SRA|SRR189772.1135742.1:3-35 HWUSI-EAS1599_0024:1:7:8948:17214.
---------aaacgcaaacactaggaacatagtgggagattc
>gnl|SRA|SRR189772.17319587.1:1-31 HWUSI-EAS1599_0024:1:111:8016:8042.
-----------acgcaaacactaggaacatagtgggagattc
>gnl|SRA|SRR189772.15427101.1:1-31 HWUSI-EAS1599_0024:1:100:4554:9415.
-----------acgcaaacactaggaacatagtgggagattc
>gnl|SRA|SRR189772.12188752.1:1-31 HWUSI-EAS1599_0024:1:80:18806:3645.
-----------acgcaaacactaggaacatagtgggagattc
>gnl|SRA|SRR189772.11993210.1:1-31 HWUSI-EAS1599_0024:1:79:16179:1290.
-----------acgcaaacactaggaacatagtgggagattc
>gnl|SRA|SRR189772.2979524.1:1-31 HWUSI-EAS1599_0024:1:17:16298:20570.
-----------acgcaaacactaggaacatagtgggagattc
>gnl|SRA|SRR189772.18721108.1:1-29 HWUSI-EAS1599_0024:1:119:14540:5383.
-------------gcaaacactaggaacatagtgggagattc
>gnl|SRA|SRR189772.17514212.1:1-29 HWUSI-EAS1599_0024:1:112:10980:7202.
-------------gcaaacactaggaacatagtgggagattc
>gnl|SRA|SRR189772.12741663.1:3-31 HWUSI-EAS1599_0024:1:84:10635:12315.
-------------gcaaacactaggaacatagtgggagattc
>gnl|SRA|SRR189772.12718417.1:1-29 HWUSI-EAS1599_0024:1:84:8078:3024.
-------------gcaaacactaggaacatagtgggagattc
>gnl|SRA|SRR189772.65104.1:1-27 HWUSI-EAS1599_0024:1:1:8007:18469.
-------------gcaaacactaggaacatagtgggagat--
>gnl|SRA|SRR189772.11685746.1:1-35 HWUSI-EAS1599_0024:1:78:1103:10969.
------gcaaaacgcaaacactaggaacatagtgggagatt-
>gnl|SRA|SRR189772.9550623.1:1-35 HWUSI-EAS1599_0024:1:64:12902:7149.
------gcaaaacgcaaacactaggaacatagtgggagatt-
>_R_gnl|SRA|SRR189772.11723437.1:1-34 HWUSI-EAS1599_0024:1:78:5232:8121.
cagcttgcaaaacgcaaacactaggaacatagtg--------
>_R_gnl|SRA|SRR189772.11128315.1:1-34 HWUSI-EAS1599_0024:1:74:12401:18000.
cagcttgcaaaacgcaaacactaggaacatagtg--------
>gnl|SRA|SRR189772.8135120.1:3-35 HWUSI-EAS1599_0024:1:56:9827:8324.
cagcttgcaaaacgcaaacactaggaacatagt---------
>gnl|SRA|SRR189772.11511072.1:7-35 HWUSI-EAS1599_0024:1:76:19311:8484.

cagcttgcaaaacgcaaacactaggaaca-------------
>gnl|SRA|SRR189772.18784121.1:1-35 HWUSI-EAS1599_0024:1:120:3209:17798.
---cttgcaaaacgcaaacactaggaacatagtgggag----
>gnl|SRA|SRR189772.7085422.1:1-35 HWUSI-EAS1599_0024:1:47:3646:14282.
---cttgcaaaacgcaaacactaggaacatagtgggag----
>_R_gnl|SRA|SRR189772.7956768.1:4-35 HWUSI-EAS1599_0024:1:55:9675:11980.
---cttgcaaaacgcaaacactaggaacatagtgg-------
>gnl|SRA|SRR189772.4440433.1:1-35 HWUSI-EAS1599_0024:1:25:18196:15488.
-agcttgcaaaacgcaaacactaggaacatagtggg------
>_R_gnl|SRA|SRR189772.18488075.1:3-35 HWUSI-EAS1599_0024:1:118:7645:2797.
--gcttgcaaaacgcaaacactaggaacatagtgg-------
>contrZMM23 .
tagcttgcaaaacgcaaacagtaggaccatagtgggagattc
>gnl|SRA|SRR189772.15436611.1:1-35 HWUSI-EAS1599_0024:1:100:5570:20500.
-agcttgcaaaacgcaaacagtaggaccatagtggg------
>gnl|SRA|SRR189772.2849058.1:1-35 HWUSI-EAS1599_0024:1:17:2842:2052.
-agcttgcaaaacgcaaacagtaggaccatagtggg------
>_R_gnl|SRA|SRR189772.18618009.1:1-32 HWUSI-EAS1599_0024:1:119:3529:4926.
-agcttgcaaaacgcaaacagtaggaccatagt---------
>_R_gnl|SRA|SRR189772.12167126.1:1-32 HWUSI-EAS1599_0024:1:80:16402:21306.
-agcttgcaaaacgcaaacagtaggaccatagt---------
>_R_gnl|SRA|SRR189772.16557257.1:8-35 HWUSI-EAS1599_0024:1:106:16479:12195.
--------------caaacagtaggaccatagtgggagattc
>_R_gnl|SRA|SRR189772.5756437.1:8-35 HWUSI-EAS1599_0024:1:33:5158:10055.
--------------caaacagtaggaccatagtgggagattc
>_R_gnl|SRA|SRR189772.885249.1:8-35 HWUSI-EAS1599_0024:1:6:1241:15696.
--------------caaacagtaggaccatagtgggagattc
>_R_gnl|SRA|SRR189772.15186005.1:1-33 HWUSI-EAS1599_0024:1:98:15202:20269.
cagcttgcaaaacgcaaacac---gaacatagtggg------
>_R_gnl|SRA|SRR189772.2133312.1:1-33 HWUSI-EAS1599_0024:1:13:2136:2059.
cagcttgcaaaacgcaaacac---gaacatagtggg------
>_R_gnl|SRA|SRR189772.11213462.1:1-35 HWUSI-EAS1599_0024:1:75:5827:6271.
----ttgcaaaacgcaaacac---gaacatagtgggagattc
>_R_gnl|SRA|SRR189772.1518107.1:1-35 HWUSI-EAS1599_0024:1:9:11851:20518.
----ttgcaaaacgcaaacac---gaacatagtgggagattc
>_R_gnl|SRA|SRR189772.13899621.1:5-35 HWUSI-EAS1599_0024:1:91:6662:4035.
--------aaaacgcaaacac---gaacatagtgggagattc
>_R_gnl|SRA|SRR189772.6787501.1:3-35 HWUSI-EAS1599_0024:1:38:18190:20338.
------gcaaaacgcaaacag---gaccatagtgggagattc

SRX058605 ZMM2

>control .
cagcttgcaaaacgcaaacactaggaacatagtgggagattc
>_R_gnl|SRA|SRR189770.28190125.1:1-35 HWI-EAS216_0001:2:114:3922:2181.
-----tgcaaaacgcaaacactaggaacatagtgggagat--
>gnl|SRA|SRR189770.18301126.1:1-35 HWI-EAS216_0001:2:74:11066:16447.
-----tgcaaaacgcaaacactaggaacatagtgggagat--
>gnl|SRA|SRR189770.17795404.1:1-35 HWI-EAS216_0001:2:72:7551:18188.

-----tgcaaaacgcaaacactaggaacatagtgggagat--
>gnl|SRA|SRR189770.6848526.1:1-35 HWI-EAS216_0001:2:27:18105:13419.
-----tgcaaaacgcaaacactaggaacatagtgggagat--
>gnl|SRA|SRR189770.4504513.1:1-35 HWI-EAS216_0001:2:18:14763:21087.
-----tgcaaaacgcaaacactaggaacatagtgggagat--
>gnl|SRA|SRR189770.1833077.1:16-35 HWI-EAS216_0001:2:8:6108:4913.
-------caaaacgcaaacactaggaa---------------
>_R_gnl|SRA|SRR189770.25852002.1:1-35 HWI-EAS216_0001:2:104:16761:6421.
---cttgcaaaacgcaaacactaggaacatagtgggag----
>_R_gnl|SRA|SRR189770.15630271.1:1-35 HWI-EAS216_0001:2:63:7721:5653.
---cttgcaaaacgcaaacactaggaacatagtgggag----
>_R_gnl|SRA|SRR189770.11319840.1:1-35 HWI-EAS216_0001:2:45:12028:2644.
---cttgcaaaacgcaaacactaggaacatagtgggag----
>_R_gnl|SRA|SRR189770.5737433.1:1-35 HWI-EAS216_0001:2:23:11726:14574.
---cttgcaaaacgcaaacactaggaacatagtgggag----
>_R_gnl|SRA|SRR189770.1333998.1:1-35 HWI-EAS216_0001:2:6:6713:3198.
---cttgcaaaacgcaaacactaggaacatagtgggag----
>gnl|SRA|SRR189770.7516338.1:1-35 HWI-EAS216_0001:2:30:11451:13214.
---cttgcaaaacgcaaacactaggaacatagtgggag----
>gnl|SRA|SRR189770.6650727.1:1-35 HWI-EAS216_0001:2:27:3940:19131.
---cttgcaaaacgcaaacactaggaacatagtgggag----
>_R_gnl|SRA|SRR189770.1690001.1:1-35 HWI-EAS216_0001:2:7:14033:16893.
--gcttgcaaaacgcaaacactaggaacatagtggga-----
>_R_gnl|SRA|SRR189770.27448026.1:1-35 HWI-EAS216_0001:2:111:4759:4117.
cagcttgcaaaacgcaaacactaggaacatagtgg-------
>_R_gnl|SRA|SRR189770.4963102.1:1-35 HWI-EAS216_0001:2:20:11066:1486.
cagcttgcaaaacgcaaacactaggaacatagtgg-------
>gnl|SRA|SRR189770.29609574.1:1-35 HWI-EAS216_0001:2:119:15670:11506.
cagcttgcaaaacgcaaacactaggaacatagtgg-------
>gnl|SRA|SRR189770.20318113.1:1-35 HWI-EAS216_0001:2:82:14847:15979.
cagcttgcaaaacgcaaacactaggaacatagtgg-------
>gnl|SRA|SRR189770.4935621.1:1-35 HWI-EAS216_0001:2:20:9112:11236.
cagcttgcaaaacgcaaacactaggaacatagtgg-------
>_R_gnl|SRA|SRR189770.20576521.1:1-34 HWI-EAS216_0001:2:83:15670:4546.
cagcttgcaaaacgcaaacactaggaacatagtg--------
>gnl|SRA|SRR189770.29763982.1:2-35 HWI-EAS216_0001:2:120:8843:12519.
cagcttgcaaaacgcaaacactaggaacatagtg--------
>gnl|SRA|SRR189770.9124659.1:3-35 HWI-EAS216_0001:2:36:17458:18056.
cagcttgcaaaacgcaaacactaggaacatagt---------
>gnl|SRA|SRR189770.25984231.1:4-35 HWI-EAS216_0001:2:105:8240:7642.
cagcttgcaaaacgcaaacactaggaacatag----------
>gnl|SRA|SRR189770.19903233.1:4-35 HWI-EAS216_0001:2:81:2846:9981.
cagcttgcaaaacgcaaacactaggaacatag----------
>gnl|SRA|SRR189770.7368721.1:5-35 HWI-EAS216_0001:2:29:19062:8139.
cagcttgcaaaacgcaaacactaggaacata-----------
>_R_gnl|SRA|SRR189770.14863010.1:1-29 HWI-EAS216_0001:2:60:3902:11026.
cagcttgcaaaacgcaaacactaggaaca-------------
>_R_gnl|SRA|SRR189770.26705149.1:1-35 HWI-EAS216_0001:2:108:5697:9927.
-agcttgcaaaacgcaaacactaggaacatagtggg------
>gnl|SRA|SRR189770.13284182.1:1-35 HWI-EAS216_0001:2:53:13540:5330.
cagcttgcaaaacgcaaacac---gaacatagtgggag----

>gnl|SRA|SRR189770.20462796.1:1-35 HWI-EAS216_0001:2:83:7455:16963.
------gcaaaacgcaaacactaggaacatagtgggagatt-
>gnl|SRA|SRR189770.5671000.1:1-35 HWI-EAS216_0001:2:23:6965:1717.
-------caaaacgcaaacactaggaacatagtgggagattc
>_R_gnl|SRA|SRR189770.28597212.1:2-35 HWI-EAS216_0001:2:115:14958:4714.
--------aaaacgcaaacactaggaacatagtgggagattc
>_R_gnl|SRA|SRR189770.21973353.1:2-35 HWI-EAS216_0001:2:89:9146:17290.
--------aaaacgcaaacactaggaacatagtgggagattc
>_R_gnl|SRA|SRR189770.19756147.1:2-35 HWI-EAS216_0001:2:80:10056:18280.
--------aaaacgcaaacactaggaacatagtgggagattc
>_R_gnl|SRA|SRR189770.14349553.1:2-35 HWI-EAS216_0001:2:58:1521:8358.
--------aaaacgcaaacactaggaacatagtgggagattc
>_R_gnl|SRA|SRR189770.13754890.1:2-35 HWI-EAS216_0001:2:55:12127:10996.
--------aaaacgcaaacactaggaacatagtgggagattc
>_R_gnl|SRA|SRR189770.2445035.1:2-35 HWI-EAS216_0001:2:10:13551:10468.
--------aaaacgcaaacactaggaacatagtgggagattc
>_R_gnl|SRA|SRR189770.28765388.1:4-35 HWI-EAS216_0001:2:116:9072:21342.
----------aacgcaaacactaggaacatagtgggagattc
>_R_gnl|SRA|SRR189770.23807646.1:4-35 HWI-EAS216_0001:2:96:14510:16979.
----------aacgcaaacactaggaacatagtgggagattc
>gnl|SRA|SRR189770.21999223.1:1-30 HWI-EAS216_0001:2:89:10983:2065.
------------cgcaaacactaggaacatagtgggagattc
>gnl|SRA|SRR189770.20315612.1:1-30 HWI-EAS216_0001:2:82:14664:2181.
------------cgcaaacactaggaacatagtgggagattc
>gnl|SRA|SRR189770.18776878.1:1-30 HWI-EAS216_0001:2:76:10302:19818.
------------cgcaaacactaggaacatagtgggagattc
>gnl|SRA|SRR189770.15301169.1:1-30 HWI-EAS216_0001:2:61:18949:5727.
------------cgcaaacactaggaacatagtgggagattc
>gnl|SRA|SRR189770.4312642.1:1-30 HWI-EAS216_0001:2:17:19288:13366.
------------cgcaaacactaggaacatagtgggagattc
>gnl|SRA|SRR189770.29807527.1:1-29 HWI-EAS216_0001:2:120:11929:12338.
-------------gcaaacactaggaacatagtgggagattc
>gnl|SRA|SRR189770.17994300.1:1-29 HWI-EAS216_0001:2:73:4899:20755.
-------------gcaaacactaggaacatagtgggagattc
>_R_gnl|SRA|SRR189770.20928636.1:2-35 HWI-EAS216_0001:2:85:5539:2542.
-----tgcaaaacgcaaacac---gaacatagtgggagattc
>_R_gnl|SRA|SRR189770.16190546.1:2-35 HWI-EAS216_0001:2:65:13882:17270.
-----tgcaaaacgcaaacac---gaacatagtgggagattc
>_R_gnl|SRA|SRR189770.13986727.1:2-35 HWI-EAS216_0001:2:56:11180:5088.
-----tgcaaaacgcaaacac---gaacatagtgggagattc
>_R_gnl|SRA|SRR189770.12736489.1:2-35 HWI-EAS216_0001:2:51:9653:8415.
-----tgcaaaacgcaaacac---gaacatagtgggagattc
>_R_gnl|SRA|SRR189770.478875.1:3-35 HWI-EAS216_0001:2:2:17723:10621.
------gcaaaacgcaaacac---gaacatagtgggagattc
>gnl|SRA|SRR189770.14243108.1:1-33 HWI-EAS216_0001:2:57:12051:11243.
------gcaaaacgcaaacac---gaacatagtgggagattc
>_R_gnl|SRA|SRR189770.25121684.1:4-35 HWI-EAS216_0001:2:101:18870:8101.
-------caaaacgcaaacac---gaacatagtgggagattc
>_R_gnl|SRA|SRR189770.24110340.1:4-35 HWI-EAS216_0001:2:97:18276:13898.
-------caaaacgcaaacac---gaacatagtgggagattc
>_R_gnl|SRA|SRR189770.8411226.1:4-35 HWI-EAS216_0001:2:34:2530:17068.

-------caaaacgcaaacac---gaacatagtgggagattc
>_R_gnl|SRA|SRR189770.5310534.1:4-35 HWI-EAS216_0001:2:21:17600:4958.
-------caaaacgcaaacac---gaacatagtgggagattc
>gnl|SRA|SRR189770.16673331.1:1-32 HWI-EAS216_0001:2:67:13962:15545.
-------caaaacgcaaacac---gaacatagtgggagattc
>gnl|SRA|SRR189770.4849579.1:1-32 HWI-EAS216_0001:2:20:2782:2126.
-------caaaacgcaaacac---gaacatagtgggagattc
>_R_gnl|SRA|SRR189770.4012710.1:2-35 HWI-EAS216_0001:2:16:16099:9871.
-----tgcaaaacgcgaacac---gaacatagtgggagattc

SRX058605 ZMM23
>control .
tagcttgcaaaacgcaaacagtaggaccatagtgggagattc
>gnl|SRA|SRR189770.685977.1:1-35 HWI-EAS216_0001:2:3:14473:1678.
------gcaaaacgcaaacagtaggaccatagtgggagatt-
>_R_gnl|SRA|SRR189770.25421958.1:1-35 HWI-EAS216_0001:2:103:4143:20404.
----ttgcaaaacgcaaac---aggaccatagtgggagattc
>gnl|SRA|SRR189770.27126893.1:1-33 HWI-EAS216_0001:2:109:17859:10078.
------gcaaaacgcaaac---aggaccatagtgggagattc
>_R_gnl|SRA|SRR189770.24655248.1:4-35 HWI-EAS216_0001:2:100:3361:4821.
-------caaaacgcaaac---aggaccatagtgggagattc
>gnl|SRA|SRR189770.15936600.1:1-32 HWI-EAS216_0001:2:64:12766:6076.
-------caaaacgcaaac---aggaccatagtgggagattc

SRX058604 ZMM2, ZMM23
>contrZMM2 .
cagcttgcaaaacgcaaacactaggaacatagtgggagattc-
>gnl|SRA|SRR189769.15365023.1:1-35 HWI-EAS216_0001:1:70:11729:15401.
------gcaaaacgcaaacactaggaacatagtgggagatt--
>gnl|SRA|SRR189769.13369164.1:1-35 HWI-EAS216_0001:1:61:11409:1772.
------gcaaaacgcaaacactaggaacatagtgggagatt--
>gnl|SRA|SRR189769.13571968.1:1-35 HWI-EAS216_0001:1:62:10190:5532.
-----tgcaaaacgcaaacactaggaacatagtgggagat---
>_R_gnl|SRA|SRR189769.7630104.1:1-35 HWI-EAS216_0001:1:36:1188:15083.
-----tgcaaaacgcaaacactaggaacatagtgggagat---
>_R_gnl|SRA|SRR189769.12100005.1:1-35 HWI-EAS216_0001:1:55:14187:13379.
--gcttgcaaaacgcaaacactaggaacatagtggga------
>gnl|SRA|SRR189769.3821063.1:1-35 HWI-EAS216_0001:1:17:8828:8058.
--gcttgcaaaacgcaaacactaggaacatagtggga------
>gnl|SRA|SRR189769.8600738.1:1-35 HWI-EAS216_0001:1:40:5275:13125.
---cttgcaaaacgcaaacactaggaacatagtgggag-----
>_R_gnl|SRA|SRR189769.1636383.1:1-35 HWI-EAS216_0001:1:8:2558:4457.
---cttgcaaaacgcaaacactaggaacatagtgggag-----
>_R_gnl|SRA|SRR189769.7166971.1:1-35 HWI-EAS216_0001:1:33:19604:19347.
-------caaaacgcaaacactaggaacatagtgggagattc-
>gnl|SRA|SRR189769.4815988.1:1-35 HWI-EAS216_0001:1:21:13427:8889.
-------caaaacgcaaacactaggaacatagtgggagattc-
>_R_gnl|SRA|SRR189769.10713180.1:2-35 HWI-EAS216_0001:1:49:10499:5355.
--------aaaacgcaaacactaggaacatagtgggagattc-

>gnl|SRA|SRR189769.7324184.1:1-34 HWI-EAS216_0001:1:34:13882:9992.
--------aaaacgcaaacactaggaacatagtgggagattc-
>gnl|SRA|SRR189769.3457755.1:1-34 HWI-EAS216_0001:1:15:16985:15659.
--------aaaacgcaaacactaggaacatagtgggagattc-
>gnl|SRA|SRR189769.10576119.1:1-32 HWI-EAS216_0001:1:48:17661:20724.
----------aacgcaaacactaggaacatagtgggagattc-
>gnl|SRA|SRR189769.25526754.1:1-30 HWI-EAS216_0001:1:114:13140:11379.
------------cgcaaacactaggaacatagtgggagattc-
>gnl|SRA|SRR189769.22359396.1:1-30 HWI-EAS216_0001:1:101:1793:14105.
------------cgcaaacactaggaacatagtgggagattc-
>gnl|SRA|SRR189769.11784019.1:1-30 HWI-EAS216_0001:1:54:6295:1405.
------------cgcaaacactaggaacatagtgggagattc-
>gnl|SRA|SRR189769.11628900.1:1-30 HWI-EAS216_0001:1:53:11794:3638.
------------cgcaaacactaggaacatagtgggagattc-
>gnl|SRA|SRR189769.2127392.1:1-30 HWI-EAS216_0001:1:10:4572:2334.
------------cgcaaacactaggaacatagtgggagattc-
>gnl|SRA|SRR189769.25991086.1:6-34 HWI-EAS216_0001:1:116:12839:20836.
-------------gcaaacactaggaacatagtgggagattc-
>gnl|SRA|SRR189769.3890113.1:1-29 HWI-EAS216_0001:1:17:14218:5160.
-------------gcaaacactaggaacatagtgggagattc-
>gnl|SRA|SRR189769.1873646.1:7-35 HWI-EAS216_0001:1:9:2920:13966.
-------------gcaaacactaggaacatagtgggagattc-
>gnl|SRA|SRR189769.25595215.1:1-28 HWI-EAS216_0001:1:114:18484:4586.
--------------caaacactaggaacatagtgggagattc-
>gnl|SRA|SRR189769.22529432.1:3-30 HWI-EAS216_0001:1:101:14971:4652.
--------------caaacactaggaacatagtgggagattc-
>gnl|SRA|SRR189769.14448921.1:1-28 HWI-EAS216_0001:1:66:9848:3201.
--------------caaacactaggaacatagtgggagattc-
>_R_gnl|SRA|SRR189769.14250629.1:8-35 HWI-EAS216_0001:1:65:11686:18778.
--------------caaacactaggaacatagtgggagattc-
>gnl|SRA|SRR189769.8597225.1:1-28 HWI-EAS216_0001:1:40:4993:4846.
--------------caaacactaggaacatagtgggagattc-
>gnl|SRA|SRR189769.5025920.1:1-28 HWI-EAS216_0001:1:22:11597:1388.
--------------caaacactaggaacatagtgggagattc-
>gnl|SRA|SRR189769.3621337.1:1-28 HWI-EAS216_0001:1:16:11495:3592.
--------------caaacactaggaacatagtgggagattc-
>gnl|SRA|SRR189769.5908391.1:1-35 HWI-EAS216_0001:1:26:7316:7694.
----ttgcaaaacgcaaacac---gaacatagtgggagattc-
>gnl|SRA|SRR189769.25746877.1:1-34 HWI-EAS216_0001:1:115:12056:4946.
-----tgcaaaacgcaaacac---gaacatagtgggagattc-
>_R_gnl|SRA|SRR189769.19447625.1:2-35 HWI-EAS216_0001:1:88:10314:19963.
-----tgcaaaacgcaaacac---gaacatagtgggagattc-
>_R_gnl|SRA|SRR189769.17251882.1:2-35 HWI-EAS216_0001:1:78:18139:1569.
-----tgcaaaacgcaaacac---gaacatagtgggagattc-
>_R_gnl|SRA|SRR189769.15936728.1:2-35 HWI-EAS216_0001:1:73:3520:13342.
-----tgcaaaacgcaaacac---gaacatagtgggagattc-
>gnl|SRA|SRR189769.6540529.1:1-34 HWI-EAS216_0001:1:29:2035:12165.
-----tgcaaaacgcaaacac---gaacatagtgggagattc-
>_R_gnl|SRA|SRR189769.17665231.1:3-35 HWI-EAS216_0001:1:80:14719:4317.
------gcaaaacgcaaacac---gaacatagtgggagattc-
>_R_gnl|SRA|SRR189769.26495510.1:9-35 HWI-EAS216_0001:1:118:15440:14698.

------------cgcaaacac---gaacatagtgggagattc-
>_R_gnl|SRA|SRR189769.1074553.1:9-35 HWI-EAS216_0001:1:5:13867:5250.
------------cgcaaacac---gaacatagtgggagattc-
>gnl|SRA|SRR189769.546814.1:1-25 HWI-EAS216_0001:1:3:9137:10770.
--------------caaacac---gaacatagtgggagattc-
>contrZMM23 .
tagcttgcaaaacgcaaacagtaggaccatagtgggagattc-
>gnl|SRA|SRR189769.6474167.1:1-35 HWI-EAS216_0001:1:28:15321:17811.
------gcaaaacgcaaacagtaggaccatagtgggagatt--
>gnl|SRA|SRR189769.5631616.1:1-34 HWI-EAS216_0001:1:25:3964:8981.
--------aaaacgcaaacagtaggaccatagtgggagattc-
>gnl|SRA|SRR189769.18219593.1:1-35 HWI-EAS216_0001:1:83:4185:13442.
----ttgcaaaacgcaaacag---gaccatagtgggagattc-
>gnl|SRA|SRR189769.24811903.1:1-33 HWI-EAS216_0001:1:111:10046:14465.
------gcaaaacgcaaacag---gaccatagtgggagattc-
>gnl|SRA|SRR189769.6011743.1:1-32 HWI-EAS216_0001:1:26:15449:1440.
-------caaaacgcaaacag---gaccatagtgggagattc-
>gnl|SRA|SRR189769.4350893.1:1-32 HWI-EAS216_0001:1:19:13584:3960.
-------caaaacgcaaacag---gaccatagtgggagattc-
>gnl|SRA|SRR189769.1688520.1:1-32 HWI-EAS216_0001:1:8:6819:8390.
-------caaaacgcaaacag---gaccatagtgggagattc-
>gnl|SRA|SRR189769.4851661.1:3-35 HWI-EAS216_0001:1:21:16231:4771.
cagcttgcaaaacgcaaacactaggaacatagt----------
>gnl|SRA|SRR189769.2664869.1:3-35 HWI-EAS216_0001:1:12:9965:15968.
cagcttgcaaaacgcaaacactaggaacatagt----------
>_R_gnl|SRA|SRR189769.23581944.1:1-32 HWI-EAS216_0001:1:106:5705:6059.
cagcttgcaaaacgcaaacactaggaacatag-----------
>gnl|SRA|SRR189769.16679792.1:4-35 HWI-EAS216_0001:1:76:8681:1553.
cagcttgcaaaacgcaaacactaggaacatag-----------
>gnl|SRA|SRR189769.19070701.1:5-35 HWI-EAS216_0001:1:86:16889:14055.
cagcttgcaaaacgcaaacactaggaacata------------
>_R_gnl|SRA|SRR189769.10946575.1:1-31 HWI-EAS216_0001:1:50:11119:8649.
cagcttgcaaaacgcaaacactaggaacata------------
>_R_gnl|SRA|SRR189769.5859480.1:1-31 HWI-EAS216_0001:1:26:3348:7613.
cagcttgcaaaacgcaaacactaggaacata------------
>_R_gnl|SRA|SRR189769.1855853.1:1-31 HWI-EAS216_0001:1:9:1345:20455.
cagcttgcaaaacgcaaacactaggaacata------------
>gnl|SRA|SRR189769.23019999.1:7-35 HWI-EAS216_0001:1:103:16635:13236.
cagcttgcaaaacgcaaacactaggaaca--------------
>_R_gnl|SRA|SRR189769.14480542.1:1-32 HWI-EAS216_0001:1:66:12404:4959.
cagcttgcaaaacgcaaacac---gaacatagtgg--------
>_R_gnl|SRA|SRR189769.10480792.1:1-32 HWI-EAS216_0001:1:48:9921:7800.
cagcttgcaaaacgcaaacac---gaacatagtgg--------
>_R_gnl|SRA|SRR189769.92894.1:1-32 HWI-EAS216_0001:1:1:9473:5041.
cagcttgcaaaacgcaaacac---gaacatagtgg--------
>_R_gnl|SRA|SRR189769.26843543.1:1-29 HWI-EAS216_0001:1:120:6158:10626.
cagcttgcaaaacgcaaacac---gaacatag-----------
>gnl|SRA|SRR189769.850967.1:9-35 HWI-EAS216_0001:1:4:14649:13962.
cagcttgcaaaacgcaaacac---gaacat-------------
>gnl|SRA|SRR189769.24479601.1:3-35 HWI-EAS216_0001:1:110:2553:20830.
-agcttgcaaaacgcaaacag---gaccatagtggga------

>gnl|SRA|SRR189769.26198450.1:7-35 HWI-EAS216_0001:1:117:10754:1358.
-agcttgcaaaacgcaaacag---gaccatagt----------
>_R_gnl|SRA|SRR189769.25067806.1:1-34 HWI-EAS216_0001:1:112:13796:12675.
-agcttgcaaaacgcaaacag---gaccatagtaggag-----
>_R_gnl|SRA|SRR189769.24245138.1:1-35 HWI-EAS216_0001:1:109:2584:19775.
--gcttgcaaaacgcaaacac---gaacatagtgggagat---
>_R_gnl|SRA|SRR189769.18088402.1:1-35 HWI-EAS216_0001:1:82:11894:10754.
--gcttgcaaaacgcaaacac---gaacatagtgggagat---
>_R_gnl|SRA|SRR189769.4902645.1:1-35 HWI-EAS216_0001:1:22:1697:20321.
--gcttgcaaaacgcaaacac---gaacatagtgggagat---
>gnl|SRA|SRR189769.8778551.1:1-27 HWI-EAS216_0001:1:40:19389:13558.
------gcaaaacgcaaacac---gaacatagcggg-------
>gnl|SRA|SRR189769.22677686.1:1-33 HWI-EAS216_0001:1:102:8379:3975.
------gcaaaacgcaaacag---gaccatagcgggagattc-
>_R_gnl|SRA|SRR189769.12292830.1:1-35 HWI-EAS216_0001:1:56:11945:4335.
-----tgcaaaacgcaaacactaggaacatagtgcgagat---
>gnl|SRA|SRR189769.12452106.1:9-35 HWI-EAS216_0001:1:57:6938:6628.
cagcttgcaaaactcaaacac---gaacat-------------
>gnl|SRA|SRR189769.350920.1:9-35 HWI-EAS216_0001:1:2:12068:7554.
cagcttgcaatacgcaaacactaggaa----------------

SRX058601 ZMM2, ZMM23
>contrZMM2 .
cagcttgcaaaacgcaaacactaggaacatagtgggagattc
>_R_gnl|SRA|SRR393017.17590643.1:1-38 HWI-EAS397_0015:7:111:16508:15737.
cagcttgcaaaacgcaaacactaggaacatagtgggag----
>_R_gnl|SRA|SRR393017.3760234.1:1-38 HWI-EAS397_0015:7:24:10770:11593.
cagcttgcaaaacgcaaacactaggaacatagtgggag----
>gnl|SRA|SRR393017.16628514.1:2-38 HWI-EAS397_0015:7:106:4677:6375.
cagcttgcaaaacgcaaacactaggaacatagtggga-----
>gnl|SRA|SRR393017.10742190.1:2-38 HWI-EAS397_0015:7:70:9017:2434.
cagcttgcaaaacgcaaacactaggaacatagtggga-----
>gnl|SRA|SRR393017.16626459.1:3-38 HWI-EAS397_0015:7:106:4452:5822.
cagcttgcaaaacgcaaacactaggaacatagtggg------
>_R_gnl|SRA|SRR393017.18606196.1:1-35 HWI-EAS397_0015:7:117:14656:11829.
cagcttgcaaaacgcaaacactaggaacatagtgg-------
>_R_gnl|SRA|SRR393017.12875818.1:1-35 HWI-EAS397_0015:7:83:9682:3000.
cagcttgcaaaacgcaaacactaggaacatagtgg-------
>_R_gnl|SRA|SRR393017.6168462.1:1-35 HWI-EAS397_0015:7:40:3033:8783.
cagcttgcaaaacgcaaacactaggaacatagtgg-------
>_R_gnl|SRA|SRR393017.8499196.1:1-31 HWI-EAS397_0015:7:55:16621:15198.
cagcttgcaaaacgcaaacactaggaacata-----------
>gnl|SRA|SRR393017.226785.1:9-38 HWI-EAS397_0015:7:2:8844:18656.
cagcttgcaaaacgcaaacactaggaacat------------
>gnl|SRA|SRR393017.7433388.1:1-27 HWI-EAS397_0015:7:48:11168:7566.
---cttgcaaaacgcaaacactaggaacat------------
>_R_gnl|SRA|SRR393017.5314055.1:1-38 HWI-EAS397_0015:7:34:10481:17638.
--gcttgcaaaacgcaaacactaggaacatagtgggagat--
>gnl|SRA|SRR393017.16006184.1:1-38 HWI-EAS397_0015:7:102:9301:14486.

---cttgcaaaacgcaaacactaggaacatagtgggagatt-
>_R_gnl|SRA|SRR393017.138225.1:15-38 HWI-EAS397_0015:7:1:16480:6118.
----------aacgcaaacactaggaacatagtg--------
>_R_gnl|SRA|SRR393017.15690721.1:1-32 HWI-EAS397_0015:7:100:11022:14225.
cagcttgcaaaacgcaaacac---gaacatagtgg-------
>_R_gnl|SRA|SRR393017.9685530.1:1-38 HWI-EAS397_0015:7:63:15498:16749.
----ttgcaaaacgcaaacactaggaacatagtgggagattc
>gnl|SRA|SRR393017.12141876.1:1-37 HWI-EAS397_0015:7:79:1762:4606.
-----tgcaaaacgcaaacactaggaacatagtgggagattc
>_R_gnl|SRA|SRR393017.12018731.1:2-38 HWI-EAS397_0015:7:78:6284:16623.
-----tgcaaaacgcaaacactaggaacatagtgggagattc
>_R_gnl|SRA|SRR393017.9970872.1:2-38 HWI-EAS397_0015:7:65:12262:7274.
-----tgcaaaacgcaaacactaggaacatagtgggagattc
>gnl|SRA|SRR393017.9110628.1:1-37 HWI-EAS397_0015:7:60:3308:18687.
-----tgcaaaacgcaaacactaggaacatagtgggagattc
>_R_gnl|SRA|SRR393017.2147850.1:2-38 HWI-EAS397_0015:7:14:11069:16272.
-----tgcaaaacgcaaacactaggaacatagtgggagattc
>gnl|SRA|SRR393017.17282431.1:1-36 HWI-EAS397_0015:7:110:2259:7854.
------gcaaaacgcaaacactaggaacatagtgggagattc
>gnl|SRA|SRR393017.13309104.1:1-36 HWI-EAS397_0015:7:86:3308:6672.
------gcaaaacgcaaacactaggaacatagtgggagattc
>gnl|SRA|SRR393017.12442682.1:1-36 HWI-EAS397_0015:7:80:16322:14780.
------gcaaaacgcaaacactaggaacatagtgggagattc
>gnl|SRA|SRR393017.10650258.1:1-36 HWI-EAS397_0015:7:69:16542:15582.
------gcaaaacgcaaacactaggaacatagtgggagattc
>gnl|SRA|SRR393017.8779979.1:1-36 HWI-EAS397_0015:7:57:15845:10751.
------gcaaaacgcaaacactaggaacatagtgggagattc
>gnl|SRA|SRR393017.7461403.1:1-36 HWI-EAS397_0015:7:48:14212:20346.
------gcaaaacgcaaacactaggaacatagtgggagattc
>gnl|SRA|SRR393017.7376822.1:1-36 HWI-EAS397_0015:7:48:4350:4803.
------gcaaaacgcaaacactaggaacatagtgggagattc
>gnl|SRA|SRR393017.3961798.1:1-36 HWI-EAS397_0015:7:25:15155:9034.
------gcaaaacgcaaacactaggaacatagtgggagattc
>gnl|SRA|SRR393017.2503224.1:1-36 HWI-EAS397_0015:7:16:14686:2055.
------gcaaaacgcaaacactaggaacatagtgggagattc
>gnl|SRA|SRR393017.7553914.1:1-35 HWI-EAS397_0015:7:49:8340:18736.
-------caaaacgcaaacactaggaacatagtgggagattc
>_R_gnl|SRA|SRR393017.2334035.1:4-38 HWI-EAS397_0015:7:15:13882:1697.
-------caaaacgcaaacactaggaacatagtgggagattc
>gnl|SRA|SRR393017.19097442.1:1-30 HWI-EAS397_0015:7:120:11186:4667.
------------cgcaaacactaggaacatagtgggagattc
>gnl|SRA|SRR393017.18396626.1:1-30 HWI-EAS397_0015:7:116:10976:19816.
------------cgcaaacactaggaacatagtgggagattc
>gnl|SRA|SRR393017.15306176.1:1-30 HWI-EAS397_0015:7:98:5669:7677.
------------cgcaaacactaggaacatagtgggagattc
>gnl|SRA|SRR393017.7927977.1:1-30 HWI-EAS397_0015:7:51:17192:13098.
------------cgcaaacactaggaacatagtgggagattc
>gnl|SRA|SRR393017.3569147.1:1-30 HWI-EAS397_0015:7:23:8315:16317.
------------cgcaaacactaggaacatagtgggagattc
>gnl|SRA|SRR393017.258624.1:1-30 HWI-EAS397_0015:7:2:12224:18377.
------------cgcaaacactaggaacatagtgggagattc

>gnl|SRA|SRR393017.5244279.1:1-29 HWI-EAS397_0015:7:34:2215:19294.
-------------gcaaacactaggaacatagtgggagattc
>_R_gnl|SRA|SRR393017.12926748.1:2-38 HWI-EAS397_0015:7:83:15038:4619.
--gcttgcaaaacgcaaacac---gaacatagtgggagattc
>_R_gnl|SRA|SRR393017.17453659.1:3-38 HWI-EAS397_0015:7:111:2337:11810.
---cttgcaaaacgcaaacac---gaacatagtgggagattc
>_R_gnl|SRA|SRR393017.6301324.1:4-38 HWI-EAS397_0015:7:40:17924:14974.
----ttgcaaaacgcaaacac---gaacatagtgggagattc
>gnl|SRA|SRR393017.5765792.1:1-33 HWI-EAS397_0015:7:37:9532:5566.
------gcaaaacgcaaacac---gaacatagtgggagattc
>_R_gnl|SRA|SRR393017.833318.1:8-38 HWI-EAS397_0015:7:6:6133:15267.
--------aaaacgcaaacac---gaacatagtgggagattc
>gnl|SRA|SRR393017.5530410.1:1-25 HWI-EAS397_0015:7:35:16911:20641.
--------------caaacac---gaacatagtgggagattc
>_R_gnl|SRA|SRR393017.14998857.1:15-38 HWI-EAS397_0015:7:96:8686:17264.
----------aacgcaaacactaggaacatggtg--------
>contrZMM23 .
tagcttgcaaaacgcaaacagtaggaccatagtgggagattc
>gnl|SRA|SRR393017.7776896.1:1-34 HWI-EAS397_0015:7:50:16569:14012.
-----tgcaaaacgcaaacag---gaccatagtgggagattc
>gnl|SRA|SRR393017.941477.1:1-27 HWI-EAS397_0015:7:6:17707:8383.
------------cgcaaacag---gaccatagtgggagattc
>_R_gnl|SRA|SRR393017.2444379.1:11-38 HWI-EAS397_0015:7:16:8504:9748.
---cttgcaaaacgcaaacac---gaacatggtg--------

SRX058599 ZMM2, ZMM23
>contrZMM2 .
cagcttgcaaaacgcaaacactaggaacatagtgggagattc
>_R_gnl|SRA|SRR189763.18271165.1:2-38 HWI-EAS397_0015:4:120:18847:4473.
-----tgcaaaacgcaaacactaggaacatagtgggagattc
>_R_gnl|SRA|SRR189763.15044037.1:2-38 HWI-EAS397_0015:4:99:18108:8723.
-----tgcaaaacgcaaacactaggaacatagtgggagattc
>_R_gnl|SRA|SRR189763.14750984.1:2-38 HWI-EAS397_0015:4:98:1884:12978.
-----tgcaaaacgcaaacactaggaacatagtgggagattc
>_R_gnl|SRA|SRR189763.9224123.1:2-38 HWI-EAS397_0015:4:61:11727:5903.
-----tgcaaaacgcaaacactaggaacatagtgggagattc
>gnl|SRA|SRR189763.14611478.1:1-35 HWI-EAS397_0015:4:97:3498:13094.
-------caaaacgcaaacactaggaacatagtgggagattc
>gnl|SRA|SRR189763.15438828.1:2-34 HWI-EAS397_0015:4:102:9942:19872.
---------aaacgcaaacactaggaacatagtgggagattc
>gnl|SRA|SRR189763.14398063.1:1-30 HWI-EAS397_0015:4:95:14117:11961.
------------cgcaaacactaggaacatagtgggagattc
>gnl|SRA|SRR189763.14951975.1:1-27 HWI-EAS397_0015:4:99:7255:9473.
---------------aaacactaggaacatagtgggagattc
>gnl|SRA|SRR189763.6164493.1:2-38 HWI-EAS397_0015:4:41:10645:19513.
cagcttgcaaaacgcaaacactaggaacatagtggga-----
>gnl|SRA|SRR189763.12762240.1:5-38 HWI-EAS397_0015:4:84:18002:18110.
cagcttgcaaaacgcaaacactaggaacatagtg--------
>_R_gnl|SRA|SRR189763.6631016.1:1-32 HWI-EAS397_0015:4:44:10913:18630.

cagcttgcaaaacgcaaacactaggaacatag----------
>_R_gnl|SRA|SRR189763.3181984.1:1-31 HWI-EAS397_0015:4:21:9869:7181.
cagcttgcaaaacgcaaacac---gaacatagtg--------
>_R_gnl|SRA|SRR189763.15187619.1:3-38 HWI-EAS397_0015:4:100:16558:16042.
---cttgcaaaacgcaaacac---gaacatagtgggagattc
>gnl|SRA|SRR189763.15021458.1:9-38 HWI-EAS397_0015:4:99:15244:16471.
cagcttgcaaaacgcaaacac---gaacatagt---------
>contrZMM23 .
tagcttgcaaaacgcaaacagtaggaccatagtgggagattc
>_R_gnl|SRA|SRR189763.2066366.1:6-38 HWI-EAS397_0015:4:14:5101:1908.
---------aaacgcaaacagtaggaccatagtgggagattc
>_R_gnl|SRA|SRR189763.3150948.1:1-30 HWI-EAS397_0015:4:21:6494:16968.
-agcttgcaaaacgcaaacagtaggaccata-----------
>gnl|SRA|SRR189763.8722027.1:7-38 HWI-EAS397_0015:4:58:5075:7526.
-agcttgcaaaacgcaaacag---gaccatagtggg------
>gnl|SRA|SRR189763.16597448.1:1-32 HWI-EAS397_0015:4:110:2825:16091.
-------caaaacgcaaacag---gaccatagtgggagattc
>_R_gnl|SRA|SRR189763.5537751.1:9-38 HWI-EAS397_0015:4:37:9267:8136.
---------aaacgcaaacag---gaccatagtgggagattc

SRX058597
>contrZMM2 .
cagcttgcaaaacgcaaacactaggaacatagtgggagattc
>_R_gnl|SRA|SRR189760.15501004.1:1-34 HWI-EAS397_0015:2:102:14934:7865.
cagcttgcaaaacgcaaacactaggaacatagtg--------
>_R_gnl|SRA|SRR189760.12078160.1:1-29 HWI-EAS397_0015:2:80:17262:10275.
cagcttgcaaaacgcaaacactaggaaca-------------
>gnl|SRA|SRR189760.6931334.1:12-38 HWI-EAS397_0015:2:46:14475:5107.
cagcttgcaaaacgcaaacactaggaa---------------
>gnl|SRA|SRR189760.14298139.1:1-29 HWI-EAS397_0015:2:95:3108:8315.
-------------gcaaacactaggaacatagtgggagattc
>_R_gnl|SRA|SRR189760.14117282.1:4-38 HWI-EAS397_0015:2:93:18081:12714.
----ttgcaaaacgcaaacac---gaacatagtgggagattc
>gnl|SRA|SRR189760.17439299.1:3-38 HWI-EAS397_0015:2:115:7332:8473.
cagcttgcaaaacgcaaacac---gaacatagtgggaga---
>contrZMM23 .
tagcttgcaaaacgcaaacagtaggaccatagtgggagattc
>gnl|SRA|SRR189760.14792614.1:2-38 HWI-EAS397_0015:2:98:5488:5207.
-agcttgcaaaacgcaaacag---gaccatagtgggagatt-
>_R_gnl|SRA|SRR189760.5683583.1:9-38 HWI-EAS397_0015:2:38:11939:7818.
---------aaacgcaaacag---gaccatagtgggagattc

SRX2375352; SRX2375353; SRX2375354
>control ..
tagcttgcagaactcaaacagtaggaacttagtgggggattc---
>_R_gnl|SRA|SRR5054410.21139670.2:68-109 21139670..
tagcttgcagaactcaaacagtaggaacttagtgggggattc---

>_R_gnl|SRA|SRR5054410.6241105.2:66-107 6241105..
tagcttgcagaactcaaacagtaggaacttagtgggggattc---
>gnl|SRA|SRR5054410.6241105.1:59-100 6241105..
tagcttgcagaactcaaacagtaggaacttagtgggggattc---
>_R_gnl|SRA|SRR5054410.5198614.2:66-107 5198614..
tagcttgcagaactcaaacagtaggaacttagtgggggattc---
>gnl|SRA|SRR5054410.5198614.1:59-100 5198614..
tagcttgcagaactcaaacagtaggaacttagtgggggattc---
>gnl|SRA|SRR5054409.22586830.1:63-104 22586830..
tagcttgcagaactcaaacagtaggaacttagtgggggattc---
>_R_gnl|SRA|SRR5054409.20593811.2:66-107 20593811..
tagcttgcagaactcaaacagtaggaacttagtgggggattc---
>_R_gnl|SRA|SRR5054409.7903509.2:77-118 7903509..
tagcttgcagaactcaaacagtaggaacttagtgggggattc---
>gnl|SRA|SRR5054409.7890060.1:60-101 7890060..
tagcttgcagaactcaaacagtaggaacttagtgggggattc---
>gnl|SRA|SRR5054409.7862195.1:60-101 7862195..
tagcttgcagaactcaaacagtaggaacttagtgggggattc---
>_R_gnl|SRA|SRR5054409.7623104.2:67-108 7623104..
tagcttgcagaactcaaacagtaggaacttagtgggggattc---
>_R_gnl|SRA|SRR5054408.14831100.2:65-106 14831100..
tagcttgcagaactcaaacagtaggaacttagtgggggattc---
>_R_gnl|SRA|SRR5054408.11314701.2:66-107 11314701..
tagcttgcagaactcaaacagtaggaacttagtgggggattc---
>gnl|SRA|SRR5054408.11314701.1:83-124 11314701..
tagcttgcagaactcaaacagtaggaacttagtgggggattc---
>gnl|SRA|SRR5054408.10316106.1:78-119 10316106..
tagcttgcagaactcaaacagtaggaacttagtgggggattc---
>_R_gnl|SRA|SRR5054408.6570608.2:67-108 6570608..
tagcttgcagaactcaaacagtaggaacttagtgggggattc---
>_R_gnl|SRA|SRR5054408.5192935.2:67-108 5192935..
tagcttgcagaactcaaacagtaggaacttagtgggggattc---
>_R_gnl|SRA|SRR5054408.4003616.2:79-120 4003616..
tagcttgcagaactcaaacagtaggaacttagtgggggattc---
>gnl|SRA|SRR5054408.4003616.1:82-123 4003616..
tagcttgcagaactcaaacagtaggaacttagtgggggattc---
>_R_gnl|SRA|SRR5054408.1928582.2:66-107 1928582..
tagcttgcagaactcaaacagtaggaacttagtgggggattc---
>_R_gnl|SRA|SRR5054410.7087484.2:65-103 7087484..
tagcttgcagaactcaaacag---gaactgagtgggggattc---
>_R_gnl|SRA|SRR5054410.22385557.2:67-105 22385557..
tagcttgcagaactcaaacag---gaacttagtgggggattc---
>gnl|SRA|SRR5054410.22385557.1:57-95 22385557..
tagcttgcagaactcaaacag---gaacttagtgggggattc---
>_R_gnl|SRA|SRR5054410.22380332.2:79-117 22380332..
tagcttgcagaactcaaacag---gaacttagtgggggattc---
>_R_gnl|SRA|SRR5054410.18235040.2:67-105 18235040..
tagcttgcagaactcaaacag---gaacttagtgggggattc---
>gnl|SRA|SRR5054410.15256189.1:71-109 15256189..
tagcttgcagaactcaaacag---gaacttagtgggggattc---
>_R_gnl|SRA|SRR5054410.15006773.2:67-105 15006773..

tagcttgcagaactcaaacag---gaacttagtgggggattc---
>_R_gnl|SRA|SRR5054410.14110930.2:67-105 14110930..
tagcttgcagaactcaaacag---gaacttagtgggggattc---
>_R_gnl|SRA|SRR5054410.9096820.2:67-105 9096820..
tagcttgcagaactcaaacag---gaacttagtgggggattc---
>_R_gnl|SRA|SRR5054410.6984663.2:70-108 6984663..
tagcttgcagaactcaaacag---gaacttagtgggggattc---
>_R_gnl|SRA|SRR5054410.6730639.2:66-104 6730639..
tagcttgcagaactcaaacag---gaacttagtgggggattc---
>_R_gnl|SRA|SRR5054410.5232496.2:66-104 5232496..
tagcttgcagaactcaaacag---gaacttagtgggggattc---
>_R_gnl|SRA|SRR5054410.4489689.2:67-105 4489689..
tagcttgcagaactcaaacag---gaacttagtgggggattc---
>_R_gnl|SRA|SRR5054410.2301173.2:67-105 2301173..
tagcttgcagaactcaaacag---gaacttagtgggggattc---
>_R_gnl|SRA|SRR5054410.2079575.2:67-105 2079575..
tagcttgcagaactcaaacag---gaacttagtgggggattc---
>_R_gnl|SRA|SRR5054410.257118.2:67-105 257118..
tagcttgcagaactcaaacag---gaacttagtgggggattc---
>_R_gnl|SRA|SRR5054409.22107776.2:67-105 22107776..
tagcttgcagaactcaaacag---gaacttagtgggggattc---
>_R_gnl|SRA|SRR5054409.19807913.2:33-71 19807913..
tagcttgcagaactcaaacag---gaacttagtgggggattc---
>_R_gnl|SRA|SRR5054409.17573180.2:67-105 17573180..
tagcttgcagaactcaaacag---gaacttagtgggggattc---
>_R_gnl|SRA|SRR5054409.14009883.2:66-104 14009883..
tagcttgcagaactcaaacag---gaacttagtgggggattc---
>_R_gnl|SRA|SRR5054409.12867369.2:67-105 12867369..
tagcttgcagaactcaaacag---gaacttagtgggggattc---
>_R_gnl|SRA|SRR5054409.11527894.2:67-105 11527894..
tagcttgcagaactcaaacag---gaacttagtgggggattc---
>_R_gnl|SRA|SRR5054409.11205338.2:80-118 11205338..
tagcttgcagaactcaaacag---gaacttagtgggggattc---
>gnl|SRA|SRR5054409.11205338.1:58-96 11205338..
tagcttgcagaactcaaacag---gaacttagtgggggattc---
>_R_gnl|SRA|SRR5054409.11070121.2:67-105 11070121..
tagcttgcagaactcaaacag---gaacttagtgggggattc---
>_R_gnl|SRA|SRR5054409.10365098.2:67-105 10365098..
tagcttgcagaactcaaacag---gaacttagtgggggattc---
>_R_gnl|SRA|SRR5054409.8061402.2:67-105 8061402..
tagcttgcagaactcaaacag---gaacttagtgggggattc---
>_R_gnl|SRA|SRR5054409.6846251.2:67-105 6846251..
tagcttgcagaactcaaacag---gaacttagtgggggattc---
>_R_gnl|SRA|SRR5054409.5675659.2:62-100 5675659..
tagcttgcagaactcaaacag---gaacttagtgggggattc---
>_R_gnl|SRA|SRR5054409.5255395.2:62-100 5255395..
tagcttgcagaactcaaacag---gaacttagtgggggattc---
>_R_gnl|SRA|SRR5054409.1701083.2:66-104 1701083..
tagcttgcagaactcaaacag---gaacttagtgggggattc---
>_R_gnl|SRA|SRR5054409.1181387.2:78-116 1181387..
tagcttgcagaactcaaacag---gaacttagtgggggattc---

>_R_gnl|SRA|SRR5054409.1055836.2:67-105 1055836..
tagcttgcagaactcaaacag---gaacttagtgggggattc---
>_R_gnl|SRA|SRR5054408.22706507.2:63-101 22706507..
tagcttgcagaactcaaacag---gaacttagtgggggattc---
>_R_gnl|SRA|SRR5054408.22238964.2:66-104 22238964..
tagcttgcagaactcaaacag---gaacttagtgggggattc---
>gnl|SRA|SRR5054408.21651111.1:13-51 21651111..
tagcttgcagaactcaaacag---gaacttagtgggggattc---
>_R_gnl|SRA|SRR5054408.20875544.2:63-101 20875544..
tagcttgcagaactcaaacag---gaacttagtgggggattc---
>gnl|SRA|SRR5054408.20698229.1:83-121 20698229..
tagcttgcagaactcaaacag---gaacttagtgggggattc---
>_R_gnl|SRA|SRR5054408.19219015.2:77-115 19219015..
tagcttgcagaactcaaacag---gaacttagtgggggattc---
>_R_gnl|SRA|SRR5054408.18849588.2:81-119 18849588..
tagcttgcagaactcaaacag---gaacttagtgggggattc---
>gnl|SRA|SRR5054408.17629702.1:83-121 17629702..
tagcttgcagaactcaaacag---gaacttagtgggggattc---
>_R_gnl|SRA|SRR5054408.16269933.2:66-104 16269933..
tagcttgcagaactcaaacag---gaacttagtgggggattc---
>_R_gnl|SRA|SRR5054408.14814894.2:66-104 14814894..
tagcttgcagaactcaaacag---gaacttagtgggggattc---
>_R_gnl|SRA|SRR5054408.14404680.2:66-104 14404680..
tagcttgcagaactcaaacag---gaacttagtgggggattc---
>_R_gnl|SRA|SRR5054408.12572790.2:68-106 12572790..
tagcttgcagaactcaaacag---gaacttagtgggggattc---
>_R_gnl|SRA|SRR5054408.9206041.2:76-114 9206041..
tagcttgcagaactcaaacag---gaacttagtgggggattc---
>_R_gnl|SRA|SRR5054408.8219289.2:23-61 8219289..
tagcttgcagaactcaaacag---gaacttagtgggggattc---
>_R_gnl|SRA|SRR5054408.7846195.2:67-105 7846195..
tagcttgcagaactcaaacag---gaacttagtgggggattc---
>_R_gnl|SRA|SRR5054408.6630523.2:66-104 6630523..
tagcttgcagaactcaaacag---gaacttagtgggggattc---
>_R_gnl|SRA|SRR5054408.5411722.2:66-104 5411722..
tagcttgcagaactcaaacag---gaacttagtgggggattc---
>_R_gnl|SRA|SRR5054408.4152196.2:65-103 4152196..
tagcttgcagaactcaaacag---gaacttagtgggggattc---
>gnl|SRA|SRR5054408.4152196.1:83-121 4152196..
tagcttgcagaactcaaacag---gaacttagtgggggattc---
>_R_gnl|SRA|SRR5054408.3520940.2:67-105 3520940..
tagcttgcagaactcaaacag---gaacttagtgggggattc---
>_R_gnl|SRA|SRR5054408.3322145.2:66-104 3322145..
tagcttgcagaactcaaacag---gaacttagtgggggattc---
>_R_gnl|SRA|SRR5054408.1702959.2:66-104 1702959..
tagcttgcagaactcaaacag---gaacttagtgggggattc---
>_R_gnl|SRA|SRR5054408.800796.2:68-106 800796..
tagcttgcagaactcaaacag---gaacttagtgggggattc---
>gnl|SRA|SRR5054409.7088353.1:97-125 7088353..
tagcttgcagaactcaaacag---gaacttag-------------
>_R_gnl|SRA|SRR5054409.4007728.2:77-115 4007728..

tagcttgcagaactcaaacag---gaacttagtgggtgattc---
>_R_gnl|SRA|SRR5054410.1116522.2:1-33 1116522..
tagcttgcagaactcaaacag---gaacttaggggg---------
>_R_gnl|SRA|SRR5054409.23355810.2:68-106 23355810..
tagcttgcagaactcaaacag---gcacttagtgggggattc---
>_R_gnl|SRA|SRR5054410.18944665.2:66-104 18944665..
tagcttgcagaattcaaacag---gaacttagtgggggattc---
>_R_gnl|SRA|SRR5054410.4356545.2:66-104 4356545..
tagcttgcagaattcaaacag---gaacttagtgggggattc---
>_R_gnl|SRA|SRR5054409.17927321.2:67-105 17927321..
tagcttgcagaattcaaacag---gaacttagtgggggattc---
>_R_gnl|SRA|SRR5054410.10199344.2:76-114 10199344..
tagctggcagaactcaaacag---gaacttagtgggggattc---
>_R_gnl|SRA|SRR5054409.9409956.2:3-35 9409956..
tagcatgcagaactcaaacag---gaacttagtggg---------

SRX567871
>control3 .
tagcttgcagaactca---aacagtaggaacttagtgggggattc----
>gnl|SRA|SRR1330252.4530866.1:36-77 4530866.
tagcttgcagaactca---aacagtaggaacttagtgggggattc----
>_R_gnl|SRA|SRR1330252.3698829.2:1-38 3698829.
tagcttgcagaactca---aacagtaggaacttagtggggg--------
>_R_gnl|SRA|SRR1330252.4948026.1:11-49 4948026.
tagcttgcagaactca---aacag---gaacttagtgggggattc----
>_R_gnl|SRA|SRR1330252.2764863.2:24-62 2764863.
tagcttgcagaactca---aacag---gaacttagtgggggattc----
>_R_gnl|SRA|SRR1330252.2474649.1:36-74 2474649.
tagcttgcagaactca---aacag---gaacttagtgggggattc----
>_R_gnl|SRA|SRR1330252.2308196.2:24-62 2308196.
tagcttgcagaactca---aacag---gaacttagtgggggattc----
>gnl|SRA|SRR1330252.432320.1:25-63 432320.
tagcttgcagaactca---aacag---gaacttagtgggggattc----

SRX1639030
>control .
ttaccaccttgcagaacaacaacag---gaccataatgggggattctgta
>gnl|SRA|SRR3233339.16307605.2:19-65 16307605.
ttaccaccttgcagaacaacaacag---gaccataatgggggattctgta
>gnl|SRA|SRR3233339.14324583.2:19-65 14324583.
ttaccaccttgcagaacaacaacag---gaccataatgggggattctgta
>_R_gnl|SRA|SRR3233339.9959707.2:14-60 9959707.
ttaccaccttgcagaacaacaacag---gaccataatgggggattctgta
>_R_gnl|SRA|SRR3233339.4505331.1:19-65 4505331.
ttaccaccttgcagaacaacaacag---gaccataatgggggattctgta
>_R_gnl|SRA|SRR3233339.4426869.2:28-74 4426869.
ttaccaccttgcagaacaacaacag---gaccataatgggggattctgta
>_R_gnl|SRA|SRR3233339.4616388.1:18-67 4616388.

ttaccaccttgcagaacaacaacagtaggaccataatgggggattctgta
>gnl|SRA|SRR3233339.10287606.1:39-76 10287606.
ttaccaccttgcagaacaacaacag---gaccataatgggg---------
>gnl|SRA|SRR3233339.8970976.1:1-33 8970976.
--------------aacaacaacag---gaccataatgggggattctgta
>gnl|SRA|SRR3233339.11549154.1:1-31 11549154.
----------------caacaacag---gaccataatgggggattctnta
>_R_gnl|SRA|SRR3233339.10287606.2:50-76 10287606.
--------------------aacag---gaccataatgggggattctgta

SRX1639019
>control .
tcaccaacttgcagaactccaatagcaggactatacaggcagggga-
>gnl|SRA|SRR3233328.25675416.1:66-101 25675416.
tcaccaacttgcagaactccaatagcaggactatac-----------
>gnl|SRA|SRR3233328.14972851.1:1-36 14972851.
----------gcagaactccaatagcaggactatacaggcagggga-
>_R_gnl|SRA|SRR3233328.38644847.1:37-82 38644847.
tcaccaacttgcagaactccaatagcaggactatacaggcagggga-
>gnl|SRA|SRR3233328.36032671.1:44-89 36032671.
tcaccaacttgcagaactccaatagcaggactatacaggcagggga-
>gnl|SRA|SRR3233328.24792564.1:21-66 24792564.
tcaccaacttgcagaactccaatagcaggactatacaggcagggga-
>_R_gnl|SRA|SRR3233328.24704713.1:27-72 24704713.
tcaccaacttgcagaactccaatagcaggactatacaggcagggga-
>_R_gnl|SRA|SRR3233328.23432622.1:50-95 23432622.
tcaccaacttgcagaactccaatagcaggactatacaggcagggga-
>gnl|SRA|SRR3233328.22909563.2:42-87 22909563.
tcaccaacttgcagaactccaatagcaggactatacaggcagggga-
>_R_gnl|SRA|SRR3233328.22909563.1:27-72 22909563.
tcaccaacttgcagaactccaatagcaggactatacaggcagggga-
>_R_gnl|SRA|SRR3233328.8650944.1:27-72 8650944.
tcaccaacttgcagaactccaatagcaggactatacaggcagggga-
>_R_gnl|SRA|SRR3233328.4079588.2:27-72 4079588.
tcaccaacttgcagaactccaatagcaggactatacaggcagggga-
>_R_gnl|SRA|SRR3233328.3987800.2:35-80 3987800.
tcaccaacttgcagaactccaatagcaggactatacaggcagggga-
>gnl|SRA|SRR3233328.24704713.2:20-64 24704713.
tcaccaacttgcagaactccaatagcaggactatacaggcagggg--
>gnl|SRA|SRR3233328.8650944.2:33-77 8650944.
tcaccaacttgcagaactccaatagcaggactatacaggcagggg--
>gnl|SRA|SRR3233328.4079588.1:1-45 4079588.
-caccaacttgcagaactccaatagcaggactatacaggcagggga-
>gnl|SRA|SRR3233328.30292314.1:1-44 30292314.
--accaacttgcagaactccaatagcaggactatacaggcagggga-
>_R_gnl|SRA|SRR3233328.35063714.2:26-71 35063714.
tcaccaactggcagaactccaatagcaggactatacaggcagggga-
>_R_gnl|SRA|SRR3233328.31386113.2:26-71 31386113.
tcaccaacttgcagaactccaatagcaggactatccaggcagggga-

>_R_gnl|SRA|SRR3233328.24792564.2:49-94 24792564.
tcaccatcttgcagaactccaatagcaggactatacaggcagggga-
>_R_gnl|SRA|SRR3233328.13526112.2:56-101 13526112.
tcaccaactggcagaactccaatagcaggactatacaggcagggga-
>gnl|SRA|SRR3233328.38644847.2:32-76 38644847.
tcaccaacttgcagaactccaatagcaggactatacagggagggg--
>_R_gnl|SRA|SRR3233328.25675416.2:1-40 25675416.
tcaccaacttgcagaactccaatagcaggactatacaggc-------
>gnl|SRA|SRR3233328.29002035.2:1-39 29002035.
-------cttgcagaactccaatagcaggactatacaggcagggga-
>_R_gnl|SRA|SRR3233328.29002035.1:25-63 29002035.
-------cttgcagaactccaatagcaggactatacaggcagggga-
>gnl|SRA|SRR3233328.42931017.1:44-86 42931017.
tcaccaacttgcagaactccaatag---gactatacaggcagggga-
>_R_gnl|SRA|SRR3233328.42616953.2:30-72 42616953.
tcaccaacttgcagaactccaatag---gactatacaggcagggga-
>gnl|SRA|SRR3233328.42134125.1:6-48 42134125.
tcaccaacttgcagaactccaatag---gactatacaggcagggga-
>gnl|SRA|SRR3233328.38204888.1:20-62 38204888.
tcaccaacttgcagaactccaatag---gactatacaggcagggga-
>_R_gnl|SRA|SRR3233328.35577116.2:34-76 35577116.
tcaccaacttgcagaactccaatag---gactatacaggcagggga-
>gnl|SRA|SRR3233328.35577116.1:24-66 35577116.
tcaccaacttgcagaactccaatag---gactatacaggcagggga-
>_R_gnl|SRA|SRR3233328.34579870.2:53-95 34579870.
tcaccaacttgcagaactccaatag---gactatacaggcagggga-
>_R_gnl|SRA|SRR3233328.34383796.2:14-56 34383796.
tcaccaacttgcagaactccaatag---gactatacaggcagggga-
>gnl|SRA|SRR3233328.34383796.1:19-61 34383796.
tcaccaacttgcagaactccaatag---gactatacaggcagggga-
>_R_gnl|SRA|SRR3233328.32755164.1:46-88 32755164.
tcaccaacttgcagaactccaatag---gactatacaggcagggga-
>_R_gnl|SRA|SRR3233328.31993161.1:30-72 31993161.
tcaccaacttgcagaactccaatag---gactatacaggcagggga-
>_R_gnl|SRA|SRR3233328.31773888.1:27-69 31773888.
tcaccaacttgcagaactccaatag---gactatacaggcagggga-
>_R_gnl|SRA|SRR3233328.31578279.2:39-81 31578279.
tcaccaacttgcagaactccaatag---gactatacaggcagggga-
>gnl|SRA|SRR3233328.31578279.1:39-81 31578279.
tcaccaacttgcagaactccaatag---gactatacaggcagggga-
>_R_gnl|SRA|SRR3233328.31527239.1:27-69 31527239.
tcaccaacttgcagaactccaatag---gactatacaggcagggga-
>_R_gnl|SRA|SRR3233328.29917028.1:2-44 29917028.
tcaccaacttgcagaactccaatag---gactatacaggcagggga-
>gnl|SRA|SRR3233328.29784799.2:53-95 29784799.
tcaccaacttgcagaactccaatag---gactatacaggcagggga-
>gnl|SRA|SRR3233328.28322919.2:6-48 28322919.
tcaccaacttgcagaactccaatag---gactatacaggcagggga-
>_R_gnl|SRA|SRR3233328.28322919.1:26-68 28322919.
tcaccaacttgcagaactccaatag---gactatacaggcagggga-
>_R_gnl|SRA|SRR3233328.24884867.1:25-67 24884867.

tcaccaacttgcagaactccaatag---gactatacaggcagggga-
>gnl|SRA|SRR3233328.24737170.1:21-63 24737170.
tcaccaacttgcagaactccaatag---gactatacaggcagggga-
>gnl|SRA|SRR3233328.24319374.1:5-47 24319374.
tcaccaacttgcagaactccaatag---gactatacaggcagggga-
>_R_gnl|SRA|SRR3233328.23943929.2:27-69 23943929.
tcaccaacttgcagaactccaatag---gactatacaggcagggga-
>gnl|SRA|SRR3233328.23943929.1:20-62 23943929.
tcaccaacttgcagaactccaatag---gactatacaggcagggga-
>gnl|SRA|SRR3233328.22064617.1:33-75 22064617.
tcaccaacttgcagaactccaatag---gactatacaggcagggga-
>_R_gnl|SRA|SRR3233328.20937605.1:27-69 20937605.
tcaccaacttgcagaactccaatag---gactatacaggcagggga-
>gnl|SRA|SRR3233328.19833497.2:7-49 19833497.
tcaccaacttgcagaactccaatag---gactatacaggcagggga-
>_R_gnl|SRA|SRR3233328.19833497.1:27-69 19833497.
tcaccaacttgcagaactccaatag---gactatacaggcagggga-
>_R_gnl|SRA|SRR3233328.19276435.2:54-96 19276435.
tcaccaacttgcagaactccaatag---gactatacaggcagggga-
>gnl|SRA|SRR3233328.19276435.1:48-90 19276435.
tcaccaacttgcagaactccaatag---gactatacaggcagggga-
>gnl|SRA|SRR3233328.19125849.1:21-63 19125849.
tcaccaacttgcagaactccaatag---gactatacaggcagggga-
>gnl|SRA|SRR3233328.18765098.2:5-47 18765098.
tcaccaacttgcagaactccaatag---gactatacaggcagggga-
>gnl|SRA|SRR3233328.17556011.2:26-68 17556011.
tcaccaacttgcagaactccaatag---gactatacaggcagggga-
>_R_gnl|SRA|SRR3233328.17556011.1:27-69 17556011.
tcaccaacttgcagaactccaatag---gactatacaggcagggga-
>_R_gnl|SRA|SRR3233328.16197516.1:27-69 16197516.
tcaccaacttgcagaactccaatag---gactatacaggcagggga-
>gnl|SRA|SRR3233328.16133676.1:20-62 16133676.
tcaccaacttgcagaactccaatag---gactatacaggcagggga-
>gnl|SRA|SRR3233328.15068849.1:14-56 15068849.
tcaccaacttgcagaactccaatag---gactatacaggcagggga-
>_R_gnl|SRA|SRR3233328.14991524.2:27-69 14991524.
tcaccaacttgcagaactccaatag---gactatacaggcagggga-
>_R_gnl|SRA|SRR3233328.14127036.1:27-69 14127036.
tcaccaacttgcagaactccaatag---gactatacaggcagggga-
>gnl|SRA|SRR3233328.13892796.2:20-62 13892796.
tcaccaacttgcagaactccaatag---gactatacaggcagggga-
>_R_gnl|SRA|SRR3233328.13892796.1:45-87 13892796.
tcaccaacttgcagaactccaatag---gactatacaggcagggga-
>gnl|SRA|SRR3233328.10301704.1:21-63 10301704.
tcaccaacttgcagaactccaatag---gactatacaggcagggga-
>gnl|SRA|SRR3233328.10109743.1:5-47 10109743.
tcaccaacttgcagaactccaatag---gactatacaggcagggga-
>gnl|SRA|SRR3233328.9687644.1:19-61 9687644.
tcaccaacttgcagaactccaatag---gactatacaggcagggga-
>gnl|SRA|SRR3233328.9595516.1:44-86 9595516.
tcaccaacttgcagaactccaatag---gactatacaggcagggga-

>_R_gnl|SRA|SRR3233328.8800171.2:46-88 8800171.
tcaccaacttgcagaactccaatag---gactatacaggcagggga-
>gnl|SRA|SRR3233328.8800171.1:44-86 8800171.
tcaccaacttgcagaactccaatag---gactatacaggcagggga-
>_R_gnl|SRA|SRR3233328.8702946.1:27-69 8702946.
tcaccaacttgcagaactccaatag---gactatacaggcagggga-
>_R_gnl|SRA|SRR3233328.8614629.1:48-90 8614629.
tcaccaacttgcagaactccaatag---gactatacaggcagggga-
>gnl|SRA|SRR3233328.6062089.1:39-81 6062089.
tcaccaacttgcagaactccaatag---gactatacaggcagggga-
>_R_gnl|SRA|SRR3233328.5531269.1:26-68 5531269.
tcaccaacttgcagaactccaatag---gactatacaggcagggga-
>gnl|SRA|SRR3233328.3737771.2:19-61 3737771.
tcaccaacttgcagaactccaatag---gactatacaggcagggga-
>_R_gnl|SRA|SRR3233328.3737771.1:27-69 3737771.
tcaccaacttgcagaactccaatag---gactatacaggcagggga-
>_R_gnl|SRA|SRR3233328.3483662.1:27-69 3483662.
tcaccaacttgcagaactccaatag---gactatacaggcagggga-
>_R_gnl|SRA|SRR3233328.3340544.2:26-68 3340544.
tcaccaacttgcagaactccaatag---gactatacaggcagggga-
>gnl|SRA|SRR3233328.2722207.2:6-48 2722207.
tcaccaacttgcagaactccaatag---gactatacaggcagggga-
>gnl|SRA|SRR3233328.2439082.1:5-47 2439082.
tcaccaacttgcagaactccaatag---gactatacaggcagggga-
>_R_gnl|SRA|SRR3233328.1784738.1:56-98 1784738.
tcaccaacttgcagaactccaatag---gactatacaggcagggga-
>_R_gnl|SRA|SRR3233328.1643467.2:41-83 1643467.
tcaccaacttgcagaactccaatag---gactatacaggcagggga-
>gnl|SRA|SRR3233328.1643467.1:21-63 1643467.
tcaccaacttgcagaactccaatag---gactatacaggcagggga-
>gnl|SRA|SRR3233328.274319.1:20-62 274319.
tcaccaacttgcagaactccaatag---gactatacaggcagggga-
>gnl|SRA|SRR3233328.36588868.1:56-97 36588868.
tcaccaacttgcagaactccaatag---gactatacaggcagggg--
>gnl|SRA|SRR3233328.27534104.2:1-42 27534104.
-caccaacttgcagaactccaatag---gactatacaggcagggga-
>_R_gnl|SRA|SRR3233328.16133676.2:1-42 16133676.
tcaccaacttgcagaactccaatag---gactatacaggcagggg--
>gnl|SRA|SRR3233328.14991524.1:1-42 14991524.
-caccaacttgcagaactccaatag---gactatacaggcagggga-
>gnl|SRA|SRR3233328.14127036.2:1-42 14127036.
-caccaacttgcagaactccaatag---gactatacaggcagggga-
>gnl|SRA|SRR3233328.12125292.1:1-42 12125292.
-caccaacttgcagaactccaatag---gactatacaggcagggga-
>gnl|SRA|SRR3233328.8614629.2:21-62 8614629.
tcaccaacttgcagaactccaatag---gactatacaggcagggg--
>gnl|SRA|SRR3233328.1784738.2:1-42 1784738.
-caccaacttgcagaactccaatag---gactatacaggcagggga-
>_R_gnl|SRA|SRR3233328.40447810.2:38-80 40447810.
tcaccaacttgcacaactccaatag---gactatacaggcagggga-
>gnl|SRA|SRR3233328.36079032.2:19-59 36079032.

tcaccaacttgcagaactccaatag---gactatacaggcaggg---
>_R_gnl|SRA|SRR3233328.36079032.1:1-41 36079032.
tcaccaacttgcagaactccaatag---gactatacaggcaggg---
>_R_gnl|SRA|SRR3233328.35314695.2:27-69 35314695.
tcaccaacttgcggaactccaatag---gactatacaggcagggga-
>_R_gnl|SRA|SRR3233328.22064617.2:47-89 22064617.
tcaccaacttgcaaaactccaatag---gactatacaggcagggga-
>gnl|SRA|SRR3233328.14799943.2:1-41 14799943.
--accaacttgcagaactccaatag---gactatacaggcagggga-
>gnl|SRA|SRR3233328.9380863.1:61-101 9380863.
tcaccaacttgcagaactccaatag---gactatacaggcaggg---

SRX1639020

>control .
taccaacttgcagaactcaaataggactctactgggggattca
>gnl|SRA|SRR3233329.30234525.1:13-55 30234525.
taccaacttgcagaactcaaataggactctactgggggattca
>_R_gnl|SRA|SRR3233329.25462893.1:5-47 25462893.
taccaacttgcagaactcaaataggactctactgggggattca
>gnl|SRA|SRR3233329.18549253.2:45-87 18549253.
taccaacttgcagaactcaaataggactctactgggggattca
>_R_gnl|SRA|SRR3233329.18549253.1:5-47 18549253.
taccaacttgcagaactcaaataggactctactgggggattca
>gnl|SRA|SRR3233329.9799329.1:45-87 9799329.
taccaacttgcagaactcaaataggactctactgggggattca
>gnl|SRA|SRR3233329.1273302.1:23-65 1273302.
taccaacttgcagaactcaaataggactctactgggggattca
>gnl|SRA|SRR3233329.25462893.2:45-87 25462893.
taccaccttgcagaactcaaataggactctactgggggattca
>gnl|SRA|SRR3233329.24019660.1:40-82 24019660.
taccaacttgcagaactcaaataggactctactgggggtttca
>gnl|SRA|SRR3233329.20278117.2:1-40 20278117.
---caacttgcagaactcaaataggactctactgggggattca
>_R_gnl|SRA|SRR3233329.20278117.1:10-49 20278117.
---caacttgcagaactcaaataggactctactgggggattca
>_R_gnl|SRA|SRR3233329.18524497.2:10-49 18524497.
---caacttgcagaactcaaataggactctactgggggattca
>gnl|SRA|SRR3233329.18524497.1:1-40 18524497.
---caacttgcagaactcaaataggactctactgggggattca
>_R_gnl|SRA|SRR3233329.18227757.1:42-81 18227757.
---caacttgcagaactcaaataggactctactgggggattca
>gnl|SRA|SRR3233329.8579749.1:40-82 8579749.
taccaacttgcagaactcaaataggactctactgggggtttca
>gnl|SRA|SRR3233329.15041068.2:34-72 15041068.
taccaacttgcagaactcaaataggactctactgggggа----
>_R_gnl|SRA|SRR3233329.15041068.1:1-38 15041068.
taccaacttgcagaactcaaataggactctactggggg-----
>gnl|SRA|SRR3233329.28531535.1:27-63 28531535.

taccaacttgcagaactcaaataggactctactgggg------
>gnl|SRA|SRR3233329.18227757.2:1-40 18227757.
---caacttgcagaactcaaataggactatactgggggattca
>_R_gnl|SRA|SRR3233329.1396319.1:1-39 1396319.
taccaacttgcagaactcaaataggcctctactgggggga----
>gnl|SRA|SRR3233329.1396319.2:45-83 1396319.
taccaacttgcagcccttaaataggactctactgggggga----
>gnl|SRA|SRR3233329.26596733.1:1-28 26596733.
---------------ctcaaataggactctactgggggattca
>gnl|SRA|SRR3233329.20219371.1:1-28 20219371.
---------------ctcaaataggactctactgggggattca
>_R_gnl|SRA|SRR3233329.9799329.2:74-101 9799329.
---------------ctcaaataggactctactgggggattca
>_R_gnl|SRA|SRR3233329.3098659.2:58-82 3098659.
------------------aaataggactctactgggggattca
>gnl|SRA|SRR3233329.3098659.1:1-25 3098659.
------------------aaataggactctactgggggattca
>_R_gnl|SRA|SRR3233329.30234525.2:11-50 30234525.
taccaacttgaagaactcaaatantacnntacngnggnat---
>gnl|SRA|SRR3233329.556515.2:57-74 556515.
taccaacttgcagaactc-------------------------

ERX2099848
>control .
accaacctacagaactccaacagtaggatttttttgggggagtct---------
>_R_gnl|SRA|ERR2040791.3570301.2 FCC0DM0ACXX:8:1206:2246:118282.
accaacctacagaactccaacagtaggatttttttgggggagtctcttagcaac
>_R_gnl|SRA|ERR2040791.10237713.1 FCC0DM0ACXX:8:2206:16533:124588.
---aacctacagaactccaacag---gatttttttgggggagtctcttagcaac

SRX1639024
>control .
ctacagaactccatcag---gattttgttgggggaatgccttagc---------------

>_R_gnl|SRA|SRR3233333.27246711.2 27246711.
ctacagaactccatcag---gattttgttgggggaatgccttagcagcattccctca---

>gnl|SRA|SRR3233333.13372898.1 13372898.
ctacagaactccatcag---gattttgttgggggaatgccttagcagcattccctcaa--

>_R_gnl|SRA|SRR3233333.17949242.1 17949242.
ctacagaactccatcag---gattttgttgggggaatgccttagcagcattccctcaagg
gaactcg
>_R_gnl|SRA|SRR3233333.24885069.1 24885069.
ctacagaactccatcag---gattttgttgggggaatgccttagcagcattccctcaagg
gaactcg

>_R_gnl|SRA|SRR3233333.13372898.2 13372898.
ctacagaactccatcag---gattttgttgggggaatgccttagcagcattccctcaagg
gaactcg
>gnl|SRA|SRR3233333.14680141.2 14680141.
ctacagaactccatcag---gattttgttgggggaatgccttagcagcattccctcaagg
gaactcg
>gnl|SRA|SRR3233333.24885069.2 24885069.
ctacagaactccatcag---gattttgttgggggaatgccttagcag-------------

>gnl|SRA|SRR3233333.17949242.2 17949242.
ctacagaactccatcag---gattttgttgggggaatgccttagcag-------------

>gnl|SRA|SRR3233333.1573605.2 1573605.
ctacagaactccatcag---gattttgttgggggaatgccttagcag-------------

>gnl|SRA|SRR3233333.33347151.2 33347151.
---cagaactccatcag---gattttgttgggggaatgccttagcagcattccctcaagg
gaactcg
>_R_gnl|SRA|SRR3233333.14680141.1 14680141.
---cagaactccatcag---gattttgttgggggaatgccttagcagcattccctcaagg
gaactcg
>_R_gnl|SRA|SRR3233333.1573605.1 1573605.
-----------catcag---gattttgttgggggaatgccttagcagcattccctcaagg
gaactcg
>gnl|SRA|SRR3233333.6531454.2 6531454.
---cagaactccatcagtaggattttgttgggggaatgccttagcagcattccctcaagg
gaactcg

ERX1349704

>control
agcacattaaataacagtaataggaatttgttgggcgag
>gnl|SRA|ERR1278117.82690193.1:31-69 HWI-ST574:196:D08GAACXX:5:2316:18618:99889
agcacattaaataacagtaataggaatttgttgggcgag
>gnl|SRA|ERR1278117.82546414.1:39-77 HWI-ST574:196:D08GAACXX:5:2316:12846:87860
agcacattaaataacagtaataggaatttgttgggcgag
>gnl|SRA|ERR1278117.82481126.1:31-69 HWI-ST574:196:D08GAACXX:5:2316:10482:82273
agcacattaaataacagtaataggaatttgttgggcgag
>gnl|SRA|ERR1278117.82442043.1:5-43 HWI-ST574:196:D08GAACXX:5:2316:1916:79119
agcacattaaataacagtaataggaatttgttgggcgag
>gnl|SRA|ERR1278117.82425573.1:1-39 HWI-ST574:196:D08GAACXX:5:2316:8831:77712
agcacattaaataacagtaataggaatttgttgggcgag
>gnl|SRA|ERR1278117.82142333.1:58-96 HWI-ST574:196:D08GAACXX:5:2316:13854:53142
agcacattaaataacagtaataggaatttgttgggcgag
>gnl|SRA|ERR1278117.81808023.1:5-43 HWI-ST574:196:D08GAACXX:5:2316:1559:23856
agcacattaaataacagtaataggaatttgttgggcgag
>gnl|SRA|ERR1278117.81703867.1:37-75 HWI-ST574:196:D08GAACXX:5:2316:18580:14308
agcacattaaataacagtaataggaatttgttgggcgag
>_R_gnl|SRA|ERR1278117.81536781.2:1-39 HWI-ST574:196:D08GAACXX:5:2315:7325:98473
agcacattaaataacagtaataggaatttgttgggcgag

>gnl|SRA|ERR1278117.81490721.1:50-88 HWI-ST574:196:D08GAACXX:5:2315:15234:94229
agcacattaaataacagtaataggaatttgttgggcgag
>gnl|SRA|ERR1278117.81433338.1:5-43 HWI-ST574:196:D08GAACXX:5:2315:18369:88935
agcacattaaataacagtaataggaatttgttgggcgag
>_R_gnl|SRA|ERR1278117.81364470.2:3-41 HWI-
ST574:196:D08GAACXX:5:2315:11766:82702
agcacattaaataacagtaataggaatttgttgggcgag
>_R_gnl|SRA|ERR1278117.81230496.2:2-40 HWI-ST574:196:D08GAACXX:5:2315:8610:70484
agcacattaaataacagtaataggaatttgttgggcgag
>_R_gnl|SRA|ERR1278117.81216741.2:3-41 HWI-ST574:196:D08GAACXX:5:2315:7527:69044
agcacattaaataacagtaataggaatttgttgggcgag
>gnl|SRA|ERR1278117.80830876.1:33-71 HWI-ST574:196:D08GAACXX:5:2315:6894:33297
agcacattaaataacagtaataggaatttgttgggcgag
>gnl|SRA|ERR1278117.80754133.1:55-93 HWI-ST574:196:D08GAACXX:5:2315:17703:26226
agcacattaaataacagtaataggaatttgttgggcgag
>gnl|SRA|ERR1278117.80638993.1:32-70 HWI-ST574:196:D08GAACXX:5:2315:20890:15349
agcacattaaataacagtaataggaatttgttgggcgag
>gnl|SRA|ERR1278117.80534888.1:7-45 HWI-ST574:196:D08GAACXX:5:2315:17990:5696
agcacattaaataacagtaataggaatttgttgggcgag
>gnl|SRA|ERR1278117.80153028.1:3-41 HWI-ST574:196:D08GAACXX:5:2314:14398:68111
agcacattaaataacagtaataggaatttgttgggcgag
>_R_gnl|SRA|ERR1278117.80109636.2:3-41 HWI-ST574:196:D08GAACXX:5:2314:2267:64073
agcacattaaataacagtaataggaatttgttgggcgag
>gnl|SRA|ERR1278117.80019157.1:7-45 HWI-ST574:196:D08GAACXX:5:2314:1439:55350
agcacattaaataacagtaataggaatttgttgggcgag
>_R_gnl|SRA|ERR1278117.79935633.2:3-41 HWI-
ST574:196:D08GAACXX:5:2314:12936:47212
agcacattaaataacagtaataggaatttgttgggcgag
>gnl|SRA|ERR1278117.79673536.1:51-89 HWI-ST574:196:D08GAACXX:5:2314:13319:21671
agcacattaaataacagtaataggaatttgttgggcgag
>_R_gnl|SRA|ERR1278117.79581086.2:3-41 HWI-
ST574:196:D08GAACXX:5:2314:20339:12493
agcacattaaataacagtaataggaatttgttgggcgag
>_R_gnl|SRA|ERR1278117.79229353.2:3-41 HWI-
ST574:196:D08GAACXX:5:2313:13604:76038
agcacattaaataacagtaataggaatttgttgggcgag
>gnl|SRA|ERR1278117.78991063.1:8-46 HWI-ST574:196:D08GAACXX:5:2313:5907:52149
agcacattaaataacagtaataggaatttgttgggcgag
>gnl|SRA|ERR1278117.78589584.1:2-40 HWI-ST574:196:D08GAACXX:5:2313:4215:11128
agcacattaaataacagtaataggaatttgttgggcgag
>gnl|SRA|ERR1278117.78572112.1:39-77 HWI-ST574:196:D08GAACXX:5:2313:20824:9222
agcacattaaataacagtaataggaatttgttgggcgag
>gnl|SRA|ERR1278117.78281173.1:36-74 HWI-ST574:196:D08GAACXX:5:2312:3930:78200
agcacattaaataacagtaataggaatttgttgggcgag
>_R_gnl|SRA|ERR1278117.78264479.2:4-42 HWI-ST574:196:D08GAACXX:5:2312:4503:76438
agcacattaaataacagtaataggaatttgttgggcgag
>gnl|SRA|ERR1278117.78113953.1:3-41 HWI-ST574:196:D08GAACXX:5:2312:10130:60742
agcacattaaataacagtaataggaatttgttgggcgag
>gnl|SRA|ERR1278117.78062369.1:42-80 HWI-ST574:196:D08GAACXX:5:2312:19047:55074
agcacattaaataacagtaataggaatttgttgggcgag
>gnl|SRA|ERR1278117.77866885.1:47-85 HWI-ST574:196:D08GAACXX:5:2312:13824:34664

agcacattaaataacagtaataggaatttgttgggcgag
>gnl|SRA|ERR1278117.77860406.1:5-43 HWI-ST574:196:D08GAACXX:5:2312:19165:33971
agcacattaaataacagtaataggaatttgttgggcgag
>gnl|SRA|ERR1278117.77803784.1:34-72 HWI-ST574:196:D08GAACXX:5:2312:3478:28169
agcacattaaataacagtaataggaatttgttgggcgag
>gnl|SRA|ERR1278117.77741719.1:28-66 HWI-ST574:196:D08GAACXX:5:2312:5176:21620
agcacattaaataacagtaataggaatttgttgggcgag
>gnl|SRA|ERR1278117.77622651.1:30-68 HWI-ST574:196:D08GAACXX:5:2312:17045:8983
agcacattaaataacagtaataggaatttgttgggcgag
>gnl|SRA|ERR1278117.77537339.1:11-49 HWI-ST574:196:D08GAACXX:5:2311:2699:98816
agcacattaaataacagtaataggaatttgttgggcgag
>gnl|SRA|ERR1278117.77387150.1:36-74 HWI-ST574:196:D08GAACXX:5:2311:6725:82761
agcacattaaataacagtaataggaatttgttgggcgag
>_R_gnl|SRA|ERR1278117.77371254.2:3-41 HWI-
ST574:196:D08GAACXX:5:2311:12175:81117
agcacattaaataacagtaataggaatttgttgggcgag
>gnl|SRA|ERR1278117.77025805.1:21-59 HWI-ST574:196:D08GAACXX:5:2311:3585:44109
agcacattaaataacagtaataggaatttgttgggcgag
>gnl|SRA|ERR1278117.76911991.1:3-41 HWI-ST574:196:D08GAACXX:5:2311:18492:31520
agcacattaaataacagtaataggaatttgttgggcgag
>_R_gnl|SRA|ERR1278117.76849340.2:3-41 HWI-
ST574:196:D08GAACXX:5:2311:13856:24983
agcacattaaataacagtaataggaatttgttgggcgag
>_R_gnl|SRA|ERR1278117.76800866.2:3-41 HWI-
ST574:196:D08GAACXX:5:2311:12659:19660
agcacattaaataacagtaataggaatttgttgggcgag
>gnl|SRA|ERR1278117.76588248.1:8-46 HWI-ST574:196:D08GAACXX:5:2310:9317:95266
agcacattaaataacagtaataggaatttgttgggcgag
>_R_gnl|SRA|ERR1278117.76582472.2:3-41 HWI-
ST574:196:D08GAACXX:5:2310:19084:94683
agcacattaaataacagtaataggaatttgttgggcgag
>_R_gnl|SRA|ERR1278117.76570801.2:2-40 HWI-
ST574:196:D08GAACXX:5:2310:16344:93293
agcacattaaataacagtaataggaatttgttgggcgag
>_R_gnl|SRA|ERR1278117.76466903.2:3-41 HWI-ST574:196:D08GAACXX:5:2310:3597:82077
agcacattaaataacagtaataggaatttgttgggcgag
>gnl|SRA|ERR1278117.76456789.1:36-74 HWI-ST574:196:D08GAACXX:5:2310:14569:80974
agcacattaaataacagtaataggaatttgttgggcgag
>_R_gnl|SRA|ERR1278117.76389568.2:3-41 HWI-ST574:196:D08GAACXX:5:2310:2437:73624
agcacattaaataacagtaataggaatttgttgggcgag
>_R_gnl|SRA|ERR1278117.76262083.2:62-100 HWI-
ST574:196:D08GAACXX:5:2310:15589:59484
agcacattaaataacagtaataggaatttgttgggcgag
>gnl|SRA|ERR1278117.76131514.1:11-49 HWI-ST574:196:D08GAACXX:5:2310:20580:44888
agcacattaaataacagtaataggaatttgttgggcgag
>gnl|SRA|ERR1278117.75721170.1:36-74 HWI-ST574:196:D08GAACXX:5:2309:15227:98208
agcacattaaataacagtaataggaatttgttgggcgag
>gnl|SRA|ERR1278117.75691277.1:49-87 HWI-ST574:196:D08GAACXX:5:2309:6275:94856
agcacattaaataacagtaataggaatttgttgggcgag
>gnl|SRA|ERR1278117.75671517.1:49-87 HWI-ST574:196:D08GAACXX:5:2309:8708:92700
agcacattaaataacagtaataggaatttgttgggcgag

>gnl|SRA|ERR1278117.75563242.1:47-85 HWI-ST574:196:D08GAACXX:5:2309:6570:80338
agcacattaaataacagtaataggaatttgttgggcgag
>gnl|SRA|ERR1278117.75461727.1:7-45 HWI-ST574:196:D08GAACXX:5:2309:8525:68797
agcacattaaataacagtaataggaatttgttgggcgag
>gnl|SRA|ERR1278117.75426494.1:43-81 HWI-ST574:196:D08GAACXX:5:2309:7573:64963
agcacattaaataacagtaataggaatttgttgggcgag
>gnl|SRA|ERR1278117.75238911.1:32-70 HWI-ST574:196:D08GAACXX:5:2309:18669:43319
agcacattaaataacagtaataggaatttgttgggcgag
>_R_gnl|SRA|ERR1278117.75230335.2:3-41 HWI-
ST574:196:D08GAACXX:5:2309:19696:42272
agcacattaaataacagtaataggaatttgttgggcgag
>gnl|SRA|ERR1278117.74937188.1:7-45 HWI-ST574:196:D08GAACXX:5:2309:19143:8824
agcacattaaataacagtaataggaatttgttgggcgag
>gnl|SRA|ERR1278117.74885853.1:6-44 HWI-ST574:196:D08GAACXX:5:2309:5639:3221
agcacattaaataacagtaataggaatttgttgggcgag
>_R_gnl|SRA|ERR1278117.74809527.2:1-39 HWI-
ST574:196:D08GAACXX:5:2308:13741:93234
agcacattaaataacagtaataggaatttgttgggcgag
>_R_gnl|SRA|ERR1278117.74725927.2:1-39 HWI-
ST574:196:D08GAACXX:5:2308:19953:83364
agcacattaaataacagtaataggaatttgttgggcgag
>gnl|SRA|ERR1278117.74657307.1:49-87 HWI-ST574:196:D08GAACXX:5:2308:4124:75625
agcacattaaataacagtaataggaatttgttgggcgag
>gnl|SRA|ERR1278117.74326795.1:37-75 HWI-ST574:196:D08GAACXX:5:2308:12088:36830
agcacattaaataacagtaataggaatttgttgggcgag
>gnl|SRA|ERR1278117.74035903.1:36-74 HWI-ST574:196:D08GAACXX:5:2308:8353:2655
agcacattaaataacagtaataggaatttgttgggcgag
>gnl|SRA|ERR1278117.73924338.1:34-72 HWI-ST574:196:D08GAACXX:5:2307:20675:88013
agcacattaaataacagtaataggaatttgttgggcgag
>gnl|SRA|ERR1278117.73793674.1:6-44 HWI-ST574:196:D08GAACXX:5:2307:16734:72750
agcacattaaataacagtaataggaatttgttgggcgag
>gnl|SRA|ERR1278117.73222667.1:36-74 HWI-ST574:196:D08GAACXX:5:2307:14482:3867
agcacattaaataacagtaataggaatttgttgggcgag
>gnl|SRA|ERR1278117.73215557.1:60-98 HWI-ST574:196:D08GAACXX:5:2307:5056:3122
agcacattaaataacagtaataggaatttgttgggcgag
>gnl|SRA|ERR1278117.73129706.1:1-39 HWI-ST574:196:D08GAACXX:5:2306:18568:91440
agcacattaaataacagtaataggaatttgttgggcgag
>_R_gnl|SRA|ERR1278117.73028060.2:3-41 HWI-
ST574:196:D08GAACXX:5:2306:10759:79056
agcacattaaataacagtaataggaatttgttgggcgag
>gnl|SRA|ERR1278117.72889753.1:29-67 HWI-ST574:196:D08GAACXX:5:2306:18142:62025
agcacattaaataacagtaataggaatttgttgggcgag
>gnl|SRA|ERR1278117.72786782.1:2-40 HWI-ST574:196:D08GAACXX:5:2306:7330:49708
agcacattaaataacagtaataggaatttgttgggcgag
>gnl|SRA|ERR1278117.72752543.1:9-47 HWI-ST574:196:D08GAACXX:5:2306:8866:45500
agcacattaaataacagtaataggaatttgttgggcgag
>gnl|SRA|ERR1278117.72443988.1:46-84 HWI-ST574:196:D08GAACXX:5:2306:15470:6944
agcacattaaataacagtaataggaatttgttgggcgag
>gnl|SRA|ERR1278117.72187199.1:62-100 HWI-ST574:196:D08GAACXX:5:2305:4722:73768
agcacattaaataacagtaataggaatttgttgggcgag
>gnl|SRA|ERR1278117.72002825.1:50-88 HWI-ST574:196:D08GAACXX:5:2305:10140:50722

agcacattaaataacagtaataggaatttgttgggcgag
>_R_gnl|SRA|ERR1278117.71475863.2:3-41 HWI-ST574:196:D08GAACXX:5:2304:14409:82484
agcacattaaataacagtaataggaatttgttgggcgag
>gnl|SRA|ERR1278117.71366633.1:60-98 HWI-ST574:196:D08GAACXX:5:2304:11506:68297
agcacattaaataacagtaataggaatttgttgggcgag
>gnl|SRA|ERR1278117.71233312.1:23-61 HWI-ST574:196:D08GAACXX:5:2304:7286:51028
agcacattaaataacagtaataggaatttgttgggcgag
>gnl|SRA|ERR1278117.71050133.1:48-86 HWI-ST574:196:D08GAACXX:5:2304:2744:27405
agcacattaaataacagtaataggaatttgttgggcgag
>gnl|SRA|ERR1278117.71015908.1:36-74 HWI-ST574:196:D08GAACXX:5:2304:3934:22823
agcacattaaataacagtaataggaatttgttgggcgag
>gnl|SRA|ERR1278117.70936632.1:11-49 HWI-ST574:196:D08GAACXX:5:2304:19875:12342
agcacattaaataacagtaataggaatttgttgggcgag
>gnl|SRA|ERR1278117.70719721.1:46-84 HWI-ST574:196:D08GAACXX:5:2303:17565:82905
agcacattaaataacagtaataggaatttgttgggcgag
>gnl|SRA|ERR1278117.70672026.1:29-67 HWI-ST574:196:D08GAACXX:5:2303:14336:76682
agcacattaaataacagtaataggaatttgttgggcgag
>_R_gnl|SRA|ERR1278117.70650207.2:3-41 HWI-ST574:196:D08GAACXX:5:2303:4762:73784
agcacattaaataacagtaataggaatttgttgggcgag
>gnl|SRA|ERR1278117.70397069.1:43-81 HWI-ST574:196:D08GAACXX:5:2303:16153:40153
agcacattaaataacagtaataggaatttgttgggcgag
>gnl|SRA|ERR1278117.70160959.1:2-40 HWI-ST574:196:D08GAACXX:5:2303:3254:8687
agcacattaaataacagtaataggaatttgttgggcgag
>gnl|SRA|ERR1278117.69947094.1:31-69 HWI-ST574:196:D08GAACXX:5:2302:13594:78355
agcacattaaataacagtaataggaatttgttgggcgag
>gnl|SRA|ERR1278117.69496294.1:5-43 HWI-ST574:196:D08GAACXX:5:2302:20431:16046
agcacattaaataacagtaataggaatttgttgggcgag
>gnl|SRA|ERR1278117.69472567.1:7-45 HWI-ST574:196:D08GAACXX:5:2302:13566:12815
agcacattaaataacagtaataggaatttgttgggcgag
>gnl|SRA|ERR1278117.69470076.1:62-100 HWI-ST574:196:D08GAACXX:5:2302:6298:12506
agcacattaaataacagtaataggaatttgttgggcgag
>gnl|SRA|ERR1278117.69379870.1:29-67 HWI-ST574:196:D08GAACXX:5:2301:11246:98784
agcacattaaataacagtaataggaatttgttgggcgag
>gnl|SRA|ERR1278117.69176313.1:55-93 HWI-ST574:196:D08GAACXX:5:2301:19317:70423
agcacattaaataacagtaataggaatttgttgggcgag
>gnl|SRA|ERR1278117.69166271.1:61-99 HWI-ST574:196:D08GAACXX:5:2301:8024:69185
agcacattaaataacagtaataggaatttgttgggcgag
>_R_gnl|SRA|ERR1278117.69159513.2:1-39 HWI-ST574:196:D08GAACXX:5:2301:11891:68101
agcacattaaataacagtaataggaatttgttgggcgag
>_R_gnl|SRA|ERR1278117.69055353.2:1-39 HWI-ST574:196:D08GAACXX:5:2301:18010:53439
agcacattaaataacagtaataggaatttgttgggcgag
>gnl|SRA|ERR1278117.68943161.1:33-71 HWI-ST574:196:D08GAACXX:5:2301:16377:37633
agcacattaaataacagtaataggaatttgttgggcgag
>gnl|SRA|ERR1278117.68811933.1:30-68 HWI-ST574:196:D08GAACXX:5:2301:5022:19279
agcacattaaataacagtaataggaatttgttgggcgag
>gnl|SRA|ERR1278117.68545118.1:50-88 HWI-ST574:196:D08GAACXX:5:2216:4218:88995
agcacattaaataacagtaataggaatttgttgggcgag
>gnl|SRA|ERR1278117.68526736.1:34-72 HWI-ST574:196:D08GAACXX:5:2216:19843:87159

agcacattaaataacagtaataggaatttgttgggcgag
>gnl|SRA|ERR1278117.68069013.1:11-49 HWI-ST574:196:D08GAACXX:5:2216:8116:47714
agcacattaaataacagtaataggaatttgttgggcgag
>gnl|SRA|ERR1278117.67927131.1:9-47 HWI-ST574:196:D08GAACXX:5:2216:21026:34855
agcacattaaataacagtaataggaatttgttgggcgag
>_R_gnl|SRA|ERR1278117.67809808.2:4-42 HWI-ST574:196:D08GAACXX:5:2216:2555:24519
agcacattaaataacagtaataggaatttgttgggcgag
>gnl|SRA|ERR1278117.67486216.1:49-87 HWI-ST574:196:D08GAACXX:5:2215:4729:94164
agcacattaaataacagtaataggaatttgttgggcgag
>gnl|SRA|ERR1278117.67469973.1:30-68 HWI-ST574:196:D08GAACXX:5:2215:5185:92541
agcacattaaataacagtaataggaatttgttgggcgag
>gnl|SRA|ERR1278117.66881770.1:36-74 HWI-ST574:196:D08GAACXX:5:2215:15760:37385
agcacattaaataacagtaataggaatttgttgggcgag
>gnl|SRA|ERR1278117.66723588.1:47-85 HWI-ST574:196:D08GAACXX:5:2215:13184:22453
agcacattaaataacagtaataggaatttgttgggcgag
>gnl|SRA|ERR1278117.66589263.1:30-68 HWI-ST574:196:D08GAACXX:5:2215:9163:9712
agcacattaaataacagtaataggaatttgttgggcgag
>gnl|SRA|ERR1278117.66283742.1:59-97 HWI-ST574:196:D08GAACXX:5:2214:12678:79064
agcacattaaataacagtaataggaatttgttgggcgag
>gnl|SRA|ERR1278117.66035508.1:9-47 HWI-ST574:196:D08GAACXX:5:2214:20646:54984
agcacattaaataacagtaataggaatttgttgggcgag
>gnl|SRA|ERR1278117.66030007.1:3-41 HWI-ST574:196:D08GAACXX:5:2214:17953:54265
agcacattaaataacagtaataggaatttgttgggcgag
>gnl|SRA|ERR1278117.65911749.1:59-97 HWI-ST574:196:D08GAACXX:5:2214:8773:42865
agcacattaaataacagtaataggaatttgttgggcgag
>gnl|SRA|ERR1278117.65883580.1:44-82 HWI-ST574:196:D08GAACXX:5:2214:5775:40227
agcacattaaataacagtaataggaatttgttgggcgag
>gnl|SRA|ERR1278117.65844674.1:48-86 HWI-ST574:196:D08GAACXX:5:2214:19923:36071
agcacattaaataacagtaataggaatttgttgggcgag
>gnl|SRA|ERR1278117.65707377.1:53-91 HWI-ST574:196:D08GAACXX:5:2214:16362:22730
agcacattaaataacagtaataggaatttgttgggcgag
>_R_gnl|SRA|ERR1278117.64764809.2:3-41 HWI-
ST574:196:D08GAACXX:5:2213:18931:22625
agcacattaaataacagtaataggaatttgttgggcgag
>_R_gnl|SRA|ERR1278117.64680819.2:3-41 HWI-
ST574:196:D08GAACXX:5:2213:12140:13797
agcacattaaataacagtaataggaatttgttgggcgag
>gnl|SRA|ERR1278117.64546171.1:32-70 HWI-ST574:196:D08GAACXX:5:2212:19473:98418
agcacattaaataacagtaataggaatttgttgggcgag
>gnl|SRA|ERR1278117.64493522.1:8-46 HWI-ST574:196:D08GAACXX:5:2212:15678:92757
agcacattaaataacagtaataggaatttgttgggcgag
>gnl|SRA|ERR1278117.64490301.1:7-45 HWI-ST574:196:D08GAACXX:5:2212:8539:92617
agcacattaaataacagtaataggaatttgttgggcgag
>gnl|SRA|ERR1278117.64400574.1:59-97 HWI-ST574:196:D08GAACXX:5:2212:1448:83189
agcacattaaataacagtaataggaatttgttgggcgag
>gnl|SRA|ERR1278117.64391638.1:11-49 HWI-ST574:196:D08GAACXX:5:2212:6353:82143
agcacattaaataacagtaataggaatttgttgggcgag
>gnl|SRA|ERR1278117.64256761.1:37-75 HWI-ST574:196:D08GAACXX:5:2212:20694:67565
agcacattaaataacagtaataggaatttgttgggcgag
>gnl|SRA|ERR1278117.64194664.1:6-44 HWI-ST574:196:D08GAACXX:5:2212:11721:61204
agcacattaaataacagtaataggaatttgttgggcgag

>gnl|SRA|ERR1278117.64160814.1:49-87 HWI-ST574:196:D08GAACXX:5:2212:20012:57403
agcacattaaataacagtaataggaatttgttgggcgag
>gnl|SRA|ERR1278117.63939688.1:62-100 HWI-ST574:196:D08GAACXX:5:2212:1124:33953
agcacattaaataacagtaataggaatttgttgggcgag
>gnl|SRA|ERR1278117.63682976.1:47-85 HWI-ST574:196:D08GAACXX:5:2212:3133:6210
agcacattaaataacagtaataggaatttgttgggcgag
>gnl|SRA|ERR1278117.63641769.1:21-59 HWI-ST574:196:D08GAACXX:5:2211:14982:100372
agcacattaaataacagtaataggaatttgttgggcgag
>gnl|SRA|ERR1278117.63606829.1:61-99 HWI-ST574:196:D08GAACXX:5:2211:10613:96714
agcacattaaataacagtaataggaatttgttgggcgag
>gnl|SRA|ERR1278117.63576566.1:33-71 HWI-ST574:196:D08GAACXX:5:2211:6412:93253
agcacattaaataacagtaataggaatttgttgggcgag
>gnl|SRA|ERR1278117.63502090.1:6-44 HWI-ST574:196:D08GAACXX:5:2211:14447:85156
agcacattaaataacagtaataggaatttgttgggcgag
>gnl|SRA|ERR1278117.63465768.1:8-46 HWI-ST574:196:D08GAACXX:5:2211:17712:81149
agcacattaaataacagtaataggaatttgttgggcgag
>gnl|SRA|ERR1278117.63232804.1:36-74 HWI-ST574:196:D08GAACXX:5:2211:11366:55567
agcacattaaataacagtaataggaatttgttgggcgag
>gnl|SRA|ERR1278117.63037335.1:59-97 HWI-ST574:196:D08GAACXX:5:2211:13317:34011
agcacattaaataacagtaataggaatttgttgggcgag
>gnl|SRA|ERR1278117.62650438.1:7-45 HWI-ST574:196:D08GAACXX:5:2210:5065:90115
agcacattaaataacagtaataggaatttgttgggcgag
>_R_gnl|SRA|ERR1278117.62536218.2:4-42 HWI-
ST574:196:D08GAACXX:5:2210:19221:77233
agcacattaaataacagtaataggaatttgttgggcgag
>gnl|SRA|ERR1278117.62477046.1:36-74 HWI-ST574:196:D08GAACXX:5:2210:10610:70584
agcacattaaataacagtaataggaatttgttgggcgag
>_R_gnl|SRA|ERR1278117.62371984.2:3-41 HWI-
ST574:196:D08GAACXX:5:2210:10794:58943
agcacattaaataacagtaataggaatttgttgggcgag
>gnl|SRA|ERR1278117.62275718.1:30-68 HWI-ST574:196:D08GAACXX:5:2210:2534:48237
agcacattaaataacagtaataggaatttgttgggcgag
>gnl|SRA|ERR1278117.62179585.1:49-87 HWI-ST574:196:D08GAACXX:5:2210:18772:37012
agcacattaaataacagtaataggaatttgttgggcgag
>gnl|SRA|ERR1278117.62165315.1:3-41 HWI-ST574:196:D08GAACXX:5:2210:9097:35501
agcacattaaataacagtaataggaatttgttgggcgag
>gnl|SRA|ERR1278117.62157102.1:11-49 HWI-ST574:196:D08GAACXX:5:2210:15285:34644
agcacattaaataacagtaataggaatttgttgggcgag
>gnl|SRA|ERR1278117.62122582.1:62-100 HWI-ST574:196:D08GAACXX:5:2210:2710:30938
agcacattaaataacagtaataggaatttgttgggcgag
>_R_gnl|SRA|ERR1278117.62065228.2:4-42 HWI-
ST574:196:D08GAACXX:5:2210:18683:24131
agcacattaaataacagtaataggaatttgttgggcgag
>gnl|SRA|ERR1278117.62030303.1:20-58 HWI-ST574:196:D08GAACXX:5:2210:20493:20164
agcacattaaataacagtaataggaatttgttgggcgag
>_R_gnl|SRA|ERR1278117.61920410.2:3-41 HWI-ST574:196:D08GAACXX:5:2210:1159:7948
agcacattaaataacagtaataggaatttgttgggcgag
>gnl|SRA|ERR1278117.61887226.1:32-70 HWI-ST574:196:D08GAACXX:5:2210:18964:3796
agcacattaaataacagtaataggaatttgttgggcgag
>_R_gnl|SRA|ERR1278117.61886725.2:3-41 HWI-ST574:196:D08GAACXX:5:2210:14070:3957
agcacattaaataacagtaataggaatttgttgggcgag

>gnl|SRA|ERR1278117.61884374.1:10-48 HWI-ST574:196:D08GAACXX:5:2210:12451:3538
agcacattaaataacagtaataggaatttgttgggcgag
>gnl|SRA|ERR1278117.61676301.1:11-49 HWI-ST574:196:D08GAACXX:5:2209:13578:78624
agcacattaaataacagtaataggaatttgttgggcgag
>gnl|SRA|ERR1278117.61492482.1:20-58 HWI-ST574:196:D08GAACXX:5:2209:15537:57254
agcacattaaataacagtaataggaatttgttgggcgag
>gnl|SRA|ERR1278117.61457957.1:49-87 HWI-ST574:196:D08GAACXX:5:2209:15369:53447
agcacattaaataacagtaataggaatttgttgggcgag
>gnl|SRA|ERR1278117.61385992.1:29-67 HWI-ST574:196:D08GAACXX:5:2209:11942:45238
agcacattaaataacagtaataggaatttgttgggcgag
>gnl|SRA|ERR1278117.60844923.1:6-44 HWI-ST574:196:D08GAACXX:5:2208:21343:80885
agcacattaaataacagtaataggaatttgttgggcgag
>gnl|SRA|ERR1278117.60800527.1:8-46 HWI-ST574:196:D08GAACXX:5:2208:4163:75763
agcacattaaataacagtaataggaatttgttgggcgag
>gnl|SRA|ERR1278117.60780589.1:30-68 HWI-ST574:196:D08GAACXX:5:2208:17860:73440
agcacattaaataacagtaataggaatttgttgggcgag
>gnl|SRA|ERR1278117.60764026.1:62-100 HWI-ST574:196:D08GAACXX:5:2208:3074:71600
agcacattaaataacagtaataggaatttgttgggcgag
>gnl|SRA|ERR1278117.60751202.1:2-40 HWI-ST574:196:D08GAACXX:5:2208:2792:70052
agcacattaaataacagtaataggaatttgttgggcgag
>gnl|SRA|ERR1278117.60681790.1:33-71 HWI-ST574:196:D08GAACXX:5:2208:8560:61972
agcacattaaataacagtaataggaatttgttgggcgag
>gnl|SRA|ERR1278117.60645462.1:41-79 HWI-ST574:196:D08GAACXX:5:2208:6768:57505
agcacattaaataacagtaataggaatttgttgggcgag
>gnl|SRA|ERR1278117.60625774.1:30-68 HWI-ST574:196:D08GAACXX:5:2208:18095:55189
agcacattaaataacagtaataggaatttgttgggcgag
>_R_gnl|SRA|ERR1278117.60378279.2:3-41 HWI-ST574:196:D08GAACXX:5:2208:6014:25853
agcacattaaataacagtaataggaatttgttgggcgag
>gnl|SRA|ERR1278117.60195860.1:20-58 HWI-ST574:196:D08GAACXX:5:2208:9749:4030
agcacattaaataacagtaataggaatttgttgggcgag
>gnl|SRA|ERR1278117.59889961.1:62-100 HWI-ST574:196:D08GAACXX:5:2207:15348:66099
agcacattaaataacagtaataggaatttgttgggcgag
>gnl|SRA|ERR1278117.59727389.1:6-44 HWI-ST574:196:D08GAACXX:5:2207:14575:46292
agcacattaaataacagtaataggaatttgttgggcgag
>gnl|SRA|ERR1278117.59689378.1:59-97 HWI-ST574:196:D08GAACXX:5:2207:3023:41798
agcacattaaataacagtaataggaatttgttgggcgag
>gnl|SRA|ERR1278117.59626482.1:50-88 HWI-ST574:196:D08GAACXX:5:2207:6981:34109
agcacattaaataacagtaataggaatttgttgggcgag
>gnl|SRA|ERR1278117.59486153.1:42-80 HWI-ST574:196:D08GAACXX:5:2207:18555:16818
agcacattaaataacagtaataggaatttgttgggcgag
>gnl|SRA|ERR1278117.59359845.1:53-91 HWI-ST574:196:D08GAACXX:5:2206:14706:100406
agcacattaaataacagtaataggaatttgttgggcgag
>gnl|SRA|ERR1278117.59355308.1:1-39 HWI-ST574:196:D08GAACXX:5:2206:10166:99940
agcacattaaataacagtaataggaatttgttgggcgag
>gnl|SRA|ERR1278117.59052614.1:6-44 HWI-ST574:196:D08GAACXX:5:2206:16394:62713
agcacattaaataacagtaataggaatttgttgggcgag
>gnl|SRA|ERR1278117.59004024.1:2-40 HWI-ST574:196:D08GAACXX:5:2206:14610:56505
agcacattaaataacagtaataggaatttgttgggcgag
>gnl|SRA|ERR1278117.58775472.1:42-80 HWI-ST574:196:D08GAACXX:5:2206:20698:28197
agcacattaaataacagtaataggaatttgttgggcgag
>gnl|SRA|ERR1278117.58759507.1:36-74 HWI-ST574:196:D08GAACXX:5:2206:1565:26285

agcacattaaataacagtaataggaatttgttgggcgag
>gnl|SRA|ERR1278117.58695786.1:2-40 HWI-ST574:196:D08GAACXX:5:2206:7321:18482
agcacattaaataacagtaataggaatttgttgggcgag
>gnl|SRA|ERR1278117.58567070.1:47-85 HWI-ST574:196:D08GAACXX:5:2206:14279:2072
agcacattaaataacagtaataggaatttgttgggcgag
>gnl|SRA|ERR1278117.58445437.1:36-74 HWI-ST574:196:D08GAACXX:5:2205:2868:85902
agcacattaaataacagtaataggaatttgttgggcgag
>gnl|SRA|ERR1278117.58351843.1:5-43 HWI-ST574:196:D08GAACXX:5:2205:17518:73942
agcacattaaataacagtaataggaatttgttgggcgag
>_R_gnl|SRA|ERR1278117.57917400.2:1-39 HWI-ST574:196:D08GAACXX:5:2205:4488:18905
agcacattaaataacagtaataggaatttgttgggcgag
>gnl|SRA|ERR1278117.57894938.1:37-75 HWI-ST574:196:D08GAACXX:5:2205:11145:15824
agcacattaaataacagtaataggaatttgttgggcgag
>gnl|SRA|ERR1278117.57799115.1:12-50 HWI-ST574:196:D08GAACXX:5:2205:11021:3546
agcacattaaataacagtaataggaatttgttgggcgag
>gnl|SRA|ERR1278117.57663421.1:10-48 HWI-ST574:196:D08GAACXX:5:2204:6605:85052
agcacattaaataacagtaataggaatttgttgggcgag
>gnl|SRA|ERR1278117.57636652.1:47-85 HWI-ST574:196:D08GAACXX:5:2204:11094:81513
agcacattaaataacagtaataggaatttgttgggcgag
>gnl|SRA|ERR1278117.57536218.1:30-68 HWI-ST574:196:D08GAACXX:5:2204:9374:68731
agcacattaaataacagtaataggaatttgttgggcgag
>gnl|SRA|ERR1278117.57399230.1:50-88 HWI-ST574:196:D08GAACXX:5:2204:10973:50910
agcacattaaataacagtaataggaatttgttgggcgag
>gnl|SRA|ERR1278117.57370915.1:4-42 HWI-ST574:196:D08GAACXX:5:2204:17920:47081
agcacattaaataacagtaataggaatttgttgggcgag
>gnl|SRA|ERR1278117.57241096.1:5-43 HWI-ST574:196:D08GAACXX:5:2204:4252:30425
agcacattaaataacagtaataggaatttgttgggcgag
>gnl|SRA|ERR1278117.57217864.1:44-82 HWI-ST574:196:D08GAACXX:5:2204:1289:27266
agcacattaaataacagtaataggaatttgttgggcgag
>gnl|SRA|ERR1278117.57185691.1:4-42 HWI-ST574:196:D08GAACXX:5:2204:6970:23037
agcacattaaataacagtaataggaatttgttgggcgag
>gnl|SRA|ERR1278117.57026929.1:34-72 HWI-ST574:196:D08GAACXX:5:2204:20815:2208
agcacattaaataacagtaataggaatttgttgggcgag
>_R_gnl|SRA|ERR1278117.56961702.2:3-41 HWI-ST574:196:D08GAACXX:5:2203:4867:92721
agcacattaaataacagtaataggaatttgttgggcgag
>gnl|SRA|ERR1278117.56929191.1:6-44 HWI-ST574:196:D08GAACXX:5:2203:17244:88029
agcacattaaataacagtaataggaatttgttgggcgag
>gnl|SRA|ERR1278117.56698899.1:28-66 HWI-ST574:196:D08GAACXX:5:2203:19102:57577
agcacattaaataacagtaataggaatttgttgggcgag
>gnl|SRA|ERR1278117.56585060.1:36-74 HWI-ST574:196:D08GAACXX:5:2203:4428:42726
agcacattaaataacagtaataggaatttgttgggcgag
>gnl|SRA|ERR1278117.56466306.1:36-74 HWI-ST574:196:D08GAACXX:5:2203:20875:26603
agcacattaaataacagtaataggaatttgttgggcgag
>gnl|SRA|ERR1278117.56146089.1:48-86 HWI-ST574:196:D08GAACXX:5:2202:13466:82449
agcacattaaataacagtaataggaatttgttgggcgag
>gnl|SRA|ERR1278117.56034956.1:11-49 HWI-ST574:196:D08GAACXX:5:2202:17126:67080
agcacattaaataacagtaataggaatttgttgggcgag
>gnl|SRA|ERR1278117.56033096.1:49-87 HWI-ST574:196:D08GAACXX:5:2202:16227:66768
agcacattaaataacagtaataggaatttgttgggcgag
>gnl|SRA|ERR1278117.55749627.1:7-45 HWI-ST574:196:D08GAACXX:5:2202:20552:27706
agcacattaaataacagtaataggaatttgttgggcgag

>gnl|SRA|ERR1278117.55736060.1:9-47 HWI-ST574:196:D08GAACXX:5:2202:11166:25777
agcacattaaataacagtaataggaatttgttgggcgag
>gnl|SRA|ERR1278117.55724260.1:61-99 HWI-ST574:196:D08GAACXX:5:2202:19184:24131
agcacattaaataacagtaataggaatttgttgggcgag
>gnl|SRA|ERR1278117.55708784.1:31-69 HWI-ST574:196:D08GAACXX:5:2202:6955:22015
agcacattaaataacagtaataggaatttgttgggcgag
>_R_gnl|SRA|ERR1278117.55651317.2:4-42 HWI-ST574:196:D08GAACXX:5:2202:9730:14112
agcacattaaataacagtaataggaatttgttgggcgag
>gnl|SRA|ERR1278117.55443530.1:30-68 HWI-ST574:196:D08GAACXX:5:2201:11491:84207
agcacattaaataacagtaataggaatttgttgggcgag
>gnl|SRA|ERR1278117.55283899.1:9-47 HWI-ST574:196:D08GAACXX:5:2201:21225:61706
agcacattaaataacagtaataggaatttgttgggcgag
>gnl|SRA|ERR1278117.55233566.1:10-48 HWI-ST574:196:D08GAACXX:5:2201:13204:54505
agcacattaaataacagtaataggaatttgttgggcgag
>_R_gnl|SRA|ERR1278117.55120158.2:3-41 HWI-ST574:196:D08GAACXX:5:2201:6293:38839
agcacattaaataacagtaataggaatttgttgggcgag
>gnl|SRA|ERR1278117.55026349.1:6-44 HWI-ST574:196:D08GAACXX:5:2201:13807:25749
agcacattaaataacagtaataggaatttgttgggcgag
>gnl|SRA|ERR1278117.54984185.1:49-87 HWI-ST574:196:D08GAACXX:5:2201:18750:19630
agcacattaaataacagtaataggaatttgttgggcgag
>gnl|SRA|ERR1278117.54358921.1:2-40 HWI-ST574:196:D08GAACXX:5:2116:4024:58218
agcacattaaataacagtaataggaatttgttgggcgag
>gnl|SRA|ERR1278117.54070526.1:33-71 HWI-ST574:196:D08GAACXX:5:2116:4411:32365
agcacattaaataacagtaataggaatttgttgggcgag
>gnl|SRA|ERR1278117.53516040.1:49-87 HWI-ST574:196:D08GAACXX:5:2115:9243:80283
agcacattaaataacagtaataggaatttgttgggcgag
>gnl|SRA|ERR1278117.53402121.1:31-69 HWI-ST574:196:D08GAACXX:5:2115:13924:69563
agcacattaaataacagtaataggaatttgttgggcgag
>gnl|SRA|ERR1278117.53177420.1:59-97 HWI-ST574:196:D08GAACXX:5:2115:16036:48444
agcacattaaataacagtaataggaatttgttgggcgag
>gnl|SRA|ERR1278117.53101683.1:49-87 HWI-ST574:196:D08GAACXX:5:2115:19198:41177
agcacattaaataacagtaataggaatttgttgggcgag
>gnl|SRA|ERR1278117.52995796.1:38-76 HWI-ST574:196:D08GAACXX:5:2115:9320:31072
agcacattaaataacagtaataggaatttgttgggcgag
>gnl|SRA|ERR1278117.52932206.1:7-45 HWI-ST574:196:D08GAACXX:5:2115:3918:25213
agcacattaaataacagtaataggaatttgttgggcgag
>gnl|SRA|ERR1278117.52834335.1:28-66 HWI-ST574:196:D08GAACXX:5:2115:15367:15739
agcacattaaataacagtaataggaatttgttgggcgag
>gnl|SRA|ERR1278117.52696346.1:14-52 HWI-ST574:196:D08GAACXX:5:2115:9308:2335
agcacattaaataacagtaataggaatttgttgggcgag
>gnl|SRA|ERR1278117.52668753.1:7-45 HWI-ST574:196:D08GAACXX:5:2114:3228:98674
agcacattaaataacagtaataggaatttgttgggcgag
>gnl|SRA|ERR1278117.51958591.1:55-93 HWI-ST574:196:D08GAACXX:5:2114:7036:28282
agcacattaaataacagtaataggaatttgttgggcgag
>gnl|SRA|ERR1278117.51935241.1:47-85 HWI-ST574:196:D08GAACXX:5:2114:1204:26206
agcacattaaataacagtaataggaatttgttgggcgag
>gnl|SRA|ERR1278117.51812188.1:55-93 HWI-ST574:196:D08GAACXX:5:2114:3508:13584
agcacattaaataacagtaataggaatttgttgggcgag
>gnl|SRA|ERR1278117.51675729.1:48-86 HWI-ST574:196:D08GAACXX:5:2113:19401:97669
agcacattaaataacagtaataggaatttgttgggcgag
>gnl|SRA|ERR1278117.51064849.1:31-69 HWI-ST574:196:D08GAACXX:5:2113:6998:33698

agcacattaaataacagtaataggaatttgttgggcgag
>gnl|SRA|ERR1278117.51017684.1:31-69 HWI-ST574:196:D08GAACXX:5:2113:11061:28551
agcacattaaataacagtaataggaatttgttgggcgag
>_R_gnl|SRA|ERR1278117.50764198.2:3-41 HWI-
ST574:196:D08GAACXX:5:2112:12842:100529
agcacattaaataacagtaataggaatttgttgggcgag
>gnl|SRA|ERR1278117.50720921.1:32-70 HWI-ST574:196:D08GAACXX:5:2112:8740:96066
agcacattaaataacagtaataggaatttgttgggcgag
>gnl|SRA|ERR1278117.50630346.1:10-48 HWI-ST574:196:D08GAACXX:5:2112:10771:86436
agcacattaaataacagtaataggaatttgttgggcgag
>gnl|SRA|ERR1278117.49723381.1:44-82 HWI-ST574:196:D08GAACXX:5:2111:5004:87434
agcacattaaataacagtaataggaatttgttgggcgag
>gnl|SRA|ERR1278117.49700179.1:49-87 HWI-ST574:196:D08GAACXX:5:2111:19895:84524
agcacattaaataacagtaataggaatttgttgggcgag
>gnl|SRA|ERR1278117.49680073.1:34-72 HWI-ST574:196:D08GAACXX:5:2111:3538:82564
agcacattaaataacagtaataggaatttgttgggcgag
>gnl|SRA|ERR1278117.49540254.1:42-80 HWI-ST574:196:D08GAACXX:5:2111:11935:67031
agcacattaaataacagtaataggaatttgttgggcgag
>_R_gnl|SRA|ERR1278117.49382354.2:1-39 HWI-ST574:196:D08GAACXX:5:2111:9516:49732
agcacattaaataacagtaataggaatttgttgggcgag
>gnl|SRA|ERR1278117.49339521.1:60-98 HWI-ST574:196:D08GAACXX:5:2111:9919:44896
agcacattaaataacagtaataggaatttgttgggcgag
>gnl|SRA|ERR1278117.48927108.1:47-85 HWI-ST574:196:D08GAACXX:5:2110:19104:97450
agcacattaaataacagtaataggaatttgttgggcgag
>gnl|SRA|ERR1278117.48897772.1:2-40 HWI-ST574:196:D08GAACXX:5:2110:14910:94152
agcacattaaataacagtaataggaatttgttgggcgag
>gnl|SRA|ERR1278117.48474092.1:47-85 HWI-ST574:196:D08GAACXX:5:2110:4643:46180
agcacattaaataacagtaataggaatttgttgggcgag
>gnl|SRA|ERR1278117.48366672.1:2-40 HWI-ST574:196:D08GAACXX:5:2110:20153:33649
agcacattaaataacagtaataggaatttgttgggcgag
>gnl|SRA|ERR1278117.48079361.1:49-87 HWI-ST574:196:D08GAACXX:5:2109:11184:99272
agcacattaaataacagtaataggaatttgttgggcgag
>gnl|SRA|ERR1278117.48008988.1:2-40 HWI-ST574:196:D08GAACXX:5:2109:14308:91184
agcacattaaataacagtaataggaatttgttgggcgag
>gnl|SRA|ERR1278117.47982410.1:49-87 HWI-ST574:196:D08GAACXX:5:2109:5076:88002
agcacattaaataacagtaataggaatttgttgggcgag
>gnl|SRA|ERR1278117.47909412.1:13-51 HWI-ST574:196:D08GAACXX:5:2109:3710:79745
agcacattaaataacagtaataggaatttgttgggcgag
>gnl|SRA|ERR1278117.47844590.1:30-68 HWI-ST574:196:D08GAACXX:5:2109:19388:71951
agcacattaaataacagtaataggaatttgttgggcgag
>gnl|SRA|ERR1278117.47670435.1:5-43 HWI-ST574:196:D08GAACXX:5:2109:3091:51726
agcacattaaataacagtaataggaatttgttgggcgag
>gnl|SRA|ERR1278117.47643997.1:5-43 HWI-ST574:196:D08GAACXX:5:2109:12306:48264
agcacattaaataacagtaataggaatttgttgggcgag
>gnl|SRA|ERR1278117.47459497.1:31-69 HWI-ST574:196:D08GAACXX:5:2109:15612:26636
agcacattaaataacagtaataggaatttgttgggcgag
>gnl|SRA|ERR1278117.47043842.1:12-50 HWI-ST574:196:D08GAACXX:5:2108:15172:76038
agcacattaaataacagtaataggaatttgttgggcgag
>gnl|SRA|ERR1278117.47012409.1:10-48 HWI-ST574:196:D08GAACXX:5:2108:11883:72380
agcacattaaataacagtaataggaatttgttgggcgag
>gnl|SRA|ERR1278117.46973619.1:36-74 HWI-ST574:196:D08GAACXX:5:2108:17483:67581

agcacattaaataacagtaataggaatttgttgggcgag
>gnl|SRA|ERR1278117.46783121.1:14-52 HWI-ST574:196:D08GAACXX:5:2108:3319:44770
agcacattaaataacagtaataggaatttgttgggcgag
>_R_gnl|SRA|ERR1278117.46709338.2:3-41 HWI-
ST574:196:D08GAACXX:5:2108:14202:35997
agcacattaaataacagtaataggaatttgttgggcgag
>gnl|SRA|ERR1278117.46582350.1:7-45 HWI-ST574:196:D08GAACXX:5:2108:9511:20707
agcacattaaataacagtaataggaatttgttgggcgag
>gnl|SRA|ERR1278117.46519829.1:44-82 HWI-ST574:196:D08GAACXX:5:2108:4558:13048
agcacattaaataacagtaataggaatttgttgggcgag
>gnl|SRA|ERR1278117.46474548.1:30-68 HWI-ST574:196:D08GAACXX:5:2108:2342:7631
agcacattaaataacagtaataggaatttgttgggcgag
>_R_gnl|SRA|ERR1278117.46048259.2:1-39 HWI-ST574:196:D08GAACXX:5:2107:8221:53868
agcacattaaataacagtaataggaatttgttgggcgag
>gnl|SRA|ERR1278117.46022957.1:21-59 HWI-ST574:196:D08GAACXX:5:2107:15349:50523
agcacattaaataacagtaataggaatttgttgggcgag
>gnl|SRA|ERR1278117.45644761.1:62-100 HWI-ST574:196:D08GAACXX:5:2107:4910:3392
agcacattaaataacagtaataggaatttgttgggcgag
>gnl|SRA|ERR1278117.45541431.1:30-68 HWI-ST574:196:D08GAACXX:5:2106:7198:89203
agcacattaaataacagtaataggaatttgttgggcgag
>gnl|SRA|ERR1278117.45326673.1:4-42 HWI-ST574:196:D08GAACXX:5:2106:19033:61751
agcacattaaataacagtaataggaatttgttgggcgag
>_R_gnl|SRA|ERR1278117.45050076.2:3-41 HWI-ST574:196:D08GAACXX:5:2106:8904:26683
agcacattaaataacagtaataggaatttgttgggcgag
>gnl|SRA|ERR1278117.44952384.1:9-47 HWI-ST574:196:D08GAACXX:5:2106:20915:13808
agcacattaaataacagtaataggaatttgttgggcgag
>_R_gnl|SRA|ERR1278117.44950483.2:3-41 HWI-
ST574:196:D08GAACXX:5:2106:21242:13518
agcacattaaataacagtaataggaatttgttgggcgag
>gnl|SRA|ERR1278117.44785978.1:3-41 HWI-ST574:196:D08GAACXX:5:2105:1184:91700
agcacattaaataacagtaataggaatttgttgggcgag
>gnl|SRA|ERR1278117.44651144.1:59-97 HWI-ST574:196:D08GAACXX:5:2105:3984:74246
agcacattaaataacagtaataggaatttgttgggcgag
>gnl|SRA|ERR1278117.44112363.1:48-86 HWI-ST574:196:D08GAACXX:5:2105:20286:3478
agcacattaaataacagtaataggaatttgttgggcgag
>_R_gnl|SRA|ERR1278117.44033251.2:4-42 HWI-
ST574:196:D08GAACXX:5:2104:11150:91936
agcacattaaataacagtaataggaatttgttgggcgag
>_R_gnl|SRA|ERR1278117.44008515.2:3-41 HWI-ST574:196:D08GAACXX:5:2104:9740:88595
agcacattaaataacagtaataggaatttgttgggcgag
>gnl|SRA|ERR1278117.43982838.1:36-74 HWI-ST574:196:D08GAACXX:5:2104:16250:85165
agcacattaaataacagtaataggaatttgttgggcgag
>gnl|SRA|ERR1278117.43745908.1:5-43 HWI-ST574:196:D08GAACXX:5:2104:20861:53709
agcacattaaataacagtaataggaatttgttgggcgag
>gnl|SRA|ERR1278117.43298573.1:60-98 HWI-ST574:196:D08GAACXX:5:2103:13213:92287
agcacattaaataacagtaataggaatttgttgggcgag
>_R_gnl|SRA|ERR1278117.43150954.2:3-41 HWI-
ST574:196:D08GAACXX:5:2103:12634:72277
agcacattaaataacagtaataggaatttgttgggcgag
>gnl|SRA|ERR1278117.43088367.1:2-40 HWI-ST574:196:D08GAACXX:5:2103:12880:63844
agcacattaaataacagtaataggaatttgttgggcgag

>gnl|SRA|ERR1278117.43085771.1:30-68 HWI-ST574:196:D08GAACXX:5:2103:4955:63586
agcacattaaataacagtaataggaatttgttgggcgag
>gnl|SRA|ERR1278117.42956780.1:49-87 HWI-ST574:196:D08GAACXX:5:2103:13720:45842
agcacattaaataacagtaataggaatttgttgggcgag
>gnl|SRA|ERR1278117.42746268.1:47-85 HWI-ST574:196:D08GAACXX:5:2103:15746:16757
agcacattaaataacagtaataggaatttgttgggcgag
>gnl|SRA|ERR1278117.42687779.1:30-68 HWI-ST574:196:D08GAACXX:5:2103:3329:8825
agcacattaaataacagtaataggaatttgttgggcgag
>gnl|SRA|ERR1278117.42683807.1:30-68 HWI-ST574:196:D08GAACXX:5:2103:19545:8098
agcacattaaataacagtaataggaatttgttgggcgag
>gnl|SRA|ERR1278117.42660367.1:48-86 HWI-ST574:196:D08GAACXX:5:2103:20537:4941
agcacattaaataacagtaataggaatttgttgggcgag
>gnl|SRA|ERR1278117.42453745.1:6-44 HWI-ST574:196:D08GAACXX:5:2102:20721:74939
agcacattaaataacagtaataggaatttgttgggcgag
>gnl|SRA|ERR1278117.42427509.1:10-48 HWI-ST574:196:D08GAACXX:5:2102:2869:71288
agcacattaaataacagtaataggaatttgttgggcgag
>gnl|SRA|ERR1278117.42344165.1:5-43 HWI-ST574:196:D08GAACXX:5:2102:5240:59720
agcacattaaataacagtaataggaatttgttgggcgag
>gnl|SRA|ERR1278117.42315527.1:30-68 HWI-ST574:196:D08GAACXX:5:2102:3603:55727
agcacattaaataacagtaataggaatttgttgggcgag
>gnl|SRA|ERR1278117.42223925.1:5-43 HWI-ST574:196:D08GAACXX:5:2102:5401:42515
agcacattaaataacagtaataggaatttgttgggcgag
>_R_gnl|SRA|ERR1278117.41713116.2:3-41 HWI-ST574:196:D08GAACXX:5:2101:8727:68237
agcacattaaataacagtaataggaatttgttgggcgag
>gnl|SRA|ERR1278117.41614253.1:37-75 HWI-ST574:196:D08GAACXX:5:2101:19766:53575
agcacattaaataacagtaataggaatttgttgggcgag
>gnl|SRA|ERR1278117.41585505.1:7-45 HWI-ST574:196:D08GAACXX:5:2101:4452:49709
agcacattaaataacagtaataggaatttgttgggcgag
>gnl|SRA|ERR1278117.41483498.1:36-74 HWI-ST574:196:D08GAACXX:5:2101:1505:34825
agcacattaaataacagtaataggaatttgttgggcgag
>gnl|SRA|ERR1278117.41054514.1:30-68 HWI-ST574:196:D08GAACXX:5:1316:9386:83028
agcacattaaataacagtaataggaatttgttgggcgag
>gnl|SRA|ERR1278117.41016517.1:59-97 HWI-ST574:196:D08GAACXX:5:1316:2917:79797
agcacattaaataacagtaataggaatttgttgggcgag
>_R_gnl|SRA|ERR1278117.40972771.2:3-41 HWI-
ST574:196:D08GAACXX:5:1316:14815:75878
agcacattaaataacagtaataggaatttgttgggcgag
>gnl|SRA|ERR1278117.40799087.1:48-86 HWI-ST574:196:D08GAACXX:5:1316:15533:60274
agcacattaaataacagtaataggaatttgttgggcgag
>gnl|SRA|ERR1278117.40760855.1:7-45 HWI-ST574:196:D08GAACXX:5:1316:19839:56825
agcacattaaataacagtaataggaatttgttgggcgag
>gnl|SRA|ERR1278117.40751708.1:1-39 HWI-ST574:196:D08GAACXX:5:1316:14662:56065
agcacattaaataacagtaataggaatttgttgggcgag
>gnl|SRA|ERR1278117.40750357.1:9-47 HWI-ST574:196:D08GAACXX:5:1316:4579:56137
agcacattaaataacagtaataggaatttgttgggcgag
>gnl|SRA|ERR1278117.40536074.1:5-43 HWI-ST574:196:D08GAACXX:5:1316:9646:36565
agcacattaaataacagtaataggaatttgttgggcgag
>gnl|SRA|ERR1278117.40500634.1:49-87 HWI-ST574:196:D08GAACXX:5:1316:10409:33471
agcacattaaataacagtaataggaatttgttgggcgag
>gnl|SRA|ERR1278117.40132237.1:46-84 HWI-ST574:196:D08GAACXX:5:1315:19997:98040
agcacattaaataacagtaataggaatttgttgggcgag

>_R_gnl|SRA|ERR1278117.39759157.2:3-41 HWI-ST574:196:D08GAACXX:5:1315:20788:63215
agcacattaaataacagtaataggaatttgttgggcgag
>gnl|SRA|ERR1278117.39745183.1:49-87 HWI-ST574:196:D08GAACXX:5:1315:15599:61783
agcacattaaataacagtaataggaatttgttgggcgag
>gnl|SRA|ERR1278117.39680853.1:49-87 HWI-ST574:196:D08GAACXX:5:1315:6177:55921
agcacattaaataacagtaataggaatttgttgggcgag
>gnl|SRA|ERR1278117.39639173.1:7-45 HWI-ST574:196:D08GAACXX:5:1315:9871:51786
agcacattaaataacagtaataggaatttgttgggcgag
>gnl|SRA|ERR1278117.39573882.1:30-68 HWI-ST574:196:D08GAACXX:5:1315:12717:45699
agcacattaaataacagtaataggaatttgttgggcgag
>gnl|SRA|ERR1278117.39364042.1:7-45 HWI-ST574:196:D08GAACXX:5:1315:1399:25751
agcacattaaataacagtaataggaatttgttgggcgag
>_R_gnl|SRA|ERR1278117.39350068.2:3-41 HWI-ST574:196:D08GAACXX:5:1315:16910:24276
agcacattaaataacagtaataggaatttgttgggcgag
>gnl|SRA|ERR1278117.39301575.1:11-49 HWI-ST574:196:D08GAACXX:5:1315:7332:19792
agcacattaaataacagtaataggaatttgttgggcgag
>gnl|SRA|ERR1278117.39277038.1:30-68 HWI-ST574:196:D08GAACXX:5:1315:20671:17348
agcacattaaataacagtaataggaatttgttgggcgag
>gnl|SRA|ERR1278117.39137095.1:55-93 HWI-ST574:196:D08GAACXX:5:1315:11981:4070
agcacattaaataacagtaataggaatttgttgggcgag
>gnl|SRA|ERR1278117.39039766.1:30-68 HWI-ST574:196:D08GAACXX:5:1314:7322:93688
agcacattaaataacagtaataggaatttgttgggcgag
>gnl|SRA|ERR1278117.38386079.1:2-40 HWI-ST574:196:D08GAACXX:5:1314:1734:29739
agcacattaaataacagtaataggaatttgttgggcgag
>gnl|SRA|ERR1278117.38038421.1:30-68 HWI-ST574:196:D08GAACXX:5:1313:7978:93723
agcacattaaataacagtaataggaatttgttgggcgag
>gnl|SRA|ERR1278117.37834420.1:55-93 HWI-ST574:196:D08GAACXX:5:1313:13906:72883
agcacattaaataacagtaataggaatttgttgggcgag
>gnl|SRA|ERR1278117.37827119.1:20-58 HWI-ST574:196:D08GAACXX:5:1313:14906:72099
agcacattaaataacagtaataggaatttgttgggcgag
>gnl|SRA|ERR1278117.37619204.1:51-89 HWI-ST574:196:D08GAACXX:5:1313:3615:51040
agcacattaaataacagtaataggaatttgttgggcgag
>gnl|SRA|ERR1278117.37459300.1:7-45 HWI-ST574:196:D08GAACXX:5:1313:18400:34688
agcacattaaataacagtaataggaatttgttgggcgag
>gnl|SRA|ERR1278117.37433765.1:7-45 HWI-ST574:196:D08GAACXX:5:1313:9171:32194
agcacattaaataacagtaataggaatttgttgggcgag
>gnl|SRA|ERR1278117.37341841.1:2-40 HWI-ST574:196:D08GAACXX:5:1313:16534:22704
agcacattaaataacagtaataggaatttgttgggcgag
>gnl|SRA|ERR1278117.37161259.1:5-43 HWI-ST574:196:D08GAACXX:5:1313:7356:4087
agcacattaaataacagtaataggaatttgttgggcgag
>gnl|SRA|ERR1278117.37110450.1:47-85 HWI-ST574:196:D08GAACXX:5:1312:17973:97693
agcacattaaataacagtaataggaatttgttgggcgag
>gnl|SRA|ERR1278117.37087770.1:14-52 HWI-ST574:196:D08GAACXX:5:1312:11186:95475
agcacattaaataacagtaataggaatttgttgggcgag
>gnl|SRA|ERR1278117.36975011.1:7-45 HWI-ST574:196:D08GAACXX:5:1312:11676:83526
agcacattaaataacagtaataggaatttgttgggcgag
>gnl|SRA|ERR1278117.36962519.1:37-75 HWI-ST574:196:D08GAACXX:5:1312:7616:82431
agcacattaaataacagtaataggaatttgttgggcgag

>_R_gnl|SRA|ERR1278117.36961020.2:1-39 HWI-
ST574:196:D08GAACXX:5:1312:15113:82245
agcacattaaataacagtaataggaatttgttgggcgag
>gnl|SRA|ERR1278117.36938859.1:36-74 HWI-ST574:196:D08GAACXX:5:1312:10941:79760
agcacattaaataacagtaataggaatttgttgggcgag
>gnl|SRA|ERR1278117.36829011.1:50-88 HWI-ST574:196:D08GAACXX:5:1312:18348:68254
agcacattaaataacagtaataggaatttgttgggcgag
>gnl|SRA|ERR1278117.36815190.1:36-74 HWI-ST574:196:D08GAACXX:5:1312:2645:67156
agcacattaaataacagtaataggaatttgttgggcgag
>gnl|SRA|ERR1278117.36658924.1:28-66 HWI-ST574:196:D08GAACXX:5:1312:21156:50708
agcacattaaataacagtaataggaatttgttgggcgag
>gnl|SRA|ERR1278117.36645105.1:5-43 HWI-ST574:196:D08GAACXX:5:1312:4891:49423
agcacattaaataacagtaataggaatttgttgggcgag
>gnl|SRA|ERR1278117.36631560.1:49-87 HWI-ST574:196:D08GAACXX:5:1312:12640:47824
agcacattaaataacagtaataggaatttgttgggcgag
>gnl|SRA|ERR1278117.36596833.1:6-44 HWI-ST574:196:D08GAACXX:5:1312:19266:44232
agcacattaaataacagtaataggaatttgttgggcgag
>gnl|SRA|ERR1278117.36562123.1:48-86 HWI-ST574:196:D08GAACXX:5:1312:7597:40736
agcacattaaataacagtaataggaatttgttgggcgag
>gnl|SRA|ERR1278117.36519795.1:48-86 HWI-ST574:196:D08GAACXX:5:1312:10324:36112
agcacattaaataacagtaataggaatttgttgggcgag
>gnl|SRA|ERR1278117.36445348.1:9-47 HWI-ST574:196:D08GAACXX:5:1312:5090:28466
agcacattaaataacagtaataggaatttgttgggcgag
>gnl|SRA|ERR1278117.36270746.1:32-70 HWI-ST574:196:D08GAACXX:5:1312:10688:9850
agcacattaaataacagtaataggaatttgttgggcgag
>gnl|SRA|ERR1278117.36201983.1:49-87 HWI-ST574:196:D08GAACXX:5:1312:10636:2619
agcacattaaataacagtaataggaatttgttgggcgag
>gnl|SRA|ERR1278117.35812303.1:13-51 HWI-ST574:196:D08GAACXX:5:1311:14323:59924
agcacattaaataacagtaataggaatttgttgggcgag
>_R_gnl|SRA|ERR1278117.35582207.2:3-41 HWI-
ST574:196:D08GAACXX:5:1311:12545:35186
agcacattaaataacagtaataggaatttgttgggcgag
>gnl|SRA|ERR1278117.35504663.1:62-100 HWI-ST574:196:D08GAACXX:5:1311:5275:26914
agcacattaaataacagtaataggaatttgttgggcgag
>_R_gnl|SRA|ERR1278117.35441755.2:4-42 HWI-
ST574:196:D08GAACXX:5:1311:21137:19989
agcacattaaataacagtaataggaatttgttgggcgag
>gnl|SRA|ERR1278117.35419619.1:47-85 HWI-ST574:196:D08GAACXX:5:1311:7628:17520
agcacattaaataacagtaataggaatttgttgggcgag
>gnl|SRA|ERR1278117.35043452.1:31-69 HWI-ST574:196:D08GAACXX:5:1310:6315:75700
agcacattaaataacagtaataggaatttgttgggcgag
>gnl|SRA|ERR1278117.35035834.1:59-97 HWI-ST574:196:D08GAACXX:5:1310:20238:74699
agcacattaaataacagtaataggaatttgttgggcgag
>gnl|SRA|ERR1278117.34840145.1:59-97 HWI-ST574:196:D08GAACXX:5:1310:2866:53463
agcacattaaataacagtaataggaatttgttgggcgag
>gnl|SRA|ERR1278117.34780060.1:62-100 HWI-ST574:196:D08GAACXX:5:1310:16690:46675
agcacattaaataacagtaataggaatttgttgggcgag
>gnl|SRA|ERR1278117.34766448.1:14-52 HWI-ST574:196:D08GAACXX:5:1310:16412:45206
agcacattaaataacagtaataggaatttgttgggcgag
>gnl|SRA|ERR1278117.34630282.1:7-45 HWI-ST574:196:D08GAACXX:5:1310:6615:30002
agcacattaaataacagtaataggaatttgttgggcgag

>gnl|SRA|ERR1278117.34395399.1:49-87 HWI-ST574:196:D08GAACXX:5:1310:13652:3892
agcacattaaataacagtaataggaatttgttgggcgag
>gnl|SRA|ERR1278117.34371891.1:39-77 HWI-ST574:196:D08GAACXX:5:1309:12358:100171
agcacattaaataacagtaataggaatttgttgggcgag
>gnl|SRA|ERR1278117.34301691.1:2-40 HWI-ST574:196:D08GAACXX:5:1309:5882:92420
agcacattaaataacagtaataggaatttgttgggcgag
>gnl|SRA|ERR1278117.34007640.1:2-40 HWI-ST574:196:D08GAACXX:5:1309:17213:59029
agcacattaaataacagtaataggaatttgttgggcgag
>gnl|SRA|ERR1278117.33619726.1:48-86 HWI-ST574:196:D08GAACXX:5:1309:1412:15167
agcacattaaataacagtaataggaatttgttgggcgag
>gnl|SRA|ERR1278117.33557511.1:11-49 HWI-ST574:196:D08GAACXX:5:1309:14703:7921
agcacattaaataacagtaataggaatttgttgggcgag
>gnl|SRA|ERR1278117.33477200.1:49-87 HWI-ST574:196:D08GAACXX:5:1308:6311:97599
agcacattaaataacagtaataggaatttgttgggcgag
>gnl|SRA|ERR1278117.33307349.1:30-68 HWI-ST574:196:D08GAACXX:5:1308:20544:77761
agcacattaaataacagtaataggaatttgttgggcgag
>gnl|SRA|ERR1278117.33163771.1:7-45 HWI-ST574:196:D08GAACXX:5:1308:7577:61381
agcacattaaataacagtaataggaatttgttgggcgag
>_R_gnl|SRA|ERR1278117.33155911.2:3-41 HWI-
ST574:196:D08GAACXX:5:1308:14348:60482
agcacattaaataacagtaataggaatttgttgggcgag
>gnl|SRA|ERR1278117.32971775.1:62-100 HWI-ST574:196:D08GAACXX:5:1308:16627:38972
agcacattaaataacagtaataggaatttgttgggcgag
>gnl|SRA|ERR1278117.32939987.1:33-71 HWI-ST574:196:D08GAACXX:5:1308:19591:35126
agcacattaaataacagtaataggaatttgttgggcgag
>gnl|SRA|ERR1278117.32728768.1:39-77 HWI-ST574:196:D08GAACXX:5:1308:4244:10627
agcacattaaataacagtaataggaatttgttgggcgag
>gnl|SRA|ERR1278117.32660887.1:59-97 HWI-ST574:196:D08GAACXX:5:1308:9028:2647
agcacattaaataacagtaataggaatttgttgggcgag
>gnl|SRA|ERR1278117.32622718.1:49-87 HWI-ST574:196:D08GAACXX:5:1307:16689:96842
agcacattaaataacagtaataggaatttgttgggcgag
>gnl|SRA|ERR1278117.32503933.1:5-43 HWI-ST574:196:D08GAACXX:5:1307:10619:82838
agcacattaaataacagtaataggaatttgttgggcgag
>gnl|SRA|ERR1278117.32248561.1:5-43 HWI-ST574:196:D08GAACXX:5:1307:16308:52301
agcacattaaataacagtaataggaatttgttgggcgag
>gnl|SRA|ERR1278117.31915193.1:14-52 HWI-ST574:196:D08GAACXX:5:1307:7835:12251
agcacattaaataacagtaataggaatttgttgggcgag
>gnl|SRA|ERR1278117.31612993.1:8-46 HWI-ST574:196:D08GAACXX:5:1306:10525:74563
agcacattaaataacagtaataggaatttgttgggcgag
>gnl|SRA|ERR1278117.31456133.1:46-84 HWI-ST574:196:D08GAACXX:5:1306:15009:55490
agcacattaaataacagtaataggaatttgttgggcgag
>gnl|SRA|ERR1278117.31397841.1:5-43 HWI-ST574:196:D08GAACXX:5:1306:4243:48312
agcacattaaataacagtaataggaatttgttgggcgag
>gnl|SRA|ERR1278117.31366462.1:39-77 HWI-ST574:196:D08GAACXX:5:1306:15617:44464
agcacattaaataacagtaataggaatttgttgggcgag
>gnl|SRA|ERR1278117.31358973.1:30-68 HWI-ST574:196:D08GAACXX:5:1306:21400:43288
agcacattaaataacagtaataggaatttgttgggcgag
>gnl|SRA|ERR1278117.31356690.1:11-49 HWI-ST574:196:D08GAACXX:5:1306:18533:43057
agcacattaaataacagtaataggaatttgttgggcgag
>gnl|SRA|ERR1278117.31340177.1:2-40 HWI-ST574:196:D08GAACXX:5:1306:14904:41085
agcacattaaataacagtaataggaatttgttgggcgag

>gnl|SRA|ERR1278117.31096340.1:47-85 HWI-ST574:196:D08GAACXX:5:1306:1197:11167
agcacattaaataacagtaataggaatttgttgggcgag
>_R_gnl|SRA|ERR1278117.30969271.2:1-39 HWI-
ST574:196:D08GAACXX:5:1305:10685:94239
agcacattaaataacagtaataggaatttgttgggcgag
>gnl|SRA|ERR1278117.30862194.1:30-68 HWI-ST574:196:D08GAACXX:5:1305:10362:80992
agcacattaaataacagtaataggaatttgttgggcgag
>gnl|SRA|ERR1278117.30589256.1:50-88 HWI-ST574:196:D08GAACXX:5:1305:7183:46734
agcacattaaataacagtaataggaatttgttgggcgag
>gnl|SRA|ERR1278117.30573468.1:10-48 HWI-ST574:196:D08GAACXX:5:1305:7834:44708
agcacattaaataacagtaataggaatttgttgggcgag
>gnl|SRA|ERR1278117.30527088.1:7-45 HWI-ST574:196:D08GAACXX:5:1305:20680:38519
agcacattaaataacagtaataggaatttgttgggcgag
>gnl|SRA|ERR1278117.30437752.1:2-40 HWI-ST574:196:D08GAACXX:5:1305:19925:27477
agcacattaaataacagtaataggaatttgttgggcgag
>gnl|SRA|ERR1278117.30428625.1:49-87 HWI-ST574:196:D08GAACXX:5:1305:7351:26392
agcacattaaataacagtaataggaatttgttgggcgag
>gnl|SRA|ERR1278117.30192310.1:10-48 HWI-ST574:196:D08GAACXX:5:1304:19239:95039
agcacattaaataacagtaataggaatttgttgggcgag
>gnl|SRA|ERR1278117.29808541.1:30-68 HWI-ST574:196:D08GAACXX:5:1304:9987:45880
agcacattaaataacagtaataggaatttgttgggcgag
>gnl|SRA|ERR1278117.29493858.1:37-75 HWI-ST574:196:D08GAACXX:5:1304:14999:4777
agcacattaaataacagtaataggaatttgttgggcgag
>gnl|SRA|ERR1278117.29408636.1:47-85 HWI-ST574:196:D08GAACXX:5:1303:12143:92505
agcacattaaataacagtaataggaatttgttgggcgag
>_R_gnl|SRA|ERR1278117.29304795.2:3-41 HWI-ST574:196:D08GAACXX:5:1303:1610:79185
agcacattaaataacagtaataggaatttgttgggcgag
>gnl|SRA|ERR1278117.29200089.1:28-66 HWI-ST574:196:D08GAACXX:5:1303:21402:65137
agcacattaaataacagtaataggaatttgttgggcgag
>gnl|SRA|ERR1278117.29195875.1:44-82 HWI-ST574:196:D08GAACXX:5:1303:16653:64629
agcacattaaataacagtaataggaatttgttgggcgag
>gnl|SRA|ERR1278117.29165966.1:31-69 HWI-ST574:196:D08GAACXX:5:1303:1351:60992
agcacattaaataacagtaataggaatttgttgggcgag
>gnl|SRA|ERR1278117.28983222.1:6-44 HWI-ST574:196:D08GAACXX:5:1303:4624:36733
agcacattaaataacagtaataggaatttgttgggcgag
>_R_gnl|SRA|ERR1278117.28919198.2:1-39 HWI-ST574:196:D08GAACXX:5:1303:6936:28044
agcacattaaataacagtaataggaatttgttgggcgag
>gnl|SRA|ERR1278117.28906023.1:62-100 HWI-ST574:196:D08GAACXX:5:1303:8267:26305
agcacattaaataacagtaataggaatttgttgggcgag
>gnl|SRA|ERR1278117.28805056.1:30-68 HWI-ST574:196:D08GAACXX:5:1303:6502:12963
agcacattaaataacagtaataggaatttgttgggcgag
>gnl|SRA|ERR1278117.28792334.1:7-45 HWI-ST574:196:D08GAACXX:5:1303:12575:11181
agcacattaaataacagtaataggaatttgttgggcgag
>_R_gnl|SRA|ERR1278117.28344640.2:1-39 HWI-
ST574:196:D08GAACXX:5:1302:15288:49187
agcacattaaataacagtaataggaatttgttgggcgag
>gnl|SRA|ERR1278117.28275022.1:29-67 HWI-ST574:196:D08GAACXX:5:1302:5555:39601
agcacattaaataacagtaataggaatttgttgggcgag
>gnl|SRA|ERR1278117.28050338.1:47-85 HWI-ST574:196:D08GAACXX:5:1302:2053:8306
agcacattaaataacagtaataggaatttgttgggcgag
>gnl|SRA|ERR1278117.28027117.1:58-96 HWI-ST574:196:D08GAACXX:5:1302:4048:5073

agcacattaaataacagtaataggaatttgttgggcgag
>gnl|SRA|ERR1278117.27967718.1:9-47 HWI-ST574:196:D08GAACXX:5:1301:7453:95635
agcacattaaataacagtaataggaatttgttgggcgag
>gnl|SRA|ERR1278117.27651558.1:6-44 HWI-ST574:196:D08GAACXX:5:1301:10664:51600
agcacattaaataacagtaataggaatttgttgggcgag
>gnl|SRA|ERR1278117.27440622.1:62-100 HWI-ST574:196:D08GAACXX:5:1301:10083:22095
agcacattaaataacagtaataggaatttgttgggcgag
>_R_gnl|SRA|ERR1278117.27435322.2:3-41 HWI-ST574:196:D08GAACXX:5:1301:11121:21321
agcacattaaataacagtaataggaatttgttgggcgag
>gnl|SRA|ERR1278117.27358590.1:46-84 HWI-ST574:196:D08GAACXX:5:1301:9347:10684
agcacattaaataacagtaataggaatttgttgggcgag
>gnl|SRA|ERR1278117.27322000.1:11-49 HWI-ST574:196:D08GAACXX:5:1301:19212:5413
agcacattaaataacagtaataggaatttgttgggcgag
>gnl|SRA|ERR1278117.27094568.1:59-97 HWI-ST574:196:D08GAACXX:5:1216:6508:83331
agcacattaaataacagtaataggaatttgttgggcgag
>gnl|SRA|ERR1278117.27081384.1:30-68 HWI-ST574:196:D08GAACXX:5:1216:13171:82092
agcacattaaataacagtaataggaatttgttgggcgag
>gnl|SRA|ERR1278117.26939695.1:7-45 HWI-ST574:196:D08GAACXX:5:1216:12611:69680
agcacattaaataacagtaataggaatttgttgggcgag
>gnl|SRA|ERR1278117.26475643.1:34-72 HWI-ST574:196:D08GAACXX:5:1216:13149:27293
agcacattaaataacagtaataggaatttgttgggcgag
>gnl|SRA|ERR1278117.26292575.1:49-87 HWI-ST574:196:D08GAACXX:5:1216:11332:10456
agcacattaaataacagtaataggaatttgttgggcgag
>gnl|SRA|ERR1278117.26188716.1:30-68 HWI-ST574:196:D08GAACXX:5:1215:6262:99705
agcacattaaataacagtaataggaatttgttgggcgag
>gnl|SRA|ERR1278117.26062302.1:6-44 HWI-ST574:196:D08GAACXX:5:1215:14106:87528
agcacattaaataacagtaataggaatttgttgggcgag
>gnl|SRA|ERR1278117.25740354.1:1-39 HWI-ST574:196:D08GAACXX:5:1215:20405:56768
agcacattaaataacagtaataggaatttgttgggcgag
>gnl|SRA|ERR1278117.25493556.1:36-74 HWI-ST574:196:D08GAACXX:5:1215:15453:33208
agcacattaaataacagtaataggaatttgttgggcgag
>gnl|SRA|ERR1278117.25448799.1:60-98 HWI-ST574:196:D08GAACXX:5:1215:7410:28876
agcacattaaataacagtaataggaatttgttgggcgag
>gnl|SRA|ERR1278117.25411263.1:11-49 HWI-ST574:196:D08GAACXX:5:1215:16722:25072
agcacattaaataacagtaataggaatttgttgggcgag
>_R_gnl|SRA|ERR1278117.25218800.2:4-42 HWI-ST574:196:D08GAACXX:5:1215:17694:6495
agcacattaaataacagtaataggaatttgttgggcgag
>_R_gnl|SRA|ERR1278117.24879754.2:3-41 HWI-ST574:196:D08GAACXX:5:1214:14175:72034
agcacattaaataacagtaataggaatttgttgggcgag
>gnl|SRA|ERR1278117.24719735.1:30-68 HWI-ST574:196:D08GAACXX:5:1214:4375:56427
agcacattaaataacagtaataggaatttgttgggcgag
>gnl|SRA|ERR1278117.24431541.1:61-99 HWI-ST574:196:D08GAACXX:5:1214:13978:27465
agcacattaaataacagtaataggaatttgttgggcgag
>gnl|SRA|ERR1278117.24288829.1:61-99 HWI-ST574:196:D08GAACXX:5:1214:6409:13106
agcacattaaataacagtaataggaatttgttgggcgag
>gnl|SRA|ERR1278117.24061399.1:12-50 HWI-ST574:196:D08GAACXX:5:1213:3865:88947
agcacattaaataacagtaataggaatttgttgggcgag
>gnl|SRA|ERR1278117.23419294.1:55-93 HWI-ST574:196:D08GAACXX:5:1213:17731:22306
agcacattaaataacagtaataggaatttgttgggcgag

>gnl|SRA|ERR1278117.23156920.1:21-59 HWI-ST574:196:D08GAACXX:5:1212:9351:93892
agcacattaaataacagtaataggaatttgttgggcgag
>_R_gnl|SRA|ERR1278117.23024045.2:3-41 HWI-
ST574:196:D08GAACXX:5:1212:11278:79998
agcacattaaataacagtaataggaatttgttgggcgag
>gnl|SRA|ERR1278117.22987877.1:10-48 HWI-ST574:196:D08GAACXX:5:1212:4658:76222
agcacattaaataacagtaataggaatttgttgggcgag
>gnl|SRA|ERR1278117.22718281.1:4-42 HWI-ST574:196:D08GAACXX:5:1212:4070:47571
agcacattaaataacagtaataggaatttgttgggcgag
>_R_gnl|SRA|ERR1278117.22545860.2:3-41 HWI-
ST574:196:D08GAACXX:5:1212:20529:29174
agcacattaaataacagtaataggaatttgttgggcgag
>gnl|SRA|ERR1278117.22227671.1:36-74 HWI-ST574:196:D08GAACXX:5:1211:6874:94028
agcacattaaataacagtaataggaatttgttgggcgag
>gnl|SRA|ERR1278117.22174904.1:2-40 HWI-ST574:196:D08GAACXX:5:1211:10543:88462
agcacattaaataacagtaataggaatttgttgggcgag
>gnl|SRA|ERR1278117.22036599.1:61-99 HWI-ST574:196:D08GAACXX:5:1211:10526:73288
agcacattaaataacagtaataggaatttgttgggcgag
>gnl|SRA|ERR1278117.21927514.1:8-46 HWI-ST574:196:D08GAACXX:5:1211:2667:61671
agcacattaaataacagtaataggaatttgttgggcgag
>gnl|SRA|ERR1278117.21617352.1:30-68 HWI-ST574:196:D08GAACXX:5:1211:18876:27527
agcacattaaataacagtaataggaatttgttgggcgag
>gnl|SRA|ERR1278117.21416246.1:36-74 HWI-ST574:196:D08GAACXX:5:1211:8062:5590
agcacattaaataacagtaataggaatttgttgggcgag
>gnl|SRA|ERR1278117.21374091.1:7-45 HWI-ST574:196:D08GAACXX:5:1210:1880:99973
agcacattaaataacagtaataggaatttgttgggcgag
>gnl|SRA|ERR1278117.21170355.1:29-67 HWI-ST574:196:D08GAACXX:5:1210:17942:77224
agcacattaaataacagtaataggaatttgttgggcgag
>gnl|SRA|ERR1278117.21041639.1:5-43 HWI-ST574:196:D08GAACXX:5:1210:12802:62855
agcacattaaataacagtaataggaatttgttgggcgag
>gnl|SRA|ERR1278117.20783994.1:38-76 HWI-ST574:196:D08GAACXX:5:1210:8818:34093
agcacattaaataacagtaataggaatttgttgggcgag
>gnl|SRA|ERR1278117.20609415.1:30-68 HWI-ST574:196:D08GAACXX:5:1210:14072:14257
agcacattaaataacagtaataggaatttgttgggcgag
>gnl|SRA|ERR1278117.20591030.1:48-86 HWI-ST574:196:D08GAACXX:5:1210:8516:12304
agcacattaaataacagtaataggaatttgttgggcgag
>gnl|SRA|ERR1278117.20419655.1:5-43 HWI-ST574:196:D08GAACXX:5:1209:6028:91792
agcacattaaataacagtaataggaatttgttgggcgag
>gnl|SRA|ERR1278117.20353159.1:20-58 HWI-ST574:196:D08GAACXX:5:1209:1365:84356
agcacattaaataacagtaataggaatttgttgggcgag
>_R_gnl|SRA|ERR1278117.20173688.2:2-40 HWI-ST574:196:D08GAACXX:5:1209:5910:63973
agcacattaaataacagtaataggaatttgttgggcgag
>_R_gnl|SRA|ERR1278117.20100475.2:4-42 HWI-
ST574:196:D08GAACXX:5:1209:15405:55454
agcacattaaataacagtaataggaatttgttgggcgag
>gnl|SRA|ERR1278117.19971519.1:1-39 HWI-ST574:196:D08GAACXX:5:1209:15554:40511
agcacattaaataacagtaataggaatttgttgggcgag
>gnl|SRA|ERR1278117.19945622.1:49-87 HWI-ST574:196:D08GAACXX:5:1209:16086:37746
agcacattaaataacagtaataggaatttgttgggcgag
>_R_gnl|SRA|ERR1278117.19834169.2:3-41 HWI-
ST574:196:D08GAACXX:5:1209:13271:24983

agcacattaaataacagtaataggaatttgttgggcgag
>gnl|SRA|ERR1278117.19501894.1:39-77 HWI-ST574:196:D08GAACXX:5:1208:10250:85398
agcacattaaataacagtaataggaatttgttgggcgag
>gnl|SRA|ERR1278117.19215661.1:7-45 HWI-ST574:196:D08GAACXX:5:1208:16624:51805
agcacattaaataacagtaataggaatttgttgggcgag
>gnl|SRA|ERR1278117.19054414.1:12-50 HWI-ST574:196:D08GAACXX:5:1208:1173:33009
agcacattaaataacagtaataggaatttgttgggcgag
>gnl|SRA|ERR1278117.19044248.1:6-44 HWI-ST574:196:D08GAACXX:5:1208:4150:31813
agcacattaaataacagtaataggaatttgttgggcgag
>gnl|SRA|ERR1278117.18924060.1:49-87 HWI-ST574:196:D08GAACXX:5:1208:8616:17654
agcacattaaataacagtaataggaatttgttgggcgag
>gnl|SRA|ERR1278117.18372758.1:31-69 HWI-ST574:196:D08GAACXX:5:1207:7974:50651
agcacattaaataacagtaataggaatttgttgggcgag
>gnl|SRA|ERR1278117.18347044.1:48-86 HWI-ST574:196:D08GAACXX:5:1207:19100:47420
agcacattaaataacagtaataggaatttgttgggcgag
>gnl|SRA|ERR1278117.18275386.1:12-50 HWI-ST574:196:D08GAACXX:5:1207:7576:38843
agcacattaaataacagtaataggaatttgttgggcgag
>gnl|SRA|ERR1278117.18234736.1:4-42 HWI-ST574:196:D08GAACXX:5:1207:17451:33804
agcacattaaataacagtaataggaatttgttgggcgag
>gnl|SRA|ERR1278117.18108626.1:44-82 HWI-ST574:196:D08GAACXX:5:1207:2946:18774
agcacattaaataacagtaataggaatttgttgggcgag
>gnl|SRA|ERR1278117.18025550.1:9-47 HWI-ST574:196:D08GAACXX:5:1207:2923:8757
agcacattaaataacagtaataggaatttgttgggcgag
>gnl|SRA|ERR1278117.17610394.1:5-43 HWI-ST574:196:D08GAACXX:5:1206:1667:56820
agcacattaaataacagtaataggaatttgttgggcgag
>gnl|SRA|ERR1278117.17428725.1:13-51 HWI-ST574:196:D08GAACXX:5:1206:9524:34301
agcacattaaataacagtaataggaatttgttgggcgag
>gnl|SRA|ERR1278117.17255477.1:10-48 HWI-ST574:196:D08GAACXX:5:1206:11075:12785
agcacattaaataacagtaataggaatttgttgggcgag
>_R_gnl|SRA|ERR1278117.17197070.2:3-41 HWI-ST574:196:D08GAACXX:5:1206:9242:5685
agcacattaaataacagtaataggaatttgttgggcgag
>gnl|SRA|ERR1278117.16857501.1:10-48 HWI-ST574:196:D08GAACXX:5:1205:19470:61944
agcacattaaataacagtaataggaatttgttgggcgag
>gnl|SRA|ERR1278117.16551114.1:54-92 HWI-ST574:196:D08GAACXX:5:1205:15068:23188
agcacattaaataacagtaataggaatttgttgggcgag
>gnl|SRA|ERR1278117.16517898.1:2-40 HWI-ST574:196:D08GAACXX:5:1205:18756:18801
agcacattaaataacagtaataggaatttgttgggcgag
>gnl|SRA|ERR1278117.16280106.1:46-84 HWI-ST574:196:D08GAACXX:5:1204:13036:87383
agcacattaaataacagtaataggaatttgttgggcgag
>gnl|SRA|ERR1278117.16219983.1:48-86 HWI-ST574:196:D08GAACXX:5:1204:13716:79671
agcacattaaataacagtaataggaatttgttgggcgag
>gnl|SRA|ERR1278117.16112593.1:27-65 HWI-ST574:196:D08GAACXX:5:1204:8506:65884
agcacattaaataacagtaataggaatttgttgggcgag
>_R_gnl|SRA|ERR1278117.15976400.2:1-39 HWI-
ST574:196:D08GAACXX:5:1204:20191:48107
agcacattaaataacagtaataggaatttgttgggcgag
>gnl|SRA|ERR1278117.15829138.1:61-99 HWI-ST574:196:D08GAACXX:5:1204:11861:29117
agcacattaaataacagtaataggaatttgttgggcgag
>gnl|SRA|ERR1278117.15665392.1:36-74 HWI-ST574:196:D08GAACXX:5:1204:8843:7839
agcacattaaataacagtaataggaatttgttgggcgag
>gnl|SRA|ERR1278117.15579081.1:30-68 HWI-ST574:196:D08GAACXX:5:1203:7197:95261

agcacattaaataacagtaataggaatttgttgggcgag
>gnl|SRA|ERR1278117.15530686.1:54-92 HWI-ST574:196:D08GAACXX:5:1203:15272:88800
agcacattaaataacagtaataggaatttgttgggcgag
>gnl|SRA|ERR1278117.15341805.1:51-89 HWI-ST574:196:D08GAACXX:5:1203:18331:63830
agcacattaaataacagtaataggaatttgttgggcgag
>gnl|SRA|ERR1278117.15285370.1:3-41 HWI-ST574:196:D08GAACXX:5:1203:3333:56711
agcacattaaataacagtaataggaatttgttgggcgag
>gnl|SRA|ERR1278117.15277517.1:4-42 HWI-ST574:196:D08GAACXX:5:1203:20911:55450
agcacattaaataacagtaataggaatttgttgggcgag
>gnl|SRA|ERR1278117.14924837.1:9-47 HWI-ST574:196:D08GAACXX:5:1203:18057:8395
agcacattaaataacagtaataggaatttgttgggcgag
>gnl|SRA|ERR1278117.14835896.1:37-75 HWI-ST574:196:D08GAACXX:5:1202:12227:95375
agcacattaaataacagtaataggaatttgttgggcgag
>gnl|SRA|ERR1278117.14630400.1:42-80 HWI-ST574:196:D08GAACXX:5:1202:13410:67704
agcacattaaataacagtaataggaatttgttgggcgag
>gnl|SRA|ERR1278117.14490447.1:31-69 HWI-ST574:196:D08GAACXX:5:1202:2968:48674
agcacattaaataacagtaataggaatttgttgggcgag
>gnl|SRA|ERR1278117.14189200.1:21-59 HWI-ST574:196:D08GAACXX:5:1202:8495:6967
agcacattaaataacagtaataggaatttgttgggcgag
>gnl|SRA|ERR1278117.14092716.1:31-69 HWI-ST574:196:D08GAACXX:5:1201:10217:92295
agcacattaaataacagtaataggaatttgttgggcgag
>gnl|SRA|ERR1278117.13860749.1:36-74 HWI-ST574:196:D08GAACXX:5:1201:14941:60233
agcacattaaataacagtaataggaatttgttgggcgag
>gnl|SRA|ERR1278117.13699671.1:36-74 HWI-ST574:196:D08GAACXX:5:1201:5864:37753
agcacattaaataacagtaataggaatttgttgggcgag
>gnl|SRA|ERR1278117.13645806.1:30-68 HWI-ST574:196:D08GAACXX:5:1201:6690:30327
agcacattaaataacagtaataggaatttgttgggcgag
>gnl|SRA|ERR1278117.13534922.1:59-97 HWI-ST574:196:D08GAACXX:5:1201:6665:14899
agcacattaaataacagtaataggaatttgttgggcgag
>gnl|SRA|ERR1278117.13480380.1:60-98 HWI-ST574:196:D08GAACXX:5:1201:16646:7081
agcacattaaataacagtaataggaatttgttgggcgag
>gnl|SRA|ERR1278117.13279779.1:36-74 HWI-ST574:196:D08GAACXX:5:1116:9533:86134
agcacattaaataacagtaataggaatttgttgggcgag
>gnl|SRA|ERR1278117.13276080.1:7-45 HWI-ST574:196:D08GAACXX:5:1116:3108:85799
agcacattaaataacagtaataggaatttgttgggcgag
>gnl|SRA|ERR1278117.13248545.1:44-82 HWI-ST574:196:D08GAACXX:5:1116:20976:83066
agcacattaaataacagtaataggaatttgttgggcgag
>gnl|SRA|ERR1278117.13138773.1:2-40 HWI-ST574:196:D08GAACXX:5:1116:11730:73225
agcacattaaataacagtaataggaatttgttgggcgag
>gnl|SRA|ERR1278117.13105210.1:2-40 HWI-ST574:196:D08GAACXX:5:1116:6806:70220
agcacattaaataacagtaataggaatttgttgggcgag
>gnl|SRA|ERR1278117.12998366.1:62-100 HWI-ST574:196:D08GAACXX:5:1116:8940:60114
agcacattaaataacagtaataggaatttgttgggcgag
>gnl|SRA|ERR1278117.12966774.1:27-65 HWI-ST574:196:D08GAACXX:5:1116:11996:57184
agcacattaaataacagtaataggaatttgttgggcgag
>gnl|SRA|ERR1278117.12755453.1:20-58 HWI-ST574:196:D08GAACXX:5:1116:8289:37100
agcacattaaataacagtaataggaatttgttgggcgag
>gnl|SRA|ERR1278117.12334103.1:59-97 HWI-ST574:196:D08GAACXX:5:1115:6158:95426
agcacattaaataacagtaataggaatttgttgggcgag
>gnl|SRA|ERR1278117.12236915.1:60-98 HWI-ST574:196:D08GAACXX:5:1115:5953:85818
agcacattaaataacagtaataggaatttgttgggcgag

>gnl|SRA|ERR1278117.12235907.1:47-85 HWI-ST574:196:D08GAACXX:5:1115:18310:85580
agcacattaaataacagtaataggaatttgttgggcgag
>gnl|SRA|ERR1278117.12195052.1:7-45 HWI-ST574:196:D08GAACXX:5:1115:19240:81554
agcacattaaataacagtaataggaatttgttgggcgag
>_R_gnl|SRA|ERR1278117.12170234.2:3-41 HWI-ST574:196:D08GAACXX:5:1115:2717:79325
agcacattaaataacagtaataggaatttgttgggcgag
>gnl|SRA|ERR1278117.11968223.1:21-59 HWI-ST574:196:D08GAACXX:5:1115:7293:59313
agcacattaaataacagtaataggaatttgttgggcgag
>gnl|SRA|ERR1278117.11478053.1:62-100 HWI-ST574:196:D08GAACXX:5:1115:3296:10531
agcacattaaataacagtaataggaatttgttgggcgag
>gnl|SRA|ERR1278117.11339864.1:6-44 HWI-ST574:196:D08GAACXX:5:1114:14762:95283
agcacattaaataacagtaataggaatttgttgggcgag
>gnl|SRA|ERR1278117.11308955.1:47-85 HWI-ST574:196:D08GAACXX:5:1114:5987:92422
agcacattaaataacagtaataggaatttgttgggcgag
>gnl|SRA|ERR1278117.11184748.1:49-87 HWI-ST574:196:D08GAACXX:5:1114:5999:79949
agcacattaaataacagtaataggaatttgttgggcgag
>gnl|SRA|ERR1278117.11075043.1:48-86 HWI-ST574:196:D08GAACXX:5:1114:18376:68554
agcacattaaataacagtaataggaatttgttgggcgag
>gnl|SRA|ERR1278117.11015378.1:61-99 HWI-ST574:196:D08GAACXX:5:1114:12341:62662
agcacattaaataacagtaataggaatttgttgggcgag
>gnl|SRA|ERR1278117.10999592.1:43-81 HWI-ST574:196:D08GAACXX:5:1114:3964:61230
agcacattaaataacagtaataggaatttgttgggcgag
>gnl|SRA|ERR1278117.10938213.1:36-74 HWI-ST574:196:D08GAACXX:5:1114:20221:54557
agcacattaaataacagtaataggaatttgttgggcgag
>gnl|SRA|ERR1278117.10898534.1:51-89 HWI-ST574:196:D08GAACXX:5:1114:14417:50721
agcacattaaataacagtaataggaatttgttgggcgag
>gnl|SRA|ERR1278117.10805808.1:8-46 HWI-ST574:196:D08GAACXX:5:1114:16187:41010
agcacattaaataacagtaataggaatttgttgggcgag
>gnl|SRA|ERR1278117.10554329.1:5-43 HWI-ST574:196:D08GAACXX:5:1114:3790:15281
agcacattaaataacagtaataggaatttgttgggcgag
>gnl|SRA|ERR1278117.10276525.1:3-41 HWI-ST574:196:D08GAACXX:5:1113:20248:85032
agcacattaaataacagtaataggaatttgttgggcgag
>gnl|SRA|ERR1278117.10149810.1:8-46 HWI-ST574:196:D08GAACXX:5:1113:14759:71771
agcacattaaataacagtaataggaatttgttgggcgag
>gnl|SRA|ERR1278117.9999363.1:10-48 HWI-ST574:196:D08GAACXX:5:1113:19528:55895
agcacattaaataacagtaataggaatttgttgggcgag
>gnl|SRA|ERR1278117.9749828.1:2-40 HWI-ST574:196:D08GAACXX:5:1113:5636:29508
agcacattaaataacagtaataggaatttgttgggcgag
>gnl|SRA|ERR1278117.9655902.1:50-88 HWI-ST574:196:D08GAACXX:5:1113:8860:19520
agcacattaaataacagtaataggaatttgttgggcgag
>gnl|SRA|ERR1278117.9616028.1:47-85 HWI-ST574:196:D08GAACXX:5:1113:6638:15488
agcacattaaataacagtaataggaatttgttgggcgag
>gnl|SRA|ERR1278117.9611685.1:42-80 HWI-ST574:196:D08GAACXX:5:1113:9574:14840
agcacattaaataacagtaataggaatttgttgggcgag
>gnl|SRA|ERR1278117.9587277.1:46-84 HWI-ST574:196:D08GAACXX:5:1113:1302:12447
agcacattaaataacagtaataggaatttgttgggcgag
>gnl|SRA|ERR1278117.9203231.1:37-75 HWI-ST574:196:D08GAACXX:5:1112:17675:69597
agcacattaaataacagtaataggaatttgttgggcgag
>gnl|SRA|ERR1278117.9038338.1:11-49 HWI-ST574:196:D08GAACXX:5:1112:10210:51801
agcacattaaataacagtaataggaatttgttgggcgag
>gnl|SRA|ERR1278117.9029901.1:47-85 HWI-ST574:196:D08GAACXX:5:1112:17253:50817

agcacattaaataacagtaataggaatttgttgggcgag
>gnl|SRA|ERR1278117.8874063.1:60-98 HWI-ST574:196:D08GAACXX:5:1112:5728:34076
agcacattaaataacagtaataggaatttgttgggcgag
>_R_gnl|SRA|ERR1278117.8854017.2:7-45 HWI-ST574:196:D08GAACXX:5:1112:11004:31949
agcacattaaataacagtaataggaatttgttgggcgag
>gnl|SRA|ERR1278117.8697682.1:43-81 HWI-ST574:196:D08GAACXX:5:1112:8909:14794
agcacattaaataacagtaataggaatttgttgggcgag
>gnl|SRA|ERR1278117.8631817.1:47-85 HWI-ST574:196:D08GAACXX:5:1112:6799:7682
agcacattaaataacagtaataggaatttgttgggcgag
>_R_gnl|SRA|ERR1278117.8391603.2:3-41 HWI-ST574:196:D08GAACXX:5:1111:11349:79992
agcacattaaataacagtaataggaatttgttgggcgag
>gnl|SRA|ERR1278117.8318948.1:28-66 HWI-ST574:196:D08GAACXX:5:1111:7640:71962
agcacattaaataacagtaataggaatttgttgggcgag
>_R_gnl|SRA|ERR1278117.8077111.2:3-41 HWI-ST574:196:D08GAACXX:5:1111:15806:44924
agcacattaaataacagtaataggaatttgttgggcgag
>gnl|SRA|ERR1278117.8051802.1:8-46 HWI-ST574:196:D08GAACXX:5:1111:8284:42118
agcacattaaataacagtaataggaatttgttgggcgag
>gnl|SRA|ERR1278117.7918195.1:36-74 HWI-ST574:196:D08GAACXX:5:1111:8570:27175
agcacattaaataacagtaataggaatttgttgggcgag
>gnl|SRA|ERR1278117.7764420.1:49-87 HWI-ST574:196:D08GAACXX:5:1111:8040:9844
agcacattaaataacagtaataggaatttgttgggcgag
>gnl|SRA|ERR1278117.7696888.1:59-97 HWI-ST574:196:D08GAACXX:5:1111:2265:2453
agcacattaaataacagtaataggaatttgttgggcgag
>gnl|SRA|ERR1278117.7660656.1:7-45 HWI-ST574:196:D08GAACXX:5:1110:20885:96808
agcacattaaataacagtaataggaatttgttgggcgag
>gnl|SRA|ERR1278117.7457324.1:49-87 HWI-ST574:196:D08GAACXX:5:1110:2512:74138
agcacattaaataacagtaataggaatttgttgggcgag
>gnl|SRA|ERR1278117.7207909.1:6-44 HWI-ST574:196:D08GAACXX:5:1110:15908:45319
agcacattaaataacagtaataggaatttgttgggcgag
>_R_gnl|SRA|ERR1278117.7129880.2:3-41 HWI-ST574:196:D08GAACXX:5:1110:16007:36371
agcacattaaataacagtaataggaatttgttgggcgag
>gnl|SRA|ERR1278117.7081953.1:4-42 HWI-ST574:196:D08GAACXX:5:1110:14754:30886
agcacattaaataacagtaataggaatttgttgggcgag
>_R_gnl|SRA|ERR1278117.6928468.2:3-41 HWI-ST574:196:D08GAACXX:5:1110:6307:13237
agcacattaaataacagtaataggaatttgttgggcgag
>_R_gnl|SRA|ERR1278117.6709870.2:3-41 HWI-ST574:196:D08GAACXX:5:1109:16651:86361
agcacattaaataacagtaataggaatttgttgggcgag
>gnl|SRA|ERR1278117.6567483.1:31-69 HWI-ST574:196:D08GAACXX:5:1109:8479:69761
agcacattaaataacagtaataggaatttgttgggcgag
>gnl|SRA|ERR1278117.6498781.1:49-87 HWI-ST574:196:D08GAACXX:5:1109:2537:61852
agcacattaaataacagtaataggaatttgttgggcgag
>gnl|SRA|ERR1278117.6428629.1:36-74 HWI-ST574:196:D08GAACXX:5:1109:20653:53495
agcacattaaataacagtaataggaatttgttgggcgag
>gnl|SRA|ERR1278117.6174323.1:12-50 HWI-ST574:196:D08GAACXX:5:1109:15395:23366
agcacattaaataacagtaataggaatttgttgggcgag
>_R_gnl|SRA|ERR1278117.5949601.2:3-41 HWI-ST574:196:D08GAACXX:5:1108:9550:95569
agcacattaaataacagtaataggaatttgttgggcgag
>_R_gnl|SRA|ERR1278117.5836724.2:1-39 HWI-ST574:196:D08GAACXX:5:1108:15918:82046
agcacattaaataacagtaataggaatttgttgggcgag
>_R_gnl|SRA|ERR1278117.5793866.2:3-41 HWI-ST574:196:D08GAACXX:5:1108:6028:77133
agcacattaaataacagtaataggaatttgttgggcgag

>gnl|SRA|ERR1278117.5765152.1:12-50 HWI-ST574:196:D08GAACXX:5:1108:12327:73728
agcacattaaataacagtaataggaatttgttgggcgag
>gnl|SRA|ERR1278117.5602112.1:47-85 HWI-ST574:196:D08GAACXX:5:1108:19639:53822
agcacattaaataacagtaataggaatttgttgggcgag
>gnl|SRA|ERR1278117.5590105.1:59-97 HWI-ST574:196:D08GAACXX:5:1108:3508:52518
agcacattaaataacagtaataggaatttgttgggcgag
>gnl|SRA|ERR1278117.5516452.1:60-98 HWI-ST574:196:D08GAACXX:5:1108:12708:43724
agcacattaaataacagtaataggaatttgttgggcgag
>gnl|SRA|ERR1278117.5497742.1:38-76 HWI-ST574:196:D08GAACXX:5:1108:14687:41443
agcacattaaataacagtaataggaatttgttgggcgag
>gnl|SRA|ERR1278117.5395532.1:30-68 HWI-ST574:196:D08GAACXX:5:1108:5768:29234
agcacattaaataacagtaataggaatttgttgggcgag
>gnl|SRA|ERR1278117.5312158.1:30-68 HWI-ST574:196:D08GAACXX:5:1108:18048:18906
agcacattaaataacagtaataggaatttgttgggcgag
>gnl|SRA|ERR1278117.5220257.1:29-67 HWI-ST574:196:D08GAACXX:5:1108:5037:7868
agcacattaaataacagtaataggaatttgttgggcgag
>gnl|SRA|ERR1278117.5152357.1:37-75 HWI-ST574:196:D08GAACXX:5:1107:8053:98385
agcacattaaataacagtaataggaatttgttgggcgag
>gnl|SRA|ERR1278117.4983805.1:5-43 HWI-ST574:196:D08GAACXX:5:1107:17727:77715
agcacattaaataacagtaataggaatttgttgggcgag
>gnl|SRA|ERR1278117.4914146.1:41-79 HWI-ST574:196:D08GAACXX:5:1107:6692:69185
agcacattaaataacagtaataggaatttgttgggcgag
>gnl|SRA|ERR1278117.4887713.1:44-82 HWI-ST574:196:D08GAACXX:5:1107:6552:65911
agcacattaaataacagtaataggaatttgttgggcgag
>gnl|SRA|ERR1278117.4884181.1:62-100 HWI-ST574:196:D08GAACXX:5:1107:12354:65258
agcacattaaataacagtaataggaatttgttgggcgag
>gnl|SRA|ERR1278117.4810193.1:50-88 HWI-ST574:196:D08GAACXX:5:1107:3657:56352
agcacattaaataacagtaataggaatttgttgggcgag
>gnl|SRA|ERR1278117.4796530.1:5-43 HWI-ST574:196:D08GAACXX:5:1107:7928:54712
agcacattaaataacagtaataggaatttgttgggcgag
>gnl|SRA|ERR1278117.4748696.1:11-49 HWI-ST574:196:D08GAACXX:5:1107:13926:48519
agcacattaaataacagtaataggaatttgttgggcgag
>gnl|SRA|ERR1278117.4727967.1:30-68 HWI-ST574:196:D08GAACXX:5:1107:5193:46217
agcacattaaataacagtaataggaatttgttgggcgag
>gnl|SRA|ERR1278117.4606231.1:49-87 HWI-ST574:196:D08GAACXX:5:1107:9697:30838
agcacattaaataacagtaataggaatttgttgggcgag
>gnl|SRA|ERR1278117.4582753.1:61-99 HWI-ST574:196:D08GAACXX:5:1107:14846:27878
agcacattaaataacagtaataggaatttgttgggcgag
>gnl|SRA|ERR1278117.4576212.1:33-71 HWI-ST574:196:D08GAACXX:5:1107:10226:27209
agcacattaaataacagtaataggaatttgttgggcgag
>_R_gnl|SRA|ERR1278117.4381939.2:3-41 HWI-ST574:196:D08GAACXX:5:1107:6070:2872
agcacattaaataacagtaataggaatttgttgggcgag
>gnl|SRA|ERR1278117.4242403.1:8-46 HWI-ST574:196:D08GAACXX:5:1106:20383:83961
agcacattaaataacagtaataggaatttgttgggcgag
>gnl|SRA|ERR1278117.3829843.1:47-85 HWI-ST574:196:D08GAACXX:5:1106:8603:31536
agcacattaaataacagtaataggaatttgttgggcgag
>gnl|SRA|ERR1278117.3778315.1:23-61 HWI-ST574:196:D08GAACXX:5:1106:1380:25148
agcacattaaataacagtaataggaatttgttgggcgag
>gnl|SRA|ERR1278117.3267943.1:60-98 HWI-ST574:196:D08GAACXX:5:1105:20843:57855
agcacattaaataacagtaataggaatttgttgggcgag
>gnl|SRA|ERR1278117.3140139.1:30-68 HWI-ST574:196:D08GAACXX:5:1105:10326:41292

agcacattaaataacagtaataggaatttgttgggcgag
>gnl|SRA|ERR1278117.3084294.1:60-98 HWI-ST574:196:D08GAACXX:5:1105:5675:34103
agcacattaaataacagtaataggaatttgttgggcgag
>gnl|SRA|ERR1278117.3066637.1:36-74 HWI-ST574:196:D08GAACXX:5:1105:21088:31738
agcacattaaataacagtaataggaatttgttgggcgag
>gnl|SRA|ERR1278117.3021038.1:27-65 HWI-ST574:196:D08GAACXX:5:1105:3885:25837
agcacattaaataacagtaataggaatttgttgggcgag
>gnl|SRA|ERR1278117.2936376.1:9-47 HWI-ST574:196:D08GAACXX:5:1105:15888:14742
agcacattaaataacagtaataggaatttgttgggcgag
>gnl|SRA|ERR1278117.2831689.1:3-41 HWI-ST574:196:D08GAACXX:5:1104:21027:99691
agcacattaaataacagtaataggaatttgttgggcgag
>gnl|SRA|ERR1278117.2657788.1:2-40 HWI-ST574:196:D08GAACXX:5:1104:15089:76554
agcacattaaataacagtaataggaatttgttgggcgag
>gnl|SRA|ERR1278117.2592510.1:5-43 HWI-ST574:196:D08GAACXX:5:1104:2246:68190
agcacattaaataacagtaataggaatttgttgggcgag
>gnl|SRA|ERR1278117.2522309.1:4-42 HWI-ST574:196:D08GAACXX:5:1104:11905:58634
agcacattaaataacagtaataggaatttgttgggcgag
>_R_gnl|SRA|ERR1278117.2445081.2:1-39 HWI-ST574:196:D08GAACXX:5:1104:1371:48291
agcacattaaataacagtaataggaatttgttgggcgag
>_R_gnl|SRA|ERR1278117.2375427.2:3-41 HWI-ST574:196:D08GAACXX:5:1104:16493:38977
agcacattaaataacagtaataggaatttgttgggcgag
>gnl|SRA|ERR1278117.2201462.1:35-73 HWI-ST574:196:D08GAACXX:5:1104:6387:15688
agcacattaaataacagtaataggaatttgttgggcgag
>gnl|SRA|ERR1278117.1990375.1:12-50 HWI-ST574:196:D08GAACXX:5:1103:3038:85772
agcacattaaataacagtaataggaatttgttgggcgag
>gnl|SRA|ERR1278117.1900509.1:7-45 HWI-ST574:196:D08GAACXX:5:1103:3388:73615
agcacattaaataacagtaataggaatttgttgggcgag
>gnl|SRA|ERR1278117.1596442.1:48-86 HWI-ST574:196:D08GAACXX:5:1103:5658:31937
agcacattaaataacagtaataggaatttgttgggcgag
>gnl|SRA|ERR1278117.1561916.1:7-45 HWI-ST574:196:D08GAACXX:5:1103:4772:27150
agcacattaaataacagtaataggaatttgttgggcgag
>_R_gnl|SRA|ERR1278117.1484380.2:3-41 HWI-ST574:196:D08GAACXX:5:1103:2757:16357
agcacattaaataacagtaataggaatttgttgggcgag
>gnl|SRA|ERR1278117.1455173.1:62-100 HWI-ST574:196:D08GAACXX:5:1103:17827:12087
agcacattaaataacagtaataggaatttgttgggcgag
>gnl|SRA|ERR1278117.1058098.1:34-72 HWI-ST574:196:D08GAACXX:5:1102:20041:55389
agcacattaaataacagtaataggaatttgttgggcgag
>gnl|SRA|ERR1278117.975281.1:60-98 HWI-ST574:196:D08GAACXX:5:1102:11405:43528
agcacattaaataacagtaataggaatttgttgggcgag
>gnl|SRA|ERR1278117.933164.1:49-87 HWI-ST574:196:D08GAACXX:5:1102:8709:37607
agcacattaaataacagtaataggaatttgttgggcgag
>gnl|SRA|ERR1278117.854491.1:6-44 HWI-ST574:196:D08GAACXX:5:1102:1962:26353
agcacattaaataacagtaataggaatttgttgggcgag
>gnl|SRA|ERR1278117.619259.1:8-46 HWI-ST574:196:D08GAACXX:5:1101:19520:91286
agcacattaaataacagtaataggaatttgttgggcgag
>gnl|SRA|ERR1278117.592381.1:48-86 HWI-ST574:196:D08GAACXX:5:1101:12402:87542
agcacattaaataacagtaataggaatttgttgggcgag
>gnl|SRA|ERR1278117.473038.1:3-41 HWI-ST574:196:D08GAACXX:5:1101:15541:70343
agcacattaaataacagtaataggaatttgttgggcgag
>gnl|SRA|ERR1278117.360319.1:8-46 HWI-ST574:196:D08GAACXX:5:1101:18930:54015
agcacattaaataacagtaataggaatttgttgggcgag

>gnl|SRA|ERR1278117.235212.1:8-46 HWI-ST574:196:D08GAACXX:5:1101:18575:36067
agcacattaaataacagtaataggaatttgttgggcgag
>gnl|SRA|ERR1278117.74706335.1:63-100 HWI-ST574:196:D08GAACXX:5:2308:19501:81022
agcacattaaataacagtaataggaatttgttgggcga-
>_R_gnl|SRA|ERR1278117.73712402.2:1-38 HWI-
ST574:196:D08GAACXX:5:2307:20001:62790
agcacattaaataacagtaataggaatttgttgggcga-
>gnl|SRA|ERR1278117.69861225.1:1-38 HWI-ST574:196:D08GAACXX:5:2302:3242:66884
-gcacattaaataacagtaataggaatttgttgggcgag
>gnl|SRA|ERR1278117.68937275.1:5-42 HWI-ST574:196:D08GAACXX:5:2301:10399:36841
-gcacattaaataacagtaataggaatttgttgggcgag
>gnl|SRA|ERR1278117.67148672.1:1-38 HWI-ST574:196:D08GAACXX:5:2215:16745:62654
-gcacattaaataacagtaataggaatttgttgggcgag
>gnl|SRA|ERR1278117.54935527.1:63-100 HWI-ST574:196:D08GAACXX:5:2201:13329:12865
agcacattaaataacagtaataggaatttgttgggcga-
>gnl|SRA|ERR1278117.54393339.1:63-100 HWI-ST574:196:D08GAACXX:5:2116:6463:61071
agcacattaaataacagtaataggaatttgttgggcga-
>gnl|SRA|ERR1278117.54327664.1:3-40 HWI-ST574:196:D08GAACXX:5:2116:1951:55287
-gcacattaaataacagtaataggaatttgttgggcgag
>gnl|SRA|ERR1278117.39878567.1:1-38 HWI-ST574:196:D08GAACXX:5:1315:3214:74529
-gcacattaaataacagtaataggaatttgttgggcgag
>gnl|SRA|ERR1278117.39859483.1:63-100 HWI-ST574:196:D08GAACXX:5:1315:18556:72614
agcacattaaataacagtaataggaatttgttgggcga-
>gnl|SRA|ERR1278117.31674748.1:3-40 HWI-ST574:196:D08GAACXX:5:1306:8461:82212
-gcacattaaataacagtaataggaatttgttgggcgag
>gnl|SRA|ERR1278117.15520524.1:1-38 HWI-ST574:196:D08GAACXX:5:1203:6697:87526
-gcacattaaataacagtaataggaatttgttgggcgag
>gnl|SRA|ERR1278117.14731737.1:63-100 HWI-ST574:196:D08GAACXX:5:1202:12294:81341
agcacattaaataacagtaataggaatttgttgggcga-
>gnl|SRA|ERR1278117.10804055.1:1-38 HWI-ST574:196:D08GAACXX:5:1114:21161:40868
-gcacattaaataacagtaataggaatttgttgggcgag
>gnl|SRA|ERR1278117.9751971.1:1-38 HWI-ST574:196:D08GAACXX:5:1113:4209:29927
-gcacattaaataacagtaataggaatttgttgggcgag
>gnl|SRA|ERR1278117.4788583.1:63-100 HWI-ST574:196:D08GAACXX:5:1107:8817:53708
agcacattaaataacagtaataggaatttgttgggcga-
>_R_gnl|SRA|ERR1278117.81601485.2:1-37 HWI-ST574:196:D08GAACXX:5:2316:7881:5414
agcacattaaataacagtaataggaatttgttgggcg--
>gnl|SRA|ERR1278117.81409019.1:7-43 HWI-ST574:196:D08GAACXX:5:2315:20457:86504
--cacattaaataacagtaataggaatttgttgggcgag
>_R_gnl|SRA|ERR1278117.81245986.2:5-41 HWI-ST574:196:D08GAACXX:5:2315:1690:71936
agcacattaaataacagtaataggaatttgttgggcg--
>_R_gnl|SRA|ERR1278117.77558458.2:1-37 HWI-ST574:196:D08GAACXX:5:2312:13146:2152
agcacattaaataacagtaataggaatttgttgggcg--
>gnl|SRA|ERR1278117.77378133.1:1-37 HWI-ST574:196:D08GAACXX:5:2311:9816:81806
--cacattaaataacagtaataggaatttgttgggcgag
>_R_gnl|SRA|ERR1278117.72208635.2:1-37 HWI-
ST574:196:D08GAACXX:5:2305:19209:76269
agcacattaaataacagtaataggaatttgttgggcg--
>gnl|SRA|ERR1278117.69257456.1:64-100 HWI-ST574:196:D08GAACXX:5:2301:6242:81787
agcacattaaataacagtaataggaatttgttgggcg--

>_R_gnl|SRA|ERR1278117.65588347.2:1-37 HWI-
ST574:196:D08GAACXX:5:2214:19090:10568
agcacattaaataacagtaataggaatttgttgggcg--
>gnl|SRA|ERR1278117.64796384.1:11-47 HWI-ST574:196:D08GAACXX:5:2213:4484:26221
--cacattaaataacagtaataggaatttgttgggcgag
>_R_gnl|SRA|ERR1278117.63741563.2:1-37 HWI-
ST574:196:D08GAACXX:5:2212:12268:12320
agcacattaaataacagtaataggaatttgttgggcg--
>gnl|SRA|ERR1278117.60459527.1:1-37 HWI-ST574:196:D08GAACXX:5:2208:20203:35395
--cacattaaataacagtaataggaatttgttgggcgag
>_R_gnl|SRA|ERR1278117.57362750.2:1-37 HWI-
ST574:196:D08GAACXX:5:2204:13043:46058
agcacattaaataacagtaataggaatttgttgggcg--
>gnl|SRA|ERR1278117.50597912.1:64-100 HWI-ST574:196:D08GAACXX:5:2112:15976:82769
agcacattaaataacagtaataggaatttgttgggcg--
>_R_gnl|SRA|ERR1278117.46098469.2:1-37 HWI-ST574:196:D08GAACXX:5:2107:3559:60061
agcacattaaataacagtaataggaatttgttgggcg--
>_R_gnl|SRA|ERR1278117.37890067.2:1-37 HWI-ST574:196:D08GAACXX:5:1313:6456:78743
agcacattaaataacagtaataggaatttgttgggcg--
>_R_gnl|SRA|ERR1278117.37598636.2:1-37 HWI-
ST574:196:D08GAACXX:5:1313:15345:48967
agcacattaaataacagtaataggaatttgttgggcg--
>gnl|SRA|ERR1278117.37180455.1:1-37 HWI-ST574:196:D08GAACXX:5:1313:6416:6087
--cacattaaataacagtaataggaatttgttgggcgag
>gnl|SRA|ERR1278117.33377510.1:1-37 HWI-ST574:196:D08GAACXX:5:1308:6998:86110
--cacattaaataacagtaataggaatttgttgggcgag
>gnl|SRA|ERR1278117.31188054.1:1-37 HWI-ST574:196:D08GAACXX:5:1306:12067:22391
--cacattaaataacagtaataggaatttgttgggcgag
>gnl|SRA|ERR1278117.21461506.1:1-37 HWI-ST574:196:D08GAACXX:5:1211:8646:10683
--cacattaaataacagtaataggaatttgttgggcgag
>_R_gnl|SRA|ERR1278117.18640925.2:5-41 HWI-ST574:196:D08GAACXX:5:1207:2757:82849
agcacattaaataacagtaataggaatttgttgggcg--
>_R_gnl|SRA|ERR1278117.17806270.2:5-41 HWI-ST574:196:D08GAACXX:5:1206:8697:80919
agcacattaaataacagtaataggaatttgttgggcg--
>gnl|SRA|ERR1278117.15063261.1:1-37 HWI-ST574:196:D08GAACXX:5:1203:21037:26838
--cacattaaataacagtaataggaatttgttgggcgag
>gnl|SRA|ERR1278117.10628628.1:1-37 HWI-ST574:196:D08GAACXX:5:1114:17734:22876
--cacattaaataacagtaataggaatttgttgggcgag
>gnl|SRA|ERR1278117.82513068.1:36-74 HWI-ST574:196:D08GAACXX:5:2316:6707:85230
agcacattaaataacagtaataggaatttgttgggagag
>_R_gnl|SRA|ERR1278117.82393577.2:1-36 HWI-
ST574:196:D08GAACXX:5:2316:10919:74754
agcacattaaataacagtaataggaatttgttgggc---
>gnl|SRA|ERR1278117.82109146.1:1-36 HWI-ST574:196:D08GAACXX:5:2316:21329:50143
---acattaaataacagtaataggaatttgttgggcgag
>_R_gnl|SRA|ERR1278117.81616624.2:3-41 HWI-ST574:196:D08GAACXX:5:2316:16215:6703
agcacattaaataccagtaataggaatttgttgggcgag
>gnl|SRA|ERR1278117.81359020.1:1-36 HWI-ST574:196:D08GAACXX:5:2315:12259:82100
---acattaaataacagtaataggaatttgttgggcgag
>gnl|SRA|ERR1278117.76611905.1:2-40 HWI-ST574:196:D08GAACXX:5:2310:15936:97989
agtacattaaataacagtaataggaatttgttgggcgag

>gnl|SRA|ERR1278117.72428809.1:1-36 HWI-ST574:196:D08GAACXX:5:2306:5788:5138
---acattaaataacagtaataggaatttgttgggcgag
>gnl|SRA|ERR1278117.71048243.1:1-36 HWI-ST574:196:D08GAACXX:5:2304:1994:27135
---acattaaataacagtaataggaatttgttgggcgag
>_R_gnl|SRA|ERR1278117.67584195.2:1-36 HWI-ST574:196:D08GAACXX:5:2216:9093:4003
agcacattaaataacagtaataggaatttgttgggc---
>gnl|SRA|ERR1278117.67211959.1:32-70 HWI-ST574:196:D08GAACXX:5:2215:10502:68525
agcaaattaaataacagtaataggaatttgttgggcgag
>gnl|SRA|ERR1278117.64794249.1:1-36 HWI-ST574:196:D08GAACXX:5:2213:6125:25782
---acattaaataacagtaataggaatttgttgggcgag
>_R_gnl|SRA|ERR1278117.62394966.2:1-36 HWI-
ST574:196:D08GAACXX:5:2210:17467:61387
agcacattaaataacagtaataggaatttgttgggc---
>gnl|SRA|ERR1278117.62132407.1:1-36 HWI-ST574:196:D08GAACXX:5:2210:10106:31768
---acattaaataacagtaataggaatttgttgggcgag
>gnl|SRA|ERR1278117.61272931.1:49-87 HWI-ST574:196:D08GAACXX:5:2209:4172:32064
agcacattaaataagagtaataggaatttgttgggcgag
>gnl|SRA|ERR1278117.59269135.1:59-97 HWI-ST574:196:D08GAACXX:5:2206:9435:89342
agcacattaaataacagtaataggaatctgttgggcgag
>gnl|SRA|ERR1278117.57752333.1:1-36 HWI-ST574:196:D08GAACXX:5:2204:19094:96333
---acattaaataacagtaataggaatttgttgggcgag
>gnl|SRA|ERR1278117.56936687.1:1-36 HWI-ST574:196:D08GAACXX:5:2203:17333:89109
---acattaaataacagtaataggaatttgttgggcgag
>gnl|SRA|ERR1278117.56903503.1:12-50 HWI-ST574:196:D08GAACXX:5:2203:4860:84791
agcacattaaataacagtaataggaatttattgggcgag
>gnl|SRA|ERR1278117.52600785.1:1-36 HWI-ST574:196:D08GAACXX:5:2114:13310:91765
---acattaaataacagtaataggaatttgttgggcgag
>gnl|SRA|ERR1278117.52548217.1:7-45 HWI-ST574:196:D08GAACXX:5:2114:2639:86803
agcccattaaataacagtaataggaatttgttgggcgag
>gnl|SRA|ERR1278117.52004898.1:1-36 HWI-ST574:196:D08GAACXX:5:2114:19601:32920
---acattaaataacagtaataggaatttgttgggcgag
>gnl|SRA|ERR1278117.50489502.1:1-36 HWI-ST574:196:D08GAACXX:5:2112:7250:71294
---acattaaataacagtaataggaatttgttgggcgag
>gnl|SRA|ERR1278117.47690776.1:60-98 HWI-ST574:196:D08GAACXX:5:2109:12844:53914
agcccattaaataacagtaataggaatttgttgggcgag
>gnl|SRA|ERR1278117.46084855.1:2-40 HWI-ST574:196:D08GAACXX:5:2107:10704:58466
agcacattaaacaacagtaataggaatttgttgggcgag
>_R_gnl|SRA|ERR1278117.45698772.2:1-36 HWI-ST574:196:D08GAACXX:5:2107:6970:10064
agcacattaaataacagtaataggaatttgttgggc---
>gnl|SRA|ERR1278117.43067610.1:7-45 HWI-ST574:196:D08GAACXX:5:2103:7255:61033
agcacattaaataacagtaataggaatttgttggccgag
>gnl|SRA|ERR1278117.42449369.1:59-97 HWI-ST574:196:D08GAACXX:5:2102:10868:74287
agcacattaaacaacagtaataggaatttgttgggcgag
>gnl|SRA|ERR1278117.41619080.1:47-85 HWI-ST574:196:D08GAACXX:5:2101:15025:54333
agcacattaaagaacagtaataggaatttgttgggcgag
>gnl|SRA|ERR1278117.38248227.1:60-98 HWI-ST574:196:D08GAACXX:5:1314:3551:15897
agcacattaaataacagtaataggaatatgttgggcgag
>_R_gnl|SRA|ERR1278117.37063698.2:1-36 HWI-
ST574:196:D08GAACXX:5:1312:10523:92792
agcacattaaataacagtaataggaatttgttgggc---
>gnl|SRA|ERR1278117.36773791.1:42-80 HWI-ST574:196:D08GAACXX:5:1312:19271:62743

agcacattaaataacagtaataggagtttgttgggcgag
>_R_gnl|SRA|ERR1278117.35440245.2:1-36 HWI-ST574:196:D08GAACXX:5:1311:8115:19840
agcacattaaataacagtaataggaatttgttgggc---
>gnl|SRA|ERR1278117.33998661.1:48-86 HWI-ST574:196:D08GAACXX:5:1309:14952:58164
agcacattaaataacagtaatagcaatttgttgggcgag
>gnl|SRA|ERR1278117.33439790.1:1-36 HWI-ST574:196:D08GAACXX:5:1308:2055:93287
---acattaaataacagtaataggaatttgttgggcgag
>gnl|SRA|ERR1278117.33109180.1:1-36 HWI-ST574:196:D08GAACXX:5:1308:17884:54835
---acattaaataacagtaataggaatttgttgggcgag
>_R_gnl|SRA|ERR1278117.29978106.2:1-36 HWI-
ST574:196:D08GAACXX:5:1304:19818:67656
agcacattaaataacagtaataggaatttgttgggc---
>gnl|SRA|ERR1278117.28759815.1:34-72 HWI-ST574:196:D08GAACXX:5:1303:4750:6980
agcacattatataacagtaataggaatttgttgggcgag
>gnl|SRA|ERR1278117.27918700.1:36-74 HWI-ST574:196:D08GAACXX:5:1301:5629:88776
agcacattaagtaacagtaataggaatttgttgggcgag
>_R_gnl|SRA|ERR1278117.27518527.2:1-36 HWI-ST574:196:D08GAACXX:5:1301:3202:33009
agcacattaaataacagtaataggaatttgttgggc---
>_R_gnl|SRA|ERR1278117.26229677.2:3-41 HWI-ST574:196:D08GAACXX:5:1216:5925:4590
agcacattgaataacagtaataggaatttgttgggcgag
>gnl|SRA|ERR1278117.25475858.1:1-36 HWI-ST574:196:D08GAACXX:5:1215:17825:31305
---acattaaataacagtaataggaatttgttgggcgag
>_R_gnl|SRA|ERR1278117.14613306.2:2-40 HWI-ST574:196:D08GAACXX:5:1202:7379:65361
agcacattaaataacagtaataggaatttgttcggcgag
>gnl|SRA|ERR1278117.8136673.1:1-36 HWI-ST574:196:D08GAACXX:5:1111:8302:51571
---acattaaataacagtaataggaatttgttgggcgag
>gnl|SRA|ERR1278117.4644378.1:1-36 HWI-ST574:196:D08GAACXX:5:1107:11323:35603
---acattaaataacagtaataggaatttgttgggcgag
>_R_gnl|SRA|ERR1278117.1418896.2:3-41 HWI-ST574:196:D08GAACXX:5:1103:16277:7225
agcacattaaataacagtaataggaatttgttgggcaag
>_R_gnl|SRA|ERR1278117.65694418.2:66-100 HWI-
ST574:196:D08GAACXX:5:2214:11886:21292
----cattaaataacagtaataggaatttgttgggcgag
>gnl|SRA|ERR1278117.44700422.1:5-42 HWI-ST574:196:D08GAACXX:5:2105:14163:80275
-gcacattaaataacagtaataggaatttgttgggtgag
>_R_gnl|SRA|ERR1278117.28480207.2:66-100 HWI-
ST574:196:D08GAACXX:5:1302:17193:67641
----cattaaataacagtaataggaatttgttgggcgag
>gnl|SRA|ERR1278117.25001633.1:1-35 HWI-ST574:196:D08GAACXX:5:1214:8771:84043
----cattaaataacagtaataggaatttgttgggcgag
>gnl|SRA|ERR1278117.16825509.1:1-35 HWI-ST574:196:D08GAACXX:5:1205:13326:57950
----cattaaataacagtaataggaatttgttgggcgag
>_R_gnl|SRA|ERR1278117.4856264.2:66-100 HWI-
ST574:196:D08GAACXX:5:1107:18098:61977
----cattaaataacagtaataggaatttgttgggcgag
>_R_gnl|SRA|ERR1278117.81873476.2:46-83 HWI-
ST574:196:D08GAACXX:5:2316:4830:29508
agcacattaaataacagtaataggaattgttgggcgag-
>_R_gnl|SRA|ERR1278117.80792942.2:29-66 HWI-
ST574:196:D08GAACXX:5:2315:5938:29784
agcaca-ttaataacagtaataggaatttgttgggcgag

>_R_gnl|SRA|ERR1278117.80404581.2:29-66 HWI-
ST574:196:D08GAACXX:5:2314:17957:92054
agcaca-ttaataacagtaataggaatttgttgggcgag
>_R_gnl|SRA|ERR1278117.80180440.2:29-66 HWI-
ST574:196:D08GAACXX:5:2314:3107:70961
agcaca-ttaataacagtaataggaatttgttgggcgag
>_R_gnl|SRA|ERR1278117.68395229.2:29-66 HWI-
ST574:196:D08GAACXX:5:2216:6762:76195
agcaca-ttaataacagtaataggaatttgttgggcgag
>_R_gnl|SRA|ERR1278117.65680767.2:30-67 HWI-
ST574:196:D08GAACXX:5:2214:2177:20225
agcaca-ttaataacagtaataggaatttgttgggcgag
>_R_gnl|SRA|ERR1278117.65034004.2:30-67 HWI-
ST574:196:D08GAACXX:5:2213:17066:50841
agcaca-ttaataacagtaataggaatttgttgggcgag
>_R_gnl|SRA|ERR1278117.60634328.2:29-66 HWI-
ST574:196:D08GAACXX:5:2208:20781:56163
agcaca-ttaataacagtaataggaatttgttgggcgag
>_R_gnl|SRA|ERR1278117.59562407.2:30-67 HWI-
ST574:196:D08GAACXX:5:2207:20990:26230
agcaca-ttaataacagtaataggaatttgttgggcgag
>_R_gnl|SRA|ERR1278117.57192110.2:29-66 HWI-
ST574:196:D08GAACXX:5:2204:14813:23956
agcaca-ttaataacagtaataggaatttgttgggcgag
>_R_gnl|SRA|ERR1278117.56310488.2:46-83 HWI-ST574:196:D08GAACXX:5:2203:9733:5849
agcacattaaataacagtaataggaattgttgggcgag-
>gnl|SRA|ERR1278117.56103892.1:1-34 HWI-ST574:196:D08GAACXX:5:2202:10532:76545
-----attaaataacagtaataggaatttgttgggcgag
>_R_gnl|SRA|ERR1278117.55869575.2:28-65 HWI-
ST574:196:D08GAACXX:5:2202:12656:44257
agcaca-ttaataacagtaataggaatttgttgggcgag
>_R_gnl|SRA|ERR1278117.53465530.2:29-66 HWI-
ST574:196:D08GAACXX:5:2115:9076:75545
agcaca-ttaataacagtaataggaatttgttgggcgag
>_R_gnl|SRA|ERR1278117.53111869.2:27-64 HWI-
ST574:196:D08GAACXX:5:2115:16373:42216
agcaca-taaataacagtaataggaatttgttgggcgag
>_R_gnl|SRA|ERR1278117.51916160.2:29-66 HWI-
ST574:196:D08GAACXX:5:2114:7019:24010
agcaca-ttaataacagtaataggaatttgttgggcgag
>_R_gnl|SRA|ERR1278117.50782388.2:29-66 HWI-
ST574:196:D08GAACXX:5:2113:20311:3551
agcaca-ttaataacagtaataggaatttgttgggcgag
>_R_gnl|SRA|ERR1278117.45367142.2:29-66 HWI-
ST574:196:D08GAACXX:5:2106:6634:67069
agcaca-ttaataacagtaataggaatttgttgggcgag
>_R_gnl|SRA|ERR1278117.43202480.2:29-66 HWI-
ST574:196:D08GAACXX:5:2103:10542:79339
agcaca-ttaataacagtaataggaatttgttgggcgag
>_R_gnl|SRA|ERR1278117.42749237.2:29-66 HWI-
ST574:196:D08GAACXX:5:2103:8549:17412

agcaca-ttaataacagtaataggaatttgttgggcgag
>_R_gnl|SRA|ERR1278117.40056714.2:29-66 HWI-
ST574:196:D08GAACXX:5:1315:15627:91174
agcaca-ttaataacagtaataggaatttgttgggcgag
>_R_gnl|SRA|ERR1278117.37914473.2:29-66 HWI-
ST574:196:D08GAACXX:5:1313:5892:81102
agcaca-ttaataacagtaataggaatttgttgggcgag
>_R_gnl|SRA|ERR1278117.37851667.2:46-83 HWI-
ST574:196:D08GAACXX:5:1313:13700:74744
agcacattaaataacagtaataggaattgttgggcgag-
>_R_gnl|SRA|ERR1278117.35202128.2:29-66 HWI-
ST574:196:D08GAACXX:5:1310:9071:92868
agcaca-ttaataacagtaataggaatttgttgggcgag
>_R_gnl|SRA|ERR1278117.34530425.2:29-66 HWI-
ST574:196:D08GAACXX:5:1310:15565:18786
agcaca-ttaataacagtaataggaatttgttgggcgag
>gnl|SRA|ERR1278117.30001538.1:1-34 HWI-ST574:196:D08GAACXX:5:1304:1523:70954
-----attaaataacagtaataggaatttgttgggcgag
>_R_gnl|SRA|ERR1278117.29595641.2:29-66 HWI-
ST574:196:D08GAACXX:5:1304:17667:18076
agcaca-ttaataacagtaataggaatttgttgggcgag
>_R_gnl|SRA|ERR1278117.28336208.2:28-65 HWI-
ST574:196:D08GAACXX:5:1302:2516:48203
agcaca-ttaataacagtaataggaatttgttgggcgag
>_R_gnl|SRA|ERR1278117.27376574.2:46-83 HWI-
ST574:196:D08GAACXX:5:1301:10803:13042
agcacattaaataacagtaataggaattgttgggcgag-
>_R_gnl|SRA|ERR1278117.26554916.2:30-67 HWI-
ST574:196:D08GAACXX:5:1216:18351:34678
agcaca-ttaataacagtaataggaatttgttgggcgag
>_R_gnl|SRA|ERR1278117.25541877.2:29-66 HWI-
ST574:196:D08GAACXX:5:1215:6140:37819
agcaca-ttaataacagtaataggaatttgttgggcgag
>_R_gnl|SRA|ERR1278117.22924509.2:29-66 HWI-
ST574:196:D08GAACXX:5:1212:8889:69452
agcaca-ttaataacagtaataggaatttgttgggcgag
>_R_gnl|SRA|ERR1278117.21213955.2:46-83 HWI-
ST574:196:D08GAACXX:5:1210:6292:82032
agcacattaaataacagtaataggaattgttgggcgag-
>_R_gnl|SRA|ERR1278117.19193174.2:36-73 HWI-
ST574:196:D08GAACXX:5:1208:7446:49459
agcacattaaataaca-taataggaatttgttgggcgag
>_R_gnl|SRA|ERR1278117.16049395.2:29-66 HWI-
ST574:196:D08GAACXX:5:1204:15399:57713
agcaca-ttaataacagtaataggaatttgttgggcgag
>_R_gnl|SRA|ERR1278117.15288288.2:46-83 HWI-
ST574:196:D08GAACXX:5:1203:14708:56834
agcacattaaataacagtaataggaattgttgggcgag-
>_R_gnl|SRA|ERR1278117.9484924.2:29-66 HWI-
ST574:196:D08GAACXX:5:1112:3353:100064
agcaca-ttaataacagtaataggaatttgttgggcgag

>_R_gnl|SRA|ERR1278117.8523196.2:29-66 HWI-
ST574:196:D08GAACXX:5:1111:19240:94452
agcaca-ttaataacagtaataggaatttgttgggcgag
>_R_gnl|SRA|ERR1278117.4078407.2:29-66 HWI-
ST574:196:D08GAACXX:5:1106:19229:63140
agcaca-ttaataacagtaataggaatttgttgggcgag
>_R_gnl|SRA|ERR1278117.151631.2:28-65 HWI-ST574:196:D08GAACXX:5:1101:7865:24113
agcaca-ttaataacagtaataggaatttgttgggcgag
>gnl|SRA|ERR1278117.69779889.1:1-33 HWI-ST574:196:D08GAACXX:5:2302:17484:55750
------ttaaataacagtaataggaatttgttgggcgag
>gnl|SRA|ERR1278117.51834665.1:1-33 HWI-ST574:196:D08GAACXX:5:2114:5318:15973
------ttaaataacagtaataggaatttgttgggcgag
>gnl|SRA|ERR1278117.34446481.1:68-100 HWI-ST574:196:D08GAACXX:5:1310:9047:9536
agcacattaaataacagtaataggaatttgttg------
>gnl|SRA|ERR1278117.32625027.1:68-100 HWI-ST574:196:D08GAACXX:5:1307:18634:97209
agcacattaaataacagtaataggaatttgttg------
>gnl|SRA|ERR1278117.21285371.1:68-100 HWI-ST574:196:D08GAACXX:5:1210:19115:89995
agcacattaaataacagtaataggaatttgttg------
>gnl|SRA|ERR1278117.16683584.1:68-100 HWI-ST574:196:D08GAACXX:5:1205:14790:39912
agcacattaaataacagtaataggaatttgttg------
>_R_gnl|SRA|ERR1278117.5056805.2:1-36 HWI-ST574:196:D08GAACXX:5:1107:13756:86579
agcacattaaataacagtaataggaatttgtggggc---
>gnl|SRA|ERR1278117.655355.1:1-33 HWI-ST574:196:D08GAACXX:5:1101:11851:96678
------ttaaataacagtaataggaatttgttgggcgag
>gnl|SRA|ERR1278117.645826.1:68-100 HWI-ST574:196:D08GAACXX:5:1101:2885:95425
agcacattaaataacagtaataggaatttgttg------
>gnl|SRA|ERR1278117.81778376.1:1-32 HWI-ST574:196:D08GAACXX:5:2316:10210:21100
-------taaataacagtaataggaatttgttgggcgag
>gnl|SRA|ERR1278117.80274015.1:1-32 HWI-ST574:196:D08GAACXX:5:2314:2717:79909
-------taaataacagtaataggaatttgttgggcgag
>gnl|SRA|ERR1278117.76285775.1:1-32 HWI-ST574:196:D08GAACXX:5:2310:3782:62160
-------taaataacagtaataggaatttgttgggcgag
>gnl|SRA|ERR1278117.71892452.1:1-32 HWI-ST574:196:D08GAACXX:5:2305:9568:36519
-------taaataacagtaataggaatttgttgggcgag
>gnl|SRA|ERR1278117.65072971.1:69-100 HWI-ST574:196:D08GAACXX:5:2213:3635:55110
agcacattaaataacagtaataggaatttgtt-------
>gnl|SRA|ERR1278117.63738987.1:1-32 HWI-ST574:196:D08GAACXX:5:2212:9584:12118
-------taaataacagtaataggaatttgttgggcgag
>gnl|SRA|ERR1278117.57669641.1:1-32 HWI-ST574:196:D08GAACXX:5:2204:9513:85788
-------taaataacagtaataggaatttgttgggcgag
>gnl|SRA|ERR1278117.50384716.1:1-32 HWI-ST574:196:D08GAACXX:5:2112:14646:60147
-------taaataacagtaataggaatttgttgggcgag
>gnl|SRA|ERR1278117.47657029.1:1-32 HWI-ST574:196:D08GAACXX:5:2109:17169:49946
-------taaataacagtaataggaatttgttgggcgag
>gnl|SRA|ERR1278117.39400929.1:1-32 HWI-ST574:196:D08GAACXX:5:1315:2604:29337
-------taaataacagtaataggaatttgttgggcgag
>gnl|SRA|ERR1278117.38460672.1:1-32 HWI-ST574:196:D08GAACXX:5:1314:17697:36944
-------taaataacagtaataggaatttgttgggcgag
>gnl|SRA|ERR1278117.36231123.1:1-32 HWI-ST574:196:D08GAACXX:5:1312:15647:5598
-------taaataacagtaataggaatttgttgggcgag
>gnl|SRA|ERR1278117.33432279.1:1-32 HWI-ST574:196:D08GAACXX:5:1308:12777:92472

-------taaataacagtaataggaatttgttgggcgag
>gnl|SRA|ERR1278117.30411801.1:69-100 HWI-ST574:196:D08GAACXX:5:1305:16883:24159
agcacattaaataacagtaataggaatttgtt-------
>gnl|SRA|ERR1278117.22819911.1:69-100 HWI-ST574:196:D08GAACXX:5:1212:6752:58331
agcacattaaataacagtaataggaatttgtt-------
>gnl|SRA|ERR1278117.19452555.1:1-32 HWI-ST574:196:D08GAACXX:5:1208:8036:79635
-------taaataacagtaataggaatttgttgggcgag
>gnl|SRA|ERR1278117.10900716.1:1-32 HWI-ST574:196:D08GAACXX:5:1114:12116:50808
-------taaataacagtaataggaatttgttgggcgag
>gnl|SRA|ERR1278117.8154550.1:69-100 HWI-ST574:196:D08GAACXX:5:1111:8528:53548
agcacattaaataacagtaataggaatttgtt-------
>gnl|SRA|ERR1278117.3524691.1:1-32 HWI-ST574:196:D08GAACXX:5:1105:6822:91320
-------taaataacagtaataggaatttgttgggcgag
>gnl|SRA|ERR1278117.2850737.1:69-100 HWI-ST574:196:D08GAACXX:5:1105:16669:3449
agcacattaaataacagtaataggaatttgtt-------
>gnl|SRA|ERR1278117.2424280.1:69-100 HWI-ST574:196:D08GAACXX:5:1104:17449:45390
agcacattaaataacagtaataggaatttgtt-------
>gnl|SRA|ERR1278117.78242035.1:1-31 HWI-ST574:196:D08GAACXX:5:2312:16816:73865
--------aaataacagtaataggaatttgttgggcgag
>gnl|SRA|ERR1278117.75609691.1:1-31 HWI-ST574:196:D08GAACXX:5:2309:7172:85622
--------aaataacagtaataggaatttgttgggcgag
>gnl|SRA|ERR1278117.71180520.1:1-31 HWI-ST574:196:D08GAACXX:5:2304:19485:44015
--------aaataacagtaataggaatttgttgggcgag
>gnl|SRA|ERR1278117.69329282.1:1-31 HWI-ST574:196:D08GAACXX:5:2301:6850:91845
--------aaataacagtaataggaatttgttgggcgag
>gnl|SRA|ERR1278117.63301413.1:1-31 HWI-ST574:196:D08GAACXX:5:2211:16144:63111
--------aaataacagtaataggaatttgttgggcgag
>gnl|SRA|ERR1278117.62080205.1:1-31 HWI-ST574:196:D08GAACXX:5:2210:15102:25965
--------aaataacagtaataggaatttgttgggcgag
>gnl|SRA|ERR1278117.61578055.1:70-100 HWI-ST574:196:D08GAACXX:5:2209:9238:67275
agcacattaaataacagtaataggaatttgt--------
>gnl|SRA|ERR1278117.59452474.1:1-31 HWI-ST574:196:D08GAACXX:5:2207:12629:12935
--------aaataacagtaataggaatttgttgggcgag
>gnl|SRA|ERR1278117.59395348.1:70-100 HWI-ST574:196:D08GAACXX:5:2207:14394:5942
agcacattaaataacagtaataggaatttgt--------
>gnl|SRA|ERR1278117.59074420.1:1-31 HWI-ST574:196:D08GAACXX:5:2206:11710:65399
--------aaataacagtaataggaatttgttgggcgag
>gnl|SRA|ERR1278117.58855331.1:70-100 HWI-ST574:196:D08GAACXX:5:2206:15104:38246
agcacattaaataacagtaataggaatttgt--------
>gnl|SRA|ERR1278117.49103552.1:1-31 HWI-ST574:196:D08GAACXX:5:2111:13482:18362
--------aaataacagtaataggaatttgttgggcgag
>gnl|SRA|ERR1278117.48880579.1:1-31 HWI-ST574:196:D08GAACXX:5:2110:20349:92092
--------aaataacagtaataggaatttgttgggcgag
>gnl|SRA|ERR1278117.45650240.1:70-100 HWI-ST574:196:D08GAACXX:5:2107:19196:3855
agcacattaaataacagtaataggaatttgt--------
>gnl|SRA|ERR1278117.40368087.1:70-100 HWI-ST574:196:D08GAACXX:5:1316:18487:21236
agcacattaaataacagtaataggaatttgt--------
>gnl|SRA|ERR1278117.39658901.1:70-100 HWI-ST574:196:D08GAACXX:5:1315:19120:53682
agcacattaaataacagtaataggaatttgt--------
>gnl|SRA|ERR1278117.38360117.1:1-31 HWI-ST574:196:D08GAACXX:5:1314:16703:26909
--------aaataacagtaataggaatttgttgggcgag

>gnl|SRA|ERR1278117.37349253.1:70-100 HWI-ST574:196:D08GAACXX:5:1313:16894:23483
agcacattaaataacagtaataggaatttgt--------
>gnl|SRA|ERR1278117.35574514.1:1-31 HWI-ST574:196:D08GAACXX:5:1311:5268:34333
--------aaataacagtaataggaatttgttgggcgag
>gnl|SRA|ERR1278117.35057172.1:1-31 HWI-ST574:196:D08GAACXX:5:1310:6792:77004
--------aaataacagtaataggaatttgttgggcgag
>gnl|SRA|ERR1278117.30717189.1:1-31 HWI-ST574:196:D08GAACXX:5:1305:14505:62748
--------aaataacagtaataggaatttgttgggcgag
>gnl|SRA|ERR1278117.25226545.1:70-100 HWI-ST574:196:D08GAACXX:5:1215:19128:7233
agcacattaaataacagtaataggaatttgt--------
>gnl|SRA|ERR1278117.24031351.1:70-100 HWI-ST574:196:D08GAACXX:5:1213:16421:85634
agcacattaaataacagtaataggaatttgt--------
>gnl|SRA|ERR1278117.21244702.1:70-100 HWI-ST574:196:D08GAACXX:5:1210:19961:85361
agcacattaaataacagtaataggaatttgt--------
>gnl|SRA|ERR1278117.20146503.1:1-31 HWI-ST574:196:D08GAACXX:5:1209:16265:60691
--------aaataacagtaataggaatttgttgggcgag
>gnl|SRA|ERR1278117.17411681.1:1-31 HWI-ST574:196:D08GAACXX:5:1206:20844:32097
--------aaataacagtaataggaatttgttgggcgag
>gnl|SRA|ERR1278117.17185019.1:70-100 HWI-ST574:196:D08GAACXX:5:1206:9915:4194
agcacattaaataacagtaataggaatttgt--------
>gnl|SRA|ERR1278117.14592136.1:1-31 HWI-ST574:196:D08GAACXX:5:1202:13998:62486
--------aaataacagtaataggaatttgttgggcgag
>gnl|SRA|ERR1278117.11366517.1:1-31 HWI-ST574:196:D08GAACXX:5:1114:9831:98126
--------aaataacagtaataggaatttgttgggcgag
>gnl|SRA|ERR1278117.4753376.1:1-31 HWI-ST574:196:D08GAACXX:5:1107:19354:49078
--------aaataacagtaataggaatttgttgggcgag
>gnl|SRA|ERR1278117.2652579.1:1-31 HWI-ST574:196:D08GAACXX:5:1104:19744:75879
--------aaataacagtaataggaatttgttgggcgag
>gnl|SRA|ERR1278117.69760418.1:1-30 HWI-ST574:196:D08GAACXX:5:2302:3724:53240
---------aataacagtaataggaatttgttgggcgag
>gnl|SRA|ERR1278117.58903291.1:71-100 HWI-ST574:196:D08GAACXX:5:2206:11047:44028
agcacattaaataacagtaataggaatttg---------
>gnl|SRA|ERR1278117.56892972.1:71-100 HWI-ST574:196:D08GAACXX:5:2203:13622:83462
agcacattaaataacagtaataggaatttg---------
>gnl|SRA|ERR1278117.49514471.1:1-30 HWI-ST574:196:D08GAACXX:5:2111:2312:64468
---------aataacagtaataggaatttgttgggcgag
>gnl|SRA|ERR1278117.32860725.1:1-30 HWI-ST574:196:D08GAACXX:5:1308:19221:25861
---------aataacagtaataggaatttgttgggcgag
>gnl|SRA|ERR1278117.24534225.1:1-30 HWI-ST574:196:D08GAACXX:5:1214:19582:37529
---------aataacagtaataggaatttgttgggcgag
>gnl|SRA|ERR1278117.22792361.1:70-99 HWI-ST574:196:D08GAACXX:5:1212:13670:55437
agcacattaaataacagtaataggaatttg---------
>gnl|SRA|ERR1278117.8088471.1:71-100 HWI-ST574:196:D08GAACXX:5:1111:15787:46088
agcacattaaataacagtaataggaatttg---------
>gnl|SRA|ERR1278117.6936745.1:1-30 HWI-ST574:196:D08GAACXX:5:1110:2809:14249
---------aataacagtaataggaatttgttgggcgag
>gnl|SRA|ERR1278117.6162257.1:1-30 HWI-ST574:196:D08GAACXX:5:1109:2596:22008
---------aataacagtaataggaatttgttgggcgag
>gnl|SRA|ERR1278117.79883450.1:1-29 HWI-ST574:196:D08GAACXX:5:2314:9099:42106
----------ataacagtaataggaatttgttgggcgag
>gnl|SRA|ERR1278117.77738419.1:1-29 HWI-ST574:196:D08GAACXX:5:2312:16777:21060

----------ataacagtaataggaatttgttgggcgag
>gnl|SRA|ERR1278117.73589401.1:72-100 HWI-ST574:196:D08GAACXX:5:2307:16887:48217
agcacattaaataacagtaataggaattt----------
>gnl|SRA|ERR1278117.57356664.1:72-100 HWI-ST574:196:D08GAACXX:5:2204:9902:45370
agcacattaaataacagtaataggaattt----------
>gnl|SRA|ERR1278117.43880621.1:1-29 HWI-ST574:196:D08GAACXX:5:2104:16033:71735
----------ataacagtaataggaatttgttgggcgag
>gnl|SRA|ERR1278117.33374986.1:1-29 HWI-ST574:196:D08GAACXX:5:1308:3038:85884
----------ataacagtaataggaatttgttgggcgag
>gnl|SRA|ERR1278117.32122751.1:72-100 HWI-ST574:196:D08GAACXX:5:1307:3865:37286
agcacattaaataacagtaataggaattt----------
>gnl|SRA|ERR1278117.32101046.1:72-100 HWI-ST574:196:D08GAACXX:5:1307:16907:34643
agcacattaaataacagtaataggaattt----------
>gnl|SRA|ERR1278117.31977423.1:1-29 HWI-ST574:196:D08GAACXX:5:1307:5260:19826
----------ataacagtaataggaatttgttgggcgag
>gnl|SRA|ERR1278117.28156214.1:72-100 HWI-ST574:196:D08GAACXX:5:1302:5146:23121
agcacattaaataacagtaataggaattt----------
>gnl|SRA|ERR1278117.26465929.1:72-100 HWI-ST574:196:D08GAACXX:5:1216:1639:26639
agcacattaaataacagtaataggaattt----------
>gnl|SRA|ERR1278117.26266540.1:72-100 HWI-ST574:196:D08GAACXX:5:1216:19069:7872
agcacattaaataacagtaataggaattt----------
>gnl|SRA|ERR1278117.22991104.1:1-29 HWI-ST574:196:D08GAACXX:5:1212:12055:76461
----------ataacagtaataggaatttgttgggcgag
>gnl|SRA|ERR1278117.17120197.1:72-100 HWI-ST574:196:D08GAACXX:5:1205:12951:94841
agcacattaaataacagtaataggaattt----------
>gnl|SRA|ERR1278117.16971668.1:1-29 HWI-ST574:196:D08GAACXX:5:1205:6632:76395
----------ataacagtaataggaatttgttgggcgag
>gnl|SRA|ERR1278117.16125108.1:72-100 HWI-ST574:196:D08GAACXX:5:1204:18499:67420
agcacattaaataacagtaataggaattt----------
>gnl|SRA|ERR1278117.11669018.1:72-100 HWI-ST574:196:D08GAACXX:5:1115:6215:29613
agcacattaaataacagtaataggaattt----------
>gnl|SRA|ERR1278117.10697474.1:72-100 HWI-ST574:196:D08GAACXX:5:1114:4871:30119
agcacattaaataacagtaataggaattt----------
>gnl|SRA|ERR1278117.9653964.1:1-29 HWI-ST574:196:D08GAACXX:5:1113:12312:19401
----------ataacagtaataggaatttgttgggcgag
>gnl|SRA|ERR1278117.1480339.1:1-29 HWI-ST574:196:D08GAACXX:5:1103:18095:15530
----------ataacagtaataggaatttgttgggcgag
>gnl|SRA|ERR1278117.790124.1:1-29 HWI-ST574:196:D08GAACXX:5:1102:6132:17037
----------ataacagtaataggaatttgttgggcgag
>gnl|SRA|ERR1278117.79608418.1:1-31 HWI-ST574:196:D08GAACXX:5:2314:18502:15245
--------aaataacagtaataagaatttgttgggcgag
>gnl|SRA|ERR1278117.78871996.1:1-31 HWI-ST574:196:D08GAACXX:5:2313:14891:39952
--------aaataacagtaatgggaatttgttgggcgag
>gnl|SRA|ERR1278117.67565510.1:1-28 HWI-ST574:196:D08GAACXX:5:2216:13977:2260
-----------taacagtaataggaatttgttgggcgag
>gnl|SRA|ERR1278117.62452659.1:1-28 HWI-ST574:196:D08GAACXX:5:2210:13681:67761
-----------taacagtaataggaatttgttgggcgag
>gnl|SRA|ERR1278117.58740893.1:73-100 HWI-ST574:196:D08GAACXX:5:2206:17974:23788
agcacattaaataacagtaataggaatt-----------
>gnl|SRA|ERR1278117.57062332.1:73-100 HWI-ST574:196:D08GAACXX:5:2204:12055:6985
agcacattaaataacagtaataggaatt-----------

>gnl|SRA|ERR1278117.53686124.1:1-28 HWI-ST574:196:D08GAACXX:5:2115:11476:96177
-----------taacagtaataggaatttgttgggcgag
>gnl|SRA|ERR1278117.53146842.1:1-28 HWI-ST574:196:D08GAACXX:5:2115:4355:45739
-----------taacagtaataggaatttgttgggcgag
>gnl|SRA|ERR1278117.48568244.1:1-28 HWI-ST574:196:D08GAACXX:5:2110:12404:56926
-----------taacagtaataggaatttgttgggcgag
>gnl|SRA|ERR1278117.46923889.1:1-28 HWI-ST574:196:D08GAACXX:5:2108:18757:61677
-----------taacagtaataggaatttgttgggcgag
>gnl|SRA|ERR1278117.45940670.1:1-28 HWI-ST574:196:D08GAACXX:5:2107:6329:40442
-----------taacagtaataggaatttgttgggcgag
>gnl|SRA|ERR1278117.42806306.1:73-100 HWI-ST574:196:D08GAACXX:5:2103:20389:25235
agcacattaaataacagtaataggaatt-----------
>gnl|SRA|ERR1278117.41019240.1:73-100 HWI-ST574:196:D08GAACXX:5:1316:1752:80156
agcacattaaataacagtaataggaatt-----------
>gnl|SRA|ERR1278117.39013094.1:1-28 HWI-ST574:196:D08GAACXX:5:1314:3470:91015
-----------taacagtaataggaatttgttgggcgag
>gnl|SRA|ERR1278117.34335121.1:73-100 HWI-ST574:196:D08GAACXX:5:1309:3494:96065
agcacattaaataacagtaataggaatt-----------
>gnl|SRA|ERR1278117.17902217.1:1-28 HWI-ST574:196:D08GAACXX:5:1206:7750:92599
-----------taacagtaataggaatttgttgggcgag
>gnl|SRA|ERR1278117.7336568.1:73-100 HWI-ST574:196:D08GAACXX:5:1110:18759:60209
agcacattaaataacagtaataggaatt-----------
>gnl|SRA|ERR1278117.354665.1:73-100 HWI-ST574:196:D08GAACXX:5:1101:13456:53389
agcacattaaataacagtaataggaatt-----------
>gnl|SRA|ERR1278117.80537646.1:1-27 HWI-ST574:196:D08GAACXX:5:2315:19097:5775
------------aacagtaataggaatttgttgggcgag
>gnl|SRA|ERR1278117.77082923.1:1-27 HWI-ST574:196:D08GAACXX:5:2311:15912:50196
------------aacagtaataggaatttgttgggcgag
>gnl|SRA|ERR1278117.76952095.1:1-27 HWI-ST574:196:D08GAACXX:5:2311:4792:36022
------------aacagtaataggaatttgttgggcgag
>gnl|SRA|ERR1278117.76849116.1:1-27 HWI-ST574:196:D08GAACXX:5:2311:12210:24817
------------aacagtaataggaatttgttgggcgag
>gnl|SRA|ERR1278117.75992551.1:1-27 HWI-ST574:196:D08GAACXX:5:2310:8089:29582
------------aacagtaataggaatttgttgggcgag
>gnl|SRA|ERR1278117.74822000.1:1-27 HWI-ST574:196:D08GAACXX:5:2308:8464:94679
------------aacagtaataggaatttgttgggcgag
>gnl|SRA|ERR1278117.73772283.1:1-27 HWI-ST574:196:D08GAACXX:5:2307:11953:70030
------------aacagtaataggaatttgttgggcgag
>gnl|SRA|ERR1278117.71400197.1:1-27 HWI-ST574:196:D08GAACXX:5:2304:16871:72673
------------aacagtaataggaatttgttgggcgag
>gnl|SRA|ERR1278117.70701940.1:1-27 HWI-ST574:196:D08GAACXX:5:2303:9820:80597
------------aacagtaataggaatttgttgggcgag
>gnl|SRA|ERR1278117.65999767.1:1-27 HWI-ST574:196:D08GAACXX:5:2214:1355:51525
------------aacagtaataggaatttgttgggcgag
>gnl|SRA|ERR1278117.65554903.1:1-27 HWI-ST574:196:D08GAACXX:5:2214:4704:7490
------------aacagtaataggaatttgttgggcgag
>gnl|SRA|ERR1278117.64179962.1:1-27 HWI-ST574:196:D08GAACXX:5:2212:3487:59718
------------aacagtaataggaatttgttgggcgag
>gnl|SRA|ERR1278117.62726989.1:1-27 HWI-ST574:196:D08GAACXX:5:2210:8921:98713
------------aacagtaataggaatttgttgggcgag
>gnl|SRA|ERR1278117.55162966.1:1-27 HWI-ST574:196:D08GAACXX:5:2201:4288:44911

------------aacagtaataggaatttgttgggcgag
>gnl|SRA|ERR1278117.53524771.1:1-27 HWI-ST574:196:D08GAACXX:5:2115:14373:81234
------------aacagtaataggaatttgttgggcgag
>gnl|SRA|ERR1278117.51987653.1:1-27 HWI-ST574:196:D08GAACXX:5:2114:21310:31198
------------aacagtaataggaatttgttgggcgag
>gnl|SRA|ERR1278117.51302706.1:1-27 HWI-ST574:196:D08GAACXX:5:2113:1534:58591
------------aacagtaataggaatttgttgggcgag
>gnl|SRA|ERR1278117.48151976.1:1-27 HWI-ST574:196:D08GAACXX:5:2110:20455:8760
------------aacagtaataggaatttgttgggcgag
>_R_gnl|SRA|ERR1278117.45410226.2:1-27 HWI-ST574:196:D08GAACXX:5:2106:1800:72692
agcacattaaataacagtaataggaat------------
>gnl|SRA|ERR1278117.41692313.1:1-27 HWI-ST574:196:D08GAACXX:5:2101:9125:65179
------------aacagtaataggaatttgttgggcgag
>gnl|SRA|ERR1278117.39251424.1:1-27 HWI-ST574:196:D08GAACXX:5:1315:4544:15016
------------aacagtaataggaatttgttgggcgag
>gnl|SRA|ERR1278117.38658942.1:74-100 HWI-ST574:196:D08GAACXX:5:1314:19763:56313
agcacattaaataacagtaataggaat------------
>gnl|SRA|ERR1278117.37303506.1:1-27 HWI-ST574:196:D08GAACXX:5:1313:19407:18623
------------aacagtaataggaatttgttgggcgag
>gnl|SRA|ERR1278117.34951288.1:1-27 HWI-ST574:196:D08GAACXX:5:1310:15288:65260
------------aacagtaataggaatttgttgggcgag
>gnl|SRA|ERR1278117.33178916.1:1-27 HWI-ST574:196:D08GAACXX:5:1308:8162:63240
------------aacagtaataggaatttgttgggcgag
>gnl|SRA|ERR1278117.31463577.1:1-27 HWI-ST574:196:D08GAACXX:5:1306:8548:56487
------------aacagtaataggaatttgttgggcgag
>gnl|SRA|ERR1278117.30186842.1:74-100 HWI-ST574:196:D08GAACXX:5:1304:2229:94704
agcacattaaataacagtaataggaat------------
>gnl|SRA|ERR1278117.30167466.1:1-27 HWI-ST574:196:D08GAACXX:5:1304:21113:91965
------------aacagtaataggaatttgttgggcgag
>gnl|SRA|ERR1278117.28285792.1:1-27 HWI-ST574:196:D08GAACXX:5:1302:5766:41102
------------aacagtaataggaatttgttgggcgag
>gnl|SRA|ERR1278117.28181097.1:1-27 HWI-ST574:196:D08GAACXX:5:1302:4019:26661
------------aacagtaataggaatttgttgggcgag
>gnl|SRA|ERR1278117.26444425.1:1-27 HWI-ST574:196:D08GAACXX:5:1216:3049:24679
------------aacagtaataggaatttgttgggcgag
>gnl|SRA|ERR1278117.26151120.1:1-27 HWI-ST574:196:D08GAACXX:5:1215:3402:96248
------------aacagtaataggaatttgttgggcgag
>gnl|SRA|ERR1278117.25899247.1:1-27 HWI-ST574:196:D08GAACXX:5:1215:16208:72148
------------aacagtaataggaatttgttgggcgag
>gnl|SRA|ERR1278117.25402053.1:1-27 HWI-ST574:196:D08GAACXX:5:1215:4959:24352
------------aacagtaataggaatttgttgggcgag
>gnl|SRA|ERR1278117.24683436.1:1-27 HWI-ST574:196:D08GAACXX:5:1214:14760:52605
------------aacagtaataggaatttgttgggcgag
>gnl|SRA|ERR1278117.23862570.1:74-100 HWI-ST574:196:D08GAACXX:5:1213:13017:68446
agcacattaaataacagtaataggaat------------
>gnl|SRA|ERR1278117.20345736.1:1-27 HWI-ST574:196:D08GAACXX:5:1209:14203:83357
------------aacagtaataggaatttgttgggcgag
>gnl|SRA|ERR1278117.20158550.1:1-27 HWI-ST574:196:D08GAACXX:5:1209:7977:62115
------------aacagtaataggaatttgttgggcgag
>gnl|SRA|ERR1278117.19443380.1:1-27 HWI-ST574:196:D08GAACXX:5:1208:1492:78588
------------aacagtaataggaatttgttgggcgag

>gnl|SRA|ERR1278117.19410062.1:74-100 HWI-ST574:196:D08GAACXX:5:1208:7829:74739
agcacattaaataacagtaataggaat------------
>gnl|SRA|ERR1278117.16482956.1:1-27 HWI-ST574:196:D08GAACXX:5:1205:2899:14571
------------aacagtaataggaatttgttgggcgag
>gnl|SRA|ERR1278117.15488781.1:1-27 HWI-ST574:196:D08GAACXX:5:1203:10307:83381
------------aacagtaataggaatttgttgggcgag
>gnl|SRA|ERR1278117.14984636.1:1-27 HWI-ST574:196:D08GAACXX:5:1203:2473:16646
------------aacagtaataggaatttgttgggcgag
>gnl|SRA|ERR1278117.14446405.1:1-27 HWI-ST574:196:D08GAACXX:5:1202:14078:42490
------------aacagtaataggaatttgttgggcgag
>gnl|SRA|ERR1278117.12070286.1:1-27 HWI-ST574:196:D08GAACXX:5:1115:17827:69284
------------aacagtaataggaatttgttgggcgag
>gnl|SRA|ERR1278117.11901895.1:1-27 HWI-ST574:196:D08GAACXX:5:1115:14359:52679
------------aacagtaataggaatttgttgggcgag
>gnl|SRA|ERR1278117.11847551.1:1-27 HWI-ST574:196:D08GAACXX:5:1115:4400:47283
------------aacagtaataggaatttgttgggcgag
>gnl|SRA|ERR1278117.9556826.1:1-27 HWI-ST574:196:D08GAACXX:5:1113:19601:8805
------------aacagtaataggaatttgttgggcgag
>gnl|SRA|ERR1278117.8248741.1:1-27 HWI-ST574:196:D08GAACXX:5:1111:7837:64183
------------aacagtaataggaatttgttgggcgag
>gnl|SRA|ERR1278117.6157612.1:1-27 HWI-ST574:196:D08GAACXX:5:1109:19685:21411
------------aacagtaataggaatttgttgggcgag
>gnl|SRA|ERR1278117.4939136.1:1-27 HWI-ST574:196:D08GAACXX:5:1107:16676:72166
------------aacagtaataggaatttgttgggcgag
>gnl|SRA|ERR1278117.3805736.1:1-27 HWI-ST574:196:D08GAACXX:5:1106:1968:28677
------------aacagtaataggaatttgttgggcgag
>gnl|SRA|ERR1278117.2503608.1:74-100 HWI-ST574:196:D08GAACXX:5:1104:12864:56106
agcacattaaataacagtaataggaat------------
>gnl|SRA|ERR1278117.458246.1:1-27 HWI-ST574:196:D08GAACXX:5:1101:5472:68380
------------aacagtaataggaatttgttgggcgag
>gnl|SRA|ERR1278117.82405407.1:1-26 HWI-ST574:196:D08GAACXX:5:2316:11714:75901
-------------acagtaataggaatttgttgggcgag
>gnl|SRA|ERR1278117.82220037.1:75-100 HWI-ST574:196:D08GAACXX:5:2316:13742:59919
agcacattaaataacagtaataggaa-------------
>gnl|SRA|ERR1278117.78355342.1:1-26 HWI-ST574:196:D08GAACXX:5:2312:20227:85646
-------------acagtaataggaatttgttgggcgag
>gnl|SRA|ERR1278117.77003758.1:1-26 HWI-ST574:196:D08GAACXX:5:2311:12886:41615
-------------acagtaataggaatttgttgggcgag
>gnl|SRA|ERR1278117.75611708.1:1-26 HWI-ST574:196:D08GAACXX:5:2309:5392:85884
-------------acagtaataggaatttgttgggcgag
>gnl|SRA|ERR1278117.75392120.1:1-26 HWI-ST574:196:D08GAACXX:5:2309:14244:60890
-------------acagtaataggaatttgttgggcgag
>gnl|SRA|ERR1278117.67947356.1:1-26 HWI-ST574:196:D08GAACXX:5:2216:3871:36767
-------------acagtaataggaatttgttgggcgag
>gnl|SRA|ERR1278117.67079184.1:1-26 HWI-ST574:196:D08GAACXX:5:2215:14445:56040
-------------acagtaataggaatttgttgggcgag
>gnl|SRA|ERR1278117.66668711.1:1-26 HWI-ST574:196:D08GAACXX:5:2215:13315:17242
-------------acagtaataggaatttgttgggcgag
>gnl|SRA|ERR1278117.65601082.1:1-26 HWI-ST574:196:D08GAACXX:5:2214:3102:12237
-------------acagtaataggaatttgttgggcgag
>gnl|SRA|ERR1278117.49916754.1:1-26 HWI-ST574:196:D08GAACXX:5:2112:8799:9597

-------------acagtaataggaatttgttgggcgag
>gnl|SRA|ERR1278117.39627669.1:72-100 HWI-ST574:196:D08GAACXX:5:1315:21228:50635
agcacattaaataacagtaatagaaattt----------
>gnl|SRA|ERR1278117.35442204.1:1-26 HWI-ST574:196:D08GAACXX:5:1311:4826:20089
-------------acagtaataggaatttgttgggcgag
>gnl|SRA|ERR1278117.34378774.1:1-26 HWI-ST574:196:D08GAACXX:5:1309:19705:100868
-------------acagtaataggaatttgttgggcgag
>gnl|SRA|ERR1278117.31451074.1:1-26 HWI-ST574:196:D08GAACXX:5:1306:4331:54919
-------------acagtaataggaatttgttgggcgag
>gnl|SRA|ERR1278117.20766614.1:1-26 HWI-ST574:196:D08GAACXX:5:1210:12280:32003
-------------acagtaataggaatttgttgggcgag
>gnl|SRA|ERR1278117.19826205.1:1-26 HWI-ST574:196:D08GAACXX:5:1209:19549:23939
-------------acagtaataggaatttgttgggcgag
>gnl|SRA|ERR1278117.9539747.1:1-26 HWI-ST574:196:D08GAACXX:5:1113:13341:7174
-------------acagtaataggaatttgttgggcgag
>gnl|SRA|ERR1278117.2355207.1:1-26 HWI-ST574:196:D08GAACXX:5:1104:19675:36015
-------------acagtaataggaatttgttgggcgag

SRX1465570; SRX1465595
>control .
caacctccagaattcaaacaggaatttactgggtgagtctct
>_R_gnl|SRA|SRR2976091.10491237.1:50-91 HWI-
D00258:15:C28DUACXX:3:2316:13172:100741.
caacctccagaattcaaacaggaatttactgggtgagtctct
>_R_gnl|SRA|SRR2976091.10411888.1:14-55 HWI-
D00258:15:C28DUACXX:3:2316:5555:49542.
caacctccagaattcaaacaggaatttactgggtgagtctct
>_R_gnl|SRA|SRR2976091.10311734.1:20-61 HWI-
D00258:15:C28DUACXX:3:2315:3288:83157.
caacctccagaattcaaacaggaatttactgggtgagtctct
>_R_gnl|SRA|SRR2976091.10244787.1:50-91 HWI-
D00258:15:C28DUACXX:3:2315:12053:37597.
caacctccagaattcaaacaggaatttactgggtgagtctct
>_R_gnl|SRA|SRR2976091.10108539.1:38-79 HWI-
D00258:15:C28DUACXX:3:2314:14661:39994.
caacctccagaattcaaacaggaatttactgggtgagtctct
>_R_gnl|SRA|SRR2976091.10046119.1:15-56 HWI-
D00258:15:C28DUACXX:3:2313:4953:94510.
caacctccagaattcaaacaggaatttactgggtgagtctct
>_R_gnl|SRA|SRR2976091.10025797.1:16-57 HWI-
D00258:15:C28DUACXX:3:2313:10050:79670.
caacctccagaattcaaacaggaatttactgggtgagtctct
>_R_gnl|SRA|SRR2976091.9930101.1:2-43 HWI-D00258:15:C28DUACXX:3:2313:6659:8936.
caacctccagaattcaaacaggaatttactgggtgagtctct
>_R_gnl|SRA|SRR2976091.9830814.1:26-67 HWI-D00258:15:C28DUACXX:3:2312:2879:29697.
caacctccagaattcaaacaggaatttactgggtgagtctct
>_R_gnl|SRA|SRR2976091.9809694.1:21-62 HWI-
D00258:15:C28DUACXX:3:2312:16075:12871.
caacctccagaattcaaacaggaatttactgggtgagtctct
>_R_gnl|SRA|SRR2976091.9554752.1:55-96 HWI-D00258:15:C28DUACXX:3:2309:6348:96406.
caacctccagaattcaaacaggaatttactgggtgagtctct

>_R_gnl|SRA|SRR2976091.9547033.1:2-43 HWI-D00258:15:C28DUACXX:3:2309:5572:89614.
caacctccagaattcaaacaggaatttactgggtgagtctct
>_R_gnl|SRA|SRR2976091.9445749.1:3-44 HWI-D00258:15:C28DUACXX:3:2308:12907:99205.
caacctccagaattcaaacaggaatttactgggtgagtctct
>_R_gnl|SRA|SRR2976091.9207586.1:55-96 HWI-
D00258:15:C28DUACXX:3:2306:20523:76223.
caacctccagaattcaaacaggaatttactgggtgagtctct
>_R_gnl|SRA|SRR2976091.9138185.1:21-62 HWI-D00258:15:C28DUACXX:3:2306:14447:7786.
caacctccagaattcaaacaggaatttactgggtgagtctct
>_R_gnl|SRA|SRR2976091.9126037.1:1-42 HWI-D00258:15:C28DUACXX:3:2305:2292:95368.
caacctccagaattcaaacaggaatttactgggtgagtctct
>_R_gnl|SRA|SRR2976091.8883486.1:19-60 HWI-D00258:15:C28DUACXX:3:2303:8058:41866.
caacctccagaattcaaacaggaatttactgggtgagtctct
>_R_gnl|SRA|SRR2976091.8868024.1:30-71 HWI-
D00258:15:C28DUACXX:3:2303:13491:25030.
caacctccagaattcaaacaggaatttactgggtgagtctct
>_R_gnl|SRA|SRR2976091.8772992.1:40-81 HWI-D00258:15:C28DUACXX:3:2302:5991:19257.
caacctccagaattcaaacaggaatttactgggtgagtctct
>_R_gnl|SRA|SRR2976091.8709346.1:15-56 HWI-
D00258:15:C28DUACXX:3:2301:13657:45784.
caacctccagaattcaaacaggaatttactgggtgagtctct
>_R_gnl|SRA|SRR2976091.8636895.1:4-45 HWI-D00258:15:C28DUACXX:3:2216:9170:79573.
caacctccagaattcaaacaggaatttactgggtgagtctct
>_R_gnl|SRA|SRR2976091.8571525.1:30-71 HWI-D00258:15:C28DUACXX:3:2216:8119:37645.
caacctccagaattcaaacaggaatttactgggtgagtctct
>_R_gnl|SRA|SRR2976091.8404937.1:50-91 HWI-
D00258:15:C28DUACXX:3:2215:11694:26608.
caacctccagaattcaaacaggaatttactgggtgagtctct
>_R_gnl|SRA|SRR2976091.8329741.1:30-71 HWI-
D00258:15:C28DUACXX:3:2214:17794:74321.
caacctccagaattcaaacaggaatttactgggtgagtctct
>_R_gnl|SRA|SRR2976091.8259854.1:35-76 HWI-
D00258:15:C28DUACXX:3:2214:12242:25077.
caacctccagaattcaaacaggaatttactgggtgagtctct
>_R_gnl|SRA|SRR2976091.8166858.1:3-44 HWI-D00258:15:C28DUACXX:3:2213:20794:57185.
caacctccagaattcaaacaggaatttactgggtgagtctct
>_R_gnl|SRA|SRR2976091.8084412.1:15-56 HWI-D00258:15:C28DUACXX:3:2212:9358:95882.
caacctccagaattcaaacaggaatttactgggtgagtctct
>_R_gnl|SRA|SRR2976091.7914768.1:2-43 HWI-D00258:15:C28DUACXX:3:2211:1454:65435.
caacctccagaattcaaacaggaatttactgggtgagtctct
>_R_gnl|SRA|SRR2976091.7788960.1:15-56 HWI-
D00258:15:C28DUACXX:3:2210:14977:62613.
caacctccagaattcaaacaggaatttactgggtgagtctct
>_R_gnl|SRA|SRR2976091.7452186.1:50-91 HWI-
D00258:15:C28DUACXX:3:2207:19072:63738.
caacctccagaattcaaacaggaatttactgggtgagtctct
>_R_gnl|SRA|SRR2976091.7362053.1:50-91 HWI-
D00258:15:C28DUACXX:3:2206:19770:77567.
caacctccagaattcaaacaggaatttactgggtgagtctct
>_R_gnl|SRA|SRR2976091.7267524.1:55-96 HWI-
D00258:15:C28DUACXX:3:2205:14503:85347.

caacctccagaattcaaacaggaatttactgggtgagtctct
>_R_gnl|SRA|SRR2976091.7168833.1:29-70 HWI-D00258:15:C28DUACXX:3:2204:1912:85815.
caacctccagaattcaaacaggaatttactgggtgagtctct
>_R_gnl|SRA|SRR2976091.7066723.1:2-43 HWI-D00258:15:C28DUACXX:3:2203:19915:79238.
caacctccagaattcaaacaggaatttactgggtgagtctct
>_R_gnl|SRA|SRR2976091.6975233.1:35-76 HWI-D00258:15:C28DUACXX:3:2202:17288:81480.
caacctccagaattcaaacaggaatttactgggtgagtctct
>_R_gnl|SRA|SRR2976091.6867402.1:15-56 HWI-D00258:15:C28DUACXX:3:2201:2621:62730.
caacctccagaattcaaacaggaatttactgggtgagtctct
>_R_gnl|SRA|SRR2976091.6759589.1:36-77 HWI-D00258:15:C28DUACXX:3:2116:1887:65492.
caacctccagaattcaaacaggaatttactgggtgagtctct
>_R_gnl|SRA|SRR2976091.6757004.1:7-48 HWI-D00258:15:C28DUACXX:3:2116:21008:63403.
caacctccagaattcaaacaggaatttactgggtgagtctct
>_R_gnl|SRA|SRR2976091.6681122.1:43-84 HWI-D00258:15:C28DUACXX:3:2116:11382:12161.
caacctccagaattcaaacaggaatttactgggtgagtctct
>_R_gnl|SRA|SRR2976091.6556834.1:15-56 HWI-D00258:15:C28DUACXX:3:2115:1535:24824.
caacctccagaattcaaacaggaatttactgggtgagtctct
>_R_gnl|SRA|SRR2976091.6433586.1:2-43 HWI-D00258:15:C28DUACXX:3:2114:10716:35474.
caacctccagaattcaaacaggaatttactgggtgagtctct
>_R_gnl|SRA|SRR2976091.6387090.1:21-62 HWI-D00258:15:C28DUACXX:3:2113:8277:100438.
caacctccagaattcaaacaggaatttactgggtgagtctct
>_R_gnl|SRA|SRR2976091.6184910.1:55-96 HWI-D00258:15:C28DUACXX:3:2112:4805:44214.
caacctccagaattcaaacaggaatttactgggtgagtctct
>_R_gnl|SRA|SRR2976091.6183549.1:55-96 HWI-D00258:15:C28DUACXX:3:2112:17659:42791.
caacctccagaattcaaacaggaatttactgggtgagtctct
>_R_gnl|SRA|SRR2976091.6168126.1:58-99 HWI-D00258:15:C28DUACXX:3:2112:16569:30307.
caacctccagaattcaaacaggaatttactgggtgagtctct
>_R_gnl|SRA|SRR2976091.5883139.1:16-57 HWI-D00258:15:C28DUACXX:3:2109:4837:85082.
caacctccagaattcaaacaggaatttactgggtgagtctct
>_R_gnl|SRA|SRR2976091.5743077.1:23-64 HWI-D00258:15:C28DUACXX:3:2108:18501:55778.
caacctccagaattcaaacaggaatttactgggtgagtctct
>_R_gnl|SRA|SRR2976091.5566029.1:55-96 HWI-D00258:15:C28DUACXX:3:2106:13855:83260.
caacctccagaattcaaacaggaatttactgggtgagtctct
>_R_gnl|SRA|SRR2976091.5548368.1:52-93 HWI-D00258:15:C28DUACXX:3:2106:9189:65904.
caacctccagaattcaaacaggaatttactgggtgagtctct
>_R_gnl|SRA|SRR2976091.5529413.1:7-48 HWI-D00258:15:C28DUACXX:3:2106:14558:46512.
caacctccagaattcaaacaggaatttactgggtgagtctct
>_R_gnl|SRA|SRR2976091.5387446.1:59-100 HWI-D00258:15:C28DUACXX:3:2104:12687:97167.
caacctccagaattcaaacaggaatttactgggtgagtctct
>_R_gnl|SRA|SRR2976091.5379949.1:50-91 HWI-D00258:15:C28DUACXX:3:2104:2909:89130.
caacctccagaattcaaacaggaatttactgggtgagtctct
>_R_gnl|SRA|SRR2976091.5312474.1:55-96 HWI-D00258:15:C28DUACXX:3:2104:19667:14518.

caacctccagaattcaaacaggaatttactgggtgagtctct
>_R_gnl|SRA|SRR2976091.5247197.1:15-56 HWI-D00258:15:C28DUACXX:3:2103:10127:41311.
caacctccagaattcaaacaggaatttactgggtgagtctct
>_R_gnl|SRA|SRR2976091.5043024.1:15-56 HWI-D00258:15:C28DUACXX:3:1316:17346:100641.
caacctccagaattcaaacaggaatttactgggtgagtctct
>_R_gnl|SRA|SRR2976091.5028131.1:21-62 HWI-D00258:15:C28DUACXX:3:1316:13768:90832.
caacctccagaattcaaacaggaatttactgggtgagtctct
>_R_gnl|SRA|SRR2976091.4996799.1:55-96 HWI-D00258:15:C28DUACXX:3:1316:10729:70346.
caacctccagaattcaaacaggaatttactgggtgagtctct
>_R_gnl|SRA|SRR2976091.4976025.1:7-48 HWI-D00258:15:C28DUACXX:3:1316:4188:56620.
caacctccagaattcaaacaggaatttactgggtgagtctct
>_R_gnl|SRA|SRR2976091.4916581.1:21-62 HWI-D00258:15:C28DUACXX:3:1316:11139:16064.
caacctccagaattcaaacaggaatttactgggtgagtctct
>_R_gnl|SRA|SRR2976091.4886968.1:21-62 HWI-D00258:15:C28DUACXX:3:1315:1842:95249.
caacctccagaattcaaacaggaatttactgggtgagtctct
>_R_gnl|SRA|SRR2976091.4795368.1:21-62 HWI-D00258:15:C28DUACXX:3:1315:12679:30396.
caacctccagaattcaaacaggaatttactgggtgagtctct
>_R_gnl|SRA|SRR2976091.4751749.1:15-56 HWI-D00258:15:C28DUACXX:3:1314:15897:98039.
caacctccagaattcaaacaggaatttactgggtgagtctct
>_R_gnl|SRA|SRR2976091.4744122.1:15-56 HWI-D00258:15:C28DUACXX:3:1314:19032:92617.
caacctccagaattcaaacaggaatttactgggtgagtctct
>_R_gnl|SRA|SRR2976091.4431591.1:13-54 HWI-D00258:15:C28DUACXX:3:1312:14461:50105.
caacctccagaattcaaacaggaatttactgggtgagtctct
>_R_gnl|SRA|SRR2976091.4221528.1:57-98 HWI-D00258:15:C28DUACXX:3:1310:15117:70216.
caacctccagaattcaaacaggaatttactgggtgagtctct
>_R_gnl|SRA|SRR2976091.4182109.1:21-62 HWI-D00258:15:C28DUACXX:3:1310:3782:34823.
caacctccagaattcaaacaggaatttactgggtgagtctct
>_R_gnl|SRA|SRR2976091.4150369.1:43-84 HWI-D00258:15:C28DUACXX:3:1310:9952:5832.
caacctccagaattcaaacaggaatttactgggtgagtctct
>_R_gnl|SRA|SRR2976091.4144342.1:14-55 HWI-D00258:15:C28DUACXX:3:1309:2937:99934.
caacctccagaattcaaacaggaatttactgggtgagtctct
>_R_gnl|SRA|SRR2976091.3913802.1:54-95 HWI-D00258:15:C28DUACXX:3:1307:12172:75178.
caacctccagaattcaaacaggaatttactgggtgagtctct
>_R_gnl|SRA|SRR2976091.3847254.1:15-56 HWI-D00258:15:C28DUACXX:3:1307:13217:6603.
caacctccagaattcaaacaggaatttactgggtgagtctct
>_R_gnl|SRA|SRR2976091.3692639.1:35-76 HWI-D00258:15:C28DUACXX:3:1305:5187:37932.
caacctccagaattcaaacaggaatttactgggtgagtctct
>_R_gnl|SRA|SRR2976091.3468879.1:19-60 HWI-D00258:15:C28DUACXX:3:1302:5365:71287.
caacctccagaattcaaacaggaatttactgggtgagtctct
>_R_gnl|SRA|SRR2976091.3342646.1:2-43 HWI-D00258:15:C28DUACXX:3:1301:20830:8518.

caacctccagaattcaaacaggaatttactgggtgagtctct
>_R_gnl|SRA|SRR2976091.3316983.1:13-54 HWI-D00258:15:C28DUACXX:3:1216:7780:87934.
caacctccagaattcaaacaggaatttactgggtgagtctct
>_R_gnl|SRA|SRR2976091.3270810.1:16-57 HWI-D00258:15:C28DUACXX:3:1216:14360:57083.
caacctccagaattcaaacaggaatttactgggtgagtctct
>_R_gnl|SRA|SRR2976091.3196486.1:21-62 HWI-D00258:15:C28DUACXX:3:1216:12008:6503.
caacctccagaattcaaacaggaatttactgggtgagtctct
>_R_gnl|SRA|SRR2976091.3056775.1:35-76 HWI-D00258:15:C28DUACXX:3:1215:13523:7397.
caacctccagaattcaaacaggaatttactgggtgagtctct
>_R_gnl|SRA|SRR2976091.2928313.1:55-96 HWI-D00258:15:C28DUACXX:3:1214:15499:11455.
caacctccagaattcaaacaggaatttactgggtgagtctct
>_R_gnl|SRA|SRR2976091.2888942.1:26-67 HWI-D00258:15:C28DUACXX:3:1213:18146:80850.
caacctccagaattcaaacaggaatttactgggtgagtctct
>_R_gnl|SRA|SRR2976091.2817376.1:34-75 HWI-D00258:15:C28DUACXX:3:1213:20768:25400.
caacctccagaattcaaacaggaatttactgggtgagtctct
>_R_gnl|SRA|SRR2976091.2690426.1:40-81 HWI-D00258:15:C28DUACXX:3:1212:11226:22749.
caacctccagaattcaaacaggaatttactgggtgagtctct
>_R_gnl|SRA|SRR2976091.2681122.1:55-96 HWI-D00258:15:C28DUACXX:3:1212:4922:15178.
caacctccagaattcaaacaggaatttactgggtgagtctct
>_R_gnl|SRA|SRR2976091.2423925.1:49-90 HWI-D00258:15:C28DUACXX:3:1209:6082:88880.
caacctccagaattcaaacaggaatttactgggtgagtctct
>_R_gnl|SRA|SRR2976091.2344231.1:2-43 HWI-D00258:15:C28DUACXX:3:1209:2178:13796.
caacctccagaattcaaacaggaatttactgggtgagtctct
>_R_gnl|SRA|SRR2976091.2311625.1:43-84 HWI-D00258:15:C28DUACXX:3:1208:4561:81889.
caacctccagaattcaaacaggaatttactgggtgagtctct
>_R_gnl|SRA|SRR2976091.2299404.1:43-84 HWI-D00258:15:C28DUACXX:3:1208:8159:70093.
caacctccagaattcaaacaggaatttactgggtgagtctct
>_R_gnl|SRA|SRR2976091.2227684.1:3-44 HWI-D00258:15:C28DUACXX:3:1207:4540:98490.
caacctccagaattcaaacaggaatttactgggtgagtctct
>_R_gnl|SRA|SRR2976091.2132031.1:21-62 HWI-D00258:15:C28DUACXX:3:1206:2872:99348.
caacctccagaattcaaacaggaatttactgggtgagtctct
>_R_gnl|SRA|SRR2976091.1932686.1:13-54 HWI-D00258:15:C28DUACXX:3:1204:6571:76388.
caacctccagaattcaaacaggaatttactgggtgagtctct
>_R_gnl|SRA|SRR2976091.1900495.1:24-65 HWI-D00258:15:C28DUACXX:3:1204:13700:38727.
caacctccagaattcaaacaggaatttactgggtgagtctct
>_R_gnl|SRA|SRR2976091.1865839.1:38-79 HWI-D00258:15:C28DUACXX:3:1203:5023:96369.
caacctccagaattcaaacaggaatttactgggtgagtctct
>_R_gnl|SRA|SRR2976091.1767598.1:1-42 HWI-D00258:15:C28DUACXX:3:1202:10160:74524.
caacctccagaattcaaacaggaatttactgggtgagtctct
>_R_gnl|SRA|SRR2976091.1754199.1:2-43 HWI-D00258:15:C28DUACXX:3:1202:1486:57786.
caacctccagaattcaaacaggaatttactgggtgagtctct
>_R_gnl|SRA|SRR2976091.1326504.1:7-48 HWI-D00258:15:C28DUACXX:3:1114:4676:77968.
caacctccagaattcaaacaggaatttactgggtgagtctct
>_R_gnl|SRA|SRR2976091.1245452.1:20-61 HWI-D00258:15:C28DUACXX:3:1114:15559:15151.

caacctccagaattcaaacaggaatttactgggtgagtctct
>_R_gnl|SRA|SRR2976091.1058150.1:37-78 HWI-
D00258:15:C28DUACXX:3:1112:10294:62089.
caacctccagaattcaaacaggaatttactgggtgagtctct
>_R_gnl|SRA|SRR2976091.1040931.1:16-57 HWI-
D00258:15:C28DUACXX:3:1112:17137:47505.
caacctccagaattcaaacaggaatttactgggtgagtctct
>_R_gnl|SRA|SRR2976091.933837.1:2-43 HWI-D00258:15:C28DUACXX:3:1111:15571:53375.
caacctccagaattcaaacaggaatttactgggtgagtctct
>_R_gnl|SRA|SRR2976091.901333.1:40-81 HWI-D00258:15:C28DUACXX:3:1111:12032:24164.
caacctccagaattcaaacaggaatttactgggtgagtctct
>_R_gnl|SRA|SRR2976091.885679.1:43-84 HWI-D00258:15:C28DUACXX:3:1111:3342:10169.
caacctccagaattcaaacaggaatttactgggtgagtctct
>_R_gnl|SRA|SRR2976091.762687.1:43-84 HWI-D00258:15:C28DUACXX:3:1109:13521:94040.
caacctccagaattcaaacaggaatttactgggtgagtctct
>_R_gnl|SRA|SRR2976091.743932.1:17-58 HWI-D00258:15:C28DUACXX:3:1109:3624:76380.
caacctccagaattcaaacaggaatttactgggtgagtctct
>_R_gnl|SRA|SRR2976091.550771.1:14-55 HWI-D00258:15:C28DUACXX:3:1107:7148:80035.
caacctccagaattcaaacaggaatttactgggtgagtctct
>_R_gnl|SRA|SRR2976091.549017.1:5-46 HWI-D00258:15:C28DUACXX:3:1107:20030:78108.
caacctccagaattcaaacaggaatttactgggtgagtctct
>_R_gnl|SRA|SRR2976091.497269.1:6-47 HWI-D00258:15:C28DUACXX:3:1107:6209:23156.
caacctccagaattcaaacaggaatttactgggtgagtctct
>_R_gnl|SRA|SRR2976091.445257.1:11-52 HWI-D00258:15:C28DUACXX:3:1106:11583:65141.
caacctccagaattcaaacaggaatttactgggtgagtctct
>_R_gnl|SRA|SRR2976091.445171.1:33-74 HWI-D00258:15:C28DUACXX:3:1106:3968:65247.
caacctccagaattcaaacaggaatttactgggtgagtctct
>_R_gnl|SRA|SRR2976091.374821.1:20-61 HWI-D00258:15:C28DUACXX:3:1105:2877:84327.
caacctccagaattcaaacaggaatttactgggtgagtctct
>_R_gnl|SRA|SRR2976091.368650.1:2-43 HWI-D00258:15:C28DUACXX:3:1105:7962:77246.
caacctccagaattcaaacaggaatttactgggtgagtctct
>_R_gnl|SRA|SRR2976091.366228.1:14-55 HWI-D00258:15:C28DUACXX:3:1105:6869:74453.
caacctccagaattcaaacaggaatttactgggtgagtctct
>_R_gnl|SRA|SRR2976091.312870.1:47-88 HWI-D00258:15:C28DUACXX:3:1105:7117:11557.
caacctccagaattcaaacaggaatttactgggtgagtctct
>_R_gnl|SRA|SRR2976091.252382.1:54-95 HWI-D00258:15:C28DUACXX:3:1104:13282:37244.
caacctccagaattcaaacaggaatttactgggtgagtctct
>gnl|SRA|SRR2976065.23677038.1:16-57 HWI-ST330:282:C1C61ACXX:1:2211:4023:74131.
caacctccagaattcaaacaggaatttactgggtgagtctct
>gnl|SRA|SRR2976065.22929328.1:58-99 HWI-ST330:282:C1C61ACXX:1:2209:5606:48057.
caacctccagaattcaaacaggaatttactgggtgagtctct
>gnl|SRA|SRR2976065.22868121.1:35-76 HWI-ST330:282:C1C61ACXX:1:2209:9650:29453.
caacctccagaattcaaacaggaatttactgggtgagtctct
>_R_gnl|SRA|SRR2976065.20623770.1:36-77 HWI-
ST330:282:C1C61ACXX:1:2201:7128:62759.
caacctccagaattcaaacaggaatttactgggtgagtctct
>_R_gnl|SRA|SRR2976065.18116830.1:23-64 HWI-
ST330:282:C1C61ACXX:1:2110:16599:41662.
caacctccagaattcaaacaggaatttactgggtgagtctct
>gnl|SRA|SRR2976065.16404646.1:53-94 HWI-ST330:282:C1C61ACXX:1:2104:17578:98974.
caacctccagaattcaaacaggaatttactgggtgagtctct

>_R_gnl|SRA|SRR2976065.16216986.1:43-84 HWI-
ST330:282:C1C61ACXX:1:2104:16482:36038.
caacctccagaattcaaacaggaatttactgggtgagtctct
>_R_gnl|SRA|SRR2976065.15742205.1:24-65 HWI-
ST330:282:C1C61ACXX:1:2102:10880:70505.
caacctccagaattcaaacaggaatttactgggtgagtctct
>_R_gnl|SRA|SRR2976065.14613609.1:43-84 HWI-
ST330:282:C1C61ACXX:1:1315:13193:11296.
caacctccagaattcaaacaggaatttactgggtgagtctct
>gnl|SRA|SRR2976065.14399356.1:59-100 HWI-ST330:282:C1C61ACXX:1:1314:14566:50230.
caacctccagaattcaaacaggaatttactgggtgagtctct
>_R_gnl|SRA|SRR2976065.11549629.1:43-84 HWI-
ST330:282:C1C61ACXX:1:1305:11043:81228.
caacctccagaattcaaacaggaatttactgggtgagtctct
>_R_gnl|SRA|SRR2976065.11203171.1:50-91 HWI-
ST330:282:C1C61ACXX:1:1304:12812:64101.
caacctccagaattcaaacaggaatttactgggtgagtctct
>gnl|SRA|SRR2976065.9640805.1:30-71 HWI-ST330:282:C1C61ACXX:1:1215:5486:36606.
caacctccagaattcaaacaggaatttactgggtgagtctct
>_R_gnl|SRA|SRR2976065.8448042.1:19-60 HWI-
ST330:282:C1C61ACXX:1:1211:15215:93984.
caacctccagaattcaaacaggaatttactgggtgagtctct
>gnl|SRA|SRR2976065.7502374.1:33-74 HWI-ST330:282:C1C61ACXX:1:1209:5490:6038.
caacctccagaattcaaacaggaatttactgggtgagtctct
>_R_gnl|SRA|SRR2976065.5564024.1:50-91 HWI-
ST330:282:C1C61ACXX:1:1202:10958:46853.
caacctccagaattcaaacaggaatttactgggtgagtctct
>gnl|SRA|SRR2976065.5165711.1:2-43 HWI-ST330:282:C1C61ACXX:1:1116:14410:99198.
caacctccagaattcaaacaggaatttactgggtgagtctct
>_R_gnl|SRA|SRR2976065.5151299.1:55-96 HWI-ST330:282:C1C61ACXX:1:1116:6528:94809.
caacctccagaattcaaacaggaatttactgggtgagtctct
>_R_gnl|SRA|SRR2976065.2096444.1:55-96 HWI-
ST330:282:C1C61ACXX:1:1108:11067:10544.
caacctccagaattcaaacaggaatttactgggtgagtctct
>_R_gnl|SRA|SRR2976065.1996251.1:21-62 HWI-
ST330:282:C1C61ACXX:1:1107:16876:78744.
caacctccagaattcaaacaggaatttactgggtgagtctct
>gnl|SRA|SRR2976065.1772425.1:9-50 HWI-ST330:282:C1C61ACXX:1:1107:7292:8118.
caacctccagaattcaaacaggaatttactgggtgagtctct
>gnl|SRA|SRR2976065.1465899.1:36-77 HWI-ST330:282:C1C61ACXX:1:1106:4593:9149.
caacctccagaattcaaacaggaatttactgggtgagtctct
>gnl|SRA|SRR2976065.1112990.1:4-45 HWI-ST330:282:C1C61ACXX:1:1104:8282:91390.
caacctccagaattcaaacaggaatttactgggtgagtctct
>_R_gnl|SRA|SRR2976091.10356065.1:54-95 HWI-
D00258:15:C28DUACXX:3:2316:18614:13211.
caacctccagacttcaaacaggaatttactgggtgagtctct
>_R_gnl|SRA|SRR2976091.6459448.1:54-95 HWI-
D00258:15:C28DUACXX:3:2114:14585:54307.
caacctccagaattcaaacaggaatttactgggtcagtctct
>_R_gnl|SRA|SRR2976091.4114719.1:23-64 HWI-D00258:15:C28DUACXX:3:1309:5771:72162.
caacctccagaattcaaacaggaatttactgggggagtctct

>gnl|SRA|SRR2976065.21062365.1:61-100 HWI-ST330:282:C1C61ACXX:1:2203:4816:22153.
caacctccagaattcaaacaggaatttactgggtgagtct--
>gnl|SRA|SRR2976065.16622267.1:61-100 HWI-ST330:282:C1C61ACXX:1:2105:3597:72217.
caacctccagaattcaaacaggaatttactgggtgagtct--
>gnl|SRA|SRR2976065.11641552.1:61-100 HWI-ST330:282:C1C61ACXX:1:1306:4679:12520.
caacctccagaattcaaacaggaatttactgggtgagtct--
>gnl|SRA|SRR2976065.10724989.1:61-100 HWI-ST330:282:C1C61ACXX:1:1302:5017:86869.
caacctccagaattcaaacaggaatttactgggtgagtct--
>gnl|SRA|SRR2976065.23972778.1:63-100 HWI-ST330:282:C1C61ACXX:1:2212:1957:61044.
caacctccagaattcaaacaggaatttactgggtgagt----
>gnl|SRA|SRR2976065.22075713.1:63-100 HWI-ST330:282:C1C61ACXX:1:2206:16823:75521.
caacctccagaattcaaacaggaatttactgggtgagt----
>_R_gnl|SRA|SRR2976091.10438582.1:1-37 HWI-D00258:15:C28DUACXX:3:2316:4183:67152.
caacctccagaattcaaacaggaatttactgggtgag-----
>_R_gnl|SRA|SRR2976091.5950737.1:1-31 HWI-D00258:15:C28DUACXX:3:2110:2517:45480.
caacctccagaattcaaacaggaatttactg-----------
>_R_gnl|SRA|SRR2976065.11573024.1:1-30 HWI-
ST330:282:C1C61ACXX:1:1305:18322:88597.
caacctccagaattcaaacaggaatttact------------
>gnl|SRA|SRR2976065.4924514.1:71-100 HWI-ST330:282:C1C61ACXX:1:1116:14902:27393.
caacctccagaattcaaacaggaatttact------------
>_R_gnl|SRA|SRR2976091.8578034.1:1-29 HWI-D00258:15:C28DUACXX:3:2216:4655:41953.
caacctccagaattcaaacaggaatttac-------------
>_R_gnl|SRA|SRR2976091.7966334.1:1-29 HWI-D00258:15:C28DUACXX:3:2212:4765:6334.
caacctccagaattcaaacaggaatttac-------------
>_R_gnl|SRA|SRR2976091.6251480.1:1-29 HWI-D00258:15:C28DUACXX:3:2112:11891:96522.
caacctccagaattcaaacaggaatttac-------------
>_R_gnl|SRA|SRR2976091.5795929.1:1-29 HWI-D00258:15:C28DUACXX:3:2109:9357:6175.
caacctccagaattcaaacaggaatttac-------------
>_R_gnl|SRA|SRR2976091.5716792.1:1-29 HWI-D00258:15:C28DUACXX:3:2108:3285:31421.
caacctccagaattcaaacaggaatttac-------------
>_R_gnl|SRA|SRR2976065.30405995.1:6-44 HWI-ST330:282:C1C61ACXX:1:2316:4815:45906.
---cctccagaattcaaacaggaatttactgggtgagtctct
>_R_gnl|SRA|SRR2976091.10412250.1:65-101 HWI-
D00258:15:C28DUACXX:3:2316:4867:49904.
-----tccagaattcaaacaggaatttactgggtgagtctct
>_R_gnl|SRA|SRR2976091.9415278.1:65-101 HWI-
D00258:15:C28DUACXX:3:2308:7672:71988.
-----tccagaattcaaacaggaatttactgggtgagtctct
>_R_gnl|SRA|SRR2976091.9024700.1:65-101 HWI-
D00258:15:C28DUACXX:3:2304:1607:92282.
-----tccagaattcaaacaggaatttactgggtgagtctct
>_R_gnl|SRA|SRR2976091.8381534.1:65-101 HWI-
D00258:15:C28DUACXX:3:2215:10622:10594.
-----tccagaattcaaacaggaatttactgggtgagtctct
>_R_gnl|SRA|SRR2976091.8228202.1:65-101 HWI-
D00258:15:C28DUACXX:3:2214:13928:2548.
-----tccagaattcaaacaggaatttactgggtgagtctct
>_R_gnl|SRA|SRR2976091.7656126.1:65-101 HWI-
D00258:15:C28DUACXX:3:2209:21010:48765.
-----tccagaattcaaacaggaatttactgggtgagtctct

>_R_gnl|SRA|SRR2976091.7476003.1:65-101 HWI-D00258:15:C28DUACXX:3:2207:7615:85925.
-----tccagaattcaaacaggaatttactgggtgagtctct
>_R_gnl|SRA|SRR2976091.7316961.1:65-101 HWI-D00258:15:C28DUACXX:3:2206:5088:34554.
-----tccagaattcaaacaggaatttactgggtgagtctct
>_R_gnl|SRA|SRR2976091.7257172.1:65-101 HWI-D00258:15:C28DUACXX:3:2205:6063:75374.
-----tccagaattcaaacaggaatttactgggtgagtctct
>_R_gnl|SRA|SRR2976091.6809315.1:65-101 HWI-D00258:15:C28DUACXX:3:2116:18983:98073.
-----tccagaattcaaacaggaatttactgggtgagtctct
>_R_gnl|SRA|SRR2976091.6398749.1:65-101 HWI-D00258:15:C28DUACXX:3:2114:4495:9722.
-----tccagaattcaaacaggaatttactgggtgagtctct
>_R_gnl|SRA|SRR2976091.6063536.1:65-101 HWI-D00258:15:C28DUACXX:3:2111:8736:42911.
-----tccagaattcaaacaggaatttactgggtgagtctct
>_R_gnl|SRA|SRR2976091.5804465.1:65-101 HWI-D00258:15:C28DUACXX:3:2109:12595:13919.
-----tccagaattcaaacaggaatttactgggtgagtctct
>_R_gnl|SRA|SRR2976091.5783894.1:65-101 HWI-D00258:15:C28DUACXX:3:2108:20550:94091.
-----tccagaattcaaacaggaatttactgggtgagtctct
>_R_gnl|SRA|SRR2976091.4635561.1:65-101 HWI-D00258:15:C28DUACXX:3:1314:14647:11875.
-----tccagaattcaaacaggaatttactgggtgagtctct
>_R_gnl|SRA|SRR2976091.4458090.1:65-101 HWI-D00258:15:C28DUACXX:3:1312:15490:71579.
-----tccagaattcaaacaggaatttactgggtgagtctct
>_R_gnl|SRA|SRR2976091.4313741.1:65-101 HWI-D00258:15:C28DUACXX:3:1311:15057:51263.
-----tccagaattcaaacaggaatttactgggtgagtctct
>_R_gnl|SRA|SRR2976091.3333841.1:65-101 HWI-D00258:15:C28DUACXX:3:1216:16507:98846.
-----tccagaattcaaacaggaatttactgggtgagtctct
>_R_gnl|SRA|SRR2976091.3120518.1:65-101 HWI-D00258:15:C28DUACXX:3:1215:4737:53054.
-----tccagaattcaaacaggaatttactgggtgagtctct
>_R_gnl|SRA|SRR2976091.3020707.1:65-101 HWI-D00258:15:C28DUACXX:3:1214:5232:80587.
-----tccagaattcaaacaggaatttactgggtgagtctct
>_R_gnl|SRA|SRR2976091.2762010.1:65-101 HWI-D00258:15:C28DUACXX:3:1212:5063:80923.
-----tccagaattcaaacaggaatttactgggtgagtctct
>_R_gnl|SRA|SRR2976091.2610016.1:65-101 HWI-D00258:15:C28DUACXX:3:1211:15484:54525.
-----tccagaattcaaacaggaatttactgggtgagtctct
>_R_gnl|SRA|SRR2976091.1717049.1:65-101 HWI-D00258:15:C28DUACXX:3:1202:12077:11097.
-----tccagaattcaaacaggaatttactgggtgagtctct

>_R_gnl|SRA|SRR2976091.1601477.1:65-101 HWI-
D00258:15:C28DUACXX:3:1116:8943:78787.
-----tccagaattcaaacaggaatttactgggtgagtctct
>_R_gnl|SRA|SRR2976091.1228733.1:65-101 HWI-
D00258:15:C28DUACXX:3:1114:10761:2250.
-----tccagaattcaaacaggaatttactgggtgagtctct
>_R_gnl|SRA|SRR2976091.847239.1:65-101 HWI-
D00258:15:C28DUACXX:3:1110:15677:73902.
-----tccagaattcaaacaggaatttactgggtgagtctct
>_R_gnl|SRA|SRR2976091.786827.1:65-101 HWI-D00258:15:C28DUACXX:3:1110:4280:17623.
-----tccagaattcaaacaggaatttactgggtgagtctct
>_R_gnl|SRA|SRR2976091.736907.1:65-101 HWI-
D00258:15:C28DUACXX:3:1109:20906:69304.
-----tccagaattcaaacaggaatttactgggtgagtctct
>_R_gnl|SRA|SRR2976091.406632.1:65-101 HWI-D00258:15:C28DUACXX:3:1106:6336:21665.
-----tccagaattcaaacaggaatttactgggtgagtctct
>_R_gnl|SRA|SRR2976091.80738.1:65-101 HWI-D00258:15:C28DUACXX:3:1102:14430:14722.
-----tccagaattcaaacaggaatttactgggtgagtctct
>_R_gnl|SRA|SRR2976091.58839.1:65-101 HWI-D00258:15:C28DUACXX:3:1101:19080:84142.
-----tccagaattcaaacaggaatttactgggtgagtctct
>_R_gnl|SRA|SRR2976091.2137691.1:65-101 HWI-
D00258:15:C28DUACXX:3:1207:16512:5780.
-----tccagaatttaaacaggaatttactgggtgagtctct
>_R_gnl|SRA|SRR2976091.995102.1:65-101 HWI-D00258:15:C28DUACXX:3:1112:15790:8340.
-----tccaggattcaaacaggaatttactgggtgagtctct
>_R_gnl|SRA|SRR2976091.9805818.1:67-101 HWI-D00258:15:C28DUACXX:3:2312:6752:9845.
-------cagaattcaaacaggaatttactgggtgagtctct
>_R_gnl|SRA|SRR2976091.8990495.1:67-101 HWI-
D00258:15:C28DUACXX:3:2304:12215:56951.
-------cagaattcaaacaggaatttactgggtgagtctct
>_R_gnl|SRA|SRR2976091.7141528.1:67-101 HWI-
D00258:15:C28DUACXX:3:2204:18513:57513.
-------cagaattcaaacaggaatttactgggtgagtctct
>_R_gnl|SRA|SRR2976091.5537867.1:67-101 HWI-
D00258:15:C28DUACXX:3:2106:15965:55048.
-------cagaattcaaacaggaatttactgggtgagtctct
>_R_gnl|SRA|SRR2976091.2505436.1:67-101 HWI-
D00258:15:C28DUACXX:3:1210:13010:62879.
-------cagaattcaaacaggaatttactgggtgagtctct
>_R_gnl|SRA|SRR2976091.1343542.1:67-101 HWI-
D00258:15:C28DUACXX:3:1114:3386:90725.
-------cagaattcaaacaggaatttactgggtgagtctct
>_R_gnl|SRA|SRR2976091.1760754.1:68-101 HWI-
D00258:15:C28DUACXX:3:1202:13537:65945.
--------agaattcaaacaggaatttactgggtgagtctct
>_R_gnl|SRA|SRR2976091.5312964.1:69-101 HWI-
D00258:15:C28DUACXX:3:2104:1760:15330.
---------gaattcaaacaggaatttactgggtgagtctct
>_R_gnl|SRA|SRR2976091.4119041.1:69-101 HWI-
D00258:15:C28DUACXX:3:1309:16995:76010.
---------gaattcaaacaggaatttactgggtgagtctct

>_R_gnl|SRA|SRR2976091.9899664.1:70-101 HWI-
D00258:15:C28DUACXX:3:2312:5044:82761.
----------aattcaaacaggaatttactgggtgagtctct
>_R_gnl|SRA|SRR2976091.9652745.1:70-101 HWI-
D00258:15:C28DUACXX:3:2310:11283:80615.
----------aattcaaacaggaatttactgggtgagtctct
>_R_gnl|SRA|SRR2976091.8248297.1:70-101 HWI-
D00258:15:C28DUACXX:3:2214:13147:16816.
----------aattcaaacaggaatttactgggtgagtctct
>_R_gnl|SRA|SRR2976091.8200875.1:70-101 HWI-
D00258:15:C28DUACXX:3:2213:17871:82086.
----------aattcaaacaggaatttactgggtgagtctct
>_R_gnl|SRA|SRR2976091.8143134.1:70-101 HWI-
D00258:15:C28DUACXX:3:2213:14392:39952.
----------aattcaaacaggaatttactgggtgagtctct
>_R_gnl|SRA|SRR2976091.7145990.1:70-101 HWI-
D00258:15:C28DUACXX:3:2204:1951:62319.
----------aattcaaacaggaatttactgggtgagtctct
>_R_gnl|SRA|SRR2976091.6602105.1:70-101 HWI-
D00258:15:C28DUACXX:3:2115:18896:56701.
----------aattcaaacaggaatttactgggtgagtctct
>_R_gnl|SRA|SRR2976091.6226371.1:70-101 HWI-
D00258:15:C28DUACXX:3:2112:1787:77179.
----------aattcaaacaggaatttactgggtgagtctct
>_R_gnl|SRA|SRR2976091.5925125.1:70-101 HWI-
D00258:15:C28DUACXX:3:2110:20213:22642.
----------aattcaaacaggaatttactgggtgagtctct
>_R_gnl|SRA|SRR2976091.4862661.1:70-101 HWI-
D00258:15:C28DUACXX:3:1315:17272:77763.
----------aattcaaacaggaatttactgggtgagtctct
>_R_gnl|SRA|SRR2976091.3864328.1:70-101 HWI-
D00258:15:C28DUACXX:3:1307:14366:24474.
----------aattcaaacaggaatttactgggtgagtctct
>_R_gnl|SRA|SRR2976091.2465849.1:70-101 HWI-
D00258:15:C28DUACXX:3:1210:14445:27292.
----------aattcaaacaggaatttactgggtgagtctct
>_R_gnl|SRA|SRR2976091.1706321.1:70-101 HWI-
D00258:15:C28DUACXX:3:1201:9786:96603.
----------aattcaaacaggaatttactgggtgagtctct
>_R_gnl|SRA|SRR2976091.1689569.1:70-101 HWI-
D00258:15:C28DUACXX:3:1201:15752:74999.
----------aattcaaacaggaatttactgggtgagtctct
>_R_gnl|SRA|SRR2976091.1136259.1:70-101 HWI-
D00258:15:C28DUACXX:3:1113:14011:27610.
----------aattcaaacaggaatttactgggtgagtctct
>_R_gnl|SRA|SRR2976091.855719.1:70-101 HWI-
D00258:15:C28DUACXX:3:1110:17419:81709.
----------aattcaaacaggaatttactgggtgagtctct
>_R_gnl|SRA|SRR2976091.145555.1:70-101 HWI-
D00258:15:C28DUACXX:3:1102:12546:99628.
----------aattcaaacaggaatttactgggtgagtctct

>_R_gnl|SRA|SRR2976091.29654.1:70-101 HWI-D00258:15:C28DUACXX:3:1101:15014:43764.
----------aattcaaacaggaatttactgggtgagtctct
>_R_gnl|SRA|SRR2976091.9556.1:70-101 HWI-D00258:15:C28DUACXX:3:1101:9250:15713.
----------aattcaaacaggaatttactgggtgagtctct
>_R_gnl|SRA|SRR2976065.7546866.1:69-100 HWI-
ST330:282:C1C61ACXX:1:1209:12299:19548.
----------aattcaaacaggaatttactgggtgagtctct
>_R_gnl|SRA|SRR2976091.5120721.1:71-101 HWI-
D00258:15:C28DUACXX:3:2101:9175:93729.
-----------attcaaacaggaatttactgggtgagtctct
>_R_gnl|SRA|SRR2976065.5350811.1:70-100 HWI-
ST330:282:C1C61ACXX:1:1201:4387:68623.
-----------attcaaacaggaatttactgggtgagtctct
>_R_gnl|SRA|SRR2976065.5139731.1:70-100 HWI-
ST330:282:C1C61ACXX:1:1116:4836:91446.
-----------attcaaacaggaatttactgggtgagtctct
>_R_gnl|SRA|SRR2976091.2354421.1:71-101 HWI-
D00258:15:C28DUACXX:3:1209:6039:23277.
-----------attcaaacaggaatgtactgggtgagtctct
>_R_gnl|SRA|SRR2976091.9952347.1:72-101 HWI-
D00258:15:C28DUACXX:3:2313:20695:25385.
------------ttcaaacaggaatttactgggtgagtctct
>_R_gnl|SRA|SRR2976091.8883193.1:72-101 HWI-
D00258:15:C28DUACXX:3:2303:4039:41681.
------------ttcaaacaggaatttactgggtgagtctct
>_R_gnl|SRA|SRR2976091.6906836.1:72-101 HWI-
D00258:15:C28DUACXX:3:2202:11895:6899.
------------ttcaaacaggaatttactgggtgagtctct
>_R_gnl|SRA|SRR2976091.4781524.1:72-101 HWI-
D00258:15:C28DUACXX:3:1315:3297:20620.
------------ttcaaacaggaatttactgggtgagtctct
>_R_gnl|SRA|SRR2976091.4642841.1:72-101 HWI-
D00258:15:C28DUACXX:3:1314:7726:17606.
------------ttcaaacaggaatttactgggtgagtctct
>_R_gnl|SRA|SRR2976091.3739734.1:72-101 HWI-
D00258:15:C28DUACXX:3:1305:12345:90069.
------------ttcaaacaggaatttactgggtgagtctct
>_R_gnl|SRA|SRR2976091.2373702.1:72-101 HWI-
D00258:15:C28DUACXX:3:1209:3470:41940.
------------ttcaaacaggaatttactgggtgagtctct
>_R_gnl|SRA|SRR2976091.959760.1:72-101 HWI-
D00258:15:C28DUACXX:3:1111:17238:76568.
------------ttcaaacaggaatttactgggtgagtctct
>_R_gnl|SRA|SRR2976091.8985640.1:73-101 HWI-
D00258:15:C28DUACXX:3:2304:5368:51971.
-------------tcaaacaggaatttactgggtgagtctct
>_R_gnl|SRA|SRR2976091.8298921.1:73-101 HWI-
D00258:15:C28DUACXX:3:2214:9196:52830.
-------------tcaaacaggaatttactgggtgagtctct
>_R_gnl|SRA|SRR2976091.6457998.1:73-101 HWI-
D00258:15:C28DUACXX:3:2114:5526:53475.

-------------tcaaacaggaatttactgggtgagtctct
>_R_gnl|SRA|SRR2976065.29100484.1:72-100 HWI-ST330:282:C1C61ACXX:1:2312:16248:72359.
-------------tcaaacaggaatttactgggtgagtctct
